# Novel intron markers to study the phylogeny of closely related mammalian species

**DOI:** 10.1186/1471-2148-10-369

**Published:** 2010-11-30

**Authors:** Javier Igea, Javier Juste, Jose Castresana

**Affiliations:** 1 Institute of Evolutionary Biology (CSIC-UPF), Passeig Maritim de la Barceloneta 37, 08003 Barcelona, Spain; 2 Estacion Biologica de Donana (CSIC), Avda. Americo Vespucio s/n, 41092 Seville, Spain

## Abstract

**Background:**

Multilocus phylogenies can be used to infer the species tree of a group of closely related species. In species trees, the nodes represent the actual separation between species, thus providing essential information about their evolutionary history. In addition, multilocus phylogenies can help in analyses of species delimitation, gene flow and genetic differentiation within species. However, few adequate markers are available for such studies.

**Results:**

In order to develop nuclear markers that can be useful in multilocus studies of mammals, we analyzed the mammalian genomes of human, chimpanzee, macaque, dog and cow. Rodents were excluded due to their unusual genomic features. Introns were extracted from the mammalian genomes because of their greater genetic variability and ease of amplification from the flanking exons. To an initial set of more than 10,000 one-to-one orthologous introns we applied several filters to select introns that belong to single-copy genes, show neutral evolutionary rates and have an adequate length for their amplification. This analysis led to a final list of 224 intron markers randomly distributed along the genome. To experimentally test their validity, we amplified twelve of these introns in a panel of six mammalian species. The result was that seven of these introns gave rise to a PCR band of the expected size in all species. In addition, we sequenced these bands and analyzed the accumulation of substitutions in these introns in five pairs of closely related species. The results showed that the estimated genetic distances in the five species pairs was quite variable among introns and that this divergence cannot be directly predicted from the overall intron divergence in mammals.

**Conclusions:**

We have designed a new set of 224 nuclear introns with optimal features for the phylogeny of closely related mammalian species. A large proportion of the introns tested experimentally showed a perfect amplification and enough variability in most species, indicating that this marker set can be very helpful in multilocus phylogenetics of mammals. Due to the lower variability and stronger stochasticity of nuclear markers with respect to mitochondrial genes, studies should be designed to make use of several markers like the ones designed here.

## Background

Phylogenetic analyses of closely related species are affected by specific problems that are different from those present in phylogenies of more distant species [1]. The first and most obvious difficulty is that nucleotide sequences must have enough variability within and among the studied species to obtain an adequate resolution of the phylogenetic tree. For this reason, mitochondrial genes, which show a high rate of nucleotide substitution, have been the main choice for the reconstruction of phylogenetic trees at the genus and family levels in all animals [2,3]. Another distinctive feature of the phylogenetic reconstruction of closely related species is that gene coalescence and the stochasticity associated with population genetic processes must be taken into account. For example, incomplete lineage sorting may cause the gene tree to have a different topology than the species tree [4-6]. This leads to the necessity of using multiple, unlinked genes, together with the integration of coalescent-based techniques, in the reconstruction of the species tree [7-10]. Phylogenies based on a broad representation of the genome can also help in species delimitation or analyses of genetic variability. Therefore, in these approaches it is essential to make use of the nuclear genome, where there are plenty of unlinked genes. However, nuclear genes in animals usually have much lower evolutionary rates than mitochondrial genes and, sometimes, they are not informative enough for assessing the variability within species or for phylogenetic reconstruction. In addition, due to the larger population size of nuclear genes with respect to mitochondrial genes, the former are more affected by coalescent stochasticity, so the necessity of using multiple genes is stronger [11,12]. Thus, the progress of multilocus phylogenetics requires, as a first step, an important effort of developing unlinked nuclear markers able to provide enough differences within and among species [13].

Systematic efforts to find novel markers for phylogenetic studies have been performed in different groups of organisms [14-21]. Markers selected include exons, introns and intergenic regions. Exons show very little variability for the phylogenies of closely related species, whereas intergenic regions present difficulties for the design of primers of wide-specificity. On the other hand, introns are a part of the genome with large nucleotide variability and, at the same time, they can be easily amplified with primers placed in the adjacent exons [20,22,23]. Thus, they are ideal candidates for multilocus phylogenies of closely related species. However, not all introns are equally valid for this task. Some introns are highly conserved due to their involvement in certain functions and they may not show enough differences between closely related species [24,25]. In fact, introns show a wide range of evolutionary rates. For example, a comparison of human and mouse introns showed genetic distances that ranged between 0.2 and 1.7 substitutions/site [26]. In addition, some introns show disparate rates of evolution in some lineages but not in others [27], indicating an imperfect molecular clock that may affect the measurement of genetic diversity in some species. Different processes, such as a change in chromosome position of the gene or the development of a new isoform by alternative splicing in certain lineages [28], may cause such variations in the evolutionary dynamics and thus in the molecular clock of the introns.

In mammals, introns have been mainly used to resolve deep groupings but also to study more recent phylogenies [29-33]. However, no attempt has been made so far to systematically select an optimal set of introns for mammals. In this work, we have devised a protocol to extract the best introns from the complete mammalian genomes of five species: human, chimpanzee, macaque, dog and cow [34-39]. We deliberately did not use the available genomes of rodents (mouse and rat) because they have genomic features that would have made the comparisons of all mammals problematic. For example, rodents have very attenuated isochors and show very fast evolutionary rates when compared to other mammals [40,41]. This is also true for introns, as previously shown [27]. The high evolutionary rates of rodent introns can complicate the alignments, phylogenetic reconstructions and measurement of genetic distances. To avoid the same problems we also decided not to include marsupials and monotremes. Thus, in this work we concentrated on the analysis of non-rodent eutherian genomes. From these genomes we obtained one-to-one orthologues, constructed alignments and trees from each individual intron and filtered out introns with inadequate features for shallow phylogenies. In addition, introns were selected to come from single-copy genes in order to avoid multiple bands in PCR reactions, to have an adequate length for PCR amplification, and to be surrounded by exons with enough space for primer design. From the resulting introns, we selected a small set that we used to experimentally verify that they work according to the expected features of ease of amplification and high evolutionary rate. Finally, we studied the variability of intron divergence in different species pairs.

## Results and Discussion

### Intron set acquisition

**Figure 1:**
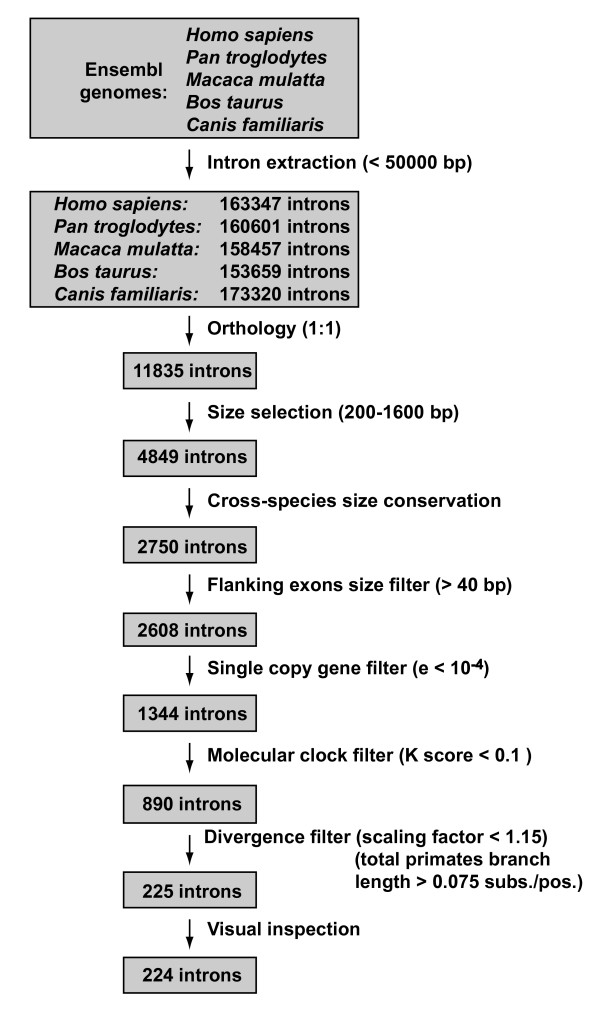
**Scheme of the intron extraction and filtering processes**. bp indicates base pairs. subs./pos. indicates substitutions/position.

We extracted all introns smaller than 50,000 nucleotides from the genomes of human, chimpanzee, macaque, cow and dog, which comprise three mammalian orders (Primates, Carnivora and Cetartiodactyla). The total number of extracted introns per genome ranged between 153,659 in the cow and 173,320 in the dog. Using information from the ENSEMBL database, we arrived at an initial set of 11,835 one-to-one orthologous introns in the five mammalian species (Figure 1).

Most of these introns had an inadequate length for their amplification and were therefore discarded in our initial filtering processes. First, we restricted the intron length in *Homo sapiens *to a minimum of 200 and a maximum of 1600 nucleotides. Second, we controlled for intron length conservation among the five species used in this study, taking human as a referent and relaxing the constraints as the phylogenetic distance between the compared species increased. After the application of these size filters, 2750 introns remained. In addition, the introns flanked by small exons (<40 nucleotides) were discarded because the design of PCR primers could be difficult in them. This filter affected 5.2% of the introns available in the previous step.

Mammalian genomes have a large number of duplicated genes due to different gene or genome duplication processes [42]. These genes constitute a severe problem because primers could hybridize in a large number of genomic places, giving rise to multiple PCR products. Thus, a crucial step was to check for the duplication of the introns using BLAST over the different genomes. In fact, we performed the BLAST searches using the flanking exons instead of the introns because the exonic sequences are easier to detect by BLAST, even in divergent genes [43]. Only those introns that had both flanking exons present just once in the five genomes were considered to be single-copy. This step ruled out approximately half of the remaining introns. At this point, there were 1344 introns with a preliminary orthology filter that can be used to study different evolutionary processes. However, for these introns to be most useful in phylogenetic studies of closely related species we applied several additional filters.

**Figure 2:**
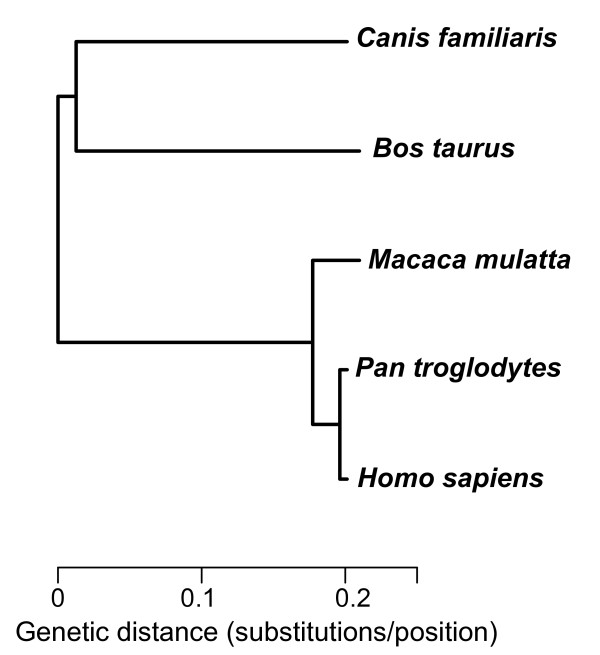
**Maximum-likelihood reference tree representing the global genomic evolution**. The root of the tree was placed at the midpoint.

Next, we eliminated introns that were too accelerated or decelerated in some lineages (and that could have been affected by changes in evolutionary processes in particular lineages such as a change of function, a change to a chromosome position with different evolutionary dynamics, alternative splicing, etc). That is, we selected introns whose rates of evolution were similar to the global genomic rate and therefore with a largely neutral molecular clock in mammals. Furthermore, trees with large branches in particular lineages may correspond to hidden paralogues that may have remained undetected up to this point. These paralogues are very problematic for estimations of evolutionary rates and other parameters necessary for their use in phylogenetic analyses. To detect this type of introns, we first constructed a global genomic tree from the concatenation of all introns available at this step (Figure 2). This tree was then used as a reference to assess if the phylogenetic tree of each individual intron had a rate of evolution similar to the global one in every lineage. This calculation was performed with the K tree score measured by the Ktreedist software, which reflects the topological and relative differences in branch length between a given tree and a reference tree [27]. That is, a high K tree score is indicative of a tree that has some highly accelerated or decelerated branches with respect to the reference tree regardless of the overall tree divergence. This score is also influenced by wrong topologies when the affected branches are large. By setting an arbitrary K tree score limit of 0.1 we removed approximately 34% of the introns that were most different in shape from the global genomic tree.

The next step was designed to eliminate the most conserved introns, which could be involved in some function and are therefore not variable enough for the phylogeny of closely related species. To do this, we employed two different measures of divergence. First, we calculated with the Ktreedist software the scaling factor from each intronic phylogenetic tree to the global genomic tree. This measure allowed us to discard the introns that showed the slowest overall evolutionary rates. Second, we calculated another measure of divergence, the primates total branch length, by using the corresponding alignments and trees with only the three primate species (human, chimpanzee and macaque). This measure is more accurate and less affected by alignment imperfections possibly generated when comparing sequences that are too divergent. We then selected the introns that had a scaling factor lower than 1.15 and a primates total branch length higher than 0.075 substitutions per position. This rendered a dataset of 225 introns that excluded the most conserved ones.

Finally, a visual inspection step was performed to detect any poorly aligned sequences caused by wrong annotations or clearly incorrect orthology assignments. Only one intron had to be eliminated in this step. The resulting final set was thus composed of 224 new phylogenetic markers (Figure 1). In the additional file 1 we show all relevant information for each marker. This includes the alignments of both the intron and the flanking exons, the phylogenetic tree of the five mammals constructed with the intron sequences, and the genomic location and function of the gene to which the intron belongs. The examination of this information allows the selection of optimal markers for specific purposes and the design of exon primers with different degrees of specificity. Interestingly, one of the introns in our final set turned out to be intron 1 of transthyretin (TTR-1), which is one of the most widely used introns in mammalian phylogenetics [44-48]. To our knowledge, no other markers in our dataset have been used so far.

### Analysis of genomic features of the new set of introns

**Figure 3:**
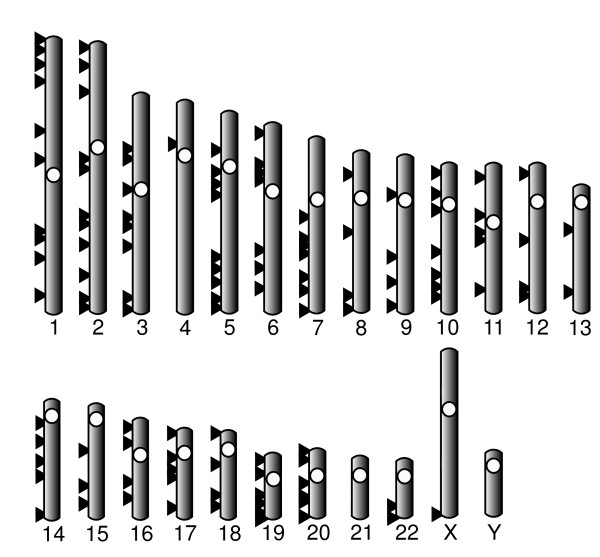
Human karyotype showing the genomic location of the genes to which the 224 introns of the final set belong

The genomic location in *Homo sapiens *of each new marker is represented in Figure 3. All the human chromosomes carry at least one intron in our set, except chromosomes 21 and Y. The latter chromosome was expected to be missing from our set because no sequence for this chromosome was present in the available genome sequences of macaque, cow and dog. Regarding the X chromosome, only one intron was present in the final set. The rest of X-linked introns were discarded in the different filtering process, mainly in the single-copy test and divergence filters steps.

Repetitive sequences present in the intron set were analyzed with the RepeatMasker software. Of the 224 introns, 163 were found to contain repetitive elements. The most frequent elements were SINEs, which were present in 70% of the introns that bore any kind of element, followed by LINEs, which were found in 26% of them. Introns with repeats had, on average, 18% of their sequence corresponding to repetitive elements. These types of repetitive sequences normally evolve in a neutral way, mostly by point mutations, and therefore their presence is not a problem for sequence-based analysis methods that assume a normal nucleotide substitution model. If all species in the alignment have the repeat (which is the normal situation in closely related species) this fragment can be used normally as any other sequence. When only some species have the repetitive element (as it happened often in our set of five mammalian species), we observed that alignment programs do not have problems dealing with these repetitive elements, and therefore the alignments can also be used for further sequence or phylogenetic analyses.

More problematic are microsatellites (defined here as two or more contiguous, approximate copies of a pattern of 1 to 6 nucleotides [49]). Since microsatellites evolve by a slippage mechanism instead of by point mutations [50], they cannot be used with phylogenetic or coalescence methods that assume a normal point substitution model. We checked for the presence of microsatellites with the Tandem Repeats Finder program and found that 43 of the 224 introns had at least one microsatellite in some species. Microsatellites are, in general, specific for particular lineages, and their presence in one species does not mean that the intron cannot be used in other taxa. Therefore, introns with some microsatellites were not eliminated from the data set. However, when these sequences are found within the introns in a species to be studied it may be better not to use this intron and rather select a new one from the set designed here. Alternatively, the positions belonging to the microsatellites can be eliminated from the alignment for further processing with sequence analysis programs that assume a point mutation mechanism of evolution.

### Analysis of genetic distances and single nucleotide polymorphisms

**Figure 4:**
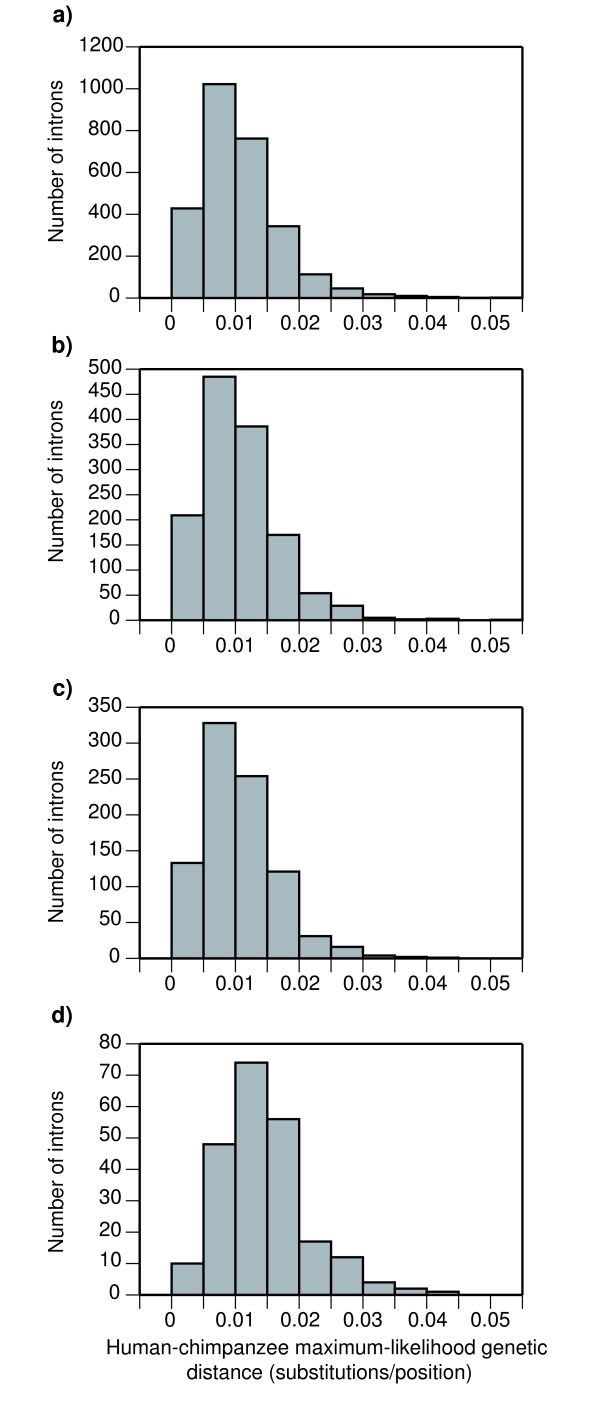
**Human-chimpanzee genetic distances measured in the primates tree**. The different sets correspond to: a) 2750 orthologous, size-constrained introns; b) 1344 single-copy introns; c) 890 orthologous introns of neutral evolution; and d) the final set of 224 introns.

In order to test the degree of variability of the 224 final introns with respect to the initial data sets, we estimated the genetic distance between human and chimpanzee for each intron. To do this, we constructed the alignments of the primate species (human, chimpanzee and macaque), which could be used without further cleaning. Then, we estimated the corresponding maximum-likelihood trees and measured the patristic distances between human and chimpanzee. We then compared the distances obtained for the final set of 224 introns with the sets corresponding to the different filtering processes (Figure 4). The mean divergence between human and chimpanzee was around 0.011 substitutions/position in the three initial data sets: the size-constrained introns set, the single-copy gene introns set, and the set obtained after the application of the neutral evolution test. As expected, after the application of the divergence filter this distance increased, but only up to 0.014 substitutions/position. Thus, the overall gain in genetic distance in the final set was small. However, the main effect of the divergence filters was the elimination of highly conserved introns (as reflected in the relative decrease of the first bar of the histograms in Figure 4d), which could be involved in some important function and would be practically useless for studies of closely related species. Similarly, the analysis of human single nucleotide polymorphisms (SNPs) in the introns of our final set revealed that they had 4.35 SNPs on average. In comparison, the set of 2750 size-constrained orthologous introns (see Figure 1) contained 3.19 SNPs on average. When scaling these results by the length of each intron to obtain the SNP density, the difference between the means was still maintained: 0.0055 SNPs per nucleotide for the final set of introns *versus *0.0043 for the initial set. Thus, human SNPs also showed a slight increase in the genetic variability of the introns in the final set.

To compare the mean intronic genetic distance with the divergence of cytochrome *b*, which is the most popular marker for mammalian phylogenetics [51], we obtained the cytochrome *b *distance between human and chimpanzee. To do this, we measured the patristic distance in a maximum-likelihood phylogenetic tree similarly reconstructed with human, chimpanzee and macaque cytochrome *b *sequences obtained from GenBank [52]. The resulting distance was 0.169 substitutions/position, which implies that the final set of 224 introns has, on average, 12.1 times less divergence than the mitochondrial cytochrome *b *gene. Furthermore, alignments containing the exons flanking the 224 introns were constructed for the three primates, maximum likelihood trees were built as above, and the resulting trees were used to calculate the distance between *Homo sapiens *and *Pan troglodytes *for both the upstream and downstream exons of each intron. The resulting mean genetic divergence was 0.006 substitutions/position, which implies that the 224 selected introns have, on average, 2.3 more divergence than their corresponding flanking exons.

### Experimental validation of the newly developed phylogenetic markers: Primer design and PCR amplification

The intron filtering processes carried out above were designed to select a set of optimal introns for the phylogeny of closely related mammalian species. However, many unidentified factors may affect the amplification of these introns in different species. To experimentally test the real performance of these introns and the validity of the designed bioinformatic analysis we randomly selected twelve introns among the largest ones in the final data set for sequencing in different mammals.

In order to design primers of wide spectrum, the flanking exonic sequences already gathered (human, chimpanzee, macaque, cow and dog) were complemented by (whenever possible) a few others from the ENSEMBL database: horse (*Equus caballus*), Sumatran orangutan (*Pongo abelii*), little brown bat (*Myotis lucifugus*), European hedgehog (*Erinaceus europaeus*), domestic cat (*Felis catus*), Northern tree shrew (*Tupaia belangeri*), gray mouse lemur (*Microcebus murinus*), African bush elephant (*Loxodonta africana*), lesser hedgehog tenrec (*Echinops telfairi*), nine-banded armadillo (*Dasypus novemcinctus*) and common shrew (*Sorex araneus*). These genomes mostly corresponded to low-coverage genome projects but many of them were valid for certain exons. Exonic alignments, containing between 11 and 15 different species, were constructed. These alignments allowed us to design the 3' end of every primer in the most conserved part of the exonic alignment. Degenerate bases (up to a limit of 48 per primer) were used to make the primers suitable for as many different mammalian species as possible.

To test these primers, we used genomic DNA extracted from six mammalian species: Iberian mouse-bat (*Myotis escalerai*), Bornean orangutan (*Pongo pygmaeus*), snow leopard (*Uncia uncia*), tiger (*Panthera tigris*), least weasel (*Mustela nivalis*), and European polecat (*Mustela putorius*). This set included several closely related species in order to analyze their intronic divergence. Table 1 shows the primers used and the amplification results for this panel of species. Five out of the 12 introns failed to produce a single, clean PCR band of the expected size in one or more of the six analyzed species. This was due to several reasons, the main one being PCR misamplification in the form of gel smear. Some other problems were the excessive length of the amplified band in some species or the generation of double bands in the PCR reaction, which can reflect the existence of gene duplications in a particular lineage (Table 1). Some optimizations with different primers or hybridization temperatures did not solve the problems in these introns. The seven remaining introns did produce a clear single PCR band of the expected size in the six chosen species. It should be noted that, even for the successful primers, the annealing temperature had to be optimized for different species. Moreover, there were a few cases, namely SLC38A7-8 for *Panthera tigris *and CARHSP-1 and PNPO-3 for *Myotis escalerai*, where more specific primers had to be designed to improve the PCR band. The PCR bands of these introns were subsequently sequenced, and the sequences were compared to the known introns, which confirmed their correct amplification. In addition to the sequence of the PCR band, the exact sequence of one allele for each specimen was obtained by cloning the sequenced PCR product in a plasmid vector.

**Table 1:** Introns selected for amplification and sequencing in six species, and final outcome

Intron name	ENSEMBL Code	Length (*H. sapiens*)	Primer sequences (Forward and Reverse)	Result
**SLC38A7-8**	ENSG00000103042	877	RGGCCTRGCYGSCTGCTTCATCTT TCVGASAGYTTGGCTTGRATGAGGCA	+
**COPS7A-4**	ENSG00000111652	952	TACAGCATYGGRCGRGACATCCA TCACYTGCTCCTCRATGCCKGACA	+
**CARHSP1-1**	ENSG00000153048	698	ACYCGCCGSACSAGGACCTTCT GTRATGAAGCCRTGGCCCTTGGA	+
**GAD2-1**	ENSG00000136750	724	GGCTCHRGCTTYTGGTCYTTYGG YCCGAKGCCKCCSGTGAACTTCT	+
**JMJD5-2**	ENSG00000155666	1124	ACCABTGGCCVTGCATGMAGARGT TGATGAACTCRYTGACBGTCATGAG	+
**OSTA-5**	ENSG00000163959	535	TGMWGGYCATGGTGGAAGGCTTTG AGATGCCRTCRGGGAYGAGRAACA	+
**PNPO-3**	ENSG00000108439	843	GATGGCTTCCRHTTCTWCACTAACTT GGYTCCCARTAGAAGACMAKSGA	+
**GAD2-3**	ENSG00000136750	1051	TGCTCTAYGGRGAYKCMGAGAAG CAGAAACGCCARMGTGGSCCTTT	(1)
**CLCN6-17**	ENSG00000011021	1376	GTGGCCAAATGGACAGGGGACTTT TTGCCCTTCATGAACTCCTTCTCGT	(2)
**TFPI2-2**	ENSG00000105825	913	TACTACTAYGACAGGYACWYGCAGA CATGTCATRGAWSTTAGATTRAAGAA	(2)
**CSE1L-12**	ENSG00000124207	1496	CATGGRATYACAMAAGCWAATGA TAYTTRATRCCRTCAGCTTTAAG	(3)
**TBC1D21-8**	ENSG00000167139	1007	TCTTYCCCTGGTTCTGYYTCTGCTT CAKGCWGTAGGCCACCAGCACCT	(4)

The intron name is taken from the gene name according to the HUGO Gene Nomenclature Committee followed by a dash and the intron number. In the "Result" column, the (+) symbol indicates successful sequencing in the whole panel of species; (1), non-specific amplification in one species; (2), non-specific amplification in several species; (3), intron of very large size in some species; (4), double PCR band in some species.

We can conclude that a large part of the markers tested were valid, with a minimum optimization, for a wide variety of mammals. Of course, the success rate will vary depending on the taxa of choice, but, to increase the chances that a primer works, it is important to take into account that this optimization may be necessary in all pilot studies and may include the adjustment of the hybridization temperature or the modification of the primer specificity with a different degree of degeneracy. In our experimental test of selected introns, we designed primers of broad specificity so that they could be used in a wide range of mammalian groups, but primers intended for specific taxa need not be based on such a variety of species and can have less degenerate bases. In addition to the species tested here, selected to include pairs of closely related species, we have successfully amplified other intron markers from our data set in other mammals, mainly belonging to Erinaceomorpha and Soricomorpha, as part of ongoing studies. They also produced the expected PCR band and the corresponding sequence, indicating again the usefulness of this set of introns.

### Use of the selected introns as markers for the phylogeny of closely related species

The sequenced introns were added to the introns already downloaded for human, chimpanzee, macaque, dog and cow, and this set was complemented by three additional genomes that had information for the seven successful introns: the Sumatran orangutan (*Pongo abelii*), the horse (*Equus caballus*) and the little brown bat (*Myotis lucifugus*). This extended species set allowed us to assess the variability of our introns in five pairs of closely related mammalian species, which comprised four different mammalian families: Mustelidae, which included *Mustela nivalis *and *M. putorius*, with an estimated divergence time of 2.8 million years [53]; Felidae, with *Panthera tigris *and *Uncia uncia*, which diverged 2.9 million years ago [54]; Hominidae, which included two pairs, namely, *Pongo pygmaeus *and *P. abelii*, with 3.8 million years of estimated divergence [55], and *Homo sapiens *and *Pan troglodytes*, with 6 million years of estimated divergence [56]; and Vespertilionidae, with *Myotis lucifugus *and *M. escalerai*, which diverged 12.2 million years ago [57].

All of the sequenced alleles for the seven introns were different in both members of every pair of closely related species, except, surprisingly, the PNPO-3 sequence obtained for *Pongo pygmaeus*, which was identical to the one downloaded from ENSEMBL for *P. abelii*. This can be due to several reasons such as a recent introgression, the existence of a constraint in the evolution of this particular marker in the orangutan lineage or the fact that, by mere chance, no mutations have accumulated in any of the two species since their recent divergence. All other intron pairs showed one or several substitutions between the species pairs selected, thus providing useful information for phylogenetic reconstruction.

**Figure 5:**
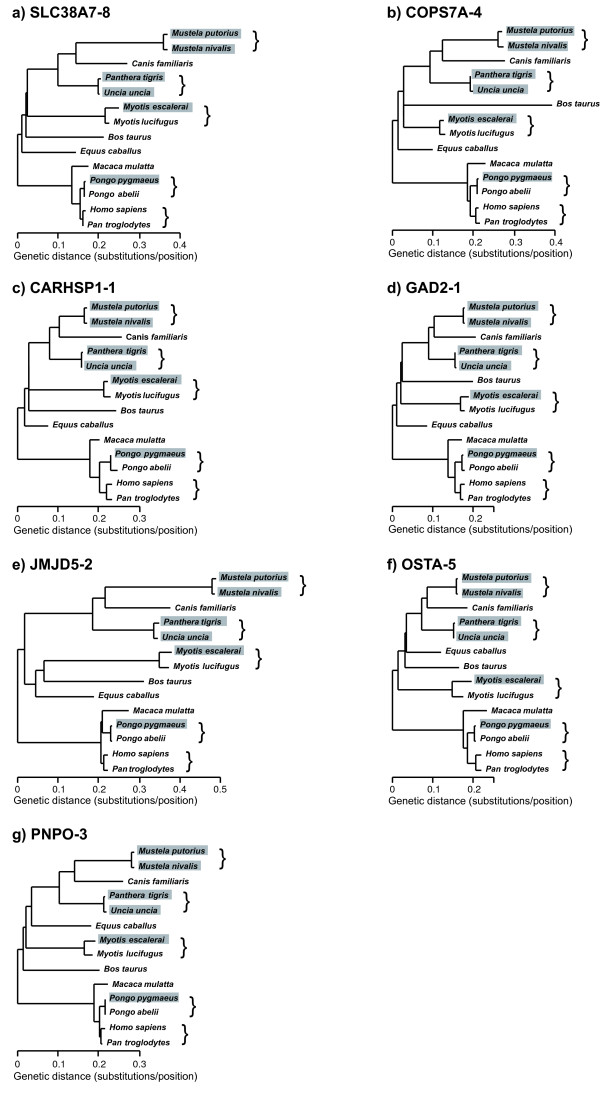
**Maximum-likelihood phylogenetic trees of seven selected introns**. Grey boxes indicate the new sequences obtained in this study and brackets show the pairs of closely related species specifically analyzed. The root was placed at 7% of the branch separating primates and laurasiatherians (coinciding with the midpoint of the global genomic tree). All trees are drawn at the same scale.

Alignments and the corresponding maximum-likelihood phylogenetic trees were reconstructed for each of the seven intronic markers (Figure 5). The phylogenies obtained were largely congruent with the known taxonomy of the species, particularly within each order, showing that these introns have normal evolutionary dynamics, not only in the five species used for filtering the introns, but also in other mammals. In addition, it is interesting to note that we have four representative orders (Carnivora, Chiroptera, Cetartiodactyla and Perissodactyla) within laurasiatherians that could help resolve the phylogeny of this group. However, the relationships among them were different in each tree, and the bootstrap values for the different clades were very low (results not shown), indicating that individual introns may not have enough information for inter-ordinal mammalian phylogenies [58-62]. It is also important to observe that the trees estimated with the different markers show very apparent differences in their overall divergence. As can be seen from the scale of the trees, the most extreme examples were GAD2-1 and OSTA-5, which were the slowest introns, and JMJD5-2, which was the intron with the fastest rate of evolution.

**Figure 6:**
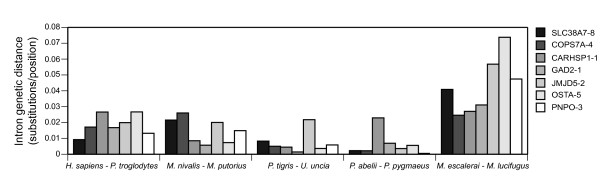
Pairwise maximum-likelihood distances of the different introns for the selected species pairs

To analyze the differences in divergence among introns in more detail, we constructed new alignments for each pair of closely related species to avoid possible problems associated with the alignments of more divergent taxa. We then estimated the intron genetic distance between species pairs from the corresponding intron pairwise alignment. Figure 6 compares the divergences between species pairs for the different introns. As expected, species pairs separated for longer times such as the two *Myotis *species accumulate an overall higher number of substitutions than the closest species such as *Panthera tigris *and *Uncia uncia*. It can also be deduced from this figure that there are important differences in intron divergence in different species pairs, indicating that it is not possible to predict that one intron with a high number of variable positions in one lineage will be equally variable in other lineages. For example, the most informative introns for *H. sapiens *and *P. troglodytes *would be CARHSP1-1 and OSTA-5 whereas for *Mustela *the best ones would be SLC38A7-8 and COPS7A-4. Furthermore, the intron JMJD5-2, despite being the fastest one according to the overall divergence in mammals (Figure 5), was not always the most variable between sister species. Therefore, the most variable introns are different for every lineage. This can be due to the stochasticity of mutations, which can specially affect to short branches, and to differences in population size and ancestral polymorphisms, which may constitute a large part of the divergence among genes in closely related species [13,63]. Thus, given the range of intron variability of our data set (with all introns being quite variable) it may not be worthwhile favoring introns with high overall rates in mammals. Rather, it may be better to use several unlinked introns to overcome different stochastic processes in any phylogenetic study of closely related species.

## Conclusions

The development of multilocus phylogenetics requires the availability of a large number of adequate markers for different taxa. In this study, we have designed 224 intronic markers with optimal conditions for the phylogeny of closely related mammalian species (excluding rodents, marsupials and monotremes). Among the important criteria used to select these markers were that they belonged to single-copy genes, were not highly conserved, and did not show disparate rates of evolution in different lineages. The experimental validation of these introns showed that, after some optimizations, they could be amplified in different mammalian species, yielding a single PCR band. In addition, an analysis of the genetic distances estimated in several pairs of closely related mammalian species revealed that introns may show different degrees of divergence in different pairs, and that it is not possible to predict which introns will be more variable in each group. In any case, it may be necessary to carry out initial pilot studies with several introns to decide which ones perform best for each species or group of closely related species. In addition, the use of several introns will reduce the stochasticity associated to mutational and coalescence processes, which is particularly large for nuclear sequences. The availability of a large set of introns like the one provided here will greatly facilitate this work.

## Methods

### Databases and filters

The following mammalian genomes were downloaded from the ENSEMBL database [64] in GenBank format: human (*Homo sapiens*) version NCBI 36 [36,39], chimpanzee (*Pan troglodytes*) version Pan_troglodytes-2.1 [35], rhesus macaque (*Macaca mulatta*) version Mmul_1 [38], dog (*Canis familiaris*) version CanFam2.0 [37] and cow (*Bos taurus*) version Btau_3.1 [34]. These genomes are sequenced with high coverage and thoroughly annotated. There were also several low-coverage genomes available, but their lack of many annotated features made their use in studies like the present one very inconvenient [64,65]. The genome of the marsupial *Monodelphis domestica *[66] was also available and well annotated at the time this study was performed but we decided not to include it to avoid problems with alignments and phylogenetic reconstruction due to its large distance from the other mammalian species used. In addition, the mouse and rat genomes, which are also very divergent due to their peculiar genomic features [40,41], were also excluded from this study.

We developed a pipeline of Perl scripts to extract all the introns and exons of each mammalian genome (Figure 1). At the same time, features like the gene description and the genomic location and length of every intron and exon were stored. In this initial step, introns of more than 50,000 nucleotides were discarded.

The ENSEMBL database includes orthology information obtained from a phylogenetic analysis, which makes the determination of the orthology relationships more accurate [64]. Therefore, we used this information to determine preliminary one-to-one orthologous genes between human, chimpanzee, macaque, dog and cow. To achieve this, we downloaded orthology tables containing the whole list of one-to-one orthologous genes between pairs of species. By crossing the information of the human-chimpanzee and human-macaque pairs we obtained a set of one-to-one orthologous genes for primates. From the table of dog and cow we obtained the corresponding set of orthologues for laurasiatherians. Finally, we crossed these two tables to obtain a set of putative one-to-one orthologous genes for the five considered species. The corresponding lists of introns were then assembled from this table. In addition, only those introns belonging to genes with equal numbers of introns and whose relative position inside the corresponding gene was conserved in every compared species were considered to be true one-to-one orthologues.

The size variation of each intron across the studied species was also controlled. To do this, the maximum difference in length allowed between human and chimpanzee, human and macaque, human and cow, human and dog, and cow and dog was established at 10%, 20%, 100%, 100% and 50%, respectively.

Multiple alignments of the orthologous intronic sequences were built using MAFFT version 5.8 [67]. Gblocks version 0.91 [68] was used with relaxed parameters [69] to discard poorly aligned positions. Maximum-likelihood phylogenetic trees were reconstructed using PhyML version 2.4.4, with the GTR model of evolution and four substitution rate categories [70].

To detect duplicated genes, BLAST searches [71] of the pair of flanking exons were performed for every intron. For each species, the exons were used as query against its own genome. The e-value limit was set at 10^-4^. We selected only the introns with exons that produced a single hit (itself) against one region of the genome. Since each genome is subdivided into large fragments, normally chromosomes, we checked that every single hit was also composed of a single subalignment and therefore corresponded to a single exon.

A reference tree reflecting the global genomic evolution was constructed using all single-copy introns. To do so, the 1344 intron alignments that passed the BLAST filter were concatenated to generate an alignment of 768,745 positions. This alignment was used to build a maximum-likelihood phylogenetic tree using RaxML version 2.4.4, which can handle big alignments [72]. Phylogenetic trees of every individual intron were compared to this reference tree using the K tree score implemented in the Ktreedist program [27].

Repetitive sequences present in the final set of introns were analyzed with the program RepeatMasker version 3.2.8 using the corresponding library for each species [73]. The intron sequences were also scanned for microsatellites with Tandem Repeats Finder version 4.04 [49] using the following program parameters: 2 7 7 80 10 50 6. Moreover, the presence of human SNPs in these introns was analyzed using information obtained from the ENSEMBL database. To do this, the whole list of annotated intronic SNPs of the NCBI 36 version of the *Homo sapiens *genome was downloaded using the Biomart platform [74], and the corresponding SNPs were mapped in our set of introns.

### Samples and laboratory procedures

Samples of six mammalian species spanning different orders were obtained. The chosen species were the Iberian mouse-bat (*Myotis escalerai*); the Bornean orangutan (*Pongo pygmaeus*), obtained from the INPRIMAT Consortium and the Biomedical Primate Research Centre in Rijswijk; the snow leopard (*Uncia uncia*) and the tiger (*Panthera tigris*), obtained from the Animal Tissue Bank of Catalunya; and the least weasel (*Mustela nivalis*) and the European polecat (*Mustela putorius*), obtained from Collection of Tissues and DNA of the Museo Nacional de Ciencias Naturales.

DNA extractions were performed using the DNEasy Blood and Tissue Kit (QIAGen), following manufacturer's instructions. PCR reactions were carried out in 25 μl of final reaction volume, containing 50 - 100 ng of template DNA, 0.5 - 1 μM of each primer, and 0.75 units of Promega GoTaq. Amplification conditions were as follows: initial denaturation at 95°C for 5 minutes, followed by 28 to 35 cycles comprising 30 seconds of denaturation at 95°C, 30 seconds of annealing at 52-65°C (this temperature turned out to be highly variable among different species and introns and had to be adjusted for each particular case) and extension at 72°C for 30-60 seconds. The resulting PCR products were loaded in 1% agarose gels stained with SYBR Safe DNA Gel Stain (Invitrogen). Bands were manually excised from the gel and subsequently purified using Illustra GFX PCR and DNA Gel Band Purification Kit (GE Healthcare). The resulting products were then sequenced using different sequencing services. Both heterozygous point mutations and indels that caused difficulties in assigning a clear sequence to each allele of the individuals were observed in several sequences. Therefore, we cloned each PCR product into the pstBlue-1 vector (Invitrogen) to be able to isolate one of the alleles. The plasmid containing one of the alleles was isolated from the bacterial cultures, purified using the GenElute Plasmid Miniprep Kit (Sigma Aldrich), and subsequently sequenced. Resulting sequences were analyzed using Geneious version 4.5.5 [75]. To correct the errors induced by the polymerase and evidenced in the cloning process, both the PCR product and its corresponding cloned sequence were assembled. Intron sequences have been deposited in GenBank under accession numbers HM147892-HM147933.

### Phylogenetic analyses of the new markers

For each successfully amplified and sequenced intron, a new multiple sequence alignment was created containing the six newly obtained sequences plus further sequences from ENSEMBL. As above, MAFFT was used to build these alignments, which were then cleaned using Gblocks with the same parameters as above. We constructed the phylogenetic trees using PhyML with the GTR model of evolution and four substitution rate categories. In addition, for each of these introns we analyzed the divergence for five pairs of closely related species. To do this, we constructed new pairwise intronic alignments for each species pair using MAFFT as above, but without applying Gblocks, and estimated maximum-likelihood distance using PAUP [76] with the GTR substitution model.

## Authors' contributions

JC and JI conceived and designed the experiments. JI did the experimental work and the main analyses. JJ contributed to the original design of the problem, participated in discussions and collaborated with samples. JC and JI wrote the paper. All authors reviewed and approved the final manuscript.

## Supplementary Material

Additional file 1 **Final set of 224 introns selected for the phylogeny of closely related mammalian species**. The first page of each intron lists the following information: description (function of the gene to which the intron belongs), gene name according to the HUGO Gene Nomenclature Committee (in parenthesis), intron number, chromosome where it is located in *Homo sapiens*, intron start (the location of the first base of the intron in the corresponding human chromosome), human intron length, intron alignment length (size of the alignment of the five species: human, chimpanzee, rhesus macaque, dog and cow), flanking exons length (the size of both the upstream and the downstream exons that flank this intron in *Homo sapiens*), SNP density (the number of single nucleotide polymorphisms described for human in this intron divided by its length), K tree score (a calculation that reflects topological and rate divergence of the intronic tree with respect to the genomic reference tree), scaling factor (the value that shows the global divergence of the intronic tree with respect to the genomic reference tree), human-chimpanzee distance in substitutions per position (the maximum-likelihood distance between the introns of *Homo sapiens *and *Pan troglodytes *measured in the primates phylogenetic tree), and total primate branch length in substitutions per position (the sum of all the branches of the corresponding phylogenetic tree built using only the three available primate species). For the representation of the exon and intron alignments, a few wrong definitions of intron ends and starts that we found were manually corrected. The five-species alignments of both upstream and downstream exons are represented, but only the 160 bases closest to the intron are displayed. To help assess sequence conservation, positions with more than 50% identities are highlighted. The maximum-likelihood phylogenetic tree of the intron for the five species is also shown. The root was placed at 7% of the branch separating primates and laurasiatherians (coinciding with the midpoint of the global genomic tree). The second page of each intron shows the five-species alignment of the intron. Introns are ordered in this document by total primate branch length. Click here for file
