# Supplementary material for: Novel intron markers to study the phylogeny of closely related mammalian species
Source: BMC Evol Biol. 2010 Nov 30;10:369. doi: 10.1186/1471-2148-10-369 (PMC3087552; doi:10.1186/1471-2148-10-369)
Supplement: Additional file 1 — Final set of 224 introns selected for the phylogeny of closely related mammalian species. The first page of each intron lists the following information: description (function of the gene to which the intron belongs), gene name according to the HUGO Gene Nomenclature Committee (in parenthesis), intron number, chromosome where it is located in Homo sapiens, intron start (the location of the first base of the intron in the corresponding human chromosome), human intron length, intron alignment length (size of the alignment of the five species: human, chimpanzee, rhesus macaque, dog and cow), flanking exons length (the size of both the upstream and the downstream exons that flank this intron in Homo sapiens), SNP density (the number of single nucleotide polymorphisms described for human in this intron divided by its length), K tree score (a calculation that reflects topological and rate divergence of the intronic tree with respect to the genomic reference tree), scaling factor (the value that shows the global divergence of the intronic tree with respect to the genomic reference tree), human-chimpanzee distance in substitutions per position (the maximum-likelihood distance between the introns of Homo sapiens and Pan troglodytes measured in the primates phylogenetic tree), and total primate branch length in substitutions per position (the sum of all the branches of the corresponding phylogenetic tree built using only the three available primate species). For the representation of the exon and intron alignments, a few wrong definitions of intron ends and starts that we found were manually corrected. The five-species alignments of both upstream and downstream exons are represented, but only the 160 bases closest to the intron are displayed. To help assess sequence conservation, positions with more than 50% identities are highlighted. The maximum-likelihood phylogenetic tree of the intron for the five species is also shown. The root was placed at 7% of the branch separating p [file 1471-2148-10-369-S1.PDF]

Additional file 1 – Final set of 224 introns selected for the phylogeny of closely related mammalian species

The first page of each intron lists the following information: description (function of the gene to which the intron belongs), gene name according to the HUGO Gene Nomenclature Committee (in parenthesis), intron number, chromosome where it is located in *Homo sapiens*, intron start (the location of the first base of the intron in the corresponding human chromosome), human intron length, intron alignment length (size of the alignment of the five species: human, chimpanzee, rhesus macaque, dog and cow), flanking exons length (the size of both the upstream and the downstream exons that flank this intron in *Homo sapiens*), SNP density (the number of single nucleotide polymorphisms described for human in this intron divided by its length), K tree score (a calculation that reflects topological and rate divergence of the intronic tree with respect to the genomic reference tree), scaling factor (the value that shows the global divergence of the intronic tree with respect to the genomic reference tree), human-chimpanzee distance in substitutions per position (the maximum-likelihood distance between the introns of *Homo sapiens* and *Pan troglodytes* measured in the primates phylogenetic tree), and total primate branch length in substitutions per position (the sum of all the branches of the corresponding phylogenetic tree built using only the three available primate species). For the representation of the exon and intron alignments, a few wrong definitions of intron ends and starts that we found were manually corrected. The five-species alignments of both upstream and downstream exons are represented, but only the 160 bases closest to the intron are displayed. To help assess sequence conservation, positions with more than 50% identities are highlighted. The maximum-likelihood phylogenetic tree of the intron for the five species is also shown. The root was placed at 7% of the branch separating primates and laurasiatherians (coinciding with the midpoint of the global genomic tree). The second page of each intron shows the five-species alignment of the intron. Introns are ordered in this document by total primate branch length.

Novel intron markers to study the phylogeny of closely related mammalian species. Javier Igea, Javier Juste and Jose Castresana

ENSG00000101194 intron 5

Description: Solute carrier family 17 member 9 (SLC17A9)
Intron number: 5
Human chromosome: 20
Intron start (bp): 61064552
Human intron length : 518
Intron alignment length: 627
Flanking exons length (upstream/downstream): 131/97
SNP density: 0.007722
K tree score: 0.0804
Scaling factor: 0.4564
Human-chimpanzee distance: 0.005884
Total primate branch length: 0.2152

ENSG00000101194 exon 5

```
Homo    GACGCTGCTGACCGGGGCGGTGGGCTCCCTGCTCCTGGAATGGTACGGCTGGCAGAGCATCTTCTATTTCTCCGGCGGCC 80
Pan     GACGCTGCTGACCGGGGCGGTGGGCTCCCTGCTCCTGGAATGGTACGGCTGGCAGAGCATCTTCTATTTCTCCGGCGGCC 80
Macaca  GACGCTGCTGACCGGGGCAGTGGGCTCCCTGCTCCTGGAATGGTACGGCTGGCAGAGCGTCTTCTATTTCTCCGGCGGCC 80
Bos     GACACTGGTGACCGGGGCTGTGGGCTCCCTGCTCCTGGACTGGTACGGCTGGCCGAGTGTCTTCTACTTTTCGGGTGGAC 80
Canis   GACGCTGGTGACGGGGGCCGTGGGCTCCCTGCTCCTGGACTGGTACGGCTGGCCGAGCGTCTTCTACTTCTCCGGCGGGC 80

Homo    TCACCTTGCTTTGGGTGTGGTACGTGTACAGGTACCTGCTGAGTGAAAAAG 131
Pan     TCACCTTGCTTTGGGTGTGGTACGTGTACAGGTACCTGCTGAGTGAAAAAG 131
Macaca  TCACCTTGCTTTGGGTGTGGTACGTGTACAGGTACCTGCTGAGTGGAAAAG 131
Bos     TCACCCTGCTGTGGGTGGGTTACGTGTACAGGTGTCTCCTGAGTGAGAGAG 131
Canis   TCACCCTGCTGTGGGTGTGTTACGTGTACAGGTACCTGCTCACTGGAAAAG 131
```

ENSG00000101194 exon 6

```
Homo    ATCTCATCCTGGCCTTGGGTGTCCTGGCCCAAAGCCGGCCGGTGTCCAGGCACAACAGAGTCCCCTGGAGACGGCTCTTC 80
Pan     ATCTCATCCTGGCCTTGGGTGTCCTGGCCCAAAGCCGGCCGGTGTCCAGGCACAGCAGAGTCCCCTGGAGACGGCTCTTC 80
Macaca  ATCTCGTCCTGGCCTTGGGAGTCCTGGCCCAAGGCCTGCCGGTGTCCAGGCACAACAGAGTCCCCTGGAGACGGCTCTTC 80
Bos     ATCTCATCCTGGCCCTGGGCATCCTGGCGCAAGGCCTGCCCGTGTCTAGACACACCAAGGTGCCCTGGAGACAGCTCTTC 80
Canis   AGCTCATCCTGGCCTTGGGCGTTCTGGCGCAAGGCCTGCCGGTGTCCAGGCACACCAAGGTTCCCTGGAGACAGCTCTTC 80

Homo    CGGAAGCCTGCTGTCTG 97
Pan     CGGAAGCCTGCTGTCTG 97
Macaca  CGGAAGCCTGCTGTCTG 97
Bos     CGAAAGCCTTCTGTCTG 97
Canis   CGGAAGCCTTCCGTCTG 97
```

ENSG00000101194 intron 5

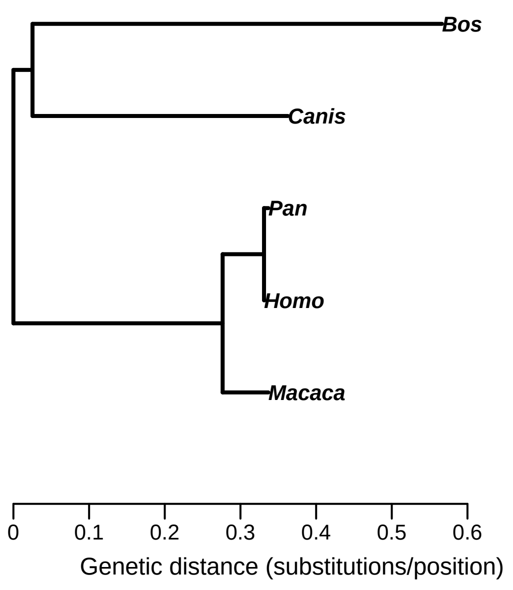

ENSG00000101194 intron 5

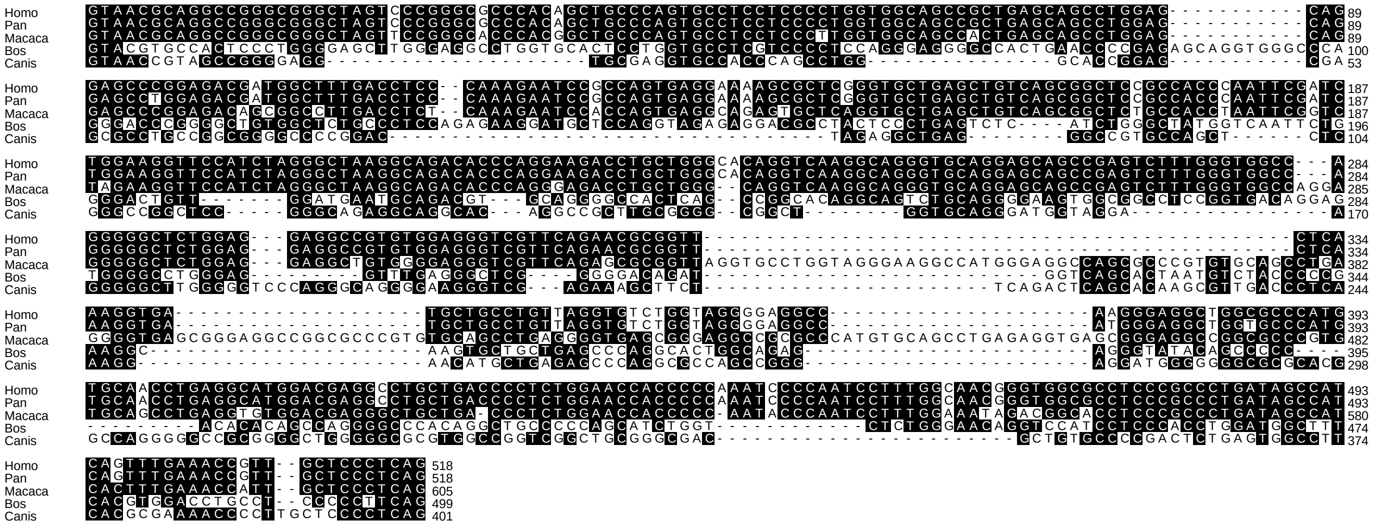

ENSG00000124602 intron 2

Description: unc-5 homolog C-like (UNC5CL)
Intron number: 2
Human chromosome: 06
Intron start (bp): 41108864
Human intron length : 734
Intron alignment length: 958
Flanking exons length (upstream/downstream): 301/263
SNP density: 0.000000
K tree score: 0.076
Scaling factor: 0.6067
Human-chimpanzee distance: 0.008836
Total primate branch length: 0.2105

ENSG00000124602 exon 2

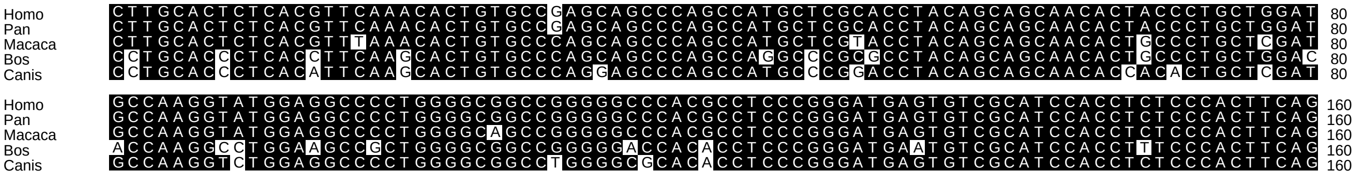

ENSG00000124602 exon 3

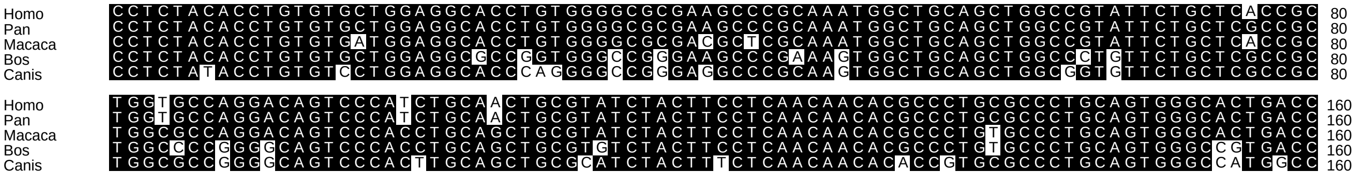

ENSG00000124602 intron 2

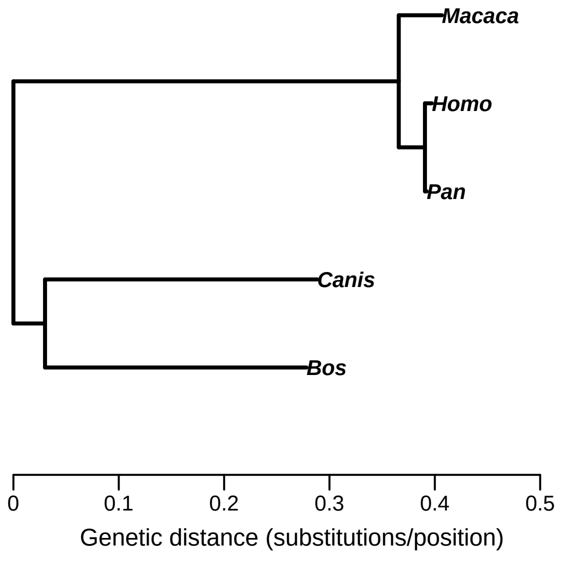

ENSG00000124602 intron 2

```
Homo    GTAGGCACATGCCATTGGCCTGTCTTCCCACACCCATCTCTCTGAGGACTGTCTTTCCTGCCCACTTTCTCTGTCTGCCTTATCTTCATCTCTCTGTATA 100
Pan     GTAGGCACATGCCATTGGCCTGTCTTCCCACACCCATCTCTCTGGGGACTGTCTTTCCTGCCCACTTTCTCTGTCTGCCTTATCTTCATCTCTCTGTATA 100
Macaca  GTAGGCACATGCCATTGGCCTGTCTTCCCACACCCATCTCTCTGGGGACTGTCTTTCCTGCCCACTTTCTCTGTCTGCCTTATCTTCAGCTCTCTGTATA 100
Bos     GTGGGCACCCACCTGTGGGC----------------------------CTGGACTTCCCGCCCCCTGTCTCTGCCTGCCTCGTCCTCACCTCTCTGCG-G 71
Canis   GTAGG------CCCATGGAC----------------------------CCGGGCTTGCTGCCCATTTTCTCAGTCTGCCTCATCTTCGCCTTTCTGTATG 66

Homo    GA--------------------AGATGGTCTGCAAATCCTTTATCCCCT-GGTGTCTCTTTCTGGCCCCTCTGTCCTAATGTTTGTAGCAGTTTTTCTCA 179
Pan     GA--------------------AGATGGTCTGCAAATCCTTTATCCCCT-GGTGTCTCTTTCTGGCCCCTCTGTCCTAATGTCTGTAGCAGTTTTTCTCA 179
Macaca  GA--------------------AGATGGTCTGCAAATCCTTTATACCCT-GGTGACCCTTTCTGGCCCCTCTGTCCTAATGTCTGTAGCAGTTTTTCTCA 179
Bos     GGACTGCCTCTTATTCTGTCCAAGCTGGCCTGCAAAGCCCATATCCCCC----ATCCTTCCCCACTCCCTCTGTTCCAGTGCCTATGGCTGCTTATCTTG 167
Canis   GAACTGCCCCTGGATCTGTTCCAGATGGCTTGCAAATCCCTTCTCTCCCCGAGTCCCCTTCCTGGTCCCACTGGCCTAGTGTGTAGAGCAGTTTGTCTCA 166

Homo    CATCAATCATGTCCATGACTGCAAATCCCATTGCTGTCTCTCTGTCTGTCTGGAAGTGTCTGACTCAGCACCATCTCTCCATCTAAGAGGGACCTGT-CT 278
Pan     CATCAATCATGTCCACGACTGCAAATCCCATTGCTGTCTCTCTGTCTGTCTGGAAGTGTCTGACTCAGCACCATCTCTCCATCTAAGAGGGACCTGT-CT 278
Macaca  CACTAATCATGTCCAGGACTGCAAATCCCACCGCTG--TATCTGTCTGTCTGGAAGTGTCTGACTCAGCCCCATCTCTCCACCTAGAAGGGACCTGTCCT 277
Bos     CTCCCACCCCATTC-GGACTGTGCATCCCCCCATGGTCCCTCTGTCTGTCTAGAAAGGTCTGGCTCAGCCCTGGTCGTCTGTCTAGAGGGGCCTGTCCCA 266
Canis   CTCCAACCCTGTCCAAGACTATGCACCCTGCTGTAGTCTCTCTACTGGTCTATGACTACC-------ACCCCAGCCCTCTTTCTGGAAGAATCTGTCCTA 259

Homo    TTCCCATCTCTCTCTGTCTTTCAAATTATCTCTC------------------------------------------------------------------ 312
Pan     TCCCTATCTCTCTCTGTCTTTCAAATTATCTCTC------------------------------------------------------------------ 312
Macaca  TCTCCACCTCTCTCTATCTCTCAAATTATCTCTC------------------------------------------------------------------ 311
Bos     TCTCCACCTCTGTC----TCTAATATTGTCTGTC------------------------------------------------------------------ 296
Canis   TCCCCACCTTTGTC--TTTCTAAAATTATCTGTCTCAGGTGCCTGGGTGGCTCAGTCAATTAAGTGTCTGCCTTCGGCTCAGGTCATGGTCCCCGGTCCT 357

Homo    --------------------------------------------------------TCTACACTGTGCCC------------------------------ 326
Pan     --------------------------------------------------------TCTACACTGTGCCC------------------------------ 326
Macaca  --------------------------------------------------------TCTACTGTGTGCCT------------------------------ 325
Bos     --------------------------------------------------------CCTTCCCTGTGCCC------------------------------ 310
Canis   AGGATTGAGCCCCACATTAGGCTTAGTGGGAGCCTGCTTCTACCACTCCCTCTGTTCCTCCCCTCTGCTTGTGGTCTCCCTCTCCCTCAAATAAATAAAT 457

Homo    --------------------------------CTTTCAGGACTGTCCCCAGGCTTAGCA-----------------CCAGTTCTCTAAGA-CTCCATTCC 376
Pan     --------------------------------CTTTCAGGACTGTCCCCAGGCTTAGCA-----------------CCAGTTCTCTAAGA-CTCCATTCC 376
Macaca  --------------------------------CTTTCAGGACTGTCCCCAAACTTAGTG-----------------CCAGTTCTCTAAGA-CTCCATTCC 375
Bos     -------------------------------TGGCTCAGGACTGTCTCCCCGCTGTCTAGGACACGTTGCCCCCCTCCAGTTCTTTAAGA-CTCCACCCC 378
Canis   AAAATCTTTTAAAAAAAATTGTCAGTCTCTATTGTCCCTGACTGTCCCTGGACTGTCTAGGGCA-GCCTATCCCCTCCAGTTCTCCAAGAGCCCCCTCCC 556

Homo    ATCTCTGTCC--TTCTTCTCTTTGTCTTTCTAGAGTCCTAGGTCCTCTGCCTCCCAGCCTCCTTGCCACCCATTGACAGGAATAGGGCC----ATGTGCG 470
Pan     ATCTCTGTCC--TTCTTCTCTTTGTCTTTCTAGAGTCCTAGGTCCTCTGCCTCCCAGCCTCCTTGCCACCCATTGACAGGAATAGGGCC----ATGTGCG 470
Macaca  ATCTTTGTCC--TTCATCTCTTTGTCTTTCTAGAGTCTTAGGTCCTCTGCCTCCCAGCCTCCTTCCTAGCCATTGACAGGAGTAGAGCC----ATGTGCA 469
Bos     ATGTCATGTCTGTCCTTAGCTCTGTCCTTCTAGGATCTTAGATCCTCTGCCTCCCAGTCTCCTTGCCACCCA-TGACCAGAGCAGGGCC---------- 466
Canis   ATCTCATGCCTGTCCTTAGCTCTGTCTTTCTAGGATCTTAGGCCCTTCTGCC-----TCTCCTTCCCAC----TGAGCAGAGCAGGGCCAGCCATGTTTG 647

Homo    GGTGCCCTCCCAAGCATGGTCACCCTGAGCCCACTTTCCCTCTCTCCCCTGCCAGCTCT----------CCTCCCCATTCAGTCACCAGTCACTGTGACA 560
Pan     GGTGCCCTCCCAAGCATGGTCACCCTGAGCCCACTTTCCCTCTCTCCCCTGCCAGCTCT----------CCTCCCCATTCAGTCACCAGTCACTGTGACG 560
Macaca  GGTGCCCTCCCAAGCATGGTCACCCTGAGCCCACTTCCCCTCTTTCCCCTGCCAGCTCT----------CCTCCCCATTCAGTCACCAGTCACCATGACG 559
Bos     -----------ATGCATGGTGGCCCGAAGCTCAGCTTCCATCTTTCCTACACTGACACA-----------------------GTGACAGTCAC--TGACA 530
Canis   GGACCCCTCTGAAGCGTAGTGGCCCTGAGCCCACCTTCCCTGTCTCCCATGCTGGAGGGTGCGTGCTGGCTCTTCTCCCCATGTGATGGTCACTGTGACG 747

Homo    TG-CTCACTTTTCATCC------------CCAACCTACCAGTTCCTCCCTAAGTGCTCCCCTCGAGTGTTCTCTGAACCTCCTTGAAGG--CCCCACAGA 645
Pan     TG-CTCACTTTTCATCC------------CCAACCTACCAGTTCCTCCCTAAGTGCTCCCCTCGAGTGTTCTCTGAACCTCCTTGAAGG--CCCCACAGA 645
Macaca  TA-CTCACTTTTCATCC------------CC-CCCTACCAGTTCCTCCCTAAGTGCTCCCCTCGAGTGCTCTCCGAACCTCCCTGAAGG--CCCCACAGA 643
Bos     TGACTCACATTCCGTCCCTCTAGCCAACCCCTCCACCCTAGTTCTTCCCTACATG---GCCTGGAATGTTCTCCTTTCCTCTCCGGAGGCCCCCCACAGA 627
Canis   TGACTCACTCTTCGTCCCCCTACTTG---CCTGACTCCCTGCCGGACTGTACATG---CCCTGGACCACTCTACTCTCCCC------------------- 822

Homo    GGCCCCTCAGGTGGCAGGAGGCTGGTGGCAG-TGTGTTGGTGTCTTAGCCTTCCCTGCACAT-------TAGTGCCTGCCACTG--GCTCCCTCTGCAG 734
Pan     GGCCCCTCAGGTGGCAGGAGGCTGGTGGCAG-TGTGTTGGTGTCTTAGCCTTCCCTGCACAT-------TAGTGCCTGCCACTG--GCTCCCTCTGCAG 734
Macaca  GGCCCCTCAGGTGGCAGGAGGCTGGTGGCAG-TGTGCTGGTGTCTTAGCCTTCCCTGCACAT-------GAGTGCCTGCCAATG--GCTCCCTCTGCAG 732
Bos     GGCCCCTGGTGTGGTGGGAGCCGGGTGGCAGGTGGCCTGGTGTCCCAGCCTCCCCAGTCCCATCGCACGTGGTCCCTGCCACCG--TCTCTCTCTGCAG 724
Canis   GGCTCCTAGAGCAGTAGGAGCCAAGTGGCAGATACACTGGGGTCCCAGGGTCCCCTGTATCA---------GTTCCTGTCACCGTCTCTCTCTCTGCAG 912
```

ENSG00000130222 intron 1

Description: GADD45 gamma protein (GADD45G)
Intron number: 1
Human chromosome: 09
Intron start (bp): 91409910
Human intron length : 257
Intron alignment length: 297
Flanking exons length (upstream/downstream): 53/102
SNP density: 0.011673
K tree score: 0.0722
Scaling factor: 0.7495
Human-chimpanzee distance: 0.004262
Total primate branch length: 0.1916

ENSG00000130222 exon 1

```
Homo    ATGACTCTGGAAGAAGTCCGCGGCCAGGACACAGTTCCGGAAAGCACAGCCAG 53
Pan     ATGACTCTGGAAGAAGTCCGCGGCCAGGACACAGTTCCGGAAAGCACAGCCAG 53
Macaca  ATGACTCTGGAAGAAGTCCGCGGCCAGGACACAGTTCCGGAAAGCACAGACAG 53
Bos     ATGACTCTGGAAGAACTTCGTGGCCAGGACACGGTTCCAGAAAGCACAGCCAG 53
Canis   ATGACTCTGGAGGAGATCCGAGGCCAAGACACGGTCCCCGAGAGCACCGCCAG 53
```

ENSG00000130222 exon 2

```
Homo    GATGCAGGGTGCCGGGAAAGCGCTGCATGAGTTGCTGCTGTCGGCGCAGCGTCAGGGCTGCCTCACTGCCGGCGTCTACG 80
Pan     GATGCAGGGTGCCGGGAAAGCGCTGCACGAGTTGCTGCTGTCGGCGCAGCGTCAGGGCTGCCTCACTGCCGGCGTCTACG 80
Macaca  GATGCAGGGTGCCGGGAAAGCGCTGCACGAGTTGCTGCTGTCCGCGCAGCGTCAGGGCTGCCTCACTGCCGGCGTCTACG 80
Bos     GATGCAGGGCGCCGGGAAAGCGTTGCACGAGCTGCTGTTGTCGGCGCAGCGCCAAGGCTGCCTCACGGCCGGCGTCTACG 80
Canis   GATGCAGGGCGCCGGGAAAGCACTCCACGAGCTGCTGCTGTCCGCGCAGCGCCAGGGCTGCCTCACCGCCGGCGTCTATG 80

Homo    AGTCAGCCAAAGTCTTGAACGT 102
Pan     AGTCAGCCAAAGTCTTGAACGT 102
Macaca  AGTCAGCCAAAGTCCTGAACGT 102
Bos     AGTCAGCCAAAGTCCTGAACGT 102
Canis   AGTCCGCCAAAGTCCTGAATGT 102
```

ENSG00000130222 intron 1

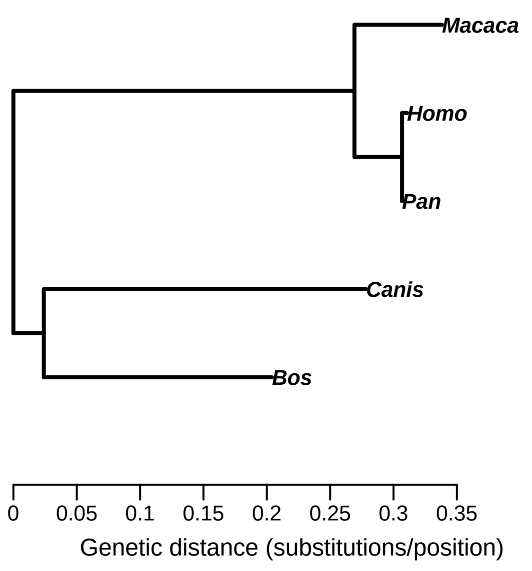

ENSG00000130222 intron 1

```
Homo    GTGG--------------------GTTTCAGGGCGCTGAGAAAGCC-------------------GCTGGTCGGTCGGCGACCGTCAGGGTTTTCCAGGG 61
Pan     GTGG--------------------GTTTCAGGGCGCTGAGAAAGCC-------------------GCTGGTCGGTCGGCGACCGTCAGGGTTTTCCAGGG 61
Macaca  GTGG--------------------GTTTCAGGGCGCTGAGAAAGCG-------------------GCTGGTCAGTCGGCGACCGTCAGGGTTCTCCAGGG 61
Bos     GTAGGTCGGCGCCGAACGCCGGACGTCTCGGGGCGCGCGGGGATCCTTGTCCTGCCCTGGCCGC-GTTGGTTGGGCGATAGTCTTTGGGGCCCGCCTGGG 99
Canis   GTGGGTCGGCGCGGGGCGCAGGACGTCTC--GGCGCGCGGGGCGCCTGTGCCCGTGCGGGCTGCAGCGGGTCGGGCGGCCGCCGCGGGGGCCCGCCGGGA 98

Homo    T-GGGAATCCGCGGGGTAGGAGGGGAGCGCGGGGGTCCGGGCGCCAGATCCCGGTTGGAGAGCCGGGGTGCAGGCGCTGAGCCGGGATTGGAGTGTGGTT 160
Pan     T-GGGAATCCGCGGGGTAGGAGGGGAGCGCGGGGGTCCGGGCGCCAGATCCCGGTTGGAGAGCCGGGGTGCAGGCGCTGAGCCGGGATTGGAGTGTGGTT 160
Macaca  T-GGGAATCCGCGGGATAGGAGCCGAGCCCGGGGGTGCGGGCGTCAGATCCGGGTTGGAGAGCCGGGGTGCGGGCGCCAAGCCGGGATTGGAGTGTGGTT 160
Bos     T-GGAAACCGGCTGGGTGGAAGACGGGCGCGAGTGTGCCGGC----------------TGGGCCGAAGGTCCGGCCCTGGGCTG----------GCGCGC 172
Canis   TGGGGAGCTCGCTGGGCGGGGGCTGG-------------GGC----------------ACGGACGGGGGTGCGTCCC----------------------C 147

Homo    GGAGTTGGGGAGCCAAGGGTGTGTGCCGGTGGCCGGGGCTGGGGTCTCCGCCGCGCCCTCCGGCCGGCTCCCGCTCACTGCGCTGGCTCCTCCGCAG 257
Pan     GGAGTTGGGGAGCCAAGGGTGTGTGCCAGTGGCCGGGGCTGGGGTCTCCGCCGCGCCCTCCGGCCGGCTCCCGCTCACTGCGCTGGCTCCTCCGCAG 257
Macaca  GGAGTTGGAGAGCCAAGGGTGAATCCCCGTGGCCGGGGCTCGGGGCTCCACCGCGCCCCACGGCCGTCTCCCGCTCACTA-GCTGGCTCCTCCGCAG 256
Bos     AGGGCTGGGGCGCTGAGGGTGAGTCTCA------GGGTCCAGGGATCTCAGCGCACCCTCCGGCCCG-TCCGGCTCACCGTGCTGGCTTGTCCGCAG 262
Canis   GGGGCCGGGTCGCCG-----------CA------GGGGCTGGGGATCCCTGGCCGCTCCCCG-------CGCGCTCACGGCGCTGGCTCGTCCGCAG 220
```

ENSG00000151657 intron 6

Description: HsKin17 protein (KIN)
Intron number: 6
Human chromosome: 10
Intron start (bp): 7856861
Human intron length : 859
Intron alignment length: 1075
Flanking exons length (upstream/downstream): 49/61
SNP density: 0.001164
K tree score: 0.0671
Scaling factor: 0.795
Human-chimpanzee distance: 0.029203
Total primate branch length: 0.1853

ENSG00000151657 exon 6

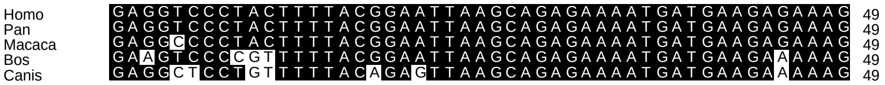

ENSG00000151657 exon 7

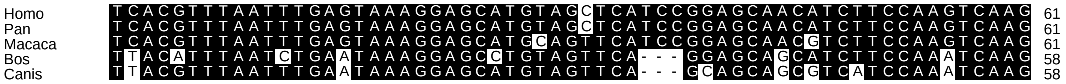

ENSG00000151657 intron 6

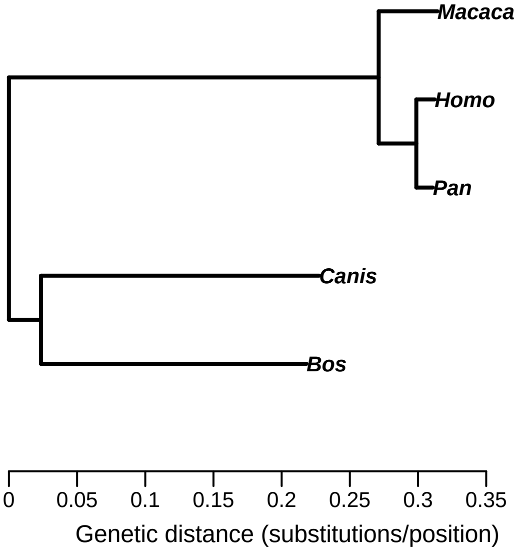

ENSG00000151657 intron 6

```
Homo    GTAGTTTATTTTTTATTT--TTTCTTTTTTTAAACATAGATGCTTTTATATT-GGCTTTGTTAAAAATGCAACTTGTCAACATATCTGAGAATAAGACA 97
Pan     GTAG--------------T--TTTCTTTTTTTAAACATAGATGCTTTTATATT-GGCTTTGTTAAAAATGCAACTTGTCAACATATCTGAGAGTAAGACA 83
Macaca  GTAGTTTATTTTTCTTTTCTTTCTTTTTTTAAACATAGATGCTTTTATATT-GGTTTTGTCAAAAATGCAACTTGTCAACATATCTGGGAGTAAGACA 99
Bos     GTACTT----TC---------TTCCCTGTTTGAAAATGGGATGCTTTTATATTGAACCCTGCCAAAAA------CTAGCAACATGTTTGGGAATAGGACA 81
Canis   GTAGCTTTACTT---------TTTTCTTCTTTTAATATGGATGATTTTGTATT-AGCGCTACCAAAAA------TTAGCAACATGTTTGCGGACAAGACA 84

Homo    TCGAATGTGTCTGAGTTACATTGTTCTTATTAATTTAATGAAA-AGGTAATACTAATGAATTGATTGGATAGGATAGCTGAAGCCACAAGTAACAAAACT 196
Pan     TCTAATGTGTCTGAGTTACATTGTTCTTATTAGTTTAATGAAA-AGGTAATACTAATAAATTGATTGGATAGGGTAGCTGAAGCTACAAGTAACAAAACT 182
Macaca  TCCAATCTGTCTGAGTTACATTATTCTTATTGATTTAATGAAA-AGATAATACTAATGAATTAATTAGATAGGCTAACTGAAGCTGCAAGTAACAAAATT 198
Bos     TATTATCTACCTGGACTGCTTTATTCTCATTAGTAAAATGAAAAAGATGATAACAATGA----ATTAGATGGAATGCCTTCAGCAATGAATAACCAGACT 177
Canis   TG--ATCTGTCTGGATTGCATTGTTTTTACCACTTTATGAAA-----GGTGATAATGA----ATCAGATAGGATGCCTTCAGCTACAAGTGAAAAAAC 173

Homo    GAC--------TACAATTAACT-----------TAAACAATAAGGAAATATCTTGCCATAATAA------GAATCCCAGAGGTAAGGTGGTTCTGCATGC 271
Pan     GAC--------TACAATTAACT-----------TAAACAATAAGGAAATATCTTGCCATAATAA------GAATCCCAGAGGTAAGGTGGTTCTGCATGC 257
Macaca  GAC--------TACAGTTAGCT-----------TAAACAATAAGGAAATATCTTGCCATAATAA------GAATCTCAGAGGTAAGGTTCTGCATGCTGC 273
Bos     GAG--------TAAAATTAGCC-----------TAAACGGTAAGGAAATACTTTGCCATACCAAGACTCCGACTCCCAGAGATAGGGTGGTTCTGGATGT 258
Canis   AAAAACACAACTAAAATCAGCCTAAATAGTATGTAAATAGTATGTAAATACTTTGCTGTAACAA------GAGCCCCAGAGGTTGGGCGCCTCTGGATGC 267

Homo    CATC----------------TAGACACAGGGTAGCTGCCAACAC-TCCAGCCGTCACATGCAGATTCTGCATTGTCGTGAGGATGGCAGAGAGCATCTCT 354
Pan     CATA----------------TGGACACAGGGTAGCTGCCAACAC-TCCAGCCATCACATGCAGATTCTTCATTGTTGTGAGGATGGCAGAGAGCATCTCT 340
Macaca  TGTA----------------TGGACACAAGGTAGCTGCCAACAC-ACCAGCCATCACATGCAGACTCTGCATTGTCGTGAGGGTGGCAGAGAGCATCTCT 356
Bos     CGCA-----CTGTCAGCAGCTGGACACAAGGTGGCTGCCAGCAC-TCCAGCCATCATATGCAGACCCAGC-CTGTCCAGAGGAAAACAGAGAGCCT---- 347
Canis   CATGGTATCCTGTCAACAGCTGAATGTGAGGTGGCTGCGGGCACTTTGGCCATCGTATGCACACCCAGTATTGTCCAGAGGATGACGGGG--------- 358

Homo    GGATGCTCTTCTAGAATCTTGTTAAGAGCAAGGATACTTTTCCAAGAAGACACTCCTCCCTTCAATAGATTTCCTTTCAAATGTCATTGGCCAGAACTGT 454
Pan     GGATGCTCTTCTAGAATCTTGTTAGGAGCAAGGATACTTTTCCAAGAAGACACTCCTCCCTTCAATAGATTTCCTTTCAAATGTCATTGGCCAGAACTGT 440
Macaca  GGATGCTCTTCTAGAATCCTGTTAGGAGCAAGGATACTTTTCCAAGAAAACACTCCTCCCTTCAATAGATTTCCTTTCAGATGTCATTGGCCAGAACTGT 456
Bos     -GTTTCTCTTCTGGAGTCCC-CTATGAGCAAGTAAGCTTTTCCTAGAAGACCTTCTTCCTAGCAGCAGGTTTCCTTTTGAATGTCATTGGTCAG-ACTGT 444
Canis   -GCAACTTTTCTGGAGTCCC-CGGTGAGCAAGGAAACTTTTCCTATAAGACACTCCTCTGGCCTGCAGACTTCCTTTTGAATGTCACTGACCAG-TCTGT 455

Homo    GTCACATG-TCCACTCAAAAAGCAGTCACTGGG------------ATTATGTTGATTGGATTAGA----------------------------------- 506
Pan     GTCACATG-TCCACTCAAAAAGCAGTCACTGGG------------ATTATGCTGATTGGATTAGA----------------------------------- 492
Macaca  GTCACACG-TCCACCCAAAAAGCAGTCACTGGG------------ATTATGTTGATTGGTATAGA----------------------------------- 508
Bos     ATCTTGTGTCCCCCCTCAAAAACAGTCACTGGGAAAGGAAATGGAATGATTTTGATTGGTTTAGA-CCAAAGAACCATACTTATCCCCTCCCACAGG-TC 542
Canis   A--TTAGCCTCCAGCTTACGAGCAGTCACTGGGAAAGGAAGTGAGATGATTTTGGTTGGTTTAGAGCAAAAGAACCTTACTGATTACCTCTAGAAGGCTC 553

Homo    --------------------------CAATCAGAATTCACCCCAGAGATGAAAATGGTGTTATG------------------TCCCCTGAGCCACAGGCA 562
Pan     --------------------------CAATCAGAATTCACCCCAGAGATGAAAATGGTGTTATG------------------TCCCCTGAGCCGCAGGCA 548
Macaca  --------------------------CAATCAGAATTTACCCCAGAGATGAAAATGGTGTTATG------------------TCCCCCGAGCCACAGGCA 564
Bos     CCACCTCTTAGTCCCATCCCCTTGGGTGTTAGGTTTTCAACGTTTAGATTTGGGAAGGGACACACCCGTTCAGACCATAGCATCCTCCAAGCCATGTGTG 642
Canis   CCATCTCTTAATCCCATCACTTTGGCCATTAGGTTTTCAACATACGAATTTGAG-GGGGATACGAACATTCATACCCTAGCATCCTCCAAGCCATATATG 652

Homo    AGAGGCGTGGAGACCTAAACAGAATTGGAGTACTGT----TCCCCAGGGGAAATGGGG--ATAGAGCAGGGAAGCGCCGGTGTGGTCTGCCACGGTGACA 656
Pan     AGAGGCGTGGAGACCTAAACAGAATTGGAGTACTGT----TCCTCAGGGGAAATGGGG--ATAGAGCGGGGAAGCGCCCGTGTGGTCTGCCACGGTGACA 642
Macaca  AGAGGTATGGAGACCTAAACAGAATTGGAGTACTGT----TCCCCAGGGGAAATGGGG--ATAGAGCAGGGAAGTGCCTGTGTGGTCTGCCACGGTGACA 658
Bos     AGGCCTTCCGACACCGAAACAAGATGGGATTCCTGT----TACCAAAGGGAGAGGGGAGGCGGAGCAGGGAAGGGGTCAAGGCCACAT------GCTCTG 732
Canis   ACAGGTATGAACACCTCAACAAAATTGCAATCCTGTCCCCTAGTAAGGGGAGAGGGGGCTATGGAGCCGGAAGTGGCCATGGCTGTCCCCTACAGGTCAA 752

Homo    CGGTGTGCAA-GTTCCTCCTGTTCTATGGCTTTCAGTGTGGGACGTTT---TACAGGAATGTGTGGTCTCCGTATAGGAACATTTTAGCTCTCGTTTATT 752
Pan     TGGTGTGCAA-GTTCCTCCTGTTCTATGGCTTTCAGTCTGGGACGTTT---TACAGGAATGTGTGGTCTCCGCATAGGAACATTTTAGCTCTCATTTATT 738
Macaca  TGGCGTGCAAGGTTCCTCCTGTTCTATGATTGTCAGTCTGGGACGTTT---TACAGGAATATGTGGTCTCCGCATAGGAACATTTTAGCTCTCGTTTATT 755
Bos     TCCAGTGAAGATCTCTCCTGCTCTGTAAGCACCACTTCAGTATATTT---TACGGGAGTTTATGATCTCTGGGTAGGAGCATTCCAGCTCTCATTTATT 829
Canis   TCATGTGTAGATCT-TCCCAGCTCTGTGAGCATCCGCCCAGTATTCTTAACTGCAGAAATTTATCATCTCAGGATGGGAGCATTCCAGCTCTCGTTTATT 851

Homo    GAAAATGTCCTGGAGTTCCTCTGTGAACACTTTGTAGTATCTTATCTGAGAAGTTTTATTATGATTTTCTTGAA-------------------------- 826
Pan     GAAAATGTCCTGGAGTTCCTCTGTGAACACTTTGTAGTATCTTATCTGAGAAGTTTTATTATGATTTTCTTGAA-------------------------- 812
Macaca  GAAAATGTCCCTGAGATCCTCTGTGAACACTTTGTAG-----TATCTGAGAAGTTTTATTATGATTTTCCTGAA-------------------------- 824
Bos     CAAAATGTCCATGAATTTCTGTGTGGCTTCTTTGTGGTATTTACCT---GATGTTTTGCTAGAATTTTCCCCAA-------------------------- 900
Canis   CAAAATGTCCATGAACTTCTATGTGGAGATATTGTGGTATCTTACT---GTTAAGTT-TTATAATTTTTCTGAAAAAAAAAATACACTTGTGTGTGTGTA 947

Homo    -----------------------ATGTATGTATGGAA-----------------TAACATTT--TTTTCTTCTAG 859
Pan     -----------------------ATGTATGTATGGAA-----------------TAACATTT--TTTTCTTCTAG 845
Macaca  -----------------------ATGTACATGTGGAA-----------------TAACATTT--TTTTCTTCTAG 857
Bos     -------------------------ATATATATCGAA-----------------TACCAGTTC-TTTTCTTCCAG 932
Canis   TATATATATGTATGTATGTATATGTATATATAATGAAATATACATGCAAAAATGTATCATTTCTTTTTCTTCCAG 1022
```

ENSG00000157315 intron 2

Description: Transmembrane emp24 domain-containing protein 6 precursor (TMED6)
Intron number: 2
Human chromosome: 16
Intron start (bp): 67939341
Human intron length : 1588
Intron alignment length: 2074
Flanking exons length (upstream/downstream): 127/149
SNP density: 0.007557
K tree score: 0.0798
Scaling factor: 0.9601
Human-chimpanzee distance: 0.012808
Total primate branch length: 0.1458

ENSG00000157315 exon 2

```
Homo    GTTCAGCGGACAGTGGGGATGTCACATGACCGGCATGTTGCTGCCACGGCACATAACCCACAGGGATTTCTCATAGACAC 80
Pan     GTTCAGCGGACAGTGGGGATGTCACATGACCGGCATGTTGCTGCCACGGCACATAACCCACAGGGATTTCTCATAGACAC 80
Macaca  GTTCAGCGGACGGTGGGGATGTCACATGACCGGCATGTTGCTGCCACGGCACATAACCCACAGGGATTTCTCATAGACAC 80
Bos     GTTCAGCGGACAATAGGAATGTCACATGACCGGCATGTTGCTGCCACTGCACATACCCCACAGGGTTTCCTTATAGAGAC 80
Canis   GTGCAGCGGACAGTGGGAATGTCACATGACCGGCATGTTGCTGCCACTGCACATACGCCACAGGGTTTCCTCATAGACTC 80

Homo    CTCCCAGGGTGTTCGGGGCCAGATTAACTTCTCTACCCAAGAGACAG 127
Pan     CTCCCAGGGTGTTCGGGGCCAGATTAACTTCTCTACCCAAGAGACAG 127
Macaca  CTCCCAGGATGTTCGGGGCCAGATTAACTTCTCTACCCAAGAGACAG 127
Bos     CTCTAAGAATGTTCGGGGACAGATTAACTTCTCTACCCACGAGACAG 127
Canis   CTCTCAGAATGTTCGGGGCCAGATTAACTTCTCTACCAAAGAGACAG 127
```

ENSG00000157315 exon 3

```
Homo    GTTTTTATCAGCTTTGTCTAAGTAATCAGCATAATCACTTCGGTTCTGTGCAAGTGTACCTCAACTTTGGGGTCTTCTAT 80
Pan     GTTTTTATCAGCTTTGTCTAAGTAATCAGCATAATCACTTCGGTTCTGTGCAAGTGTACCTCAACTTTGGAGTCTTCTAT 80
Macaca  GTTTTTATCAGCTTTGTCTAAATAATCAGCATAATCACTTCGGTTCTGTGCAAGTGTACCTCAACTTTGGAGTCTTCTAT 80
Bos     GTTTTTATCAGCTTTGTCTAGCAAACCAGCAAAATCGCTTTGCTTCCGTCCAAGTATACCTCAACTTTGGAGTCTTCTAT 80
Canis   GTTTTTATCAGCTTTGTCTAAAAAATCAGCAAAATCACTTTGGTTCTGTGCAAGTGTACCTCAATTTTGGAGTCTTCTAT 80

Homo    GAGGGGCCTGAGACTGATCACAAACAGAAG---GAAAGAAAACAACTGAATGATACTCTGGATGCAATTGAG 149
Pan     GAGGGGCCTGAGACTGATCACAAACAGAAG---GAAAGAAAACAACTGAATGATACTCTGGATGCAATTGAG 149
Macaca  GAGGGGCCTGAGACTGATCACAAACAGAAG---GAAAGAAAACAACTGAACGATATTCTGGATGCAATAGAG 149
Bos     GAGGGGCCTGAGATGGACCACAAAGAGAAT---GAAAGAAAACAACTGAATGATACTCTGGATGCAATTGAG 149
Canis   GAGGGGCCTGAAATGGACCACAAACAGAAGAATGAAAGAAAGCGACTGAATGATACTCTGGATGCAATTGAG 152
```

ENSG00000157315 intron 2

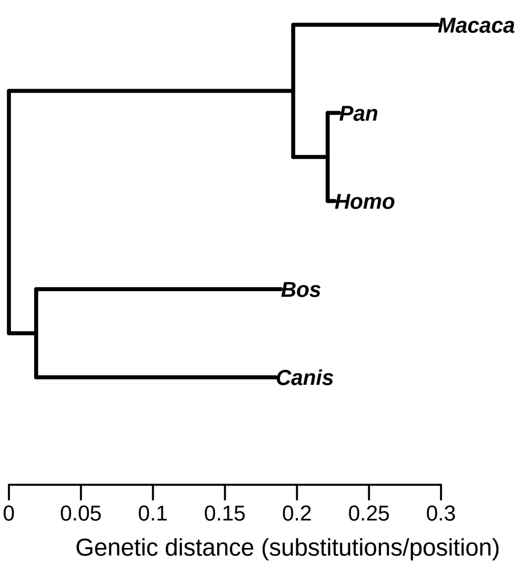

ENSG00000157315 intron 2

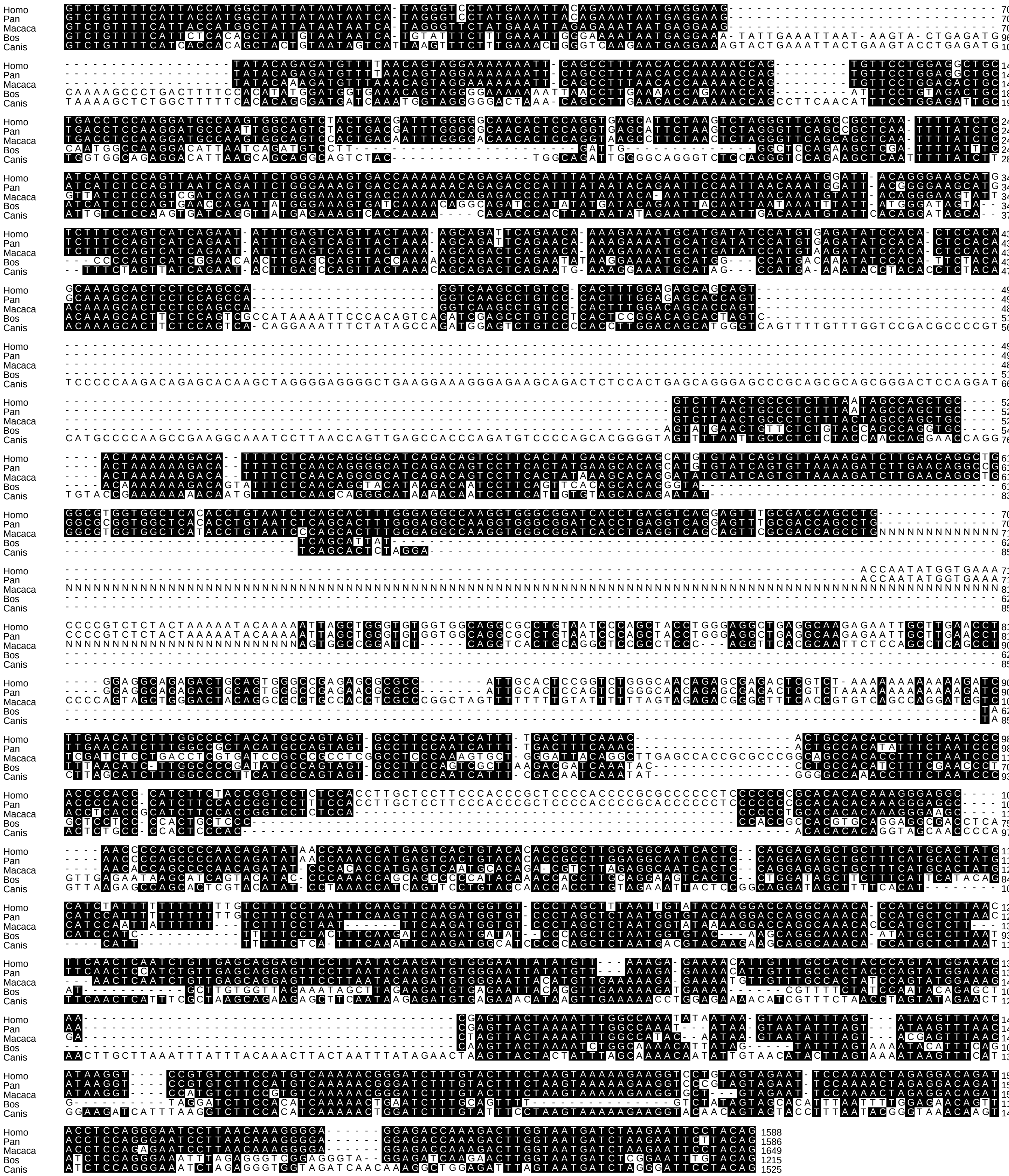

ENSG00000120685 intron 8

Description: C13orf23 protein (C13orf23)
Intron number: 8
Human chromosome: 13
Intron start (bp): 38494550
Human intron length : 639
Intron alignment length: 715
Flanking exons length (upstream/downstream): 79/87
SNP density: 0.001565
K tree score: 0.0727
Scaling factor: 0.9708
Human-chimpanzee distance: 0.026885
Total primate branch length: 0.1405

ENSG00000120685 exon 8

```
Homo    CCTCCTTCAACATATAATCCACATAAACCTGTTCCTTATCCGATACCTCCATGCCGACCACATGCAACTATTGCACCAA 79
Pan     CCTCCTTCAACATATAATCCACATAAACCTGTTCCTTATCCGATACCTCCATGCCGACCACATGCAACTATTGCACCAA 79
Macaca  CCTCCTTCAACATATAATCCACATAAACCTGTTCCTTACCCAATACCTCCATGCCGACCACATGCAACTATTGCACCAA 79
Bos     CCTCCTTCAACATACAATCCACATAAACCTGTCCCTTACCCGATACCTCCATGCCGACCACACGCAACTATTGCACCAA 79
Canis   CCTCCTTCAACGTACAATCCCCATAAGCCTGTTCCTTATCCAATACCTCCATGCCGGCCACACGCAACTATTGCACCAA 79
```

ENSG00000120685 exon 9

```
Homo    GTGCTTATAACAATGCAGGTCTGGTACCATTAGCGAATGTCATAGCTCCA---------CCTCCACCTCCATATACTCCT 71
Pan     GTGCTTATAACAATGCAGGTCTGGTACCATTAGCGAATGTCATAGCTCCA---------CCTCCACCTCCATATACTCCT 71
Macaca  GTGCTTATAACCATGCAGGTCTGGTACCATTAGCCAGTGTCATAGCTCCAGGTACGCCCCCTCCACCTCCATATACTCCT 80
Bos     GTGCTTATAACAATGCAGGTCTGGTACCGTTAGCCAACGTCATAGCTCCAGGAGTGCCCCCGCCACCTCCATATACTCCT 80
Canis   GTGCTTATAACAATGCGGGTCTGGTACCATTAGCCAACGTCATAGCTCCAGGTGTACCCCCTCCACCTCCATATACTCCT 80

Homo    AATCCTGTAGGAACAG 87
Pan     AATCCTGTAGGAACAG 87
Macaca  AATCCTGTAGGAACAG 96
Bos     AATCCAGTAGCAGCAG 96
Canis   AATCCAGTAGGAACAG 96
```

ENSG00000120685 intron 8

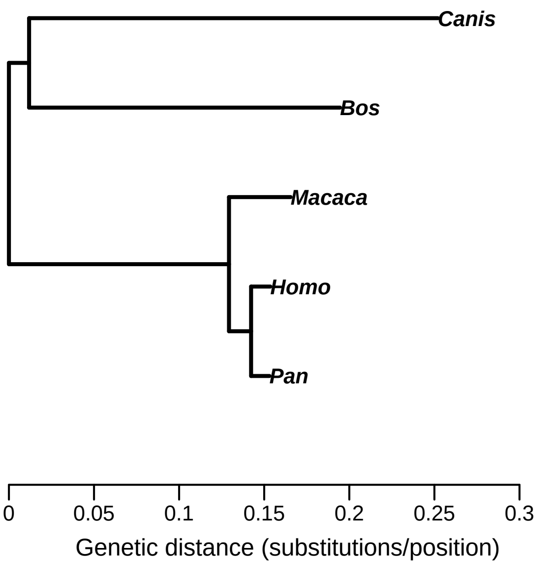

ENSG00000120685 intron 8

```
Homo    GTAAGGAGTTCCCTTA-TTTTTTACTTGTTTTTCTTTTTTGCTGTTTATTTATTAAATTGAAAGTAAATGCTGGTCTATGTGG------------------ 82
Pan     GTAAGGAGTTCCGTTATTTTTTTACTTGTTTTTCTTTTTTGCTGTTTATTTATTAAATTGAAAGTAAGTGCTGGTCTATGTGG------------------ 83
Macaca  GTAAGGAGTTCCCTTA-TTTTTTACTTGTTTTTCTTTTTTGCTGTTTATTTATTAAATTGAAAGTAAGTGCTGGTCTATGTGC------------------ 82
Bos     GTAAGGAGCTCTTTCA-GAGTTCACTT-TTTTTCCTTTTTGCTCTTTATTTATTAATTTGAAAGCAAAGGTTGGTATATATAC------------------ 81
Canis   GTAAGCAGTTCATTCA-TTTTTAAA---TTTTTTTTTCTTGCTCTTTATTTACTAAGTTGAAAGAAAATGTTCGTGTGTGTGTGTGTGTGTNATACACAC 96

Homo    -------------------TTGGCAAATCTGTAGCACTTATGAA--ATGCTAC-------TGTGAGCTTAATTATGCCCATAGTTAGCTA--GTAGTGTC 152
Pan     -------------------TTGGCAAATCTGTAGCACTTATGAA--ATGCTAC-------TGTGAGCTTAATTATGCCCATAGTTAGCTA--GTTGTGTC 153
Macaca  -------------------TTGGGAAATCTGTAGCACCTCTGAT--ATGCTAC-------TGTGAGCTTAATTATGCCCATAGTTAGCTA--GTAGTGTC 152
Bos     -------------------TCTGGAAATCTGGAGCACTCATGAAATATGCTAT-------GCTGAGCTTTCCTTTGCCCATAGTTGGTTACTCTAGGGTA 155
Canis   ACACACACACACACACACATTGGGAAAATTAGAGCACTTACGAAATAGACTATAGGCTCTGCTGAGTTTATTTCTGCCCACAGTAGCCTAC-ACAGTGTC 195

Homo    CTGGCCTACAACACTGCAGCAGAT-------------AGTTTATTTAGGATATTTTCTTTATGATAAATTAAT-TAGA---TTATTTTTAGTCATTCATT 235
Pan     CTGGCCTACAACACTGCAGCAGAC-------------AGTTTATTTAGGATATTTTCTTTATGATAAATTAAT-TAGA---TTATTTTTAGTCATTCATT 236
Macaca  CTGGCCTCCAACACTGTAGCAGAC-------------AGTTTATTTAGGATATTTTCTTTATGATAAATTA---CAGA---TTATTTTTAGTCATTCATT 233
Bos     TTGGTCTACAACAGTGCAGAGGACTAAGAGGATTAATATCTCATTTAGGATATTTTCCTTTTGATAAATTAAT-TGTAGATTTTTTTTCATTGTTACTT 254
Canis   TTGGACTATAGCAGTGCAGCACAC----------AGAGTTAGTTTAGGGTATTAGCTTTTTAATAAATTCATATAGA---TTCTTTTTCATTGTTAT-- 280

Homo    ATT--TATGGGTTTTCACT--TTGGTAAATCATCTTGAATTCCTTTTTTTGGTAGTTGGTAGTTCAGAGGCATCATTCATGCAT-TTAACTTCAAAAAGT 330
Pan     ATT--TATGGGTTTTCACT--TTGGTAAATCATCTTGAATTCCTATTTTTGGTAGTTGGTAGTTCAGAGGCATCATTCATGCAT-TTAACTTTAAAAAGT 331
Macaca  ATT--TATGGGTTTTCACT--TTGGTAAATCATCTTGAATTCCT-TTTTTGGTAGTTGGT-GTTCGGAGGAGTCATTAATGCAT-TTAACTTAAAAAAGT 326
Bos     ATTTATATAAGCTTTTGCTTATGGGTAAATCATCTTGAATCCCT-TTTTTGATAGTTGGT-ATTCAAAGACATCATTAGTGCAT-TTAACTTGAAAGGGC 351
Canis   -----TATGGGTTTTCACT--TTGGTAAATCATCATGAATCCC--TTTTCAGTAGTTGGT-ATTTGAAGGTATCCTCAAGACGTCTTAACTTAAAAAGGT 370

Homo    CAAGTATATGCGTTTGGCAGAAAAGACAAATAATGTAAGTATAGCAGGTTCTGCCTGCCATGCCATTCACTGAACTTATCCCTGCAATGAGCATGCAGTG 430
Pan     CAAGTATATGGGTTTGGCAGAAAAGACAAATAATGTAAGTATAGCAGGTTCTGCCTGCCATGCCATTCACTGAACTTATCCCTGCAATGAATATGCAGTG 431
Macaca  CAAGTATATGTGTTTGGCAGGAAAGACAAATAATTTAAGTATAGCAGGTTCTGCCTGCCATGCCATTCACTGAGCTTATCCCTGCAATGAGGATGCAGTG 426
Bos     CAAGTGTGTGTGCTTGACATAAAAGGCAAATCATACACATCTGGCAGGTTCTGCCCACCGTGCCCTTCAGTGAG-TTAGGCCTGGAGAGAGCCTGCACT- 449
Canis   CACCTGTATGTGCTTGACAGAAAAGGCAGATCATTTAAATATGGCAGGATCTCCCTGCCATGCCAGTTACTGAGCTTATGTTTGGAATGAGCATGCAATG 470

Homo    GGAAATAGGAATCTCTGGTTGTTTCATTTGGTGTCAGTTTTCACTTAACTCTAGACATAAACCTCCATATAAAAT-CAACTGTATTAAATA--GGAGTTT 527
Pan     GGAAATAGGAATCTCTGGTTGTTTCATTTGGTGTCAGTTTTCACTTAACTCTAGACATAAACCTCCATGTAAAAT-CAACTCTATTAAATA--GGAGTTT 528
Macaca  GGAAATAGGAATCTCTGGTTGTTTCATTTGGTGTCAGTTTTCACGTAACTCTAGACCTAGACCTCTATCTAAAAT-CAACTCTATTAAATA--GGAGTTT 523
Bos     GGAAATAGAAGTCTCTGCTTGTCTAATTCGAGCTCACTCTTCACCCAA------------GCCTCCATGTAAAACACAACTGTACTAGATGACACAGTTT 537
Canis   GGAAAT--CAATCTGCAACTGTCTCATTTGATCTTGACTTTGTCCTTACACTAGACCTAAAACTCCATGTAAAATGCAATGTTACATTA----GCAATTT 564

Homo    TTATAATAAATTGAATAGCTGCATTAATATAGTTTGAACTCCTAGTCACAGAACAATACCAGTCACAGAACAGTCCTAAGGTATA---TTTTTTTCTATT 624
Pan     TTATAATAAATTGAATAGCTGCATTAATATAGTTTGAACTCCTAGTCACAGAACAATACTAGTCACAGAACAGTACTAACGTATA---TTTTTTTCTATT 625
Macaca  TTATAA-----TGCATAGCTGCATTAATATAGTTTGAACTCCTAGTCACAGAACAATATTAGTCACAGAACAGTGCTAACTTATA---TTTATTTCTGTT 615
Bos     TAGAAATAAAGTCGGTGGCTGTGTCAATACAGCTTGAACT-------------------------CGGAATGATGCTAAATTGTACTTTTTTTTTCTATT 612
Canis   TAAAAAT----CAGATAGCTGTGTTAACATGGTTTGAACTCTTGGCCA------------------CAGAATGATACTAAA--------TTCTTTTTTCCT 635

Homo    GTGTTTTTATTTTAG 639
Pan     GTGTTTTTATTTTAG 640
Macaca  TTGTTTTTATTTTAG 630
Bos     ATATTTTTATTTTAG 627
Canis   ATAATTGTATTTTAG 650
```

ENSG00000141560 intron 5

Description: Ketosamine-3-kinase (FN3KRP)
Intron number: 5
Human chromosome: 17
Intron start (bp): 78277768
Human intron length : 230
Intron alignment length: 248
Flanking exons length (upstream/downstream): 123/339
SNP density: 0.008696
K tree score: 0.0466
Scaling factor: 0.5394
Human-chimpanzee distance: 0.036106
Total primate branch length: 0.1405

ENSG00000141560 exon 5

```
Homo    GTGAATGACTGGCAGGAGGACTGGGTCGTGTTCTATGCCCGGCAGCGCATTCAGCCCCAGATGGACATGGTGGAGAAGGA  80
Pan     GTGAATGACTGGCAGGAGGACTGGGTCGTGTTCTATGCCCGGCAGCGCATTCAGCCCCAGATGGACATGGTGGAGAAGGA  80
Macaca  GTGAATGACTGGCAGGAGGACTGGGTTGTGTTCTATGCCCGGCAGCGCATTCAGCCCCAGATGGACATGGTGGAGAAGGA  80
Bos     GTGAACGACTGGCAGCAGGACTGGGTCACTTTCTATGCCCGGCAGCGCATTCAGCCCCAGATGGACCTGGTAGAGCAGAG  80
Canis   GTGAATGACTGGCAGGACGACTGGGTGACCTTCTATGCCCGGCAGCGCATTCAGCCCCAGATGGACATGGTGGAGAAGGG  80

Homo    GTCTGGGGACAGGGAGGCCCTCCAGCTTTGGTCTGCTCTGCAG  123
Pan     GTCTGGGGACAGGGAGGCCCTCCAGCTTTGGTCTGCTCTGCAG  123
Macaca  GTCTGGGGACAGGGAGGCCCTCCAGCTTTGGTCTGCTCTGCAG  123
Bos     GTCTGGTGACAGGGAGGCCCGTGAGCTCTGGGCTGCTCTGCAG  123
Canis   GTCTGGGGACAGGGAGGCCCGGGAACTCTGGTCTGCTCTGCAG  123
```

ENSG00000141560 exon 6

```
Homo    TTAAAGATCCCTGACCTGTTCCGTGACCTGGAGATCATCCCAGCCTTACTCCACGGGGACCTCTGGGGTGGAAACGTAGC  80
Pan     TTAAAGATCCCTGACCTGTTCCATGACCTGGAGATCATCCCAGCCTTGCTCCACGGGGACCTCTGGGGTGGAAACGTAGC  80
Macaca  TTAAAGATCCCTGACCTGTTCCGTGACCTGCAGATCATCCCAGCCTTACTCCACGGGGATCTCTGGGGCGGAAACGTAGC  80
Bos     TTAAAGATCCCCGACCTGTTCCATGATCTGGACATCATCCCAGCCCTGCTCCACGGAGACCTCTGGGGAGGAAACGTAGC  80
Canis   CTGAAGATCCCTGACCTGTTTCGTGACCTGGACATCGTCCCGGCCCTGCTCCACGGGGACCTCTGGGCCGGAAACGTCGC  80

Homo    AGAGGATTCCTCTGGGCCGGTGATTTTTGACCCAGCTTCTTTCTACGGCCACTCGGAATATGAGCTGGCAATAGCTGGCA  160
Pan     AGAGGATTCCTCTGGGCCGGTGATTTTTGACCCGGCTTCTTTCTACGGCCACTCGGAATATGAGTTGGCAATAGCTGGCA  160
Macaca  AGAGGATTCCTCTGGGCCGGTCATTTTTGACCCAGCTTCTTTCTATGGCCACTCGGAATATGAGCTGGCAATAGCTGGCA  160
Bos     GGAGGATTCCTCTGGGCCCATCATCTTTGACCCTGCTTCCTTCTATGGCCACTCGGAGTATGAGCTGGCAATAGCTGGCA  160
Canis   GGAGGACTCCTCCGGGCCTGTCATCTTCGACCCGGCTTCCTTCTACGGCCACGCGGAGTACGAGCTGGCGATAGCCGGCA  160
```

ENSG00000141560 intron 5

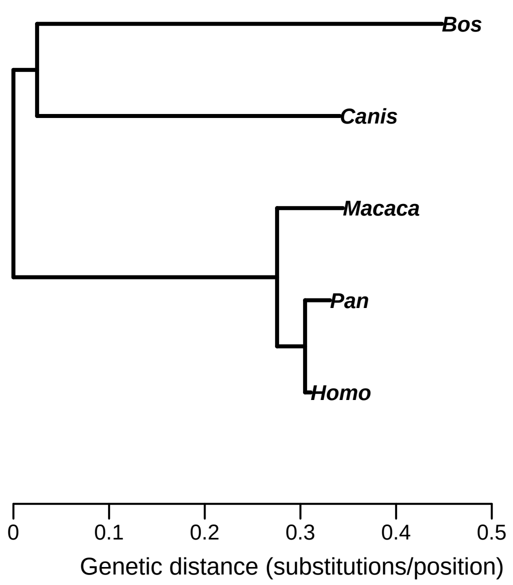

ENSG00000141560 intron 5

```
Homo    GTGAGTGGGGCCCCACTGCATGCCCAGCACCTGCCACACACCCCACTTGCCTGAGGTAGGGGAGGCACCAAGGCAGGTGAGGCCACCTCTGAGTCTGATT 100
Pan     GTGAGTGTGGCCCCACTGCATGCCCAGCACCTGCTACATGCCCCACTTGCCTGAGGTAGGGGAGGCACCAAGGCAGGTGAGGCCACCTCTGAGTCTGATT 100
Macaca  GTGAGCGGGGCCTCACTGCACGCCCAGCACCTGCCACACGCCCCTCTTGCGTGAGCTCGGGGAGGTGCCAGGGCAGGTGAGGCCACCTCTGAGTCTGGTT 100
Bos     GTGAGTGTCCATC-TCCCCGCGCCCTCCCCCCCGACCCCAACCTGTGCCCATCATGTCCTGCAGGTGCTGTGGCCAACGCAGCCA--CCTTGGTGTGGGT 97
Canis   GTGAGGGGAGGCC----------------------------------------------------------------TGAGGCAGGGCCTGGGGGCGGGT 36

Homo    CTGGGTGGGAAGCGGGTGCCTGGTGGTGTGCTATCCGCTGCTGCCTGGGCTGGGGGCGGTGGGGACCTGGAGC------------------GGCGCCCCT 182
Pan     CTGGGTGGGAAGCGGGTGCCTGGCGGTGTGCTATCCGCTGCTGCCTGGGCTGGGGGCGGCGGGGACCTGGAGC------------------AGCGCCCCT 182
Macaca  CTGGATGGGAAGCGGGTGCCTGGCGGTGTCCTATCCGCTGCTGCCTGGGCTGGGGGC---AGGCACCTGGAGCGGTGCTTAGGGAAACATGGGCACTCCT 197
Bos     CAGGACAGGAGGTGGGTGT-----------------------GTCTGTGTACCAACC---AGGCACCGGGAGCCACACTCAAAAAAGCCCATGTGTCCCT 171
Canis   CTGCACAGGAGGAG----------------------------GCCTGGGCCAGCCGT----GGGGGCTCGTGCGCCCTGCAGGAAAGCATCCTTGTCCCT 104

Homo    CATGCACGCGTTGATCAATTTGCTGAGGCTCCATTTTCCTCCCTGCAG 230
Pan     CATGCTCGCGTTGATCAATTTGCTGAGGCTCCATTTTCCTCCCTGCAG 230
Macaca  CGTGCATGCTTTGATCAATTTGCTGAGGCTCCATTTTCCTCCCTGCAG 245
Bos     CCCACACGCATCGATCACTTTGCTGAGGCTGCATTTAC--CCTGGCAG 217
Canis   CCCACACGTGTCGCTCACTTTGTTGAGGGTGC-CTGTCCTCCCTGCAG 151
```

ENSG00000090512 intron 1

Description: Fetuin-B precursor (FETUB)
Intron number: 1
Human chromosome: 03
Intron start (bp): 187841169
Human intron length : 344
Intron alignment length: 353
Flanking exons length (upstream/downstream): 225/111
SNP density: 0.000000
K tree score: 0.05
Scaling factor: 1.0194
Human-chimpanzee distance: 0.031372
Total primate branch length: 0.1369

ENSG00000090512 exon 1

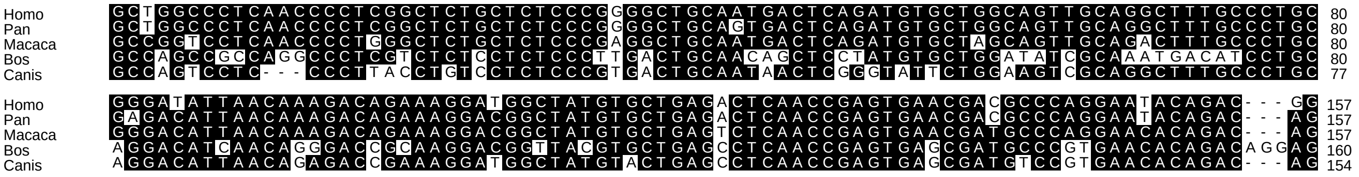

ENSG00000090512 exon 2

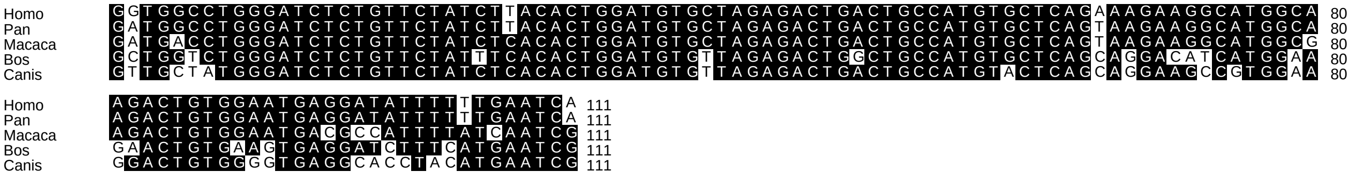

ENSG00000090512 intron 1

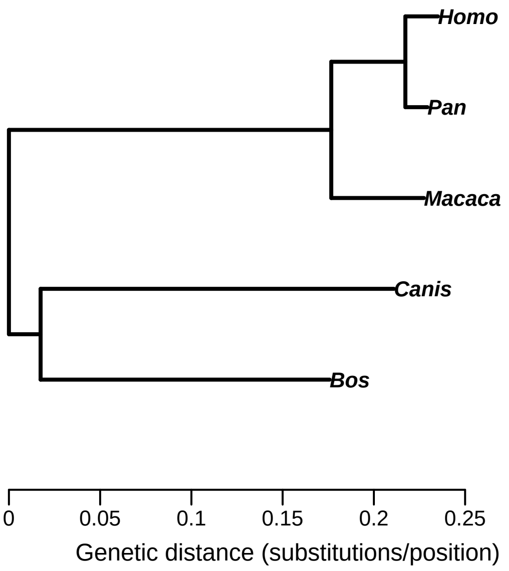

ENSG00000090512 intron 1

```
Homo    GCAAGTAGGGACAGTTCCCACTCTGGGGCAGGGATGTGGGCAAGCTGGAGAGACTGGCCAGGGTGAGGGAACCCAGAGACAC--CCTGGCAGGTGTTTTC 98
Pan     GCAAGTAGTGACGGTTCCCACTCTGGGGCAGGGATGTGGGCAAGCTGGAGAGTCTGGCCAGGGTGAGGGAACCCAGAGACAC--CCTGGCAGGTGTTTTC 98
Macaca  GCAAGTAATGACGGTCCCCACTC--GGGCAGGAATGTGGGCAAGCTGGAGAGACTGGCCAGGGTGAGGGAACCCAGAGACACATGTTGGCAGGTGTTTTC 98
Bos     GTAAGTGGTG---GTCCCC--GCCGGGGCAGGTGTTTGCACCAGCTGGGGAGGCTGGCCAGGGCAAGGGAACCCAGAGGCACCTGTTGGCTGGTGCTTTC 95
Canis   GCAAGTAATG---GTCCCCTATCCAGGACAGGTGCTTGCTGAAGTTGAGGAGGCCAACCAGGGGATGGGAACCCAAAGACACATGTTGGCAGGTGCTTTC 97

Homo    TGTATTGGAAGCCCTGCCGAGAACTCTCTGTTCTCCTCTGCCAGGGTCAGCAACAACGCACTTATTGTTAGTAAAAACAAGTGTTAACAGTGATCCTACT 198
Pan     CGTATTGGAAGTCCTGCCGAGAACTCTCTGTTCTCCTCTGCCAGGGTCAGCGACAACGCACTTATTATTAGTAAAAACAAGTGTTAACAGTGATCCTACT 198
Macaca  CGTATTGGAAGCCCTGCCAAGAACACTCTGTCATCCTCTGCCAGGGTCAGCGACAATGCACTTATTGTTAATAAAAACACATGTTAACAGTGATCCTACT 198
Bos     TGAGTCAGAAACCCTGCTGAGTACATGCTGCTCTCCTCTGCTAGGGT--GCTACTGCCT-CTTAGTAGTATTAGAAACAAGTGCTAATGGT--ATCCACT 190
Canis   CAAATCGGAAACCCTGCTAAGAACGTTCCTCTATTCTCTGCCAGTGT--GTT-CTATGT-GTTTTTACTGTT----TTTTGTGCTAATAGTTCTCCTACT 189

Homo    CTGACACTTGTGTCAGATTTAATGGTTTACGGGGAATCTTCACAAATATT-----CTTTGGCTTTGCCATAAAGCAGGGGTTTT--TTTGTGTGGTCTTT 291
Pan     CTGACACTTGTGTCAGATTTAATGGTTTACGGGGCATCTTCACAAACATT-----CTTTGGCTTTGCCATAAAGCAGGGGTTTT--TTTGTGTGGTCTTT 291
Macaca  CTGACACTTGTGTCAGATTCAGCAGTTTACAGGGTATCTCCACAAACATC-----CTTTGGCTTTCCCATAAAGCAGGGTTTTTTGTGTGTGTGGTCTTT 293
Bos     CTGAAACTT-----GTGTCCCATCATTTATAGGAACACTTCACAAACATCAGAGCCTTTGGCTTTGCCATAAAACAGAAAGTTT--CTTGGCTGGTCGTT 283
Canis   CTGACACTTGTCTAGTATCCCACAGGTTATCAGAAATCTTCACAAACATCACAGAGTTTGGCTTTGTCATAAAAGAGAGA-CTT--TTTGGCCAGTCTTT 286

Homo    CTAGGTTTCTACTTCCTGTGAAATGTATTGCATTTCTCTACCCCTTCCCACAG 344
Pan     CTGGGTTTCTACTTCCTGTGAAATGTATTGCATTTCTCTACCCCTTCCCACAG 344
Macaca  CTCGGTTTCTGCTTCCTGTGAAATGTGTTGTATTTCTCTA-CCCTTCCCACAG 345
Bos     AGAAGTTTCTGCTTCATGTGAGCTG---TGCATTTCTCTTCCCCCTTGCACAG 333
Canis   AAGGGTTTCTGCTTCATGTGGGATGCGATGCATTTCTCAACCCCCTTCCACAG 339
```

ENSG00000100906 intron 5

Description: NF-kappa-B inhibitor alpha (NFKBIA)
Intron number: 5
Human chromosome: 14
Intron start (bp): 34941018
Human intron length : 333
Intron alignment length: 414
Flanking exons length (upstream/downstream): 270/48
SNP density: 0.021021
K tree score: 0.0695
Scaling factor: 0.9136
Human-chimpanzee distance: 0.013433
Total primate branch length: 0.1289

ENSG00000100906 exon 5

```
Homo    GGGCTATTCTCCCTACCAGCTCACCTGGGGCCGCCCAAGCACCCGGATACAGCAGCAGCTGGGCCAGCTGACACTAGAAA 80
Pan     GGGCTATTCTCCCTACCAGCTCACCTGGGGCCGCCCAAGCACCCGGATACAGCAGCAGCTGGGCCAGCTGACACTAGAAA 80
Macaca  GGGCTATTCTCCCTACCAGCTCACCTGGGGCCGCCCAAGCACCCGGATACAGCAGCAGCTGGGCCAGCTGACACTGGAAA 80
Bos     GGGCTACTCCCCGTACCAGCTCACGTGGGGCCGCCCCAGCACCCGGATACAGCAGCAGCTGGGCCAGCTGACCCTAGAGA 80
Canis   GGGCTACTCCCCGTACCAGCTCACGTGGGGCCGCCCAAGCACACGGATACAGCAGCAGCTGGGCCAGCTGACCCTAGAAA 80

Homo    ACCTTCAGATGCTGCCAGAGAGTGAGGATGAGGAGAGCTATGACACAGAGTCAGAGTTCACGGAGTTCACAGAGGACGAG 160
Pan     ACCTTCAGATGCTGCCAGAGAGTGAGGATGAGGAGAGCTATGACACAGAGTCAGAGTTCACGGAGTTCACAGAGGACGAG 160
Macaca  ACCTTCAGATGCTGCCGGAGAGTGAGGATGAGGAGAGCTATGACACAGAGTCAGAGTTCACGGAGTTCACGGAGGATGAG 160
Bos     ACCTTCAGACACTGCCGGAGAGCGAGGATGAGGAGAGCTATGACACCGAATCA---------GAGTTCACAGAGGACGAG 151
Canis   ACCTTCAGATGCTGCCAGAGAGTGAGGATGAGGAGAGCTATGACACAGAGTCA---------GAGTTCACAGAGGACGAG 151
```

ENSG00000100906 exon 6

```
Homo    CTGCCCTATGATGACTGTGTGTTTGGAGGCCAGCGTCTGACGTTATGA 48
Pan     CTGCCCTATGATGACTGTGTGTTTGGAGGCCAGCGTCTGACGTTATGA 48
Macaca  CTGCCCTATGACGACTGTGTGTTTGGAGGCCAGCGTCTGACATTATGA 48
Bos     CTTCCCTACGATGACTGTGTGCTTGGAGGCCAGCGTCTGACGTTATGA 48
Canis   CTGCCCTATGATGACTGTGTGCTTGGAGGCCAGCGCTTGACGTTATGA 48
```

ENSG00000100906 intron 5

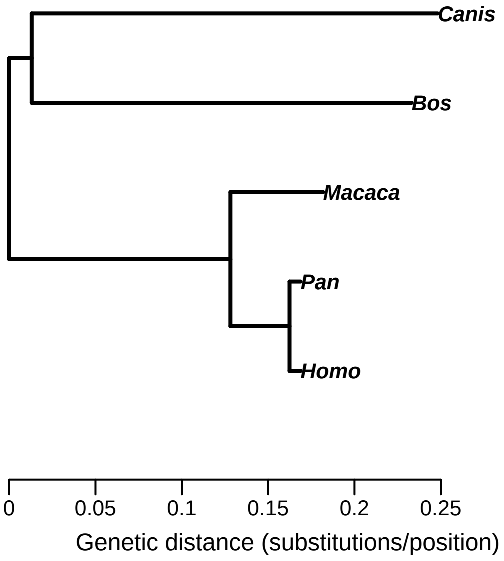

ENSG00000100906 intron 5

```
Homo    GTGAGTCT--GTGAACTCCTTCGGCGCTCT---------AACTAATGAGGTGCCATTCCCTTCACCCTCCCAGGCCCCTAGAGCTGCTCCTTATCAGAGG 89
Pan     GTGAGTCT--GTGAACTCCTTCGTTGCTCT---------AACTAATGAGGTGCCATTCCCTTCACCCTCCCAGGCCCCTAGAGCTGCTCCTTATCAGAGG 89
Macaca  GTGAGTCT--GTGAACTCCTTGGGCTCTCT---------AACTAATGAGGGACCATTCCCTTCACCCTCCCGGGCCCCTAGAACTGCTCCTTATCAGAGG 89
Bos     GTGAGTCCCAATTAACTTGTTCATGGCTCTGCAAGAAGGAAGAAGAGAGGCCATTCTCCCTCCCCCTTCTAAGGCTCCTAGAGTTCCTCCTTATCCTGAG 100
Canis   GTGAG--T--GTCAACTCCATCG---CTCT---------GCACAAAGCAGT------------ACCCTCCCAGGCCTCTAGAGCTCCTCTTTATCCAAAG 72

Homo    GG-TATCTACATAATGAGTCTCTCAAATTT--CTGTGCATAACC---AGTATCCCAAGAATGTACCTGCCCCCC-----------------------CTC 160
Pan     GGTTATCTACATAATGAGTCTCTCAAATTT--CTGTGCATAACC---AGTATCCCAAGAATGTACCTGCCCCCCCCCCCTTTTTTTTAAATAGGTTACTC 184
Macaca  GG-TATCTACATAATGAGTCTCTCAAATTT--CTGTGCATAACC---AGTATCCCAATAATGTACCTTTTTTTT--------TTTTTAAAATAGCTTACC 175
Bos     GG-TCTCAATGTAATGAGCCTCTCAAGCTTGAGTATGTGTAAGC---AGAATCCCAAGAGCATGTTTTTTTTTT------------------TTTTTTGC 178
Canis   AGCTCAATGCAATGAAGTTCTCCCACTTTA--GTGTTCATGACCAGGAGTCTCCCAAAAGTGTGTCTTTGTTTT----------------------TTGC 148

Homo    TTTTTTTAAAGAGAAGCA---------------------------TTAAAGGCAAACTCTCTCAGCCTGTAAAGTTCA-TTATATTTGGGCTATGGAGAA 232
Pan     TTTTTTTAAAGAGAAGCA---------------------------TTAAAGGCAAACTCCCTCAGCCTGTAAAGTTCA-TTATATTTGGGCTATGGAGAA 256
Macaca  CTTTTTTAAAGAAAAGCATTTTTTAAAACATTTTTAAAATGGGAGTTAAAGGCAAACTCCCTCAGCCTGTAAAGTTCA-TTGTATTTGGGCTATGGAGAA 274
Bos     TCTTCTTGAAGGAGAGGA------------------------TCCTTAAAGGAAAGCTCCCTCAGCCCAGAAAATTCATTTACATTCAGTGTATAGAGAA 254
Canis   TCTTTTTAAAGGAGAGGA------------------------GCATTAAAGAAAAACTTCCTTAGCCCCTGAGGTTAATTTATACTTGGGTTATGGAGAA 224

Homo    TGGAGTCCAA--------GAGTTATTTCCAGTAGTGGCC-TCCC---CATCCCGGTAGCTTGGCAGAGCTCCGCTCCGGAAGCTTAACGT-GTCTTTTTT 319
Pan     TGGAGTCCAA--------GAGTTATTTCCAGTAGTGGCC-TCCC---CATCCCAGTAGCTTGGCAGA-----GCTCCGGAAGCTTAACGT-GTCTTTTTT 338
Macaca  TGGAGTCCAA--------GGGTTATTTCCAGTAGCGGCC-TCCC---CATCCCAGTGGCTTGGCAGA-----GCTTCGGAAGTTTAATGT-GTCTTCTTT 356
Bos     TGGAGTCCAAGAACACTTGAGTTATTTCCAGTAGCAGCCCTCCCCATCATCCCAGCACCCTTGGCAA----AGCCCTGCGAGTTTAACCTGCCTTTTTTT 350
Canis   TGGAATCCAA--------GAGTACTTGATAATGGCCCTCACCCT---TATCCCAGTACCTTTGACAA------AACTGGAAGCTTAACATGGCTTTTTTT 307

Homo    CCCCTTGTTTTCAG 333
Pan     CCCCTTGTTTTCAG 352
Macaca  CCCCTTGTTTTCAG 370
Bos     TCTC---TTTTTAG 361
Canis   TCTCTTGTTTTTAG 321
```

ENSG00000105669 intron 6

Description: Coatomer subunit epsilon (COPE)
Intron number: 6
Human chromosome: 19
Intron start (bp): 18875233
Human intron length : 1347
Intron alignment length: 1447
Flanking exons length (upstream/downstream): 82/156
SNP density: 0.001485
K tree score: 0.0912
Scaling factor: 0.3946
Human-chimpanzee distance: 0.007479
Total primate branch length: 0.1285

ENSG00000105669 exon 6

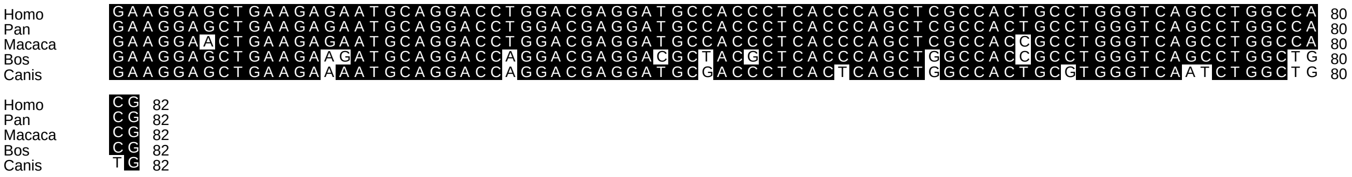

ENSG00000105669 exon 7

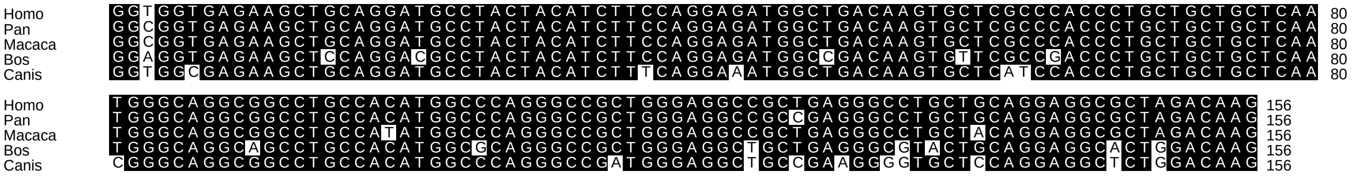

ENSG00000105669 intron 6

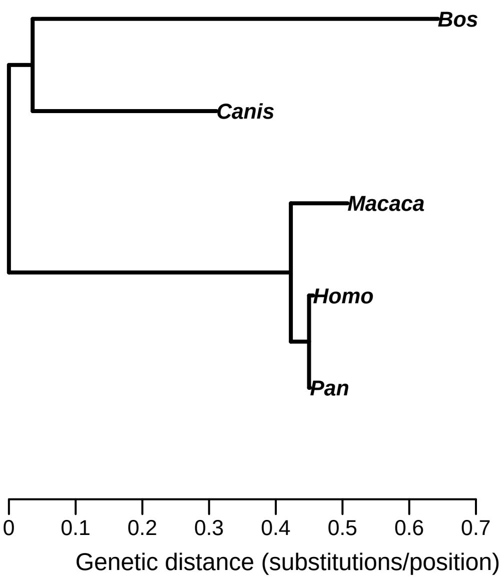

ENSG00000105669 intron 6

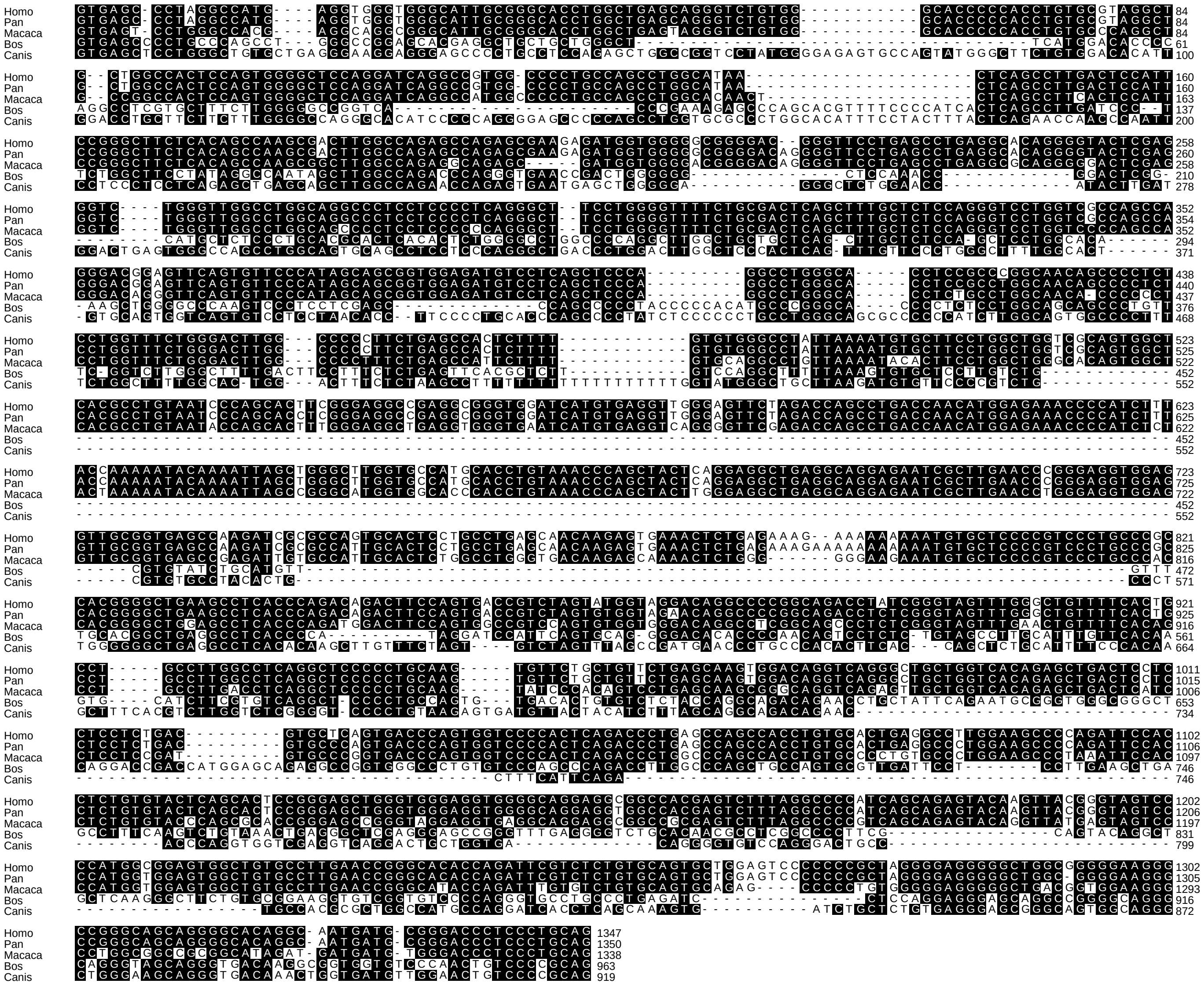

ENSG00000165695 intron 6

Description: Putative adenylate kinase-like protein C9orf98 (C9orf98)
Intron number: 6
Human chromosome: 09
Intron start (bp): 134693293
Human intron length : 560
Intron alignment length: 677
Flanking exons length (upstream/downstream): 82/72
SNP density: 0.001786
K tree score: 0.0973
Scaling factor: 0.7677
Human-chimpanzee distance: 0.016952
Total primate branch length: 0.1279

ENSG00000165695 exon 6

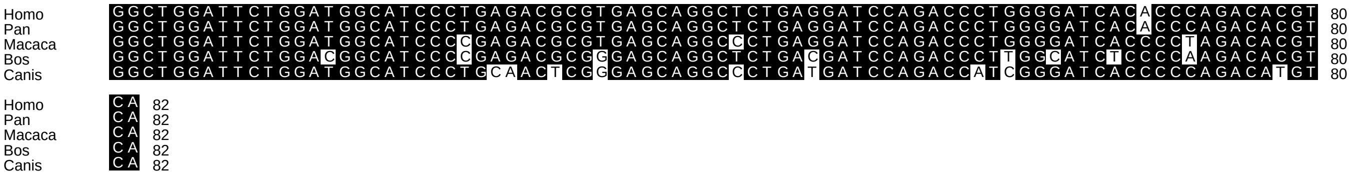

ENSG00000165695 exon 7

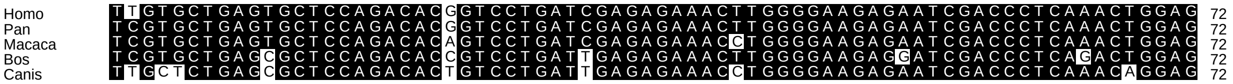

ENSG00000165695 intron 6

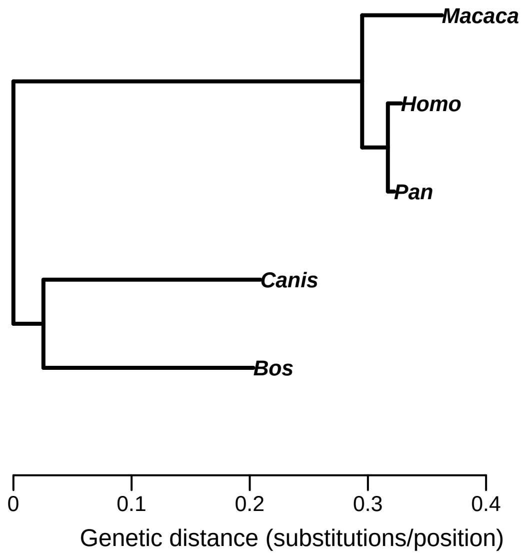

ENSG00000165695 intron 6

```
Homo    GTGAGTAGCTCCCAAGGGTCCCCCACCTGCCAGAGCTGCAGCCCCAAGGGGTGGCCAGTGAGCGCCCCGCTCATGCTG------------CCTGCTCCTG 88
Pan     GTGAGTAGCTCCCAAGGGTCCCCCACCTGCCAGAGCTGCAGCCCCAAGGGGTGGCCAGTGAGCGCCCCACTCATGCTG------------CCTGCTCCTG 88
Macaca  GTGAGTAGCTCCCAAGGGTCCCCCACCTGCCACAGCTGCAGCCCCAAGGGGTGGCCAGTGAGCACCCCGCTCATGCCG------------CCTGCTCCCG 88
Bos     GTGAGTAGC------------CCCGCCCACCAGAGCTG-------CAGACCCTGAGAGTGAGTGCCCAGCCTGTGCTGTGTCAGCGGTCCTCAGTGCCC- 80
Canis   GTGAGTAGCTTGCGAAGG--GCCCTCCCACCAGAGCTGCAGTCCCCAGACCCCAGGAGTGAGGAC----CCTGCTCTGTGTTGCTGGTTCCCGGTGCCCA 94

Homo    ACCTCAGCCCAGG--CACCGTCTGA---------------------GACCCAGCAAGG---AAGAGGACAGCACAACTGAATTTTTCACAGAGCTAAATA 162
Pan     ACCTCAGCCCAGG--CACCGTCTGA---------------------GACCCAGCAAGG---AAGAGGACAGCACAACTGAATTTTTCACAGAGCTAAATA 162
Macaca  ACCTCAGCCCAGG--CACTGTCCGA---------------------GACCCAGCAAGG---AAGAGGACAGCACAGCTGAATTTTTCACAGAGCTAACTA 162
Bos     CCCTCAGCCCAGGGCCACCATCCAGCAGAGGGGTTGATGGGCCTGTGACCCGGCAGGAACCAGGAGGACCGCTCAGCCGAGTTCTCCA-AGAGCTAAATG 179
Canis   CTCTCAGCCCCA---TACCATCCAA---------------------GACCCAGCAGGAAAGAGGAGGACAGCACAGTTGAATTTTTCACAAAGCTAAAGG 170

Homo    TATTCAGGGCTGGTTCTTAAGAAAAGTGGGGTTTCGTTTTCAGCCTGGCGATTCCTTGTCTCAGGAA--------------------------------- 229
Pan     TATTCAGGGCTGGTTCTTAAGAAAAGTGGGGTTTTGTTTTCAGCCTGGCGATTCCTTGTCTCAGGAA--------------------------------- 229
Macaca  TATTCAGGGCTGGTTCTTAAGAAAAGTGGGGTTTTGCTTTCAGCCTGGTGATTCCTTGTCCCAGGAA--------------------------------- 229
Bos     CACTCGGGGCTTGTGCTG-AGGAACCTGGGGTCTTGCTCTGACCCTGGGGATTCCCTGTTTCAGGAAGCCCCCATCTCAACCATGGGAAACTCTGAGAAA 278
Canis   CCTTCAGGGATTGCTCTT-AGAGAAAGGGGGTTTTGCTTTGGCCCTGGTGGTTCCTTGTTGCAGGAATCCC----------------------------- 240

Homo    ----------------------GGAAATGCATATTCTATGCATAGCATAGAATGCTGTGCAAGCATTTAGTGCGCACAGAGATGCCCATTGTGGCGTTAT 307
Pan     ----------------------GGAAATGCATATTCTATGCATAGCATAGAATGCTATGCAAGCATTTAGTGCGCACAGAGATGCCCATTGTGGCGTTAT 307
Macaca  ----------------------GAAAATCAAC---------------TAGAATGCTATGCAGGCGTTCAGTGCACGCAGAGATGCCCATCATGGCGTTAT 292
Bos     CCACTGGAAGCTGGAAACCACTGAGAAGCTCTCTATAGGAAATAATCCCAAGTGTGGCAAAAGCCTTC----TGTACAGGGATGT------CACAGTTAT 368
Canis   ----------------ATTTCTGAGAAACTCTCTTTAGGAAATAATCCAAAATATGGCAAGAACATTC----TGCACAAGAGCAT------TACAG---- 310

Homo    CTGTGACGAGGGAAATTGTTAACAGGCAGAACTTTC--------------------CGTCATGGGCCGTGCTCTGTCCCAACGTTACACTCTTTGTGTTT 387
Pan     CTGTGACGAGGGAAATTGTTAACAGGCAGAACTTTCCGTCACGGGCCGTGCTCTGTCGTCACGGGCCGTGCTCTGTCCCGACGTTACGCTGTTTGTGTTT 407
Macaca  CTGTGACGAGGGAAATTGCTAACAGGCAGAACTTTCAGTCATGGGCCTGGCTCTGTC--CCGAGGTTACACTCTGTCCCGATGTTACGCTCTTTGTGTTT 390
Bos     TGGTGGCCCTGAAGGCGGTCGGCAGGCAGTGTTCGTG----------------------CATGGATGACCT-CTGTCCTGAGATTCCATGTCTGTGTTCT 445
Canis   ---------TGAAAATTGTTAG-ATGAGATGTTCAAG----------------------TGTGGGCTGTCTGCTGTCCTGAGACTGAAGATCT------- 371

Homo    GGGTGCGAGAAGACCTTTCTCTTAGGAATTCACGGGGATGGGAGATGATTGTTGTTTTAATGTCATTATACAG----AAAAGCAAAAAACGTAAAGCAAG 483
Pan     GGGTGCGAGAAGACCTTTCTCTTAGGAATTCACGGGGATGGGAGATGATTGTTGTTTTAATGTCATTATACAG----AAAAGCAAAAAACCTAAAGCAAG 503
Macaca  GAGTGCAAGAAGACCTTTCTCTTAGGAAATCATGGGCATGGGAGATGATTGTTGTTTTAATGTCATTATACAGAAAAAAAAAAAAAAAACCTAAAGCAAG 490
Bos     GA----------CCTCTCTCTTAAGGGGTTGATGGGTAT-GGAGGTGGTGGGTGTTTTAGTGT-------------------------------------- 497
Canis   ------------------CTCGGAGGGATTCTCAGGGGT-GGGGGTGGGTGTTGTGTTAGTGTTGTTACCCAGCAAGAGAGACATAAGACA------AGC 446

Homo    TATTGGTCTGTGATATTCAAATGTGTGGCACCCGCTGCCCGACATGCCTGGTTTTGCTCCTTTTCTTCTCTGCCCAG 560
Pan     TATTGGTCCGTGATATTCAAATGTGTGGCACCCGCTGCCCGACATGCCTGGTTTTGCTCCTTTTCTTCTCTGCCCAG 580
Macaca  TATTGGTCCGTGCTATTCATATGTGTGGCACCCGCTGCCTGACATGCCTGCTTTT-CTCCTTTTCTTCTCTGTCCAG 566
Bos     ------------------------------CCTGCTGCCCACTGTGTCTGGTTTGGCTTCTTTCCTTCTCTGCCCAG 544
Canis   TATTGGCCCATGAGATCCAAATGTTTGGGACCTGCTACCTGACATGTC-AGTTGTGCTGTTGTCCTTCTCCATCCAG 522
```

ENSG00000175315 intron 1

Description: Cystatin M precursor (CST6)
Intron number: 1
Human chromosome: 11
Intron start (bp): 65536332
Human intron length : 541
Intron alignment length: 761
Flanking exons length (upstream/downstream): 240/126
SNP density: 0.009242
K tree score: 0.0617
Scaling factor: 0.45
Human-chimpanzee distance: 0.019419
Total primate branch length: 0.1253

ENSG00000175315 exon 1

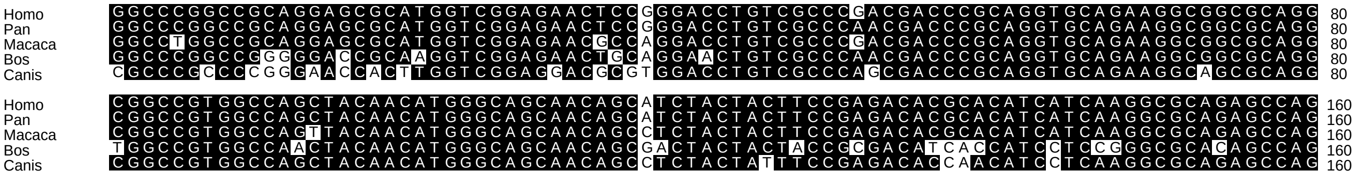

ENSG00000175315 exon 2

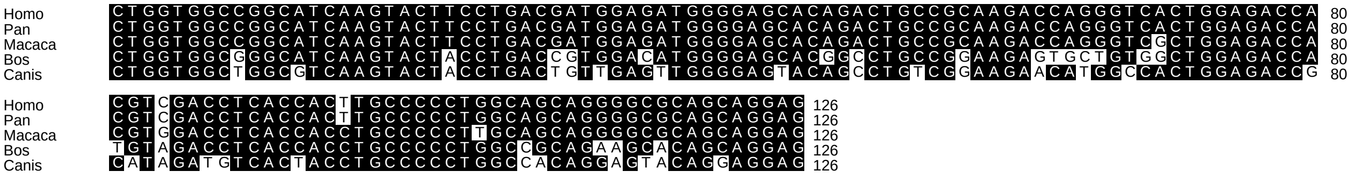

ENSG00000175315 intron 1

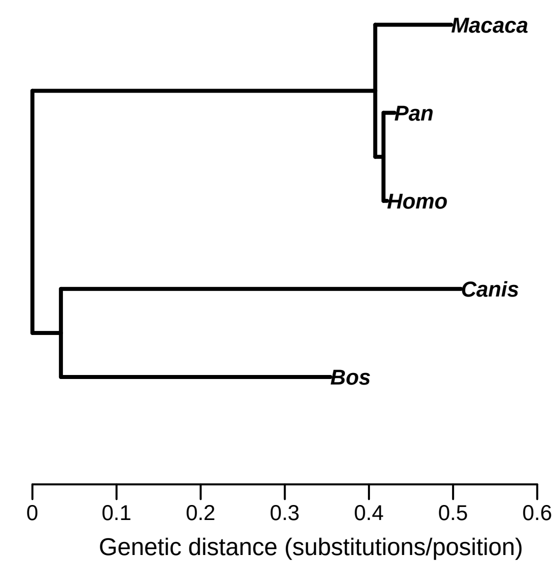

ENSG00000175315 intron 1

```
Homo    GTGC--GGCGGGCGGGGTG-----------------------CTGGGAGGGGACACCCGGCCCAGATGGGGGA----------GGCCACAGGCGCTGCCC 65
Pan     GTGC--GGCGGGCGGGGTG-----------------------CTGGGAGGGGACACCCGCCCCAGATAGGGGA----------GGCAACAGGCGCTGCCC 65
Macaca  GTGC--GGCGGGCGGGGTG-----------------------CTGGCAGGGGATA-CTGGCCCAGGCGGGGGA----------GGCCACAGGCGCTGCCC 64
Bos     GTGCGGGGCGGGCGGGCCGGGCCGGGGAAGGGGCGGCGCCGACGGGGGGAGGGGGGACGGAGCGGACGGGGGAGAGGGGGCGGGGAGGCGAGCGCGGCCC 100
Canis   GTGT--GCAGGGGGGAGGAGGGGGGGCGTGGGACAGGGGCGAGGGGTGGGGTGGTGGGGCGCCGGCTAGGGCA------------AGACCAGTGCGGCAT 86

Homo    CAG------CGTGCATGAAGGGGGCCTAA-AAGCGCAATCGG---GATATTTTCATGCAATA--TTTTAAAAATCGAATTAATGCTAAAACTCCACGATG 153
Pan     CAG------CGTGCATGAAGGGGGCCTAA-AAGCGCAATCGG---GATATTTTCATGCAATA--TTTTAAAAATCGAATTAATGCTAAAACTCCACGATG 153
Macaca  GGG------CTTGCATGGAGGGGGCCCGA-AAGCGCTACCAG---GATATTCTCATGCAATA--TTTAAAAAATCGAATTAATGCCAAAACTCCACAATG 152
Bos     CAGCCGGGCCCCAGATGGAGGGGGCGACT-AAGAGTAATCAGGGCGGCATCTTAATACAACA-GTTTTCAAAATCCAATTAATGTCAAAAGTCCATGCGG 198
Canis   TCGCAGGTGACCAGGTTAAAGGGATGCCACGAGAGTATTCAG---GACACTTTAATACAATGTATTTTTTTAGTTCAATTAATGCCACAAATTTATGAAC 183

Homo    GACA-ACCATCTAAATTTCAAGGAAAGACATAATTAAACCCTGTAT----TGCACTACCTGCCTTCCTGGCTTGCCTGGTCCTAGTCTCTGCCCTGATGG 248
Pan     GACA-ACCATCTAAATTTCAAGGAAAGACATAATTAAACCCTGTAT----TGCACTACCTGCCTTCCTGGCTTGCCTGGTCCTAGTCTCTGCCCTGATGG 248
Macaca  GGCA-GCCATCTAAATTTCAAGGAAAGACATCATTCAATCCTGTATTGCATGCACTACCTGCCTTCCTGGCTTGCCTGGTCCTAGTCTCTGCCCTGATGG 251
Bos     AGCAGAATCTCAGAATTTCAAATAAGG-----TTCAACCCTGAAT-------------------------CGGCCTGGTCCTGA--------------AA 254
Canis   AACA---CATCGATATTTCAATAGGA------TCTAGCCCTGACT----TACACAGCGGCTCGCTAGCCTCAAGGTGCTCCTGCTCTGG---------GG 261

Homo    GGTGGATCGGGGAGGAGGGAAGGCAGGTGGGGACAGTGGGCAGCTCCCTAGATGGGGCATGTCTGATGTCT---GCCAAGGCTGCTGGTGGGGCTCAGCC 345
Pan     GGTGGATCAGGGAGGAGGGAAGGCAGGTGGGGACAGTGGGCAGCTCCCTAGATGGGGCATGTCTGATGTCT---GCCAAGGCTGCTGGTGGGGCTCAGCC 345
Macaca  GGTGGATCGGGGAGAAGGGAAGGCAGATGGGGACAGTGGGCAGTTCCCCAGGTGGGGCATGTCTGATGTCT---GCCCAGGCTGCTGGTGGGGCTCAGCC 348
Bos     GGCGGATTGGGGTGCGATGGGGTGA----GGGAGAAGGGGCAGCGGTGTGGACTTGA-GTCTCAGGCTCCT---GCCT-------------GGCCCAGGC 333
Canis   GGTGGATTGGGGAGACGGGAGTGA-----GGGAGATAGGGCAGTG----GGCTTCAGGATGCCTGCACCCCTGGGTCTCAGCTTCCTGCGTGGCCCAGGC 352

Homo    TCCAGGCCTCTCCACCCTCCATCCCCACCAGAACTCCCCCCCCCACCCCCCCCCACCCCCCCCACCCCGTCTGAATCATCCCCTCTCCCTCTCTCCCCA 445
Pan     TCCAGGCCTCTCCACCCTCCATCCCCACCAGAACT-------------TCCCCCACCCCCCACCCCCCCATCTGAATCATCCCCTCTCCCTCTCTCCCCA 432
Macaca  TCCAGGCCTCTCCTCCCTCCACACCCACCAGAACT-------------TCCACCA--CCCGGACCCCAGGTCTGAATCATTCCTTCCCCCTCTCTCCCCA 433
Bos     TCTGCCCGCCTCACCACAGGACCCCCACCAGAACT-------------CCCTGCTGCCCTCTCCCCGCCTTTCCAGTCCCCCCATCTCAGGTTTGAATCC 420
Canis   CTCTGCTTGTCTTCCCAGGAATCCCCACCAGAACT----------------------TCCCCATCCAGGGTCTGAATC--------------CCGAGGCC 416

Homo    CCTGAGTGCCTGGGGGCAA----CAGGAGAATATATCAAGAGGTGAGAAGTGAGGATCAAGGG---TCAAG----------------------------- 509
Pan     CCTGAGTGCCTGGGGGCAA----CAGGAGAATATATCAAGAGGTGAGAAGTGACGATCAAGGG---TCAAG----------------------------- 496
Macaca  CCTGAGTGCCTGGGGGCAA----CAGGAGAATATGCTGGGAGGTGAGAAGTGAGGATCAAGGG---TCAAG----------------------------- 497
Bos     AAAGGCGGTGTGCTGGCTATGCCCAGGGGAAGGTGATGGGTGATGGGGGGACACGGAGGGAGG---TGAGANNNNNNNNNNNNNNNNNNNNNNNNNNNNN 517
Canis   CAGGCCAGTGTGCT--------------GAATGTGGAGAGAGGTGAGAGGTCCGGGTCAAGGGCATCCAGA----------------------------- 473

Homo    ---------------------------------------------------------------------------------------------------- 509
Pan     ---------------------------------------------------------------------------------------------------- 496
Macaca  ---------------------------------------------------------------------------------------------------- 497
Bos     NNNNNNNNNNNNNNNNNNNNNNNNNNNNNNNNNNNNNNNNNNNNNNNNNNNNNNNNNNNNNNNNNNNNNNNNNNNNNNNNNNNNNNNNNNNNNNNNNNNN 617
Canis   ---------------------------------------------------------------------------------------------------- 473

Homo    -----------------------------ACCCCTGACCTGCCCCTACCCTATGCCCCCAG 541
Pan     -----------------------------ACCCCTGACCTGCCCCTACCCTATGTCCCCAG 528
Macaca  -----------------------------ACCCCCGACCTGCCCCTACCCTATGTCCCCAG 529
Bos     NNNNNNNNNNNNNNNNNNNNNNNNNNNNNNNNNNNNNNNCACCTCTG--------CCCCAG 670
Canis   -----------------------------ATCCCTGACCTGCTCCTGTCCTCCATCCCCAG 505
```

ENSG00000162891 intron 3

Description: Interleukin-20 precursor (IL20)
Intron number: 3
Human chromosome: 01
Intron start (bp): 205106605
Human intron length : 747
Intron alignment length: 780
Flanking exons length (upstream/downstream): 153/75
SNP density: 0.017403
K tree score: 0.0918
Scaling factor: 0.8703
Human-chimpanzee distance: 0.009901
Total primate branch length: 0.1247

ENSG00000162891 exon 3

```
Homo    CCTGCGAATCGATGCTGCCTCCTGCGCCATTTGCTAAGACTCTATCTGGACAGGGTATTTAAAAACTACCAGACCCCTGA  80
Pan     CCTGCGAATCGATGCTGCCTCCTGCGCCATTTGCTAAGACTGTATCTGGACAGGGTATTTAAAAACTACCAGACCCCTGA  80
Macaca  CCTGCGGATCAGTGCTGTCTTCTACGCCATTTGCTACGACTCTATCTGGACAGAGTATTTAAAAACTACCAGACCCCTGA  80
Bos     CCTGCAGATCAGTGCTGCCTCCTCCATCATATACTGAGACTTTACCTGGACAGGGTATTCAAAAACTATCAACCCCCTGA  80
Canis   CCTGCAGATCAGTGCTGCCTCCTTCGCCACGTACTGAGACTCTACCTGGACAGGGTATTCAAAAACTATCAGACTCCTGA  80

Homo    CCATTATACTCTCCGGAAGATCAGCAGCCTCGCCAATTCCTTTCTTACCATCAAGAAGGACCTCCGGCTCTGT  153
Pan     CCATTATACTCTCCGGAAGATCAGCAGCCTTGCCAATTCCTTTCTTACCATCAAGAAGGACCTCCGGCTCTGT  153
Macaca  CCATTATACTCTCCGGAAGATCAGCAGCCTCGCCAATTCCTTTCTTACCATCAAGAAGGACCTCCGGCTCTGT  153
Bos     CCACCATATCTTCCGGAAGGTCAGCCGTCTTGCCAACTCTCTTCTCACCATCAAGAAGGACCTCCGGCTGTGT  153
Canis   CCACCATATCCTCCGGAAGACCAGCAGTCTTGCCAACTCTTTTCTTACCATCAAGAAGGACCTCCGGCTCTGT  153
```

ENSG00000162891 exon 4

```
Homo    CATGCCCACATGACATGCCATTGTGGGGAGGAAGCAATGAAGAAATACAGCCAGATTCTGAGTCACTTTGAAAAG  75
Pan     CATGCCCACATGACATGCCATTGTGGGGAGGAAGCAATGAAGAAATACAGCCAGATTCTGAGTCACTTTGAAAAG  75
Macaca  CATGCCCACATGACATGCCATTGTGGGGAGGAAGCAATGAAGAAATACGGCCAGATTCTGAGTCACTTTGAAGAG  75
Bos     CATGCCCATATGTCATGCCCTTGTGGGGAAGAAGCAAAGGAGAAATACAGCCAGATTCTGAGTCACTTCGAAGAG  75
Canis   CATGCACATATGACATGCTCTTGTGGAGAGGAAGCAACGGAGAAATACAGCCAGATTCTGAGTCACTTCGAAGAG  75
```

ENSG00000162891 intron 3

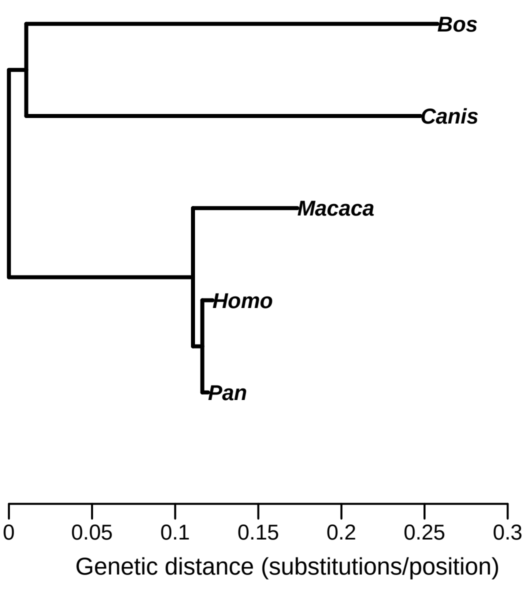

ENSG00000162891 intron 3

```
Homo    GTGAGTGTGGGTCTTGGGTGACAGGATGCATCTCAGCACACAGCTTCAATGGCTTAGCAACTAAACTCTCTTTCCTACCTCCATTTAATGGATGGAGAAA 100
Pan     GTGAGTGTGGGTCTTGGGTGACAGGATGCATCTCAGCACACAGCTTCAATGGCTTAGCAACTAAA--CTCTTTCCTACCTCCATTTAATGGATGGAGAAA 98
Macaca  GTGAGTGTGGATCTTGGGTAACAGGATGCCTCTCAGCACACAGCTTCAATGGCTTAGCAACTAAACTCTCTTTCCTGCCTCCATTTAATGGAGGGAGAAA 100
Bos     GTGAGTATGGGTCTTGGGTAAAAGGATCCACCTCAGCACATA---------ACTTAGCAATCAGG---CTCATCTTAGACCCATTTAATGGATGGAAAAA 88
Canis   GTGAGTAAAGGGTAAAAGCATC-CATTGCTGCTTCATCCATAACTTCAGTGACTTAGCAACCAAG--CTCTTTCTTGCCTCCATTTAATGGATAAAAAAG 97

Homo    CTGAGTCCAAAAGTTCTAA--TAATCTGTGTTGAG--------ACATGTGAC----TAGGTAATAAGAACTCAGTTTTATTGACTTTTCGGTATATGCTC 186
Pan     CTGAGTCCAAAAGTTCTAA--TAATCTGTGTTGAG--------ACATGTGAC----TAGGTAATAAGAACTCAGTTTTATTGACTTTTTGGTATACGCTC 184
Macaca  CTGAGTCCAAAAATTCTAA--TAATCTGTGTTGAGGTTACAACACAGGTTATAATATAGGTTATAATAACTCAGTTTTATTGACTTTTGGGTATATGCTC 198
Bos     CTGAGTTCGAGAGTTCTAAATTAATCTGTGTTGAG--------CTATGTGAC----TAGGTAATAAGAACTCAGTTATATTGACTTTTGGATACATGCTC 176
Canis   CTGAGTCCAAAAGTTCTCA--TAATCTGTCTTGTA--------GTGCGTGAC----TAGCTAATAAGAATTCAGTTTTACTGAGTTTTGGGTATACGCTC 183

Homo    TAGGCAAAAAGTA--CTTTGCAAAGTCTAAAGAACTATAAGATGCTAACTATTTGATATTAATGATAAC--TCTGT---TGTCTTTGAAATTATACTTTT 279
Pan     TAGGCAAAAAGTA--CTTTGCAAAGTCTAAAGAACTATAAGATGCTAACTATTTGATATTAATGATAAC--TCTGT---TGTCTTTGAAATTATACTTTT 277
Macaca  TGGACAAAAAGTACTCTTTGCAAAGTCTAAAGAACTATAAGGTG----CTACTTGATATTAATGATAAC--TCTAT---TATCTTTGAAATTATACTTTT 289
Bos     TATGCAA--------------AATGTCTAAAGAACTATAAAGTGCTGGCTCTTTGATGTCACCAACAATCATCACTATATGTGAATGAAATCATGGTTCT 262
Canis   CATGCAAAAAAAA------AAAAAATCTAAATAACTACAAAATG----TTATTTGGTGTTTATGATAATTATCTAT---TATGCATGAAATTACACTTTT 270

Homo    TCTGTAGGTGAGGTATCCTACAGTATATTAGTGCGTCCTCTGTCTAGGCAGTCAATTAGTAGACCATTGAGCTTGACCTCAGAATATAGTCTGAATAGGA 379
Pan     TCTGTAGGTGAGGTATCCTACAGTATATTAGTGCATCCTCTGTCTAGGCAGTCAATTAGTAGACCATTGAGCTTGACCTCAGAATATAGTCTGAATAGGA 377
Macaca  CCTGTAGGTGAGGTATTCTACAGTATATTAGTGCATCCTCTGTCTGGGCAGTCAATTAACAGACCATTGAACTT------------------------GA 365
Bos     TCTGTGAGT----TATCCTATGGTGTTTAGGGCATCCTGAGCCCAGGCAGCTAATCAGCAGACCACCAGACTCAACCTCAAG------------ATAGGA 347
Canis   -CTGTAGGTGAAGGATCCTACCACATATTAGTGTATCCTGTGTCCAGGTAGCTTCGCAGTAAGCCATTGGACTCGATATCAAGATAAGGTCTGAATAGGA 369

Homo    CCTAGGAATTCAATTCTTTTTTTTTTTTTTCTCATGGGGGCTCAAAGAGCTCTGGGATAGAGCTCCTAGACTACAGCTGGGGGTTGTGGGGAGGCCAGAT 479
Pan     CCTAGGAATTCAATTC---TTTTTTTTTTTCTCATGGGGGCTCAAAGAGCTCTGGGATAGAGCTCCTAGACTACAGCTGGGGGTTGTGGGGAGGCCAGAT 474
Macaca  CCTAGGAATTCAAATT---TCTTTTTTTCCTTCATGGGGACTCAAAGAGCTCTGGGATAGAGCTCCTAGAGTACAGCTGGGGGTTGTGGGGAGGCCAGAT 462
Bos     CCTCAGAACCCAATTCGCTCTTCCCATGACGCTCTCTGGGAGGGAAATAGATTAGAACAGGG-------------------------GTGCAGGCCAGAA 422
Canis   CCTGGGGATCCAA--------------------------------------------------------------------------------------- 382

Homo    GGGGTACGGGGATGGCAAATGCCTTCAGTACTGCCTGCCTATTTCTAAAAAAGAAGTGATGAGTTCCATGTTTGAGCCTA-AAAGGTGGCTTCCTCTCCT 578
Pan     GGGGTACGGGGATGGCAAATGCCTTCAGTACTGCCTGCCTATTTCTAAAAAAGAAGTGATGAGTTCCATGTTTGAGCCTA-AAAGATGGCTTCCTCTCCT 573
Macaca  GGGGTACGGGAACGGCAAATGCCTTCAGCACTGCCTGCCTGTTTCTAAAAAAGAAGCAATGAGTTCCATGTTTGAGCCTA-AAAGGTAGCTTCCTCTCCT 561
Bos     GGGACACAGGGCTCACAAACTCCTTCAGTGCTGCCTG-----TTTCTGAACAGAAGCAACGAGCTCTATATTTGAGCGTAAAACGCTGGCTTCCTTTTCT 517
Canis   --------------------------------------------------------CAAAGAGCCCCATATTTGAGCCTACGAAGCTGGCCTCCTCATCT 426

Homo    AGCTGATGATGAACTTAGTGATTCCAAATG-TGAGGTCTGAAAGAGCTTTTCTATAGGAAT-AAGCATCCTCAGGGTTGTGGGTGAAAGAGTAGAGTTTC 676
Pan     AGCTGATGATGAACTTAATGATTCCAAATG-TGAGGTCTGAAAGAGCTTTTCTATAGGAAT-AAGCATCCTCAGGGTTGTGGGTGAAAGAGTAGAATTTC 671
Macaca  AGCTGATGATGAACTTAATGATTCCAAATG-TGAGGTCTGAAAGAGCTTCTCTATAGGAAT-AAGCATCCTCAGGGTTGTGGGTGAAAGAGTATAATTTC 659
Bos     AGCTGATGTACACTTTAGTGATCCCAAATAAAGAAGTATGAAAAGATT--TCTACAGGTG------------------------GAAAGAACAGAACTCC 591
Canis   GGTTGATCATTAGTTTTATAATCTCAAATGATGAGGTCTGAAAAGACTT-TCTATAGGAATGGAGTATGTACAAGAATGCAGATGAAAGAG------TTC 519

Homo    TGCCTGCTTCATGTCAAT------GGCAAAAAATCAGAATCTGTAAT--ATAATCTATTATTCTTTGG-GTCCTTTTCAG 747
Pan     TACCTGCTTCATGTCAAT------GGCAAAAAATCAGAATCTGTAAT--ATAATCTATTATTCTTTGG-GTCCTTTTCAG 742
Macaca  TACCTGCTTCATGTCAATGGCAAGGGCAAAAAATCAGAATCTGTAAT--ATAATCTATTACTCTTTGG-GTCCCTCCCAG 736
Bos     TACCTACTTCATGTCAAT------GGCAGAAGATTGCAATCTTTAAT--AACATCTATCATTCTTTGGTATTCTTTTCAG 663
Canis   TCCTTACTTTATGACAGTGGCAAGGGCAGAAAATCAGAATCTTTAATAAAAAATCTATTATTCTGTGGTATTCTTTTCAG 599
```

ENSG00000167900 intron 5

Description: Thymidine kinase, cytosolic (TK1)
Intron number: 5
Human chromosome: 17
Intron start (bp): 73682846
Human intron length : 359
Intron alignment length: 390
Flanking exons length (upstream/downstream): 90/120
SNP density: 0.008357
K tree score: 0.0577
Scaling factor: 0.4485
Human-chimpanzee distance: 0.019910
Total primate branch length: 0.1241

ENSG00000167900 exon 5

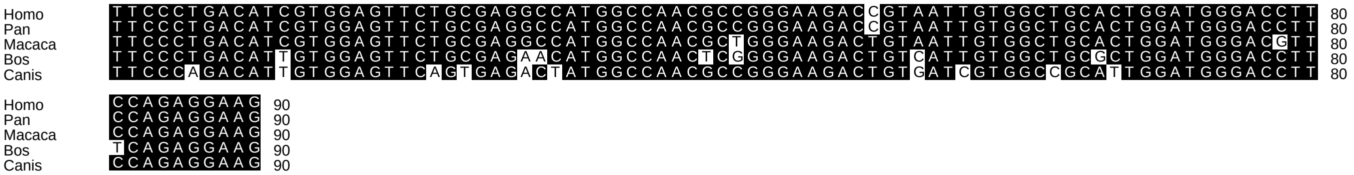

ENSG00000167900 exon 6

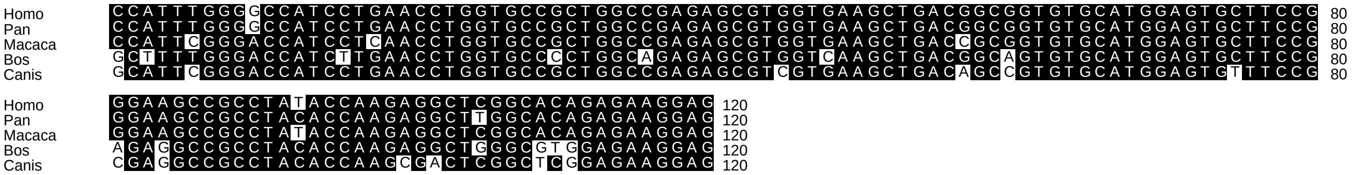

ENSG00000167900 intron 5

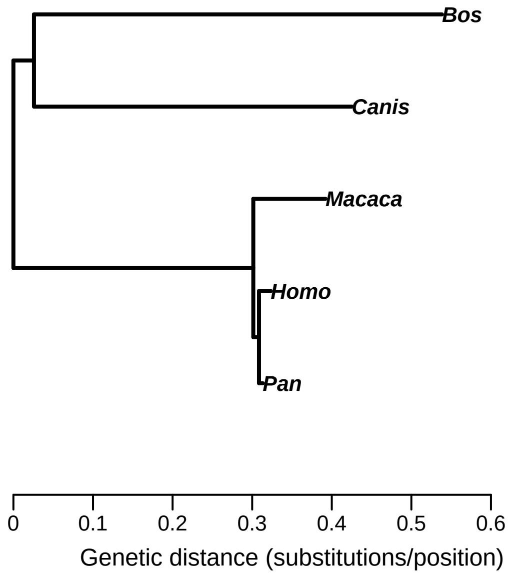

ENSG00000167900 intron 5

```
Homo    GTAAGGCGTCTGATCCAGGTCTGGAGCTGGGATTGAGGAGGGCAAG--AGGCTTCTGGATGGGCACAGAGACACC---------------AGCTCTGGGT 83
Pan     GTAAGGCGTCTGATCCAGGTCTGGAGCTGGGATCAAGGAGGGCAAG--AGGCTTCTGGATGGGCACAGAGACACC---------------AGCTCTGGGT 83
Macaca  GTAAGGCGTCTGATCCAGGTCTGGAGCTGGGATCCAGGAGGGGAAG--GGGCTTCTGGGGGGGTACACAGACACC---------------AGCTCTGGGT 83
Bos     GTGAAGTGTCTGGTCCAGGTCTTAGGGTGGGTCCGGGGACCTA-----GGGGCCTGGGCAGTGCACAGAAATGGA-----CAGCCCAGCGTGCCCTGGAT 90
Canis   GTGAGGGGTCTGAGCTGGGCCTTGGGAAGGGATCGGGAGGGAGGGGGCGAGGGGTGGGGCAGGAACAGGGCCTCCTCTCTCAGCACAGTGCTCCCTGGAT 100

Homo    GACCAGGGCTCAGCCACCACAGGGTTACGGCCGAGCTGCT-----CAGGCCTTGGCTGAGCCAAGGGACTC---CATGGTCTGTGCAG---ACTGCGTGC 172
Pan     GACCAGGGCTCAGCCACCACAGGGGTACGGCCGAGCTGCT-----CAGGCCTTGGCTGAGCCGAGGGACTC---CATGGTCTGTGCAG---ACTGCGTGC 172
Macaca  GACCAGGGCTCAGCCACCACAAGGGTATGGCCGAGCTGCTCAGACCAGGCCTTGTCTGAGCCCAGGGACTC---CGTGGTCTGTGCAG---ACTG-GTTC 176
Bos     GCC------------------------------------------CACACCATGCCCAAGCCCAGGCACTCGGGCCAGCCAGTCCCAGCTCCCTGGGGTG 148
Canis   GACCAGGGCTTGGT--CCTTGGGCACACGTTCAG-----------CCGGGGGTGCCTTAGCCTGGGCACTCAGATGAGTCCATCCCAG---ACTGGATGC 184

Homo    CATCTGTTGCGGCAGGTGCTTTGAATTGGCAAAGGGACAGAGCCGGGCATGGTGCTCTGGGGGTTGGGGGAAGGACTAAGGTCAGAGCAAACTCTCCTGG 272
Pan     CATCTGTTGTGGCAGGTGCTTTGAATTGGCAAAGGGACAGAGCCGGGCATGGTGCTCTGGGGGTTGGGGGAAGGACTAAGGTCAGAGCAAACTCTCCTGG 272
Macaca  CATCTGTC-TGACAGGTGCTTTGAATCAGCAGAGGGACAGAGCTGGGCATGGTGCTCTGGGGGTTGGGGGAAGGACTCCGGGCAGAGAGAA--CTCCTGG 273
Bos     CCCCGACTCCACCCG--------------------------CCTAGGGCAGGTGCTCTGGGGGAAGG---------CGAGGTCCAGGCTTAGTTTCCTGG 213
Canis   TGTCTGCT---------------------------------ACAGGGGATGGCACTCTGGGGAAAGCAAGCCGAGCCAAGGT-----------TTCCGTG 240

Homo    CTTCAGTGCTTGTGAATCAGAGGGTTTAAAAGAAAAACCCACCTGGTAAGGTGGCTGAG--CGCCCTCTGTCTTTCCATGGG-AGCGCAG 359
Pan     CTTCAGTGCTTGTGAATCAGAGGGTTTAAAAGAAAAACCCACCTGGTAAGGTGGCTGAG--CGCCCTCTGTCTCTCCATGGG-AGCACAG 359
Macaca  CTTTAGTGCTTGTGAATCAGAGGGTTTAAAAGAAAAACCCGCCTGGTGAGGTGGCTGAG--CGCCTGCTGTCCTTCCCTGGG-AGCACAG 360
Bos     CTTCAGGGCTG-TGAGTC--AGGGTCTCCAGGAAAAGCCCACCCGGCGAAATGAGTGTG-----CACTCGTCTTTCTGTGGATTGTACAG 295
Canis   CCTCCCTGCTC-TGAGTCCAAGGATTT-CAAGGAAAACCCACCTGGTCAGGTAGATGTGCTCACCTGCTGTCTTCCCCGGGG-AGTGCAG 327
```

ENSG00000105404 intron 1

Description: Prenylated Rab acceptor protein 1 (RABAC1)
Intron number: 1
Human chromosome: 19
Intron start (bp): 47154941
Human intron length : 288
Intron alignment length: 330
Flanking exons length (upstream/downstream): 56/213
SNP density: 0.000000
K tree score: 0.0434
Scaling factor: 0.7132
Human-chimpanzee distance: 0.010826
Total primate branch length: 0.1236

ENSG00000105404 exon 1

```
Homo    ATGGCAGCGCAGAAGGACCAGCAGAAAGATGCCGAGGCGGAAGGGCTGAGCGGCAC 56
Pan     ATGGCAGCGCAGAAGGACCAGCAGAAAGATGCCGAGGCGGAAGGGCTGAGCGCCAC 56
Macaca  ATGGCAGCCCAGAAGGACCAGCAGAAAGATGTCGAGGCGGAAGGGCTGAGCGCCAC 56
Bos     ATGGCGGTCGAGAAGGACCAGCAGAAGGATGCCGAGGCGGAAGGACTGAGCGCCGC 56
Canis   ATGGCGGCCGAGAAGGACCAGCAGAAGGATGCTGAGCCGGAAGGGCTGAGCGCCAC 56
```

ENSG00000105404 exon 2

```
Homo    GACCCTGCTGCCGAAGCTGATTCCCTCCGGTGCAGGCCGGGAGTGGCTGGAGCGGCGCCGCGCGACCATCCGGCCCTGGA 80
Pan     GACCCTGCTGCCGAAGCTGATTCCCTCCGGTGCAGGCCGGGAGTGGCTGGAGCGGCGCCGCGCGACCATCCGGCCCTGGA 80
Macaca  GACCCTGCTGCCGAAGCTGATTCCCTCCGGTGCAGGCCGGGAGTGGCTGGAGCGGCGCCGCGCGACTATCCGGCCGTGGG 80
Bos     GACCCTGCTGCCGAAACTGATTCCATCTGGCGCGGGCCGCGAGTGGCTGGAGCGGCGCCGTGCGACCATCCGGTCCTGGG 80
Canis   GACCCTGCTGCCGAAACTGATCCCATCTGGCGCGGGCCGTGAGTGGCTGGAGCGGCGCCGCGCGACCATCCGGCCCTGGA 80

Homo    GCACCTTCGTGGACCAGCAGCGCTTCTCACGGCCCCGCAACCTGGGAGAGCTGTGCCAGCGCCTCGTACGCAACGTGGAG 160
Pan     GCACCTTCGTGGACCAGCAGCGCTTCTCACGGCCCCGCAACCTGGGCGAGCTGTGCCAGCGCCTCGTACGNCACGTGGAG 160
Macaca  GCACCTTCGTGGACCAGCAGCGTTTCTCACGGCCCCGCAACCTGGGCGAGCTGTGCCAGCGCCTCGTACGCAACGTGGAG 160
Bos     GCTCCTTCGTGGACCAGCGGCGCTTCTCGCGGCCCCGCAACCTGGGCGAGCTGTGCCAGCGCCTCGTACGCAACGTGGAG 160
Canis   GCTCCTTCGTGGACCAGCGGCGCTTCTCGCGGCCCCGCAACCTGGGCGAGCTGTGCCAGCGCCTCGTACGCAACGTGGAG 160
```

ENSG00000105404 intron 1

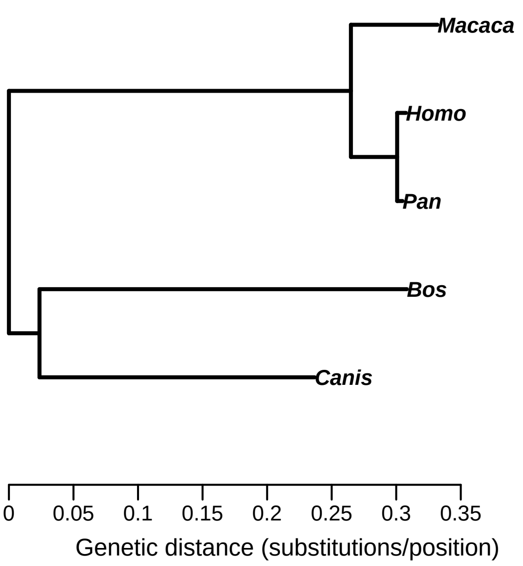

ENSG00000105404 intron 1

```
Homo    GTGAGCGAGCTGGCTCTAGG-----CCCTCGGACCAGAGACCCGGGAGTCCGGGCCCCCGGCCCCCTCCTCCCTCCTCCCTCGAGAGCCTGGAGTCCCAG 95
Pan     GTGAGCGAGCTGGCTCTAGG-----CCCTCGGACCAGAGACCCGGGAGTCCGGGCCCCCGGCCCCCTCCTCCCTCCTCCCTCGAGAGCCTGGAGTCCCAG 95
Macaca  GTGAGCGAGCCGGCTCTAGG-----CCCTCGGACCCGAGGCCCGGGAGTCCGGGACCCTGGCC-------CCCTCCTCCCTCGAGTGCCCGGAGTCCCAG 88
Bos     GTGAGCAA--------------------------CCGAGACCCGGGAGTCCTGGTCCCCCGACC------CCTTCCCCCTGGGGGTCCTAGGAATCCTGG 68
Canis   GTGAGCGAGCCGGCTCTAGGCACCCCCTCCCCGACCCAAACCCGGGAGCCCCGGCCCCCCGCC-------CCCTTCTCCTTCGGGTCCCAGGAATCCGGG 93

Homo    CCCCGAGCCGCAGAAGTGTTGGCCCCGGTCTCCTCTAATGGGACCCTGGAGTCTGGGCCCCTAGTCCCCTCCCTGCCTGCTGTACAGGAATCTGGGGTCC 195
Pan     CCCCGAGCCGCAGAAGTGTTGGCCCCGGTCTCCTCTAATGGCACCCTGGAGTCTGGGCCCCTAGCCCCCTCCCTGCCTGCTGTCCAGGAATCTGGGGTCC 195
Macaca  CCCCGAGACGCACAAGCGCTGGCCCCGGTCTCCTCTAATGGGACCCTGGCGTCTGGGCCCCTACCCCCCTCCCCGCCTGTGATTCAGTAATCTGGGGTCC 188
Bos     CCTCTAGACCTAAGAGCTTGGGCCTCTGTTGTCCTCTTCGG------GGAGTCTGGGCCCTCAGCCCCTTTCTCCCCTGAGATCCAGGAGCCTGGGACCA 162
Canis   CTCTGGACACTAGAGTCTGGGCCCCTGGTCTCCAGTTCAAGGACTCTGGAGTCTGGGCCCTCCTCGCCCTCCTCCTCTGGGATCCAGGGGTCTTGGACCT 193

Homo    C-------------GGGAGGGAGTCCAGGCACCCTTAGTCCCCTTCTTTTGCGGGGGT-------------CTGGGGCTCCATCCCGGTCCACCACTGAC 269
Pan     C-------------GGGAGGGAGTCCAGGCACCCTTAGTCCCCTTCTTTTGCGGGGGT-------------CTGGGGCTCCATCCCGGTCCACCACTGAC 269
Macaca  C-------------GGGAGGGAGTCCAAGCCCCCTTAAACCCCTTCTTTTGCGGGGGT-------------CTGAGGCTCCATCCCGGTCCACCACTGAC 262
Bos     TTATCCACCCCAGAACCCCGGAGTCCGGCCCCCCTCGGTCCTCTTCTTGCTGGAGGGT--------------CCGGACTCCATCCCGGTCGGTCGATCGC 248
Canis   CCTTCCACCCTAGGGCGCCGGAGTCCAGCCCCCCTCGGCACCCTTTTTGCTCGGGGGTGGGGACTGGGGGCTCCGGGCTCCACCCCGGTCTCCCACTGAC 293

Homo    C-----------CGGCCCCACCCCCCGCAG 288
Pan     C-----------CGGCCCCACCCCCCGCAG 288
Macaca  C-----------CGGCCCCACCCCTCGCAG 281
Bos     TGACGTGACCCGTGACCCCTCACCCATCAG 278
Canis   C-----------CGGCGCCGCCCAC--CAG 310
```

ENSG00000124194 intron 2

Description: Ganglioside-induced differentiation-associated protein 1-like 1 (GDAP1L1)
Intron number: 2
Human chromosome: 20
Intron start (bp): 42319400
Human intron length : 1088
Intron alignment length: 1514
Flanking exons length (upstream/downstream): 193/174
SNP density: 0.003676
K tree score: 0.0899
Scaling factor: 0.6124
Human-chimpanzee distance: 0.010353
Total primate branch length: 0.1228

ENSG00000124194 exon 2

```
Homo    TGCGAGGAGCGGGACGTGAGCCTGCCACAGAGCGAGCACAAGGAGCCCTGGTTCATGCGGCTCAACCTGGGCGAGGAGGT  80
Pan     TGCGAGGAGCGGGACGTGAGCCTGCCACAGAGCGAGCACAAGGAGCCGTGGTTCATGCGGCTCAACCTGGGCGAGGAGGT  80
Macaca  TGCGAGGAGCGGGACGTGAGCCTGCCGCAGAGCGAGCACAAGGAGCCATGGTTCATGCGGCTCAACCTGGGCGAGGAGGT  80
Bos     TGTGAGGAGCGGGACGTGAGCCTGCCACAGAGCGAGCACAAGGAGCCCTGGTTCATGCGGCTCAACCTGGGCGAGGAGGT  80
Canis   TGTGAGGAGCGGGACGTGAGCCTGCCACAGAGTGAGCACAAGGAGCCCTGGTTCATGCGGCTCAACCTGGGGGAGGAGGT  80

Homo    GCCCGTCATCATCCACCGCGACAACATCATCAGTGACTATGACCAGATCATTGACTATGTGGAGCGCACCTTCACAGGAG  160
Pan     GCCCGTCATCATCCACCGCGACAACATCATCAGTGACTATGACCAGATCATTGACTATGTGGAGCGCACCTTCACAGGAG  160
Macaca  GCCCGTCATCATCCACCGCGACAACATCATCAGTGACTATGACCAGATCATTGACTATGTGGAGCGCACCTTCACAGGAG  160
Bos     GCCCGTCATCATCCACCGCGACAACATCATCAGCGACTACGACCAGATCATCGACTACGTGGAGCGCACGTTCACTGGAG  160
Canis   GCCCGTCATTATCCACCGAGACAATATCATCAGCGACTATGACCAGATCATCGACTACGTGGAGCGAACCTTCACAGGAG  160
```

ENSG00000124194 exon 3

```
Homo    AGCACGTGGTGGCCCTGATGCCCGAGGTGGGCAGCCTGCAGCACGCACGGGTGCTGCAGTACCGGGAGCTGCTGGACGCA  80
Pan     AGCACGTGGTGGCCCTGATGCCCGAGGTGGGCAGCCTGCAGCACGCACGGGTGCTGCAGTACCGGGAGCTGCTGGACGCG  80
Macaca  AGCACGTGGTGGCCCTGATGCCCGAGGTGGGCAGCCCACAGCATGCGCGGGTGCTGCAGTACCGGGAGCTGCTGGACGCG  80
Bos     AGCATGTGGTGGCGCTGATGCCGGAGGCGGGCAGCCCCCAGCACGCGCGGGTGCTGCAGTACCGCGAGCTGCTGGATGCG  80
Canis   ATCACGTGGTGACCCTGATGCCTGAGGCGGGCAGCCCACAGCACGCGAGGGTGCTGCAGTACCGTGAGCTGCTGGACGCG  80

Homo    CTGCCCATGGATGCCTACACGCATGGCTGCATCCTGCATCCCGAGCTCACCACCGACTCCATGATCCCCAAGTACGCCAC  160
Pan     CTGCCCATGGATGCCTACACACATGGCTGCATCCTGCATCCCGAGCTCACCACCGACTCCATGATCCCCAAGTACGCCAC  160
Macaca  CTGCCCATGGACGCCTACACACACGGCTGCATCCTGCACCCCGAGCTCACCACCGACTCCATGATTCCCAAGTACGCCAC  160
Bos     CTGCCCATGGACGCCTACACGCACGGCTGCATTCTGCACCCCGAACTCACCACCGACTCCATGATCCCCAAGTACGCCAC  160
Canis   CTGCCCATGGACGCCTACACACACGGCTGCATCCTGCACCCCGAGCTCACCACTGACTCCATGATCCCCAAGTACGCCAC  160
```

ENSG00000124194 intron 2

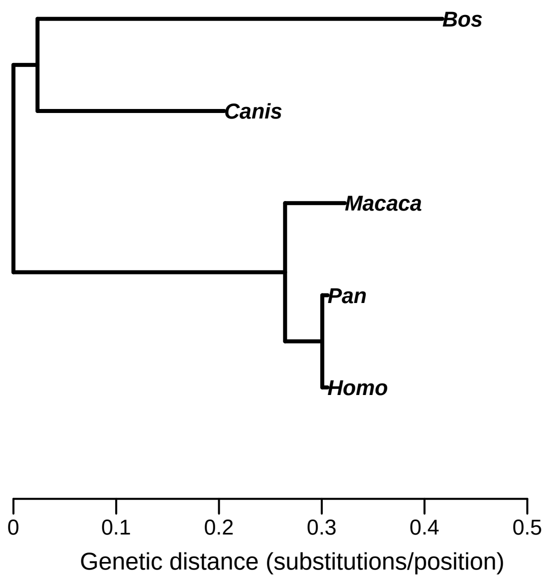

ENSG00000124194 intron 2

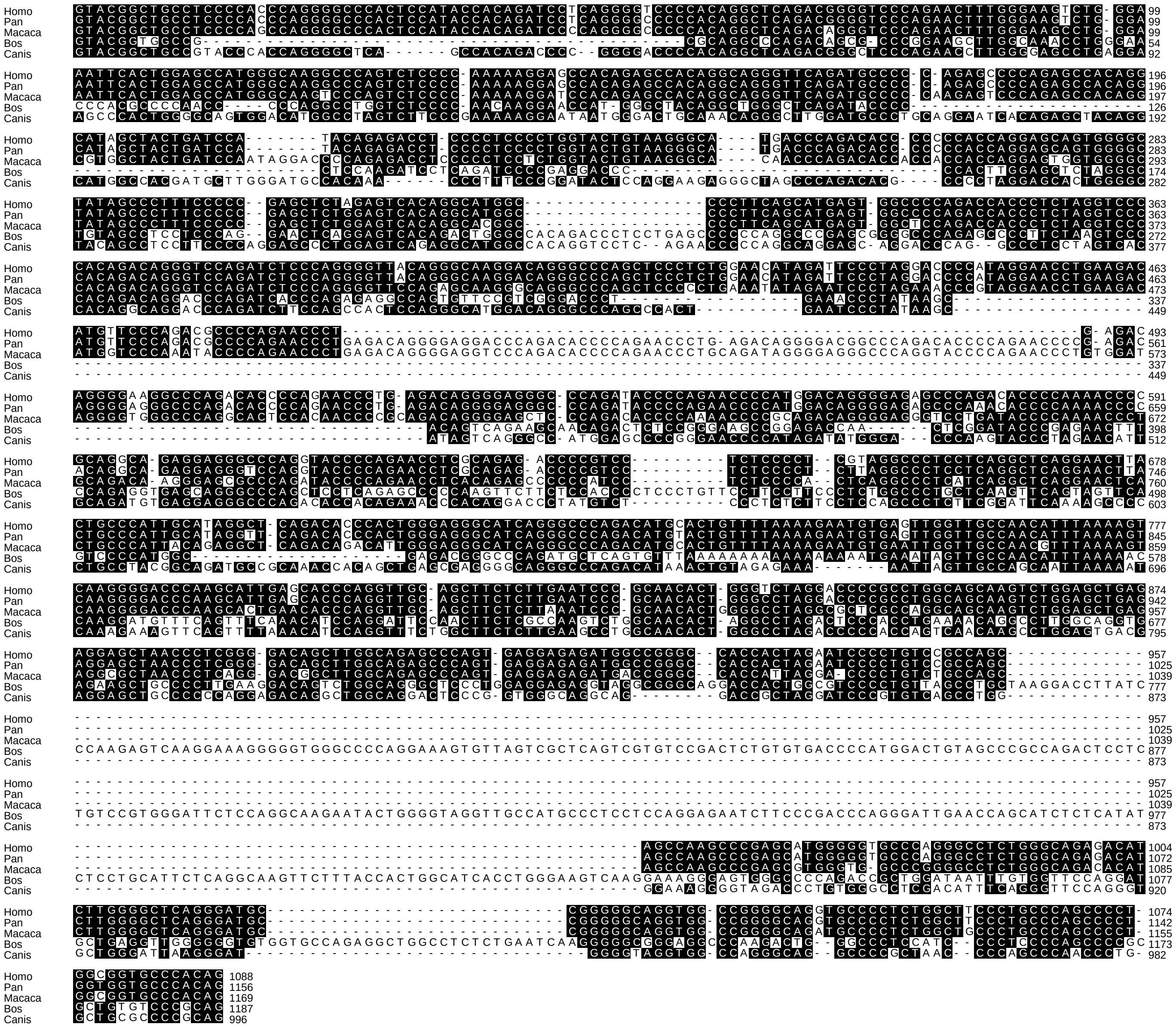

ENSG00000157911 intron 3

Description: Peroxisome assembly protein 10 (PEX10)
Intron number: 3
Human chromosome: 01
Intron start (bp): 2328255
Human intron length : 1496
Intron alignment length: 1589
Flanking exons length (upstream/downstream): 407/236
SNP density: 0.007353
K tree score: 0.0974
Scaling factor: 0.3885
Human-chimpanzee distance: 0.012257
Total primate branch length: 0.1197

ENSG00000157911 exon 3

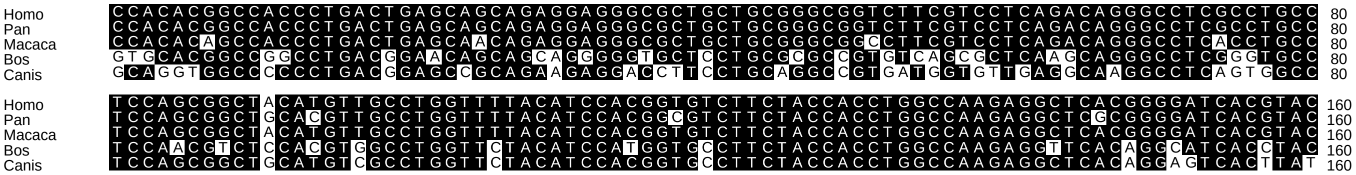

ENSG00000157911 exon 4

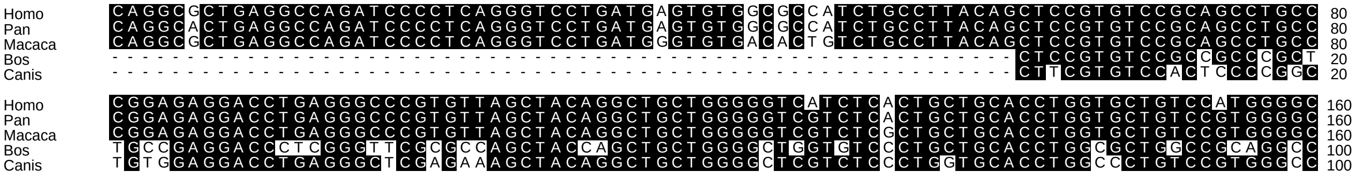

ENSG00000157911 intron 3

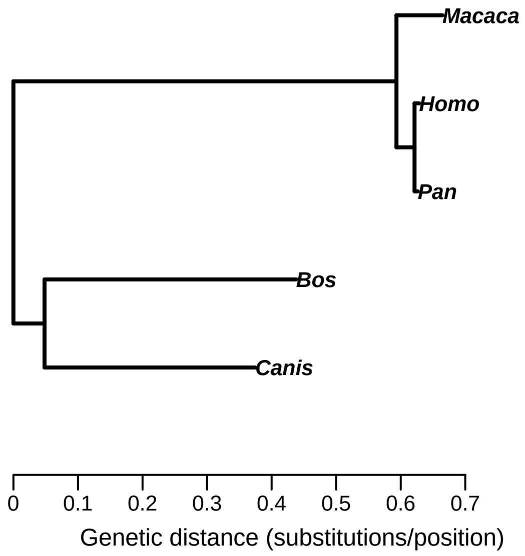

ENSG00000157911 intron 3

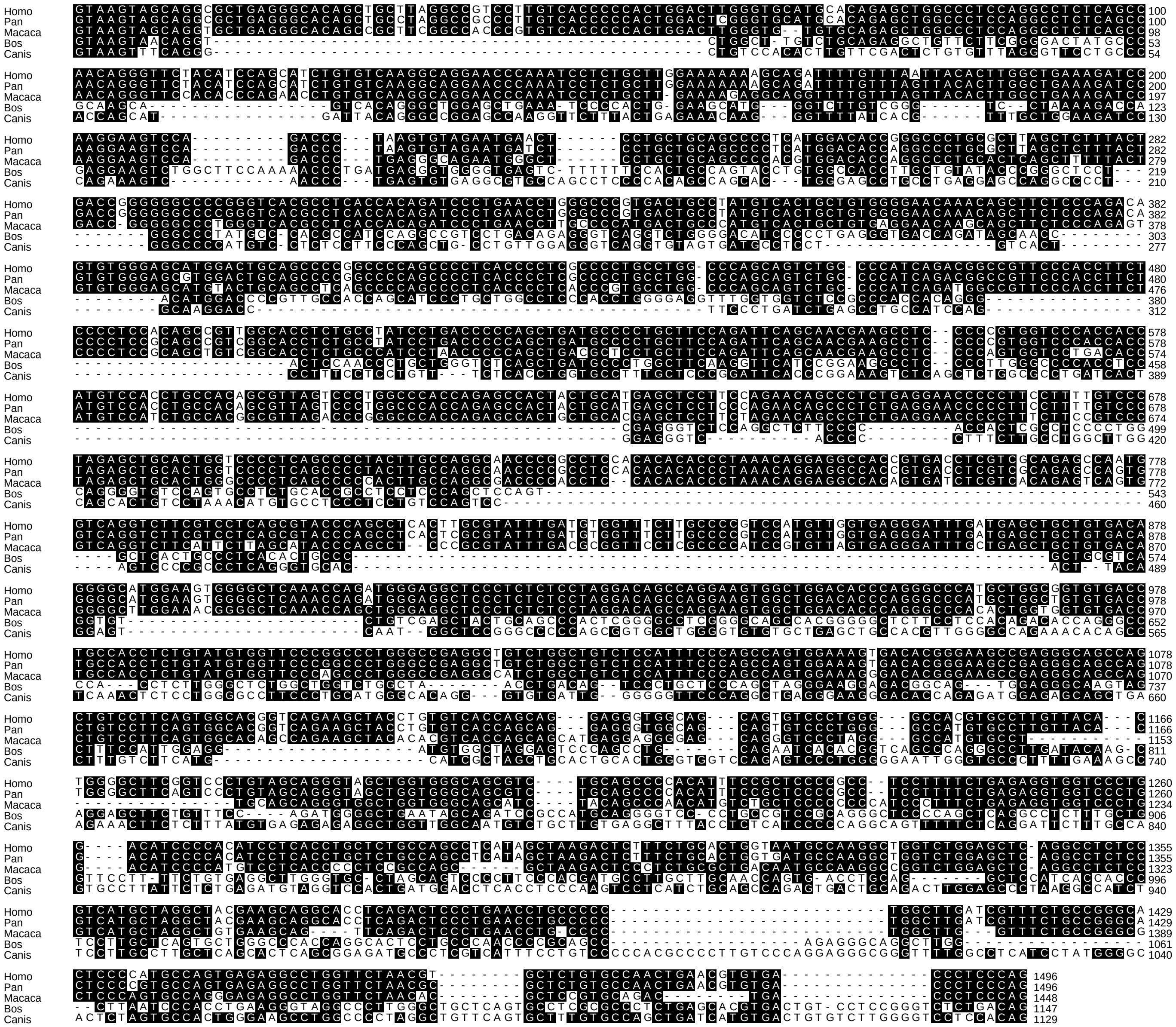

ENSG00000105053 intron 9

Description: Serine/threonine-protein kinase VRK3 (VRK3)
Intron number: 9
Human chromosome: 19
Intron start (bp): 55183562
Human intron length : 1146
Intron alignment length: 1466
Flanking exons length (upstream/downstream): 133/121
SNP density: 0.009599
K tree score: 0.0959
Scaling factor: 0.8277
Human-chimpanzee distance: 0.010642
Total primate branch length: 0.1187

ENSG00000105053 exon 9

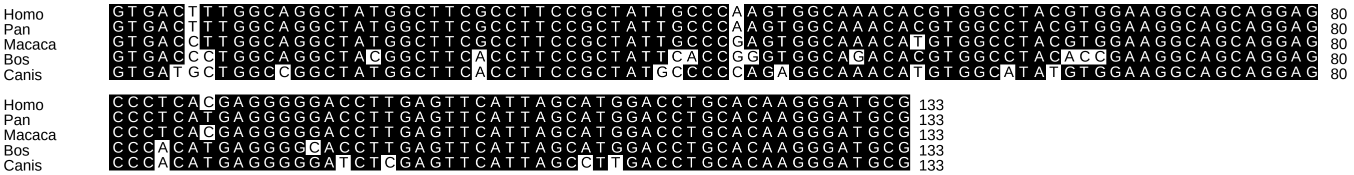

ENSG00000105053 exon 10

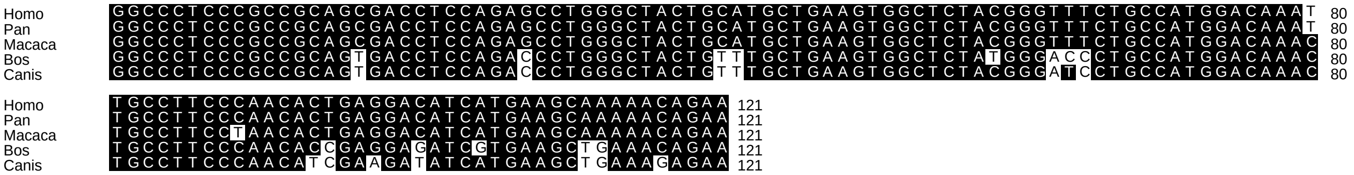

ENSG00000105053 intron 9

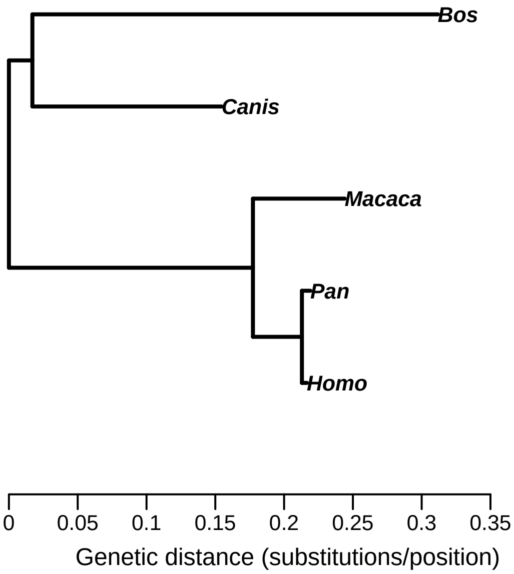

ENSG00000105053 intron 9

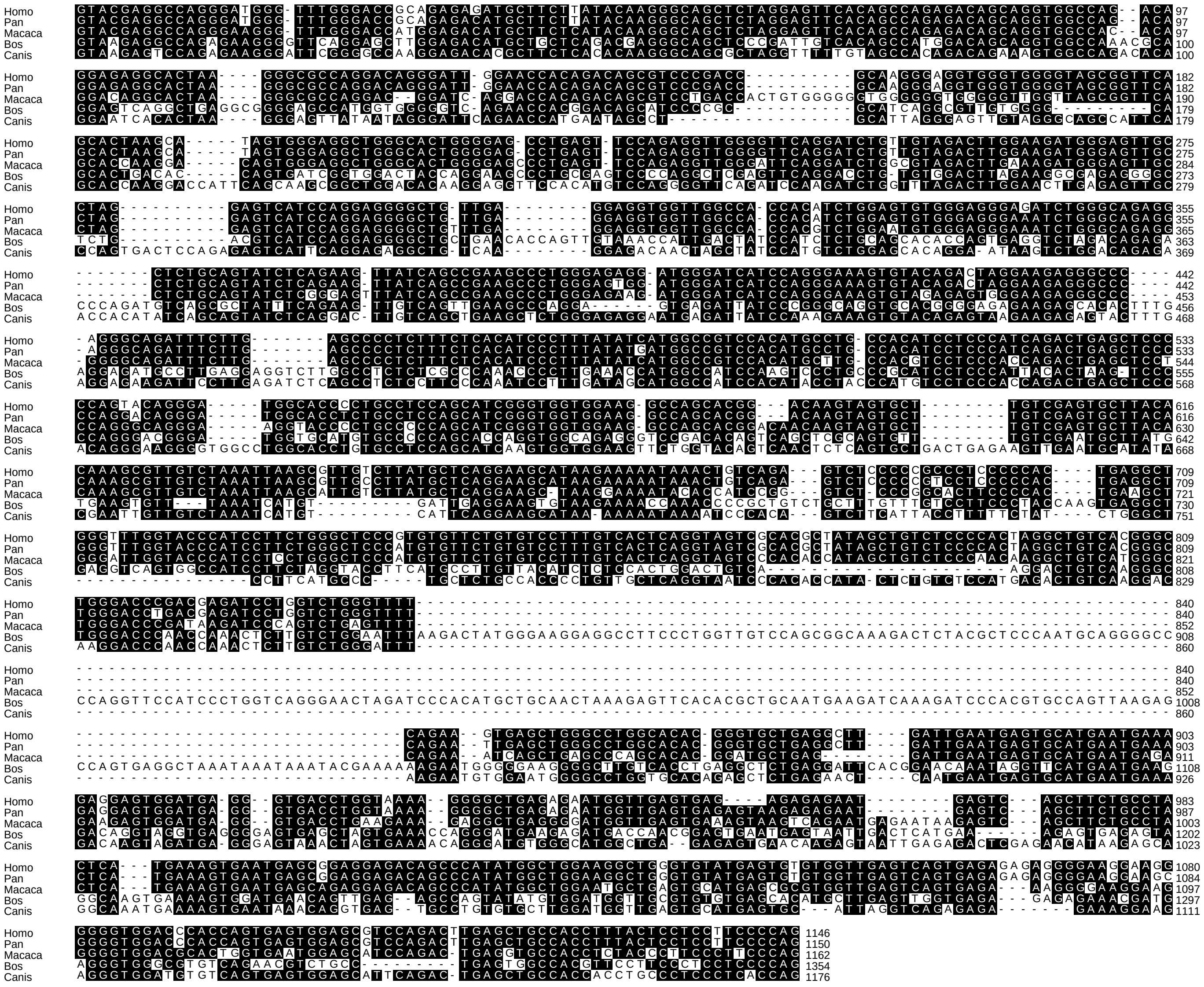

ENSG00000166825 intron 1

Description: Aminopeptidase N (ANPEP)
Intron number: 1
Human chromosome: 15
Intron start (bp): 88149699
Human intron length : 506
Intron alignment length: 598
Flanking exons length (upstream/downstream): 614/143
SNP density: 0.003953
K tree score: 0.0891
Scaling factor: 0.5998
Human-chimpanzee distance: 0.030659
Total primate branch length: 0.1181

ENSG00000166825 exon 1

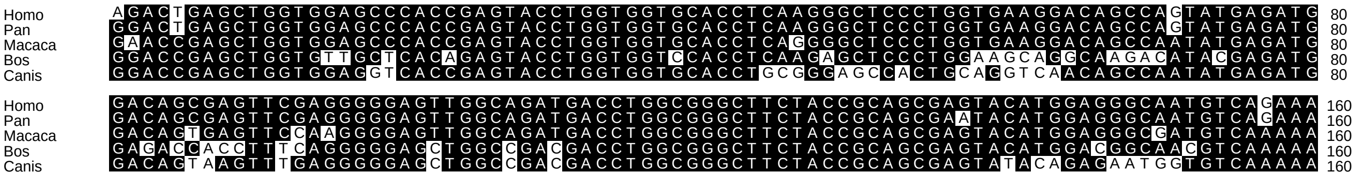

ENSG00000166825 exon 2

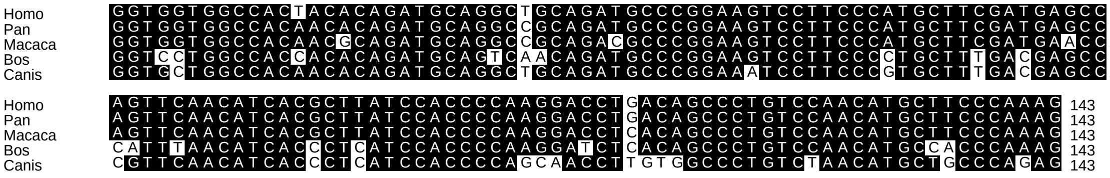

ENSG00000166825 intron 1

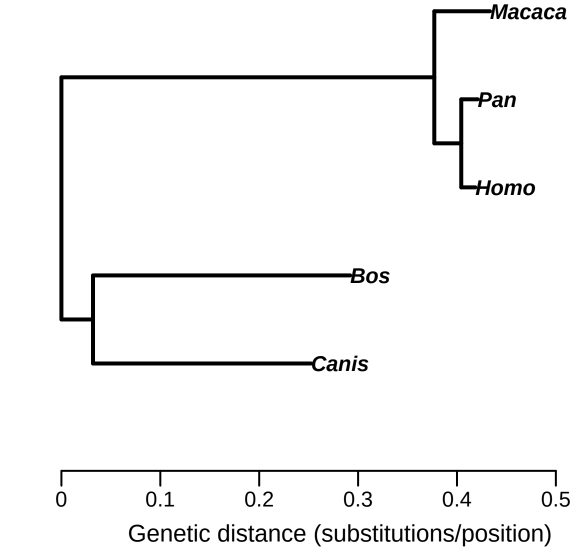

ENSG00000166825 intron 1

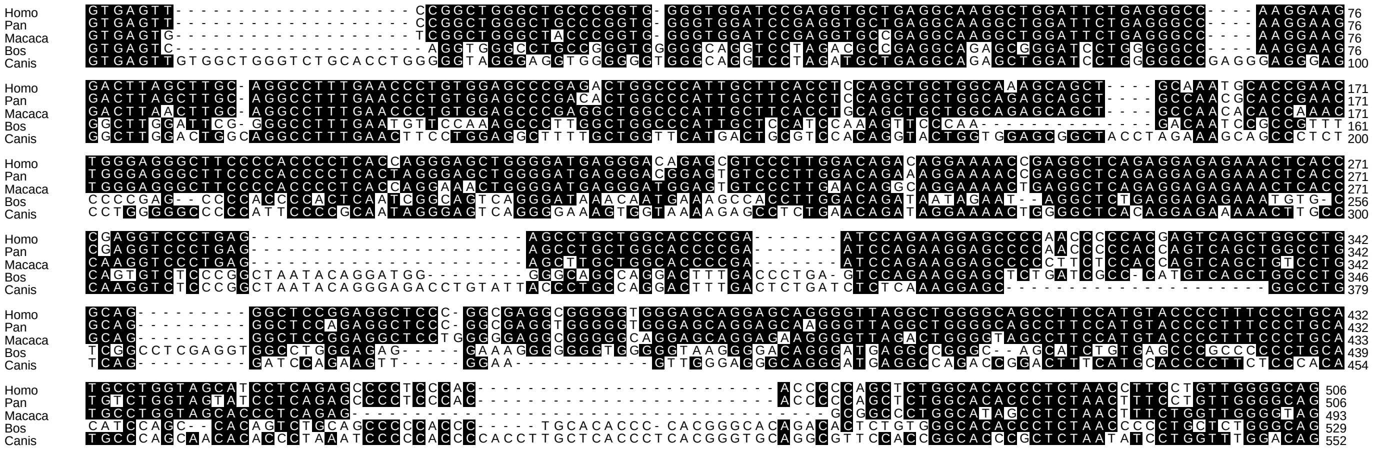

ENSG00000106077 intron 5

Description: abhydrolase domain containing 11 isoform 1 (ABHD11)
Intron number: 5
Human chromosome: 07
Intron start (bp): 72788958
Human intron length : 237
Intron alignment length: 245
Flanking exons length (upstream/downstream): 182/133
SNP density: 0.000000
K tree score: 0.0723
Scaling factor: 0.6798
Human-chimpanzee distance: 0.008554
Total primate branch length: 0.1179

ENSG00000106077 exon 5

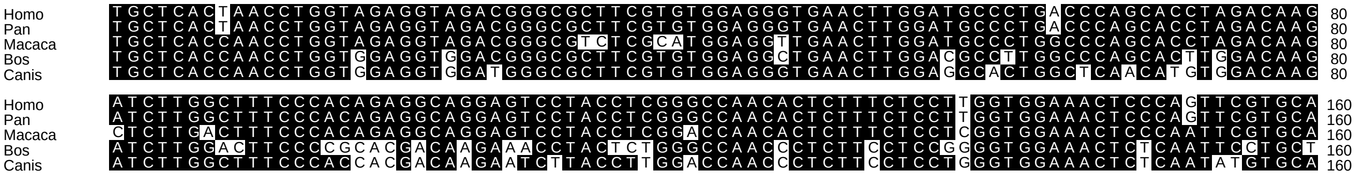

ENSG00000106077 exon 6

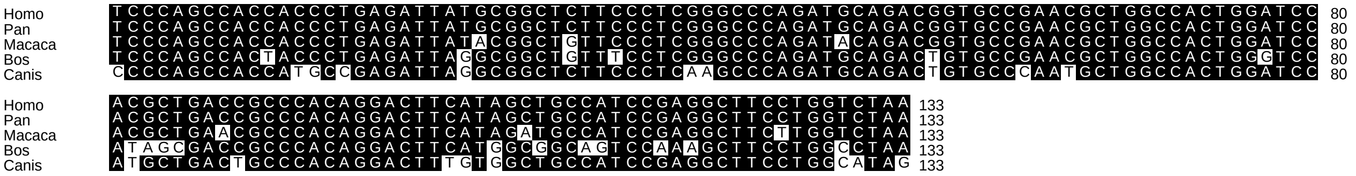

ENSG00000106077 intron 5

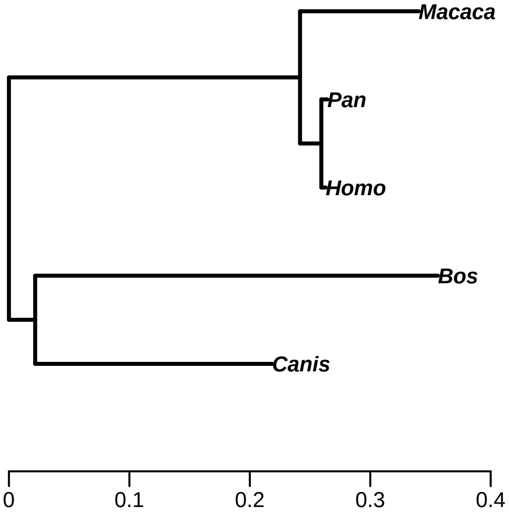

Genetic distance (substitutions/position)

ENSG00000106077 intron 5

```
Homo    GTAAGCTAGCCT------GGGTAGTAGGAG-GGGCATGCTTTACTCGCCCAGGCCC-CAGCCTGGGGACCCTACCAGGGGAGGCCTTGGGTGTGGAAGGG 92
Pan     GTAAGCTAGCCT------GGGTAGTAGGAG-GGGCATGCTTTACTCGCCCAGGCCC-CGGCCTGGGGACCCTACCAGGGGAGGCCTTGGGTGTGGAAGGG 92
Macaca  GTAAGCCAGCTT------GGGTAGTGGGAG-GGGCATGCTTTACTCACCCAGGCCC-TAGCCTGGGGACCCTACCAGGGAAGGCCTTGGGTGTGGAAGGT 92
Bos     GTAAGCCAGGCT------GGGTGGTAGGGACGGGCATACCCGACTTACCCAGCCCCTCAGCCTG--------CCTTGGGGAGGGCTGGGGTGTGGGTAAG 86
Canis   GTAAGCCAGTTTTACTGGTGGTGGCAGGAG-GGGCATGCCTTACTTACCCAGGCCC-CAGCCTGGGGCTCCTGCTTGGGGAGGGCTTGCATGCAGGCAGG 98

Homo    GGGTGGCTGAGAGGCCAGAAATACCACCGGGCATATGGGCTCACACAGCTCTCTCCATTTCTCCCCGTTCTGCTTCCACGTGTCTCTTTCACTCCTCCTC 192
Pan     GGGTGGCTGAGAGGCCAGAAATACCACCGGGCATATGGGCTCACACAGCTCTCTCCATTTCTCCCCGTTCTGCTTCCACGTGTCTCTTTCACTCCTCCTC 192
Macaca  GGGTGGCTGAGAGGCCAGAAATACCATTGGGCATGTGGACTTATGCAGCTCTCTCCGTTTCTCCCCACTCTGCTTACACTTGTCTCTTTCACTCCTCCTT 192
Bos     GGGTGCCTGAAAA-CCGGAAACACCACTGGGCACATAGACTCACGTGGTTCTCCC--TCTTTCCCCGCTTGGCCTCTGTGTCCCTCCTTCTCTCTCCACT 183
Canis   CAGTGCCTGAGAGTTCA-AAACACCACTGGGCATGTGGGCTTATGTAGCTCTCTTTTCCCTGCTCTCTTCTACT----------TCTCTCTTTCCCCATC 187

Homo    ATCCCACTTTCACCCGTTTTTGATGGCCGTCGCTGCGATGCACAG 237
Pan     ATCCCACTTTCACCCGTTTTTGATGGCCATCGCTGCGATGCACAG 237
Macaca  ATCCCACTTTCACCCATTTTCGATGGCCATCACTGCAATGCACAG 237
Bos     TTCCTCCTGTCATCCCTTCTCGGCTGCCTTCTCTCTGGTGTTCAG 228
Canis   TTCCCACTTTGACCCCTTCTGGGTGGCCTTCTCTTCGATGGACAG 232
```

ENSG00000011021 intron 10

Description: Chloride channel protein 6 (CLCN6)
Intron number: 10
Human chromosome: 01
Intron start (bp): 11809866
Human intron length : 884
Intron alignment length: 1077
Flanking exons length (upstream/downstream): 133/114
SNP density: 0.005656
K tree score: 0.062
Scaling factor: 0.4066
Human-chimpanzee distance: 0.015066
Total primate branch length: 0.1174

ENSG00000011021 exon 10

```
Homo    AGACAAGAGAGACTTTGTATCAGCAGGAGCGGCTGCTGGAGTTGCTGCAGCTTTCGGGGCGCCAATCGGGGGTACCTTGT 80
Pan     AGACAAGAGAGACTTTGTATCAGCAGGAGCGGCTGCTGGAGTTGCTGCAGCTTTCGGGGCGCCAATCGGGGGTACCTTGT 80
Macaca  AGACAAGAGAGACTTTGTATCAGCAGGAGCAGCTGCTGGAGTTGCTGCAGCTTTCGGGGCCCCAATCGGGGGTACCTTGT 80
Bos     AGACAAGCGGGACTTTGTATCAGCAGGGGCAGCTGCCGGAGTTGCTGCGGCTTTTGGGGCCCCCATTGGGGGCACCCTGT 80
Canis   AGACAAGCGAGACTTTGTATCAGCAGGGGCGGCTGCTGGAGTTGCTGCAGCTTTCGGGGCTCCCATCGGGGGTACCCTGT 80

Homo    TCAGTCTAGAGGAGGGTTCGTCCTTCTGGAACCAAGGGCTCACGTGGAAAGTG 133
Pan     TCAGTCTAGAGGAGGGATCGTCCTTCTGGAACCAAGGGCTCACGTGGAAAGTG 133
Macaca  TCAGTCTAGAGGAGGGTTCGTCCTTCTGGAACCAAGGGCTCACATGGAAAGTG 133
Bos     TCAGTCTGGAGGAGGGTTCGTCCTTCTGGAACCAAGGGCTCACATGGAGAGTG 133
Canis   TCAGTCTGGAAGAGGGCTCGTCCTTCTGGAACCAGGGGCTCACGTGGAAAGTG 133
```

ENSG00000011021 exon 11

```
Homo    CTCTTTTGTTCCATGTCTGCCACCTTCACCCTCAACTTCTTCCGTTCTGGGATTCAGTTTGGAAGCTGGGGTTCCTTCCA 80
Pan     CTCTTTTGTTCCATGTCTGCCACCTTCACCCTCAACTTCTTCCGTTCTGGGATTCAGTTTGGAAGCTGGGGTTCCTTCCA 80
Macaca  CTCTTTTGTTCCATGTCTGCCACCTTCACCCTCAACTTCTTCCGTTCTGGGATTCAGTTTGGAAGCTGGGGTTCCTTCCA 80
Bos     CTCTTCTGCTCCATGTCTGCCACCTTCACCCTCAATTTCTTTCGTTCTGGAATTCAGTTTGGAAGTTGGGGTTCCTTCCA 80
Canis   CTCTTTTGTTCCATGTCTGCCACCTTCACCCTCAACTTCTTTCGTTCTGGGATTCAGTTTGGAAGTTGGGGTTCCTTCCA 80

Homo    GCTCCCTGGATTGCTGAACTTTGGCGAGTTTAAG 114
Pan     GCTCCCTGGATTGCTGAACTTTGGCGAGTTTAAG 114
Macaca  GCTCCCTGGATTGCTGAACTTTGGCGAGTTTAAG 114
Bos     GCTCCCTGGGCTGCTGAACTTCGGCGAGTTTAAG 114
Canis   GCTCCCCGGATTGCTGAACTTTGGCGAGTTTAAG 114
```

ENSG00000011021 intron 10

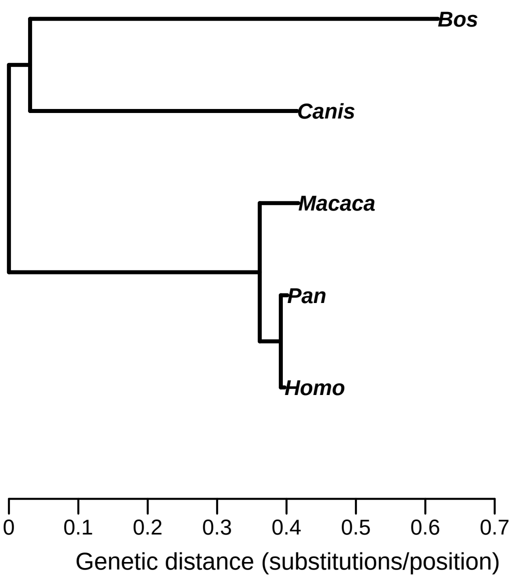

ENSG00000011021 intron 10

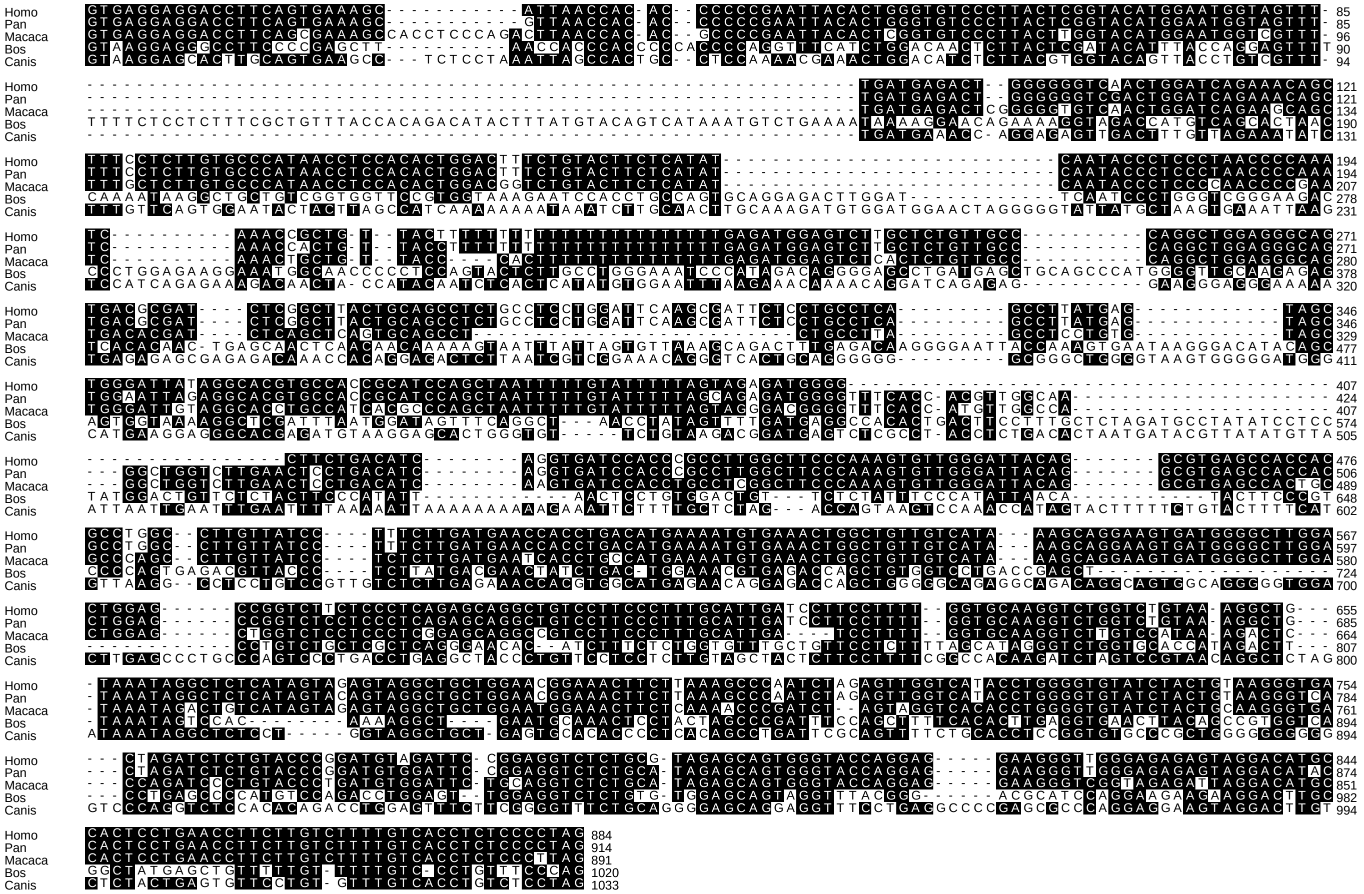

ENSG00000177728 intron 7

Description: KIAA0195 (KIAA0195)
Intron number: 7
Human chromosome: 17
Intron start (bp): 70997045
Human intron length : 207
Intron alignment length: 243
Flanking exons length (upstream/downstream): 103/101
SNP density: 0.000000
K tree score: 0.0895
Scaling factor: 0.6765
Human-chimpanzee distance: 0.010037
Total primate branch length: 0.1151

ENSG00000177728 exon 7

```
Homo    ATGGTGCCTGGACATGGCCCTGTCCCGACCAGTCACTGCCCTGGACAATGAGCGGTTCACAGTGCAGTCGGTGATGCTAC 80
Pan     ATGGTGCCTGGACATGGCCCTGTCCCGACCAGTCACTGCCCTGGACAATGAGCGGTTCACAGTGCAGTCGGTGATGCTAC 80
Macaca  GTGGTGCCTGGACATGGCCCTGTCCCGACCAGTCACCGCCCTGGACAATGAGCGGTTCACAGTGCAGTCGGTGATGCTAC 80
Bos     GTGGTGCCTGGACATGGCCCTGTCCCGCCCGGTGACCGCTCTGGACAACGAGAGGTTCACAGTGCAGTCGGTGATGCTGC 80
Canis   ATGGTGCCTGGACATGGCCCTGTCCCGCCCAGTCACTGCCCTGGACAACGAGAGGTTCACGGTGCAGTCGGTGATGTTGC 80

Homo    ACTATGCTGTGCCCGTGGTCCTG 103
Pan     ACTATGCTGTGCCCGTGGTCCTG 103
Macaca  ACTATGCTGTGCCTGTGGTCCTG 103
Bos     GCTACGCGGTGCCTGTGGTCCTG 103
Canis   ACTACGCCGTGCCTGTGGTCCTG 103
```

ENSG00000177728 exon 8

```
Homo    GCCGGCTTCCTCATCACCAATGCCCTGCGCTTCATCTTCAGTGCCCCGGGGGTCACTTCCTGGCAGTACACCCTCCTCCA 80
Pan     GCCGGCTTCCTCATCACCAATGCCCTGCGCTTCATCTTCAGTGCCCCGGGGGTCACTTCCTGGCAGTACACCCTCCTCCA 80
Macaca  GCCGGCTTCCTCATCACCAATGCCCTGCGCTTCATCTTCAGTGCCCCGGGGGTCACTTCCTGGCAGTACACCCTCCTGCA 80
Bos     GCCAGCTTCCTCATCACCAACGCCCTGCGCTTCATGCTCAATGCCCCGGGTGTCACCACCTGGCAATACACTCTCCTCCA 80
Canis   GCTGGCTTCCTTATCACCAATGCCCTGCGCTTCATGCTGAATGCCCCTGGCGTCACATCCTGGCAGTACACCCTCCTCCA 80

Homo    GCTCCAGGCAAGGACCACCCT 101
Pan     GCTCCAGGCAAGGACCACCCT 101
Macaca  GCTCCAGGCAAGGACCGCCCT 101
Bos     GCTGCAG-------------- 87
Canis   GCTACAG-------------- 87
```

ENSG00000177728 intron 7

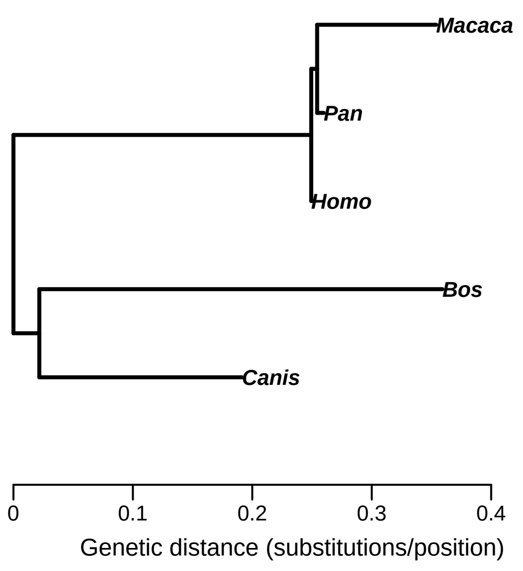

ENSG00000177728 intron 7

```
Homo    GTGCGTGTGGCGGGGCTGTGCGGGGCTGCATGGGGCAGAGGAGAGGGCTGGACACGGGGGG----GTCTCAGGGCCACTCACATGAGCGGGAGTGAATGC 96
Pan     GTGCGTGTGGCGGGGCTGTGCGGGGCTGCATGGGGCAGAGGAGAGGGCTGGACACGGCGGG----GTCTCAGGGCCACTCACATGAGCGGGAGTGAATGC 96
Macaca  GTGCGTGCAGCAG--------GGGGCTGCAC-GGGCAGAGGAGAGGGCTGGACTTGGTGGGGGGAGTCTCAGGGCCACTCACGTGAGCGGGAGTGAATGC 91
Bos     GTGTGTGCGGCCG----------GGCCGTGGCGGGGGCAGCTGGGGGCTGGGGGCTCGGGG---ACCCTGGGAGCCAGACTCAGGCAAGCCTGTGAGCAA 87
Canis   GTGCGTGAGGCGG-----------GCCTCGCGGGGCAGGGGAGAGGGCCAGGAAATGGGGG---AGCCTCAGGGCCAGATTCAGGTGAGCGTGTGAGGGA 86

Homo    AGA-----GGGTCCCAGAGTGAGCCAGCC----------------------TGTGGAGTAGCAAAGGAAGGGGAACGGCAGTGCCTGGGTCCCTCT--AG 167
Pan     AGA-----GGGTCCCAGAGTGAGCCAGCC----------------------TGTGGAGTAGCAAAGGAAGGGGAACGGCAGTGCCTGGGTCCCTCT--AG 167
Macaca  AGA-----GGGTCGCTGAGCGAGCCAGCC----------------------TGTGGAGTAGCAAAGGAAGGAAAATGGCAGTGCCTATGACGCTCT--AG 162
Bos     AGACAGGAGGGTCCAGGTGTGAGCCGGCCAGTGCCAGGGGTGGGGGAG---TGTGAGTGGTCAGAAGGTGGGGTGCCTTAGAGCCTAAGTCCCCCCTGGG 184
Canis   AGATGGGGGTGGTCCAGAGTGAGATGGCCAGTGCTGGGGTTGGGGGGTGGGTGTGAGTGGGCAGAGGAAAGGGAGCCTTAGAGCCTAGGTCCTTCT--CT 184

Homo    AGGGGCGGGGTCAA---GGCTGTGCCTCTGCTGTTCCCAACAG 207
Pan     AGGGGTGGGGTCAA---GGCTGTGCCTCTGCTGTTCCCAACAG 207
Macaca  AGGGGCGGGGTCAA---GGCTGTGCCTCTGCTGTTCCCAACAG 202
Bos     AGGGGTGGCGTTAA---GGCAGTGTCTCTCCTGTCCCCAACAG 224
Canis   GGGTGGGGAGCTAATAGGGCTGCACTTCTGCTGTTCCCAA-AG 226
```

ENSG00000021488 intron 9

Description: B(0,+)-type amino acid transporter 1 (SLC7A9)
Intron number: 9
Human chromosome: 19
Intron start (bp): 38025064
Human intron length : 1537
Intron alignment length: 1744
Flanking exons length (upstream/downstream): 97/150
SNP density: 0.005856
K tree score: 0.072
Scaling factor: 0.8108
Human-chimpanzee distance: 0.010685
Total primate branch length: 0.1141

ENSG00000021488 exon 9

```
Homo    ACTCATTTACGTGGCGGGCCGGGAGGGTCACATGCTCAAAGTGCTTTCTTACATCAGCGTCAGGCGCCTCACTCCAGCCC 80
Pan     ACTCATTTACGTGGCGGGCCGGGAGGGCCACATGCTCAAAGTGCTTTCTTACATCAGCGTCAGGCGCCTCACTCCAGCCC 80
Macaca  ACTCATTTATGTGGCGGGCCGGGAGGGCCACATGCTCAAAGTGCTTTCTTACATCAGCGTCAAGCGCCTCACTCCAGCCC 80
Bos     ACTTGTTTTCGTTGCGGGCCGGGAAGGCCACATGCTGAAAGTGCTGTCTTACATCAGCGTCAGGCGCCTCACTCCAGCTC 80
Canis   GCTTGTGTACGTGGCGGGCCGGGAGGGCCACATGCTCAAAGTGCTCTCCTACATTAGTGTCAAGCGCCTGACTCCAGCCC 80

Homo    CCGCCATCATCTTTTAT 97
Pan     CCGCCATCATCTTTTAT 97
Macaca  CCGCCATCATCTTCCAT 97
Bos     CTGCCATCATGTTTCAT 97
Canis   CCGCCATCATCTTTTAT 97
```

ENSG00000021488 exon 10

```
Homo    GGTATCATAGCAACGATTTATATCATCCCTGGTGACATAAACTCGTTAGTCAATTATTTCAGCTTTGCCGCATGGCTGTT 80
Pan     GGTATCATAGCAACGATTTATATCATCCCTGGTGACATAAACTCGTTAGTCAATTATTTCAGCTTTGCCGCATGGCTGTT 80
Macaca  GGTATCATAGCAACGATTTATATCATCCCTGGTGACATAAACTCGTTAGTTAATTATTTCAGCTTTGCCGCATGGCTCTT 80
Bos     GGCATGATAGCGATCATTTACATCATCCCTGGTGACATAAACTCATTAGTCAATTACTTCAGTTTCGCTGCATGGCTGTT 80
Canis   GGCATCATAGCGACTATTTACATCATCCCCGGTGACATAAACTCGTTGGTCAATTACTTCAGTTTTGCTGCGTGGCTCTT 80

Homo    TTATGGCCTGACGATTCTAGGACTCATCGTGATGAGATTTACAAGGAAAGAGCTGGAAAGGCCTATCAAG 150
Pan     TTATGGCCTGACGATTCTAGGACTCATCGTGATGAGATTTACAAGGAAAGAGCTGGAAAGGCCTATCAAG 150
Macaca  TTATGGCCTGACGATTCTAGGACTCATCGTGATGAGATTTACAAGGAAAGAGCTGGAAAGGCCTATCAAG 150
Bos     TTATGGTTTGACGATCACCGGACTCATTGTGATGAGGTTTACAAGGAAAGAGCTGAAAAGACCTATCAAG 150
Canis   TTACGGCCTGACGATTCTGGGACTGGTTGTGATGAGGTTCACAAAGAAAGACCTGGAAAGGCCTATCAAG 150
```

ENSG00000021488 intron 9

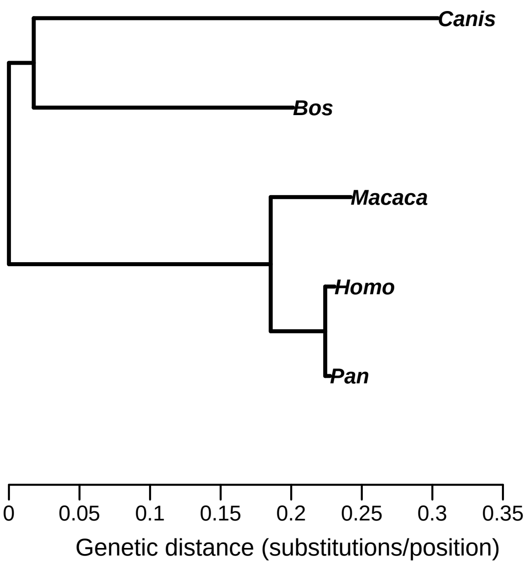

ENSG00000021488 intron 9

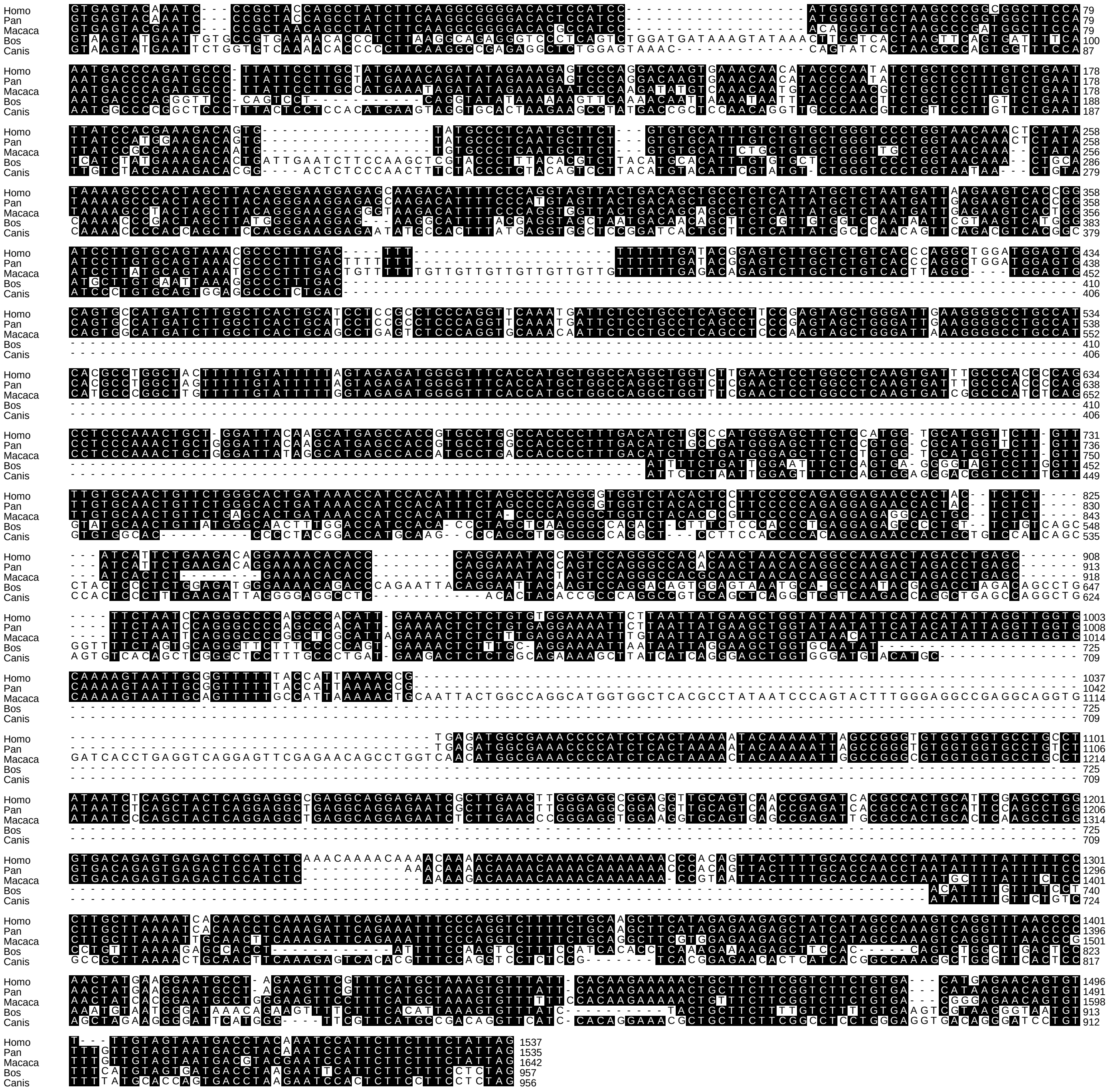

ENSG00000136542 intron 4

Description: Polypeptide N-acetylgalactosaminyltransferase 5 (GALNT5)
Intron number: 4
Human chromosome: 02
Intron start (bp): 157860557
Human intron length : 595
Intron alignment length: 680
Flanking exons length (upstream/downstream): 136/120
SNP density: 0.003361
K tree score: 0.0847
Scaling factor: 1.1199
Human-chimpanzee distance: 0.006767
Total primate branch length: 0.1137

ENSG00000136542 exon 4

```
Homo    GTGATGTGTTGACATTTTTAGATTCTCATGTGGAATGTAACGTTGGTTGGTTGGAACCTCTTCTGGAAAGAGTTTATTTA 80
Pan     GTGATGTGTTGACATTTTTAGATTCTCATGTGGAATGTAACGTTGGTTGGTTGGAACCTCTTCTGGAAAGAGTTTATTTA 80
Macaca  GTGATGTGTTGACATTTTTAGATTCTCACGTGGAATGTAACGTTGGTTGGTTGGAACCTCTTCTGGAAAGAGTGTATTTA 80
Bos     GTGATGTGTTGACATTCTTAGATTCTCACGTGGAATGTAACATTGGCTGGTTGGAACCTCTCCTGGAAAGAGTTTACTTA 80
Canis   GTGATGTGTTGACATTCTTAGATTCTCACGTGGAATGTAACGTTGGTTGGTTGGAACCTCTTCTGGAAAGAGTTTATTTA 80

Homo    AGTAGAAAGAAAGTGGCCTGTCCAGTAATCGAAGTCATCAATGATAAGGATATGAG 136
Pan     AGTAGAAAGAAAGTGGCCTGTCCAGTAATCGAAGTCATCAATGATAAGGATATGAG 136
Macaca  AGTAGAAAGAAAGTGGCCTGTCCAGTAATCGAAGTCATCAATGATAAGGATATGAG 136
Bos     AGCAGAAAGAAAGTAGCCTGTCCAGTAATTGAAGTCATCAATGATAAGGATATGAG 136
Canis   AGTAGAAAGAAAGTAGCCTGTCCAGTAATTGAAGTCATCAATGATAAGGATATGAG 136
```

ENSG00000136542 exon 5

```
Homo    TTACATGACAGTGGATAACTTTCAAAGAGGCATCTTTGTGTGGCCCATGAACTTTGGTTGGAGAACAATTCCTCCAGATG 80
Pan     TTACATGACAGTGGATAACTTTCAAAGAGGCATCTTTGTGTGGCCCATGAACTTTGGTTGGAGAACAATTCCTCCAGATG 80
Macaca  TTACATGACAGTGGACAACTTTCAAAGAGGCATCTTTGTGTGGCCCATGAACTTTGGTTGGAGAACAATTCCTCCAGATG 80
Bos     TTACATGACAGTGGACAACTTTCAAAGAGGCATATTTGTGTGGCCAATGAACTTTGGTTGGAGAACAATTCCTCCAGACG 80
Canis   TTACATGACAGTGGACAACTTTCAAAGAGGCATCTTTGTGTGGCCCATGAACTTTGGTTGGAGAACAATTCCTCCAGACG 80

Homo    TCATTGCAAAAAAACAGAATTAAAGAAACTGATACAATAAG 120
Pan     TCATTGCAAAAAAACAGAATTAAAGAAACTGATACAATAAG 120
Macaca  TCATTGCAAAAAAACAGAATTAAAGAAACTGATGCAATAAA 120
Bos     TTGTTGCAAAAAAACAAAATTAAAGAGACTGATATAATAAG 120
Canis   TTGTTGCAAAAAAACAGAATTAAAGAAACAGATATAATAAG 120
```

ENSG00000136542 intron 4

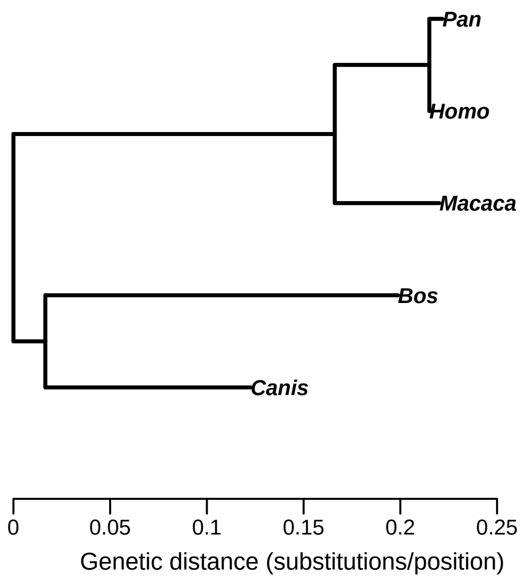

ENSG00000136542 intron 4

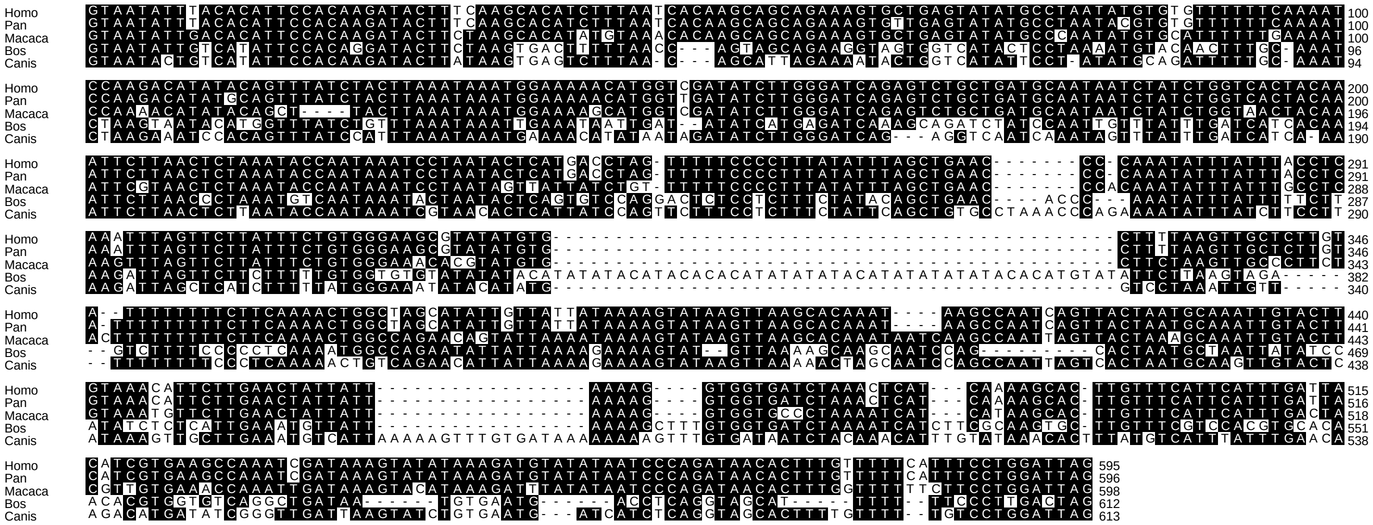

ENSG00000163959 intron 5

Description: organic solute transporter alpha (Osta)
Intron number: 5
Human chromosome: 03
Intron start (bp): 197439542
Human intron length : 535
Intron alignment length: 583
Flanking exons length (upstream/downstream): 159/112
SNP density: 0.003738
K tree score: 0.0783
Scaling factor: 1.1157
Human-chimpanzee distance: 0.026789
Total primate branch length: 0.1131

ENSG00000163959 exon 5

```
Homo    GTTTTATGCCGTGTGCTTTTACCTGCTGATGCTGGTCATGGTGGAAGGCTTTGGGGGGAAGGAGGCAGTGCTGAGGACGC 80
Pan     GTTTTATGCCGTGTGCTTTTACCTGCTGATGCTGGTCATGGTGGAAGGCTTTGGGGGGAAGGAGGCAGTGCTGAGGACGC 80
Macaca  GTTTTATGCCGTGTGCTTTTACCTGCTGATGCTGGTCATGGTGGAAGGCTTTGGGGGGAAGGAGGCAGTGCTGAGGACGC 80
Bos     GTTTTACTCGATGTGCTTTTACCTGCTGATGCAGGCCATGGTGGAAGGCTTTGGTGGGAAGGAGGCGGTGTTGAGGACAC 80
Canis   GTTTTACTCGGTATGCTTTTACTTGATGATGATGGCCATGGTGGAAGGCTTTGGTGGGAAGGAGGCAGTACTGAGGACAC 80

Homo    TGAGGGACACCCCGATGATGGTCCACACAGGCCCCTGCTGCTGCTGCTGCCCCTGCTGTCCACGGCTGCTGCTCACCAG 159
Pan     TGAGGGACACCCCGATGATGGTCCACACAGGCCCCTGCTGCTGCTGCTGCCCCTGCTGTCCGCGGCTGCTGCTCACCAG 159
Macaca  TGAGGGACACCCCGATGATGGTCCACACAGGCCCCTGCTGCTGCTGCTGCCCCTGCTGTCCGCGGCTGCTGCTCACCAG 159
Bos     TGAAGGACACCCCGGTGATGATCCATACAGGCCCCTGCTGCTGCTGTTGCCCCTGCTGCCCCCGAATCAAGATCACCAG 159
Canis   TGAAGGACACTCCGATGATGATCCACACAGGTCCCTGCTGCTGCTGTTGCCCCTGCTGCCCTCCACTCATGCTCACCAG 159
```

ENSG00000163959 exon 6

```
Homo    GAAGAAGCTTCAGCTGCTGATGTTGGGCCCTTTCCAATACGCCTTCTTGAAGATAACGCTGACCCTGGTGGGCCTGTTTC 80
Pan     GAAGAAGCTTCAGCTGCTGATGTTGGGCCCTTTCCAATACGCCTTCTTGAAGATAACGCTGACCCTGGTGGGCCTGTTTC 80
Macaca  GAAGAAGCTTCAGCTGCTGATGCTGGGTCCTTTCCAATACGCCTTCCTGAAGATAACGCTGAGCCTGGTGGGCCTGTTTC 80
Bos     GAAGAGACTTCAGCTGCTGCTGTTGGGCCCCATCCAGTACGCCTTCTTCAAGATATCATTGACCCTGGTGGGCCTGTTTC 80
Canis   GAAAAAGCTTCAGCTGCTGATGTTAGGCCCATTCCAGTATGCCTTCTTCAAGATAACACTGAGCCTGGTGGGCCTGTTTC 80

Homo    TCGTCCCCGACGGCATCTATGACCCAGCAGAC 112
Pan     TCATCCCCGACGGCATCTATGACCCAGCAGAC 112
Macaca  TCATCCCCGACGGCATCTATGACCCGGCAGAC 112
Bos     TCATCCCTGATGGCATCTTTGACCCATCAGAC 112
Canis   TCATCCCCGATGGCATCTATGACCCAGCAGAT 112
```

ENSG00000163959 intron 5

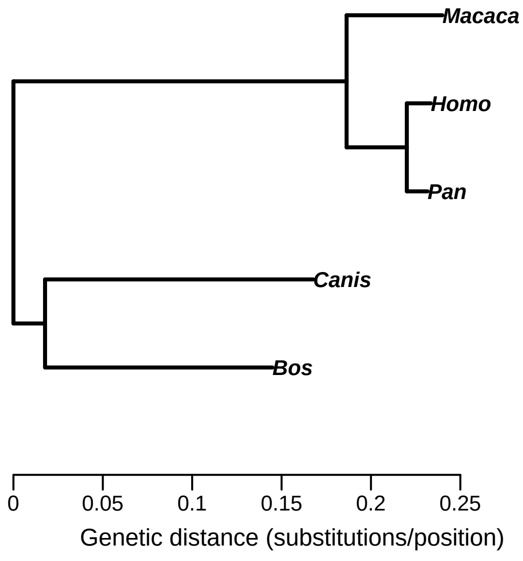

ENSG00000163959 intron 5

```
Homo    GTGAGGCGGGGCCAAGGTGCCTTCCCCAGGAGCCGG------------------------GGAGCCTCTCCTGGACCCCT--GGTCCCCTTCAAGGCTCT 74
Pan     GTGAGGCGGGGCCAAGGTGCCTTCCCCAGGAGCCGG------------------------GGAGCCTCTCCTGGACCCCT--GGCCCCCTTCAAGGCTCT 74
Macaca  GTGAGGCAGGGCCAAGGTGCCTCCTCCAGGAGCTGG------------------------GGAGCTTCTGCTGACCCCCT--AGCCCTCTTCAAGGCTCT 74
Bos     GTGAGGCGGGAGGGAGAGGCTGCCT-GGAGAGCAGACTGGCCTCT-TCTGCACTCTCTCAAGAGCCTCTGCTGGCTCTCTCAGTCCTGCTTCAAGCCTCA 98
Canis   GTAAGGTGGGAGGGAGAGGCTCCTTCAAAGAACAGGTTGGCCTCTACCTGCACTCTCCCAAGAGCCTCT-CCGGCCCCCTCAGCCCTGCTTCAAGCCTCT 99

Homo    GGGAATTAGGCGTGAATAGGCCAAAGCCAGTGACGGAAGGGTGGCATCCCACGGCCAGGACCCAC-----GGGCGCCACCCTCAGGGGACAGGGGAGCA 169
Pan     GGGAATTAGGCGTGAATAGGCCAAAGCCAGTGACGGAAGGGTGGGTATCCCACAGCCAGGACCCAC-----GGGCGCCACCCTCAGGGGACAGGGGAGCA 169
Macaca  GGGAATTCGGCGTGAATGGGCCAAAGCCCGTGACGGAAAGGTGGCATCCCACAGCCAGGACTCAG-----GGGCACCAACCTCAGGGGACAGGGGAGCA 169
Bos     TGGAATTTGGCTTGTATGGAACAAAGCCAGTGATGAAAGGGTGGCATCCCACAGCCAGGACCCACACACTGGGCACC-CAAACAGGGGTAGGAGGAACA 197
Canis   TGGGATCAGGCTTGTATGGAACAAAGCCGGTGATGAAAGGGTGGCATCCCACAGCCAACATCCACACATTGGGCACC-CGTTCAGGGGTGAGGATGGCA 198

Homo    ----GAGGAAGGAGAGCTCCTCAATTGTCATCACTGAACTTCACTGCACCCTGCAAGCCTTAGCCACACAGAGAAAACTGGACTTGGGGCCAT----TCG 261
Pan     ----GAGGAAGGAGAGCCCCTCAGTTCTCATCACTGAACTTCACTGCACCCTGCAAGCCTTAGCCACACAGAGAAAACTGGACTTGGGGCCAT----TCG 261
Macaca  ----GAGGAAAGAGAGGTCCTAAATTCTCATCACTGAACTTCACTGCACCCTGCAAGCCTTAGCCACACAGAGAAAACTGGACTTGGGGCCATTTGCTTG 265
Bos     ----GAGGAAGGAGAACTCCAGAGTTCCCATCACTTACGCTCACTGTCCCCTACAAACTTTA-TCACAGAGAACAAACTGCACTTTGGACTAT----TTA 288
Canis   GAGGGAGGAAAGAGAACTCC--AATTTCATCATTTAAGCTCACTGCTGCCTACAAACTTTAGCTACATGGAGTAAAGTGGACTTTGGTCTAC----ATA 292

Homo    CTTGCCCCTTCTGCCCACTTTGGTGAATGAGGTTGAGGTCACACTCC---CAGACCTCGCTCCAAAGACGGAATTCTTGTCTGGAGGTGAGTGGGAGAC- 357
Pan     CTTGCCCCTTCTGCCCACTTTGGTGAATGAGGTTGCGGTCACACTCC---CAGACCTCGCTCCAAAGACGGAATTCTTGTCTGGAGGTGAGTGGGAGAC- 357
Macaca  CTTGCCCCTTCTGCCCACTTTGGTGAATGAGGTTGAGGTCACACTCC---CAGACCTCGCTCCAAAGACAGCATTCTGGTCTGGAGGTGAGCGGGAGAC- 361
Bos     CTTTCCCCTTCCATCCGTTTCAGTTAATGAGGTTGGGGTCAATCCTCAGATAGTTCTTGCTCAAAAGGCATCATTCTGGTCTGAGGGTGAGTGGGAGGCA 388
Canis   CTTGTCCCTGCTGTCCATTTTGGTTGATGAGGCTGGGGTCAACATTCTGATAGTTCCTGCTCAAAAGGCAGAATTCTGGTCTGGGGCTGAGGGGGAGGCA 392

Homo    --CAAGAGGGTTCCCAGCACTCTCAACATTCTGCTTTTTTCCTCTGTCCCCATCCACTTAACCTGGTTGGTGTTTATGACAGCAGCCGAGCCTCAGC-CC 454
Pan     --TAAGAGGGTTCCCAGCACTCTCCACATTCTGCTTTTTTCCTCTGTCCCCATCCACTTAACCTGGTTGGTGTTTATGATGGCAGCCGAGCCTCAGC-CT 454
Macaca  --TAAGAGGGTTCCCAGCACTCTCAACATTCTGGTTTTTTCCTCCGTCCCCACCCACTTAACCTGGCTGGTGTTTATGGTGGCAGCCAAGCCTCGGC-GC 458
Bos     ATAAAGATGGTTCCCAATG----------------CTCTTCCTAAGTCCCCACCCACTTGACTATGTTGGTGTCTATGATGGTGGCCAAGCCTTG---CC 469
Canis   A-GACGAGGGTTCCCAGTGTTCTCAACATTCCGGCTTCTTCCTATATCCCCATCCATTTAGCCATTTCGTTATCTATTATGGCAGCCAAGCCTCAGCTCA 491

Homo    AGGAGCCCT-GTGAG-GAGCCGGGGCGTCTTCCTGGGGCAGGGGGTTTGTGGGCCCATGTTCCTAACCCTCTTCCCCACTCAG 535
Pan     AGGAGCCCA-GTGAG-GAGCCGGGGCGTCTTCCTGGGGCAGCGGGTTTGTGGGCCCATGTTCCTAACCCTCTTCCCCACTCAG 535
Macaca  AGGAGCCCA-GCGAG-GAG-CGGGGGGTCTTCCTGGGACAGCTGGTTTGT-GGCCTGTGTTCCTCACCCTCTTCCCCACGCAG 537
Bos     AGGAACCCA-ATGAGAGAGCTGGAAGTTCTGGTTGGGGTGGGTGATTTGTAGGCCTATGTTCCTAATCCTCTTCCTACTTCAG 551
Canis   AGGAGCCCAGGTGAGGGAGGTGGAAGGTGTTCTTGAGGTGGGCAGTTTGTGGGTTCATGTTTCCAACTCTCCTTTCCTTCAG 574
```

ENSG00000092929 intron 27

Description: Unc-13 homolog D (UNC13D)
Intron number: 27
Human chromosome: 17
Intron start (bp): 71338338
Human intron length : 436
Intron alignment length: 473
Flanking exons length (upstream/downstream): 72/84
SNP density: 0.000000
K tree score: 0.0933
Scaling factor: 0.3943
Human-chimpanzee distance: 0.023886
Total primate branch length: 0.1129

ENSG00000092929 exon 27

```
Homo    AACCTGGAGATCTGCTTCCACGCTGAGGGCTGTGGCCTGCCACCCAAGGCCCTGCACACTGCCACCTTCCAG 72
Pan     AACCTGGAGATCTGCTTCCACGCTGAGGGCTGTGGCCTGCCACCCGAGGCCCTGCACACTGCCACCTTCCAG 72
Macaca  AACTTGGAGATCTGCTTCCACGCTGAGGGCTGTGGCCTGCCGCCCGAGGCTCTGCACACTGCCACCTTCCAG 72
Bos     AACCTGGAGGTCTGCTTCTACGCAGAGGGATGTGGCCTGCCGCCCGAGGCCCTGCACACCGCCACTTTTCAG 72
Canis   AATCTGGAGATCTGCTTCTATGCGGAGGGCTGTGGCCTGCCACCTGCGGCGCTGCACACAGTCACCTTCCAG 72
```

ENSG00000092929 exon 28

```
Homo    GCTCTGCAGAGGGACCTGGAGCTGCAGGCGGCCTCCAGCCGGGAACTCATCCGGAAGTACTTCTGCAGCCGAATCCAGCA 80
Pan     GCTCTGCAGAGGGACCTGGAGCTGCAGGCGGCCTCCAGCCGGGAACTCATCCGGAAGTACTTCTGCAGCCGAATCCAGCA 80
Macaca  GCTCTGCAGAGGGACCTGGAGCTGCAGGCAGCCTCCAGCCGGGAGCTCATCCGGAAGTACTTCTGCAGCCGCATCCAGCA 80
Bos     GCTCTGCAGAGGGACCTGGAGCTGCAGGCGGCCTCCAGTCGGGAGCTCATCCACAAGTACTTCTGCAGCCGCCTCCAGCA 80
Canis   GCTCTGCGGAGGGACCTGGAGCTGCAGGCAGCTTCCAGCCGGGAGCTCATCCAGAAGTACTACTGCAGCCGCATCCAGCA 80

Homo    GCAG 84
Pan     GCAG 84
Macaca  GCAG 84
Bos     GCAG 84
Canis   GCAG 84
```

ENSG00000092929 intron 27

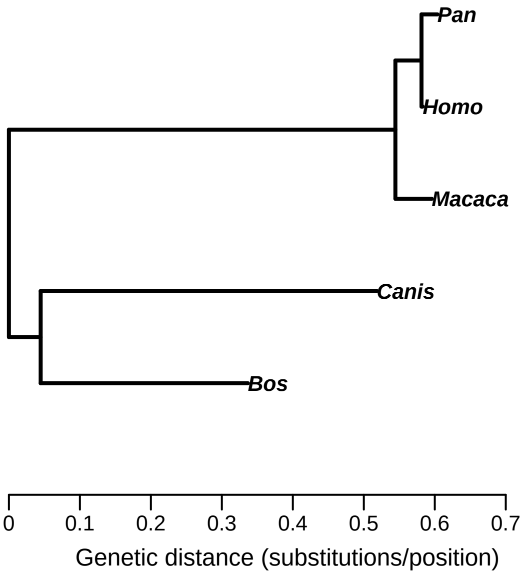

ENSG00000092929 intron 27

```
Homo    GTAGGGGCTTTGGGTGAGCTTCCCCGTAGGAAGTCTGGGGAGCCTGCCTTTTGGCACTCACCTATGTCCAGGGTCGGCTACATAATTTATGGGGCCCAGT 100
Pan     GTAGGGGCTTTGGGTGAGCTTCCCCGTAGGAGGTCCGGGGAGCCTGTCTTTTGGCACTCACCTATGTCCAAGGTCGGCTACATAATTTATGGGGCCCAGT 100
Macaca  GTAGGGGCTTTGGGTGAGCTTCCCCGCAGGAGGTCCGGGGAGCCTGCCTTTTGGCACTCACCTATGTCCAGGGCCAGCTACATAATTTATGGGGCCCAGT 100
Bos     GTAGGCCCTTTGGGTGAGCTGGC------GGCATTG-GGGAGCCTGCCTTCCTGTGCTCGCCTGTGTCCAGGGCCTGCTGCATAATTTACAGGGCTCAGC 93
Canis   GTGGGGGTCTTTGGTGAGCTGGT------GGGGTGGAGGGAGCCTGCCTTTCTGTGT---CCTGTGTCCCGTATTAACTACATAATTTACAGGGCCCAGC 91

Homo    ACAAAATGAAAAACTGGGCCCCTTATTCAAAAGTTA---T----------TACAAATTTTAGAACGGTGACGACAGAACCTGAAACCAAGCCTGGGTCCT 187
Pan     ACAAAATGAAAAACTGGGCCCCTTATTCAAAAGTTA---T----------TACAAATTTTAGAACGGTGACGACAGAACCTGAAACCAAGCCTGGGTCCT 187
Macaca  ACAAAATGAAAAGCTGGGCCCCTTATTCAAAAGTTA---TTCAAAAGTTATTCAAATTTTAAAACGGTGACAACAGAACCTGAAACCAAGCCTGGGTCCT 197
Bos     ACAAAATGAAAATATGGGTCCTTTCTTAAAAAAAAAAAAAGAAAAAATTTTGAAAAATTTAAGACAGCAACAGCAGGAGCTTAAACCGAGTGTGAGGTTC 193
Canis   ACAAAAAGAAAATGTGGGCCCCTTTTCCAAAAATCA-------------TTAAAAATTTAAAGACAGCAACGGCAGAGCATTAAACCAAACATAAAGCTC 178

Homo    CCTAAGCACAGGGGCCCTGTGTGCCCGCA-TGGGG-GGTGGGGTCGGGGAAGCACGCCTGTGAAGCCAGCCCTGTCTGTGCCTCTCCTGCTGTCTGTCTG 285
Pan     CCTAAGCACAGGGGCCCTGTGTGCCCACA-TGCGG-GGTGGGGTCGGGGAAGCATGCCTGTGAAGCCAGCCCTGTCTGTGCCTCTCCTGCTGTCTGTCTG 285
Macaca  CCTAAGCACA-GGGCCCTGTGTGCCTGCACGGGGGCGGTGGGGTCAGGGGAGCACACC-GTGAAGCCAGCCTTGTCTGTGCCTCTCCTGCTGTCTGCCTG 295
Bos     TTC--------------------TAAAGCCGGTTGGGGCAGGGCAGCTGAGACACACCTGCGGAGCCCGCTGCGTCGGTGCCT---CTG-------GCTG 263
Canis   TTCAGGCACG----------GTGAGGGGGTGGCCGTGCCATGGTAAGGGTGGC-CACCCTCGAGGCCCATTGCGTCCGTGCCG---CGGCAGGCCTGCCG 264

Homo    GCCTGAGG--GTGGCCCCAGCCCGTTGTCCCATG--ACCCAATACTTTCCATTTCAAACGGACATG-----------TTACCCTTGACAGTGAGCTCCT- 369
Pan     GCCTGAGG--GTGACCCCAGCCCGTTGTCCCATG--ACCCAATACTTTCCATTTCAAACGGACATG-----------TTACCCTTGACGGTGAGCTCCT- 369
Macaca  GCCTGAGG--GTGGCTCCTGCCCGTGGCCCCATG--ACCCAATACTTTCCATTTCAAACGGGCATC-----------TTACCCTTGACCGCGAGCTCCT- 379
Bos     TCGCAGGG--ACAACCCTGGCTTGTGGTCCCAGGGCACCCGGTGCTTTCCACATCA---CTCTGTGGGCT-------TTCCCATCGGCCGTGACCTTCT- 350
Canis   TCCTGAGGGTGCGTTCCCCAGGTGAGCATCTGGGGCACCCGGTGCTTTCTGTATCAAATTGCTGTTGGCTGAATCAAACCCTCTGGGCTGCGTTTTCCCG 364

Homo    -----GGAGGGCAGGCCTGGGCTGTGGTTGGGCGTG-CAGTCCAGGTGGATCCTCAGCTGTATGTGTTCCCAG 436
Pan     -----GGAGGGCAGGCCTGGGCTGTGGTTGGGCGTG-CAGTCCAGGTGGATCCTTAGCTGTATGTGTTCCCAG 436
Macaca  -----GGAGGGCGGGCCCTTGCTGTGGTTGGGCTTG-CGGTCCATTTGGATTCTCAGCTGTATGTGTTCCCAG 446
Bos     -----GAAGGGCAGCCCTGGGCTGCGGTTGGGGGTGACGGTTCAGGGTGT----------GTCCCCTCCTCAG 408
Canis   AAGGGGAAGGGCGGCCCCGGGCTGCGGTGGAGAATGGCAGTTCGCGGGACTGCTCGGCTATGCCTGTTCGCAG 437
```

ENSG00000105618 intron 10

Description: U4/U6 small nuclear ribonucleoprotein Prp31 (PRPF31)
Intron number: 10
Human chromosome: 19
Intron start (bp): 59323565
Human intron length : 679
Intron alignment length: 696
Flanking exons length (upstream/downstream): 73/129
SNP density: 0.014728
K tree score: 0.0646
Scaling factor: 0.4942
Human-chimpanzee distance: 0.044246
Total primate branch length: 0.1125

ENSG00000105618 exon 10

```
Homo    GTACCGCAAGATGAAGGAGCGGCTGGGGCTGACGGAGATCCGGAAGCAGGCCAACCGTATGAGCTTCGGAGAG 73
Pan     GTACCGCAAGATGAAGGAGCGGCTGGGGCTGACGGAGATCCGGAAGCAGGCCAACCGTATGAGCTTCGGAGAG 73
Macaca  GTACCGCAAGATGAAGGAGCGGCTGGGGCTGACAGAGATCCGGAAGCAGGCCAACCGGATGAGTTTCGGAGAG 73
Bos     GTACCGCAAGATGAAGGAGCGGCTGGGGCTGACAGAGATCCGGAAGCAGGCCAACCGCATGAGCTTCGGAGAG 73
Canis   GTACCGCAAGATGAAGGAGCGGCTAGGGCTGACCGAGATCCGGAAGCAGGCCAACCGCATGAGCTTCGGAGAG 73
```

ENSG00000105618 exon 11

```
Homo    ATCGAGGAGGACGCCTACCAGGAGGACCTGGGATTCAGCCTGGGCCACCTGGGCAAGTCGGGCAGTGGGCGTGTGCGGCA 80
Pan     ATCGAGGAGGACGCCTACCAGGAGGACCTGGGATTCAGCCTGGGCCACCTGGGCAAGTCGGGCAGTGGGCGTGTGCGGCA 80
Macaca  ATTGAGGAGGACGCCTACCAGGAGGACCTGGGATTCAGCCTGGGCCACCTGGGCAAGTCGGGCAGCGGGCGCGTGCGGCA 80
Bos     ATCGAGGAGGACGCCTACCAGGAGGACCTGGGTTTCAGCCTGGGCCACCTGGGCAAGTCGGGCAGCGGGCGGGTGCGGCA 80
Canis   ATCGAGGAAGACGCCTACCAAGAGGACCTGGGCTTCAGCCTGGGCCATCTGGGCAAATCAGGCAGCGGGCGGGTACGGCA 80

Homo    GACACAGGTAAACGAGGCCACCAAGGCCAGGATCTCCAAGACGCTGCAG 129
Pan     GACGCAGGTAAACGAGGCCACCAAGGCCAGGATCTCCAAGACGCTGCAG 129
Macaca  GACGCAGGTCAACGAGGCCACCAAGGCGAGGATCTCCAAGACGCTGCAG 129
Bos     GACGCAGGTGAACGAGGCCACCAAAGCCAGGATTTCCAAGACGTTGCAG 129
Canis   GACACAGGTGAACGAGGCCACCAAAGCCAGGATCTCTAAGACACTGCAG 129
```

ENSG00000105618 intron 10

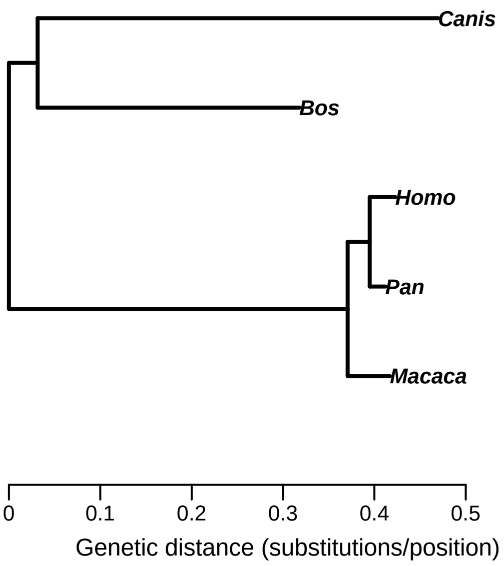

ENSG00000105618 intron 10

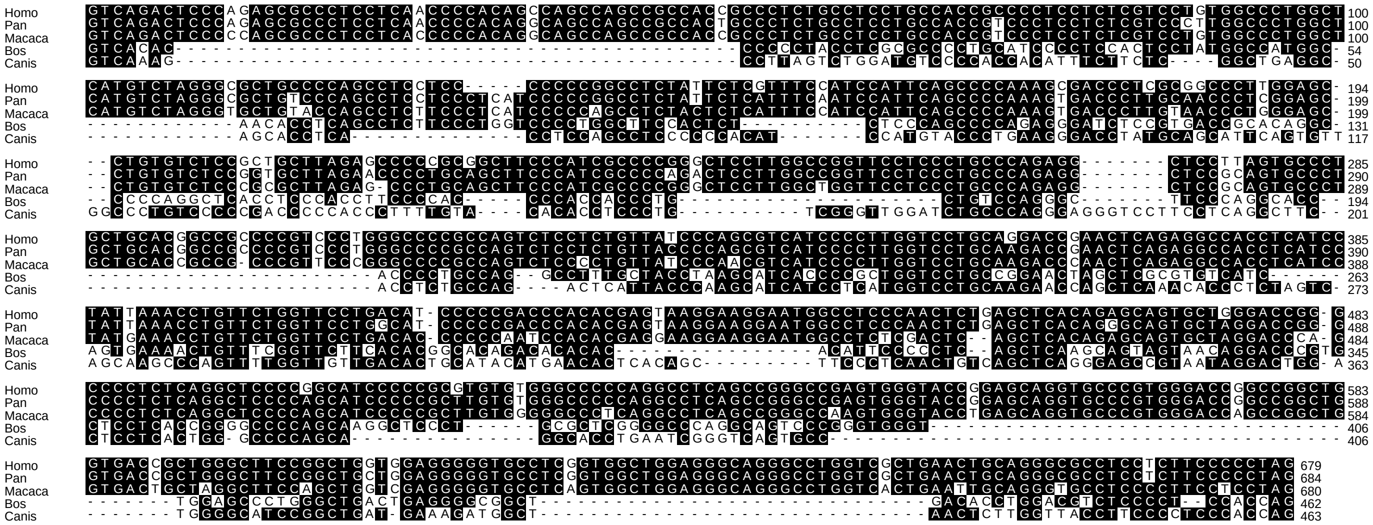

ENSG00000123454 intron 3

Description: Dopamine beta-hydroxylase precursor (DBH)
Intron number: 3
Human chromosome: 09
Intron start (bp): 135497408
Human intron length : 948
Intron alignment length: 1143
Flanking exons length (upstream/downstream): 258/177
SNP density: 0.006329
K tree score: 0.0508
Scaling factor: 0.6727
Human-chimpanzee distance: 0.017283
Total primate branch length: 0.1117

ENSG00000123454 exon 3

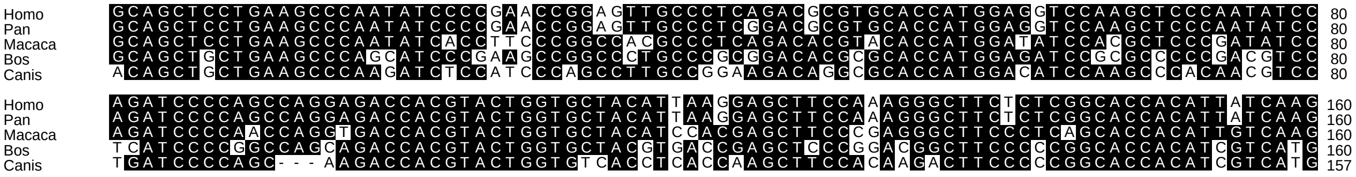

ENSG00000123454 exon 4

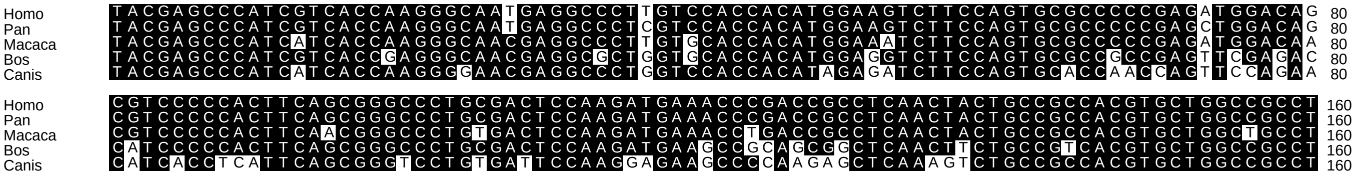

ENSG00000123454 intron 3

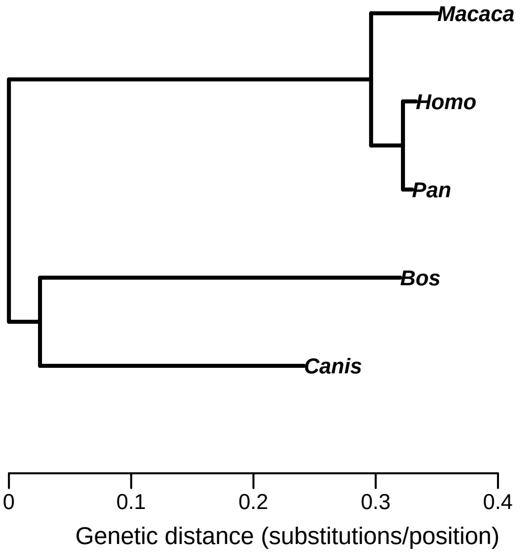

ENSG00000123454 intron 3

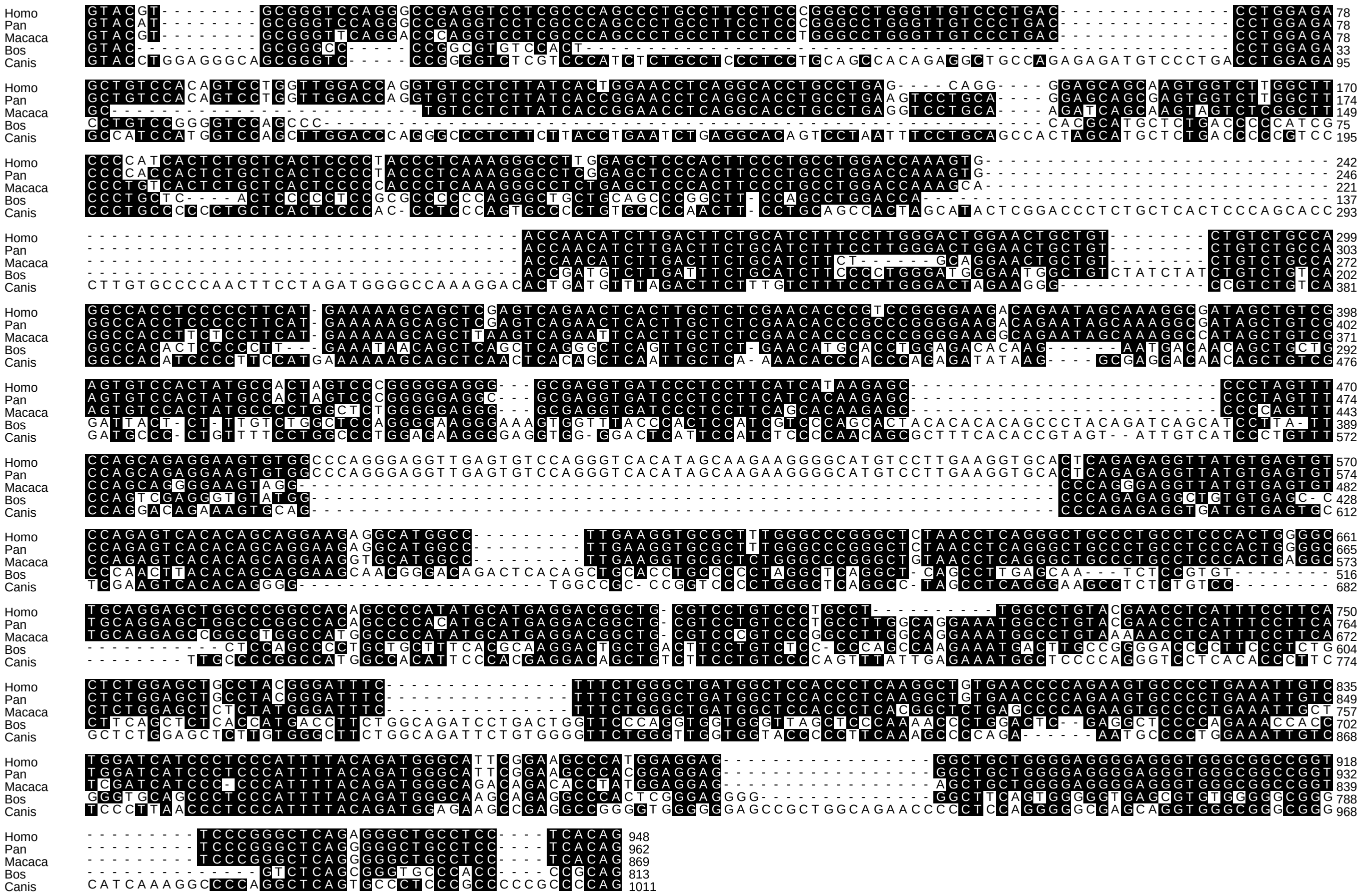

ENSG00000174343 intron 1

Description: Neuronal acetylcholine receptor protein sub alpha-9 precursor (CHRNA9)
Intron number: 1
Human chromosome: 04
Intron start (bp): 40032306
Human intron length : 295
Intron alignment length: 331
Flanking exons length (upstream/downstream): 64/146
SNP density: 0.003390
K tree score: 0.0776
Scaling factor: 0.894
Human-chimpanzee distance: 0.019918
Total primate branch length: 0.1116

ENSG00000174343 exon 1

```
Homo    ATGAACTGGTCCCATTCCTGCATCTCCTTTTGCTGGATCTACTTTGCTGCTTCCAGACTGAGAG 64
Pan     ATGAACTGGTCCCATTCCTGCATCTCCTTTTGCTGGATTTACTTTGCTGCTTCCAGACTGAGAG 64
Macaca  ATGAACTGGTCCCATTCCTGCATCTCCTTTTGCTGGATTTACTTTGCTGCTTCCAGACTGAGAG 64
Bos     ATGAACTGGCCCCATTCCTGCATCTCCTTTTGCTGGATTTACTTTGCTGCCTCCAGACTGAGAG 64
Canis   ATGAACTGGCCCCATTCCTGCATCTCCTTTTGCTGGATTTACTTTGCTGTCTCTAGGCTGAGAG 64
```

ENSG00000174343 exon 2

```
Homo    CTGCAGAGACGGCAGATGGAAAATATGCTCAGAAGTTGTTTAATGACCTTTTTGAAGATTATTCTAATGCTCTTCGTCCA 80
Pan     CTGCAGAGACGGCAGATGGAAAATATGCTCAGAAGTTGTTTAATGACCTTTTTGAAGATTATTCCAATGCTCTTCGTCCA 80
Macaca  CTGTGGAGACGGCAGATGGAAAATATGCTCAGAAGTTGTTTAATGACCTTTTTGAAGATTATTCCAATGCTCTTCGTCCA 80
Bos     CTGTAGAACCAGCAGATGGAAAATATGCTCAGAAGTTGTTCAATGACCTTTTTGAAGACTACTCTAATGCTCTTCGTCCA 80
Canis   CTGTAGAAACAGCAGATGGAAAATATGCTCAGAAGTTGTTTAATGACCTTTTTGAAGATTATTCCAATGCTCTTCGTCCA 80

Homo    GTGGAAGATACAGATAAAGTCCTGAATGTGACCCTGCAGATTACGCTCTCTCAGATTAAGGATATG 146
Pan     GTGGAAGATACAGATAAAGTCCTGAATGTGACCCTGCAGATTACGCTCTCTCAGATTAAGGATATG 146
Macaca  GTGGAAGATACAGATAAAGTCCTGAATGTCACACTGCAGATTACGCTCTCTCAGATTAAGGATATG 146
Bos     GTAGAGGATACAGATAAAGTCCTGAACGTCACACTCCAGATTACACTTTCTCAGATTAAGGATATG 146
Canis   GTAGAAGATACAGATAAAGTTCTGAATGTCACCCTGCAGATTACACTTTCTCAGATTAAGGACATG 146
```

ENSG00000174343 intron 1

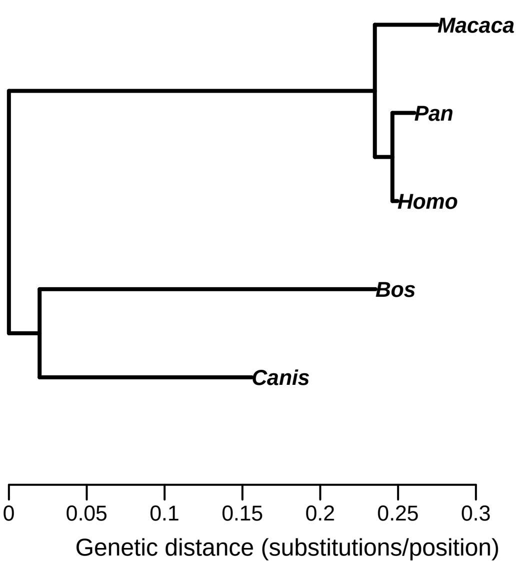

ENSG00000174343 intron 1

```
Homo    GTGAGAGGCTGGT-GACACGTAACCTATCTTGTCTCATCTGGGGCTATTGCTTTGGTGGGAGGTGGGGCAGGACAGGGAAGACAAAACAGATGATTCAAC 99
Pan     GTGAGAGGCTGGT-GACATGTAACCTATCTTGTCTCATCTGGGGCTATTGCTTTGGTGGGAGGTGGGGCAGGACAGGGAAGACAAAACAGATGATTCAAC 99
Macaca  GTGAGCGGCTGGT-GACATGTAACC-ATCTTGTCTCATCTGGGGCTATTGCTTTGGTGGGAGGTGGGGCAGGGCGGGGAAGACAAAACAGATGATTCCAC 98
Bos     GTAAGAGGCTGTTGGGTGTGCAACT-CTCTTAGCCCA---GGGATCACTTCTTTGGTGGAATGTTGGGTGGGCCAGG--AAA-AGAACAGACGCTCTAAC 93
Canis   GTAAGAGGCTGATGGGTGTGTAATC-ATCTCAGTTCACCTGGGGCCCCTTCTTTGGTGG------GAGTGGGCCAGC--AAACAGAACAAATATTCTAAC 91

Homo    CGCATGGAAGTGTTCTGTGCTTCCTACTGACTCTGAT---CTTGGTAGGGCATGCCAGAAAAACAACTCATCCATCCACCCAAAGCTTGTCCCTCGCCAG 196
Pan     CGCATGGAAGTATTCTGTGCTTCCTACTGACTCTGAT---CTTGGAAGGGCATGCCAGAAAAACAACTCATCCACCCACCCAAAGCTTGTCCCTCGCCAG 196
Macaca  CGCATGGAAGGGCTCCGTGCTTGCTACTGCCTCTGAC---CTTGGTAGGGCATGCCAGAAAAACAACTCATCCATCCACCCAAAGC-TGTCCCTCGCCAG 194
Bos     TGTGAGGAAGTATTTC-AGCTTCTTACAGTCTCATGCTGATCCTGTAGGGCCTGCCAGAGAAATAACTC--CACTTGACCCAGAGCTTGTCCCCAGCTGA 190
Canis   TGCAAGGAAGTGTTCT-TGCCTCTT--------------------CTTGACTGGCCGGAAAAATGACTC--TCATATACCCAAAGCTTGTCCCTAGCTGA 168

Homo    TGTTGGGC--------------------------------CATCCCCCATTTTAGACCTCAGTCTTGTGTTGTTTGTCTTGAAATACACACAACACTCTG 264
Pan     TGTTGGGC--------------------------------CATCCCCCATTTTAGACCTCAGTCTTGTGTTGTTTGTCTTGAAATACACACAACACTCTG 264
Macaca  TGCTGGGC--------------------------------CATCCCCCATTTTAGACCTCAGTCTTGTGTTCTTTGTCTTGAAATACATACAACACTCTG 262
Bos     TGGTAGGTAGGTAGATAGCTAGGATGGTAGGCAGGGATGATAGAACCAATGCTAGTCCTTGGTCTGTTGCTCTTTGCCTTGAAACACTCATA--ACACTG 288
Canis   TGCCAGGT--------------------------------CATGCCCAGTTCTAGTCCTCAGTTTGTTGTTCTTTACCTTGAAATATGCATG--ACTTTG 234

Homo    AATGTTAATCCAGATCCCTCTTGTTGAGTAG 295
Pan     AACGTTAATCCAGATCCCTCTTGTTGAGTAG 295
Macaca  AAGGTTGATCCAGATCCCTCTTGTTGAGTAG 293
Bos     AAGCTTGATCCAGATCCCGCTTTTTGAGCAG 319
Canis   AATGTTGATCCAGGTCCCTCTTTTTGAGCAG 265
```

ENSG00000064835 intron 5

Description: Pituitary-specific positive transcription factor 1 (POU1F1)
Intron number: 5
Human chromosome: 03
Intron start (bp): 87391945
Human intron length : 1168
Intron alignment length: 1388
Flanking exons length (upstream/downstream): 61/211
SNP density: 0.001712
K tree score: 0.0526
Scaling factor: 1.0185
Human-chimpanzee distance: 0.018920
Total primate branch length: 0.1098

ENSG00000064835 exon 5

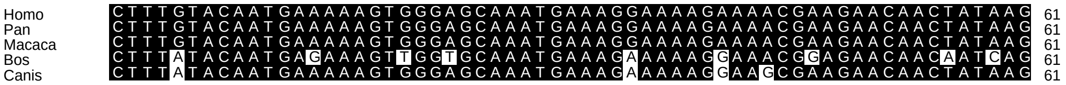

ENSG00000064835 exon 6

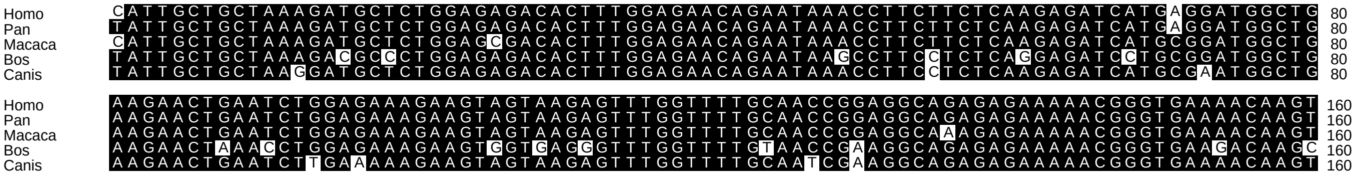

ENSG00000064835 intron 5

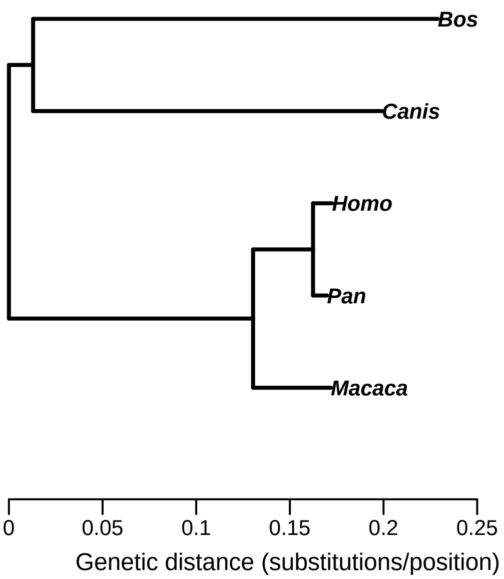

ENSG00000064835 intron 5

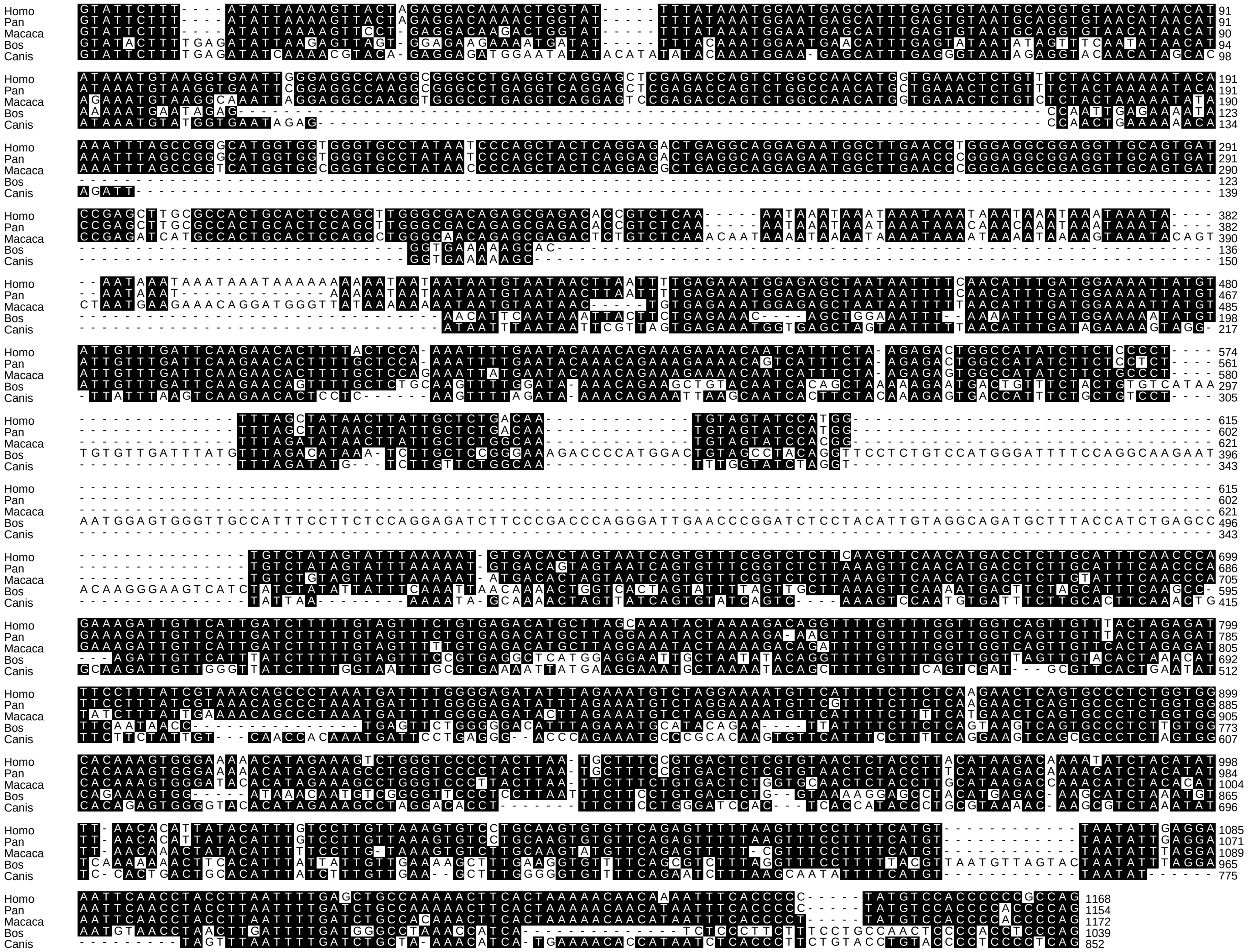

ENSG00000106638 intron 2

Description: Transducin beta-like 2 protein (TBL2)
Intron number: 2
Human chromosome: 07
Intron start (bp): 72626389
Human intron length : 260
Intron alignment length: 275
Flanking exons length (upstream/downstream): 131/185
SNP density: 0.000000
K tree score: 0.0727
Scaling factor: 0.839
Human-chimpanzee distance: 0.011691
Total primate branch length: 0.1097

ENSG00000106638 exon 2

```
Homo   GCCAAAAAGCAAATGGATTTCCACCTGACAAATCTTCGGGATCCAAGAAGCAGAAACAATATCAGCGGATTCGGAAGGAG 80
Pan    GCCAAAAAGCAAATGGATTTCCACCTGACAAATCTTCGGGATCCAAGAAGCAGAAACAATATCAGCGGATTCGGAAGGAG 80
Macaca GCCGAAAAGCAAATGGATTTCCACCTGACAAATCCTCAGGATCCAAGAAGCAGAAACAGTATCAGCGGATTCGGAAGGAG 80
Bos    GCCAAAAGGCAAACGGATGTCCACCTGACAAGTCCTTGAAATCCAAGAAGCAGAAACTGCAACAGCGGGTTCGCAAGGAG 80
Canis  GCCAAAAAGCAAATGGATTTCCACCTGACAAGTCCTTGAAGTCCAAGAAGCAGAAACAGCATCAACGAATTCGCAAGGAG 80

Homo   AAGCCTCAACAACACAACTTCACCCACCGCCTCCTGGCTGCAGCTCTGAAG 131
Pan    AAGCCTCAACAACACAACTTCACCCACCGCCTCCTGGCTGCAGCTCTGAAG 131
Macaca AAGCCTCAACAACACAACTTCACCCACCGCCTCCTGGCTGCAGCTCTGAAG 131
Bos    AAGCCTCAGCAACACAACTTCACCCACCGCCTTCTGGCTGCAGCACTGAAG 131
Canis  AAGCCTCAGCAGCACAATTTCACTCACCGCCTCCTGGCTGCGGCATTAAAG 131
```

ENSG00000106638 exon 3

```
Homo   AGCCACAGCGGGAACATATCTTGCATGGACTTTAGCAGCAATGGCAAATACCTGGCTACCTGTGCAGATGATCGCACCAT 80
Pan    AGCCACAGCGGGAACATATCTTGCATGGACTTTAGCAGCAATGGCAAATACCTGGCTACCTGTGCAGATGATCGCACCAT 80
Macaca AGCCACAGCGGGAACATATCTTGCATGGACTTTAGCAGCAATGGCAAATACCTGGCCACCTGTGCAGATGATCGCACCAT 80
Bos    AGCCACAGTGGGAACATAACTTGCATGGACTTCAGCAGCAATGGCAAGTACCTGGCTACCTGTGCAGACGACCGTACCGT 80
Canis  AGCCACAGTGGGAACATATCTTGCATGGACTTTAGCAGCAATGGCAAGTACCTGGCCACCTGTGCAGATGACCGCACTGT 80

Homo   CCGCATCTGGAGCACCAAGGACTTCCTGCAGCGAGAGCACCGCAGCATGAGAGCCAACGTGGAGCTGGACCACGCCACCC 160
Pan    CCGCATCTGGAGCACCAAGGACTTCCTACAGCGAGAGCACCGCAGCATGAGAGCCAACGTGGAGCTGGACCACGCCACCC 160
Macaca CCGCATTTGGAGCACCAAGGACTTTCTGCAGCGAGAGCACCGCAGCATGAGAGCCAACGTGGAGCTGGACCATGCCACCC 160
Bos    CCGCATCTGGAGCACAAAGGACTTCCTGCAGCGGGAGCACCGCAGCATGAGAGCCAACGTGGAACTGGACCATGCCATCC 160
Canis  CCGCATCTGGAGCACCAAGGACTTCCTGCAGCGGGAGCACCGCAGCCTGAGAGCCAACGTGGAGCTGGACCATGCTACCT 160
```

ENSG00000106638 intron 2

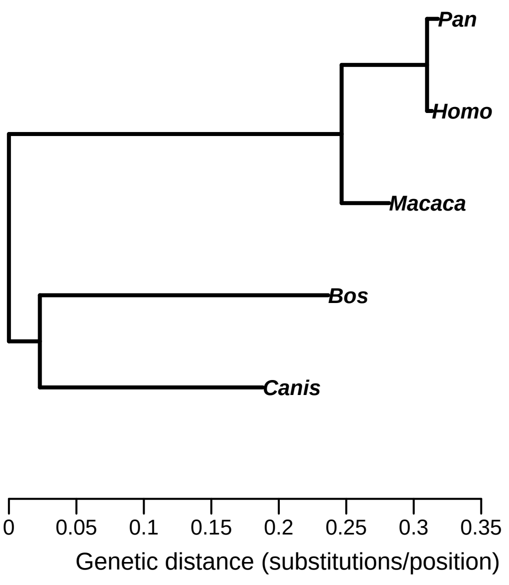

ENSG00000106638 intron 2

```
Homo    GTACGTGGGGTACCTGGCACAGCGTGC--ACCCTGAGACTCAG-------------CCCCACAGGCTTTTCCTCTGGAGTATTCCATGCATGTTACAGGC 85
Pan     GTACGTGGGGTACCTGGCACAGCGTGC--ACCCTGAGACTCAG-------------CCCCACAGGCTTTTCCTCTGGAGTATTCCATGCATGTTACAGGC 85
Macaca  GTACGCGGGGTACCCAGCGCAGTGCTC--ACTCTGAGACTCAG-------------ACCCACAGGCTTTTCCTCTGTAGTATTCTGTGCATGTTATAGAC 85
Bos     GTACACGGGTACCCA-GCACAGCATGTATATATGAAGACTCATGG-----------CCCCGCAGACTTCTCCTTTGTAGGATTCTGTGCATGTTACAGAC 88
Canis   GTACATGGATACCCATGCTCAGCATACATATACAGAGACTCACAGCCCCCCCCCCCCCCCATAGACTTTTCCTTTTTAAGGGTCTGCACATG--ACAGAC 98

Homo    TCTCCTGCCTCTGATCAGAATCAGCGGGGGTGCTTAATTCTGTCCCCTCTCATCGCAGCCCAATCTCAGCTTGCTGTTAATCCAGGCCACAGGGCCCCAT 185
Pan     TCTCCTGCCTCTGATCAGAATCAGCGGGGATGCTTAATTCTGTCCCCTCTCACCGCAGCCCAATCTCAGCTTGCTGTTAATCCAGGCCACAGGGCCCCAT 185
Macaca  CCTCCTGCCTCTGATCAGAATCAGCGGGGATGCTTAATTCTGTCCCCTCTCATCGCAGCCCAACCTCAGCTTGCTGTTAATCCAGGCAACGGGGCCCCAT 185
Bos     TCCCCTGCCTCTGACCCAGATCAGGGGAGACGCCCTGTCCCATCTC--CTCCTGACGTCCCATCAGGAGTTTGCTGTTGATCTAAGCAACAGGGTCCCTG 186
Canis   CCTTCTGCCTCTGACTAAAGTTAGTGGGGATGCCCCATCCAATC-C--CTCATCATACCCCATCAGCAGCTTGTCGTTAATCCAGGGAGCAAA------- 188

Homo    CTCCCCGGCTCCAGGTTTGGCTTCCCCATCTCCCCCAGGAGCTCCTATTGACACTTCCTGCCACCCTTCCCCTAG 260
Pan     CTCCCCGGCTCCAGGTTTGGCTTCCCTATCTCCCCCAGGAGCTCCTATTGACACTTCCTGCCACCCTTCCCCTAG 260
Macaca  CTCCCTGGTTCCAGGTTTGGCTTCCCCATCTCCCCAAGGAGCTCCTATTCACACTTCCTGCCACCCTTTCCCTAG 260
Bos     AGTCATGGTTGCACG---GGTTTCCGCATTTGCCCAGGGGGTTCCTGTTCATACCTGCTGCTGCCCTTGCCCTAG 258
Canis   -----------------------CCCCATCTGCCCAAGGGGTCCCTGTTCATACTTCCTGCAACCCTTCCCTTAG 240
```

ENSG00000166135 intron 5

Description: Hypoxia-inducible factor 1 alpha inhibitor (HIF1AN)
Intron number: 5
Human chromosome: 10
Intron start (bp): 102295797
Human intron length : 468
Intron alignment length: 479
Flanking exons length (upstream/downstream): 107/64
SNP density: 0.004274
K tree score: 0.0487
Scaling factor: 1.1381
Human-chimpanzee distance: 0.015118
Total primate branch length: 0.1089

ENSG00000166135 exon 5

```
Homo    GTGGACTTTGACAATCCCGACTACGAGAGGTTCCCTAATTTCCAAAATGTGGTTGGTTACGAAACAGTGGTTGGCCCTGG 80
Pan     GTGGACTTTGACAATCCCGACTACGAGAGGTTCCCTAATTTCCAAAATGTGGTTGGTTACGAAACAGTGGTTGGCCCTGG 80
Macaca  GTGGACTTTGACAATCCCGACTACGAGAGGTTCCCTAATTTCCAAAATGTGGTTGGTTACGAAACAGTGGTTGGCCCTGG 80
Bos     GTGGACTTTGACAATCCTGACTACGAGAGGTTTCCCAATTTCCAGAACGTGGTTGGTTACGAAACAGTGGTTGGCCCCGG 80
Canis   GTGGACTTTGACAATCCTGACTACGAGAGGTTTCCCAATTTCCAGAACGTGGTTGGTTATGAAACAGTGGTTGGCCCTGG 80

Homo    TGATGTTCTTTACATCCCAATGTACTG 107
Pan     TGATGTTCTTTACATCCCAATGTACTG 107
Macaca  TGATGTTCTTTACATCCCAATGTACTG 107
Bos     TGATGTTCTTTACATCCCAATGTACTG 107
Canis   TGATGTTCTTTACATCCCAATGTACTG 107
```

ENSG00000166135 exon 6

```
Homo    GTGGCATCACATAGAGTCATTACTAAATGGGGGGATTACCATCACTGTGAACTTCTGGTATAAG 64
Pan     GTGGCATCACATAGAGTCATTACTAAATGGGGGGATTACCATCACTGTGAACTTCTGGTATAAG 64
Macaca  GTGGCATCACATAGAGTCATTACTAAATGGGGGGATTACCATCACTGTGAACTTCTGGTATAAG 64
Bos     GTGGCATCACATAGAGTCATTACTAAATGGGGGGATAACCATCACTGTGAACTTCTGGTATAAG 64
Canis   GTGGCATCACATAGAGTCATTACTAAATGGGGGGATTACCATCACTGTGAACTTCTGGTATAAG 64
```

ENSG00000166135 intron 5

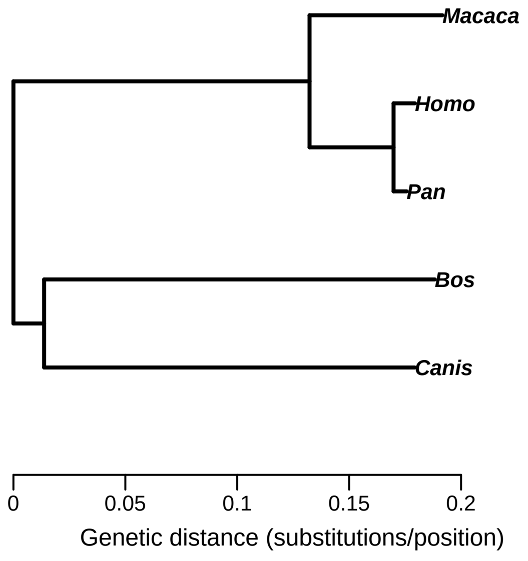

ENSG00000166135 intron 5

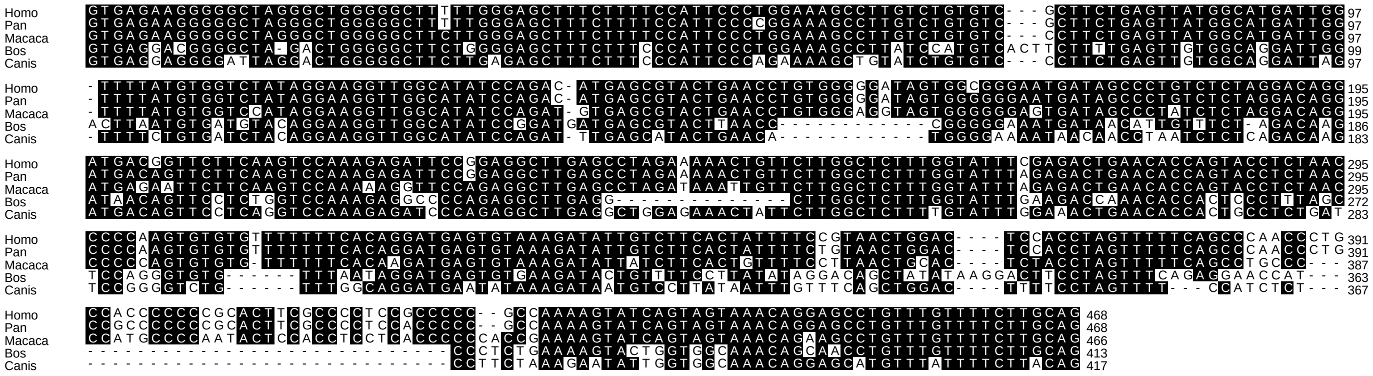

ENSG00000067836 intron 6

Description: leucine zipper domain protein (ROGDI)
Intron number: 6
Human chromosome: 16
Intron start (bp): 4788670
Human intron length : 1018
Intron alignment length: 1280
Flanking exons length (upstream/downstream): 96/99
SNP density: 0.002947
K tree score: 0.0824
Scaling factor: 0.6267
Human-chimpanzee distance: 0.019190
Total primate branch length: 0.1085

ENSG00000067836 exon 6

```
Homo    ATCCAGGATGCCAGAAACCATGTGAGCCAAGCCATTTACCTGCTTACCAGCCGGGACCAGAGCTACCAGTTCAAGACGGG 80
Pan     ATCCAGGATGCCAGAAACCATGTGAGCCAAGCCATTTACCTGCTTACCAGCCGGGACCAGAGCTACCAGTTCAAGACGGG 80
Macaca  ATCCAGGATGCCAGAAACCATGTGAGCCAAGCCATTTACCTGCTGACCAGCCGGGACGAGAGTTACCAGTTCAAGACGGG 80
Bos     ATCCAAGATGCCAGGAACCACGTGAGCCAAGCCATTTACCTCCTCGCCAACCGAGATGAGAGCTACCAGTTCAGGACGGG 80
Canis   ATCCAGGATGCCAGGAACCACGTAAGCCAGGCTATGTACCTCCTTGCCAACCGGGATGAAAGCTACCAGTTCAGGACAGG 80

Homo    CGCTGAGGTCCTCAAG 96
Pan     CGCTGAGGTCCTCAAG 96
Macaca  TGCTGAGGTCCTCAAG 96
Bos     AGCAGAGGTCCTCAAG 96
Canis   AGCAGAGGTCCTCAAG 96
```

ENSG00000067836 exon 7

```
Homo    CTGATGGACGCAGTGATGCTGCAGCTGACCAGAGCCCGAAACCGGCTCACCACCCCCGCCACCCTCACCCTCCCCGAGAT 80
Pan     CTGATGGACGCAGTGATGCTGCAGCTGACCAGAGCCCGAAACCGGCTCACCACCCCCGCCACCCTCACCCTCCCCGAGAT 80
Macaca  CTGATGGATGCAGTGATGCTGCAGCTGACCAGAGCCCGAAACCGCCTCACCACCCCCGCCACCCTCACCCTCCCTGAGAT 80
Bos     CTGATGGACGCCGTGATGCTGCAGCTGACCAGAGCCCGCAACCGGCTCACCACTCCCGCCACCCTCACTCTGCCGGAGAT 80
Canis   CTGATGGATGCTGTGATGCTGCAGCTGACCAGAGCCCGCAACCGCCTCACCACCCCAGCCACCCTTACTCTGCCTGAGAT 80

Homo    CGCCGCCAGCGGCCTCACG 99
Pan     CGCCGCCAGCGGCCTCACG 99
Macaca  CGCCGCCAGTGGCCTCACG 99
Bos     TGCTGCCAGCGGCCTCACG 99
Canis   CGCTGCCAGTGGCCTCACG 99
```

ENSG00000067836 intron 6

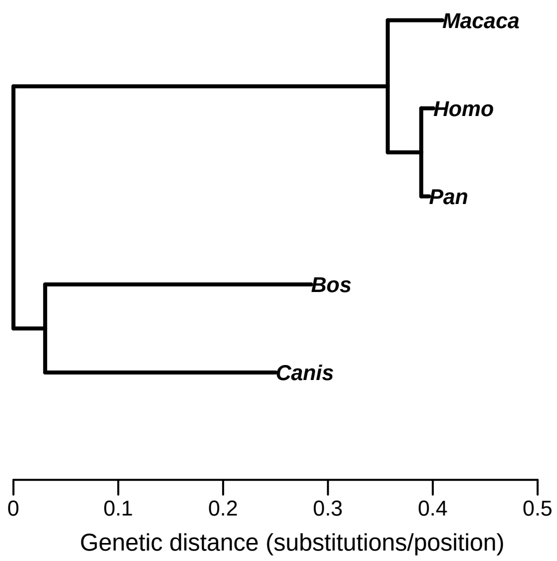

ENSG00000067836 intron 6

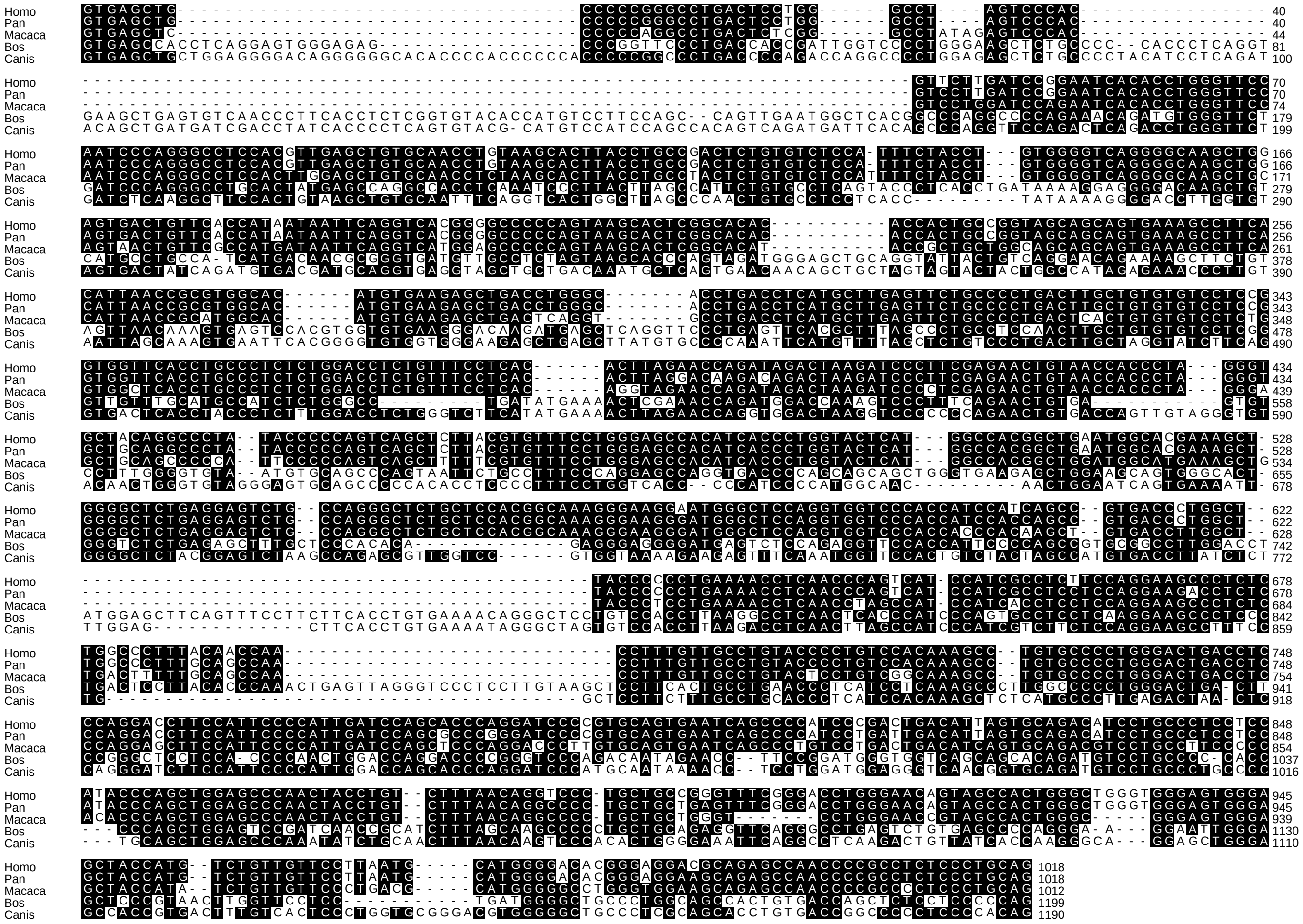

ENSG00000152670 intron 8

Description: Probable ATP-dependent RNA helicase DDX4 (DDX4)
Intron number: 8
Human chromosome: 05
Intron start (bp): 55112732
Human intron length : 787
Intron alignment length: 1556
Flanking exons length (upstream/downstream): 81/48
SNP density: 0.005083
K tree score: 0.0959
Scaling factor: 0.6377
Human-chimpanzee distance: 0.014575
Total primate branch length: 0.108

ENSG00000152670 exon 8

```
Homo    ATAATGACTTAGACCCAGACGAATGTATGCAGCGCACTGGTGGCCTTTTTGGTTCTAGAAGACCAGTATTAAGTGGCACA 80
Pan     ATAATGACTTAGACCCAGACGAATGTATGCAGCGCACTGGTGGCCTTTTTGGTTCTAGAAGACCAGTATTAAGTGGCACA 80
Macaca  ATAATGACTTAGACCCAGACGAATGTATGCAGCGCACTGGTGGCCTTTTTGGTTCTAGGAGACCAGCATTAAGTGGCACA 80
Bos     ATAGTGATTATGAACAAGATGAGGGGACCCAGCGCTCTGGTGGCATTTTTGGTTCTAGAAGATCAGCATTAAGTGGTGCA 80
Canis   ATCGTGAATATGAACAAGATGAGGGGACACAGCGCACTGGTGGCCCTTTTGGTTCTAGAAGACCAGTATTAAGTGGTGCA 80

Homo    G 81
Pan     G 81
Macaca  G 81
Bos     G 81
Canis   G 81
```

ENSG00000152670 exon 9

```
Homo    GTAATGGTGATACTTCTCAAAGCAGAAGTGGCAGTGGAAGTGAACGAG 48
Pan     GTAATGGTGATACTTCTCAAAGCAGAAGTGGCAGTGGAAGTGAACGAG 48
Macaca  GTAATGGTGATACTTCTCAAAGCAGAAGTGGCAGTGGAAGTGAACGAG 48
Bos     GTAATGGTGACACTTTCCAAAGCAGAAGTGGTGGTGGAAGTGGACGAG 48
Canis   GTAATGGTGACACTTACCAAAGCAGAAGTGGCAGTGGAAGTAGACGAG 48
```

ENSG00000152670 intron 8

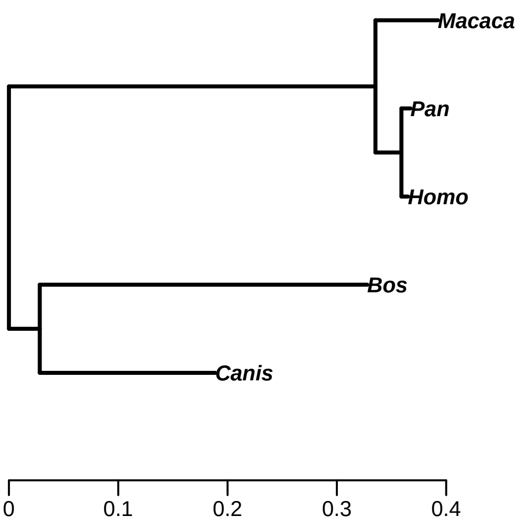

Genetic distance (substitutions/position)

ENSG00000152670 intron 8

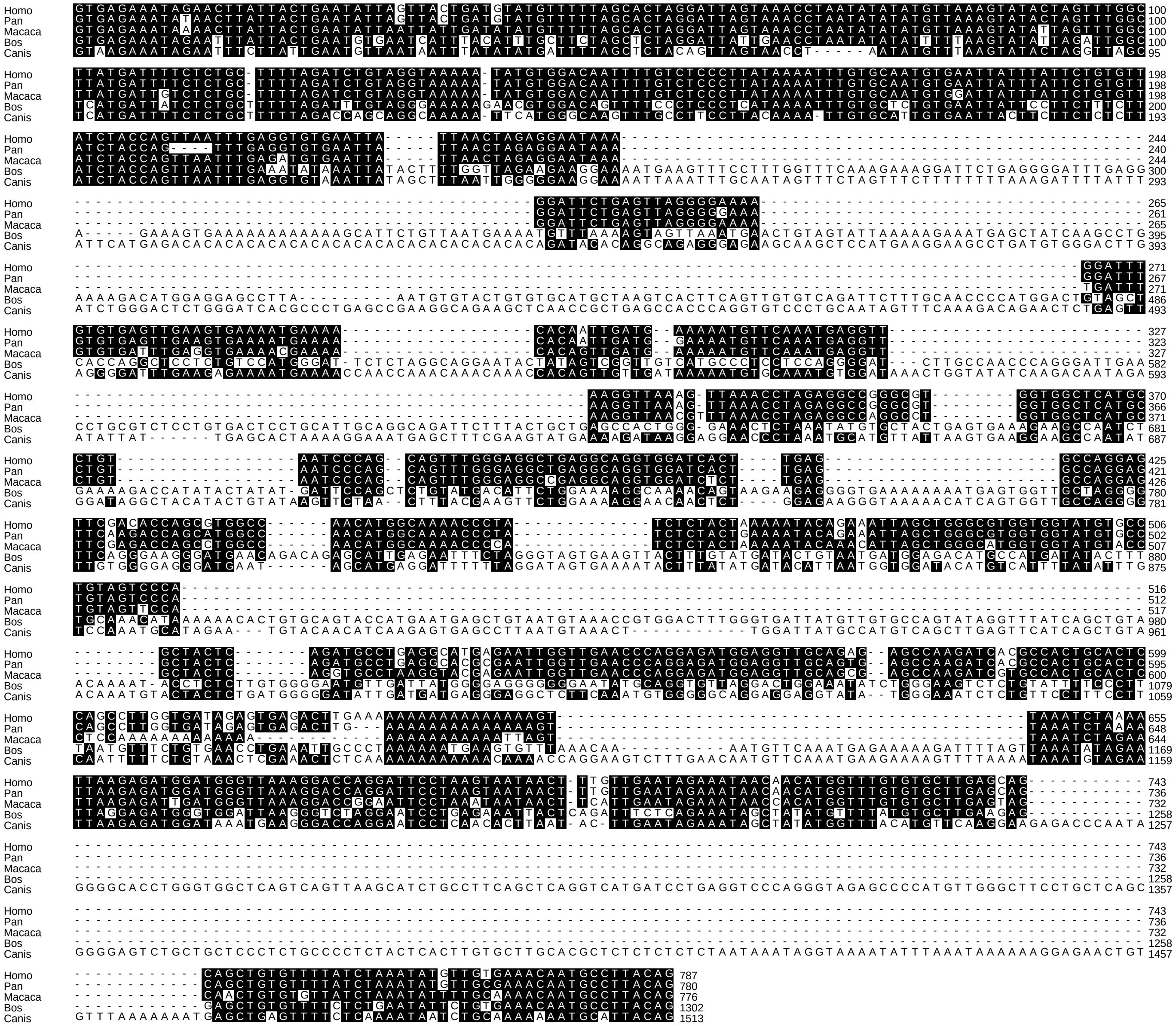

ENSG00000187783 intron 4

Description: Transmembrane protein 72 (TMEM72)
Intron number: 4
Human chromosome: 10
Intron start (bp): 44749231
Human intron length : 879
Intron alignment length: 1494
Flanking exons length (upstream/downstream): 140/476
SNP density: 0.001138
K tree score: 0.0771
Scaling factor: 0.6146
Human-chimpanzee distance: 0.018030
Total primate branch length: 0.1074

ENSG00000187783 exon 4

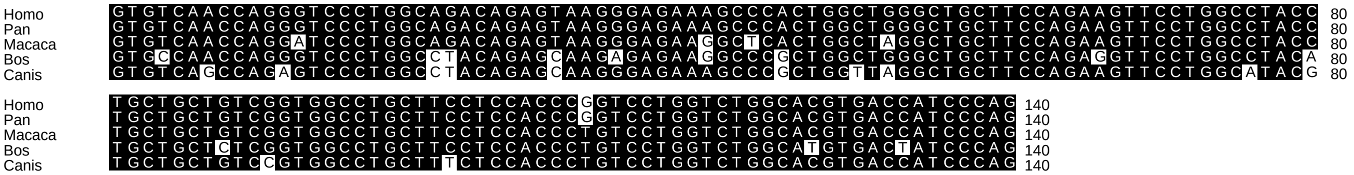

ENSG00000187783 exon 5

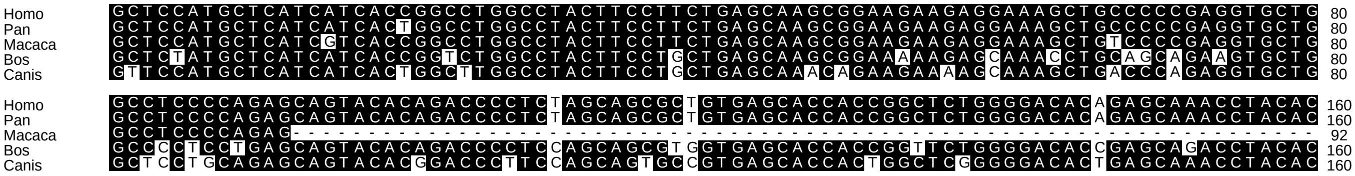

ENSG00000187783 intron 4

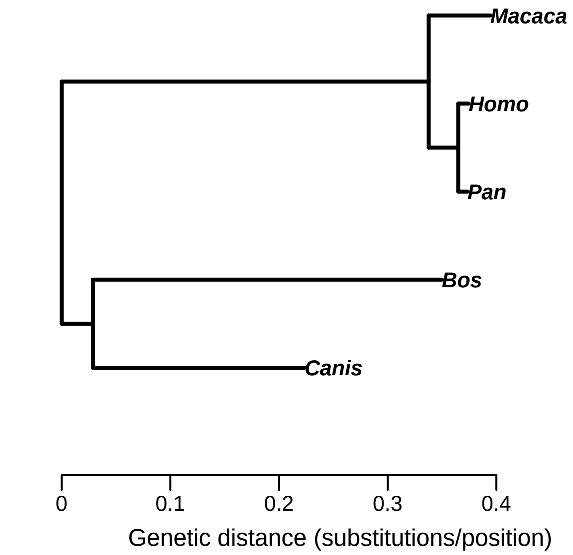

ENSG00000187783 intron 4

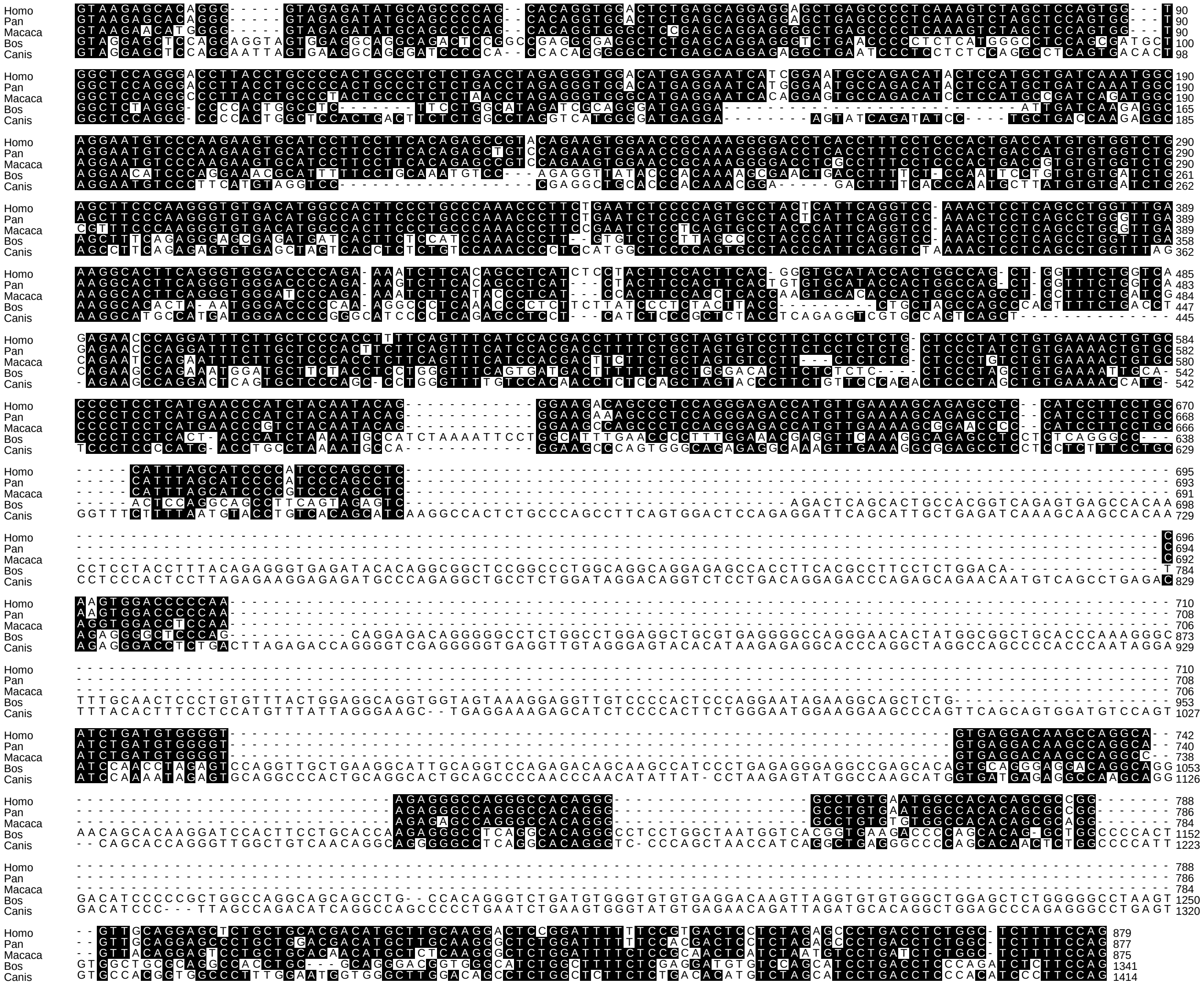

ENSG00000112118 intron 2

Description: DNA replication licensing factor MCM3 (MCM3)
Intron number: 2
Human chromosome: 06
Intron start (bp): 52255619
Human intron length : 432
Intron alignment length: 466
Flanking exons length (upstream/downstream): 113/209
SNP density: 0.016204
K tree score: 0.078
Scaling factor: 0.9123
Human-chimpanzee distance: 0.004727
Total primate branch length: 0.1052

ENSG00000112118 exon 2

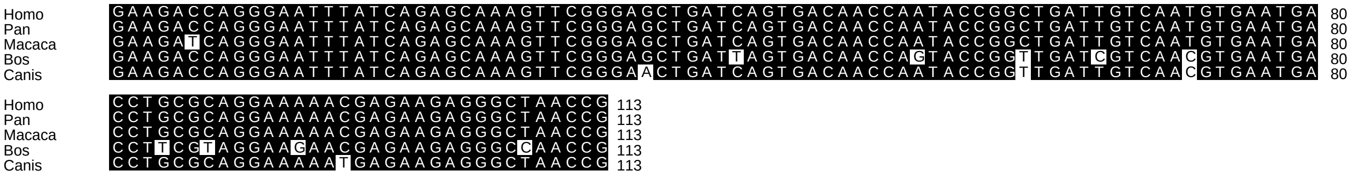

ENSG00000112118 exon 3

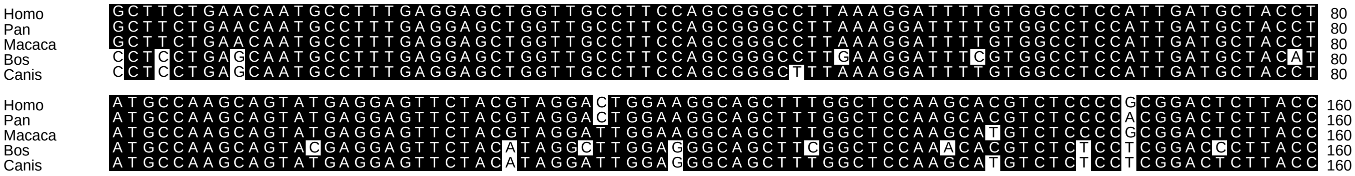

ENSG00000112118 intron 2

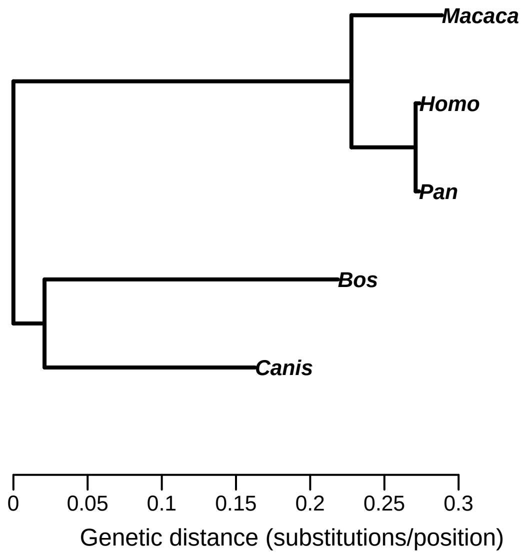

ENSG00000112118 intron 2

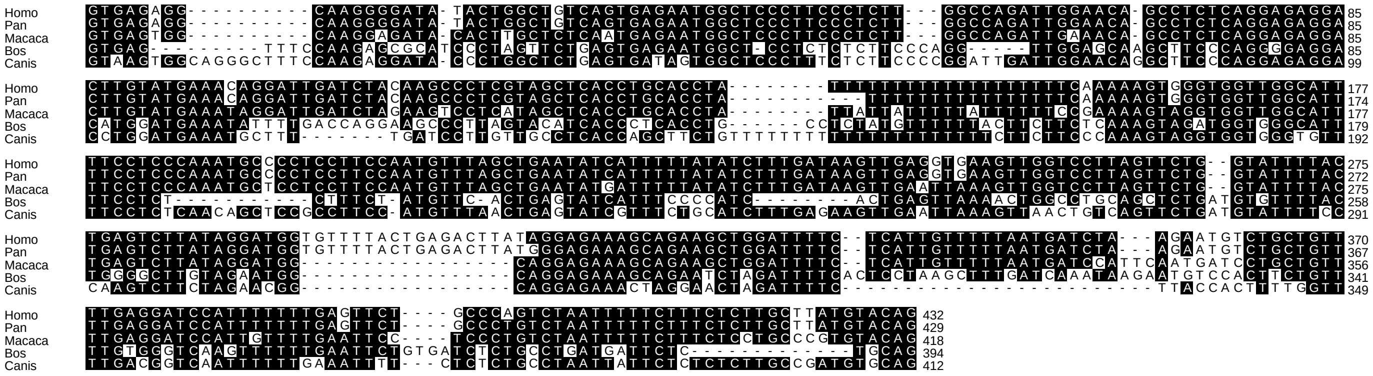

ENSG00000158941 intron 9

Description: p30 DBC protein (KIAA1967)
Intron number: 9
Human chromosome: 08
Intron start (bp): 22527962
Human intron length : 334
Intron alignment length: 361
Flanking exons length (upstream/downstream): 114/164
SNP density: 0.008982
K tree score: 0.0374
Scaling factor: 0.7951
Human-chimpanzee distance: 0.021355
Total primate branch length: 0.1033

ENSG00000158941 exon 9

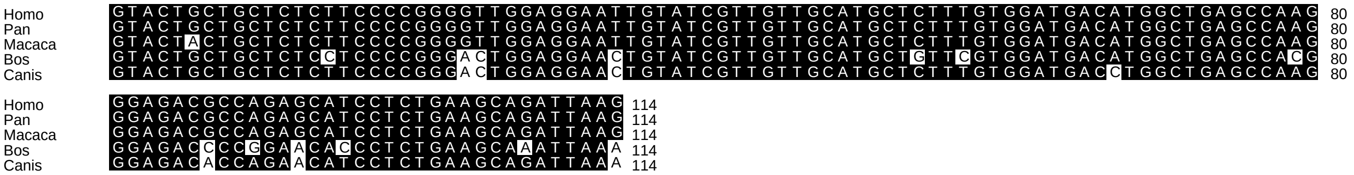

ENSG00000158941 exon 10

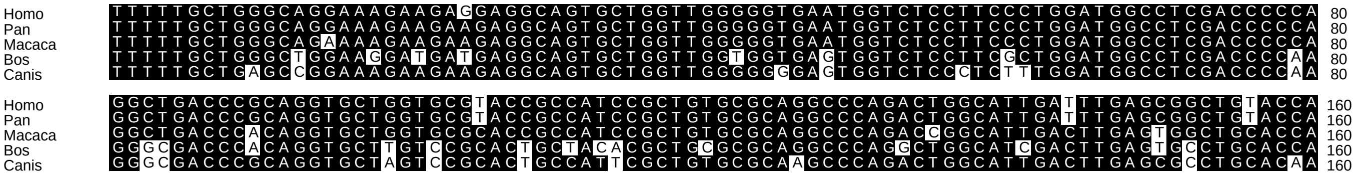

ENSG00000158941 intron 9

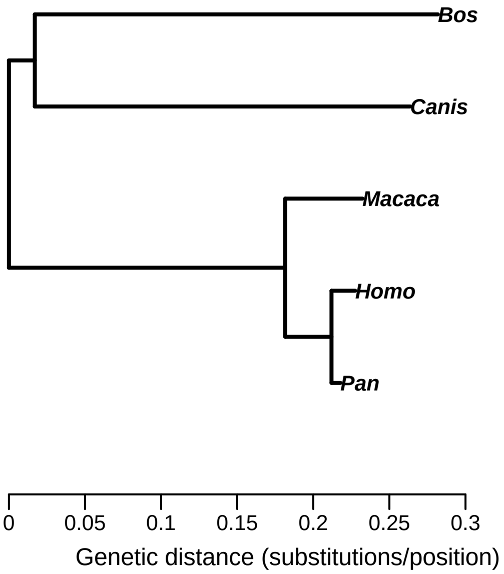

ENSG00000158941 intron 9

```
Homo    GTAAGAGCTGGAGAGCAGGAGG--AGATGCATTCACGGGTAATGTGCACAACCTGTCATGTTTTGAGT-GTTCGGCTCCTGTTCCTGAATGCATA-AAAC 96
Pan     GTAAGAGCTGGAGAGCAGGAGG--AGATGCATTCACTGGTAATGTGCACAACCTGTCATGTTTTGAGT-GCTCGGCTCCTGTTCCTGAATGCATA-AAAC 96
Macaca  GTAAGAGCTGGAGGGCAGGAGG--AGATGCATTCACTGGTAATGTCCAGAACCTGTCATGTTTCGAGT-GCTCGGCTCCTGTTCCTGAATGCACAGAAAC 97
Bos     GTAAGAGCTGGAGCCCAGGAGGCAAGACACACTTACTGACGATGGTCAGTCCCTGTCA-----------ACTGAGATGCTGCTCATGAGCTCAAAGAAAC 89
Canis   GTAAGAGCTGGAGTTCATGAGGCAGGAGGTAGTCCTGGATGACGGCCAGGCCCTGTCACGTTTTCAGCAGCCTGGCTCCTGTTCACGAGCTCGTAGAAAC 100

Homo    GAGGCATCTCAGAGCCGAAC-ACCCCTCATTTCACCCAGGAGGAAGGTGCAGCTCAGAGCTGTTACGACTAGTCAGAGAGAGCGAGGTGGCTGGTTCATG 195
Pan     GAGGCATCTCAGAGCCGAAC-ACCCCTCATTTCACCCAGGAGGCAGGTGCAGCTCAGAGCTGTTACGACTAGTCAGAGAGAGCTAGGTGGCTGGTTCATG 195
Macaca  GAAGCATCTCGGAGCTGAAT-GCCCCTCATTTTACCCAGGAGGAAGGTACAGCTCAGAGCTGTTCTGACTAGTCAGAGAGAGCTAGGTGGCTGGCTCATG 196
Bos     AGAGT---------TGAGAT-ACTAGTCGTTCTGCCC-GGAGCAGGGTGTGGCCCAGAGATGGTCTTACT--------AGATTTACGTGG--GGTTCAAG 168
Canis   AAGGAGCCCAGAACTGAAATGACCACTCATTTTACCA-GAAAGACAGCATGGCTTAG-GATGTTCTTGTC----AGAAAAAGTTACATGGCTAGTTCATG 194

Homo    TTTGCAGATAGAGA----------CCT----------CACTGTGTGGTGGA--CTTCCCCACTTCCGGCTGCCTTCATCCTGATGGGTGGCAGCCTTGCC 273
Pan     TTTGCAGATAGAGA----------CCT----------CACTGTGTGGTGGA--CTTCCCCACTTCCTGCTGCCTTCATCCTGATGGGTGGCAGCCTTGCC 273
Macaca  TTAGCAGATAGAGA----------CCT----------CACTGTGTGATGGA--CTTCCCCACTTCCTGCTCCCTTCATCCTGATGTGCGGCAGCCTTGCC 274
Bos     TTCATGGGTAGAGA----------GCTGCCCACCACGTGCAGTGTGGTGGA--CTCTGTTCCCCAGTGGTGC--------------------AGGTTGTT 236
Canis   TTTGTAGGTGGAGAGATCCATCCTCCCCTGCTTTCCACCCCGTGTGGCAGACTCTTCACCATATCCTGCTGCTTTCATTCCCCAGGGGTGGTAAGCTGCC 294

Homo    CTGCAGTGGGAGACCCAGGTAATGTAGTTTTTTGTTTTGTATCCCTGATCTCTTCTTGCAG 334
Pan     CTGCAGTGGGAGACCCAGGTAATGTAGTTTTTTGTTTTGTATCCCTGACCTCTTTTTGCAG 334
Macaca  CTGCAGTGGGAGACCCAGGTAACGGAGTTTTTTGTTTCATATCCCTGATGTCTTTTTGCAG 335
Bos     TCCCAGTAGGAAGCCCAGATGAT-TGGTTTTTCGTGTCTTATCCCTGGTCTCTCTTTGCAG 296
Canis   TCCTGGTGAGAGATCCAGATAAT-TGTTTTTTTGTTTTTT-TATCTCATTTATTTTTGCAG 353
```

ENSG00000067836 intron 7

Description: leucine zipper domain protein (ROGDI)
Intron number: 7
Human chromosome: 16
Intron start (bp): 4788187
Human intron length : 384
Intron alignment length: 423
Flanking exons length (upstream/downstream): 99/114
SNP density: 0.005208
K tree score: 0.0693
Scaling factor: 0.5487
Human-chimpanzee distance: 0.015805
Total primate branch length: 0.1033

ENSG00000067836 exon 7

```
Homo    CTGATGGACGCAGTGATGCTGCAGCTGACCAGAGCCCGAAACCGGCTCACCACCCCCGCCACCCTCACCCTCCCCGAGAT 80
Pan     CTGATGGACGCAGTGATGCTGCAGCTGACCAGAGCCCGAAACCGGCTCACCACCCCCGCCACCCTCACCCTCCCCGAGAT 80
Macaca  CTGATGGATGCAGTGATGCTGCAGCTGACCAGAGCCCGAAACCGCCTCACCACCCCCGCCACCCTCACCCTCCCTGAGAT 80
Bos     CTGATGGACGCCGTGATGCTGCAGCTGACCAGAGCCCGCAACCGGCTCACCACTCCCGCCACCCTCACTCTGCCGGAGAT 80
Canis   CTGATGGATGCTGTGATGCTGCAGCTGACCAGAGCCCGCAACCGCCTCACCACCCCAGCCACCCTTACTCTGCCTGAGAT 80

Homo    CGCCGCCAGCGGCCTCACG 99
Pan     CGCCGCCAGCGGCCTCACG 99
Macaca  CGCCGCCAGTGGCCTCACG 99
Bos     TGCTGCCAGCGGCCTCACG 99
Canis   CGCTGCCAGTGGCCTCACG 99
```

ENSG00000067836 exon 8

```
Homo    CGGATGTTCGCCCCTGCCCTGCCGTCCGACCTGCTGGTCAACGTCTACATCAACCTCAACAAGCTCTGCCTCACGGTGTA 80
Pan     CGGATGTTCGCCCCTGCCCTGCCATCCGACCTGCTGGTCAACGTCTACATCAACCTCAACAAGCTCTGCCTCACGGTGTA 80
Macaca  CGGATGTTCGCCCCCGCCCTGCCCTCCGACCTGCTGGTCAATGTCTACATCAACCTCAATAAGCTCTGCCTCACGGTGTA 80
Bos     CGGATGTTCGCCCCCACCCTGCCTTCTGACCTCCTGGTCAACGTCTACATCAACCTCAACAAGCTCTGCCTCACCGTGTA 80
Canis   CGGATGTTTGCCCCTGCCCTGCCCTCCGACCTGCTTGTCAACGTCTACATCAACCTCAACAAGCTCTGCCTCACCGTGTA 80

Homo    CCAGCTGCATGCCCTGCAGCCCAACTCCACCAAG 114
Pan     CCAGCTGCATGCCCTGCAGCCCAACTCCACCAAG 114
Macaca  CCAGCTGCATGCCCTGCAGCCCAACTCCACCAAG 114
Bos     CCAGCTGCACACCCTGCAGCCCAATTCCACCAAG 114
Canis   CCAGCTGCACGCCCTGCAGCCCAATTCCACCAAG 114
```

ENSG00000067836 intron 7

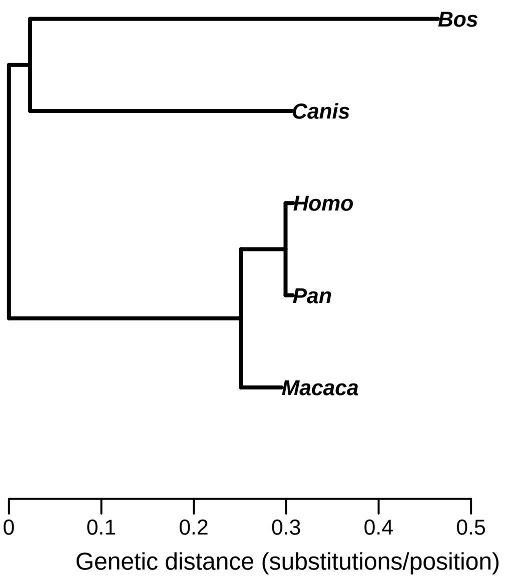

ENSG00000067836 intron 7

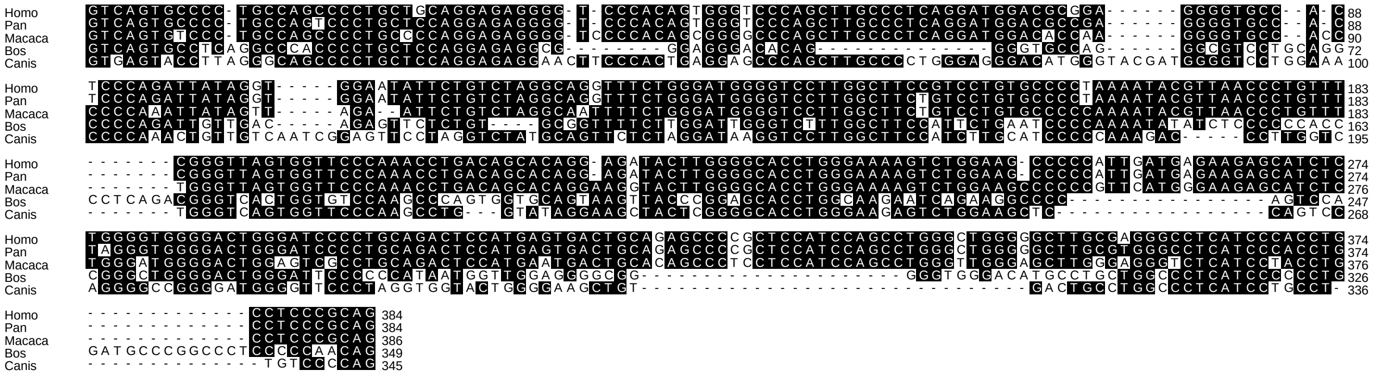

ENSG00000125869 intron 1

Description: Uncharacterized protein C20orf103 precursor (C20orf103)
Intron number: 1
Human chromosome: 20
Intron start (bp): 9443564
Human intron length : 536
Intron alignment length: 586
Flanking exons length (upstream/downstream): 64/173
SNP density: 0.007463
K tree score: 0.0706
Scaling factor: 1.0246
Human-chimpanzee distance: 0.019327
Total primate branch length: 0.1033

ENSG00000125869 exon 1

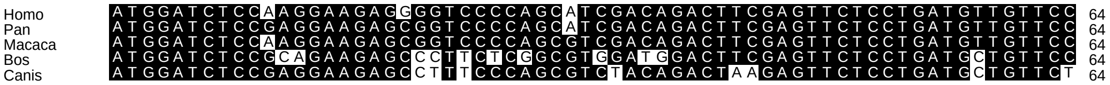

ENSG00000125869 exon 2

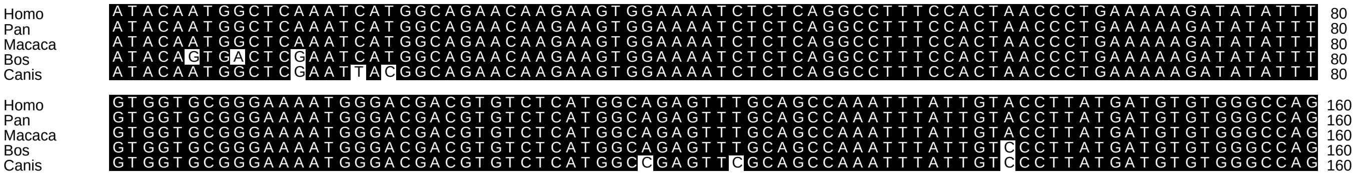

ENSG00000125869 intron 1

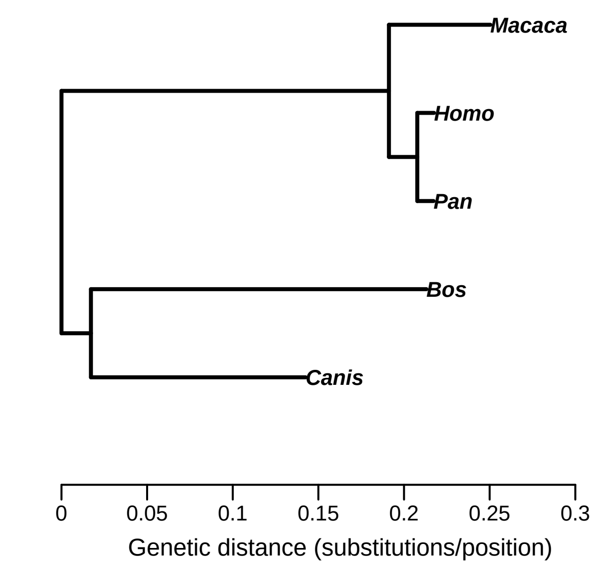

ENSG00000125869 intron 1

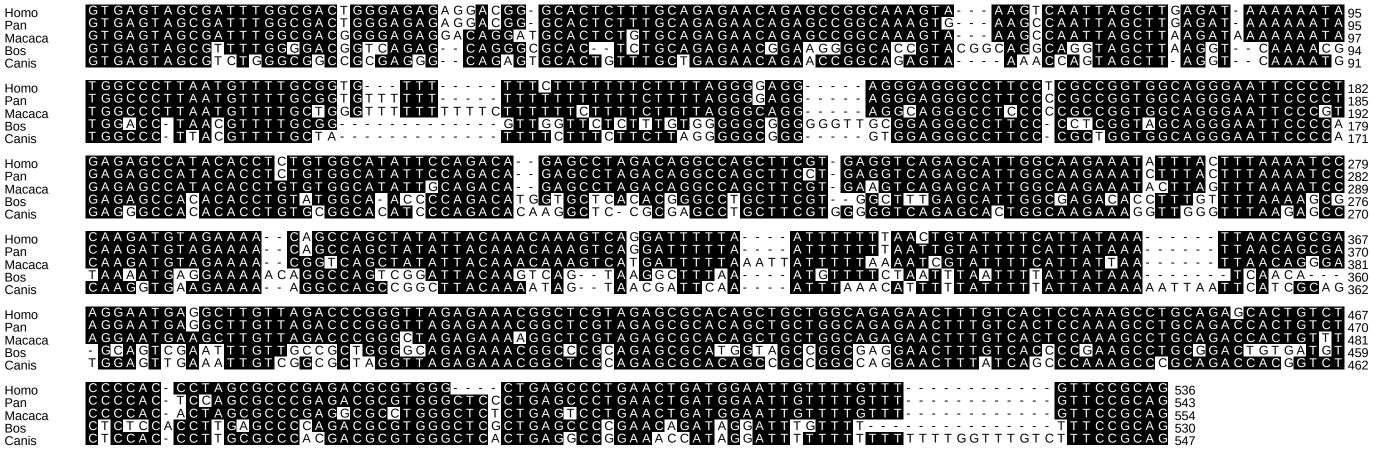

ENSG00000157368 intron 3

Description: Interleukin-34 Precursor (IL34)
Intron number: 3
Human chromosome: 16
Intron start (bp): 69248084
Human intron length : 280
Intron alignment length: 298
Flanking exons length (upstream/downstream): 78/162
SNP density: 0.000000
K tree score: 0.0654
Scaling factor: 0.5273
Human-chimpanzee distance: 0.007226
Total primate branch length: 0.1031

ENSG00000157368 exon 3

```
Homo    AAACACTACTTCCCCATCAACTACAAGATCAGTGTGCCTTACGAGGGGGTGTTCAGAATCGCCAACGTCACCAGGCTG 78
Pan     AAACACTACTTCCCCATCAACTACAAGATCAGTGTGCCTTACGAGGGGGTGTTCAGAATCGCCAACGTCACCAGGCTG 78
Macaca  AAACACTACTTCCCCATCAACTACAAGATCAGTGTGCCTTACGAGGGGGTGTTCAGAATCGCCAATGTCACCAGGCTG 78
Bos     AAACACTATTTCCCCATCAACTACAGGGTCAGTGTGCCTTATGAGGGGGTGCTCCGAACAGCCAACGTCACCAGGCTG 78
Canis   AAACACTACTTCCCCATCAACTACAGAGTCAGCGTGCCTTATGAGGGGGTGCTCAGAATGGCCAACATCACCAGGCTG 78
```

ENSG00000157368 exon 4

```
Homo    CAGAGGGCCCAGGTGAGCGAGCGGGAGCTGCGGTATCTGTGGGTCTTGGTGAGCCTCAGTGCCACTGAGTCGGTGCAGGA 80
Pan     CAGAGGGCCCAGGTGAGCGAGCGGGAGCTGCGGTATCTGTGGGTCTTGGTGAGTCTCAGTGCCACTGAGTCGGTGCAGGA 80
Macaca  CAGAGGGCCCGGGTGAGCGAGCGGGAGCTGCGGTATCTGTGGGTCTTGGTGAGTCTCAGTGCCACTGAGTCGGTGCAGGA 80
Bos     CAGCGGGCCCAGGTAAGCCAGCAGGAGCTGCGGTATCTGTGGGTCCTGGTGAGTCTCAGTGCTACTGAGTGGGTGCAGGA 80
Canis   CAGAGGGCCCGAGTCAGCCAGCAGGAGCTGCGCTATCTGTGGGTCTGGGTGAGCCTCAGTGCTACTGAGTCGGTGCAGGA 80

Homo    CGTGCTGCTCGAGGGCCACCCATCCTGGAAGTACCTGCAGGAGGTGGAGACGCTGCTGCTGAATGTCCAGCAGGGCCTCA 160
Pan     CGTGCTGCTCGAGGGCCACCCATCCTGGAAGTACCTGCAGGAGGTGCAGATGCTGCTGCTGAATGTCCAGCAGGGCCTCA 160
Macaca  CGTGCTGCTCGACGGCCACCCGTCCTGGAAGTACCTGCAGGAGGTGCAGACGCTGCTGCTGAATGTCCAGAAGGGCCTCA 160
Bos     GGTGCTGCTTGAGGGCCACCCGTCCTGGAAGTACCTGGAGGAGGTGCACACACTACTGCTGGACGTCAAGCAGGGCCTGG 160
Canis   AGTACTGCTTGAGGGCCACCCATCCTGGAAGTACCTGGAGGACGTACATACGCTGCTGCTGGATGTGCAGCAGAGCCTCA 160
```

ENSG00000157368 intron 3

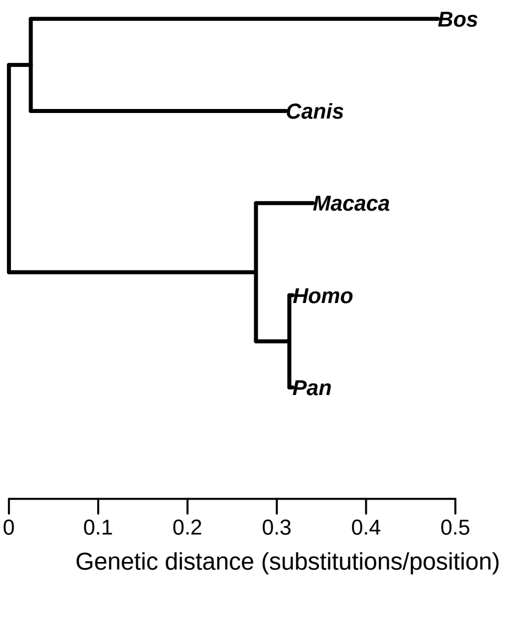

ENSG00000157368 intron 3

```
Homo    GTGAGAATCCCTTCCT---GGGCTGGGGGGACCCTGCCTCCTGCGACATCCGGTGGGCCCCAGCCTCAGAGGCGCGCTGGGACTGGCAGGTGGTGCTGCT 97
Pan     GTGAGAATCCCTTCCT---GGGCTGGGGGGACCCTGCCTCCTGCGACATCCGGTGGGCCCCAGCCTCAGAGGCGCGCTGGGACTGGCAGGTGGTGCTGCT 97
Macaca  GTAAGAATCCCTTCCT---GGGCTGGGGGGACCCTGCCTCCTGCAACGTCCGGTGGG-CCCAGCCTCAGAGGCGCGCTGGCATTGGCAGGTGGTGCTGCT 96
Bos     GTGAGAATCCCCTCTTCTGGAGTGGGGAGCCCCCTGCCTCCCGGGACACCCTGGGCG-CCCTGCCTGGGAAGCA-------------------------- 73
Canis   GTGAGAATCCTGTCCT---GGGCT-AGGGACCTCTGCCTCCTGGGACACCCCACGGA-CCCAGCCC--AAGGCACACTGGCCTCGCCA----GTCCTGTT 89

Homo    GGCCTGGGGCCTCTCCTCAGCCCCGAGAGCAGGCTGGCCCTTGGCCCAGGCACATGTTTGTCCACTGTCCACAGCGGACGGCCCGCGTCTGCTCCCTATG 197
Pan     GGCCTGGGGCCTCTCCTCAGCCCCGAGAGCAGGCTGGCCCTTGGCCCAGGCACATGTTTGTCCACTGTCCACAGCGGACGGCCCACGTCTGCTCCCTATG 197
Macaca  GGCCTGGGGCCTCTCCTCAGCCCCAAGAGCAGGCTGGCCCTTGGCCCAGGCACACGTTTGTTCACTGTCCGCAGTGCACAGCCCGTGTCTGCTCCCTGTG 196
Bos     ---CCAGGAGGCTTTCCTGGCCCCTCAGGCAAGCCCACCCTTGGCTCAGGCCCAAGCTTGCCCCCCACCCCTGG-GGGCTGCCGTGGTCTCCCCCCTGTG 169
Canis   G--CCAGGGGCCTTTCCCAGCTCTGCAAGCAAGCCTGCCCTTGGTGTCGGCCCAGGGGTGCTCCATGCCCACAGTGGATGCCTGTGGTCTGCTCTTGTTG 187

Homo    CAGGGGCAAGTCCCTGGTGAGGGAGTGG---------T------CAGAGCCCAGAGGCCCATGTCTCCCGAGTTGCAGTGACTTCCCTGTTTCCGCAG 280
Pan     CAGGGGCAAGTCCCTGGTGAGGGGGTGG---------T------CAGAGCCCAGAGGCCCATGTCTCCCGAGTTGCAGTGACTTCCCTGTTTCCGCAG 280
Macaca  CAGGGGCAAGTTCCTGGTGAGGGGCTGGTCAGATTGCT------CAGAACCCAGAGGCCTGTGTCTCTGGGGTTGCAGTGACTTCTCTGTCTCCGCAG 288
Bos     CAGGTCAAATCT----GGGGGATGTTGGATCTGGGGGCGGGGGTAAGGGCCCGGAGGCC--TCTTTCCCGGGCTTCAGTAGTTTCTCTGTTCTTGCAG 261
Canis   TGGGGACAAGTT----GGGTGGGGGTGGTCAGGGTGGTGGCAGGAAGGGCCCAGAGCCCCGTGTCTGTGGGCTTTAATGATTTCTCCTGTCCCCACAG 281
```

ENSG00000111652 intron 4

Description: COP9 signalosome complex subunit 7a (COPS7A)
Intron number: 4
Human chromosome: 12
Intron start (bp): 6708877
Human intron length : 952
Intron alignment length: 982
Flanking exons length (upstream/downstream): 203/106
SNP density: 0.005252
K tree score: 0.0379
Scaling factor: 0.4613
Human-chimpanzee distance: 0.017408
Total primate branch length: 0.1026

ENSG00000111652 exon 4

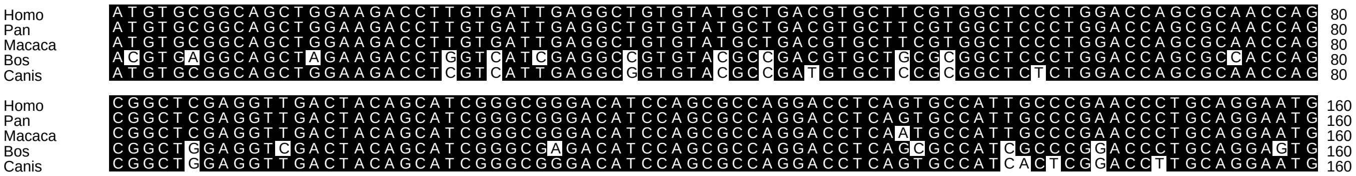

ENSG00000111652 exon 5

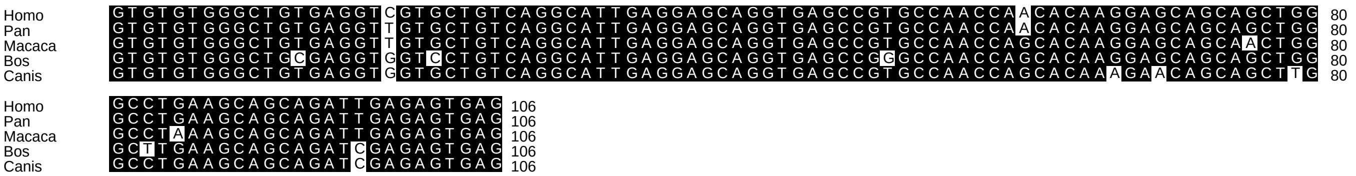

ENSG00000111652 intron 4

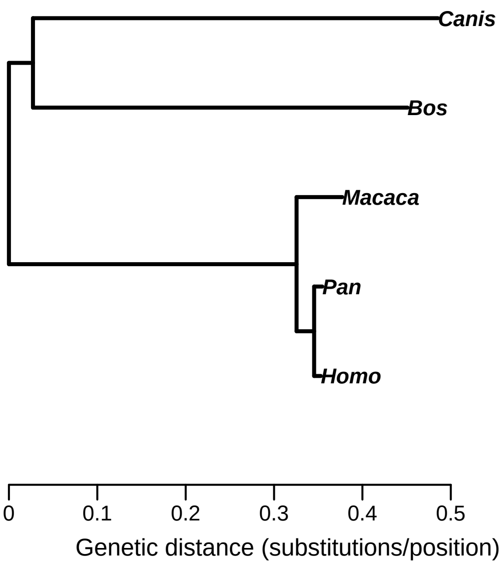

ENSG00000111652 intron 4

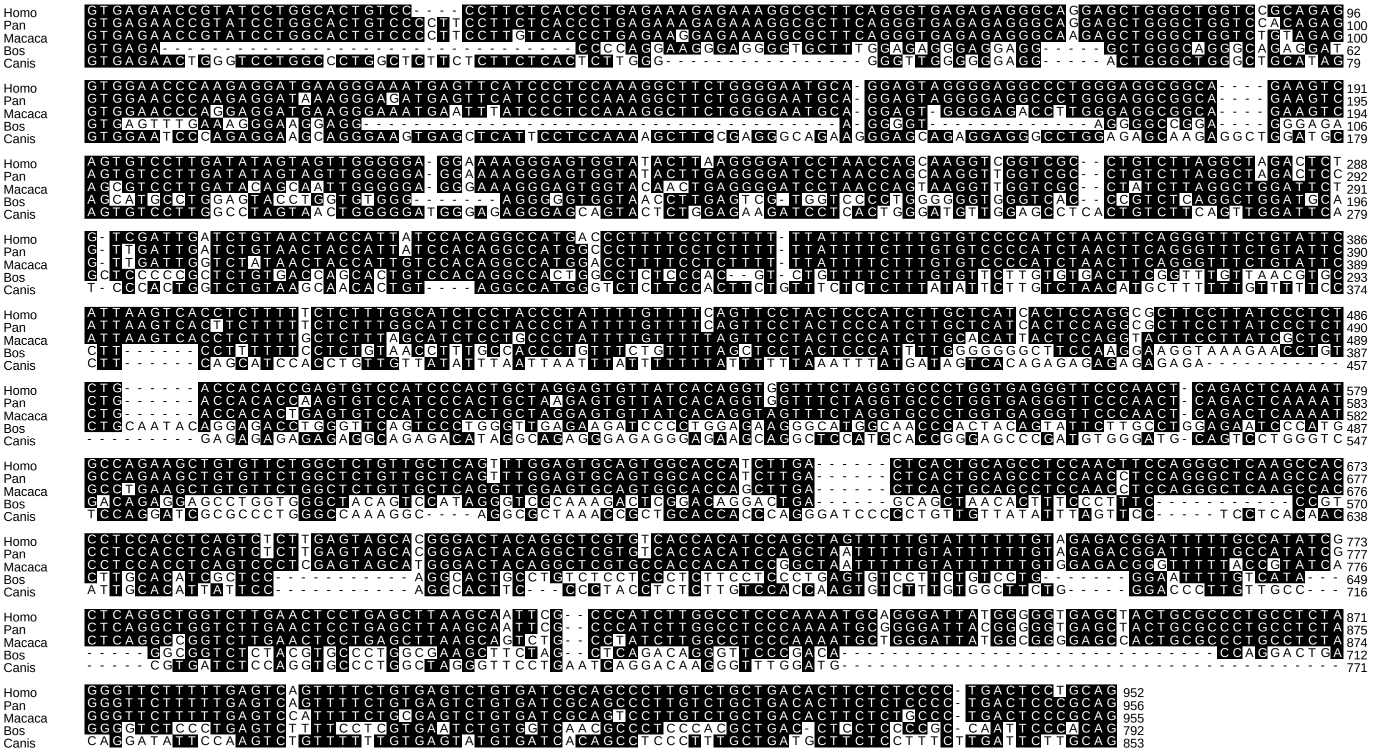

ENSG00000135902 intron 3

Description: Acetylcholine receptor protein subunit delta precursor (CHRND)
Intron number: 3
Human chromosome: 02
Intron start (bp): 233100400
Human intron length : 816
Intron alignment length: 872
Flanking exons length (upstream/downstream): 45/110
SNP density: 0.002451
K tree score: 0.0465
Scaling factor: 0.8482
Human-chimpanzee distance: 0.021208
Total primate branch length: 0.1015

ENSG00000135902 exon 3

```
Homo    AAAGAAGTTGAGGAGACCCTCACTACCAATGTGTGGATAGAGCAC 45
Pan     AAAGAAGTTGAGGAGACCCTCACTACCAATGTGTGGATAGAGCAC 45
Macaca  AAAGAAGTTGAGGAGACCCTCACTACCAATGTGTGGATAGAGCAC 45
Bos     AAAGAAGTCGAAGAGACCCTCACCACGAATGTGTGGATCGAGCAG 45
Canis   AAAGAAGTAGAAGAGACCCTGACCACTAACGTGTGGATTGAGCAC 45
```

ENSG00000135902 exon 4

```
Homo    GGCTGGACAGACAACCGGCTGAAGTGGAATGCTGAAGAATTTGGAAACATCAGTGTCCTGCGCCTCCCCCCGGACATGGT 80
Pan     GGCTGGACAGACAACCGGCTGAAGTGGAATGCTGAAGAATTTGGAAACATCAGTGTCCTGCGCCTCCCCCCGGACATGGT 80
Macaca  GGCTGGACAGACAACCGGCTGAAGTGGAATGCTGAAGAATTTGGAAACATCAGTGTCCTGCGCCTCCCCCCAGACATGGT 80
Bos     GGCTGGACAGACAGCCGGCTGCAGTGGGATGCAGAAGACTTTGGGAACATCAGCGTCCTGCGTCTGCCCGCTGACATGGT 80
Canis   GGCTGGACAGACAGCCGGCTGCAGTGGAACGCTAAAGACTTTGGAAACATCAGTGTCCTGCGCCTGCCGCCTGACATGCT 80

Homo    GTGGCTCCCAGAGATTGTGCTGGAGAACAA 110
Pan     GTGGCTCCCAGAGATTGTGCTGGAGAACAA 110
Macaca  GTGGCTCCCAGAGATTGTGCTGGAGAACAA 110
Bos     GTGGCTCCCAGAGATTGTGCTGGAGAACAA 110
Canis   GTGGCTCCCAGAGATTGTGCTAGAGAACAA 110
```

ENSG00000135902 intron 3

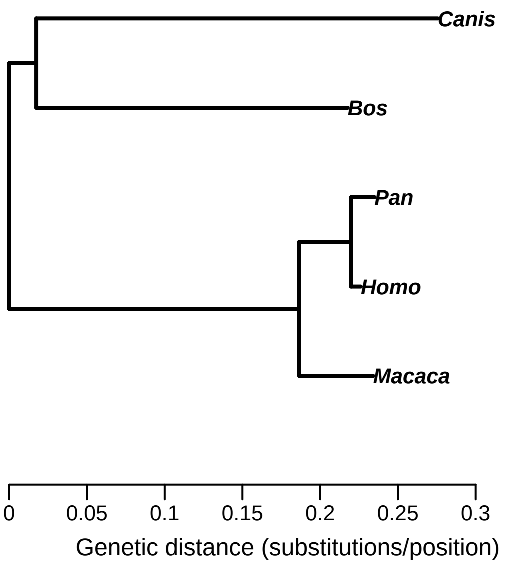

ENSG00000135902 intron 3

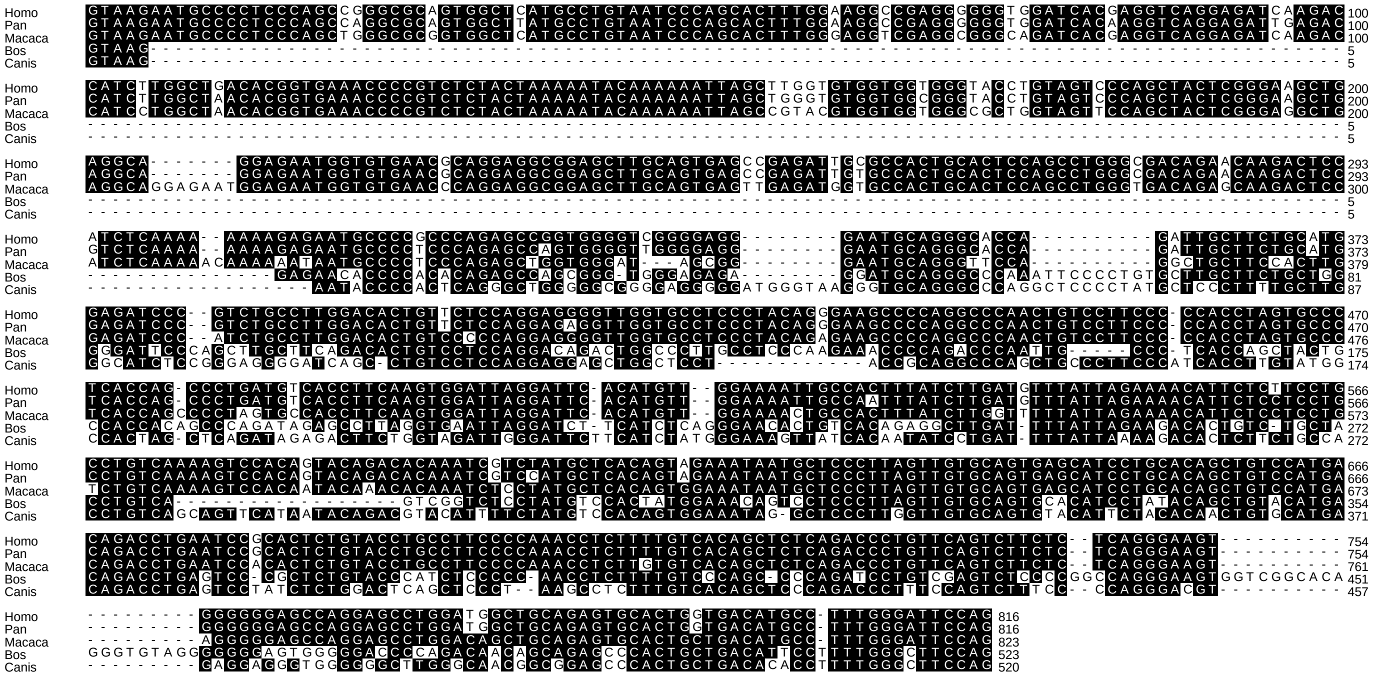

ENSG00000113492 intron 8

Description: Alanine--glyoxylate aminotransferase 2, mitochondrial precursor (AGXT2)
Intron number: 8
Human chromosome: 05
Intron start (bp): 35061718
Human intron length : 554
Intron alignment length: 589
Flanking exons length (upstream/downstream): 101/93
SNP density: 0.003610
K tree score: 0.0616
Scaling factor: 1.1311
Human-chimpanzee distance: 0.028044
Total primate branch length: 0.1012

ENSG00000113492 exon 8

```
Homo    ACTGCTGCCAAGCTAAAGATCAGTATATTGAGCAATTCAAAGATACGCTGAGCACATCTGTGGCCAAGTCAATTGCTGGA 80
Pan     ACTGCTGCCAAGCTAAAGATCAGTATATTGAGCAATTCAAAGATACGCTGAGCACATCTGTGGCCAAGTCAATTGCTGGA 80
Macaca  ACTGCTGCCAAGCTAAAGACCAGTATATTGAGCAGTTCAAAGACACGCTGAGCACATCCGTGGCCAAGTCAATTGCAGGA 80
Bos     ACTGCTGTCAAGCTAAAGACCAGTATATTGAACAATTCAAAGATACTCTGAGCACTTCTGTGGCCAAATCAATTGCTGGA 80
Canis   ACTGCTGCCAAGCAAAAGACCAGTATATTGAACAGTTCAAAGATACTCTGAACAGTTCTGTGGCCAAATCAATTGCTGGA 80

Homo    TTTTTCGCAGAACCTATTCAA 101
Pan     TTTTTCGCAGAACCTATTCAA 101
Macaca  TTTTTTGCAGAACCTATTCAA 101
Bos     TTTTTTGCAGAGCCAATTCAA 101
Canis   TTTTTTGCAGAACCAATTCAA 101
```

ENSG00000113492 exon 9

```
Homo    GGTGTGAATGGAGTTGTCCAGTACCCAAAGGGGTTTCTAAAGGAAGCCTTTGAGCTGGTGCGAGCAAGGGGAGGCGTGTG 80
Pan     GGTGTGAATGGAGTTGTCCAGTACCCAAAGGGGTTTCTAAAGGAAGCCTTTGAGCTGGTGCGAGCAAGGGGAGGCGTGTG 80
Macaca  GGTGTGAATGGAGTTGTCCAGTACCCAAAGGGGTTTCTAAAGGAAGCCTTTGAGCTGGTGCGAGCAAGGGGAGGTGTGTG 80
Bos     GGGGTGAATGGCGTTGTTCAGTACCCGAAGGGGTTTCTAAAGGAAGCCTTCGAGCTGGTGCGCGAGCGGGGAGGCGTGTG 80
Canis   GGGGTGAATGGAGTTGTTCAGTACCCAAAGGGGTTTTTAAAGGAAGCCTTTGAGCTGGTGCGAGAACGAGGAGGCCTGTG 80

Homo    CATTGCAGATGAA 93
Pan     CATTGCAGATGAA 93
Macaca  CATTGCAGATGAA 93
Bos     CATTGCAGATGAA 93
Canis   CATCGCAGATGAG 93
```

ENSG00000113492 intron 8

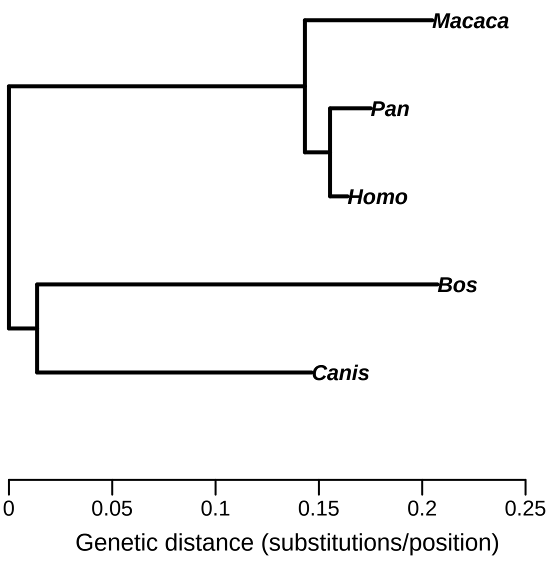

ENSG00000113492 intron 8

```
Homo    GTGGGTATATGAACCTTAGACATTAATGGAGCTGAATCTTCAATTATCAGAAAGTTTTAACTCATATATTCTAGAGAATTCCAAATGAATTAAGTGCTCA 100
Pan     GTGGGTATATGAACCTTAGACATTAATGGAGCTGAATCTTCAATTATCAGAAAGTTTTAACTCATATATTCTAGAGAATTCCAAATGAATTAAGTGCTCA 100
Macaca  GTGGGTACATGAATCTAAGACATTAATGGAGCTGAATCTTCAATTATCAGAAAGTTTTAACTCATACTTTCTAGAGAATTCCAAATGAATTAAGTGTTTA 100
Bos     GTAAGTGTGTTAACCTTAAACATTAACTGAACTCAGTTGTAAATTATCA----ATTTTAACTCATACATTCTAGTGACTTCCAAATTATTTAAGTGATCA 96
Canis   GTGGGTATACTGACCTTAGACCTTAATTGGGCTGACTTTCAAATTATCAGAGCATTTTAATTCATATATTCTAGAGAATTCCAAAATATTTAAGTGACCA 100

Homo    ---CTGTGTCCCTCTCTA-----AATTCATTCTCTTGGTCTCCTTG-AAAAAAGAAG-ACGCTGACTTTCAAATGACCTTTTAAAAACCCTCTCCTCCAT 190
Pan     ---CTATGTCCCTCTCTA-----AATTCATTCTCTTGGTCTCCTTGAAAAAAAAGAAG-ACGCTGACTTTCAAATGACTTTTTAAAAACCCTCTCCTCCAT 191
Macaca  ---CTGTGTCTGTCTCTA-----AATTCGTTCTCTTGGTCTCCTTGAAAGAAAGAAG-ACACTGATTTTCAAATGATCTTTTAAAAACCCTCTCCTCCAT 191
Bos     CTGTTATGTCCCTTTATG--------TCATTCTCCTCATCCCCTTGAAAAAAAAGAAGGCCCCTGGTTTTCAACTGACCTTTAAAAGTCCATCTGCTGTAC 188
Canis   TTGCTGCGTCCCTCTCCAAATTCAATTCATTCTCTTGGCTCCCTTAAAAAAAAAAGAGTGCCCTCAATTTTCTACTGACCTTCAAAAACTATCTCCTGTAT 200

Homo    TCTC------ACCAGAC----AGATGAAACTACCGAACTCCTAAAATTCTTAAGAAGAGGAAACCCTGGCCATTTTTAGCTGCTTAATAACATCCGCATT 280
Pan     TCTC------ACCAGAC----AGATGAAACTACTGAACTCCTAAAATTCTTAAGAAGAGGAAACCCTGGCCATTTTTAGCTGCTTAATAACATCCGCATT 281
Macaca  TTCC------ACCAGAC----AGGTGAAACTACTGAACTCCTAAAATTCTTAACAAAAGGAAACCCTGGCCGTTTTTAGCTGCTTAGTAACATCCACATT 281
Bos     TCCCAGACTGAACAGACAGATAGATGAAACTACTGAATTTGT-AAATCTGTAAAAATAGAAAACCCCAGCAGTTTTCAGTTGCTTATT---ACCCACACT 284
Canis   TCTTGGGCTGAACAGAT----AGATGAAACTATTGAATTCCTAAAATTCTGAAGAAAAGGAAACCCCAGCAATCTTTAGTTGCTTAAT---ACCCACACT 293

Homo    GTCCAAAGAACTCTTTCTTTAGTCTCACCAATTTCTTTTATTCTACTGT-TTTCTAATCTCAGAAAATAATCAATGTTTGCATCTTTTTTCCAGAAGTCA 379
Pan     GTCCAAAGAACTCTTTCTTTAGTCTAACCAATTTATTTTATTCTACTGT-TTTCTAATCTCAGAAAATAATCAATGTTTGCATCTTTTTTGCAGAAGTCA 380
Macaca  GTCCAAAGAACTCTTTCGTTAGTCTCACCAAATTCCTCTATTGTACTGT-TTTCTAATCTCGGAAAATAATCAATGTTTGCATCTTTTTC-CAGAAGTCA 379
Bos     GTCAGGGTGAGCCGTTCTGTAATCTTACTAAATCCCCTTAATCTGTTTT-TCTCTGCTCTCAGGAAAGA--CCACATTTGCAACCATTTCCCAGAAGGCA 381
Canis   ATCAGGGGGAGCCTTTCTCTAATGTTACCAAATCCCCTTAATCTACTTTGTTTCTAATCTCAGAAGAGAAAAAAGTTTTGTGTCTTTTTCCCAAAAGTCA 393

Homo    TGGTA-T-AGAGTAATAAGTCCTCTTTTCACCCACAGTGGAAACTGTTCTACAGACTGTTGTTAAAATTGCTAGTGTTACTGTCATGATAATGATGATAA 477
Pan     TGGTA-T-AGAGTAATAAGTTCTCTTTTCACCCATAGTGGAAACTGTTCTATAGACTGTTGTTAAAATTGCTAGTGTTACTATCACGATAATGATGATAA 478
Macaca  TGGTG-T-AGAGTAATAACTCCTCTTTTCACCCGTAGTGAAAACTGTTCTATAGACTGTTGTTAAAATTGCTAGTGTTACTATCATGATATTGATGATAA 477
Bos     TGGCATTAAGAGGAATGGCTCTTCTTTTCACTCACAGTTGAAACATTTCTAGAAACGGCTTCAAAAATTGCTAGTATCACTA-----ATGCTAATATTGA 476
Canis   TGGCA-CAAGAGTAATGGCCCCTCTATTCACCCATAGTTGGAACAGTTCTAAAGACCATTTAAAAAATTGCTGGTATTACTA-----ATGCTAATGATGA 487

Homo    TGATGATCTAACT------------TTTTTATATACTTTGAAAGGGCTGATGCTATTATTATAGGTCTTCTTGGTCTGGTCTTTTGCAG 554
Pan     TGATGATCTAACT------------ATTTTACATACTTTGAAAGGGCTGATGCTGTTATTATAGGTCTTCTTGGTCTGTTCTTTTGCAG 555
Macaca  TGACGATCTAACT-------------TTTTGTATACTTTGAAA-GCCTGATGCTGTTATTATAGGTCTTCTTGGTCTGGTCTTTTGCAG 552
Bos     TCATGGTCTGTCTTCAGTTGTTTCTTTTTTTAATACTTTCAAGGCCCT-GTGCTGTT-TTCTAGGTCTTCTTGGTTTGGTCTTTTGCAG 563
Canis   TAATGATCTATCTTC-----TTTCATTTTTTAATAGTTTCAAAGGGCT-ATGCTGTTATTATAGGACTCCCTGGTTTGGTCTTTTGCAG 570
```

ENSG00000067836 intron 4

Description: leucine zipper domain protein (ROGDI)
Intron number: 4
Human chromosome: 16
Intron start (bp): 4790581
Human intron length : 688
Intron alignment length: 734
Flanking exons length (upstream/downstream): 55/81
SNP density: 0.001453
K tree score: 0.0278
Scaling factor: 0.9434
Human-chimpanzee distance: 0.010407
Total primate branch length: 0.1012

ENSG00000067836 exon 4

```
Homo    CACAGACCAGGTGAAGGGTGTGCTGACTCTGCAGGGGGATGCCCTCAGCCAGGCG 55
Pan     CACAGACCAGGTGAAGGGTGTGCTGACTCTGCAGGGGGATGCCCTCAGCCAGGCG 55
Macaca  CACAGACCAGGTGAAGGGTGTGCTGACTCTGCAGGGGGATGCCCTCAGCCAGGCG 55
Bos     CACAGACCAGGTGAAGGGCGTGCTGACTCTGCAGGGAGATGCGCTGAGCCAGGCG 55
Canis   CACAGACCAGGTGAAGGGCGTGCTGACTCTGCAAGGGGACGCGCTGAGCCAGGCG 55
```

ENSG00000067836 exon 5

```
Homo    GATGTGAACCTGAAGATGCCCCGGAACAACCAGCTGCTGCACTTCGCCTTCCGGGAGGACAAGCAGTGGAAGCTGCAGCA 80
Pan     GATGTGAACCTGAAGATGCCCCGGAACAACCAGCTGCTGCACTTCGCCTTCCGGGAGGACAAGCAGTGGAAGCTGCAGCA 80
Macaca  GACGTGAACTTGAAGATGCCCCGGAACAACCAGCTGCTGCACTTCGCCTTCCGGGAGGACAAGCAGTGGAAGCTGCAGCA 80
Bos     GACGTGAACCTGAAGATGCCCAGGAACAACCAGCTGCTGCACTTTGCCTTCCGGGAGGACAAGCAATGGAAACTGCAGCA 80
Canis   GACGTGAACCTGAAGATCCCGCGGAACAACCAGTTGCTGCACTTTGCCTTCCGAGAGGACAAGCAGTGGAAGCTGCAGCA 80

Homo    G 81
Pan     G 81
Macaca  G 81
Bos     G 81
Canis   G 81
```

ENSG00000067836 intron 4

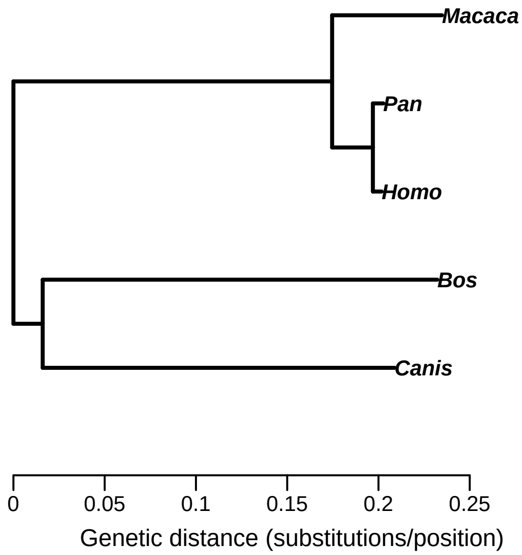

ENSG00000067836 intron 4

```
Homo    GTGAGTCCCCCGGCC--CTGTCCCCTCCCATCCTG---------CCAATGGGGAAGAGCATGGGAGC----------------TCCCACTTTCTCTCTGT 73
Pan     GTGAGTCCCCCGGCC--CTGTCCCCTCCCATCCTG---------CCAATGGGGAAGAGCATGGGAGC----------------TCCCACTTTCTCTCTGT 73
Macaca  GTGAGTCCCCCAGCC--CTGTCCCCTCCCATCCTG---------CCAGTGGGGAAGAGCATGGGGGC----------------TCCCACGATCTCTCTAT 73
Bos     GTGAGTCCCCCGGCCCCTGGCCCCTTCCCATGCCACCAGAGCAGCCAGCGGGCAAGAGTCGGGGGGTGGGGGGAGGCTTCCTGCTTTAGCTCAGCTCTGC 100
Canis   GTGAGTCCCCCAGCCCTCTTTCCCTTTCCCTGCTGCTGGAGGATCCAGGGGGGAAGAGTATGGGGGC----------------CTCCTGCTGAGCTCTGC 84

Homo    -----ACAAGCCCTGCGACTCTGGGCGGGACCCTTGATCTCTCTGGCCTCAGTTTTCTCCTTTGGAAAATGACAGCAACCCTCAC---ACCTTCTCCCGG 165
Pan     -----ACAAGCCCTGCGACTCTGGGCGGGACCCTTGATCTCTCTGGCCTCAGTTTTCTCCTTTGGAAAATGACAGCAACCCTCAC---ACCTCCTCCCGG 165
Macaca  -----ACAAGCCCTGCGACTCTGAGCGGGACCCTTGATGTCTCTGGCCTCAGTTTTCTCCTTTGGAAAATGACAGCAGCCCTCAC---ACCTCC-CCCAG 164
Bos     CACCCACTAGCTGTGTGACTCTGGGCAGGATCCTTGACCTTTCTGGGCCTCGGTTTCCTTGTTAGAAAATGACAACAAGCCCCGT--AACCTGCTTCTGA 198
Canis   CACCAGTTGGCTCTGTGACCCTGGGCAGGATCTTGACTTCTCCGGGCCTCAGTTTCCTCATTTGTAAAATAACAACCCACCCCACCAAACCTCCTCATGG 184

Homo    GATCGGTGTGCAGATTCAGTAAGAGGTGCAGGA-GAGGGGTTCTTTGCACACGGCCTGGCCTGGCGTCCGGA-CCAGTGAAACATGGGCATTGG-TGGAT 262
Pan     GATCGGTGTGCAGATTCAGTAAGAGGTGCAGGA-GAGGGGTTCTTTGCACACGGCCTGGCCTGGCGTCCGGA-CCAGTGAAACATGGGCATTGG-TGGAT 262
Macaca  GATGGGTGTGTGGATTCAGGAGGACATGCAGGAGGGGGGGTTCTTTGCACACGGCCTGGCCTGATGTCTGGA-CCAGTGAAACATGGGCATTGG-CGGAT 262
Bos     GATTG--ATGTTACTCCATTGAGAGGTGGGTGGTGGGTGGTGCTTTGCACACGGCACTACCTATAGCCTGGCCCC--------------------GGGGT 276
Canis   AATTGGCGTGTGGCTTCAATGAGAGGGACAGGAAGGAGGATGCTTTGCACACAGCCCGGCCCTGAGTCTGGGCCCAGTGAAGCGTGGGCAATGGCTGGAT 284

Homo    TAGGCTGGCCACACATCACAGTTGAATCAGCCAATGCCTGAACTCCAGTCTGCACCCGACTCTGTGCCAGGCACCAAGGCGTGGGGGGAGGCCCCCCTCC 362
Pan     TAGGCTGGCCACACATCACAATTGAATCAGCCAATGCCTGAACTCCAGTCTGCACCCGACTCTGTGCCAGGCACCAAGGCGTGGGGGGAGGCCCCCCTCC 362
Macaca  TAGGCTGGCCACACATCACAGTTGAATCAGCCAACGCCTGAGCTCCAGTCTGCACCCAACCCTGTGCCAGGCACCAAGGCGTGGGGGGCGGCCCCCCTCC 362
Bos     TAGGCTGGCCCCA---------------GACCAACATCTTAACTCTC-TCTGCACTTGATCCTGTAGCCGGTGCCAAGGCCTGGGGGAAGGTCCTCCTCC 360
Canis   TAGGCTGGCCCCAATTCAAAGTGGAATCCGCCAACATCTGAGCTCCCGT------------CCGTGCCAGGCGCCAAGATGTGGGGGAAGGTGCCTCTCT 372

Homo    T-------------GGGCTCCATCTGTTGCCCTTTGTAGACA----CTAGATGCACCCCGTTCG------CACAGAGCCTCACAGAGGCTGCCTTTCCTC 439
Pan     T-------------GGGCTCCATCTGTTGCCCTTTGTAGACA----CTAGATGCACCCCGTTCG------CACAGAGCCTCACAGAGGCTGCCTTTCCT- 438
Macaca  T-------------GGGCTCCATCTGTTGGCCCCTGTAGACG----CTAGATGCACCCGGTTCG------CACAGAGCCTCACAGAGGCTGCCTGCTGTT 439
Bos     TCCTCCTCGGGTGGGCATTACGTCCCTTGGCACCTGCAAATGTTCTCCATCTGCCCACAACCACTCACCTCCTCCCTCCCAGCAGAAGCACCTTTCCC- 459
Canis   T-------------CCAGTCCATTGGCCTGGACCCATGGGTC----CTGTCTGCCCACAGTCAC------ACCTCCCCCCGGCTGAGGCCACCTTCCCTT 449

Homo    TTCCCCTTCCCCTTTCTTCACCTCTTTCCATTTCACTTCTTTGTGGCTCTTCTTGGGCCTGGTTTCCTGTTTCCTGACCTCCCTGTTCAACC---CCCAA 536
Pan     -----CTTCCCCTTTGTTCACCTCTTTCCATTTCACTTCTTTGTGGCTCTTGTTGGGCCTGGTTTCCTGTTTCCTGACCTCCCTGTTCAACC---CCCAA 530
Macaca  TTCCCCTTCCCCTTTCTTCACCTCTTTCAATTTCACTTCTTCGTGGCTCCTCTTGGGCTTGTTTTCCTGTTTCCTGACCTCCCTGTTCACCC---CCCAA 536
Bos     ------------------------------TTTCATTTCTTTGTGGTTCCTCTTG-GCCTGAGTTCCTGTTTCCTG--TTCCCTGTTTGCCCCAGCCCAA 526
Canis   TCTCTTTTCCC-------------------TTTCATTTCTTTGTGGTTTCTCTGGAGCCTGAGTTCCTGTTTCCTGACCTCCCTGTGCACAC---CCCAA 527

Homo    ACGGCCAAGGCATTGGGAGACCCTCTGCCCTATACAGCGGCCAAGGCCTGAACCACCACACACCCCATTTCTGCCCTGG--GCTTAAGGGCTCAGAGTGG 634
Pan     ACGGCCAAGGCATTGGGAGACCCTCTGCCCTATACAGCGGCCAAGGCCTGAGCCACCACACACCCCATTTCTGCCCTGG--GCTTAAGGGCTCAGAGTGG 628
Macaca  ACGGCCAAGGCATTGGGAGTCTCTCTGCCCTACACAGCAGCCAAGGCCTGAGCCACCACACACCCCATTTCTGCCCCAC--GCTTAAGGGCTCAGAGTGG 634
Bos     ATGGCCATGTTCTTC----CCCCTCTCCCACATGGAGTGGCTCGGGCCCGAGCCAACACACACACAGTTTCTGCCCCCCTGGCTCAAGGGCTCAGAGTGG 622
Canis   ATGGCC-----------AGATCTCCTGCCCCCTGGAATGGCCAGGACCTGAGCACACACA---CCCGTCTCTGCCCCCT--GCTTCAAGGCTTGGGGTGG 611

Homo    CTG-TGGCAGGAGGTGCCCCCCCACTGCCCAGCTCACCCCACTCTTGCCTGTCAG 688
Pan     CTG-TGGCAGGAGGCGCCCCCTCACTGCCCAGCTCACCCCACTCTTGCCTGTCAG 682
Macaca  CTG-TGGCAGGAGGCGCCCCCC--GTGCCTGTTTCACCCCACTCTTGCCTGTCAG 686
Bos     CCAGCGGCAGGAGGACATCCTT--CTGCCCGGCTTAGCCCACGCTTGCCTGGCAG 675
Canis   CCGAAAGCAGGAGGGTGTCCTT--CCGCTTA------TACACTCTTGCCCGTCAG 658
```

ENSG00000140274 intron 5

Description: dual oxidase activator 2 (DUOXA2)
Intron number: 5
Human chromosome: 15
Intron start (bp): 43196796
Human intron length : 410
Intron alignment length: 517
Flanking exons length (upstream/downstream): 215/194
SNP density: 0.000000
K tree score: 0.05
Scaling factor: 0.8661
Human-chimpanzee distance: 0.017981
Total primate branch length: 0.1011

ENSG00000140274 exon 5

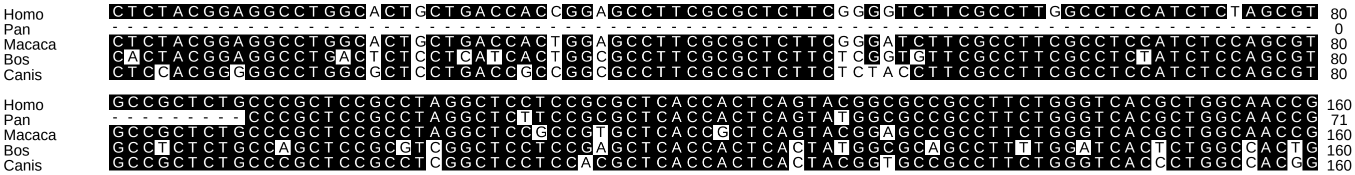

ENSG00000140274 exon 6

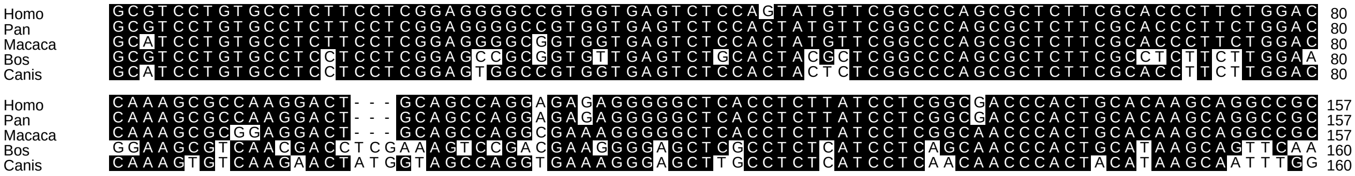

ENSG00000140274 intron 5

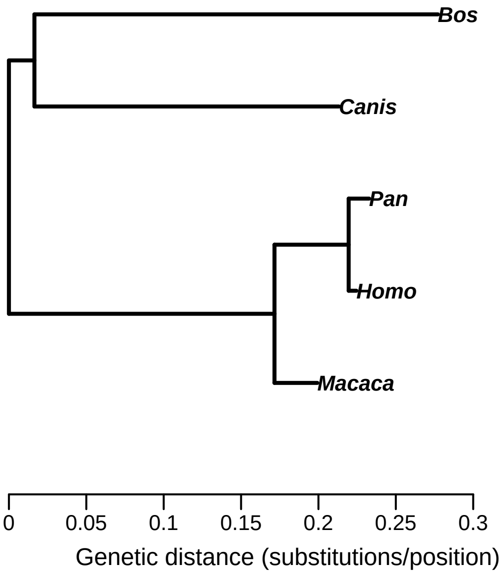

ENSG00000140274 intron 5

```
Homo    GTGAGGACCGAGAGAATGGGCCCCGGG-GGCTAAGGGTGGAGACAGGATTCACACCGGGTGTGCACTTTCCAGTTTACAGAATGAATTCACATCT---AT 96
Pan     GTGAGGACCTAGAGAACGGGCCCCGGG-GGCTAAGGGTGGAGACAGGATTCACACCGGGTGTGCACTTTCCAGTTTACAGAATGAATTCACATCT---AT 96
Macaca  GTGAGGACCGAGGGAATGGGCCCTGGG-GGCTAAGGGTGGGGACAGGATTCATACCCGGTGTGCACTTTCCAGTTTACAGAATGAATTCAC-------AT 92
Bos     GTGAGGACCAAGGGAACACGCCCCCGGTGGCTGGGAGCCGGGACGCGATTTATACC-AGCGTGCGCTTTCCAGTTTGCATAGTGAGTTCACATAA---AT 96
Canis   GTGAGGACCGAGGGGGCGGATCCCGGGAGGCCCGGGGAGGGCACGGGACTCATACCGAGGTGCGCTTTCCCCGTTTACAAAATGAGTCCACATACTAGAT 100

Homo    TACCCTATTTGCCCCTCTCAATAGTTCGCAGAAACAGGCACTGTTATGACCATTTTACAGATGAAAAGT-GGG--GGGCTCAGAAGGGTTTGGTGT---- 189
Pan     TACCCTATTTGCCCCTCTCAATAGTTCGCAGAAACAGGCACTGTTATGACCATTTTACAGATGAAAAGTGGGG--GGGCTCAGAAGGGTCTGGTGT---- 190
Macaca  TATCTGAATTGCCCCTCTCAATAGTTCACAGAAACAGGCACTGTTATGACCATTTTACAGATGAAAAGA-GGG--GGTCTTAGAAGGGGTGGGTGT---- 185
Bos     TATCTGATCTGTCCCGCTCAATAGTTCGTAGACGCGGACATTGTTACACCTATTTTGCAGATGAGGGAGGGGAACGGAGTTCGGAAGGGTTCCTTCGGTT 196
Canis   CATCTGATTTGCTCCTCTTAATAGCTCCTACAAGCAGGCACTGTTATACCCATTTTCCAGAGGAGGC---CAG--GGGCTTCTGAAGGGCGCTTGC---- 191

Homo    -----CTTG--------------CCGTGTTTCATGTAATTCAGATTAGAGGTGTGTG------------------------------------------- 227
Pan     -----CTTG--------------CCGTGTTTCATGTAATCCAGATTAGAGGTGTGTGGG----------------------------------------- 230
Macaca  -----CTTG--------------CCGTGTTTCATGTAATCCAGATTAGAGGTGTGTGTA----------------------------------------- 225
Bos     TCTTACCCACTGAGCACA-GAACCCGCGTATCATGT-ATCCATACTAGAGATCTTTCTGCGGGTTGTGTGTGTGTGTGTGTGTATGTGTCTGTGTATGTG 294
Canis   -----CCCAGTTAGCACATGAACTCGTGTATCACGTAATCCGTATTAGAGGTCTTTCTGC------------------------------------TGCA 250

Homo    ------GCGGGAGGTAACACAAGGGGTAGGCTCCAAAAGATGGAAGAAGGCCCGGGCA-------TCACGCCTGTAATCCCAGCACTTTGGGAGGTCGAG 314
Pan     -GGGCCGGGGGAGGTAACATAAGGCGTAGGCTCCAAAAGATGGAAGAAGGCCCGGGCA-------TCACGCCTGTAATCCCAGCACTTTGGGAGGTCGAG 322
Macaca  -GGGCCCGGGGAGGTAATACAAGGGGTAGGCTCCAAAAGATTGAAGAGGGCCCGGGCATGGTGGCTCAAGCCTGTAATGCCAGCACTTTGGGAGGTCAAG 324
Bos     TGTGTCTGGGGAGGAAATGCAGA----TACTTCTAATAGATGGAGATGGGGA------------------------------------------------ 342
Canis   TGTGCTGGGGGAGGAAATGCAAGGTGTAGCTCCCGGGAGATGGAAGCGGGCT------------------------------------------------ 302

Homo    GCAGGAAGGTCGTTTGAGGCCA-GAGTTCGAGACCAGCCTGGGCAAC----ATAGCCCTGACTCCACATGCCCTCCTTTCTTTCGATCCCCACCGCCACA 409
Pan     GCAGGAAGGTCGTTTGAGGCCA-GAGTTCGAGACCAGCCTGGGCAAC----ATAGCCCTGACTCCACATGCCCTCCTTTCTTTCGATCCCCACCGCCACA 417
Macaca  GCAGGAAGGTCGCTTGAGGCCAGGAGTTCGAGACCAGCCTAGGCAAC----ATAGCCCTGACTCCACATGCCTTCCTTTCTTTCCGTCCCCGCCGCCACA 420
Bos     -------------------------------GGCCGGGCTCTGAAGCCCTAACAGCCTCCTTTACCCAC-CCCCAACCCCGCCCCGACTGCGCTATCGCA 410
Canis   -------------------------------GGCCAGGTTCTGGCCT----GCAGACCTGACT-------ACCCTGTTTCTTTCTGTCGGGGTCACCGCA 360

Homo    G 410
Pan     G 418
Macaca  G 421
Bos     G 411
Canis   G 361
```

ENSG00000168306 intron 3

Description: Acyl-coenzyme A oxidase 2, peroxisomal (ACOX2)
Intron number: 3
Human chromosome: 03
Intron start (bp): 58494320
Human intron length : 441
Intron alignment length: 468
Flanking exons length (upstream/downstream): 152/108
SNP density: 0.000000
K tree score: 0.0778
Scaling factor: 0.8176
Human-chimpanzee distance: 0.016106
Total primate branch length: 0.1008

ENSG00000168306 exon 3

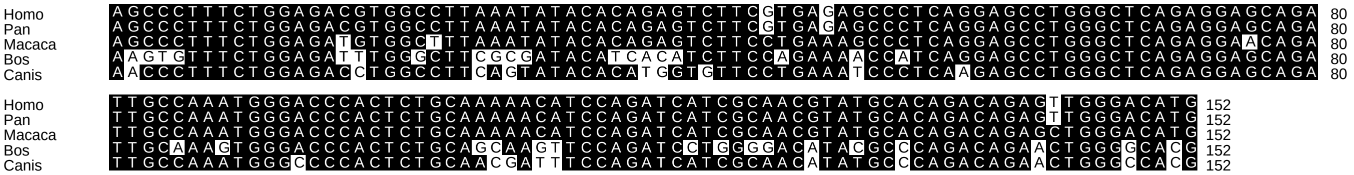

ENSG00000168306 exon 4

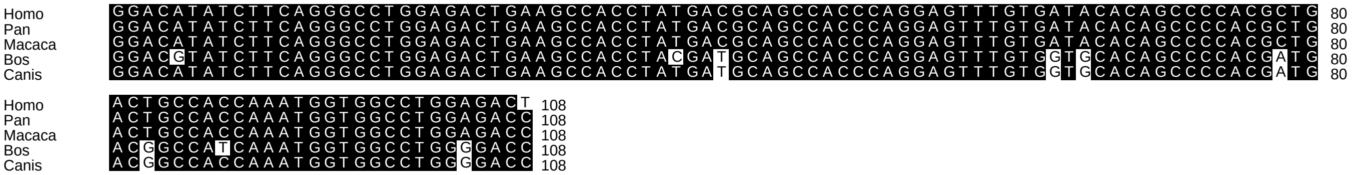

ENSG00000168306 intron 3

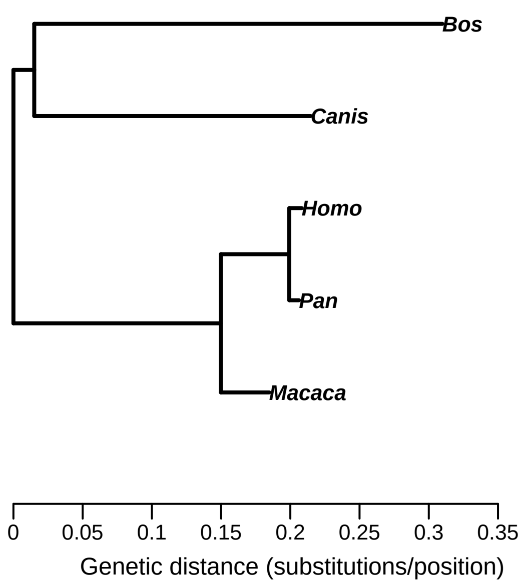

ENSG00000168306 intron 3

```
Homo    GTGAGCTAGGACTGCTGCGTGTGGTGTTTAGGTTGTGCACTGCAAAACTCCAGAGGGCCCATTTACATCTAGGTAGTCATAGGTTCATATGCTCCGAGGG 100
Pan     GTGAGCTAGGACTGCTGCGTGTGGTGTTTAGGTTGTGCACTGCAAAACTCCAGAGGGCCCATTTACATCTAGGTAGTCATAGGTTCATATGCTCCGAGGG 100
Macaca  GTGAGCCAGGGCTGCTGCATGTGGTGTTTAGGTTGTGCACTGCAAAACTCCAGAGGGCCCATTTACATCTCGGTAGTCACAGGTTCATAGGCTTCAAGGG 100
Bos     GTGAGCAGGGGCTGCTGCATGCAGCGTACAGGTAATGCCCTGCTCAGCTCCAGAGGGCCCATTTCCATCTCAGTCGTCACAGGCTGATATACT------- 93
Canis   GTGAGCCAGGGCTTCTGCCTGCAGAGTTCAGGTGGTGCAC-----------------------------------------AGTTCCGGAGCGTCAGAGA 59

Homo    ACATATGTTTAATAAGAAAACATTCCAGCAGCTGGCAGTCAAGGGTCCTGAGGA-AGGGGTGCCACCTTCTAATTTGCACGTA------TTGTATGAGCT 193
Pan     ACATATGTTTAATAAGAAAACATTCCAGCAGCTGGCAGTCAAGGGTCTTGAGGA-AGGGGTGCCGCCTTCTAATTTGCACGTA------TTGTATGAGCT 193
Macaca  ACATATGTTTAATATGAAAACATTCCAGCAGATGGCAGTCAAGGGTCTTGAGGA-AGGGATGCTGCCTTCTAATTTGCACATA------TTGTGTGAGCT 193
Bos     -------------AGCAAGATGTCACAGCAGGTGGCAGTGTAAAGGTCTTAAGAGGGGGTGATTTTTTTCTAATTTACACGAACATA--CCGTATGAACT 178
Canis   CTGTGTATTTCA-ATGCAAATGTTCCAGCAGGTGGCAGTGAAGGGTCTCGAAGGCGGGGGTTCCTTTTTCTAATTGATACAAACACCGCCCGTATGAGCC 158

Homo    ------------CCCAGTGGGGCAAGTCATTCCTTCTGGAAACATCAGCAGCCACCCCTTCTCATGGACTGGCTATAGGGGACTGTGTGTCCCACACACA 281
Pan     ------------CCCAGTGGGGCAAGTCGTTCCTTCTGGAAACATCAGCAGCCACCCCTTCTCACGGACTGGCTATAGGGGACTGTGTGTCCCACACACA 281
Macaca  ------------CCCAGTGGGGCAAGTAATTCCTTCTGGAAACATCAGCAGCCATCCCTTGTCACAGACTGGCTATAGGGGACTGTGTGACCCCCACACG 281
Bos     ------------GGTGGTGGCACAAGTCCTTCCTACTGGAAATGTCAGCCACCACCCGCTATCGTGGGCTGGCCATGTG-----------TCCCCGCAGA 255
Canis   AGCTATGAGCCAGCGTGTGGGGCAAGTTGTCCCTTCTGGAAATGACAGCAGGCATCCCTTGTCACGGGCTGGCCATGTGGG---------------CACA 243

Homo    GCCCACAGATGAAAGGGGAGAACTGCCTGCAAGTACAGCTG---GGAGCTGGGCCCCACTCCATACTGCGCTGATGCCTAAGAGTT-CAGACCCACCTAG 377
Pan     GCCCACAGATGAAAGGGGAGAACTGCCTGCAAGTACAGCTG---GTAGCTGGGCCCCACTCCATACTGCGCTGTTGCCTAAGAGTT-CAGACCCACCTAG 377
Macaca  GCCCACAGATGAAAGGGGAGAACTGCCTGCAAGTACAGCTG---GGAGCTGAGCCCCACTCTGTACCGCACTAATGCCTAAGAGTA-CAGGCCCACCTAG 377
Bos     GCCCACCAGTGAAGGGAGAGACATGCC-GGGAACACACTGGAGTGGGAGTGTGCCCCACTCCACACTG-GCTGGTGCCTGGGAGCTCCAGGGACACCCAG 353
Canis   GCCCACGGGTGAAATCCTGGAAATGCCTGTGAGTATAGCTG---CGAGCT----CCGACTCCAGACTGGGCTGGTACCTGGCAGTTCCCGGCCCACCAGG 336

Homo    AAGCAGCTTCACAGGTAAGAACCCCACCTCAGGCCAATGT----CTAACACCTCTCCTTGATCCTAAG 441
Pan     AAGCAGCTTCACAGGTAAGAACCCCACCTCAGGCCAATGT----CTAACACCCCTCCTTGATCCTAAG 441
Macaca  AAGCAGCTTCCCAGGTAAGATCCCCACCTTGGGCCAAT-T----CTAACATCCCTCCTTGATCCTAAG 440
Bos     ACGCTGCTAGCCCAGC-AGACTCCCGCCCTGGCCCAACGT----CTACCTACCCGCCTTGATTCCCAG 416
Canis   ACGCTGCTCCCCAGGTGAGATCCCTGTCCTGACCCCCTGTCTAACTAACTCCTCTCCTTGATTCCCAG 404
```

ENSG00000100162 intron 1

Description: Centromere protein M (CENPM)
Intron number: 1
Human chromosome: 22
Intron start (bp): 40672447
Human intron length : 523
Intron alignment length: 574
Flanking exons length (upstream/downstream): 57/80
SNP density: 0.005736
K tree score: 0.0599
Scaling factor: 0.5539
Human-chimpanzee distance: 0.009742
Total primate branch length: 0.1005

ENSG00000100162 exon 1

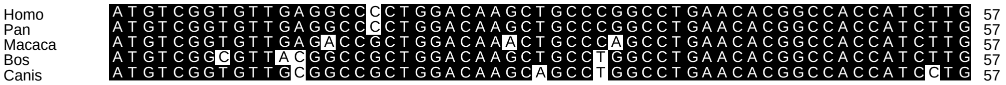

ENSG00000100162 exon 2

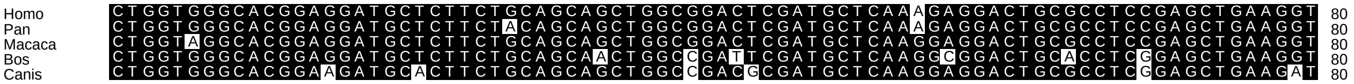

ENSG00000100162 intron 1

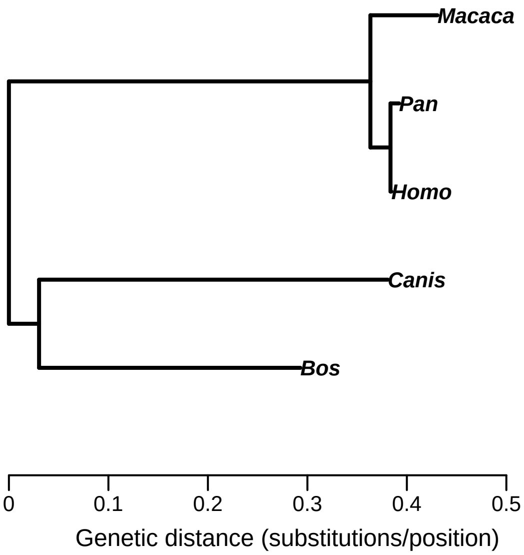

ENSG00000100162 intron 1

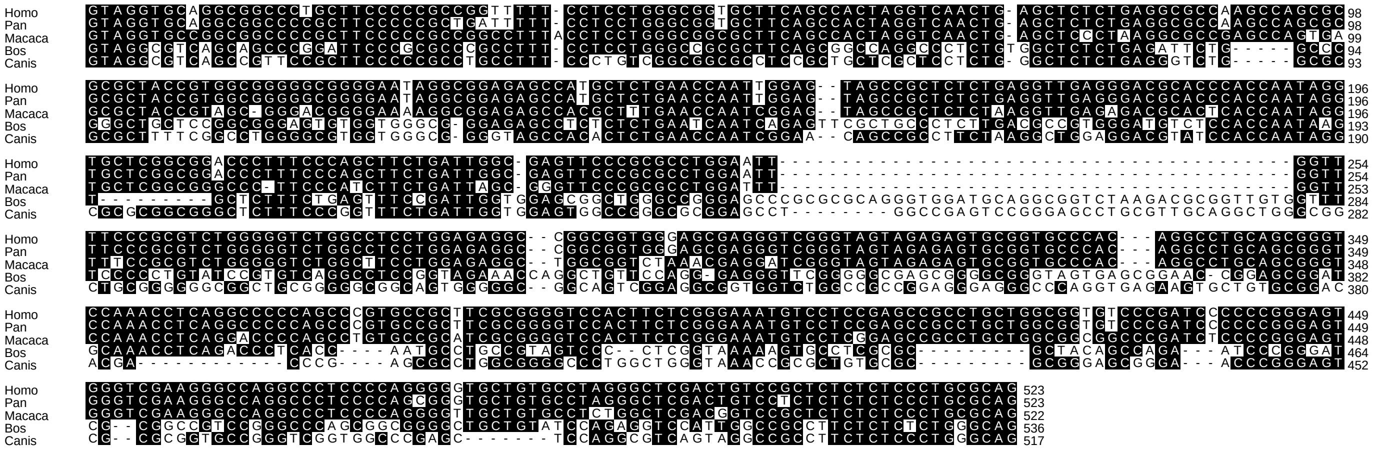

ENSG00000123454 intron 10

Description: Dopamine beta-hydroxylase precursor (DBH)
Intron number: 10
Human chromosome: 09
Intron start (bp): 135511594
Human intron length : 419
Intron alignment length: 498
Flanking exons length (upstream/downstream): 128/160
SNP density: 0.000000
K tree score: 0.0525
Scaling factor: 0.6262
Human-chimpanzee distance: 0.022917
Total primate branch length: 0.0997

ENSG00000123454 exon 10

```
Homo    GGGGGCTTCGGGATCCTGGAGGAGATGTGTGTCAACTACGTGCACTACTACCCCCAGACGCAGCTGGAGCTCTGCAAGAG 80
Pan     GGGGGCTTTGGGATCCTGGAGGAGATGTGTGTCAACTACGTGCACTACTACCCCCAGACGCAGCTGGAGCTCTGCAAGAG 80
Macaca  GGCGGCTTTGGGATCCTGGAGGAGATGTGTGTCAACTATGTGCACTACTACCCCCAGACGCAGCTGGAGCTCTGCAAGAG 80
Bos     GGGGGCTTCGGGATCCTGGAGGAGATGTGCGTCAACTATGTGCACTACTACCCCCAGACGCAGCTGGAGCTCTGCAAGAG 80
Canis   GGGGGCCTGGGGACCCAGGAGGAGATGTGCGTCAACTACATCCACTACTACCCGCAGACACAGCTGGAGCTCTGCAAGAG 80

Homo    CGCTGTGGACGCCGGCTTCCTGCAGAAGTACTTCCACCTCATCAACAG 128
Pan     CGCTGTGGACGCCGGCTTCCTGCAGAAGTACTTCCACCTCATCAACAG 128
Macaca  TGCTGTGAACGCCGGCTTCCTGCAGAAGTACTTCCACCTCATCAACAG 128
Bos     CGCCGTGGACCCTGGCTTCCTGCACAAGTACTTCCGCCTCGTGAACAG 128
Canis   CCATATAGACCCCTGCTTCCTGCAGAAGTATTTCCACCTAGTGAACAG 128
```

ENSG00000123454 exon 11

```
Homo    GTTCAACAACGAGGATGTCTGCACCTGCCCTCA----------------------------------------------- 33
Pan     GTTCAACAACGAGGACGTCTGCACCTGCCCTCA----------------------------------------------- 33
Macaca  GTTCAACAATGAGGAGGTCTGCACCTGCCCTCA----------------------------------------------- 33
Bos     GTTCAACAGCGAGGAAGTCTGCACCTGCCCCCA----------------------------------------------- 33
Canis   GTCCAACCTAGGGGAATACTGCACCTGCCCTCAGGCTTCTGGCACCACCTGCCCTCAGGCCTCTGGCACCACCTGCCCCC 80

Homo    -GGCGTCCGTGTCTCAGCAGTTCACCTCTGTTCCCTGGAACTCCTTCAACCGCGACGTACTGAAGGCCCTGTACAGCTTC 112
Pan     -GGCGTCCGTGTCTCAGCAGTTCACCTCTGTTCCCTGGAACTCCTTCAACCGCGATGTACTGAAGGCCCTGTACAGCTTC 112
Macaca  -GGCATCCGTGTCTCAGCAGTTCGCCTCTGTTCCCTGGAACTCCTTCAACCGCGAAGTACTGAAGGCCCTGTACGCCTTC 112
Bos     -GGCGTCTGTCCCTGAGCAGTTTGCCTCCGTGCCCTGGAACTCCTTCAACCGCGAGGTGCTCAAGGCCCTGTACGGCTTC 112
Canis   GGGCCTCCGTCCCCGAGCAGTTTGCTTCGGTTCCCTGGAACTCCTTCAGCCGAGTGGTGCTCAAGGCCCTGTATGACTTC 160
```

ENSG00000123454 intron 10

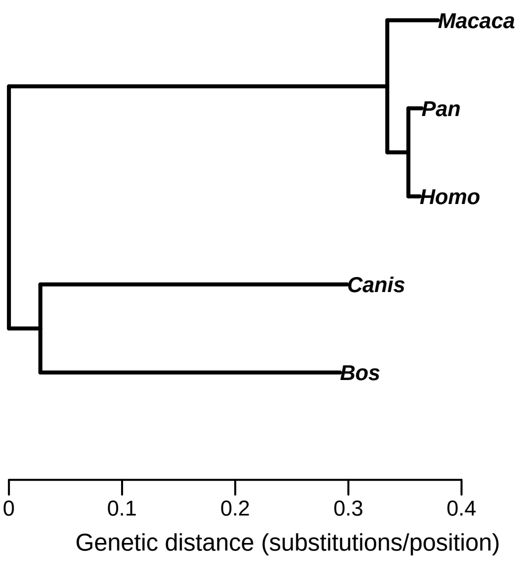

ENSG00000123454 intron 10

```
Homo    GTGAGGGCTCCCTGCA-----------CAAGCTCCCTGCCCCCAGGGAA------------------------------CCCCGACACAGAACCTC-GGG 58
Pan     GTGAGGGCTCCCTGCA-----------CAAGCTCCCTGCCCCCAGGGAA------------------------------CCCCGACACAGAACCTC-AGG 58
Macaca  GTGAGGGCTCCCTGCA-----------CAAGCTCCCTGCCCCCAGGGAG------------------------------CCCCGACACAGAACCTCTGGG 59
Bos     GTGATGGCTCCACGGG-GCATGCCCGCTGTGCCTTCCATCCT--GGGCATGGCAGGGCAACCCCGGCCCCGAACACTCCCCCCAACCCCAAACCCTCCAG 97
Canis   GTGACAGCTCTGCGGGCGTGTGCCTGCCATGCCCTCCGTCCCCGAGGCAGGGTGGGGGGGGGGCGATCAGAGGAA----CCCCAACATAGGAGGTGCTTG 96

Homo    CCTGTTAGGCGGCTGGGCAGATTGGAGGAGTCCAGGCTAAGTTCTAGGA------------------------------GCAGAGACCTGTGGC-GGCAT 127
Pan     CCTGTTAGGCGGCTGGGCAGATTGGAGGAGTCCAGGCTAAGTTCTAGGA------------------------------GCAGAGACCTGTGGC-AGCAT 127
Macaca  CCTGTCAGGCAGCTGGGCAGATTGGAGGAGTCCAGGCTAAGTTCTAGGA------------------------------G------CCTGTGGC-GGCAT 122
Bos     GCC---------CTGGGCGAAAGCAGGGCGCCCAGGAGCAAGGTGAGCAGGAACCTGGCGATCAGGCCCCCACCCACCTTCAGGCGCCGGTGGCAGGCAT 188
Canis   TCC---------CTGGGTAAAAGTGAGGAATCCAGGCTGAGTTCTGGGA--------GCAGCCGTGCCTCCATTTCCCTCTGAGAGCCGA-GGTCAACAT 178

Homo    CACTCACCCCTCCCCTCACTCGTTTCCTGCTGAGTCTGGATTTGGCTTCAGAGCC-TCCTCAACCCAGCCTGCAGCAGCCATTACCCACCC--GCCAAGG 224
Pan     CACTCACCCCTCCCCTCACTCGTTTCCTGCTGAGTTTGGATTTGGCTTGAGAGCC-TCCTCAACCCAGCCTGGAGCAGCCATTACCCACCC--GCCAAGG 224
Macaca  CACTCACCCCTCCCCTCACTCATTTCCTACTGAGTCTGGATTTGGCTTGAGAGCC-TCCTCAACCCAGCCCGGAGC-------ACCCACCC--GCCAAGG 212
Bos     CCCCAGGCGACCCCCATTCTTGCTTCCTATCGAGTCTGGACTTGGCCCGAGAAGC-CCCTCCACCCAGCCCTGGAGCAGCCGTTCCCACCTGAGCCAAGA 287
Canis   CGCTAATCCAGCCCCATACACTCTTCCAACCAAGCCTGGACTTGGCCCAAGAAGCTCCCTCGGCCCAGCCCTGGAGTGGCCCTTCCCAGCCCCTGCAGGG 278

Homo    TGACAGGAGAGGTGAGGCCG--TTGCCGGTGAAGGCAGCATCTGGGGTGGCCTGGCGGAGGCAGCGGGGCTGGGGAGGAGGTGGCAGGACGGTTGCCCCA 322
Pan     TGACAGGAGAGGTGAGGCCG--TTGCCGGTGAAGGCAGCATCTGGGGTGGCCTGGCGGAGGCAGTGGGGCTGGGGAGGAGGTGGCAGGACGGTCGCCCCA 322
Macaca  TGCCAGGAGAGGTGAAGCCG--TTGCTGGTGAAGGCAGTATCTGGGACGGCCTGGTGGAGGCAGCGGGGCTGAGGAGGAGGCGGCAGGAGGGTCTCCCCA 310
Bos     TGGCACAAGAGATGAAGCCTATTCGTCGT--CAGGG-------CGGGAGGCAGGGCCAGGGCAGT---------------GTGCTAGAAGGGCCGCTCTG 363
Canis   -GACTCAAGAGGTGAAGCCGACCAGCCATTGCAGGC-------GAGGAGCCACAGCCTAGACAG-----------------------------CACTTTG 341

Homo    GGGACAGGACTCGAGTTGCAGGGAGGTGTTGCTGGTGCC-CACTGGGCTCCCTGCCCGTCAGCTGCTCCCCAGCCTGGCTGTTCCTTGTCCCCACCAG 419
Pan     GGGACAGGACTCGAGCTGCAGGGAGGTGTTGCTGGTGCT-CACTGGGCTCCCTGCCCGTCAGCTGCTCCCCAGCCTGGCTGTTCCTTGTCCCCACCAG 419
Macaca  GGGACAGGACTTGACCTGCAGGGAGGTGTCGCTGGTGCC-CACTGGGGTCCTTGCCCATCGGCTGCTCCCCAGCCTGGCTGTTCCTTGTCCCCACCAG 407
Bos     GGGACCCCAC-CCCTCTTCAGGGAGGCATCACGGGTGCCTTGTTGGACTCCCCTCCGCTCCCCCAGACCCCACTCTGGACGGCTCCTTCTCTCACCAG 460
Canis   GTGGCAGCAG-GGC----CAGGGAGGTGTCAC-----CCACAGCCACCCACCGGCGCCGCCACAG---------CTGGGCTCTCTCCTCCCTCACCAG 421
```

ENSG00000130810 intron 2

Description: Suppressor of SWI4 1 homolog (PPAN)
Intron number: 2
Human chromosome: 19
Intron start (bp): 10078348
Human intron length : 833
Intron alignment length: 865
Flanking exons length (upstream/downstream): 171/102
SNP density: 0.003601
K tree score: 0.0626
Scaling factor: 0.8747
Human-chimpanzee distance: 0.025810
Total primate branch length: 0.0995

ENSG00000130810 exon 2

```
Homo    GAAGCGCGCCCGCGCCCAGGCGCAGCTCCGCAACCTCGAGGCCTATGCCGCGAACCCGCACTCGTTCGTGTTCACGCGAG 80
Pan     GAAGCGCGCCCGCGCCCAGGCGCAGCTCCGCAACCTCGAGGCCTATGCCGCGAACCCGCACTCGTTCGTGTTCACGCGAG 80
Macaca  GAAGCGCGCCCGCGCTCAGGCGCAGCTCCGCAACCTCGAGGCCTATGCCGCGAACCCGCACTCGTTCGTGTTCACGCGGG 80
Bos     GAAGCGCGCGCGCGCCCAGGCCCATCTCCGGAACCTCGAAGCCTATGCCGCGCAGCCGCACTCGTTCGTGTTTGCTCGGG 80
Canis   GAAGCGTGCGCGCGCCCAGGCGCAGCTCCGCAACCTCGAGGCCTACGCCGCGCAGCCGCACTCGTTCGTGTTCGCTCGGG 80

Homo    GCTGCACGGGTCGCAACATCCGGCAGCTCAGCCTGGACGTGCGGCGGGTCATGGAGCCGCTCACTGCCAGCCGTCTGCAG 160
Pan     GCTGCACGGGTCGCAACATCCGGCAGCTCAGCCTGGACGTGCGGCGGGTCATGGAGCCGCTCACTGCCAGCCGTCTGCAG 160
Macaca  GCTGTACGGGTCGCAACATCCGGCAGCTCAGCCTGGACGTGCGGCGGGTCATGGAGCCGCTCACTGCCAGCCGTCTGCAG 160
Bos     GCCGCGTGGGTCGCAGCGTCCAGCAGCTTAGTATGGACTTGAGGCGGGTCATGGAGCCGCTCACCGCCACCCGCCTGCAG 160
Canis   GCCGCGCGGGTCGCGGCGTCCGGCAGCTCAGCCTGGATCTGCGCCGGGTCATGGAGCCGCTGACCGCCACCCGCCTGCAG 160
```

ENSG00000130810 exon 3

```
Homo    GTTCGTAAGAAGAACTCGCTGAAGGACTGCGTGGCAGTGGCTGGGCCCCTCGGGGTCACACACTTTCTGATCCTGAGCAA 80
Pan     GTTCGTAAGAAGAACTCGCTGAAGGACTGCGTGGCAGTGGCTGGGCCCCTCGGGGTCACACACTTTCTGATCCTGAGCAA 80
Macaca  GTTCGTAAGAAGAACTCGCTGAAAGACTGTGTGGCAGTGGCTGGGCCCCTTGGGGTCACACACTTTCTGATCCTGAGCAA 80
Bos     ATACGGAAGAAAAACTCTCTGAAAGATTGTGTAGCAGTGGCTGGGCCCCTTGGGGTCACACACTTCCTGATTGTGAGCAA 80
Canis   ATACGAAAGAAAAACACCCTGAAGGATTGTGTGGCAGTGGCCGGGCCTCTTGGGGTCACCCATTTCCTGATCTTGAGTAA 80

Homo    AACAGAGACCAATGTCTACTTT 102
Pan     AACAGAGACCAATGTCTACTTT 102
Macaca  AACAGAGACCAGTGTCTACTTT 102
Bos     AACAGAGACCAACGTCTACTTT 102
Canis   AACAGAAACGAACATCTACTTT 102
```

ENSG00000130810 intron 2

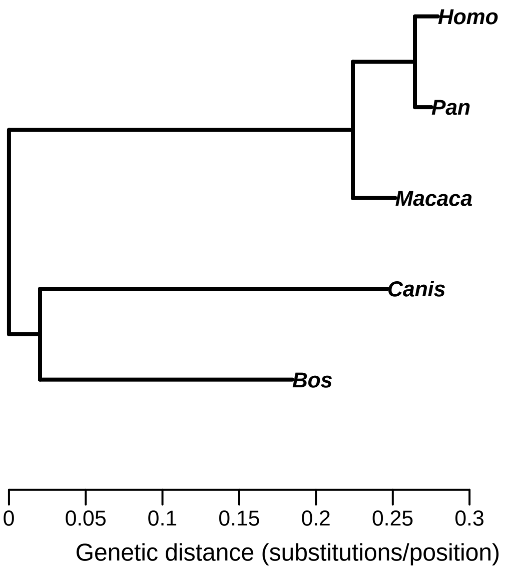

ENSG00000130810 intron 2

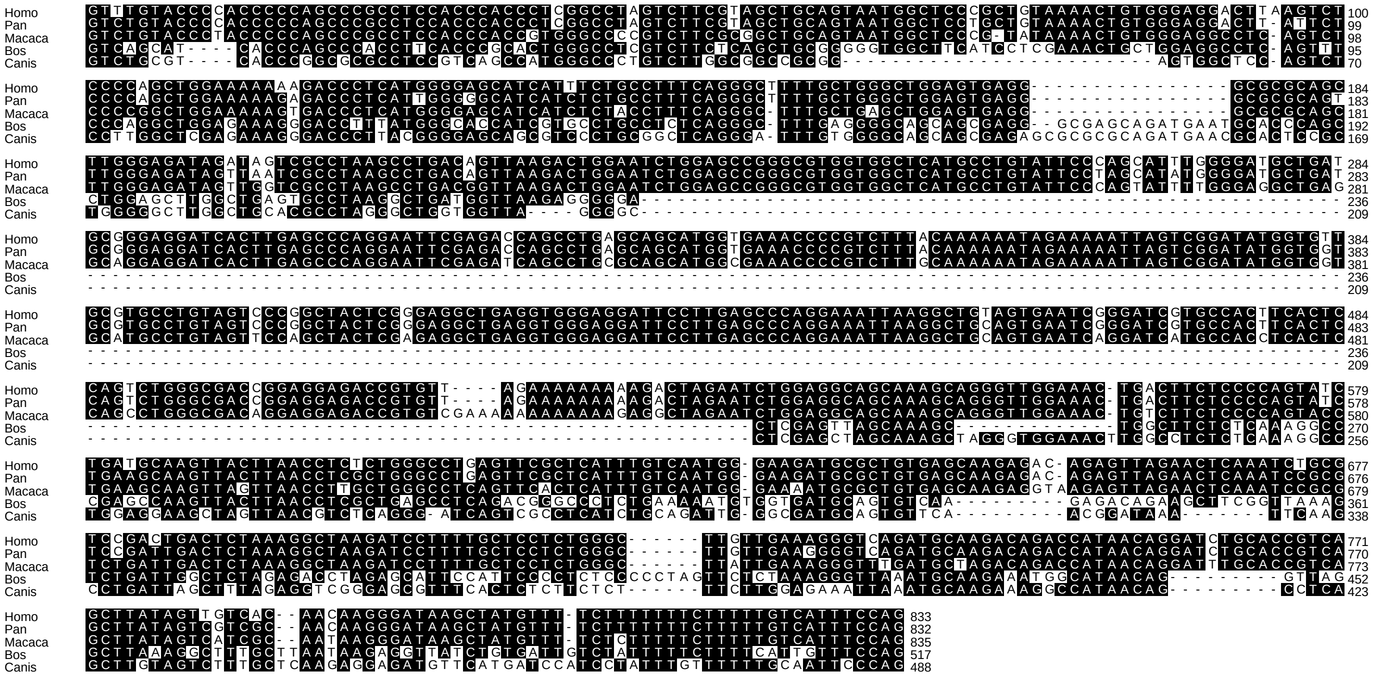

ENSG00000074317 intron 2

Description: Beta-synuclein (SNCB)
Intron number: 2
Human chromosome: 05
Intron start (bp): 175986124
Human intron length : 203
Intron alignment length: 231
Flanking exons length (upstream/downstream): 42/119
SNP density: 0.009852
K tree score: 0.0241
Scaling factor: 0.6579
Human-chimpanzee distance: 0.031016
Total primate branch length: 0.0995

ENSG00000074317 exon 2

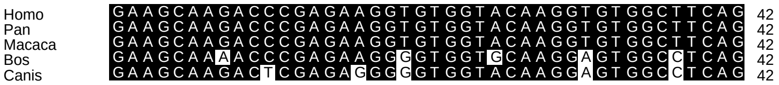

ENSG00000074317 exon 3

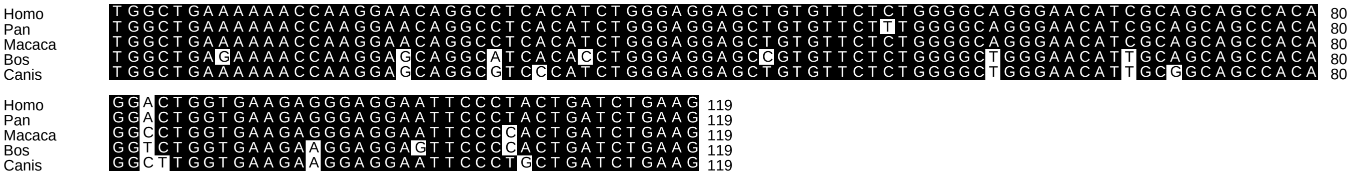

ENSG00000074317 intron 2

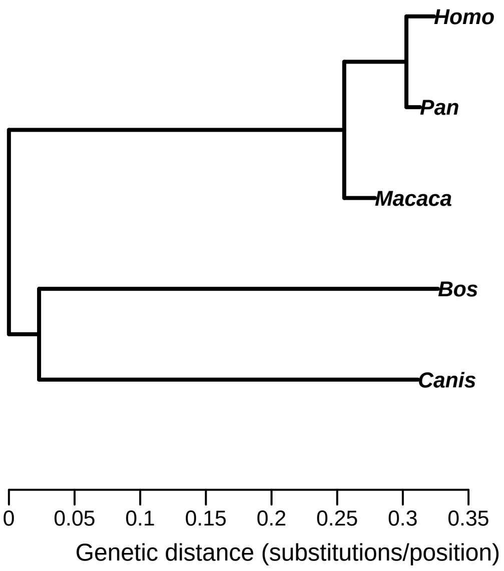

ENSG00000074317 intron 2

```
Homo    GTACTAGCCCAGCCCTGGCACCAG-CCCTTCTCTCA-CTTAGGCGGATGATCTGGCCGGGAACCAGAGGGCGGGGGCGGGGGA--------------GAC 84
Pan     GTACCAGCCCAGCCCTGGCACCAGCCCCTTCTCTCA-CTTAGGCGGATGATCTGGCCGGGAACCAGAGGGCGGGGGCGGGGGA--------------GAC 85
Macaca  GTACCAGCCCAGCCCTGGCACCAGCCCCTTCTCTCA-CTTAGGCCGGTGATCTGGCCGGGAACCAGAGGGCAGGGGCGGGGGA--------------GAC 85
Bos     GTACTAGCCCAGCCCTG---CCCACTTGTTCCCTTA-CCTTGGCCTAGGGTCTG-CAGACATCCAGAGAGTGAGGGCAGAGGGCGGCGGGAGAGGGCACC 95
Canis   GTACCAGCCCAG-CTGGGCACAGCCCCCTTCCCTCACCCCGGGCTGGAGATCTGTCTGGGATCTGGAGCGTGGGCGGTGGGGG----------------- 82

Homo    TCCCAAGGCTTCTGCGGG----AATGCTCCGTGGGGAGGGCAGGCCCTGGGATACTACAAGGCAGGGCA--------TCGGTGTTTCCCCCTGGCTCCCA 172
Pan     TCCCAAGGCTTCCGCGGG----AATGCGCCGTGGGGAGGGCAGGCCCTGGGATACTACAAGGCGGGGCA--------TCAGTGGTTCCCCCTGGCTCCCA 173
Macaca  CCCCAAGGCTTCTGCGGG----AATGCGCTGTGGGAAGGGCAGGTCCCGGGA---TACAAGGTGGGGCA--------TCGATGGTTCCCCCTTGCTCCCA 170
Bos     CCCAGAGCTTTCTGCGGGGTGTAGTGCTCTGTGGGAA-GTCAGGCCCTGGGACACTATGGAGGAGGAGG-GCATCTATGTCTGCTTGACCTTTGC----- 188
Canis   ----TGGATTCCTGAGAG----TATGCTCTGTGAGAGAGGCAAGCCCTGGGACACCACGAGGGAGAGAAGATGCATCTGTCTGCTTCACCTTAGCCCCCC 174

Homo    AACCCCTTCCTCAACCCCCTCCCTGCTCCAG 203
Pan     AACCCCTTCCTCAACCCCCTCCCTGCTCCAG 204
Macaca  AACCCCTTCCTCAACTCCCTCCCTGCTCCAG 201
Bos     --------CCCCAACCCTCTCCCTGACACAG 211
Canis   ACCCCCTTCCCCAATCCTCTCCCTGCTCCAG 205
```

ENSG00000165281 intron 2

Description: Fanconi anemia group G protein (FANCG)
Intron number: 2
Human chromosome: 09
Intron start (bp): 35068734
Human intron length : 414
Intron alignment length: 513
Flanking exons length (upstream/downstream): 91/132
SNP density: 0.000000
K tree score: 0.0776
Scaling factor: 0.778
Human-chimpanzee distance: 0.019625
Total primate branch length: 0.0994

ENSG00000165281 exon 2

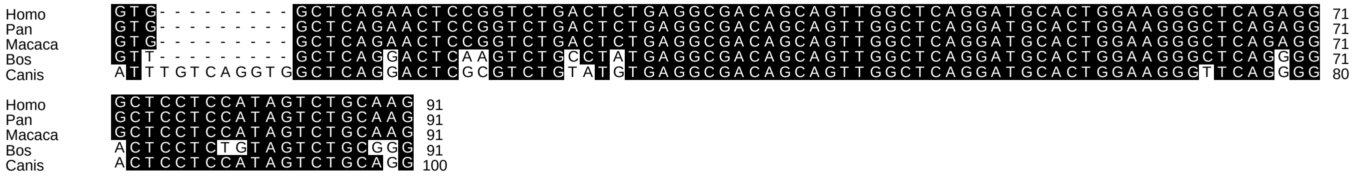

ENSG00000165281 exon 3

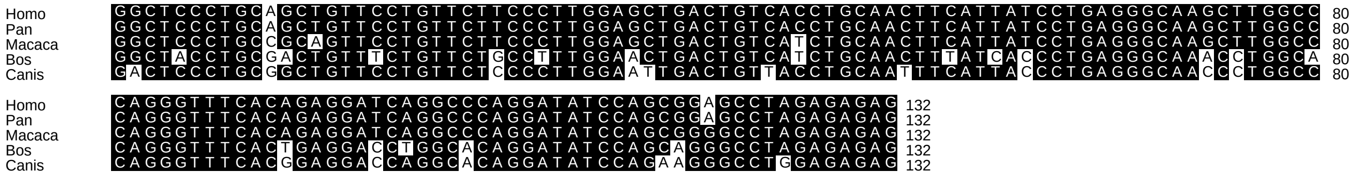

ENSG00000165281 intron 2

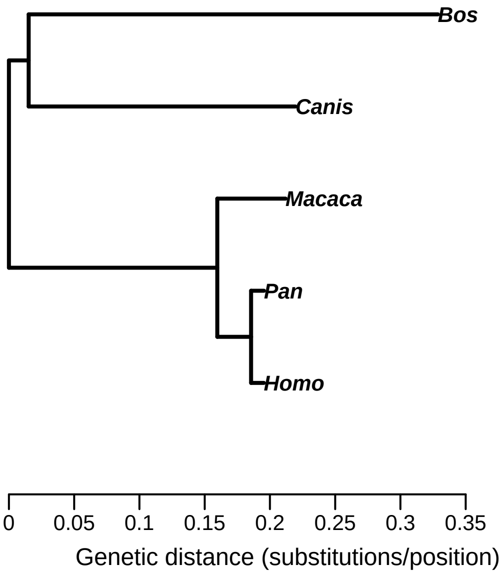

ENSG00000165281 intron 2

```
Homo    GTAGGCGGGTCCTCCCC----------------------------------------AGGATGGTCAGTTCCCCTCTTCCATAGCCAGAGAAACA-TCCG 59
Pan     GTAGGCGGGTCCTCCCC----------------------------------------AGGATGGTCAGTTCCCCTCTTCCATAGCCAGAGACACA-TCCG 59
Macaca  GTAAGCAGGTCCTCCCCTTACCTGCTTACCTTGCAGACTATGGAGGTCAGGTAAGCAGGTAAGGGGAGTTCCCCTCCTCCATAGCCAGAGACTCA-TCTG 99
Bos     GTAAGTAGGGCCCTCCCC---------------------------------------AGGATGGTC-ACTCCTGCCCTCCACATCCAGAGACCAGTTCCT 60
Canis   GTAAGCAGGATCCTCCTC---------------------------------------AGGATTGTCTGTTACCTTCCTCCACAGCCAGAGACCAGTTCCT 61

Homo    CTCCTGCGTTTTTGGGATCGATATAATTACTC--GGGGCA-GGGAGTCCTGTTTAAGGCACAGAGGAGACTGGAGTGGAATCATCTTT-GTACAGGC--- 152
Pan     CTCCTGCGTTTCTGGGATCGATATAATTACTC--GGGGCA-GGGAGTCCCGTTTAAGGCACAGAGGAGACTAGAGTGGAATCATCTTT-GTACAGGCAAA 155
Macaca  CTCCTGCATTTCTGGCATCGACAAAATTACTTAGGGGGCA-GGGAGTCCTGTTTAAGGCACAGAGGGGGCTGGAGT-GAATCATCTTT-GTACAGGC--- 193
Bos     GTTCTGCATGTCTAGAATTGAAGCGATTACTCAGGGGGTA----AGTCCTGTTTAAGACACAGACAGGACTGGAATGGAATCATCATT-GTACAGGT--- 152
Canis   CTTCTGAATTTCTGGGACTGAGAAAATTACTCAGGGGGCAGGGGAATCCTATTTAAGTCACAGAAGGGATTGGAGTGGAATTCTTTTTGGTACTGGT--- 158

Homo    -AAATCCCTCTCTTCCTTACACACTCACAGAGTGGCATTT---------------------GAAAAATGGTTTCCAAGATGGTCTGATCTGGATAACTTT 230
Pan     TAAATCGCTCTCTTCCTTACACACTCACAGAGTGGCATTT---------------------GAAAAATGGTTTCCAAGATGGTCTGATCTGGATAACTTT 234
Macaca  -AAATCCCTCTCTTCCTTACG----CACAGAGTGGCATTT---------------------GAAAAATGATTTCCAGGATGGTCTGATCTGGATAACTTT 267
Bos     -GAATCTCTCTCTTCCTTAGGCACACACACACATACATACATCGGCACACAGAAGCGTGTGAAGAAGTGATTTCCAAGACGGTCTATTTGGAA-AACATT 250
Canis   -GAATCTCTACGCCCT---GCCACGCCCCCCCCCCCCCCCCCC----------------------AGTGGTTTCCGGGATGGTCCAATCTAAA-GGACTT 231

Homo    TTTGCCCTTTCCAGACTCTCTGTCCAAAGGTTGGGTTCTTTTTTTAAATCCTTAGCTAATTGGCTGGAGGGTGGGTTCTTT-------ATTGTAGCTG-T 322
Pan     ATTGCCCTTTCCAGACTCTCTGTCCAAAGGTTGGGTTCTTTTTTTTAATCCTTAGCTAATCGGCTGGAGGGTGGGTTCTTT-------ATTGTAGCTG-T 326
Macaca  TTTGCACTTTCCAGACTCTCTGTCCAAAGGTTGGGTTCTTTTTTTTAATCCTTAGCTAATCGGCTGGAGGGTGGGTTCTTT-------ATTTTGGCCATT 360
Bos     CTGGCTGTTCCATTACACACACACACACACACACACACACGGAAGCA--TTTGAGGAAACAGTTTCCAAGATGGCCTATTTGGAAAACATTGTGGCCG-T 347
Canis   TCTGCCCTTTGTGTGCAAAC-------------------------------------------------GGTGATTTCTTT-------ATTGAGGCCA-T 274

Homo    TTTG--------------GTTGGAGTGGGGTGATCTCTGAG-----TTGGGATTTTATTTGGAGGA-TCTTCCATGGGGGAGCAGTCTGTAAGACCAACA 402
Pan     TTTG--------------GTTGGAGTGGGGTGATCTCTGAG-----TTGGGATTTTATTTGGAGGA-TCTTCCATGGGGGAGCAGTCTGTAAGACCAACA 406
Macaca  TTTG--------------GTTGGAGGGGGGTGATCTCTGTG-----TTGGTATCTTATTTGGAGGA-TCTTCCATGGGGGAGCAGTCTGTAAGACCAACT 440
Bos     TCTATAATCTACCAGAGTACCAGAGTGGGTTCTTTATTGAGGACATTTTGGTTGGGGTGGGGTGATCTCTTTCACCTGAGAGTGGTCTGTGAGATCAGCA 447
Canis   TTTG--------------GTTGGGGTGAGATGCCCTTTGAG-----TTTGGTTCTTATTTGGGGAATCTTTCTGGAGGGGAGTGGTCTATAAGGCCAGCA 355

Homo    T-TGTTGCTCCAG 414
Pan     T-TGTTGCTCCAG 418
Macaca  T-TGTTGCTCCAG 452
Bos     TTTGTGGCTCCAG 460
Canis   TGTGTTGCTCCAG 368
```

ENSG00000087903 intron 16

Description: DNA-binding protein RFX2 (RFX2)
Intron number: 16
Human chromosome: 19
Intron start (bp): 5945962
Human intron length : 650
Intron alignment length: 690
Flanking exons length (upstream/downstream): 43/116
SNP density: 0.009231
K tree score: 0.095
Scaling factor: 0.4762
Human-chimpanzee distance: 0.018807
Total primate branch length: 0.0989

ENSG00000087903 exon 16

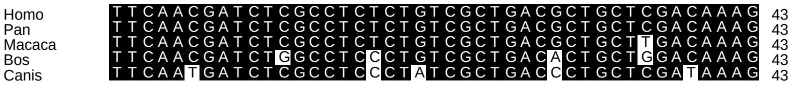

ENSG00000087903 exon 17

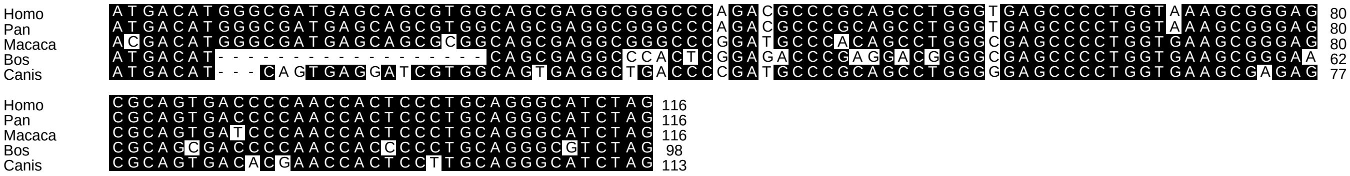

ENSG00000087903 intron 16

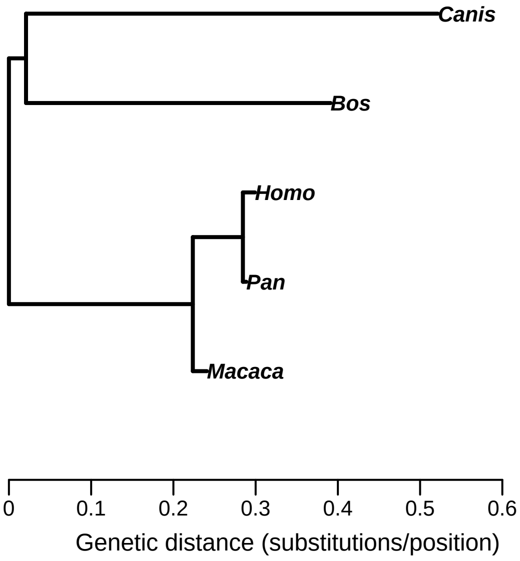

ENSG00000087903 intron 16

```
Homo    GTAGGTGCGTCTGCGGCTTTCCCACCACGACCCTCCCAGGAGGGTGGTACTCCTGGCCGTGGCCTGTCTGCCCCATCTCATGATGAACAGGTGGAAATGC 100
Pan     GTAGGTGCGTCTGCGGCTTTCCCACCACGGCCCTCCCAGGAGGGTGGTACTCCTGGCCGTGGCCTGTCTGCCCCATCTCATGATGAACAGGTGGAAATGG 100
Macaca  GTGGGTGTGTCTGCAGCTTTCCCGCCACGGCCCTCCCAGGAGGGTGGTACTCCTGGCCGTGGCCTGTCTGCCTCATCTCATGATGAGCAGGTGGAAATGG 100
Bos     GTGGGTGTGTCTG--------------------------------GGCGCTCCCTCCCGTGCAC-------------------------AGTGGAGGTGG 43
Canis   GTGGGTATGTCTGTAGCTTTTCCGGGAGG--CTCTCCCAGGGGCTATTCCTCCCACCTTTGGCCT------------------------AGTGGAAATCG 74

Homo    GGGCAGCCCCTTGGGGGG---TGAGGGCCCTGGGATCTGTCATCCGGACAGT-GGGTGATG--CCTGAGCAGCAGGTGACACTGCAGACTGGCTCGGCTG 194
Pan     GGGCAGCCCCTTGGGGGG---TGAGGGCCCTGGGATCTGTCATCCAGACAGT-GGGTGATG--CCTGGGCAGCAGGTGACACTGCAGACTGGCTCGGCTG 194
Macaca  GGGCAGCCCC-TGGGGGG---TGAGGGCCCTGGGATCTGGCGTCCAGACAGT-GGGTGAGGGCCCTGGGCAGCAGGCGCCACTGCAGACTGGCTCGGCTG 195
Bos     GGGCAGCCCCTAAGGCGG---GGCTGGGC--GGCAGCCTGCATGTCCACCGG-GGCTCAGA----------GCAGAAGCCCCTTCCCTCCTCCTAGATC 127
Canis   AGGCAGCTCCTGGGGTGGGTTGAGGGGCTTTAGGAGTCAGCATCCAGATAGGCTGGTGAGA--CCTGAGCAGCAGGAGGTGTCCTACTGATGTCCTACTG 172

Homo    GGGC---TCAGACTGCCGCTGGCCTCTGGG---------TAGCCGCTGCCCAGGGACCTCAGCACCAGGCCCAGACTAGGCAGCCCCTGGTCTCCTGCGC 282
Pan     GGGC---GCAGAGTGCCGCTGGCCTCTGGG---------TAGCCGCTGCCCAGGGACCTCAGCACCAGGCCCAGACTAGGCAGCCCCTGGTCTCCTGCGC 282
Macaca  GGGC---TCAGAGTGCTGCCGGCCTCTGGG---------TAGCGGCTGCCCAGGGACCTCAGCACCAGGTCCGGACCAGGCAGCCTCTGGTCTCCTGCGC 283
Bos     ACTA---TCCTGCTCCCCGGCCGTCTACA----------CGAGGCCCG---GAGGAGCAGAGCCATGGACGGGACTGGGGCGGCTCACTGCCTCCTCCCT 212
Canis   ATTCAGGCCTGGCCCCTGCTGGGGCTTAGAGGAGCAGCCCCGCACCTCCTTGGGTGCTTTGGGACGACATCCAATCC---TGCTCCCCGGGCACCTCCAC 269

Homo    CCACGCCCAGCAGGAGAGTGGGACAGGCAGGCCA--GAGGGACC-----------CCCGGGCCCCGTCTCTGTCCCATGCGGGTG---GG---AGGACCC 363
Pan     CCACGCCCAGCAGGAGAGTGGGACAGGCAGGCCA--GAGGGACC-----------CCTGGGCCCCGTCTCTGCCCCATGCGGGTG---GG---AGGACCC 363
Macaca  CCATGCCCAGCAGTAGAGTGGGACAGGCAGGCCA--GAGGAGCC-----------CCTGGGCCCCGTCCCTGCTCCACGCGGGTGGGAGG---AGGACCC 367
Bos     CCATGTCTGGAGAGGGAGTGGGGCAGGTGGGGCAATGAGCACCC-----------CCAGGGCCCATTCCTTGCCCCACGGCGATGGGAGGCCCAGTGCCC 301
Canis   ATCAGGCCAGGAGTAGAAC--CACAGCCCTGTCTGCCTGCATCCACTTCCCCTTACCCCAACCCAGGCCCTCCCCTACAGGATTAGGGTACCAACTGCTG 367

Homo    CAGAGGCCCCAGCCCTGTGGGGAGCAGGCCCCAGGCCCGTCGGCGCTGGGAAAGGGAGGCACAGGGCAGAGGCTCTTTCGCCTGGCCCGAGCTGGGGGCA 463
Pan     CAGAGGCCCCAGCCCTGTGGGGAGCAGGCCCCAGGCCCGTCGGCGCTGGGAAAGGGAGGCACAGGGCAGAGGCTCTTTCGCCTGGCCCAAGCTGGGGGCA 463
Macaca  CAGAGGCCCCAGCCCTGTGGGGAGCAGGCCCCAGGCCTATCGGCCCTGGG-GAGAGAGGCACAGGGCAGAGGCTCTTTCGCCTGGCCCGACCTGGGGGCA 466
Bos     AAAGGG----GACCCTACCAGGCGCATG-ACCAGGCGCATGGGCACTGGT-------------------------------------------------- 346
Canis   AAGGG-----AACCCTGCCAGGAGTGTG-ACCCAGCAAATGGACACTGGGCAAGGGAGGTCTCCAGTGAGAAGGCCTCCCT--------------GAGCA 447

Homo    CCTCTGGGTAAC--GGCAGGTGGG-GAGGACAGGTCAGCCTGGAGCAGAGGCCGAGGTGGGGTCCACAGGCTGCCATGTATCCTCGGTGCCCTGCCTGGC 560
Pan     CCTCTGGGTGAT--GGCAGGTGGG-GAGGACAGGTCAGCCTGGAGCAGAGGCCGAGGTGGGGTCCACAGGCTGCCATGTATCCTCGGTGCCCTGCCTGGC 560
Macaca  CCTCTGGGTGACCAGGTAGGTGGGAGAGGACAGGTCAGCCTGGAGCAGAGGCCAAGGCGGGGTCCACGGGCTGCCATGTGTCCTTAGTACCCTGCCTGGC 566
Bos     -CCGAGGGTGGTC-GCAGGGAGGAAGACCCCAGTCAACCCAGACCCGA-GCCAAGGCAGGGCCCTCA---------------------------CTTGGC 417
Canis   CTTCAGGGTGGCC--------GGGGGGGCCTGGTGTGTCCAA-------GCCTGTGCACCTCCCTGA--------------------------------- 500

Homo    GTGCCGGAGCAGTTCTCGGAACGTGCACTTCCTTCTCTCCCTCTCGTGCCCTCCTGATGATGGACACTGACCCTACCCCTCCCTCCGCAG 650
Pan     GTGCCGGAGCAGTTCTCGGAACGTGCACTTCCTTCTCTCCCTCTCGTGCCCTCCTGATGATGGACACTGACCCTACCCCTCCCTCCACAG 650
Macaca  GTGCCGGAGCGGCTCTTGGAACATGCGCTTCCGTCTCTCCCTCCCATGCCCTCCTGATGATGGACACTGACCCTACCCCTCCCTCCACAG 656
Bos     CTG---------------------------------------TTCATGCCCCCC---------------ACCCCACCCGACC--CCACAG 451
Canis   ------------------GAACATGCTTCTCTCTCCCTCCCTCCTGCACCCCAC-TCCCACACCCCTCATCCCCAATGTTAACCCTGCAG 571
```

ENSG00000144837 intron 10

Description: phospholipase A1 member A (PLA1A)
Intron number: 10
Human chromosome: 03
Intron start (bp): 120830403
Human intron length : 518
Intron alignment length: 854
Flanking exons length (upstream/downstream): 165/85
SNP density: 0.003861
K tree score: 0.0422
Scaling factor: 1.111
Human-chimpanzee distance: 0.011806
Total primate branch length: 0.0989

ENSG00000144837 exon 10

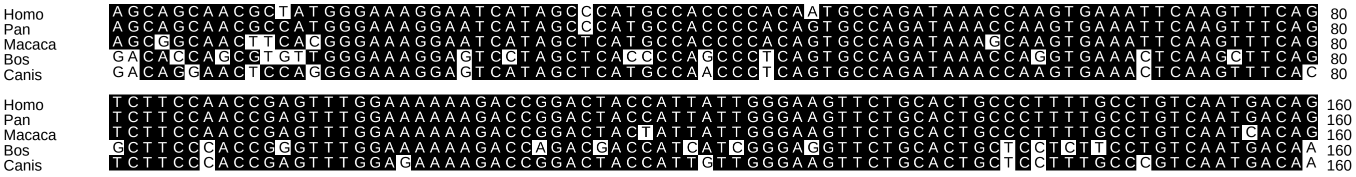

ENSG00000144837 exon 11

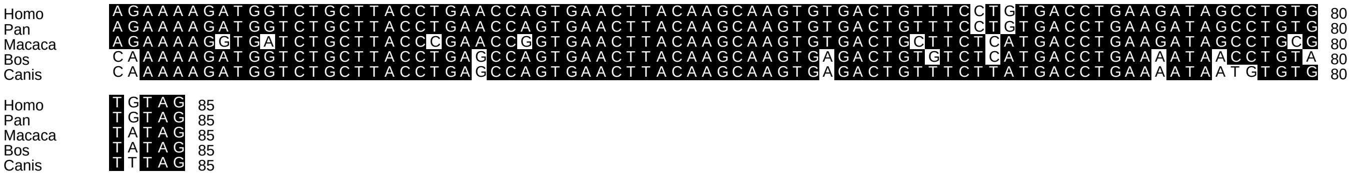

ENSG00000144837 intron 10

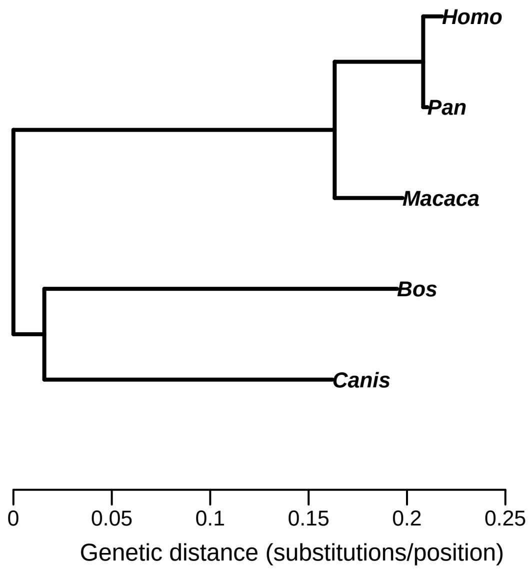

ENSG00000144837 intron 10

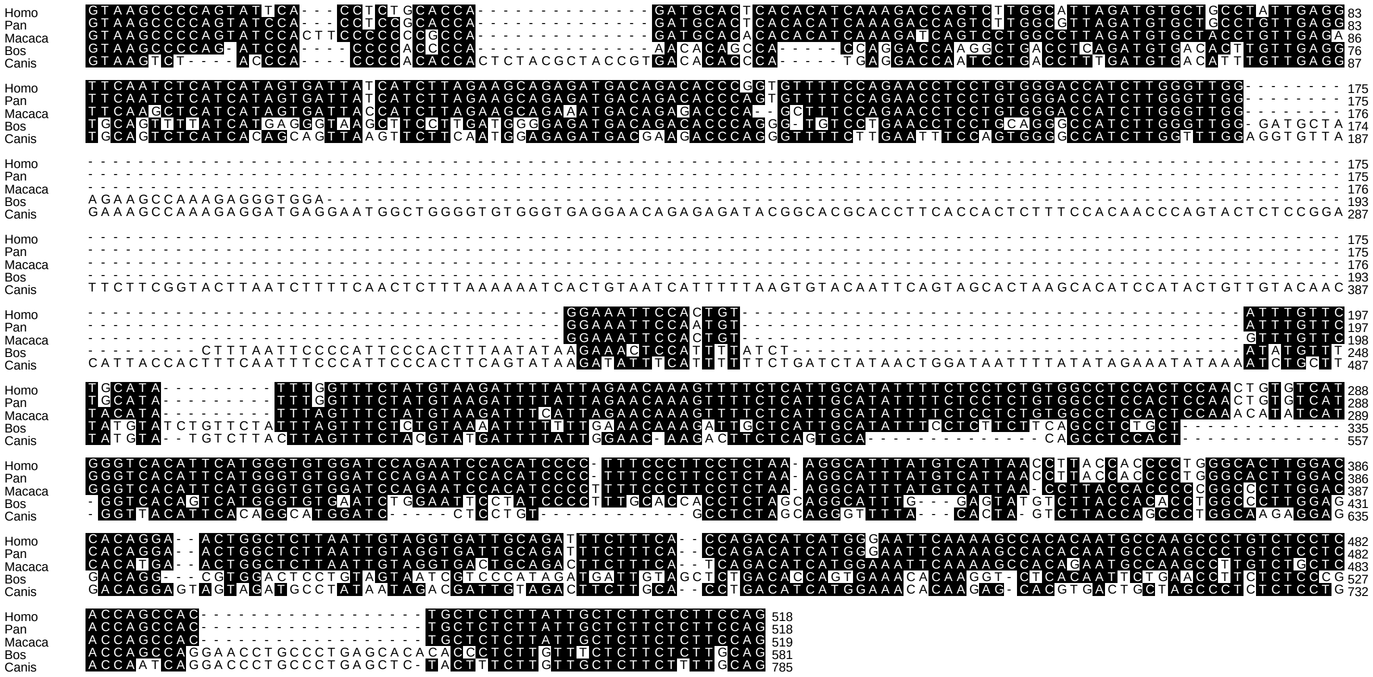

ENSG00000159899 intron 10

Description: Atrial natriuretic peptide receptor B precursor (NPR2)
Intron number: 10
Human chromosome: 09
Intron start (bp): 35792281
Human intron length : 219
Intron alignment length: 241
Flanking exons length (upstream/downstream): 78/105
SNP density: 0.004566
K tree score: 0.0653
Scaling factor: 0.9473
Human-chimpanzee distance: 0.009311
Total primate branch length: 0.0983

ENSG00000159899 exon 10

```
Homo    GGAAATGTTGTCGCCATCAAACATGTGAATAAGAAGCGCATTGAGCTGACCCGGCAGGTTCTGTTTGAACTCAAACAT 78
Pan     GGAAATGTTGTCGCCATCAAACATGTGAATAAGAAGCGCATTGAGCTGACCCGGCAGGTTCTGTTTGAACTCAAACAT 78
Macaca  GGAAATGTTGTCGCCATCAAACACGTGAATAAGAAGCGCATTGAGCTAACCCGGCAGGTTCTGTTCGAACTCAAACAT 78
Bos     GGAAACGTTGTGGCCATCAAGCATGTGAATAAGAAGCGCATCGAGCTGACCCGGCAGGTTCTGTTTGAACTGAAACAT 78
Canis   GGAAATGTTGTCGCCATCAAACACGTGAATAAGAAGCGCATTGAGCTGACCCGGCAGGTTCTGTTTGAACTCAAACAT 78
```

ENSG00000159899 exon 11

```
Homo    ATGAGAGATGTTCAGTTCAACCATCTCACTCGCTTCATTGGCGCCTGCATAGACCCTCCCAACATTTGCATTGTCACTGA 80
Pan     ATGAGAGATGTTCAGTTCAACCATCTCACTCGCTTCATTGGCGCCTGCATAGACCCTCCCAACATTTGCATTGTCACTGA 80
Macaca  ATGAGAGATGTTCAGTTCAACCACCTCACTCGCTTCATTGGCGCCTGCATAGACCCTCCTAACATTTGCATTGTCACTGA 80
Bos     ATGAGAGATGTTCAGTTTAACCATCTCACTCGCTTCATTGGTGCCTGCATAGACCCTCCCAACATTTGCATTGTCACCGA 80
Canis   ATGAGAGATGTTCAGTTCAACCACCTCACGCGCTTCATTGGTGCCTGCATCGACCCTCCCAACATTTGCATTGTCACTGA 80

Homo    ATACTGTCCTCGTGGGAGTTTACAG 105
Pan     ATATTGTCCTCGTGGGAGTTTACAG 105
Macaca  ATATTGTCCTCGTGGGAGTTTACAG 105
Bos     GTATTGTCCTCGTGGGAGTTTACAG 105
Canis   GTATTGTCCTCGTGGGAGTTTACAG 105
```

ENSG00000159899 intron 10

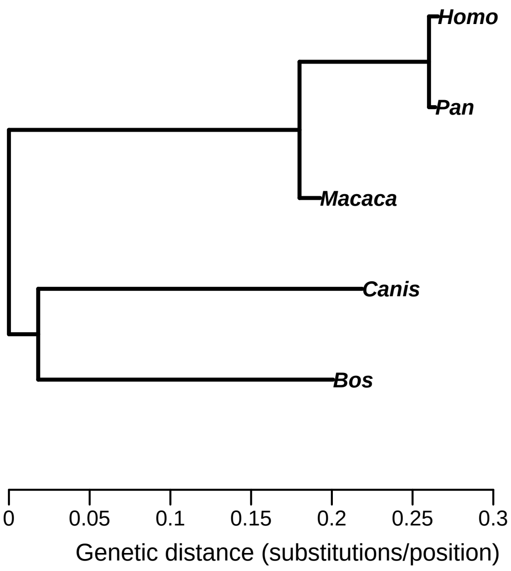

ENSG00000159899 intron 10

```
Homo    GTATGTAACAGAGGATGGACT------------CTAACATGTATGTAATGGAGGAG----TGGGGAAAACTATGAAATAGTAAAATTGGCTAGATGGGCA 84
Pan     GTATGTAACAGAGGATGGACT------------CAAACATGTATGTAATGGAGGAG----TGGGGAAAACTATGAAATAGTAAAATTGGCTAGATGGGCA 84
Macaca  GTATGTAACGGAGGATGGGTTGAGGGTGAGGGGTAAACAGGGATGGGGAAGAGGAG----TGGGGAAAACTACGAAATAGTAAAATTGGCTGGACGGGCA 96
Bos     GTAAGTCCTGGTGTATGGGTTGAGGGCCCAGGATAGACACGGATGTGGAAGGGGAA-----GGAGAGGACTAGGGAATAGTAATACTGGCTGGGAGGGCA 95
Canis   GTATGTAACGGTGTATGAGTTAAGGCTGAGGGGTAGATAGGGATGGAGGAGAGGAAAGGGGGAAGAGGAGAGGGGAATAGTAACCTTGGCTAGGAAGGAA 100

Homo    AGGGGTTGATTTATAAATGATTTTAAAATCGTAGATACAACTAAGAGAAAGGTCCTCTTATGTAGTGGTAAGTTTCTGTATCTAATTTTT-AACTCTTTC 183
Pan     AGGGGTTGATTTATAAATGATTTTAAAATCGTAGATACAACTAAGAGAAAGGTCCTCTTATGTAGTGGTAAGTTTCTGTATCTAATTTTT-AACTCTTTC 183
Macaca  AGGGGTTGATTTATAAATGATTTTAAAATTGTAGATACAACTAAGAGAAAGGTCCTCGTATGTAGTGGTAAGTTTCTGTATCTAATTTTT-AACTCTTCC 195
Bos     AGTGGGTCATTTGTAAATGACTTT-GAGTTGTGAGTACAATTAAGAGAAAGGTCCTCTCCTGTAGTGGTAGATTTCTGTATCTAATTTTT-AGCTCTTTG 193
Canis   AGGGGCTTATTTATAAAGGATTGT-AAGTTGCAGGTACAATTAAGAGTAAGGTCCTGTCATGTAGTGGTATATTTCTGTATCTACTTTTTCATCTCCTTC 199

Homo    AATTTTCTT-----ATCCTTCCCATTGTTTTTTTCTGCCAG 219
Pan     AATTTTCTC-----ATCCTTCCCATTGTTTTTTTCTGCCAG 219
Macaca  AATGTTCTT-----ATCCTTCCCATTGTTTTTTTCTGCCAG 231
Bos     AATTCTCTTT----TCTTCCTTTTTTTTTCCTTTTGGCTAG 230
Canis   AATTCTTTTTCTCCTCTCCCATCTTTGGTTGTTGTTGTTAG 240
```

ENSG00000160613 intron 5

Description: Proprotein convertase subtilisin/kexin type 7 precursor (PCSK7)
Intron number: 5
Human chromosome: 11
Intron start (bp): 116600143
Human intron length : 484
Intron alignment length: 532
Flanking exons length (upstream/downstream): 55/142
SNP density: 0.004132
K tree score: 0.0821
Scaling factor: 0.646
Human-chimpanzee distance: 0.023250
Total primate branch length: 0.0982

ENSG00000160613 exon 5

```
Homo    CTGGGGACCAGATGACGATGGGAAGACAGTGGATGGCCCCCATCAGCTTGGAAAG 55
Pan     CTGGGGACCAGATGACGATGGGAAGACAGTGGATGGCCCCCATCAGCTTGGAAAG 55
Macaca  CTGGGGACCAGATGATGATGGGAAGACAGTGGATGGCCCCCATCAGCTTGGAAAG 55
Bos     CTGGGGACCAGACGATGACGGGAAGACAGTGGATGGACCCCACCAGCTGGGCAAG 55
Canis   CTGGGGCCCAGATGATGATGGGAAGACGGTGGATGGTCCCCATCAGCTTGGAAAG 55
```

ENSG00000160613 exon 6

```
Homo    GCTGCCTTACAACATGGGGTGATTGCTGGTCGCCAGGGCTTTGGGAGCATCTTTGTGGTAGCCAGTGGCAACGGAGGCCA 80
Pan     GCTGCCTTACAACATGGGGTGATTGCTGGTCGCCAGGGCTTTGGGAGCATCTTTGTGGTAGCCAGTGGCAACGGAGGCCA 80
Macaca  GCTGCCCTACAACACGGAGTGATTGCTGGTCGCCAGGGCTTTGGGAGTATCTTTGTGGTAGCCAGTGGCAACGGAGGCCA 80
Bos     GCTGCCTTGCAGCACGGGGTGATCGCTGGCCGCCAGGGCTTTGGGAGCATCTTTGTGGTGGCCAGTGGCAATGGGGGCCA 80
Canis   GCCGCCTTGCAACATGGGGTGATTGCTGGCCGCCGGGGCTTTGGGAGCATCTTCGTGGTGGCCAGTGGCAACGGAGGCCA 80

Homo    ACACAACGACAACTGCAACTACGATGGCTACGCCAACTCCATCTACACCGTCACCATAGGTA 142
Pan     ACACAACGACAACTGCAACTACGATGGCTACGCCAACTCCATCTACACAGTCACCATAGGTA 142
Macaca  ACACAACGACAACTGCAACTACGATGGCTACGCCAACTCCATCTACACGGTCACCATAG--- 139
Bos     GCACAACGACAACTGCAACTACGATGGCTATGCCAACTCCATCTACACTGTCACCATAG--- 139
Canis   GCACAATGACAACTGCAACTATGACGGCTATGCCAACTCCATCTACACTGTCACCATAG--- 139
```

ENSG00000160613 intron 5

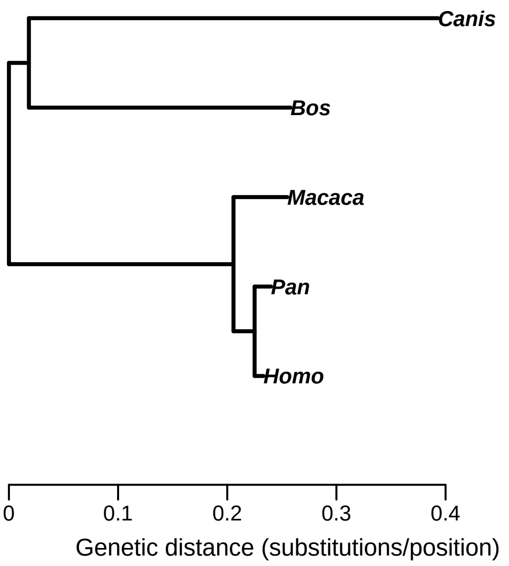

ENSG00000160613 intron 5

```
Homo    GTAACTGGCCTGCACAAAGACTCTGAGAAACGGGAT-GCCCTCTTCAT-ACAGTCCCGGGAGAACTGCTGCAAGTTGACCCATCTTCCCACTCCACCTCC 98
Pan     GTAACTGGCCTGCACAAAGACTCTGAGAAACGGGAC-GCCCTCTTCAT-ACAGTCCCAGGAGAACTGCTGCAAGTTGACCCATCTTCCCACTCCACCTCC 98
Macaca  GTAACTGGCCTGCACGGAGA--CTGAGAGGCAGGAT-GCGCTCTTCAT-TCAGTCCCAGAAGACCTGCTGCAAGCTGAGCCATCTTCCCACTCCACCTCC 96
Bos     GTAGCTGGGAACCGTGGAGACCTTGGGGACCAAGTTCACCCTCGTCATCCCATTCCCGGAAGTGCTCCCG---------CCCTCCCCCAGACCTGCCTTG 91
Canis   GTAACTGAACTGCATGAAGG--TTGAGAG--------GCCTTCTCCATTCGGGTCCTAGAACTGCTCTAAGCCACTCCCTTTCCTACCCTACCCGCCCCC 90

Homo    A----------------------------------CGCTCAGTC--TCCTTTCAGGAAGAGAACACCTAACGTCGGGGCAGGAAGCCTAGGGGACGTTTG 162
Pan     A----------------------------------CGCTCAGTC--TCCTTTCAGGAAGAGAACACCTAACGTCGGGGCAGGAAGCCTAGGGGACGTTTG 162
Macaca  A----------------------------------TGCGCAGTC--TCCTTTCAGGAAGAGAAAACCGAACGTCGGGGCAGGAAGCCTGGGGGACTTTTG 160
Bos     T----------------------------------AGCCCCGACCACTCCTTTGGGAACAGCACTCCTGCCCTGGGGTGAAGGGGTGAGGGGGACATTCT 157
Canis   TCCCCCCCATCTGTGGTGCCAGACTCCTGTCATCCAGCCTTACCACCTCCTTTTGGGCAAGTCCTCATAACCTAGGGGTGGGGAGTGCAGGGGACACTAG 190

Homo    TTCCTAGAGAGGGAAATGGAACAGTGCTTTGAGATTGTTTCCCTTTCATCTTATCCTCAGCTCCCATGCCTGGCCCCACCCTGTCCTCTGATAAGCAGAG 262
Pan     TTCCTAGACAGGGAAATGGAACAGTGCTTTGAGATTGTTTCCCTTTCATCTTATCCTCAGCTCCCATGCCTGGCCCCACCCTGTCCTCTGATAAGCAGAG 262
Macaca  TTCCTAGAGAGGGAAAGGGAACAGTACTTTGAGATTGTCTCCCTTTCATCTTGTCCTCAGCTCCCATGCCCGGCCCCACCCTGTCCTCTGATAAGTGGAG 260
Bos     T-----------AGAATGGAGCAGTGCTTTG-----------CTCTGATTTTGTGCTCAGCTCCGCATTTCTGGCCCACC---TTCTCTGATAAGCAGAG 232
Canis   TTCTTGGA----GAAGTGGAAGGATACCTTGAGCT--GTTCCCTTTGGTTTTGTGCTTGTGCCCCC-----------------TCCCCCCATCACTGGG- 266

Homo    GGTGTCCATCAGTCA-TCATTCAACTCACAGCTGCCCCACATCCTGGGTCAACTCACAGCTC---CCTCACATGCGGGGTCCATCTTCCAA--TCTTTCC 356
Pan     GGTGTCCATCAGTCA-TCATTCAACTCACAGATGCCCCAAATCCTCAGTCAACTCACAGCTC---CCTCACATCCAGGGTCCATCTTCCAA--TCTTTCC 356
Macaca  GGCGTCCATCAGTCA-TCATTCAACTCACAGCTGCCCCACATCCTGGGCCAACTCCTAGCTCCCTCCTCACATCCAGGGTCCATCTTCCAG--TCTTTTC 357
Bos     CACATCCATCAAGCAGTTGTTCCACTAATAGCCCCTAACCTCTTA--------------------------------AGTCCATCTTTAAGTCTCTTTCT 302
Canis   --------------------------------------------------------------------------------------CCCAGGCGATTCCC 280

Homo    CCCATGCCCAGTGCCCTTTGAACTTTTTT-CCCTTTGTTTCTAGAAGGGGTGGAGAGGGTGACTGATGTGGTAGGCGGAGGT---CTTTCTGTGGGTTCC 452
Pan     CCCATGCCCAGTGCCCTTTGAACTTTTTT-CCTTTTGTTTCTAGAAGGGGTGGAGAGGGTGACTGATGTGGTAGGCAGAGGT---CTTTCTGTGGGTTCC 452
Macaca  CCCAT-CCCAGTGCCCTTTGAACTTTTTT-CCTTTTGTTTCTAGAAGGGATGGAGAGGGTGACTGATGTAGTAGGTGGAGGT---CTTTCTGTGGGTTCC 452
Bos     CCCTTGTCCAATGCCCTTGAGG--TTTTT-TGTTTTCCTTCTAGAAGGGGACAGGAGGGCGAGTGGCGTGGTAGGTGG-GGT---CTTCCTGTGAGTTCC 395
Canis   CCTTGCTCCAGTGCCTTTGGGCTTTTTTCCCTTTTCTTTCTAGAAGAGGAGGA----GCTGTTGGTATGGAAAGTAGAGATTCCTTTTCTGGGCGTTCC 376

Homo    TCAACTGACACCCTGATGTTTTGGCTCCTCAG 484
Pan     TCAACTGACACCCTGATGTTTTGGCTCCTCAG 484
Macaca  TCAACTGACACCCTGATGTTTTGGCTCCTCAG 484
Bos     TCAGCTGA---CCTGGGTGTTTTGCTCCTCAG 424
Canis   TTGGCTGA-CCCAGGACATTTTTGCTCTTCAG 407
```

ENSG00000126775 intron 6

Description: KIAA0831 (KIAA0831)
Intron number: 6
Human chromosome: 14
Intron start (bp): 54917162
Human intron length : 1271
Intron alignment length: 2121
Flanking exons length (upstream/downstream): 230/116
SNP density: 0.004721
K tree score: 0.0231
Scaling factor: 0.9524
Human-chimpanzee distance: 0.018283
Total primate branch length: 0.098

ENSG00000126775 exon 6

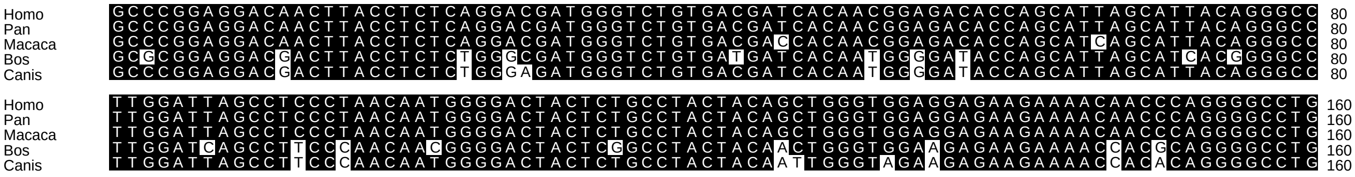

ENSG00000126775 exon 7

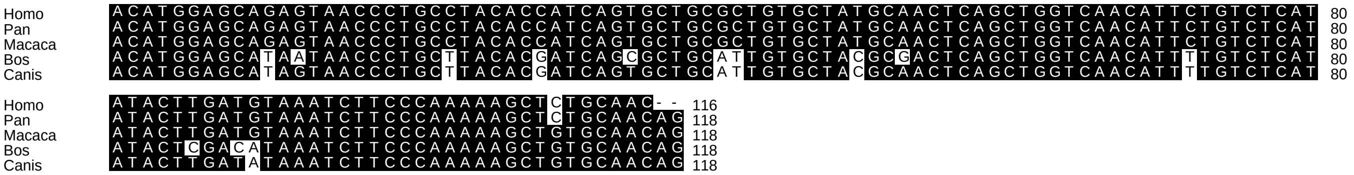

ENSG00000126775 intron 6

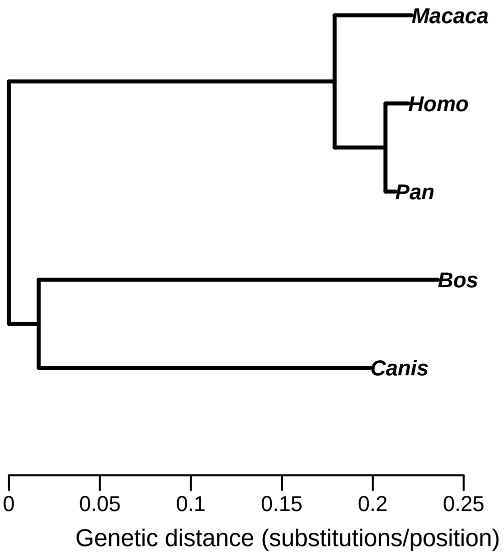

ENSG00000126775 intron 6

```
Homo    GTGAGAAGCAGCATATCCTTTGAAATCTATATATAGAGAGAGTTAT----------AGGTAAAATTGACATAACAAAATGAAGAGCTTTCAAGTGAACCA 90
Pan     GTGAGAAGCAGCATATCCTTTGAAATCTATATATAGAGAGAGTTAT----------AGGTAAAATTGACGTAACAAAATGAACAGCTTTCAAGTGAACAA 90
Macaca  GTGAGAAGCAGCATATCCTTTGAAATGTATAGAGAGAGATAGTTAT----------AGGTAAAATTGACATAACAAAATGAATAACTTTCAAGTGAACAA 90
Bos     GTGAGAAGCAAGATAGTGTTCGAAAACTTTTATAAATTTTTAATTG----------AGGTGAAACTGATGTAACAAAATGAACAGTT----AGAGAGTCA 86
Canis   GTGAGAATCAAAATAGTCTTTGAAAACTCTTAATTATGTGTTTTTTTTTTAAATTGAGGTAAAATTTACATAACAGGATG-ACAGTTTTAAAGTGAACAG 99

Homo    TTCAGGGGCATTTAGTACATTCACAGCACTGTGCTCCCATCACCTCCATGTCATTTCAAAACATTTTGATCACCCCAGACAGAAACCCCATGGCATGAAA 190
Pan     TTCAGGGGCATTTAGTACATTCACAGCACTGTGCTCCCATCACCTCCATGTCATTTCAAAACATTTTGATCACCCCAGACAGAAACCCCATGGCATGAAA 190
Macaca  TTCAGGGGCATTTAGTACATTCACAGCACTGTGCTCCCATCACCTCTGTGTTGTTTCAAAACATTTTGATCATCCCAGACAGAAACCCCATGGCATGAAA 190
Bos     GTGAGCGGGA-CTAGTACATTCACAGTGTTGTGCAGCCATCACCTTGTCAAGATCAAAAGCACATTTTCATCCCAGACAAAAACTCTGGGGCTCGAAT 185
Canis   TTTAGTGGCA-TTAATAGATTGACAGTGTTGTGCAGTGACCACCTCTGTTGAATTCCAGAGCTCCTGCATCACCCCAAACGGGAACTCCAGGGCACAAAG 198

Homo    GTGTCACATTTCCCCACTCTCCCTTCCCCTCAGCTCCTGGCAACCAC----------------------------------------------------- 237
Pan     GTGTCACATTTCCCCACTCTCCCTTCCCCTCAGCTCCTGGCAACCAC----------------------------------------------------- 237
Macaca  GTGTCACATTCCCCCACTCTCCCTTCCCCTCAGCTCCTGGCAGCCAC----------------------------------------------------- 237
Bos     CAGTCA---------------------------------------------------------------------------------------------- 191
Canis   ATGTCA---TTCCCCTGTCCCCTCTCCTCCCATGTCATGGCATCCCCTACAGATGATCCCAACTTGTGATTGTTCAACTTAAAATTTTTTGACTTTATGA 295

Homo    ---------------------------------------------------------------------------------------------------- 237
Pan     ---------------------------------------------------------------------------------------------------- 237
Macaca  ---------------------------------------------------------------------------------------------------- 237
Bos     ---------------------------------------------------------------------------------------------------- 191
Canis   TGATACAAAAGCAGTACACATTCAGTAGAAACCATGCTTTGAATTTTGAATTTGCATCTTTTCCTGGGCTAGTGGTCTGCCATGCATACTCTCAGGGTGC 395

Homo    ---------------------------------------------------------------------------------------------------- 237
Pan     ---------------------------------------------------------------------------------------------------- 237
Macaca  ---------------------------------------------------------------------------------------------------- 237
Bos     ---------------------------------------------------------------------------------------------------- 191
Canis   TGGGCACTGTGGCTCCTAGGCAGCTCCGTGGTCCTGAGGGTCCACAGCCTGTAGATATACTGATAACTGTTCTGTGTTTCACTTTCAGTATAGTATTCAA 495

Homo    ---------------------------------------------------------------------------------------------------- 237
Pan     ---------------------------------------------------------------------------------------------------- 237
Macaca  ---------------------------------------------------------------------------------------------------- 237
Bos     ---------------------------------------------------------------------------------------------------- 191
Canis   GACGTGGCATGAGGTATTCAACACTTTATTATAAATAGGCTTTGCATTAGATGATTTTGCCCGACTGTAGGCTAATGTCAGTGTTCTGGGCCTGTGTAAG 595

Homo    ---------------------------------------------------------------------------------------------------- 237
Pan     ---------------------------------------------------------------------------------------------------- 237
Macaca  ---------------------------------------------------------------------------------------------------- 237
Bos     ---------------------------------------------------------------------------------------------------- 191
Canis   GCAGGTCAGGCTAAACTTTGGTGTTTAGTTGTTTAGGTGCATTAAATGTATTTTTCACTTAGGATATTTCCAGTTTATGATGAGTTTATCATGACATAAC 695

Homo    ---------------------------ACATTGCTTTCTGTGTCTATAGATTTGCCTGTTCTGGGCAGCTTATATAAATGGAATCACAGAACATATAGCT 310
Pan     ---------------------------ACATTGCTTTCTGTGTCTATAGATTTGCCTGTTCTGGGCAACTTATATAAATGGAATCACAGAATATATAGCT 310
Macaca  ---------------------------ACATCGCTTTCTGTGTCTGTAGATTTGCCTGTTCTGGGCAACTTATATAAATGGAATCACAGAATATATAGCT 310
Bos     ---------------------------CTCCTGCTTTCTGTGTCTCTATGGATG----CTTTCTGTTTGTTTCATAGAAATGGGATCATACAACATGTGGCC 260
Canis   CCCATCGTACATTGAGGAGGATCTGTAATTTTGTTTCTGTAACTATAGATGTGTCTATTTTGGATATTTTATAGAAATGGAATCATACAATGCGTGGCC 795

Homo    TTTTGTGTCAGACTTCTTTCACTTAGGGTAATGTTTTCAAGATTCATCCACATGATAGTATATATTAGTGTTTCATTCGTTTTTATGGTTGAGTAATATT 410
Pan     TTTTGTGTCAGACTTCTTTCACTTAGCATAATGTTTTCAAGATTCATCCACATGATAGTATATATTAGTGTTTCATTCGTTTTTATGGTTGAGTAATATT 410
Macaca  TTTTGTGTCAGACTTCTTTCGCTTAGCATAATGTTTTCAAGGTTCATCCGCATGA---TATGTA-TAGTGTTTCATTCCTTTTCATGGTTGAATAATATT 406
Bos     TTTTGTGTCAGGCTTTTTTCACGTAGTGTATGGTTTCAAGGTTCATCTACATTGTGGCATATA----CATTTCATTCCTTTT-ATGACTGAATAATACT 355
Canis   TTTTGTGTCTGGCTTTTTTACATAGCATGATG--TTCAAGGTTCATCCATATTGTAGCATGAATTAGCATTTCATTCTTTTT----ATGATTAATATT 888

Homo    CCATC-------ATATGGATAAGACACATTTTATTTATTCATTCATCACTTGATGGACTGAAAACA-----------------TTTTTTAGATGTCTCAC 486
Pan     CCATC-------ATATGGATAAGCCACAT----TTGTTTATTCATTCATCACTTGATGGACTGAAAACA-----------------TTTTTTAGATGTCTCAC 482
Macaca  CCATT-------GTGTGGATAAGCCACAT----GTTGTTTATTCATTCATCACTTGATGGACTGAAAACA-----------------TTTTTTAGATGTCTCAT 478
Bos     CCTTT-------GTGTGGCTATGCCACAT-------TTGTTAATTGATGAATGGACTGAAAACA-----------------TTTTTTATATATTTCAC 440
Canis   TCATTGTATGTAATGTGGGTATATTGCGTTTT-ATATTCAATCCATCAGTGGATTTACTGAAAACATTTTTTTTTTCCGGTATTTTTTTAGATATTTTAC 970

Homo    CTTTTGGGGGTTAATATTGTC------------------------------------------------------------------------------- 507
Pan     CTTTTGAGGGTTAATATTGTC------------------------------------------------------------------------------- 503
Macaca  CTTTTGGGGGTTAATATTGCC------------------------------------------------------------------------------- 499
Bos     CTTTTGGGGTCAAATGTTACTTACATCAGAGAAGGCAATGGCACCCCACTCCACTACTCTTGCCTGGAAAATCCATGGGTGGAGGAGCCTGGTAGGCTGC 540
Canis   TTTTGGGGAGCCAATATTGCC------------------------------------------------------------------------------- 991

Homo    ---------------------------------------------------------------------------------------------------- 507
Pan     ---------------------------------------------------------------------------------------------------- 503
Macaca  ---------------------------------------------------------------------------------------------------- 499
Bos     AGTCCATGGGGTCGCTAAGGGTTGGACACGACTGAGCGACTTCACTTTCATGCATTGGAGAAGGAAATGGCAACCCACTCCAGTGTTCTTGCCTGGAGAA 640
Canis   ---------------------------------------------------------------------------------------------------- 991

Homo    ---------------------------------------------------------------------------------------------------- 507
Pan     ---------------------------------------------------------------------------------------------------- 503
Macaca  ---------------------------------------------------------------------------------------------------- 499
Bos     TCCCAGGGACGGGGGAGCCTGGTGAACTGCTGTCTATGGGGTCACACAGAGTTGGACACAACTGAAGTGACTTAGTAGTAGTAGTAGTAGTAGTAGTAGT 740
Canis   ---------------------------------------------------------------------------------------------------- 991

Homo    --TTGTTTATGTAAGAGGA---CGTTATTTAAAGAAGTATATTTAACCCGTTTAGACCCATATTTATAGTGTATAACTTTGTAA-----------TGTGT 591
Pan     --TTGTTTATATAAGAGGA---CATTATTTAAAGAAGTATATTTAACCCGTTTAGACCCATATTTATAGTGTATAACTTTGTAA-----------TGTGT 587
Macaca  --TTGTTTATGCAAGAAGA---CATTATTTAAAGAAGTATATTTCACCTGTTGAGACCCGTATTTTTAGAGTATAACTTTGTAA-----------CGTGT 583
Bos     AGTAACTTATATAAAAGATCTCACTATTTAAAGAAGTGTATTTAGCCTGTTGGGTCCCATTTTTATAGCTTCTAACTTTGTAAATAACTTTTTTTTTTT 840
Canis   --TTATTTATATAAAAGT---CCTCAGT--AGTTAGTATATATAATCTGGTTGAGTCCTATTTTTATAGTTTGTAA----AGAG-----------CTTGT 1069

Homo    TTTAGTAGTCCTAAATGGAGACTGAAAGGCCCCAATGGGAAAACCCCTTAAAACCCCTGCAGTTTCTCTTTGGTGGAGAAAGATTCTTTGTTCATTTCGT 691
Pan     TTTAGTAGTCCTAAATGGAGATTGAAAGGCCCCAATGGGAAAACCCCTTAAAACCCCTGCAGTTTCTCTTTGGTGGAGAAAGA----TTGTTCATTTCGT 683
Macaca  TTTAGTAGTCCTAAATGGAGATTGAAAGGCCCCGATGGAAAAACCCTTTAAGACCCGCAGTTTCTCTTTGGTGGAGAAAGA----TTGTTCATTTAGT 679
Bos     TTTACCAGCCCTAAGTGGAGATTGAAATACCCCAGTAGAAAAACCCCTTAAATGCCAGTAGCTTCTCTTTGGTGGAGAAAGA----TGATGCATTAGT 936
Canis   TTTAGCAGCCCTAAATGGAG--------------ATAGAAAAACCCTATAAAACCCCTATAGCTT-TCTTTGGTGGAGAAAAA-----ATTGTTTAAGT 1148

Homo    CACTCAGTAAATATTTAGTGAGCTCATGCTTTGTGTCATGCACATTGCTGGGTGCTGGAGATACAAAGGTG-----ACCAAGACAGAGGT---------- 776
Pan     CACTCAGTAAATACTTAGTGAGCTCATGCTTTGTGTCATGCACATTGCTGGGTGCTGGAGATATAAAGGTG-----ACCAAGACAGAGGT---------- 768
Macaca  CACTCAGTAATATTTAGTGAGCTCATGCTTTGTGTTACGCACATTGCCGGGTGCTGGAGATAAAAAGGTG-----ACCGAGACAGATGT---------- 764
Bos     TATCCAGCCAGTATTTAATGAG---TGCTTTCTGTTATGCACGCTGCTGG-------TGATAC-AAGGCGATGTCACCAAGGCATGGGTTAGATCCCTG 1024
Canis   CATTTTCCAAATACACAGTGAGTATCTGCACTCTGTCATGCACAGTGCTGGGTACTGATGCTACAAAGGTGATGTCACCAAGACAGGCGT---------- 1238

Homo    ---GGCCCTTTGCCCTTCTCAGGGGAGAAAGGTAGGAAGCAAGTAAATGATCGAGATGCTGTACATTGAGTGGGTGCCATGGAAGGAACTAGGGGAGCAT 873
Pan     ---GGCCCTTTGCCCTTCTCAGGGGAGAAAGGTAGGAAGCAAGTAAATGATCGAGATGCTGTACATTGAGTGGGTGCCGTGGAAGGAACTAGGGGAGCAT 865
Macaca  ---GGCCCTTTGCCCTTCTCAGGGGAGAAAGGTAGGAAGCAAGTAAATGATCAAGGTGCTGTCCACTGAGTGGGTGCCGTGGAAGGAACTAGGGGAGCAT 861
Bos     ACCAGCCCTTTACCTCTCTGTGGAGAGGAAAGGAAGCAAC----TAATGGTCAAAGGCATTACAACTGAGTGTGGCTGTAGGCAGAACCAGCGA----- 1115
Canis   ---AGCC--------------------------------------------------------------------------------------------- 1242

Homo    CCTGGGGCAGGATGGGGTACAGAGGAAGGGATGACTCTTCCGTAGGGTTGTCAGGAGAGCCCTCTCTGGAGAGGTGACATTCAAGTTGGGCATTTATTGT 973
Pan     CCTGGGGCAGGATGGGGTACAGAGGAAGGGATGACTCTTCCGTAGGGTTGTCAGGAGAGCCCTCTCTGGGGAGGTGACATTCAAGTTGGGCATTTATTGT 965
Macaca  CCTTGGGTGGGATGGGGTACAGAGGAAGGGATGACTCTTCCGTAGGGTTGTCAGGAGAGCCCTTTCTGGGGAGGTGCCATTCGAGTTGGGCCTTTATTGT 961
Bos     -CTTCATGGGGTGGGGCTACAGAGGAAGGAACTATTTCTCTGTAGGGTAGTCAGGGGAGGCTTCTCTGAGGAGGTGACTTTAAGTTGGTCATTAATTGC 1214
Canis   -----------------------------------------------CAGGTGAGACCTCTCTGAGGAGGTGACATTGAAGTTAGGCATTTGCTGT 1291

Homo    TTTG----ATATTACGTTATCAGGTTCACACAGGTCAGACTTTAATTTCATTTATGTTGGGTCTATTTA-GGCCCATTTGCTC-TGTTACATTGCTCTCA 1067
Pan     TTTG----ATATTACGTTATCAGGTTCACACAGGTCAGACTTTAATTTCATTTATGTTGGGTCTATTTA-GGCCCATTTGCTC-TGTTACATTGCTCTCA 1059
Macaca  TTTG----ACATTACATTATCAGGTTCATGCAGGTCAGACTTTAATTTCATTTATGCTGGGTCTATTTAGGGCCCATTTGCCC-TGTTACATTGCTCTCA 1056
Bos     ATAA----ATAATGTGTTATC-----CAAGCAGTTCAGATTTCAATTTCATTTATGCTGGGTCTGCTTA-GACCCATTTGCTTAAGTTATATTCCACTC- 1303
Canis   TCTGATAAATAATACATTATCAGGTCCAAGTGGTTCTGACTTTCATTTCATTTATGTTGGGTCTGTCTA-GGCCCATTTGCTTAGGTTACATTAC----- 1385

Homo    GCAAAAAAAAAAAAAAAAATATATATATATATATATATACACACAAAGAATGGACCATTTATGTCTGGTCATGGAGCATCTATTA-------AGGAAGCA 1160
Pan     GCAAAAAAAAAAAAAAAA----ATATATATATATATATACACACAAATAATGGACCATTTATGTCTGGTCATGGAGCATCTATTA-------AGTAAGCA 1148
Macaca  GAAAAAAAAAAAAAAAAA------------------AAAAATACACAAATAAGGGACTATTTATGTCTGGTCATGGAGCATCTATTG-------AGTAAGCA 1132
Bos     ---------ATCAAAAA---------------------TTATAAATAATGAAATATTAATATCTGGCCATGGACTTTTATT--------AGTAAAGA 1363
Canis   ---------AACAAAAAA-------------------CCATAAACAATGAAACATTAATGTGTGGCTATGAACATTTTATTAGTATATTAGTAAGGA 1454

Homo    CTAAAACGTAA----CAGTGCTATCATGATAAAATCATAAATGATA-AACTGGTGGATCATTAGTA-GTTTTGTTGGAATTAAGGTTTTGTACA--TGGT 1252
Pan     CTAAAACGTAA----CAGTGCTATCATGATAAAATCATAAATGATA-AACTGGTGGATCATTAGTA-GTTTTGTTGGAATTAAGGTTTTGTACA--TGGT 1240
Macaca  CTAAAACGTAA----CAGTGCTATCGTGATAAAATCATAAATGATA-AACTCTTGGCTCATTAGTA-GTTTTGCTGGAATTAAGGTTTTGTACG--TGGT 1224
Bos     GTAAAGCTTAGCTGCCAGTAATGTATTGATAAAACCACACATCCCAGAAATCTAGGTTCTTTAATG-TTTTTGTTGAAATTGAAGTTTTTGTGAATGGC 1462
Canis   GCAAAGCATAACCATCAATAATGTGATGATAAAATTATAAATCATA-AAATACAAGTTCATTGGTATTTTTTGTTGAAATTAAAGTTGTTTGTG--T-AT 1550

Homo    GGTTGTTTTCTTAC--TGCAG 1271
Pan     GGTTGTTTTCTTAC--TGCAG 1259
Macaca  GGTTGTTTTCCTGC--TGCAG 1243
Bos     TGTTGTTTATCTACTTTTTAG 1483
Canis   GGTTGTTTTTATAC--TTTAG 1569
```

ENSG00000111678 intron 1

Description: Putative C10 protein (C12orf57)
Intron number: 1
Human chromosome: 12
Intron start (bp): 6923598
Human intron length : 302
Intron alignment length: 347
Flanking exons length (upstream/downstream): 52/177
SNP density: 0.003311
K tree score: 0.0918
Scaling factor: 0.5361
Human-chimpanzee distance: 0.026254
Total primate branch length: 0.098

ENSG00000111678 exon 1

```
Homo    ATGGCGTCCGCCTCGACCCAACCGGCGGCCTTGAGCGCTGAGCAAGCAAAGG  52
Pan     ATGGCGTCCGCCTCGACCCAACCGGCGGCCCTGAGCGCTGAGCAAGCGAAGG  52
Macaca  ATGGCGTCCGCCGCGTCCCAACCCGCGGCCCTCAGCGCTGAGCAGGCAAAGG  52
Bos     ATGGCGTCCGCCTCGGCTCAGTCCGCGGCCCTGAGCGCCGAGCAGGCCAAGG  52
Canis   ATGGCGTCCGCCCCGGCCCCGCCCGCGGCCCTGAGCGCCGAACAAGCCAAGG  52
```

ENSG00000111678 exon 2

```
Homo    TGGTCCTCGCGGAGGTGATCCAGGCGTTCTCCGCCCCGGAGAATGCAGTGCGCATGGACGAGGCTCGGGATAACGCCTGC  80
Pan     TGGTCCTGGCGGAGGTGATCCAGGCGTTCTCCGCCCCGGAGAACGCAGTGCGCATGGACGAGGCTCGGGATAACGCCTGC  80
Macaca  TGGTCCTGGCGGAGGTGATCCAGGCGTTCTCCGCCCCTGAGAATGCAGTGCGCATGGACGAGGCTCGGGATAACGCGTGC  80
Bos     TGGTCCTGGCCGAGGTGATCCAGGCGTTCTCGGCGCCGGAGAATGCTGTTCGCATGGACGAGGCTCGGGACAACGCGTGT  80
Canis   TGGTCCTGGCCGAGGTAATCCAGGCGTTCTCGGCCCCGGAGAACGCCGTGCGCATGGACGAGGCTCGGGACAACGCCTGC  80

Homo    AACGACATGGGTAAGATGCTGCAATTCGTGCTGCCCGTGGCCACGCAGATCCAGCAGGAGGTTATCAAAGCCTATGGCTT  160
Pan     AACGACATGGGTAAGATGCTGCAATTCGTGCTGCCCGTGGCCACGCAGATCCAGCAGGAGGTTATCAAAGCCTATGGCTT  160
Macaca  AACGACATGGGTAAGATGCTGCAATTCGTGCTGCCCGTGGCCACGCAGATCCAGCAGGAGGTTATCAAAGCCTATGGCTT  160
Bos     AACGACATGGGCAAAATGCTGCAATTCGTGCTGCCCGTGGCCACGCAGATTCAGCAGGAGGTTATTAAAGCCTACGGCTT  160
Canis   AACGACATGGGCAAGATGCTGCAGTTCGTGCTGCCCGTGGCCACGCAGATCCAGCAGGAGGTTATCAAGGCCTATGGCTT  160
```

ENSG00000111678 intron 1

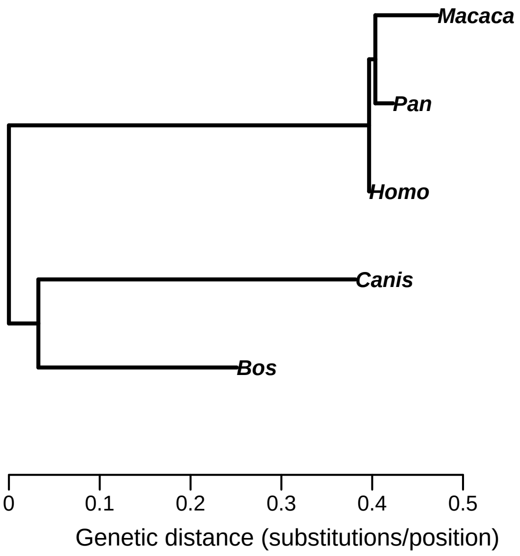

ENSG00000111678 intron 1

```
Homo    GTGAGAATCGTCCTAGTCAAGGCATAGGCTGCTGGCCTGGGGTAGTCAAGGCATGGGCTGCTGGTCTGGGGAGGATGCGGGCGGAGGATGTGGGGCGACA 100
Pan     GTGAGAATCATCC-----------------------------TAGTCAAGGCATGGGCTGCTGGTCTGGGGAGGATGCGGGCGGAGGATGTGGGGCGACA 71
Macaca  GTGAGAATAATCG-----------------------------TAGTCAACGCATGGGTTGCCGGTCCGGGGAGGATGCGGGCAGAGGATGTGGGGCGACA 71
Bos     GTGAGAAGCCTCC-----------------------------CATTTGCCGGGTGGGCTCCTTTCTCGAGAAGGGCGC-------------TGGGCGAGA 58
Canis   GTGAGGAGCGTTC-----------------------------TAGCCGCCCGGCGGGCTCCCTGCTGGAGAAGGGTGC-------------GGGGGGAGG 58

Homo    AACCTGGCTACGTCCGCCGGGAAAATGGGGTAGGGGACGCCGGGTACCGT------------------CCTTCTAAGTGGGG--CGCTTGCCCCC----- 175
Pan     AACCTGGCTATGTCCGCCGGGAAAATGGGGTAGGAGACGCCGGGTACCGTAGGGGACGCCGGGTACCGTCATTTAAGTGGGG--CGCTTGCCCCC----- 164
Macaca  AACCTGGCTTCGTCCGCCGGGAAAACAGGGTAGGGGACGCCGGGTCCCGT------------------CGTTCTAAGTGGGG--CGCTTGCCCCC----- 146
Bos     GGTGTGACTGCATTCGCTGGGAAGTGGGGGCGCG------CGGGGACCAT----------------CCTCTTTTTAGAGGGGCCTCTTTGCCCTCAGCGA 136
Canis   TACAGAGC----------GTGGGGTGTGGGTGCG------CCGGTACCCC----------------CCTCCTCCAGGAG-----CCCTGGCCCCC----- 116

Homo    -----AAGACTTGGGATCTTATTGGGTTACCTACAGCCTCAATCCACTAATTCCTTGCGCTCTCCGCTGGGCCCGCTGTCTGTTCTCCGACGCCT----- 265
Pan     -----AAGACTTGGGATCTTATTGGGTTACCTACAGCCTCAATCCACTAATTCCTTGCGCTCTCCGCTGGGCCCGCTGTCCGTTCTCCGACGCCT----- 254
Macaca  -----AAGACTCGGGATCTTATTGGGTTACCTATAGCCTCAATCCACTAATTCCCTGCGCTCTCCACTGGGCCCGCTCTCCGTTCTCCGACGCCT----- 236
Bos     GGAATTCGGGGGCCGAATTTATCGGGTTA-TTACGGCTTCAGTTCACTAATCCGCTGCCCTTTTCCTTGCGCTGTCGCTCGCGTCGGCCGGACTCGGATT 235
Canis   ----TGGAGCGACGGAGTTTATCGGGCTG-CTATGCCTGCAGTCCACTAATTGCCCGCCCTCTTCCCTGCGTTGCCGGCCGGGCCCGCCTGCCCT----- 206

Homo    ---------ACCCGGGACGCCTCCCTGGGATGCTTCTGG-CGCGCAG 302
Pan     ---------ACCCGGGACGCCTCCCTGGGATGCTTCTGG-CGCGCAG 291
Macaca  ---------ACCCGGGACGCCTCCCTGGGATGCTTCTGA-CGCGCAG 273
Bos     CCCACTCGCGGCGGCGACTCCTCCCTGGGCTGCATCCG--TGCACAG 280
Canis   ----------GCTCCGCGCCTACCCGGGGACGCGTCTGATTTCGCAG 243
```

ENSG00000105825 intron 2

Description: Tissue factor pathway inhibitor 2 precursor (TFPI2)
Intron number: 2
Human chromosome: 07
Intron start (bp): 93356472
Human intron length : 913
Intron alignment length: 1443
Flanking exons length (upstream/downstream): 183/189
SNP density: 0.000000
K tree score: 0.0614
Scaling factor: 0.9153
Human-chimpanzee distance: 0.010322
Total primate branch length: 0.0978

ENSG00000105825 exon 2

```
Homo    CTGCCCCTAGACTACGGACCCTGCCGGGCCCTACTTCTCCGTTACTACTACGACAGGTACACGCAGAGCTGCCGCCAGTT  80
Pan     CTGCCCCTAGACTACGGACCCTGCCGGGCCCTACTTCTCCGTTACTACTACGACAGGTACACGCAGAGCTGCCGCCAGTT  80
Macaca  CTGCCCCTAGACTACGGACCCTGCCGGGCCCTACTTCCCCGTTACTACTACGACAGGTACACGCAGAGATGCCGCCAGTT  80
Bos     CTGCCCCGGACGACGGGCCCTGCCGGGCGCGGATCCCCAGTTACTACTACGACAGGTACACGCAGAGCTGCCGCGAGTT  80
Canis   CTGCCGCCCGACGTGGGGCCCTGCCGGGCGCTGATCCCCAGTTACTACTACGACAGGTACACGCAGAGCTGCCTCCCCTT  80

Homo    CCTGTACGGGGGCTGCGAGGGCAACGCCAACAATTTCTACACCTGGGAGGCTTGCGACGATGCTTGCTGGAGGATAGAAA  160
Pan     CCTGTACGGGGGCTGCGAGGGCAACGCCAACAATTTCTACACCTGGGAGGCCTGCGACGAGGCTTGCTGGCGGATAGAAA  160
Macaca  CTTGTACGGGGGCTGCGAGGGCAACGCCAACAATTTCTACACCTGGGAGGCCTGCGACGAGGCTTGCTGGCGGATAGAAA  160
Bos     CATGTACGGAGGCTGCGAGGGCAATGCCAACAATTTTGAAACTTTGGAGGCCTGCAACGAAGCGTGCTGGAAGATTGAGA  160
Canis   CACCTATGGGGGCTGCCGGGGCAACGCCAACAACTTCGAAACTTGGGAGGCCTGCGACGAGGCTTGCTGGAGGATTGAGA  160
```

ENSG00000105825 exon 3

```
Homo    AAGTTCCCAAAGTTTGCCGGCTGCAAGTGAGTGTGGACGACCAGTGTGAGGGGTCCACAGAAAAGTATTTCTTTAATCTA  80
Pan     AAGTTCCCAAAGTTTGCCGGCTGCAAGTGAGTGTGGACGACCAGTGTGAGGGGTCCACAGAAAAGTATTTCTTTAATCTA  80
Macaca  AAGTTCCCAAAGTTTGCCGGCTGCAAGTGAGCGTGGACGACCAGTGTGAGGGGTCCACAGAAAAGTATTTCTTTAATCTA  80
Bos     AAGTTCCCAAAATTTGCCGGTTAAAAGTGAAT---AAGAAGCAGTGTGGGGAGCTCAGAGAACAGTATTTCTTCAATCTA  77
Canis   AAGTTCCCAAAATTTGCAGGTTGGAAGTCAAT---GAGGAGAAGTGTGGAGAGAACAAAGAGGAGTATTTCTTCAATCTA  77

Homo    AGTTCCATGACATGTGAAAAAATTCTTTTCCGGTGGGTGTCACCGGAACCGGATTGAGAACAGGTTTCCAGATGAAGCTAC  160
Pan     AGTTCCATGACATGTGAAAAAATTCTTTTCCGGTGGGTGTCACCGGAACCGGATTGAGAACAGGTTTCCAGATGAAGCTAC  160
Macaca  AGTTCCATGACATGCGAAAAAATTCTCATCTGGTGGGTGTCACCGGAACTGGATTGAGAACAGGTTTCCAGATGAAGTTAC  160
Bos     AGTTCTATGACATGTAAAAAGTTCATATCTGGCGGGTGTCACAGCAA------TGAGAACCGGTTCCCGGATGAAGCTAC  151
Canis   ACATCCATGACATGTGAAAAAATTCCTATCTGGCGGTTGTCACAGCAA------TGAGAACAGGTTCCCGGATGAGGCTGC  151
```

ENSG00000105825 intron 2

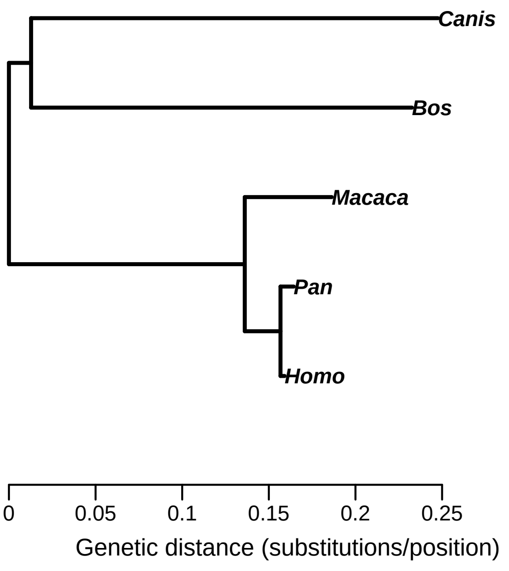

ENSG00000105825 intron 2

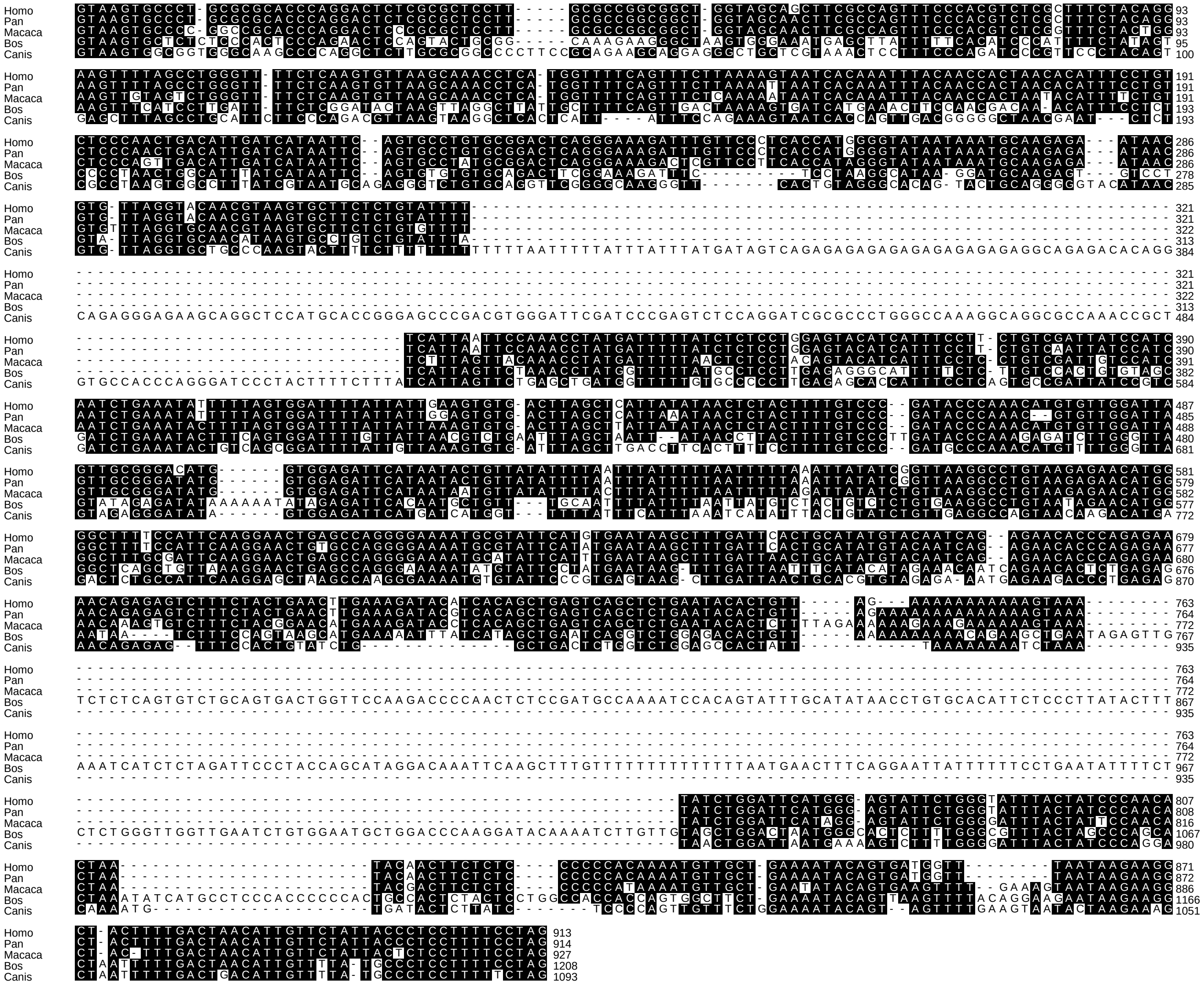

ENSG00000160917 intron 5

Description: Cleavage and polyadenylation specificity factor 30 kDa subunit (CPSF4)
Intron number: 5
Human chromosome: 07
Intron start (bp): 98886355
Human intron length : 1572
Intron alignment length: 1867
Flanking exons length (upstream/downstream): 94/73
SNP density: 0.000000
K tree score: 0.0607
Scaling factor: 0.6242
Human-chimpanzee distance: 0.013746
Total primate branch length: 0.0969

ENSG00000160917 exon 5

```
Homo    GTCCCCTCTGCAGGCACCGGCACACACGGAGAGTCATCTGTGTGAATTACCTCGTGGGATTCTGCCCGGAGGGGCCCTCG 80
Pan     GTCCCCTCTGCAGGCACCGGCACACACGGAGAGTCATCTGTGTGAATTACCTCGTGGGATTCTGCCCAGAGGGGCCCTCG 80
Macaca  GTCCCCTCTGCAGGCACCGGCACACACGGAGAGTCATCTGTGTGAATTACCTCGTGGGATTCTGCCCAGAGGGGCCCTCA 80
Bos     GCCCCCTGTGCAGGCACCGGCACACGCGGAGGGTCATCTGTGTCAATTACCTCGTGGGATTCTGCCCGGAGGGGCCCTCG 80
Canis   GTCCCCTGTGCAGGCACCGACATACTCGAAGAGTCATCTGTGTGAATTACCTCGTGGGATTCTGCCCGGAGGGGCCCTCG 80

Homo    TGTAAATTCATGCA 94
Pan     TGTAAATTCATGCA 94
Macaca  TGTAAATTCATGCA 94
Bos     TGTAAATTCATGCA 94
Canis   TGTAAATTCATGCA 94
```

ENSG00000160917 exon 6

```
Homo    CCCTCGATTTGAACTGCCCATGGGAACCACCGAGCAGCCCCCACTGCCGCAGCAGACACAGCCTCCAGCAAAG 73
Pan     CCCTCGATTTGAACTGCCCATGGGAACCACCGAGCAGCCCCCACTGCCGCAGCAGACACAGCCTCCAGCAAAG 73
Macaca  CCCTCGATTTGAACTGCCCATGGGAACCACCGAGCAGCCCCCACTGCCGCAGCAGACCCAGCCTCCGGCAAAG 73
Bos     CCCTCGATTTGAACTGCCGATGGGAACCACCGAGCAGCCCCCACTGCCGCAGCAGACGCAGCCACCCACGAAG 73
Canis   CCCTCGATTTGAACTGCCAATGGGAACCACCGAGCAGCCCCCACTGCCACAGCAGACGCAACCTGCAACAAAG 73
```

ENSG00000160917 intron 5

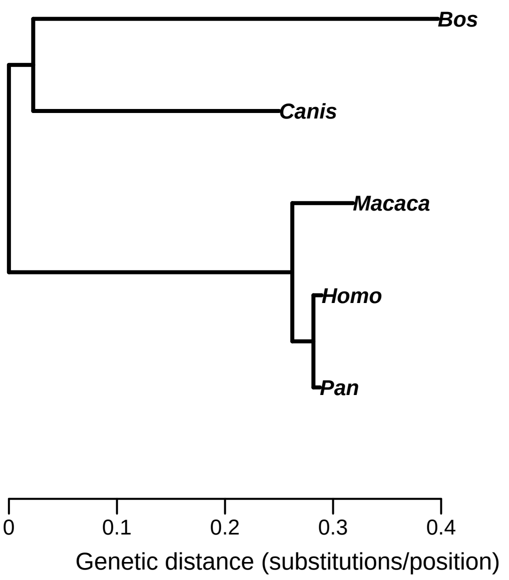

ENSG00000160917 intron 5

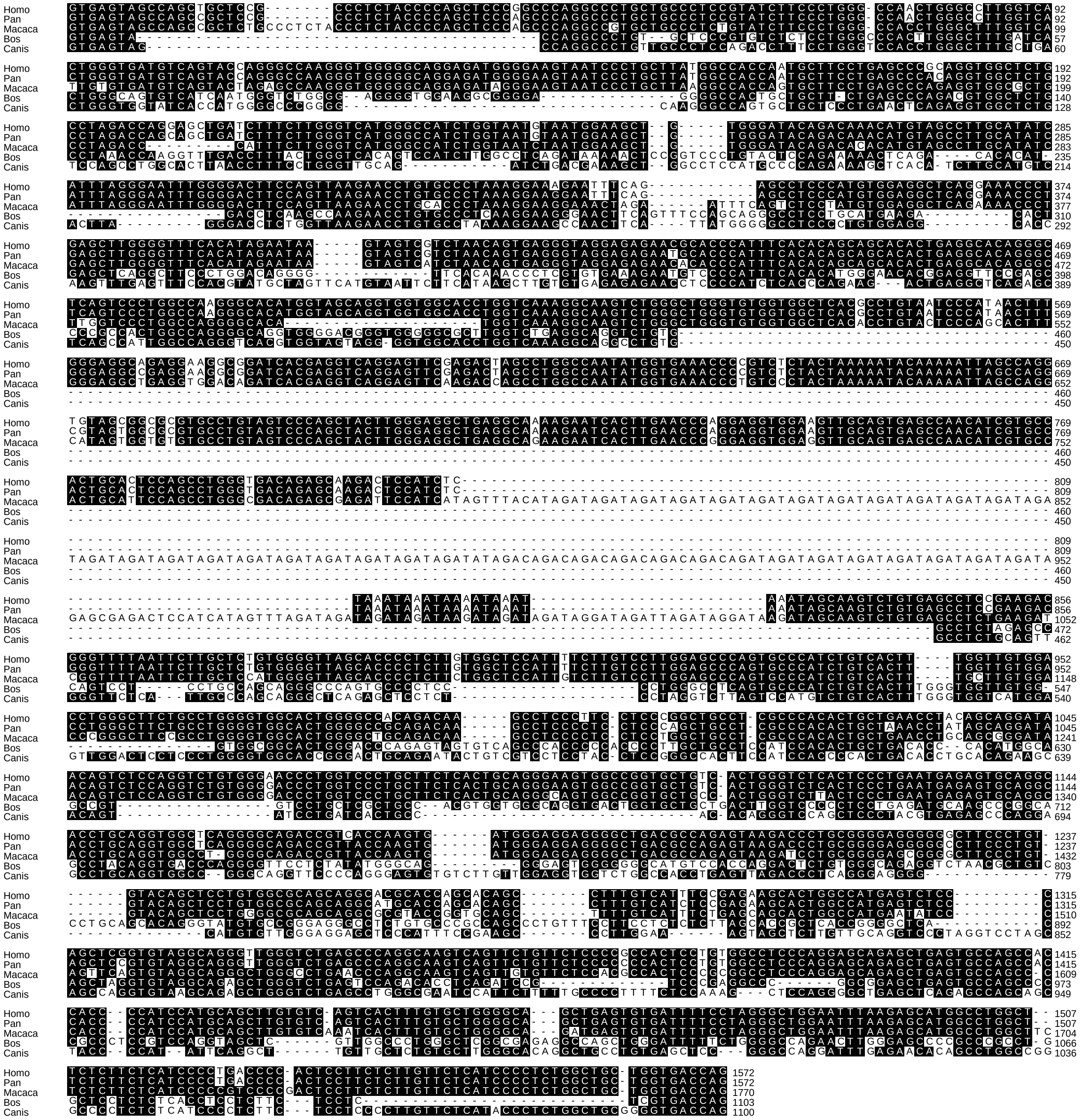

ENSG00000118655 intron 1

Description: DNA cross-link repair 1B protein (DCLRE1B)
Intron number: 1
Human chromosome: 01
Intron start (bp): 114249921
Human intron length : 1220
Intron alignment length: 1293
Flanking exons length (upstream/downstream): 189/166
SNP density: 0.008197
K tree score: 0.0919
Scaling factor: 0.4467
Human-chimpanzee distance: 0.009174
Total primate branch length: 0.0967

ENSG00000118655 exon 1

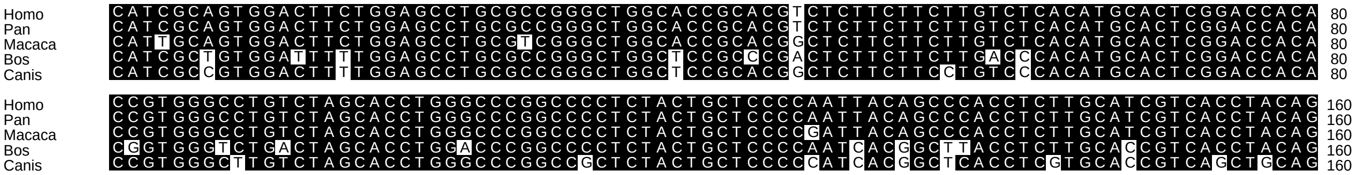

ENSG00000118655 exon 2

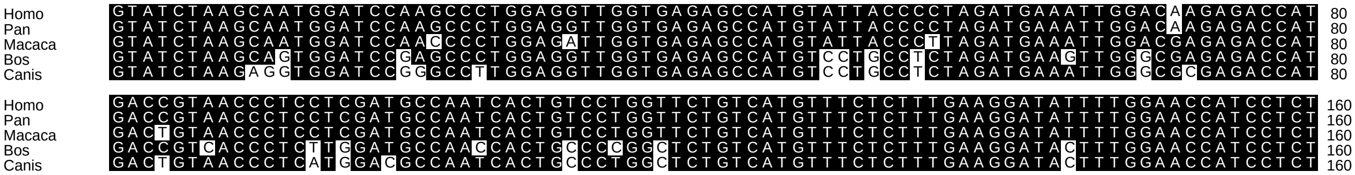

ENSG00000118655 intron 1

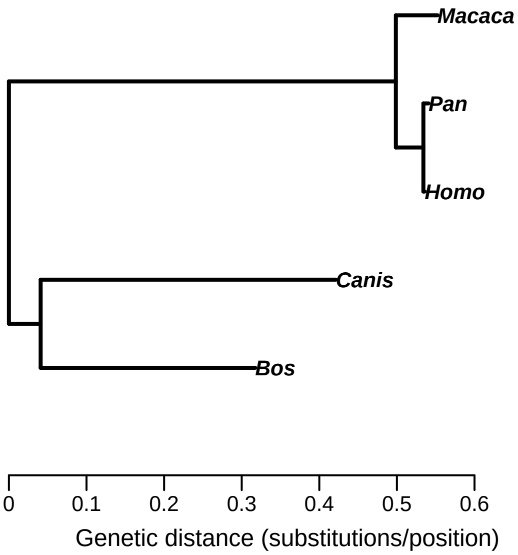

ENSG00000118655 intron 1

```
Homo    GTAT----------------GGGGCTGGAGTCGGTCTCCGAGAGCTGGTCCAGGCATCTGGGTTGGGACTTCAACCTGT--CATTCTTGGAGTCATAGAA 82
Pan     GTAT----------------GGGGCTGGAGTCGGTCTCCGAGAGCTGGTCCAGGCATCTGGGTTGGGCCTTCAACCTGT--CATTCTTGGAGTCATAGAA 82
Macaca  GTAT----------------GGGGCTGGAGTCAGTCTCCAAGAGGTGGTCCAGGCATCTGGGTTGGGACTTCGACCTGT--CATTCTTTGAGTCATAGAA 82
Bos     GTAT--------------GGGGGGCTGGAGTCGGCCTCTG----------------G----GTGGGCCTTCCGATCTGT--CAGTCTTGGAGTCATAGAG 65
Canis   GTACCCGGGGGCTCGGGGGAGGGGAAGGGGGCGGGGCGGA----------------GTCCCGTGGCGCTGTGCGATCCGTCACAGCCTCGGGCGCGTAGAG 85

Homo    TGGGGGCCACGAAAACTGATTT----------GGAAGTTGT-TTGAACCCAAAAATGCATCTTT--AACCCAGAATAGGAACGT-GTTTTCGAAGTGACC 168
Pan     TGGGGGCCACGAAAACTGATTT----------GGAAGTTGT-TTGAACCCAAAAATGCATCTTT--AACCCAGAATAGGAACGT-GTTTTCGAAGTGACC 168
Macaca  TGGGGGCCACCAAGACTGATGC----------GGAAGTTGT-TTGAACCCAAAAATGCATCTTT--AACTCAGAATAGGAACGT-GGTTTCCAAGTGACC 168
Bos     TTGTGGCCAGGAGAACTGATTTTTACAAAAGCGACAGTTTTGGGGAACCCAGAAATGCATATATTAAACCCAGAACAAGAGT---GGTTTCCAACTGGCT 162
Canis   -CGGGGACAGGAGGGCCGAGTTTTACAAATGCGGGAGTGGT-TTGAATCCAGAAATGCGCCTAT---AGCTAGAATGGGAGTCTGGCTTCTTGACTGGCC 180

Homo    AACTTGAGATAGTT-AGCCA-TAAGTTGAGGAGATATTTTAATCCAGCCAAGGAAACTATTCCTACCTTTATAAGTAAGCTAGGCCGATGTTTCCCAAAC 266
Pan     AACTTGAGATAGTT-AGCCA-AAAGTTGAGGAAATATTTTAATCCAGCCAAGGAAACTATTCCTACCTTTATACGTAAGCTAGGCCGATGTTTCCCAAAC 266
Macaca  AACTTGAGATATTT-AGCTA-TAAGTTGAGGAGATATTTTAGTCCAGCCAAGGAAACTATTGCTACCTTTATACATAAGCTAGGCTGATGTTTCCCAAAC 266
Bos     GGCTTGGGATGTTTCAGCGATAAAACTGGGAGGACTTCTAGGCCTTTCAAGGAAACTGTTCCTACTTCTTCACGTAAGCTAGGCCAGTGTTTTCCAAAC 262
Canis   GACTTGGGATGTTTCAGCTA-GAAGTCGGGGAGATCTTTTAGTCCACCCAAGGAAACTA--CCTACCTTTTCATGTAAACCAGGCCAGTGCTTCCTTAGT 277

Homo    TTGCCTTTTTTTTTTTTTTTTTTTTTTTTTTTGAGACGGAGTCTCGCTC-TGTTGC--CCAGGCTGGAGTGCAGTGGCGCAATCTCGGCTCACTGCAGC 363
Pan     TTGCC-----TTTTTTTTTTTTTTTTTTTTTTTGAGACGGAGTCTCGCTC-TGTTGC--CCAGGCTGGAGTGCAGTGGCGCAATCTCGGCTCACTGCAGC 358
Macaca  TTGCT---------TTTTTTTTTTTTTTTTCCTGATACGGAGTCTCGCTCTTGTTGCTGCCAGGCTGGAGTGCGGTGGCACGATCTCGGCTCACTATAAC 357
Bos     TTGGC----------------------------------------------------------------------------------------------- 267
Canis   ----C----------------------------------------------------------------------------------------------- 278

Homo    CTCCGCCTCCCGGGTTCAAGCGATTCTCCTGTCTCGGCCTCCTGAGTAGCTGAGATTACAGGCGCACGCCACCACGCCCAGCTAATTTTTTGTATTTTTT 463
Pan     CTCCGCCTCCCGGGTTCAAGCGATTCTCCTGTCTCGGCCTCCTGAGTAGCTGAGATTACAGGCGCACGCCACCACGCCCAGCAAATTTTTTGTATTTTTT 458
Macaca  CTCCGCCTCCCGGGTTCAAGAGATTCTCCTGTCTCAGCCTCCTGAGTAGCTGAGATTACAGGCGCACGCCACCACGCCCAGCTAATTTTTTGTATTTTTC 457
Bos     ---------------------------------------------------------------------------------------------------- 267
Canis   ---------------------------------------------------------------------------------------------------- 278

Homo    GTGGAGACGGGGTTTCACCATGTTGGCCAGGCTGTTCTCTAACTCCTGACCTTAAGTGATCCGCCCGCCTCAGCCTCCCCAAGTGCTGGGATTACAAGCG 563
Pan     GTGGAGACGGGGTTTCACCATGTTGGCCAGGCTGTTCTCTAACTCCTGACCTTAAGTGATCCGCCCGCCTCAGCCTCCCCAAGTGCTGGGATTACAAGCG 558
Macaca  GTAGAGACGGGGTTTCACCATGTTGGCCAGGCTGTTCTCAAACTCCTGACCTTAAGTGATCCGCCCGCCTCAGCCTCCCCAAGTGCTGGGATTACAAGCG 557
Bos     ---------------------------------------------------------------------------------------------------- 267
Canis   ---------------------------------------------------------------------------------------------------- 278

Homo    TGAGCCACCACGCCCCGCCTAAACTTGCCTCATCTTAAGAATCCACCTAATGTGCTGGG---------TGGTTCTCCAGCT----CTCTCCCCACCCCAC 650
Pan     TGAGCCACCACGCCCCGCCTAAACTTGCCTCATCTTAAGAATCCACCTAATGTGCTGGG---------TGGTTCTCCAGCT----CTCTCCCCACCCCAC 645
Macaca  TGAGCCACCACGCCCCGCCTAAATATGCCTTATCTTAAGAATCCACCTAACGTGCTGGG---------TGGTTCTCCAGCT----CTCTCCCCACCCCAC 644
Bos     -------------------------------TCCTAAGAATTCATCTAATGTTCTGAG----TAAATAGATTGTCCAGTG----CCTCCCTCCTCCCGC 327
Canis   -------------------------------TCATCAGAATCCACCTAATGTGCTGGGGGACTACACATGTTCTCCAGCGGCCCCCACCCTACCTCCGC 346

Homo    CCTTAAGTCTCCAAGAGATTAAGATCAGGAGTAAGTTTGGGAATTGCTGACTAGACTGTTTGCTGAACTC---TTTTTTTTTTTTTTGAGATGGAGTCTCG 747
Pan     CCTTAAGTCTCCAAGAGATTAAGATCAGGAGTAAGTTTGGGAATTGCTGACTAGACTGTTTGCTGAACTCTTTTTTTTTTTTTTTTTGAGATGGAGTCTCG 745
Macaca  CCTTAAGTCTCCAAGATATTAAGATCAGGAGTAAGTTTGAAAATTGCTGACTAGACTGTTTGATGAACTC-TCCCCCACCCCACCCAGACGGAGTGTCC 743
Bos     ATT-----TGAATCAGTAGACACAGCTGGTGCAGGTCCTGGAAAT-------------CTGGCTGGAATTTTTTTCACAGGTACCCCCTAAGGCTTCCC 409
Canis   TCTCCCACCGATCCAGTAGATCTAGCTGGAGCAGGTCTAGGAATC-------------TGGGTTGGA---------CCAGGTGCTCCCTAGGGATTATGA 424

Homo    CTCTGTCGCCCAGGCTGGAGTGCAGTGGCGCAAACTCGGCTCACTGCAAGCTCCGCCTCCCGGGTTCACGCCATTCTCCTGCCTCAGCCTCCCGAGTAGC 847
Pan     CTCTGTCGCCCAGGCTGGAGTGCAGTGGCGCAAACTCGGCTCACTGCAAGCTCCGCCTCCCGGGTTCACACCATTCTCCTGCCTCAGCCTCCCGAGTAGC 845
Macaca  CTCTGTTGCCCAGGCTGGAGGACACT---------CCAGTTCACTGCAAGCTCCGCCTCCCGGGTTCACGCCATTCTCCTGCCTCAGCCTCCCGAGTACC 834
Bos     TGGTGGCTCAGACGGTAAAGAATCCGCCTGCAATTCGGGAGACCTG---GGTCCGATCCCGGGGTCGGGAAAATCCCGTGG------------------- 487
Canis   TGAT--CAGGAATGTTTGAGAATCT---------CTTGGCTAGACTG---GTTTTGGACTCATAATT---TAAACTGTACAA------------------- 489

Homo    TGGGACTACAGGCGCCCGCCACCACGCCTGGCTAA-TTTTTTTTGTATTTTTTAGTAGAGACGGGGTTTCACCG----TGTTAGCCAGGATGGTCTCGAT 942
Pan     TGGGACTACAGGCGCCCGCCACCACGCCCAGCTAATTTTTTTTTGTATTTTTTAGTAGAGACGGGATTTCACCG----TGTTAGCCAGGATGGTCTCGAT 941
Macaca  TGGTACTACAGGTGCCCGCCACCTTTTTT-------TTTTATTATTATTTTTAAGTAGAGATGGGGTTTCACCG----TGTTAGCCAGGACAGTCCCGAT 923
Bos     -------AGAAGGGCGTGGTAACACCCTCCAGTATTCTTGCCTGGAGAATTCCATGGACAGAGGAATCTAGTGGGCTGTAGTCCATGGGGTGGCAAAGAG 580
Canis   -------AGAGTGGAGGGGA---------------------GAGTAAGAGAAAGAAGGGGGGATTTGAGGG---------------------------- 533

Homo    CTCCTGACCTCGTGATCCACCCGCCTCAGCCTCC-------CAAAGTGCTGGGATTACAGGCGTGAGCCACCACGCCCGGCCAGCTGAACTC----TTAA 1031
Pan     CTCCTGACCTCGTGATCCACCCGCCTCAGCCTCC-------CAAAGTGCTGGGATTACAGGCATGAGCCACCACGCCCGGCCAGCTGAACTC----TTAA 1030
Macaca  CTCCTGACCTCATGATCCTCCCGCCTCGGCCTCT-------CAAAATGCTGGGATTACAGGCATGAGCCACCGCGCCCGGCCAGCTGAACTCTTAATTAA 1016
Bos     TTGGCCACGACTGAGTGATTAAGCACAGCACTCCAAGGGATTATTATTTGGAAAGTTGAGGAATGGTTGAATAGACTT-GCTTTCTGGACTATTAATTTA 679
Canis   -----------------GACTCTCCATAGAGCTCC-------TGCTCCACGGTGGCTTAGGATGTGGTCTGCTTTCCCT-CCCTCCT--------------- 595

Homo    A--TTGTACAAAGGGTGGGATGGGAAGTGGAAGGAGGAAGGGCAGATTGCTTGGGAATCCCCATTACAGCTTCTGCTCCCGGTGGCTCAGGATTTGGTCA 1129
Pan     A--TTGTACAAAGGGTGGGATGGGAAGTGGAAGGAGGAAGGGCAGATTGCTTGGGAATCCCCATTACAGCTTCTGCTCCTGGTGGCTCAGGATTTGGTCA 1128
Macaca  A--TTGTACAAAGGGTGGGATGGGGAGTGGAAGGAGGAAGGGCAGATTGCTTGGGAATCCCCATTACAGCTTCTGCTCGCGGTGACTCAGGATTTGGTCA 1114
Bos     GCCTTGTAGGAAGGATGGGGTAGAGAGAGGGAGAAGGAAAAGAGGACTG--------------------------------------------------- 728
Canis   ----------------TGCCGTAAAGGG------------------------------------------------------------------------ 607

Homo    GCTTTCCTGATTCATGTAAGGGATAGTCCCTGACCCGGGGAGGTAAC--GGAGAGGAGTCAGTGGTCACTGGGATGACTAACTGTTTTCTCAG 1220
Pan     GCTTTCCTGATTCATGTAAGGGATAGTCCCTGACCCGGGGAGGTAACAGGGAGAGGAGTCAGTGGTCACTGGGATGACTAACTGTTTTCTCAG 1221
Macaca  GCTTTCTTGATTCATGTAAAGCATAGTCCCTGACCCGGGGAAGTAACAGGGAGGGGAGTCGGTGGTCACTGGGATGACTAACTGTTTTCTCAG 1207
Bos     -----------TGATCCCTATCACAGCTTCTGATCCAGGGAGGTACCAGGGAGAGGCGTCTGTGTTTCCTGAGATGACCTGCTGTTTCTTTAG 810
Canis   ----------------------TTAGTTCCTGATCCAGGGCAGTATCAAGGAGA---TTCTGTGTTCACTAGAATGACCACCTTTTCCTCAG 675
```

ENSG00000105618 intron 3

Description: U4/U6 small nuclear ribonucleoprotein Prp31 (PRPF31)
Intron number: 3
Human chromosome: 19
Intron start (bp): 59317135
Human intron length : 553
Intron alignment length: 695
Flanking exons length (upstream/downstream): 84/98
SNP density: 0.001808
K tree score: 0.0901
Scaling factor: 0.8248
Human-chimpanzee distance: 0.016535
Total primate branch length: 0.0962

ENSG00000105618 exon 3

```
Homo    TGATGGGACCAGTGGAGGCCGCGCCTGAATACCGCGTCATCGTGGATGCCAACAACCTGACCGTGGAGATCGAAAACGAG 80
Pan     TGATGGGACCAGTGGAGGCAGCACCTGAATACCGCGTCATCGTGGATGCCAACAACCTGACCGTGGAGATCGAAAATGAG 80
Macaca  TGATGGGACCGGTGGAGGCAGCGCCTGAATACCGCGTCATCGTGGATGCCAACAACCTGACCGTGGAGATCGAAAACGAG 80
Bos     TGATGGGACCGGTGGAGGCGGCCCCTGAGTATCGGGTCATCGTAGATGCCAACAACCTAACAGTAGAGATCGAGAATGAG 80
Canis   TGATGGGACCTGTTGAGGCGGCCCCTGAATACCGAGTCATCGTGGATGCTAACAACCTGACCGTGGAGATCGAGAATGAG 80

Homo    CTGA 84
Pan     CTGA 84
Macaca  CTGA 84
Bos     CTCA 84
Canis   CTGA 84
```

ENSG00000105618 exon 4

```
Homo    ACATCATCCATAAGTTCATCCGGGATAAGTACTCAAAGAGATTCCCTGAACTGGAGTCCTTGGTCCCCAATGCACTGGAT 80
Pan     ACATCATCCATAAGTTCATCCGGGATAAGTACTCAAAGAGATTCCCTGAACTGGAGTCCTTGGTCCCCAATGCACTGGAT 80
Macaca  ACATCATCCATAAGTTCATCCGGGATAAGTACTCGAAGAGATTCCCTGAACTGGAGTCCTTGGTCCCCAATGCACTGGAT 80
Bos     ACATCATCCACAAGTTCATCCGGGATAAGTACTCAAAGCGATTCCCCGAGCTGGAGTCCCTGGTCCCCAACGCCCTGGAT 80
Canis   ACATCATCCATAAGTTCATCCGGGATAAGTACTCAAAGCGCTTCCCTGAACTGGAGTCTCTGGTCCCTAATGCACTGGAT 80

Homo    TACATCCGCACGGTCAAG 98
Pan     TACATCCGCACGGTCAAG 98
Macaca  TACATCCGCACGGTCAAG 98
Bos     TACATCCGCACTGTCAAG 98
Canis   TACATCCGCACGGTCAAG 98
```

ENSG00000105618 intron 3

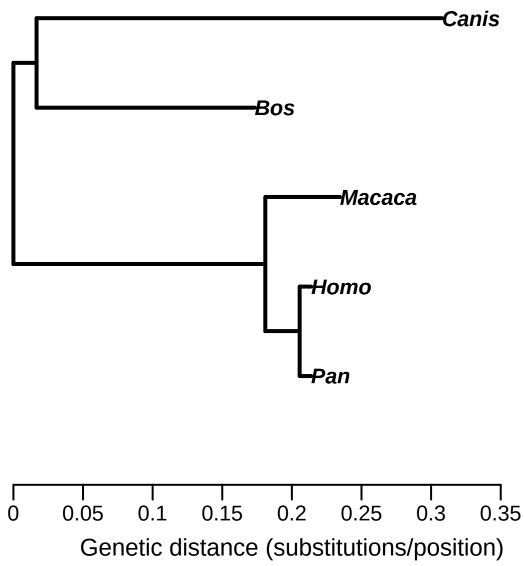

ENSG00000105618 intron 3

```
Homo    GTGAGTGCTGGG----------------------------------------------GGGCAGGCGGAGACAGCCCCGTGTGACGTCCCTCACGCCCCC 54
Pan     GTGAGTGCTGGG----------------------------------------------GGGCAGGCGGAGACAGCCCTGTGTGACGTCCCTCACGCCCCC 54
Macaca  GTGAGTGCT-GG----------------------------------------------GGGCAGGCGGAGACAGCCCCCTGTGACGCCCCTCATGCCCC- 52
Bos     GTGAGTACTGGGAAGGGCAACCAGTGAAGATCGAGAACGAGCTGAGTGAGCACTGGGAGGGCAAGCAGAAACAGCCCTGCCTGGTGTCCCCCACAGCCTC 100
Canis   GTGAGTGGCAGG------------------------------------------------AAGGGCAGAGATGGCCCTGCCTGCTGTCCCTTCCACTCTC 52

Homo    TCTC-----------------------------------CCTTCCCCACTGGCCTTTCCCAGGGTCCTGCCCCTAAGCCC------AAGCTCAGATCGAG 113
Pan     TCTC-----------------------------------CCTTCCCCACTGGCCTTTCCCAGGGTCCTGCCCCTAAGCCC------AAGCTCAGATCGAG 113
Macaca  -CTC-----------------------------------CCTTCCCCACGGGCCTTTCCCAGGGTCCTGCCGCTGAGCCC------AAGCTCAGATCGAG 110
Bos     TCCCGTCCCCATTTGAGGGGCGGGCAGGATCCCAGGCCACCGTCCCTGCTGGCCTTGCCCAGGGTCTTGCCCCTAAGCCCCTGTATTAGCTCAAACATAG 200
Canis   CTCCCATCCACAAGGAGAGGACAGGCAGGACCCTGGTTACTTCCCCACTGGCCTTCCCCAAGATCCTGCCCCAGACTCCTCATACTAGCTGTAGTCCAG 152

Homo    GTTGACCTGCTGTCACAGAGTGGCTGAAATAAGAAGGAAGTGCGTTCT---CTCGCGTATGAGTCTGAG-GAGCACTCGGGGATGGTGTGGCCGCTTGGC 209
Pan     GTTCACCTGCTGTCACAGAGTGGCTGAAATAAGAAGGAAGTGCGTTCT---CTCGCGTATGAGTCTGAG-GAGCACTTGGGGATGGTGTGGCCGCTTGGC 209
Macaca  GTTCACCTGCTGTAACGGAGTGGTGTAAGTAAGAAGGAAGTGTGTTCTCC-CTCACGTATGAGTCTGAGCGAGCACTCGGGGACGGCGTGGCCGCTTGGC 209
Bos     GTTCAGCTACTG-AACAGAATGGT-GACATGAGAAGGAAGTTTATTTTCCTCTCATGGGAAAGTCTGGATGAG---------------------------- 271
Canis   ATTCAGCTGCCATAACAGCAGCTT---AAAAAAAAGAAAGTGTGCTTTTCTCCGATGTGAGAATCTGGATGAGGAGTCCAGCCCT--------------- 234

Homo    TGCCTGTAGGGCCCCGGCTCTTTCCATCCTGTTGGTCGGCCACCTGCCTCACGGTGCGAGGTGACTGCCCCACCTCCAGCCATCACCTCCGCATTCCCAC 309
Pan     TGCCTGTAGGGCCCCGGCTCTTTCCATCCTGTTGGTCGGCCACCTGCCTCACGGTGCGAGGTGACTGCCCCACCTCCAGCCATCACCTCCGCATTCCCAC 309
Macaca  TGCCTGTAAGGCCCCAGCCCTTTCCATCTTGTTGGTCGGCCACCTGCCTCGTGGTGCGAGGGGACTGCCCCACGTCCAGCCGTCGCCTCTGCATTCCCAC 309
Bos     --------GAGCCCAGGCTGTCTGAGTCTTACTGATCTGTCACCTGTTTCGTGGTACAAAGTGACTA-CCTACCTCCAGCCATCACCTCTACATCCCCAT 362
Canis   --------GGAGTCAGGCTCTTTCTGTCT---TGACCTGCCACCTGTGTCAGGGCACAAAGTGACTG-TCCACCTCCAGTCATCACCTCCACATCCCCAC 322

Homo    CAGCAA---------------------------GGCGCTTCTTTTCTTTAAGAA-------------------CATGTCACTGCAGCTCACGTTTTACAG 363
Pan     CAGCAA---------------------------GGCGCTTCTTTTCTTTAAGAA-------------------CATGTCACTGCAGCTCACGTTTTACAG 363
Macaca  CAGCAA---------------------------GGCACTTCTTTTTTTTAAGAA-------------------CATGTCACTGCAGCTCACATTTTACAG 363
Bos     CAGCAAAAAAAAATCAGGAGAGAAGAGGAGGAGGGCACATTTTTTCCTTTAAAAGAGTGACCTGGAAGTTGCCCGTGTGACTTGCTCTCACGCCTTATGG 462
Canis   CAGCAG---------AACAGGGAAGAAAAAGAGGGCACAGTT----CTTTAAGGGGGTGACCTGGAAATTGCATGCATCACTTCATATGATATCCTGTAG 409

Homo    ACCAGAACTAATTCCCCTGGTCACACCTAGCGGTAAGGACGGCTGAGAAA----GGCTGTATGCTG-GTGCCCGTGTGCCAGGCCACAAGCCAGGGCTTC 458
Pan     ACCAGAACTAATTCCCCTGGTCACACCTAGCGGTAAGGGCGGCTGAGAAA----GCCTTTATGCTG-GTGCCCGTGTGCCAGGCTACAAGCCAGGGCTTC 458
Macaca  ACCAGAACTAATTCCCCTGGTCACACCTAGCGGTAAGGGCGGCTGAAAAATGGGGCCTGTATGCTG-GTGCCCGTATGCCAGGCTACATGCCAGGGCTTC 462
Bos     ACCAGAACTGATCTACACGGCCGCAGCCAGCAGTAGGGGCA-------------GCCTTTGTGCTGTGTGTCTGTGAACCCAGCTGCCAGGAGGGGCTCC 549
Canis   ACCAGAACTAACTTGCAGGGCCACACCTGGCAGCAGGGGAGTA-----------GCGTTTTTGCTGGGTACCCACGAACCCAGCTAAATTCAGGGGTTCA 498

Homo    AGTTACTAAAGGAAGAAGGGGACATGGGTGTTAGGGCCAACCAGCAGAGTCTACCTTCCATCTCACCCGACAACCTCCTGTCCCGTTTACCCTAG 553
Pan     AGTTACTAAAGGAGGAAGGGGACATGGGTGTTAGGGCCAACCAGCAGAGTCTATCTTCCATCTCACCCGACAACCTCCTGTCCCGTTTACCCTAG 553
Macaca  AGTTACTAAAGGAAGAAGGGGACGTGGGTGTTAGGGCCAACCAGCAGAGTCTGCCTCCCGTCTCACCTGACACCCGCCTGTCCCGTTTACCCTAG 557
Bos     -CTCACTGCAGGACGAAGGGCAGATGGGTGCTGGGG-GAGCCCGCGGCATCTGCCTGCC-CCTCATCAGGCTCCTGCCCGCCCTGTGC-CCCCAG 640
Canis   TTTATTTAAAAAGAAGCAGGGAGATGGGTGTTGCGGACAGCCAGTGGAGTCTGCCTGCCCCTTATCAG----CTACCTGTTCTCCCTACCTTAG 589
```

ENSG00000175707 intron 2

Description: C1orf172 protein (C1orf172)
Intron number: 2
Human chromosome: 01
Intron start (bp): 27149230
Human intron length : 530
Intron alignment length: 577
Flanking exons length (upstream/downstream): 75/83
SNP density: 0.003774
K tree score: 0.0707
Scaling factor: 0.7885
Human-chimpanzee distance: 0.009716
Total primate branch length: 0.0961

ENSG00000175707 exon 2

```
Homo    AGCTGACAGTGCAGATCTCCCAGGAGACGACTGCAGATGCCATCGCCCGGAAGCTGAGGCCTTATGGAGCTCCAG 75
Pan     AGCTGACAGTGCAGATCTCCCAGGAGACGACTGCAGATGCCATCGCCCGGAAGCTGAGGCCTTATGGAGTTCCAG 75
Macaca  AGCTGACAGTGCAGATCTCCCAGGAGACGACTGCAGATGCCATCGCCCGGAAGCTGAGGCCTTATGGAGCTCCAG 75
Bos     AACTCACGGTGCAGATCTCCCAGGAGACGACCGCAGATGCCATTGCCAGGAAGCTGAGACCCTATGGAGCCCCAG 75
Canis   AGCTCACAGTGCAGATCTCCCAGGAGACTACTGCAGATGCCATCGCCAGGAAGCTGAGGCCTTATGGAGCTCCAG 75
```

ENSG00000175707 exon 3

```
Homo    GGTACCCAGCAAGCCATGACTCATCCTTCCAGGGCACCGACACAGACTCGTCGGGGGCACCCTTGCTCCAGGTGTACTGC 80
Pan     GGTACCCAGCAAGCCATGACTCATCCTTCCAGGGCACCGACACAGACTCGTCGGGGGCACCCTTGCTCCAGGTGTACTGC 80
Macaca  GGTACCCAGCCAGCCATGACTCATCCTTCCAGGGCACCGACACAGACTCATCGGGGGCACCCTTGCTCCAGGTGTACTGC 80
Bos     GATACCCTGCCAGCCACGACTCATCCTTCCAGGGCACCGACACAGACTCTTCAGGGGCACCCCTGCTCCAGGTGTATTGC 80
Canis   GATACCCAGCCAGCCATGATTCATCCTTCCAGGGTACAGACACAGACTCATCAGGAGCACCGCTGCTCCAGGTGTACTGC 80

Homo    TAA 83
Pan     TAA 83
Macaca  TAA 83
Bos     TAA 83
Canis   TAA 83
```

ENSG00000175707 intron 2

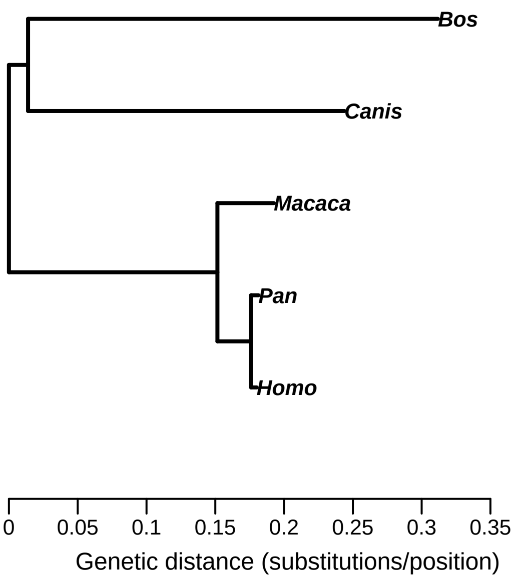

ENSG00000175707 intron 2

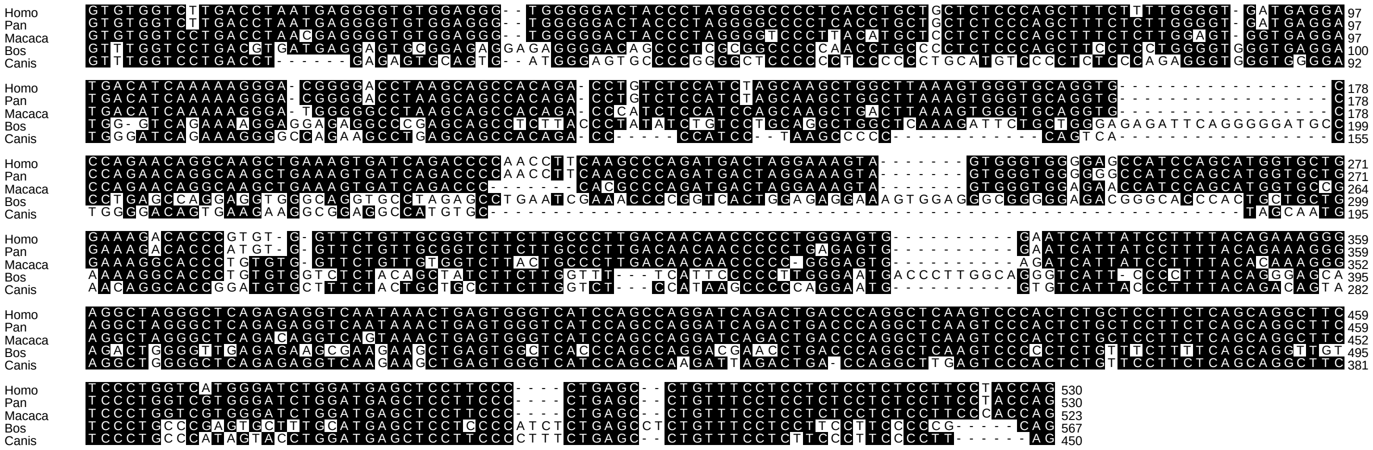

ENSG00000131653 intron 4

Description: E3 ubiquitin protein ligase TRAF7 (TRAF7)
Intron number: 4
Human chromosome: 16
Intron start (bp): 2160733
Human intron length : 533
Intron alignment length: 539
Flanking exons length (upstream/downstream): 117/93
SNP density: 0.007505
K tree score: 0.0835
Scaling factor: 0.416
Human-chimpanzee distance: 0.013441
Total primate branch length: 0.096

ENSG00000131653 exon 4

```
Homo    CCCCCCATCAGCACTCCCCGCCGCTCCGACTCCGCCATCTCTGTCCGCTCCCTGCACTCAGAGTCCAGCATGTCTCTGCG 80
Pan     CCCCCCATCAGCACTCCCCGCCGCTCCGACTCCGCCATCTCCGTCCGCTCCCTGCACTCAGAGTCCAGCATGTCTCTGCG 80
Macaca  CCCCCCATCAGCACTCCCCGCCGCTCCGACTCTGCCATCTCCGTCCGCTCCCTGCACTCAGAGTCCAGCATGTCCCTGCG 80
Bos     CCCCCTATCAGCACGCCTCGCCGCTCCGACTCAGCCATCTCCGTGCGCTCCCTGCACTCTGAGTCTAGCATGTCCCTCCG 80
Canis   CCCCCCATCAGCACCCCACGCCGCTCCGACTCGGCTATCTCCGTCCGCTCCCTGCACTCAGAGTCCAGCATGTCCCTGCG 80

Homo    CTCCACATTCTCACTGCCCGAGGAGGAGGAGGAGCCG 117
Pan     CTCCACATTCTCACTGCCCGAGGAGGAGGAGGAGCCG 117
Macaca  CTCCACATTCTCGCTGCCCGAGGAGGAGGAGGAGCCG 117
Bos     CTCCACGTTCTCGCTGCCGGAGGAGGAGGAGGAGCCG 117
Canis   CTCCACGTTCTCGCTGCCTGAGGAGGAGGAGGAGCCG 117
```

ENSG00000131653 exon 5

```
Homo    GAGCCACTGGTGTTTGCGGAGCAGCCCTCGGTGAAGCTGTGCTGTCAGCTCTGCTGCAGCGTCTTCAAAGACCCCGTGAT 80
Pan     GAGCCACTGGTGTTTGCGGAGCAGCCCTCGGTGAAGCTGTGCTGTCAGCTCTGCTGCAGCGTCTTCAAAGACCCCGTGAT 80
Macaca  GAGCCACTGGTGTTTGCGGAGCAGCCCTCGGTGAAGCTGTGCTGTCAGCTCTGCTGCAGTGTCTTCAAAGACCCCGTGAT 80
Bos     GAGCCGCTGGTGTTTGCCGAGCAGCCCTCGGTGAAGCTGTGCTGCCAGCTCTGCTGCAGCGTGTTCAAGGACCCCGTGAT 80
Canis   GAGCCGCTGGTGTTTGCTGAGCAGCCCTCGGTGAAGCTCTGCTGCCAGCTTTGCTGCAGCGTGTTCAAGGACCCTGTGAT 80

Homo    CACCACGTGTGGG 93
Pan     CACCACGTGTGGG 93
Macaca  CACCACGTGTGGG 93
Bos     CACCACGTGTGGG 93
Canis   CACCACGTGTGGG 93
```

ENSG00000131653 intron 4

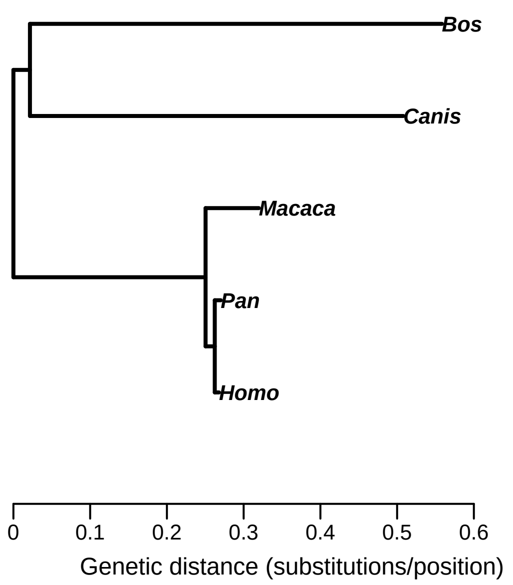

ENSG00000131653 intron 4

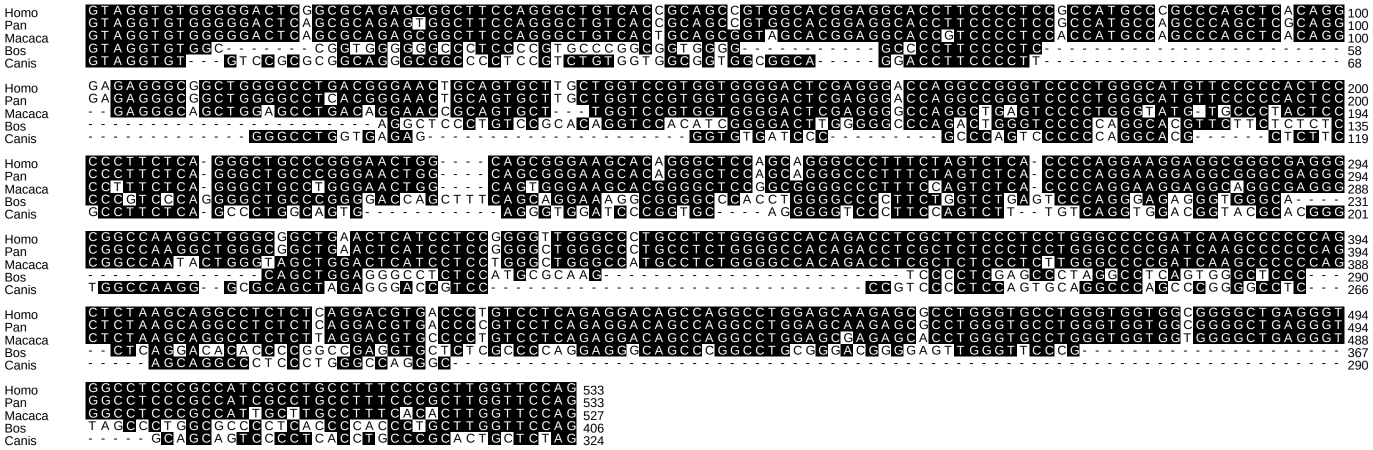

ENSG00000094755 intron 3

Description: Gamma-aminobutyric-acid receptor subunit pi precursor (GABRP)
Intron number: 3
Human chromosome: 05
Intron start (bp): 170153881
Human intron length : 909
Intron alignment length: 936
Flanking exons length (upstream/downstream): 68/218
SNP density: 0.005501
K tree score: 0.0808
Scaling factor: 0.9496
Human-chimpanzee distance: 0.009095
Total primate branch length: 0.0958

ENSG00000094755 exon 3

```
Homo    GAGAACCCGTACAGATAGCGCTGACTCTGGACATTGCAAGTATCTCTAGCATTTCAGAGAGTAACATG 68
Pan     GAGAACCCGTACAGATAGCACTGACTCTGGACATTGCAAGTATCTCTAGCATTTCAGAGAGTAACATG 68
Macaca  GAGAACCCGTTCAGATAGCGCTGACTCTGGACATTGCAAGTATCTCTAGCATTTCAGAGAGTAACATG 68
Bos     GAGAACCTGTTCAGATAGCACTGACTCTGGACGTTGCAAGTATTTCCAGTATTTCAGAGAGTAATATG 68
Canis   GAGAACCAGTTCAGATAGCACTGACTATGGACATTGCAAGTATTTCTAGTATTTCAGAGAGTAACATG 68
```

ENSG00000094755 exon 4

```
Homo    GACTACACAGCCACCATATACCTCCGACAGCGCTGGATGGACCAGCGGCTGGTGTTTGAAGGCAACAAGAGCTTCACTCT 80
Pan     GACTACACAGCCACCATATACCTCCGACAGCGCTGGATGGACCAGCGGCTGGTGTTTGAAGGCAACAAGAGCTTCACTCT 80
Macaca  GACTACACAGCCACCATATACCTCCGACAGCGCTGGATGGACCAGCGGCTGGTGTTTGAAGGCAACAAGAGCTTCACTCT 80
Bos     GACTACACAGCCACCATATACCTCAGACAGCGCTGGACGGACCAGCGGCTGGTGTTTGAAGGCAACAAGAGCTTCACTCT 80
Canis   GACTACACAGCCACCATATACCTCAGACAACGCTGGACGGACCAGCGGCTGGTGTTCGAAGGCAACAAGAGCTTTACTCT 80

Homo    GGATGCCCGCCTCGTGGAGTTCCTCTGGGTGCCAGATACTTACATTGTGGAGTCCAAGAAGTCCTTCCTCCATGAAGTCA 160
Pan     GGATGCCCGCCTCGTGGAGTTCCTCTGGGTGCCAGACACTTACATTGTGGAGTCCAAGAAGTCCTTCCTCCATGAAGTCA 160
Macaca  GGATGCCCGCCTCGTGGAGTTCCTCTGGGTGCCGGACACTTACATTGTGGAGTCCAAGAAGTCCTTCCTCCATGAAGTCA 160
Bos     GGATGCACGCCTAGTGGAATTCCTCTGGGTGCCAGACACCTACATCGTGGAGTCCAAGAAGTCCTTCCTCCATGAAGTCA 160
Canis   GGATGCACGCCTCGTGGAGTTCCTCTGGGTGCCGGATACATACATCGTGGAGTCCAAGAAGTCCTTCCTCCATGAAGTCA 160
```

ENSG00000094755 intron 3

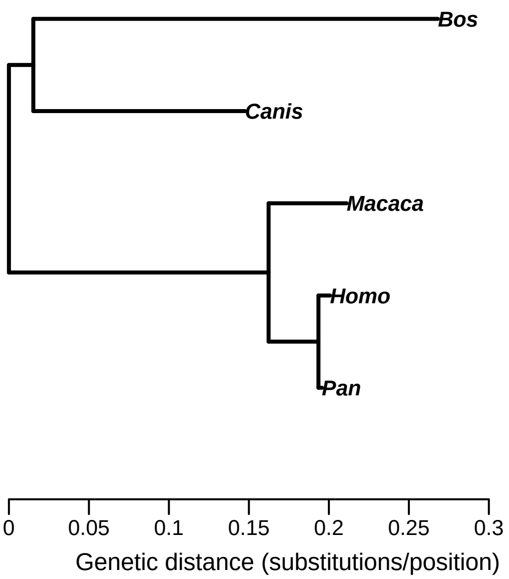

ENSG00000094755 intron 3

```
Homo    GTAAGCGCTGTTCCTTTGTACTCTACCCAAGTAGTCCCTCTCTGTGTGTTTAAAGGGGATATGCTTTCTAGCCGCTCAAAAAAAAAAAAAAAAAAAAAGA 100
Pan     GTAAGCGCTGTTCCTTTGTACTCTACCCAAGTAGTCCCTCTCTGTGTGTTTAAAGGGGATATGCTTTCTAGCCGCTC---------AAAAAAAAAAAAGA 91
Macaca  GTAAGTGCTGTTCCTTTGTACTCCACCGAAGTAGTCCCTCTCCGTGTGTTTAATGGGAATATGCTTTCTAGCCACTC----------AAAAAAAAAAAAA 90
Bos     GTAAGTGTGCTTCCCTCGCCTTCCACCTATTGAGCCCCTTCCC-TGTGTTTTATGAGGAAGCCCATTCTAGCTATGC------------AAAACAGAACA 87
Canis   GTAAGTG-TGGCCCTTTCCTCTTTACCTGAGGAGTCCTTCT---TGTGTTTTATGGGGATATACTTTCTAGCTACTC-----------AAAAACAGAATA 85

Homo    ATGGCTCATGGCCCTCCTCCATTTGGAATGGCAGAAGCTGGAGTGCTATCTCCAAACTCAAGACAGTGGGAGATTCTTGTAGAGCAGATTTTCTGAGTAA 200
Pan     ATGGCTCATGGCCCTCCTCCATTTGGAATGGCAGAAGCTGGAGTGCTATCTCCAAACTCAAGACAGTGGGAGATTCTTGTAGAGCAGATTTTCTGAGTAA 191
Macaca  ATGGCTCACGGCCCTCCTGCATTTGAAATGGCAGAAGTTGGAGTGCTGTCTCCAAACTC---------------------------------------AA 151
Bos     GAGACTTA--------------TTGGAATGGCAGGAACTGGAGACCTATCCTGAAACTC---------------------------------------AA 134
Canis   GGGACTCACAGCCCCCTTGTACTTGAAATGACAGAAACTGGAGACCTATCCCAAAACTC---------------------------------------AA 146

Homo    GACAATGGAAGATTCTTGTAGAGCAGATTTCCTGAGTAGCAGGAAGAAAATCGGTTCAAGAGCCAGAAGGAGAGAAACCAGGCAATGAAGTGGGTACACT 300
Pan     GACAATGGAAGATTCTTGTAGAGCAGATTTTCTGAGTAGCAGGAAGAAAATCGGTTCAAGAGCCAGAAGGAGAGAAACCAGGCAATGAAGTGGGTACACT 291
Macaca  GACAATGGAAGATTCTTATAGAGCCGATTTTCTGAGTATCAGGAAGAAAATCGGTTCAAGAGCCAGGAGGAGAGAAACCAGGCGATGAAGTGGGTACACT 251
Bos     GACAGTGGAAGATGCTTGTAAAACAAATCTTCTGAGTATCAGAGGAAAAGTGGATTCAAAGGCTAGGAGGCGGGGG----GATGAGGGAGTGGGTGTGCT 230
Canis   GACAGTGGAAGATTCTTGTAAAACAGATCTTCTGAGTATCAGGAAGAAAGTGGATTCAAGGGCCAGGAAGGAAAAGATGAGGGAATGGGGCGGGTGCATT 246

Homo    TGGTGATGGATAAAATTTGGGGAAAGGAATGAAAGTGTAGTAGGCAAGCCTGGCCATTACATGGTCTTTTATAATGGGGGAGACAACCCGAG-----GAA 395
Pan     TGGTGATGGATAAAATTTGGGGAAAGGAATGAAAGTGTAGTAGGCAAGCCTGGCCATTACATGGTCTTTTATAATGGGGGAGACAACCCGAG-----GAA 386
Macaca  TGGTAATGGATAAAATCTGGGGAAAGGAATAGAAGTGTAGTAGGCAAGCCAGGCCATCACGTGGTCTTTCATAACAGGGGAGACAACCCAAG-----GAA 346
Bos     GGGTGAGGGATAAGAATGGGGAAAAATA----AAATGCAGGGAGGAAGCCTAAATATCACATGGTTCCTCACAGAGCAGGAGACAAGCTGAT-----GAA 321
Canis   TGATGAGGAATAAAAACT-GGGTAATGAATGAAAGCACAGTGGAGAAGCCTGAATATCAAGTGGACCCTCCCAGGGTGGAAGCTAAGCTAAGCTGATGAA 345

Homo    AACTGACAGCT-----GATGCTGACAACGACTGTCTCCCTTAAGAGAACATATTCGAGACAAGGGCAAACTAAAGGCAGTTTGCCAAGAGGGTATGCTGT 490
Pan     AACTGACAGCT-----GATGCTGACAACGACCCTCTCCCTTAAGAGAACATATTCGAGACAAGGGCAAACTAAAGGCAGTTTGCCAAGAGGGTATGCTGT 481
Macaca  AACTGACAGCT-----GGTGCTGACAACGACCCTCTCCCTTAAGAGAACACATTTGAGACAAGGGCAAACTAAAGGCAGTTTGCCAAGAGGGCATGCTGT 441
Bos     AACCAATAATCACGTAACTTTTTCCAACCATCTTCTCACTTAAGAGAACACATTTTAGGCAAGGTGACACTACAGGCAGTTTGCCATGAGAGTCCGCTGT 421
Canis   AACTGGCAATCACATAAGTGCTTTCCACCATTCTCTCACTTAAGAGAACCCATTAAAGACAAAGTGACACTAAAGGCAGGTTGCCAAGGGGGCCTGCTGT 445

Homo    GCATTCCAGATAAATCGTGGACAACCTAAGCACTTAGCCCACCAAAAAATTCAACAATTTGGTGGGGCCTTAGTGATCAGTGCAATGTGAGCTGTCACAC 590
Pan     GCATTCCAGATAAATCGTGGACAACCTAAGCACTTAGCCCACCAAAAAATTCAACAATTTGGTGGGGCCTTAGTGATCAGTGCAATGTGAGCTGTCACAC 581
Macaca  GCATTCCAGATAAATCATGGACAACCTGAGCACTTAGCCCACCAAAAAATTCAACCATTTGATGGGGCCTTAGTGATCAGTGCAATGTGAGCTGTCACGC 541
Bos     AGACCCCAGGTAAAC--TGGGCAGCC-----------------------TTGAAAAACTTACTGTGGCCTTTGTGATCA-----------------AGGG 479
Canis   GCATTCTGGGTAAATCCTGGACAACCTAAGCACTTAGCTCACCCAAAACTTCAACCACTCGGAGGGGCCTTTGGGTTAG-----------------AAGG 528

Homo    CAAGTAAGTTAAAGTCTTGAACA--------TGGCGTTCCCTTTTGGCAATAGGAGCTATTATCTAGAATAAATAAGGCGATTTGTTCTCTTATTGCAAT 682
Pan     CAAGTAAGTTAAAGTCTTGAACA--------TGGGGTTCCCTTTTGGCAATGGGAGCTATTATCTAGAATAAATAAGGCGATTTGTTCTCTTATTGCAAT 673
Macaca  CACGTAAGTTAAAGTCTCAAACA--------TAGGGCTCCCTTTTGGCAGGGGGAGCTATTATCTCAAATAAATAAGGTGATTTGTTCTCTTATTGCAAT 633
Bos     ATAGTAAGTTAAGGCCCTGAACATACTCCTATGTGGCTCCCTTTGGGCAGTGGGTGTTGTTATCTGGAATAAATACGGTGCTTTGTCCTCATATAGCTGC 579
Canis   CAAGCAAGTTAAGGTCCTGAACATGCTCCTCTGGGGCTCCCTTTTGGCAGTGAGAGTTCTGGTGGAGAATAAATAAGGTGCTTTGGTCTC--ATTGCTAC 626

Homo    ATGCCACTAACCCAAGACTGGGGTAACTGGAGTGACTTATCAGTTACCCCAGTCTTGGGGTAGTGGCATATTGCAATTAGAATGGTAACTTTATTCACCA 782
Pan     ATGCCACTAACCCAAGACTAGGGTAACTGGAGTGACTTATCAGTTACCCCAGTCTTGGGGTAGTGGCATATTGCAATTAGAATGGTAACTTTATTCGCCA 773
Macaca  ATGCCACTACCCCAAGACTAGGGTAACTAGAGTGACTTATCAGTTACCCCAGTCTTGGGGTAGTGGCATATTGCAATTAGAATGGTACCTTTATTCACCA 733
Bos     AGGCCACCACCTCGAGGCAA-------------------------------------------------------------------GCTTCGTTCACCC 612
Canis   ACGCCCCTCCCCCAAGAATAAG-----------------------------------------------------------------ACTTTATTCACCA 661

Homo    AAGTC--------TGTGTATTCTTGCTCTAAGTGGAGGTGGGGAGGGGAGTAAACTTTGACCACTTTCCTCC-ATTTGGTGGGGTTTTCTCACCCACTAC 873
Pan     AAGTC--------TGTGTATTCTTGCTCTAAGTGGAGGTGGGGAGGGGAGTAAACTTTGACCACTTTACTCC-ATTTGGTGGGGTTTTCTCACCCACTAC 864
Macaca  ACATC--------TGTGTACTCTTGCTCTAAGTGGAGGTGGGGAGAGGAATAAACCTTGACCATTTTTCTCCTGTTTGGTGGGGTTTTCTCACCCACCAC 825
Bos     CTGTCTACTGTGGCCTGTGCTCTTGATCTCAGTGGCGGTGGGGAGGGAAGTAAGACGGGACCCTTTTCCTTGCACTTTCTGAGCTTT---------CTCC 703
Canis   ATGTCTATTATAGTATGTACTCCTGACCTAAGTGCAGGTGGGAAGGGGAGTAAGACATCACCCCTTTCCTTCCATTTTCAG----TCTTTCTCCCCTTCC 757

Homo    CCCCATCCATTTCCATACCCTGCCTCCCCCTCCCAG 909
Pan     CCCCATCCATTTCCATACCCTGCCTCCCCCTCCCAG 900
Macaca  CCCCATCCATTTCCATACCTTGCCTCTCCGTCCCAG 861
Bos     TTCCGTCCACTTCCTCTCCCCGGATGCCC--CTCAG 737
Canis   CCCCATCCATTTCCACTGCCCTTCTTCCCC-CTCAG 792
```

ENSG00000128510 intron 7

Description: Carboxypeptidase A4 precursor (CPA4)
Intron number: 7
Human chromosome: 07
Intron start (bp): 129733974
Human intron length : 1409
Intron alignment length: 1642
Flanking exons length (upstream/downstream): 111/91
SNP density: 0.009936
K tree score: 0.0594
Scaling factor: 1.0905
Human-chimpanzee distance: 0.019556
Total primate branch length: 0.0956

ENSG00000128510 exon 7

```
Homo    ATTGTATCTGATTACCAGAGGGATCCAGCTATCACCTCCATCTTGGAGAAAATGGATATTTTCTTGTTGCCTGTGGCCAA 80
Pan     ATTGTATCTGATTACCAGAGGGATCCAGCTATCACCTCCATCTTGGAGAAAATGGATATTTTCTTGTTGCCTGTGGCCAA 80
Macaca  ATTGTATCTGATTACCAGAGGGATCCAGCTATCACCTCCATCTTGGAAAAAATGGATATTTTCTTGTTGCCTGTGGCCAA 80
Bos     ATTGCATGTGATTATGGGAGAGATCTAGTCATCACCTCCATCTTGGAGAAAATGGATATATTCTTGTTGCCGGTGGCCAA 80
Canis   ATTGTATCTGATTATGGGAAGGACCCAGCTCTCACCTCCATCTTGGAGAAAATGGATATTTTCTTGTTGCCTGTGGCCAA 80

Homo    TCCTGATGGATATGTGTATACTCAAACTCAA 111
Pan     TCCTGACGGATATGTGTATACTCAAACTCAA 111
Macaca  TCCTGATGGATATGTGTATACTCAAACTCAA 111
Bos     TCCTGATGGATATGTGTACACTCATACTCAC 111
Canis   TCCTGATGGATATGTGTACACACAAACTCAA 111
```

ENSG00000128510 exon 8

```
Homo    AACCGATTATGGAGGAAGACGCGGTCCCGAAATCCTGGAAGCTCCTGCATTGGTGCTGACCCAAATAGAAACTGGAACGC 80
Pan     AACCGATTATGGAGGAAGACGCGGTCCCGAAATCCTGGAAGCTCCTGCATTGGTGCTGACCCAAATAGAAACTGGAACGC 80
Macaca  AACCGATTATGGAGGAAGACGCGGTCCCTAAATCCTGGAAGCTCCTGCGTTGGTGCTGACCCAAATAGAAACTGGAACGC 80
Bos     AACCGATTATGGAGGAAGACACGGTCTGTAAATCCTAGAAGCACCTGCATTGGTGCTGATCCAAATAGAAATTGGGATTC 80
Canis   AACCGATTATGGAGGAAGACACGGTCCTTAAATCCTGGAAGTGCCTGCGTTGGTGCTGATCCAAATAGAAATTGGAATGC 80

Homo    TAGTTTTGCAG 91
Pan     TAGTTTTGCAG 91
Macaca  TAGTTTTGCAG 91
Bos     TCATTTTGGAG 91
Canis   TAGTTTTGCAG 91
```

ENSG00000128510 intron 7

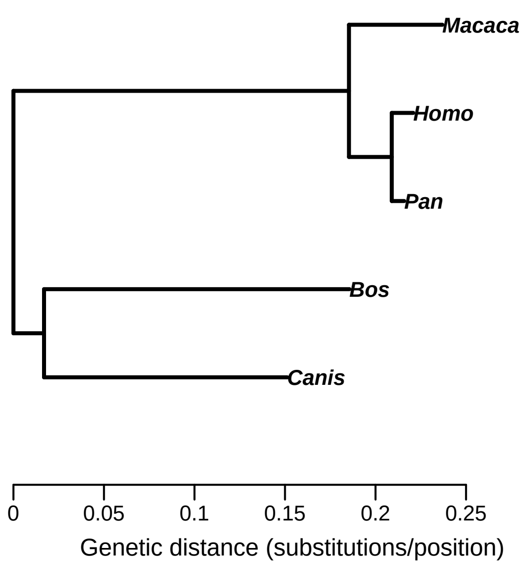

ENSG00000128510 intron 7

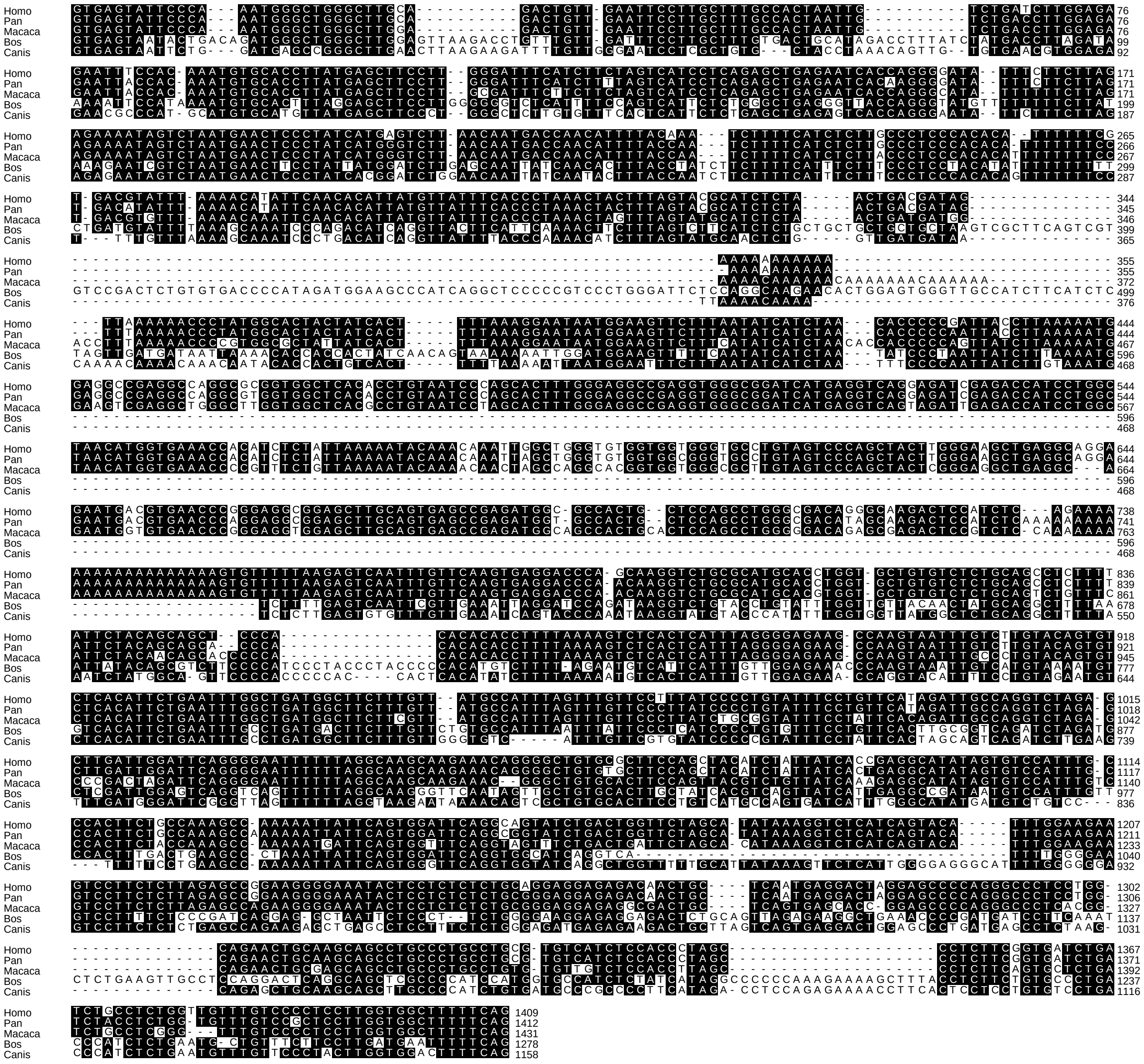

ENSG00000162496 intron 3

Description: Short-chain dehydrogenase/reductase 3 (DHRS3)
Intron number: 3
Human chromosome: 01
Intron start (bp): 12561572
Human intron length : 336
Intron alignment length: 368
Flanking exons length (upstream/downstream): 120/239
SNP density: 0.023810
K tree score: 0.0657
Scaling factor: 0.7087
Human-chimpanzee distance: 0.018161
Total primate branch length: 0.0953

ENSG00000162496 exon 3

```
Homo    GTGGGTGACATCACCATCCTGGTGAACAATGCCGCCGTGGTCCATGGGAAGAGCCTAATGGACAGTGATTGATGATGCCCT  80
Pan     GTGGGTGACATCACCATCCTGGTGAACAATGCCGCCGTGGTCCATGGGAAGAGCCTAATGGACAGTGACGATGATGCCCT  80
Macaca  GTGGGTGACATCACCATCCTGGTGAACAATGCCGCCGTGGTCCACGGGAAGAGCCTAATGGACAGTGACGACGATGCCCT  80
Bos     GTGGGTGACATCACCATCCTGGTGAACAACGCCGCCGTGGTCCACGGGAAGAGCCTGATGGACAGTGACGATGATGCCCT  80
Canis   GTGGGCGACATCACCATCCTGGTGAACAACGCGGCCGTGGTCCACGGGAAGAGCCTGATGGACAGTGACGACGATGCCCT  80

Homo    CCTCAAGTCCCAACACATCAACACCCTGGGCCAGTTCTGG  120
Pan     CCTCAAGTCCCAACACATCAACACCCTGGGCCAGTTCTGG  120
Macaca  CCTCAAGTCCCAACACATCAACACCCTGGGCCAGTTCTGG  120
Bos     TCTCAAGTCCCAGCACATCAACACCCTGGGCCAGTTCTGG  120
Canis   CCTCAAGTCCCAGCACATCAACACACTGGGCCAGTTCTGG  120
```

ENSG00000162496 exon 4

```
Homo    ACCACCAAGGCCTTCCTGCCACGTATGCTGGAGCTGCAGAATGGCCACATCGTGTGCCTCAACTCCGTGCTGGCACTGTC  80
Pan     ACCACCAAGGCCTTCCTGCCGCGTATGCTGGAGCTGCAGAATGGCCACATCGTGTGCCTCAACTCCGTGCTGGCACTGTC  80
Macaca  ACCACCAAGGCCTTCCTGCCGCGTATGCTGGAGCTGCAGAACGGCCACATCGTGTGCCTCAACTCCGTGCTGGCACTGTC  80
Bos     ACCACCAAGGCCTTCTTGCCGCGGATGCTGGAGCTACAGAACGGCCACATCGTGTGTCTGAACTCCGTGTTGGCACTCTC  80
Canis   ACCACCAAGGCCTTCCTGCCCCGCATGCTGGAGCTGCAGAACGGCCACATCGTGTGTCTCAACTCCGTGCTGGCGCTCTC  80

Homo    TGCCATCCCCGGTGCCATCGACTACTGCACATCCAAAGCGTCAGCCTTCGCCTTCATGGAGAGCCTGACCCTGGGGCTGC  160
Pan     TGCCATCCCCGGTGCCATCGACTACTGCACATCCAAAGCGTCAGCCTTCGCCTTCATGGAGAGCCTGACCCTGGGGCTGC  160
Macaca  TGCCATCCCCGGTGCCATCGACTACTGCACATCCAAAGCGTCAGCCTTTGCCTTCATGGAGAGCCTGACCCTGGGGCTGC  160
Bos     TGCCATCCCTGGCGCCATCGACTACTGCACGTCCAAAGCCTCAGCCTTCGCCTTCATGGAGAGCCTGACCCTGGGGCTGC  160
Canis   TGCCATCCCCGGTGCCATCGACTACTGCACGTCCAAAGCCTCGGCCTTCGCCTTCATGGAGAGCCTGACCCTGGGGCTGC  160
```

ENSG00000162496 intron 3

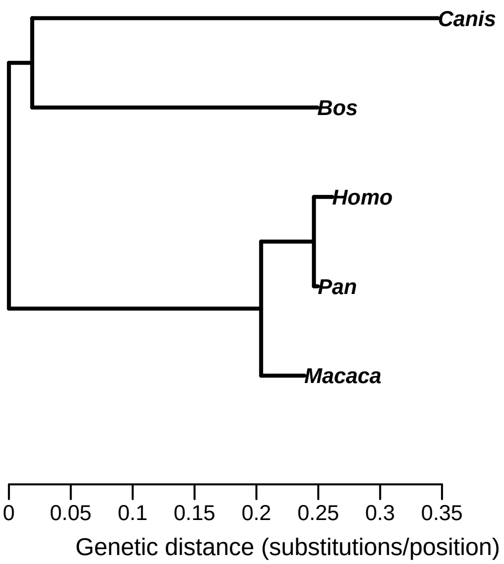

ENSG00000162496 intron 3

```
Homo    GTAAGGG-CTATCCGGGGCCAGAGCCAGCCCCGGGCGTG----------------------CAGGGAGCCCAGGCAGGATGGAGGGGAGGGGATGATTTG 77
Pan     GTAAGGG-CTATCCGGGGCCAGAGCCAGCCCCGGGCGTG----------------------CAGGGAGCCCAGGCAGGATGGAGGGGAGGGGATGATTTG 77
Macaca  GTAAGGG-CTGTCCCAGGCCAGAGCCAGCCCCCGGTGTG----------------------CAGGGAGCCCAGGCAGGATGGAGGGGAGGGGATG-CTTG 76
Bos     GTAA-GG-TTTTCCTGAGCCAGAGCCAGCGCTGGGCATGTCAATGACATGGTCTTCCCTTCCAGGGAGCCTGGGGCTATAGGGGAGTGGGGAGAGGCTCA 98
Canis   GTAAGGGCCTGTCCCGGGCCCCGGGCAGCCCCGGG-GTGACAGTGACCTGTTCCTTCCCTCCTGGGTGCC-------GTGGGGGCGGGGGGAGAGGCTC- 91

Homo    CCAGGGCTTGGGCTCAGGCCAGCAGGGATGTCCACGAACAAGGTGGCCAGACAAGACGAATGACCAG--CTCTAGGATACATC-AGACTTTGTGAAATAT 174
Pan     CCAGGGCTTGGGCTCAGGCCAGCAGGGATGTCCACGAACGAGGTGGCCAGACAAGACGAATGACCAG--CTCCAGGACACATC-AGACTTTGTGAAATAC 174
Macaca  CCAGGGCTTGGGCTCAGGCCAGCAGGGATGTCCACGAACGAGGTGGCCAGACAAGACAAATGACCGG--CTCCAGGACGCATC-AGACTTTGTGAAATGC 173
Bos     CCAGGGCTTGGGCTTGGGTCTAGAG----------------GGTGGCTGGACAAGAAGAATGACAGGCTCTCCAGGAGTCATCTGGACTTTCTGAAATGT 182
Canis   ---------------------AGGGGGATGTCCGAGGACGAGGCGACCTGCCAG---------------CCCCAGGAGACATCCAGACGTACAGAAATGT 155

Homo    ---GCTCGGTGTCCTGGGGCAGGGAGCCCTCGGCCCTCTCCCTCGCAGGAAGGCTCGGGCCCTAGAGCATCATGTGTGTCCGGGGTGGGCTGTTCTCCCC 271
Pan     ---GCTCAGTGTCCTGGGGCAGGGAGCCCACGGCCCTCTCCCTCGCAGGAAGGCTCGGGCCCTAGAGCATCATGTGTGTCCGGGGTGGGCTGTTCTCCCC 271
Macaca  ---GCTCACTGTCCCGGGGCCGGGAGCCCTCAGCCCTCTCCCTTGCAGGAAAGCTCGGGTACTAGAGCATCGTGTGTGTCCAGGGTGGGCTGTTCTCCCC 270
Bos     GGGGGTTGGTGCCCTCAGCCTCCACCCCATCA--CCTCCCCATCGCAGGAAAACCCAGTGGCTGGAGGGGTGCCCGTGTCAAGGGTAGTCTGTTCTCCCC 280
Canis   GGAGGTCAGCGCCCTCGG-------------CGCCCCCTCCCGTCCTAGGAGTGCCCGCCACCGGAGGGGTGCATGTGCCCGGGGCGGCCTGTCCTCCCC 243

Homo    GAAAGAA-AGGTGGAGGGGTTGTCAG-AGCTGCGGCTGCTCCACCCTGA-CACCCCATCTGCCCTCAG 336
Pan     GAAAGAA-AGGTGGAGGGGTTGTCAG-AGCTGCGGCTGCTCCACCCTGA-CACCCCATCTGCCCTCAG 336
Macaca  AAAAGAA-AGGTGGAGGGGTT-TCAG-AGCTGCGGCTGCTCCACCCTGA-CACTCCATCCACCCTCAG 334
Bos     AAGAGGGTAAGTCGAGGGGCTGTTGGAAGCTGTGTCTGCCGCACACTAACCTCCCCATCCACCCTCAG 348
Canis   AGAAGCT----TAGAGGGGCTGCCGGAGGCCGCGG-TGCCCCACACTGA-CCTCCCGCCCGCCCCCAG 305
```

ENSG00000148297 intron 1

Description: Surfeit locus protein 5 (MED22)
Intron number: 1
Human chromosome: 09
Intron start (bp): 135201929
Human intron length : 1287
Intron alignment length: 1765
Flanking exons length (upstream/downstream): 123/81
SNP density: 0.010101
K tree score: 0.0866
Scaling factor: 0.4629
Human-chimpanzee distance: 0.014928
Total primate branch length: 0.0951

ENSG00000148297 exon 1

```
Homo    ATGGCCCAGCAGAGAGCCCTGCCCCAGAGCAAGGAGACGCTGCTGCAGTCCTACAACAAGCGGCTGAAGGACGACATTAA 80
Pan     ATGGCCCAGCAGAGAGCCCTGCCCCAGAGCAAGGAGACGCTGCTGCAGTCCTACAACAAGCGGCTGAAGGACGACATTAA 80
Macaca  ATGGCCCAGCAGAGAGCCCTGCCCCAGAGCAAGGAGACGCTGCTGCAGTCCTACAACAAGCGGCTGAAGGATGACATTAA 80
Bos     ATGGCCCAGCAGAGAGCCCTGCCGCAGAGCAAGGAGACGCTGCTGCAGTCTTACAACAAGCGGCTAAAGGACGATGTCAA 80
Canis   ATGGCCCAGCAGAGAGCCCTGCCGCAGAGCAAGGAGACGCTGCTGCAGTCCTACAACAAGCGGCTCAAGGACGATGTCAA 80

Homo    GTCCATCATGGACAACTTCACCGAGATCATCAAGACCGCCAAG 123
Pan     GTCCATCATGGACAACTTCACCGAGATCATCAAGACCGCCAAG 123
Macaca  GTCCATCATGGATAACTTCACCGAGATCATCAAGACCGCCAAG 123
Bos     GTCCATCATGGACAACTTCACTGAGATCATCAAGACCGCCAAG 123
Canis   GTCCATCATGGACAACTTCACCGAGATCATCAAGACCGCCAAG 123
```

ENSG00000148297 exon 2

```
Homo    ATTGAGGACGAGACGCAGGTGTCACGGGCCACTCAGGGTGAACAGGACAATTACGAGATGCATGTGCGAGCCGCCAACAT 80
Pan     ATTGAGGACGAGACGCAGGTGTCACGGGCCACTCAGGGTGAACAGGACAATTACGAGATGCATGTGCGAGCCGCCAACAT 80
Macaca  ATTGAGGACGAGACGCAGGTGTCACGGGCCACTCAGGGTGAACAGGACAATTACGAGATGCACGTGCGAGCCGCCAACAT 80
Bos     ATTGAGGACGAGACGCAGGTGTCAAGGGCTACTCAGGGCGAGCAGGACAACTACGAGATGCACGTGAGAGCTGCCAACAT 80
Canis   ATCGAGGACGAGACACAAGTGTCGAGAGCCACTCAGGGCGAGCAGGATAATTACGAGATGCATGTTCGAGCTGCCAACAT 80

Homo    C 81
Pan     C 81
Macaca  T 81
Bos     C 81
Canis   C 81
```

ENSG00000148297 intron 1

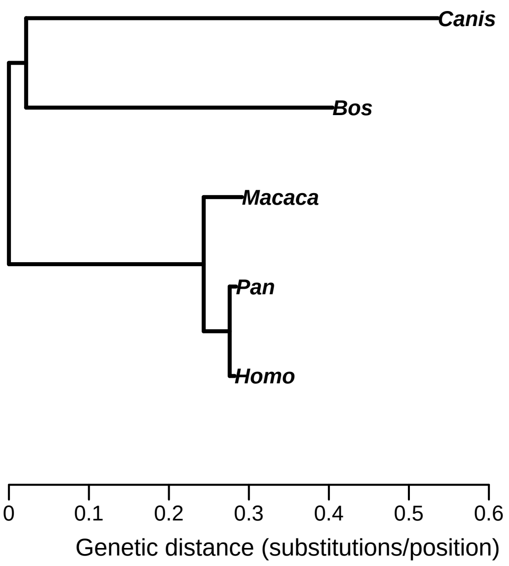

ENSG00000148297 intron 1

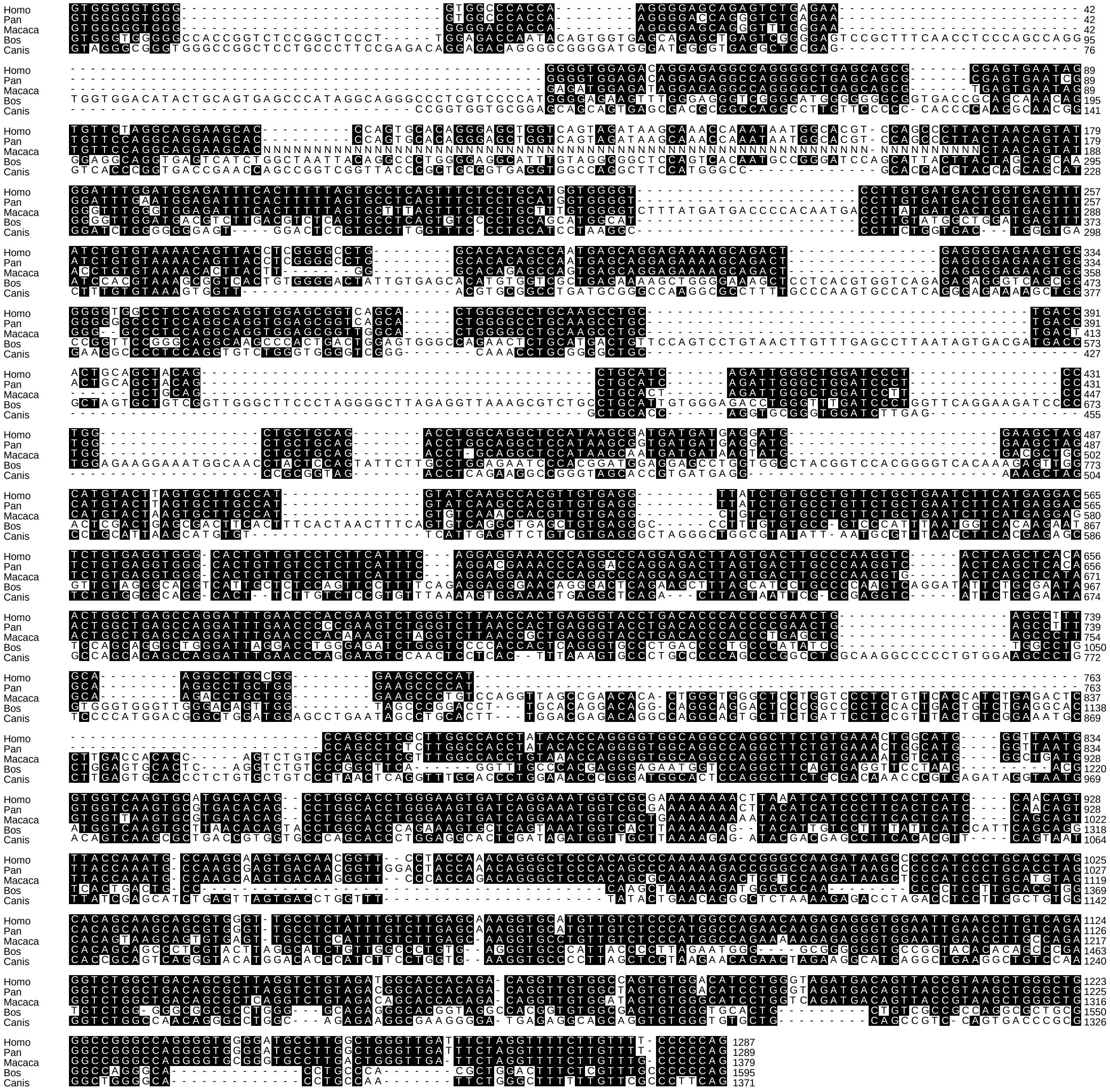

ENSG00000116977 intron 2

Description: Galectin-8 (LGALS8)
Intron number: 2
Human chromosome: 01
Intron start (bp): 234767509
Human intron length : 1293
Intron alignment length: 1333
Flanking exons length (upstream/downstream): 89/211
SNP density: 0.020108
K tree score: 0.0865
Scaling factor: 0.7162
Human-chimpanzee distance: 0.030778
Total primate branch length: 0.095

ENSG00000116977 exon 2

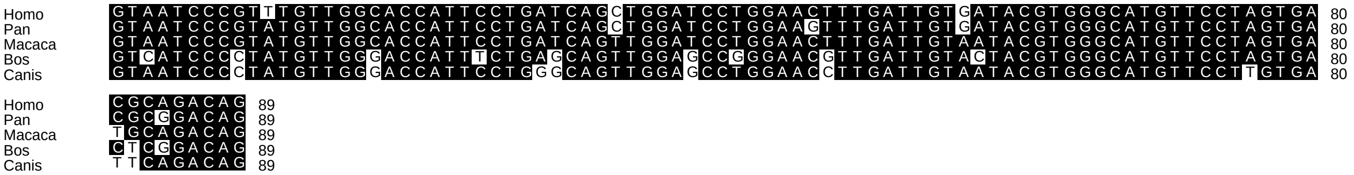

ENSG00000116977 exon 3

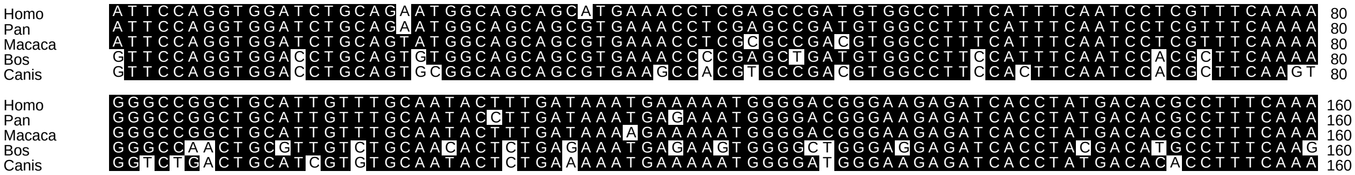

ENSG00000116977 intron 2

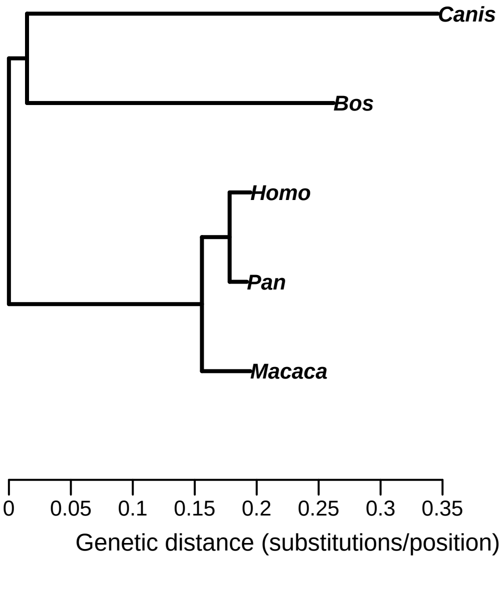

ENSG00000116977 intron 2

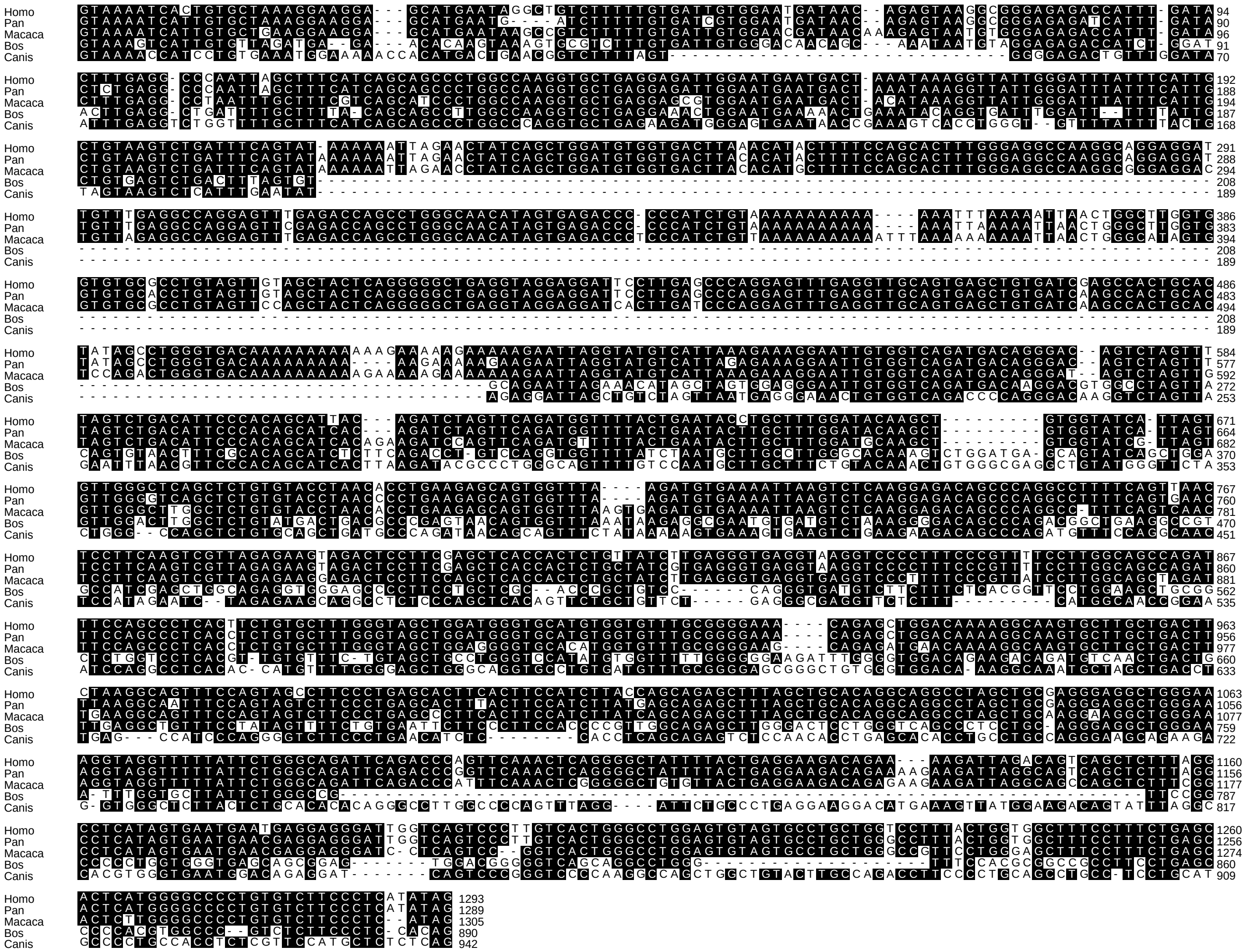

ENSG00000116977 intron 3

Description: Galectin-8 (LGALS8)
Intron number: 3
Human chromosome: 01
Intron start (bp): 234769013
Human intron length : 1474
Intron alignment length: 1743
Flanking exons length (upstream/downstream): 211/120
SNP density: 0.028494
K tree score: 0.087
Scaling factor: 0.4233
Human-chimpanzee distance: 0.017971
Total primate branch length: 0.0949

ENSG00000116977 exon 3

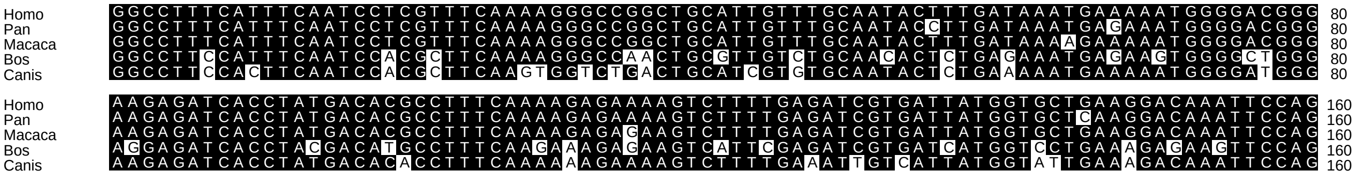

ENSG00000116977 exon 4

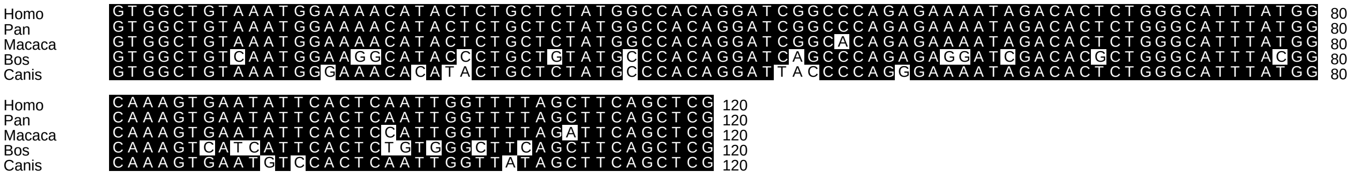

ENSG00000116977 intron 3

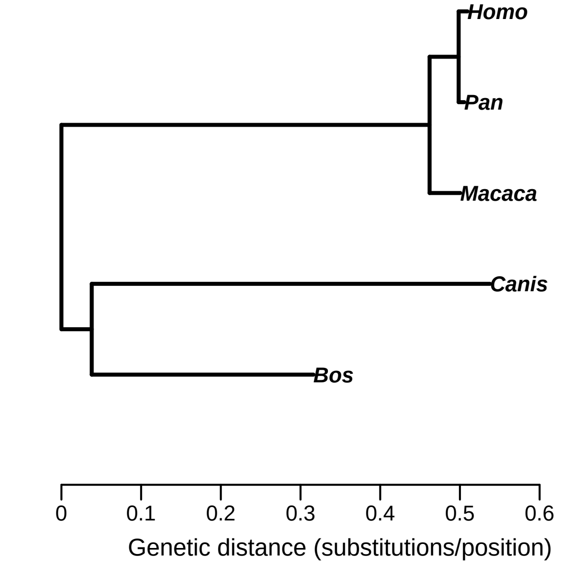

ENSG00000116977 intron 3

```
Homo    GTAGGTTTTGGAGAGGGACAGGTTGAGTCCTCATTAGTGAGCAGGAGTGCACAGGGGTGCTTTTCACATTTGTGAGCCCAGCCTTATATTTCCTACACCT 100
Pan     GTAGGTTTTGGAGAGGGACAGGTTGAGTCCTCATTAGTGAGCAGGAGTGCACAGGGGTGCTTTTCACATTTGTGAGCCCAGCCTTATATTTCCTACACCT 100
Macaca  GTAGGTTTTGGAGAGGACCAGGTTGAGTCCTCATGAGCGAGCGGGACTGCACAGGGGCGCTTTTCACGTTTGTGAGCCCGGCCTTCTATTTCCTACACCT 100
Bos     GCAGGTTTTGGAGGGTGG-AGGTTGACTCCTCACTCATGA-CAGTGAGCATGAGGGAAGCCCTTCACACACGTGAACTCGCCCATACATCTCCTAAATCC 98
Canis   GTAGGTTTTGGAGAATGA-AGATGAAATCCTCATTAATGA-CATAAGCGCCAGGGTGGGTCAGCACATCAGTGGACTCACCCTCCTGTTACTTCAGTCC 98

Homo    GAGATATAGTTTGTTTGGCTTTGTAGTTTTTCTCCATAAAAGGACCAGG-----AAGGCACCTAAATGTGAGGTATGGCACCACTACTCTCCAGCCAGTT 195
Pan     GAGATATAGTTTGTTTGGCTTTGTAGTTTTTCTCCATAAAAGGACCAGG-----AAGGCACCTAAATGTGAGGTATGGCACCACTACTCTCCAGCCAGTT 195
Macaca  GAGACATGGTTTGTTTGGCTTTGTAGTTTTTCTCCATAAAAGGACCAGG-----AAGGCACCTAAATGTGAGGTGTGGCACCACTACTCTCCAGCCAGTT 195
Bos     AGAGGAAGG---CTTTGGCTTTATAACTCTCCTCAGAAGTAGGGCACGG-----CGGGTACCTTCCCTTGAGGTGGG------CACAGCCTGGCTCCTGT 184
Canis   AGAGAAAAA---CAGTGGCTTTATCATTTTGCTCTAAAGTAGGGAGGGGCCAACCGGGTACCTAAATGTGAGATGGGGCACACCTCCATCTGCCCCTGGT 195

Homo    GTTGCCATGCAGAAATATGGTCCACTGTGACCAGATC---TTTTTATTAGATCCTATTTCTCCTAGCAGGGCTGAGTTC-----CGAATTGACACAGTAT 287
Pan     GTTGCCATGCAGAAATATGGTCCACTGTGACCAGATC---TTTTTATTAGATCCTATTTCTCCTAGCAGGGCTGAGTTC-----CGAATTGACACAGTAT 287
Macaca  GTTGCCGTGCAGAAATATGGTCCATTGTGACCAGATC---TTTTTATTAGATCCTGTTTCTCCTAGCAGGGCTGAGTTC-----TGAATTGACACAGTAT 287
Bos     GTCCCTGCTCATGTCACCATGCAGGTCCAGTGTGACTGGATCTTTGCTGGATT---GTTCTCCTTGCAGGACTGAGTTC-GTTCTGAACTGGCTCAGGCT 280
Canis   GTTTCATCTAGAATATGGATGCACG-GCAGAGAAACT---TTTTTATTGGATCCCATTTCTGCTAGCTCAGCTCAGCTTAGGTCTGAACTGGCACAGGAA 291

Homo    TATGTTCATGATGGGAGGGTAAG-----------TTATAATATAACCGCCACCACCCGAAG------------------AGCTAACAAGGGCAATCCCAG 358
Pan     TATGTTCATGATGGGAGGGTAAG-----------TTATAATATAACCGCCACCACCCGAAG----------------------AACAAGGGCAATCCCAG 354
Macaca  TATGTTAATGATGGGAGGGTAAG-----------TTATAATATAACCGCCACCATCCGAAG------------------AGCTAACAAGGGCAATCCTAG 358
Bos     TACAC---TGATGATGAAGA------------GTTAACTGCAGGCACTAGAACCCTGAGTGTGGGGAGTCTGAACTGACAGACACATGGGATCCTGT 363
Canis   TAAATATGGGATAGTAAAAAAAAAAAAAGGGGATAGTAAAACTGGTCACCATGACCCAAAGTGTCAGGGCATTTGTACTAGTAGATGGATAGGAAGTCAA 391

Homo    CGAAGAAATC-------AGAAGGGTTTTGTAAATTCAAGTCTTGCCACAAGACAGTTCCGTAGGATCATGAGATTTTAGACCCAGAGGAGATCCTAGAAA 451
Pan     CATAGAAATC-------AGAAGGGTTTTGTAAATTCAAGTCTTGCCACAAGACAGTTCTGTAGGATCATGAGATTTTAGACCCAGAGGAGATCCTAGAAA 447
Macaca  CATAGAAATC-------AGAAGGGTTTTATAAATTCAAGTCTTGCCACAAGATAGTTCTGTAGGATC--GAGAGTTTAGATCCAGAGGAGATCCTAGAAA 449
Bos     CGTTGGGGCC-------AGGGGGGTTGTTCAGTTCC---GTCCTGTGCTGCCTCGTCCCGTAGATCC---AGAGATTCTAATCAGAGGAGATCTTAGAAA 450
Canis   AGTTAAATCCTGCCATAAGTTTGTTTGTTTGTTTTT---TTTTTTTTTAGATTTTATTTATTTATTTCATGAGAGACACACACAGAGAGAGAGAGAGGCGG 488

Homo    TCCTTGATGTCAGTTCCATCTCTGGCTTCATGG---AGTGTCTTGTACCTAGCGTGTATGTGTACGGTTGAATTTGGTCCCAGAAG--CTTAC------- 539
Pan     TCCTTGATGTCAGTTCCATCTCTGGCTTCATGG---AGTGTCTTATACCTAGCGCGTATGTGTACGGTTGAATTTGGTCCCGGAAGCTCTTAC------- 537
Macaca  TCCTTGATGTCACTTCCATCTCTGGCTTCATGG---AGTGTCTTATACCTAGCGCGTATGTGTACGGTTGAATTTGGTCCTAGAGGCTCTTAC------- 539
Bos     GCCCACGTATCGTCAGGACTGCTG--------------AGCACTTATCCCTAGTG----TGTGCAGAGGTGGAATGTGGTCCCGAGGCTGCTCCNNNNNNN 534
Canis   AGACACAGGCAGAGGGAGAAGCAGGCTCCATGCAGTGGGACTCAATCCC--GGG----TCTCCAGGATCATGCCCTGGGCTGCAGGCGGCGCT------- 575

Homo    -------------------------------------ACCTGCTGGCCCTCTGGCCTGTGGAGCTTGCCCACAGTAGAGGTATGTACCAACGCGAGAGAA 602
Pan     -------------------------------------ACCTGCTGGCCCTCTGGCCTGTGGAGCTTGCCCACAGTAGAGGTTTGTACCAACGCGAGAGAA 600
Macaca  -------------------------------------ACCGGCTGGCACTCCGGCCTGTGGAGCTTTCCCAGGGTAGAGGTTTGTACCAGTGTGAGAACA 602
Bos     NNNNNNNNNNNNNNNNNNNNNNNNNNNNNNNNNNNNNNNNNNNAAACCTCCCCTCCCCCACC--CCTCCTCTTCTGGCTGTTGGAGTCCT------- 625
Canis   -------------------------------------------AAACCACTGCGCCACCGGGGCTGCCCCCTGCCACAAGTTCTTTAGGATTT------- 625

Homo    GACTCGAATGCCTCTGGCGCAGATCCTTTCTGATCTTCGGGATACTGCTCCTGCCCGAAAGTCTTTCTGAATCTCCCAAACTCCATTCACCTCTCCCTTC 702
Pan     GACTCACATGCCTCTGGCGCAGATCCTTTCTGATCTTCGGGATACTGCTCCGCCCCGAAAGTCTTTCTGAATCTCCCAAACTCCATTCACCTCTCCCTTC 700
Macaca  GACCCACATGTCTTTGGCACAGATCCTTTCTGATCTTTAGGATACCGCTCCCGCCCAAAAATCTTTCTGAATCTCCCAAACTCCATTCACCTCTCCCTTC 702
Bos     --------TGCGTCTGTATCATTTTTTTT----------------------------------------------------------------------- 647
Canis   --------TATTTTTAAAAAAGATTTTATTTATTCATTCATGAGAGACACAGAGAGAGGCAGAGACATAGGCAGAGGGAGAAGCA--------AGCTTTC 709

Homo    TCTGGCCTTTTGAGCCCGTGTCTGTATCATTCTTTTTCA-CAGTTTTTAACAGTTGTGCTTTGGCTTTACGTGTTTATTTTGCCTCCACAATGGGATTTT 801
Pan     TCTGGCCTTTTCAGCCCATGTCTGTATCATTCTTTTTCA-CAGTTTTTAACAGTTGTGCTTTGGCTTTATGTGTTTATTTTGCCTCCACAATGGGATTTT 799
Macaca  TCTGGCCTTTCCAG-CCGTGTCTGTATCATTCTTTTTAACCATTTTTTAACAGTTGTGCTTTGGCTTGATGTGTTTATTTTGCCCCCACAATGGGATTTG 801
Bos     -----------AACGCCTGTCTGTACTGT---------TGTGCTTTGGTTTTTTTTCCT---------GCCTGCCTTTTGCCTTC-CATTGGGATTTG 715
Canis   TGCAGGGGCCCCAATGTAGGACTAGATCTC----------AGGACCCCAGGATCACTACCTGAGCCAAAGGCAGACATCTGCTCAAC-TATTGAGCCACC 798

Homo    AAG------CTCCCTGAGTCAGAGACTATATTGTATGCTGCTCGCGTTTTCTGCCTATAACCTAACGTGGTA---------------------------- 867
Pan     AAG------CTCCTTGAGTCAGAGACTATATTGTATGCTGCTCGTGTTTTCTGCCTATAACCTAACGTGGTA---------------------------- 865
Macaca  AAG------CTCCTTGAGTCAGAGACTGTATTGTATGCTGCTTGCGTTTTCTGCCTATAACCTAACCTGGTA---------------------------- 867
Bos     CAG-----CTCCCTGGGGTCTGAGAC--CATACTGTGTTCTGCGTGTCTTCCTGACACTCCGTACACGTGTA---------------------------- 780
Canis   CAGGCACCCTCTTTAGGATTTCAGGTGAGAGATTTAGATGCAGAGGAGTTCTTAGAAGTCCG---ATGTGTAGTAAACACCAGTGGCTCCAAGCGGTAAA 895

Homo    ------CCTGGCATTTGAGAGGGAGGAGGAGGAGCCTCGTAGCGTGCCG------AGGACCTGCA----------------------------------- 921
Pan     ------CCTGGCATTTGAGAGGGAGGAGGAGGAGCCTTGTAGCGTGCCG------AGGACCTGCA----------------------------------- 919
Macaca  ------CCTGGCATTTGAGAGGGAGGAGGAGGAGCAACGTAGAGTGCTG------AGGACCTGCG----------------------------------- 921
Bos     -------CTGGCATCTGGGAGGGAGGAAGAAGGAGCCCGGAGCACTGGTGCCCTCTCGGTCAGGCA--------------CTGGGCTGGAGTCTCAC--- 856
Canis   TACCTAGCCAGTGTTAAGTGTGAGGTAGAAGGTGGCCTGGAGAGGCTCTGGCCCACAGGCCTTGTAGAACCTTGAGCCTCCCAGACTAGAGTTGCTCCGC 995

Homo    --GAAGCTACTTTCTCGTCATCT---TACTG---------TAGTCTGTTGAGGTAGAGACTG-------------------------------------- 969
Pan     --GAAGCTACTTTCTCGTCATCT---TACTG---------TAGTCTGTTGAGGTAGAGATTG-------------------------------------- 967
Macaca  --GAAGCTACTTTCTCGTCATCTTGCTACTG---------TAGTCTGTTGAGGTAGAGATTG-------------------------------------- 972
Bos     -GAGAGGGACGGGGTCATCTCCTCCCAACAG---------CAGCGTCTGGAGGCGCATGC---------------------------------------- 907
Canis   TGAGAGTGAGAATTTCCCCTCTTCTTGTGTGGGTCCTTTCTGGCCTTTGGGATATGCTCCCACCAAAACACTTCCCCTGAATCTCCCAAACTCTATTCAT 1095

Homo    --------TTCCTACTTCAGAATAAGAAAACCGAATTC---AAATATGTTGGGTAACTTGTCCATATTAA-TTTATTTAGCAAATACAACAGATTTTGAG 1057
Pan     --------TTCCTACTTCAGAATAAGAAAACCGAATTC---AAATATGTTGGGTAAGTTGTCCAT---A-TTTATTTAGCAAATACAACGGATTTTGAG 1051
Macaca  --------TTCCTACTTCAGAATAAGAAAACCGAATCTTAAAATATGTTGAGTAACTTGTCCATATTAGTTTTATTTAGCAAAGTCAACAGATTTT--- 1061
Bos     --------TTCCTGCTCACAGATAGGAATTTC---------AGAGAACTTGGGTCATTGCCTGGGTTCA-TTCATTCAGCAAATTACCAAATGTG--- 986
Canis   CCCTCCCTTCCTGGCTTACAATAGGAATCTC---------AGATAAACTCAGTAGCTTGCCCACGTCCG-TTCATTGATCAGATTCAACAAATATT--- 1182

Homo    TGTCTGCCACATGGTGGTCTCCAGGGACAGTGTTGTGGGGAGCTCGCAGGC-AGATCTTTAACCTGGGTTCACAATCTCCAGGGCA--CCTGTGCCTGG 1154
Pan     TGTCTGCCACATGGTAGTCTCCAGGGACAGTGTTGTGGGGAGCTCGCCGGC-AGATCTTTAACCTGGGTTCACAATCTCCAGGGCA--CCTGTGCCTGG 1148
Macaca  -----------------------------------------------------GATCTTTAACCTGGGTGCACGACCTCCAGGGCA--CCTGTGCCTGG 1105
Bos     --TGTGGAGCACCTGCCTTGTGCGGGGTGGTCTCC---AGAGTCTGGTCCTC-AGGACAGCA----GGGTGATGAGGTCGTGGGGCAGGTCTCTGCCAGG 1076
Canis   --TATTGAACGCCTGCCATGTGCAAGGTGGTCTTCCTTAGAATGTGGTCCTCAAGGACAGCAGGTGGGGTGGCAAGCTTGCAGGGCAGATCGCAAACCAG 1280

Homo    GCTTCCAGGCGACCTTCGAACCCAGATGTCTCACATGTATGCAGAGGC-GCACACAAGCACACGCA-------CATATACTTATGACTGGCT-GTTTGTC 1245
Pan     GCTTCCAGGCGACCTTCGAACCCAGATGTCTCACATGTATGCAGAGGC-GCACACAAGCACACACACATATACCATATACTTATGACTGGCT-GTTTGTC 1246
Macaca  GCTTCCAGGAGACCTTCGAACCCAGATGTCTCACATGTGTGCAGAGGC-TCACAGAGGCACACGCA-------CCTGGATTTGTGACTGGCT-GTTTGTC 1196
Bos     GCTCATGGGCACCCA-----------GGTGTCTCATGTACAGGCCTGCTACACATGGGCATGTGGT-------CGTGTGTTCACCGATGTGTGATCCATC 1159
Canis   GGTCTTCACAGGCCT-----------CGACCATCCGTGTATACACATGC-----------------A-------CATACATTCATGAATGCATCATTTGTC 1346

Homo    TGGGGAGAGACAGTTCCTGGTGCTTAATCAAATCAGGAACTCAAAAGAAGTTTGGAAGCACTGCTAGTGTTTTGGGTGCTTTCGGTTACCATTTGGTC-- 1343
Pan     TGGGGAGAGACAGTTCCTGGTGCTTAATCAAATCAGGAACTCAAAAGAAGTTCGGAAGCACTGCTAGTGTTTTGGGTGCTTTCGGTTACCATTTGGTC-- 1344
Macaca  TGGGGAGAGACAGTTCCTGGTGCTTAATCAGATCAGGAACTC----GAAGTTCAGAAGCACTGCTAGTGTTTTGGGTGCTTTGGTTACAGATGTGGTCAG 1292
Bos     TGGAGAGAG--TGTCCCTGGTGCTCAGTCAGAGGAGAACTCAGAGGAAGTTAAGAGTGCTGTCT---GCTCTGGTTGCTTTC-ATCACAGTTTGGTCAG 1253
Canis   CGGGGGGAG--GATCCATGCTAATGAATCAGACCAGGACTCAGAGGAAGTTAAGAAACACCACTAGAGTTATGGATGCTTTC-A---------GGTCAG 1434

Homo    ATGTG---TGTGGAGACCTGTGGGAACAGGTATAAAACTGGACGCAAGGAAACATTTAAATTTGGATAATAAGTTA------ATTTATTAACTG--TTTT 1432
Pan     ACGTG---TGTGGAGACCTGTGGGAACAGGTATAAAATTAGACGCAAGGAAACATTTAAATTTGGATAATAAGTTA------ATTTATTAACTGTTTTTT 1435
Macaca  ATGTG---TGTGGAGACCTGTGGGAAAGGTATAAAATTGGATGCAAGGAAAC------ATTTGGATAAGAAGTTA------ATTTATTAACTTCTTTTT 1377
Bos     ACGTGTCATGGGAGACCTGTGGGAAAAAGTCTCAAAATCTGAGTCAGCAG--ATTTAAACTTGGATAAATTAATAAAATTCATTTATTAACTGCTTTTAT 1351
Canis   ACATACAATGTAGAGACCTGTGGGAAAAGGTATAAAATAAGCAAAAGTAAACATTTAAATTTGGATACATTAATA-----CATGTATTAACTGTTTTTTT 1529

Homo    TTTTTGGT-GGCGGGGGGGGCTCTGTCTTCTGTATCTCTCTAG 1474
Pan     TTTTTGGT-GGC-GGGGGGGCTCTGTCTTCTGTATCTCTCTAG 1476
Macaca  TT-----------GGGGGGGGTGTCTTCTGTATCTTCTAG 1408
Bos     T-------GGAGGGGGCT--GTTGTCTTC--TGTTGTTCAG 1382
Canis   TTTTTCCCCTGGGGGTGCTGGGCTGTCTTCTGTGTCCACATAG 1572
```

ENSG00000147378 intron 4

Description: Fetal and adult testis-expressed transcript protein (FATE1)
Intron number: 4
Human chromosome: X
Intron start (bp): 150641110
Human intron length : 646
Intron alignment length: 811
Flanking exons length (upstream/downstream): 79/132
SNP density: 0.000000
K tree score: 0.0585
Scaling factor: 0.5268
Human-chimpanzee distance: 0.014105
Total primate branch length: 0.0949

ENSG00000147378 exon 4

```
Homo    CAACCCAGGGACAGATGCAGTGGCGCAGACTAGCCTGGAAGAGTTCAATGTACTGGAGATGGAAGTCATGAGAAGACAG 79
Pan     CAACCCAGGGACAGATGCAGTGGCGCAGACTAGCCTGGAAGAGTTCAATGTACTGGAGATGGAGGTCATGAGAAGACAG 79
Macaca  CAACCCAGGTACAGATGCAGTGGCACAGACTAGCCTGGAAGAGCTCAATGTACTGGAGATGGAAGTCATGAGAAGACAG 79
Bos     ---CCGAGAGTTAAATACACTGGCTGAGATTGGTCTGGAAGAGCTCAATGAGCTGGAGATGGAGATCATGAGAAGACAG 76
Canis   CAACCCAGAGACTGATATGATGACAGAGATTGGCCTAGAAGAGCTCAATGGACTGGAGATGGAAGTCATGAAAAGGCAG 79
```

ENSG00000147378 exon 5

```
Homo    CTGTATGCAGTCAACCGGCGTCTGCGCGCCCTGGAGGAACAGGGCGCCACCTGGCGCCACAGGGAGACCCTGATCATCGC 80
Pan     CTGTATGCAGTCAACCGGCGTCTGCGCGCCCTGGAGGAACAGGGCGCCACCTGGCGCCACAGGGAGACCCTGATCATCGC 80
Macaca  CTATATGCAGTCAACCGGCGTCTGCGCGCCCTGGAGGAGCAGGGCGCCACCTGGCGCCAGAGGGAGACCCTGATCATCGC 80
Bos     CTGTTCATGATTACTGAGCGTCTGCGCTACCTGGAGGACCAGAGTGCCACCTGGCACCAACGGGAGGTGCTGTTATTCAC 80
Canis   CTGCAGGTGATCACCAGCCGCCTGCGAGCCCTGGAGGACCAGGGCGCCACCTGGCGCCACAGGGAAGCTCTGTTCTTCAC 80

Homo    CGTGCTGGTGTCGGCCAGCATTGCCAACCTGTGGCTGTGGATGAACCAGTGA 132
Pan     CGTGCTGGTGTCGGCCAGCATTGCCAACCTGTGGCTGTGGATGAACCAGTGA 132
Macaca  CGTGCTGGTGTCAGCCAGCATAGCCAACCTGTGGCTGTGGATGAACCAGTGA 132
Bos     CATGCTGCTGTCTTCCTGCATCACCAACCTCTGGCTGTGGATGCGCCAGTGA 132
Canis   CGTGCTGGTGTCGGCCTGCATTGCCAACCTGTGGCTGTGGATGCGCCAGTGA 132
```

ENSG00000147378 intron 4

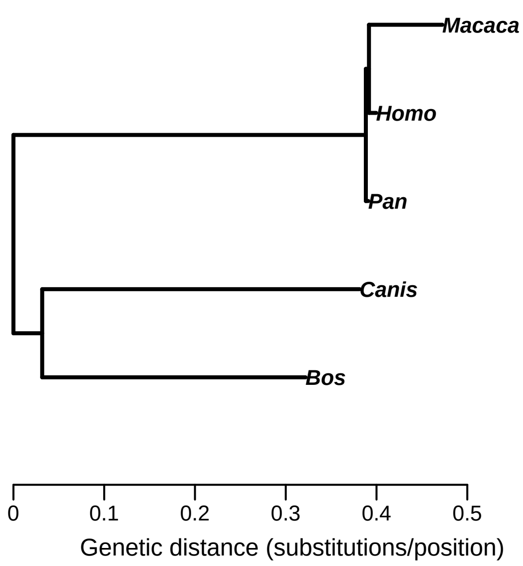

ENSG00000147378 intron 4

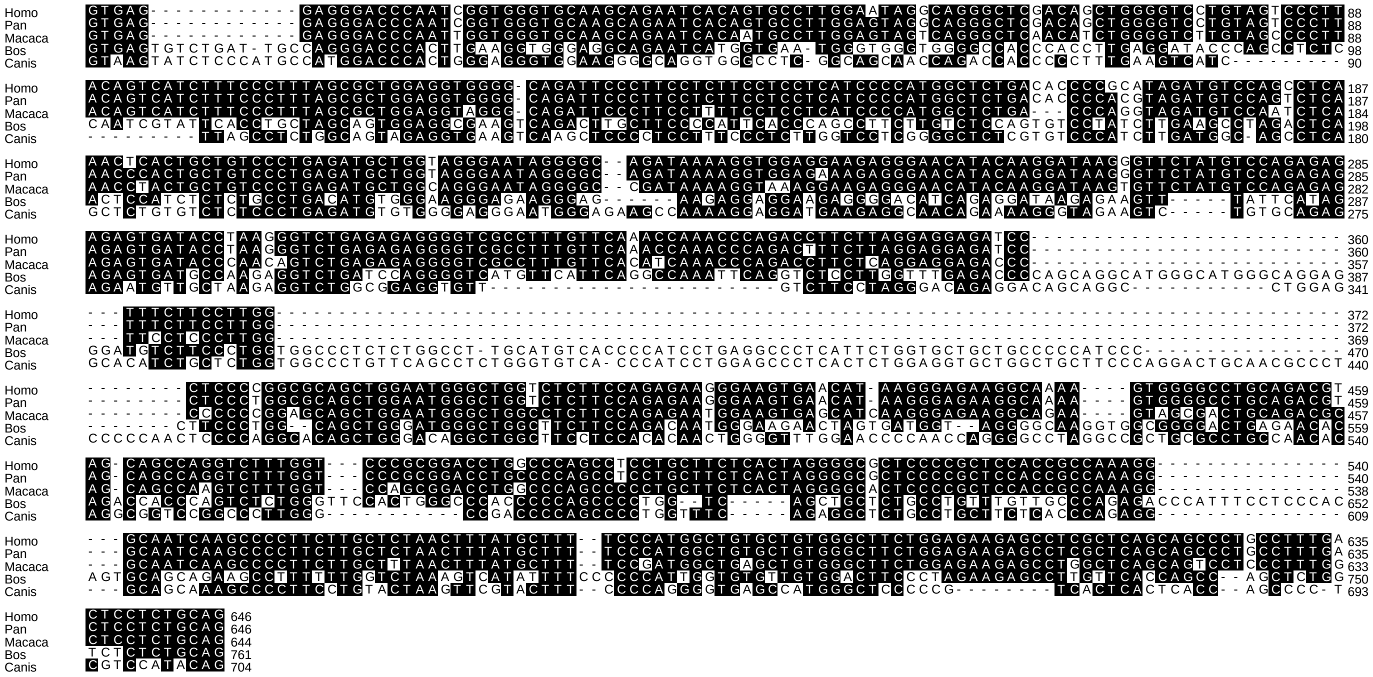

ENSG00000102970 intron 2

Description: Small inducible cytokine A17 precursor (CCL17)
Intron number: 2
Human chromosome: 16
Intron start (bp): 56006612
Human intron length : 566
Intron alignment length: 642
Flanking exons length (upstream/downstream): 118/97
SNP density: 0.000000
K tree score: 0.0607
Scaling factor: 0.5481
Human-chimpanzee distance: 0.021724
Total primate branch length: 0.0949

ENSG00000102970 exon 2

```
Homo    CTCGAGGGACCAATGTGGGCCGGGAGTGCTGCCTGGAGTACTTCAAGGGAGCCATTCCCCTTAGAAAGCTGAAGACGTGG 80
Pan     CTCGAGGGACCAATGTGGGCCGGGAGTGCTGCCTGGAGTACTTCAAGGGAGCCATTCCCCTTAGAAAGCTGAAGACGTGG 80
Macaca  CTCGAGGGACCAACGTGGGCCGGGAGTGCTGCCTGAAGTACTTCAAGGGAGCCATTCCCCTTAGAAAGCTGAAGACGTGG 80
Bos     CTCGAGCAGGCAACGTGGGCCGGGAGTGCTGCCTGCAGTTCTACAAAGGATCCATTCCCCAAAAGGTGCTGGTGGGTTGG 80
Canis   CTCGAGGCACCAACGTGGGCCGGGAGTGCTGCCTAGAGTACTTCAAAGGAGCCATTCCTATCAGCAGGCTGACAAGGTGG 80

Homo    TACCAGACATCTGAGGACTGCTCCAGGGATGCCATCGT 118
Pan     TACCAGACATCTGAGGACTGCTCCAGGGATGCCATCGT 118
Macaca  TACCAGACATCCGAGGACTGTTCCAGGGATGCCATTGT 118
Bos     TACCAGACCTCGGACGATTGTCCCAACAAAGCCATTGT 118
Canis   TACAAGACTTCAGGGGAGTGTCCCAAGGATGCCATCGT 118
```

ENSG00000102970 exon 3

```
Homo    TTTTGTAACTGTGCAGGGCAGGGCCATCTGTTCGGACCCCAACAACAAGAGAGTGAAGAATGCAGTTAAATACCTGCAAA 80
Pan     TTTTGTAACTGTGCAGGGCAGGGCCATCTGTTCGGACCCCAACAACAAGAGAGTGAAGAATGCAGTTAAATACCTGCAAA 80
Macaca  TTTTGTAACTGTCCAGAACAAGGCCATCTGTTCGGACCCCAACGACAAGAAAGTGAAGAAGGCACTTAAATACCTGCAAA 80
Bos     GCTGGTGACTCGCTCTGGTAGAACCATTTGTGCAAACCCCAAGGACAAAACGGTGAAGAAGGCAATGAAATACTTGCAGA 80
Canis   GTTTGTAACTGTCCAAGGCAAGTCCATCTGTTCCGACCCCAAGGACAAGAGGGTGAAGAAGGCGGTCAGATATTTACAAA 80

Homo    ---------------GCCTTGAGAGGTCTTGA 97
Pan     ---------------GCCTTGAGAGGTCTTGA 97
Macaca  ---------------GCCT---------CTAG 88
Bos     ---------------------AACAGAAGAAG 91
Canis   GAACCTGGAAGGGAGGGCCCCAAGAGTCCTGA 112
```

ENSG00000102970 intron 2

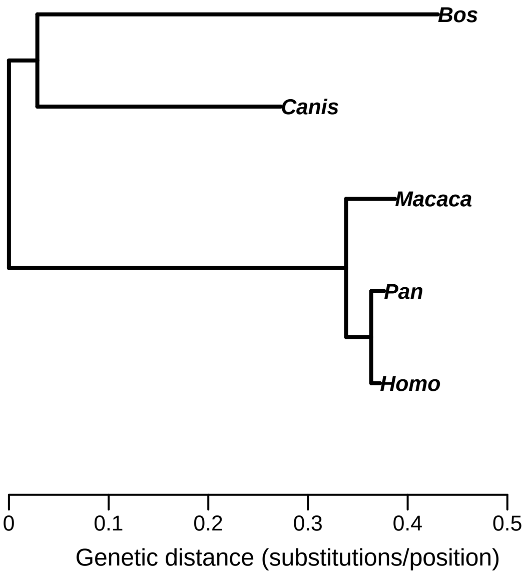

ENSG00000102970 intron 2

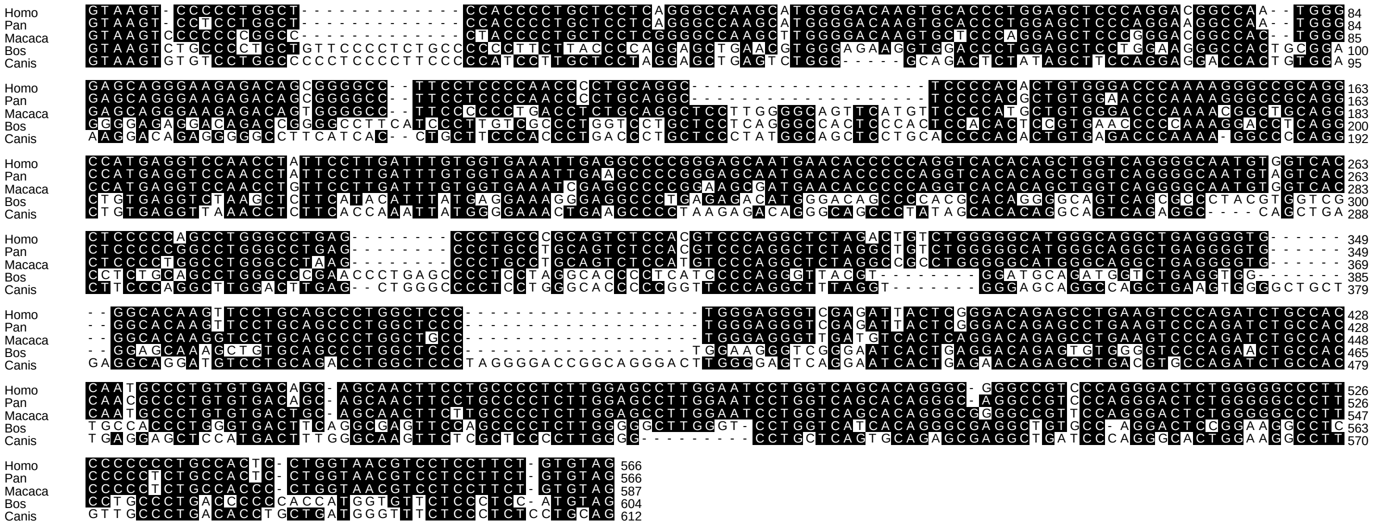

ENSG00000149534 intron 5

Description: High affinity immunoglobulin epsilon receptor subunit beta (MS4A2)
Intron number: 5
Human chromosome: 11
Intron start (bp): 59617608
Human intron length : 405
Intron alignment length: 426
Flanking exons length (upstream/downstream): 159/99
SNP density: 0.000000
K tree score: 0.0534
Scaling factor: 0.7865
Human-chimpanzee distance: 0.015491
Total primate branch length: 0.0944

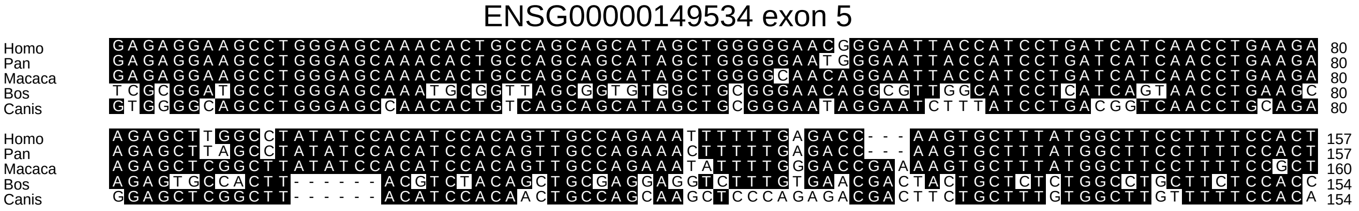

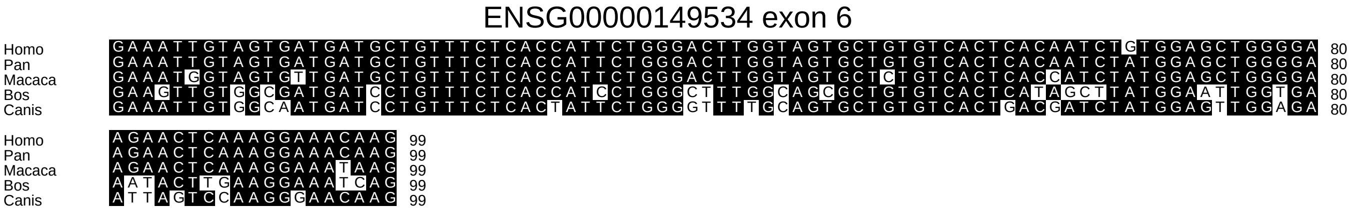

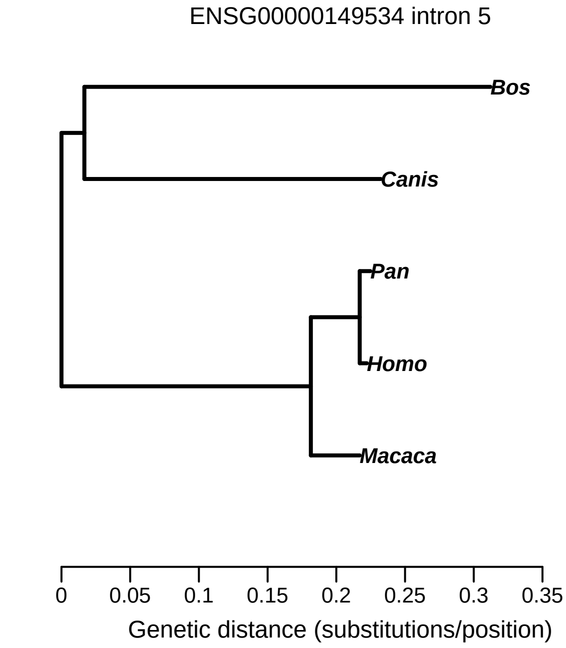

ENSG00000149534 intron 5

```
Homo    GTATGTATTTTTTTTTGTGTGGGAAGACTAAGATTCTGGGTCCTAATGTAAGTAAGAAGCCCTCTTCTCCTGTTCCATGAACACCATCCTTTTCTGTAAC 100
Pan     GTATGTATTTTTTTGTGTGTGGGAAGACTAAGATTCTGGGTCCTAATGTAAGTAAGAAGCCCTCTTCTCCTGTTCCATGAACACCATCCTTTTCTGTAAC 100
Macaca  GTATGTATTATTT--TGTGTGGGAAGACTAAGATTCTGGGTCCTAATGTAAGTAAGAAGCCCTCTTTTCTTGTTCCATAAACACCATCCTTTTCTGTAAC 98
Bos     GTGTGTATTTCTC-----ATGGGAAGGCTGAGA-CCCTGGTCCTGATCTAAGTGGGACGCC-TTCTTTCCCTTTCCATGAACACCACCCTTTCCTGCCAC 93
Canis   GTACGTATTTCTT-----GTGAGGGGGCTAAGG-TCTGGGTCCTACTCTAAGTACAAG----CCCTCTCCTTTCCCACGAACGTCGTCCTTTGCTGTAAC 90

Homo    TTCTATTACACAGTATAGTGGTTCTGTAAGTTCACACAGCCCAGGGAGATGCTGGCTGCCCACTCCCCTCAACCCAGGCAAATTCCTCGGGGTTAAAGTT 200
Pan     TTCTATTACACAGTATAGTGGTTCTGTAAGTTCACACAGCCCAGGGAGATGCTGGCTGCCCACTCCCCTCAACCCAGGCAGATTCCTCGGGGTTAAAGTT 200
Macaca  TTCCATTACATGGTATAGT-GTTCTGTAAGTTCACACAGTCCAGGGAGATGCTGGCTGCCCGCTCCCCTCAACCCAGGCAAATTCCTTGGGGTTAAAGTT 197
Bos     GGC-ATCACGTAATGCAGT-GTTCTGGAGGCTCGTG---CCCAGGGTCGTGCCAGCTCTTTCCCCCACC--TTGACCTGAGTCCTGCAAGGGTGGTGCCC 186
Canis   CTCTATCACATAGTGTAGT-GTTCGGGGGGTTCATG-----GACAAAGATGCTGGCTGCCGCTCCCTC----CCCAGCTGGGTTCTGTGAGGCTGGAGTC 180

Homo    ATCTACTGCAAGTGACGATCTCTGGGTTTTTCTGTG------CCTGTGTTTGTGTGTGTGTGTGTGTGTGTGTGTGTGTGTGTATGTGTCACTTTAAA 294
Pan     ATCTCCTGCAAGTGACGATCTCTGGGTTTTTCTGTG------CCTGTGTT------TGTGTGTGTGTGTGTGTGTGTGTGTGTGTATGTGTCACCTTAAA 288
Macaca  ACCTACTACAAGTAACCATCTCTGGGTTTTCCTGTGTCTCTCTCTCTCTCTCTGTGTGTGTGTGTGTGTGTGTGCGTGTGTGTGTGTGTGTGTCACTTTAGA 297
Bos     GGGGGCTTTGAGTAACCACCTCCTGGTTTTCGCACG----------------------------------------------------TGTCATTCTATA 234
Canis   ACCCGCCTTGACCTCCCTTTTTGTGGCTTTCCCACG------------------------------------------------TATGTGTCACTTCATT 232

Homo    AGGACTGGTCAGATGGTA---------GGGAGATGAAAACAGGAGATGCTATAAGAAAATAAACTTTTGGGGCGAATACCATGTGACTCTT------TTT 379
Pan     AGGACTGGTCAGATGGTA---------GGGAGATGAAAACAGGAGATGCTATAAGAAAATAAACTTTTGGGGCGCATACCATGTGACTCTT------TTT 373
Macaca  AGGACTGGTCAGATGATG---------GGGAGATGAAAGCAGGAGATGCTATAAGAAAATAAACTTTTGGGGCACCTACCATGTGACTC--------TTT 380
Bos     GGAACTGGCCATGTGCTGAGTGGGCATGGGTGATGAAAACAGGGGCTGCTGTAAGGGAATGCGCTTT-------CCCACCCCTTGACTCCATTGTCCTTT 327
Canis   GGGAGTGATCACAT--CG---------GGGTGATGAAATCAGGGGCTTCTGTTAG-GAACACACCTTTGGGGCACCTGCCTTGTGACTCTA------CTT 314

Homo    GTTTGTCATTTGTTGCTGTTCAATAG 405
Pan     GTTTGTCGTTTGTTGCTGTTCAATAG 399
Macaca  GTTTATCGTTTGTTGCTGTTCAATAG 406
Bos     GGTTGTTGTTGGCTGTTGCTCAACAG 353
Canis   GTC--TTGTTGGTTACTGTTCAACAG 338
```

ENSG00000186815 intron 4

Description: two pore segment channel 1 (TPCN1)
Intron number: 4
Human chromosome: 12
Intron start (bp): 112190145
Human intron length : 785
Intron alignment length: 878
Flanking exons length (upstream/downstream): 114/131
SNP density: 0.003822
K tree score: 0.0594
Scaling factor: 0.7457
Human-chimpanzee distance: 0.020238
Total primate branch length: 0.0941

ENSG00000186815 exon 4

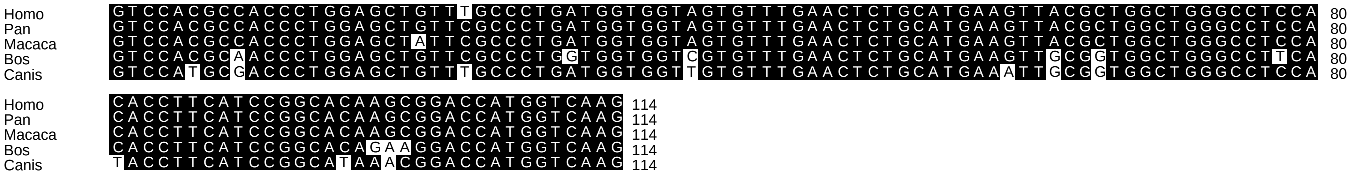

ENSG00000186815 exon 5

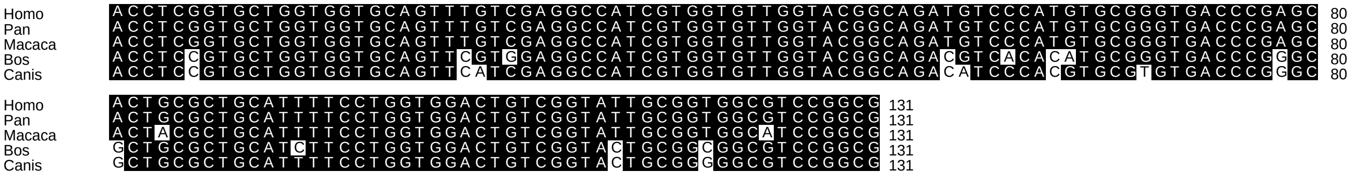

ENSG00000186815 intron 4

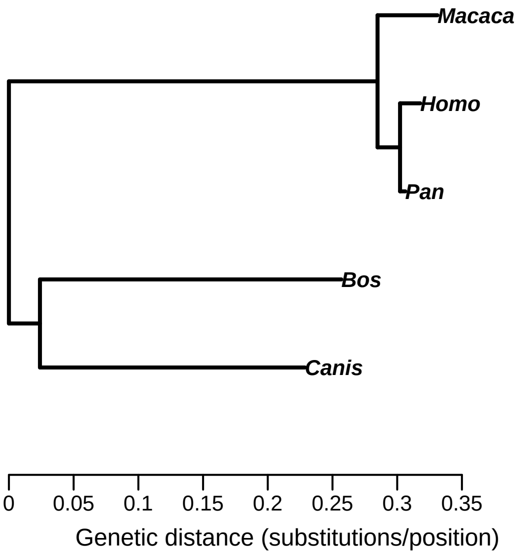

ENSG00000186815 intron 4

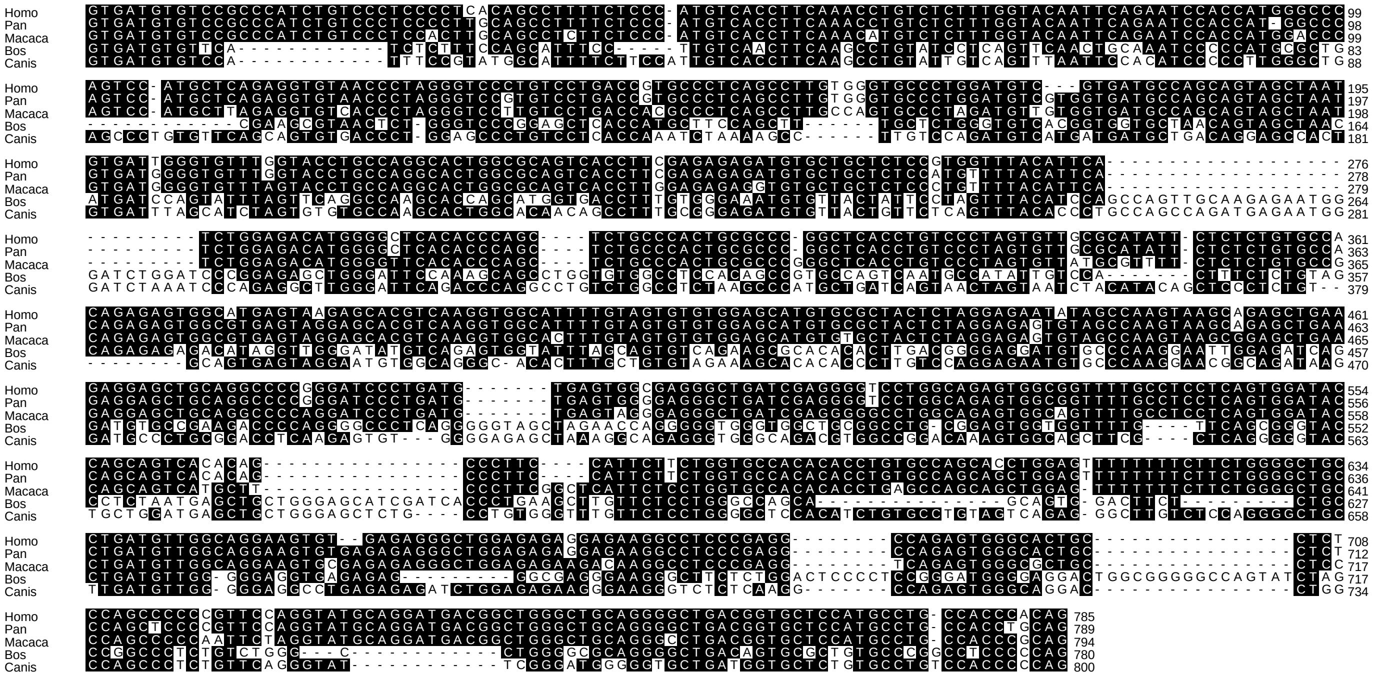

ENSG00000143921 intron 10

Description: ATP-binding cassette sub-family G member 8 (ABCG8)
Intron number: 10
Human chromosome: 02
Intron start (bp): 43955127
Human intron length : 662
Intron alignment length: 813
Flanking exons length (upstream/downstream): 77/268
SNP density: 0.013595
K tree score: 0.0536
Scaling factor: 0.7625
Human-chimpanzee distance: 0.015298
Total primate branch length: 0.0941

ENSG00000143921 exon 10

```
Homo    GTTACTCAGAGAGGGCAATGCTTTACTATGAACTGGAAGACGGGCTGTACACCACTGGTCCATATTTCTTTGCCAAG 77
Pan     GTTACTCAGAGAGGGCAATGCTTTACTGTGAACTGGAAGACGGGCTGTACACCACTGGTCCATATTTCTTTGCCAAG 77
Macaca  GTTACTCGGAGAGGGCAATGCTTTACTATGAACTGGAAGACGGGCTGTACACCACTAGTCCATATTTCTTTGCCAAG 77
Bos     GTCACTCAGAGAGGGCACTGCTTTACTATGAACTAGAAGATGGGCTGTACACGGCGGGGCCATATTTCTTTGCCAAG 77
Canis   GTCACTCAGAGAGGGCAATGCTTTACTATGAACTAGAAGATGGACTGTACACTGCTGGTCCATATTTCTTTACCAAG 77
```

ENSG00000143921 exon 11

```
Homo    ATCCTCGGGGAGCTTCCGGAGCACTGTGCCTACATCATCATCTACGGGATGCCCACCTACTGGCTGGCCAACCTGAGGCC 80
Pan     ATCCTCGGGGAGCTTCCGGAGCACTGTGCCTACATCATCATCTACGGGATGCCCACCTACTGGCTGGCCAACCTGAGGCC 80
Macaca  ATCCTCGGCGAGCTTCCGGAGCACTGTGCCTACATCATCATCTATGGGATGCCCACCTACTGGCTGGCCAACCTGAGGCC 80
Bos     ATCCTGGGGGAGTTTCCTGAGCACTGTGTCTACATCATCATCTATGGAATGCCCATCTACTGGCTGGCCAACCTGCGCCC 80
Canis   ATCCTAGGGGAGCTTCCAGAGCACTGTGTCTACATCATGATCTGTGGGATGCCTACCTACTGGCTGGCCAACCTGCGCCC 80

Homo    AGGCCTCCAGCCCTTCCTGCTGCACTTCCTGCTGGTGTGGCTGGTGGTCTTCTGTTGCAGGATTATGGCCCTGGCCGCCG 160
Pan     AGGCCTCCAGCCCTTCCTGCTGCACTTCCTGCTGGTGTGGCTGGTGGTCTTCTGTTGCAGGATTATGGCCCTGGCCGCCG 160
Macaca  GGGCCTCCAGCCCTTCCTGCTGCACTTCCTGCTGGTGTGGCTGGTGGTCTTCTGTTGCAGGATTATGGCCCTGGCCACCG 160
Bos     AGGCCTCGAGCCCTTCCTGCTGCACTTCCTGCTGGTGTGGCTGGTGGTCTTCTGCTGCCGGGTCATGGCCCTGGCCGCCG 160
Canis   AGGCCTTGAGCCCTTTCTGCTGCACCTTCTGCTGGTGTGGCTAGTGGTCTTCTACTGTAGGATCATGGCCCTGGGTGTTG 160
```

ENSG00000143921 intron 10

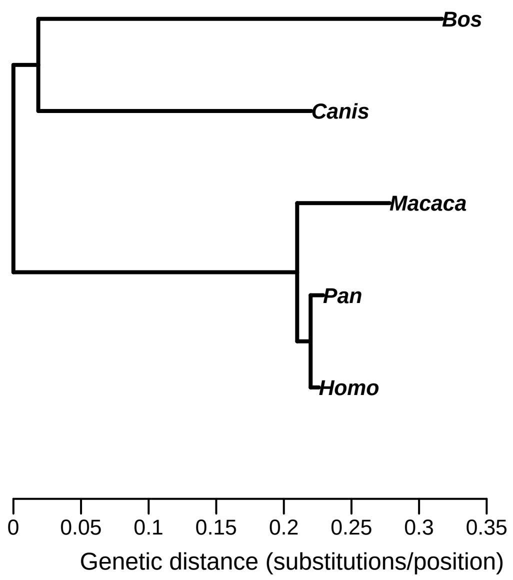

ENSG00000143921 intron 10

```
Homo    GTGACTGGGCAGGGTTGAGAGCAAGTG-CCCCCCACCCACCAGGGTGGGGTAAGTGTGGAGAAAACGTTGCTACA------------------AGGAAGG 82
Pan     GTGACTGGGCAGGGTTGAGAGCAAGTGCCCCCCCACCCACCAGGGTGGGGTAAGTGTGGAGAAAACATTGCTACA------------------AGGAAGG 83
Macaca  GTGACTGGGCAGGGTTGAGAGCAAGTG-CCCCCCGCCCACCAGGGTGGGCGTAAGTGTGGAGAAAACACTGCCACC------------------AGGAAGC 82
Bos     GTGACTGGTCAGGCTTCAGAGTGAGTG-------------CCACATGGAACAGTGGGCAGAAGAATCTTCTCCACATTATACAGATGTTACAGGGAAGCA 87
Canis   GTGACTGGTCAGGGCTGAGAGCAAGTG------------ACAAGGTGGGGG-ATGGGTAGACAAAGTCACACTGCATTG-ACAGAAGCTCCTGAGAAGTA 86

Homo    CTTTTCTGAACCATGGGGCCGTGGGTCATGTGAAGTCACCGATGCCACCAGATGCCACATTTTCTAGGGGAGTTTCAGAGACTTGAACTTT---------- 173
Pan     CTTTTCTGAACCATGGGGCCGTGGGTCATGTGAAGTCACAGATGCCACCAGATGCCACATTTTCTAGGGGAGTTTCAGAGACTTGAACTTT---------- 174
Macaca  CTTTTCTGAACCATGGGGCTATGGGTCATGTGAAGTCGCAGATGCCACCAGATGTCACATTTTCTAGGGGAGTTTCGGAGACTTGAAC------------ 170
Bos     TTTTGCTCAGCGGTGGGGCTGAGCATCATCTGTGGTTG----------CAGATGCCAGAATCCTTAGGAGAGTTTCAGAGACTTGATGAGTCTCTGATAA 177
Canis   TTTTGCTGAACCAGGGGACCGTGCATCATCTGTGGTTA----------TAGATATCATATTTCCTAGGGGAGTTTCAGAGACTTGATGT----------- 165

Homo    -TATTTTAAACATTTACCAAAACACCAGGACTGTGGACCAGGAATGATAGTTTTCTGGCCAGAAGGAATCTAAAAAAAAAGA------------------- 253
Pan     -TATTTTAAACATTTGCCAAAACACCAGGACTGTGGACCAGGAATGATAGTTTTCTGGCCAGAAGGAATCTAAAAAAAAAGA------------------- 254
Macaca  ---TTTTAAACATTTACCAAAACACCAGGATTGTGGACCAGGAATGATAGTTTTCTGGTCAGAAGGAGTCTAAAAAAAAAGA------------------- 248
Bos     ATTTTTCAAACATTTATCAGAAGGCTAGGACCATGGACCAGGAATGACAATTTTCTGCCCACCAGAAACCTAACAGAGAAG-------------------- 258
Canis   ---TTTAAAATATTTACCTAAAGACCAGGACTGTGGATGAGGAATGACACTTTCCTGGTCAGCAGAAACCAAAAAAAGAGAGAAAGACAGACAGACAGGA 262

Homo    --------------------------------------------------AGTCCTCATTGTATTTTACCTTTTCAGAGCCTCACTGTGGCCCCCCAGCAG 304
Pan     --------------------------------------------------AGTCCTCATTGTATTTTACCTTTTCAGAGCCTCACTGTGGCCCCCTAGCAG 305
Macaca  --------------------------------------------------AATCCTCTTTGTATTTTACCTCTTCGGAGCCTCACTGTGGCCCC--GAGCG 297
Bos     ------------------------------------------AGGAGGGAAACACAACTAGCATACTTCACCTCTCCAGTCTTTCCACGTCCCT---GGAA 314
Canis   AGGAAGGAAAGAAAGAGGGAGGGAAGAAGGAAGGAAAGAAAGAAAGTGGGAAACATCCAGCATACTCCCCTTTCAGA----TATCATGACATCTCCAGGA 358

Homo    GCCAGGCAGG----------------TGTTTCCAGGAAGCAGAGGTTCAGAGAGGCTACGTGGCTCTC-CAAGGCCACACAGCTACTTGATCTGGTCAGG 387
Pan     GCCAGGCAGG----------------TGTTTCCAGGAAGCAGAGGTTCAGAGAGGCTACGTGGCTCTC-CAAGGCCACACAGCTACTTGATCTGGTCAGG 388
Macaca  GCCAGGCAGG----------------CGTTTCCAGGAAGCAGAGGTTCGGAGAGGCTGCTCGGCTCTC-CAAGGCCACACAGCTACTTG-TCTGGTCAGG 379
Bos     AGCAGATTGGAAGGTTTGACGGTCCTCATTTCAAAGAAGTGGAGGCTCAGAGAAGTTAAGTGGCTGCCTCCAGGCCACACAGCTACCATGTCTGGGTAGG 414
Canis   AGCAGGTCAGCAACTATTACTATACTTATTTCCAGGATGCAAAGGCTCAGAGAGGTTATGCAGCTGTCTCAAAGCCCCACACCTATTAGGTCTAGGCAGG 458

Homo    GGGAGTAGGGACTTGAAACCAG---------GTCTTAGGACAGCAGGTGCCCAT---------TGCTGCCCTGGGGGAAACAGGCTGAACTGCCCTAGAG 469
Pan     GGGAGTAGGGACTTGAAAGCAG---------TTCTTAGGACAGCAGGTGCCCAT---------TGCTGCCCTGGGGGAAACAGGCTGAACTGCCCTAGAG 470
Macaca  GGGAGTAGGGACTTGAAACCAG---------GTCTTAGGACAGCAGGTGCCCAG---------TGCTGCCCCGGGAGAAACAGGCTGAACTGCCCTAGAG 461
Bos     CAGAGTAGGGATTTGAAACCAG---------GTCTCCAGA------------------------------------------GCTCTGAACTGCCCCAGAG 464
Canis   CAGGCGGGAACTAGAAACAAGAAAACAGATACCTTACTGCAGTTTTCACCCGGCTACACTGCCCCCACCCCGGGGGAAATGACTCTAACTGTCCCAGAG 558

Homo    GGAGCATTTGGTATCTGCAAGGGGGAGGCCAGCA--CTGAGCTCATGCTCCTGGGTCCCA-------------GCACACCCTCCTGCCACAGCCTCA-TC 553
Pan     GGAGCACTTGGTATCTGCAAGGAGGAGGCCAGCA--CTGAGCTCATGCTCCTGGGTCCCA-------------GCACACCCTCCTGCCACAGCCTCC-TC 554
Macaca  GGAACGCTTGGCATCTGCAAGGGGGAAGCCAGCA--CTGAGCGTG-----CCGGGTCCCA-------------GCACACCCTCCTGCCACAGCCTCA-TC 540
Bos     GGAGCATACAGCATCTGCAGGGGGAAG-TCAGCA--GTGAGTTCATGCT-GTGGGTCCCACACACCTGTGCCCACTAACCCTCCAGTGGCAACCCAGTC 560
Canis   GGAGCACATGGCATCTGCAGGGAGCAA-CCAGCAAGGAGAGCTCATGTTCCAGGGTCCCACA---------CCTACTGCCCTTCCTGCTGCAGGCTGA-TA 648

Homo    ATCACCAGGAGGGAAGGTTGCTATCTGGGGGCACTGC-TACTTTTAAACGTTT--ATAATAATGGC-AGTGAAGGTGCTGGCTTCATATCCTTGCAAGGG 649
Pan     ATCACCAGGAGGGAAGGTTGCCATCTGGGGGCACTGC-TACTTTTAAACGTTT--ATAATAATGGC-AGTGAAGGTGCTGGCTTCATATCCTTGCAAGGG 650
Macaca  ATCACCAGGAGGGAAGGTTGCCACTGGAGGGCACAGA-TACTTTTAAACATTT--ATTATAATGGC-AGTGAAGGTGCTGGCTTCACATCCTTGCAAGGG 636
Bos     ACCATCAGGAGGGAAGGCTGGGACACTGGGGGCTGC-TGCCTTTGAACACTGAAAGATCAGCAGCAAATAAAGGCGCTGGCTC---------------- 643
Canis   ATCAGTGGGAGGGAAGATTGGCATCACAGAGGATTACTTATCTTTAAACACTTAAAGAACAATGAT-AGTGAAGGCATTAGTTCCACATCCTTGCAAGGG 747

Homo    CTGTTCTTTGCAG 662
Pan     CTGTTCTTTGCAG 663
Macaca  CTGTTCTTTGCAG 649
Bos     ACATCCTTTGCAG 656
Canis   CTATTCTTTGCAG 760
```

ENSG00000113522 intron 15

Description: DNA repair protein RAD50 (RAD50)
Intron number: 15
Human chromosome: 05
Intron start (bp): 131967638
Human intron length : 759
Intron alignment length: 803
Flanking exons length (upstream/downstream): 127/194
SNP density: 0.003953
K tree score: 0.0987
Scaling factor: 0.53
Human-chimpanzee distance: 0.014786
Total primate branch length: 0.094

ENSG00000113522 exon 15

```
Homo    ATGGAACTTAAAGATGTTGAAAGAAAAATTGCACAACAAGCAGCTAAGCTACAAGGAATAGACTTAGATCGAACTGTCCA 80
Pan     ATGGAACTTAAAGATGTTGAAAGAAAAATTGCACAACAAGCAGCTAAGCTACAAGGAATAGACTTAGATCGAACTGTCCA 80
Macaca  ATGGAACTTAAAGATGTTGAAAGAAAAATTGCACAACAAGCAGCTAAGCTACAAGGAATAGACTTAGATCGAACTGTCCA 80
Bos     ATGGAACTAAAAGATGTTGAAAGAAAAATTGCACAGCAAGCAGCTAAACTCCAAGGACTAGATTTGGATCGAAGTGTTCA 80
Canis   ATGGAACTTAAAGATGTTGAAAGAAAAATTGCACAACAAGCAGCTAAGCTGCAAGGACTAGACTTGGATAGAACTGTTCA 80

Homo    ACAAGTCAACCAGGAGAAACAAGAGAAACAGCACAAGTTAGACACAG 127
Pan     ACAAGTCAACCAGGAGAAACAAGAGAAACAGCACAAGTTAGACACAG 127
Macaca  ACAAGTCAACCAGGAGAAACAAGAGAAACAGCACAAGTTAGACACAG 127
Bos     GCAAGTAAACCAGGAAAAACAAGAAAAACAACACAAATTAGATACAG 127
Canis   ACAAGTAAACCAGGAAAAACAAGAAAAACAACACAAATTAGATACAG 127
```

ENSG00000113522 exon 16

```
Homo    TTTCTAGTAAGATTGAATTGAATCGTAAGCTTATACAGGACCAGCAGGAACAGATTCAACATCTAAAAAGTACAACAAAT 80
Pan     TTTCTAGTAAGATTGAATTGAATCGTAAGCTTATACAGGACCAGCAGGAACAGATTCAACATCTAAAAAGTACAACAAAT 80
Macaca  TTTCTAGTAAGATTGAATTGAATCGTAAGCTTATACAGGACCAGCAGGAACAGATTCAACATCTAAAAAGTATAACAAAT 80
Bos     TTTCTAGTAAGATAGAATTGAACCGTAAGCTTATACAGGACCAGCAGGAACAGATTCAACACCTAAAAAGCACAACAAAT 80
Canis   TTTCCAGTAAGATGGAATTGAATCGTAAGCTTATACAGGACCAGCAGGAACAGATTCAATACCTAAAAAGTACAACAAAT 80

Homo    GAGCTAAAATCTGAGAAACTTCAGATATCCACTAATTTGCAACGTCGTCAGCAACTGGAGGAGCAGACTGTGGAATTATC 160
Pan     GAGCTAAAATCTGAGAAACTTCAGATATCCACTAATTTGCAACGTCGTCAGCAACTGGAGGAGCAGACTGTGGAATTATC 160
Macaca  GAGCTAAAATCTGAGAAACTTCAGATATCCACTAATTTGCAACGTCGTCAGCAACTGGAGGAGCAGACTGTGGAATTATC 160
Bos     GAACTTAAATCAGAGAAACTTCAGATATCCACTAATTTACAACGTCGTCAGCAGCTAGAGGAACAGACTGTGGAATTATC 160
Canis   GAACTTAAATCAGAGAAACTTCAGATATCCACTAATTTACAACGTCGACAGCAAATGGAGGAACAGACTGTGGAATTATC 160
```

ENSG00000113522 intron 15

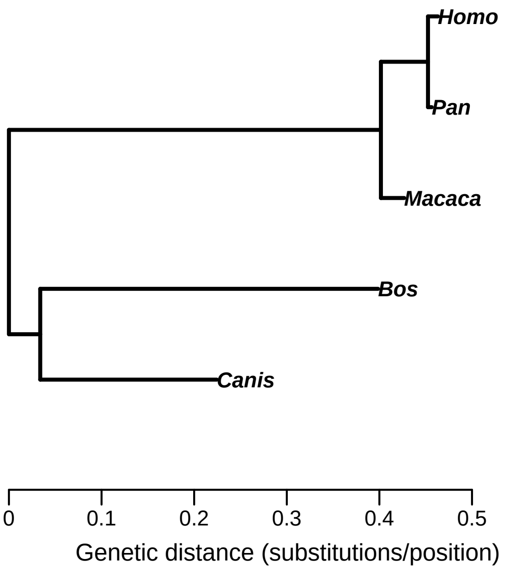

ENSG00000113522 intron 15

```
Homo    GTAATACAGTCTGTGTCCT----------------------------------TCTGTACTCATAGAGACTTTGACATTGCGAGCACATGCCTTTTACAG 66
Pan     GTAATACAGTCTGTGTCCT----------------------------------TCTGTACTCATAGAGACTTTGACATTGCGAGCACATGCCTTTTACAG 66
Macaca  GTAATACAGTCTGTGTCCT----------------------------------TCTGTACCCATAGAGACTTCAACATTGCCAGCACATGCCTTTTATAC 66
Bos     GTAATAGAGTGTGTTTCTT------------------------------------------TATAAAGACTTCAACATTCCAAGCATATGGTTTTTACCA 58
Canis   GTAATATAGTTAGTTTTTTCTTTCTCTTTTTTTTTATATATAGTTAGTTTTTTCATGTACCCATAAAGACTTCAACATCCT-AGCATATGCTTTTTATCA 99

Homo    TCAATTTTATATCTGTTACATGGACAACGAAGTTCCTTCCTGTCCACA-TATATTTGCTCTTTTTTTCTGAAAAGCATCACTTCTCTTTGTTCTCTTACT 165
Pan     TCAATTTTATATCTGTTACATGGACAACGAAGTTCCTTCCAGTCCACAGTATATTCGCTCTTTTTTTCTGAAAAGCATCACTTCTCTTTTTTCTCTTACT 166
Macaca  TCAATTTTATACCTGTTACATAGACAAGGAAGTTCCTTCCAGTCCACAGTATATTCGCTCTTTTTTTCTGAAAAGCATCACTTCTCTTTGTTCTCTTACT 166
Bos     CTAATTTTATAACTATTATGTGGACAAGGAACAGTCTTCCAGTCAATGGTATAGTCACTTTCTTTTCTC-AAAAGCATCACT--TCTTTGTTTCCTTTCT 155
Canis   TACATTTTATATCTATCATGTGGACAGGGAACTGTCTTTCTTCCAGTTCGCAATATTCATCCTCTTTCT-AAAAGC----CT--TACTTGTTTCCTTGCT 192

Homo    TTGCACAACAGATTTTGAACAGTAGGAGAGTATGAAATAAGCTGTATTCTCTTTTCCTTTTTTTTTTTTTTTAAGATAGAGTTTCACTTTTGTCGCCCAGG 265
Pan     TTGCACAACAGATTTTGAACAGTAGGAGAGTATGAAATAAGCTGTATTCTCTTTTC----TTTTTTTTTTTTAAGATAGAGTTTCACTCTTGTCGCCCAGG 262
Macaca  TTGCACAACAGATTTTGAATAGTAGGAGACTGTCAAATAAGCTGTATTCTTTTTCT----TTTTTTTTTTTTAAGATGGAATTTTGCTCTTGTTGCCTAGG 262
Bos     GTCCAGAACAGACTTTGAATAATAGGAACTTTAA-------------------------ATAATTAGTAATAGATGGAAAGTAAAGTTGCTCAGTCAT 230
Canis   TTGCATAACAGATTTTGAATGATGG--------------------------------------------------------------------------- 217

Homo    CTGGAGTGCAATGGTGCAATCTTGGCTCACTGCAACCTCCGCCTCCCGGGTTCAAGTGATTCTCCTGCCTCACCCTCCTGAGTAGCTGGGGTTACAGGCA 365
Pan     CTGGAGTGCAGTGGTGCAATCTCGGCTCACTGCAACCTCCGCCTCCCGGGTTCAAGTGATTCTCCTGCCTCACCCTCCTGAGTAGCTGGGGTTACAGGCA 362
Macaca  CTGGAGTGCAGTGGTGCAATCTCAGCTCACTGCAACCTCCGCCTCCCAGGTTCAAGTGATTCTCCTGCCTCACCCTCCTGAGTAGCTGGGATTACAGGCA 362
Bos     GTCCAGCTCTTGTGACCACATGGACTATAGAGTCCATGGATTTCTCCAGGCCAGAATACTGGAGTGGGTAGCCTTTCCTTTCTCCAGGGGAT------- 323
Canis   ---------------------------------------------------------------------------------------------------- 217

Homo    CACATCACTATGCCCAGCTAATTTTTGTATTTTTTCGTAGAGATGGGGTTTCACTGTGTTTGTCAGGCTGATCTCGAACTCCTGACCTCAGGTGATCCAC 465
Pan     CACATCACTACGCCCAGCTAATTTTTGTATTTTTTCGTAGAGATGGGGTTTCACTGTGTTTGTCAGGCTGATCTCGAACTCCTGACCTCAGGTGATCCAC 462
Macaca  CTCGTCACCACGCCCAGCTAATTTTTGTATTTTTTAGTAGAGGCGGGGTTTCACTGTGTTTGTCAGGCTGGTCTCAAACTCCTGACCTCGG--GATTCAC 460
Bos     ----CTTCCCAACCCAGCGATTGAACCCAGGTCTCCTGCATTGCAGGCGGATTCTTTACCAGCTGAGCCACAAGGAAACCCCAAATAATAG--------- 410
Canis   ------------------------------------------------------------------------------------ATATCAG--------- 224

Homo    CTGCCTCAGTCTCCCAAAGTGCTGGGATTACAAGTGTGAGCCAGTGCGCCTGGCCATGAGCTGTATTCTTTCTCATTCATACCTGCCCTGTAAGCT---- 561
Pan     CTGCCTCAGTCTCCCAAAGTGCTGGGATTACAAGTGTGAGCCAGTGCGCCTGGCCATGAGCTGTATTCTTTCTCATTCATACCTGCCCTGTAAGCT---- 558
Macaca  CCGCCTCGGCCTCCCAAAGTGCTGGGATTACAAGCATGAGCCAGTGCGCCTGGCCATGAGCTATATTCTTTCTCAGTCATACCTGCCCTGTGAGCT---- 556
Bos     -------------------------------------------------------ATAAGCTGTTCTCTTTCTCAGTCATACCTGTCCTGTGAGCTCACG 455
Canis   -------------------------------------------------------ATGAGCTCTACTCTTTCTTAGTCCTGCCTGCCCTGTGATTT---- 265

Homo    --TTCCCTGTGAGTGGCCAGCAGGGAACATCAAGCTGATTTGAGAACTAGCAGGGTTCTCATTGTATTTTTGTTGCAGTGGGTGGGGCCCACAGAAA--T 657
Pan     --TTCCCTGTGAGTGGCCAGCAGGGGACATAAAGCTGATTTGAGAACTAGCAGGGTTCTCATTATATTTTTGTTGCAGTGGGTGGGGCCCACAGAAA--T 654
Macaca  --TTCCCTGTGAGTGGTCAGCAGGGGACATCAAGCTGATTTGGGAGCTAGCAGGGTTCTCGTTATATGTTTGTTGCAGTGGGTGGGGCCCACAGAAACTC 654
Bos     CATGGCTGTTGGGGCAGCAGGCGGG-------------TTTGGAGCTGGATAAATTGTTCTGGCATTTTTCTTGCAGTGGGTGGAACCCACAGAAACTC 542
Canis   --TAACCCATGGGTGACCAGGAGGG-------------ATT-----------------CTGTATCACTTTTCTTGCAGTGGGTGGGATCCACAGAAGCTG 333

Homo    AGCATTTGTGGATTCCATAGACCGATAAAAAATGGGAAGAATGATACAAATAGTA-TTTTCTATGCCCTTACATTAATTACTGTGATAATATGTTTTTGTG 756
Pan     AGCATTTGTGGATTCCATAGACCGATAAAAAATGGGAAGAATGATACAAATAGTA-TTTTCTATGCCCTTACATTAATTATTGTGATAATATGTTTTTGTG 753
Macaca  AGCATTTGTGGATTCCATAGACTAATAAAAAATGGGAAGAATTATACAAATAGTA-TTTTCTATACCCTTACATTAATTACTGTGTTAATATGTTCTTGTG 753
Bos     AGCCTTTGTGAATGCCACAGACCTATAAACG--GGAAAGATGTTACTTGTAGTA-TTTTCTGTGCCCTTCCACTAATCACTGTATGGATGTGTTCTCATG 639
Canis   AGCCTTTGTGAATTCCATAGAGCTATAAAAAT-GGAAGCTTGCTGATCATAGTATTTTTCTGTATCCTTAAACTAATCGCTGTGTGACTATGTTTTCATG 432

Homo    TAG 759
Pan     TAG 756
Macaca  TAG 756
Bos     CAG 642
Canis   TAG 435
```

ENSG00000039650 intron 6

Description: Bifunctional polynucleotide phosphatase/kinase (PNKP)
Intron number: 6
Human chromosome: 19
Intron start (bp): 55058830
Human intron length : 203
Intron alignment length: 332
Flanking exons length (upstream/downstream): 108/72
SNP density: 0.004926
K tree score: 0.0738
Scaling factor: 0.6898
Human-chimpanzee distance: 0.020117
Total primate branch length: 0.0939

ENSG00000039650 exon 6

```
Homo    CTGGTGATCTTCACCAACCAGATGAGCATCGGGCGCGGGAAGCTGCCAGCCGAGGAGTTCAAGGCCAAGGTGGAGGCTGT 80
Pan     CTGGTGATCTTCACCAACCATATGAGCATCGGGCGCGGGAAGCTGCCAGCCGAGGAGTTGAAGGCCAAGGTGGAGGCTGT 80
Macaca  CTGGTGATCTTCACCAACCAGATGAGCATCGGGCGCGGGAAGCTGCCAGCCGAAGAGTTCAAGGCCAAGGTGGAGGCTGT 80
Bos     CTGGTGATCTTCACCAACCAGATGGGCATCGGGCGCGGAAAGCTGCGAGCGGAGGAGTTCAAAGCTAAGGTGGAGGCTGT 80
Canis   CTGGTGATCTTCACCAACCAGATGGCCATTGGGCGCGGGAAGCTGCCGGCAGAGGAGTTCAAGGCTAAAGTGGAGGCTGT 80

Homo    GGTGGAGAAGCTGGGGGTCCCCTTCCAG 108
Pan     GGTGGAGAAGCTGGGGGTCCCCTTCCAG 108
Macaca  GGTGGAGAAGCTGGGGGTCCCCTTCCAG 108
Bos     GGTGGAGAAGCTAGGGGTCCCCTTTCAG 108
Canis   GGTGGAGAAACTGGGCCTCCCCTTTCAG 108
```

ENSG00000039650 exon 7

```
Homo    GTGCTGGTGGCCACGCACGCAGGCTTGTACCGGAAGCCGGTGACGGGCATGTGGGACCATCTGCAGGAGCAG 72
Pan     GTGCTGGTGGCCACGCACGCAGGCTTGTACCGGAAGCCGGTGACGGGCATGTGGGACCATCTGCAGGAGCAG 72
Macaca  GTGCTGGTGGCCACGCACGCAGGCTTGTACCGGAAGCCAGTGACAGGCATGTGGGACCATCTGCAGGAGCAG 72
Bos     GTGCTGGTGGCCACGCACGCAGGCTTGTACCGGAAGCCAGTGAGCGGCATGTGGGACCACCTGCAGGAGCAG 72
Canis   GTGCTGGTGGCCACACACGCCGGCTTGTACCGGAAGCCAGTGATCGGCATGTGGGACCATCTGCGGGAACAG 72
```

ENSG00000039650 intron 6

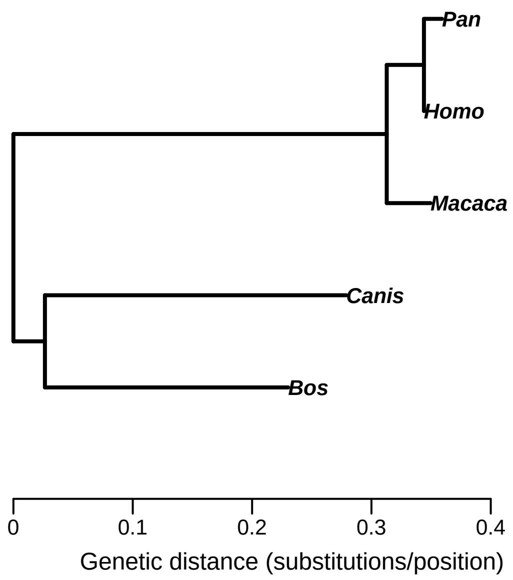

ENSG00000039650 intron 6

```
Homo    GTATGGCTGGAAGGGAGGCTGGGAGCT--ATGTGGGGGCACAGAGATCCAGAGCGAG------------------------------------------- 55
Pan     GTATGGCTGGAAGGGAGGCTGGGAGCT--ATGTGGGGGCACAGAGATCCAGAGCAAG------------------------------------------- 55
Macaca  GTATGGCTGGAAGGGAGGCTGGGAGCT--ATGTGGGGGCACACAGATCTGGAGAGAG------------------------------------------- 55
Bos     GTATGGCTGCACGGGAGGCTGAAGGAC-TATGTAGAGGGACAGAGAGGCTGAGACAG---GGGCCAGAGATAGCGCGGTGG-----------GCCGGAAA 85
Canis   GTATGGCTGCAAGGGAGCTTGAGTCATAGAGGTTGGGGGACAGGGACCCAGACAGAGGCAGGGACAGAGTTAGTGTCACGGTGTCAGACCGTGACAGAGA 100

Homo    -----GGCTTTAGACTCCAGGGTAAG--------------------------CGGGGCGGAGGCCTCAGGCAGTGTGATGCTGATAGGAACCCATCATCA 124
Pan     -----GGTTTTAGACTCCAGGGTAAG--------------------------CAGGGCGGAGGCCTCAGGCAGTGTGATGCTGATAGGAACCCATCATCA 124
Macaca  -----GGCTTCAGACTCCAGGATAAG--------------------------TGGGGCAGAGGCCTCAGGCGGTGTGATGCTGATAAGAACCCATCATCA 124
Bos     GGAGGAAACAAAGACCCGGGGAGAAGGTGTGACATGGTGAGCCAGTTGGGGAGTGGGTGGGAGCCCCAGGGGTGGTTATGCTGACAGATGCC--TTGTAA 183
Canis   GGAGGGAACAGAGACCCAGGGAGAAGGCAT----------ACGTACCAGAAGGCAGTGGAGGCCCCCCGGCAGGGTTATGCTGGTAGAAACCCATCATAA 190

Homo    GCTACAAATTCT----------------------GGGCTGGGGTGGCATC--------------------------CAGTAGGTGCTTGAGGGGGAGCTA 176
Pan     GCTACAAATTCT----------------------GGGCTGGGGTGGCATC--------------------------CAGTAGGTGCTTGACGGGGAGCTA 176
Macaca  GCTACAAATTCTGTAGCTCTGCTTGGCTGGGTGAGGGCTGGGGTAGCATC--------------------------CAGTAGGTGCTTGAGGAGGAGCTG 198
Bos     GTG---------------GTGCTCACCTTGGTGAGGGTTGGAGTGGTGTCTTTGGGATTCAGGTGCCCTTGAAGGACTCTGGGTGTCGGAGCAAGAGTCA 268
Canis   ATGGGTTCTTGGGCATCTGGAGTCCTCTGGGTTGGTGAAGGGGTAGCATCCCTGGCACCCATGCACCCTCGAAGGAT--------TTGGGGAAGGAGCCC 282

Homo    AAGCTGGTGGCCCCC-----TCCCATCCACAG 203
Pan     AAGCTGGTGGCCCCC-----TCCCATCCACAG 203
Macaca  AGGCTGGTGGCCCCC-----TCCCATCCACAG 225
Bos     AGGATGGTGTCTGACCCCCACCCCACCCACAG 300
Canis   AGGAGGGTGTCTGAC-----CTTCACCCACAG 309
```

ENSG00000121931 intron 3

Description: Uncharacterized protein C1orf103 (C1orf103)
Intron number: 3
Human chromosome: 01
Intron start (bp): 111292545
Human intron length : 1451
Intron alignment length: 1752
Flanking exons length (upstream/downstream): 273/441
SNP density: 0.004824
K tree score: 0.0812
Scaling factor: 0.8301
Human-chimpanzee distance: 0.016015
Total primate branch length: 0.0935

ENSG00000121931 exon 3

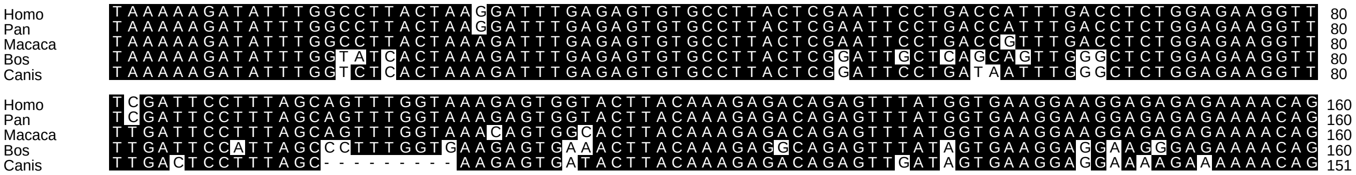

ENSG00000121931 exon 4

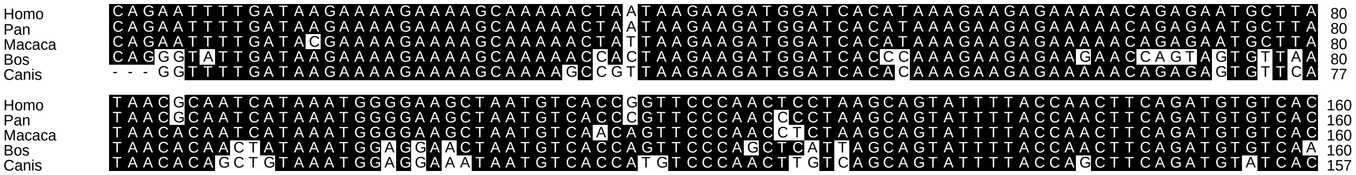

ENSG00000121931 intron 3

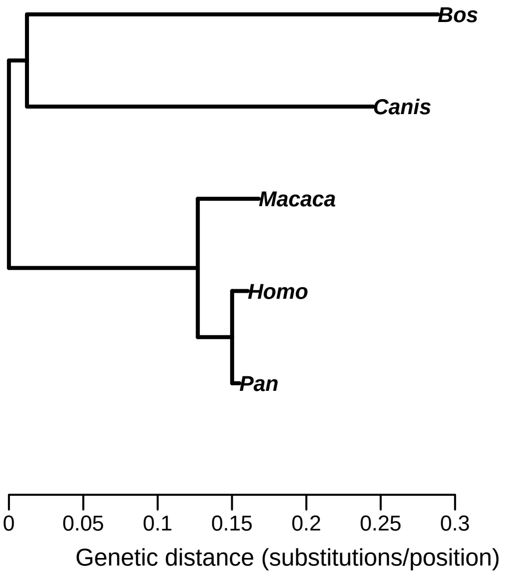

ENSG00000121931 intron 3

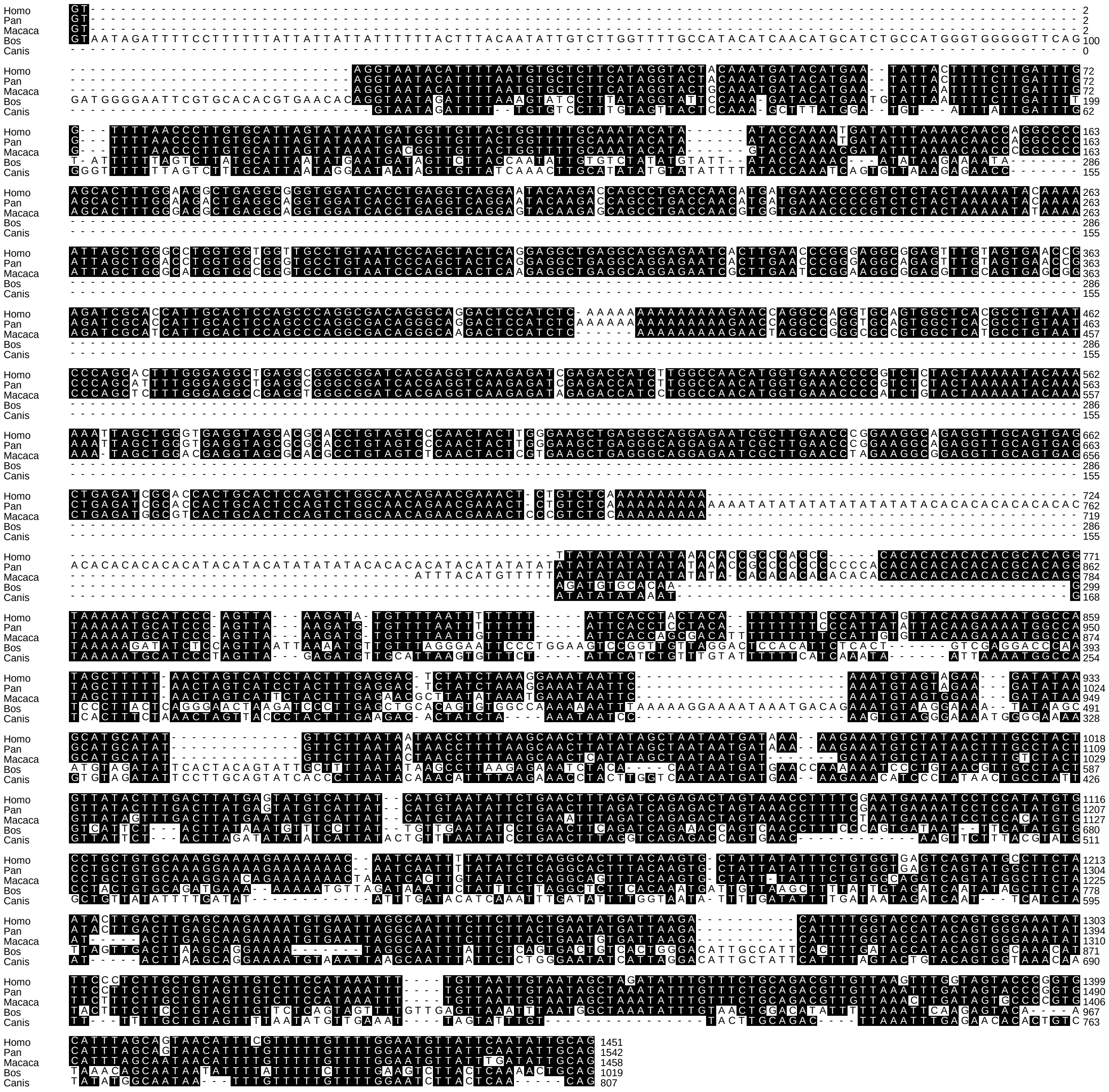

ENSG00000153048 intron 1

Description: Calcium-regulated heat stable protein 1 (CARHSP1)
Intron number: 1
Human chromosome: 16
Intron start (bp): 8859831
Human intron length : 698
Intron alignment length: 772
Flanking exons length (upstream/downstream): 158/123
SNP density: 0.005731
K tree score: 0.0794
Scaling factor: 0.7891
Human-chimpanzee distance: 0.026730
Total primate branch length: 0.0935

ENSG00000153048 exon 1

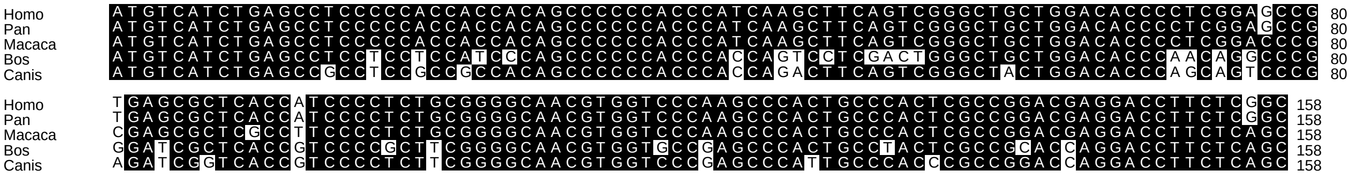

ENSG00000153048 exon 2

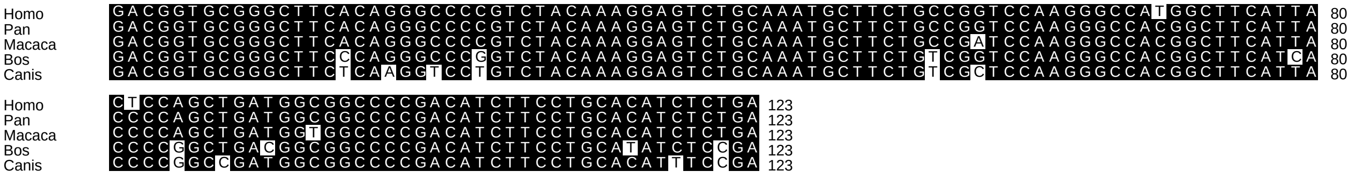

ENSG00000153048 intron 1

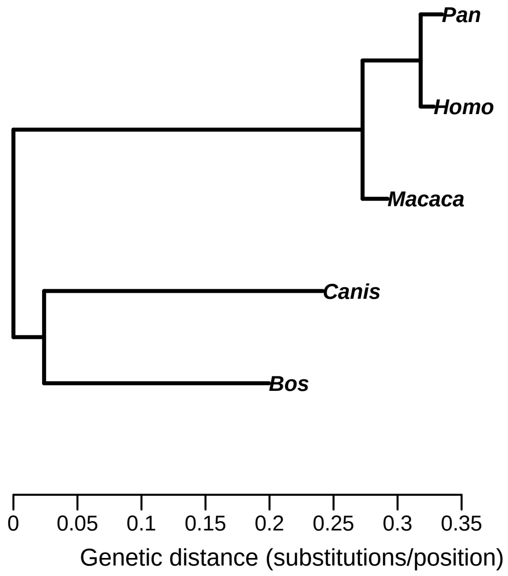

ENSG00000153048 intron 1

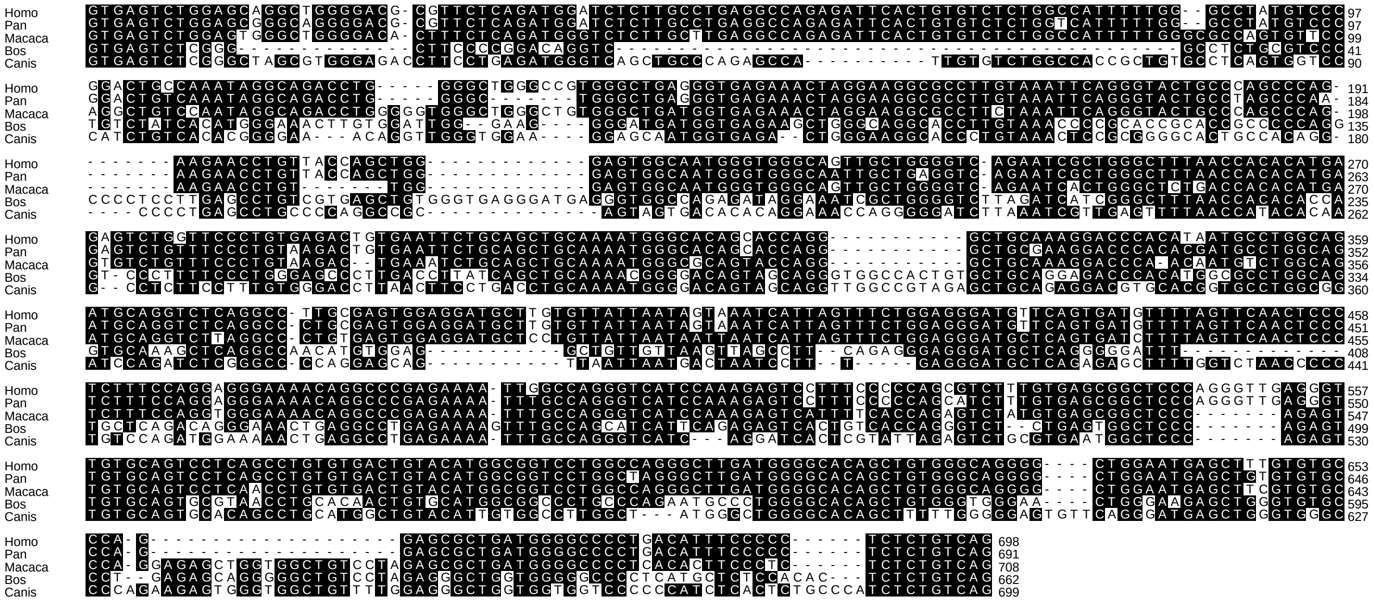

ENSG00000143921 intron 8

Description: ATP-binding cassette sub-family G member 8 (ABCG8)
Intron number: 8
Human chromosome: 02
Intron start (bp): 43952950
Human intron length : 1480
Intron alignment length: 1514
Flanking exons length (upstream/downstream): 84/200
SNP density: 0.007432
K tree score: 0.078
Scaling factor: 0.7883
Human-chimpanzee distance: 0.005483
Total primate branch length: 0.0935

ENSG00000143921 exon 8

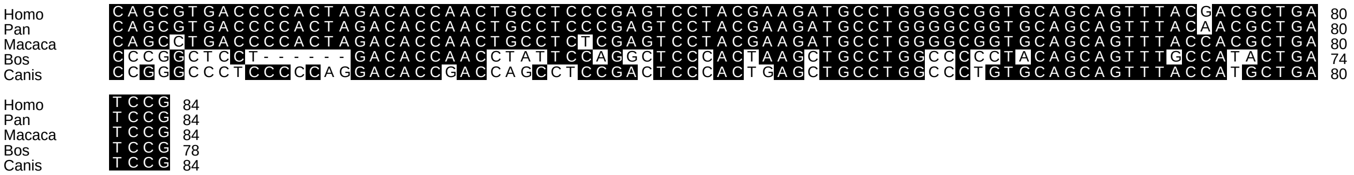

ENSG00000143921 exon 9

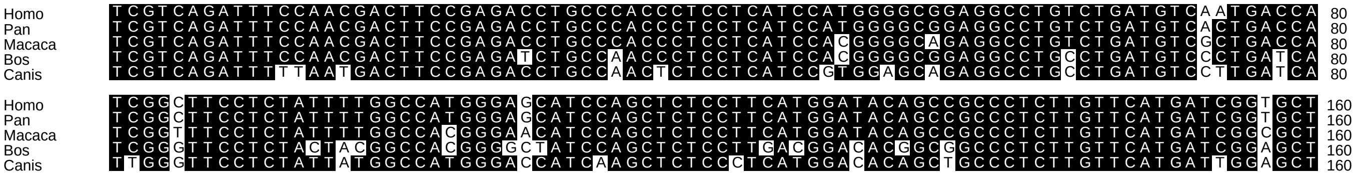

ENSG00000143921 intron 8

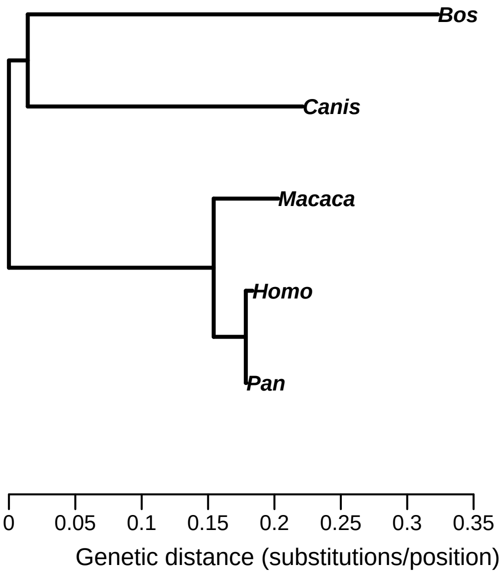

ENSG00000143921 intron 8

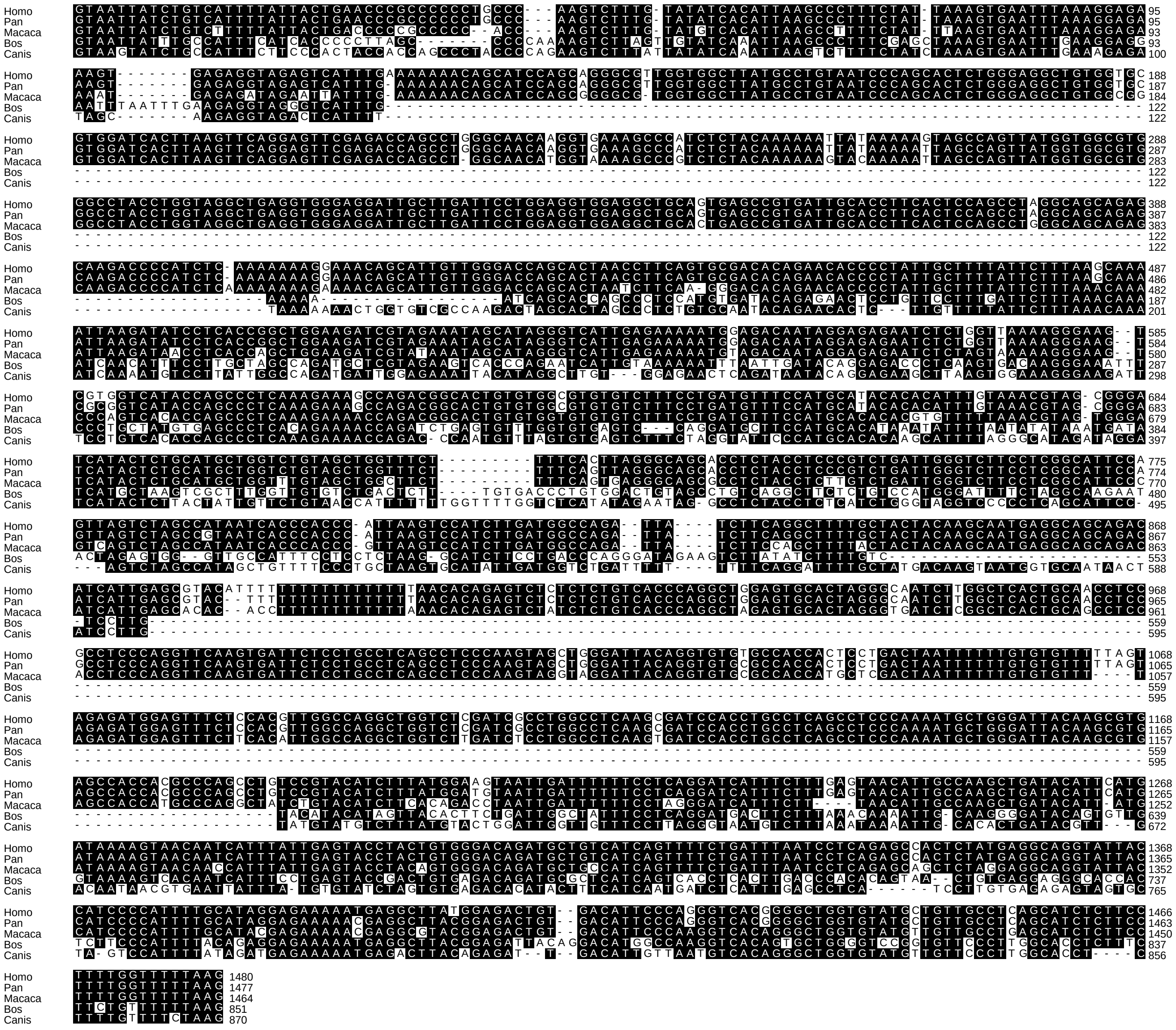

ENSG00000088451 intron 10

Description: dTDP-D-glucose 4,6-dehydratase (TGDS)
Intron number: 10
Human chromosome: 13
Intron start (bp): 94026667
Human intron length : 959
Intron alignment length: 992
Flanking exons length (upstream/downstream): 59/98
SNP density: 0.003128
K tree score: 0.0795
Scaling factor: 0.9716
Human-chimpanzee distance: 0.009478
Total primate branch length: 0.093

ENSG00000088451 exon 10

```
Homo    ATCAAAGAGACCAATTCAGAGTCTGAAATGGAAAATTGGGTTGATTATGTTAATGATAG 59
Pan     ATCAAAGAGACCAATTCAGAGTCTGAAATGGAAAATTGGGTTGATTATGTTAATGATAG 59
Macaca  ATCAAAGAGACCAATTCAGAGTCCGAAATGGAAAATTGGGTTGATTATGTTAATGATAG 59
Bos     ATCAAAGAGACCAATTCAGAGTCTGAAATGGAAAACTGGGTTGATTATGTTGATGATAG 59
Canis   ATCAAAGAGACCAGTTCAGAGTCTGAAATGGAAACCTGGGTGGATTATGTTAATGATAG 59
```

ENSG00000088451 exon 11

```
Homo    ACCCACCAATGACATGAGATACCCAATGAAGTCAGAAAAAATACATGGCTTAGGATGGAGACCTAAAGTGCCTTGGAAAG 80
Pan     ACCCACCAATGACATGAGATACCCAATGAAGTCAGAAAAAATACATGGCTTAGGATGGAGACCTAAAGTGCCTTGGAAAG 80
Macaca  ACCTACCAATGACATGAGATATCCAATGAAGTCAGAAAAAATACATGGCTTGGGATGGAGACCTAAAGTGCCTTGGAAAG 80
Bos     ACCTACCAATGACATGAGATATCCAATGAAGTCAGAAAAAATACATGGCTTGGGATGGAGACCTAAAGTACCTTGGAAAG 80
Canis   ACCTACCAATGACATGAGATATCCAATGAAGTCAGAAAAAATACAGGGCTTAGGATGGAGACCCAAAGTGCCTTGGAAAG 80

Homo    AAGGAATAAAGAAAACAA 98
Pan     AAGGAATAAAGAAAACAA 98
Macaca  AAGGAATAAAGAAAACAA 98
Bos     AAGGAATAAAAAAAACAA 98
Canis   AAGGAATAAAGAAAACAA 98
```

ENSG00000088451 intron 10

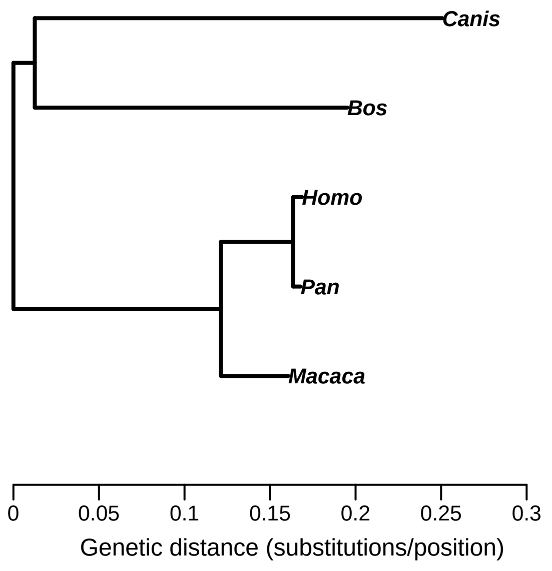

ENSG00000088451 intron 10

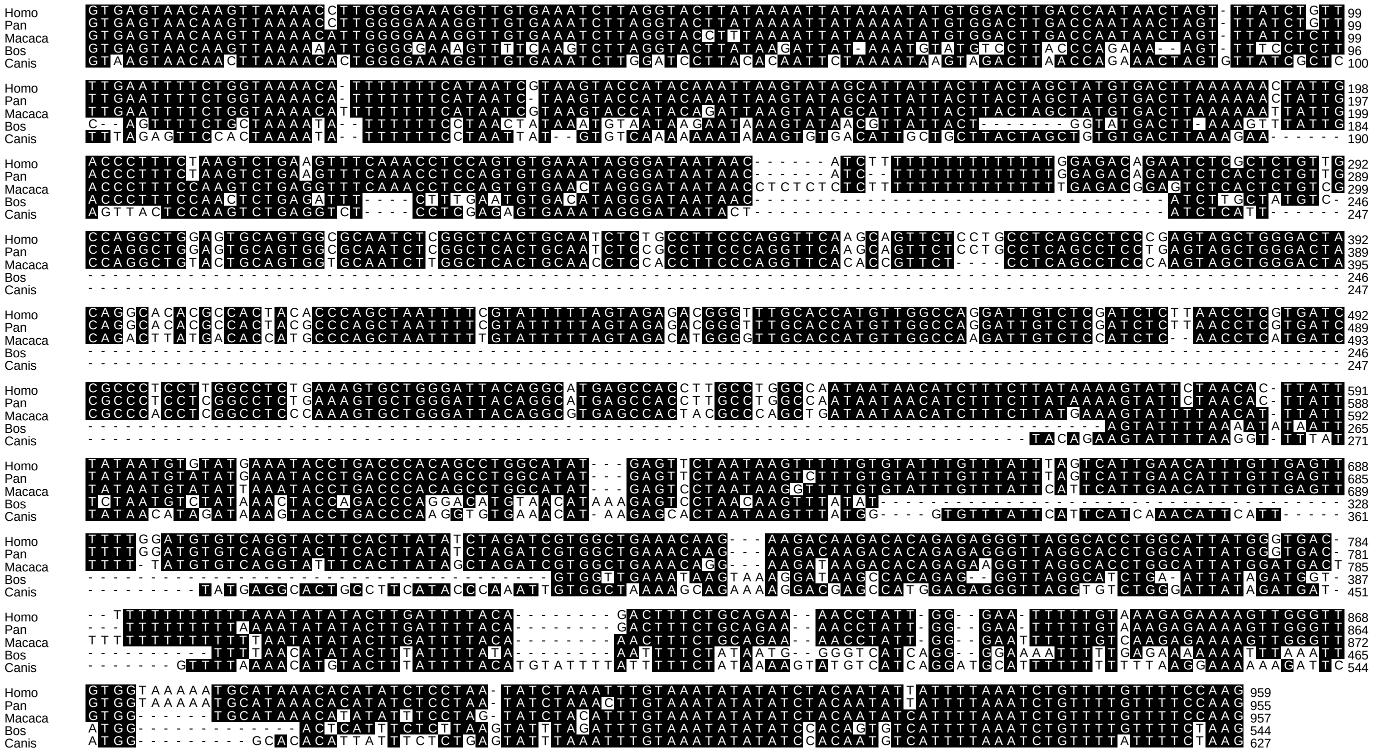

ENSG00000100941 intron 1

Description: Pinin (PNN)
Intron number: 1
Human chromosome: 14
Intron start (bp): 38714348
Human intron length : 685
Intron alignment length: 706
Flanking exons length (upstream/downstream): 113/72
SNP density: 0.011679
K tree score: 0.0402
Scaling factor: 1.1092
Human-chimpanzee distance: 0.015011
Total primate branch length: 0.0923

ENSG00000100941 exon 1

```
Homo    ATGGCGGTCGCCGTGAGAACTTTGCAGGAACAGCTGGAAAAGGCCAAAGAGAGTCTTAAGAACGTGGATGAGAACATTCG 80
Pan     ATGGCGGTCGCAGTGAGAACTTTGCAGGAGCAGCTGGAAAAGGCCAAAGAGAGTCTTAAGAACGTGGATGAGAATATTCG 80
Macaca  ATGGCGGTCGCAGTGAGAACTTTGCAGGAACAGCTAGAAAAGGCCAAAGAGAGCCTTAAGAACGTGGATGAGAATATTCG 80
Bos     ATGGCGGTCGCAGTGAGAACTTTGCAGGAGCAGCTAGAAAAGGCCAAAGAGAGCCTAAAGAATGTGGATGAGAATATTCG 80
Canis   ATGGCGGTCGCGGTGAGAACTTTGCAGGAGCAGCTAGAAAAGGCCAAAGAGAGCCTAAAGAACGTGGATGAGAATATTCG 80

Homo    CAAGCTCACCGGGCGGGATCCGAATGACGTGAG 113
Pan     CAAGCTCACCGGGCGGGATCCGAATGACGTGAG 113
Macaca  CAAGCTCACCGGGCGGGATCCGAATGACGTGAG 113
Bos     CAAGCTCACCGGGCGGGATCCGAATGATGTGAG 113
Canis   CAAGCTCACCGGACGGGATCCGAATGACGTAAG 113
```

ENSG00000100941 exon 2

```
Homo    GCCCATCCAAGCCAGATTGCTGGCCCTTTCTGGTCCTGGTGGAGGTAGAGGACGTGGTAGTTTATTACTGAG 72
Pan     GCCCATCCAAGCCAGATTGCTGGCCCTTTCTGGTCCTGGTGGAGGTAGAGGACGTGGTAGTTTATTACTGAG 72
Macaca  GCCCATCCAAGCCAGATTGCTGGCCCTTTCTGGTCCTGGTGGAGGTAGAGGACGTGGTAGTTTATTACTGAG 72
Bos     GCCCATCCAAGCCAGGTTGCTCGCCCTTTCTGGTCCTGGTGGAGGTAGAGGACGTGGTAGTTTATTGCTGAG 72
Canis   GCCCATCCAAGCCAGATTGCTGGCCCTTTCTGGTCCTGGTGGAGGTAGAGGACGTGGTAGTTTATTGCTGAG 72
```

ENSG00000100941 intron 1

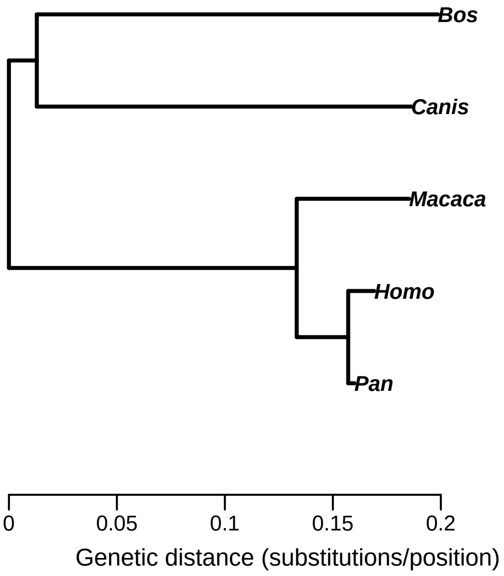

ENSG00000100941 intron 1

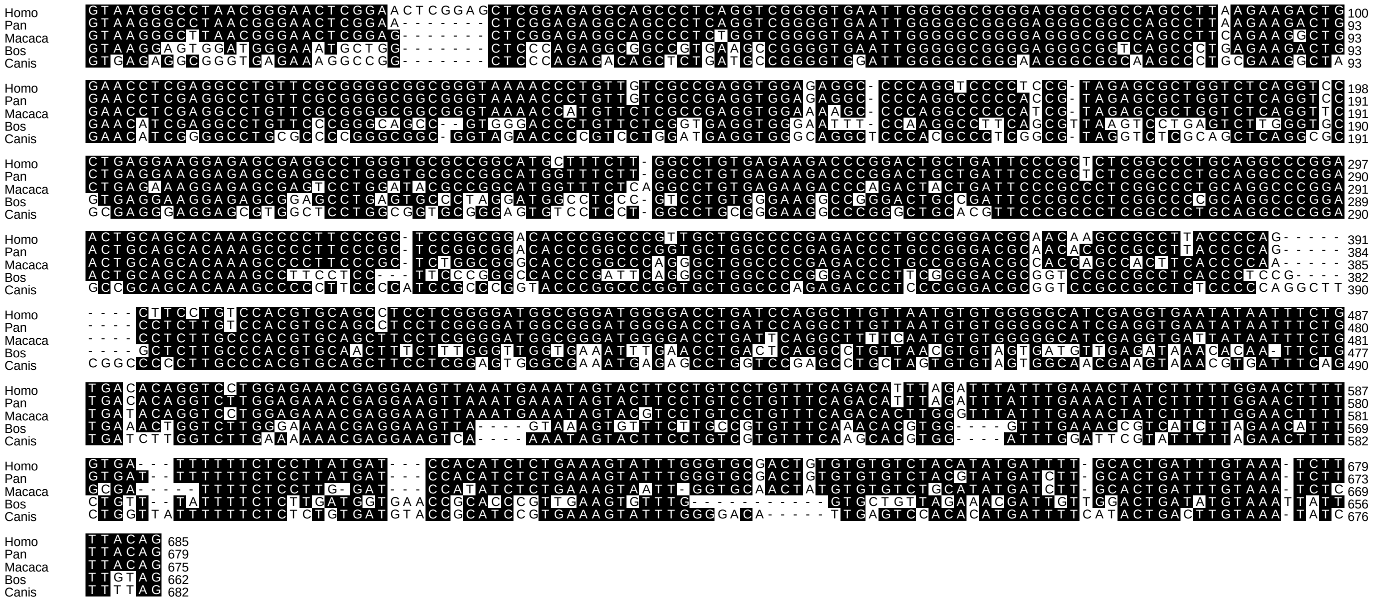

ENSG00000100985 intron 2

Description: Matrix metalloproteinase-9 precursor (MMP9)
Intron number: 2
Human chromosome: 20
Intron start (bp): 44072145
Human intron length : 384
Intron alignment length: 419
Flanking exons length (upstream/downstream): 233/149
SNP density: 0.010417
K tree score: 0.0658
Scaling factor: 0.8673
Human-chimpanzee distance: 0.013557
Total primate branch length: 0.0921

ENSG00000100985 exon 2

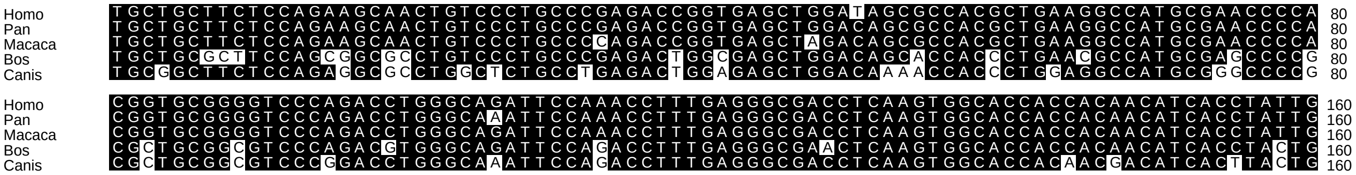

ENSG00000100985 exon 3

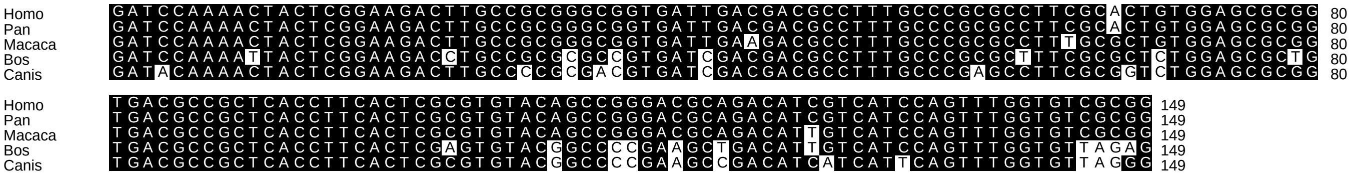

ENSG00000100985 intron 2

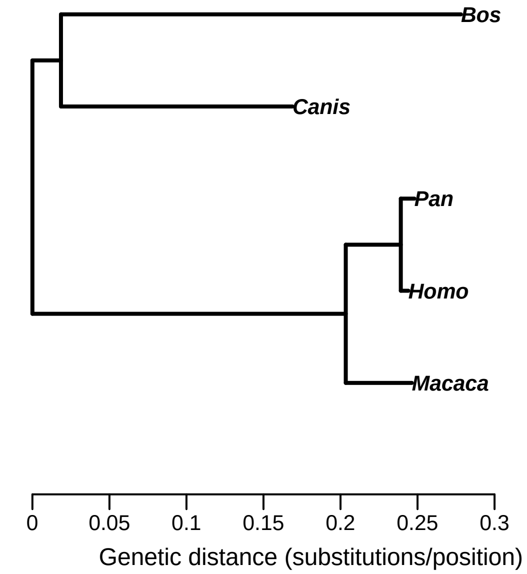

ENSG00000100985 intron 2

```
Homo    GTGAGCCGGGG----CCGTGGGGGC--------AGCGGGGTGGGGCGGGGAGGCCAGGTCTGGCTCTTGGG------CCAGCGGTGAACATGTCCTGTCT 82
Pan     GTGAGCCGGGG----CCGTGGGGGC--------AGCGGGGTGGGGCGGGGAGGGCGGGTCTGGCTCTTGGG------CCAGGGGTGAACATGTCCTGTCT 82
Macaca  GTGAGCCGCGG----CCGCGGGGACGGGGTGGGGGGGCGGTGGGGCGGGGAGGGCAGGTCTGGCTCTCGGG------CCAGCGGTGAACACGTCCTGTCT 90
Bos     GTGCGCGGCTGGGCCCCGCGGGGGC-------GAGCGGGGTGCAGAGGGTGGCGAGAGGCGGGTTCCCAAGCGCCGCCCCTTGGAGAACACATCCCGTCT 93
Canis   GTGCGCGGCCGGGCTCCGCGGAGGC--------------GCGGGACCGGGGCGGGAGGGCCAGACCCCGCG------CGGGCGGTGATCTCATC----CT 76

Homo    TGGACGCGTCCCTGGGTTTCACTATTTAATGTG--TGGCCCCTGGGGAGTGTCCCCACCTCTGAGCCTCTGTTTCTCC--TTCAGGGAAATGGCTCTTGC 178
Pan     TGGACGCGTCCCTGGGTTTCACTATTTAATGTG--TGGCCCCTGGGGAGTGTCCCCACCTCTGAGCCTCTGTTTTTCC--TTCAGGGAAATGGCTCTTGC 178
Macaca  TTTACAGGTCCCTGGGTTTCACTATTTAACGTG--TGACCCCGGGTGAGTGTCTCCACCTCTGAGCCTCAGTTTTTCC--TTCAGGGAAATGGCTCTTGC 186
Bos     CAGGCCAATCCATGGCTTTCA---CTGAATGTGCCCGCCTTGAGGCAAGTGGCTCCCCCTCAGAGCCTCCGTCTCCCCCATCGGGGGAAATGGACCTCGC 190
Canis   TAGACGGGTCCCTGGGT-------TTAAATGGG--CAGCCTGGGGCAAGTGGCTCCACCTCTGAGCCCCTGTTTCTCC--TTCAGGGAAATGGGTCTCGC 165

Homo    A-----ATCCAAGTCCTCCTGCCAGGGCCATTGTGAGGGTCTAAGTAGACAAAAAAAAAAAAAAAAAAAAAACAGTCTGGAAGCAATTTATAGATGAGAGCG 273
Pan     A-----ATCCAAGTCCTCCTGCCAGGGCCATTGTGAGGGTCTAAGTAGAC------------AAAAAAAACAGTCTGGAAGCAATTTATAGATGAGAGCG 261
Macaca  A-----ATCCAAGTCCTCCTGCCAGGGCCATTGTGAAGGTCTAAGTAGAT--------------AAAAAACAGTCTGGAAGCAATTTATAGATGAGAGCG 267
Bos     C-----AACTGACTCC--------------------------AAAGAG----------------AAAAGTGCGTCTGAAAGTGCTTTCGAGATGAAAGCG 243
Canis   ACGCACCCTTGAATCTTCCTGCTAGGGCCATTGTGAGCGTCCCAAGAG----------------AAAAATGCGTCTGAGGGCGCTTTGTAGATGCAAGCG 249

Homo    TGGACGGCAGAGAGCATTGTGTATGTTGAAGTCTCTGCGATATGGGGTGTCCCTGCTGCCCC-----GCTCCAGCCTTTCACTTCTGACCTCCTTCCTCT 368
Pan     TGGACGGTAGAGAGCATTGTGTATGTTGAAGTCTCTGCGATATGGGGTGTCCCTGCTGCCCC-----GCTCCAGCCTTTCACTTCTGACCTCCTTCCTCT 356
Macaca  TGGACGGCAGGGAACATTGTGTATGTTGAAGTCTCTGCCATATGGGGCGTCCCTGCTGCCCC-----GCTCCAGCCTTTCACTTCTGACCTGCTTCCTCT 362
Bos     TGGAGCGCAGGCAGGGCTATGTAT-TTGAAGTCGCGGAGACACCGGGTGGCACTGCTGGCCC----GGCTCCCACCTCTCACTTCTGCCCTTTATCCTCC 338
Canis   CGGAGCGCAGGGAGCACTGCGCTTGCTGAGGTCGCTGACACACCGGGTGTCGCTGCTACTGCCCCGGGCTCCGGCC--TCACTTCTGACCTATAGCCTCC 347

Homo    GGCTCTTACGC---TACAG 384
Pan     GGCTCTTACGC---TACAG 372
Macaca  GGCTTTTACTC---TACAG 378
Bos     TGCCTTTTCCT---TCCAG 354
Canis   CGCCTTTCCCCCCGCCCAG 366
```

ENSG00000155666 intron 2

Description: jumonji domain containing protein 5 (JMJD5)
Intron number: 2
Human chromosome: 16
Intron start (bp): 27132574
Human intron length : 1124
Intron alignment length: 1285
Flanking exons length (upstream/downstream): 167/133
SNP density: 0.001779
K tree score: 0.0471
Scaling factor: 0.6684
Human-chimpanzee distance: 0.020129
Total primate branch length: 0.092

ENSG00000155666 exon 2

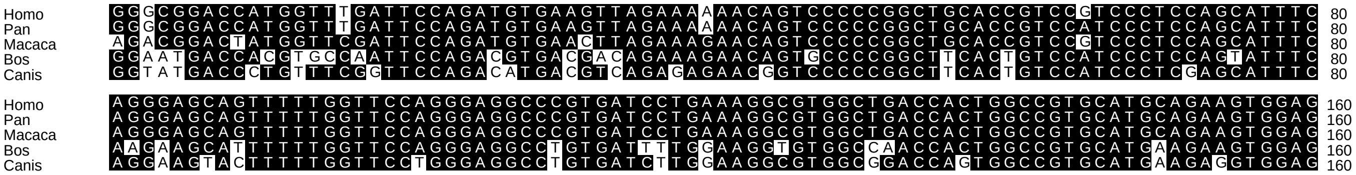

ENSG00000155666 exon 3

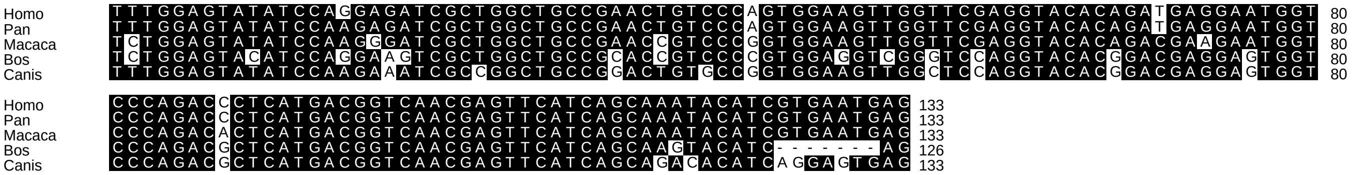

ENSG00000155666 intron 2

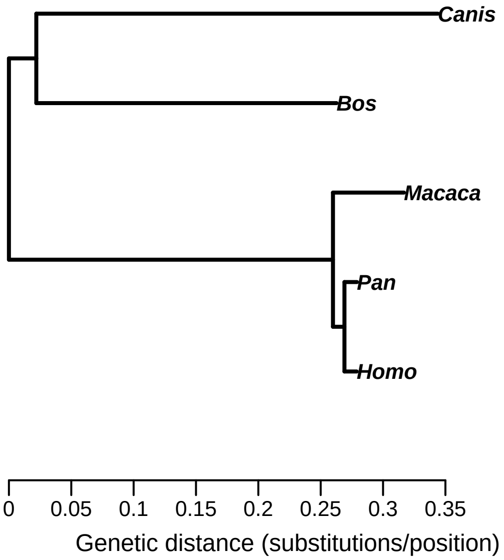

ENSG00000155666 intron 2

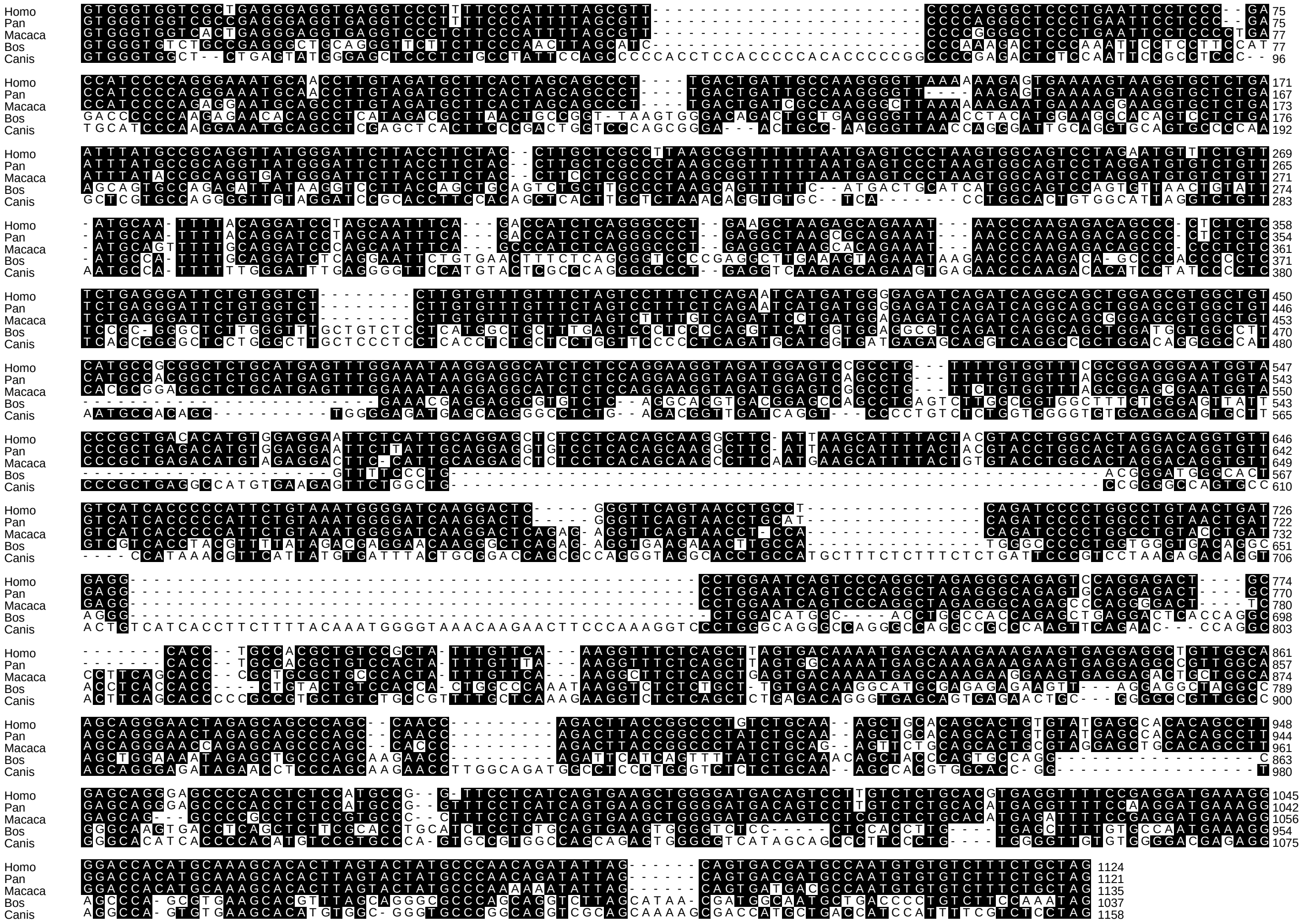

ENSG00000155906 intron 6

Description: Required for meiotic nuclear division protein 1 homolog (RMND1)
Intron number: 6
Human chromosome: 06
Intron start (bp): 151785434
Human intron length : 919
Intron alignment length: 997
Flanking exons length (upstream/downstream): 107/65
SNP density: 0.006529
K tree score: 0.0258
Scaling factor: 1.0313
Human-chimpanzee distance: 0.009999
Total primate branch length: 0.0918

ENSG00000155906 exon 6

```
Homo    GGGACAGTCAAAACTTCACAGGGGGGAAATCAAGTTAAATTCAGAGCTGGATTTAGATGATGCCATTCTAGAGAAGTTTG 80
Pan     GGGACAGTCAAAACTTCACAGGGGGGAAATCAAGTTAAATTCAGAGCTGGATTTAGACGATGCCATTCTAGAGAAGTTTG 80
Macaca  GGGACAGTCAAAACTTCACAGGGGGGAAATCAAGTTAAATTCAGAGCTGGATTTAGATGATGCCATTCTAGAGAAGTTTG 80
Bos     GGGGCAGTCAAAACTTCACAGAGGGGAAATCCGGTTAAATTCAGAGCTGGATTTAGATGATGTCATTCTAGAGAAATTTG 80
Canis   GGGACAGTCGAGACTTCACAGAGGGGAAATCAGATTAAATTCAGAGCTAGATTTAGATGACGCCATTCTAGAGAAATTTG 80

Homo    CTTTCTCCAATGCTCTATGCCTTTCTG 107
Pan     CTTTCTCCAATGCTCTATGCCTTTCTG 107
Macaca  CTTTCTCCAATGCTCTCTGCCTTTCTG 107
Bos     CTTTCTCGAATGCCCTTTGCCTCTCAG 107
Canis   CTTTCTCTAATGCTCTTTGTCTTTCAG 107
```

ENSG00000155906 exon 7

```
Homo    TAAAACTGGCAATTTGGGAAGCATCACTGGATAAATTTATTGAATCTATTCAGTCAATTCCTGAG 65
Pan     TAAAACTGGCAATTTGGGAAGCATCACTGGATAAATTTATTGAATCTATTCAGTCAATTCCTGAG 65
Macaca  TAAAACTGGCAATTTGGGAAGCATCACTGGATAAATTTATTGAATCTATTCAGTCAATTCCTGAG 65
Bos     TGAAACTTGCTATCTGGGAAGCATCACTGGATAAATTTGTTGAATCTATTCAGTCGATTCCAGAG 65
Canis   TGAAACTGGCTATTTGGGAGGCATCACTGGATAAATTTGTTGAGTCTATTCAGTCAATTCCAGAG 65
```

ENSG00000155906 intron 6

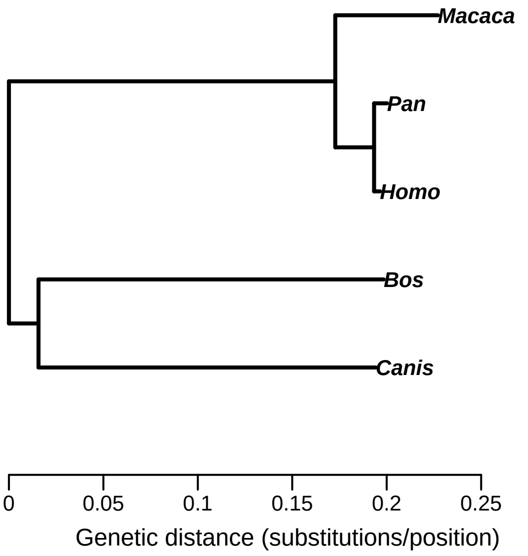

ENSG00000155906 intron 6

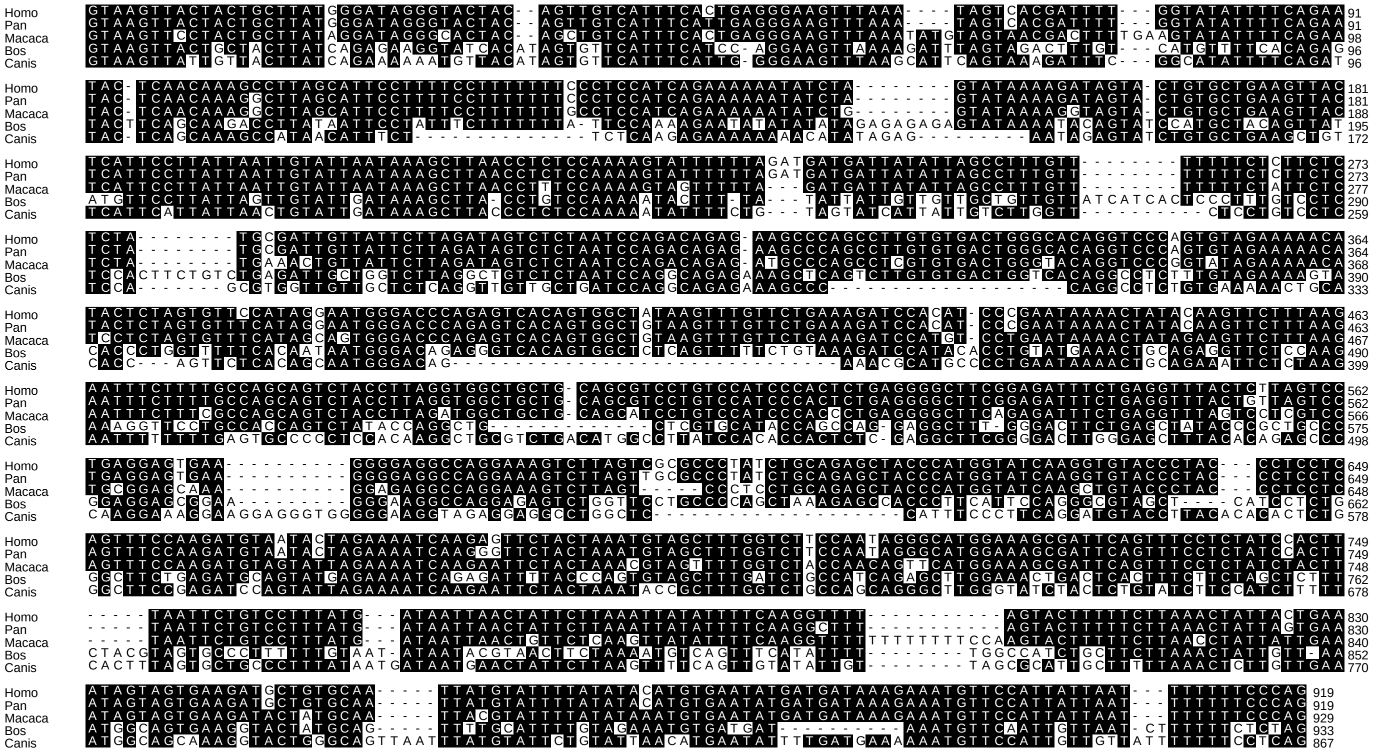

ENSG00000205073 intron 3

Description: Unknown (C1orf222)
Intron number: 3
Human chromosome: 01
Intron start (bp): 1844799
Human intron length : 275
Intron alignment length: 281
Flanking exons length (upstream/downstream): 155/133
SNP density: 0.000000
K tree score: 0.0672
Scaling factor: 0.4276
Human-chimpanzee distance: 0.003660
Total primate branch length: 0.0917

ENSG00000205073 exon 3

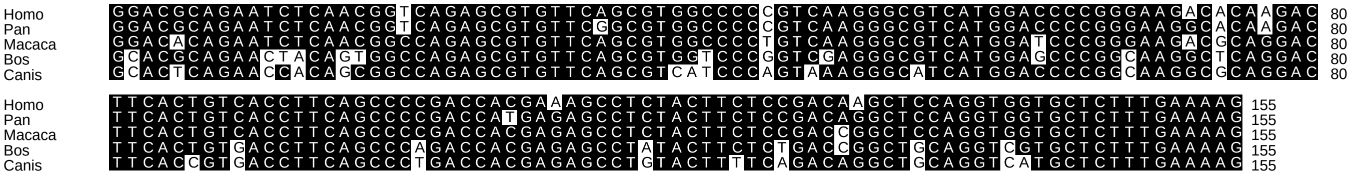

ENSG00000205073 exon 4

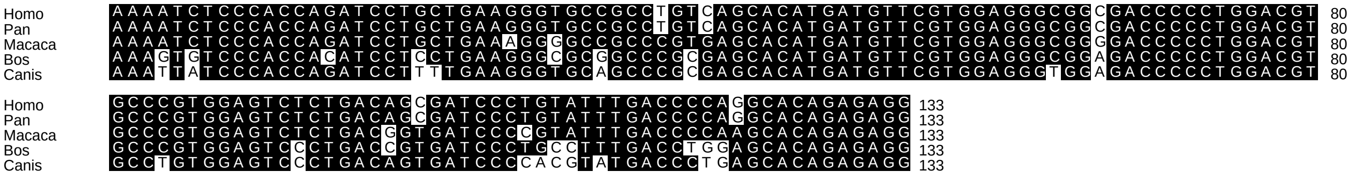

ENSG00000205073 intron 3

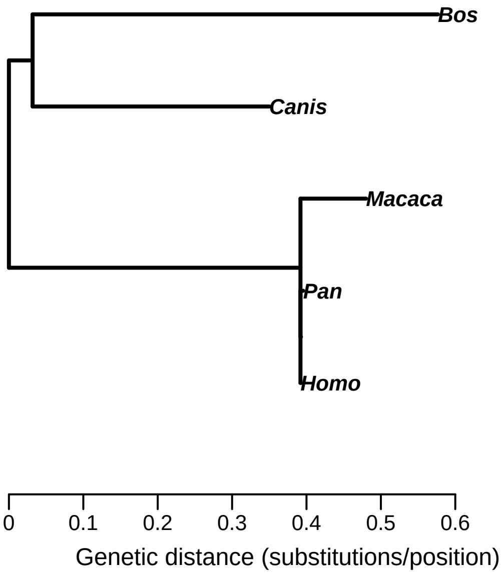

ENSG00000205073 intron 3

```
Homo    GTGCGGCTCTGGCTCTGCAAGCTCAGCCCGGCCCGGCCCTGGCCCCACGCAGGGCCTTGCCTTTGGCTGCACTGCTCCACTTTGGAGGCCCTGAGGGTAC 100
Pan     GTGCGGCTCTGGCTCTGCAAGCTCAGCCCGGCCTGGCCCTGGCCCCACGCAGGGCCTTGCCTTTGGCTGCACTGCTCCACTTTGGAGGCCCTGAGGGTAC 100
Macaca  GTGCGGCTCTGGCTCTGCAAGTTCAGCTGGGCCCGGCCCTGGCCCCAACCAGAGCCTTGCCTTTCGCTGCACTGCCCCACTTTGGAGACCCGGAGGGTAC 100
Bos     GCAGGACTCCG---------ACCCAGCCTGGACCCCTCCTCCCCACCCGCATGTCCATTTGTCCAAGCCCTTTGGCTGCCTGAGCGGCCCCACATTGGAG 91
Canis   GTG------TGGTTCCTCCAGCCCAGTCGTGACCCCCTTCCCGAGTGCCCAGGGAAGGGGGCCTAACCTGCCTGCCCTACTTTGAAGGCCCTGG----CC 90

Homo    CATGG----CTGGGGGACTCAGCACCACACTGAGGGCCGTC-AGTGGAAAGACCAGAGTCCAGGGCCCCACGGGGCCCCCAGGCCGTCCCTGCTTCCTTC 195
Pan     CATGG----CTGGGGGACTCAGCACCACACTGAGGGCCGTC-AGTGGAAAGACCAGAGTCCAGGGCCCCACGGGGCCCCCAGGCCGTCCCTGCTTCCTTC 195
Macaca  CATGG----CTGGGGGACTCAGCACCACACTGAGGGTCATG-GGTCGAGAGACCAGAGTCCAGGGCCCCACGGGGCCCCCAGGCCATCCCTGCCTCCTTC 195
Bos     CTTGGTGCCTGGGGGGGCTCCACATCAGGCTGGGGGTCATCAGGTGGAGAGACCAATGTCTGGGGTCCCACAGGCCCACATCACAGCCCCGC--CCCCTC 189
Canis   CGTGG----CC--AGGACTCCACACCA---TGGGTGTTGTCTGGTATAGAGGCCAGTGTCCCAGGTCCCACGGGCCCAC---------------ATCCTC 166

Homo    CCTGAGCCAGCCCAGCTCTCACCAGCAGGACCCCTGCCGCCGAGCTTCGGGGACA-AGGCCACTCCACTTCTTGTCCCCAG 275
Pan     CCTGAGCCAGCCCAGCTCTCACCAGCAGGACCCCTGCCGCCGAGCTTCGGGGACA-AGGCCACTCCACTTCTTGTCCCCAG 275
Macaca  CCTGAGCCAGCCCAGCTCTCACCAGCAGGGCCCCTGCTGCGGAGCTTTGGGGACA-AGGCCACTCCACTTCTTGTCCCCAG 275
Bos     CCAGAGCC--------------------------------CCACATGCAGGGTTGCAAACCGCTGTGCTCCT-GCCCCCAG 237
Canis   TTGGAGCTGGGTCA--------------------------TGATGTTGCAGGACG-AGGCTACTGTGCTCTTCGTCCCCAG 220
```

ENSG00000166825 intron 15

Description: Aminopeptidase N (ANPEP)
Intron number: 15
Human chromosome: 15
Intron start (bp): 88136798
Human intron length : 472
Intron alignment length: 886
Flanking exons length (upstream/downstream): 92/111
SNP density: 0.012712
K tree score: 0.0589
Scaling factor: 0.9494
Human-chimpanzee distance: 0.025480
Total primate branch length: 0.0913

ENSG00000166825 exon 15

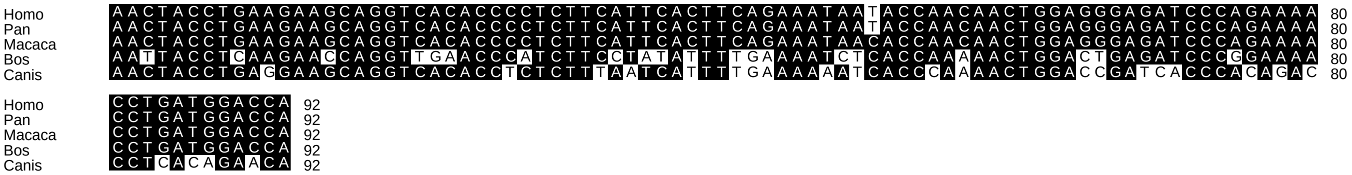

ENSG00000166825 exon 16

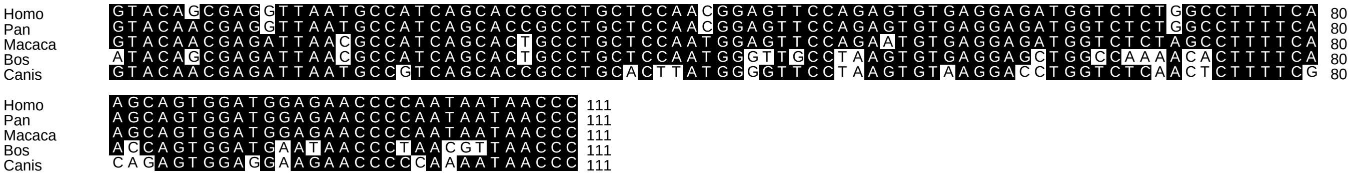

ENSG00000166825 intron 15

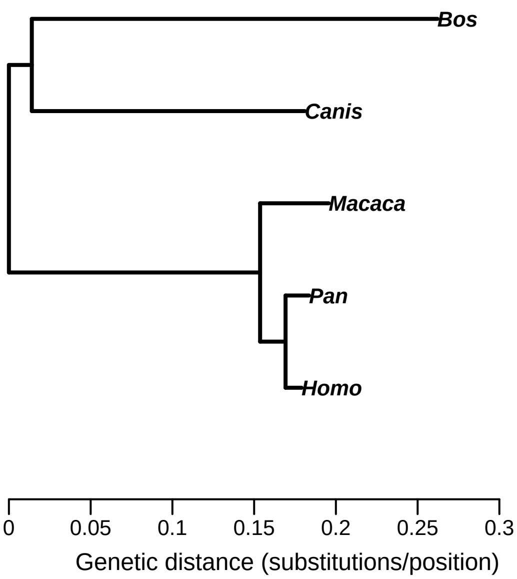

ENSG00000166825 intron 15

```
Homo    GTGAGTG-------GGAGCTCTCATGGGTTTG------------------GAAGTCCTGGGCACCCCGG-------------GGAGTCAAGAGCAACATC 62
Pan     GTGAGTG-------GGAGCTCTCATGGGTTTG------------------GAAGTCCTGGGCACCCCGG-------------GTTGTCAAGAGCAACATC 62
Macaca  GTGAGTG-------GGAGCTCTCATGGGTTTG------------------GAGGTCCTGGGCATCCCAG-------------AGAGTCAAGAGCAACATC 62
Bos     GTGAGTA-------TGACCTGTCTTGGGTCTT---------------------GGCCCAGACACCCTGA--------CCTGGGAAGTCTAGAGCAAGTTC 64
Canis   GTGAGTATGCAGTTGAGAAGCTCAGGGCCTTGCTCGTTTCCTCATTCCCAGAGGTCATGGGCGGCCCAGCCACCCTGACTGAGGAGTCCAGAGCACAGCC 100

Homo    CTG--GCAGTGTTCTCTG-------CCGCAGCTCTCAATGCGGGGGCACCAGGCAGGGAGAGA----------------CCCCCACCCAGGAGCAGGGAG 137
Pan     CTG--GCAGTGTTCTCTG-------CCGCAGCTCTCAATGCAGGGGCACCAGGCAGGGAGAGA----------------CCCCCACCCAGGAGCAGGGAG 137
Macaca  CTG--GCAGTGTTCTTTG-------CCGCAGCTTTCAATGCGGGGGCACCAGGCAGGGAGAGG----------------CCCCCACCCAGGAGCAGGGAG 137
Bos     CCACAGCAGCCTCCTCCGAACTTCCCAATTCCTGGCTAGACCAGGGCCACCAGTCAGGGAGGAAACCGCCCCCTCCCTGACCCCACCCAGGAGTGGGGG- 163
Canis   CTGCAGTATCCTCCTTTG-------GAAGACATCTCTGGACCTGGGCAACCAGCAGGGAAAGA----------------ACCCCTCCTAGGAGTAGGAGG 177

Homo    -GTCCTGCCCTGAGGCTCCATGTGGCCTGAGGGCCCATCGGACCTCTTTATACAAAGACCCAAACTGAAACTCT-AGCTCACCCCACTGGCTTGA----- 230
Pan     -GTCCTGCCCTGAGGCTCCATGTGGCCTGAGGGCCCCTCGGACCTCTTTATACAAAGACCCAAACTGAAACTCT-AGCTCACCCCACTGGCTTGA----- 230
Macaca  -GCCCTGCCCTGAGGCTCCATGTGGCCTGTGGGTCCATCGGACCTCTTTATACAAAGACCCAAACTGAAACTCTGAGCTCATCCCACTGGCTTGA----- 231
Bos     -GCCCTGCCCCAAGGCTCCATGTGGCCTGTGAACCCGTCTGGCCTCTCTGTGTGAAGGACCAAACTGAACCTCCCTAGTCCCTTCACTGGCAT------- 255
Canis   TGGCCTGCCCTAAGGCTCTTTGTGGCCTGTGGGGCCATCTGGCC-CTTTGTATAAGAGACCAAACTGAAACTCTGAGGTC-CCTCACTGGCTTGGTCATT 275

Homo    ---------------------------------------------------------------------------------------------------- 230
Pan     ---------------------------------------------------------------------------------------------------- 230
Macaca  ---------------------------------------------------------------------------------------------------- 231
Bos     ---------------------------------------------------------------------------------------------------- 255
Canis   TATTTTATTTTAAAGATTTATTTATATATTTGAGAGCAAGAGAGCAAGAGCACATGAGCTGGGGAGGGACAGAGGGGGAGAGAGAGAGAGAGAGAGAGAA 375

Homo    ---------------------------------------------------------------------------------------------------- 230
Pan     ---------------------------------------------------------------------------------------------------- 230
Macaca  ---------------------------------------------------------------------------------------------------- 231
Bos     ---------------------------------------------------------------------------------------------------- 255
Canis   CCCCCAGCAGATTCCCCACTGAGCACAGAGCCCAATGTGGGGCTCGATCCCAGGACCCCCTGAAATCAGGACCTGAGCTGAAATCAAGAGCTGGCTGTTT 475

Homo    ------------------------GGTTATACAGTTTCTAGGTGTTGAAACTGGGGCTGGAGCCTGTTCCTGCCCTTCTGGGTCACAGGGCTGCTTTGGG 306
Pan     ------------------------GGTTATACAGTTTCTAGGTGTTGAAACTGGGGCTGGAGCCTGTTCCTGCCCTTCTGGGTCATGGGGCTGCTTTGGG 306
Macaca  ------------------------AGTTATATGGTTTGCAGATGTTGAAACTGGGGCTAGAGCCTGTTCCTGCCCTTTTGGGTCTCTGGGCTGCTTTGGG 307
Bos     ------------------------GGTCATGCAGATTCTAGGTGTTAAAGGTGGGGCTGGGGTCTGTGCCAGCCC-------TCCCTGGGCT-------- 316
Canis   AACAGAGCCACCCATGCGCCCCTTGGTCATTTAGATTCTAAATGTTCAAATTGGGGCTGGAGCCTGTGCTGACCCCTCTAGGTCCCTGGGCT-------- 567

Homo    GACCAAGGAACCTCAGGCCCAAGGCACAAAGCACATACAGCGGGGGTCAGAGGGCCAGTTA-----GGCCACAGAGCCTTAGGCAGG---------GTGC 392
Pan     GACCAAGGAACCTCAGGCCCAAGGCACAAACCACATACAGGGGGGCCCAGAGGGCCAGTTA-----GGCCACAGAGCCTTAGGCAGG---------GTGC 392
Macaca  GACCAAGGAACCTCAGGCCCAAGGCACAAAACACATACCGGGGGGCCCAGAGGGCCAGTTA-----GGCCACAGAGCCTTAGGCAGG---------GTGC 393
Bos     -----------------------GCACAAGGCAAGAAGCAAAGCACCCAAAGGGTAGGTGGGCTGTGGCCTTAGAGCCCCACAC------------CTGC 381
Canis   ----------------GTCCAAGGCACAGA--GCAAACAGAGGAACCCAGTGGGTGGGATG-----GACTGC--AGCCTCAGACAGAGCCTGGGCCGTGC 642

Homo    CGGCCT----GGGGTTGTGTGTCAAGAGGT---------------------------------------------------------------------- 418
Pan     CGGCCT----GGGGTTGTGTGTCAAGAGGT---------------------------------------------------------------------- 418
Macaca  CGTCCT----GGGATTGTGTGTCAAGAGGT---------------------------------------------------------------------- 419
Bos     TGCCCTACCTGGAGTTATATGCCAGCAGGTGGGCTACAGTCCACAGGGTAGCAAAGAGTCAGACACAAAGAGTCAGACACGACTGAAGTGACTGAGCGTA 481
Canis   CATCCTCTCCAGAGCTCTGCATCAGCAGGG---------------------------------------------------------------------- 672

Homo    ----------------GAATCTTGGCTGGAGTT--CGCCAGGTG-----------TGCTAACCGCGTG---TCACCCCTGGGGCAG 472
Pan     ----------------GAATCTTGGCTGGAGTT--GGCCAGGTG-----------TGCTAACCGCGTG---TCACCCCTGGGGCAG 472
Macaca  ----------------GAATCTTGGCTGGAGGT--GGCCAGGTG-----------TGCTAACCGCGTG---TCACCCCTGGGGCAG 473
Bos     AGTACGAGCACAGGGAGAGTCTTTGCTGGAGCCAGGGCCAGGCA-----------TGCTGACAGAGTGTCATCACCCCTGGTGCAG 556
Canis   ----------------GAATCTTTGCCAGAGTCTAGGCCAGGTGACCCATGGGCCTGCTGACCAAGTG---TCACCCCTGGGACAG 739
```

ENSG00000136878 intron 17

Description: Ubiquitin carboxyl-terminal hydrolase 20 (USP20)
Intron number: 17
Human chromosome: 09
Intron start (bp): 131677056
Human intron length : 354
Intron alignment length: 712
Flanking exons length (upstream/downstream): 127/170
SNP density: 0.002825
K tree score: 0.0875
Scaling factor: 0.5303
Human-chimpanzee distance: 0.023037
Total primate branch length: 0.091

ENSG00000136878 exon 17

```
Homo    GTGGGCACTACATCGCCTACTGCCAGAACGTGATCAATGGGCAGTGGTACGAGTTTGATGACCAGTACGTCACAGAAGTC 80
Pan     GTGGGCACTACATCGCCTACTGCCAGAACGTGATCAATGGGCAGTGGTACGAGTTTGATGACCAGTACGTCACAGAAGTC 80
Macaca  GTGGGCACTACATCGCCTACTGCCAGAATGTGATCAATGGGCAGTGGTACGAATTCGACGACCAGTACGTCACAGAAGTC 80
Bos     GTGGCCACTACATCGCCTACTGCCAGAACGTGATCAACGGGCAGTGGTATGAGTTCGACGACCAGTACGTCACCGAGGTC 80
Canis   GTGGCCACTACATCGCCTACTGCCAAAATGTGATCAACGGGCAGTGGTATGAGTTCGACGACCAGTATGTCACTGAGGTC 80

Homo    CACGAGACGGTGGTGCAGAACGCCGAGGGCTACGTACTCTTCTACAG 127
Pan     CACGAGACAGTGGTGCAGAACGCTGAGGGCTACGTACTCTTCTACAG 127
Macaca  CATGAGACAGTGGTGCAGAACGCCGAGGGCTACGTCCTCTTCTACAG 127
Bos     CACGAGACGGTGGTGCAGAACGCGGAGGCCTACGTGCTCTTCTACAG 127
Canis   CACGAGACGGTGGTGCAGAGCGCTGAGGCCTACGTTCTCTTCTACAG 127
```

ENSG00000136878 exon 18

```
Homo    GAAGAGCAGCGAGGAGGCCATGCGGGAGCGACAGCAGGTGGTGTCCCTGGCCGCCATGCGGGAGCCCAGCCTGCTGCGGT 80
Pan     GAAGAGCAGCGAGGAGGCCGTGCGGGAGCGACAGCAGGTGGTGTCCCTGGCCGCCATGCGGGAGCCCAGCCTGCTGCGGT 80
Macaca  GAAGAGCAGCGAGGAGGCCGTGCGGGAGCGGCAGCAGGTGGTGTCCCTGGCCGCTATGCGGGAGCCCAGCCTGCTGCGGT 80
Bos     GAAGAGCAGCGAGGAAGCTGTGCGGGAGCGACAGCAGGTGGTGTCCCTGGCCGCCATGCGGGAGCCCAGCCTGCTGCGGT 80
Canis   GAAGAGCAGTGAGGAGGCTGTGCAGGAGCGGCAGCAAGTGGTGTCCCTGGCTGCCATGCGGGAGCCCAGCCTACTGCGGT 80

Homo    TCTACGTGTCCCGCGAGTGGCTCAACAAGTTCAACACCTTCGCGGAGCCAGGCCCCATCACCAACCAGACCTTCCTCTGC 160
Pan     TCTACGTGTCCCGCGAGTGGCTCAACAAGTTCAACACCTTCGCGGAGCCAGGCCCCATCACCAACCAGACCTTCCTCTGC 160
Macaca  TCTACGTGTCCCGCGAGTGGCTCAACAAGTTCAACACCTTCGCAGAGCCAGGACCCATCACCAACCAGACCTTCCTCTGC 160
Bos     TCTACGTGTCCCGGGAATGGCTCAACAAGTTTAACACCTTCGCGGAGCCAGGGCCCATCACCAACCACACGTTCCTCTGC 160
Canis   TCTACGTGTCTCGAGAATGGCTCAACAAGTTCAACACGTTTGCTGAGCCAGGGCCCATCACGAATCACACCTTCCTCTGC 160
```

ENSG00000136878 intron 17

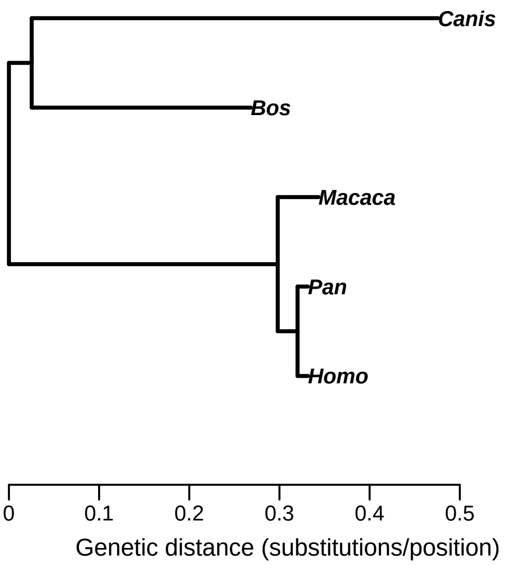

ENSG00000136878 intron 17

```
Homo   GTGGG--------C-----------GCTGGGCCAGGCCTGGTGGAGGAACCTCACCATCCCCGTCCCCTGGGAC--CCATGGGCCTTCTGGGTGTGTGTA 79
Pan    GTGGG--------C-----------GCCAGGCCGGGCCTGGTGGACGAACCTCACCATCCCCGTCCCCTGGGAC--CCGTGGGCCTTCTGGGTGTGTGTA 79
Macaca GTGGG--------C-----------GCCGGGCCGGGCCTGGTGGAGGAACCTCACCACCCCCGTCCCCTAGGAC--CCATGGGCCTTCTGGGTGTATGTA 79
Bos    GTGCGGTTCTGCCA-----------GCGCGCCGGGGCCCTGCACAGCAACCCGGGACCTAGCGTTCCGCGCCACCTCCACGGGACTTC---ACCCGGGTG 86
Canis  GTGGGACCCTGGGCAGGCCGTCGTGGTAGGGCTGGGTCTGGCTAAGGAAC----AGACCAGTGACCTGGGGGCC--------------TGGATGTGCGTG 82

Homo   GGGG---ACAGCGCCTGGA------TTAAGCCAGGGAAGTAAGG-ACTTGGATTGTTAAGAAACAGCC-----CTCTGGCTGCTGTGGGGAACCCAGGAA 164
Pan    GGGG---ACAGCGCCTGGA------TTAAGCCAGGGAAGTAAGG-ACTTGGATTGTTAAGAAACAGCC-----CTCTGGCTGCTGTGGGGAACCCAGGAA 164
Macaca GGGG---ACAGTGCCTGGAAGGGTTTTGAACCAGGGAAGTAAGG-ACTTGGA---TTAAGAAAGAGCT-----CTCTGGCTGCTGTGGGGAATGCAGGAA 167
Bos    CAGG---GGGGCGCCTGAGAGGGCTTTAGGCCAGGGAAGAGTGATGATTGGGGGTTTTAGAAAGAGCCTGCTGCTCTGGCTGCTGTTGGGAAAGCAGGAA 183
Canis  GAGGCAAGTGGTGCCTGGGAGGGTCCTAGGCCCGGGCAGTTAGG-GTTTCAGTTTGTAAGAAAGAATC-----TTCTG---GCTGAGGGGAAAGTGGGAA 173

Homo   GGCAGCTTG---------GAGCCTTCTCCAG-GGCCCTGGCTGGCGGCAC-------------------------------------------------- 204
Pan    GGCAGCTTG---------GAGCCTTCTCCAG-GGCCCTGGCCGGCGGCAC-------------------------------------------------- 204
Macaca GGCAGCTTG---------GAGCCTTCTCCAG-GGCCCTGGCTGGTGCACA-------------------------------------------------- 207
Bos    ACCCACGTAGTTGAG-TTGGGTTTTCTGCAGTGGTCCTGGTGGGAGGTGATGACGCTGGAATTCTGGCAGGGGTGGTGGGGCCTATGAGATGTGGAGGAT 282
Canis  ACCCAGGAAGTTGAGTTTGGGCTTTCTGTGGGGGTCCT-TTGGAAGGTGATATGGCCAGAACTCTGGCAGGGGTG------------------------- 247

Homo   ---------------------------------------------------------------------------------------------------- 204
Pan    ---------------------------------------------------------------------------------------------------- 204
Macaca ---------------------------------------------------------------------------------------------------- 207
Bos    GCTGAGATGGGAGGATGCTGAGATATGGAGGGGTGCTAAAATGCGAGGGTGCTGAGATGAGGGCGCTGAGATGAGGAAGGTGCTGAGATGAGGGGTGCTG 382
Canis  ---------------------------------------------------------------------------------------------------- 247

Homo   -----AGGGGGCTGCTCACATGTCCAGGACCC-GTGAAGGACCCAGGAG--------------------------------------------------- 247
Pan    -----AGGGGGCTGCTCACATGTCCAGGACCC-CTGAGGGACCCAGGAG--------------------------------------------------- 247
Macaca -----GGGGCGCTGCTCACATGTCCAGGACCC-CTGAGGAACCCAGGA---------------------------------------------------- 249
Bos    AGATGAGGAGGGTGCTGAGATGCAGAAGGGTC-CTGAGATACGGAGGGGTGCTGAGACGTGGAGGGTGCTGAGGTCGGGAGGGGTGCTGAGATGAGGAGG 481
Canis  -----GCAAGGCTGCTGGGATGCTGCTGGGTCGCTGAGGGGTTGGGGAGGGCAGTGAT------------------------------------AGGTGG 306

Homo   ------------------------------------GTGGTGGACAGGGCCGGTAGCCCGAGGTGCACTGGGATGGGGGCTCTGCA-------------- 297
Pan    ------------------------------------GTGGTGGACAGGGCCGGTAGCCCGAGGTGCACTGGGATGGGGGCTCTGCA-------------- 297
Macaca --------------------------------------GGTGGACAGGGCCGGTAGCCCCAGGTGCACTGGGATGAGGGCTCTGCA-------------- 297
Bos    GTGCTGAGATGAAGGGTGCTGAGATGGGAGGATGCTGAGATATGGAGGGGTGCTGAGATGAGGGGTGCTGAGATGAGGGTGCTGAAATGAGGGTGCTGAG 581
Canis  CTCCAGACACGCCCCTGGCAGGTGTGGCTGGTGCCCCAGCATGGAGAGAGTCTGAACACAGCAGCTCCTGATTTTCCTGCCCCACCCCTGTCCCACCCCT 406

Homo   --------TGTGTCTGAAGGTGGCAG--------------------------------CCGTCAGGCACAGCTT----------CTCGCCCCCTCCTCA- 346
Pan    --------TGTGTCTGAAGGTGGCAG--------------------------------CCGTCAGGCACAGCTT----------CTCGCCCCCTCCTCA- 346
Macaca --------TGTGTCTGGAGGTGGCAG--------------------------------CCGTCAGGCACAGCTT----------CTCGCCCCCTCCTCA- 346
Bos    ATGAGGAGGGTGCTGAGATGTGGAGGGGTGCTGAGGGGCCTGAGACGTGGGGCTTCCTGCACGGGGCGTGGCTCCCACCCCTGACCCGCCCCCGTGCCG- 680
Canis  GTCCCATCCTTGCCCCATCTCTGCTC-----------------------TACCCTGCCGTACCCCTTGCTCTGCCTACCTGTGCCCCACCCCTGTCCCAT 483

Homo   -CCCCA---CAG 354
Pan    -CCCCA---CAG 354
Macaca -CCCCG---CAG 354
Bos    -CCCCGCCCCAG 691
Canis  CCCCTGCTGCAG 495
```

ENSG00000011021 intron 17

Description: Chloride channel protein 6 (CLCN6)
Intron number: 17
Human chromosome: 01
Intron start (bp): 11817235
Human intron length : 1376
Intron alignment length: 1409
Flanking exons length (upstream/downstream): 107/187
SNP density: 0.003634
K tree score: 0.0309
Scaling factor: 0.6753
Human-chimpanzee distance: 0.009606
Total primate branch length: 0.091

ENSG00000011021 exon 17

```
Homo    GTGGCCAAATGGACAGGGGACTTTTTCAATAAGGGCATTTATGATATCCACGTGGGCCTGCGAGGCGTGCCGCTTCTGGA 80
Pan     GTGGCCAAATGGACAGGGGACTTTTTCAATAAGGGCATTTATGATATCCACGTGGGCCTGCGAGGTGTGCCGCTTCTGGA 80
Macaca  GTGGCCAAATGGACAGGGGACTTTTTCAATAAGGGCATTTATGATATCCACGTGGGCCTGCGAGGCGTGCCACTTTTGGA 80
Bos     GTGGCCAAATGGACAGGGGACTTTTTCAATAAAGGCATTTATGACATCCACGTGGGCCTGCGCGGCGTGCCGCTGCTAGA 80
Canis   GTGGCCAAATGGACAGGGGACTTTTTCAACAAAGGCATTTATGATATCCACGTGGGCTTGCGGGGCGTGCCGCTCCTGGA 80

Homo    ATGGGAGACAGAGGTGGAAATGGACAA 107
Pan     ATGGGAGACAGAGGTGGAAATGGACAA 107
Macaca  ATGGGAGACAGAGGTGGAAATGGACAA 107
Bos     ATGGGAGACAGAGGTGGAAATGGACAA 107
Canis   ATGGGAGACAGAGGCGGAAATGGACAA 107
```

ENSG00000011021 exon 18

```
Homo    GCTGAGAGCCAGCGACATCATGGAGCCCAACCTGACCTACGTCTACCCGCACACCCGCATCCAGTCTCTGGTGAGCATCC 80
Pan     GCTGAGAGCCAGCGACATCATGGAGCCCAACCTGACCTACGTCTACCCGCACACCCGCATCCAGTCTCTGGTGAGCATCC 80
Macaca  GCTGAGAGCCAGCGACATCATGGAGCCCAACCTGACCTACGTCTACCCGCACACCCGCATCCAGTCTCTGGTGAGCATCC 80
Bos     ACTGCGAGCCAGCGACATCATGGAGCCCAATCTGACCTACGTCTACCCGCACACCCGCATCCAGTCCCTGGTCAGCATCC 80
Canis   ACTGAGAGCCAGTGACATCATGGAGCCCAACCTCACCTACGTCTACCCACACACCCGCATCCAGTCTCTAGTGAGCATCC 80

Homo    TGCGCACCACGGTCCACCATGCCTTCCCGGTGGTCACAGAGAACCGCGGTAACGAGAAGGAGTTCATGAAGGGCAACCAG 160
Pan     TGCGCACCACGGTCCACCACGCCTTCCCGGTGGTCACAGAGAACCGCGGTAACGAGAAGGAGTTCATGAAGGGCAACCAG 160
Macaca  TGCGCACCACAGTCCACCACGCCTTCCCGGTGGTCACAGAGAACCGCGGCAACGAGAAGGAGTTCATGAAGGGCAACCAG 160
Bos     TACGCACCACCGTCCACCACGCCTTCCCGGTGGTCACAGAGAACCGGGGCAACGAGAGGGAGTTCATGAAGGGCAACCAG 160
Canis   TGCGCACCACGGTCCACCACGCCTTCCCGGTGGTCACGGAGAACCGGGGCAACGAGAAGGAGTTCATGAAGGGCAACCAG 160
```

ENSG00000011021 intron 17

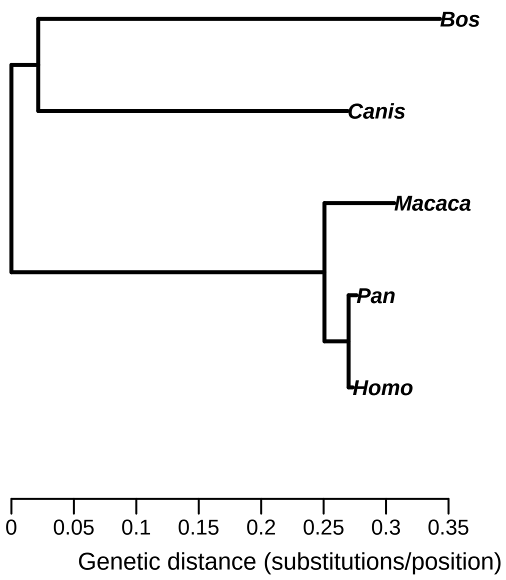

ENSG00000011021 intron 17

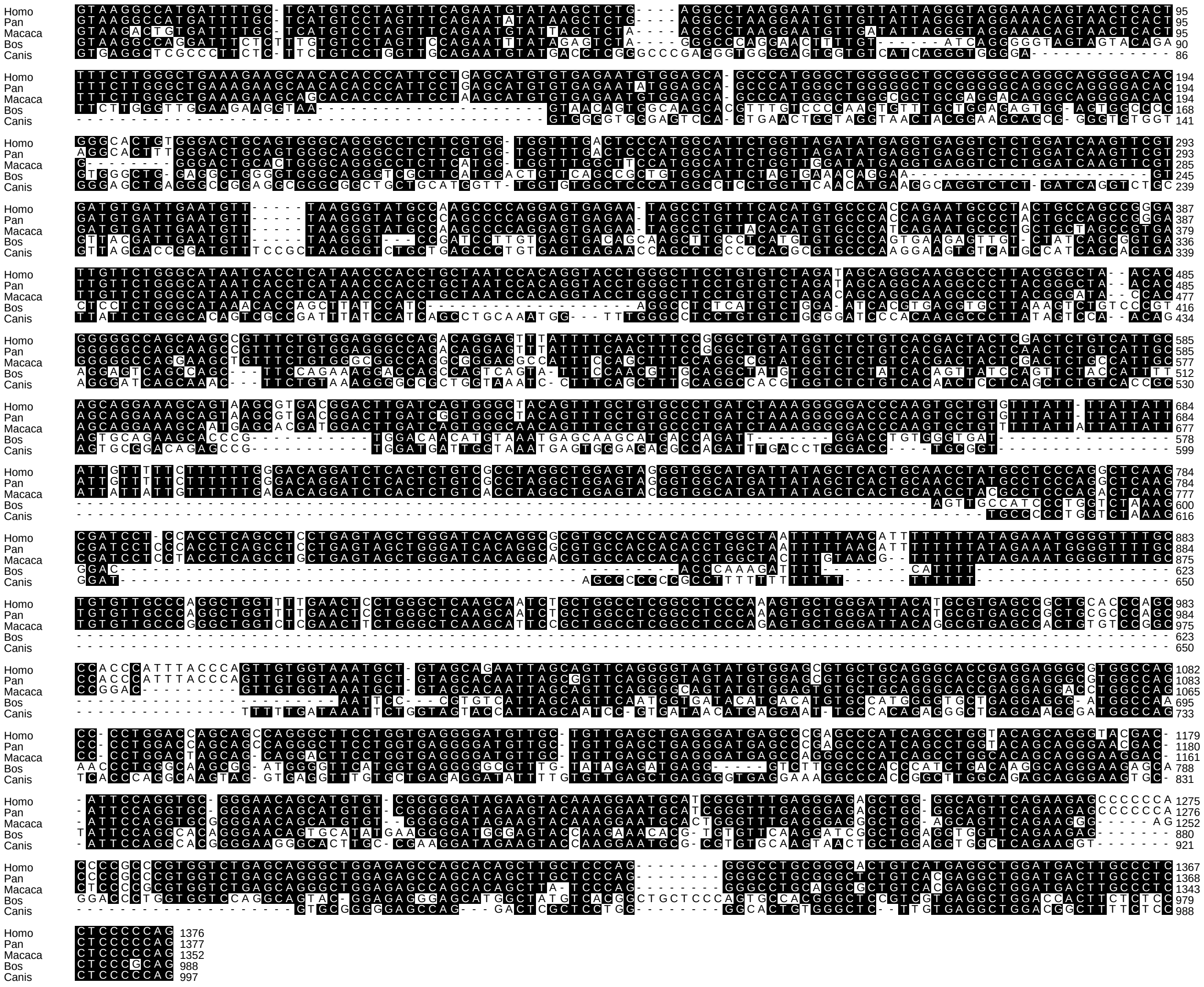

ENSG00000016490 intron 11

Description: chloride channel, calcium activated, family member 1 precursor (CLCA1)
Intron number: 11
Human chromosome: 01
Intron start (bp): 86732720
Human intron length : 1056
Intron alignment length: 1137
Flanking exons length (upstream/downstream): 262/171
SNP density: 0.006629
K tree score: 0.0578
Scaling factor: 0.9086
Human-chimpanzee distance: 0.014457
Total primate branch length: 0.0907

ENSG00000016490 exon 11

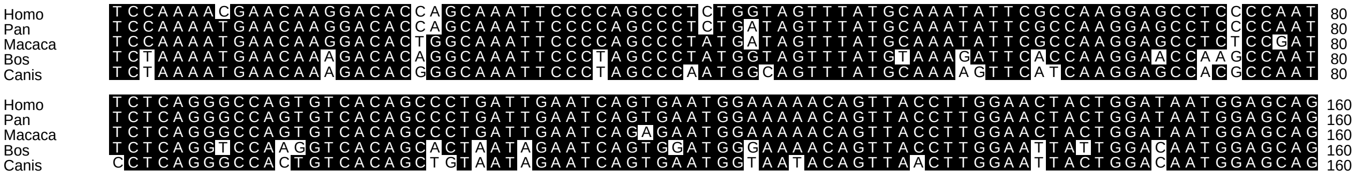

ENSG00000016490 exon 12

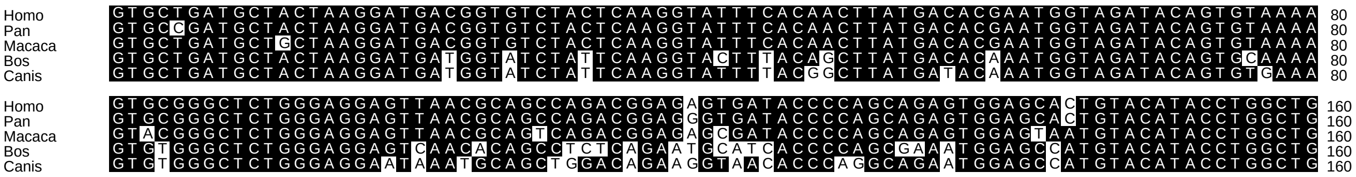

ENSG00000016490 intron 11

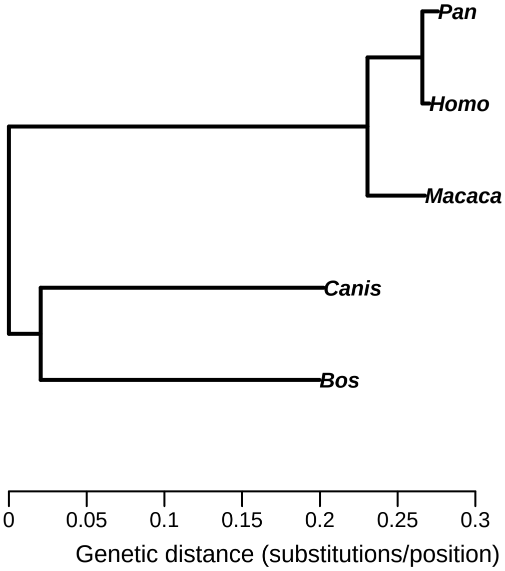

ENSG00000016490 intron 11

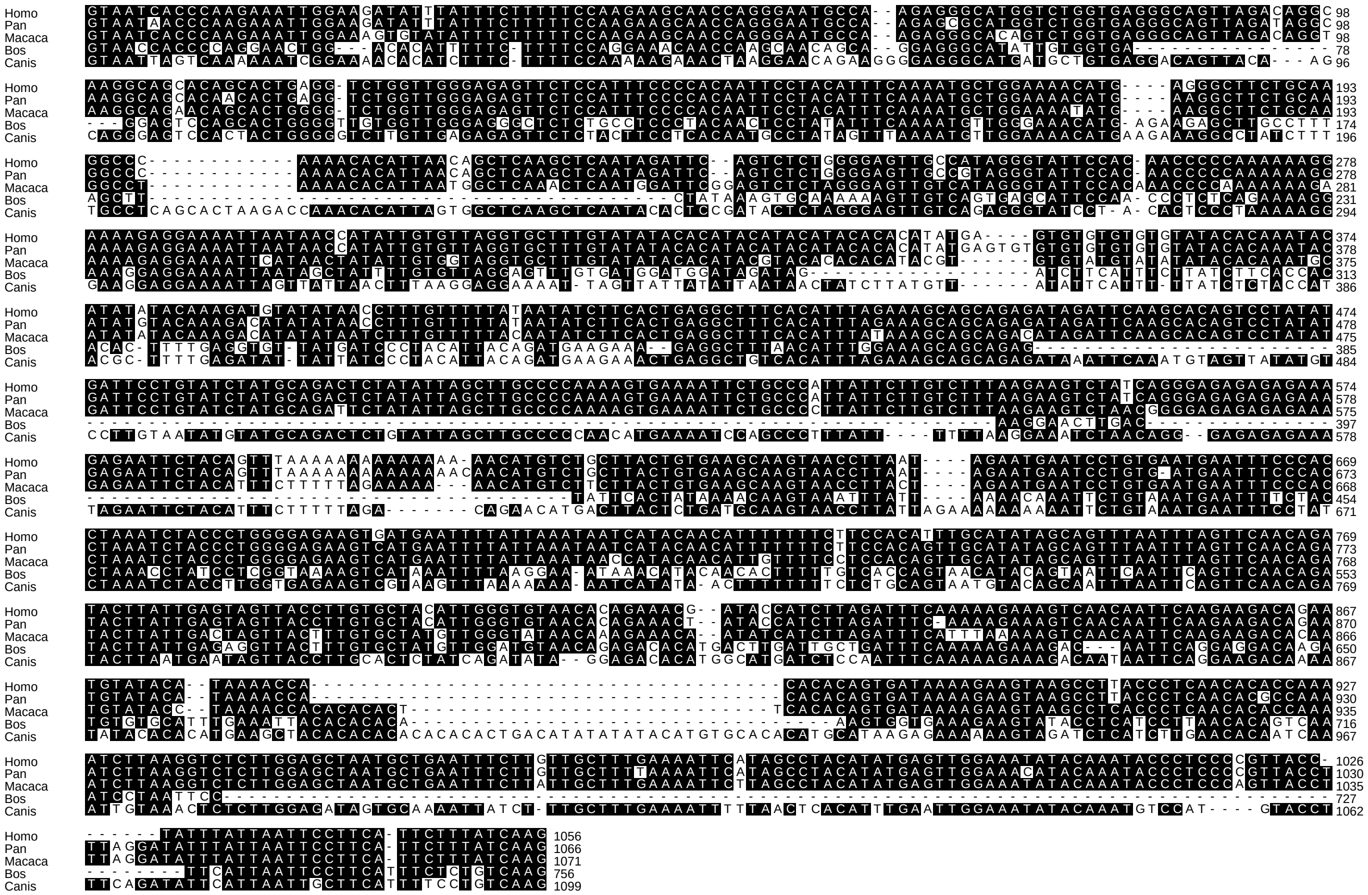

ENSG00000158941 intron 2

Description: p30 DBC protein (KIAA1967)
Intron number: 2
Human chromosome: 08
Intron start (bp): 22519635
Human intron length : 430
Intron alignment length: 448
Flanking exons length (upstream/downstream): 92/92
SNP density: 0.004651
K tree score: 0.0838
Scaling factor: 0.8193
Human-chimpanzee distance: 0.014056
Total primate branch length: 0.0907

ENSG00000158941 exon 2

```
Homo    GCACAGCTTCAACATCTCTTCTGGGCCCTCCTCCTGGTTTGCTCACTCCTCCTGTGGCCACAGAACTGTCCCAGAATGCC 80
Pan     GCACAGCTTCAACATCTCTTCTGGGCCCTCCTCCTGGTTTGCTCACTCCTCCTGTGGCCACAGAACTGTCCCAGAATGCC 80
Macaca  GCACAGCTTCAACATCTCTTCTGGGCCCTCCTCCTGGTTTGCTCACTCCTCCTGTGGCCACAGAACTGTCCCAGAATGCC 80
Bos     GCGCAGCTTCGACATCCCTTCTGGGCCCTCCTCCTGGTTTGCTCACTCCTCCTGTGGCCACAGACCTGTCCCAAAATGCC 80
Canis   GCGCAGCTTCAACATCTCTTCTGGGTCCTCCTCCTGGTTTGCTCACTCCTCCTGTGGCCACAGACCTGTCCCAAAATGCC 80

Homo    AGGCACCTTCAG 92
Pan     AGGCACCTTCAG 92
Macaca  AGGCACCTTCAG 92
Bos     AGGCACCTTCAG 92
Canis   AGGCACCTTCAG 92
```

ENSG00000158941 exon 3

```
Homo    GGTGGGGAGAAACAGCGGGTCTTCACTGGTATTGTTACCAGCTTGCATGACTACTTTGGGGTTGTGGATGAAGAGGTCTT 80
Pan     GGTGGGGAGAAACAGCGGGTCTTCACTGGTATTGTTACCAGCTTGCATGACTACTTTGGGGTTGTGGATGAAGAGGTCTT 80
Macaca  GGTGGGGAGAAACAGCGGGTCTTCACTGGTATTGTTACCAGCTTGCATGACTACTTTGGGGTTGTGGATGAAGAGGTCTT 80
Bos     GGTGGAGAGAAACAGCGGGTCTTCACTGGCATTGTTACCAGCTTGCACGACTACTTTGGGGTGGTGGATGAAGAAGTCTT 80
Canis   GGTGGGGAGAAGCAGCGGGTCTTCACTGGCATTGTTACCAGCTTGCATGACTACTTTGGGGTGGTGGATGAAGAGGTCTT 80

Homo    TTTTCAGCTAAG 92
Pan     TTTTCAGCTAAG 92
Macaca  TTTTCAGCTAAG 92
Bos     TTTTCAGCTAAG 92
Canis   TTTTCAGCTCAG 92
```

ENSG00000158941 intron 2

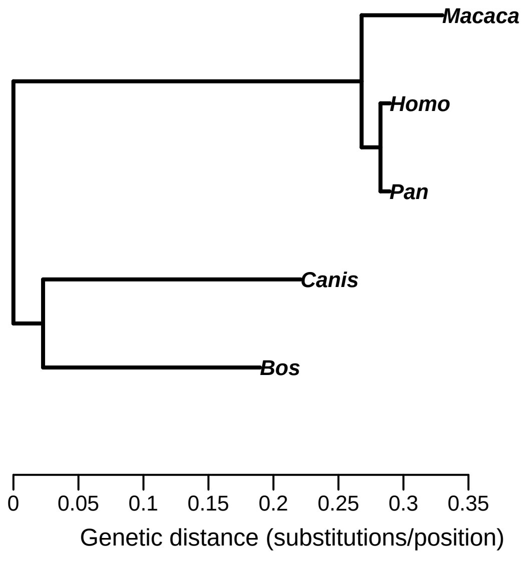

ENSG00000158941 intron 2

```
Homo    GTAGGTTCGGCATCCCTTGTAGTAAGTTTCAGCCCTTGGGA------CTGAATGCATGGAGGCCTGGTGCTCTTTTTCTCTTACATAATAGTGTTTATCA 94
Pan     GTAGGTTGGGCATCCCTTGTAGTAAGTTTCAGCCCTTGGGA------CTGAATGCATGGAGGCCTGGTGCTCTTCTTCTCTTACGTAATAGTGTTTATCA 94
Macaca  GTAGGTTGGGCATCCCTTGT----AGTTTCTGCCCTTGGGA------CTGGATGCATGGAGGCCTGGTGCTCTTCATCTCTTACATAATAGTGTTTATCA 90
Bos     GTAAGTTGGGCATTCCGTCTAGTGAGTTTCAGCACTTGGGAGGACCTCAGCATGCATGGTTGTCCGATGCTTTCAT--TCTTACATGACATTGTTCATCC 98
Canis   GTAGGTTGTGCATTTTTTCTAGTGAGCTTCAGCACTTGGGACTAGTGCAGCATGCATGGGGCCTGGTGCTTTCAT--TCTTGCATTGCCATGTTCATCC 98

Homo    TTCCTGATCCCTCTCCTGGTAGTGGGCTAGTATGGGGTTGCCCAGATAGCCTTCAGACTGGGGCATCTTCATTTCTCGCTCCAGGGAAATCTTTAGCTTA 194
Pan     TTCCTGATCCCTCTCCTGGTAGTGGGCTAGTATGGGGTTGCCCAGATAGCCTTCAGACTGGGGCATCTTCATTTCTCGCTCCAGGGAAATCTTTAGCTTA 194
Macaca  TTTCTGACCCCTCTCCTGGTAGTAGGCTAATACGGAGTTTCCCAGATAGCCTTCAGACTGGGGCATCTTCATTTCTCGCTCCAGGGAAATCTTTAGCATA 190
Bos     TTCTTGACACTCCT----ATCGTCAACCAGTCCAGAGGTAACTAG--AACTCTCTGACTGGGGAGTCTTTGGTTTTGATTCTAGGGAAATCTCTAGGGTG 192
Canis   TTCCTGGAGCTCCT----GGCCTGGGCCGGGATGGAGGTAACTAG--ACACCTCTGACC-GGGAGTCCTAAGTTCTGGCT-----GAAATCTCTTGGGTG 186

Homo    CGTCTTGGAATTA---ATGAACTGGAAATCCCAGAAGAAGCTTGTGGGTCAAGAACCAGCTCAAACATGCCCCAACCCCTAATGATGGAGTATGCCCAC- 290
Pan     CGTCTTGGAATTA---ATGAAGTGGAAATCCTAGAAGAAGCTTGTGGGTCAAGAACCAGCTCAAACATGCCCCAACCCCTAATGATGGAGTATGCCCAC- 290
Macaca  CGTCTTGGAATTA---ATGAACTGGAAGTCCTAGAAGAAGGTTGTGGGTCAAGAACCAGTTCAAACATGCCCCGAGCCTTAATGATGGAGTATGCCCACC 287
Bos     CTTTTTGGAGTTAAGGAAGAACTGGGAATCCTAGAAGAAGCTTATGGCCCAAGAATCAGCAT-GACTTCTCCCACCTT----CGGTAGATTATGTCCACT 287
Canis   CTTCTTGGAATTA---AAGAACTGGAAATCCCAGAAGAAGCCTGTGAGCTAAGGTTCAGTGT-GATCTCTTCCTCCTCCCGGCAGTAGATTATGACTGTT 282

Homo    -CACACCTGTACTTCACTCTGTGCGAACTCTTGATGCAGTACGCTG-------AGCTGGGGCGTGGGTTGAGATGAGACCTTCATGGCAGAGTGTGATTT 382
Pan     -CACACCTGTACTTCATTCTGTGCGAACTCTTGATGCAGTACGCTG-------AGCTGGGGCGTGGGTTGAGATGAGACCTTCATGGCAGAGTGTGATTT 382
Macaca  ACACACTTGTACTTCACTCTGTGTGAACTCTTGATGCAGTACGCTG-------AGCAGGCGTGTGGGTTGAGATGAGACTTTCATGGCAGAGTGTGATTT 380
Bos     AGACACATGTGCTTCCTTCTACAGGAAG-CTCCGTGCAGTACTTTGATCTGGGGCTGGAGGGGTGGAGAAAGATGAGATGCTAATGGCAGAGCATGGATA 386
Canis   CTTTCTGTGTGCTTCCTTC-ATGTGACC-CTTGATGCCATCCTCTGTTGAGCTAGATGGGGTTTGGCTGGAGATGAGATGCTAATGGCAGAGCGTAGACA 380

Homo    TGTCCAGTTTCCTCCCTTTTACTTTTCAGCTGTCTCCTTCTCTTACAG 430
Pan     TGTCCAGTTTCCTCCCTTTTACTTTTCAGCTGTCTCCTTCTCTTACAG 430
Macaca  TGTCCAGCTTCCTC-----TATTTTTCAACTGCTCCCTTTTCTTACAG 423
Bos     GATGCTTCCTCT-------TACCTTTAAACTTCACTCTTCTCATATAG 427
Canis   AATG----CTGT-------TACTTTCAAACTGCACTATTCTCTTTCAG 417
```

ENSG00000115365 intron 4

Description: LanC-like protein 1 (LANCL1)
Intron number: 4
Human chromosome: 02
Intron start (bp): 211013714
Human intron length : 563
Intron alignment length: 584
Flanking exons length (upstream/downstream): 136/147
SNP density: 0.003552
K tree score: 0.098
Scaling factor: 0.9439
Human-chimpanzee distance: 0.019923
Total primate branch length: 0.0906

ENSG00000115365 exon 4

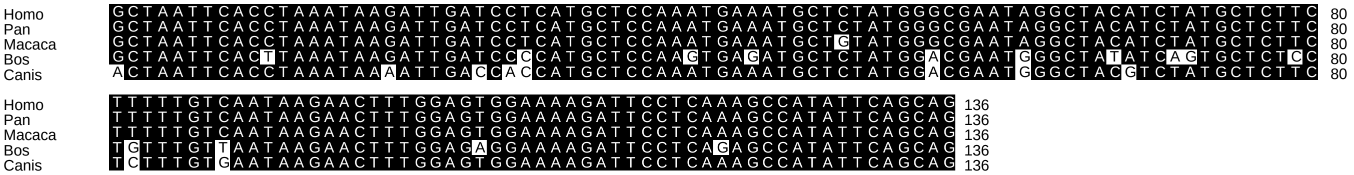

ENSG00000115365 exon 5

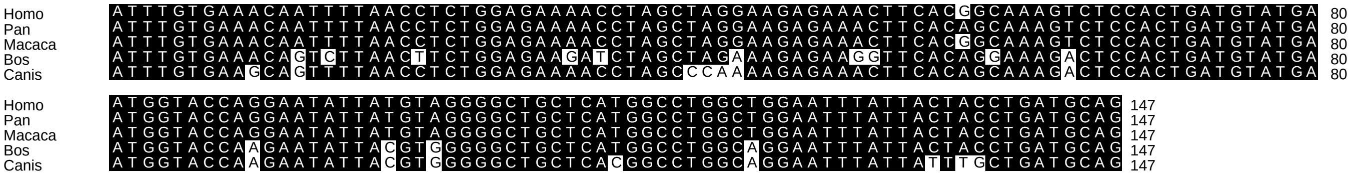

ENSG00000115365 intron 4

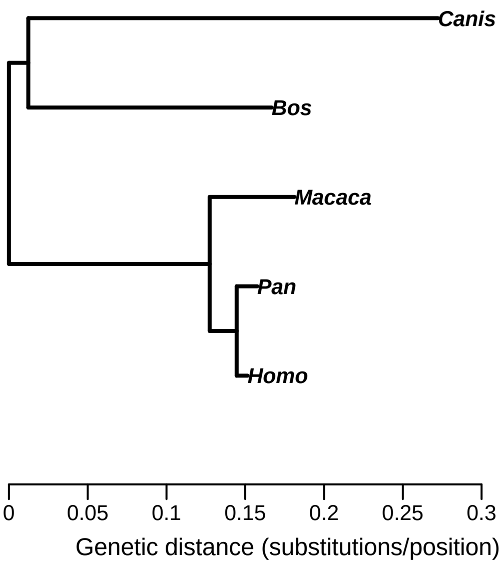

ENSG00000115365 intron 4

```
Homo    GTACCACGTTTTTGTGTTTTTAGGAGCCTTTTTAGGATCCCATTTACTTACTATCTGCCTAAACTATTGTTTATGTCTCTGTAGTTAGCACATTCCATAT 100
Pan     GTACCACGTTTTTGTGTTTTTAGGAGTCTTTTTAGGATCCCATTTACTTACTATCTGCCTAAACTATTGTTTGTGTCTCTGTAGTTAGCACGTTCCATAT 100
Macaca  GTACCACGTTTTTGTGTTTTTAGGAGTCTCTTTAGGATCCCATTTACTTACTGTCTGCCTAAACTATTGTTTGTGTCTCTCCAGTTAGCACATTCCAAAT 100
Bos     GTACCACG-TTTTGTGCTTTCTGGCGTCTCTTTACGACCCC----ACTTCCTATCTGCCTAAACTGTTGGT------TTCCCTGTTGGCACATCCCAGGT 89
Canis   GTACCACATTTTTGTGCTTTTTGGAGTCTTGTTAGGATCCC----AGTTGTTATCTATGTGAACT-TTGGTTTTGTTTCTCCAGTGAGCATACCCCAGGT 95

Homo    AA------AGGAAGGTGTATCTGGACCTCTAAAGGTTGGGCACAGTCCAAGAACCAATAGCTTTCTACGGAGTGCTGTTGGAAAC----ATTTCTGTCTC 190
Pan     AA------AGGAAGGTGTATCTGGACCTCTAAAGGTTGGGCACAGTCCAAG-ACCAATAGCTTTCTACGGAGTGCTGTTGGAAAC----ATTTCTGTCTC 189
Macaca  AA------AGGAAGGTGTATCTGGACCTCTAAAGGTTGGGCACAGTCCAAGAACCAATAGCTTCCTACGGAGTGCTGTTGGAAAC----ATTTCTGACTC 190
Bos     AGCTGGCCAGGAAGGTGTATCTTGACCTCTGAAGGTCAGGCATGATCCCAGCACCACCAGCTTCACATGGGGTGCTGTGGGACACTGAAGGGAGTGACTC 189
Canis   AG-----AAGGAAAGTGTCAGTTGGCTTCTGAAGGTCAGGTGGGATCAGAGAGCGGACAGTGTCACGCTGAGTGCTGTTGGAAACACTTAGGGAGTTCTA 190

Homo    AAGAGTCCGAAGGCCAGTCTGCTCCATTTCAGAATTTAGAGGCCCTCTCCAAAGAGGATTAGAGCCAAATTTCACCTCCCAGATGGAGGATCTCCTTTGG 290
Pan     AAGAGTCAGAAGGCCAGTCTGCTCCATTTCAGAATTTAGAGGCCCTCTCCAAAGGGGATTAGAGCCAAATTTCACCTCCCAGATGGAGGATCTCCTTTGG 289
Macaca  ---AGTCAGGCGGCCAGTCTGCTCCATTTCAGAATTTAGAGGTCCTCTCCAAAGAAGATTAGAGCCAAATTTCACCTCCCAAATGGAGGGTCTGCTTTGG 287
Bos     -AGAGTAAGAAGTCCAG-CTACTCCATTTGAGAGTTCAGGGGCCAT-TCAGAGGAAGATAAGGGCCAGA------------------------------- 255
Canis   -AGAGTGGGAAGTCCAGCCCACTCTGTT--GGAGTGTCGAGGCCCTTC----AGAAGATGAGGGCCAGATTTCACCTCCCAGGTGCAGGCGTCTCCTT-- 281

Homo    TGGTGCCTAGAGGAGTTTATGCTTTTCTTTGACAGGCGAACTCTTGTAAACTGTAATGCAAAGAGCTCAAGATCCTCTACGCCCTCCCCCTTCTAGGCTA 390
Pan     TGGTTCCTAGAGGAGTTTGTGCTTTTCTTTGACAGGCGAACTCTTGTAAACTGTAATGCAAAGAGCTCAAGATCCTCTATGCCCTCCCCCTTCTAGGCTA 389
Macaca  TGGTGCCTAGAGGATTTTATGCT----TTTGACAGGTGAACTCTTGTAAACTGTAATGCAAAGAGCTCACGCTCCTCTATGCCCTCCCCGTTCTAGGCTG 383
Bos     ---------------------------------------------------------------------------------------------------- 255
Canis   TCGTTCCTGAAGGAATTCAAGCT-TTCCTGGTCAAGTGAATTCTTGTAAGCTCCAGTAAAGAGAGTTAAGGATTGCC--ATCTCTCCCTCTTTTAGGCAG 378

Homo    AATACAGTCCCACACACTTCTCTGA-TGGAGAATTAAACTGTGCCTTTCCCTGGCTATAAGCTGGTGTCTAATTTGTCATGTTCCCCA-GTACCTCTTAG 488
Pan     AATACAGTCCCACACACTTCTCTGA-TGGAGAATTAAACTGTGCCTTTCCCTGGCTATAAGCTGGTGTCTAATTTGTCATGTTACCCA-GTACCTCTTAG 487
Macaca  AATATAGTCCCAC-----TCTCTGA-TGAAGAATTAAACTGTACCTTTCCCTGGCTGAAAGCTAGTGTCTAATTTGTCGTGTTCCTCA-GTACCCCTTAG 476
Bos     ---ATAATCCCATGTGCTTCTCTGAGTACAGAGTCAAACTGTGCCCATTCATGGCCATAAGGTGATGTCTAACTTGTCATGTTCCCCA-GTACCTGTTAG 351
Canis   AATGCAGTCCCCCCTGC-TTTCTGAGCACAGAACTAAACTG--CCTGCCCATGGCCATAAGAGTGGGTCTGATTCACCATGTTCTCCATACCTCTCTTAG 475

Homo    CAAAAAAGTCTTTACCTCCTCCAGGAAGATTT---------TTGTTCAGTTAACTTGTTATTTTGGTTGGTTTTCTCCCTGTAG 563
Pan     CAAAAAAGTCTTTACCTCCTCCAGGATGATTT---------TTGTTCAGTTAACTTGTTATTTTTGTTGGTTTTCTCCCTGTAG 562
Macaca  CAAAAAAGTCATTACCTCCTCCAGGAAGGTTT--------ATTGTTCAGTTCACTTGTGATTTTGGTTGGTTCTCTCTCTGCAG 552
Bos     CAAAAATCCCTTGTCTCCAT------------------------TCTAATTCTTTCCTTATTCTGGTTGGGTTTCTCTCTGTAG 411
Canis   CGAAAAGTTCCTTATCTCCGCCGGGAAGATTTTTATTCTGATTCTCTGATTCTCTCATTATTTCAATCCGTTTTCTCCCTGTAG 559
```

ENSG00000111445 intron 2

Description: Replication factor C subunit 5 (RFC5)
Intron number: 2
Human chromosome: 12
Intron start (bp): 116941325
Human intron length : 495
Intron alignment length: 498
Flanking exons length (upstream/downstream): 65/137
SNP density: 0.002020
K tree score: 0.0521
Scaling factor: 0.9649
Human-chimpanzee distance: 0.016880
Total primate branch length: 0.0904

ENSG00000111445 exon 2

```
Homo    GGTTGAAAAATACCGGCCACAGACCCTGAATGATCTCATTTCTCATCAGGACATTCTGAGTACCA 65
Pan     GGTTGAAAAATACCGGCCACAGACCCTGAATGATCTCATTTCTCATCAGGACATTCTGAGTACCA 65
Macaca  GGTTGAAAAATACCGGCCACAGACCCTGAATGATCTCATTTCTCATCAGGACATTCTGAGTACCA 65
Bos     GGTTGAAAAATATCGGCCACAGACACTGGATGATCTCATTTCTCATCAGGACATCTTGAGTACTA 65
Canis   GGTTGAAAAATACCGGCCACAGACGCTGAATGATCTGATTTCTCATCAGGACATTTTGAGTACCA 65
```

ENSG00000111445 exon 3

```
Homo    TTCAGAAGTTTATCAATGAAGACCGACTGCCACACTTGCTTCTCTACGGTCCCCCAGGGACAGGCAAGACATCTACCATC 80
Pan     TTCAGAAGTTTATCAATGAAGACCGACTGCCACACTTGCTTCTTTACGGTCCCCCAGGGACAGGCAAGACATCTACCATC 80
Macaca  TTCAGAAGTTTATCAGTGAAGACCGACTGCCACACTTGCTTCTCTATGGTCCCCCAGGGACAGGCAAGACATCTACCATC 80
Bos     TTCAGAAGTTTATCAGTGAGGACCGCCTGCCGCACCTCCTTCTGTATGGTCCTCCAGGGACAGGAAAGACATCCACCATC 80
Canis   TTCAAAAATTTATCAGTGAAGACCGTTTGCCCCACCTGCTCCTCTATGGTCCTCCAGGGACAGGAAAGACTTCCACCATC 80

Homo    CTAGCCTGTGCGAAACAGCTATATAAAGACAAAGAATTTGGCTCCATGGTCTTGGAG 137
Pan     CTAGCCTGTGCGAAACAGCTATATAAAGACAAAGAATTTGGCTCCATGGTCTTGGAG 137
Macaca  CTAGCCTGTGCGAAACAGCTGTATAAAGACAAAGAATTTGGCTCCATGGTCTTGGAG 137
Bos     CTGGCCTGTGCTAAACAGCTGTACAAAGATAAGGAATTTGGCTCCATGGTCTTGGAG 137
Canis   CTGGCCTGTGCAAAACAGCTATACAAAGATAAGGAATTTGGCTCCATGGTCTTGGAG 137
```

ENSG00000111445 intron 2

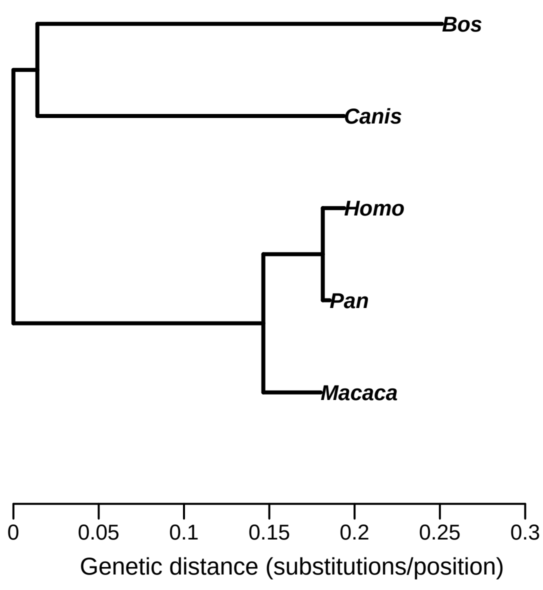

ENSG00000111445 intron 2

```
Homo    GTAAGTATTCATGTGTCAGTTGCTCTTGTCCTGCTTGAGCGACTTGTATAAATTGCTTGGTGATGTTGTCTCTCCTA-AGTAATAACTTCAAAGAATCTG 99
Pan     GTAAGTATCCATGTGTCAGTTGCTCTTGTCCTGGTTGAGGGACTTGTATAAATTTCTTGGCGATGTTGTCTCTCCTA-AGTAATAACTTCAAAGAATCTG 99
Macaca  GTAAGTATCCATGTGTCAGTTGCTCTTGTCCTGGTTGAGGGACCTGTGTGAATTTCTTGGTGA---GGTCTCTCCTA-AGTAATAACTTCAAAGAATCTG 96
Bos     GTAAGTATCCATGTGTCAGTGGCTGGTATCCTAGTTGAAGGACCTGGATATATTTCTTAGGGA---TGTTGTCTCTGCTGTAATAACTTTAAAGAATCTG 97
Canis   GTAAGCATCCATGTGTCAGTTGCTGTCATTCTAGTTGGAAGACTTGTATAAATTTCTTGGTTA---TGTCTCTGCTG-AGTAATAACCTCGAAGTATCTG 96

Homo    ATTGCGCTTTGTAGAATGTGGCTTTAAGATCATTCATTCATTTTCTTCAGGTTGCTACAGTCCAGTGGGTAAGTCACAAACATGAGCATAAAATTGCAAC 199
Pan     ATTGCGCTTTGTAGAATGTGGCTTTAAGATCATTCATTCATTTTCTTCAGGTCGCTACAGTCCAGTGGGTAAGTCACAAACATGAGCATAAAATTGCAAC 199
Macaca  ATTGAGCTTTGTAGAATGTGGCTTTAAGATCATTCATTCATCTTCTTCAGATAGCTGCAGTCTAGTGGGTAAGTCACGAACATGAGCATAAAATTGCAAC 196
Bos     ATTAAGCTCTTTGGGATGTGGCTTTGACAGC-------TGCATCCTTCAGAT------------------------------------------------ 142
Canis   ACTGAGCTGTATGGAATGTTGCTCTGTGAGC-------TAGGTCCTTCGGAT------------------------------------------------ 141

Homo    ACAGTGGAGTAAAAATAGCATTACGGAGAATCATTTAAGTTCACAGATGTTGCCAGCACCCAGAAAGGTGCCTGTAACACAGTAGGCACTCAATAAATAC 299
Pan     ACAGTGGAGTAAAAATAGCATTACGGAGAATCATTTAAGTTCACAGATGTTGCCAGCACCCCGAAAGGTGCCTGTAACACAGTAGGCACTCAATAAATAC 299
Macaca  TCAGTGGAGTAAAAATAGCATTACAGCGAATCATTTAAGTTTACAGATGTTGTCAGCACCCAGAAAGGTGCCTGTAACACAGTAGGCCCTCAATAAATAC 296
Bos     ---------------------------------TTTTCATTC---------------------------------------------------------- 151
Canis   ---------------------------------TTTTCTTTC---------------------------------------------------------- 150

Homo    TTGTTGACTGGCTGCGCTATTGAGTGAATTCCAGTTGTTCCACGAAAGTAGATATGAGGCCCCTACATCAAGTTGTATCTGCCTCTGATTTGGACATTGA 399
Pan     TTGTTGACTGGCTGCGCTATTGAGTGAATTCCAGTTGTTCCACGAAAGCAGATATGAGGCCCCTACATCAAGTTGTATCTGCCTCTGATTTGGACATTGA 399
Macaca  TTGCTGGCTGGCTGCACTATTGAGTGAATTCCAGTTGCCCCAGGAACGCAGAGACAAGGCCCCTACATCAAGTTGCATCTGCCTCTGATTTGGACATTGC 396
Bos     ------------------------------------------------------------------------CTGCCTGAGCCCTGGATTTGAAC----- 174
Canis   ------------------------------------------------------------------------CTGCATCTGTCTCTGATTTGAAC----- 173

Homo    ATTGGGTTGAAGAGCTTTCAGCCCAACTCA-GGAGCCCAGGGTGAATGAGGCAGCTCCCAAAGACTGATGGTGCCCTTCTTCCT-TCCTGATCCTCAG 495
Pan     ATTGGGTTGAAGAGCTTTCAGCCCAACTCA-GGAGCCCAGGGTGAATGAGGCAGCTCCCAAAGACTGATGGTGCCCTTCTTCCT-TCCTGATCCTCAG 495
Macaca  ATTGGGTTGAAGAGCTTTCAGCCCAACTCA-GGGGCCCAGGGTGAATGAGGCGGCTCCCAAAGACTGATGGTGCCCTTCTTCCT-TCCTGATCCTCAG 492
Bos     ATAGAGTTGAAGTAGATTCAGTCCAACTCT---GGCCCGCAGTGAATGAGGCGACTC-T-AAGACTGATAGCATCTCTCTTTTTCCCCCCATCTTCAG 267
Canis   ATGGGATTGAAGTGCATTCAGGCCAACCCCAAGGGCCCAAGGTAAATCAGGTAACTCTT-GAAACTGATGGGCTCTCTCATTCT-------TCCTCAG 263
```

ENSG00000078898 intron 8

Description: Bactericidal/permeability-increasing protein-like 1 precursor (BPIL1)
Intron number: 8
Human chromosome: 20
Intron start (bp): 31070290
Human intron length : 239
Intron alignment length: 256
Flanking exons length (upstream/downstream): 186/54
SNP density: 0.012552
K tree score: 0.0757
Scaling factor: 0.9249
Human-chimpanzee distance: 0.004208
Total primate branch length: 0.0903

ENSG00000078898 exon 8

```
Homo    CATCATCCTGCCCACGGATGCCACCCCTTTTGTGTTGCCAAGGCATGTGGGTACCGAGGGCTCCATGGCCACCGTGGGCC 80
Pan     CATCATCCTGCCCACGGATGCCACCCCTTTTGTGTTGCCAAGGCATGTGGGTACCGAGGGCTCCATGGCCACCGTGGGCC 80
Macaca  CATCATCCTGCCCACGGATGCCACCCCTTTTGTGTTGCCAAGGCATGTGGGCACTGAGGGCTCCATGGCCACTGTGGGCC 80
Bos     CATCGTTCTGCCTGTGGATACTACCCCCTTCGTGCTGCCACAGCACATGGGCACCAAGGGTGCCATGGCAACCGTGGGCC 80
Canis   CATCGTCCTGCCTGTGGATGTCACCCACTTTGTGCTGCCGCCTAACGTGGGTGCTGATGGTGCCATGGCAACCGTGGGCC 80

Homo    TCTCCCAGCAGCTGTTTGACTCTGCGCTCCTGCTGCTGCAGAAGGCCGGTGCCCTCAACCTGGACATCACAGGGCAGCTG 160
Pan     TCTCCCAGCAGCTGTTTGACTCTGCGCTCCTGCTGCTGCAGAAGGCCGGTGCCCTCAACCTGGACATCACAGGGCAGCTG 160
Macaca  TCTCCCAGCACCTGTTTGACTCTGCGCTCCTGCTGCTGCAGAAGTCCGGCGCCCTCAACCTGGACATCACAGGGCAGCTG 160
Bos     TCTCCCAGGACCTGTTTGACTCTGTCATCATGCTGCTGCAGAAGGCTGGTGCGCTCAACCTGGACGTCACAGCGCAGCTG 160
Canis   TCTCCCAAGACCTGTTTGACTCCGCCCTCCTGCTGCTGCAGAAAGCTGGTGCCCTCAACCTGGACATCACAGGGCAGCTG 160
```

ENSG00000078898 exon 9

```
Homo    AGGTCGGATGACAACCTGCTGAACACCTCTGCTCTGGGCCGGCTCATCCCGGAG 54
Pan     AGGTCGGATGACAACCTGCTGAACACCTCTGCTCTGGGCCGGCTCATCCCGGAG 54
Macaca  AGGTCGGATGACAACCTGCTGAACACCTCTGCACTGGGCTGGCTCATCCCGGAG 54
Bos     AATTCGAGTAACAACCCGCTGAACACCTCTGTGCTGGGCCAGTTCATCCCCGAG 54
Canis   AAGTCTGAGGACAACCTGCTGAACACCTCTGTGCTGGGCCAGCTCATCCCCGAG 54
```

ENSG00000078898 intron 8

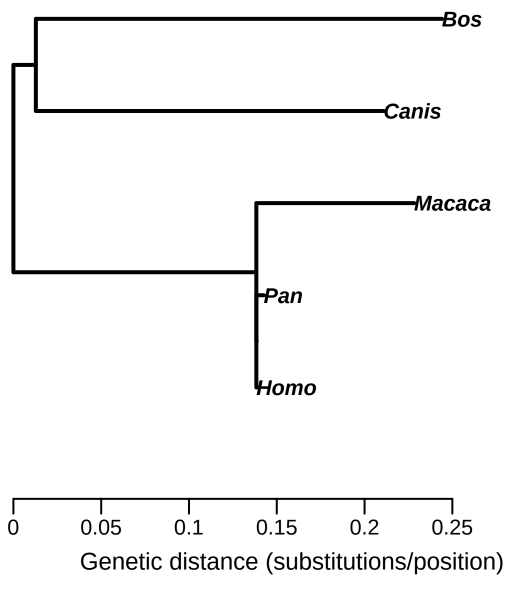

ENSG00000078898 intron 8

```
Homo    GTGAGGGCCCGACCTGCAGCCCAGGGCCTGTGGGGCAAGAGCTCCCTGTGGCCCAGCCTAGGGAGACCTCCCAGAGGCCAAATCATGGGTGGGTGGGGCC 100
Pan     GTGAGGGCCCGACCTGCAGCCCAGGGCCTGTGGGGCAAGAGCTCCCTGTGGCCCAGCCTAGGGAGACCTCCCAGAGGTCAAATCATGGGTGGGTGGGGCC 100
Macaca  GTGAGGGCCTGACCTGCAGCCCAGGGTCTGTGGGGCAAGAGCTCCCTGTGGCCCAGCCTAGGGAGACCCCCCAGAGGCCATATTGTGGGTGGGTGGGACT 100
Bos     GTGAGAGCTGGTCCTTCCGCCCTGGGCACGTGGGGCAGGAGTGCCCTGTTGCCCAGCTTGGGGAGACCCTGCTGAGGCCAAATCCTGAGTGGGTGGGGCA 100
Canis   GTGAGGCTCGGACCTGCCG-CCAGAGCCTGTGGGACAGGCATGCCCTGCGGCCCAGCCTGGGGAGGCCCCGCAAAGGCCAAATT----GTGGGTGGGGCA 95

Homo    GCTGAAGC--TGGCGCCACAGGGTGGCTGTTGGAAGGGTTAAACGGGGGCTATGGGGAGCGATTGCTCAGGGG--------------GAA-AGTGGTGAT 183
Pan     GCTGAAGC--TGGCGCCACAGGGTGGCTGTTGGAAGGGTTAAACGGGGGCTATGGGGAGCGATTGCTCAGGGG--------------GAA-AGTGGTGAT 183
Macaca  GCTAGAGT--TGGCGCCACAGGGTGGCTGCTGGAAGGATTAAACAGGGGCTATGGGGAGCGATTGCTCGGGGG--------------GGACAGTGGTGAT 184
Bos     GTTGAAGCTTTGGGGCCACAGGGTGGCTATTGTAAGGGTTAGACAGAGGGGTAGGGTGGGATTAGACAGAGGAGTTGGGATGGAGAGACAAGAGGCGGT 200
Canis   GCCCAAGTTTTGCGGCTACAGGGTGGCTGTTGCAAAGGTTCAAAGGCGGTTGTGGGGA-----------------------------GGCAACAGGTGGT 166

Homo    CTGTCTTGGGGAGCAGCTGTCCTAAAACCTGTTGTGGCCTGGGGTGCTGATTTCAG 239
Pan     CTGTCTTGGGGAGCAGCTGTCCTAAAACCTGTTGTGGCCTGGGGTGCTGATTTCAG 239
Macaca  CTATCTT-GGGAGCAGCTGCCCTAAAACCTGTTGTGGCCTGGGGGGCTGATTTCAG 239
Bos     CTGGCTTGGGGAGTGACTGTTCTAAAACCTGTCGTGTGTTGGGA-GTTGATTTCAG 255
Canis   CTAGCTTGAGGAGTGGCTGCCCTAAAACCTGCCATGACGTGGGG-GCTAATTTCAG 221
```

ENSG00000141076 intron 8

Description: Cirhin (CIRH1A)
Intron number: 8
Human chromosome: 16
Intron start (bp): 67745085
Human intron length : 712
Intron alignment length: 809
Flanking exons length (upstream/downstream): 97/65
SNP density: 0.005618
K tree score: 0.0627
Scaling factor: 0.8771
Human-chimpanzee distance: 0.010137
Total primate branch length: 0.0903

ENSG00000141076 exon 8

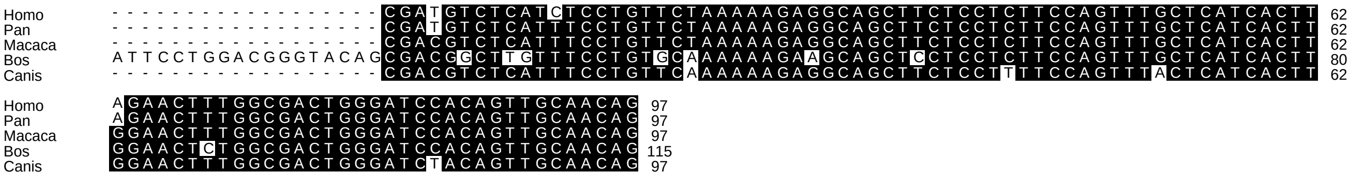

ENSG00000141076 exon 9

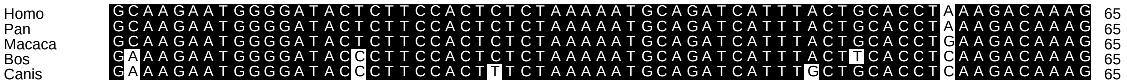

ENSG00000141076 intron 8

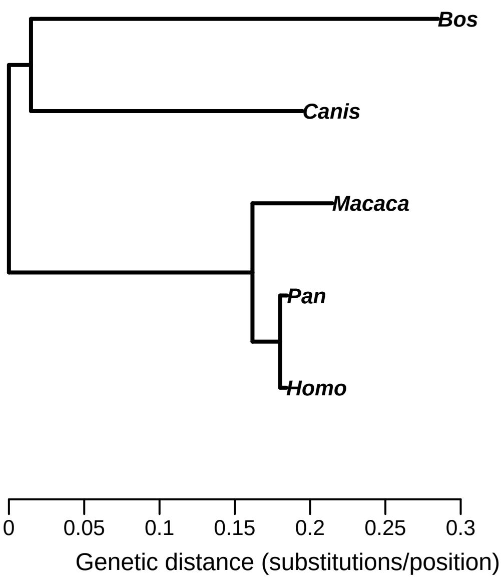

ENSG00000141076 intron 8

```
Homo    GTAAGATGGGAGCACGTTTTTTT-CAATAAGAAAACT-GGAGAGAGAAGCCAGAAATCAGAATTGCGGTTGGAATTCTGCTTATAGAAAAACTGAAGTAG 98
Pan     GTAAGATGGGAGCACGTTTTTTT-CAATAAGAAAACT-GGAGAGAGAAGCCAGAAATCAGAATTGCGGTTGGAATTCTGCTTATAGAAAAACTGAAGTAG 98
Macaca  GTAAAATGAGAGCACGTTTTTTT-CAATAAGAAAACT-GGAGAGAGAAGCCAGAAATCAGAATTGCGGTTGGAATTCTGCTTATAGAAAAACTGAAGTAG 98
Bos     GTAAGCTGGGAGAAGGTTTTTTCACAACAAGAAAACTGGGAGAGAGAAGCCAGA------AATTGCAGTTGAAATTCTGCTTGTGGAAAAATGGAAGTCA 94
Canis   GTACAATGAGAGTTTA-CTTTTC-CAATAAGAAAACT-GGAGAGAGAAGTAGAAAATTAGAATTGCAGTTGAAATTTTGCTTGTGGAAAAATGGAAGTAG 97

Homo    ACTTTTGTGTCCATTTTAACTAATGGT-TTTCCCACCCATTAGATAAAGCAAATAAGCTGTTTAGAGTATTAGAATCTTTAGCAGGATATTGGATCATCT 197
Pan     ACTTTTGTGTCCATTTTAACTAATGGT-TTTCCCACCCATTAGATAAAGCAAATAAGCTGTTTAGAGTATTAGAATCTTTAGCAGGATATTGGATCATCT 197
Macaca  ACTTTTATGTCCATTTTAACTAATGGT-TTACCCACCCATTAGATAAAGCAAATAAGTTATTTAGAGTATTAGAATCTTTAGTAGGATATTGAATCATCT 197
Bos     ATTATTTTTCTGTTTGACATCCTTACTGTTTCCTGTCCACTAGATAGAGCAGAGAAGTTCCTCAGAGTGTGAAAATCATTAGCTGGACACCGAATCACCT 194
Canis   ATTTTTCTG--------------TGTC-TTTGGCAGCTTATGGGCAGAGCAAAAGAGTTCTTC--AGTGTTAGAATCATTAGCAGGATAATGATTC-TAT 179

Homo    CTTAAAAGTTTATGGCTCTAGGTGGTGCGCTGCAGGGTGTAGTCAAGGTAAGGGTTGAAGAATCGTAGTTTGCGCTAGCTTAACTGAGGTGAAAACAGGC 297
Pan     CTTAAAAGTTTATGGCTCTAGGTGGTGCGCTGCAGGGTGTAGTCAAGGTAAGGGTTGAAGAATCGTAGTTTGCGCTAGCTTAACTGAGGTGAAAACAGGC 297
Macaca  CTTAAAGGTTTATGGCTTGTGGTGGTGCGCTGCAGGGTGTAG--------------------------------------TAACTGAGAAGAAAACAGGC 259
Bos     TTGGAAAGTGTGCAGTTCCATACAGTGCACCACAGGGTATCAGGGAGGCAGGGGTAGTGGAAT---AGTTCGCCCTGGCTGAACTGGGATCAGAACAGGC 291
Canis   GTTAAAAGTTGATGATTCTATAAGGTACACTGTAAGGCATAGTGGAGGTAAAGGTTATAGAATC--AGTTTGCCCCATCTTGACTGGGATCAAAACAAGC 277

Homo    TGTTGAAAAACAACAAGATTTAATGATGGGTAATGATAATCCTGTA--GAAAAAGAAGATCACATGGATCTTATTGGAGTGACAGCCTAGTGGCCTTTT- 394
Pan     TGTTGAAAAACAACAAGATTTAATGATGGGTAATGATAATCCTGTA--GAAAAAGAGGATCACATGGATCTTATTGGAGTGACAGCCTAGTGGCCTTTT- 394
Macaca  TGTTGGAAAATAACAAGATTTAATGGTGGGTAATGATGATCCTGTA-CGAAAAAGAAGATCACATGGATCTTATTGGAGTGGCAGCCTAGTGGCCTTTTT 358
Bos     TGTTGAAAAATGACAGATTTTCATGCTGAGTTCTGTCAAGCCTTCA-TGAAAAAGAAGGTCATGCAGATCTTACCAGGGTGACAGCCTCGTGGCCTTAG- 389
Canis   TGCTG-AATATGATAAGGTTAATGTTGGGGAGTGACAGTCCTACAGTGAAAAAGAAGGTCACGTTGATCTTACTAGGGTGATAGCCTTAGTGGCCTTGT 376

Homo    ---TGACTTATTAAGGAAATGTTTGAGTCCT-CTCATTTGCAGTGG-----------------------------------------ATTAAAATCGAGG 449
Pan     ---TGACTTATTAAGGAAATGTTTGAGTCCT-CTCATTTGCAGTGG-----------------------------------------ATTAAAATCGAGG 449
Macaca  GTGTGACCTATTAAGGAAATGTGTGAGTCCT-CTCTTTTGCAGTGG-----------------------------------------ATTAAAATTGAGG 416
Bos     GTATGAC---------ACATGTGTGGGTGCTGTTGTTTTATAGTAG-----AGGCTTTGGCTTTAGGCTCCACGATGAACAAAAGAAGTTGAAGTTGAGG 475
Canis   GTATGACCTGTTCTGGAAGTGTTTGGGCTCTGTTGTTTTATAGTAGGTGAGAGGTTTTTGCTTTAGATTCCAAAATGAGCAAATGAAGTTGTAGTCAAAG 476

Homo    CCACACAAACTCC----------ACCTGTGTCCCTGGCTATGGACTAGAGCTTCAGGGAATTAGTGATGTATCAG-----------------------AG 516
Pan     CCACACAAACTCC----------ACCTGTGTCCCTGGCTATGGAGTAGAGCTTCAGGGAATTAGTGATGTATCAG-----------------------AG 516
Macaca  CCACACAGACTCC----------ACCTGCGTGCCTGGCTATGGAATAGAGCTTCAGAGAATTAGTGATGTATCAG-----------------------AG 483
Bos     CCACACAAACTTCACATCCATCCACCTGCGTGCCTGGCTGTGGGGAGCAGCTTCAGAAAAGAAGCGCCATGGCCA----------CATTCAGGCCCCCA 565
Canis   CCACACAAACCCCACTTCCACCTACCTGCATGCCTGGCTGTGGGTTGTAGCTTCAGAGAATTAATGATATACCTACCATAGGCCACATCCAAGGCACCCA 576

Homo    GATAGAAGCAAAAGAAGTACTCACTGCTTT-CTCTTCATATACC----CCTCAAAAATTGTGAGCTTACTCAGTGAGCTTGTATTTTTCTGATTTCCCAC 611
Pan     GATAGAAGCAAAGGAAGTACTCACTGCTTT-CTCTTCATATACC----CCTCAAAAATTATGAGCTTACTCAGTGAGCTTGCATTTTTCTGATTTCCCAC 611
Macaca  G-TAGAAGCAAAGGAAGTACTCACTGTTTTCCTCTTCATATACA----CCTCAAAAGTTGTGAGCTTAC-------------ATTTTTCTGATTTCTCCC 565
Bos     CGGAGAGGCCAAGAAAGTGCTCGCTGTTCTCCTCTTCGTATACATACCCCAGAAGACTCTAAGCTCACTCTGTCAGAC---ATGTTTCTGATTTCCCAG 662
Canis   AATAGAAGCTAAGAAAGTACTCAGTGCTTTCTGCCTCCTATAAG----CCCAGAAAACTGTGGGTTTACTAAGTCAGAC---ATTTTTCTGATTTCCTAG 669

Homo    TGA-TAGAGATTCCATTTTTCCAAGGAGTAACTTTTTAG------AAG-AAAAATGTACAAATACTCTTTCTTTCATAAGAAAAATCTCAGGGAAGTTTC 703
Pan     TGA-TAGCGATTCCATTTTTCCAAGGAGTGACTTTTTAG------AAGAAAAAAATGTACAAATACTCTTTCTTTCATAAGAAAAATCTCAGGGAAGTTTC 704
Macaca  AGA-TAGAGATTCCATTCTTCCAGGGAGTGACTTTTTAG------AAGAAAAAAAATGTACAAATTC--TCTTTCATAAGAGAAATCTCAGGGAAGTTTC 656
Bos     TGG-TAGAGAGTCCATTCTACCCATTTTTTCTTTTTAAA------AGAAAAAAACATTTGTATAGCCTTCCTTTGGGAAGAAAAATATCAGAGATGTTTC 755
Canis   TGGTTAGCGATTCCATTCTACTCAGGAGTGACTTTTTAGTTAAAAAAAAAAAAAGTACAAATGATCTTTCTTTGAGGAAGGAAAACCTCAGGGAAATTTC 769

Homo    TTGTTTCAG 712
Pan     TTGTTTCAG 713
Macaca  TTGTTTCAG 665
Bos     TTGTTCCAG 764
Canis   TTGTTCCAG 778
```

ENSG00000073756 intron 3

Description: Prostaglandin G/H synthase 2 precursor (PTGS2)
Intron number: 3
Human chromosome: 01
Intron start (bp): 184914160
Human intron length : 653
Intron alignment length: 829
Flanking exons length (upstream/downstream): 144/144
SNP density: 0.006126
K tree score: 0.0476
Scaling factor: 1.0661
Human-chimpanzee distance: 0.020611
Total primate branch length: 0.0894

ENSG00000073756 exon 3

```
Homo    CGGAATTTTTGACAAGAATAAAATTATTTCTGAAACCCACTCCAAACACAGTGCACTACATACTTACCCACTTCAAGGGA 80
Pan     CGGAATTTTTGACAAGAATAAAATTATTTCTGAAACCCACTCCAAACACAGTGCACTACATACTTACCCACTTCAAGGGA 80
Macaca  CGGAATTTCTGACAAGAATAAAATTACTTCTGAAACCCACTCCAAACACAGTGCACTACATACTTACCCACTTCAAGGGA 80
Bos     CCGAATTTCTGACAAGAATAAAATTACTCCTGAAACCCACTCCCAACACAGTGCACTACATACTTACCCACTTCAAAGGA 80
Canis   CGGAATTTCTGACAAGAATAAAATTATACCTGAAACCCACTCCAAATACAGTACACTACATACTTACCCACTTCAAGGGA 80

Homo    TTTTGGAACGTTGTGAATAACATTCCCTTCCTTCGAAATGCAATTATGAGTTATGTGTTGACAT 144
Pan     TTTTGGAACGTTGTGAATAACATTCCCTTCCTTCGAAATGCAATTATGAGTTATGTGTTGACAT 144
Macaca  TTTTGGAACGTTGTGAATAACATTCCCTTCCTTCGAAATGCAATTATGAGTTATGTGTTGACGT 144
Bos     GTCTGGAACATTGTCAATAAGATCTCCTTCCTGCGAAATATGATTATGAGATATGTGTTGACGT 144
Canis   GTCTGGAACATTGTCAATAACATCCCCTTCCTGCGAAATACAATTATGAAATATGTGTTGACAT 144
```

ENSG00000073756 exon 4

```
Homo    CCAGATCACATTTGATTGACAGTCCACCAACTTACAATGCTGACTATGGCTACAAAAGCTGGGAAGCCTTCTCTAACCTC 80
Pan     CCAGATCACATTTGATTGACAGTCCACCAACTTACAATGCTGACTATGGCTACAAAAGCTGGGAAGCCTTCTCTAACCTC 80
Macaca  CCAGATCACATTTGATTGACAGTCCACCAACTTACAATGCTGACTATGGCTACAAAAGCTGGGAAGCCTTCTCTAACCTC 80
Bos     CGAGATCACATTTGATTGAGAGTCCACCAACTTATAATGTGCACTACAGCTATAAAAGCTGGGAAGCCTTTTCTAACCTG 80
Canis   CCCGGTCACATTTGATTGAGAGTCCACCAACTTATAATGTGAACTACGGCTATAAAAGCTGGGAAGCCTTTTCTAACCTC 80

Homo    TCCTATTATACTAGAGCCCTTCCTCCTGTGCCTGATGATTGCCCGACTCCCTTGGGTGTCAAAG---- 144
Pan     TCCTATTATACTAGAGCCCTTCCTCCTGTGCCTGATGACTGCCCGACTCCCTTGGGTGTCAAAG---- 144
Macaca  TCCTATTATACCAGAGCCCTTCCTCCTGTGCCTGATGACTGCCCAACTCCCTTGGGTGTGAAAG---- 144
Bos     TCTTATTATACCAGAGCTCTTCCTCCTGTGCCTGATGACTGCCCAACACCCATGGGTGTGAAAGGTGA 148
Canis   TCCTATTATACCAGAGCTCTTCCCCCTGTACCTGATGACTGTCCAACACCCATGGGTGTGAAAG---- 144
```

ENSG00000073756 intron 3

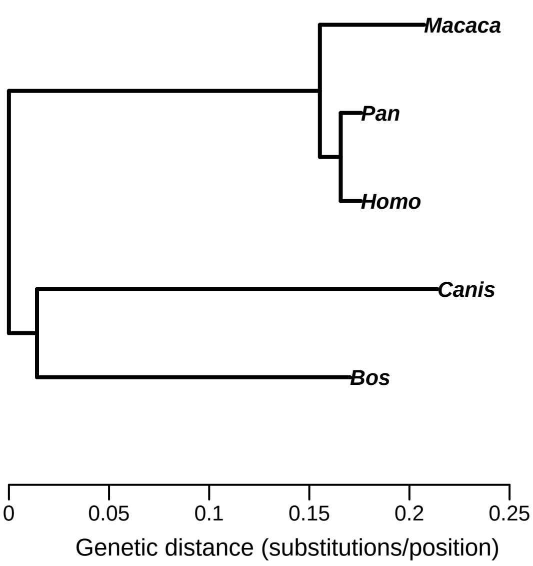

ENSG00000073756 intron 3

```
Homo    GTAAGTACAAGTGTCTTTCTAAGGTTTTTAGCCTTCTCAAAGAAAAATATGCTTTATAATACTGTAAGCCT----------------------------- 71
Pan     GTAAGTACAAGTGTCTTTCTAAGGTTTTTAGCTTTCTCAAAGAAAAATATGCTTTATAATACTGTAAGCCT----------------------------- 71
Macaca  GTAAGTACAAGTGTCTTTCTAAGG-TTTTAGTTTTCTCAAAGAAAAATATGCTTTATAATACTGTAAGCCT----------------------------- 70
Bos     GTAAGTACACATCACTTTCTAGTGTTCTCAGCTGCCTCAAAGAGAAATGTACTTTACAA------ATTTTA----------------------------- 65
Canis   GTAAGTACAAGTCTCTTTCTAAGGTTTTCAATTTCCTCAAAGAAAAATGTTCTTTATAAGACTTTAGATTATTAAACCTTTTAAGATTTTATTTATTTGA 100

Homo    ----------------------------------------------------------------AATCTAAAAACATATTTCCAAGCTTATCAAAA-AGA 106
Pan     ----------------------------------------------------------------AATCTAAAAACATATTTCCAAGCTTATCAAAA-AGA 106
Macaca  ----------------------------------------------------------------AATCTAAAAACATATTTCCTAGTTTATCAAAATGAA 106
Bos     ----------------------------------------------------------------GATTTTAAAACTTAAT----------TCAAAACATG 91
Canis   GAGAATGAACCTGAGTGGGGGGAGGGGTGGAGGGAGAAGCAGACTCCCTGCCGAGCAGGGAGCCAGACAAGGGACTCAATCCAAGGACCCTGGGATCATG 200

Homo    CTTTAAGATAGCTTTTAAG---------------TTTGCCTTCCATCTTAATCGCCAAAAATATTGACATTT-AGTCCCATCCAGTTTATACAG------ 184
Pan     CTTTAAGATACCTTTTAAG---------------TTTGCCTTCCATCTCAATCGCCAAAAATATTGACATTT-AGTCCCATCCAGTTTATACAG------ 184
Macaca  TTTTAAGATACCTTTTAAG---------------ATTGCCTTCCATCTTAACGGCCAAAACTATTGACATTT-AGTCCCATCCAGTTTATACAG------ 184
Bos     TTTAAATATACCATTTTAG---------------TTTGCCTACTATCTTAATGGCCAAAAATATTGACATCTGAATCCCTTGAAGTTTCTAAAGTTCTGT 176
Canis   ACCTGAGCCAAGGGCAGAGCTACCCAGCCGCCCCTTTGCCCTCTATCTTAATGGCCCAAAATATTGACATTTCGGTCCCATTCAGTTTGTAAAGTTCTGT 300

Homo    --------------TCTGCTCACAACTCTGTATACCTCTTCTAACCTTTACTGTTTGGT-CAGTTTGTGGAGGTAGCATGGTCCAGCTGTTTATTGAATG 269
Pan     --------------TCTGCTCACAACTCTGTATACCTCTTCTAACCTTTACTGTTTGGT-CCGTTTGTGGAGGTAGTATGGTCCAGCTGTTTATTGAATG 269
Macaca  --------------TCTACTCACAACTCTGTATACGTCTTCCAACTTTTACTATTTGGT-CAGTTTGTGGAGGTAGCACGGTCCAGCTGTTTATTAAATG 269
Bos     CTCACAGGCAGAATTCTACTTAAAATACTGCATGCTTCTGCTGACCTTCACTGTTCTCCACAGTTTGTGAAAGATGCCTGGTCCAGTTGTTTATTGAATG 276
Canis   CTCAAATTCAGAATTCTACTTACAATCCTGTATACCTCTTCTAA-ATTCACTGCTCTTACCAATC--TGAAAGAAGCGTGGTCCAGTTGTTAATTGAATA 397

Homo    CCCATGGGCCACAGAATTGTTCTGAACATGTAGCACCCATTAAAATA-------AATTTGGATTTGGATCAGCAAGAAAATAACTTTCCATGATTCTAAA 362
Pan     CCCATGGGCCACAGAATTGTTCTGAACATGTAGCACCCATTAAAATA-------AATCTGGATTTGGATCAGCAAGAAAATAACTTTCCATGATTCTAAA 362
Macaca  CCCATGGGTCACAGAATTGTTCTGAACATGTAGCACCCATTAAAATA-------AATTTATATTTGGATCAGCAAGAAAATAACTTTCCATGATTCTAAA 362
Bos     GCCATGGGGCACAGAATT--------------GCACCCATTAAAATAAATGAATAATTTGGATTGGGGTCGGCGAGAAAGTAACTTT---TATTTCTAAT 359
Canis   TCCATGGACCAC---------------------------------------------------------------------------------------- 409

Homo    GTGGGTGCCATACTCAGCCATTCCTTTCATAGGCCTCTTGGATAGTGAGCAGATGGCTACCTG--AAAAATCAATATTGCCAGATTATAATG-----TGC 455
Pan     GTGGGTGCCATACTCAGCCATTCCTTTCATAGGCCTCTTGGATAGTGAGCAGATGGCTACCTG--AAAAATCAATATTGCCAGATTATAATG-----TGC 455
Macaca  GTAGGTTCCATGCTCAGCCATTCCCTTCATAGGCCTCTTGGACAGGGAGCAGATGGCTACCTG--AAAAATCAATATTGTCAGATTATAATG-----TGC 455
Bos     GTGAGTGTCAAGCACAGACGTTTTGCTCATGGGCCTCTTGAACAATAAGTGGATGGTTATTTGAAAAAAATGAAAATGGCTAGACCAAAACATCTTCTGT 459
Canis   ---------------------------------------------------------------------------------------------------- 409

Homo    AGAGTATATGTATTTTATTAAAGATGTATTTCAAGTGGCCATT---------------------------AGACTATAAAGTGTAGTTGTTTAAAAATAG 528
Pan     AGAGTATATGTATTTTATTAAAGATGTATTTCAAGTGGCCGTT---------------------------AGACTATAAAGTGTAGTTGTTTTAAAATAG 528
Macaca  AGAGTATATGTATTTTATTAAAGATGCATTTCAAATGGCCATT---------------------------AGATTTTAAAATGTAGTTGTTTTAAACTAG 528
Bos     AAAGAATATATACTTTATCAAAGATGCCTTTTAATGCATCATTCTTTCCTCTTTTTTGAGCACCAGTAGCAGGCTTTAAAGTATGATTGCTTTAAAATAG 559
Canis   ---------------------------------------------------------------------------------------TGGTTTACAATAG 422

Homo    ATT-TTTTT-TA-TTTTGGAGTTACATTCAACCTCAGGTGCCACTTTCCACATTTTACAATAAAAATAATGGTTGATTTACTTAACAAATGAGAATAAAT 625
Pan     ATT-TTTCT-TATTTTTGGAGTTAAATTCAACTTCTAGTGCCACTTTCCACATTTTACAATAAAAATAATGGTTGATTTACTTAACAAATGAGAATAAAT 626
Macaca  ATTATTTTT-GATTTTTGGAGTTAAATTCAACATCTAGTGCCACTTTCCACATTTTACAATAAAAATAATGATTGATTTACTTAGCAAATGAGAATAAAT 627
Bos     AGTATCTTTGTATTAATTAAATTAAATTTA--TTCTAGTGCCACTTTGTACATTATACAATAAAAATGAGCATTGATTTGCCTTGTGGATGGGAACAAAT 657
Canis   GTTATCTTT-----GATGGAATTAAATTCGGTCTTTAGTGCCACTTTGCACTTTGTACAATAAAAGTGAACATTGATATGTCTTGCGTATAGGAACAAAT 517

Homo    AAAAC-ATTTTTTTCTTTGAAAATTTCAG 653
Pan     AAAAC-ATTTTTTTCTTTGAAAATTTCAG 654
Macaca  ACAAC--TTTTTTTCTTTGAAAATTTCAG 654
Bos     AACAT--TTTTTTTCCTCTAAAATTTCAG 684
Canis   AAAACAATATTTTTTTTCTTAAATTTCAG 546
```

ENSG00000136878 intron 6

Description: Ubiquitin carboxyl-terminal hydrolase 20 (USP20)
Intron number: 6
Human chromosome: 09
Intron start (bp): 131663713
Human intron length : 1573
Intron alignment length: 2041
Flanking exons length (upstream/downstream): 70/114
SNP density: 0.016529
K tree score: 0.0437
Scaling factor: 0.5776
Human-chimpanzee distance: 0.010343
Total primate branch length: 0.0893

ENSG00000136878 exon 6

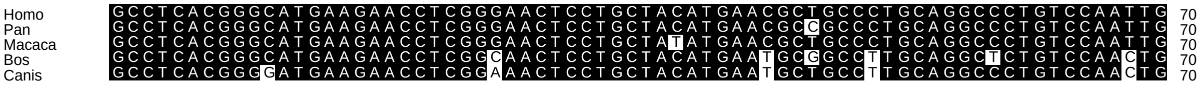

ENSG00000136878 exon 7

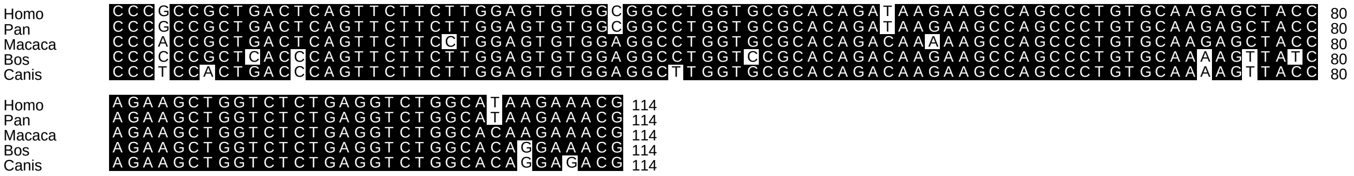

ENSG00000136878 intron 6

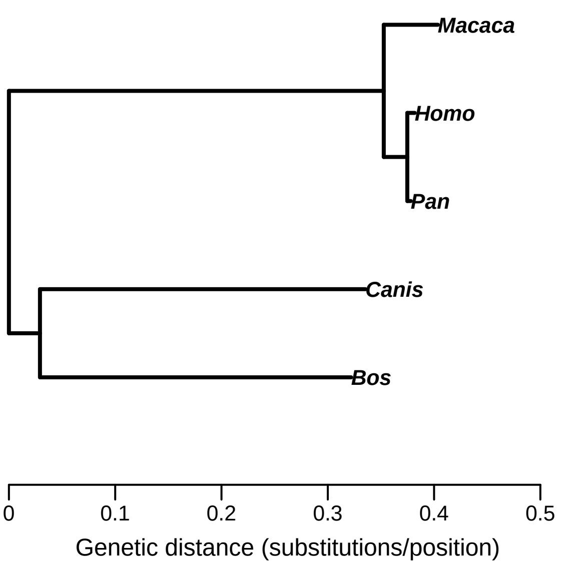

ENSG00000136878 intron 6

```
Homo    GTAGGTCGACACTTTGTCCGAGGGCCGAGAGCTG-----TCCCTGGCAAAGCCCCGGAAACAGCAGCAGTAGCAGCC--------CCCAGCCGGTTGCCC 87
Pan     GTAGGTCGACACTTTGTCCGAGGGCCGAGAGCTG-----TCCCTGGCAAAGCCCCGGAAACAGCAGCAGTAGCAGCC--------CCCAGCCGGTTGCCC 87
Macaca  GTAGGTCGACACTTTGTCCAAGGGCCGAGAGCTG-----TCCCTGGCAAAGCCCCAGAAACAGCAGCAGTAGCAGCT--------CCCAGCCTGTTGTCC 87
Bos     GTAGGT-TCGCCTCATCTGGGATTTGGGGG---------------------GCCCTGGGAATAGCAGCTGCCGCCCCAAGTTGGGGCGTGTGTGTTGCTC 80
Canis   GTAGGTTGTTACATCGTCTGGGAGCTGGAGGCCAGGAGCTGCCGCCCGGCGCCCTGGAAGTAGCA---GCAGCCCCCAGGCTGTGCTGTGTGTGTTTCCC 97

Homo    ACAAACACATGTATA-GGATGCA---CAGCAAAATGCCTGAAAGCATGTATACCCTTTGACCCAGCAATCCTCCCAGTCAGAATGTATCCTGAGGAGATA 183
Pan     ACAAACACATGTATA-GGATGCA---CAGCAAAATGCCTGAAAGCATGCATACCCTTTGACCCAGCAATCCCCCCAGTCAGAATGTATCCTGAGGAGATA 183
Macaca  ACAAACACATGTATA-GGATGCACAGCAGCAAAATTCCTAAAAACATGCGTGCCCTTTGACCCAGCAATCTCCCCAGTCGAAATGTGTCCTGAGGAGATA 186
Bos     ATAGAAGCCTGGATGTGGGTACA---CATCAAAACGCCCCAAGCCACACCTGCCCTTTGACCCAGCAATCCCTCCAATGGGAATTTCTCCTAAAGGAGGT 177
Canis   ACAGATGCATGTATACGGTTGTG---CGTCAGAAC-CCTTAGAACACACATGCCCTTTGACCCAGCAATCCCTCCTGTCAGAATTAATCCTGGCAAGGTC 193

Homo    A-------CCGGAGATGCTGGGGAAGTTGTACGTGCAAGGAGGCTCAGTGCTCTGTTGTTTACTGGAAATGATTTAAAGGCCCATCTGCAA---------- 267
Pan     A-------CCGGAGATGCTGGGGAAGTTGTACATGCAAGGAGGCTCAGCGCTCTGTTGTTTACTGGAAATGATTTAAAGGCCCATCTGCAA---------- 267
Macaca  A-------CCAGAGATGCTGGGGAAGTTGTACATGCAAGGAGGCCCACCGCTCTATTGTTTATTGGAAACGATTTAAAGGCCCATCTGCAA---------- 270
Bos     ACCAGTACCCAGAGAGGTTGAAAAAGCTGTCTGTGCT-----GTTCCTCACCCTGCCATTTATTGGAAACATGTTAAAGGCCCATA-------------- 258
Canis   A-------TCTGAGAAGTGGAGCACGCCCTGGGGGA-----TGTTCCTATCTTGTTGTTTATGGGGAACCGTTTAATGGTCTACATTTAGTTTTTTAAA 281

Homo    ---------------------------------------------------------------------------------------------------- 267
Pan     ---------------------------------------------------------------------------------------------------- 267
Macaca  ---------------------------------------------------------------------------------------------------- 270
Bos     ---------------------------------------------------------------------------------------------------- 258
Canis   AGATTTTATTTATTTATTTGAGAGAAAGAGAGAGCATAAGCAGGAGGAGGGGCAGAGGCAGAGGGAGAAGCAGATTCCCTGCTGAGCAGGGAGCTCGATG 381

Homo    ----------------------GAGAGTATAAATT---CTCATAGC------------------------------------------------------ 288
Pan     ----------------------GGGAGTATAAATT---CTCATAGC------------------------------------------------------ 288
Macaca  ----------------------GGGAGTATAAATT---CTCATAGC------------------------------------------------------ 291
Bos     ----------------------AAAATCACAAATTCAGTACAGACCAG---------------------------------------------------- 284
Canis   TGGGGCTTGATCCCAGGACCCTGAGATCATGACCTGAACCTAAATCAAGAGTTGGCCGCTCAATTGACTGAGCCACCCAGGTGCCCCAATGGTCCACATA 481

Homo    ------------------------------------------------------------------------------CCTTGCAGCAAGTGCAGGAAAA 310
Pan     ------------------------------------------------------------------------------CCTTGCAGCAAGTGCAGGAAAA 310
Macaca  ------------------------------------------------------------------------------CCTTGCAGCAAGTGCAGGAAAA 313
Bos     ------------------------------------------------------------------------------CTTTACGGTGAGTTCAGGGAAA 306
Canis   TTTTTTTTTTTTAAGATTTTATTTATTCATGAGAGACAGAGAGAGAGGCAGAGACACAGGCAGAGGGAGAAGCAGGCTCCCTGCAGGGAGCCCGATGTGG 581

Homo    AT-------------------------------------------------------------------------------------------------- 312
Pan     AT-------------------------------------------------------------------------------------------------- 312
Macaca  AT-------------------------------------------------------------------------------------------------- 315
Bos     AAT------------------------------------------------------------------------------------------------- 309
Canis   GACTCCATCCCGGGTCTCCAGGATCATGCCCTGGGCTGAAGGCGGCACTAAACCACTGAGCCACCCGGGCTGCCCTCAATGGTCCACATTTAAAGGCCAG 681

Homo    ----------GGCACAAGACAACATGCATTGAAACAAGAAGGGGTTCAGAATCTGGTGAGTGGGGGAAAAAGGAGGTTATAAAGCATTGCATGGTACAAT 402
Pan     ----------GGCACAAGACAACATGCATTGAAACAAGAAGGGGTTCAGAATCTGGTGAGTGGGGGAAAAAGGAGGTTATAAAGCATTGCATGGTACAAT 402
Macaca  ----------GGCACAAGACAGCATGCATTGAAACAAAACAGGTTTCAGAATCTGGTGAGTGGGGGAAAAAGGAGGTTATAAAGCATTGTAAGGTAAAAT 405
Bos     ----------GGTGCAGGACAATATTTACTGAAACGGGGAGGAGTTCAGAATATGATAA------GTAGGAAAGGTTGTGATCCTTTATATGGTGCAGT 393
Canis   CTTTACAGTGAGTGCAGGACAGTATTTACTAAATTGGAGGGGG--TCAGAATATGGCGA---GGGGAGGAAGGAGGTCATGAAGCTTTGTGTGGCGCAGT 776

Homo    CCCGTTTTATGGGAGAAATGCGATTTTCCATTAAGGATAAGGCTGTGAGCTCCATGAGGCCAAGGATGTCTCCTGTGTCTGACGTGCT---TCTCCTACC 499
Pan     CCCGTTTTATGGGAGAAATGCGATTTTCCATTAAGGATAAGGCTGTGAGCTCCATGAGGCCAAGGATGTCTCCTGTGTCTGACGTGCT---TCTCCTACC 499
Macaca  CTCATTTTACGAGAGAAACGCGATTTTCCATTAAGGA-AAGGCTATCAGCTCCATGAGGCCAAGGATGCCTCCTGTATCTGACATGCT---TCTCCTACC 501
Bos     CCCATTTTAGGGGTGAAAATATTTTTTTCCATCTAAGACA--CCCTCTACTCCCTGAGGCT-GGGCCATCTCCTCTGCCTGGCACTCAGGGTCTCACACC 490
Canis   CCCA-TTTGGGGGAGAAATGCCTAGTTTCTCTCTAGGATAGGCTGCTAGCTCCGCGAGGCC-AGGGCATTTCCTATGCCTGGTACACA-GACGTCTCACC 873

Homo    CAGTGGAGGTTAGAGATGCAGTATATGTAT-----------------GCGGGAAGCAGAGAGGGAATGGGTA---CAAATGCGTAGAAAAGTCTGGAAGGC 580
Pan     CAGTGGAGGTTAGAGATGCAATATATGTAT-----------------GCGGGAAGCAGAGAGGGAATGGGTA---CAAATGCGTAGAAAAGTCTGGAAGGC 580
Macaca  CAGTGGAGATTAGAGATGCTGTATATGTAT-----------------GCAGGAAGGAGAGAGGGAATGGGTA---CAAATGTGTAGAAAAGTCTGGGAGAC 582
Bos     TACTAAGGGTTCTGTGTGT-GTGTGTGTGTGTGTGTGTGAGAGAGAGAGAGAAGGAGGGCAAGGATGGGGGCTCTGCATGCATAGAAAATTCTAGGAGG- 588
Canis   TGGTAAGGATTCAGTACCT-GCATATGTGT-----------------------AGGAGGGAGAGGA---------TGTGTGCCTAGGAAAGTCTGGAAGGC 941

Homo    TATG-TCCCAAAATGTTCCTAGTAACTATCCTCA--GGTGGCAGGATGCAGGTCAATTATTGTTAATAATTGACCACACCCAGGCTGGGCGCAGTAGCCC 677
Pan     TATG-TCCCAAAATGTTCCTAGTAACTATCCTCA--GGTGGCAGGATGCAGGTCAATTATTGTTAATAATTGACCACACCCAGGCTGGGCGCAGTAGCCC 677
Macaca  TATG-TCCCAAAATGTTCCTAGTAACTATCCTCA--GGTAGCAGGATGCAGGTCAATTATTGTTAATAATTGACCGCACCCAGGCCGGGCGCAGTGGCTC 679
Bos     ----------ATATCTTCAGAGTAGCTATCCCCACTCATGGTAGGATTAAGGTCAGTTACTAT---TGTTTGCTTAT------------------------ 652
Canis   TATGAGCCTAAAATCTTCAGAGTAGTTGTCTTCA--CATGGTAGGATTAAACTCCCTTAC------TGTTTGCCCATCTTCAGGTAGGGGAAGGTTTCC- 1032

Homo    ACGCCTGTAATCCCAGCACTTTGGTAGGCCGAGGCAGATGGATCACCAGGTCAGGAGATAGAGACCATTCTGGCTAACATGGTGAAACCCTGTCTCTACT 777
Pan     ACGCCTGTAATCCCAGCACTTTGGTAGGCCGAGGCAGGTGGATCACGAGGTCAGGAGATAGAGACCATTCTGGCTAACATGGTGAAACCCTGTCTCTACT 777
Macaca  ACGCCTGTAATCCCAGCACTTTGGGAGGCCGAGGCAGGTGGATCACGAGGTCAGGAGATCAAGACCATCCTGGCTAACACGGTGAAACCCTGTCTCTACT 779
Bos     ---------------------------------------------------------CTGTATTTCCTAATTTTT------------------------- 670
Canis   ----------------------------ACAGAGTGGGCTGGTGGGAGGCAGCAGGTACTGGATCCTGTCACGGCT------------------------ 1079

Homo    AAAAATACAAAAAACTAGCTGGACGTGGTGGCACGCACCTGTAGTCCCAGCTACTGGGGAGGCTGAGGCAGGAGAATCGCTTGAACCCGGGAGGCGGAGG 877
Pan     AAAAATACAAAAAACTAGCTGGACGTGGTGGCACGCACCTGTAGTCCCAGCTACTGGGGAAGCTGAGGCAGGAGAATCACTTGAACCCGGGAGGCGGAGG 877
Macaca  AAAAATACAAAAAACTAGCTGGACTTGGTGGCACGCACCTGTAATCCCAGGTACTGGGGAGGCTGAGGCAGAAGAATCACTTGAACCCAGGAGGCGGAGG 879
Bos     ---------------------------CTACAATAAATGACTATTTCTTA-----------TGTAACGAGGTAACAAAAACAAAGAAAAAAGAAGCGAGG 732
Canis   ---------------------------GGGCAGCAGCGTCCATCCCCTGACCCCACGGGCCCTGAAGTGGACAACTCGGCAGCCCTGAGAGAGGGAAGG 1152

Homo    TTGCAGTGAGCTGAGATCATGCCATTGCATTCCAGCCTGGGTGACAGAGCGAGACTCCGTCTC-AAAAAAAAAAAGAAAAATTGACCGCACCCAATCCGT 976
Pan     TTGCAGTGAGCTGAGATCATGCCATTGCATTCCAGCCTGGGTGACAGAGCGAGACTCCGTCTC-AAAAAAAAAAAA-AAAAATTGACCGCACCCAATCAGT 975
Macaca  TTGCAGTGAGCTGAGATCATGCCATTGCACTCCAGCCTGGGTGACAGAGCAAGCCTCCATCTCAAAAAAAAAAAAAAAAAAATTGACTGCACCTAGTCCGT 979
Bos     GAGAGGC----------------------CTTCAGGTTCAG----------------------------------------------------------- 751
Canis   GGGCCGT----------------------TTAGTGTCTCGG----------------------------------------------------------- 1171

Homo    TTTAACTTTAATAACCTCTTTAAAGGCCCTGTCTTGGCTGGGCACCGTGGCTTACGCCTGTAATCCCAGCACTTTGGGAGGTCAAGGCAAGTGGATCGCC 1076
Pan     TTTAACTTTAATAACCTCTTTAAAGGCCCTGTCTTGGCTGGGCACCGTGGCTTACGCCTGTAATCCCAGCACTTTGGGAGGTCAAGGCAAGTGGATCGCC 1075
Macaca  TTTAACTTTAATAACCTCTATAAAGGCCTTGTCTTGGCTGGGCACGGTGGCTTACGCCTGTAATCCCAGCACTTTGGGAGGCCAAGGCAAGTGGATCACC 1079
Bos     ---------GAAGGTTTCTAAGCAGGGGCCG------------ATGA------------------------------------AAGGAGGGGTGAT---- 790
Canis   ---------GGTGGCCTCTCTTGACTTCTAG------------ATGGCGACCTTCTCTTTGTGTCTTCATGCCATCTTCTCTCTGGGCATGTTTAT---- 1246

Homo    TGAGGTCAGGAGTTCAAGACCAGCCTGGCCAACTAG--TAGAGCCTGTCTCTACTAAAAATACAAAAAAATTAGCCAGGCATTGTGGCATGTGCCTGTAA 1174
Pan     TGAGGTCAGGAGTTCAAGACCACCCTGGCCAACTAG--TAGAGCCTGTCTCTACTAAAAATACAAAAAAATTAGCCAGGCATTGTGGCATGCGCCTGTAA 1173
Macaca  TGAGGTCAGGATTTCAAGATCAGCCTGGCCAACATGGTGAAACCCCGTCTCTACTAAAAATACAAAAAAATTAGCCAGGTGTGGTGGCATGCGCCTGTAA 1179
Bos     -------------------------------------------GCTTCCTCTAATTTTTACACAA------CAGTCT----------------------- 818
Canis   -------------------------------------------GTCCCATCTCCTCCTCATGTAAGGACACCAGTCA----------------------- 1280

Homo    TCCCAGCTACTCGGGAGGCTAAGGCAGGAGAAGCACTTGAACCCGGGAGGCAGAGGTTGCAATGAACCAAGATTGCACCACTGGACTCCAGCCTGGGCAA 1274
Pan     TCCCAGCTACTCGGGAGGCTAAGGCAGGAGAAGCACTTGAACCCGGGAGGCAGAGGTTGCAATGAACCAAGATTGCACCACTGGACTCCAGCCTGGGCAA 1273
Macaca  TCCCAGCTACTTGGGAGGCTAAGGCAGGAGAAGCACTTGAACCCAGGAGGCAGAGGCTGCGGTGAACCAAGACTGCACCACTGCACTCCAGCCTGGGCAA 1279
Bos     -------------------------------------------------------------------------TTAAAGA-------------------- 825
Canis   -------------------------------------------------------------------------TACCAGACTGGGGCCCACCCTG----- 1302

Homo    CAGAGGGAGACTCTGTTTCAAAATAATAATAATAATAATAAAAAATAAGGTAAGATAGATAAGATGAG-----ATAAAATAAAAATGCCCTGTCT---- 1365
Pan     CAGAGGGAGACTCTGTTTCAAAATAATAATAATAATAATAATAAAATAAGGTAAGATAGATAAGATGAGATAAAATAAAATAAAAATGCCCTGTCT---- 1369
Macaca  CAGAGTGAGACTCTGTTTCAAAATAATAAAAATAATAATAAAAGA-------------AGATAAGATGAGATAAAATAAAATAAAAATACGCTGTCTCTAG 1367
Bos     ------AAGACCTTATCTCCAGTGCAT------------------------------------------------------------------TCACATT 853
Canis   ------AGGACCTCATTTGAACCTAATGA-------------------------------------------------------------CCTGTCTCGAA 1336

Homo    ---------------------CTGGGAGTTAGGGCTTCG-CAGATGAATTTT--GGAGGATACAATTT--TCTCATAACTGGTAGGAGCCTTCTAGTCAC 1439
Pan     ---------------------CTGGGAGTTAGGGCTTCA-CAGATGAATTTT--GGAGGATACAATTT--TCTCATAACTGGTAGGAGCCTTCTAGTCAC 1443
Macaca  ATACGGTCACACTCTGAGGTACTGGGGATTAGGGCTTCA-CAGATGAATTTT--GGAGGATACAATTT--TCTCATAACTGGTAGGAGCCTTCTAGTCAC 1462
Bos     CTACGGT--------------GGGGGGTTAGGACTTCAGTGTATGAATTTGCAGGGGGATACAATTCAGCCCCATAACAGGTGGGGTCTGTCAAGTCAC 939
Canis   ATACAGT--------------CACCTTCTGAAGTCTCAACATGTGAATTTG--GGGAGACACATTTCA-CCCCATGACAG--------------GTCAT 1405

Homo    CTTCAGGAGTTTCTGGGTCCAGGGCCACTGTGCTCCCTGTGCGAGCACTCAGGGCT-CCCCGCTATGCAGCCCTTTCTAAAC--TGCCGCATGCCT-CAT 1535
Pan     CTTCAGGAGTTTCTGGGTCCAGGGCCACTGTGCTCCCTGTGCGAGCACTCAGGGCT-CCCCGCTATGCAGCCCTTTCTAAAC--TGCCGCGTGCCT-CAT 1539
Macaca  CTTTAGGCGTTTCTGGGTCCAGGGTCACTGTGCTCCCTGCGCGAGCACTCAGGGCT-CCCCGCTACGCAGCCCTTTCTAAACCGTGCCACCTGCCT-CGT 1560
Bos     CTACAGGTATTTATGGCTCTGGGGGGCCCTGGTATATCCCCACACAGGACAGGTTTTCTCCTCCAGCCAGCTC-TTGTAAATGATGCCGTGTACCCTTCT 1038
Canis   CTGCAGACATTTCTTGCTCAGAGGCAGCAGTATCCCCCTCCCCCCAGAGGCTTTTGGTTCTCTACCCAGTTCTTTCTCACCAATGCTACGTGCCC---- 1501

Homo    TTGCTCTCCTGACCTGG--CTCTCTCT-CCCCTGCACCCAG 1573
Pan     TTGCTCTCCTGACCTGG--CTCTCTCT-CCCCTGCACCCAG 1577
Macaca  TTGCTTTCCTGACCTGGCTCTCTCTCT-CCCTCGCACCCAG 1600
Bos     CTTCTTTGCTGACCTGG----CTCCCTGCCCCCTCCCCCAG 1075
Canis   CTCCTTTTCTGAGCCGG----CTCTTT-CCCTTGCCCTTAG 1537
```

ENSG00000148335 intron 1

Description: UPF0351 protein C9orf32 (METTL11A)
Intron number: 1
Human chromosome: 09
Intron start (bp): 131434966
Human intron length : 1188
Intron alignment length: 1242
Flanking exons length (upstream/downstream): 162/253
SNP density: 0.012626
K tree score: 0.0983
Scaling factor: 0.4155
Human-chimpanzee distance: 0.010384
Total primate branch length: 0.0893

ENSG00000148335 exon 1

```
Homo    GACGAGCGAGGTGATAGAAGACGAGAAGCAATTCTATTCCAAGGCCAAGACCTACTGGAAACAAATCCCACCCACGGTGG  80
Pan     GACGAGCGAGGTGATAGAAGACGAGAAGCAATTCTATTCCAAGGCCAAGACCTACTGGAAACAAATCCCACCCACAGTGG  80
Macaca  GACGAGCGAGGTGATAGAAGACGAGAAGCAATTCTATTCCAAGGCCAAGACCTACTGGAAACAAATCCCACCCACGGTGG  80
Bos     GACGAGCGAGGTGATCGAGGACGAGAAGCAGTTCTACTCCAAGGCCAAGACGTACTGGAAGGAGGTCCCTGCCACCGTGG  80
Canis   GACAAGTGAGGTGATAGAAGATGAGAAACAGTTCTATTCAAAGGCCAAGACGTACTGGAAGGAAGTCCCGCCCACAGTGG  80

Homo    ACGGCATGCTTGGGGGGTATGGCCACATCTCCAGCATCGACATCAACAGCTCCCGGAAGTTTCTGCAGAGGTTTTTGAGG  160
Pan     ACGGCATGCTTGGGGGGTATGGCCACATCTCCAGCATCGACATCAACAGCTCCCGGAAGTTTCTGCAGAGGTTTTTGAGG  160
Macaca  ACGGCATGCTTGGGGGGTATGGCCACATCTCCAGCATCGACATCAACAGCTCCCGGAAGTTTCTGCAGAGGTTTTTGAGG  160
Bos     ACGGCATGCTCGGGGGGTATGGCCACATCTCCAGCATCGACATCAACAGCTCCCGGAAGTTCCTGCAGAGGTTTCTGAGG  160
Canis   ACGGCATGCTCGGGGGGTATGGCCACATCTCCAGCATCGACATCAGCAGCTCCCGGAAGTTCCTGCAGAGGTTTTTGAGG  160
```

ENSG00000148335 exon 2

```
Homo    GAAGGCCCGAACAAGACAGGAACGTCCTGTGCCCTGGACTGTGGAGCTGGCATTGGGAGGATCACCAAGCGGCTGCTCCT  80
Pan     GAAGGCCCGAACAAGACAGGAACGTCCTGTGCCCTGGACTGTGGAGCTGGCATTGGCAGGATCACCAAGCGGCTGCTCCT  80
Macaca  GAAGGCCCGAACAAGACAGGAACGTCCTGTGCCCTGGACTGTGGAGCTGGCATTGGGAGGATCACCAAGCGGCTGCTCCT  80
Bos     GAAGGCCCGAACAAGACGGGGACCTCATACGCCCTGGACTGCGGAGCCGGCATTGGGAGGATCACCAAGCGGCTGCTCTT  80
Canis   GAAGGCCCAAACAAGACGGGCACCTCCTGTGCCCTGGACTGCGGAGCCGGCATAGGCAGGATCACCAAGAGGCTGCTGCT  80

Homo    GCCGCTGTTCAGAGAGGTGGATATGGTCGACATAACGGAGGACTTCCTGGTTCAAGCCAAGACCTACCTGGGGGAGGAGG  160
Pan     GCCGCTGTTCAGAGAGGTGGATATGGTCGACATAACGGAGGACTTCCTGGTTCAAGCCAAGACCTACCTGGGGGAGGAGG  160
Macaca  GCCGCTGTTCAGAGAGGTGGATATGGTCGACATAACGGAGGACTTCCTGGTTCAAGCCAAGACCTACCTGGGGGAGGAGG  160
Bos     GCCTCTCTTTGGAGTGGTGGACATGGTGGATGTAACAGAGGACTTCCTGGTCAAGGCTAAGACCTACCTGGGCGAGGAGG  160
Canis   GCCGCTCTTTGGAGTGGTAGACATGGTGGACGTGACAGAAGATTTTCTGGTTAAAGCCAAGACGTATCTGGGGGAAGAGG  160
```

ENSG00000148335 intron 1

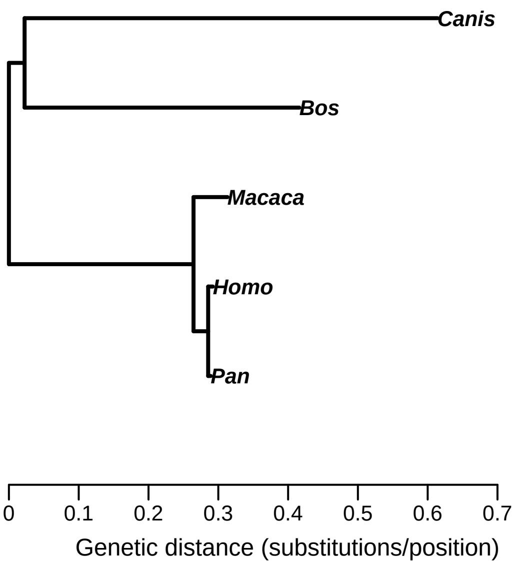

ENSG00000148335 intron 1

```
Homo    GTAGGCAGGTCTGGCGTGCTCTCCAGG---AGAGGCTGTGGCTCTGGTGGCACTGCCGAGTCCATGTGGGGTGCCACCTTTTACACATGTAACACTGCAG 97
Pan     GTAGGCAGGTCTGGCGTGCTCTCCAGG---AGAGGCTGTGGCTCTGGTGGCACTGCCGAGTCCATGTGGGGTGCCACCTTTTACACATGTAACACTGCAG 97
Macaca  GTAGGCAGGTCTGGCGTGCTCTCCAGG---AGAGGCTGTGGCTCTGGTGGCACTGCCGAGTCCATGTGGGGTGCCACCTTTT--ACATGTAACACTGCAG 95
Bos     GTAGGCAGGTCTGGTGTGCTCTCCAGG---AGGA-----------------------GAGCCCACGT--------------------------------- 41
Canis   GTAGGCAGGTCTGGTGTGCTGTCCAGGAGCAGAGGATGTGGCCCTGGTGGCCCTCCCTGTTCTGCACGGGGCGCCACTTTTC--ACACGGATCACCGCAG 98

Homo    GATTGTTGGCTATCTCTTGGTACCTACC-CCAAAGCCTTGAGGAGCTGGGCTGGGTGGCACAGTGGGGTAACTTCTAGATTGTGGACTTAGCCTCAGGG 196
Pan     GGTCATTGGCTATCTCTTGGTACCTACC-CCAAAGCCTTGAGGAGCTGGGCTGGGTGGCACAGTGGGGTAACTTCTAGATTGTGGACTTAGCCTCAAGG 196
Macaca  GCTCGTTGGCTGTCTCTTGGTACCTACC-CCAAAGCCTTGAGGAGCTGGGCTGGGTGGCACAGTGGGGTAACTTCTAGATTGTGGACTTAGCCTCAAGG 194
Bos     ----------------------CCACCA-CCCAGGCCTTGACGAGC-GGGTGGGGCGGG----------------CCAGCTCGTAGGCTTTGCCGCAAGG 101
Canis   GGTTTTTGGTTATCTGTTGGCGTCGACCACCCAGGCCTCGAAGGCTGGGGCGGGGGCAGCA--------------TGTGACTGGGGCCTCTGTCTCAGGG 184

Homo    TTACCAGACAGGAGCTGGCTGTTAGGCAGAA---TTGAGGTGCCTGAATTGGGCAAACCTCTCTTCAACTGTTGGGTTTTGCCAGGGAC-GAAACCCTTG 292
Pan     TTACCAGACAGGAGCTGGCTGTTAGGCAGAA---TTGAGGTGCCTGAATTGGGCAAACTTCTCTTCAACTGTTGGGTTTTGCCAGGGAC-GAAACCCTTG 292
Macaca  TTACCAGACAGGAGCTGGCTGTTAGGCAGAA---CTGAGGTGCCCGAATTGGGCAAACTTCTCTTCAACTGTTGGGTTTTGCCAGGGAC-AAAACCCTTG 290
Bos     TCCCCAGCCAGGAGCCGGCTGTCATGCTGGGGCCTCCTGGTGCCGGAACAGAGCAGCTTTCTCGCAGACTGTTCCATTTTTCCAGAGGCGGAAACCTTTG 201
Canis   TTGTCAGATGGGAGCTGGTCACTGTGCGAGGGT-TTCCGGCACCAGCACGGAG----TCTCACTTTAACCGTTCCGTTTTGTCTGGATT-GAATCCTTCA 278

Homo    CTCTCCTGCCATGTCCACGGGTGTTTGGACTGAGGGTGTTGGCTA---TTCTGGACGAAGACTCCTAGTTATGTGCCATGGTTTCCACCCTCTGCTCCAC 389
Pan     CTCTCCTGCCATGTCCATGGGTGTTTGGACTGAGGATGTTGGCTA---TTCTGGACGAAGACTCCTAGTTACGTGCCATGGTTTCCACCCTCTGCTCCAC 389
Macaca  CTCTCCTGCCATGCCCATGGGTGTTTGGGCTGAGGGTGTTGGCCA---TTCTGGATGAAAACTCCTAGTTACATGCCATGGTTTCTACCCTCTGCTCCAC 387
Bos     CTCTCTTGCCATGTTTGCAAATATCTGGGCTGTGGGTGTTGGCTGGTCTGCTGAACACAGACTCCCACTTTCTTGTCCTGCCACCGCCTCTGTGC----- 296
Canis   CTCTCTTGCCATGTTTGCGGGCTGTTGGGCTGTGGGTGCTGGCTGTTCTGCTGAAGGCCGGCTCCTAGTTGTTCACCTGCCCTGCCCTCCTCTGTGC--- 375

Homo    CCCCAGCCTTTTCTGTACCAGGCTGGGACCCCCAGTCCTGGAGCCCCCCACCTTCTTGTCTCATGGCTGGCACTGGAGGCTGCTGTTGGCCACCTGCTCC 489
Pan     TCCCAGCCTTTTCTGTACCAGGCTGGGACCCCCAGTCCTGGAGCCCCCCACCTTCTTGTCTCATGGCTGGCACTGGAGGCTGCTGTTGGCCACCTGCTCC 489
Macaca  CC------------------------------------------------NNNNNNNNNNNNNNNNNNNNNNNNNNNNNNNNNNNNNNNNNNNNNNNNNN 439
Bos     ------------------CAGGCTGGCAGCGCTAGGGCCAGAGCCTCCCCAGGCCCGCCTCCCTGCCTCTCCCTTGGCACACACCGTGGCTGCTGTTGC- 377
Canis   ------------------CAGGCTGGCGTGTGCCCAGCTGTGGCCTCCCCTGGTGAGCGTCAGAGGCTGGTGCTTGCTCCTCATTCTCT----------- 446

Homo    ATCCAGCGCCTCCCCTCCCTCCATGTTCCCTCTCTGCACCCTTGTTCACCTGTTGCCCCGCTTCCATTCTCCCCATTCCTCTCTGCCGTTGTTTACCTGG 589
Pan     ATCCAGCGCCTCCCCTCCCTCCATGTTCCCTCTCTGCACCCTTGTTCACCCGTTGCCCCGCTTCCATTCTCCCCATTCCTCTCTGCCGTTGTTTACCTGG 589
Macaca  NNNNNNCGCCTCCCCTCCCTCCGTGTTCCCTCTCCGCACCCTTGTTCACCCATTGTCCTACTTCCATTCTCCCTGCTCCTCTCTGCCGTTGTTTACTTGG 539
Bos     --------CCCCTGCTCCACCCGCGTGCCAGCTCCCAGGTGTGCTCACCGAGTGCCCTCTTCCTTCTTTCACTGCTTCTCTCCACGGC------CCTTG 463
Canis   --------GCTCCTCTCTGCCCACTTTCCGTCTTC-------CATTCTTTCACTGCTTAAATTTTTTCCATCCTGTTGTTTAACGGGG------CTCTG 525

Homo    AGACTCAGGAGAGATTAGTGCACCTGTTCAAGTTA-TCACCTTTAAGTGGAAATTAGCTTTCA-TTTAGAAATGTGTGGCCCAGGGCAGGGGCAGTGGCT 687
Pan     AGACTCGGGAGAGATTAGTGCACCTGTTCAAGTTA-TCACCTTTAAGTGGAAATTAGCTTTCA-TTTAGAAATGTGTGGCCCAGGGCAGGGGCAGTGGCT 687
Macaca  AGACTCAGGAGAGATTAGCGTACCTGTTCAAGTTA-TCACCTTTAAGTGGAAATTGGCTTTCATTTTAGAAATGTGTGGCCCAGGGCTGGGGCAGCAGCT 638
Bos     TGAGCGAGAGGAGATGAGTGCATCTAGTCAAGTTA-ACACCTTTAAGTGGAGGTTGGCTTTCATCTCAGGAACATGTGACCCAGA--------------- 547
Canis   GGAGTGAGAGGAGATCAATGCGCCTATTCAAATTACCCATCTTTAAGTGGAAGTCGGCTTTTT------AGACAGGTGGCCCAGG---------------- 604

Homo    CACTCCTGTCATCCCAGCCCCTTGGGATGCCAAGGTGAGAGGATTGCTTGAGCCCAGGAGTTTGAGACCAGCCTGGGCAACAGACCGAGGCCCCGTCTCT 787
Pan     CACTCCTGTCATCCCAGCCCCTTGGGATGCCAAGGTGAGAGGATTGCTTGAGCCCAGGAGTTTGAGACCAGCCTGGGCAACAGACCGAGGCCCCGTCTCT 787
Macaca  CACTCCTGTAAT-CCAGCTCTTTGGGATTTCAAGGTGGGAGGACTGCTCGAGCCCAGGAATTTGAGACCAGCCTGGGCAACAGACTGAGGCCCTGTCTCT 737
Bos     ---------------------------------------------------------------------------------------------------- 547
Canis   ---------------------------------------------------------------------------------------------------- 604

Homo    ACAAAAAATAC-AAAAAAAAAAAAATGAGTTGGGCATGGTGGCGCATGCCTGTAGTCCCAGCTACTTGGGAGGCTGAGGTGGGAAGATGGAGGATGGCTT 886
Pan     ACAAAAAATACAAAAAAAAAAAAAATGAGTTGGGCATGGTGGCGCATGCCTGTAGTCCCAGCTACTTGGGAGGCTGAGGTGGGAAGATGGAGGATGGCTT 887
Macaca  ACAAAAAAT----AAATAAAAATAAAGAGTTGGGCATGGTGGCACATGCCTGTAGTCCCAGCTACGTGGGAGGCTGAGGCGGGAAGATGGAGGATGGCTT 833
Bos     ---------------------------------------------------------------------------------------------------- 547
Canis   ---------------------------------------------------------------------------------------------------- 604

Homo    GAGCCCAGGAGTTCGAGGCTACAGTGAGCCATGATCCCACCACTGCATTCCAACCTGAACAGAATGCAGAATGCAACCT-----------GAACTTTCAA 975
Pan     GAGCCCAGGAGTTCGAGGCTACAGTGAGCCATGATCCCACCACTGCATTCCAACCTGAACAGAATGCAGAATGCAACCT-----------GAACTTTCAA 976
Macaca  GAGCCCAGGGGTCCGAGGCTACAGTGAGCCGTGATCCCACTACTGCATTCCAGCCTGGACA----ACAGAATGAGATCCTGTCTCTACAAAAACTTTCAA 929
Bos     ---------------------------------------------------------------------------------------------------- 547
Canis   ---------------------------------------------------------------------------------------------------- 604

Homo    AGATAAAGGAATGTGTGGCCCAGCAGTGTGTCCTCCCATGCTCATCCCCTC-------GACTCCGTACAAGCTGGCAGCCAGCACAGACCTCCCCACCAG 1068
Pan     AGATAAAGGAATGTGTGGCCCAGCAGTGTGTCCT-CCATGCTCATCCCCTC-------GACTCCGTACAAGCTGGCAGCCAGCACAGACCTCCCCACCAG 1068
Macaca  AGATAAAGGAATATGTGGCCCAGCAGGGTGTCCTTCCACGCTTACCCCCTTGAATCAGGACTCTGTACAAGCTGGCAGCCAGCACAGACCTCCCCACCAG 1029
Bos     ----AAACGGCTGTG--------------------CTGTGCTCACGCCC-------------------------ACCGCCGGCGC---CACCCACGCCAG 595
Canis   ----ACAGGCACAAG--------------------GTGTGCTCCTGCCC----------------------------------ACTGGCGACCCCACAGC 646

Homo    TGCTCAGCTTGTGCGGAGTCCTGGAATCCTGACTTGAGTCTAAGTCCTAGGGCTCGTGAGGAAGGG-CCGCACGTGCGGTGACAGGGTCCCTCTGA---- 1163
Pan     TGCTCAGCTTGTGCGGAGTCCTGGAATCCTGACTTGAGTCTAAGTCCTAGGGCTCGTGAGGAAGGG-CCCCACGTGCGGTGACAGGGTCCCTCTGA---- 1163
Macaca  TGCTCAGCTTGTGCAGAGCCCTGGAATCCTGACTTGAGTCTAAGTCCCAGGGCTCATGAGGAAGGGCCCCCACGTGCGGCGACAGGGTCTCTCTGA---- 1125
Bos     CCACCAGCCTGTGCTGATCTGAGTAGTTCTG-----------------GGGGCTCACAAGGAAAGGCCCT------TTGCAGAGGGGTCCCTCTGGC-TT 671
Canis   CTACATGCCTGGTCCTACTGGAGTGACCTTG-----------------GGAGCGTGGGAGGAACAGCCCTTCC---TAGGGACAGGGTCCCTCGGGCTTT 726

Homo    --------------TAGGTTATATGC---CTCTGCTTTTCAG 1188
Pan     --------------TAGGTTATATGC---CTCTGCTTTTCAG 1188
Macaca  --------------TAGGTTACATGC---CTCTGCTTTTCAG 1150
Bos     CTAACCCTTCTCTTCAGTCTGTCTTC---TCTGCTGCTTCAG 710
Canis   TTGAGCCTTCCTTACGGAGTCTGTGCCTTCTCTGCTTCTTAG 768
```

ENSG00000091140 intron 9

Description: Dihydrolipoyl dehydrogenase, mitochondrial precursor (DLD)
Intron number: 9
Human chromosome: 07
Intron start (bp): 107343378
Human intron length : 1097
Intron alignment length: 1152
Flanking exons length (upstream/downstream): 191/171
SNP density: 0.004558
K tree score: 0.0569
Scaling factor: 0.8444
Human-chimpanzee distance: 0.005502
Total primate branch length: 0.0892

ENSG00000091140 exon 9

```
Homo    TGACAGCAGTTGAATTTTTAGGTCATGTAGGTGGAGTTGGAATTGATATGGAGATATCTAAAAACTTTCAACGCATCCTT  80
Pan     TGACAGCAGTTGAATTTTTAGGTCATGTAGGTGGAGTTGGAATTGATATGGAGATATCTAAAAACTTTCAACGCATCCTT  80
Macaca  TGACAGCAGTTGAATTTTTAGGTCATGTAGGTGGAGTTGGAATTGATATGGAGATATCTAAAAACTTTCAACGCATCCTT  80
Bos     TGACAGCAGTTGAGTTTTTGGGTCATGTTGGTGGTGTTGGAATTGATATGGAAATATCTAAAAACTTTCAACGTATCCTT  80
Canis   TGACAGCAGTTGAGTTTTTAGGTCATGTTGGTGGAGTCGGAATTGATATGGAGATATCTAAAAACTTTCAACGCATCCTC  80

Homo    CAAAAACAGGGGTTTAAATTTAAATTGAATACAAAGGTTACTGGTGCTACCAAGAAGTCAGATGGAAAAATTGATGTTTC  160
Pan     CAAAAACAGGGGTTTAAATTTAAATTGAATACAAAGGTTACTGGTGCTACCAAGAAGTCAGATGGAAAAATTGATGTTTC  160
Macaca  CAAAAACAGGGGTTTAAATTTAAATTGAATACAAAGGTTACTGGTGCTACCAAGAAGTCAGATGGAAAAATTGATGTTTC  160
Bos     CAAAAACAAGGATTTAAATTTAAATTAAACACAAAAGTTACTGGTGCTACTAAGAAATCAGATGGAAAAATTGATGTTTC  160
Canis   CAAAAACAAGGATTTAAATTTAAATTGAATACAAAAGTTACTGGTGCTACCAAGAAGTCAGATGGAAAAATTGATGTTTT  160
```

ENSG00000091140 exon 10

```
Homo    TATTGAAGCTGCTTCTGGTGGTAAAGCTGAAGTTATCACTTGTGATGTACTCTTGGTTTGCATTGGCCGACGACCCTTTA  80
Pan     TATTGAAGCTGCTTCTGGTGGTAAAGCTGAAGTTATCACTTGTGATGTACTCTTGGTTTGCATTGGCCGACGACCCTTTA  80
Macaca  TATTGAAGCTGCTTCTGGTGGTAAAGCTGAAGTTATCACTTGTGATGTACTCTTGGTTTGCATTGGCCGACGACCCTTTA  80
Bos     TATTGAGGCTGCTTCTGGTGGTAAAGCTGAAGTTATCACTTGTGATGTACTGCTGGTTTGCATCGGCCGACGACCCTTTA  80
Canis   TATTGAAGGTGCTTCTGGTGGCAAAGCCGAGGTTATCACATGTGACGTACTCTTGGTTTGCATTGGCCGACGACCCTTTA  80

Homo    CTAAGAATTTGGGACTAGAAGAGCTGGGAATTGAACTAGATCCCAGAGGTAGAATTCCAGTCAATACCAGATTTCAAACT  160
Pan     CTAAGAATTTGGGACTAGAAGAGCTGGGAATTGAACTAGATCCCAGAGGTAGAATTCCAGTCAATACCAGATTTCAAACT  160
Macaca  CTAAGAATTTGGGACTAGAAGAGCTGGGAATTGAACTAGATCCCAGAGGTAGAATTCCAGTTAATACCAGATTTCAAACT  160
Bos     CTCAGAATTTGGGACTAGAAGAGCTTGGAATTGAGCTGGATACTAGAGGTAGAATTCCAGTGAATACCAGATTCCAAACT  160
Canis   CTCAGAACTTGGGACTAGAAGAGCTTGGAATTGAACTAGATCCTAGAGGTAGAATTCCAGTCAATACCAGATTCCAAACT  160
```

ENSG00000091140 intron 9

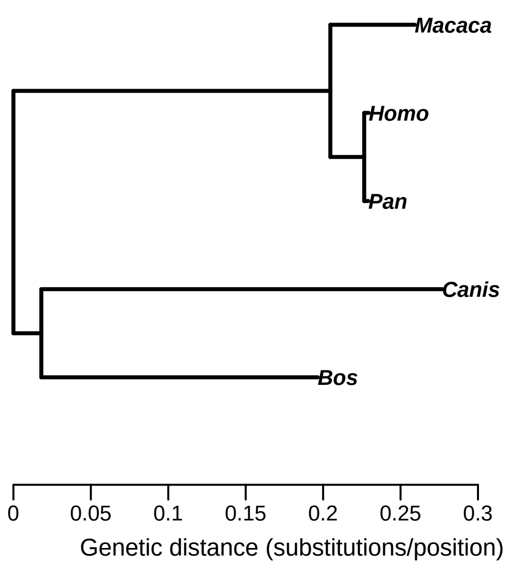

ENSG00000091140 intron 9

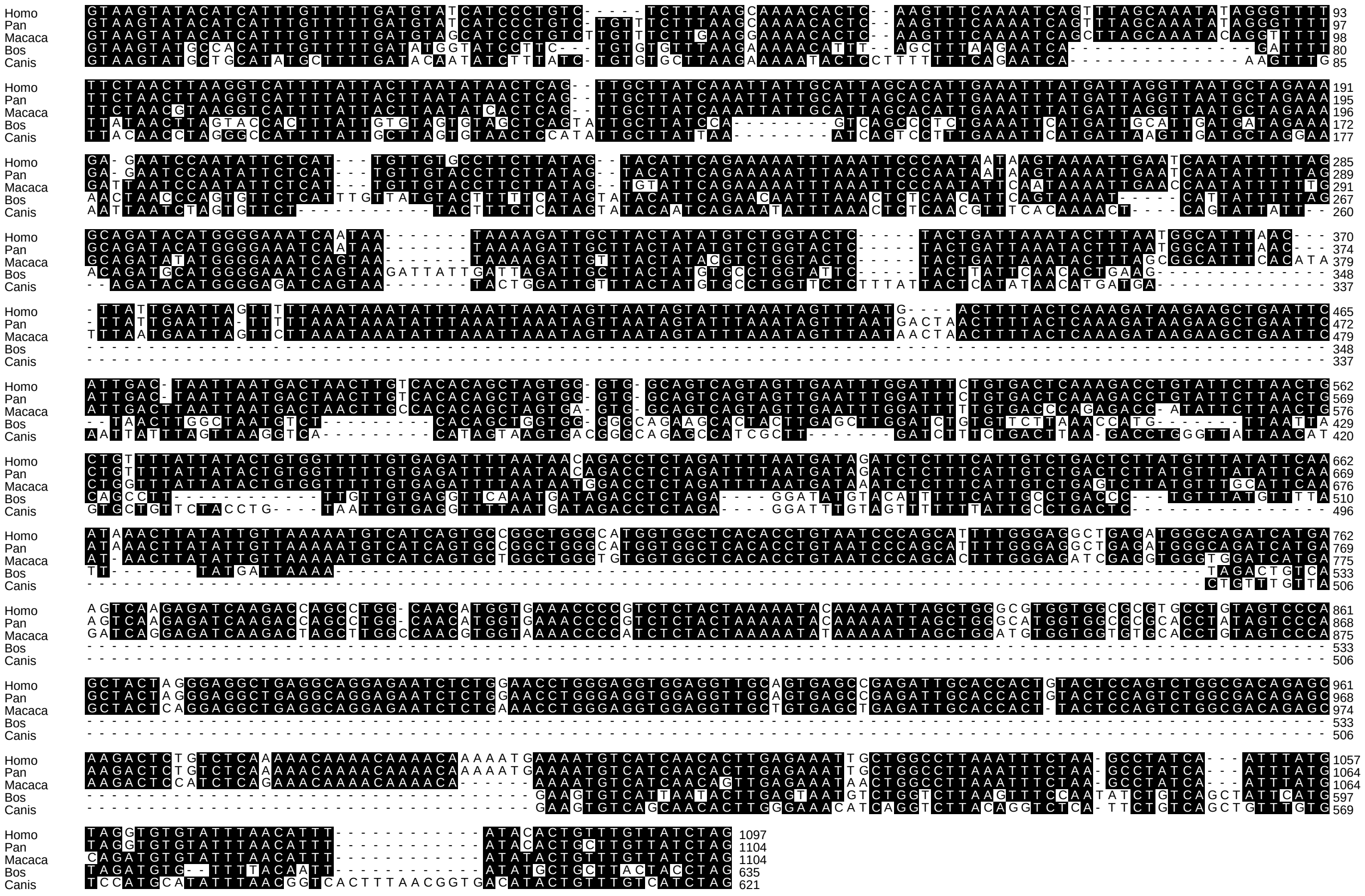

ENSG00000179636 intron 1

Description: Protein p25-beta (TPPP2)
Intron number: 1
Human chromosome: 14
Intron start (bp): 20568754
Human intron length : 257
Intron alignment length: 259
Flanking exons length (upstream/downstream): 173/154
SNP density: 0.007782
K tree score: 0.0837
Scaling factor: 0.7639
Human-chimpanzee distance: 0.019697
Total primate branch length: 0.0892

ENSG00000179636 exon 1

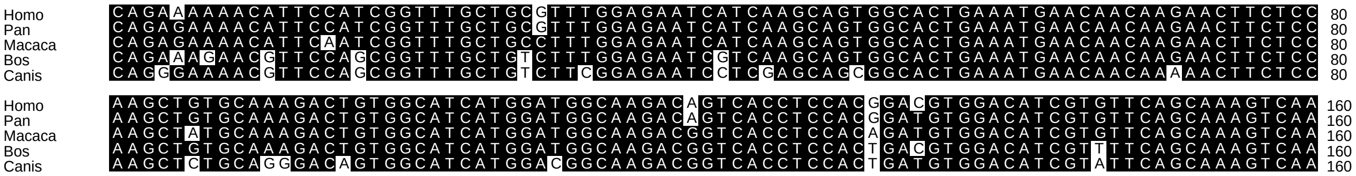

ENSG00000179636 exon 2

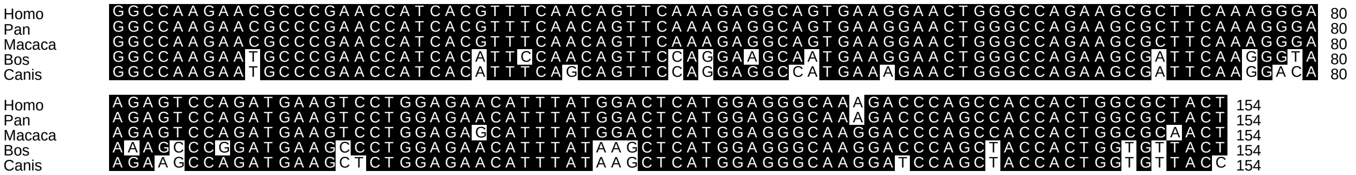

ENSG00000179636 intron 1

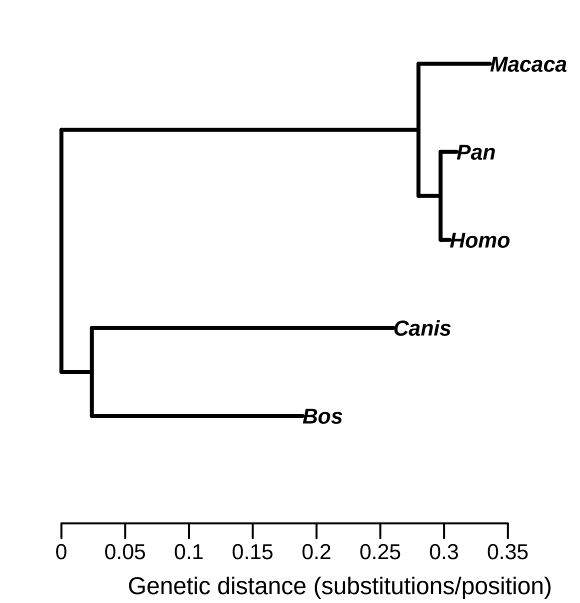

ENSG00000179636 intron 1

```
Homo    GTGAGGAGCCAAAAA-TATGGAGGTGGGGGTGAGAAGAACCTGCGAGGTAGTTGGCCACGGAAGGGTTCACGTGGTGGAACATTTGCAGTGGGCCTACCA 99
Pan     GTGAGGAGCCAAAAA-TATGGAGGTGGGGGTGAGAAGAACCTGCGAGGTAGTTGGCCACGGAAGGGTTCATGTGGTGGAACATTTGCAGTGGGCCTACCA 99
Macaca  GTGAGGACCCAAAGA-TGTGGGGGTGGGGGTGAGAAGAAAGTGCGAGGTAGTTGGCCATGGAAGGGTTCATGTGGCGGAACATTTGCAGTGGGCCTACCA 99
Bos     GTAAGGGACCACAGACGGGTGTGGGGGTGGTGAGTAAAACAAGCAAGGTCATGGGATGTGGCAGGGTTTCCGCAGTAGAACATTTGTAACAGGTCTCCTG 100
Canis   GTAAGGGACCAGAGA-GGAGGTAGGGGTGGTGAGTAGACCGAGCAAGGCAGCGGGCTATGGAAGGGTTCGTGTGGC-GAGCATGCATAATGGGTCCACTG 98

Homo    AGCACC-ACAGGGTACCTGGCGCTGTGCTATCCTTTGTCTTCGAGACACTCACTGATTGATAGGACAAAAGAGGCCAAATCTGAGTGAGGCAAGAGTTGG 198
Pan     AGCACC-ACAGGGTACCTGGCGCTGTGCTATTCTTTGTCTTCAAGACACTCACTGATTGATAGGACAAAAGAGGCCAAATCTGAGTGATGCAAGAGTTGG 198
Macaca  AGCACC-ACAGGGTACCTGGCACTGTGCTATTCTTTGTCATTGAGACACTTAC----TGATAGGACAAAAGAGGACAAATCTGAGTGAGGTAAGAGTTGG 194
Bos     AGCACCTACTGGGTGCCTGGCTC--TGCTAGGCCCTGACCTCAAGACATTCAGTGTAT-----------AGAGGCCCCGTCTGAGTAAGAGGAGGGCTGG 187
Canis   GGCACCTGCTGGGCCCATGGCCCTGTGCTAGTCCCTGTCCCCAGGACACTCAGTGT--------------------------------CAAGAGGGGTGG 166

Homo    ACCTTGGAAGGTAATGCATTCATGCTCCAAAGTTTCTGGATT--TCTGTCTAACTGACCAG 257
Pan     ACCTTGGAAGGTAGTGCATTCATGCTCCAAAGTTTCTGGATT--TCTGTCTAACTGACCAG 257
Macaca  ACCTTGGAAGGTAATGCATTTGTGCTCCAAAGTTTCTGGATT--TCTGTCTAACTGACCAG 253
Bos     ACCTTGGAAAGCAATGACTTCATGTTCCCCAGTTCCTGGATT--TCTGTCTTACTGACCAG 246
Canis   ACCTTGGAAGGGGAAGAGTTCATGTTCCAGAGCTCCTGGGTTTCTCTGTCCGACTGACCAG 227
```

ENSG00000149295 intron 3

Description: D(2) dopamine receptor (DRD2)
Intron number: 3
Human chromosome: 11
Intron start (bp): 112791544
Human intron length : 1251
Intron alignment length: 1326
Flanking exons length (upstream/downstream): 137/191
SNP density: 0.006395
K tree score: 0.089
Scaling factor: 0.7615
Human-chimpanzee distance: 0.008911
Total primate branch length: 0.0889

ENSG00000149295 exon 3

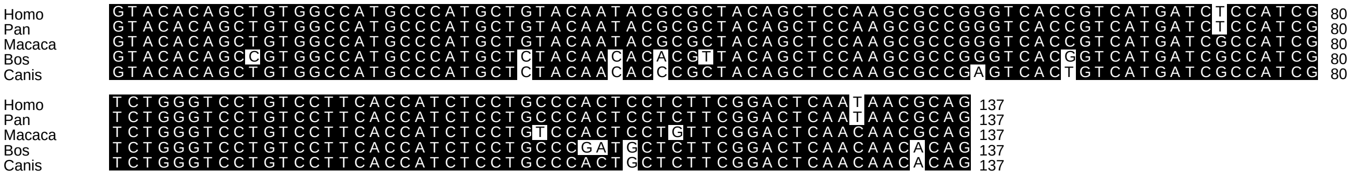

ENSG00000149295 exon 4

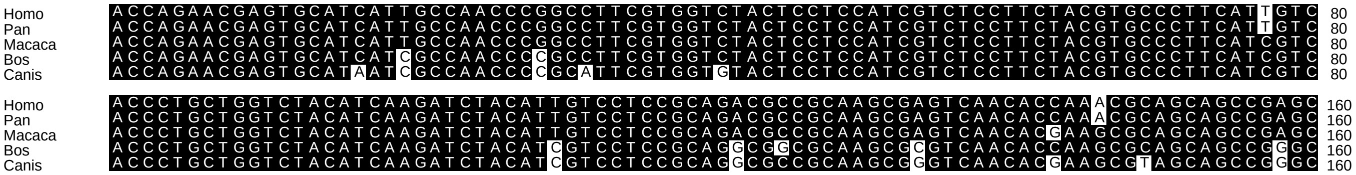

ENSG00000149295 intron 3

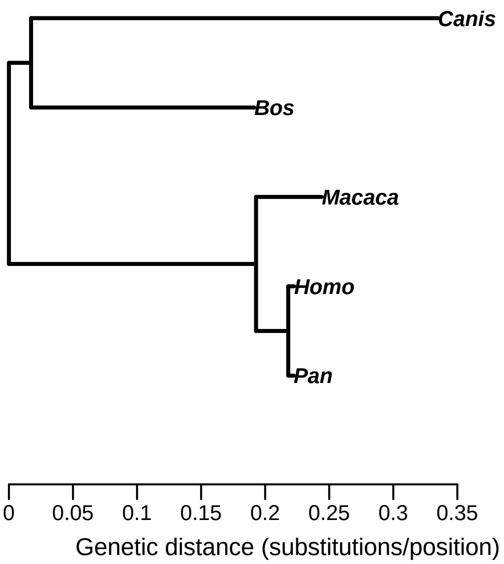

ENSG00000149295 intron 3

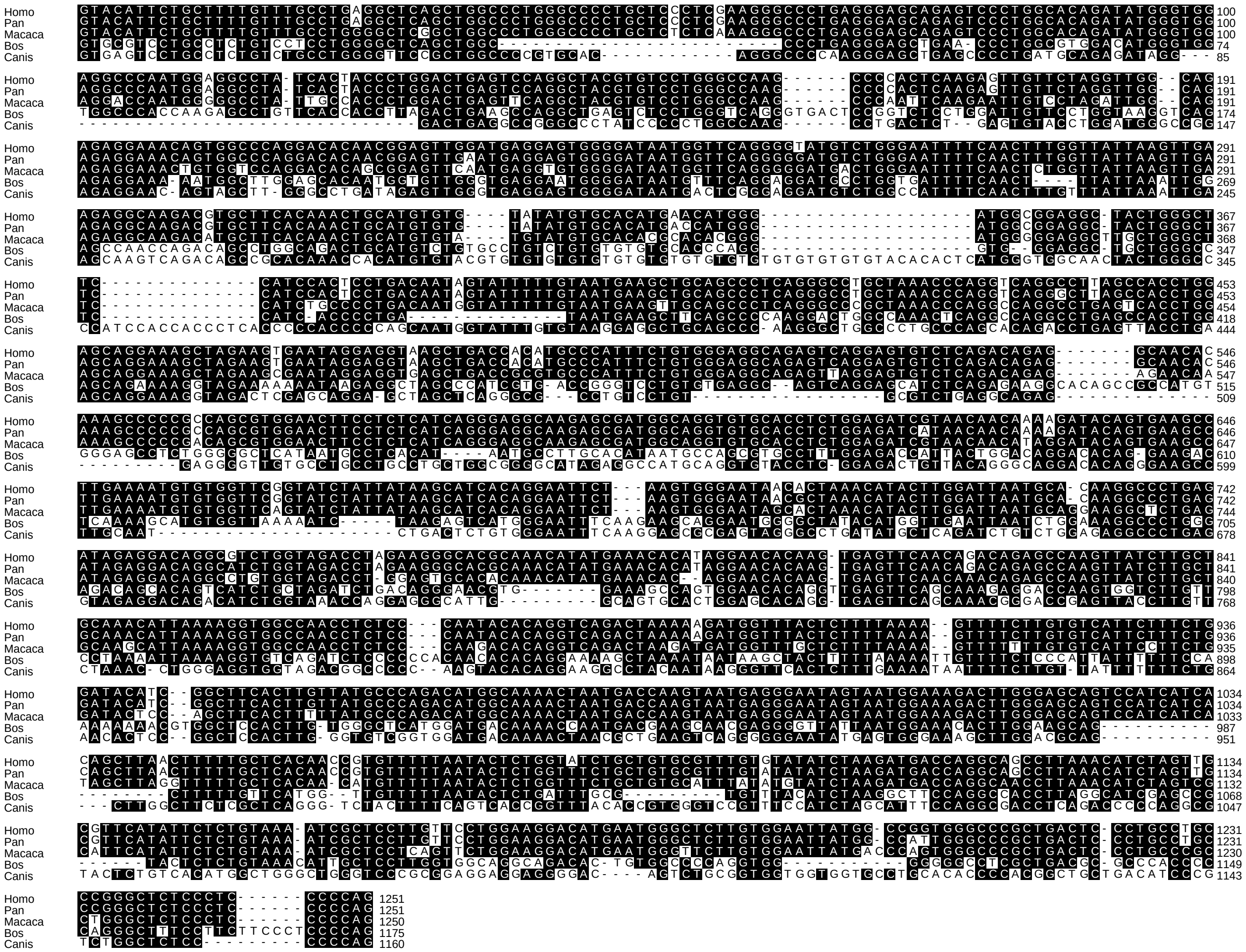

ENSG00000155542 intron 2

Description: Unknown (C5orf35)
Intron number: 2
Human chromosome: 05
Intron start (bp): 56243121
Human intron length : 1474
Intron alignment length: 2479
Flanking exons length (upstream/downstream): 368/124
SNP density: 0.003392
K tree score: 0.0394
Scaling factor: 1.0979
Human-chimpanzee distance: 0.006847
Total primate branch length: 0.0888

ENSG00000155542 exon 2

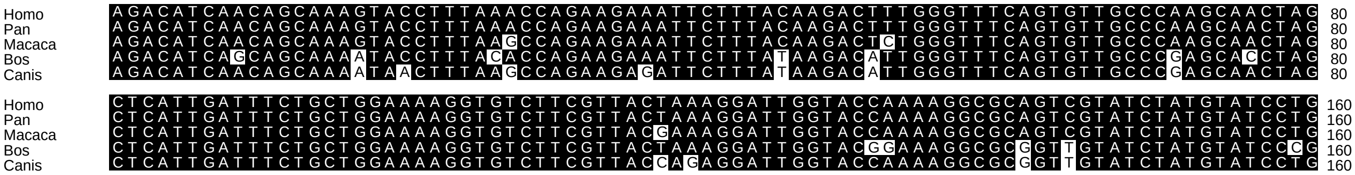

ENSG00000155542 exon 3

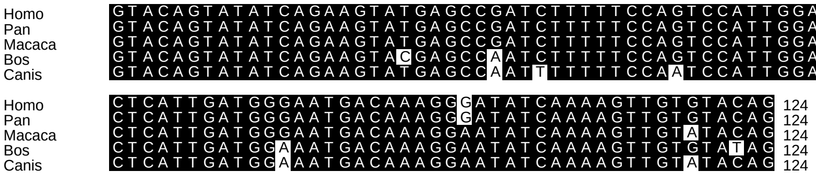

ENSG00000155542 intron 2

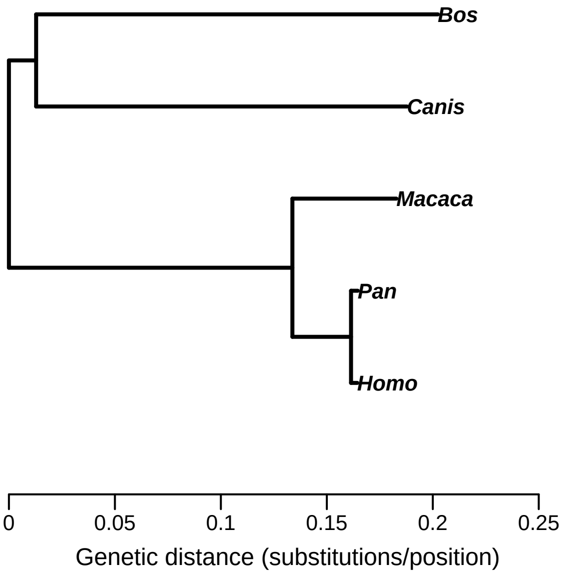

ENSG00000155542 intron 2

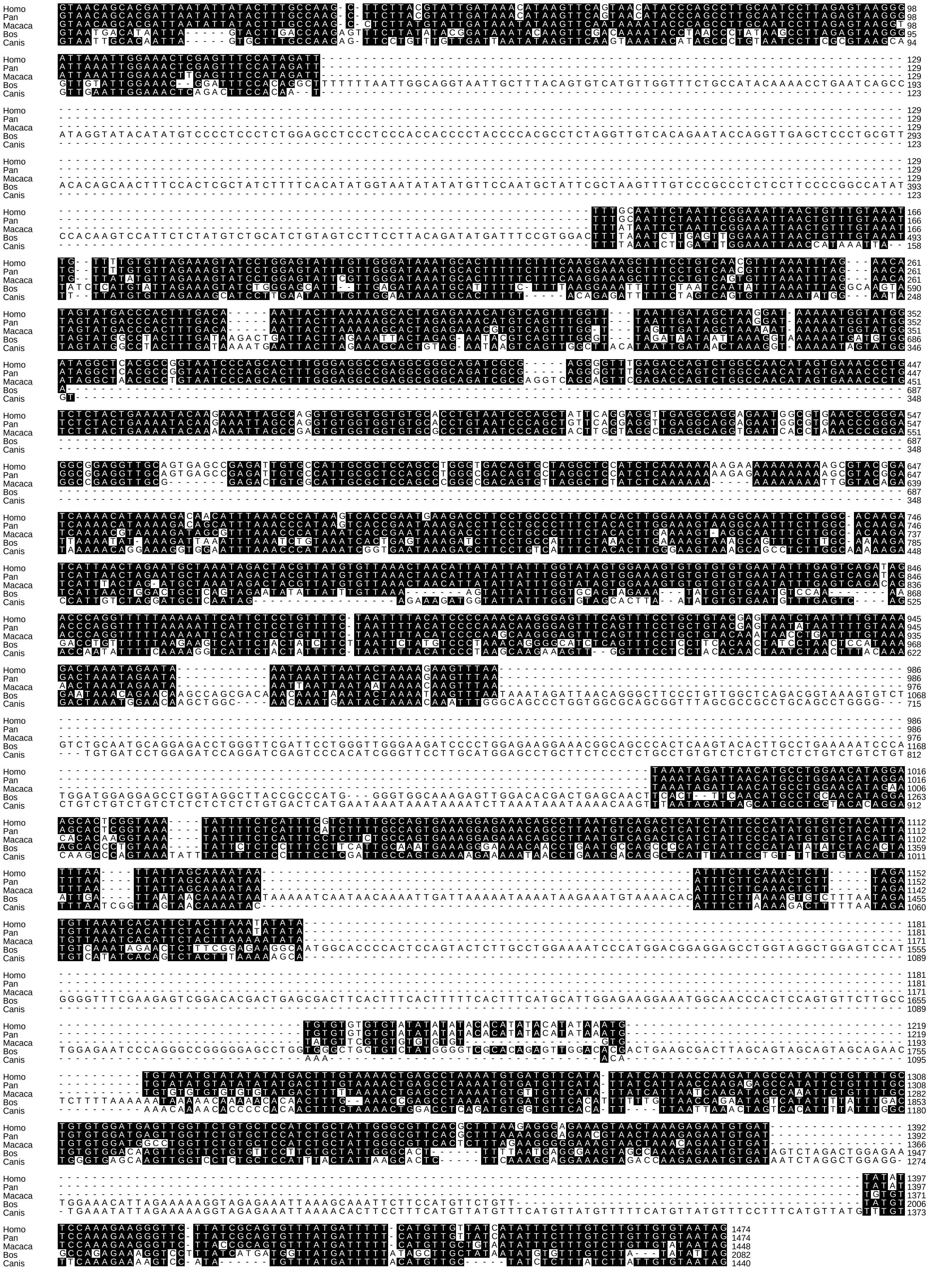

ENSG00000185674 intron 2

Description: Lysozyme g-like protein 2 precursor (LYG2)
Intron number: 2
Human chromosome: 02
Intron start (bp): 99228354
Human intron length : 1221
Intron alignment length: 1285
Flanking exons length (upstream/downstream): 141/197
SNP density: 0.003276
K tree score: 0.1
Scaling factor: 0.9219
Human-chimpanzee distance: 0.016665
Total primate branch length: 0.0882

ENSG00000185674 exon 2

```
Homo    GCACTTCCAGGGGCTCATACCCCTTCAGTCACTCAATGAAGCCTCACCTACATCCACGCCTGTACCACGGCTGCTATGGG 80
Pan     GCACTTCCAGGGGCTCGTACCCCTTCACTCACTCAATGAAGCCTCACCTACATCCACGCCTGTACCATGGCTGCTACGGG 80
Macaca  GCACTTCCAGGGGCTCGTACCCCTTCACCCACTCAATGAACCCTCACCTACATCCGCGCCTGTACCACGGCTGCTACGGG 80
Bos     GCACTTCTAGGGGCTCACACTCCTTCACTCACACAATGAGTCCTCGCCTGCACTTCCGCCTGTACCATGGCTGCTACGGA 80
Canis   GCACTTCTAGGGGCTCGTACCCTTTCACTCACTCAATGAAGCCCCACCTGCATCCCCGCCTATACCATGGCTGCTATGGT 80

Homo    GACATCATGACCATGAAGACCTCTGGGGCCACTTGTGATGCAAACAGTGTGATGAACTGCG 141
Pan     GACATCATGACCATGAAGACCTCTGGGGCCACTTGTGATGCAAACAGTGTGATGAACTGCG 141
Macaca  GACATCATGACCATGAAGACCTCTGGGGCCACTTGTGATGCAAAGAGTGTGATGAACTGCG 141
Bos     GACGTCATGATCATGGAGACCCCTGGTATCCCCTGTGATAACACCAGGATGATTGCCTGTG 141
Canis   GACATCATGACCATGGAGACCTCTGGTGCCGCCTGTGATATAACCAGGTTGATGAACTGTG 141
```

ENSG00000185674 exon 3

```
Homo    GGATCCGTGGTTCTGAAATGTTTGCTGAGATGGATTTGAGGGCCATAAAACCTTACCAGACTCTGATCAAAGAAGTCGGG 80
Pan     GGATCCGTGGTTCTGAAATGTTTGCTGAGATGGATTTGAGGGCCATAAAACCTTACCAGACTCTGATCAAAGAAGTCGGG 80
Macaca  GGATCCGTGGTTCTGAAATGTTTGCTGAGATGGATCTGAGGGCCATAAAACCCTACCAGACTCTGATCAAAGAAGTCGGG 80
Bos     GCATCCGTGGTTCTGAGATGTTTGCTGAGATGGATTTGAAGGCCTTACAGTCTTATCAAATTCTGATCAAAGAAATTGGA 80
Canis   GGATCCGTGGTTCTGAAATGTTTGCTGAGATGGATTTGAGAGCCTTAAAGACTTACCAGATTCTGATCAAAGAAGTTGGG 80

Homo    CAGAGACATTGCGTGGACCCTGCTGTCATCGCAGCCATCATCTCCAGGGAAAGCCATGGCGGATCTGTCCTGCAAGACGG 160
Pan     CAGAGACATTGCGTGGACCCTGCTGTCATCGCAGCCATCATCTCCAGGGAAAGCCATGGCGGATCTGTCCTGCAAGACGG 160
Macaca  CAGAGATATTGCGTGGACCCTGCTGTCATTGCAGCCATCATCTCCAGGGAAAGTCATGGCGGATCTGTCCTGCAAGATGG 160
Bos     CTGCGGCACTGCGTGGACCCTGCTCTCATTGCAGCCATCATCTCTAGAGAAAGCCACGGTGGAACCATCCTGCTGGATGG 160
Canis   CTGAGGCATTGTGTAGACCCTGCACTCATTGCTGCCATCATCTCCAGAGAAAGCCATGGTGGAGCCGTCCTGAAAGATGG 160
```

ENSG00000185674 intron 2

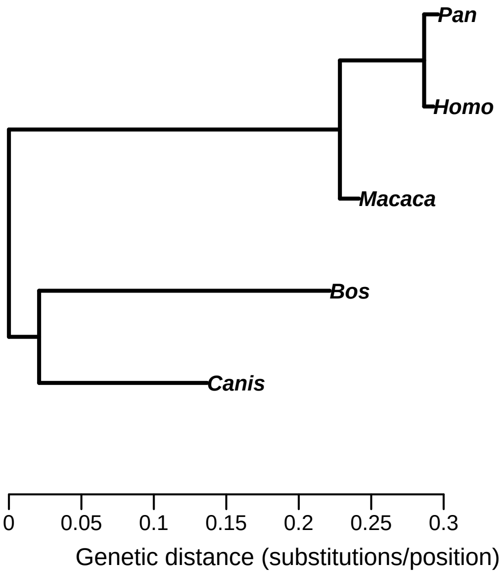

ENSG00000185674 intron 2

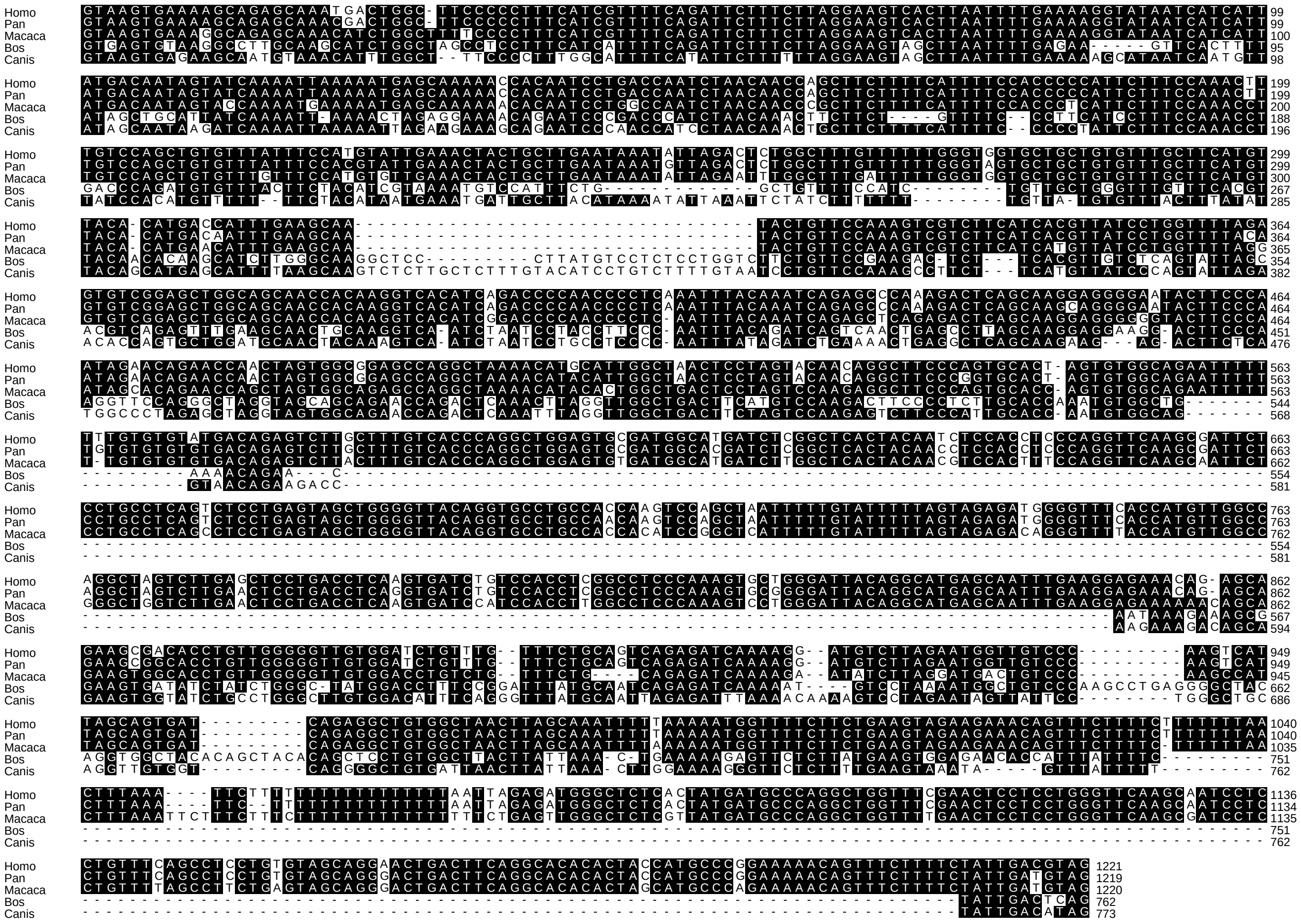

ENSG00000166394 intron 3

Description: cytochrome b5 reductase b5R (CYB5R2)
Intron number: 3
Human chromosome: 11
Intron start (bp): 7647142
Human intron length : 290
Intron alignment length: 299
Flanking exons length (upstream/downstream): 107/130
SNP density: 0.006897
K tree score: 0.0901
Scaling factor: 0.8748
Human-chimpanzee distance: 0.035241
Total primate branch length: 0.0882

ENSG00000166394 exon 3

```
Homo    GTAACTATGTCCAGCTCTTGGCAAAAATCGATAATGAATTGGTGGTCAGGGCTTACACCCCTGTCTCCAGTGATGATGAC 80
Pan     GTAACTATGTCCAGCTCTTGGCGAAAATTGGCAATGAATTGGTGGTCAGGGCTTACACCCCTGTCTCCAGTGATGATGAC 80
Macaca  GTAACTATGTCCAGCTCTTGGCGAAAATTGATAATGAATTGGTGGTCAGGGCTTACACGCCTGTCTCTAGTGATGATGAC 80
Bos     GTAACTATGTTCATCTCTTGGCCGAAATTGACAGTGTTCTGGTGGTCAGGGCTTACACGCCTGTGTCCAGTGATGACGAC 80
Canis   GTAACTATGTCCATCTCTTGGCCAAAATTGATGGCGTTATGGTGGTCAGGGCTTACACGCCTGTGTCCAGTGATGATGAC 80

Homo    AGAGGCTTTGTGGACCTAATTATAAAG 107
Pan     AGAGGCTTTGTGGACCTAATTATAAAG 107
Macaca  AGAGGCTTTGTGGACTTAATTATAAAG 107
Bos     CTAGGCTTTGTGGACTTAATCATCAAG 107
Canis   CGAGGCTTTGTGGACTTGATTATAAAG 107
```

ENSG00000166394 exon 4

```
Homo    ATCTACTTCAAAAATGTACACCCCCAATATCCTGAAGGTGGGAAGATGACTCAGTATTTGGAGAACATGAAAATCGGGGA 80
Pan     ATCTACTTCAAAAATGTACACCCCCAATATCCTGAAGGTGGGAAGATGACTCCGTATTTGGAGAACATGAAAATCGGGGA 80
Macaca  ATCTACTTCAAAAATGTACACCCCCAGTATCCTGAAGGGGGGAAGATGACTCAGTATTTGGAGAACATGAAAATCGGGGA 80
Bos     ATCTACTTCAAAAATGTACATCCTAATTACCCAGAAGGGGGGAAGATGACTCAGTACTTGGAGAACATGAAAATTGGGGA 80
Canis   GTCTACTTCAAAAATGTGCACCCCAATTATCCAGAAGGGGGGAAGATGACTCAGTACTTGGAGAACATGAAAATCGGGGA 80

Homo    GACCATCTTTTTTCGAGGGCCAAGGGGACGCTTGTTTTACCATGGGCCAG 130
Pan     GACCATCTTTTTTCGAGGGCCAAAGGGACGCTTGTTTTACCATGGGCCAG 130
Macaca  CACCATCTTTTTTCGAGGGCCAAAGGGACGCTTGTTTTACCATGGGCCAG 130
Bos     CACCATCCTTTTTCAAGGGCCATCTGGGTGCCTGTTCTATCATGGGTCAG 130
Canis   CACCATCCTTTTTCGAGGGCCAACTGGTCGCCTCTTCTATAATGGGCCAG 130
```

ENSG00000166394 intron 3

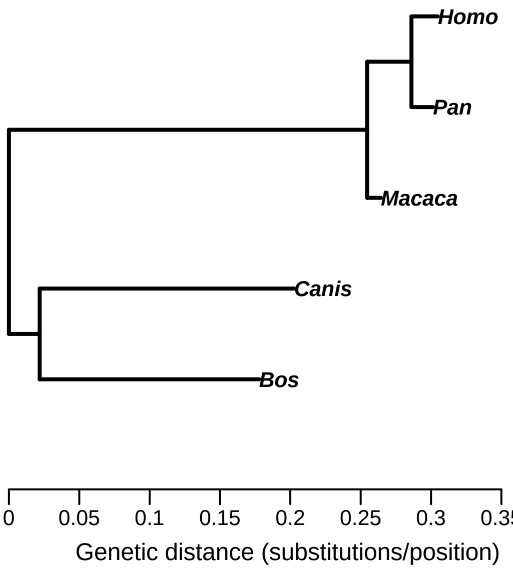

ENSG00000166394 intron 3

```
Homo    GTAAATAGTGCCCACCATATCTGGCTAGCTCGTGCTTCCAAGCCTACTGAGGGTGGTGGGGTGGAGGGGCAGTGCTTTTTCTCTGAGGGCACATATGCCC 100
Pan     GTAAATAGTGCCCACCATATCTGGCTAGCTCGTGCTTCCAAGCCTATGGAGGGTGGTGGGGTGGAGGGGCAGTGCTTTTTCTCTGAGGGCACATATGCCC 100
Macaca  GTAAATAGTGCTCACCATATCTGGCTGCCTCGTGCTTTCAAGCCTATTGAGGGTGGTGGGGTGGAGGGGCAGTGCTTTTTCTCTGAGGGGACATATGCCC 100
Bos     GTAAAGGATGCCTGCCACGCCCAGCTGCCTCACTGTCACAAGCCTGTTCAGGGTAGT-GGGTGGA-------------------GAAGGGGTGCATGCCC 80
Canis   GTAAGTGGGGCCCACCCTGTCCAGCTGCTTCACCCTCACACACCTATTAAAGGTGGTAGGGTGGA-------------------GAAGGGACACATACCC 81

Homo    TAGGCAGGGCTTTCCAGCAGTGAAGCTACAGTTAACCTTGTGGCAGCTTC---------ATCTTCAGGGATTGCGTACAACAATTCAGAGAGGAGAACAC 191
Pan     TAGACAGGGCTTTCCAGCAGTGAAGCTACAGTTAACCTTGTGGCAGCTGC---------ATCTTCAGGGATTGCGTACAGCAATTGAGAGAGGAGAACAG 191
Macaca  TAGACAGGGCTTTCCAGCAGTGAAGCTACAGTTAACCTTGAGGCAGCTTC---------ATCTTCAGGGACTGCGTACAGCAATTAAGAGAGCAGAACAT 191
Bos     TGGATAGGGCTTCCTGGCAGTG-----TCATTTAACAGTGAAGCTG-TTTCCTAGTGAAGTCTTCAGGGGTTGCTCAGAGCAACCGTGAGCACAGTGGAT 174
Canis   TGGGAAGGGCTTTCCAGAAGGG-----TCATTTAACTGTGCAGCTGATGACTTGGTGAAGTCTTCAGGGG-TGCTTAGAGCAACTGTGAATGCCAAGGAT 175

Homo    TTTCTCTGCAAAGCTTCCAGCAGTAAATGCATTTTCTAGTTGTACGTGGCTGTGGCATTGTGTCTGGGAATGTGAAAGCAGTGCTGCCTTGCTTTTCAG 290
Pan     TTTCTCTGCAAAGCTGCCAGCAGTAAATGCATTTTCTAGTTGTGCGTGGCTGTGGCATTGTGTCTGGGAATGTGAAAGCAGTGCTGCCTTGCTTTTCAG 290
Macaca  TTTCTCTGCAAAGCTTCCAGCAGTAAATGCATTTTCTGGTTGTGTGTGGCTGTGGCATTGTGTCTGGGAATGTGAAAGCAGTGCTGCCTTGCTTTTCAG 290
Bos     TTGCTCTTCACAGTTTCTGGTAGCAAATATATTCCTGAGTTGTATGTGGCTGCAGTCTCGGGTCTAAAGGTGTGAAAACTGTGCTGCTTTATTTTTCAG 273
Canis   TT-----------TCTCCAGTGGCAAATGGAATTTTGAATTGTATGCAGCTGTGGTCTCAGGTCTGACGGTATAAAAATCAGGCTGCCTTATTTTTCAG 263
```

ENSG00000173391 intron 4

Description: Oxidized low-density lipoprotein receptor 1 (OLR1)
Intron number: 4
Human chromosome: 12
Intron start (bp): 10204329
Human intron length : 323
Intron alignment length: 347
Flanking exons length (upstream/downstream): 140/116
SNP density: 0.030960
K tree score: 0.0347
Scaling factor: 0.9677
Human-chimpanzee distance: 0.025321
Total primate branch length: 0.0881

ENSG00000173391 exon 4

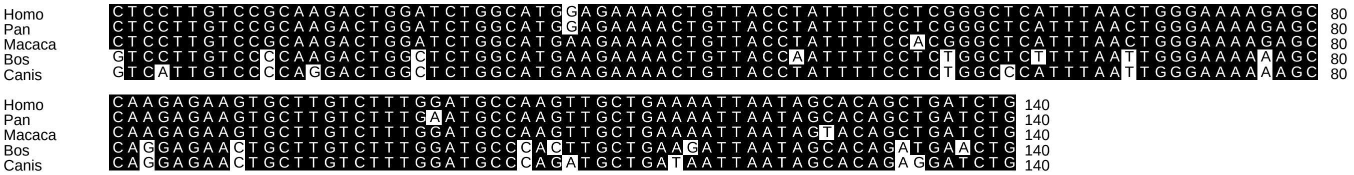

ENSG00000173391 exon 5

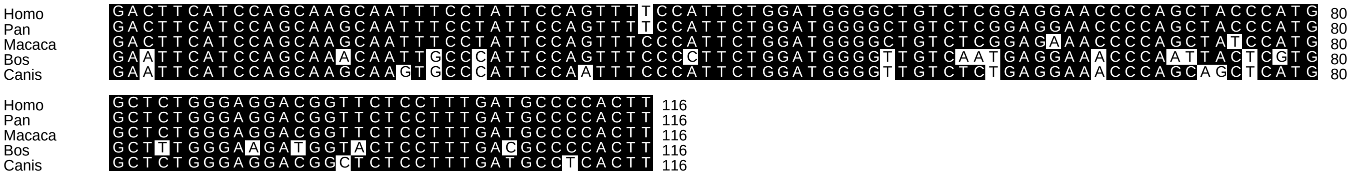

ENSG00000173391 intron 4

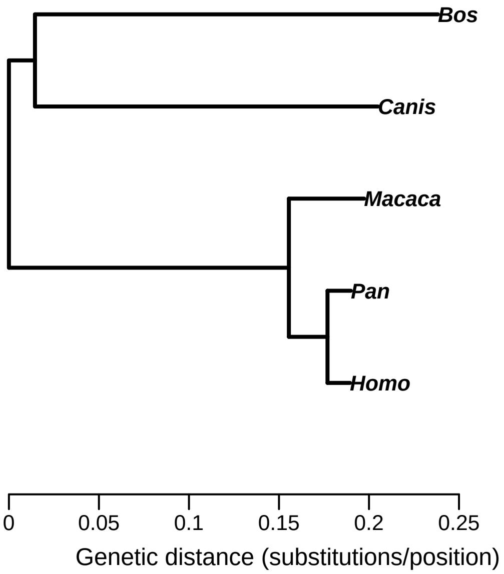

ENSG00000173391 intron 4

```
Homo    GTGAGTGTTCA----------TGGATATTTGTTGGGGTGGAGTATTGGTTGATAGGGAAATTAATCTGGTTTAAACTGTCACTGGAGGAATTCTTGTTTG 90
Pan     GTGAGTGTTCA----------TGGATATTTGTTGGGCTGGAGTATTGGTTGGTAGGGAAATTAATCTGGTTTAAACTGTCACTGGAGGAATTCTTGTTTG 90
Macaca  GTGAGTGTTTA----------TGGATGTTTGTTGGGCTGAAGTATTGGTTGACAGGGAAATGAATCTGGTTTAGACTGTCACTGCAGGAATTCTTGTTTG 90
Bos     GTGAGTGTTCAACAGATGTATGGGATGTGTGTCGGGCTGGAATATTGGGTCATAGGTAAGTAGATCTTGTTTAGACCCTCAGTAGAGGAGTGCTTGCTTA 100
Canis   GTGAGTTTTTA----------TGGTTGTATTTAAGGCTGGAGTAGTGCCTTATAGGGAAATTAATCAGGTTTGCATCCTTGGTAGAGGAGTACATGTTTG 90

Homo    AGAGCCAAAATGTCTGT---TTTTTTAGCTTAGTCTGATCACCAATTTTGTGTATGACCTTGGACGGACAGGCTCTGTAGTATCTCTAGCCTTCAGTAAG 187
Pan     AGAGACAAAATGTCTGT---TTTTTTAGCTTAATCTGATCACCAATTTTGTGTATGACCTTGGACGGACAAGCTCTGTAGTATCTCTAGCCTTCAGTAAG 187
Macaca  AGAGCCAAAATGTCTGT---TTTTTTAGCTTAATCTGATCACCAATTTTGTGCATGACCTTGGACTGACAGGCTCTGTAGTATCTCTAGGCTTCGGTAAC 187
Bos     AGAATCAGAACATGTACATGATTTCTAGCCTTGTGTGGCCACTGGATATGTGCATGACCTTGGATGGACAGGTTACTAAGCATTTCTAGGCTCCACTAGA 200
Canis   AGAGTCAGAATACTTA----TTTTCTAGCCTCATCTGGTTGCCAGTTATGTGCATGACCTTGGGTAGAGAGGTTACATAGTATCTCTAGGCTTCAGTAAA 186

Homo    TGTTTCCGTAAAACAGTCAAGGGGATGTCAAAGA---TACCTTTTGGTTCTTTAGTTTTGTGGATCCAACACTAACTGACTTTCAGATTCTAAAACCACC 284
Pan     TGTTTCCGTAAAACAATCAAGGGGATGTCAAAGA---TAGCTTTTGGTTCTTTAGTTTTGTGGATCTAACACTAACTGACTTTCAGATTCTAAAACCACC 284
Macaca  TGTTTGCATAAAACAATCAAGGGGATGTCAGAAA---TCCCTTTTGGTTCTTTAGTTTTGTGGATCTAACACTAACTGACTTTCAGATTCTAAAACCACC 284
Bos     CATCGCTGTAAAGCCATGGAGAAGATGTCAAAGT---TCTTTTTGAGTTCTTTAGTTTTGTGACTCTAA-------------------TCTAAAAAGTCC 278
Canis   CATTTTTGTAAAACCAT----GGAAAACAAGAGATTTTCTCTTTCAGTTCTTTAGTTTTCTGAATCTAG-------------------TCTAAAACAATC 263

Homo    ATGTGGATTC---CACAAACTC-----AGAAACATTACTCCCCACAG 323
Pan     ATGTGGATTC---CACAAACTC-----AGAAACATTACTCCCCACAG 323
Macaca  ACGTGGATTC---CACAAACTC-----AGAACCATTACTCCCCACAG 323
Bos     ACATCCACTCATTCATCGACAC-----AGAAATGTTACTCCCCACAG 320
Canis   ACCTGGACTT---TATCATCACTTTAGATAAACATTCCTCCCCACAG 307
```

ENSG00000119913 intron 7

Description: Beta-tectorin precursor (TECTB)
Intron number: 7
Human chromosome: 10
Intron start (bp): 114047980
Human intron length : 1260
Intron alignment length: 1667
Flanking exons length (upstream/downstream): 163/73
SNP density: 0.003175
K tree score: 0.0557
Scaling factor: 0.7949
Human-chimpanzee distance: 0.008093
Total primate branch length: 0.0881

ENSG00000119913 exon 7

```
Homo    CCCCACGGATGAAACCGTCCTCGTGCATGAGAATGGGAGAGATCACAGGGCAACCTTCCAATTCAATGCTTTCCGGTTCC 80
Pan     CCCCACGGATGAAACCGTCCTCGTGCATGAGAATGGGAGAGATCACAGGGCAACCTTCCAATTCAATGCTTTCCGGTTCC 80
Macaca  CCCCACGGATGAAACCGTCCTCGTGCATGAGAATGGGAAAGATCACAGGGCAACCTTCCAATTCAATGCTTTCCGGTTCC 80
Bos     CCCCACGGATGAAACCGTCCTTGTGCATGAGAACGGGAAAGACCACAGGGCAACCTTCCAATTCAATGCCTTCCGGTTCC 80
Canis   CCCCACAGATGAAACAGTCCTCGTACATGAGAATGGGAAAGACCACAGGGCCACCTTCCAATTCAATGCTTTCCGGTTCC 80

Homo    AGAACATCCCCAAACTCTCCAAGGTGTGGTTACACTGTGAGACGTTCATCTGCGACAGTGAGAAACTCTCCTGCCCAGTG 160
Pan     AGAACATCCCCAAACTCTCCAAGGTGTGGTTACACTGTGAGACATTCATCTGCGACAGTGAGAAACTCTCCTGCCCAGTG 160
Macaca  AGAACATCCCCAAACTCTCCAAGGTGTGGTTACACTGTGAGACGTTCATCTGCGACAGTGAGAAACTCTCCTGCCCAGTG 160
Bos     AGAACATCCCCAAACTATCCAAGGTGTGGTTACACTGCGAGACGTTCATCTGTGACAGTGAGAAGCTCTCCTGCCCCGTG 160
Canis   AGAACATCCCCAAACTCTCTAAGGTTTGGTTACACTGTGAGACATTCATCTGTGACAGCGAGAAGCTCTCCTGCCCAGTG 160
```

ENSG00000119913 exon 8

```
Homo    ACCTGCGATAAACGGAAGCGCCTCCTGCGAGACCAGACCGGGGGAGTCCTGGTCGTGGAGCTCTCCCTGCGGA 73
Pan     ACCTGCGATAAACGGAAGCGCCTCCTGCGAGACCAGACCGGGGGAGTCCTGGTTGTGGAGCTCTCCCTGCGGA 73
Macaca  ACCTGCGATAAAAGGAAGCGCCTCCTGCGAGACCAGACCGGGGGAGTCCTGGTCGTGGAGCTCTCCCTGCGGA 73
Bos     ACTTGTGACAAGCGGAAACGCCTCCTCCGGGACCAGACCGGGGGCGTGCTGGTGGTGGAGCTCTCCCTCCGTA 73
Canis   ACCTGTGACAAACGGAAGCGCCTCCTGCGGGACCAGACAGGGGGCGTCCTGGTGGTGGAGCTCTCCCTCCGTA 73
```

ENSG00000119913 intron 7

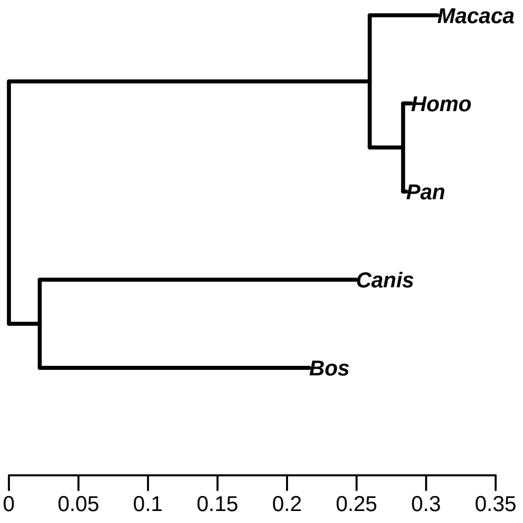

Genetic distance (substitutions/position)

ENSG00000119913 intron 7

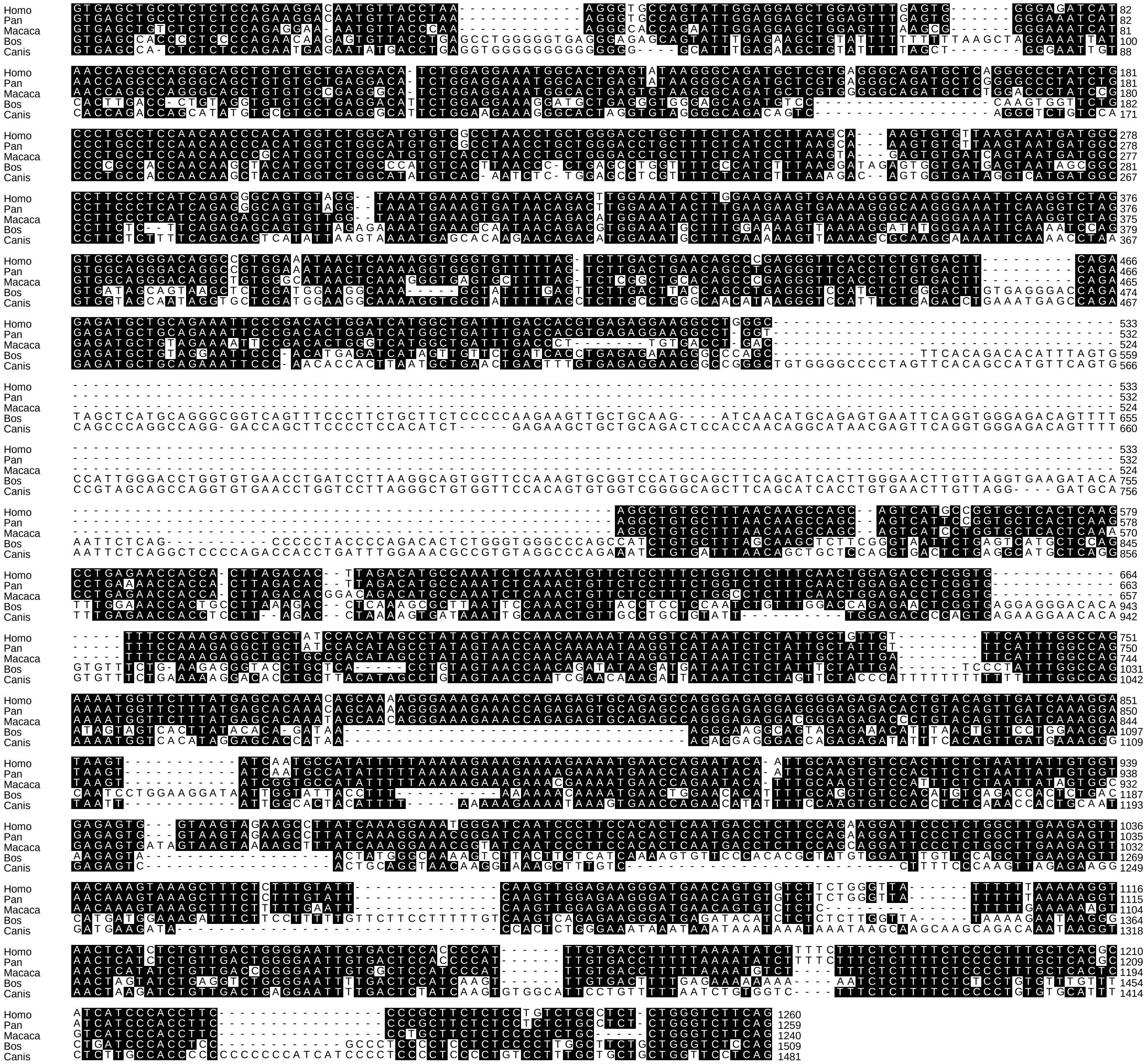

ENSG00000102967 intron 5

Description: Dihydroorotate dehydrogenase, mitochondrial precursor (DHODH)
Intron number: 5
Human chromosome: 16
Intron start (bp): 70612712
Human intron length : 1050
Intron alignment length: 1108
Flanking exons length (upstream/downstream): 188/114
SNP density: 0.003810
K tree score: 0.0968
Scaling factor: 0.794
Human-chimpanzee distance: 0.019051
Total primate branch length: 0.0879

ENSG00000102967 exon 5

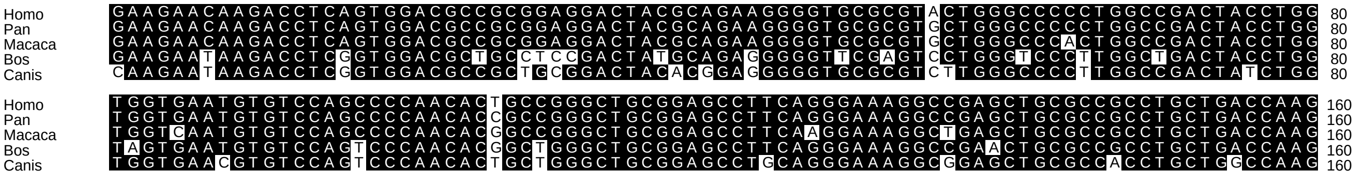

ENSG00000102967 exon 6

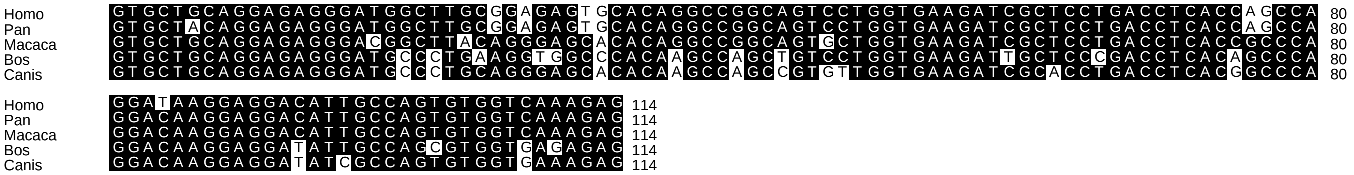

ENSG00000102967 intron 5

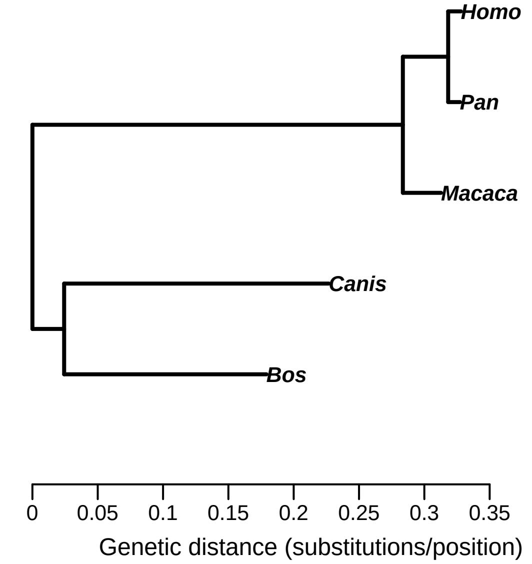

ENSG00000102967 intron 5

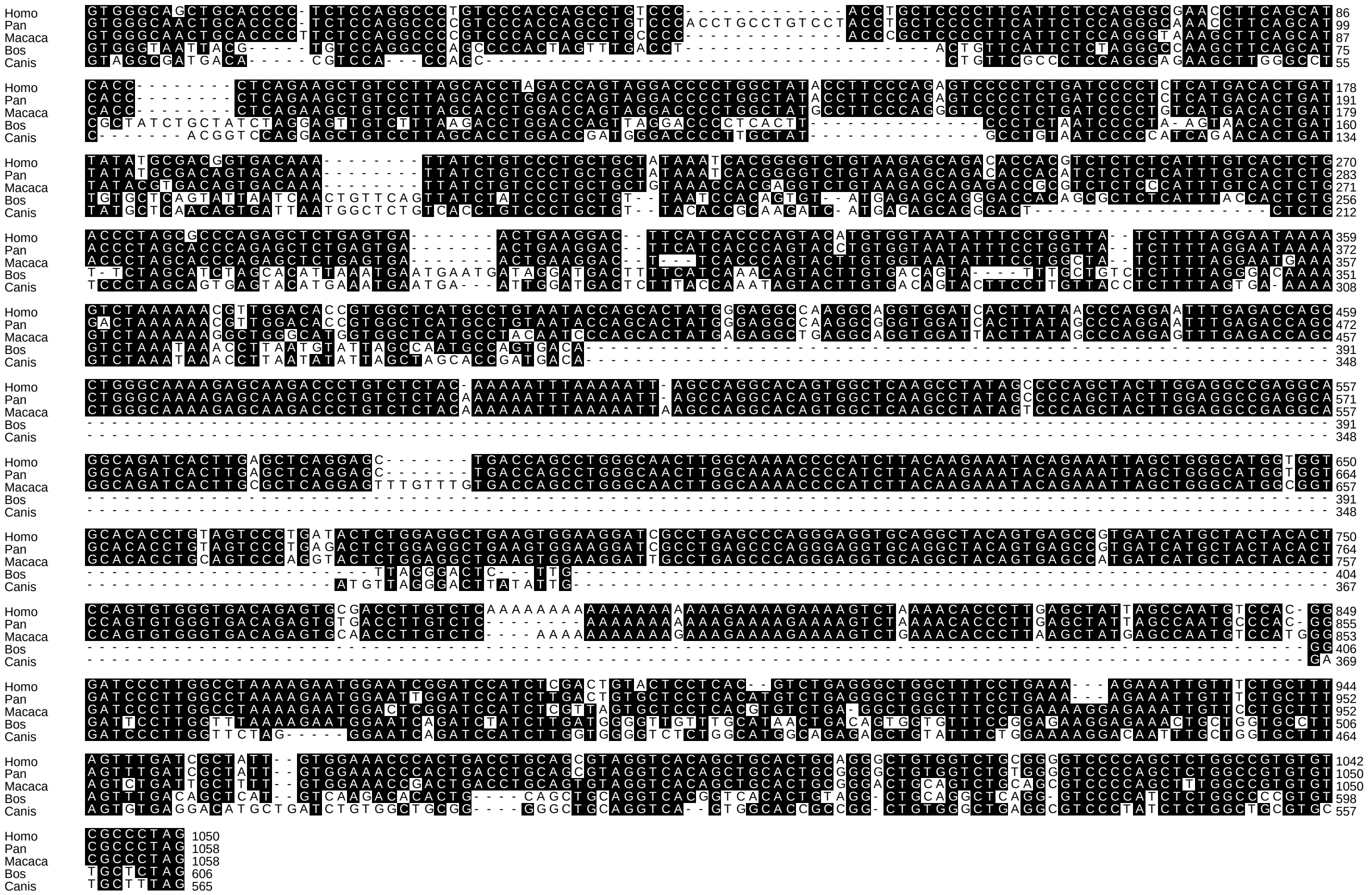

ENSG00000125652 intron 3

Description: Alkylated repair protein alkB homolog 7 precursor (ALKBH7)
Intron number: 3
Human chromosome: 19
Intron start (bp): 6325601
Human intron length : 221
Intron alignment length: 224
Flanking exons length (upstream/downstream): 125/163
SNP density: 0.004525
K tree score: 0.0958
Scaling factor: 0.7581
Human-chimpanzee distance: 0.014624
Total primate branch length: 0.0877

ENSG00000125652 exon 3

```
Homo    TTCTGCGGGGCCACCATCGCCGGCCTGTCTCTCCTGTCTCCCAGCGTTATGCGGCTGGTGCACACCCAGGAGCCGGGGGA 80
Pan     TTCTGCGGGGCCACCATCGCCGGCCTGTCTCTCCTGTCTCCCAGCGTTATGCGGCTGGTGCACACCCAGGAGCCAGGGGA 80
Macaca  TTCTGCGGGGCCACCATCGCCGGCCTGTCTCTCCTTTCCCCCAGCGTTATGCGGCTGGTGCACACCCAGGACCCGGGGGA 80
Bos     TTCTGTGGATCCACCATTGCCGGCCTGTCCCTGTTGTCTCCTAGCGTCATGCGACTAGTGCACACCCAGGAGCCGGGGGA 80
Canis   TTCTGTGGAGCCACCATCGCTGGCCTGTCCCTGCTATCTCCCAGCGTCATGCGGCTGGTGCACACCCAGGAGCCGGGGGA 80

Homo    GTGGCTGGAACTCTTGCTGGAGCCGGGCTCCCTCTACATCCTTAG 125
Pan     GTGGCTGGAACTCTTGCTGGAGCCGGGCTCCCTCTACATCCTTAG 125
Macaca  GTGGCTGGAACTCTTGCTGGAGCCGGGCTCCCTCTACATCCTTAG 125
Bos     GTGGCTGGAACTCTTGCTGGAGCCGGGCTCCCTCTACATCCTTAG 125
Canis   GTGGCTGGAACTCTTGCTGGAGCCAGGCTCCCTCTACATCCTTAG 125
```

ENSG00000125652 exon 4

```
Homo    GGGCTCAGCCCGTTATGACTTCTCCCATGAGATCCTTCGGGATGAAGAGTCCTTCTTTGGGGAACGCCGGATTCCCCGGG 80
Pan     GGGCTCAGCCCGTTATGACTTCTCCCATGAGATCCTTCGGGATGAAGAGTCCTTCTTTGGGGAACGCCGGATTCCCCGGG 80
Macaca  GGGCTCAGCCCGTTATGACTTCTCCCACGAGATCCTTCGGGACGAAGAGTCCTTCTTTGGGGAGCGCCGGATTCCCCGGG 80
Bos     GGGCTCGGCTCGTTATGACTTCTCCCACGAGATCCTTCGGGATGAAGAGTCCTTTTTTGGGGAGCGTCGGATTCCCCGGG 80
Canis   GGGCTCGGCCCGTTATGACTTCTCCCATGAGATTCTTCGGGATGAAGAGTCCTTTTTTGGAGAGCGTCGGGTACCCCGGG 80

Homo    GCCGGCGCATCTCCGTGATCTGCCGCTCCCTCCCTGAGGGCATGGGGCCAGGGGAGTCTGGACAGCCGCCCCCAGCCTGC 160
Pan     GCCGGCGCATCTCCGTGATCTGCCGCTCCCTCCCTGAGGGCATGGGGCCAGGGGAGTCTGGACAGCCGCCCCCAGCCTGC 160
Macaca  GCCGGCGCATCTCCGTGATCTGCCGCTCCCTCCCTGAGGGCATGGGGCCAGGGGAGTCTGGACAGCCGCCCCCAGCCTGC 160
Bos     GCCGTCGCATCTCGGTGATTTGCCGCTCCCTCCCTGAGGGGATGGGGCCAGGGGAGCCTGGGCAGCTGCCCCCAGCCTGC 160
Canis   GCCGACGCATCTCTGTGATCTGTCGCTCCCTCCCGGAGGGGATGGAATCAGGGGAGCCCGGACAGCCGCCCCCAGCCTGC 160
```

ENSG00000125652 intron 3

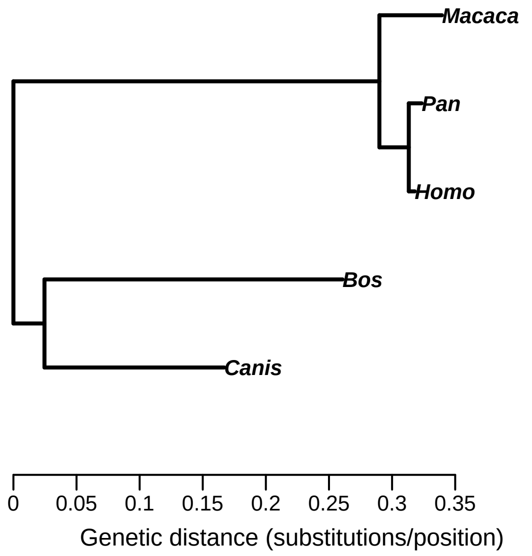

ENSG00000125652 intron 3

```
Homo    GTA-CCTCCATCCAGGCAGCACCCACCCCTCCAAGAATGTGCCATCCGCCCACCCTGTGCCCTGGGTACTTTCCCTCAAATCCAGCCCTCCCCGAAGACGC 99
Pan     GTA-CCTCCATCCAGGCAGCACCCACCCCTCCAAGAATGTGCCATCCGCCCACCCTGTGCCCTGGGTACTTTCCCTCAAATCCAGCCCTCCCCGAAGACGC 99
Macaca  GTA-CCTCCAGCCAGGCAGC-------------TGATTGTGCCATCCACCCCCCCAGTGCCCTGGGTACCTTCCCTCACTCCAGCCCTCCCCAAAGAAGC 86
Bos     GTA-TGGGCAGCCAAGGAGCCCACACTGCACCAGTAACTCACAAGACACCCACTGAACATTTGGAACACCTTCTGTCA-TTCAGCCCTGACAGCAGACTC 98
Canis   GTACTGCCCGGCCGGGGAGCCCCCTCCCCACCAGTAACTCATCAGATGCCCACCTAGCATGTTGAGCACCTTCTCTTT-TTCAGCCCTCCCGGCAGACAC 99

Homo    --CTTTACCCCAGGGTCTGTGTGGGAGGCAGCAGGGGGCTGGAATCCAGGAGGCTGGAATCCAGGTCTGCCTGGAGCAGGGGCTGCCCTCCTTGCACCCA 197
Pan     --CTTTACCCCAGGGTCTGTGTGGGAGGCAGCAGGG---------------GGCTGGAATCCTGGTCTGCCTGGCGCAGGGGCTGCCCTCCTTGCACCCA 182
Macaca  --CCTTACCCCAGGGCCTGTGTGGGAGGCAGCAGGG---------------GGCTGGAATCCAGGTCTGCCTGGAGCAGGGGCTGCCCTCCTTGCACCCA 169
Bos     -----TACCCCTAGGGCTG---------------------------------------------GTCTGCCTGGAACCAGGGCCACCCTCACCAGCCACA 148
Canis   GATCGTTCCCCCGTGGCAGGCT------------------------------------------GTCCGCCTGGCTCGGGGGCTGCCGTCACCCCACCCA 157

Homo    GTCACAGCCTGTTTCTTCCTGCAG 221
Pan     GTCACAGCCTGTTTCCTCCTGCAG 206
Macaca  GTCACAGCCTGCTTCCTCCTGCAG 193
Bos     TC----CTCTGTTTTCTCCTGCAG 168
Canis   GCGACAGCCTGTTTTCTCTTGCAG 181
```

ENSG00000164331 intron 5

Description: Ankyrin repeat family A protein 2 (ANKRA2)
Intron number: 5
Human chromosome: 05
Intron start (bp): 72885970
Human intron length : 472
Intron alignment length: 482
Flanking exons length (upstream/downstream): 126/67
SNP density: 0.006356
K tree score: 0.0486
Scaling factor: 1.0521
Human-chimpanzee distance: 0.015029
Total primate branch length: 0.0876

ENSG00000164331 exon 5

```
Homo    GGTGCTGATCCCCAACTTTTAGGAAAAGGTCGAGAAAGTGCACTGTCGTTGGCCTGTAGTAAAGGCTACACAGATATTGT 80
Pan     GGTGCTGATCCCCAACTTTTAGGAAAAGGTCGAGAAAGTGCACTGTCGTTGGCCTGTAGTAAAGGCTACACAGATATTGT 80
Macaca  GGTGCTGATCCCCAACTTTTAGGAAAAGGTCGAGAAAGTGCACTGTCGTTGGCCTGTAGTAAAGGCTACACAGATATTGT 80
Bos     GGCGCTGATCCTCAGCTTTTAGGAAAAGGTCGAGAAAGTGCTCTGTCATTGGCCTGTAGCAAGGGCTACACAGATATTGT 80
Canis   GGTGCTGATCCCCAGCTTTTAGGAAAAGGTCGAGAAAGTGCACTCTCATTGGCATGTAGTAAAGGCTACACAGATATTGT 80

Homo    CAAAATGCTGCTTGATTGTGGAGTTGATGTAAATGAATATGATTGG 126
Pan     CAAAATGCTGCTTGATTGTGGAGTTGATGTAAATGAATATGATTGG 126
Macaca  CAAAATGCTGCTTGATTGTGGAGTTGATGTAAATGAATATGATTGG 126
Bos     CAAAATGCTGCTGGATTGTGGAGTTGATGTAAATGAATATGATTGG 126
Canis   CAAAATGCTGCTTGATTGTGGAGTTGATGTAAATGAATATGATTGG 126
```

ENSG00000164331 exon 6

```
Homo    AATGGAGGAACACCTCTGCTTTATGCTGTACATGGAAATCATGTGAAATGTGTAAAGATGCTCTTAG 67
Pan     AATGGAGGAACACCTCTGCTTTATGCTGTACATGGAAATCATGTGAAATGTGTAAAGATGCTCTTAG 67
Macaca  AATGGAGGAACACCTCTGCTTTATGCTGTACATGGAAATCATGTGAAATGTGTAAAGATGCTCTTAG 67
Bos     AATGGAGGGACACCTTTGCTTTACGCTGTACACGGAAATCATGTGAAATGTGTAAAAATGCTCTTAG 67
Canis   AATGGAGGGACACCTTTGCTTTATGCTGTACATGGAAATCATGTTAAATGTGTAAAAATGCTTTTAG 67
```

ENSG00000164331 intron 5

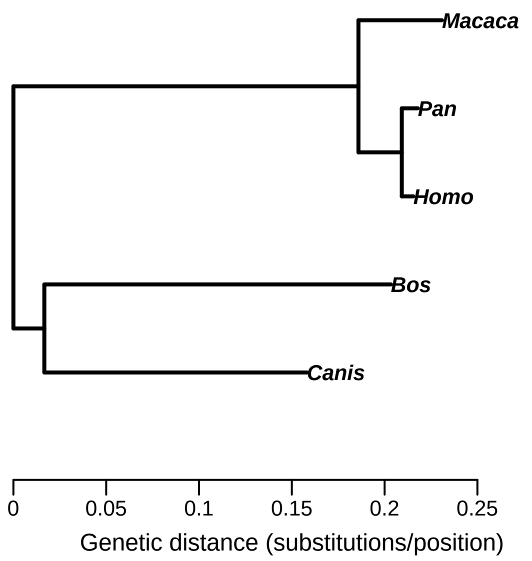

ENSG00000164331 intron 5

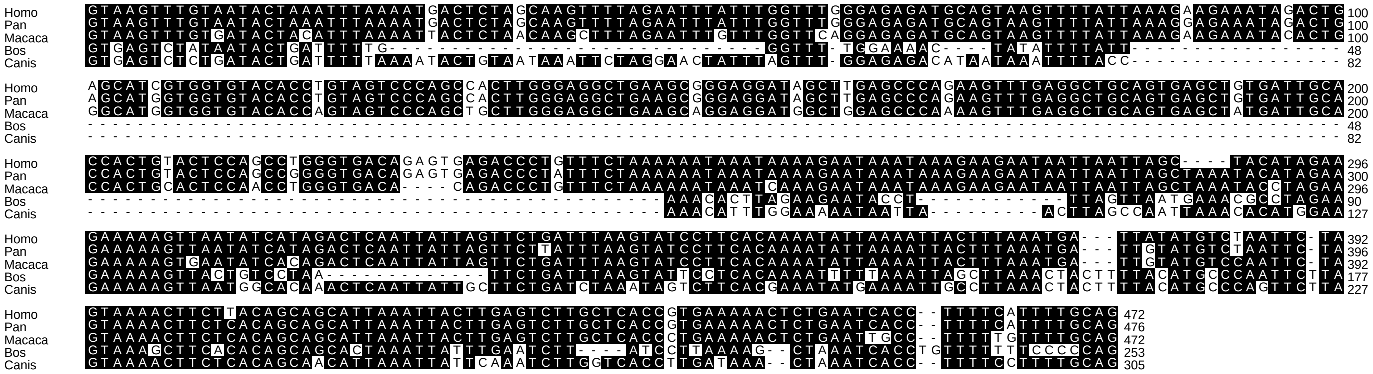

ENSG00000128510 intron 6

Description: Carboxypeptidase A4 precursor (CPA4)
Intron number: 6
Human chromosome: 07
Intron start (bp): 129732997
Human intron length : 866
Intron alignment length: 923
Flanking exons length (upstream/downstream): 105/111
SNP density: 0.001155
K tree score: 0.0681
Scaling factor: 0.7801
Human-chimpanzee distance: 0.005818
Total primate branch length: 0.0871

ENSG00000128510 exon 6

```
Homo    TTCAGCACTGGGAAAGGCGTGAGGCGGCCGGCCGTTTGGCTGAATGCAGGCATCCATTCCCGAGAGTGGATCTCCCAGGC 80
Pan     TTCAGCACTGGGAAAGGCGCGCGGCGGCCGGCCGTTTGGCTGAATGCAGGCATCCATTCCCGAGAGTGGATCTCCCAGGC 80
Macaca  TTCAGCACTGGGGAAGGCGCACGGCGGCCGGCCATTTGGCTGAATGCAGGCATCCATTCCCGAGAGTGGATCTCCCAGGC 80
Bos     TTCAGCACTGGAAAAGGCAGGCAGCGCCCGGCCATTTTGCTGAATGCAGGCATCCATGCCCGGGAGTGGATCTCATCGGC 80
Canis   TTCAGCACTGCAGAAGGCAGGCGGCGGCCAGCCATTTGGCTGAATGCGGGCATCCATTCCCGGGAGTGGATTTCGCAGGC 80

Homo    CACTGCAATCTGGACGGCAAGGAAG 105
Pan     CACTGCAATCTGGACGGCAAGGAAG 105
Macaca  CACTGCAATCTGGACGGCAAGGAAG 105
Bos     CACAGCAATCTGGACTGCGAGAAAG 105
Canis   CACAGCAATCTGGACCGCGAGGAAG 105
```

ENSG00000128510 exon 7

```
Homo    ATTGTATCTGATTACCAGAGGGATCCAGCTATCACCTCCATCTTGGAGAAAATGGATATTTTCTTGTTGCCTGTGGCCAA 80
Pan     ATTGTATCTGATTACCAGAGGGATCCAGCTATCACCTCCATCTTGGAGAAAATGGATATTTTCTTGTTGCCTGTGGCCAA 80
Macaca  ATTGTATCTGATTACCAGAGGGATCCAGCTATCACCTCCATCTTGGAAAAAATGGATATTTTCTTGTTGCCTGTGGCCAA 80
Bos     ATTGCATGTGATTATGGGAGAGATCTAGTCATCACCTCCATCTTGGAGAAAATGGATATATTCTTGTTGCCGGTGGCCAA 80
Canis   ATTGTATCTGATTATGGGAAGGACCCAGCTCTCACCTCCATCTTGGAGAAAATGGATATTTTCTTGTTGCCTGTGGCCAA 80

Homo    TCCTGATGGATATGTGTATACTCAAACTCAA 111
Pan     TCCTGACGGATATGTGTATACTCAAACTCAA 111
Macaca  TCCTGATGGATATGTGTATACTCAAACTCAA 111
Bos     TCCTGATGGATATGTGTACACTCATACTCAC 111
Canis   TCCTGATGGATATGTGTACACACAAACTCAA 111
```

ENSG00000128510 intron 6

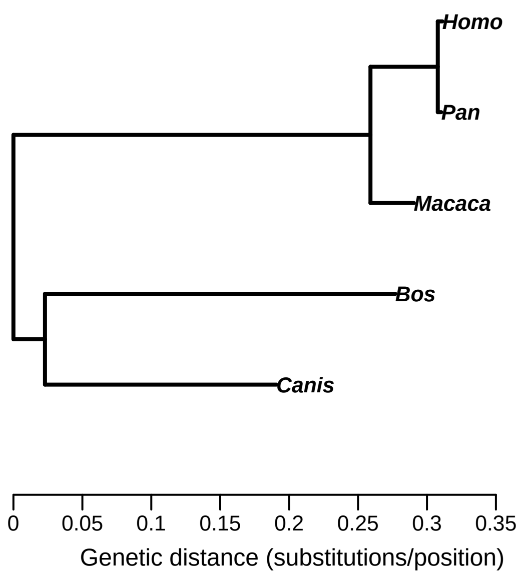

ENSG00000128510 intron 6

```
Homo    GTCATGCTGCGTGGTATTAGCCAGGAATGCTGATGGTGCCGGGGTGCCCCTGCAGCATGCCATGTGGCTGGGCCTGGGGCACGAGG------------TG 88
Pan     GTCATGCCGCGTGGTATTAGCCAGGAATGCTGATGGTGCCGGGGTGCCCCTGCAGCATGCCATGTGGCTGGGCCTGGGGCACGAGG------------TG 88
Macaca  GTCATGTCGCCTGGTATTAGCCAAGAATGCTGATGGGCCCGGGGTGCCCCTGAAGCATGCCATGTAGCTGGGCCTGGGGCACGAGG------------TG 88
Bos     GTCATGTCGCCTAGTATTAGCCCAGCATTCTGCAGGGCCAGGGGTG-CCCTGTACTGTACTATGCAGCTGTACCTGGGAGGGGTGGGATGTGGTAGGTTA 99
Canis   GTCATGTCGCCCGGTATTAGCCGAGAATTGTGGTGGGCCCAGGGTGCCCCTGCAGGGTCTCATGAGGCTGGGCTTGGGCGACGAGG------------TG 88

Homo    GGAGGGTTCTCTACTAAGACCCAGTCGGCTGGGGG-GCCTTTTCAGATTGGAGAAGCAAATGGTCAAAACCCCTTGGTCAGCT---CACTGCGGCCAAAT 184
Pan     GGAGGGTTCTCTACTAAGACCCAGTCGGCTGGGGG-GCCTTTTCAGATTGGAGAAGCAAATGGTCAAAACCCCTTGGTCAGCT---CACTGCGGCCAAAT 184
Macaca  GGAGGGTGCTCTGCTAAGACCCAGTCGGCT-GGGG-GCCTTTTCAGAGTGGAGAAGTAAATGGTCAAAGCCCCTTGGTCAGCT---CATTGCAGCCAAA- 182
Bos     GGAGGGTACTCT-CACAGGAACAATAGGCTGGGGGAGTCTTTTCAAATTGGAGAAACAAAAGATCAGAGCCCCACAGTCAGCCTCATAGTTTAGCTAAA- 197
Canis   GGAAGGCACTCTGCACAGACACAGTGGGCC-GGGG-GCCTTTTCAGATCAGAGAAGCAAAAGGTCAGAGCCCCTTGGTCAAGC---CGCTGTGGCTAAA- 182

Homo    TGATTGCGTTCCTGGTGGTGCACAAG----CTGATATGCAGCAGAGGTGGGCCTGAGCCTCTGA-GAGCAGTCCACTCCGCAGAAAGGGAGCAGAGCAAC 279
Pan     TGATTGCGTTCCTGGTGGTGCACAAG----CTGATATGCAGCAGAGGTGGGCCTGAGCCTCTGA-GAGCAGTCCACTCCGCAGAAAGGGAGCAGAGCAAC 279
Macaca  CGATTGCGTTCCTGCTGGTGCACAAG----CTGATATGCAGCAGAGGTGGGCCTGAGCCTCTGA-GCGCAGTCCACCCCACAGAAAGGGAGTGGAGCAAC 277
Bos     TGGCAATGTCCGCTCATAGCAGTGAGCATACTAATGAGCTGTAGAGGTGGCCCTGAAACACTGACACATAGTCAGTCCTGC------------------- 278
Canis   TGACAAAGTTCATTCATAGTAGCGAG----CTAATGAGCTGCAGAGGTGGCCCTGAAACTCTAACCCGTAGTCAGCACCACAGGAAGGGAGTGGAGTCTT 278

Homo    ACC--CAGACAAGGCCCCATAGACCCTCGGGGACCTCA-CGCCATCTCCATCTCCTAGAAAGGCTGCTGGGAGGGCAG-----CAGGAGGCTGAGGCAGG 371
Pan     ACC--CAGACGAGGCCCCATAGACCCTCGGGGACCTCA-CGCCATCTCCATCTCCTAGAAAGGCTGCTGGGAGGGCAG-----CAGGAGGCTGAGGCAGG 371
Macaca  ACC--CAGCGGAGGCCCCACAGGCCCTTGGGGACCTCATTGCCATCTCCATCTCCTAGAAAGGCTTCTGGGAGGGCAGCTACTCAAGAGGCTGAGGCAGG 375
Bos     -TCAGCAGCAAGGCTCAGATAATTTTTA-----CCTTCAGTCCCTTTCAGGCTCTC-------------------CTGGCTCTCAGGGACCTCATGCTA- 352
Canis   CTCATCAGTGAAACTCCGATTTAACTTTG----CCTTGGGCCCTGCCCTAGCCCTC-------------------CAGATCCTCAGGGGCCTCATGCAG- 354

Homo    AGAATCCCTTGAACCCAGGAGGCGGAGGTTGCGTTGAGCCAAGATCACGCCACTGCACTCCAGCCTGGGCAACAGAGCAAGACTCTGTCTT------AAA 465
Pan     AGAATCCCTTGAACCCAGGAGGCGGAGGTTGCGTTGAGCCAAGATCACGCCACTGCACTCCAGCCTGGGCAACAGAGCAAGACTCTGTCTT------AAA 465
Macaca  AGAATCCCTTGAACCCAGGAGGTGGAGGTTGTGGTGAGCTGAGATCATGCTATTGCACTCCAGCCTGGGCAACAGAGCGAGACCCTGTCTTAAAAAAAAA 475
Bos     -----------------------------------------TCTCCATGCTCTGTCCCTCTGG----------------AAAGTCTAGTTG--------- 386
Canis   ---------------------------------------------TCTACCATGTCCCTCTGG----------------AGAGTCTGCTTT--------- 384

Homo    A----AAAGAAAGAAAGAAAGTCTGCTGGGAGGG-GAGGAGGGGTGCATGTGGAGGACACATCTTCCTTCCCTCTTCAT-TGGAGGTTCAAATTCCTAAA 559
Pan     AAAAGAAAGAAAGAAAGAAAGTCTGCGGGGAGGG-GAGGAGGGGTGCATGTGGAGGACACATCTTCCCTCCCTCTTCAT-TGGAGGTTCAAATTCCTAAA 563
Macaca  AAAAGAAAGAAAGAAAGAAAGGCTGCTGGGA----GAGGAGGGTTGCATGTGGAGGACACATCTTCCCTCCCTCTTCAT-TGGAGGTTCAAATTCCTAAA 570
Bos     ------------------AAGCCAGTTCCAGGGAGGAGGGGTGGTGCGAGTGGAGGATCCATCTTCCCCAAC-CCTAGCACAGGGGCTCATATTCCTAAT 467
Canis   ------------------AAGGCGGCACTAGGGA-CAGGAGTGCTGCACGTGGAGGATTTATTTTCCCTGCCTGTTAGCATGGGGGCTCAGAGCCCCCAA 465

Homo    AACTATTACAATGTGTGGCTCCCTCCT--------------CCTGGAAGGAAGGAATAAAGCACGTACCCCAGGACTCACTGCAGTTGATGCCATTAGCA 645
Pan     AACTATTACAATGTGTGGCTCCCTCCT--------------CCTGGAAGGAAGGAATAAAGCACGTACCCCAGGACTCACTGCAGTTGATGCCATTAGCA 649
Macaca  AACTATTA-GATGTGTGACTCCCTCCT--------------CCTGGAAGGAAGGAATAAAGCATGTACCCCAGGACTCACTGCAGTTGATATCATTAGCA 655
Bos     AACTATTATGATGTGTGGTTCTCCTTTCCCCTGCTCCTACCCCCTGGAAGGAAGAATAAACCAGGCAGCCCAGGACTCATTGAAGTTG------------ 555
Canis   AACTGTTAGGACATGTGGCTCTTCCCTCCCTGTTCCCACCCCCTGGAAGGAATGAATGAACCAGGCAGCCCTGGACTCATTGCAGTTG------------ 553

Homo    GCCATGTCCCCAAGCAGCCGTGTCTCTCCAGGCTTTGAAGGGCTCAGAGGGCTTTGCAGAGAGCAGAGCCTCTGAGGGTGGCAGGGAGGCTT-TTGAGGG 744
Pan     GCCATGTCCCCAAGCAGCCGTGTCTCTCCAGGCTTTGAAGGGCTCAGAGGGCTTTGCAGAGAGC-GAGCCTCTGAGGGTGGCAGGGAGGCTT-TTGAGGG 747
Macaca  GCCATGTCCCCAAGCAGCTATGTCTCTCCAGGATTTCAAGGGCTCAGAGGGCTTTGCAGAGAGCAAAGCTTTTGAGGGTGGCAGGGAGGCTT-TTGAGGT 754
Bos     ---ATGTCATCAGGTAGCCATGTTCCCTCAGATTTTGAAGTGCTCCGTGGGCTTTGCAGAGAGCTGCACCTACAAGGGTGGCAGGGAGACTGCCAGAGGG 652
Canis   ---CTGTCCTCATGAAGCCACATTTCCGCAGGGTTTGAAGGGCTCCAAGAGCTTTGCAGAGAGCTGTGTCTCTGAGGGTGACAGGGAGTCTG-CAGAG-- 647

Homo    TGGATGCTGTTCTAGGAGCATTCATTCTAGCTGGTTGCTGGGCATCTTGAGAAGAT-ACATGGTTCAGCCCTAATGGCCCATCCTGCCATGGGATAGCTG 843
Pan     TGGATGCTGTTCTAGGAGCATTCATTCTAGCTGGTGGCTGGGCATCTTGAGAAGAT-ACATGGTTCAGCCCTAATGGCCCATCCTGCCATGGGATAGCTG 846
Macaca  TAGATGCTGTTCTAGGAGCATCCATTCTAGCTGGATGCCGGGCATCTTTGGAAGAT-ACATGGTTCAGCCCTAATGACCCATCCTGCTATGGGATAGCTG 853
Bos     TAGATGCTGTTCTAGGAGCT-------------------GGGCATCTGGGGAAGATACTCTGACTTAGATCTAGCAACCCAGCCTGCAAAGACATAGCTG 733
Canis   ---------------------------------------GGGCATCTGGAGAAGATGACATGGGCTTA-------GACCTTGCTGGCCAAAGCATAGCTG 701

Homo    ACAAGCATATTGTCTCTGCTTAG 866
Pan     ACAAGCATATTGTCTCTGCTTAG 869
Macaca  ACAAGCATATTGTCTCTGCTTAG 876
Bos     ACAAGCATGTTGTCTTTGCTTAG 756
Canis   ACAAGCTTCTGGTCTCTGCTTAG 724
```

ENSG00000078898 intron 10

Description: Bactericidal/permeability-increasing protein-like 1 precursor (BPIL1)
Intron number: 10
Human chromosome: 20
Intron start (bp): 31071218
Human intron length : 577
Intron alignment length: 618
Flanking exons length (upstream/downstream): 171/68
SNP density: 0.003466
K tree score: 0.0565
Scaling factor: 1.0798
Human-chimpanzee distance: 0.008696
Total primate branch length: 0.087

ENSG00000078898 exon 10

```
Homo    GTTTCCCGAGCCCATGCCTGTGGTGCTCAAGGTGCGGCTGGGTGCCACACCTGTGGCCATGCTCCACACAAACAACGCCA 80
Pan     GTTTCCCGAGCCCATGCCTGTGGTGCTCAAGGTGCGGCTGGGTGCCACACCTGTGGCCATGCTCCACACAAACAACGCCA 80
Macaca  GTTCCCCGAGCCCATGCCCGTGGTGCTCAAGGTGCGGCTGGGTACCACACCTGTGGCCATGCTCCACACCAACAACGCCA 80
Bos     ATTCCCCGAGCCCATGCCCCTGCTGCTCAAGGTGAGGCTGGGTGCCACGCCCACAGTCACGTTCCGCACCAACAACGCCA 80
Canis   GTTCCCCGAGCCCATGCCTGTGACTCTCAAGGTGCGGCTGGGTGCCACGCCCGTGGCCACACTTCGTACCAACAATGCCA 80

Homo    CCCTGCGGCTGCAGCCCTTCGTGGAGGTCCTGGCCACAGCCTCCAACTCGGCTTTCCAGTCCCTCTTCTCCCTGGATGTG 160
Pan     CCCTGCGGCTGCAGCCCTTCGTGGAGGTCCTGGCCACAGCCTCCAACTCGGCTTTCCAATCCCTCTTCTCCCTGGATGTG 160
Macaca  CCCTACGACTGCAGCCCTTCGTGGAGGTCCTGGCCGCAGCCTCCAACTCGGCTTTCCAGTCCCTCTTCTCCCTGGATGTG 160
Bos     CGCTGCAGCTGCAGCCCTTTGTGGAAGTCCTGGCCCCGACCTCCAACTCGGCTTTCCAGTACCTCTTCTCCCTCAATGTG 160
Canis   CGCTTCAGCTGCAGCCCTTTGTGGAAGTCCAGGCTGTGACCTCCAACTCAGCTTTCCAGTCCCTCTTCTCCTTTGATGTG 160
```

ENSG00000078898 exon 11

```
Homo    GTAGTGAACTTGAGACTCCAGCTCTCTGTGTCCAAGGTGAAGCTTCAGGGGACCACGTCTGTGCTGGG 68
Pan     GTAGTGAACTTGAGACTCCAGCTCTCTGTGTCCAAGGTGAAGCTTCAGGGGACCACGTCTGTGCTGGG 68
Macaca  GTAGTGAACTTGAGCCTCCAGCTCTCTGTGTCCAAGGTGAAGCTTCAGGGGACCACGTCTGTGCTGGG 68
Bos     GCAGTGAACCTGAGCCTCCAGCTTTCTGTGTCCAAGGTGAGGCTTCGGGGGACCACATCTGTGCTGGG 68
Canis   GTTGTGAACCTGGACCTCCAGCTCTCTGTGTCCAAGGTGAAGCTTCAGGGCACCACATCTGTGCTGGG 68
```

ENSG00000078898 intron 10

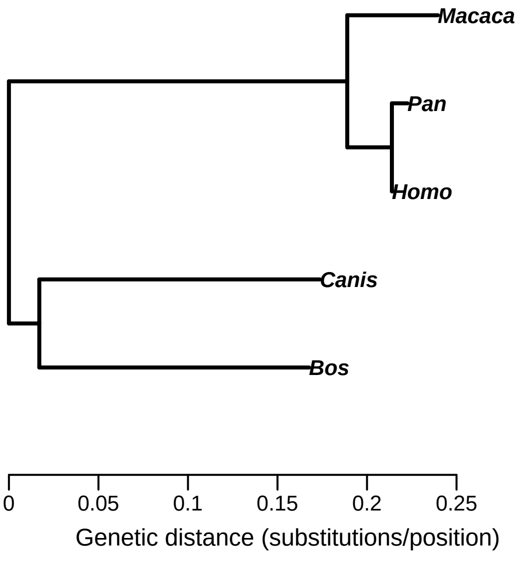

ENSG00000078898 intron 10

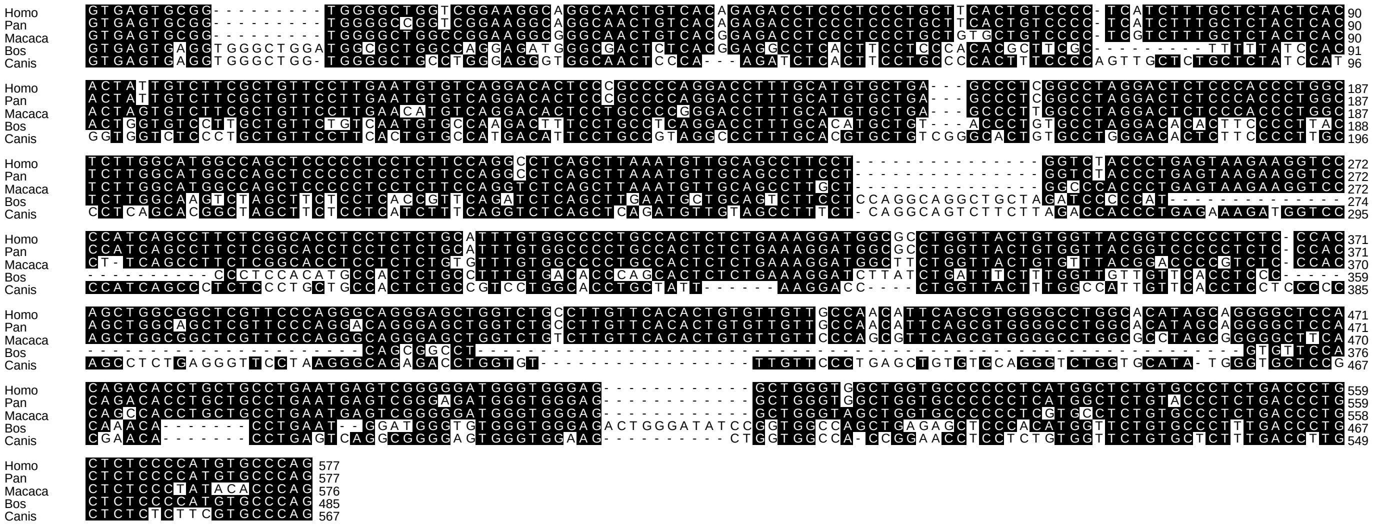

ENSG00000183921 intron 4

Description: Putative short chain dehydrogenase/reductase family 42E-2 (SDR42E2)
Intron number: 4
Human chromosome: 16
Intron start (bp): 22089774
Human intron length : 1304
Intron alignment length: 1652
Flanking exons length (upstream/downstream): 119/76
SNP density: 0.002301
K tree score: 0.0727
Scaling factor: 0.7567
Human-chimpanzee distance: 0.010282
Total primate branch length: 0.0869

ENSG00000183921 exon 4

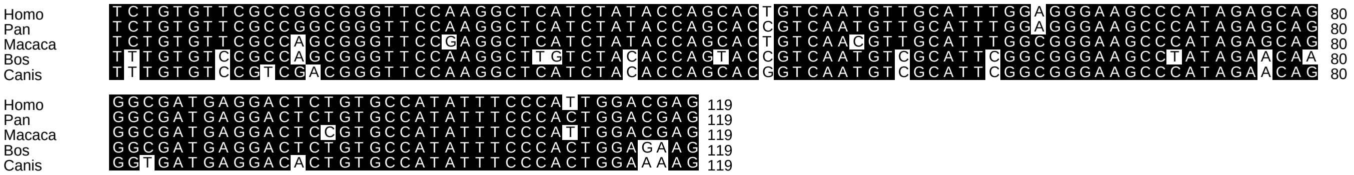

ENSG00000183921 exon 5

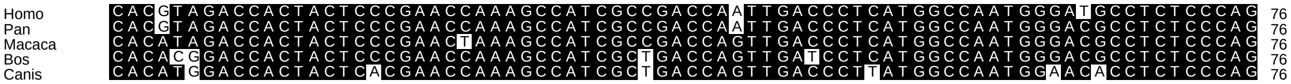

ENSG00000183921 intron 4

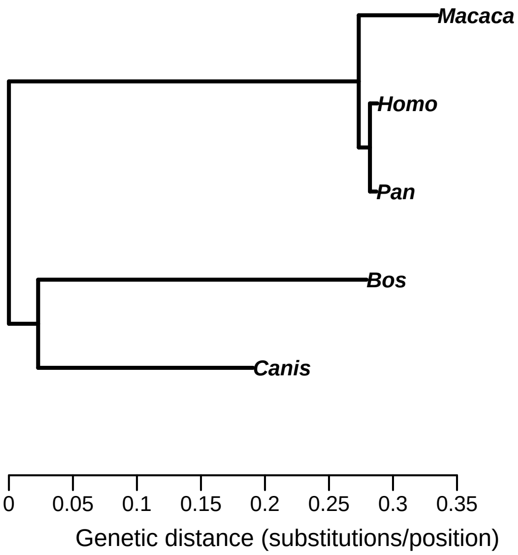

ENSG00000183921 intron 4

```
Homo   GTACCTGTCTTCCGGGAGAGGTGGCAGGACCCGCGGGCCCGGGACCCACAGCTCAGAGGGGAGGGATGGGTGGAAG-CAGGGTTGGTTTCACAACTCAG 99
Pan    GTACCTGTCTTCCGGGAGAGGTGGCAGGACCCGCGGGCCCGGGACCCACAGCTCAGAGGGGAGGGATGGGTGGAAG-CAGGGTTGGTTTCACAACTCAG 99
Macaca GTACCTGTCTTCCGGGAGAGGTGGCGGGGCCTGAGGGCCGGGGACCCACAGCTCAGAGGGTAGGAATGGGTGGAAGCCAAGATTGGTTTCACAACTCAG 100
Bos    GTACCTGGCTTCCTGGAGAGGTGGCAGGGC----GGACCTGGGACCCACAGTTCAAAGGGTAGGGATGGGCAG-AGCCAGGGTCAACTTCACAACTGAG 95
Canis  GTACCTGGCTTCCAGGAGAGGTGGCAGGGCCACTGGGCCTCGGACCCACAG------GGGTCCGGATGGGTGGAAACCAGGATCAACTTCACAACTCGG 94

Homo   GATACGTCCTAGCAGGAAGGGGCTCCCAAAACTGGGTGGCGTCAGCACTCTCCTGTTTCAGTTGGTCACACCTGCAGGGGCATTGAAATCAGACAGGTCT 199
Pan    GATACGTCCTAGCAGGAAGGGGCCCCCAAAACTGGGTGGCATCAGCACTCTCCTGTTTCAGTTGGTCACACCTGCAGGGGCATTGAAATCAGACAGGTCT 199
Macaca GATGCGTCCTAGCAGGAAGGGGCCCCCAAAACTGGCTGGCATCAGCACT--------------------------------------------------- 149
Bos    GGTGAGACCCAGGAGCAAAGAGTCCCC-AAATAGCATGGTTCAAGCCCT--------------------------------------------------- 143
Canis  GGTTAGTCCCATGAAGAGGGGATCCCTAAAACTGAGTAGCATGAGCCTT--------------------------------------------------- 143

Homo   GGCCTTAGCCTCAACCTGGCCGCATTCTACCCATGTGACCCTGACCCTGAGCAAATCACTTAAGCCCTTTGAGCC------------------------- 274
Pan    GGCCTTAGCCTCAACCTGGCCACATTCTACCCATGTGACCCTGACCCTGAGCAAATCACTTAAGCCCTTTGAGCC------------------------- 274
Macaca --------CCCCAACCTGGCCACATTCTACCCATGTGACCCTGACCCTGAGCAAATCACTTAAGCCCTTTGAGCCTCTGTTTTGTTTTTTGTTGTTTTTT 241
Bos    ---------------------------------------------------------------------------------------------------- 143
Canis  ---------------------------------------------------------------------------------------------------- 143

Homo   -------------TGTTTTTTTTTCTTTTCTTTTCTTTTGTTTTTTTGAGACAGA--------------------------------------------- 316
Pan    -------------TG-TTTTTTTTCTTTCCTTTTCTTTTCTTTTTTTGAGACAGA--------------------------------------------- 315
Macaca TACTTTTTCTTTTTCTTTTCTTTTTTTTTTTTTTTTTTTTTTTTTTGAGACGGAGTCTCGCTCTGTTGCCCAGGCTGGAGTGCAGTGGCACAATCTCAG 341
Bos    ---------------------------------------------------------------------------------------------------- 143
Canis  ---------------------------------------------------------------------------------------------------- 143

Homo   ---------------------------------------------------------------------------------------------------- 316
Pan    ---------------------------------------------------------------------------------------------------- 315
Macaca CTCACTGCAACCTCTGCCTCCCGGGTTCACGGCATCCTCCTGCCTCAGCCTCCCGAGTAGCTGGGACTACAGGCGCCGACCACCACGTCCCGTTAATTTT 441
Bos    ---------------------------------------------------------------------------------------------------- 143
Canis  ---------------------------------------------------------------------------------------------------- 143

Homo   ---------------------------------------------------------------------------------------------------- 316
Pan    ---------------------------------------------------------------------------------------------------- 315
Macaca TTGTATTTTTAGTAGAGACAGGGTTTCACGGTGTTAGCCAAGATGGTCGGTATCTCCTGACCTCATGATCCGCCTGCCTCGGCCTTCCAGAGTGCTGGGA 541
Bos    ---------------------------------------------------------------------------------------------------- 143
Canis  ---------------------------------------------------------------------------------------------------- 143

Homo   ------------------GTCTCACCCTGTCCCCAGGCTGGAGTGCAGTGGCGTGATTTTGGCTCACTGCAACCTCTGCCTCCTGGGTTCAATAGATTCT 398
Pan    ------------------GTCTCACCCTGTCCCCAGGCTGGAGTGCAGTGGCGTGATTTTGGCTCACTGCAACCTCTGCCTCCTGGGTTCAATAGATTCT 397
Macaca TTACAGGTGTGAGCCACCGTCTCACTCTGTCACCAGGCTGGAGTGCAGTGGCGCGATTTTGGCTGACTGCAACCTCCCCCTCCCAGGTTAAAGGGATTCT 641
Bos    ---------------------------------------------------------------------------------------------------- 143
Canis  ---------------------------------------------------------------------------------------------------- 143

Homo   CCTGCCTCAGCCTCCCAAGTAGCTGGGATTATAGGTGCATGCCACCACAACCAGCCAATTTTTGTATTTTTAGTAGAGACAGGGTTT-ACCATGTTGGCC 497
Pan    CCTGCCTCAGCCTCCCAAGTAGCTGGGATTATAGGTGCACGCCACCACAACCAGCCAATTTTTGTATTTTTAGTAGAGACAGGGTTT-ACCACGTTGGCC 496
Macaca CCTGCCTCAGCCTCCCAAGTAGCTGGGATTACAGGTGCACACCACCACGACCAGCCAATTTTTGTATTTTTAGTAGAGACAGGGTTTCACCATGTTGGCC 741
Bos    -CT-CCTACTTCA---------------------------------------------------------------------------GTCATGCCAGCA 166
Canis  -CTATTTTAGCTG---------------------------------------------------------------------------ACCATGCCGGCA 167

Homo   AGGATGGTCTCCATCTCTTGACCTCGTGATCTGCCAGCCTCAGCCTCCCAAAGTGCTGGGATTACAGGCGTGTTGTTTGTTTGTTTTGTTTTGTTTTGAG 597
Pan    AGGATGGTCTCCATCTCTTGACCTCGTGATCTGCCAGCCTCAGCCTCCCAAAGTGCTGGGATTACAGGCGTG----TTGTTTGTTTTGTTTTGTTTTGAG 592
Macaca AGGATGTTCTCCATCTCCTGACCTCATGATCTGCCAGCCTCAGCCTCCCAAAGTGCTGGGATTACAGGTG-GTTTCTGTTTTGTTTTGTTTTGTTTTGAG 840
Bos    AGG-----------------------------------------------------------------------------------------GCTTTGAA 177
Canis  GGG-----------------------------------------------------------------------------------------ATTTTGAA 178

Homo   ACAAGGTCTTGCTTTGCTCTGTCACTCAGACTGGAGTGCAGTGTCATGATCTCAGCTCACTGCAACCTCTGCCTCCCGGGTTCAAGTGATTCTCCTGCCT 697
Pan    ACAAGGTCTTGCTTTGCTCTGTCACTCAGACTGGAGTGCAGTGTCATGATCTCAGCTCACTGCAACCTCTGCCTCCCGGGTTCAAGTGATTCTCCTGCCT 692
Macaca ACAAGGTCTTGCTTTGCTCTGTCACTCAGACTGGAGTGCAGTGGCAGGATCTTAGCTAAATGCAACCTCCACCTCCCGGGTTCAAGTGATTTTCCTGCCT 940
Bos    A--------------------TCACACCAACCTGG----------------------------------------------------------------- 192
Canis  A--------------------TCACACAGACCTGG----------------------------------------------------------------- 193

Homo   CAGCCTCCCAGTAGCTGGGACTACAGTCGTGCGCCACCACGCCCAGCTAATTTTTGTATTTTTAGTGGATACAGGTTTCATTGAGTTGCCCAGGCTGGT 797
Pan    CAGCCTCCCAGTAGCTGGGACTACAGTCGTGCGCCACCACGCCCAGCTAATTTTTGTATTTTTAGTGGATACAGGTTTCATTGAGTTGCCCAGGCTGGT 792
Macaca CAGCCTCCCA-TAGCTGGGACT-------------ACCACGCCCAGTTAACTTTTGTATCTTTAGTGGATACAGGTTTCATTGTGTTGCCCAGGCTGGT 1026
Bos    -------------------------------------------------------------------------------CCTTGAACCAGCAAAGCAACT 213
Canis  -------------------------------------------------------------------------------CCTTGAGCCAGCCAGACCACT 214

Homo   CTCGAACTCCTGGGCTCAATCAATCCTCCCTCCTGCGGCTGTCAAAGTGCTGGAATTACAGGCATGAGCCACTGCACCCGGACAAGCCTCTGTTTCTTCA 897
Pan    CTCGAACTCCTGGGCTCAATCGATCCTCCCTCCTGGGGCTGTCAAAGTGCTGGAATTACAGGCATGAGCCACTGCACCCGGACAAGCCTCTGTTTCTTCA 892
Macaca CTCAAACTCCTGGGCTCAATCAATCCTCCCTCCTGGGCCTCCCAAAGTGCTGGGATTACAGTCATGAGCCACTGCACCCAGAAAAACCTCTGTTTCTTCA 1126
Bos    TTCTA-CTGTGGGACCC-------------------------------------------TGAGCAGATCACTTCACTTGG---AGGCTCTCCTTCTACA 266
Canis  TTCTATCTGTGGGAACC-------------------------------------------TGCGCAAGTCATTTTACTCTG---AGCCTCTATTTTTTCA 268

Homo   ACCCAGTATGGTAACACTATCACCATGGGGTTGTGCAGAGCAGGAAATAAGATAACCGATGTGAAAATGCCATGTGAACACAAAAGAGCCATGTGTAGGT 997
Pan    ACCCAGCATGGTAACACTATCACCATGGGGTTGTGCAGAGCAGGAAATAAGATAACCGATGTGAAAATGCCATGTGAACACAAAAGAGCCACGTGTAGGT 992
Macaca TCCCAGCATGGTAACACTACTGCCATGGGATTGTGCAGAGCAGGAAATAATTTAACCAATGTGAAAATGCCATGTGAACACAAAAGAGCCATGTGTAGGT 1226
Bos    CCTGGGCATGATAACACCACT-CTGTGGGGATGTGGAGAGCTGGAAATGAGACGAT-TATGTGAAAATGCCA--CGTGAGCACAAGGATTCCATGTAAAC 362
Canis  TCTGGGCATGACCACTCCACC-CCATGGGGGTGCAGAGAGCGGGAAATGAGATGATGTATGTGAAAAAAACA--CAAACACTAAAGAACTAGGTGGAAAC 365

Homo   AATTCATCCAGCATGTGTCCAGCATCAGCCTTGCAAGGACAGGTGTCTGGGGCTGGCAGGAGCCTCCCTGAGTC-GGCTGCCCCTGGTCATTGTTACTGC 1096
Pan    AATTCATCCAGCATGTGTCCAGCATCAGCCTTGCAAGGACAGGTGTCTGGGGCTGGCAGGAGCCTCCCTGAGTC-GGCTGCCCCTGGTCATTGTTACTGC 1091
Macaca AATTCATCCCGCGTGTGTCCAGCATCAGCCTTGCAAGGACAGGTGTCTGGAGCCGGCAGGAGTCTCCCTGAGTTGGGCTGCCCCTAGTCATTGTTACTGC 1326
Bos    AGTCCATCTGACATGCATCCAGCATCACATC-CCAGTGCAGCCGATGTCCCAGTGCAGGCGACGTGGCTGACG-GGAAGCTCC-----ATTGTTACTGC 455
Canis  AGGTCATCCGACTCACACGTTGCACCTGCATC-CCGGAGCAGGCCCTGGGGGCCGGCAAGAGCCTCCCTGAGTG-GAAGAGCCCTGGTTGTTGTTACTGC 463

Homo   TCCGAACCCAGTGCTCCTCTGCTCCTGTGGCTCTGGAAGGAGATGCACTGTTCTCCCACCCAGGGGGAGAGGAAAGGCCATGGGGCTCAGGGCCTGGCCA 1196
Pan    TCCGAACCCAGTGCTCCTCTTCTCCTGTGGCTCTGGAAGGAGATGTACTGTTCTCCCACCCAGGGGGAGAGGAAAGGCCATGGGGCTCAGGGCCTGGCCA 1191
Macaca TCCGAACCCAGTGCTCCTCTGCTCCTGTGGCTCTGGAAGGAGATGCACTGTTCTCCCACCC-GGGGGAGAGGAAAGGCCATGGGACTCGGGGCTTGGCCA 1425
Bos    TCTGAACT-----CTCTCCTCTGTTCGTGG----------------------CTCCCACCTAGAGGGAAAGGAGCGGCAGTAGGGCTCAGGGCTTGGCCA 528
Canis  TCTGAGCT-----CTGCTC-----CCGTGG----------------------CTCCCACCCCAAGAGAAAAGAAGGGCACTGAGGCTCAGGGCTTGGCTG 531

Homo   TATCCCAGTG-------GGAGTCCCCAGGGAAGGTGCAGCCCTGG---------------------------------GGCTGGGCAGGCATTGCCAGCA 1256
Pan    TATCCCAGTG-------GGAGTCCCCAGGGAAGGTGCAGCCCTGG---------------------------------GGCTGGGCAGGCATTGCCAGCA 1251
Macaca TATCCCAGTG-------GGAGTCCCGAGGGAAGGTGCAGCCCTGGGGCTGGGC-----------------------CAGGCTGGGCAGGCATTGCCAGCA 1495
Bos    TTTCCTGGTG-------AGGGTCCCAGAAGTGAACTCCCATATGGTCAGAAGCAAAAGTGCAGCCCTGGGGCCAGGAGGGCTGGGT-GGTGTGATCAGCA 620
Canis  CTTCCTGGTGGGGGAGTGGGGGCCCAGAATCTAGC-CATGTGTGGTCTGAAGC-----------------------AGGGCTGGCCAGGCCTTGCCAGCA 607

Homo   GCATTGCTGACCCT-CTCTGGTGGTTCTGTTTTTCCTG---GCCTCTGGCAG 1304
Pan    GCATTGCTGACCCT-CTCTGGTGGTTCTGTTTTTCCTG---GCCTCTGGCAG 1299
Macaca GCATTGCTGACCCT-CTCTGGTGGTTCTGTTTGTCCCG---GCCTCTGGCAG 1543
Bos    GTGTCCCTGACCCC-ATCTGGTAGCTCTATTCTCCCTG---TCCTTGAACAG 668
Canis  GTGTCACTGACCCCTACCTGGTGGCTCTTTTCTTCTTGTCCTCCTCCGGCAG 659
```

ENSG00000103316 intron 1

Description: Mu-crystallin homolog (CRYM)
Intron number: 1
Human chromosome: 16
Intron start (bp): 21196407
Human intron length : 497
Intron alignment length: 509
Flanking exons length (upstream/downstream): 170/154
SNP density: 0.006036
K tree score: 0.0871
Scaling factor: 1.0063
Human-chimpanzee distance: 0.008108
Total primate branch length: 0.0869

ENSG00000103316 exon 1

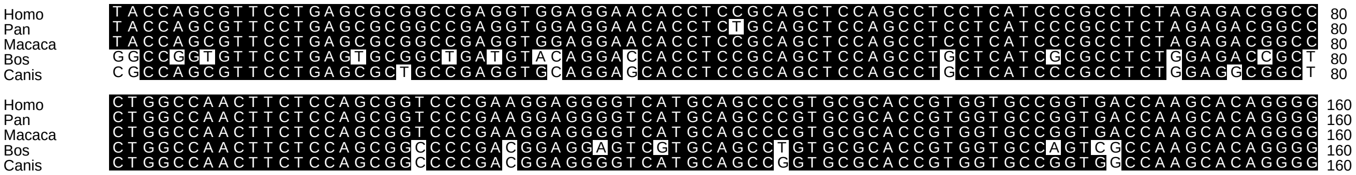

ENSG00000103316 exon 2

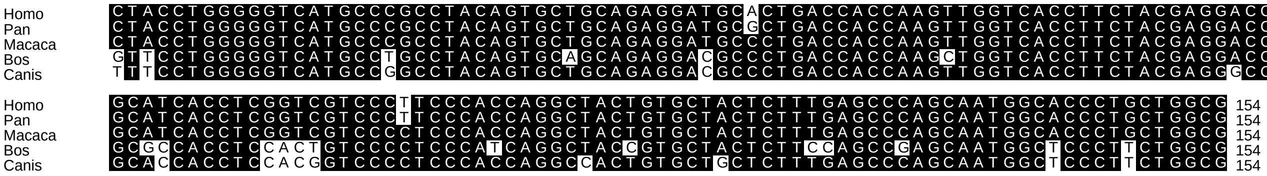

ENSG00000103316 intron 1

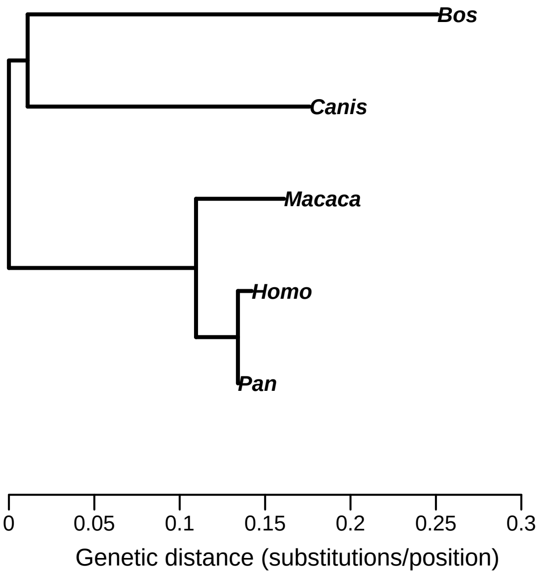

ENSG00000103316 intron 1

```
Homo    GTGAGTGAGGAGGGTCGGGGTCAGGGGCAGGAGAAACTGGAAAGCGGGGAAAGGAG----GAGCAGGAAGAGGAGGGGTGGGAGAAGGGAAGAGACGGCT 96
Pan     GTGAGTGAGGAGGGTCGGGGTCAGGGGCAGGAGAAACTGGAAAGGGGGGAAAGGAG----GAGCAGGAAGAGGAGGGGTGGGAGAAGGGAAGAGACGGCT 96
Macaca  GTGAGTGAGGAGGATCGGGGCCAGGGGCAGGAGAAACTGGAAAGGGGGAAGAGGAG----GAGCAGGAAGAGGAGCGGTGGAAGAACGGAAGAGACGGCT 96
Bos     GTAAGTGAGGAAGGCCGGGTCC-GGGGCTGGAGAAACTGGAAAGGAGGAAGGGGAG----GGGTGAGAAGGGAAGCAGCAGGAGAAAAGAAAGGAGGGCT 95
Canis   GTGAGTGAGGAAGGCCGGGGCTAAGGGCTGGAGGAACTGGAAAGGTGGAAAGGGAGGCATGCATGCGAAGGGAAGCAGCGGGGGAAAGGAAGAGAGAGCT 100

Homo    TGCCTAACCGTTGC---TAACAGCTGCCACTTTTAATAGGCGCTACCCGGTGTGCTAAGTGGGCGTTTTACAGGTGTTACCTTGTTTATTTCAGCACACT 193
Pan     TGCCTAATCGTTGC---TAACAGCTGCCACTTTTAATAGGCGCTACCCGGTGTGCTAAGTGGGCGTTTTACAGGTGTTACCTTGTTTATTTCAGCACACT 193
Macaca  TGACTAATTGTTGC---CAACAGCTGCCACTTTTAATAGGCGCTACCCGGTGTGCTAAGTGGGAGCTTTACGGGTGTTACCTTGTTTATTTCAGCGCACT 193
Bos     TCCCTAATCCCTACTAATAACACCTTTCACCT--AATTTGAGCAACCCACT-------GTGGGCGCTTTAAAGCTGTTATCTGGTTTCATTCAACACACT 186
Canis   TGCCTAATCATTGCTAGTAACAGCTGCCACCTAATATTTGCGCTCTCGGCCGTGCCAAGTGGATGCTTTTCAAGGGTTATCTTGCTTAATTCAACACATG 200

Homo    CTCCACCTAGGGAGGTATTGATACCACCATTTGATTGGTGGAAAACTGAGACTCAGAGAGGTTAAGTGACTTACCTAAGCTTAC-ACAGCTACTAAGTGG 292
Pan     CTCCACCTAGGGAGGTATTGATGCCACCATTTGATTGGTGGAAAACTGAGACTCAGAGAGGTTAAGTGACTTACCTAAGCTTAC-ACAGCTACTAAGTGG 292
Macaca  CTCCACCTGGGGAGATATTGATGCCACCGTTTTATTGATGGAAAACTGAGACTCAGAGAGGTTAAGTGACTTACCTAGGCTTAC-ACAGCTACTAAGTGG 292
Bos     CCATATGAACGTGGCTATTGATGCAGCTATTTGCTGATGGGAAAACCGAAGACTCAAGAGGTGAAGCGAATGACCCAAGGTTATGGCAGCTAGTAAGTGG 286
Canis   CTCCATATGGGTAGGTGTTGATGGAATCATTTTACTGATGGGGAAAATGGAGACTCAGAGGTTAAGTGACTTACCTAAGGCTAC-ATCGGTAGATAGTGG 299

Homo    CAGAGCTGGAAAGG-TTTTCTGATCCAGAGCCAGACTTCTGAACATACTGTGCATAGTGTCTAAGGGAAGGGCA---GAGGGATGCGGGGGCTGGAAGAG 388
Pan     CAGAGCTGGAAAGG-TTTTCTGATCCAGAGCCAGACTTCTGAACATACTGTGCATAGTGTCTAAGGGAAGGGCA---GAGGGATGCGGGGGCTGGAAGAG 388
Macaca  CAGAACTGGAATGG-TTTTCTGATCCAGAGTCAGACTTCTGAACATATTATGCATAGTGTCTAAGGGAAGGGCAGAGGAGGGATGCGGGGGCTGGGAGAG 391
Bos     CAGAACTGGGGTGGTTTTTCTCATCCAGAGGCAGACT--CGAACGTACTCTGCACACTGCTAAAGGCAACGGCAGAGGGATGGGGACAAAGGAGGGAGAA 384
Canis   CAGAACTAGAATGGTTTTTCTCATCCAAAGCCAGACTGTCCAACATACTCTGCAC--TGCCTAAAGAAAGGGCAGAGGAGAGGCAGGGGGGCGGGAG--- 394

Homo    GGCCAGTGGGGTGGTGAGGAAGGAAAGAAAAAGGAAAGTATATGGATACTTTCTAAGAGACCCCAGCATTGATGGGAAGGGGCTGAAGCTGCTCCCTCTC 488
Pan     GGCCAGTGGGGTGGTGAGGAAGGAAAGAAAAAGGAAGGTATATGGATACTTTCTAAGAGACCCCAGCATTGATGGGAAGGGGCTGAAGCTGCTCCCTCTC 488
Macaca  GGCCAGTGGCGCCGTGACGAAGGAAAGAGAAAGGAGGGTATATGGGTACTTTCTAAGAGACCCCAGCATTGATGGAAACAGGCTGAAGCTGCTCCCTCTC 491
Bos     GGCCAGTAGGGCAGTGAGTGGGGAAAGAGAAAGAAAGGGACATAGGGACTTGACAGGATGTCTCAGCATCGACAGGAAGGGGTTGAAGTTGCTCCCTCTC 484
Canis   ---------------GACACAGGAAAGAGAAAGGAAGGTCTATGGGGACTTGATCGCACACCCCAGTGTTGGTAGGAAGGGGTTGAAGCTACTCCCTCTC 479

Homo    TCCTTGTAG 497
Pan     TCCTTGTAG 497
Macaca  TCCTTGTAG 500
Bos     TTCTTCTAG 493
Canis   TCCTTGTAG 488
```

ENSG00000186815 intron 21

Description: two pore segment channel 1 (TPCN1)
Intron number: 21
Human chromosome: 12
Intron start (bp): 112212432
Human intron length : 708
Intron alignment length: 837
Flanking exons length (upstream/downstream): 116/47
SNP density: 0.001412
K tree score: 0.0537
Scaling factor: 0.6424
Human-chimpanzee distance: 0.013167
Total primate branch length: 0.0868

ENSG00000186815 exon 21

```
Homo    CACGAGTACAGTGGCAGATGCCTACCGCTGGCGCAACCACACCGTGGGCAACAGGACCGTGGTGGAGGAAGGCTACTATT 80
Pan     CACGAGTACAGTGGCAGATGCCTACCGCTGGCGCAACCACACCGTGGGCAACAGGACCGTGGTGGAGGAAGGCTACTATT 80
Macaca  CACGAGTACAGTGGCAGACGCCTACCGCTGGCGCAACCACACCGTGGGCAACAGGACCGTGGTGGAGGAAGGCTACTATT 80
Bos     TACAAGCACAGTGGCAGACGCCTACCGCTGGCTGAACCACTCCGTGGGCAACAGGACCGTCGTGGAGGAGGGCTATTACT 80
Canis   TACGAGCACAGTATCTGATGCCTACCGCTGGCTCAATCACACCGTGGGCAACAGGACCGTTGTGGACGAAGGCTACTACT 80

Homo    ATCTCAATAATTTTGACAACATCCTCAACAGCTTTG 116
Pan     ATCTCAATAATTTTGACAACATCCTCAACAGCTTTG 116
Macaca  ATCTCAATAATTTTGACAACATCCTCAACAGCTTTG 116
Bos     ATCTCAATAATTTTGACAACATCCTGAACAGCTTTG 116
Canis   ATCTTAATAACTTTGACAACATCCTAAACAGCTTTG 116
```

ENSG00000186815 exon 22

```
Homo    TGACCCTGTTTGAGCTCACAGTTGTCAACAACTGGTACATCATCATG 47
Pan     TGACCCTGTTTGAGCTCACAGTTGTCAACAACTGGTACATCATCATG 47
Macaca  TGACCCTGTTTGAGCTCACAGTTGTCAACAACTGGTACATCATCATG 47
Bos     TGACCTTGTTCGAGCTCACGGTTGTCAACAACTGGTACATCATCATG 47
Canis   TGACCTTGTTCGAGCTCACAGTTGTCAACAACTGGTACATCATTATG 47
```

ENSG00000186815 intron 21

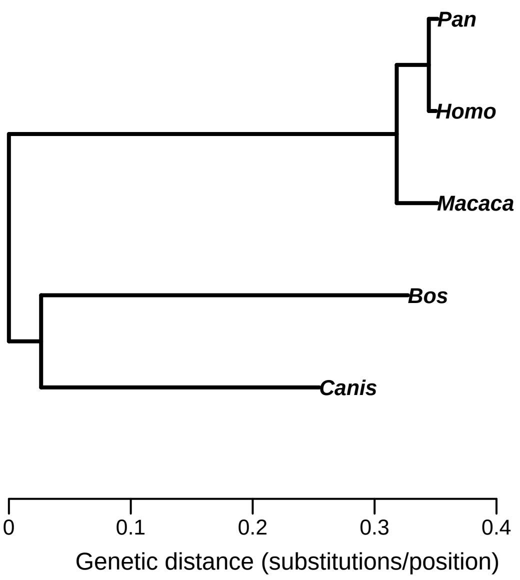

ENSG00000186815 intron 21

```
Homo    GTGAGTGGGAAAAATCACAGGGGGCACATTCCCTGGGGACCCCACCCTTGTCACCCTTGTCA-CCACGGGTCCCAGAGGTGGTGTAGC--AAGTGTCT-- 95
Pan     GTGAGTGGGAAAAATCACAGGGGGCACATTCCCTGGGGACCC---------CACCCTTGTCA-CCACGGGTCCCAGAGGTGGTGTAGC--AAGTGTCTGG 88
Macaca  GTGAGTGGGAAAAATCACAGGGGGCACATTCCCTGGGGACCC---------CGCCCTTGTCA-TCACGGGTCCCAGAGGTGTTGTAGCAAAAGTGTCTGG 90
Bos     GTGAGTACAGGAAGCCCAGGCGGG-------------------------------GGTGGCACCCACAGGTCCCAGGTGCCGGGGAGTGCAGCTGGCCGA 69
Canis   GTGAGTGCAGAGGCCACTGGTGGG-------------------------------ACAGTCA-CTGCAGGCCCCAGGTGTGGCCTAGCAGAGCTGTCTGA 68

Homo    ----------------------GGGCCACACTTTCTGGAAGCCTCCCTGGCCCAGGTGGAGGACTTGGTCTGCCCAGCTTTGGCAGTACAGTCCAGGCTG 173
Pan     G--CCTCTCCCTCCAGCACTGAGGGCCACACTTTCTGGAAGCCTCCCTGGCCCAGGTGGAGGACTTGGTCTGCCCAGCCTTGGCAGTACAGTCCAGGCTG 186
Macaca  G--CCTCTCCCTCCAGCACCGAGGGCCACACTTTCTGGAAGCCTCCCCGGCCCAGGTGGAGGACATGGTCTGCCCAGCCTTAGCAGTGAAGTCCAGGCCG 188
Bos     GGCCTCCCCCTCCCAGGGCATGTGCTACGCTTGCTCAAGTCCTCTCTGGCCCCCACGGAGCGTCTGGTTTGCCACCCCAGGGCTCACTTGGTC--GCCA 167
Canis   CGCCTCCCCCTTCCT-GGCCATTTGCTATGCTTTCTGGAGTCCTCCTGAGCCTCCATGGAGGCTGTGGTTTACCCCGCCACAGGGTGCAGGCCAGGCCA 167

Homo    GATCCTGGAGCTCACTCAGTTGCCTGATTCCACTCTTTCCTGATATTTTCTCAGTA-CCGGGGAGGACAAGAGTTGCAAGCCTTAGCTGGGCGGGGT--- 269
Pan     GATCCTGGAGCTCACTCAGTTGCCTGATTCCACTCTTTCCTGATATTTTCTCAGTA-CCGGGGAGGACAACAGTTGCAAGCCTTAGCTGGGCGGGGT--- 282
Macaca  GATCCCGGAGCTCACTCGGTTGCCTGATTCCACTCTTTCCTGATACTTTCTCAGTATTTGGGGAGGAGAAGAGCTGCAAGCCTTAGCTGGGCGGGGT--- 285
Bos     GGTTTTCCTGCATCTGCTGTCCC-----------------------TTTCCCAGCA-TTGGGGAGCAGCAGAGCTTCACGCTTGGCTGGGA-GGGGTACA 242
Canis   GATCCCAGCGCTCACTCACCACC-----------------------AGGTTTCGTT-CCTGGGAGCAGAGGGGCTCCGTGCTTTGCCGAGAGGGGGGAA 243

Homo    --------------------CCCCTCATCACAGTGCCACTTGCAGAATGGGACCCAGCCACACCACTGCTGCTGCAGCTGCACTGCC-CCCTGGCTCAGA 348
Pan     --------------------CCCCTCATCACAGTGCCACTTGCAGAATGGGACCCAGCCACACCACTGCTGCTGCAGCTGCACTGCC-CCCTGGCTCAGA 361
Macaca  --------------------CCCCTCATCACACTGCCACTTGCAGAATGGGACCCAGCCACACCAATGCTGCTGCAGCTGCACTGCC-CCCCAGCTCAGG 364
Bos     GCCTGATGCTGGGGAGCCACCCCCCG-------------TGTCACGGGGGTGCCCAGCCAC-----------AGTACCCTGTGCCA--CCCAGGCCAGG 315
Canis   GCCTGAGGCCAGGGAACCCACCCCCAGTCACAACCCCACAGTTGCAGGGTGGGACAGCCATACCAACCTGCCAAGTGCCCTATGCTGACCCCAGCTCAGG 343

Homo    CTGCACACAGCCCT-CTGCCCACTCCCATGAAGAGGC--GAACACAGCCAAACCGCCTCTCCAGAAAAAGGGTGTGCCCC-CATGACACCCCAGATCTGC 444
Pan     CTGCGCACAGCCCT-CTGCCCATTCCCATGAAGAGGC--GAACACAGCCAAACCGCCTCTCCAGAAAAAGGGTGTGCCCC-CATGACACCCCAGATCTGC 457
Macaca  CTGCCCACAGCCCT-CTGCCCACCCCCATGAAGAGGC--GAACACAGCCAAACCGCCTCTCCAGAAAAAGGGTGTGCCCC-CATGACACCCCAGATCTGC 460
Bos     CC-CACGCAGCTCTGCTCCTGGCTTCCACGGAAAAGCTGGG--------AGGGCAGGGGGCCACGGAGGGATGGGGCCTTGCCAGACACAGCTTTCTCAG 406
Canis   CC-CACATGGCAGTGCTCCTGGGTTCCATGGAGAGGCTGGGGCAGGGGCAGGGCACAGCTCAAGAGGGAAAGGGAGCCCCTCCTGGCATCCTGGATCTAC 442

Homo    ACC------------------------CAGTAAGATCCAGATAGTCTGATGT-------------------------------------GGCACAGGCTGCT 485
Pan     ACC------------------------CAGTAAGATCCAGATAGTCTGATGT-------------------------------------GGCACAGGCTGCT 498
Macaca  ACC------------------------CAGTAAGATCCAGATGGTCTCGTGT-------------------------------------GGCACAGGCTGCC 501
Bos     AACATCCCCTCATGACATCCTAGACTGAGGAAGGTCTGGGGGGCTCTTCCTCAGGATCCC---------CACTGCAGC---------ATCTCGAGCTGCT 488
Canis   ACCAAGTCAGGTTGGAGTAGCTCTCGTGGGAGAGTTTTTGTGGTCCCTCTCCTGTGTCTCCCACTCTTTCCCTGCTGCCCAGCCCCAGCCCTGGTTTGTC 542

Homo    G-AGGCAGAGGGTGTGTTCTCTTGGGGGCAGGCCATGATG--------TCCTGTGGAGCCAGCGCCCTAGGAGTGCCTTGCCTGGTCTTTCCTGGAGCTC 576
Pan     G-AGGCAGAGGGTGTGTTCTCTTGGGGGCAGGCCACAATG--------TCCTGTGGAGCCAGTGCCCTAGGAGTGCCTTGCCTGGTCTTTCCTGGAGCTC 589
Macaca  G-AGGCAGAGGGTGTGTCCTCTTGGGGCCGGGCCACAATG--------TCCTGTGGAGCCAGCGCCCTAGGAGTGCCTTGCCTGGTCCTTCCTGGAGCTC 592
Bos     G-AGGCAAAAGACGCATGCTTTGGGGGCCAGGCCGAGACGGGACCACTTCCTATGGGGGAGGCGCTCTGGAAGGGTCTTGGCCGCTCTCT-CTGGAGCTC 586
Canis   ATAGGCACAGGGAGCGCCCTTTGGGGGACAGGCCAAGATGGCAGCACTCCCTGTGGGGTGGCGCTCAGGGGAAGCTTTGGCTGATACTT-CTAGGACT- 640

Homo    ACAACCCAAGCGGTCATATGGTGACCCTCTCATCTTAGCACATGGCACCCAGGATAGGTGGCAAGAGGCCACTTGAAATTGACTTTGAAGTG--GGGTCT 674
Pan     ACAACCCAAGTGGTCATATGGTGACCCCCTCATCTTAGCACATGGCACCCAGGATAGGTGGCAAGAGGCCACTTGAAATTGACTTTGAAGTG--GGGTCT 687
Macaca  ACAACCCAAGCGGTCATATGGTGACCCCCTCATCTCAGCACACGGCACCCAGGATAGCTGGCAAGAGGCCACTTGAAGTTGACTTTGAAGTG--GGGCCT 690
Bos     ACAGCCCAGGCTGGCAGAC----------------AAGTGCCTGGTACCCAGGATAGGTGGAGAGAGGCCACT--GACCCCACTTTGCAAAAGCGGGTCT 668
Canis   -CAGCCCAGGCTGGCAGACGGGGATGCCATCATTTAGGTACATGGCACCCCGGATAGGTGGAGAGAGGCTACTTGAAATAGACTTTGAAAAAGTGATTCT 739

Homo    CATCAGGATGCTTCTTTCTCTC---TTCCCTCGGCAG 708
Pan     CATCAGGATGCTTCTTTCTCTC---TTCCCTCGGCAG 721
Macaca  CATCAGAATGCTTCTTTCTCCC---TTCCCCTGGCAG 724
Bos     GATCAAGACGCTTCTGTCTCTCCCTTCTGTTTGGCAG 705
Canis   GATTGAAATACTCCTCGCTCTCCCTTGCCTTTGGCAG 776
```

ENSG00000113492 intron 2

Description: Alanine--glyoxylate aminotransferase 2, mitochondrial precursor (AGXT2)
Intron number: 2
Human chromosome: 05
Intron start (bp): 35075371
Human intron length : 1066
Intron alignment length: 1514
Flanking exons length (upstream/downstream): 89/185
SNP density: 0.006567
K tree score: 0.0549
Scaling factor: 0.9169
Human-chimpanzee distance: 0.028843
Total primate branch length: 0.0865

ENSG00000113492 exon 2

```
Homo    TAGGTACTTCCCGGACATCAGTAACCAAGCTCAGTCTTCATACAAAGCCCAGAATGCCTCCATGTGACTTCATGCCTGAA 80
Pan     TAGGTACTTCCTGGACATCAGTAACCAAGCTCAGTCTTCATACAAAGCCCAGAATGCCTCCATGTGACTTCATGCCTGAA 80
Macaca  ---GTGCTTCCTGGACATCAGTAACCAAGCTCAGTCTTCATACAAGGCCCAGAATGCCTCCATGTGACTTCATACCTGAA 77
Bos     CCCGTGCTTTCTGGACATCTGTGACCAGGTGCGGTCTTCATACAAAGCCCAGCATGCCGCCATGTGACTTCACACCCGAA 80
Canis   TAGGTGCTTTCTGGACATCTGTGACCAAGCTTGGTCTTCATACAAAACCCAGAATGCCTCCATGTGACTTCACACCTGAA 80

Homo    AGATACCAG 89
Pan     AGATACCAG 89
Macaca  AGATACCAG 86
Bos     AGATACCAG 89
Canis   AAATACCAG 89
```

ENSG00000113492 exon 3

```
Homo    TCCCTTGGCTACAACCGTGTCCTGGAAATCCACAAGGAACATCTTTCTCCTGTGGTGACGGCATATTTCCAGAAACCCCT 80
Pan     TCCCTTGGCTACAACCGTGTCCTGGAAATCCACAAGGAACATCTTTCTCCTGTGGTGACGGCATATTTCCAGAAACCCCT 80
Macaca  TCCCTTGGCTACAACCGTGTCCTGGAAATCCACAAGGATCACCTTTCTCCTGTGGTGACGGCGTATTTCCAGAAACCCCT 80
Bos     TCCCTGGCCTATAGCCGAGTCCTGGAGATCCATAAGCAACATCTCTCTCCTGTGCACACCGCCTATTTCCCGGAGCCCCT 80
Canis   TCTCTCACCTATGAACGTGTCCTGGAGATCCACAAGCAACATCTTTCTCCCATAACTGACGGCGTATTTTCCAAAGCCCTT 80

Homo    GCTGCTCCACCAGGGGCACATGGAGTGGCTCTTTGATGCTGAAGGAAGCAGATACCTGGATTTCTTTTCCGGGATTGTTA 160
Pan     GCTGCTCCACCAGGGGCACATGGAGTGGCTCTTTGATGCTGAAGGAAACAGATACCTGGATTTCTTTTCCGGGATTGTTA 160
Macaca  GCTGCTCCATCAGGGGCACATGGAGTGGCTCTTTGATGCTGAGGGAAACAGATACCTGGATTTCTTTTCCGGGATTGTTA 160
Bos     GTTGCTCCACCAGGGACACGTGGAGTGGCTCTTTGATCATGAGGGAAACAGATACCTGGATTTCTTTTCTGGGATTGTCA 160
Canis   GTTGCTCCACCAGGGTCACAAGGAATGGCTATTTGATTATGAGGGAAACAGATACCTGGATTTCTTTTCTGGGATTGTCA 160
```

ENSG00000113492 intron 2

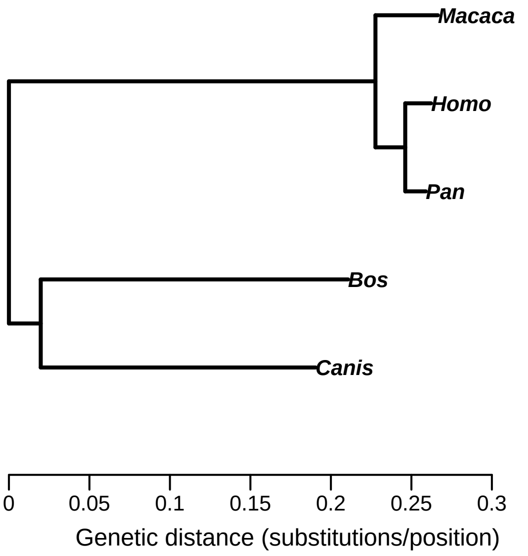

ENSG00000113492 intron 2

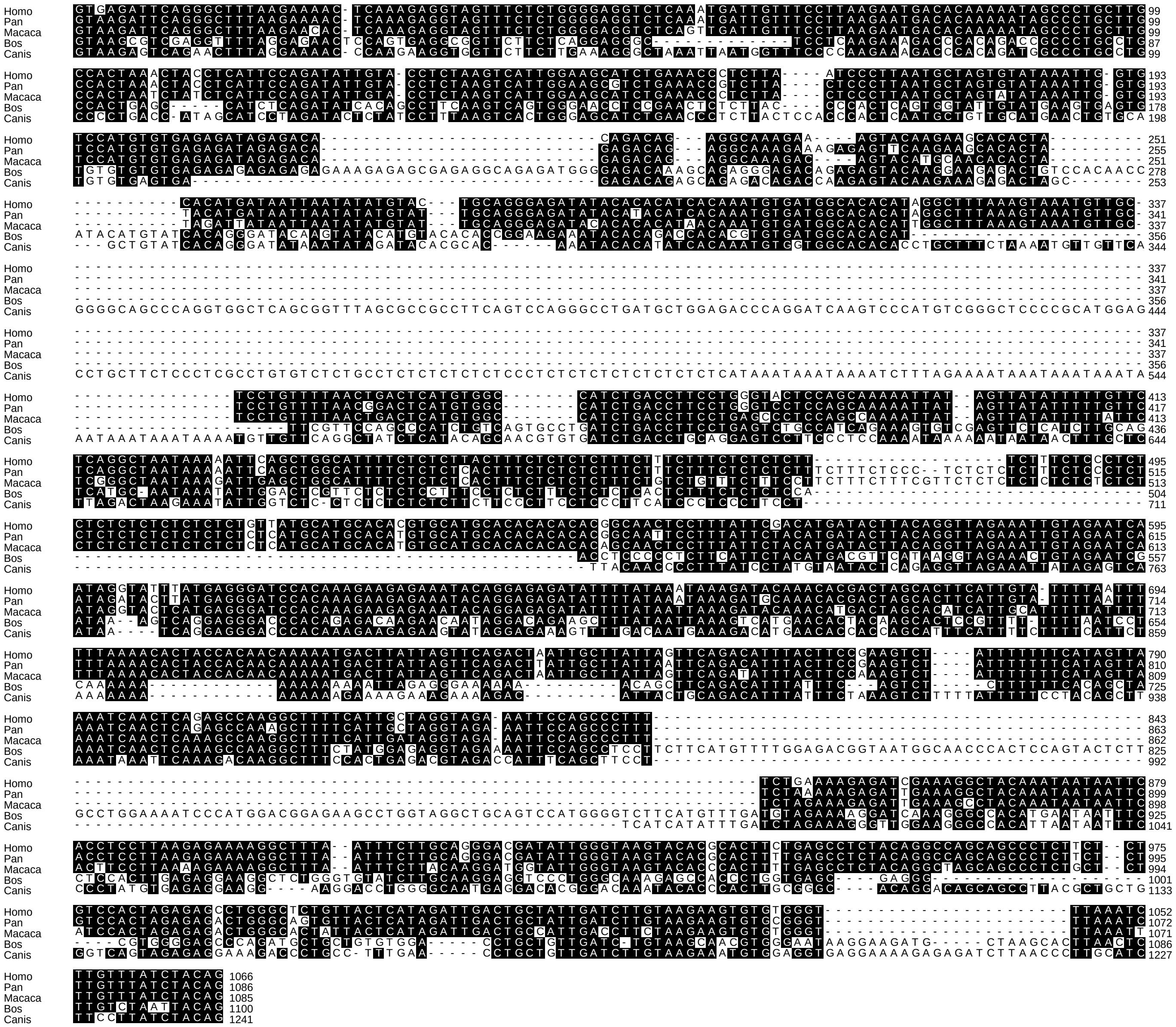

ENSG00000073756 intron 7

Description: Prostaglandin G/H synthase 2 precursor (PTGS2)
Intron number: 7
Human chromosome: 01
Intron start (bp): 184911940
Human intron length : 282
Intron alignment length: 304
Flanking exons length (upstream/downstream): 247/287
SNP density: 0.007092
K tree score: 0.075
Scaling factor: 0.8251
Human-chimpanzee distance: 0.021806
Total primate branch length: 0.0865

ENSG00000073756 exon 7

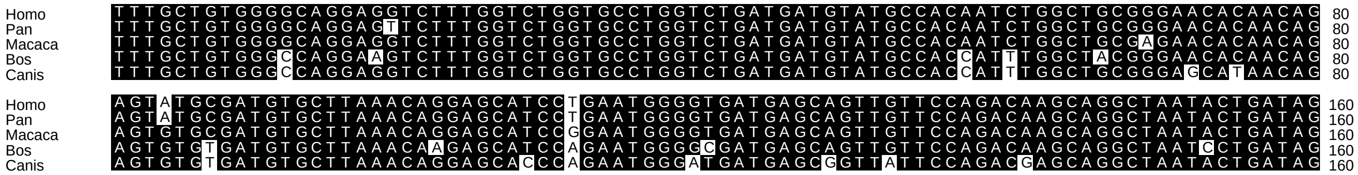

ENSG00000073756 exon 8

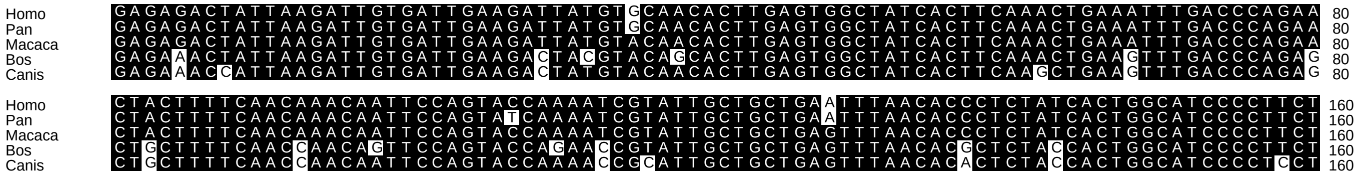

ENSG00000073756 intron 7

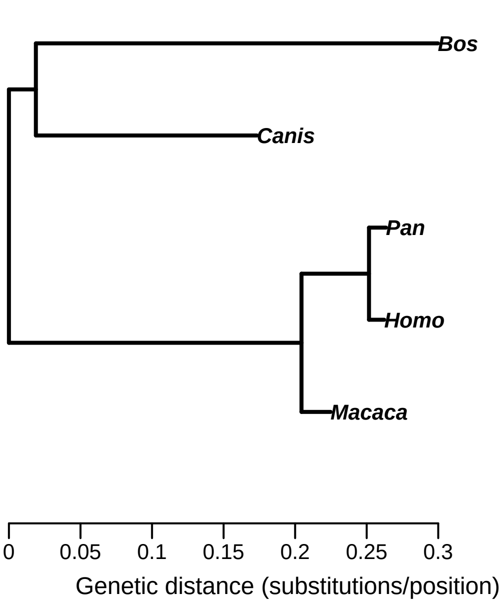

ENSG00000073756 intron 7

```
Homo    GTAAACA-----AGAAAATGATTTATATAAAACCCTCTTCCCCAGGGAAAATTAGTGTGCTATCTTTGTTATGTTTTGA-GTAA--ATGACAAGATGTGG 92
Pan     GTAAAAA-----AGAAAATGATTTATATAAAACCCTCTTCCCCAGGGAAAATTAGTGTGCTATCTTTGTTATGTTTTGA-GTAA--ATGACAAGATATGG 92
Macaca  GTAAACA-----AGAAAATGATTTATATAAAACCCTCTTCCCCGGGGAAAACTAATGTGTTACTTTTGTTATGTTTTGA-GTAA--CTGACAAGATGTGG 92
Bos     GTAAGCTAGAAGAGCAAATCACTTAAAGAAAATCCTCTTCCTCAAAGAAAACTAACTTGTTTCCTTTGCTTGGGAGGAATATAATTCTGGGGGAGTGTCA 100
Canis   GTAAGCAAGAAGAGAAAATAATTTCAATAAAACCCTCTCCCCCAGAGAAAGCTGGCATGTTACTTTTTATCCGTTTTAA-GTAATTCTGGGAGAATGTAG 99

Homo    TAAATGAAAACTCACACATTCTATATACATTAAATATGTAAGCATGACTGATAAAATAGCTATCTTTTGATACTGACAA------GGAAGAAAACAGAAA 186
Pan     TAAATGAAAACTCACACATTCTATATACATTAAATATGTAAGCATGACTGATCATATAGCTATCTTTTGATACTTACAA------GGAAGAAAACAGAAA 186
Macaca  TAAAAGAAAACTCACACACTCTATATTCATTAAATATGCAAGCATGACTGATCATATAGCTATCTTTTGATATTGACAA------GGAAGAAAACAGAAA 186
Bos     TGCATGTACAATCACCTGCTCCATATTCATTCAGTCCTTCAGCTGGATTGCTCAGACACCTACCTCTCAAT--------------GGAGGAAAAGAAAAT 186
Canis   TGAATGGAAAATCACATGCTCCATAT---TTCAGTCTTTGAGCGTGATTGAGGGTATTAATACTTTTCTGTTTTGTTGGTAAACCAGGACAAAATAGAAG 196

Homo    TGA---AGGAATAGCAAATTTTAAAAATTGCATTCCAGTTGCTTGAAAGCTTGTGATCAGATGCAATAAATGTTTTTATTATTT-----ATTTTGTGCAA 278
Pan     TGA---AGGAATAGCAAATTTTAAAAATTGCATTCCAATTGCTTGAAAGCTTGTGATCAGATGCAATAAATGTTTTTATTATTT-----ATTTTGTGCAA 278
Macaca  TGA---AGGAGTAGCAAATTTTAAAAATTGCATTCCAATTGCTTGAAAGCTTGTGATCAGA-AAAATAAATGTTTTTATTCTTT-----ATTTTATGCAA 277
Bos     CTAGATAGGAATGCCAAATGACCA--GTTGCATTTCAGTTGCTTGAACATTTGTGAGCAGAAAGAATTCATGTTTTCAATTTTTT----ATTTTGTGCCT 280
Canis   TGA---AAGAATAGCAGATTAACAAGATTGCATTTCAGTTGCT----------TGATCAGA--GAATTAATGCTCTAATTTTTTTGTTGTTGTTGTGTAA 281

Homo    ATAG 282
Pan     ATAG 282
Macaca  ATAG 281
Bos     GTAG 284
Canis   ATAG 285
```

ENSG00000164591 intron 2

Description: Myozenin-3 (MYOZ3)
Intron number: 2
Human chromosome: 05
Intron start (bp): 150030394
Human intron length : 958
Intron alignment length: 1253
Flanking exons length (upstream/downstream): 155/54
SNP density: 0.007307
K tree score: 0.0194
Scaling factor: 1.033
Human-chimpanzee distance: 0.009732
Total primate branch length: 0.0864

ENSG00000164591 exon 2

```
Homo    TCCCTACGCTGGACCTGGGCAAGAAGCTGAGCGTGCCCCAGGACCTGATGATGGAGGAGCTGTCACTACGCAACAACAGA 80
Pan     TCCCTACGCTGGACCTGGGCAAGAAGCTGAGCGTGCCCCAGGACCTGATGATGGAGGAGCTGTCACTACGCAACAACAGA 80
Macaca  TCCCTACGCTGGACCTGGGCAAGAAGTTGAGCGTGCCCCAGGACCTGATGATGGAGGAGCTGTCACTACGCAACAACAGA 80
Bos     TTCCTTTGCTGGACCTGGGCAAGAAACTGAGCGTGCCCCAGGACTTGATGATGGAGGAGCTATCGCTTCGTAACAACAGA 80
Canis   TCCCTTTGCTGGACCTGGGCAAGAAGCTGAGTGTGCCCCAAGACTTGATGATGGAGGAGCTCTCTCTACGCAACAACAGA 80

Homo    GGGTCCCTCCTCTTCCAGAAGAGGCAGCGCCGTGTGCAGAAGTTCACTTTCGAGTTAGCAGCCAGCCAGCGGGCG 155
Pan     GGGTCCCTCCTCTTCCAGAAGAGGCAGCGCCGTGTGCAGAAGTTCACTTTCGAGTTAGCAGCCAGCCAGCGGGCG 155
Macaca  GGGTCCCTCCTCTTCCAGAAGAGGCAGCGCCGTGTGCAGAAGTTCACTTTCGAGTTAGCAGCCAGCCAGCGGGCG 155
Bos     GGGTCCCTCCTCTTCCAGAAGAGACAGCGCCGCGTGCAGAAATTCACCTTTGAGTTTGCAGCCAGCCCTCGTGCT 155
Canis   GGTTCCCTCCTCTTCCAGAAGAGACAGCGCCGGGTGCAGAGATTCACCTTTGAGTTTGCAGCCAGCCCGCAGGCG 155
```

ENSG00000164591 exon 3

```
Homo    ATGCTGGCCGGAAGCGCCAGGAGGAAGGTGACTGGAACAGCGGAGTCGGGGACG 54
Pan     ATGCTGGCCGGAAGCGCCAGGAGGAAGGTGACTGGAACAGCGGAGTCGGGGACG 54
Macaca  ATGCTGGCCGGAAGCGCCAAGAGGAAGGTGACTGGAACAGCGGAGTCGGGGACG 54
Bos     GTCGTGGCAGGAAGTGCAAAGGAGAAGGTGACAGGAACAGCAGAACCTGGGATG 54
Canis   ATTGCAGATGGAAGCACCAAGGGGAAGGTGCTGGGAACAGCAGAGCTGGGGACG 54
```

ENSG00000164591 intron 2

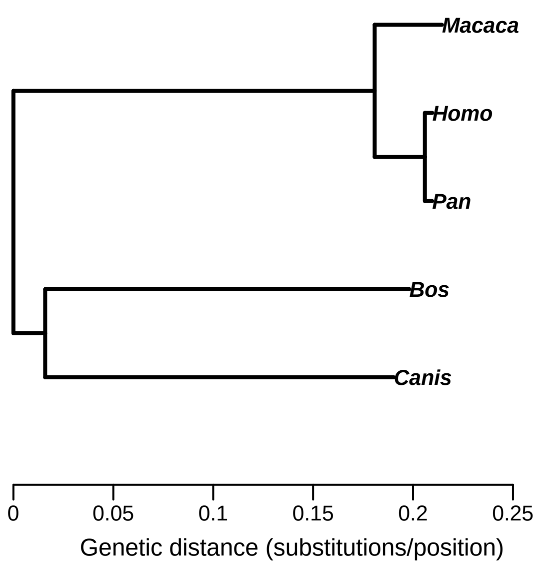

ENSG00000164591 intron 2

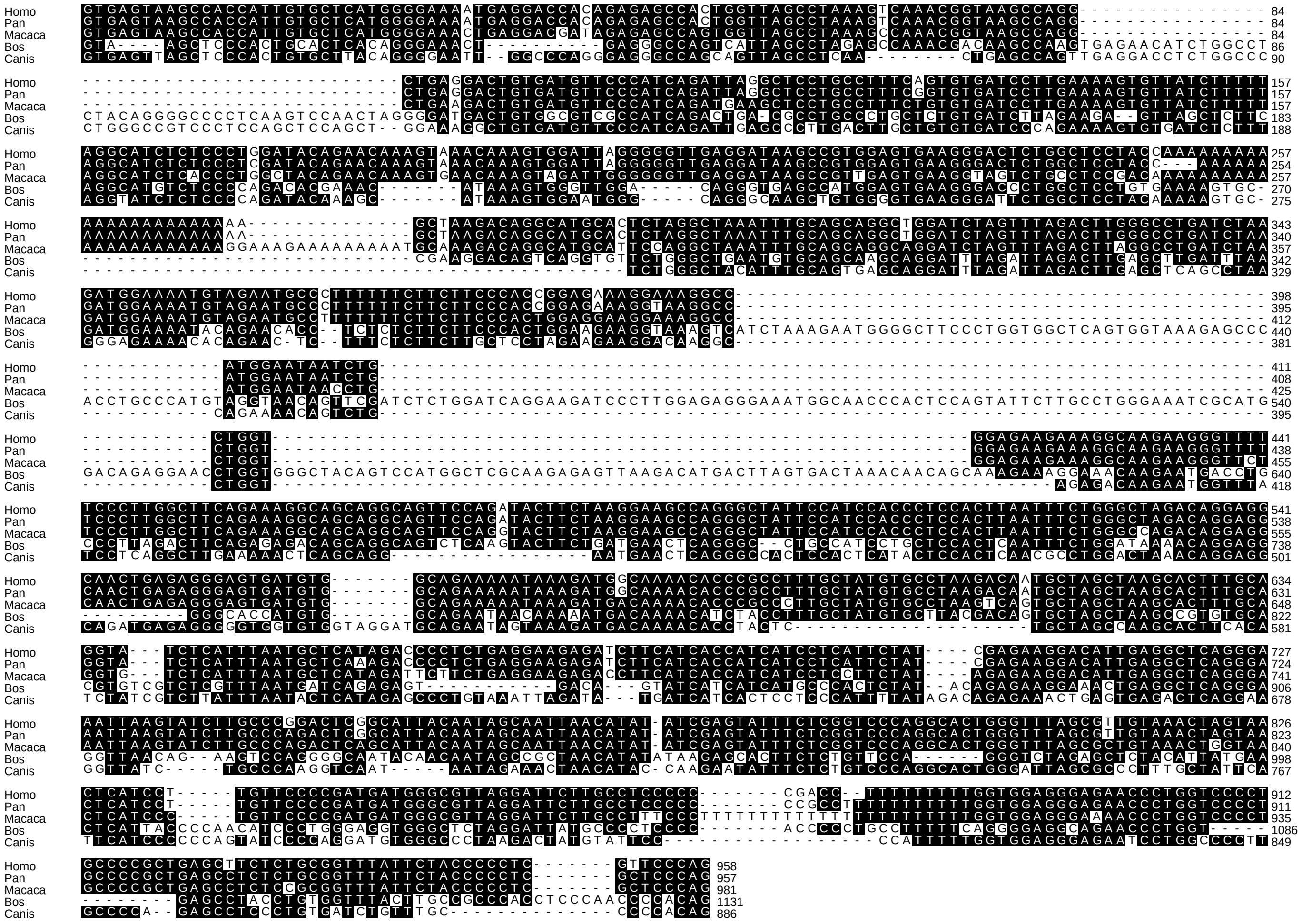

ENSG00000072682 intron 1

Description: Prolyl 4-hydroxylase alpha-2 subunit precursor (P4HA2)
Intron number: 1
Human chromosome: 05
Intron start (bp): 131581441
Human intron length : 696
Intron alignment length: 709
Flanking exons length (upstream/downstream): 82/97
SNP density: 0.001437
K tree score: 0.0523
Scaling factor: 1.1227
Human-chimpanzee distance: 0.014795
Total primate branch length: 0.0862

ENSG00000072682 exon 1

```
Homo    ------ATGAAACTCTGGGTGTCTGCATTGCTGATGGCCTGGTTTGGTGTCCTGAGCTGTGTGCAGGCCGAATTCTTCAC 74
Pan     ------ATGAAACTCTGGGTGTCTGCATTGCTGATGGCCTGGTTTGGTGTCCTGAGCTGTGTGCAGGCCGAATTCTTCAC 74
Macaca  ------ATGAAACTCTGGGTGTCTGCATTGCTGATGGCCTGGTTTGGTGTCCTGAGCTGTGTGCAGGCCGAATTCTTCAC 74
Bos     GTGACCATGAAACCCTGGGAGTCCACGTTGCTGGTGGCCTGGTTTGGTGTCCTGAGCTGCGTGCAGGCTGAATTCTTCAC 80
Canis   GTGACCATGAAACGCTGGGTGTCCACATTGCTGGTGGCCTGGTTTGGTGTCCTGAGCTGTGTGCAGGCTGAATTCTTCAC 80

Homo    CTCTATTG 82
Pan     CTCTATTG 82
Macaca  CTCTATTG 82
Bos     TTCTATTG 88
Canis   TTCTATTG 88
```

ENSG00000072682 exon 2

```
Homo    GGCACATGACTGACCTGATTTATGCAGAGAAAGAGCTGGTGCAGTCTCTGAAAAGAGTACATCCTTGTGGAGGAAGCCAAG 80
Pan     GGCACATGACTGACCTGATTTATGCAGAGAAAGAGCTGGTGCAGTCTCTGAAAAGAGTACATCCTTGTGGAGGAAGCCAAG 80
Macaca  GGCACATGACTGACCTGATTTATGCAGAAAAAGAGCTGGTGCAGTCTCTGAAGGAGTACATCCTTGTGGAGGAAGCCAAG 80
Bos     GACACATGACAGACCTGATTTATGCAGAGAAGGACCTGGTGCAGTCCCTGAAGGAGTACATCCTGGTGGAGGAAGCCAAG 80
Canis   GGCACATGACTGACCTAATTTATGCAGAGAAGGACCTGGTGCAGTCTCTGAAGGGGTACATCCTTGTGGAGGAAGCCAAA 80

Homo    CTTTCCAAGATTAAGAG 97
Pan     CTTTCCAAGATTAAGAG 97
Macaca  CTTTCCAAGATTAAGAG 97
Bos     CTCTCCAAGATTAAGAG 97
Canis   CTCTCTAAGATTAAGAG 97
```

ENSG00000072682 intron 1

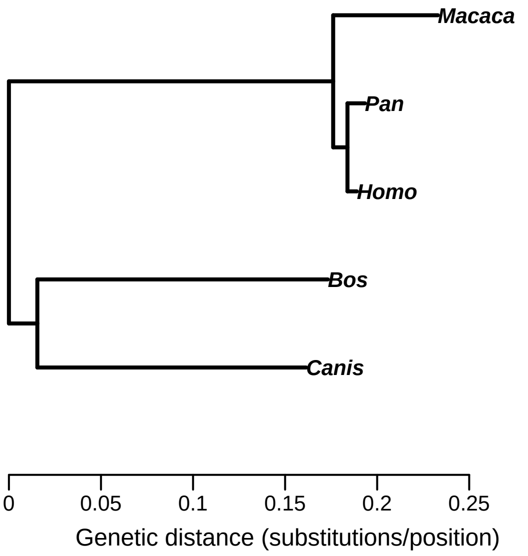

ENSG00000072682 intron 1

```
Homo    GTACGTGCCAACAGGACTGTCGTCTCCCTG---ACACCTTGGCTCACAT--GCCACGG-ATGTCTCTGGCTGCAGCCTGTTCTCATTTAGAGTGGATAG 94
Pan     GTACGTGCCAACAGGACTGTCGTCTCCCTG---ACACCTTGGCTCACAT--GCTACGG-ATGTCTCTGGCTGCAGCCTGTTCTCATTTAGAGTGGATAG 94
Macaca  GTACGTGCCAACAGGACTGTCGTCTCCCTG---ACACCTTGGCTCACCTTGGCCACGG-ATATCTCTGGCTGCAGCCTGTTCTCATTTAGAGTGGATAG 96
Bos     GTATGTGCCAACAGGACTGTCGTCTGCCTGGGCAGTCCTTGGCTCAAGT--GCCAGGGGATGGCACTGCCTGAGGCCTGTTCTCCTTCAGGGTGGGGTGG 98
Canis   GTATGTGCCAACAGGACTGTCATCTACCTGAGCAATCCTTGGCTCAAGT--GCCAGGGAATGTCACCACCTGAAGCCTGTTCTCACTCAAGGTGGAATGG 98

Homo    CCTTAACTACTGGTTTTGGCCAGTTCTGAGGAGAGTGGAACTGGCAGAGTTGCTGTTTTCCCCTATAAGATCCCAATGATCTGGATGTTC----AGGGAG 190
Pan     CCTTAACTACTGGTTTTGGCCAGTTCTGAGGAGAGTGGAACTGGCAGAGTTGCTGTTTTCCCCTATAAGATCCCAATGAGCTGGATGTTC----AGGGAG 190
Macaca  CCTTAATAACTGGTTTTGACCAGTTCTGAGAAGAGTGAAACTGGCAGAGTTGCTATTTTTCCCTATACGATCCCAGTGATCTGGTTGTTC----AGGAAG 192
Bos     CCTCGTTCACTGGTTTTGACCAGATCTGGGGAGAGTCAGGCTTGCAGAGCTGCTGTTTCCGCCTCTGA-CCCCTGATGACCTGGATGTCCTTCTTGGTAG 197
Canis   -CTCATCCACTGGTTTTGACCAGTTCTGGGGAAAGTGGGGCTCACAGAGCTGCTGTTTCCCCTTCTGAGGCCCCGATGACCTGAATGTCCTTCTTGGGAG 197

Homo    CCAGATGTCTGAATTGGGTCTTTCTTCCTGGGAAGTGCAGGCTGCACTTGGGCTCTCTGGTCTTTTTGACCACCTTGCCCATGGACCAGAGAGTGGTTCT 290
Pan     CCAGATGTCTGAAATGGGTCTTTCTTCCTGGGAAGTGCAGGCTGCACTTGGGCTCTCTGGTCTTTTTGACCACCTTGCCCATGGACCAGAGAGTGGTTCT 290
Macaca  CCAACTGTCTGAATTGGGTCTTTCTCCCTGGGAAGTGCAGGCTGCACTTGGGCTCTCTGGTCTTTTTGACCACCTTGCCCATGGACCAGAGAGTGGTTCT 292
Bos     GTGGCTGTGTCAGCTGGGCC-TCCACCTTGGAAAGTGCAGGCTGCTCCTGGGCTGCCTGGCGTTTCTGTCCAACTTTCTCCTGGACTAGAGAATGGTCCT 296
Canis   CCACCTGTCTCAACTGGGTC-TCCTCCCTGAGAAGTGCAGGTTGCTCCTGGGCTTCCTGGCCTCTCTGACCATCTCTGTTCAGGGCCAGAGAGTGGTTCT 296

Homo    GAGCAGCAAATCCTTTGTATCCTGAGGATCAAGCTTTTCCTATCCTTCCGACCTAAAGTTCAGAGCTTTTTATCCTGTGGTGAGCCCCCAGGATATCCAT 390
Pan     GAGCAGCAAATCCTTTGTATCCTGAGGATCAAGCTTTTCCTATCCTTCCGACCTAAAGTTCAGAGCTTTTTATCCTGTGGTGAGTCCCCAGGATATCCAT 390
Macaca  GAGCAGCAAATCCTTTGTATCCTGAGGATCAAGCTTTTCCTATCCTTCTGACCTGAAGTTCAGAGCTTTTTATCCTGTGATGTGCCCCCAGGATATCCAT 392
Bos     GAGCAGAGAA-------------------------------------------------------------------------GCCCTCAGGACATCCTT 323
Canis   GAGCAAAGAA-------------------------------------------------------------------------GCCCTTATGATGTCCAT 323

Homo    GCCCCAGTGTCATGA-CCAGCTATGTAACAGTCGGAGAATGAGATTTAGGGCTGCTT-CTTGAGTGACATCCAGTGCACTTATCTCAAACATCCCCTTGG 488
Pan     GCCCCAGTGTCATGA-CCAGCTATGTAACAGTCGGAGAATGAGATTTAGGGCTGCTT-CTTGAGTGACATCCAGTGCACTTATCTCAAACATCCCCTTGG 488
Macaca  GCCTGAGTGTCACGA-CCAGCTATGTAACAGTTGGAGAATGAGATTTAGGTCTACTT-CTTGAGTGACATCCAGTGCATTTATCTCAAATGTCCCACTGG 490
Bos     GTCCCAGGAGCATGACCCAGCTGGGTGACAGTGGGAGAGTGAGATGCGGGGTTTCTTCCTTAAGGTGTCCTCCGTGCA-----CTT-ACCATCTGCCTGG 417
Canis   GCCTTGGTGCCATGACCCACCTGGGTGCCAGTAGGTGAATGAGAATTAGGGCTTCTTCCTTCAGGTACTCCTAGTGCACTCATCTC-ACCATCCCCTTGA 422

Homo    TGCCTCTGCCTCTTTCTTCCTGAAGTTGCGAGATAGAGCCCCATGAGTGCCTAGGCCCCCTTTAACT-CCAAGTCCCCATAATCCTCAGAGAGCTGACAT 587
Pan     TGCCTCTGCCTCTTTCTTCCTGAAGTTGTGAGATAGGGCCCCATGAGTGCCTAGGCCCCCTTTAACT-CCAAGTCCCCATAATCCCCAGAGAGCTGACAT 587
Macaca  TGCCTCTGCCTCTTTCTCTCTGAAGTTGGGAGATAGGACCCCATGAGTGCCTAGGCCCCCTTTAACT-CCAAGTCCCCATAATCCCCAGAGATCTTACAT 589
Bos     AGCCTCTGCCTCTCTCTTCCTAAAGCTGGGGACCAGGACCATATGAGTGCCCAGGCTTCCTTTAACC-CTAGGTCCCCATGACCCCAAGAGAGCTGACAT 516
Canis   AGCCTTCATCTCTGTCTTATTGAAGCTGGGGTCCAGGACCGTGGGAGTGCCTAGGTCCCCTTAAACTGCCAAATCCTCAAGGTCCCAAGAGAAGTGATAT 522

Homo    GTTCTTATCCCAGGGGACTTGCTTCTGTGCTGGTATTCTGTGCCCCAAGGAAGGAGGCTGGACATCCCTCATCTGTTTCTCACTGGTGTCTTTCTTCTCT 687
Pan     GTTCTTATCTCAGGGGACTTGCTTCTGTGCTGGTATTCTGTGCCCCAAGGAAGGAGGCTGGACATCCCTCATCTGTTTCTTACTGGTGCCTTTCTTCTCT 687
Macaca  GTTCTTGTCCCAGGGGACTTGCTTCTGTGCTGGTATTCTGTGCTCCAAGGAAGGAGGCTGGACATCCCTCTTCTGTTTCTCACTGTTGCCTTTCTTCTCT 689
Bos     GTGCCTGGCTCAG----CTCGCCTCTGTGCTGATGTGTTTGCCTCAAGGTTGGGAGCTGGATGTCCCTCATCCACTTCTTACTGGC-CCTTTTATCTTT 611
Canis   GCCAGTGGCCCAG----AGGACTTGTGC-------TGTTGTGCCTCAAGGAAGGGGATGGACGTCCCTCATCCACTTCTTACT-GC-CCTTTTTTCTTT 609

Homo    CCCTTGCAG 696
Pan     CCCTTGCAG 696
Macaca  CTCTTGCAG 698
Bos     CCCTCGCAG 620
Canis   CCCT-ACAG 617
```

ENSG00000186532 intron 5

Description: SET and MYND domain-containing protein 4 (SMYD4)
Intron number: 5
Human chromosome: 17
Intron start (bp): 1637018
Human intron length : 448
Intron alignment length: 607
Flanking exons length (upstream/downstream): 183/164
SNP density: 0.013393
K tree score: 0.0587
Scaling factor: 1.004
Human-chimpanzee distance: 0.011309
Total primate branch length: 0.0862

ENSG00000186532 exon 5

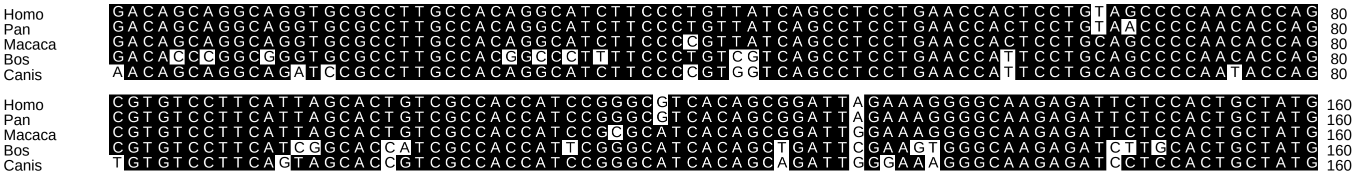

ENSG00000186532 exon 6

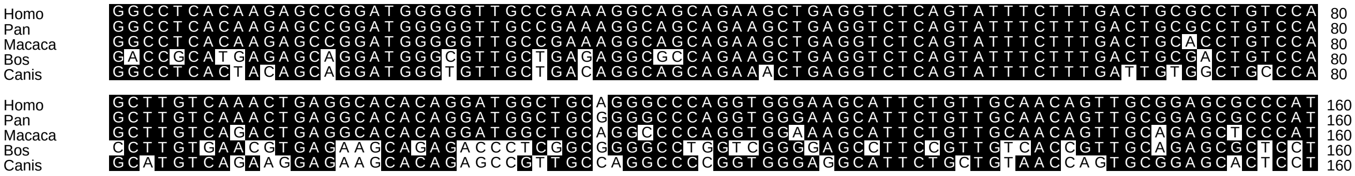

ENSG00000186532 intron 5

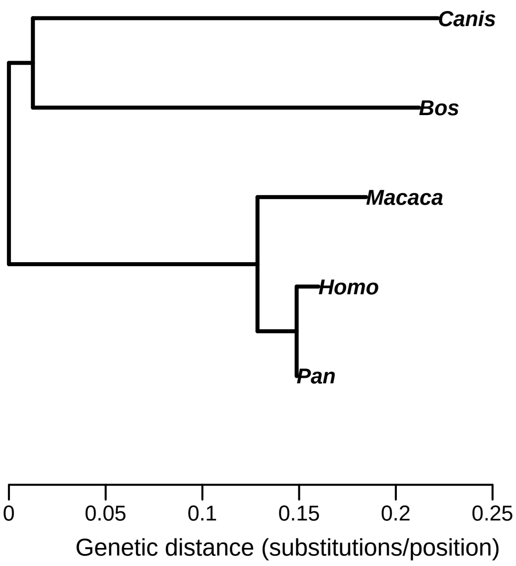

ENSG00000186532 intron 5

```
Homo    GTGAGCCATCCCTCCGCACTGCCATCCTGCCCTTCTCCAACAGAAAAGGAC--GG-GGACGCAAGTCAGCAAGCCACCAGGCATGTGACTTCACCTGTCT 97
Pan     GTGAGCCATCCCTCCGCACTGCCATCCTGCCCTTCTCCAACAGAAAAGGACAGGG-GGACGCAAGTCAGCAAGCCACCAGGCATGTGACTTCACCTGTCT 99
Macaca  GTGAGCCATCCCTCCGCACTGCTGTCCCGCC-----CCGACAGAAAAGGACAGGG-GGACGCGAGTCAGCAAGCCCCCGGGCGTGTGACTTCACCTGTCT 94
Bos     GTGAGCCGTCCATCCTCCCCGCTGCCCACCT--TCTCCAGCAGAAAAGGACGGGGTGAACTCGCATGGGCAGGCCCCAAAGCAGGTGCCTTCATCTGCCC 98
Canis   GTGAGCCCTCCCTCCTCATTACTGTCCTCAC-TTCTGTGGCAGGAAAGGACAGGGCAGATGAGTATGAGCAAACCCCTGGACAGGTGA------------ 87

Homo    A---CTGTCTGCTTCCTGGAAGAGGCAGTGAACACTTAGGCCAAAAACAAGGAAGGGAGGATGGAAGGA--------------------ATGGCCTTCGT 174
Pan     A---CTGTCTGCTTCCTGGAAGAGGCAGTGAACACTTAGGCCAAAAACAAGGAAGGGAGGATGGAAGGA--------------------AGGGCCTTCGT 176
Macaca  A---CTGTCTGCTTCCTGGAAGAGGCGGTGAACACTCAGGCCTAAAACAAGGAAGGGAGGGAGGGAGGAAGGGAGGGAGGGAGGGAGGGAGGGCCTTCGT 191
Bos     AGGGAGGGTTGTTTCCTGGTGGAGGCGGC------------------------------------------------------------ACAGAGCTCCT 138
Canis   ----------------------AGGCAGTGCATACTTAGGCCAGAAAAAGAGGCCT---------------------------------CCTGGCCTCCT 132

Homo    GCTAGGGCCCTATTGCGGCTAAACTA---TGTCTCG--CTGTTTAATCACTATAAACTTCCTATTCTGAAAA-TGGGCTAGGAACAACTTCTTGGCTTCT 268
Pan     GCCAGGGCCCTATTGCGGCTAAACTA---TGTCTCG--CTGTTTAATCACTATAAACTTCCTATTCTGAAAA-TGGGCTAGGAACAACTTCTTGGCTTCT 270
Macaca  ACTAGGGCTCTCTTGTGGCTAAACTA---TGTCTTGCTCTGTTTAATCACTATGTATTTCCTGTTCTGAAAA-TGGGCTAGGAACAACTTCTTGGCTTCT 287
Bos     CACTAGGCCCTGTTGTGTCTACACTACGGTGTGTCTCACGGCTTGATCCCTATAAGCATCCTATTCTGAAAA-TGGACTGGGAACAACTTCTTGGCTTCT 237
Canis   TCCTTGGCCCTGTTCTGTTTAAACTATCCAGTATCTTCCTGCTCAAAT-CCATAAACTTCCTATTCCAAAAACCGGACTAAGAATAACTTTGGGGTTTTT 231

Homo    CTTT-CTTCCTGTCTCAGCAACTTGGAAGCTTATTTTAGTATCCAGTTCCACAAATATTTGGGTAAAGGTTAGCTGTCACATGATAGGTAATTGTCCAGA 367
Pan     CTTT-CTTCCTGTCTCAGCAACTTGGAAGCTTATTTTAGTATCCAGTTCCACAAATATTTGAGTAAAGGTTAGCTGTCACATGATAGGTAATTGTCCAGA 369
Macaca  GTTT-CTTCCTGTCTCAGCAGCTTGGAAGCTTATTTTAGTATCCAGTTCCACAAATGTCTGAGTAAAGGTTAGCTGTCACATGATAAGTAATTGTCCAGA 386
Bos     TTTC-CTTCCTGTCCCCATGACTGTGAAGCCTGTTTC----TATAGTTACACCCGTGTTTGAGTAAAAGGTACTTCTCACATGATGTGTGATTA-CCAGA 331
Canis   TTTTCCTTCCTGTCCTTGCAACTCAGAAGCTTATGTCTACGTCTATTGCCACAAATGTTTGAGTAAAAG-TACTTGTCACATGATGAAGAATTG------ 324

Homo    CCCTCTGCTAAGCTGAAG---------------------------------------------------------------------------------- 385
Pan     CCCTCTGCTAAGCTGAAG---------------------------------------------------------------------------------- 387
Macaca  CCCTCCGCTAAGCTGAAG---------------------------------------------------------------------------------- 404
Bos     CCCTCCACTAAGCTGAAGTCTTTTTTTTTTTTTTTGACTGTGATGTGCAGGATCTTAGTTCCCTGACCAGGGAGGCAGCCCGCTGTCCCTTGCAGTGGAA 431
Canis   -CCTCCGCTAAGCTGAAG---------------------------------------------------------------------------------- 341

Homo    --------------------------------------------GTTTTTACGTAGTGTTTGACTCTCTCTTGCTTTCAATATTGGCTGACGGTGATCTC 441
Pan     --------------------------------------------GTTTTTAAGTAGTGTTTGACTCTCTCTTGCTTTCAATATTGGCTGAGGGTGATCTC 443
Macaca  --------------------------------------------GTTTTTAAGTCGTGTTTGACTCTCTCTTGCTTTCAACATTGGCTGAGGGTGATCTC 460
Bos     GCTCAGAGTCTTAACCACTGGATCGCCAGGGAAGTCCCCTGCAATCTTTTAAGCAGTGTTTGGTGCTCTCTGGGTTTCAATATTGGCCAAGCGTGGTCTT 531
Canis   --------------------------------------------TTTTAGAAGTAGTGCTCGACTCTCTCGTGTCTTCAGTATTGACCAAGTGTGGTCTC 397

Homo    CCTCCAG 448
Pan     CCTCCAG 450
Macaca  CCTCCAG 467
Bos     TCTGCAG 538
Canis   TCTGCAG 404
```

ENSG00000160932 intron 1

Description: Lymphocyte antigen Ly-6E precursor (LY6E)
Intron number: 1
Human chromosome: 08
Intron start (bp): 144173784
Human intron length : 322
Intron alignment length: 408
Flanking exons length (upstream/downstream): 52/120
SNP density: 0.000000
K tree score: 0.0957
Scaling factor: 0.415
Human-chimpanzee distance: 0.009567
Total primate branch length: 0.0862

ENSG00000160932 exon 1

```
Homo    ------------------ATGAAGATCTTCTTGCCAGTGCTGCTGGCTGCCCTTCTGGGTGTGGAGCGAG 52
Pan     ------------------ATGAAGATCTTCTTGCCAGTGCTGCTGGCTGCCCTTCTGGGTGTGGAGCGAG 52
Macaca  ------------------ATGAAGATCTTCTTGCCGGTGCTGCTGGCTGCGCTTCTGGGTGTGGAGCGAG 52
Bos     GCCTGTCCCTC---CAGAATGAAGGTCTTTCTGCCGTTGCTGCTGGCTGCCCTCCTGGGCGTGGAGCGAG 67
Canis   GCCCCTGCCTCGTCCAGAATGAAGGTGTTTCTCCCTGTGCTGCTGGCGGCCCTTCTGGGCGCGGAGCAAG 70
```

ENSG00000160932 exon 2

```
Homo    CCAGCTCGCTGATGTGCTTCTCCTGCTTGAACCAGAAGAGCAATCTGTACTGCCTGAAGCCGACCATCTGCTCCGACCAG 80
Pan     CCAGCTCGCTGATGTGCTTCTCCTGCTTGAACCAGAAGAGCAATCTGTACTGCCTGAAGCCGACCATCTGCTCCGACCAG 80
Macaca  CCAGCTCGCTGATGTGCTTCTCCTGCCTGAACCAGAAGAGCAATCTGTACTGCCTGAAGCCGACCATCTGCTCCGACCAG 80
Bos     CCCACTCCCTGGTGTGCTTCACCTGCCAGAATGTGCAAAGCAACTGGTATTGCCTGAAGCCGACCATCTGCTCCAACACC 80
Canis   CCCACTGCCTGGTGTGCTTCTCATGTACGAATCAGAACAGCAACTTCTACTGCCTGAAGCCGACCGTCTGCTCCGACTCG 80

Homo    GACAACTACTGCGTGACTGTGTCTGCTAGTGCCGGCATTG 120
Pan     GACAACTACTGCGTGACCGTGTCTGCTAGTGCTGGCATTG 120
Macaca  GACAACTACTGCGTGACCGTGTCTACTAGTGCCGGCATTG 120
Bos     GACAGTTACTGTGTGACGGTCTCTGCATCCGCTGGCCTCA 120
Canis   GACAACTACTGTGTGACGGTGTCCGCATCCGCCGGCATCG 120
```

ENSG00000160932 intron 1

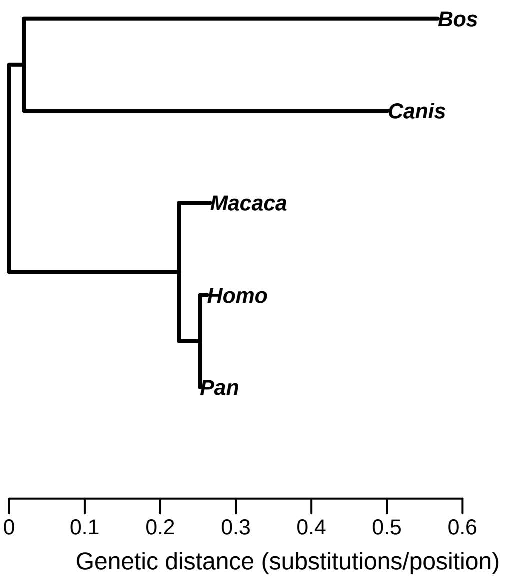

ENSG00000160932 intron 1

```
Homo    GTGAGGTGCCCTTGGGGACCCCAGACCTTTGTCCAGCTG-TGCCCTGCTCCACTCCCTCTCCACCCCTCTCCCCTGAGCAGACGCCCCAGGGGTCCTTCC 99
Pan     GTGAGGTGCCCTCGGGGACCCCAGACCTTTGTCCAGCTG-TGCCCTGCTCTACTCCCTCTCCACCCCTCTCCCCTGAGCAGACGCCCCAGGGGTCCTTCC 99
Macaca  GTGAGGTGCCCTCGGGGACCCCAGAACTTTGTCCGGCTG-TGCCCTGCTCGACT-------------------------------CCCAGGGGTCCTTCC 68
Bos     GTGAGGTGCCCC--GGGCCCCGGGCCTCCAAGCTGGCCG---------------------------------------TGGCCGTGTCACTGGTGCCACT 59
Canis   GTGAGATGGCCCGGAGGTCCCTGGCTGTGGGGTGAGCCGCTGTCCTCCTGGACTCTCTCTCTCCCTTCTCCC------TAGCTGTGCCCGGGGGCCTTCA 94

Homo    AGGCCGCTCCCAGCAGAGGG-----------------------CTCACCCGGC----CTGGCCACACTGTCTCACTGTGTGTTTGAGTGTCGCTTGACCT 172
Pan     AGGCCGCTCCCAGCAGAGGG-----------------------CTCACCCGGC----CTGGCCACACTGTCTCACTGTGTGTTTGAGTGTCGCTTGACCT 172
Macaca  AGGCCGCTCCCAGCAGAGGG-----------------------CTCCCCCGGC----CTGGCCACACTGTCCCACTGTGTGTTTGGGTGCCACTTGAGCT 141
Bos     CACTGCCTCCC--------------------------------CTGCCCCGAC---------------------------ACTTGGACATTGCTGGAACC 100
Canis   GGCCTGTTCCCGGGTGACTGTTCTGTACGGCCCTACAGGTCCTCTCCCCCAGCAAAGCGGGTCACTCCTTCCTGCTCCCACTGGAGGCCCCAGTTCTGCC 194

Homo    GCTCGACGGC---------CAGGGTGGGGTGTCACTGTCTTTGCTCTGCCTTCAGCCCAGGGCCTGGTATACAGTAATTTCTCAGTAAATGTCCAC---- 259
Pan     GCTCGACGGC---------CAGGGTGGGGTGTCACTGTCTTTGCTCTGCCTTCAGCCCAGGGCCTGGTATACAGTAATTTCTCAGTAAATGTCCAC---- 259
Macaca  GCTCGACGGC---------CAGGGTAGGGTGTCACTGTCTTCGTTCTGCCTCCACCCCAAGGGCTGCTATACGGTAATTTCTCAGTAAATGTCCAC---- 228
Bos     CCTCCAGGGC---------CGGGGTCTGGTGTCCTCGTCCTTCCAG--GTCTCCAGCCTGGGCCCCACGTACCACAGGGGCCCCATCAGTGCCTGCGCAA 189
Canis   CCTGGCACACTTATCCCTGTGCATTTGGATGTCACGGGAGTTCCTC--CTCCAGGGCCAAGTCCTGGGGCATGATAATTGCTCCATTAACATCTGCAGAT 292

Homo    ---------------------TGGGGTCAGGCCTGGGAGGGA-------------------CACTGGAGGCTT-----CCCTGAGACAGGTGTGTCCTCC 314
Pan     ---------------------TGGGGTCAGGCCTGGGAGGGA-------------------CACTGGAGGCTT-----CCCTGAGACAGGTGTG---TCC 311
Macaca  ---------------------TGGGGTCAGGCCTGGGAGGGA-------------------CACTGGAGGTTT-----CCCTGAGACAGGTGTGTCCTCC 283
Bos     GAGGGGGTGTCCTCAAGCCTCTGGGTCGGGGCCGGGAGGGGG-------------------CACCGGAGGACTCCCCGGCATGAGAC---TGCATCCCTC 267
Canis   GATTGGGAA-----------GTGAGGGGGGGCCGGAAGGGGAGGAGCGGCGGAGGGGTCACCCCTGAAGGTTCCCGGGCCCTGAGACAGGTGTCCCCTTG 381

Homo    TTCCGCAG 322
Pan     TTCCACAG 319
Macaca  TTCCACAG 291
Bos     CCCTGCAG 275
Canis   TTCTGCAG 389
```

ENSG00000011021 intron 20

Description: Chloride channel protein 6 (CLCN6)
Intron number: 20
Human chromosome: 01
Intron start (bp): 11820144
Human intron length : 835
Intron alignment length: 1700
Flanking exons length (upstream/downstream): 157/108
SNP density: 0.007186
K tree score: 0.0836
Scaling factor: 0.4546
Human-chimpanzee distance: 0.012304
Total primate branch length: 0.0859

ENSG00000011021 exon 20

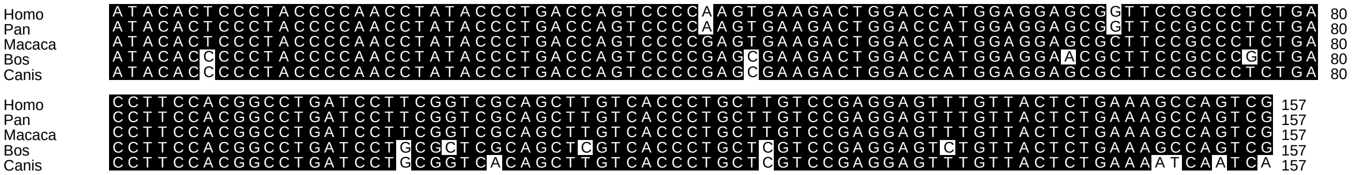

ENSG00000011021 exon 21

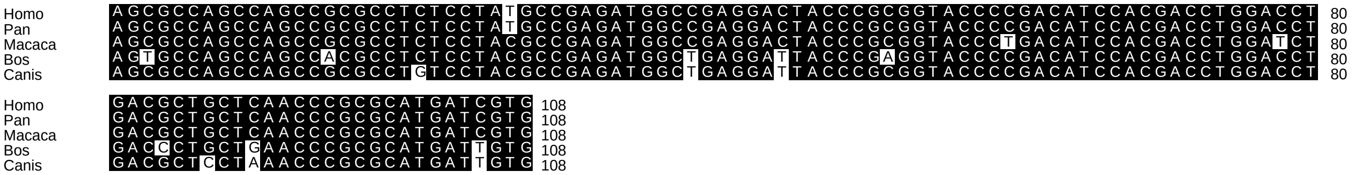

ENSG00000011021 intron 20

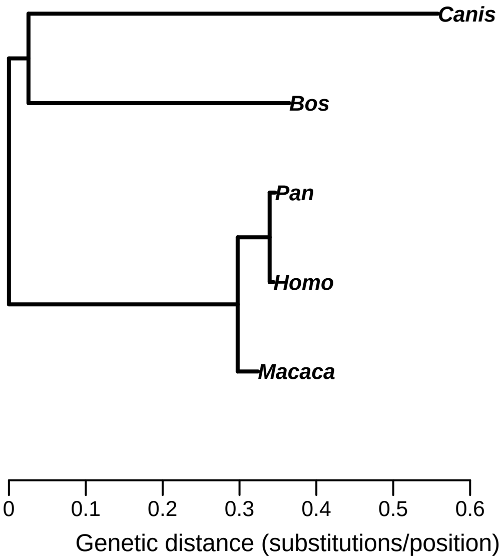

ENSG00000011021 intron 20

```
Homo    GTAAGTCT--------------------------------------------------------------------------------------------  8
Pan     GTAAGTCT--------------------------------------------------------------------------------------------  8
Macaca  GTAAGTCT--------------------------------------------------------------------------------------------  8
Bos     GTGAGTCTCCCTTCTTGTCCACACCAGGCCTCTGGAAAGCATGA---------------------------GGGTGCTTGGAACCCAGTCCCCACACCTG  73
Canis   GTGAGTCT---CTCTGGTCTGCGCTGGGATTCTGGAAGGCACAGGGTGGTAGGAGCACGGGGAACAGAAGTGGACCTTTGGGAGTCTGTGTGCCGGCGGG  97

Homo    --------------------------------------------------------CTCCGAGGC-----------------------------------  17
Pan     --------------------------------------------------------CTCCGAGGC-----------------------------------  17
Macaca  --------------------------------------------------------CTCCAAGGC-----------------------------------  17
Bos     CTGTTGAGCAGTGCGTGCAATCCCCGCTGCCCTATGCTGAGCAGGGCTCAGAGTCCCTCCAGGGCCTTGCCC---------CTTAGTCTCTGCATCTGTT  164
Canis   CTGCTGTCCCGTGCTTGTGCAGCCAGCCCCGTGGTCCCCTCGGCCGTGGCCGGCGCCTGTCAGGTGACACGCGGTGGGGGGCTCCCCCAGGCCCCCTGAC  197

Homo    ----------------------------------------------------------------------------------------------------  17
Pan     ----------------------------------------------------------------------------------------------------  17
Macaca  ----------------------------------------------------------------------------------------------------  17
Bos     GTTAAGTGTCCTTCTAGCCAGTAGCCTTTTAATATAATTTCTACACTTCAATTTCTGCCATGTATGCACTTTGTTACACGTGTTATCCAAAGAAATGCCT  264
Canis   AACTGCTGCCCTTGTAGCCTGTAGACTTTTAATAGACTTTGAGTTTTTTTAAAATGCTGTTCTTCCCACTAATTTTTTTTTTCTTTCATTTGGGTTTTGT  297

Homo    ----------------------------------------------------------------------------------------------------  17
Pan     ----------------------------------------------------------------------------------------------------  17
Macaca  ----------------------------------------------------------------------------------------------------  17
Bos     TTGTTCTTGTTCATTTCCCTGTAGTCTTTTCTTTGCTTTGGTTTATGTGTGTTCAAAGTGGTCCATATAGTGACCTGCAACACACTTCACCCCTTCAGAT  364
Canis   AGAGACAAGTGGGGGTGGGTGGGGTGTGTTGTTCAAAGTGGTCCCTCTCGTGCCCTGAAGGCCTGGCCATCCCCACACACGTCAC----CCCCTGTAGAC  393

Homo    ----------------------------------------------------------------------------------------------------  17
Pan     ----------------------------------------------------------------------------------------------------  17
Macaca  ----------------------------------------------------------------------------------------------------  17
Bos     GGAGCCTGAAAAACTCACACAGAGACCTAGTTCCCAAACTAATTTTCCTTTGGGTCTTGCCCGTGGGCAGCCAGCCTGAGGTCACCCATGGTCTGACTAG  464
Canis   AAAGCCTGCAGAGGTTGCCCAGAGGCCTGCTTTCCAGACTGACTGTCCT---------------------------------------------------  442

Homo    ----------------------------------------------------------------------------------------------------  17
Pan     ----------------------------------------------------------------------------------------------------  17
Macaca  ----------------------------------------------------------------------------------------------------  17
Bos     CCTGCAGAAGAGCAACTCACGTGGGCAGGACAGGAAGGGCCACGGATGGAGTAGCTCAGGCTTGGGGGTATGGCATGTGGGAGGCCCAACCTCACTGGAA  564
Canis   ---------------------------------------------------------------------------------------------GCTTGAG  449

Homo    ----------------------AGAAATCAGCCAGGCCA-------------------------------------------GACCTGACGAAGCTCGAG  52
Pan     ----------------------AGAAATCAGCCAGGCCA-------------------------------------------GGCCTGACGAAGCTCGAG  52
Macaca  ----------------------AGAAATCAGCCAGGCCA-------------------------------------------GGCCTGATGAAGCTTGAG  52
Bos     TGGCCCAAGGGACGAGAGTTGGAGGAATGAAGCAGGCCAGACTATACAGGGCCTCGCCTGCAGACAGAAGCCA-CCAGTGCTGGCCTGAGGAAGCCCTGG  663
Canis   CTCTTGCCCACAGGAGCACAGGAGGGCCGAGGTGGGCTG---CGTTAGAGGCCTTGTCTAAAGGCAGAAACCAGCCAGTGCTGGCCTGATGGAGCAGCGG  546

Homo    GGGTT---------------GGGCGCTG-C--------CGGCGGGCCGTGAACAGTGCCATAGG------------------------------------  92
Pan     GGGTT---------------GGGCGCTGCC--------CGGCGGGCCGTGAACAGTGCCATAGG------------------------------------  93
Macaca  GGATT---------------GGGCGCTGCC--------CGGTAGGCCATAAACAGTGCCGTAGG------------------------------------  93
Bos     GGGTC---------------AGCGGTGGACAGCAAAGACCGTGGGCCAAGGGGAGGAAGGGGAAGAAGATTGGGGAGGAGTGGTGACTGGCCTGAGAGC  748
Canis   GAGCTGCGTGCCGCTGGGCGGGCGGTGGGCGGAGAATGCAGCGTGCAGAGTGGAGCCGCGGGGAGAGTGACCAGGGCAGAGCAGTGACCAGCACTTGTGG  646

Homo    ------------------------------------------------GAAAGCTGAATGCAAGGCGAGAAG----------------------------  116
Pan     ------------------------------------------------GAAAGCTGAATGCAAGGCGAGAAG----------------------------  117
Macaca  ------------------------------------------------GAAAGCTGAATGCAAGGCAAGAAG----------------------------  117
Bos     TGATGGTCTCGGTGCCCTGTAACGGTCAGCG------------GCCTTGAGAGCTGAGGGAAAGTCCAGAAGACTCAGGATGCAGGAATAGTGGTAAAGC  836
Canis   CCACTGCGCCCAGCCTGAGACCCTGTTGGTGTGGCCCAGGTGCCCTTGGAGGGCTGAGGGCAAATGCGGGAGACGCGGGATGCAGGGGTGGG--------  738

Homo    -------------TGCAGAGTGGACTT------------------------------AGGGGAGGAGTCGCCACAG-CCGGGTGGTGAGA--------AG  164
Pan     -------------TGCAGAGTGGACTT------------------------------AGGGGAGGAGTCGCCACAG-CCGGGTGGTGAGA--------AG  165
Macaca  -------------TACAGAGTGGACTT------------------------------AGGGGAGGAGTTGCCACAG-CCGGGTGGCGAGA--------AA  165
Bos     TCCCTTGTGACTTTACTGGGCTGACTTTCCTGGGGCTGCTGTTTGCAGTCGCTGCAAACTGGGCAAAATCCCACAG-GCGGGCCCCTCAGATGCTTCCAG  935
Canis   -------------AACAGAGCCGCTGT----------------------------------GTCCAACTGCCACAGACCAGGTGGCTGCA--------AG  783

Homo    CAACAGCGGTTTATTCCTTCATAGTTC---AAGGCCAGAAGTACAAAATCCGGGTGTCACCAGGGCAGCGCTCCCT-----GGCTCT------GGTGCTG  250
Pan     CAACAGCGGTTTATTCCTTCATAGTTC---AAGGCCAGAAGTACAAAATCCAGGTGTCACCAGGGCAGCGCTCCCT-----GGGTCT------GGTGCTG  251
Macaca  CAACAGCGGTTTATTCCTTCCTAGTTC---AAGGCCAGAAGTACAAAATCCAGGTGTCACCAGGGCAGCGCTCCCT-----GGCTCTGGGAGAGGTGCTG  257
Bos     CAACAGCAGTGTGTCCTTGCTTGGTTTTGAAGGCCCTGGGACAGAACGCCCCG---GTCGGCCGGGCTGTGCTTCCTCTGAAGGCTCCAGGAGAGG----A  1028
Canis   CAGGAGGAACGCGTTCTTCCGTAGTTGTCAAGGCC--GGAGTACAAAGCCCGCACGTCAGCGGGGCCTTG------------------------------  851

Homo    TCCTTGCCCCTTCCTAGCTCATGGGAGTTGCCTGCAGACCTTGGTGCTCCTGACTGTGAACACAGCCCCCATCCTCGCCACTGTCATCACACG----GCG  346
Pan     TCCTTGCCCCTTCCTAGCTCATGGGAGTTGCCTGCAGACCTTGGTGCTCCTGACTGTGAACACAGCCCCCATCCTCGCCACTGTCATCACATG----GCA  347
Macaca  TCCTTGCCCCTTCCTAGCTCATGGGAGTTGCCTGCAGTCCTTGTTGCTCCTGAC--TGAATGCAGCCCCCATCCTCGCCACTGTCATCACAAG----GCG  351
Bos     TTCTTG--CCTTCCTCATTTGTGGTGGGTGCCCTCGGCCT------GCCCTGGCTGGAGGGCCATCAATTGTGTCTGCCTCT-TTGTTACCCG----GCC  1115
Canis   --------GCTTCCTCGTGCGTGGAGGCTGCCAGCCATGCCTGGCTCTCCTTGACCGTGGATCTGCCACTGCAGCCTCTGCTCCCGTGTCACGTGGTGTG  943

Homo    TTCTCCTGTGTATCTTCTCTCCTTTCCCTAAGGACGCTTGTCACCGGATTTGGGGCCCGCCCTAATCCAGCATGGGTTCATCTGAACTCGGTCACATCTG  446
Pan     TTCTCCTATGTATCTTCTCTCCTTTCCCTAAGGACGCTTGTCACCGGATTTGGGGCCCGCCCTAATCCAGCATGGGTTCATCTGAACTCGGTCACATCTG  447
Macaca  TT---CTGTGTATCTTCTCTCCTTTCCCTAAGGACACTTGTCACTGGATTTGGGGCCCACCCGAATCCAGCATGGGCTTATCTGAACTTGGTCACATCTG  448
Bos     CT-CCCTCTGTGCCCTCGCCTCTTTTAGC-AGGACACCAGTCCCTGGGTTTGGGGCCCACCCTCCTCCA-CGTGACCTCATTTTACCTTGACTACATCTG  1212
Canis   CT-CCCTGTGCGCCTCTCCTCTTGATATT----------GTCAGTGGA-CTGGGGCTCACCCCAATCCTGCCTGCCCTCTTCTGAACTGGCTTCTGTCTG  1031

Homo    TAGAGACCTTGTTTCCAGAAAGGTCGCATTCATA-GGTACTAGGGCTTAGGAC---TTGGCCATATCTTTTGGTGGGGGGACACATTCACCCCCCAGCCA  542
Pan     CAGAGACCTTGTTTCCAGAAAGGTCGCATTCATA-GGTACTAGGGCTTAGGAC---TTGGCCATATCTTTTGGTGGGGGGAGACATTCACCCCCCAGCCA  543
Macaca  CAGAGACCCTGTTTCTAGAAAGGTCACATTCATA-GGTACTAGGGCTTAGGAC---TTGGCCATATCTTTCGGTGGGGGGACAGATTTACCCCCCAGCC-  543
Bos     TAAAAGCCTCTTTTCCAAGAAGGCTACATTCACA-GATTCTAGGCATTAGAAT---GCAGACGAATCTTTTA--GGGGACTCAAGTCGCTACAGCTACAG  1306
Canis   CAGAGACCCTGCTTCTGACATGGCCACACCCACAGGGTAGCAGGAGTTAGCGCTAGTCAGGCGTGTCTTTTG----AGAGACACGTCAACCCACGGGAGC  1127

Homo    CTTTACTTAGTGC-TTTCTCACTTAAACTTCCTAATAAGCCATTTGCATCTCATGAATCAGGAAGAAAGGGAGTGCAGACAGATCAGGGGACTTGCCCAG  641
Pan     CTTTACTTAGTGC-TTTCTCACTTAAACTTCCAAATAAGCCATTTGCATCTCATGAATCAGGAAGAAAGGGAGTGCAGACAGATCAGGGGACTTGCCCAG  642
Macaca  ----ACTTAGCGC-TTTCTCACTTAAACTTCCAAATAAGCCATTTGCGTCTCATGAATCAGGAAGAAAGGGAGTGCAGAGAGATCAGGAGAC-GGCCCAG  637
Bos     CTACTATTAGCACTTTTCTCACTGAAACTCTAGAATAGCTCACATCTGTTCTACAGAT-----GAAAGGGACGTTCAGAGAAGTTAGGAGACCCGCCCAG  1401
Canis   TGGTAGGTAGCTCTGCTCTCACGTAGACTCCAGGGCAAGCCGTGTGCACCTCACAGAC--GGAGAGAAGCAGGTTCAGAGAGAGTAGGAGGTTCGCCGAG  1225

Homo    GGTGCCACAGCTA-GGACTTGGCCAAGGACTTGGTTT-GACTGGAGAGCTCTCACTCTGGAGCGCTTG--CTGCTGCTGCACCACCTGCCTCAGCAGCCT  737
Pan     GGTGCCACAGCTA-GGACTTGGCCAAGGACTTGGTTT-GACTGGAGAGCTCTCACTCTGGAGCGCTTG--CTGCTGCTGCACCACCTGCCTCAGCAGCCT  738
Macaca  GGTGCCACAGCTA-GGACTTGGCCAAGGACTGGGTTT-GACTGGAAAGCTCTCACTCTGGAGTGCCTG--CTGCTGCTGTGCCACCTGCCTCAGCAGCCC  733
Bos     GG-GACACAGCTAGGGAATTGGCTGAGAAATGGCTTT-GGAGCAGGAGGTCTCACTCCAGAGCACCTGTCCTGGCCCTGCACTGCCCGCCT---------  1490
Canis   G--GGCACAGCGA-------GGCTGGGAGCTGGCCTCGGGCCCCGGGCCTCTCACGGCAGAGCCCCG------TTCTGTACCACCTCGCACCA------  1304

Homo    GGAGCTGGGCATATTCAGGCATCAAGGGGAGGGGCTCTAAG-GTGACCCTGCTGGCCACTGCTGCCTGAGC-ACGGACAGTGTCTGGGTTGGAATTGCAG  835
Pan     GGAGCTGGGCGTATTCAGGCATCAAGGGGAGGGGCTCTAAG-GTGACCCTGCTGGCCACTGCTGCCTGAGC-ACGGACAGTGTCTGGGTTGGAATTGCAG  836
Macaca  AGAGCTGGGCATATTCAGGCATCAAGGGAAGAGGCTCTCGG-GTGACCCCGCTGGCCACTGCTGCCCGAGC-ACGGACAGTGTCTGGGTTGGAATTGCAG  831
Bos     ---------GGCTCTCAGACATTGAGGGAGGGTCCCCTAAACGTGGCTCCACCAGCGGCCACATCCAGTGCAAAGGACAGTGTCTGGGTTGGGATTGCAG  1581
Canis   -----GGTGGGGCTGTGGACGTGGAGAGGGAGGGGCTGTGA-ATGTCCCCGACGGTGGCTGTACTGGGTAC-AAGGAT-GTGTCTTGGTTGGGATTGCAG  1396
```

ENSG00000146409 intron 1

Description: C6orf192 protein (C6orf192)
Intron number: 1
Human chromosome: 06
Intron start (bp): 133159954
Human intron length : 1292
Intron alignment length: 1701
Flanking exons length (upstream/downstream): 43/140
SNP density: 0.003096
K tree score: 0.0793
Scaling factor: 0.8431
Human-chimpanzee distance: 0.016040
Total primate branch length: 0.0858

ENSG00000146409 exon 1

```
Homo    ATGGAGGCGCTGGGTGACCTGGAGGGACCACGCGCACCAGGAG  43
Pan     ATGGAGGCGCTGGGTGACCTGGAGGGACCACGCGCACCAGGAG  43
Macaca  ATGGACGCGCCGGGTGACCTGGAGGGCCCACGCGCACCAGGAG  43
Bos     ATGGACTCACCGGGAGACCCCGAAGGCCCACGCCCCCAAGGAG  43
Canis   ATGGACGCGCCGGGGGGTCCAGAGGGCCCACGCCCCCCAGAG  43
```

ENSG00000146409 exon 2

```
Homo    GTGATGATCCTGCAGGAAGTGCAGGAGAGACCCCCGGGTGGCTTTCGAGAGAACAGGTTTTTGTACTGATATCGGCAGCT  80
Pan     GTGATGATCCTGCAGGAAGTGCAGGAGAGACCCCCGGGTGGCTTTCGAGAGAACAGGTTTTTGTACTGATATCGGCAGCT  80
Macaca  GTGATGATCCTGCAGGAAGTGCAGGAGAGACTCCCAGGTGGCTTTCGAGAGAACAGGTTTTTGTACTGATATCGGCAGCT  80
Bos     GTGATGGCCCTCCAGCAGGTGCAATGGAGACATCCAGGCGGCTTTCAAGAGAACAGATTTTTGTACTGATATCAGCAGCT  80
Canis   GTGATGACTCTCTGGGAAATACAAGAGAGACACCCAGACAGCTTTCCAGAGAACAGTTCTTTGTACTGATATCAGCAGCT  80

Homo    TCGGTGAACTTAGGTTCCATGATGTGCTATTCTATACTTGGACCGTTTTTCCCCAAAGAG  140
Pan     TCCGTGAACTTAGGTTCCATGATGTGCTATTCTATACTTGGACCGTTTTTCCCCAAAGAG  140
Macaca  TCGATGAACTTAGGTTCCATGATGTGCTATTCTATACTTGGACCCTTTTTCCCCAAGGAG  140
Bos     TCCATTAACTTAGGTTCCATGATGTGCTATTCTATCCTTGGACCCTTTTTCCCCAAGGAG  140
Canis   TCCATTAACCTGGGTTCTATGATGTGCTATTCTATCTTAGGACCCTTTTTTCCCAAGGAG  140
```

ENSG00000146409 intron 1

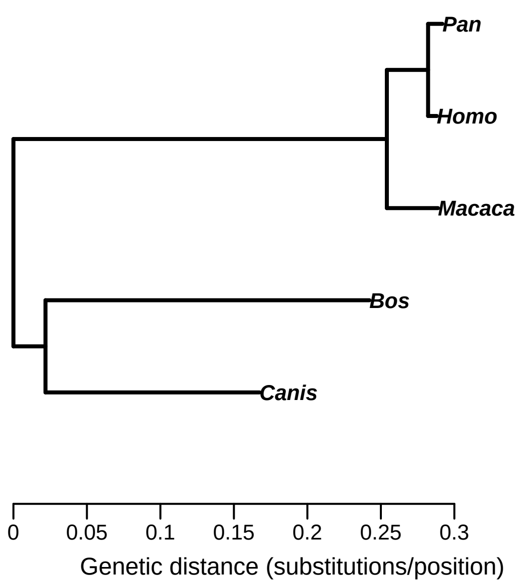

ENSG00000146409 intron 1

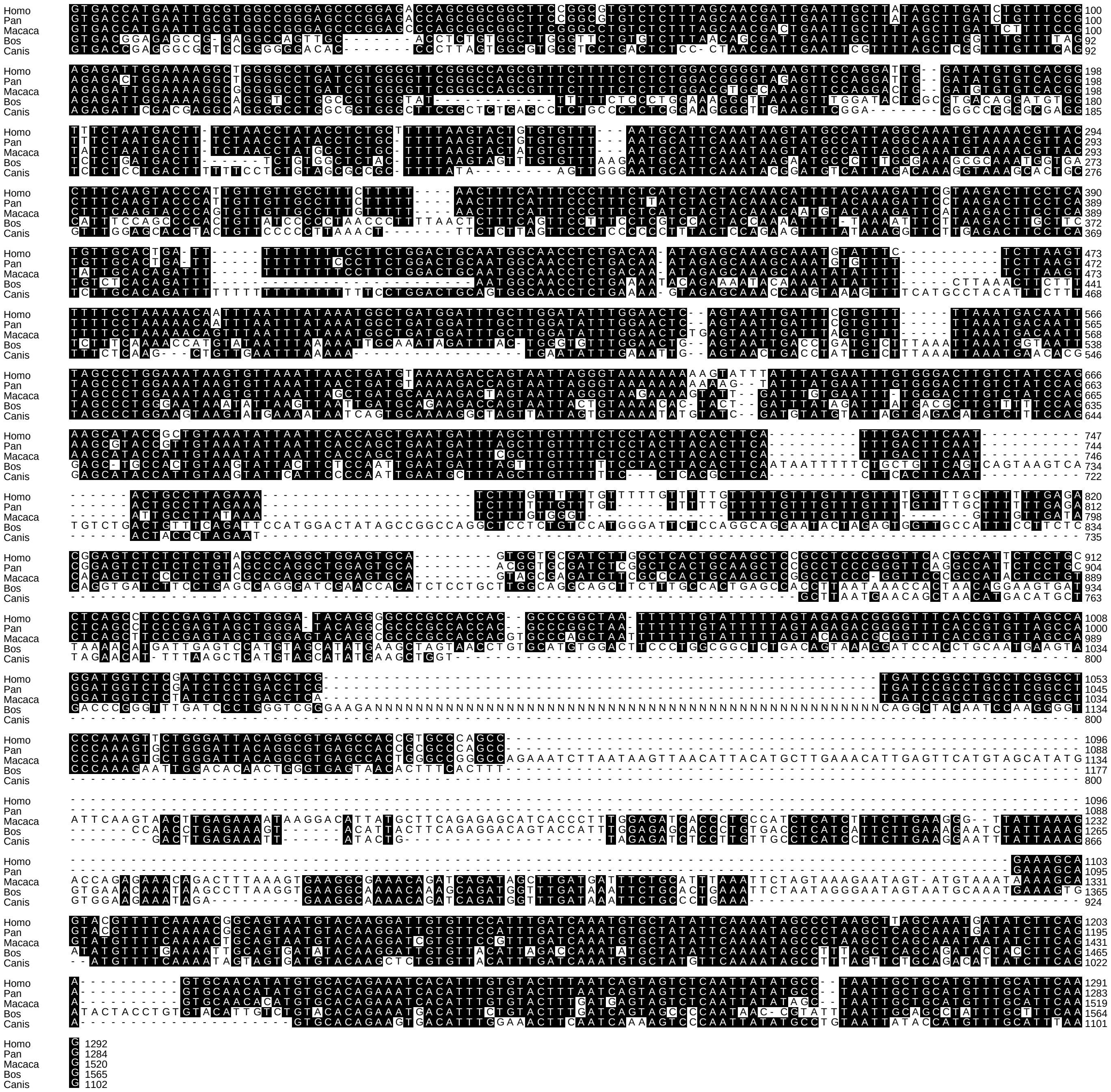

ENSG00000108405 intron 3

Description: P2X purinoceptor 1 (P2RX1)
Intron number: 3
Human chromosome: 17
Intron start (bp): 3754443
Human intron length : 458
Intron alignment length: 524
Flanking exons length (upstream/downstream): 72/70
SNP density: 0.002183
K tree score: 0.0702
Scaling factor: 0.8545
Human-chimpanzee distance: 0.011002
Total primate branch length: 0.0857

ENSG00000108405 exon 3

```
Homo    GGGGACAACTCCTTCGTGGTCATGACCAATTTCATCGTGACCCCGAAGCAGACTCAAGGCTACTGCGCAGAG  72
Pan     GGGGACAACTCCTTCGTGGTCATGACCAATTTCATCGTGACCCCGAAGCAGACTCAAGGCTACTGCGCAGAG  72
Macaca  GGGGACAATTCCTTCGTGGTCATGACCAATTTCATCGTGACCCCAAAGCAGACTCAAGGCTACTGCGCAGAG  72
Bos     GGGGACAGTTCCTTCGTAGTCATGACCAATTTTATCGCCACCCCCCGGCAGGCTCAAGGTCACTGTGCAGAG  72
Canis   GGGGACAGCTCCTTCGTGATCATGACCAATTTCATTGCGACCCTCCAGCAGGTTCAAGGCTACTGTGCGGAG  72
```

ENSG00000108405 exon 4

```
Homo    CACCCAGAAGGGGGCATATGCAAGGAAGACAGTGGCTGTACCCCTGGGAAGGCCAAGAGGAAGGCCCAAG  70
Pan     CACCCAGAAGGGGGCATATGCAAGGAAGACAGTGGCTGTACCCCTGGGAAGGCCAAGAGGAAGGCCCAAG  70
Macaca  CACCCAGAAGGGGGCATATGCAAGGATGACAGTGGCTGTACCCCTGGGAAGGCCAAGAGGAAGGCCCAAG  70
Bos     AACCCGGAAGGGGGCACGTGCACAAATAATAGCGGCTGCACCCCGGGAAAGGCAGAGAGAAAGGCCCAAG  70
Canis   CATCCAGAAGGGGGTACATGCAGAAATGACAGTGGCTGCACTCCGGGGAAGGCAGAGAGGAAGGCACAAG  70
```

ENSG00000108405 intron 3

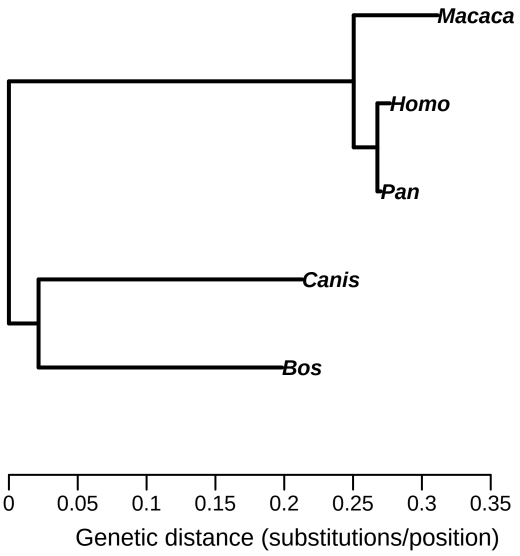

ENSG00000108405 intron 3

```
Homo    GTGAGGACTTCTGCCCGCCAGCC-----CCCTTCTGGGACTCACAGGGGAAAATGACGT-GCAGAGAAAGTCTCCAGGGGCACTGGAGGGGAAGCCAGA- 93
Pan     GTGAGGACTTCTGCCCGCCAGCC-----CCCTTCTGGGACTCACAGGGGGAAATGACGT-GCAGAGAAAGTCTCCAGGGGCACTGGAGAGGAAGCCAGA- 93
Macaca  GTGAGAACTTCTGCCCGCCAGCC-----CCGTTCTCGGACTCACGGGGGAAAATGACCT-GC--AGACAGTCTCCATGGGCACT-GAGAGGATGCCAGA- 90
Bos     GTGAGGACTGTTACCCATTCCCT------GTTTTTTGAACCTTCAGGAAAAAGTAATGTGGAAGAGGAGGTCCCCCGGAGTATTGGAGGGAAAGCCTGAT 94
Canis   GTGAGGACTGCTACCTGTCCCCTCACCCCAATTCTTGGACCCATAGGGGAAAATAACATGGAAGAGGAAGTCCCCTTGGCTATCAAAGA----------- 89

Homo    GAGGCTTCCTGCAGGGGCCATAGTGATGGAAATTTGGGCGCATATTCACACACAGCAACTGTCAACCCTT-GCAGAGCTGAGCCTTCCCGCCCAGCACAC 192
Pan     GAGGCTTCCTGCAGGGGCCATAGTGATGGAAATTTGGGCGCATATTCACACACAGCAACTGTCAACCCTT-GCAGAGCTGAGCCTTCCCGCCCAGCACAC 192
Macaca  GAGCCTTCCTGCAGGGGCCACGGTGGTGCAAACTCGGGCATATATTCACACACAGTAACTGTCAACCCTT-GCAGAGCTAAGCCTTCCCGCCCAGCA--- 186
Bos     TAAGCCTCCCTGTGGGGCCGTAATGGTGGAATTTCAGGCATAGATTCACACAGAGTGATAGCCCACCGTTGGCAGAGCTGAGGCTTCCCTCCCAGCT--- 191
Canis   TAGGCTTCCTCGGGGGAATGCGATGGTGGAATTTTAGGCATAGATTCACACAAAGTAACAGTCTGCTATTGGCTGTGGAGACGCTTCCCTCCCAGCT--- 186

Homo    GGTGTACCAGGCAGGGTGCCGAGACTTCGTGCCCACCCC--------------------------CACCGCCCAGCTGACATTGGCAGT-GGGCAGCTG 264
Pan     GGTGTACCAGGCAGGGTGCCGAGACTTGGTGCCCACCCC--------------------------CACCGCCCAGCTGACATTGGCAGT-GGGCAGCTG 264
Macaca  ----TACCAGGCAGGGTGCCGAAACT--GTGCCCACCCC--------------------------CACCGCCCAGCTGACATTGACAGT-GAGCGGCTG 252
Bos     -GTGTGTCAAGCGGGATGCTGAGCCTCAGTGCCTGCCCAGTCCCCAACACCT----TCCCTCCGAATCCCTGCCAGCTGACATCGGCCCTGGGACAGCTG 286
Canis   -ATGTGTCAAGCAAGATGCCGACCCTCAGTGCCCACCCAACCCCCAAACCCTAACATCTCTCC---TTCCTGCCAGGTAACACTGGCCATAGGCAG-TG 281

Homo    GGCATGAGCCC------GGGGGGCGCCTCGGCAGAGCAGTG-TGGGGGGAAATGGGGGAAGGAGGCTGTGAGCAGGCGTCACTCCAGAGAGGTCTGAGAC 357
Pan     GGCATGAGCCC------GGGGGGCGCCTCGGCAGAGCAGTG-TGGGGGGAAATGGGGGAAGGAGGCTGTGAGCAGGCGTCACTCCAGAGAGGTCTGAGAC 357
Macaca  GGCATGAGCCCGGCGGGGGGGGGGCCTCGGCAGAGCAGTGTTGGGGGGAAACGGGGGAAGGAGGCTGTGAGCAGGTGTCACTCCAGAGAGGTCTGAGAC 352
Bos     GGCACAAGCCC-------AGAGAGGTCCAGGCGGTGC----------GGGGACTGGGGAAAGGAGGATGTGAGCAAGGGTCACCCTGTAGGGATCTCAGAT 370
Canis   GGCATGAGCCC-------AGAGGGATACTGG----GA----------GGGGACTGAGGGAAGGAAGCTGTAAGCAGAGGTCACCCCAAAACTGTCTAAGA- 360

Homo    TCTGCCCCACGCTCTCCCTAGGGCCCTGGGGGGTGGGGAGAGGGG-----------------------CTCTTCTCAGGACCCCACCCAAGGGGCCTGGG 434
Pan     TCTGCCCCACGCTCTCCCTAGGGCCCTGGGGGGTGGGGAGAGGGG-----------------------CTCTTCTCAGGACCCCACCCAAGGGGCCTGGG 434
Macaca  TCTGCCCCACGCTCTCCCTAGGGTCCTGGG----GGCGGGAGGGG-----------------------CTCTTCTCAGGACCCCACCCAAGGGGCCTGTG 425
Bos     CCTGCCCCACACCATCCCTAGGGCCCTGGGCGGGCGGGGGGGGGGGGGCGCGGGTGGGATGTCGTGTATCTACTCAGGACCCAACCCAGAGGGCCTATG 470
Canis   -CTGCCACAAGACCTCCCTAGGGCCCTGGGGGTATGAGGGG---------------------------CTCTCCTTAGGACACTACCCTG-GGACCTGGG 431

Homo    ACCAGCAGCTTTTGCCTCCCCCAG 458
Pan     GCCAGCAGCTTTCGCCTCCCCCAG 458
Macaca  GCCAGCAGCTTTCGCCTCCCCCAG 449
Bos     GCCAGCAGCTCTCTCCTCCCC-AG 493
Canis   ACCAGCAACTCTCCCTTCCCCTAG 455
```

ENSG00000108439 intron 3

Description: Pyridoxine-5’-phosphate oxidase (PNPO)
Intron number: 3
Human chromosome: 17
Intron start (bp): 43377081
Human intron length : 843
Intron alignment length: 880
Flanking exons length (upstream/downstream): 100/54
SNP density: 0.002372
K tree score: 0.0561
Scaling factor: 0.8314
Human-chimpanzee distance: 0.013733
Total primate branch length: 0.0856

ENSG00000108439 exon 3

```
Homo    AGATGGAAAACCCTCTGCTCGCATGTTGCTGCTGAAGGGCTTCGGGAAAGATGGCTTCCGCTTCTTCACTAACTTCGAGA 80
Pan     AGATGGAAAACCCTCTGCCCGCATGTTGCTGCTGAAGGGCTTCGGGAAAGATGGCTTCCGCTTCTTCACTAACTTCGAGA 80
Macaca  AGATGGAAAACCCTCTGCTCGCATGTTGCTACTGAAGGGCTTTGGGAAAGATGGCTTCCGCTTCTTCACTAACTTCGAGA 80
Bos     AGATGGAAAACCGTCTGCCCGCATGGTGCTGTTGAAGGGCTTTGGCAAGGATGGCTTCCGCTTCTTCACTAACTTCGAGA 80
Canis   AGATGGAAAACCTTCTGCCCGCATGGTACTGCTGAAGGGCTTTGGCAAGGATGGCTTCCGCTTCTTCACTAACTTCGAGA 80

Homo    GTCGAAAAGGAAAAGAGCTG 100
Pan     GTCGAAAAGGAAAAGAGCTG 100
Macaca  GTCGAAAAGGAAAAGAGCTG 100
Bos     GTCGAAAGGGAAAAGAGCTG 100
Canis   GTCGGAAGGGAAGAGAGCTG 100
```

ENSG00000108439 exon 4

```
Homo    GACTCTAATCCCTTTGCTTCCCTTGTCTTCTACTGGGAGCCACTTAACCGTCAG 54
Pan     GACTCTAATCCCTTTGCTTCCCTTGTCTTCTACTGGGAGCCACTTAACCGTCAG 54
Macaca  GACTCGAATCCCTTTGCTTCCCTTGTCTTCTACTGGGAGCCACTTAACCGTCAG 54
Bos     GACTCGAATCCCTTTGCTTCCCTTGTCTTCTACTGGGAGCCCCTGCACCGTCAG 54
Canis   GACTCCAATCCCTTTGCCTCCCTTGTCTTCTACTGGGAGCCCCTCAACCGCCAG 54
```

ENSG00000108439 intron 3

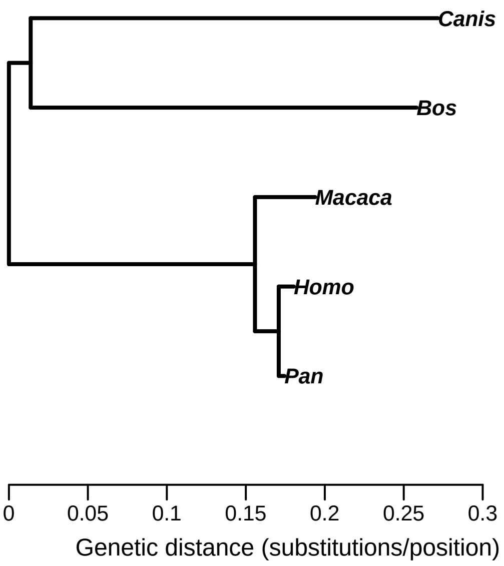

ENSG00000108439 intron 3

```
Homo    GTGGGTGAAAAGAGCTAGTAATCTTTCCCAGGGCCTGCAGGGTTTGGCTCTTGCTTCCTCAGTCACCTTCTTATGCCACCCCACCCCCCCAGTCTACTTG 100
Pan     GTGAGTGAAAAGAGCTAGTAATCTTTCCCAGGGCCTGCAGGGTTTGGCTCTTGCCTCCTCAGTCACCTTCTTATGCCACCCCACCCCCCCAGTCTACTTG 100
Macaca  GTGTGTGAAAAGAGCTGGTAATCTTTCCCAGGGCCTGTGGGATTTGGCCCTTTCCTCCTCAGTCACCTTCTCATGCTACCCCA-CCCCTCAGTCTACTTG 99
Bos     GTGAGGGTTCA----CGGTAAACCCTTCCTGGGCCTGCAGGGTCCAGCCCTGGCTT-CTTGGTCGCCTACTCATGCCACCCC--TGCCCGAGTCCACTTG 93
Canis   GTGAG-------------------------GGGCCTGCAGGGGCCTGTCCTTCCTT---------CCCTCTGGTGCTAGCCC--CACCCCTGCCCACTTG 64

Homo    CTCTCTGTGTGCAGGTGCCCTCCTGCCATGAGGCCTTGGCACTTGCTGTGCTCTTTGCCATAAATGAATGAATGCACTTTCTAACCAGCCTCACCCCATC 200
Pan     CTCTCTGTGTGCAAGTGCCCTCCTGCCATGAGGCCTTGGCACTTGCTGTGCTCTTTGCCATAAATGAATGAATGCACTTTCTAACCAGCCTCACCCCATC 200
Macaca  CTCTCTCTCTGCAGGTGCCCTCCTGCCATGAGGCCTTGGCACTTGCTGTGCTCTTTGCCATAAATGAATGAATGCACTTTCTAACCAACCTCACCCCATC 199
Bos     CCAGCCACGCTCAGGCACTGTCCTGACTAGAGGCCTTTGCACTTGCC-TTCTCTCTGCT----GTGAATGAGGGCAAGCTTTAAGCACCTCCACCTCCCT 188
Canis   CTGGCTGTGTTTGGGCACCGTCCTGCCTGCGGGCCTTGGCACTCTTCCTTTTCTTTGCC----ATGAATGAGTGCTCGCTCTGACCACCCCGCCTCTGCC 160

Homo    AGCCAT------------GACCTGTTCCTTCAGATCTCAGCCCACATGTTACCTCCTCAGGAAGGCCTTCCTTGACTCTCACTCAGACTCAGCCCCTCAT 288
Pan     AGCCAT------------GACCTGTTCCTTCAGATCTCAGCCCACATGTTACCTCCTCAGGAAGGCCTTCCTTGACTCTGACTCAGACTCAGCCCCTCAT 288
Macaca  AGCCAT-------------CACCTGTTCCTTCAGATCTCAGCCCACATGTTACCTCCTCAGGAAGGCCTTCCTTGACTCTGACTCAGACTCAGCCCCTCAT 287
Bos     AGCTGTTTATCTGAGTAGCTCCTGTTTCTTCGGGTCTCAGCCCCCACGTCACCTCCTCACGGAAGCCT--CTGGATTCCAGGCTGAGCCAGCCCCTCTC 286
Canis   ATCCACTTACCTAAGTAACTCCCAACCCTTTGGCTTTCAGCCCCTGTGCTACCTTCTAGGGAAAGTCATCCCTGATCCCTATGCTACCTCAGC------T 254

Homo    GCTGTTTGCTGCCATTATAACTTGCAACTTTCTTTTTTGTCTTCTTCTTCAGTTTGTAACTGAGCATTTGTTTGGGTGATTATGTAACTGATATTTGTCT 388
Pan     GCTGTTTGCTGCCATTATAACTTGCAACTTTCTTTTTTGTCTTTTTCTTCAGTTTGTAACTGAGCATTTATTTGGGTGATTATGTAACTGATATTTGTCT 388
Macaca  GTTGTTTGCTGCCATTATAACTTGCAACTTTCCTTTTTATCATTTTCTTCAGTTTGTAACTAAGCTTTTATTTGGGTGATTATGTAACTGATATTTGTCT 387
Bos     ACTTT----GGTCACTATAATTTACAAATTTCCTTTCTGCCATTGT-TTTAGTTTCTAACT--GCGTGTATTTGGGGGATTATGTGATTAATGTTTATGT 379
Canis   GCCATTCACCATCATCTTAACGTGTCACTTTACTTTCTACCTTCTT-TTCAGTTTGTGACT--GCATTTGTTGGTGTGTTTATGTGATTGATACTTGTCT 351

Homo    CCTCTGCTCAA----CAGTAAGCTCTGTAGGAGTGGAGGCTGC-TCTGTTTTATCTCTGCTGTATCCCCCGCACGTCTCCTGTTGTCAGACCCAGAGTGG 483
Pan     CCTCTGCTCAA----CAGTAAGCTCTGTAGGAGTGGAGGCTGC-TCTGTTTTATCTCTGCTGTATCCCCCGCACATCTCCTGTTGTCAGACCCAGAGTGG 483
Macaca  CCTCTGCTGAA----CAGTAAGCTCTGCAGGAGTGGAGGATGC-TGTGTTTTATCTCTGCTGTATCCCCCACATGTCTTCTGTTGTCAGACCCAGAGTAA 482
Bos     CCTCTGCTAAA----TGGTAAGCTCCGTGGGGGCGGGGCTGCT-TCTGAGATGTCTCTGTTAAACCCCCAGCACCTCTCCTGGTGTTGAGCACAGAGCTG 474
Canis   CCCTTGCTAAATACCCAGTAAGCTCTGTGGAGGCAGAGGCTGCATCCGTTGTACCTCTCCCATTCCCCGAGCACCTCTCCTGGGGTCAAGCCCAGAGTTG 451

Homo    GCACTTCGCGTGCATTCAATGAATGAATCCAAAATGAGTAAACCT-CCCTGAGTCCATGCCAGCCAAGCATGCATTTCTCAAGACTCACAAATCTGTGGG 582
Pan     GCACTTCGCGTGCATTCAATGAATGAATCCAAAATGAGTAAACCT-CCCTGAGTCCATGCCAGCCAAGCATGCATTTCTCAAGACTCACAAATCTGTGGG 582
Macaca  GCACTTCGTGTGCATTCAGTGAATGAATCCAAAGTGAGTAAACCT-CCCTGAGTCCATGCCAGCCAAACATGCATTTCTCAAGACTCGCAAATCTGTGGG 581
Bos     GCACTTCCTATACGTTCAGTTAATGAATCTAAAATGAGTAGCTCTCCCCCAAGTCCAGTGCAGTCAAATGTGCATTTCAGAGGATTTGCAAATCTGTGGG 574
Canis   ATACTTTGTGTGCATTCAGTC-ATGAACCCAAAGGA--------CTCCCGGAATCCATACTGGCCACAGGTGCATTTCTGAGGACTTGCAAATCTATGGA 542

Homo    TGAGAGAAATCACCCAGTCTCACTTTGGAGTCCGACTGGCTTAAACTT-------AGCTCCACCACTTCCTGCAGGAACTTGGGCACGTGA--------- 666
Pan     TGAGAGAAATCACCCAGTCTCACTTTGGAGTCCGACTGGCTTAAACTT-------AGCTCCACCACTTCCTGCAGGAACTTGGGCATGTGA--------- 666
Macaca  TGAGAGAAATCACCCAGTCTCACTTTGGAGTCTGACTGGCTTAAACTT-------AGCTCCACCACTTACTGCAGGAACTTGGGCATGTGA--------- 665
Bos     TGAGAGAAAT-GGCAAGCCTCACTTTGGAGTCCCTCTGGCTGAGGTTTTCAGCGCAGGTCTTCCATTGACTCTGTGAACCTGGGAGTGTTAGTGAACCTC 673
Canis   TGAGAGAAAT-GGCAAGCCTCACTTTGGAATCCATCTGGCTTAGGTTTTCAGCACGGCTCTAGCACTTACTGTGTGAACTTAGGCAGGTTCCTTAACCTC 641

Homo    -CTTAGCCTGTCTCT-GTGTCTGTGACAATAGGCCTACTTTTGAGGGTTTAAATGAGCTCTTACGGTGGGTGCATCTGCTGTGGGCCTGCGCACTACAGC 764
Pan     -CTTAGCCTGTCTCC-GTGTCTGTGACAATAGGCCTACTTTTGAGGGTTTAAATGAGCTCTTACGGTGGATGCATCTGCTGTGGGCCTGCGCACTACAGC 764
Macaca  -CTTAGCCTGTCTCC-GTATCTGTGACAATAGGCCTACTTTTGAGGGTTTAAATGAGCTCATACAGTGGATGCATCTGCTGTGGGCCTGCACACTACAGC 763
Bos     TCTGAGCCTATCTCC-ACGCCTGTAAAAGTAGGCCCACCTTTGGGGACTT-AACAAGATGCTACAGTAAATGCATCTGCAGTGGGCCTGCGCCGTGCAGA 771
Canis   TCTGAGTCTGTCTCCTACACCCGTAAAAGTAGGCATACCTTTGAGGGTTTAAATGAGATGTTTTGTT------ATCCCCTGTATGCCTGCATGCTGCAGA 735

Homo    TCTCCTGCCTTTTCCCTGCATGCCGGAGGCCTCCTCTCC-CTGTCCTGATGGCTGGCTGTGGATTCTCTTTTACTTCTAG 843
Pan     TCTCCTGCCTTTTCCCTGCATGCCGGAGGCCTCCTCTCC-CTGTCCTGACGGCTGGCTGTGGATTCTCTTTTACTTCTAG 843
Macaca  TCTCCTGCCTTTTCCCTGCATGCCGGAGGCCTCCTCTCC-CTATCCTGATGACTGGCTGTGGATTCTCTTTTACTTCTAG 842
Bos     CCCCCTAGCTTCCCCCTGTGCCC---AGGCCTCCTCTCTGCTGTCCTGATGACCGTCTGTGGATTCTCTTCCATTTCCAG 848
Canis   CTTCCTACCTCCTTTCTGCACGGTGGAGGTGTTCTCCCTGCTGTTTTGATGGCCGACGGCGAATCCTCTTTTATTTCCAG 815
```

ENSG00000165076 intron 4

Description: Peptidase S1 domain-containing protein LOC136242 Precursor
Intron number: 4
Human chromosome: 07
Intron start (bp): 141182805
Human intron length : 576
Intron alignment length: 616
Flanking exons length (upstream/downstream): 137/141
SNP density: 0.008681
K tree score: 0.0435
Scaling factor: 1.0134
Human-chimpanzee distance: 0.009137
Total primate branch length: 0.0854

ENSG00000165076 exon 4

```
Homo    GCCGACACCCTGACTTGCGGCAGAACCTGGAGGCCCCCGTGATGTCTGATCGAGAATGCCAAAAAACAGAACAAGGAAAA  80
Pan     GCCGACACCCTGACTTGCGGCAGAACCTGGAGGCCCCCGTGATGTCTGATCGAGAATGCCAAAAAACAGAACAAGGAAAA  80
Macaca  GCCGACACCCTGACTTGCGGCAGAACCTGGAGGCCCCTGTGATGTCTGATAAAGAATGCCAGAAAACAGAACAAGGAAAA  80
Bos     GCAGACACCCTGATTTAAGGCAGAACCTGGAGGCCCCCGTGATGTCCGACACAGCCTGCCAGGAAACCGAACAAGGAAAA  80
Canis   GTAGACATCCTGATTTAAGGCAGAACCTGGAGGCTCCTGTGATGTCTGATAAAGAGTGCCAGAAAACCGAACAAGGGAAA  80

Homo    AGCCACAGGAATTCCTTATGTGTGAAATTTGTGAAAGTATTCAGCCGAATTTTTGGG  137
Pan     AGCCACAGAAATTCCTTATGTGTGAAATTTGTGAAAGTATTCAGCCGAATTTTTGGG  137
Macaca  AGCCACAGGAATTCCTTATGTGTGAAATTTGTGAAAGTATTCAGCCGAATTTTTGGG  137
Bos     AGCCACAGAAACTCCATATGTGTTAAATTTCTAAAAGTGTTCAGTCGAATTTTTGGG  137
Canis   AGCCACAGGAACTCCTTATGTGTTAAATTTGTGAAAGTCTTCAGCCGAATTTTTGGG  137
```

ENSG00000165076 exon 5

```
Homo    GAGGTGGCCGTTGCTACTGTCATCTGCAAAGACAAGCTCCAGGGAATCGAGGTGGGGCACTTCATGGGAGGGGACGTCGG  80
Pan     GAGGTGGCCGTTGCTACTGTCATCTGCAAAGACAAGCTCCAGGGAATCGAGGTGGGGCACTTCATGGGAGGGGACGTCGG  80
Macaca  GAGGTGGCCGTTGCTACTGTCATCTGCAAAGACAAGCTCCAGGGGATCGAGGTCGGGCACTTCATGGGAGGGGATGTCGG  80
Bos     GAGCTGGCCGTTGCTACTGTCATCTGCAAAAACAAGCTGCAGGGGATCGAGGTCGGACACTTCATGGGAGGGGATGTGGG  80
Canis   GAGGTGGCCGTCGCTACCGTCATCTGCAAAAACAAGCTCCAGGGGATTGAGGTTGGACACTTCATGGGAGGAGATGTTGG  80

Homo    CATCTACACCAATGTTTACAAATATGTATCCTGGATTGAGAACACTGCTAAGGACAAGTGA  141
Pan     CATCTACACCAATGTTTACAAATATGTATCCTGGATTGAGAACACTGCTAAGGACAAGTGA  141
Macaca  CATCTACACCAATGTTTACAAATATGTATCCTGGATTGAGAACACTGCTAAGGACAAGTGA  141
Bos     CATCTACACCAACGTTCAGAAATACGTCAGCTGGATTGAGAGCACCACTAAAGACAAATGA  141
Canis   AATCTATACCGATATTCACAAATATATACCCTGGATTGAGAGTATCACTAAAGACAAATGA  141
```

ENSG00000165076 intron 4

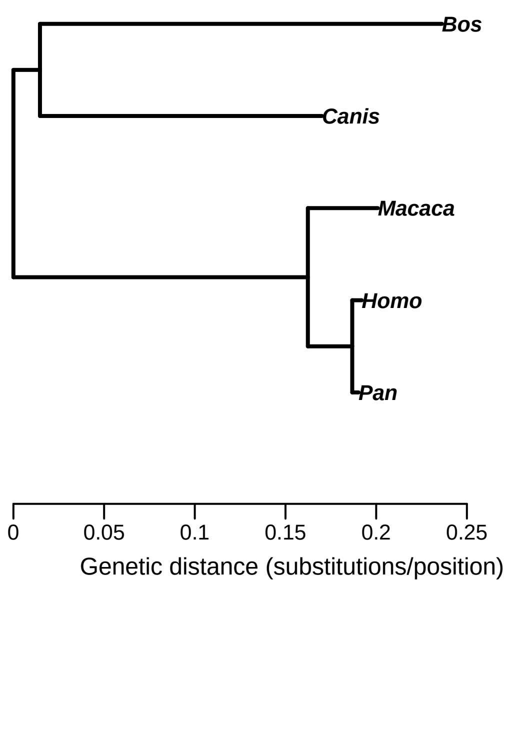

ENSG00000165076 intron 4

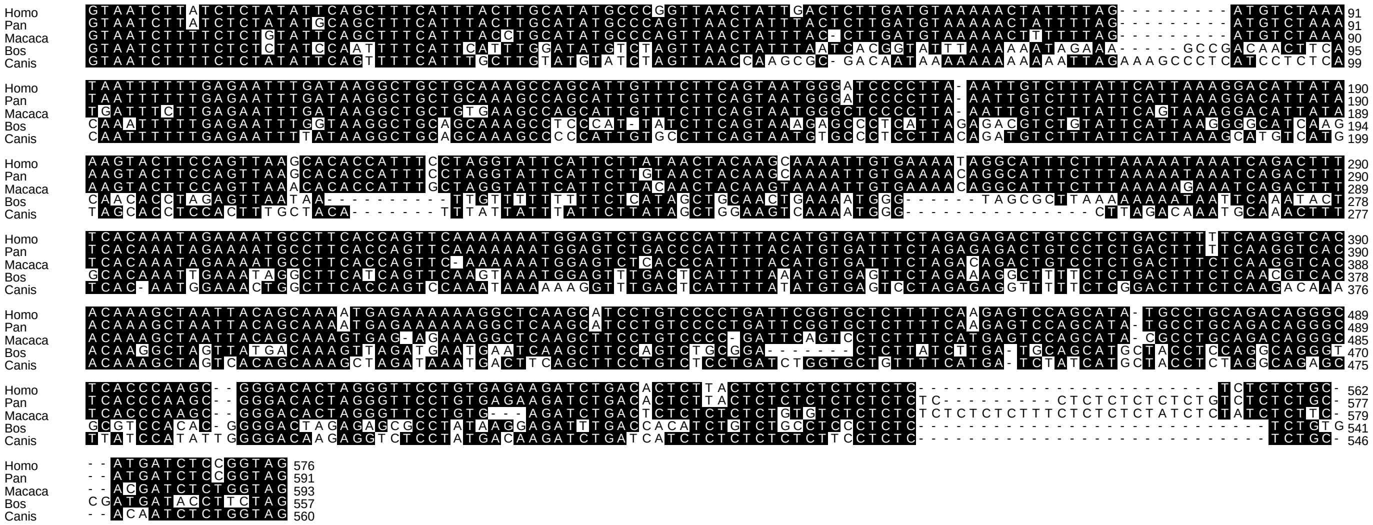

ENSG00000185662 intron 3

Description: Uncharacterized protein C5orf50 (C5orf50)
Intron number: 3
Human chromosome: 05
Intron start (bp): 171150159
Human intron length : 245
Intron alignment length: 262
Flanking exons length (upstream/downstream): 68/195
SNP density: 0.000000
K tree score: 0.0932
Scaling factor: 0.9545
Human-chimpanzee distance: 0.016554
Total primate branch length: 0.0849

ENSG00000185662 exon 3

```
Homo    GAAGCAGTTGGGAGGTGTCAGAAAGGATCAGAGAATGTAACTACTACCAGAATCTTGCAGTTCCCCAG 68
Pan     GAAGCAGTTGGGAGGTGTCAGAAAGGATCAGAGAATGTAACTACTACCAGAATCTTGCAGTCCCCCAG 68
Macaca  GAAGCAGTTGGGAGGTGTCAGAAAGGATCAGAGAATGTAACTACTACCAGAATCTTGTAGTTTCCCAG 68
Bos     GAAGAAGTTGGGAGGTGTCAGAAAGGATCAGAGAATGTAACTACCCCCAGAATCCTGTGACTTCCCAG 68
Canis   GAAGAAGTTGGGAGGTGTCAGAAAGGATCAGAGAATGTAACTATCCCCAGAATCCTGTGGCTTCCCAG 68
```

ENSG00000185662 exon 4

```
Homo    GGGCTTGAATATCAGACCAACGAGCCCTCAGAAGAACCGATAAAGACCATCAGGAACTGGCTGAAGGAGAAGTTGCATGT 80
Pan     GGGCTTGAATATCAGACCGACGAGCCCTCAGAAGAACCGATAAAGACCATCAGGAACTGGCTGAAGGAGAAGTTGCATGT 80
Macaca  GGGCTTGAATATCAGACCGATGAGCCTTCGGAAGAACCGATAAAGACCATCAGGAACTGGCTGAAGGAGAACTTGCATGT 80
Bos     GTGTTTGAATACCAGACCAGTGACTCCCCAGAAGAGCCGATCAAGGTCATGAGGACCTGGTTGAAAGAGAACTTGCATGT 80
Canis   GGATTTGAATACCAGACCAATGAACCTGCAGAAGAGCCAATAAAGGTCATCAGGACCTGGTTGAAGGAAAATTTGCATGT 80

Homo    CTTCTCGGAGAAGTTAGAGGAAGAGGTGCAGCAGCTGGAGCAGCTAGCGTGGGACCTGGAACTGTGGCTGGATGCTCTTC 160
Pan     CTTCTCGGAGAAGTTAGAGGAAGAGGTGCAGCAGCTGGAGCAGCTAGCGTGGGACCTGGAACTGTGGCTGGATGCTCTTC 160
Macaca  CTTCTTGGAGAAGTTAGAGGAAGAGGTGCGAGAGCTGGAGCAGCTAGTGCGGGACCTGGAACTGTGGCTGGATGCTCTTC 160
Bos     TTTCTTGGAGAAGCTAGAGAAAGAGGTGCAAGAGCTGGAGCAGCTGGTCCGGGACCTGGAGGAATGGCTGGATGTCCTCC 160
Canis   TTTCTTGGAGAAGCTAGAGAAAGAGGTGCAAGAACTGGAGCAGCTGGTACAGGACCTGGAGGAGTGGTTAGATGCTCTTC 160
```

ENSG00000185662 intron 3

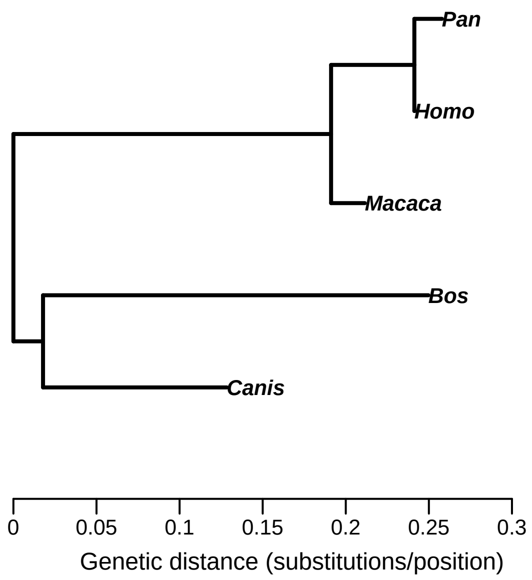

ENSG00000185662 intron 3

```
Homo    GTAACATGTCC-AGCAAGGTACTGAGG----------------TGAGGCAATCTGGGAAGGCTTTCCAGAGGAGGTGTCATTGGCAACATGGGTCATGAA 83
Pan     GTAATATGTCC-AGCAAGGTACTGAGG----------------TGAGGCAATCTGGGAAGGCTTTCCAGAGGAGGAGTCATTGGCAACATGGGTTACGAA 83
Macaca  GTAACATATCC-AGCAAGGTACTGAGG----------------TGAGGCAATCTGGGAAGGCTTTCCAGAGGAGGTGTCATTGGCAACATGGGTCATGAA 83
Bos     GTAACATATTCAAGAAGGGACCTTAAG----------CTTGGCTGAGGACATCTGAGAAGGCTTTCTG---TAGGTGTCATTGATGAACTGGGTCTTGAA 87
Canis   GTAACATATTCAAAAAAGGTACTGAGGGGATGTCAAATTTAGCTGAGGAGATCTGGGAAGGCTTCCCA---GGGGTGTCATGGGCGAGCTGGACCTTGAA 97

Homo    AGATAAGTAGTCGCTTGTCAAGTGCAGAAGGTGGGAACAGGTACTACGAGCACAATGAGTAGCATGTGCAAAGGCCCAGGGATCTTTTTGAAACCAGTGG 183
Pan     AGATAAGTAGTCGCTTGTCAAGTGCAGAAGGTGGGAACAGGTACTACGAGCACAATGAGTAGCATGTGCAAAGGCCCAGGGATCTTTTTGAAACCAGTGG 183
Macaca  AGATAAGTAGGAGCTGGCCAAGTGTAGAAGGTG------GGTACTATGAGCACGATGAATAGCACGTGCCAAGGCCCAGGGATCTTTTTGAAACCAGTGG 177
Bos     GGATAGGTAGGAGTTGGTCTGGTAGAGAAGGTGGGGATGGGTACTTGGGACAGAAGGAA-AGCATA-GCAAAGGCTCGGGGAACTTGTAGGAACCAGCAC 185
Canis   GGACATGTAGGAGCTGGCCAGGTGGAGAAGGTGGGGATGAGCACTCCAGACAGAAGGAATAACATGTTCAAACACTCAGAAAACTTTTTGAAACCAGT-- 195

Homo    GGATGGGGAGGAGGGTCCTATCTGAGAAAGCACAGGATGCCACTTCTGTGTCTGCCTTCCAG 245
Pan     GGATGGGGAGGAGGGTCCTATCTGAGAAAGCACAGGATGCCACTTCTGTGTCTGCCTTCCAG 245
Macaca  GGGCGGGGAGGAGGGTCCTATCTGAGAAA---------GCCACTTCCATGTCTGCCTTCCAG 230
Bos     A-----GCAAGAGAGGTCTACAGGAAAATGCTCAAGATACAACCTTGGTGTCTGCCTTTTAG 242
Canis   ------GACAGGGGATTCTATGTGAAAAAGCTCAAGATGCAACTTTGATGTCTGCCTTTCAG 251
```

ENSG00000078898 intron 4

Description: Bactericidal/permeability-increasing protein-like 1 precursor (BPIL1)
Intron number: 4
Human chromosome: 20
Intron start (bp): 31065424
Human intron length : 1479
Intron alignment length: 2000
Flanking exons length (upstream/downstream): 147/61
SNP density: 0.003381
K tree score: 0.0833
Scaling factor: 0.656
Human-chimpanzee distance: 0.009663
Total primate branch length: 0.0848

ENSG00000078898 exon 4

```
Homo   CGCCCCAGAGCCCCTGGAGCTGACGCTGCCTGTGGAACTGCTGGCTGACACCCGCGTGACCCAGAGCTCCATCAGGACCC 80
Pan    CGCCCCAGAGCCCCTGGAGCTGACGCTGCCTGTGGAACTGCTGGCTGACACCCGCGTGACCCAGAGCTCCATCAGGACCC 80
Macaca CGCCGCAGAGCCCCTGGAGCTGACGCTGCCTGTGGAACTGCTGGCTGACACCCGTGTGACCCAGAGCTCCATCAGGACCC 80
Bos    TGACCCGGAGCCCCTGCACCTGCTGCTCCCCATAGCACTGCTGGCTGACACCTCCGTGGAGCAGGGCTCCATCGGGACCC 80
Canis  AGTCCCGGAGCCCCTGAAGCTGGTGCTGCCCGTGGTACTGCTGGCCGACGCCCAGGTGGCCCAGGGCTCCATCCGGACCC 80

Homo   CTGTGGTCAGCATCTCTGCCTGCTCTTTATTCTCGGGCCACGCCAACGAGTTTGATGGCAGTAACAG 147
Pan    CTGTGGTCAGCATCTCTGCCTGCTCTTTATTCTCGGGCCACGCCAACGAGTTTGATGGCAGTAACAG 147
Macaca CTGTGGTCAGCATCTCTACCTGCTCTTCATTCTCAGGCCACGCCAACGAGTTTGATGGTAGTAACAG 147
Bos    CCGTGGTCAGCATCTCCAGCTGCTTTTCCATCTTCGACAAAGCCATCGTGTTTGATGGCCGCAACAG 147
Canis  CCGTGGTCAGCATCTCTGCCTGCTTCGCACTCTTCAACAGTGCCGCGGTGCTGGATGGCAGTACCAG 147
```

ENSG00000078898 exon 5

```
Homo   CACCTCCCACGCGCTGCTGGTCCTGGTGCAGAAGCACATTAAAGCTGTCTTGAGTAACAAG 61
Pan    CACCTCCCACGCGCTGCTGGTCCTGGTGCAGAAGCACATTAAAGCTGTCTTGAGTAACAAG 61
Macaca CACCTCCCACGCACTGCTGGTCCTGCTGCAGAAGCACATTAAAGCTGTCTTGAGTAACAAG 61
Bos    CACAGCCCCCGCGATGCTGGCCCCGCTGCAGAGTCACATCACAGCTGTGCTGAAGAGCAAG 61
Canis  CGTGGCCCCTGGGCTGCTGGTCCTGGTGCAGAATCATATTAAAGCTGTCCTGCGGAACAAG 61
```

ENSG00000078898 intron 4

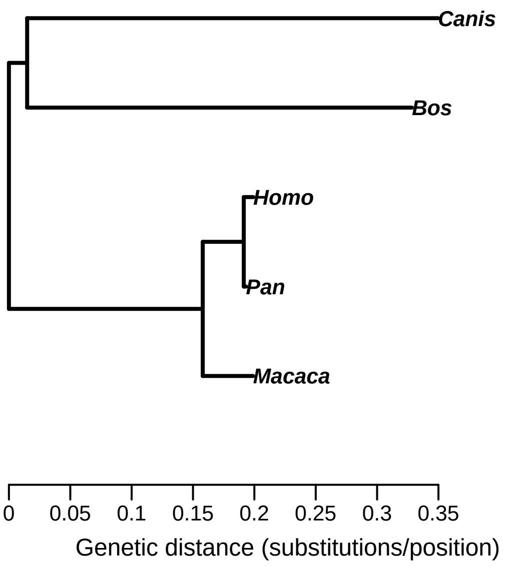

ENSG00000078898 intron 4

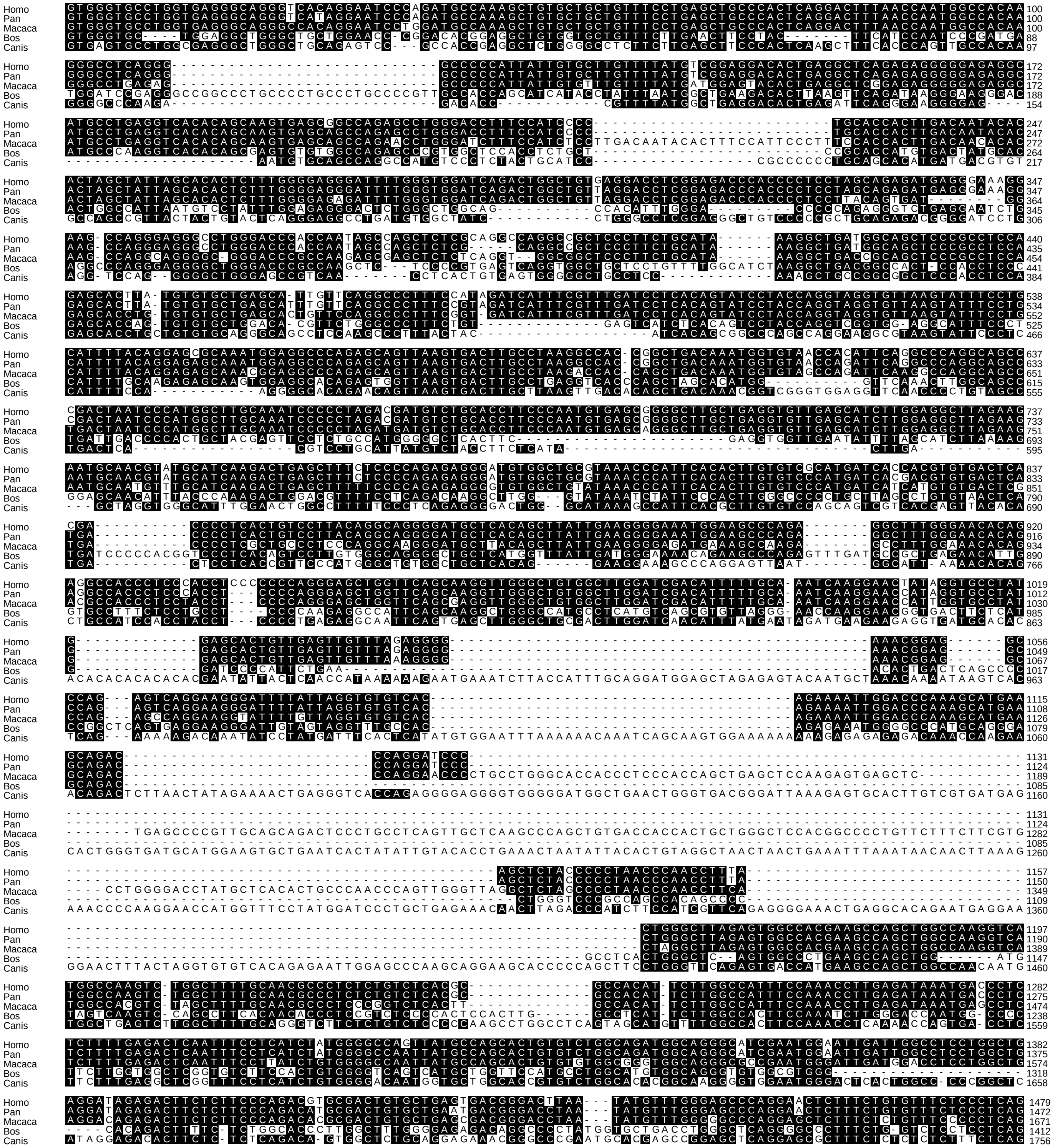

ENSG00000148331 intron 2

Description: Ankyrin repeat and SOCS box protein 6 (ASB6)
Intron number: 2
Human chromosome: 09
Intron start (bp): 131441609
Human intron length : 1032
Intron alignment length: 1143
Flanking exons length (upstream/downstream): 182/107
SNP density: 0.002907
K tree score: 0.0967
Scaling factor: 0.7168
Human-chimpanzee distance: 0.011759
Total primate branch length: 0.0843

ENSG00000148331 exon 2

```
Homo    GAGAGCCGAATCCTTGTTCTCACTGAGCTGCTGGAGAGGAAAGCCCACTCTCCCTTTTACCAGGAAGGCGTGAGCAACGC 80
Pan     GAAAGCCGAATCCTTGTTCTCACTGAGCTGCTGGAGAGGAAAGCCCACTCTCCCTTTTACCAGGAAGGCGTGAGCAACGC 80
Macaca  GAGAACCGAATCCTTGTTCTCACTGAGCTGCTGGAGAGGAAAGCCCACTCTCCCTTTTACCAGGAAGGTGTGAGCAACGC 80
Bos     GAGAGCCGCATCCTTGTTCTCACCGAGCTGCTGGAAAGAAAAGCCCACTCTCCATTTTACCAGGAAGGCGTGAGCAACGC 80
Canis   GAGAGCCGAATCCTTGTTCTCACCGAGCTGCTGGAGAGAAAGGCGCACTCTCCATTTTACCAGGAAGGTGTGAGCAATGC 80

Homo    CCTGCTCAAGATGGCTGAGCTGGGGCTGACGCGGGCGGCCGACGTTCTCTTGCGGCATGGGGCCAATCTCAACTTTGAAG 160
Pan     CCTGCTCAAGATGGCTGAGCTGGGGCTGACGCGGGCGGCCGACGTTCTCTTGCGGCATGGGGCCAATCTCAACTTTGAAG 160
Macaca  CCTGCTCAAGATGGCTGAGCTGGGGCTGACCCAGGCGGCCGACGTTCTCTTGCGGCATGGGGCCAATCTCAACTTTGAAG 160
Bos     CCTGCTGAAGATGGCCGAGCTGGGGCTGACACGCGCAGCTGCCGTGCTCCTGCGGAATGGAGCCAACCTCAACTTTGAAG 160
Canis   CCTGCTGAAGATGGCCGAGCTGGGACTGACCCGGGCCGCCGACGTTCTCCTGCGCAATGGGGCCAACCTCAACTTTGAAG 160
```

ENSG00000148331 exon 3

```
Homo    ACCCAGTCACCTACTACACGGCCTTGCACATCGCCGTCCTGCGGAACCAGCCGGACATGGTGGAGCTGCTGGTGCATCAC 80
Pan     ACCCAGTCACCTACTACACGGCCTTGCACATCGCCGTCCTGCGGAACCAGCCGGACATGGTGGAGCTGCTGGTGCATCAC 80
Macaca  ATCCAGTCACCTACTACACGGCCTTGCACATCGCCGTCCTGCGGAACCAGCCAGACATGGTGGAGCTGCTGGTGCATCAC 80
Bos     ACCCCGTCACCTACTACACGGCACTGCATATCGCCGTCCTGCGAAACCAGCCTGACATGGTGGAGCTGCTGGTGCGCCAC 80
Canis   ACCCTGTCACTTACTACACGGCCCTGCACATCGCCGTCCTGCGAAACCAGCCGGACATGGTGGAGCTGCTGGTGCGCCAC 80

Homo    GGGGCCGACGTTAATCGGAGGGACCGG 107
Pan     GGGGCCGACGTTAATCGGAGGGACCGG 107
Macaca  GGCGCTGACATTAATCGGAGGGACCGG 107
Bos     GGGGCCGACATCAACCGGAGGGACCGG 107
Canis   GGGGCCGACATTAACCGGAGGGACCGG 107
```

ENSG00000148331 intron 2

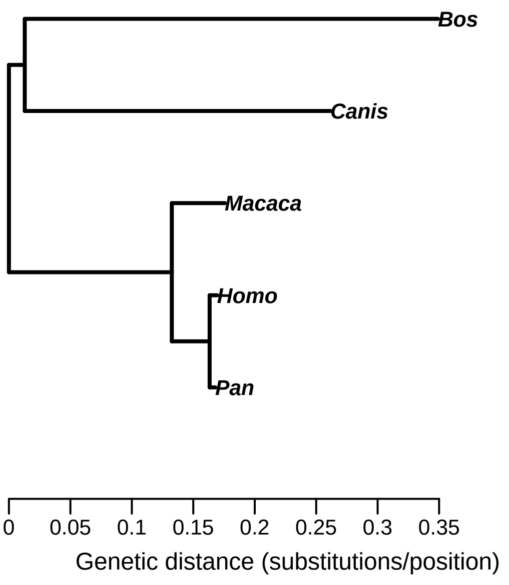

ENSG00000148331 intron 2

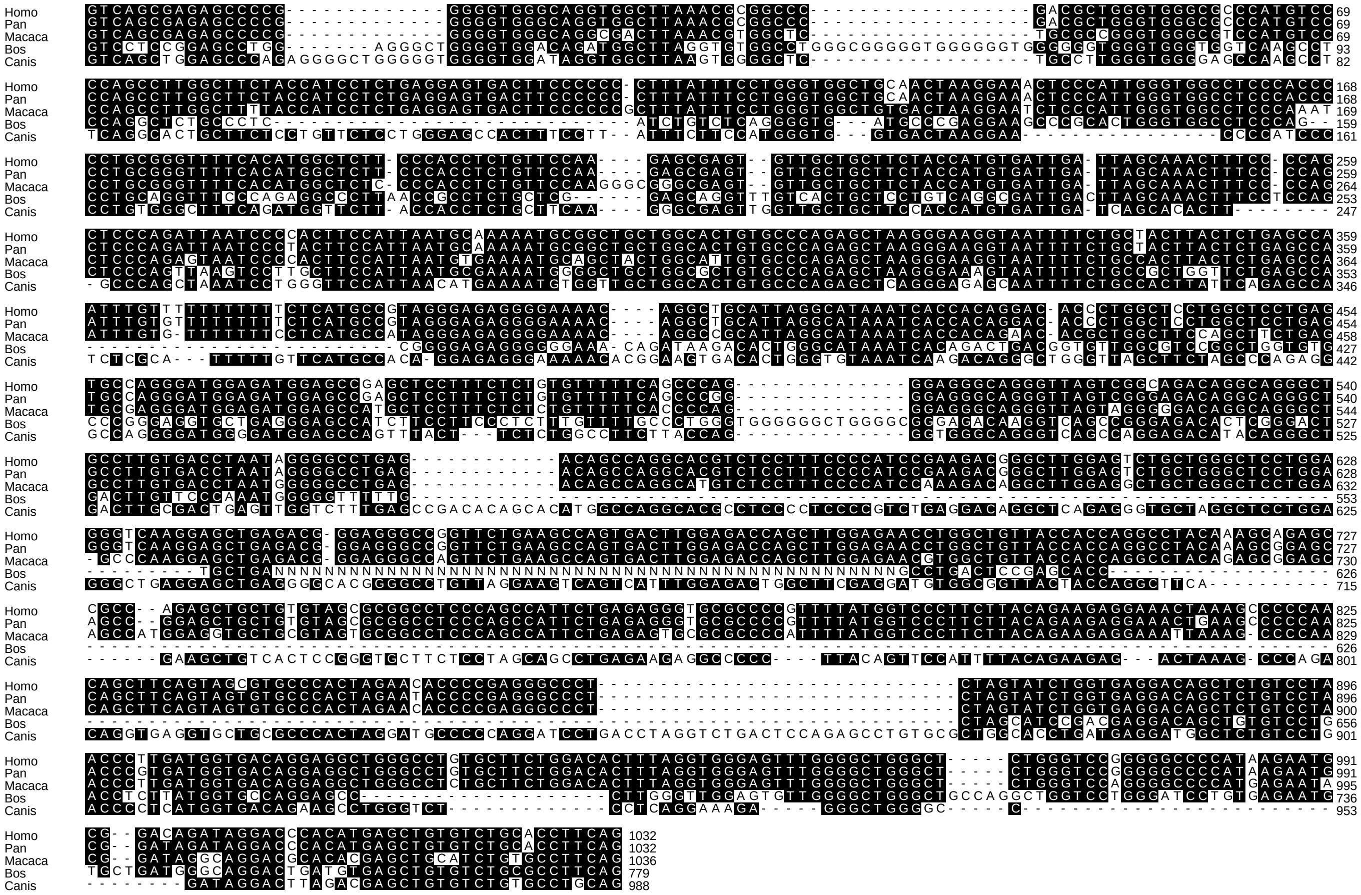

ENSG00000106638 intron 3

Description: Transducin beta-like 2 protein (TBL2)
Intron number: 3
Human chromosome: 07
Intron start (bp): 72625738
Human intron length : 466
Intron alignment length: 636
Flanking exons length (upstream/downstream): 185/152
SNP density: 0.000000
K tree score: 0.0726
Scaling factor: 1.108
Human-chimpanzee distance: 0.017479
Total primate branch length: 0.0842

ENSG00000106638 exon 3

```
Homo    TGGACTTTAGCAGCAATGGCAAATACCTGGCTACCTGTGCAGATGATCGCACCATCCGCATCTGGAGCACCAAGGACTTC  80
Pan     TGGACTTTAGCAGCAATGGCAAATACCTGGCTACCTGTGCAGATGATCGCACCATCCGCATCTGGAGCACCAAGGACTTC  80
Macaca  TGGACTTTAGCAGCAATGGCAAATACCTGGCCACCTGTGCAGATGATCGCACCATCCGCATTTGGAGCACCAAGGACTTT  80
Bos     TGGACTTCAGCAGCAATGGCAAGTACCTGGCTACCTGTGCAGACGACCGTACCGTCCGCATCTGGAGCACAAAGGACTTC  80
Canis   TGGACTTTAGCAGCAATGGCAAGTACCTGGCCACCTGTGCAGATGACCGCACTGTCCGCATCTGGAGCACCAAGGACTTC  80

Homo    CTGCAGCGAGAGCACCGCAGCATGAGAGCCAACGTGGAGCTGGACCACGCCACCCTGGTGCGCTTCAGCCCTGACTGCAG  160
Pan     CTACAGCGAGAGCACCGCAGCATGAGAGCCAACGTGGAGCTGGACCACGCCACCCTGGTGCGCTTCAGCCCTGACTGCAG  160
Macaca  CTGCAGCGAGAGCACCGCAGCATGAGAGCCAACGTGGAGCTGGACCATGCCACCCTGGTGCGCTTCAGCCCTGACTGCAG  160
Bos     CTGCAGCGGGAGCACCGCAGCATGAGAGCCAACGTGGAACTGGACCATGCCATCCTGGTGCGCTTCAGCCCTGACTGCAG  160
Canis   CTGCAGCGGGAGCACCGCAGCCTGAGAGCCAACGTGGAGCTGGACCATGCTACCTTGGTGCGCTTCAGCCCTGACTGCAG  160
```

ENSG00000106638 exon 4

```
Homo    AGCCTTCATCGTCTGGCTGGCCAACGGGGACACCCTCCGTGTCTTCAAGATGACCAAGCGGGAGGATGGGGGCTACACCT  80
Pan     AGCCTTCATCGTCTGGCTGGCCAACGGGGACACCCTCCGTGTCTTCAAGATGACCAAGAGGGAGGATGGGGGCTACACCT  80
Macaca  AGCCTTCATCGTCTGGCTGGCCAACGGGGACACCCTCCGTGTCTTCAAGATGACCAAGCGGGAGGATGGGGGCTATACCT  80
Bos     AGCATTCATCGTCTGGCTCGCTAATGGAGACACCCTCCGTGTCTTCAAGATGATGAAACGAGACGATGGAGGCTATACTT  80
Canis   AGCCTTCATCGTCTGGCTGGCCAATGGAGATACCCTTCGTGTCTTTAAGATGACCAAGCGAGAAGATGGGGGCTATACCT  80

Homo    TCACAGCCACCCCAGAGGACTTCCCTAAAAAGCACAAGGCGCCTGTCATCGACATTGGCATTGCTAACACAG  152
Pan     TCACAGCCACCCCAGAGGACTTCCCTAAAAAGCACAAGGCGCCTGTCATCGACATTGGCATTGCTAACACAG  152
Macaca  TCACAGCCACCCCAGAGGACTTCCCTAAAAAGCACAAGGCGCCTGTCATCGACGTTGGCATTGCTAACACAG  152
Bos     TCACGGCCACCCCGGAGGACTTTCCTAAAAAGCACAAGGCACCCATTGTCAACATTGGCATCGCTGACACAG  152
Canis   TCACAGCCAACCCAGAGGACTTTCCTAAAAAGCACAAGGCACCCGTCATCAACATTGGCATTGCAGACACAG  152
```

ENSG00000106638 intron 3

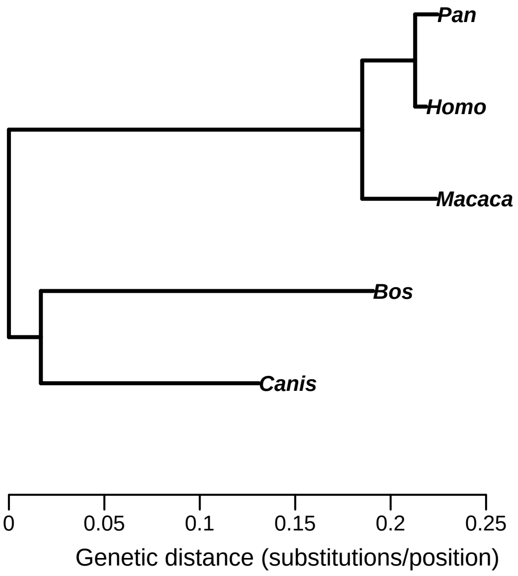

ENSG00000106638 intron 3

```
Homo    GTGGGACAAGACGGAGCCTCAGGTTTGCCTGCAGAGGCCTAGTCCCAGGCTT-TGGCCTTCCCATATCTGCTTTGAGGTAGTGAGGAGGGACCTGGACCC 99
Pan     GTGGGACAGGACGGAGCCTCAGGTTTGCCTGCAGAGGCCTAGTCCCAGGCTT-TGGCCTTCCCATATCTGCTTTGAGGTAGTGAGGAGGGACCTGGACCC 99
Macaca  GTGGGACAGGATGGAGCCTCAGGTTTGCCTGAAGAGGCCCAGTCCCAGGCAT-TGGCCTTCCCATATCTGCTTTGAGGTGGCGAGGAGGGACCCGGACCC 99
Bos     GTGGGACAGGATGGAGCCCCAGGTGTGCCTGCAGAAGCCCTGACCCAGGCCTAGGTCTTTCCCACACCTGGTTTGGGATACTGAGGGGG-----GGACCC 95
Canis   GTGGGACAGGATGGAGCCTCAGCTTTGCCTGCAGAAGCCCAGTCCCAGGCTTATGTCTTTCCCATACCTGACTTGGGATAGTGGGGGTG-----G----- 90

Homo    AGGGCCTCCCTTGGGGTGCCAGAA-AGAGCATCCACATAGTTTGCGCGCTGCCTGGGGCTCCCTGGAGATCTGGGC------------------------ 174
Pan     AGGGCCTCCCTTGGGGTGCCAGAA-AGAGCATCCACATAGTTTGTGCGCTGCCTGGGGCTCCCTGGAGGTCTGGGC------------------------ 174
Macaca  AGGGCCTCCCTTGGGGTGCCAGAA-AGAGCATCCACGTAGTTTGCACGCTGCCTGGGGCTCCCTGGAGGTCTGTGC------------------------ 174
Bos     AGGGTCTTCCTTGTGGTGGCAGAAGGGAACATCCAGACAGCTTGAACACTGTCTGAGGCTCCCTGGAGGGTTATGCAGCTACCTAACAAGGAAGTAGCAA 195
Canis   -GGGTCTCCCTGGGGATGGCAGAA-AGAGTATCTGGACAGCTTAAGCACTGCCTGGGGCTCCCTAGAGGTCTCTGCA----------------------- 165

Homo    -------------------TGCTATACAGC---------------------------------------------------------------------- 185
Pan     -------------------TACTATACAGC---------------------------------------------------------------------- 185
Macaca  -------------------TGCTATACAGC---------------------------------------------------------------------- 185
Bos     TGGCAACCCACTCCAGTACTCTTGCCTGGGAAATCCCATGGACGGAGGAGCCTGGTAGGCTACAGTCCATGGGGTCACGAAGAGTCGGACACGACTGATG 295
Canis   -------------------TTTTACACAGA---------------------------------------------------------------------- 176

Homo    --------------------------AAGGAAACAG----GGGGAGGTCTCTTCCTACCT-GGAAATACCTTGGCCTGGC----TTCCAGATGAGCCTTA 250
Pan     --------------------------AAGGAAACGG----GGGGAGGTCTCTTCCTACCC-GGAAATACCTTGGCCTGGC----TTCCAGATGAGCCTTA 250
Macaca  --------------------------AAGGAAACGG----GGGGAGGTCTTTTCCTACCT-GGAAATAACTTGGCCTGGC----TTCCAGATGAGCCTTG 250
Bos     TCACAGTTAGCAGCAGCAGCAGCTACAAGGAAGTAGAGTAGGGAAAATCTGTTCCTATTTGGGAAGTACCAAAGCCTGACTGATTTCCAGATTAGTCTTG 395
Canis   --------------------------AAGGAAGCAGAGAGGGGAAGATATGTTCCTACTTGGGAAGTACTGAAACCTGGC-----TCCAGATTCGTCCTG 245

Homo    G-------------TTTCTAAACCCTAGAGAGAAACTAAAGGAGCCAAAGCTATCAGAATCTT-ACCTTTTGAGGAGTTTTATGAGATTTGGAACCAGGG 336
Pan     G-------------TTTCTAAACCCTAGAGAGAAACTAAAGGAGCCAAAGCTATCAGAATCTT-ACCTTTTGAGGAGTTTTATGAGATTTGGAACCAGGG 336
Macaca  G-------------TTTCTAAACCCTAGAGAGAAACTAAAGAAGTCAAAGCAATGGAATCTT-ACCTTTTGAGGAGTTTTATAAGATTTGGAACCAGGG 336
Bos     GAAGCTCTCATGGTTTTCTAAACCCTAGAGAGAAGCTAAAGGAGCCAGAACTATGAGAGTCCTAACCTTCTAAGGAGTTTTAAGAGATTCTGAATTAAGG 495
Canis   GAAATTATAATGGTTTTCTAAACTCTAGAGAGACACCAAAGGAGCCAAAGCTATGCGAGTCCT-ATCTTCTGAGAAGTATTAAGAGGTTTTGAACAAAGG 344

Homo    GCTTAAGTTTTAAGGCTAGACTGTGGGGCATGAGGAGAGATCTTGACTTCCCCGAGAACCCAGCACAGGA------CCTGGAACACAGAGCCTCTTTGTC 430
Pan     GCTTAAGTTTTAAGGCTAGACTGTGGGGCATGAGGAGAGATCTTGACTTACCCGAGAACCCAGCACAGGA------CCTGGAACACAGAGCCTCTTTGTC 430
Macaca  GCTTATGTTGTAAGGCTAGACTGTGGGGCATGAGGGGAGATCTTGACTTCCCCCAGAATCCAGCACAGGA------CCAGGAACACAGAGCCTCTTTGTC 430
Bos     ------GTTATGAGGCTGGACTATAGGTCCTGAAGGGAGATCTTGATTCTCCTTAAAGCCTGCCTAGCGTAGGGCCTCACACACACAGGTCTCTGTGTCT 589
Canis   AGTTAAGTTGCAGGGCTTGAC--TGGGGCATGAAGGAAGACCATGATTTTCCTCGGAGCCTAGCATACAAA-----CCTCACACATAGGCCTCCGTGTCC 437

Homo    CAGTGAGAGCGTGACTTGTCTGCTGCCTGTCTCTAG 466
Pan     CAGTGAGAGCGTGACTTGCCTGCTGCCTGTCTCTAG 466
Macaca  TAGTGAGAGAGTGACTTGTCTGCTACCTGTCTCTAG 466
Bos     GAAGGAGTGAGAGACTTGTCTGCTCCCTGTTTCTAG 625
Canis   AAACAAATGAGTGACTTGTCTGCTACCTGCCTCTAG 473
```

ENSG00000079134 intron 10

Description: THO complex subunit 1 (THOC1)
Intron number: 10
Human chromosome: 18
Intron start (bp): 236456
Human intron length : 1393
Intron alignment length: 1828
Flanking exons length (upstream/downstream): 109/132
SNP density: 0.002871
K tree score: 0.0299
Scaling factor: 0.9617
Human-chimpanzee distance: 0.018407
Total primate branch length: 0.0841

ENSG00000079134 exon 10

```
Homo    CTCTATTCCAATTGATTACAACCTGTATCGAAAATTCTGGTCACTTCAGGATTACTTCAGGAACCCTGTGCAATGCTATG 80
Pan     CTCTATTCCAATTGATTACAACCTGTATCGAAAATTCTGGTCACTTCAGGATTACTTCAGGAACCCTGTGCAATGCTATG 80
Macaca  CTCTATTCCAATTGATTACAACCTGTATCGAAAATTCTGGTCACTTCAGGATTACTTCAGGAACCCCGTGCAATGCTATG 80
Bos     CTCCATTCCAATTGATTACAACCTGTACCGAAAATTTTGGTCACTTCAGGATTACTTTAGAAACCCTGTGCAATGCTATG 80
Canis   CTCCATTCCAATTGACTACAACCTGTATCGAAAATTCTGGTCACTTCAGGATTACTTCAGGAACCCTGTGCAATGCTATG 80

Homo    AGAAGATTTCATGGAAAACTTTTCTCAAG 109
Pan     AGAAGATTTCATGGAAAACTTTTCTCAAG 109
Macaca  AGAAGATTTCATGGAAAACTTTTCTCAAG 109
Bos     AGAAAATCTCATGGAAAACTTTTCTCAAG 109
Canis   AGAAGATTTCATGGAAAACTTTTCTTAAG 109
```

ENSG00000079134 exon 11

```
Homo    TATTCTGAAGAAGTTTTAGCTGTTTTTAAGAGTTATAAATTAGATGATACTCAGGCCTCAAGAAAAAAGATGGAAGAATT 80
Pan     TATTCTGAAGAAGTTTTAGCTGTTTTTAAGAGTTATAAATTAGATGATACTCAGGCCTCAAGAAAAAAGATGGAAGAATT 80
Macaca  TATTCTGAAGAAGTTTTAGCTGTTTTTAAGAGTTATAAATTAGATGATACTCAGGCCTCAAGAAAAAAGATGGAAGAATT 80
Bos     TATTCTGAAGAAGTCCTAGCTGTTTTTAAGAGTTATAAATTAGATGATACTCAGGCCTCAAGGAAAAAGATGGAAGAATT 80
Canis   TATTCTGAAGAAGTTCTAGCTGTTTTTAAAAGTTATAAATTAGATGATACTCAAGCCTCAAGGAAAAAAATGGAAGAATT 80

Homo    GAAAACAGGAGGAGAACATGTATATTTTGCAAAATTTTTAACAAGTGAAAAG 132
Pan     GAAAACAGGAGGAGAACATGTATATTTTGCAAAATTTTTAACAAGTGAAAAG 132
Macaca  GAAAACAGGAGGAGAGCATGTATATTTTGCAAAATTTTTAACAAGTGAAAAG 132
Bos     GAAAACAGGAGGAGAGCATGTATATTTTGCAAAATTTTTAACAAGTGAAAAG 132
Canis   AAAAACAGGAGGAGAACATGTATATTTTGCAAAATTTTTAACAAGTGAAAAG 132
```

ENSG00000079134 intron 10

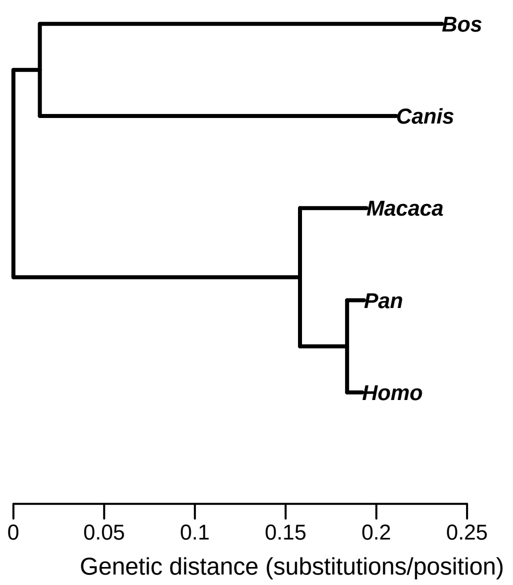

ENSG00000079134 intron 10

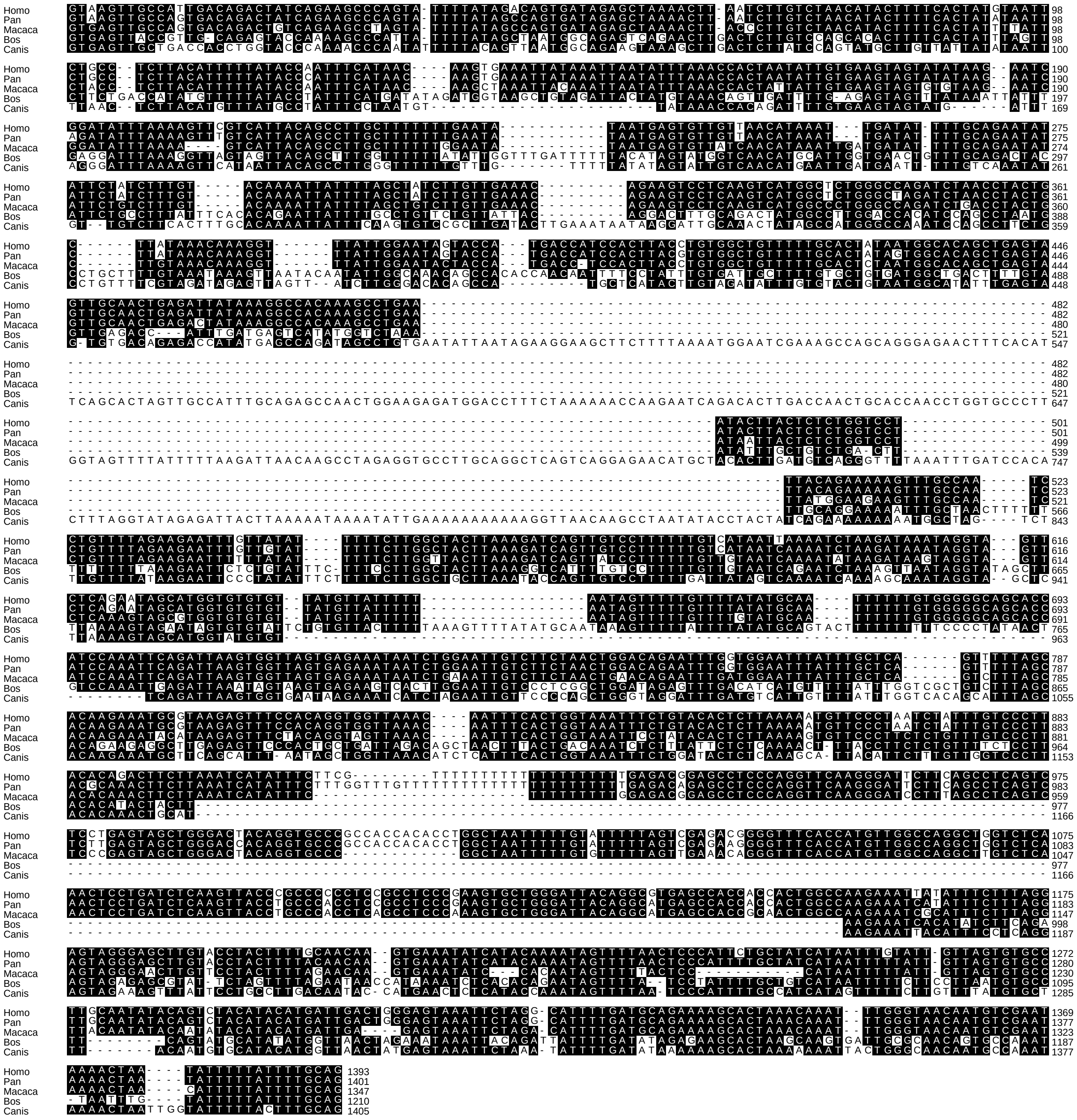

ENSG00000155926 intron 2

Description: SRC-like-adapter (SLA)
Intron number: 2
Human chromosome: 08
Intron start (bp): 134131416
Human intron length : 827
Intron alignment length: 875
Flanking exons length (upstream/downstream): 100/87
SNP density: 0.000000
K tree score: 0.0432
Scaling factor: 0.8402
Human-chimpanzee distance: 0.020224
Total primate branch length: 0.0839

ENSG00000155926 exon 2

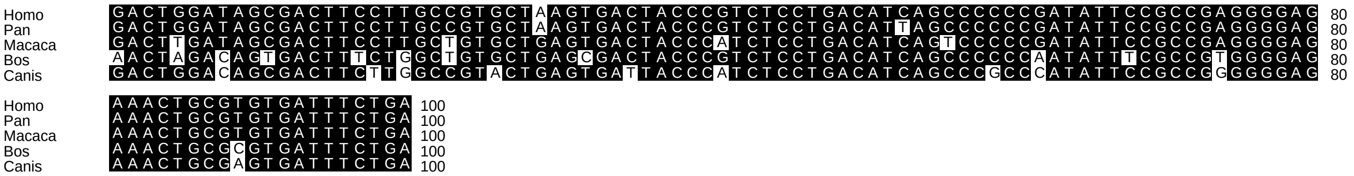

ENSG00000155926 exon 3

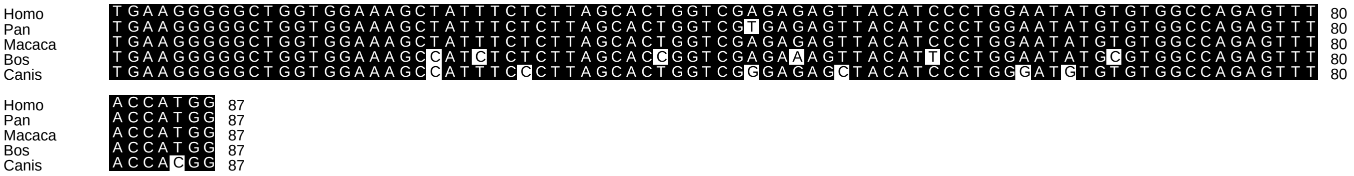

ENSG00000155926 intron 2

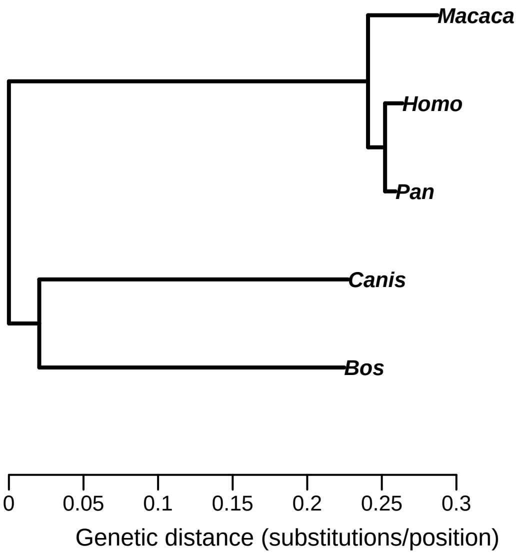

ENSG00000155926 intron 2

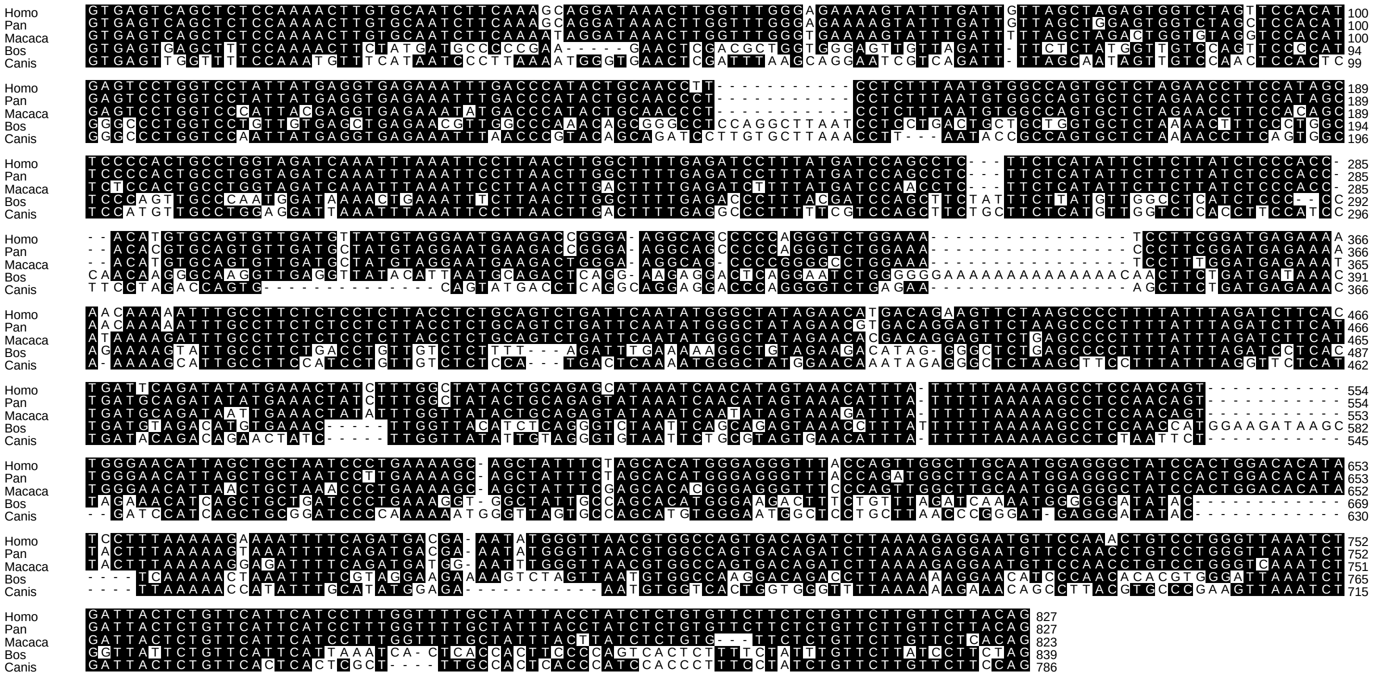

ENSG00000167139 intron 9

Description: TBC1 domain family member 21 (TBC1D21)
Intron number: 9
Human chromosome: 15
Intron start (bp): 71967131
Human intron length : 693
Intron alignment length: 722
Flanking exons length (upstream/downstream): 117/84
SNP density: 0.000000
K tree score: 0.0397
Scaling factor: 0.838
Human-chimpanzee distance: 0.016304
Total primate branch length: 0.0838

ENSG00000167139 exon 9

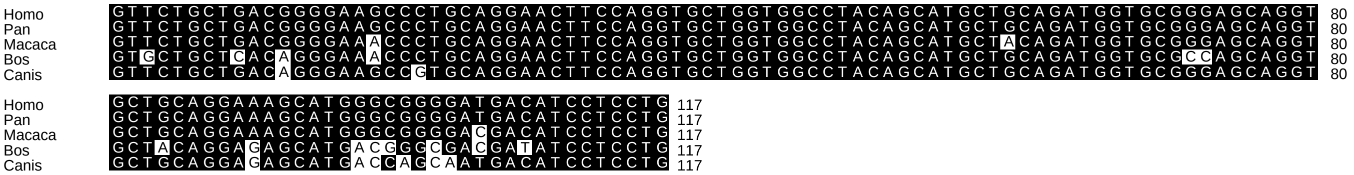

ENSG00000167139 exon 10

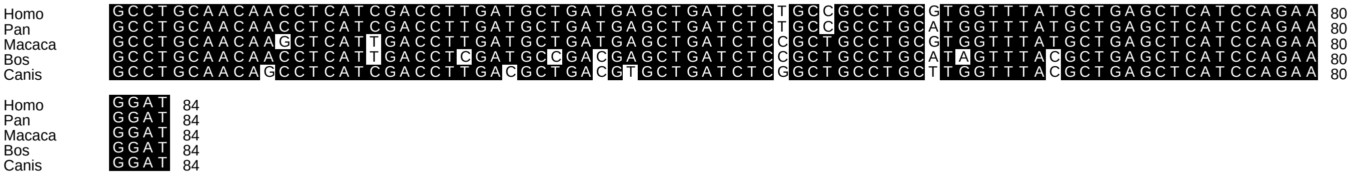

ENSG00000167139 intron 9

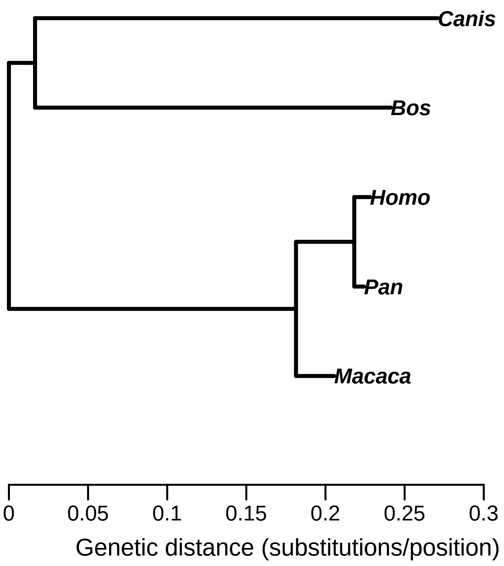

ENSG00000167139 intron 9

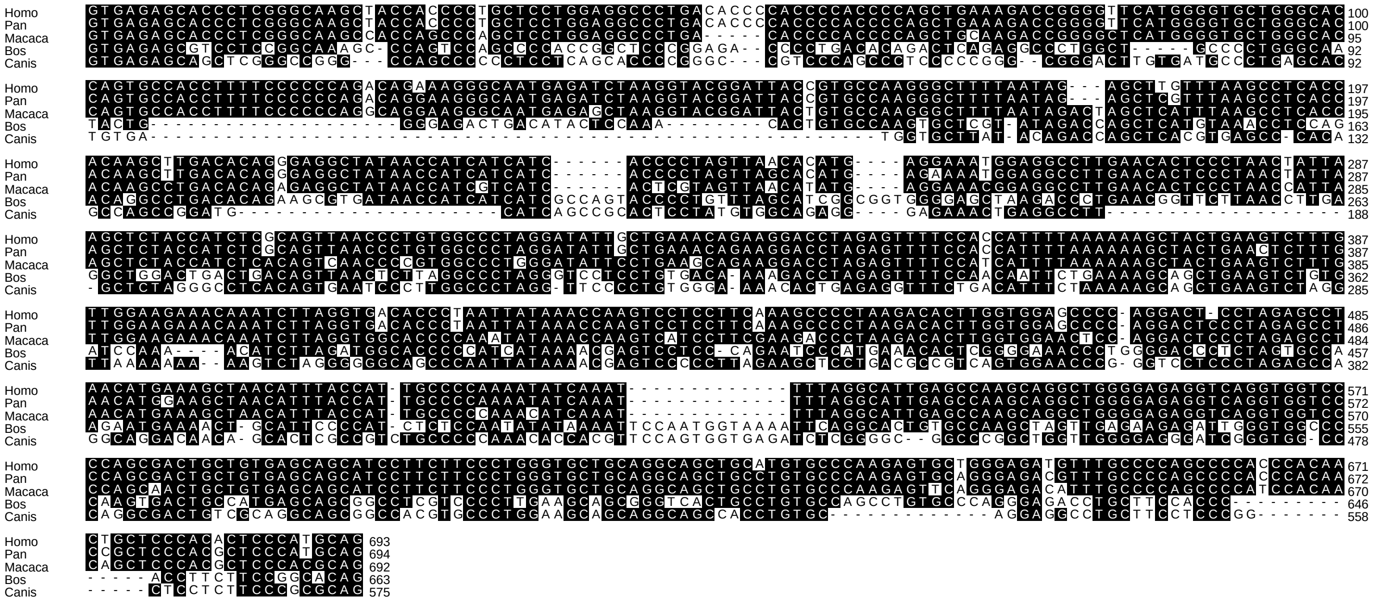

ENSG00000179761 intron 5

Description: Peroxisomal sarcosine oxidase (PIPOX)
Intron number: 5
Human chromosome: 17
Intron start (bp): 24405836
Human intron length : 371
Intron alignment length: 405
Flanking exons length (upstream/downstream): 147/159
SNP density: 0.005391
K tree score: 0.0741
Scaling factor: 0.8758
Human-chimpanzee distance: 0.011109
Total primate branch length: 0.0837

ENSG00000179761 exon 5

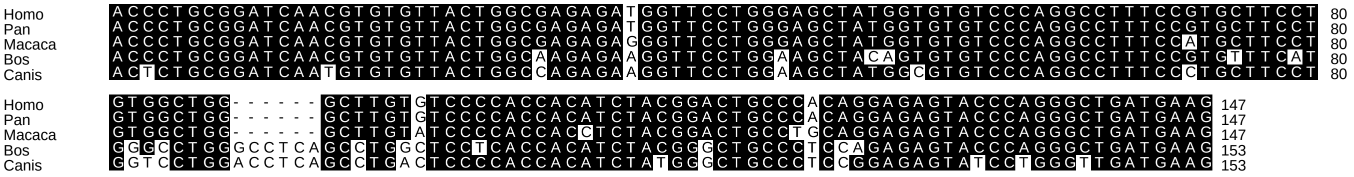

ENSG00000179761 exon 6

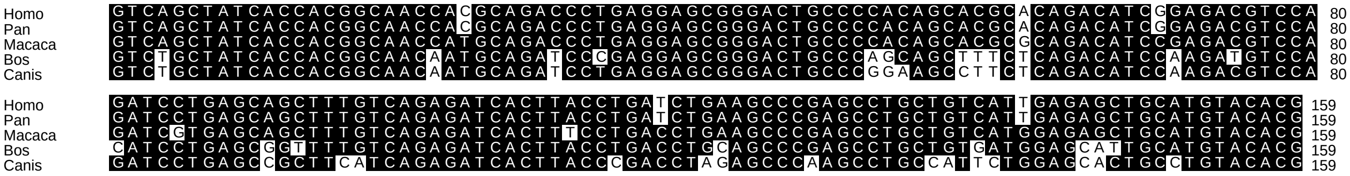

ENSG00000179761 intron 5

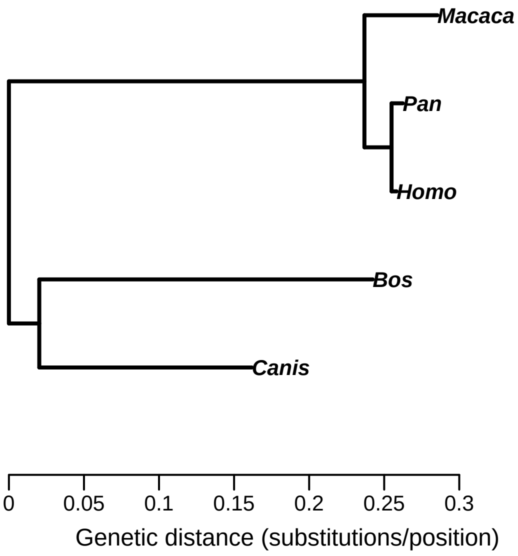

ENSG00000179761 intron 5

```
Homo    GTGAGCGGAAAGACCGAGATAAGGCAGGTAGAT------GCCAGTGCAGATTAGGGGAAGCTGCTCTAACTTTCCCCTCCATAGATGCCCTTGGGTCTCG 94
Pan     GTGAGCGGAAAGACCGAGATAAGGCAGGTAGAT------GCCAGTGCAGATTAGGGGAAGCTGCTCTAACTTTCCCCTCCATAGATGCCCTTGGGTCTCG 94
Macaca  GTGAGCAGAAAGATTGAGAAAAGGCAGGTAGAT------GCCAGTGCAGATTGGGGGAAGCAGCTCTAACTTTCCCCTCCATAGACGCCCTTGGGTCTCA 94
Bos     GTGAGGGGAGAGGCCCAGAGAAGGCTAACAGGG------TCCCATGCAGATTTGGGGCGGCTACCCTAACTTCCCTCTCAATGAGCTCCCTCAGACCCCA 94
Canis   GTGAACAGAAAGGCCCAGAGAGGGTTGGTAGGGCACCCCTCCCTTGCAGATTTGGGGCAA-TGCTCTAACTTCCCTTTCAACAGATTCCC-CAGATCCCC 98

Homo    G-GGCCAGCAGCAGGAGCCT------GCTTCACTCATGGCGATTTGCAAGGAAAGACCCTCAGCTGCCCTGAGGCC----CAGCAAAAGGAGCCCTTCCT 183
Pan     G-GGGCAGCAGCAGGAGCCT------GCTTCACTCATGGCGATTTGCAAGGAAAGACCCTCAGCTGCCCTGAGGCC----CAGCAAAAGGAGCCCTTCCT 183
Macaca  G-GGGCAGCAGAAGGAGCCT------GCTTCACTCTTGGCGATTTGCAAGGAAAGACCCTCAGCTGCCCTGAGGTC----CAGCAAAAGGAGCCCTTCCT 183
Bos     GTGAGCAGCAGCAGGAACCA------ACTTCACCATTCAGTATGTGCCGCACGAGACTCCCAGCTGTCCTGGGGCC----CAGCAAAAGGAGCCCTTCCT 184
Canis   CCAGGGAGCAGCAGGAGCAGGAGAATACTTCTCTATTCACCATTTGCAATGCATGACCCCCAGCTGTCCCAAGCCCCCCTCCCCAAAGGGAGCCCTTCCT 198

Homo    TTGCAAACATAGGGTGGTTGCAGGGACCAACAGCT-AAGCAGCCTCAGGTGACAGCCCACAGCACCTGCCAGGAGTGGGGA-AGGCTGGAATGATGGATG 281
Pan     TTGCAAACATAGGGTGGTTGCAGGGACCAATAGCT-AAGCAGCCTCAGGTGACAACCCACAGCACCTGCCAGGAGTGGGGA-AGGCTGGAATGATGGATG 281
Macaca  TTGCAAACATAGGGTGGTTGCAGGGACCAAGAGCT-AAGCAGCCTCAGGTGACAGCCCGCAGCACTTGCCAGGAGTGGGGA-AGGATGGAATGATGGATG 281
Bos     TTGCAAACAGAGGGTTAGCAGGGGAACGGAGGGCT-AGGCAACCTTGTATGGCAACAAAAAGCATCTGACAGGAGTGCGGA-AGGCTGGGGTGATGGCTG 282
Canis   TTGCAAACATGGGGCTAGTGGGGGGAGGAACAGTTAAGGCAACCTCATGTGGCAGCAAACAGCATCTGTCAGGGCCAGGGAGAGGTTGTGATGATGGATG 298

Homo    GGGCTGTCCTGTGTACACGCCTGCCC-------------TTCGGCTGTGGG-TGTGTGTGGAAGGCC-AGCCCTCACCACTCATGCCACTCTGATTGGGA 366
Pan     GGGCTGTCCTGTGTACACGCCTGCCC-------------TTCGGCTGTGGG-TGTGTGTGGAAGGCC-AGCCCGCACCACTCATGCCACTCTGATTGGGA 366
Macaca  GGGCCCTCCTGTGTACACGCCTGCCC-------------TTCGGCTGTGGC-TGTGCGCAGAAGGCC-AGCCCTCACCACTCCTGCCGCTCTGATTGGGA 366
Bos     GGGTGCCTCCGGTGTACAACCTGGCCCCCCCATCTCCTGCCGGGCTGGGGGTGGTGTGCAGGAAGCCGGGGCCTCCCAGCTCAAACCGCTTTGATTGGGA 382
Canis   GGGGCACTCTGCGTCTGAGCCTGCCCC------------CTGGGCTGGGTG-GGTGTGCAGGAGGGC-CGGCCTCGCAGCTCACACTGCTCTGATTGGGA 384

Homo    GCCAG 371
Pan     GCCAG 371
Macaca  GCCAG 371
Bos     GCCAG 387
Canis   GCTAG 389
```

ENSG00000021826 intron 12

Description: Carbamoyl-phosphate synthase (CPS1)
Intron number: 12
Human chromosome: 02
Intron start (bp): 211167576
Human intron length : 880
Intron alignment length: 1834
Flanking exons length (upstream/downstream): 99/96
SNP density: 0.000000
K tree score: 0.0369
Scaling factor: 1.1291
Human-chimpanzee distance: 0.009226
Total primate branch length: 0.0837

ENSG00000021826 exon 12

```
Homo    TACCTGTTTGATTCCTTTTTCTCACTGATAAAGAAAGGAAAAGCTACCACCATTACATCAGTCTTACCGAAGCCAGCACT 80
Pan     TACCTGTTTGATTCCTTTTTCTCACTGATAAAGAAAGGAAAAGCTACCACCATTACATCAGTCTTACCGAAGCCAGCACT 80
Macaca  TACCTGTTTGATTCCTTTTTCTCACTGATGAAGACAGGAAAAGGTACCACCATTACATCAGTCTTACCGAAGCCAGCACT 80
Bos     TATCTGTTTGATTCCTTTGTCTCACTGATAAAGAAGGAGAAAGGAACCACCATTACATCGGTACTACCAAAGCCAGGCCT 80
Canis   TACCTATTTGATTCTTTTTTCTCACTGATAAAGAAAGGTAAAGGCACCACGATTACATCGGTTCTACCAAAGCCAGCACT 80

Homo    AGTTGCATCTCGGGTTGAG 99
Pan     AGTTGCATCTCGGGTTGAG 99
Macaca  AGTTGCATCTCGAGTTGAG 99
Bos     AGTAGCATCTCGAGTTGAG 99
Canis   AGTTGCATCTCGAGTTGAG 99
```

ENSG00000021826 exon 13

```
Homo    GTTTCCAAAGTCCTTATTCTAGGATCAGGAGGTCTGTCCATTGGTCAGGCTGGAGAATTTGATTACTCAGGATCTCAAGC 80
Pan     GTTTCCAAAGTCCTTATTCTAGGATCAGGAGGTCTGTCCATTGGTCAGGCTGGAGAATTTGATTACTCAGGATCTCAAGC 80
Macaca  GTTTCCAAAGTCCTTATCCTAGGATCAGGAGGTCTGTCCATTGGTCAGGCTGGAGAATTTGATTACTCAGGATCTCAAGC 80
Bos     GTTTCCAAAGTCCTTATCCTAGGATCAGGAGGTCTGTCCATTGGTCAGGCAGGTGAATTTGATTACTCGGGATCGCAAGC 80
Canis   GTTTCCAAGGTCCTTATCCTAGGATCGGGGGGGCTGTCCATTGGTCAGGCAGGAGAATTTGATTACTCAGGATCACAAGC 80

Homo    TGTAAAAGCCATGAAG 96
Pan     TGTAAAAGCCATGAAG 96
Macaca  TGTAAAAGCCATGAAG 96
Bos     TGTAAAAGCCATGAAG 96
Canis   TGTAAAAGCCATGAAG 96
```

ENSG00000021826 intron 12

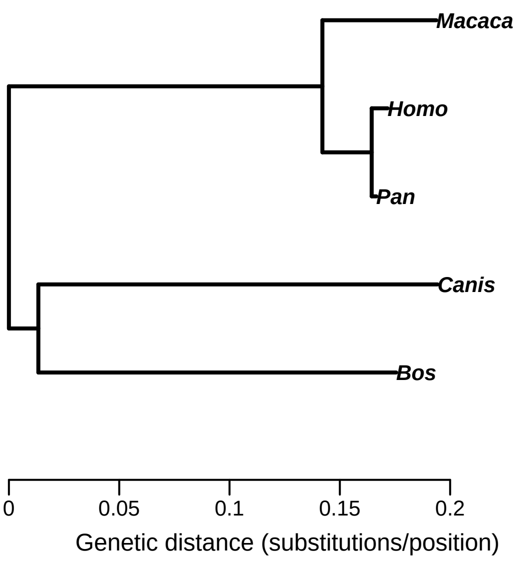

ENSG00000021826 intron 12

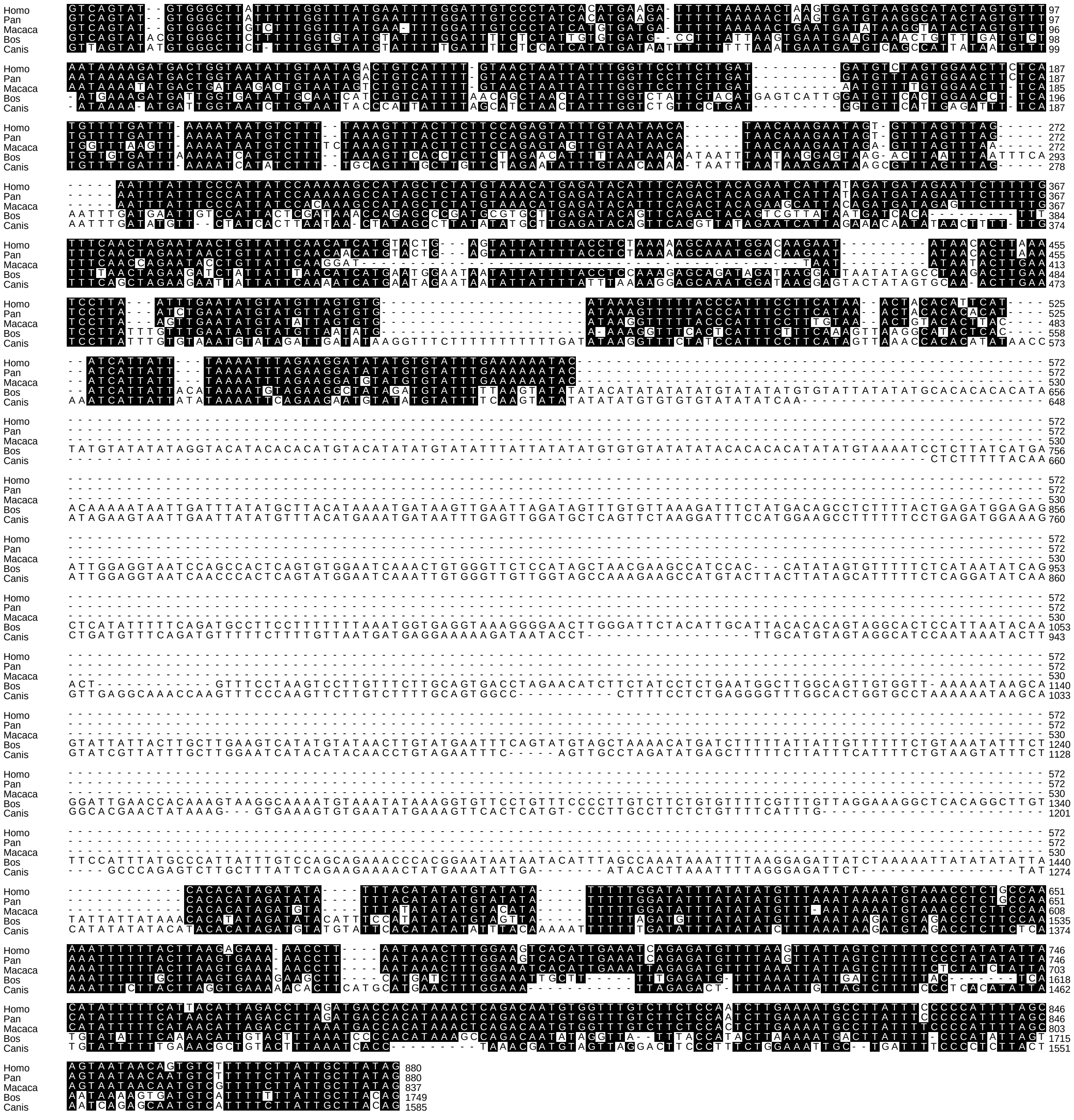

ENSG00000128604 intron 7

Description: Interferon regulatory factor 5 (IRF5)
Intron number: 7
Human chromosome: 07
Intron start (bp): 128375619
Human intron length : 244
Intron alignment length: 311
Flanking exons length (upstream/downstream): 119/246
SNP density: 0.004098
K tree score: 0.0842
Scaling factor: 0.8421
Human-chimpanzee distance: 0.018179
Total primate branch length: 0.0835

ENSG00000128604 exon 7

```
Homo    AGCTCATCCTGTTCCAAAAGGGCCAGACCAACACCCCACCACCCTTCGAGATCTTCTTCTGCTTTGGGGAAGAATGGCCT  80
Pan     AGCTCATCCTGTTCCAAAAGGGCCAGACCAACACCCCACCACCCTTCGAGATCTTCTTCTGCTTTGGGGAAGAATGGCCT  80
Macaca  AGCTCATCCTGTTCCAGAAGGGCCAGACCAACGCCCCACCACCCTTCGAGATCTTCTTCTGCTTCGGGGAAGAATGGCCT  80
Bos     AGCTCATCCTATTCCAGAAGGGCCAGACCAACACCCCACCTCCATTCGAGATCTTCTTCTGCTTTGGGGAGGAGTGGCCT  80
Canis   AGCTCATCCTGTTCCAGAAGGGCCAGACTAACACCCCACCGCCATTTGAGATCTTCTTCTGCTTTGGGGAGGAGTGGCCT  80

Homo    GACCGCAAACCCCGAGAGAAGAAGCTCATTACTGTACAG  119
Pan     GACCGCAAACCCCGAGAGAAGAAGCTCATTACTGTACAG  119
Macaca  GACCGCAAACCCCGAGAGAAGAAGCTCATTACTGTACAG  119
Bos     GACTGCAAACCCCGGGAGAAGAAGCTCATCACTGTACAG  119
Canis   GACCGCAAACCCCGAGAGAAGAAGCTCATCACTGTACAG  119
```

ENSG00000128604 exon 8

```
Homo    GTGGTGCCTGTAGCAGCTCGACTGCTGCTGGAGATGTTCTCAGGGGAGCTATCTTGGTCAGCTGATAGTATCCGGCTACA  80
Pan     GTGGTGCCTGTAGCAGCTCGACTGCTGCTGGAGATGTTCTCAGGGGAGCTATCTTGGTCAGCTGATAGTATCCGGCTACA  80
Macaca  GTAGTGCCTGTAGCAGCTCGATTGCTGCTGGAGATGTTCTCAGGGGAGCTATCTTGGTCAGCTGATAGTATCCGGCTACA  80
Bos     GTGGTACCCGTAGCAGCCCGGATGTTGCTGGAGATGTTCTCGGGGGAGCTTTCTTGGTCAGCTGATAGCATCCGGCTACA  80
Canis   GTGGTGCCTGTAGCAGCTCGGCTGTTGCTGGAGATGTTCTCAGGGGAGCTTTCTTGGTCAGCTGATAGTATCCGGCTACA  80

Homo    GATCTCAAACCCAGACCTCAAAGACCGCATGGTGGAGCAATTCAAGGAGCTCCATCACATCTGGCAGTCCCAGCAGCGGT  160
Pan     GATCTCAAACCCAGACCTCAAAGACCGCATGGTGGAGCAATTCAAGGAGCTCCATCACATCTGGCAGTCCCAGCAGCGGT  160
Macaca  GATCTCAAACCCAGACCTCAAAGACCACATGGTGGAGCAGTTCAAGGAGCTCCATCACATCTGGCAGTCCCAGCAGCGGT  160
Bos     GATCTCAAACCCAGACCTCAAAGACCGCATGGTGGAACAGTTCAAGGAGCTCCATCACATCTGGCTGTCCCAGCAGCATC  160
Canis   GATCTCAAACCCAGACCTCAAAGACCGCATGGTAGAGCAGTTCAAGGAGCTCCATCACCTCTGGCAGTCCCAGCAGCGGT  160
```

ENSG00000128604 intron 7

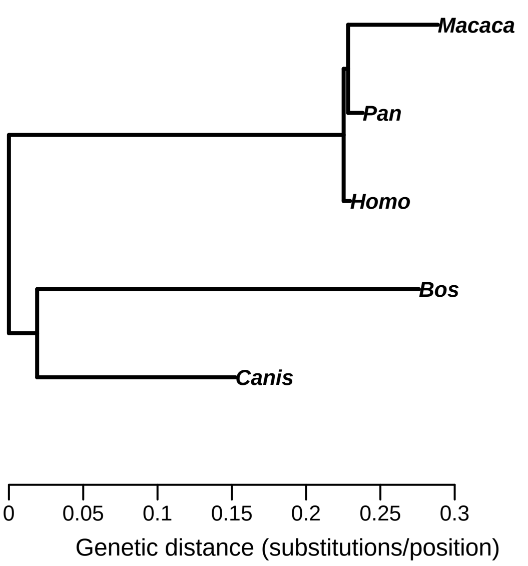

ENSG00000128604 intron 7

```
Homo    GTACATCTCC--CCTATCCCAAAGTCGGCCTTGGCTTGAAAACTGGGGAATCCTGGGGCTAGGCCCTTGCCCCAGGCTGGAGGCTCAGGGCTCCCTGAGC 98
Pan     GTACATCTCC--CCTATCCCAAAGTCGGCCTTGGCTTGAAAACTGGGGAATCCTGGGGCTAGGCCCTTGCCCCAGGCTGGAGGCTCAGGGCTCCCTGAGC 98
Macaca  GTACGTCTCC--TCTGCCCCAAAGTCGGCCTTGGCTTGAAAACTGGGGAAGCCTGGGGCCAGGCCCTTGCCCCAGGCTGGAGGCTCAGGGCTCCCTGAGC 98
Bos     GTATACTCCCATCAT-CAACCTCCTGAACTCTGCTTTGAATATTGGGAAATCCTGG------GCCCTTGCCCCAGG-AGGGG---CAGGGCTGTCTGGGT 89
Canis   GTATACCGCCATCTCACCCCCTACTCTGCTTTGGTTTGAATATTGGGGAATCCTGGGGATCAGCCGTTGCCCCAGG-TAGAGGCCCAGGGCTCTCTGAGC 99

Homo    AGTGTGAACTTGGCGGCCAGAGACCATCAAGGCTCAGAG--------------------------------------------------------CCGGA 142
Pan     AGTGTGAACTTGGCCGCCAGAGATCATCACGGCTCAGAG--------------------------------------------------------CCGGA 142
Macaca  AGCGTGAACTTGGCTGCCAGAGACCATCAAGGCTCAGGG--------------------------------------------------------CCAGA 142
Bos     AGGGTGAATTCAGTGGTCAGAGTTAATTGATGACCGGCACTGCTCACACATAAGCTGCCTTTCTACGAAGTAGAAATTGGTGCCCCTTCTT----CTGGC 185
Canis   AGTGGGAACTCCGTGGCCAGAGATAATCAAGGCCGGAGGCTGCCCACACATCAGCAGCCTTTGTATGAAGTAGAAATTGGTGCCCCTTCTTCTTCCTGGT 199

Homo    GAATGCGGTCTATTACTCACCC-CTGATGGCTGTCCTCATGCACAGCTGGATCTGGCAGCCCTGCC-------ACAGGTCTCCCTGTCTCATCTCCTCTT 234
Pan     GAATGCAGTCTATTACTCACCC-CTGATGGCTGTCCTCATGCACAGCTGGATCTGGCAGCCCTGCC-------ACAGGTCTCCCTGTCTCATCTCCTCTT 234
Macaca  GAATGCAGTCTATTACTCACCC-CTGATGGCTGCCCTCATGCACAGCTGGATCTGGCAGCCCTGTC-------ATGGGTCTCCCTGTCTCATCTCCTCTT 234
Bos     GGATGTAAACTACCACCTGCCCTCTGGTGGATCCCCTCATGCATAACTCAATCGGACAGCCCTGCCGATCCAGACAGTTCACCCCATCCTGTCTCCTCTT 285
Canis   GGATGCAGTCCACCACCTGCCCTCTGGTGAAT-GCCTCAGGCACAACTGGATTTGACTGT--------------------------TTTTGTCTCTGCCC 272

Homo    TGC-CTCCCAG 244
Pan     TGC-CTCCCAG 244
Macaca  TGC-CTCCCAG 244
Bos     CACCCGCTTAG 296
Canis   CAC------AG 277
```

ENSG00000183921 intron 3

Description: Putative short chain dehydrogenase/reductase family 42E-2 (SDR42E2)
Intron number: 3
Human chromosome: 16
Intron start (bp): 22088325
Human intron length : 1330
Intron alignment length: 1400
Flanking exons length (upstream/downstream): 58/119
SNP density: 0.001504
K tree score: 0.0985
Scaling factor: 1.0005
Human-chimpanzee distance: 0.008383
Total primate branch length: 0.0835

ENSG00000183921 exon 3

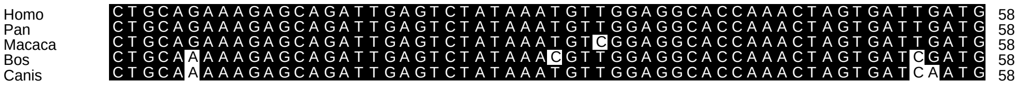

ENSG00000183921 exon 4

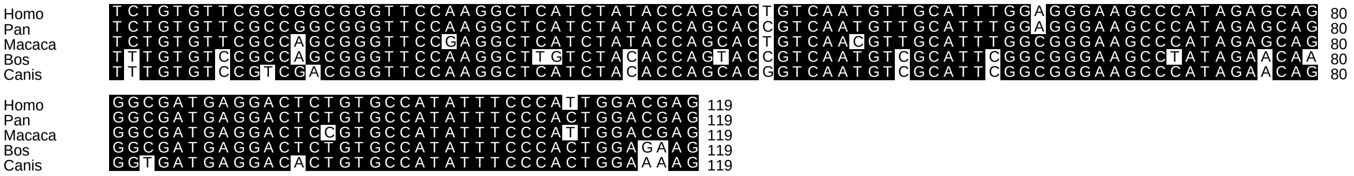

ENSG00000183921 intron 3

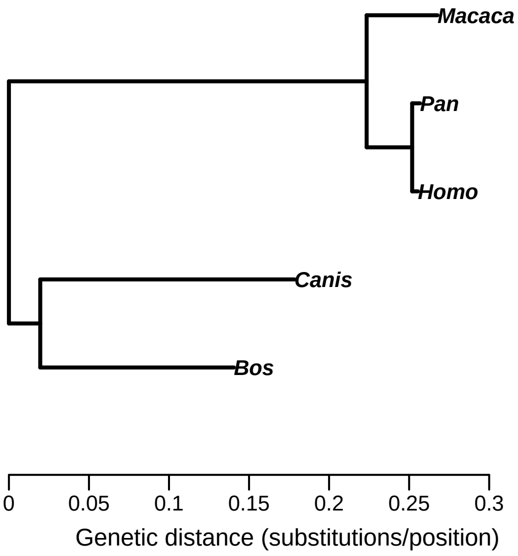

ENSG00000183921 intron 3

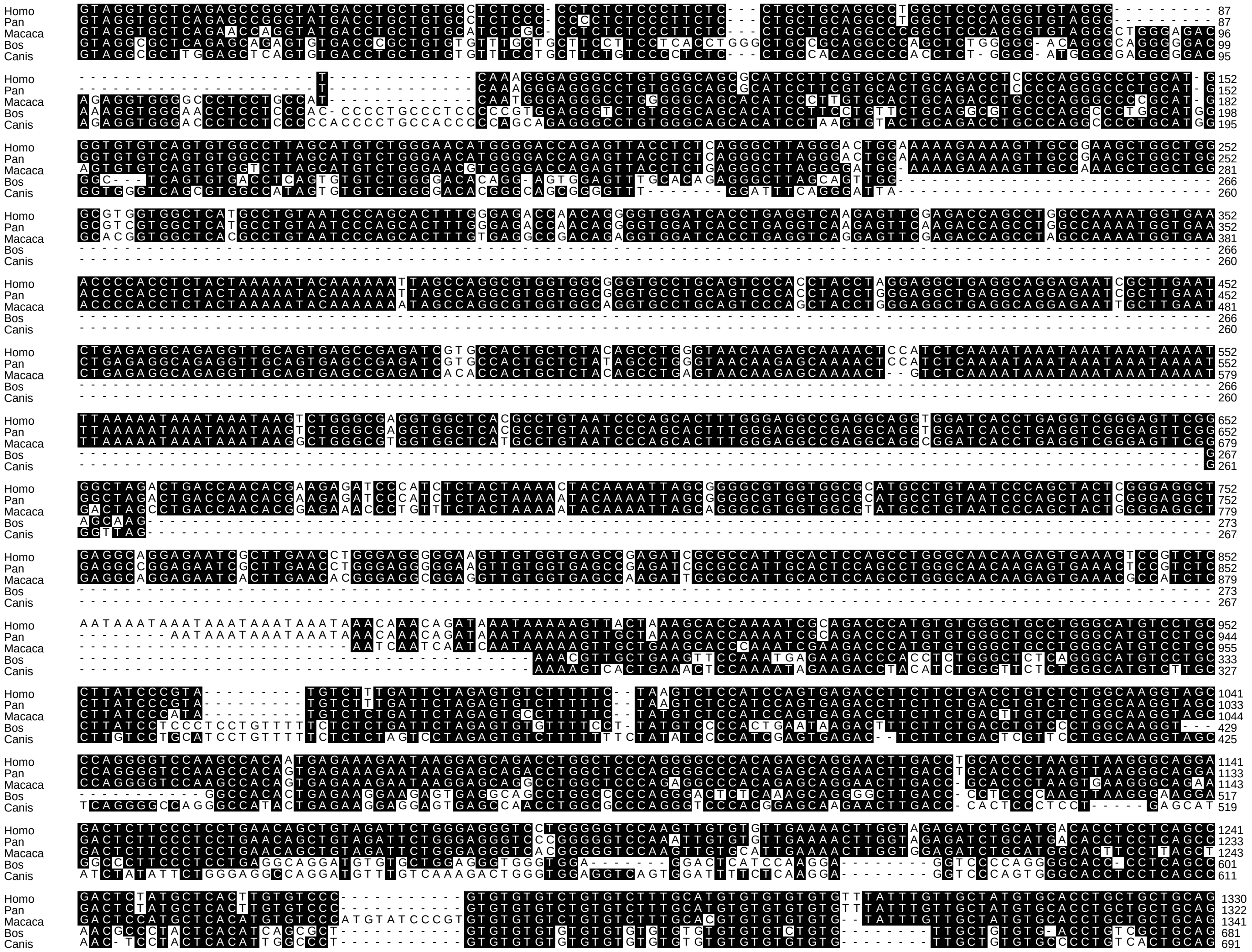

ENSG00000137857 intron 14

Description: Dual oxidase 1 precursor (DUOX1)
Intron number: 14
Human chromosome: 15
Intron start (bp): 43221603
Human intron length : 1077
Intron alignment length: 1274
Flanking exons length (upstream/downstream): 138/114
SNP density: 0.006500
K tree score: 0.0307
Scaling factor: 1.1018
Human-chimpanzee distance: 0.011358
Total primate branch length: 0.0833

ENSG00000137857 exon 14

```
Homo    GAGACCCCTGTCCGCAGCCGAGACAGCTCAGCACTGAAGGCCTGCCAGCGTGTGCTCCCTCTGTTGTTCGTGACTATTTT 80
Pan     GAGACCCCTGTCCGCAGCCGAGACAGCTCAGCACTGAAGGCCTGCCAGCATGTGCTCCCTCTGTTGTTCGTGACTATTTT 80
Macaca  GAGACCCCTGTCCGCAGCCAAGACAGCTCAGCACTGAAGGCCTGCCAGCCTGTGCTCCCTCCATTGTTCGTGACTATTTT 80
Bos     GAGACCCCTGCCCACAGCCAGGACAGCTCAGCACCAAAGGCCTGCCAGCCTGTGCTCCCCCCATCATGCGGGACTATTTT 80
Canis   GAGACCCCTGCCCACAGCCAAGACAGCTCAGCACTCAGGGCTTGCCAGCCTGTGCTCCCTCCACCATGCAGGACTATTTT 80

Homo    GAGGGCAGTGGATTTGGCTTCGGGGTCACCATCGGGACCCTCTGTTGCTTCCCTTTGG 138
Pan     GAGGGCAGTGGATTTGGCTTCGGGGTCACCATCGGGACCCTCTGCTGCTTCCCTTTGG 138
Macaca  GAGGGCAGTGGATTTGGCTTCGGGGTCACCATCGGGACCCTCTGCTGCTTCCCTTTGG 138
Bos     GAAGGCAGTGGATTTGGCTTCGGGGTCACCATCGGGACTTTGTGCTGCTTCCCCTTGG 138
Canis   GAAGGCAGTGGATTTGGCTTTGGAGTCACCATTGGGACTCTCTGCTGCTTCCCCCTCG 138
```

ENSG00000137857 exon 15

```
Homo    TGAGCCTGCTCAGTGCCTGGATTGTTGCCCGGCTCCGGATGAGAAATTTCAAGAGGCTCCAGGGCCAGGACCGCCAGAGC 80
Pan     TGAGCCTGCTCAGCGCCTGGATTGTTGCCCGGCTCCGGATGAGAAATTTCAAGAGGCTCCAGGGCCAGGACCGCCAGAGC 80
Macaca  TGAGCCTGCTCAGTGCCTGGATTGTTGCCCGGCTCCGGATGAGAAATTTCAAGAGGCTCCAGGGCCAGGACCGCCAGAGC 80
Bos     TGAGCCTGCTCAGTGCCTGGATCGTTGCCCAGCTCCGGAGGAGAAATTTAAAGAGGCTCCAGGGCCAAGACCGCCAGAGC 80
Canis   TGAGCCTGCTTAGTGCCTGGATTGTTGCCCGGCTCCGAAAGAAAAATTTCAAGAAGCTCCAGGGCCAGGACCGCAAAAGC 80

Homo    ATCGTGTCTGAGAAGCTCGTGGGAGGCATGGAAG 114
Pan     ATCGTGTCTGAGAAGTTCGTGGGAGGCATGGAAG 114
Macaca  ATCGTGTCTGAGAAACTCGTGGGAGGCATGGAAG 114
Bos     ATCATGTGTGAGAAGCTCATGGGGGGCATAAAAG 114
Canis   GTCATGTCTGAGAAGCTCGTTGGGGGCATGGAAG 114
```

ENSG00000137857 intron 14

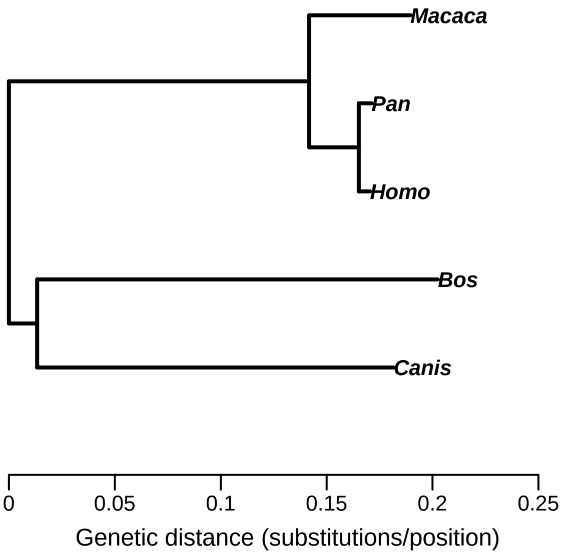

ENSG00000137857 intron 14

```
Homo    GTAAAA-TCATGGACAGAGTGGGGTGGGGTGAGAGATGCAAGCTAGGGGATGCAGTTTGGGTGGTTCCACTAATGACAACAAACCACGCAAACCACACAG 99
Pan     GTAAAA-TGATGGACAGAGTGGGGTGGGGTGAGAGATGCAAGCTAGGGGATGCAGTTTGGGTGGTTCCACTAATGACAACAAACCACGCAAACCACACAG 99
Macaca  GTAAAA-TGATGGACAGAGTGGAGTGGGGTGAGGGATGCAAGCTAGGGGATGCAGTTTGGGTGGTTCCACTAATGACAACAAACCACG---------CAG 90
Bos     GTAAAAGTGATGGGCAGGGTGGGGCTGGGTGAGAGGTATCAGCTGGGGG------TGGGGTCAGCTGCATCTATGACAACCAACCAAT---------ACAG 86
Canis   GTAAAAGTGATGGGCAGGGTGGGGTTGGGTGGAATGTGTACTCTGAGGGATGGCAGTGGGGTGGTTTCATTAAT----ACAGACTATG---------CAG 87

Homo    AGCATGGTCCCTTTGGGTAGGAGAATGAAACATTAGAGGAGGAAGGGACAAGAGAAGTGTTTGACCTGCAGAGAAGTCAGCTCCAGGGAGAATTGCCTCC 199
Pan     AGCATGGTCCCTTTGGGTAGGAGAATGAAACATTAGAGGAGGAAGGGACAAGAGAAGTGTCTGACCTGCAGAGAAGTCAACTCCAGGGAGAATTGCCTCC 199
Macaca  AGCATGGTCCCTCTGGGTAGGAGAATGAAACACTAGAGGAGGAAGGGACAAGAGAAGTG--TGACCTACAGAGAAGTCAGCTCCAGGGAGGATTGCCTCC 188
Bos     AGTACTGTTCCTCCGAGGAGATGA---AAACATCAGAGCAGG-TGGGATGTGAGAAGTATTAAAGCTACACATNNNNNNNNNNNNNNNNNNNNNNNNNNN 182
Canis   GAT----TCTATTTGGGCACATGAAGAAAACCTCAGAGCAGGAGGGACATGAGAAGTGTCTGACCTGTAGCTAAGTTAACTCCAGGGAAAATTGCCTTC 183

Homo    CTGTTTTCCTGGGACTGCCCCCAGGGCCCAGCATTGGTCAGGAGCCAGGGGGAATCTATAATCCACAGCTTTTTGCAG--GCCTGAGCCTAGAGGACTGT 297
Pan     CTGTTTTCCTGGGACTCCCCCCAGGGCCCAGCATTGGTCAGGAGCCAGGGAGAATCTATAATCCACAGCTTTTTGCAG--GCCTGAGCCTAGAGGACTGT 297
Macaca  CTGTTTTCCTGGGACTCCCCGCAGGGCCCAGCATTGGTCAGGAGCCAGGGGGAATCTGTAATCCACAGCTTTTTGCAG--GCCTGGGCCTAGAGGACTGT 286
Bos     NNNNNNNNNNNNNNNNNN------------------------------------------------------------NNNNNGGCATGGAAAACTGT 220
Canis   CTGTTCTCCTGGGG-TCCCTCTAAGGCCCAG-ATTGGTCAGGAGCCACAGGGTATCTGTATTTCACAGTTATCTGCAGAAACCTGGGCCTGGGAAACTGT 281

Homo    CAGAGGCAAATCCCTGTTTAAGAACAAGGGCTAAGACGGGGCGCGGTGGCTCATGCCTGTAATCCCAGCACTTTGAGAGGCCGAGGCAGGCGGATCACCT 397
Pan     CAGAGGCAAATCCCTGTTTAAGAACAAGGGCTAAGACGGGGCGCGGTGGCTCATGCCTGTAATCCCAGCACTTTGAGAGGCCGAGGCAGGCGGATCACCT 397
Macaca  CAGAGGCAAATCCCTGTTTAAGAACAAGGGCTAAGGCCCGGCGCGGTGGCTCACGCCTGTAATCCCAGCACTTTCAGAGGCCAAGGCAGGCGGATCGCCT 386
Bos     CAGAAGCAAATGCCTGCTTAAGAAGTAGGG---------------------------------------------------------------------- 250
Canis   TAGAGGCAAA-CCCTGCTTGAGAACCAG------------------------------------------------------------------------ 308

Homo    GAGGTCAGGAGTTTGAGACCAGCC-TGATCAACATGGAGAAACCATGTCTCTCCTAAAAATACAAAATTAGCCGGGCATGGTGGCGCATGCCTGTAATCC 496
Pan     GAGGTCAGGAGTTTGAGACCAGCC-TGATCAACATGGAGAAACCATGTCTCTACTAAAAATACAAAATTAGCCGGGCATGGTGGCGCATGCCTGTAATCC 496
Macaca  GAGGTCGGGAGTTCGAGACCAGCCATGATCAACATGGAGAAACCCTGTCTCTACTAAAAATACAAAATTAGCTGGGCATGGTGGCGCATGCCTGTAATCC 486
Bos     ------------CTGGGA---------------------------------------------------------------------------------- 256
Canis   ------------TTGAGA---------------------------------------------------------------------------------- 314

Homo    CAGCTACTCGGGAGGCTGAGGCAGGAGAATCAATTGAATCTGGGAGGCAGAGGTTGCAGTGAGCTTAGATC----------------------------- 567
Pan     CAGCTACTCGGGAGGCTGAGGCAGGAGAATCAATTGAATCTGGGAGGCAGAGGTTGCGGTGAGCTTAGATC----------------------------- 567
Macaca  CAGCTACTCGGGAGGCTGAGGTAGGAGAATCAAGTGAATCCGGGAGGCAGAGGTTGCAGTGAGCTGAGATCACACCATTGCAGTTGCAATGAGCTGAGAT 586
Bos     ---------------------------------------------------------------------------------------------------- 256
Canis   ---------------------------------------------------------------------------------------------------- 314

Homo    -GCGCCATTGCACTCCAGCCTGGGCAACAAGAGCGAAACTCTGTCAAAAAAAGAAA-------------------------------------------- 622
Pan     -GCGCCATTGCACTCCAGCCTGGGCAACAAGAGCAAAACTCTGTCAAAAAAAGAAA-------------------------------------------- 622
Macaca  TGCACCATTGCAGTCCAGCCTGGGCAACAAGAGGGAAACTCTGTGAAGAAAAGAAAAGAACAGAAAAAAAAGAAAAGAAAAGGAGGAAAGGAGGAAAGGA 686
Bos     ---------------------------------------------------------------------------------------------------- 256
Canis   ---------------------------------------------------------------------------------------------------- 314

Homo    -----------------------GAAAGAGAGAGAGAGAGAATGGAAGGAAGGA---------------------------------------------- 653
Pan     -----------------------GAAAGAGAGAGAGAGAGAATGGAAGGAAGGA---------------------------------------------- 653
Macaca  GGGAGGGAAAGAAGGAAGGAAGGGAAAGAAAGAGAGAGAGGAAGGGAGGGAGGAACGGAGGGAGGGAGGGAAGTAGGGAGGGAAGGAGGGAGGAAAGGAG 786
Bos     ---------------------------------------------------------------------------------------------------- 256
Canis   ---------------------------------------------------------------------------------------------------- 314

Homo    ------------------------------AAGAAGGAAGGAAGGAAGGAAGGGAGGGAGGAAGGGAGGGAGGAAGAGCAGGCTGGGGCAGACATAGTCG 723
Pan     ------------------------------AAGAAGGAAGGAAGGAAGGAAGGG-------AGGAAGGGAGGAAGAGCAGGCTGGGGCAGACATAGTCG 715
Macaca  GGAGGAAAGGAAGAAGGAAGGGAGGGAGGGAGGGAGGAAGGAAGGTAGGAAGGAAGGGAGGGAGGAAGGGAGGAAGGGAAGGCTGGGGCAGATCTAGTCA 886
Bos     ---------------------------------------------------------------------------------------------TGTAGCTG 264
Canis   -----------------------------------------------------------------------------------------------ACTAG 319

Homo    CTGGAGTGAGGGCTTGGGGCTTAGTCTTTGGCAAGGCTGTGTGTGGGTCAGCTAGC-AGCCTGGTGGGGTGAGTCTAGTCTCTGACACAAGGACTCTGGA 822
Pan     CTGGAGTGAGGGCTTGGGGCTTAGTCTTTGGCAAGGCTGTGTGTGGGTCAGCTAGC-AGCCTGGTGGGGTGAGTCTAGTCTCTGACACAAGGACTCTGGA 814
Macaca  CTGGAGTGA-GGCTTGGGGCTTAGTCTTTGGCAAGGCTGTGTGTGGGTCAACTAGC-AGCCTGGTGGGGTGAGTCTAGGCTCTGACACAAAGACTCTGGA 984
Bos     CTGGGGCAACGGCTTGGGACTTAGTCTTTGACTAA---GTGTGTGGATTGATCAGC-AGCCTGGTAGAGTGGGTCCAGTCTCTGGCACGAGGAC------ 354
Canis   CTGGGGCAAAGGTTTGAGACTTAGACTTTGTCATGGCTGTGTGTGAGTCCACCAGCAAGCATGGCAGAGTGGGTCCAGTAAC-AGCACAAGGACTCTGGC 418

Homo    CTTAGTCTCTGGCCAGGAAGGGACGTGAGTGGTGGAATCTGAGGAGGAAG------------GGTGGGTATCTTAGATGCTACCCAAAGCTCCCCATGGG 910
Pan     CTTAGTCTCTGGCCAGGAAGGGACGTGAGTGGTGGAATCTGAGGAAGAAG------------GGTGGGTATCTTAGATGCTACCCAAAGCTCCCCATGGG 902
Macaca  CTTAGTCTCTGGCTAGTAAGGGACATGAGTGGTAGAATATGAGGAGGCAA------------CGTGGGCATCTTAGATGCTACCCAAAGCTCCCCATGGG 1072
Bos     --CAATTTCTAGCTGGGAAGGGACCCGAGTGGTAGAAGCAGGCAAGCGTA------------GGCACGCACCTTAGATGCTCCACAAAGCTTCCTGTGGG 440
Canis   CTTAGTCTCTGGCTGGGAAGGGACCTGAGTGGTGGAAGCAGGGGAGGCTGTGCTGACAGTGCTGCAGACATCTTAGATGATACCCAAAGGCTCCCTCCTC 518

Homo    ATGCAGAGCAGCTTCC-CCCAGGGACCTTCCCAATCTGAAACAATTGAGCAAGCTGACCTAGGAGGTGGGGACAATAGGTGGTATTGCCAGGTAAGGAG- 1008
Pan     ATGCAGAGCAGCTTCC-CCCAGGGACCTTCCCAATTTGAAACAATTGAGCAAGCTGACCTAGGAGGTGGGGACAATAGGTGGTATTGCCAGGTAAGGAG- 1000
Macaca  ATACAGAGCAGCTTCC-CCCAGGGACCTTCACAATTTGAAACAATTGAGCAAGCTGACCTAGGGGGTAGGGACAATAGGTGGTACTGCCAGGAAAGGAG- 1170
Bos     ATGCAGAGCACCTCCCTCCCAGGGCACTTCATAATTTGAGACAACTGAATGGG-TAACCTAGGAGGTGAGGACAGCAGGGGTTCTTTCCAAGGAAAGAAA 539
Canis   CACC-------CCCCT-ACCAGGGAGCTTCACAATCTGAGACAATTGAATGAGCCCACCTAGGAAGTGGGGAAGGCAGGGGGTCTTGCCAAGGAAGGAG- 609

Homo    -CTGAGAAAGGAGCTGCTTCCATCCCCTAGACCCCCACGTCTCCCTGAACCT----CTGCCTCTGCCCTCCCAG 1077
Pan     -CTGAGAAAGGAGCTGCTTCCATCCCCTAGACCCCCACGTCTCCCTGAACCC----CTGCCTCTGCCCTCCCAG 1069
Macaca  -CTGAGAAAGGAGCCGCTTCCAACCCCCCGAGCCACACCTCTCCCTGAGCGTCCTACTGCCTCTGCCCTCCCAG 1243
Bos     CCTGTGAAGGAAGCCACTGATCTCCCC-AGACCTCTACCTTCCCCTGAGCATCCTACTGCCTCTG-CTTCTCAG 611
Canis   ------------GCCACTGATCTCCCCAGACTCCCACCCTTCCTCTGAGCATCCTACTGCCTCTGCCCTCTCAG 671
```

ENSG00000198171 intron 1

Description: DDRGK domain-containing protein 1 Precursor (DDRGK1)
Intron number: 1
Human chromosome: 20
Intron start (bp): 3132063
Human intron length : 1120
Intron alignment length: 1291
Flanking exons length (upstream/downstream): 91/204
SNP density: 0.005357
K tree score: 0.0319
Scaling factor: 0.7033
Human-chimpanzee distance: 0.015559
Total primate branch length: 0.0833

ENSG00000198171 exon 1

```
Homo    ATGGTGGCGCCTGTGTGGTACTTGGTAGCGGCGGCTCTGCTAGTCGGCTTTATCCTCTTCCTGACTCGCAGCCGGGGCCG 80
Pan     ATGGTGGCGCCCGTGTGGTACTTGGTAGCGGCGGCTCTGCTAGTCGGCTTTATCCTCTTCCTGACTCGCAGCCGGGGCCG 80
Macaca  ATGGTGGGGCCCGTGTGGTACTTGGTAGCGGCGGCTCTGCTAGTCGGCTTTATCCTCTTCCTGACTCGCAGCCGGGGCCG 80
Bos     ATGGTGTCCCCCGTGGTGTACTTGGTGGTGGCGGCTCTGCTTGTCGGGCTTATTCTGTTCCTGACTCGCGGCCGGGGCCG 80
Canis   ATGGTGACCCCCGTGGTATACCTGGTGGCGGCGGCTTTGCTTGTCGGCCTTATCCTTTTTCTGACTCGCAGCCGGGGCCG 80

Homo    GGCGGCATCAG 91
Pan     GGCGGCATCAG 91
Macaca  GGCGGCATCAG 91
Bos     GGCGGCAGCAG 91
Canis   GACAGCAGCAG 91
```

ENSG00000198171 exon 2

```
Homo    CCGGCCAAGAGCCACTGCACAATGAGGAGCTG---GCAGGAGCAGGCCGGGTGGCCCAGCCTGGGCCCCTGGAGCCTGAG 77
Pan     CCGGCCAAGAGCCACTGCACAATGAGGAGCTG---GCAGGAGCAGGCCGGGTGGCCCAGCCTGGGCCCCTGGAGCCTGAG 77
Macaca  CCAGCCAAGAGTCACTGCACAATGAGGAGCTG---GCAGGAGCAGGCCGGGTGGCCCAGCCTAGGCCCCTGGAGCCTGAG 77
Bos     CTGCCCAAGAGCCTTTGCACAATGAAGAGGTACCCGCAGCAGCAGGCCGAGTGGCTCGGCCTCAGCCCTTAGAGCCCGAG 80
Canis   CTGGCCAAGACCCATTGCACAATGAGGAGCAGCCCATAGTAGCAGGCTCAGTGGCCCAGCCTCGGCCCCTGGAGCCTGAG 80

Homo    GAGCCGAGAGCTGGAGGCAGGCCTCGGCGCCGGAGGGACCTGGGCAGCCGCCTACAGGCCCAGCGTCGAGCCCAGCGGGT 157
Pan     GAGCCGAGAGCTGGAGGCAGGCCTCGGCGCCGGAGGGACCTGGGCAGCCGCCTACAGGCCCAGCGTCGAGCCCAGCGGGT 157
Macaca  GAGCCGAGAGCTGAAGGCAGGCCTCGGCGCCGGAGGGACCTGGGCAGCCGCCTACAGGCCCAGCGTCGAGCCCAGCGGGT 157
Bos     GAGCAGAGAGCTGCAGGCAGGCCCCGGCGCCGGAGAGACCTGGGCAGCCGCTTGCAGGCCCAGCGTCGAGCCCAGAGAGT 160
Canis   GAGCAGAGAGCCGGGGGCAGGCCCCGGCGCCGAAGGGACCTGGGCAGCCGCCTGCAGGCCCAGCGTCGAGCCCAGCGTGT 160
```

ENSG00000198171 intron 1

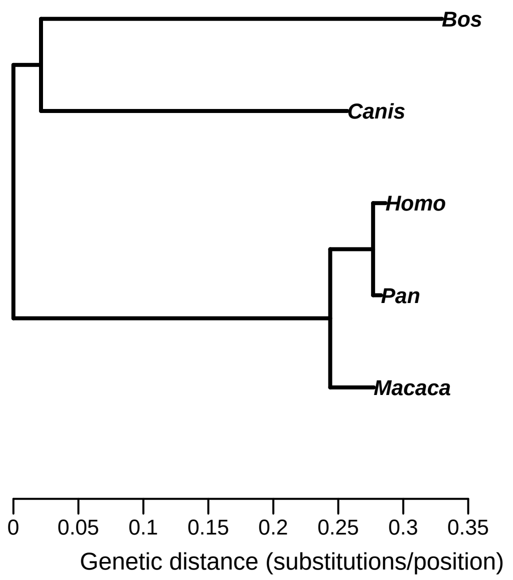

ENSG00000198171 intron 1

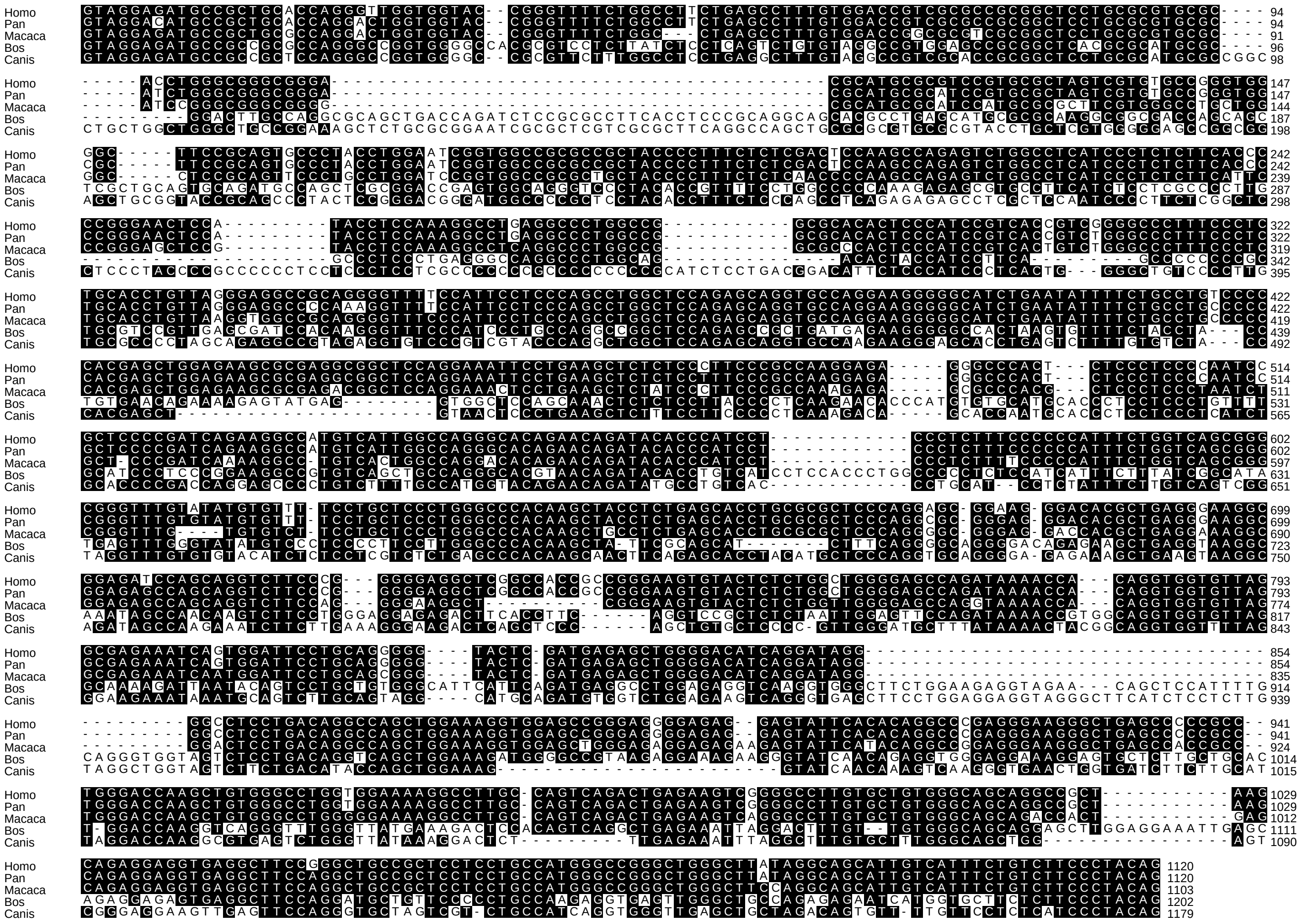

ENSG00000121486 intron 2

Description: N2,N2-dimethylguanosine tRNA methyltransferase-like (C1orf25)
Intron number: 2
Human chromosome: 01
Intron start (bp): 183386248
Human intron length : 1332
Intron alignment length: 1741
Flanking exons length (upstream/downstream): 111/114
SNP density: 0.006006
K tree score: 0.0357
Scaling factor: 1.0067
Human-chimpanzee distance: 0.012267
Total primate branch length: 0.0831

ENSG00000121486 exon 2

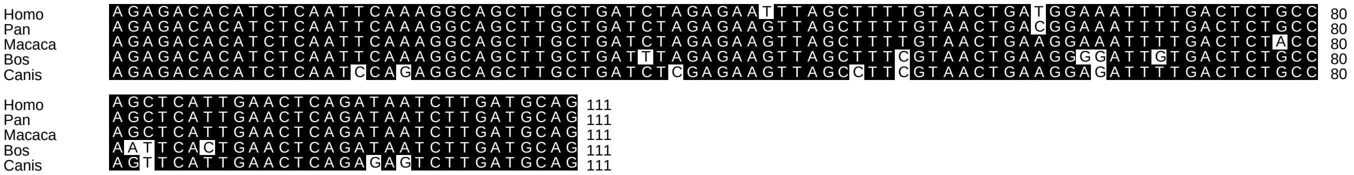

ENSG00000121486 exon 3

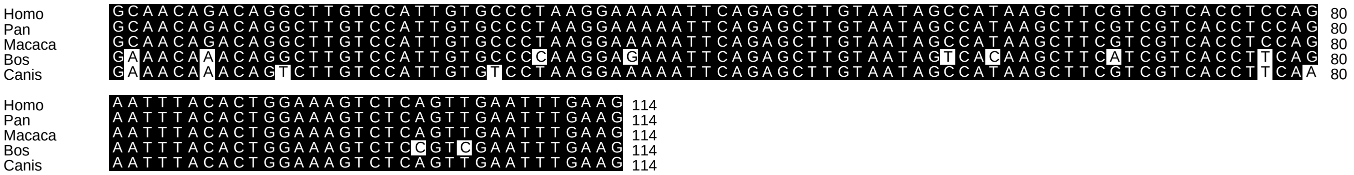

ENSG00000121486 intron 2

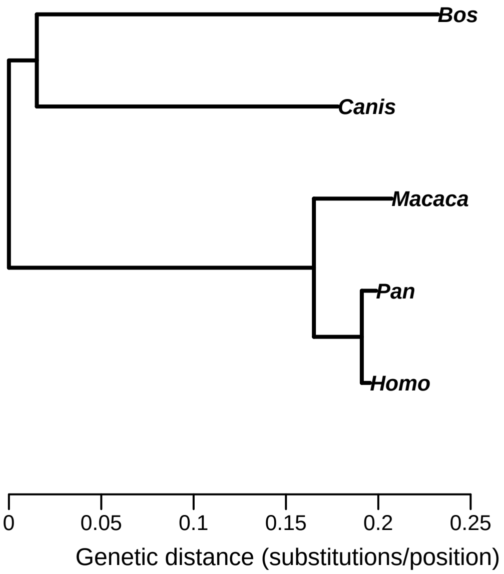

ENSG00000121486 intron 2

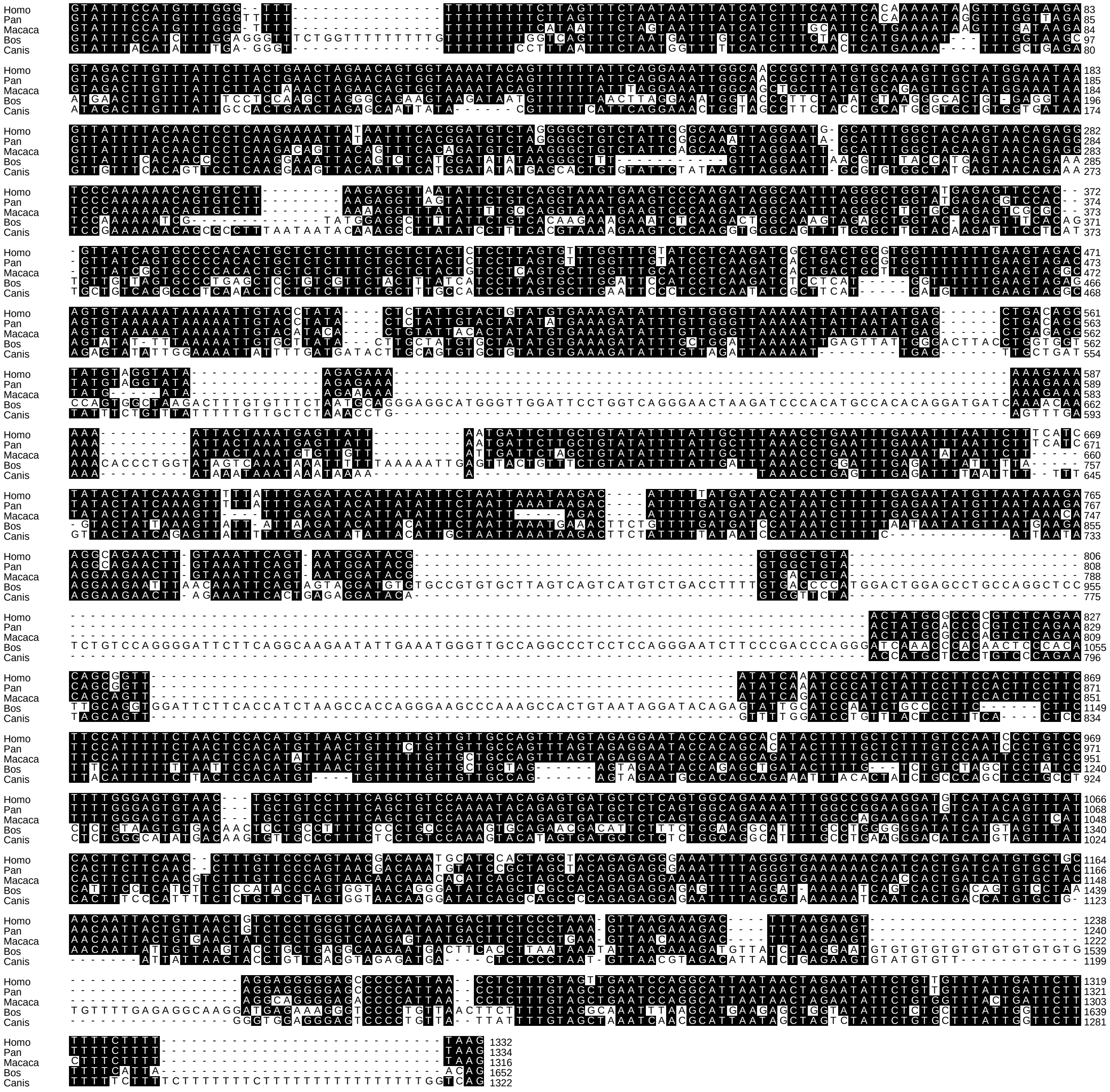

ENSG00000149292 intron 18

Description: Tetratricopeptide repeat protein 12 (TTC12)
Intron number: 18
Human chromosome: 11
Intron start (bp): 112738435
Human intron length : 1325
Intron alignment length: 1717
Flanking exons length (upstream/downstream): 102/100
SNP density: 0.003019
K tree score: 0.0955
Scaling factor: 0.7888
Human-chimpanzee distance: 0.017343
Total primate branch length: 0.0829

ENSG00000149292 exon 18

```
Homo    AGAGCTGCTGGTGTTCTGAGCCGGACCCTTTCTTCCTCTCTGAAAATTGTTGAGGAGGCCTTGCGAGCAGGAGTGGTAAA 80
Pan     AGAGCTGCTGGTGTTCTGAGCCGGACCCTTTCTTCCTCTCTGAAAATTGTTGAGGAGGCCCTGCGAGCAGGAGTGGTAAA 80
Macaca  AGAGCTGCTGGTGTTCTGAGCCGGACCCTTTCTTCCTCTCTGAAAATTGTTGAGGAGGCCTTGCAAGCAGGAGTGGTAAA 80
Bos     AGAGCTGCTGGCGTCCTCAGCCGTACCCTTTCTTCCTCCCTGAAAATTGTTGAGGAGGCCCTCAGAGCAGGAGTGGTGAA 80
Canis   AGAGCTGCTGGCGTCCTCAGCCGCACCCTTTCTTCCTCCCGGAAGATGGTTGAGGAGGCCCTGAGAGCTGGAGTGGTGAA 80

Homo    GAAAATGATGAAATTCCTGAAG 102
Pan     GAAAATGATGAAATTCCTGAAG 102
Macaca  GAAAATGATGAAATTCCTGAAG 102
Bos     GAAGATGATTAAATTCTTGAAG 102
Canis   GAAGATGATTAAGTTCCTGAAG 102
```

ENSG00000149292 exon 19

```
Homo    ACAGGAGGTGAGACTGCATCACGTTATGCTATAAAGATACTAGCTATCTGCACGAATAGTTATCATGAAGCTCGGGAAGA 80
Pan     ACAGGAGGTGAGACTGCATCACGTTATGCTATAAAGATACTAGCTATCTGCACGAATAGTTATCATGAAGCTCGGGAAGA 80
Macaca  ACAGGAGGCGAGACTGCATCACGTTATGCTGTAAAGATACTAGCTATCTGCACGAATAGTTACCACCGAAGCTCGGGAAGA 80
Bos     GCAGGAGGCCAGACTGCGTCACGTTATGCAGTCAAGATACTTGCCATCTGCACAAACAGTTATCAGGAAGCGCGAGAAGA 80
Canis   ACGGGGGGCCAGACTGCATCACGATATGCCATAAAAACACTAGCTGTCTGCACGAATAGCCATTATGAAGCACGAGAAGA 80

Homo    AGTAATAAGACTGGATAAAA 100
Pan     AGTAATAAGACTGGATAAAA 100
Macaca  AGTAATAAGACTGGATAAAA 100
Bos     AGTGTTAAGACTGGATAAAA 100
Canis   AGTGATAAGACTGGATAAAA 100
```

ENSG00000149292 intron 18

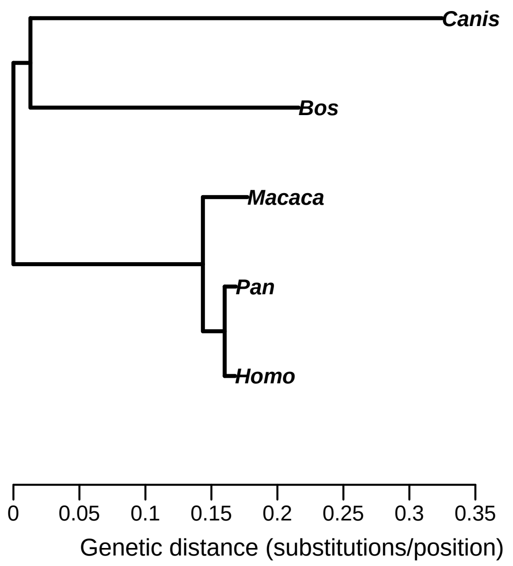

ENSG00000149292 intron 18

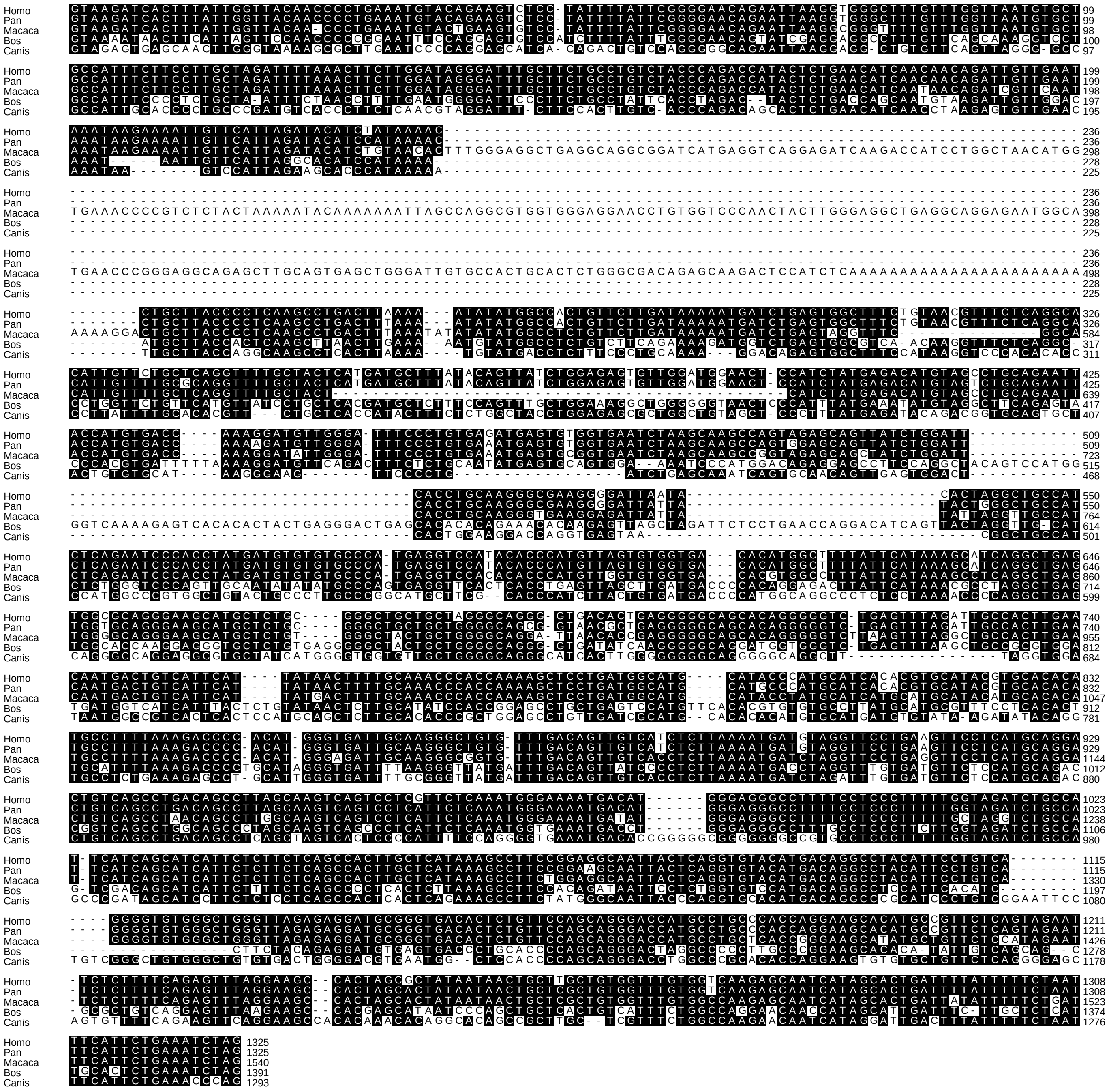

ENSG00000145826 intron 2

Description: Leukocyte cell-derived chemotaxin 2 precursor (LECT2)
Intron number: 2
Human chromosome: 05
Intron start (bp): 135314957
Human intron length : 1502
Intron alignment length: 1985
Flanking exons length (upstream/downstream): 97/146
SNP density: 0.006658
K tree score: 0.065
Scaling factor: 1.0317
Human-chimpanzee distance: 0.012183
Total primate branch length: 0.0826

ENSG00000145826 exon 2

```
Homo    CACTGGCAGGGCCATGGGCTAATATATGTGCTGGCAAGTCTTCCAATGAGATCCGGACGTGTGACCGCCATGGCTGTGGA 80
Pan     CACTGGCAGGGCCATGGGCTAATATATGTGCTGGCAAGTCTTCCAATGAGATCCGGACGTGTGACCGCCATGGCTGTGGA 80
Macaca  CACTGGCAGGGCCATGGGCTAATATATGTGCTGGCAAGTCTTCCAACGAGATCCGGACGTGTGACCGCCATGGCTGTGGA 80
Bos     CACTGGCTGGACCATGGGCTATTATATGTGCTGGCAAGTCTTCCAATGAGATCAGGACATGTGATGGCCATGGCTGTGGC 80
Canis   CACTGGCTGGACCATGGGCTAATATCTGTGCTGGCAGGTCTTCCAATGAGATCAGGACATGTGACAGCCACGGTTGTGGC 80

Homo    CAGTACTCTGCTCAAAG 97
Pan     CAGTACTCTGCTCAAAG 97
Macaca  CAGTACTCTGCTCAAAG 97
Bos     CAGTACACGGCTCAGAG 97
Canis   CAGTACACTGCCCAAAG 97
```

ENSG00000145826 exon 3

```
Homo    AAGTCAGAGGCCTCACCAGGGTGTGGACATCTTGTGCTCTGCTGGATCTACTGTGTACGCACCATTCACTGGAATGATTG 80
Pan     AAGTCAGAGGCCTCACCAGGGTGTGGACGTCTTGTGCTCTGCTGGATCTACTGTGTACGCACCATTCACTGGAATGATTG 80
Macaca  AAGTCAGAGGCCTCACCAGGGTGTGGACATCTTGTGCTCTGCTGGATCTACTGTGTATGCACCATTCACTGGAATGATTG 80
Bos     AAATCAGAAGCTTCACCAGGGTGTAGATGTCTTGTGCTCAGATGGCTCTACTGTTTACGCACCTTTCACCGGAAAGATCA 80
Canis   AAATCACAGGCTTCACCAGGGTGTGGATGTCTTGTGTTCTGATGGATCTACTGTGTATGCACCTTTCACCGGAATGATTG 80

Homo    TGGGCCAGGAGAAACCTTATCAAAACAAGAATGCTATCAATAATGGTGTTCGAATATCTGGAAGAG 146
Pan     TGGGCCAGGAGAAACCTTATCAAAACAAGAATGCTATCAATAATGGTGTTCGAATATCTGGAAGAG 146
Macaca  TGGGCCAGGAGAAACCTTATCAAAACAAAAATGCTATCAATAATGGTGTTCGAATATCTGGAAGAG 146
Bos     TGGGCCAGGAGAAACCTTATAAAAACAAAAATGCCATCAATAATGGTGTTCGGATCTCTGGAGGAG 146
Canis   TGGGCCAGGAGAAGCCTTATAAAAACAAAAATGCCATTAATAATGGTGTTCGGATATCTGGAAGAG 146
```

ENSG00000145826 intron 2

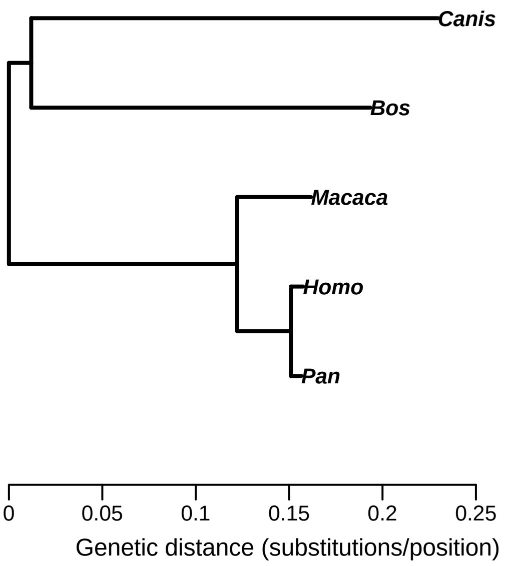

ENSG00000145826 intron 2

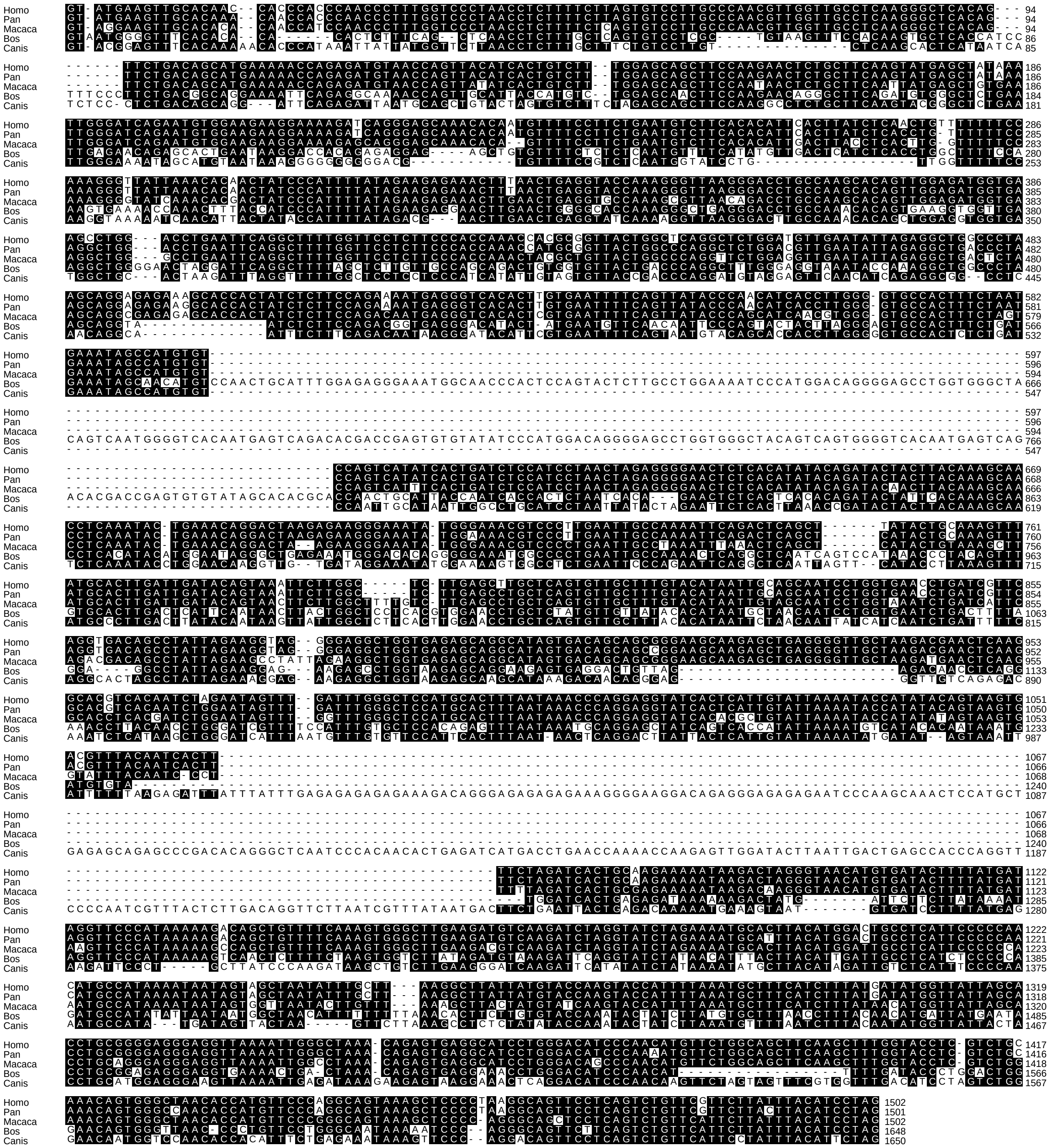

ENSG00000148218 intron 10

Description: Delta-aminolevulinic acid dehydratase (ALAD)
Intron number: 10
Human chromosome: 09
Intron start (bp): 115190463
Human intron length : 615
Intron alignment length: 675
Flanking exons length (upstream/downstream): 130/62
SNP density: 0.009756
K tree score: 0.0464
Scaling factor: 1.1021
Human-chimpanzee distance: 0.014908
Total primate branch length: 0.0824

ENSG00000148218 exon 10

```
Homo    CACCCTGACCTCCCTCTCGCCGTGTACCACGTCTCTGGAGAGTTTGCCATGCTGTGGCATGGAGCCCAGGCCGGGGCATT 80
Pan     CACCCTGACCTCCCTCTCGCCGTGTACCACGTCTCTGGAGAGTTTGCCATGCTGTGGCATGGAGCCCAGGCCGGGGCATT 80
Macaca  CACCCCGACCTCCCTCTCGCCGTGTACCATGTCTCTGGAGAGTTTGCCATGCTGTGGCACGGAGCCCAGGCTGGGGCATT 80
Bos     CACCCTGAGCTCCCCCTGGCTGTGTACCACGTTTCTGGAGAGTTTGCCATGCTGTGGCACGGGGCCCAGGCCGGGGCATT 80
Canis   CACCCTGAGCTCCCTCTTGCCGTGTACCACGTTTCCGGAGAGTTTGCCATGCTGTGGCACGGAGCCCGGGCCGGGGCGTT 80

Homo    TGATCTCAAGGCTGCCGTACTGGAGGCCATGACTGCCTTCCGCAGAGCAG 130
Pan     TGATCTCAAGGCTGCCGTACTGGAGGCCATGACTGCCTTCCGCAGAGCAG 130
Macaca  TGATCTCAAGGCTGCCGTACTGGAGGCCATGACTGCCTTCCGCAGAGCAG 130
Bos     TGATCTCAAGGCTGCTGTACTAGAGGTCATGACTGCTTTCCGCAGAGCAG 130
Canis   TGATCTCAAGGCTGCTGTTCTGGAGGCCATGACCGCCTTCCGCCGCGCAG 130
```

ENSG00000148218 exon 11

```
Homo    GTGCTGACATCATCATCACCTACTACACACCGCAGCTGCTGCAGTGGCTGAAGGAGGAATGA 62
Pan     GTGCTGACATCATCATCACCTACTACACACCACAGCTGCTACAGTGGCTGAAGGAGGAATGA 62
Macaca  GTGCTGACATCATCATCACCTACTACACACCCCAGCTGCTGCAGTGGCTGAAGAAGGAATGA 62
Bos     GTGCCGATGTCATCATTACCTACTACACACCTCAGCTACTGCAGTGGCTGA---AGGAGTGA 59
Canis   GTGCCGACATCATCATCACCTACTACACACCTCAGCTATTGCAGTGGCTGAAGGAGGAGTGA 62
```

ENSG00000148218 intron 10

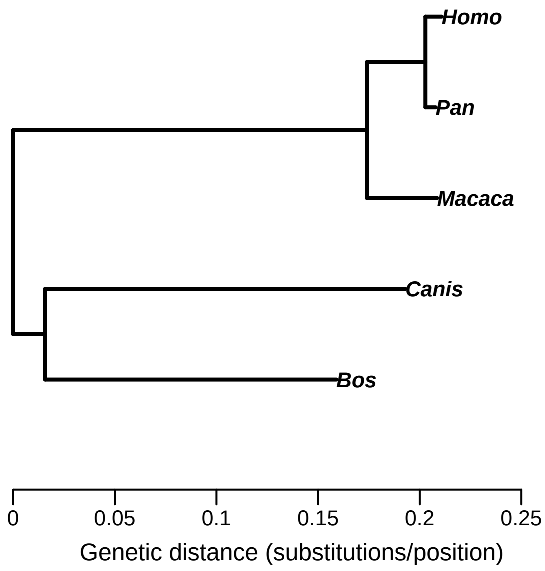

ENSG00000148218 intron 10

```
Homo    GTAGGCAGGCAAGGGTGGGG-TGTTTTGACCTGCGCCACAGGG-ACTGATAAGCACTCTGCCTAGATTGGGGAACGACGTC--CTGAGAGCTTGGGATCT 96
Pan     GTAGGCAGGCAAGGGTGGGG-TGTTTTGACCTGCGCCACAGGG-ACTGATAAGCACTCTGCCTAGATCGGGGAACGACGTC--CTGAGAGCTTGGGGTCT 96
Macaca  GTAGGCAGGCAGGGGTTGGG-TGTTTTGACCTGTGCCACAGGG-ACTGATAAGCACTCTGCCTAGATCAAGGAACGAAGTC--CTGAGAGCTTGGAGTCT 96
Bos     GTGAGCATGCAGGAGTTGGG-GGTTTTGACCTGGGCCACAGGGAACTGATAGGCACCCTGCCCAAATAGGGAAATAAAGTTAGGGGACAGCCTGGAGTCA 99
Canis   GTGAACAGGCGGGGCTGGGGTTTTTTTGACCTGGGCTACAGGG--CTGATGGGCACTTTGCCCAGAGAGGGAAATAAAGTT--GAGACAGCCTGGCGTCG 96

Homo    TATTCCGGGAATTACTAGTGATCTAAACAGACACACACTGAGGAAGAGATATGGAACTGCAGCATAGAACACGGCCCGGTGAAGCAAGCAGAGCCC---- 192
Pan     TATTCCGGGAATTACTAGTGTTCTAAACAGACACACATTGAGGAAGAGATATGGAACTGCAGCATAGAACACGGCCTGGTGAAGCAAGCAGAGCCCTTCA 196
Macaca  TATTTCGGGAATTACTAGTGATCTAAACAGACACACATTGAGGAAGAGATATGGAACTGCAGCATAGAACATGACATGGTGAAGCAAGCAGAGCCCTTCA 196
Bos     CCTGTCTGGAAATGCCAATGATCTAAACAGACAGGC--TGGGGAACGGACATGAAACTGCGGGGCAGAAGGTG-AGCAATGAACCATGCAGAGCCCTCAG 196
Canis   CATGCCTGGAAATAGCAGCGATCTAAA------------GAGGAGGGGACATGGAACCGCAGAACACAGCATGCAGCAGTGA---AGGAAGAACCCTCTG 181

Homo    TTCATTTTTGGTTGTGAGAACGTGGGCAAGCCACTTCTCTGAACCTCAGTGTCCTCACCCATAACTGGATAACTGGGGATAAGATACCTGGTGCGTGGTT 292
Pan     TTCATTTTTGGTTGTGAGAACGTGGGCAAGCCACTTCTCTGAACCTCAGTGTCCTCACCCATAACTGGATAACTGGGGATAAGATACCTGGTGCGTGGTT 296
Macaca  TTCATTTTTGGTTGTGAGAATGTGGGCAAGCCACTTCTTAGAACCTCAGTGTCCTCACCCATAACTGGATAACTGGGGACAAGATACCTGGTGCGTGATT 296
Bos     TTACTTTTTGATTTTGAGACCTTGGGCAAGTCACTTCTCT-AACTTCAGTGTCCTTGTCTGTA-------GACTGGGGATAAGACAGATGGTGCATGGTC 288
Canis   TCACTTTTTGGTTTTGAGACCTTAGCTGAGCCATGTCCGTGAACCTCAGAGTCCTTATCTGCA-------AACGGGGGATAAAACACCCGACAT-TGGTT 273

Homo    GTCCTGAGGATTAAATGA-AGTAATA---------------------TCACTCCATAAA-GGGGACTCATTTTGTTAGAATTGCACACCAGCATGGGAA- 368
Pan     GTCCTGAGGATTAAATGA-GGTAATA---------------------TCACTCCATAAA-GGGGACTCATTTTGTTAGAATTGCACACCAGCATGGGAA- 372
Macaca  GTCCTGAGGATTAAATGA-GGTAGTA---------------------TCACTCCATAAA-GGGGGCTCATTTTGTTAGAACTGTACACCAGCACAGGAAG 373
Bos     ATCAAGGGGATTAAATGA-GATGTTGCGTGTAATTGGCATATGCATGTCATTTCAGAAATGAGAGCTGGTTTTGTTGGTTAGGCACA-CAGCGTGGCGGG 386
Canis   GTCCGGAGAATTGAGTGAGGATGATATATGTCATTGGCATATGCTCTCCACTCTGCAAATGGGAGCTGGTTTTGTTGG-TGTGCATA-CAGCCTGAGGAG 371

Homo    GGAACTTGCCTCTTACCTATTTCCTTCACTGTGC-ATTTTATTCTTTGGTAAACTGAGGCCCCAAAAGAGGAAATGACTTGCCCA--------------- 452
Pan     GGAACTTGCCTCTTACCTATTTCCTTCACTGTGC-ATTTTATTCTTTGGTAAACTGAGGCCCCAAAAGAGGAAATGACTTGCCCA--------------- 456
Macaca  GGAACTTGCATCTTACCTATATCCTTCACTGTGC-ATATTATTCTTTGGTAAACTGAGGTCCCAAGAGAGAAAATGACTTGCCCA--------------- 457
Bos     GGAACTCACAGCTTGCCTATTTCCCCCACTGTGCAACTTTATACATGGACACACTGAGGCCCAGAAAGAGAAAATGCCTTCCCCAAGGTCACACAGTGTA 486
Canis   GGCCCTCACAGCTTACTCATTT-CCCCACCTTGC-ACCTTATACGTTGGCAAAC--AGGCCCAGCAAGCGAAAATGACTTGCCCAGGGTCGCACGGTGTG 467

Homo    -AGAAATAGAGTTTCCCAAAGCTGGGCTC-CGTCTCATGTGGTG-TGCCCACAGGCTGTGCTTCTTCATGGTAGCCTTCTTCCCCGCCT---------GG 540
Pan     -AGAAATAGAGTTTCCCAAAGCCGGGCTC-CGTCTCATGTGGTG-TGCCCACAGGCTGTGCTTCTTCGTGGTAGCCTTCTTCCCCGCCT---------GG 544
Macaca  -AGAAATACAGCTGCCCAAAGCTGGACTC-CGTCTCATGTGGTG-TGCCCACAGGCTGTGCCTCTTCATGGTAGCCTTCTTCCCCGCCT---------GG 545
Bos     TAGAGCTAGGACTGACCAGAACTAGACTCAGGTCTTAGGTGGTGATACCCTGGGGCTGTGCCTCTTCATAGTAGCCTTCTTCCCACCCC---------AG 577
Canis   CAGAGCTGTAACTGCCCGGAGCTTGGCTT-GATCTTATGTCATAATGCCCACGAGCTGTGCCCCTTCATGGTAGCAGCCTTCCACCCCCTCTGCTCTTGG 566

Homo    CCTTCCCATCGCAGAAGGTGTGCTCAGAGCTGATCAGCGTCCCCCCAGCAACTTTCTGCATCTCTCCCAACACAG 615
Pan     CCTTCCCATCGCAGAAGGTGTGCTCAGAGCTGATCAGCGTCCCCCCCGCAACTTTCTGCATCTCTCCCAACACAG 619
Macaca  CCTTCCCATCCCAGAAGGTGTGCTCGGAGCTGATCACCGTCCCCC--GCAACTTTCCGCATCTCTCCCAACACAG 618
Bos     CCCTCACCTCCCAGAAGGTATTTCCCAAGCTGATGC----CCTGC--CCCGCTGCCCGCTTCTTTCCCCACACAG 646
Canis   CCCTCGTCTCCCCAAAGGGGTGCCCAGAGCTGATGCCAGGCTTCT--CCCCCTTCCTGCATCTGTCCCC-CACAG 638
```

ENSG00000134028 intron 10

Description: ADAM DEC1 precursor (ADAMDEC1)
Intron number: 10
Human chromosome: 08
Intron start (bp): 24312933
Human intron length : 695
Intron alignment length: 1005
Flanking exons length (upstream/downstream): 82/131
SNP density: 0.001439
K tree score: 0.0739
Scaling factor: 0.865
Human-chimpanzee distance: 0.005791
Total primate branch length: 0.0824

ENSG00000134028 exon 10

Homo	CGGGATTAGCTTCAACAATCGACGTGTGGGACTGGCAGCTTCAAATTCCTTGTGTTCCCCATCTTCGGTTGCTGTTATTG	80
Pan	CGGGATTAGCTTCAACAATCGACGTGTGGGACTGGCAGCTTCAAATTCCTTGTGTTCCCCATCTTCGGTTGCTGTTATTG	80
Macaca	CGGGATTAGCTTCAACAATCGACGCGTGGGACTGGCAGCTTCAAATTCCTTGTGTTCCCCATCTTCGATTGCTGTTATTG	80
Bos	TGGAGTTGGCTTCAAAACTCGAATGGCAGGACTGGCGACCTCGAATTCCTTGTGCTCCCCATCATCCGTCGCTGTTATTG	80
Canis	TGGACTTGGCTTTGACAATCGACGTGTAGGAATGGCAGCATCAAATTCCTTGTGTTCTCCATCTTCAGTTGCTGTTATTG	80

Homo	AG	82
Pan	AG	82
Macaca	AG	82
Bos	AG	82
Canis	AG	82

ENSG00000134028 exon 11

Homo	GCTAAAAAAAAAGAATAATGTGGCTCTTGTAGGAGTGATGTCACATGAGCTGGGCCATGTCCTTGGTATGCCTGATGTTCC	80
Pan	GCTAAAAAAAAAGAATAATGTGGCTCTTGTAGGAGTGATGTCACATGAGCTGGGCCATGTCCTTGGTATGCCTGATGTTCC	80
Macaca	GCTAAAAAAAAAGAATAGTGTGGCTCTTGTAGCAGTGATGTCACATGAGCTGGGCCATGTCCTTGGTATGCCTGATGTTCC	80
Bos	GGTAAGAGAAAGAACAGTGTCTCTCTTGTAGGAGTGATGTCACACGAATTGGGTCATGTCCTTGGTATGCCTGATGTTCC	80
Canis	GCTAAGAAAAAGAACAATGTGGCTCTTGTAGGACTGATGTCACATGAGTTGGGTCATGTCCTTGGTATGCCTGATATTCC	80

Homo	ATTCAACACCAAGTGTCCCTCTGGCAGTTGTGTGATGAATCAGTATCTGAG	131
Pan	ATTCAACACCAAGTGTCCCTCTGGCAGTTGTGTGATGAATCAGTATCTGAG	131
Macaca	ATTCGACACCAAGTGTCCCTCCGGCAGTTGTGTGATGAATCAGTATCTGAG	131
Bos	ACATAACACCAAGTGTCCCTCTGGGAGTTGTGTGATGAATAAATATCTAAG	131
Canis	ATATTATACCAAGTGTCCCTCAGGGAGTTGTGTGATGAACCAGTATCTGAG	131

ENSG00000134028 intron 10

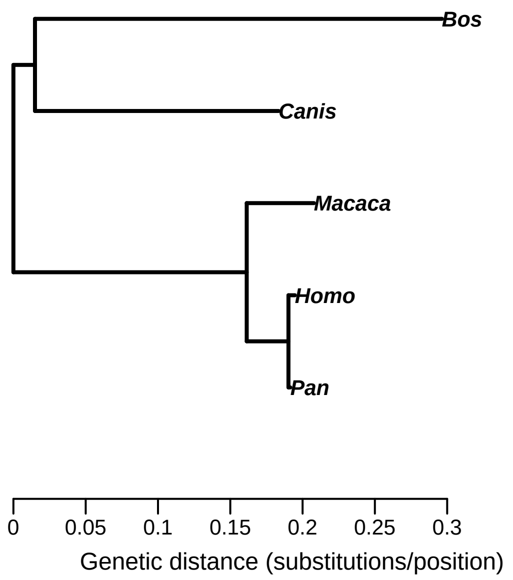

ENSG00000134028 intron 10

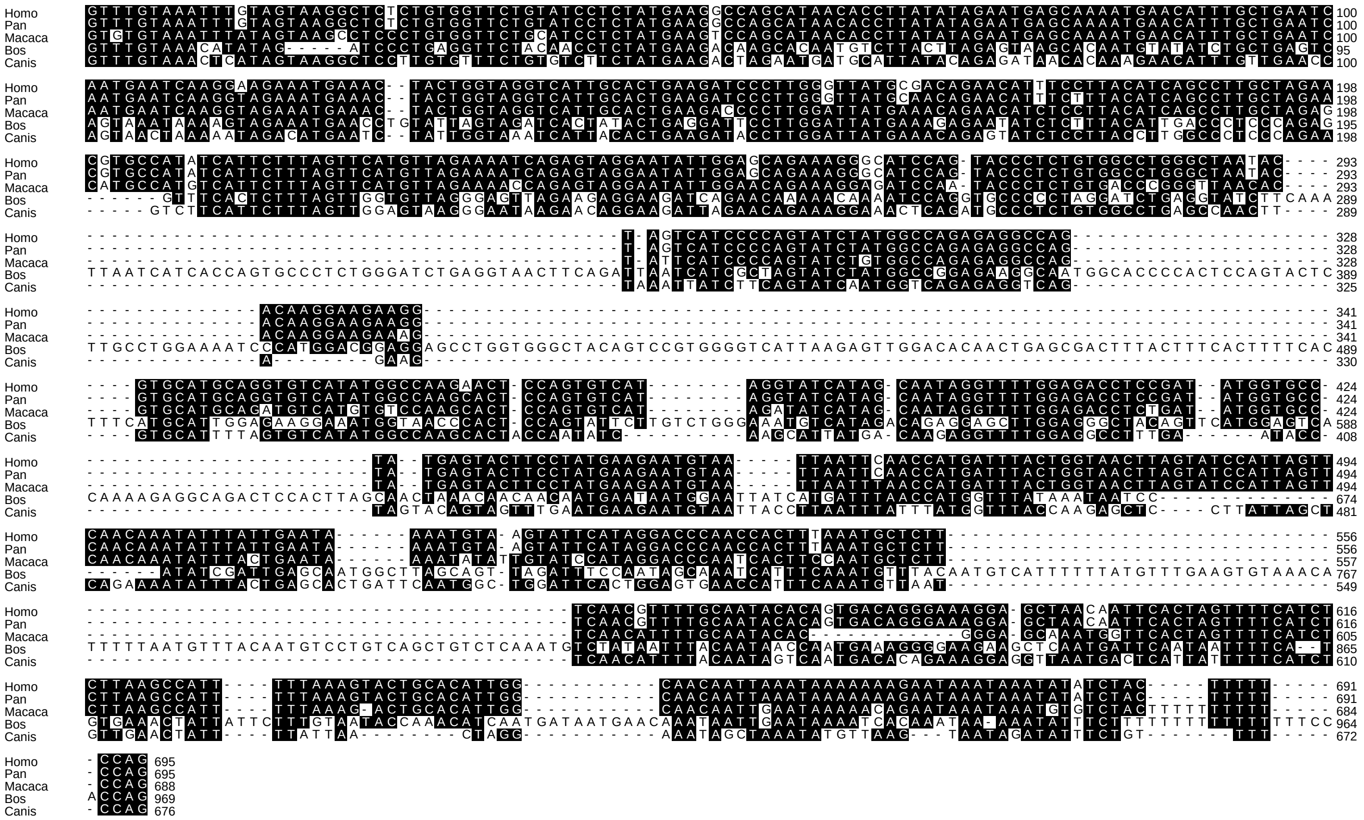

ENSG00000183862 intron 2

Description: Cyclic nucleotide-gated olfactory channel (CNGA2)
Intron number: 2
Human chromosome: X
Intron start (bp): 150658008
Human intron length : 682
Intron alignment length: 715
Flanking exons length (upstream/downstream): 93/171
SNP density: 0.004399
K tree score: 0.0513
Scaling factor: 0.7422
Human-chimpanzee distance: 0.010389
Total primate branch length: 0.0822

ENSG00000183862 exon 2

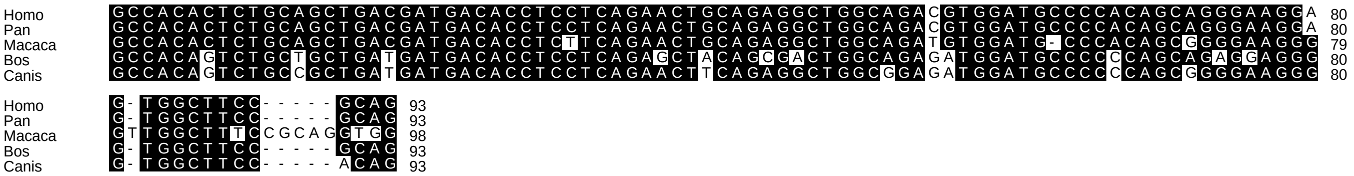

ENSG00000183862 exon 3

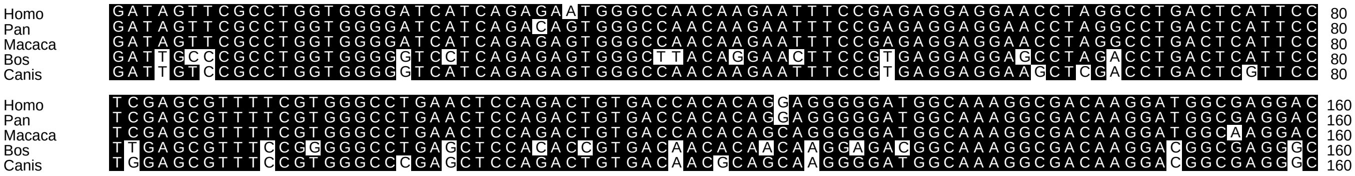

ENSG00000183862 intron 2

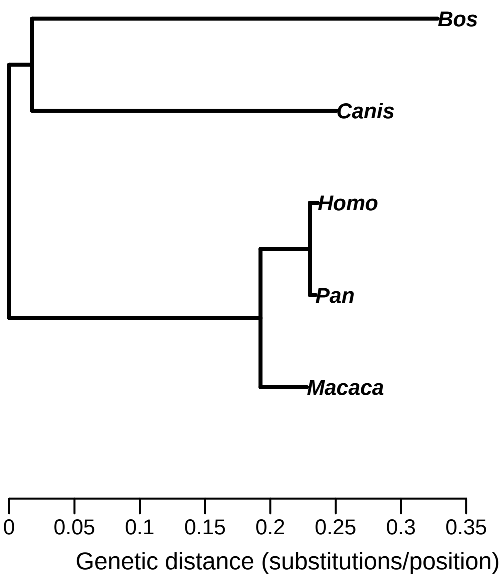

ENSG00000183862 intron 2

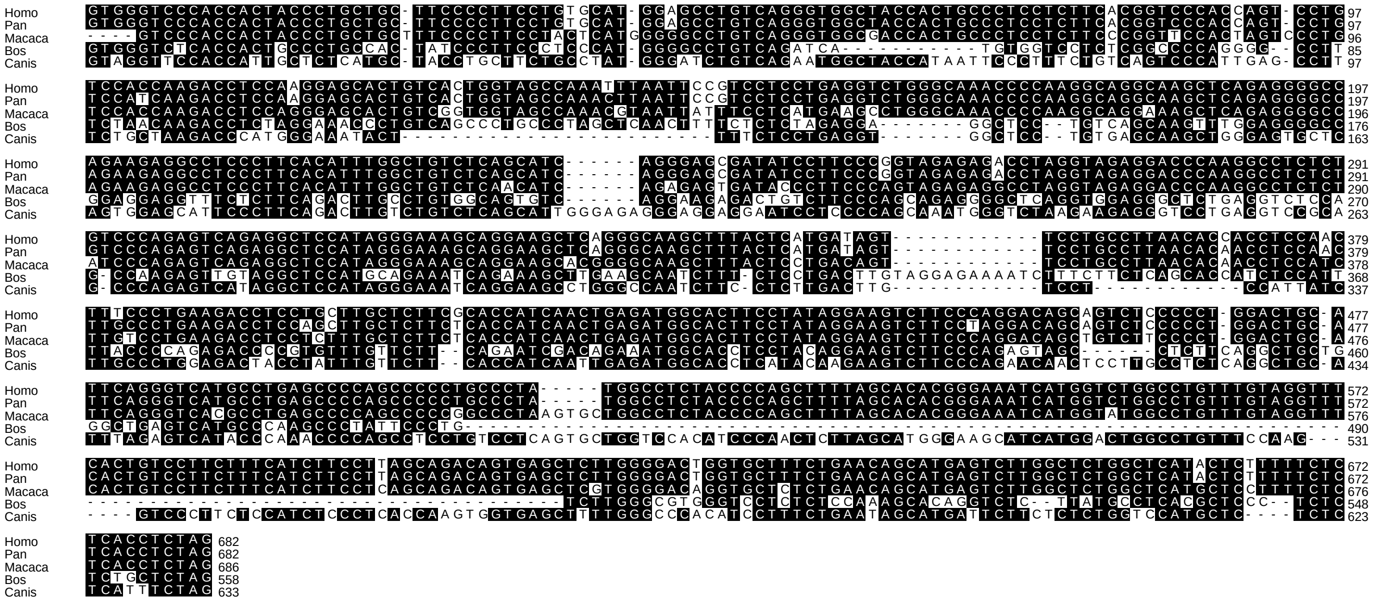

ENSG00000136878 intron 11

Description: Ubiquitin carboxyl-terminal hydrolase 20 (USP20)
Intron number: 11
Human chromosome: 09
Intron start (bp): 131671526
Human intron length : 246
Intron alignment length: 310
Flanking exons length (upstream/downstream): 116/173
SNP density: 0.000000
K tree score: 0.0862
Scaling factor: 0.5181
Human-chimpanzee distance: 0.008191
Total primate branch length: 0.0821

ENSG00000136878 exon 11

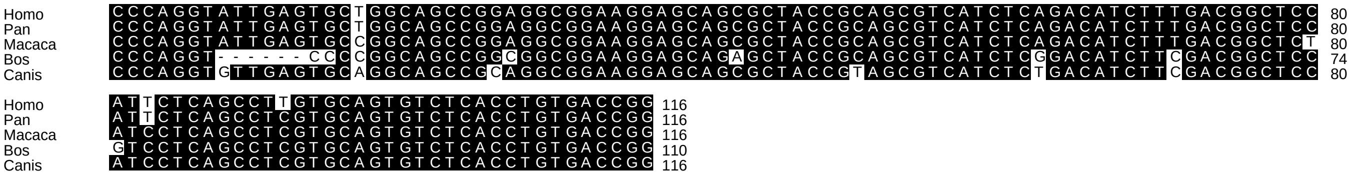

ENSG00000136878 exon 12

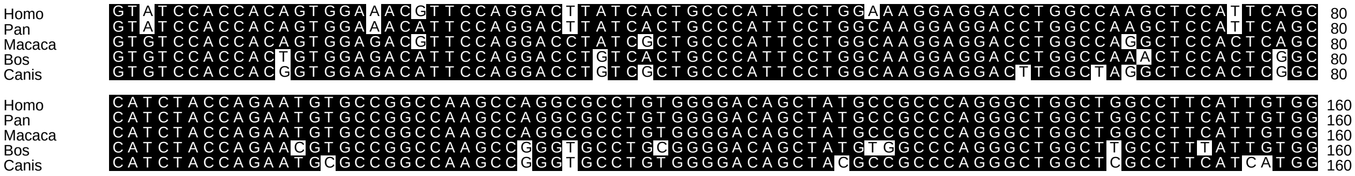

ENSG00000136878 intron 11

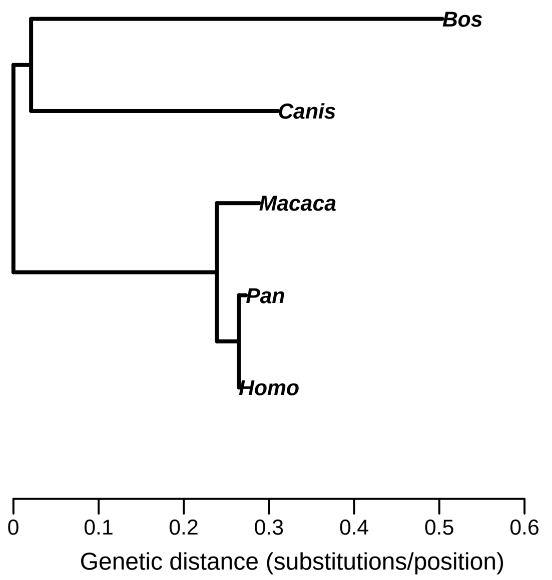

ENSG00000136878 intron 11

```
Homo    GTGGGTGC-CCCAGGGATGGGGGGAGCTGGGCCAGGCTGCCAGTGGCCTCAGCAGCTCTTGCCCTGACTGGGTGCAGGG--------------------- 78
Pan     GTGGGTGC-CCCAGGGATGGGGGGAGCTGGGCCAGGCTGCCAGTGGCCTCAGCAGCTCTTACCCTGACTGGGTGCAGGG--------------------- 78
Macaca  GTGGGTGCTCCCAGGGATGGGGGGAGCTGGGCCAGGCTGCCAGCGGCCTCAGCAGCTCTTGCCCTAACTGGGTGCAGGG--------------------- 79
Bos     GTGGGTGC---TGCGCGGGGAGGGGAGCGA-------TGCACAGGGC--------TTCCCACTCAGCTTGGGGCTGGGGCACTGGCTC------------ 70
Canis   GTGAGCCC---TGGGAGTAGAGAGGCCCAGGCCAGCCTTCAGTGGCC--------CTGGTGACCAGGTTGGGGGTGGGTGAATGGGTTGGGGTGGGTGAA 89

Homo    TGGGCTCTCCA-CGGGTGCTCCCTGCTTTTATCCA-GGGGAGGGCCCTGCCTGCGTGGATCCCGTGTCTATGGCCTGGGGAGTAGTCCCTCTGCCCCTCA 176
Pan     TGGGCTCTCCA-CGGGTGCTCCCTGCTTTTATCCA-GGGGAGGGCCCTGCCTGCGTGGATCCCCTGTCTATGGCCTGGGGAGTAGTCCCTCTGCCCCTCA 176
Macaca  TGGGCTCTCCA-TGGGTGCTCCCTGCTTTTCTCCA-GGGGAGGGCCCTGCCTGTGTGGATCCCGTGTCTGTGGCCCGGGGAGCAGGCCCTCTGCCCCTCA 177
Bos     TCCGCCCGGTACCCCCCGACCCCTGCATTTCTCTAGAGGCAGGCCCTGGTCCTGGGGCTCCCCGTGTCCCAGGACTGGGGAGCTGGCTCTCTGCCACACA 170
Canis   TGGGTTCTTCACCAGGGGGCCCCTGCCTTTCTGGATGGGGAGGGCCCTGATGGCCTGGCTCCCATGTCCAGGGGCTGGGGAGCCAACCCTCTGTCCTCTA 189

Homo    CCTGC-------TGCTTGGGGTTTGGGGTGCAGACATTG-----------------------CCAGAGGATGGGCAG-CAGACTGACCTTCAACC----- 240
Pan     CCTGC-------TGCTTGGGGTTTGGGGTGCAGACATTG-----------------------CCAGAGGATGGGCAG-CAGACTGACCTTCAACC----- 240
Macaca  CCTGC-------TGGTTGGGATTTGGGGTGCAGATATTG-----------------------CCAGAGGACGGGCAG-CAGACTAACGTTTAATC----- 241
Bos     CCTGTCATCTGGCAGCTGGGGTTTGGGGTCCAGACCTGGGCTCATCTGGGCAGGGCCGAACCCTGGCAGATGGAAAGCCAGGCTGACCTTCAACCTTCCC 270
Canis   CCTGCCATCTGGTGGCTGGGCTTCGGGCTGCAGACTCTGCTTGGTAAGGGCAGGGTGGCACCCTGGAAGGTGGAAACCCAGGCTGACCTTTAACC----- 284

Homo    ----CCACAG 246
Pan     ----CCACAG 246
Macaca  ----CCACAG 247
Bos     CCCGTCCCAG 280
Canis   ----TCACAG 290
```

ENSG00000107651 intron 11

Description: SEC23-interacting protein (SEC23IP)
Intron number: 11
Human chromosome: 10
Intron start (bp): 121669099
Human intron length : 1292
Intron alignment length: 2650
Flanking exons length (upstream/downstream): 153/96
SNP density: 0.006192
K tree score: 0.0843
Scaling factor: 0.9406
Human-chimpanzee distance: 0.012605
Total primate branch length: 0.0819

ENSG00000107651 exon 11

```
Homo    ATGCCTGAAGAGCCAAAGCTGACTTTGGATGAGTCGTATGACCTTGTTGTTGAAAATAAAGAAGTCCTAACTTTGCAAGA 80
Pan     ATGCCTGAAGAGCCAAAGCTGACTTTGGATGAGTCTTATGACCTTGATGTTGAAAATGAAGAAGTCCTAACTTTGCAAGA 80
Macaca  TTGCCTGAAGAGCCAAAGCTGACTATGGATGAGTCATGTGACCTTGATGTTGAAAATGAAGAAGTCCTAACTTTGCAAGA 80
Bos     ATACCCGAAGAGCCAAAGCTGACGTTGGATGAGTCTTGTGACCTTGATGTTGAAAATGAAGAAGTCCTGACTTTGCAAGA 80
Canis   ATACCTGAAGAGCCAAAGTTGACTTTGGATGAGTCCTGTGACCTTGACATTGAAAATGAAGAAGTCTTAAGTTTGCAAGA 80

Homo    AACTCTGGAAGCACTTAGCCTCTCTGAATATTTTAGCACTTTTGAAAAGGAAAAGATTGATATGGAGTCCCTG 153
Pan     AACTCTGGAAGCACTTAGCCTCTCTGAATATTTTAGCACTTTTGAAAAGGAAAAGATTGATATGGAATCCCTG 153
Macaca  AACTCTGGAAGCACTTAGCCTCTCTGAATACTTCAGCACTTTTGAAAAAGAAAAGATTGATATGGAATCTCTG 153
Bos     AACTCTGGAAGCACTTAGTCTCTCTGAATATGTTAGCACTTTTGAAAAGGAAAAGATTGATATGGAATCTCTG 153
Canis   AACTCTGGAAGCACTTAGCCTCTCTGAATATGTTAGCATATTTGAAAAGGAAAAGATTGATATGGAATCTCTG 153
```

ENSG00000107651 exon 12

```
Homo    CTTATGTGTACAGTTGATGACCTGAAGGAAATGGGGATACCCCTTGGACCCAGAAAGAAGATAGCTAACTTTGTAGAACA 80
Pan     CTTATGTGTACAGTTGATGACCTGAAGGAAATGGGGATACCCCTTGGACCCAGAAAGAAGATAGCTAACTTTGTAGAACA 80
Macaca  CTTATGTGTACAGTTGATGACCTGAAGGAAATGGGGATACCCCTTGGACCCAGAAAGAAGATAGCTAACTTTGTAGAACA 80
Bos     CTTATGTGTACAGTGGATGATCTGAAGGAAATGGGGATACCACTTGGACCCAGAAAGAAGATAGCTAACTTTGTAAAACA 80
Canis   CTTATGTGTACAGTTGATGACCTGAAGGAAATGGGCATACCTCTTGGACCCAGAAAGAAGATAGCTAACTTTGTAAAACA 80

Homo    TAAAGCAGCCAAACTG 96
Pan     TAAAGCAGCCAAACTG 96
Macaca  TAAAGCAGCCAAACTG 96
Bos     TAAAGCTGTCAAACTG 96
Canis   GAAAGCAGTTAAACTG 96
```

ENSG00000107651 intron 11

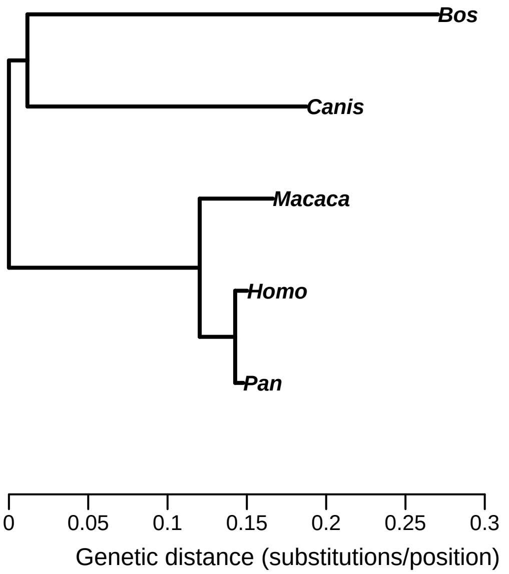

ENSG00000107651 intron 11

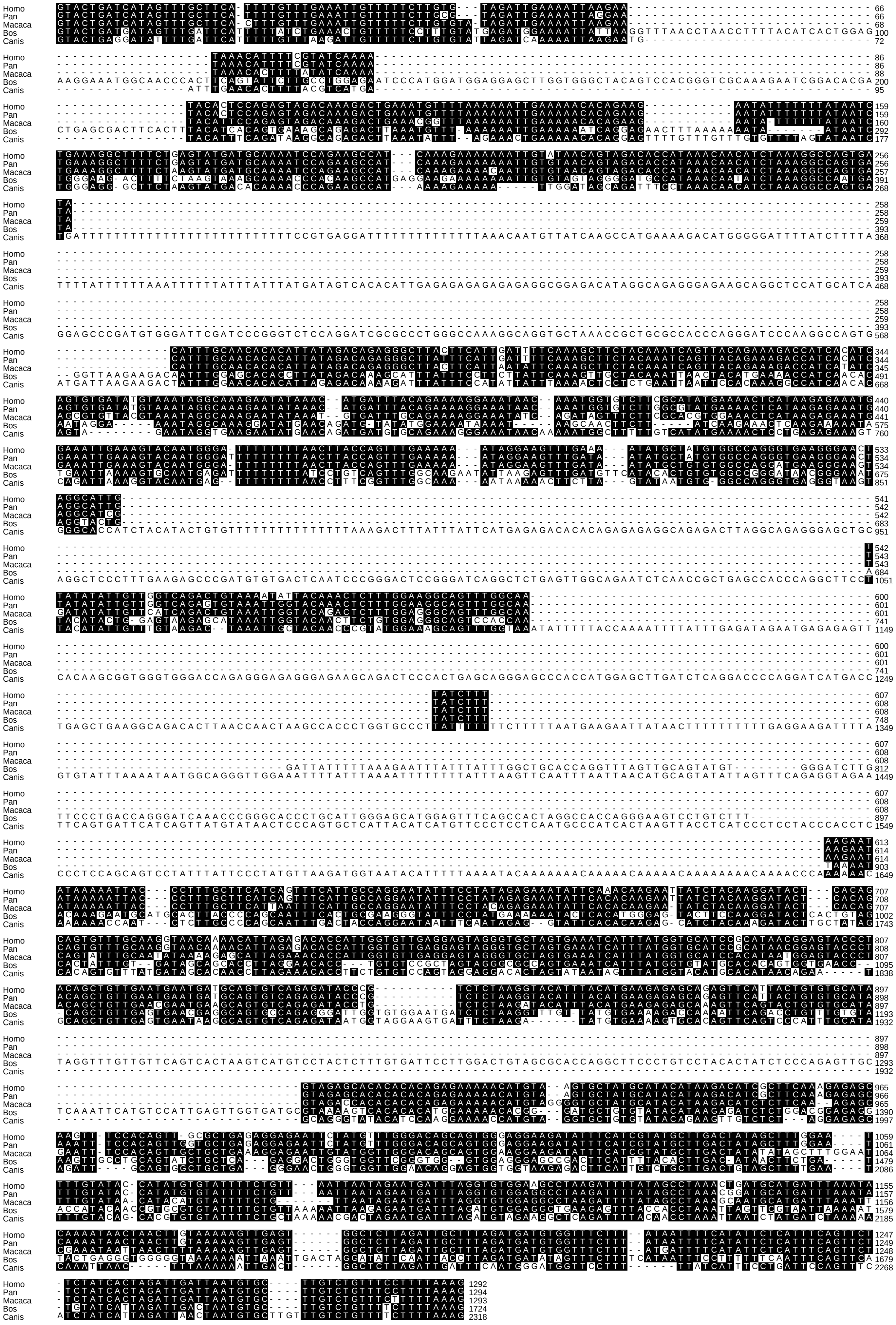

ENSG00000119913 intron 1

Description: Beta-tectorin precursor (TECTB)
Intron number: 1
Human chromosome: 10
Intron start (bp): 114033559
Human intron length : 724
Intron alignment length: 1110
Flanking exons length (upstream/downstream): 76/191
SNP density: 0.005525
K tree score: 0.0562
Scaling factor: 0.751
Human-chimpanzee distance: 0.012637
Total primate branch length: 0.0819

ENSG00000119913 exon 1

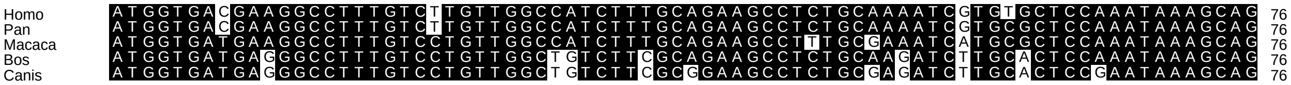

ENSG00000119913 exon 2

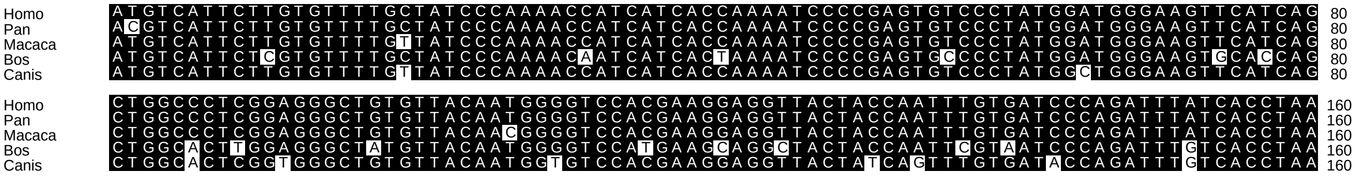

ENSG00000119913 intron 1

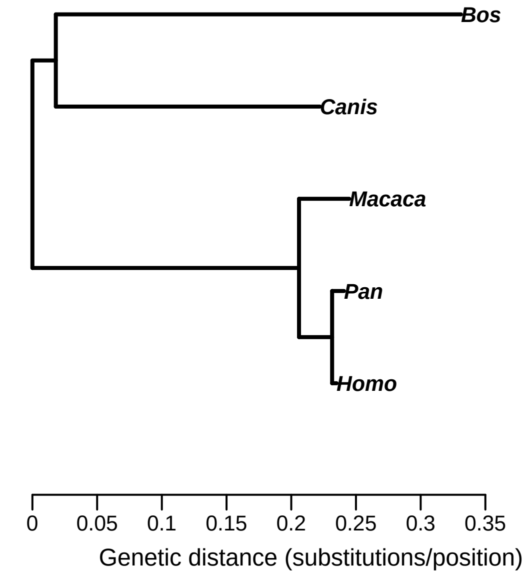

ENSG00000119913 intron 1

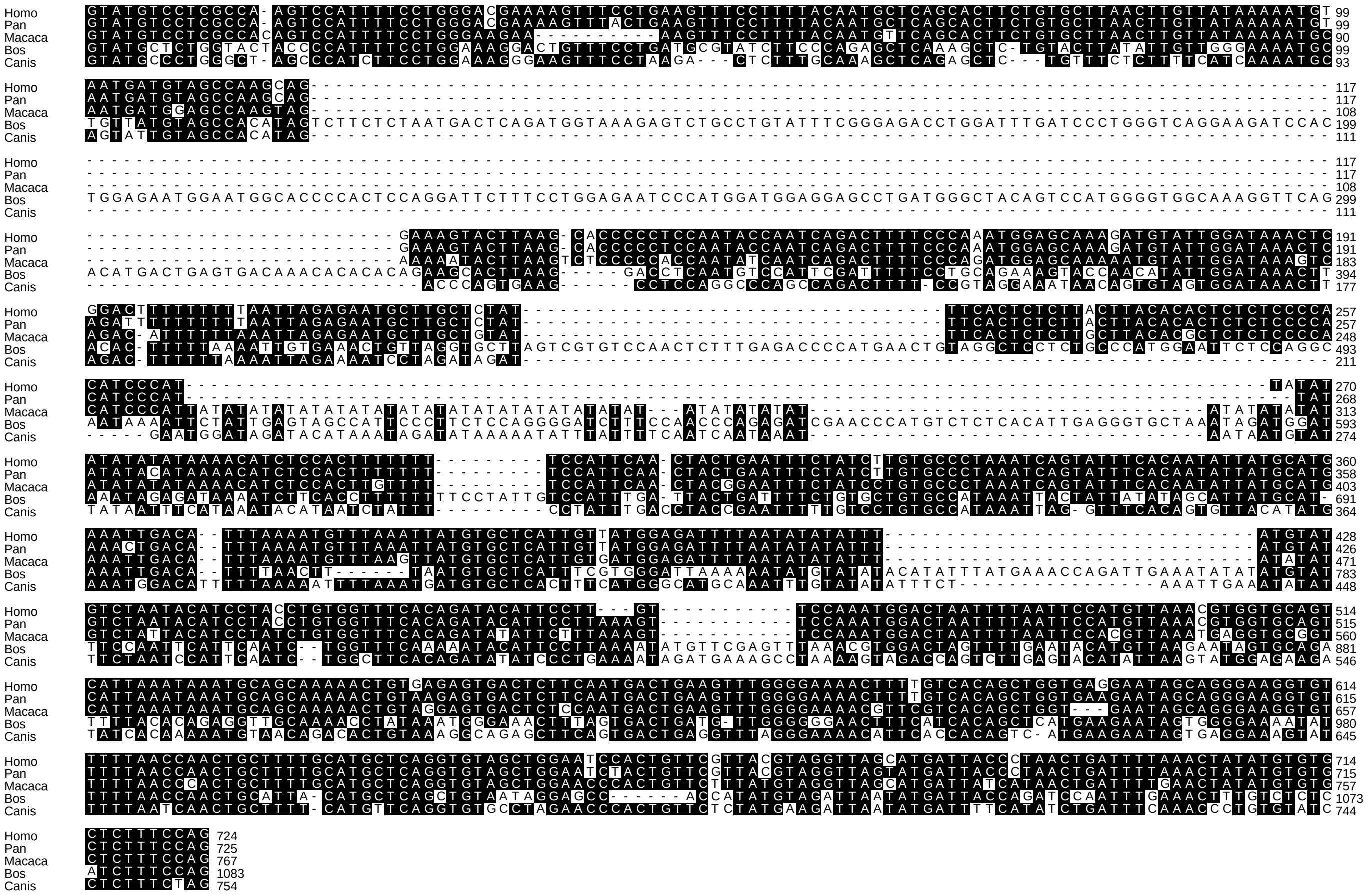

ENSG00000125538 intron 5

Description: Interleukin-1 beta precursor (IL1B)
Intron number: 5
Human chromosome: 02
Intron start (bp): 113304622
Human intron length : 718
Intron alignment length: 1005
Flanking exons length (upstream/downstream): 131/213
SNP density: 0.019499
K tree score: 0.0673
Scaling factor: 1.1149
Human-chimpanzee distance: 0.014146
Total primate branch length: 0.0819

ENSG00000125538 exon 5

```
Homo    TGGTGTTCTCCATGTCCTTTGTACAAGGAGAAGAAAGTAATGACAAAATACCTGTGGCCTTGGGCCTCAAGGAAAAGAAT 80
Pan     TGGTGTTCTCCATGTCCTTTGTACAAGGAGAAGAAAGTAATGACAAAATACCTGTGGCCTTGGGCCTCAAGGAAAAGAAT 80
Macaca  TGGTGTTCTCCATGTCCTTTGTACAAGGAGAAGAAAGTAATGACAAAATACCTGTGGCCTTGGGCCTCAAGGCAAAGAAT 80
Bos     TGGTGTTCTGCATGAGCTTTGTGCAAGGAGAGGAAAGAGACAACAAGATTCCTGTGGCCTTGGGTATCAAGGACAAGAAT 80
Canis   TGGTGTTCCACATGAGCTTTGTGCACGGGGATGAAAGTAATAACAAGATACCTGTGGTCTTGGGCATCAAACAAAAGAAT 80

Homo    CTGTACCTGTCCTGCGTGTTGAAAGATGATAAGCCCACTCTACAGCTGGAG 131
Pan     CTGTACCTGTCCTGCGTGTTGAAAGATGATAAGCCGACTCTACAGCTGGAG 131
Macaca  CTGTACCTGTCCTGTGTGTTGAAAGATGATAAGCCCACGCTACAGCTGGAG 131
Bos     CTATACCTGTCTTGTGTGAAAAAAGGTGATACGCCCACCCTGCAGCTGGAG 131
Canis   CTGTACCTGTCCTGTGTGATGAAGGATGGAAAGCCCACCCTACAGCTAGAG 131
```

ENSG00000125538 exon 6

```
Homo    AGTGTAGATCCCAAAAATTACCCAAAGAAGAAGATGGAAAAGCGATTTGTCTTCAACAAGATAGAAATCAATAACAAGCT 80
Pan     AGTGTAGATCCCAAAAATTACCCAAAGAAGAAGATGGAAAAGCGATTTGTCTTCAACAAGATAGAAATCAATAACAAGCT 80
Macaca  AGTGTAGATCCCAAAAACTACCCAAAGAAGAAGATGGAAAAGCGATTTGTCTTCAACAAGATAGAAATCAATAACAAGCT 80
Bos     GAAGTAGACCCCAAAGTCTACCCCAAGAGGAATATGGAAAAGCGCTTTGTCTTCTACAAGACAGAAATCAAGAATACAGT 80
Canis   AAGGTAGACCCCAAAGTCTACCCAAAGAGGAAGATGGAAAAGCGATTTGTCTTCAACAAGATAGAAATCAAGAACACAGT 80

Homo    GGAATTTGAGTCTGCCCAGTTCCCCAACTGGTACATCAGCACCTCTCAAGCAGAAAACATGCCCGTCTTCCTGGGAGGGA 160
Pan     GGAATTTGAGTCTGCCCAGTTCCCCAACTGGTACATCAGCACCTCTCAAGCAGAAAACATGCCCGTCTTCCTGGGAGGGA 160
Macaca  GGAATTCGAGTCTGCCCAATTCCCCAACTGGTACATCAGCACCTCTCAAGCGGAAAACATGCCCGTCTTCCTGGGAGGGA 160
Bos     TGAATTTGAGTCTGTCCTGTACCCTAACTGGTACATCAGCACTTCTCAAATCGAAGAAAGGCCCGTCTTCCTGGGACATT 160
Canis   GGAATTTGAGTCTTCTCAGTACCCTAACTGGTACATCAGCACCTCTCAAGTCGAAGGAATGCCTGTCTTCCTAGGAAATA 160
```

ENSG00000125538 intron 5

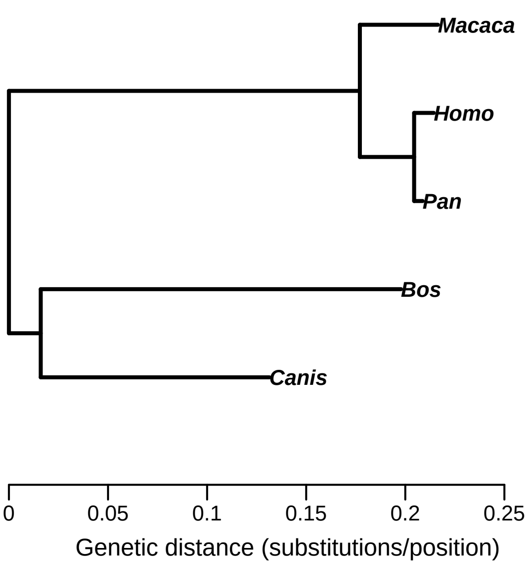

ENSG00000125538 intron 5

```
Homo    GTAAGTGAATGCTATGGAATGAAGC-CCTTCTCAGCCTCCT--------------GCTACCACTTAT------------TCCCAGACAACCACCTTCTCC 73
Pan     GTAAGTGAATGCTATGGAATGAAGC-CCTTCTCAGCCTCCT--------------GCTACCACTTAT------------TCCCAGACAACCACCTTCTCC 73
Macaca  GTAAGTGAATGCTATGGAATGAAGC-CCTTCTCAGTCTCCA--------------GCTATCACTTATAACCACCTTCTATCACTCACAACCACCTTCTCC 85
Bos     GTGAGTGAATACCAGGGGCTGAAGGCCCTTCTCAGGCTTTTCTGGGGCATCCCTAGCCATCACTTAC-----------TTCACAGACAACTAACTTTCCT 89
Canis   GTGAGTGAGTACGAGTGGCTAAAGG--CTTCCCAGGCTCCTCTCAAACAACCTTAGCCCTCACTTAC-----------TTCCAAGACAACCACTTTTCC- 86

Homo    CCGCCCCCATCCCTAGGAAAAGCTGGGAACAGGTCTATTTGACAATTTTGCATTAATGTAAATAAATTTAACATAATTTTTAACTGCGTGCAACCTTCAA 173
Pan     CCGCCCCCATCCCTAGGAAAAGCTGGGAACAGGTCTATTTGACAATTTTGCATTAATGTAAATAAATTTAACATAATTTTTAACTGCGTGCAACCTTCAA 173
Macaca  CCGCCCCCATCCCTAGGAAAAGCTGGGAACAGGTCTATTTGACAATGTTGCATTAATGTAAATAAATTTAACATAATTTTTAAGTGCATGCAACTTTCAA 185
Bos     CCTTCCCCA-------GAACCTCTGGGAGTAGATCTACATGATAATTTTGCATAAATGGAAATCAATTTAATGTAAGTGTCAAATGCATGTAGCTTTCAC 182
Canis   ----CCCCA-------GGAAATCTGGG---AGATCTACTTGATAATTCTGCATAAATGTCAACAAATTTAATATAATTGTTAAATGTATGCAACTTCCAC 172

Homo    TCCTGCTGCA-GAAAATTAAATCATTTTGCCGATGTTATTAT------GTCCTACCATAGTTACAACCCCAACAGATTATATATTGTTAGGGCTGCTC-- 264
Pan     TCCTGCTGCA-AAAAATTAAATCATTTTGCTGATGTTATTAT------GTCCTACCATAGTTACAACCCCAACAGATTATATATTGTTAGGGCTGCTC-- 264
Macaca  TCCTGCTGCA-GAAAATTAAATCATTTTTCTGATGTTATCGT------ATCCTACCATAGTTATGACCCCAACAGATTATATATTGTCAGGGCTGCTC-- 276
Bos     TCCCATAACACAAAAAGTAAATTATTCTGCAGATTTTTTTCC------GTCTTGCCA----------CCAAACAGATTATACATTGTTAGAGTTCCTCCA 266
Canis   CTCCACCACA-GAAAATTAAGTCATTTTTCTGATATTATGTCCTGGCATTGTTACCA----------CCTAACAGATTATACAATGTTAGGGTT------ 255

Homo    ---------------------------------------------------------------------------------------------------- 264
Pan     ---------------------------------------------------------------------------------------------------- 264
Macaca  ---------------------------------------------------------------------------------------------------- 276
Bos     TTTGATGGGACTTCCGTGGTGGCTCAGAGGTAAAGAATCTGCCTGCAAACGCAAGAGATTCCAATTAGACCTCTGCATCGGGAAGAAACCCTGGAGTAGG 366
Canis   --------------------------------------------------------------------------------AGAAGG-------------- 261

Homo    ---------------------------------------------------------------------------------------------------- 264
Pan     ---------------------------------------------------------------------------------------------------- 264
Macaca  ---------------------------------------------------------------------------------------------------- 276
Bos     AAATGGCAACTCACTCCAGTATTCTTGCCTGGAAATGCTATGGACAGAGGAGCCTGGTGGGTTACAGTCCATGGGATCACATAAGAGTTGGACACTTCTG 466
Canis   ------------ATTATAACATTGTTG------------------------------------------------------------------------G 277

Homo    -------------------TCATTTGATA---GACACCTTGGGAAATAGATGACTTAAAGGGTCCCAT----TATCATGTCCACTCCACTCCCAAAATTA 338
Pan     -------------------TCATTTGATA---GACACCTTGGGAAATAAATGACTTAAAGGGTCCCAT----TATCACGTCCACTCCACTCCCAAAATCA 338
Macaca  -------------------TCATTTGATA---GACACTTTGGGAAATAAATGACTTAAAGGATCCCAT----TGTCACATCCTCTCCACTCCCAAAATCA 350
Bos     AGTGACTAAACAACAACCACCATTTGATAGATGACACTTTGAGGACTTAATCGGTTCAAGGGACCAGTCAC-TGCCATCTCCACTGCACTCCCAAAACCA 565
Canis   AGTTACT------------CCATTTGATAAATGACACCTTGGGAAATTAATCAATTCAAGGGAGCTATCACTTGTCATCTC--CTCCACTCCCAAAATCA 363

Homo    CCACCACTATCACC----TCCAGCTTTCTCAGCAAAAGCTTCATTTCCAAGT----TGATGTCATTCT----AGGACCATAAGGAAAAATACAATAAAAA 426
Pan     CCACCACTATCACC----TCCAGCTTTCTCAGCAAAAGCTTCATTTCCAAGT----TGATGTCATTCT----AGGACCATAAGGAAAAATACAATAAAAA 426
Macaca  CCACCACTATCACC----TCCAGCTTTCCCAGCAAAAGCTACATTTCCAAGT----TGATGTCATTCT----AGGACCATAGGGAAAAATACAGTAAAAA 438
Bos     CACCTCCTTTTCCC----ACCAGTTTTCCCAGCAAAAATTTCATGTCCAACT----TGAAGTCATTCTAGCCAGAGCCATGAGGAAAAACACAGGATAAG 657
Canis   CCTTCCCCTCTCCTGACAACCAGCTGTCCCAGCAAAAGTTTCATGTCTAGTCATTGTGAAGTCATTCTAGCCAGGGCCATATGGAAAAATACAGCAAAAG 463

Homo    GCCCCTGGAAACTAGGTACTTCAAGAAGCTCTAGCTTAATTTTCACCCC--CCCAAAAAAAAAAAATTCTCACCTACATTATGCTCCTCAGCATTTGGCA 524
Pan     GCCCCTGGAAACTAGGTACTTCAAGAAGCTCTAGCTTAATTTTCACCCCAAAAAAAAAAAAAAAAAATTCTCACCTACATTATGCTCCTCAGCATTTGGCA 526
Macaca  GCCCCTGGAAAATAGGTACTTCAAGAAGCTCTAGCCTAATTTTCACTT----------AAAAAAAAAATTCACCTACATTATGCTCCTCAGCATTTGGCA 528
Bos     TCCCTGGAAA-CTAGCTACACCTAGAAGCTTTTACCTAATTTTCACTTTT--------GAA-AACATTTCCACCTATATTATACACACTAGCATTTGATG 747
Canis   TCGCTGGAAAGCTAGCTACTCTTAGAAATTTTAATCTAATTCTCACTTTT--------TAATAAAATTCCCACCTATATTTTGCTCCCTATCATTTGATA 555

Homo    CTAAGTTTTAGAAAAGAAGAAGGGCTCTTTTAATAATCACACGGAAAGTTGGGGGCCCAGTTACAACTCAGGAGTCTGGCTCCTGATCATGTGACCTGCT 624
Pan     CTAAGTTTTAGAAAAGAAGAAGGGCTCTTTTAATAATCACACGGAAAGGTGGGGGCCCAGTTACAACTCAGGAGTCTGGCTCCTGATCATGTGACCTGCT 626
Macaca  CTAAGCTTTAGAAAAGAAGAAGAGCTCTTTTAGTAACCACACAGAAAGTTGGGGGCCCAGTTCTAACTCAGGAG------TCCTGATCCTGTGACCCGCT 622
Bos     CTAAGTTTCAGAGAAGGATAAGTGTGCCTTCTGTCACCGCACAGAAGGCCGGGGTCACAAAGACAACTCAGGAGTCTGGCTCCTGGACCCG-A----GCC 842
Canis   CTAAGTTTCAGAAAAGAGTAAGAGCTCTTTCAATCACTACACAGAAAATTGGGGCCACAAAGATAAATCGGGAGTCTGGATCCTGGTCCTATG----ATT 651

Homo    CGTCAGTTTCCTTTCTGGCCAACCCAAAGAACATCTTTCCC-----------ATAGCATCTTTGTCCCTTGCCCCACAAAAATTCTTCTTTCTCTTTCGC 713
Pan     CATCAGTTTCCTCTCTGGTCAACCCAAAGAACATCTTTCCC-----------ATAGCATCTTTGTCCCTTGCCTCACAAAAATTCTTCTTTCTCTTTCGC 715
Macaca  CATCAGTTTCCTCTCTGGCCAACCCAAAGAACATCTTTCCC-----------ATGGCATCTTTGTCCCTTGCCTCACAAAAATTCTTCTTTCTCTTTTGC 711
Bos     CTGCTGATCCATTTTTAAATGACCCAGAGAACAACTTTGTCCAGACCAGGGTAGAATGTCTTTGCCCTTTCCTTCTTAAAGATGTTTATTTCTGTTTTGC 942
Canis   CCTCAGTTTCCTCTCTAGCTGAACTAGAAAATAACCTCACC-----------AGAGTGTCTTTGTCCCTTCCCTGATAAAAATTGTTATTTCTGTTTTGT 740

Homo    TGCAG 718
Pan     TGCAG 720
Macaca  TGCAG 716
Bos     TGCAG 947
Canis   TGCAG 745
```

ENSG00000165281 intron 9

Description: Fanconi anemia group G protein (FANCG)
Intron number: 9
Human chromosome: 09
Intron start (bp): 35065752
Human intron length : 207
Intron alignment length: 315
Flanking exons length (upstream/downstream): 67/290
SNP density: 0.000000
K tree score: 0.0982
Scaling factor: 1.0685
Human-chimpanzee distance: 0.009630
Total primate branch length: 0.0819

ENSG00000165281 exon 9

```
Homo    GGCAGGAGACGCTGCAGAGCATTACTTGGACCTGCTGGCCCTGTTGCTGGATAGCTCGGAGCCAAGG  67
Pan     GGCAGGAGACGCTGCAGAGCATTACTTGGACCTGCTGGCCCTGTTGCTGGATAGCTCGGAGCCAAGG  67
Macaca  GGCAGAAGACGCTGCAGAGCATTACTTGGACCTGCTGGCCCTGTTGCTGGATAGCTCGGAGCCAAGG  67
Bos     GGCAGCAGACGCTGCAGAACATTACCTGGACCTGCTCGCCCTGTTGCTGGATGGCTCAGAGCCAAAG  67
Canis   GGCTGAGGACGCTGCAGAGCATTACTTGGATCTGCTGGCTCTGTTGCTGTCTGACTCAGAGCCAAAG  67
```

ENSG00000165281 exon 10

```
Homo    TTCTCCCCACCCCCCTCCCCTCCAGGGCCCTGTATGCCTGAGGTGTTTTTGGAGGCAGCGGTAGCACTGATCCAGGCAGG  80
Pan     TTCTCCCCACCCCCCTCCCCTCCAGGGCCCTGTATGCCTGAGGTGTTTTTGGAGGCAGCGGTAGCACTGATCCAGGCAGG  80
Macaca  TTCTCCCCGCCCCCCTCCCCTCCAGGGCCCTGTATGCCTGAGGTGTTTTTGGAAGCAGCGGTAGCACTGATCCAGGCAGG  80
Bos     TTCTCCCCACCACCTTGCCCTCCAGGGCCCTGTGTACCTGAGTTGTTTTTGGAGGCAGCAGCAGCGTTGATCCAGGCAGG  80
Canis   TTCTCCCCACCTCCTACCCACCCAGGGCCCTGTATGCCTGAGGTGTTCTTAGAGGCAGCAGCAGCGCTGATCCAGGCAGG  80

Homo    CAGAGCCCAAGATGCCTTGACTCTATGTGAGGAGTTGCTCAGCCGCACATCATCTCTGCTACCCAAGATGTCCCGGCTGT  160
Pan     CAGAGCCCAAGATGCCTTGACTCTATGTGAGGAGCTGCTCAGCCGCACATCATCTCTGCTACCCAAGATGTCCCGGCTGT  160
Macaca  CAGAGCCCAAGATGCCTTGACTCTATGTGAGGAGTTGCTCAGCCGCACATCGTCTCTGCTACCCAAGATGTCCCGGCTGT  160
Bos     CCGAGCCCAGGATGCTTTGACCGTATGTGAGGAGCTGCTTAGCCGTACATCATTTCTGCTTCCCAAGAGGCCTCGGCTGT  160
Canis   CCGAGCCCAGGATGCCTTGACCGTATGTGAGGAGCTGCTCAGCCGCACGTCATCTCTGCTTCCCAAGATGCCCCAGCTGT  160
```

ENSG00000165281 intron 9

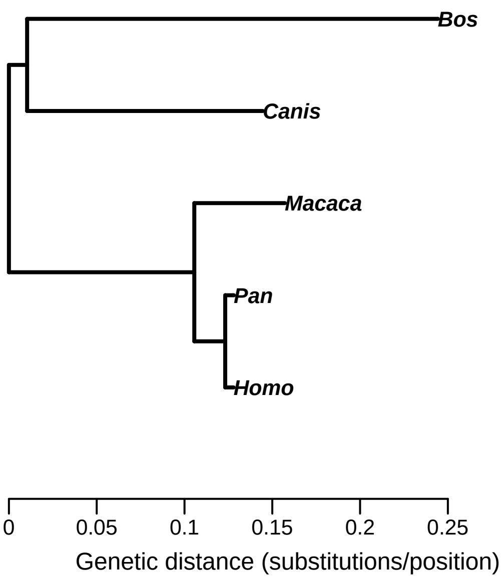

ENSG00000165281 intron 9

```
Homo    GTGGGTGTGTCTTCAAGCTTCTCTGCAATGGGGTAGACGGGTTGGTGTCCCCTTTGAAATACAGCAAGA------------------------------- 69
Pan     GTGGGTGTGTCTTCAAGCTTCTCTGCAATGGGGTAGACGGGTTGGTGTCCCCTTTGAAATACAGCAAGA------------------------------- 69
Macaca  GTGAGTGTGTCTTCAAGCTTCTCTGCAGTGGGGTAGAAGGGTTGGTGTCCCCTTTGGAATAAAGGGGGATTTTGTTTTTCCCTCTTTTTTTTTTTTTTTT 100
Bos     GTGGGTATG---CCACGTTTCTATGTAGTGGGGTGGGAGAGTTGGAGTTTCCTTTGGAGTAGAGCAGGA------------------------------- 66
Canis   GTGAGTATATCGTCAAGGTTCTCTATAATTGGGTAGGAGGGTTGGAGTCTCCTTTGGAGTACAGCAGGA------------------------------- 69

Homo    --------CTGCAATTTTTTGTTTTTTCCTCTA---TTCTGTCTGTCCAGTCTGGGGGCCCA-------------------------------------- 120
Pan     --------CTGCAATTTTTTGTTTTTTCCTCTA---TTCTGTCTGTCCAGTCTGGGGGCCCA-------------------------------------- 120
Macaca  TTTTTTTTTTGCAATTTTTTGTTTTTCCCTCTT---TTCTGTCTGTCCAGTCTGGGGGCCCA-------------------------------------- 159
Bos     --------GTGCTTTTTTCTGACCTTTCCTCTGCTCTTTTTTTTGTCCATTCTGGGAGACCAGATTGGGAAGGTTCCCAGAGCAAGGGAGATCCAGGGCA 158
Canis   --------CTGCTGTTTTCTCTTTTTATTTCTG---CTTT-TCTGTCCAAGTTGGAAGGCCAGACCAGGAAGGTTCCCAGAGCAAAG--GGTCCAGTGTA 155

Homo    -------------GGACTCTGCATGGTACCAGAGCCTGG-TGAAGAGG--------TTGGGGATGGTGGCTCATGCCT-TTCAAGACAC-----TGCTTC 192
Pan     -------------GGACTCTGCATGGTACCAGAGCCTGG-TGAAGAGG--------TTGGGGATGGTGGTTCATGCCT-TTCAAGACAC-----TGCCTC 192
Macaca  -------------GGACTCTGCATAGTACCAGAGCCTGG-TGAAGAGG--------TTGGGGATGGTGGCTTATGCCT-TTGAAGACAC-----TGCCTC 231
Bos     GCAAGCAGTGGATGGTCCCTGCATGGTACTAGAGCCTGG-TGATGATACTAGAGCCTTGGAGGTAGTGGTTCATGTCTCCTCAAGACACTGCATTGCATT 257
Canis   GCAAGCAATGGGTGGTCTCTGTGTGGTACCAGAGCCTGGTTGAAGAGG--------TTGGGGA---TGGCTAATGCCTCTTTAAGACAC-----TGCATC 239

Homo    TTCTTCCCACTCCAG 207
Pan     TTCTTCCCACTCCAG 207
Macaca  TTCTTCCCACTCCAG 246
Bos     CTCTTCCCACCGCAG 272
Canis   CTCCTCCCACCCCAG 254
```

ENSG00000166479 intron 14

Description: Protein disulfide-isomerase TXNDC10 precursor (TMX3)
Intron number: 14
Human chromosome: 18
Intron start (bp): 64497749
Human intron length : 1449
Intron alignment length: 1518
Flanking exons length (upstream/downstream): 130/69
SNP density: 0.002070
K tree score: 0.0927
Scaling factor: 0.8529
Human-chimpanzee distance: 0.014148
Total primate branch length: 0.0817

ENSG00000166479 exon 14

```
Homo    TGAATTGACAGTCCCAACTGTAGTTGTACTGAATACTTCAAACCAGCAATATTTCTTGCTAGATAGACAGATTAAGAATG 80
Pan     TGAATTGACAGTCCCAACTGTAGTTGTACTGAATACTTCAAACCAGCAATATTTCTTGCTAGATAGACAGATTAAGAATG 80
Macaca  TGAATTGACAGTCCCAACTGTAGTTGTACTGAATACTTCAAACCAACAATATTTCTTGCTGGATAGACAGATTAAGAATG 80
Bos     TGAATTGAAAGTCCCAACTGTGGTTGTACTGAATACTTCAAACCAACAGTACTTTTTACTAGATAGACAGATTAAGAATG 80
Canis   TGAACTCAAAGTCCCAACGGTGGTTGTACTAAATACTTCCAACCAACAGTACTTTTTACTAGACAGACAGATCAAGAATG 80

Homo    TTGAAGACATGGTCCAGTTTATTAATAACATTTTGGATGGCACAGTAGAA 130
Pan     TTGAAGACATGGTCCAGTTTATTAATAACATTTTGGATGGCACAGTAGAA 130
Macaca  TTGAAGACATGGTCCAGTTTATAAATAACATTTTGGATGGCACAGTAGAA 130
Bos     CAGAAGACATGGTCCAGTTCATTAATAACATCTTGGATGGCACAGTAGAT 130
Canis   CCGAGGACATGGTGCAGTTCATTAATAACATCTTGGATGGCACAGTGGAG 130
```

ENSG00000166479 exon 15

```
Homo    GCCCAAGGAGGTGATAGCATTTTGCAGAGATTGAAAAGAATAGTATTTGATGCCAAATCTACTATTGTG 69
Pan     GCCCAAGGAGGTGATAGCATTTTGCAGAGATTGAAAAGGATAGTATTTGATGCCAAATCTACTATTGTG 69
Macaca  GCCCAAGGAGGTGATAGCATTTTGCAGAGATTGAAAAGAATAGTATTTGATGCCAAATCTACTATTGTG 69
Bos     GCCCAAGGAGGTGACAGCATTTTGCAGAGATTGAAACGAATAGTATTTGATGCCAAATCTACTGTTGTG 69
Canis   GCCCAAGGAGGTGATAGCATATTGCAGAGATTGAAGAGGATAGTATTTGATGCCAAATCTACTATTGTG 69
```

ENSG00000166479 intron 14

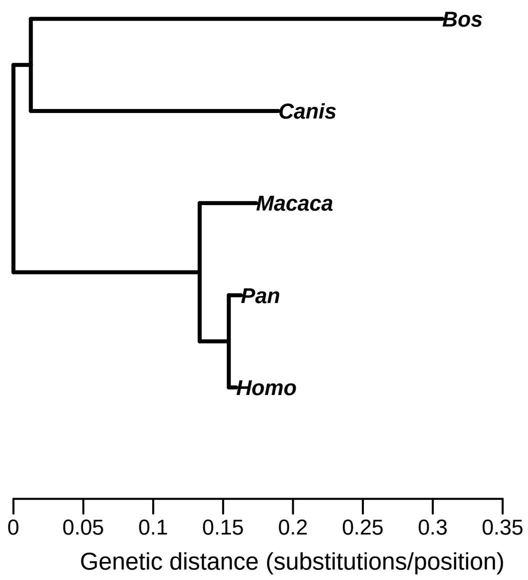

ENSG00000166479 intron 14

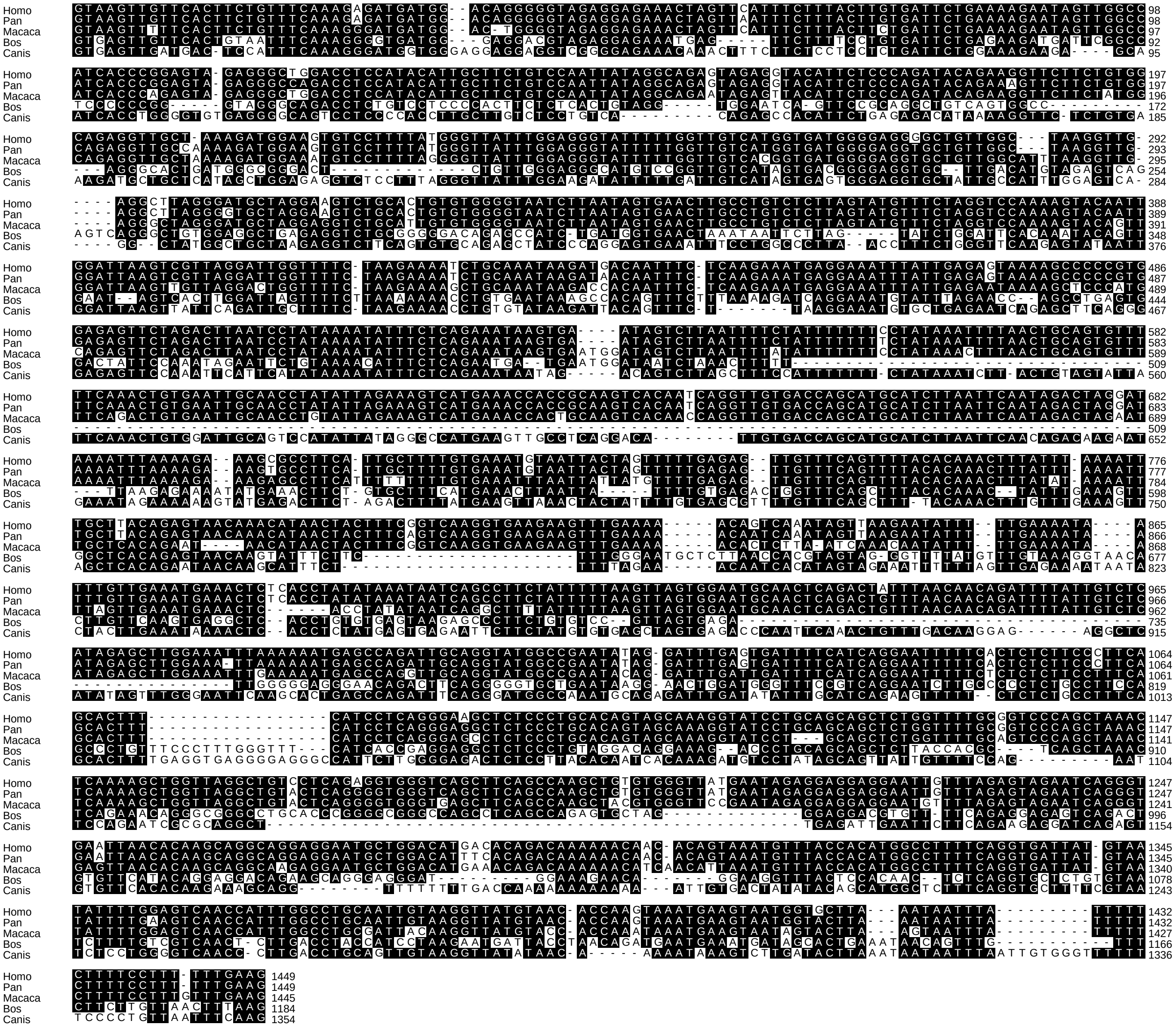

ENSG00000158525 intron 1

Description: Carboxypeptidase A5 precursor (CPA5)
Intron number: 1
Human chromosome: 07
Intron start (bp): 129773679
Human intron length : 1164
Intron alignment length: 1253
Flanking exons length (upstream/downstream): 116/82
SNP density: 0.004296
K tree score: 0.0558
Scaling factor: 0.7896
Human-chimpanzee distance: 0.013941
Total primate branch length: 0.0815

ENSG00000158525 exon 1

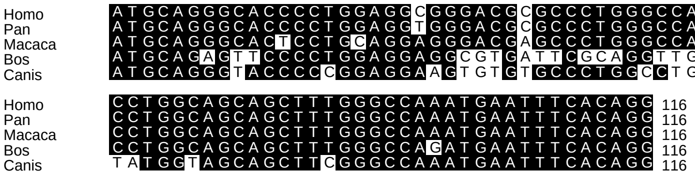

ENSG00000158525 exon 2

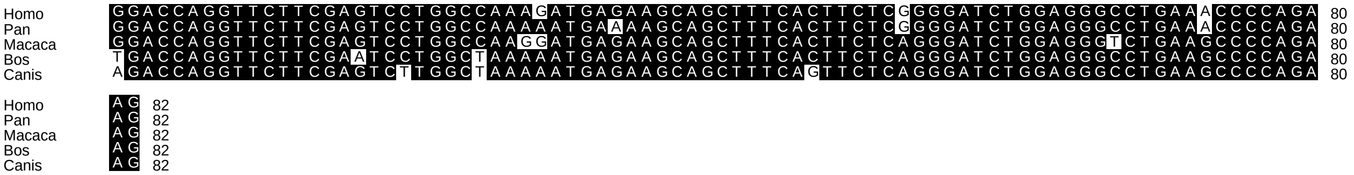

ENSG00000158525 intron 1

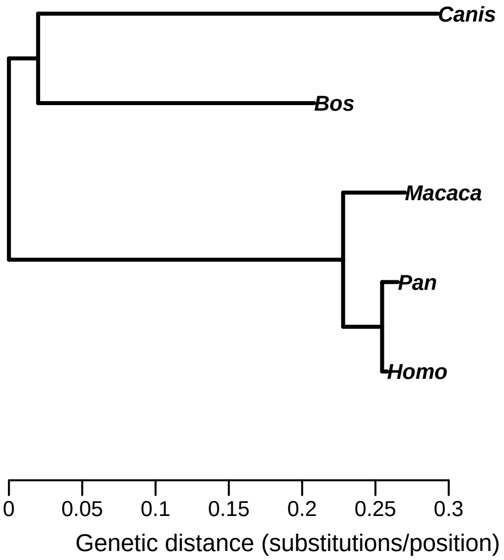

ENSG00000158525 intron 1

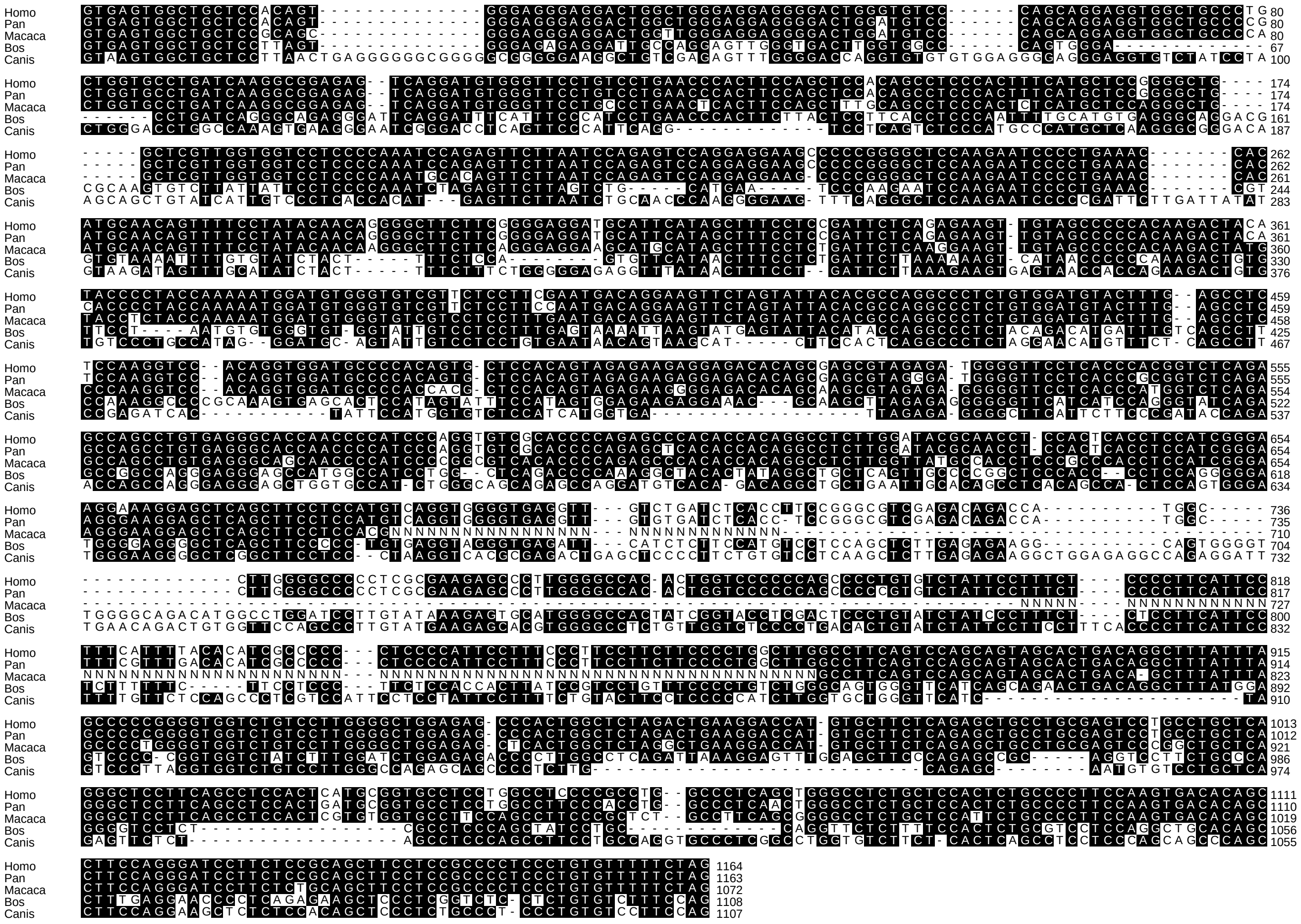

ENSG00000173991 intron 1

Description: Telethonin (TCAP)
Intron number: 1
Human chromosome: 17
Intron start (bp): 35075249
Human intron length : 246
Intron alignment length: 253
Flanking exons length (upstream/downstream): 110/394
SNP density: 0.020325
K tree score: 0.0528
Scaling factor: 0.822
Human-chimpanzee distance: 0.012411
Total primate branch length: 0.0812

ENSG00000173991 exon 1

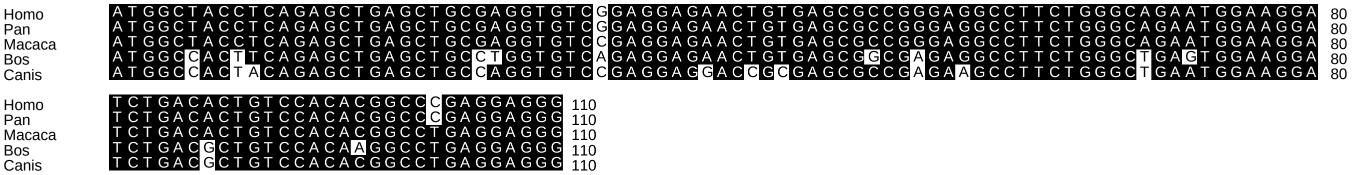

ENSG00000173991 exon 2

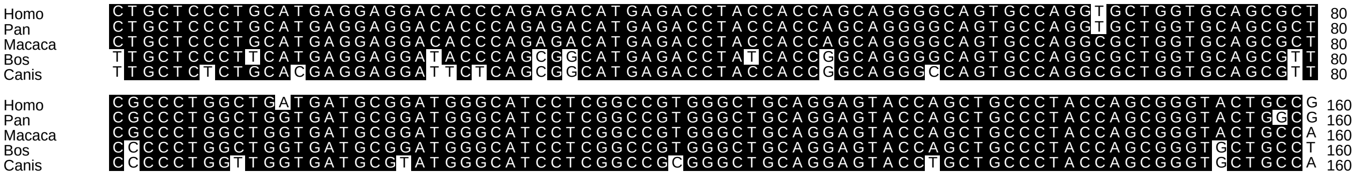

ENSG00000173991 intron 1

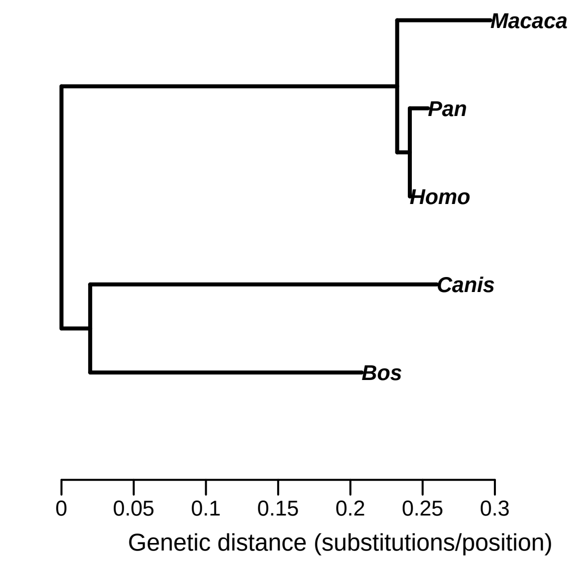

ENSG00000173991 intron 1

```
Homo    GTGAGTGTGGGTCTGCTAGAGCCCTGCCTCTGCTCCCCCAGAGCACCCTCACTGAG--CCATGAGGCCAGAGCATGAAGCCCTGGAGAAATTTCTGGGGG 98
Pan     GTGAGTGTGGGTCTGCTAGAGCCCTGCCTCTGCTCCCCCAGAGCACCCTCACTGAG--CCATGAGGCCAGATCGTGAAGCCCTGGAGAAATTTCTGGGGG 98
Macaca  GTGAGTGTGGGTCTGCCAGAGTCCTGCCTCTGCTCCTCCAAAGCACCCTCACTGAG--CCCTGAGGCCAGAGCATGAAGCCCTGGAGAAATTTCTGGGGG 98
Bos     GTGAGTGCTGGCCAGTCAGGGGT---CCCCCATCCTCTCAGAGCACCTTTCCTGAGACTCATGAGACCAGAGCACAAAGCCTGGGAGAGGTTT-TGGGGG 96
Canis   GTGAGTGCCAGCCGGCTGGGAGT---CCCTC--TCCCCCAGAACACCCCTCCTGAGCCTCAGGAGGCCAGAACACAAAGCCCTGCAGAAGTTTCTGGGGG 95

Homo    TGGGGGCAGGAAGAATGCCCCATGGGGAGAGCAAAGGGGAACCACCCTTCCTGCCCCCAGGTCCCAGCAGCCCAGGGGAGCCCC-CCACCCAGCCTGTGC 197
Pan     TGTGGGCAGGAAGAATGCCCCATGGGGAGAGCAAAGGGGAACCACCCTTCCTGCCCCCAGGTCCCAGCAGCCCAGGGGAGCCCC-CCACCCAGCCTGTGC 197
Macaca  TGGGGGAAGGAAGAATGCCCCATGGGAAGAGCAAAGGGGAACCACCCCTCCCGCTCCCAGGTCCCAGCAGCCCTGGGGAGCCCC-CCACCCAGCCTGTTC 197
Bos     TGGGGGCCAGGAGAGTGCCTGCTGGGGAAAGTGGAGTTGCCCC-------------------TCCAGCAGCCCCAGGGAGCTCTGCTGCCCCTCTGGTGA 177
Canis   TAGGGACAAGGAGAGGGTACCCCTTCTGTATCCCAGGT------------------------CCTGGAAGTCCCAGGGAGCCCC-CTGCCCAGCTCATTA 170

Homo    CCAGAGAGCAACAGCTCCCAGGAGCTCACTGCCCCTCC----CCTCTCCCCAG 246
Pan     CCAGAGAGCAACAGCTCCCAGGAGCTCACTGCCCCTCC----CCTCTCCCCAG 246
Macaca  CCGGAGAGCAACAGCTCCCAAGAACTCACTGCCCCTCC----CCTCTCCCTAG 246
Bos     GTGGCGGGCAGCAGCTCTC--GGGTTCAGCATCCCTTCGCTTCTCCTTCCCAG 228
Canis   GTAGAGGGTAGCAGATTTCAGGAGCTCAGTGTCCCCTCCCGTCTCCTCCCCAG 223
```

ENSG00000106178 intron 2

Description: Small inducible cytokine A24 precursor (CCL24)
Intron number: 2
Human chromosome: 07
Intron start (bp): 75279219
Human intron length : 1341
Intron alignment length: 1861
Flanking exons length (upstream/downstream): 118/169
SNP density: 0.000000
K tree score: 0.0901
Scaling factor: 0.4344
Human-chimpanzee distance: 0.015173
Total primate branch length: 0.0811

ENSG00000106178 exon 2

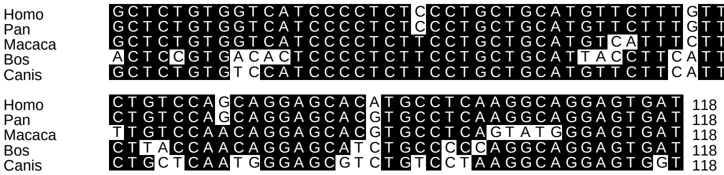

ENSG00000106178 exon 3

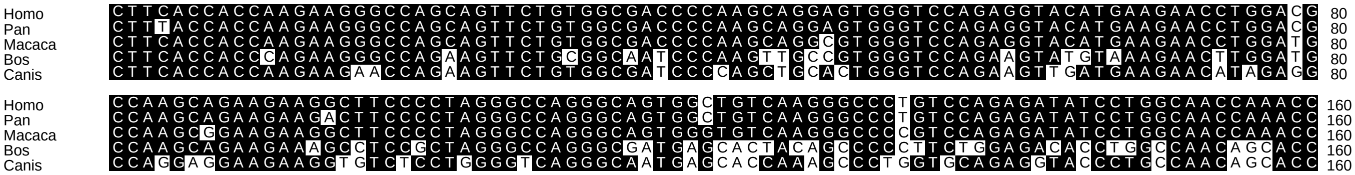

ENSG00000106178 intron 2

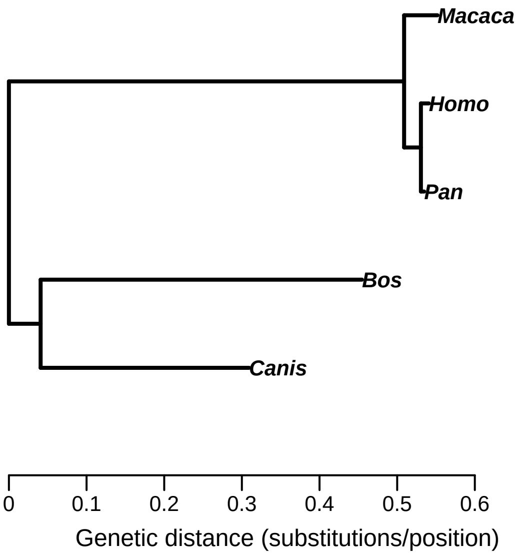

ENSG00000106178 intron 2

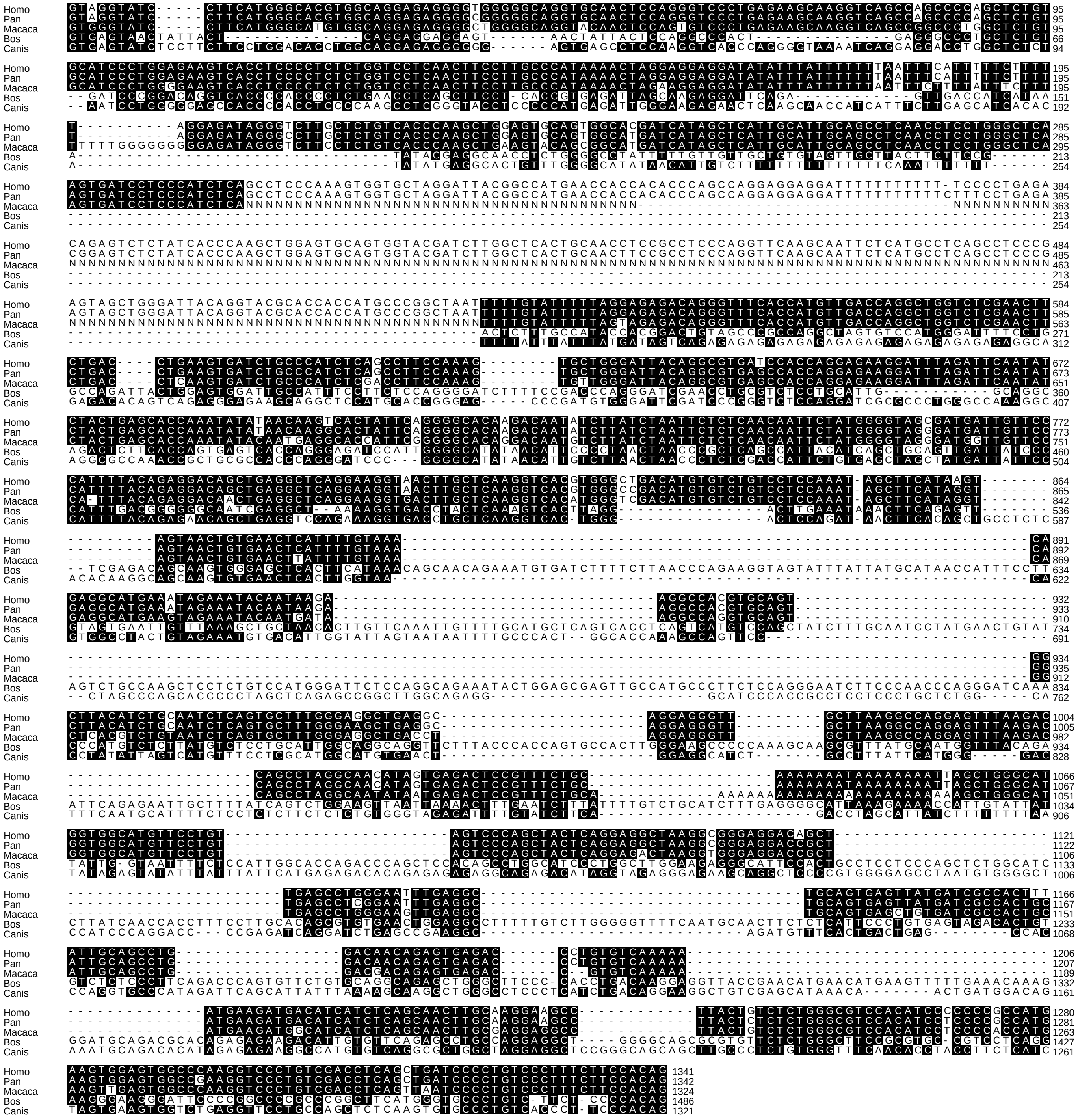

ENSG00000113272 intron 1

Description: interphase cytoplasmic foci protein 45 (THG1L)
Intron number: 1
Human chromosome: 05
Intron start (bp): 157091218
Human intron length : 1236
Intron alignment length: 1281
Flanking exons length (upstream/downstream): 191/177
SNP density: 0.005663
K tree score: 0.0223
Scaling factor: 1.0164
Human-chimpanzee distance: 0.016097
Total primate branch length: 0.081

ENSG00000113272 exon 1

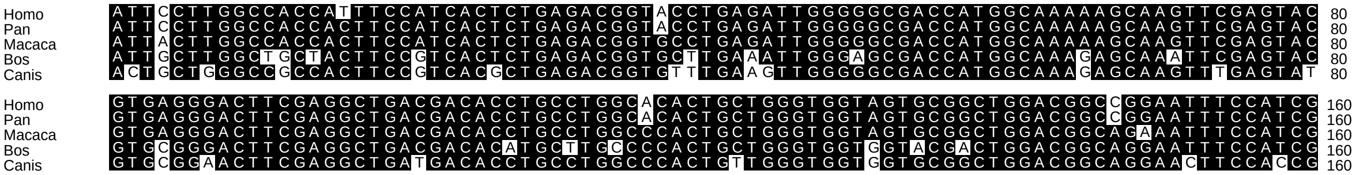

ENSG00000113272 exon 2

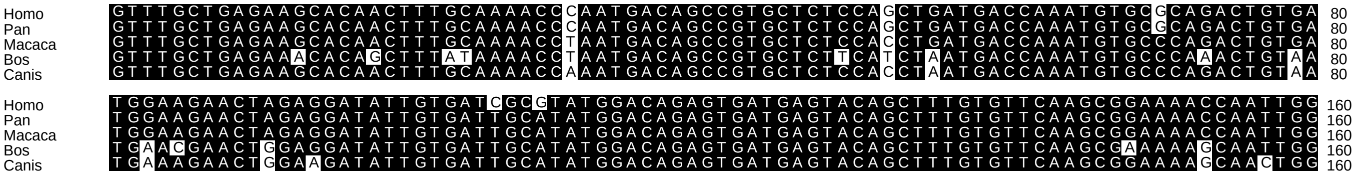

ENSG00000113272 intron 1

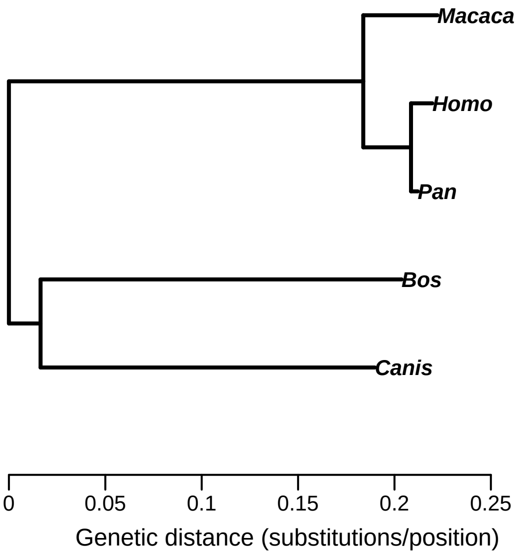

ENSG00000113272 intron 1

```
Homo    GTGAGCGAGCTCGACTCGGGGCGTCGCGATGCGCCAGCGCTTCCGGGGAATCCAGCTTCTTCCCTTGCAAGTCCTCCCGCCCGCTCCAGTCACGGTTACC 100
Pan     GTGAGCGAGCTCGACTCGGGGCGTCGCGATGCGCCAGCGCTTCCGGGGAATCCAGCTTCTTCCCTTGCAAGTCCTCCCGCCCGCTCCAGTCACGGTTACC 100
Macaca  GTGAGCGAGCTCGGCCCGGGGCGTCGCGGTGCGCCAGCGCTTCTGGGAAATCCGGCTTCTACCCTTACAAATCGTCCCGGCAGCCCCAGTCACGGTTACC 100
Bos     GTGAGCGAGCTCGGCCCGGGGTG-CGTGCTTCGCAGGCGTTTCCGGTGAACGCGGCCTCTCC----------GTAGCCGCCCGTAGC--TCGTGGTTACC 87
Canis   GTGAGCGGTTTCGCCTCAGGCCA-CGTGGT--GCCCGCGCTTCCGG--------------------------GGACTCGGTCGCGGC--TGGCGGTTCCC 69

Homo    AGGGTAACGCGGTAGCCAATGAGTGCGCAGCATGGAGCATTGCCCCGCCCTGCGTGTTGGCTAGGACAGTGCCTGAACGCTGG-CTGTTAGCGTAATTGA 199
Pan     AGGGTAACGCGGTAGCCAATGAGTGCGCAGCATGGAGCATTGCCCCGCCCTGCGTGTTAGCTAGGAAAGTGCCTGAACGCTGG-CTGTTAGCGTAATTGA 199
Macaca  GGGGTAACGCGGTAGCCAATGAGTGCGCAGCATGGAGCATCGCCCCGCCCTGCGTGTTGGCTAGGACAGTGCTTGAACGCTGG-CTGTTAGCGTAATTGA 199
Bos     GTGGTAACGTGGCAGCCAATGACTGCGCGGCTTTAAGCAT-------CACTCTGTGCTGGCTACAAC-TGGCTTGAACGCGAG-TAGTCTGGATAGTTAA 178
Canis   G-GGTAACGCGGCAGCCAATGAGCGCGCGGCATTAAGCAT-------CCCCGAGTGCTGGCTACCAG-GCGCTTGAGCGCGAGCTGGTCTGAGTAGCTAA 160

Homo    AT---GGGCGATGTGCTCCCGTGATGAGGATCTGGCTTGGAGTGCCCGTGTTAAGTGAACAGAGACTTGTTAGCAAGGACCTGTTGTTAAAAGTCACTCT 296
Pan     AT---GGGCGATGTGCTCCCA-GATGAGGATCTGGCTTGGAGTGCCCGTGTTAAGTGAACAGAGACTTGTTAGCAAGGACCTGTTGTTAAAAGTCACTCT 295
Macaca  AT---GGGTGACGTGCTCCCCTGATGAGGATCCCGCTTGGAGTGGCCGTGTTAAGTGAACAGAGACCTGTTAGCAAGGACGTGTTGTTAAAAGTCACTCT 296
Bos     AAATGAGACAGGGTGCTCCCCTG-------------------------------GTGACGAGCTGTCCATTGGCAAGGCTTTGGTGGGAGAAGTCACT-C 246
Canis   ACCTGAGGCGCCGCGCTCTC----------------------------------GTGAGCAGAGACCTGTTAGTAAGGCCTTGCTGGGAAAAATCACTCC 226

Homo    CATGCACAATATTTAAGCTCTTAAGCCAATTGCCTAGCTATTCTGTTAGATATCTTTGAATGCTGACTGTTCAGAAGTCTGTGTAAGCCTTGCTTGTGGG 396
Pan     CATGCACAATATTTAAGCTCTTAAGCCAATTGCCTAGCTATTCTGTTAGATATCTTTGAATGCTGACTGTTCAGAAGTCTGTGTAAGCCTTGCTTGTGGG 395
Macaca  CATGCACAGTATTTAAGCTCTTAAGCCAATCGCCTAGCT-TTCTGTTAGATATCTTTGAATGCTGACTGTTCAGAAGTCTGTGTAAGCCTTGCTTGTGGG 395
Bos     TTGAAGCTTTACTTCAGCTCTTCAACCGATGGCCAAACTGGT-TGTTAGGTATCTTTGAG----GG----------------------CTTG-CTGTGGA 318
Canis   CTGGCGCAGTATTTCAGCTCTTTAGCCAATTGCCTGGCTATTCTGTTAACTGTCATCGAG----GGCCGTGTAGAGGCCTGTTTAGGTCTTA-TTGTGGG 321

Homo    CAATACTTAAC-------GTCTGTGCTTCATTATCAGTAACAGGCCACATGGACTAGATTCAACAGAGAGTCAGAAAAAAAGAGAAGGCTGGGCGCGGGA 489
Pan     CAATACTTAAC-------GTCTGTGCTTCATTATCAGTAACAGGCCACATGGACTAGATTCAACAGAGAGTCAGAAAAAAAGAGAAGGCTGGGCGCGGGA 488
Macaca  CAATACTT-AT-------GTCTGTGCTTCATTATCAGTAACAGGCCACATGGATTATTTTCAACAGAGAGTCAGAAAAAAAGAGAAGGCTGGGCGCGGAA 487
Bos     CAACGCTATACAGAGCATATTCTTGTTTCATTATAAGTAACAGGGCACATGGACTGGATTCAGGAGG--------------------------------- 385
Canis   CGATGCTTTAC--AATATGTTCTTGTTTCGTTATCGGTAACTGGCTGCATCGACTGGACTCAGGAGG--------------------------------- 386

Homo    GGCAGGAGGATCACTTAAGGCCAGGAGTTGGAGACCAAG-TTTTTGTTTTTGTTTTGTTTTTT--CTGTGGTTGTTGTTTGTGAAACTCCGCCACTACAA 586
Pan     GGCAGGAGGATCACTTAAGGCCAGGAGTTGGAGACCAAGTTTTTTGTTTTTGTTTTGTTTTTTTGTGGTTGTTGTTTGTGAAACT---CCGCCACTA 585
Macaca  GGCAGGAGGATCACTAAAGGCCAGGAGTTGGAGACCAGG--------------------TTTTTGTTGTTGTTGTTGTTTCTGAAACT---CTGCCACTA 564
Bos     ---------------------------------------------------------------------------------------------------- 385
Canis   ---------------------------------------------------------------------------------------------------- 386

Homo    AAAAAAAAAAAAAAAAAAAAAAAAAAAGAGAGAGAGAGAGAGAAAGAAGGAAGAGAGAAAGAAAGAAAAAG--AAAAAATTTAATTTGCCCGGCGCAGTG 684
Pan     CAAAAAAAAAAAAAAAAAAAA---------------------AGAGAAGGAAGAGAGAAAGAAAGAAAAAG--AAAAAATTTAATTTGCCCGGCGCAGTG 662
Macaca  CAAAAACAGAGAGAGAGAGAA---------------------GGAAAAGCAAGAGAGAAAGAAAGAAAAAGAAAAAAAATTTAATTTGCCAGGCGCAGTG 643
Bos     ------------------------------------------GGAAAAGTCAGAAAAAAACAAA------------------------------------ 407
Canis   ------------------------------------------AGACAAATCAGAAAAAAAAGGGG----------------------------------- 409

Homo    GTGCACACATGTAGTTCCAGCTACTGGGAAGGCTGAGGTGGAAGGATCGCTTGAGCCTGGGAGGTCAAGGCTGCAGTGAGCGGTGATTGGA-CACTGCAC 783
Pan     GTGCACACATGTAGTTCCAGCTACTCGGAAGGCTGAGGTGGAAGGATCGCTTGAGCCTGGGAAGTCAAGGCTGCAGTGAGCGGTGATTGGA-CACTGCAC 761
Macaca  GTGCACACCTATAGTCCCAGCTACTCGGGAGGCTGAGGTGGGAGGATCGCTTGAGCCTGGGAGGTCAAGGCTGCAGTGAGCGGTGATCGGACCATTGAAC 743
Bos     ---------------------------------------------------------------------------------------------------- 407
Canis   ---------------------------------------------------------------------------------------------------- 409

Homo    TCCAGCTTGGGTGACAGAGCAAGACCCTGTCTCAGAAAAAGAGGGAGGGGGAGAAAGAAATATAACAGAAAGATGGGAATTTAATCTTTGAAGAGGGCTG 883
Pan     TCCAGCTTGGGTGACAGAGCAAGACCCTGTCTCAGAAAAAGAGGGAGGGGGAGAAAGAAATATAACAGAAAGATGGGAATTTAATCTTTAAAGAGGGCTG 861
Macaca  TCCAGCTTGGGTGACAGAGCAAGACCCTGTCTCAGAAAAAGAGGGAGGGGGAGAA--AAATACAACAGAAAGATGGGAATTTAATTTTTAAAAAGGGCTG 841
Bos     -------------------------------------------------------------GTAACAGAAAGATGGGAATT---TCTTTAAAAA-TACTA 442
Canis   ---------------------------------------------------------AAGTGTAACGGAAATGTGGGAATTTAATTCTTAAAAA-TGTTA 451

Homo    CATTGTTAATGATTATTATAACTAACATTTATTGAGCACTTACTAGGAGC---TAGGTATGATTACTTTTAT--CTTCTCCCTAATCTTCAGGACAACCC 978
Pan     CATTATTAATGATTATTATAACTAACATTTATTGAGCACTTACTAGGAGC---TAGGTATGATTACTTTTAT--CTTCTCCCTAATCTTCAGGACAACCC 956
Macaca  CATTATTAATGATCATTATAACTAACATTTATTGAGCACTTACTAGGAGC---TAGATATGATTGCTTTTAT--CTTCCCTCTAATCTTCAGGACAACCC 936
Bos     CTTTGGTAATGATTACTGTAGTTAAAATTTATTGAGCA-GTCCAAGGAGC---CAGGCATAGTTACGTTTGTTACCTCCCTCTAACCTGCAGGACAATCC 538
Canis   CTTTAATAATGGTTATTATAACTAACATTTATTGAGCACTTTCATGGAGCCTGCTGGCATAGTTACATTT-TAACTTCTTTCTAATCTTCAGAACAGTCC 550

Homo    AATGAGGTAAATATTCTTTTATTCCCATTTCACAGAAAGGAGAGTTGAGGCTTAGCATTTCTGTCTGGCTTTCTCAATT--TCAGGGC---------AGG 1067
Pan     AATGAGGTAAATATTCTTTTATTCCCATTTCACAGAAAGGAGAGTTGAGGCTTAGCATTTATGTCTGGTTTTCTCAATT--TCAGGGC---------AGG 1045
Macaca  AATGAGGTAAATATTCTTTT-TTCCCATTTCACAGAAAGGAGAGTTGAGGCTTAGCATTTATGTCTGTTTTTCTCAATT--TCAGGGC---------AGG 1024
Bos     TATGAAGTAAA-----TTTA-TCCCCATTTCACAGAAAAGGGAATGGAGGATTAGTATATATGTTCCTTTTTCTCAGTTGGTCAGAGCTAAAACAAAAAA 632
Canis   TATGAAGTAGGTATACTTTA-TCTCCATTTCACAGAAAAGGGAATAGGGACTTAGAAATTATGCCCCTTCTTCTCAGTTGGTCAGGGC----------AA 639

Homo    -AAAAAAACTAAGTTCTTAAGAAAATGGATCAGGTTGTGGCTAGTTGAGCATTTGAGTTCACAGTGCTATTACATTATCTTCCTCCA--AACAGCCTCTG 1164
Pan     -AAAAAAACTAAGTTCTTAAGAAAATGGATCAGGTTGTGGCTAGTTGAGCATTTGGGTTCAGAGTGCTATTACATTATCTTCCTCCA--AACAGCCTCTG 1142
Macaca  AAAAAAAACTAAGTTCTTAAGGAAATGGGTCAGGTTGTGGCTAGTTGAGCATTTGGGTTCACAGTGCTGTTACATTATCTTCCTCCA--AACAGCCTCTG 1122
Bos     CAAAAAACTGGAGTTCTTAACGAAATGGATTGGGTTGTGGCTCCTGGAATGTTTGGACTCTCAGCACTATTACAGTGTCTTCCTTTATGAACAGCCTATT 732
Canis   GAAAAAACTAGAGTTCTTAAGGAAGTGAGTCAGGTTGTGGCT---------AGTAGACTCACAGCATTATTAC-CTATCTTCCATTGTGTGCATTCTTTT 729

Homo    TTATCTAGCAACAGAGCGTGTTGTTAATGACAGG----CTTCCATCCATGCTTTCTTCCCTT-----TTTCCCCTGTGAAG 1236
Pan     TTATCTAGCAACAGAGCGTGTTGTTAATGACAGG----CTTCCATCCATGCTTTCTTCCCTTTTCTCTTTCCCCTGTGAAG 1219
Macaca  TTATCTAGCAACAGAGCTTGTTGTTAATGACAGG----CTTCCATCCATGCTTTCTTCCCTTTTCTCTTCCCCCTACGAAG 1199
Bos     TAATCTG------------TGTATTAATGACAAAGTCTCTTGCAGCCATGCCCTTTTCCCTTTTCTTTTTCCCATTTGAAG 801
Canis   TGATAAAA---------GTGGTGTTAATGACAAG--CTCTTTCATTCATGCCTTTTTCTCCTC-----------TATGGAG 788
```

ENSG00000118271 intron 1

Description: Transthyretin precursor (TTR)
Intron number: 1
Human chromosome: 18
Intron start (bp): 27425933
Human intron length : 924
Intron alignment length: 1583
Flanking exons length (upstream/downstream): 69/131
SNP density: 0.003247
K tree score: 0.0818
Scaling factor: 0.9404
Human-chimpanzee distance: 0.008832
Total primate branch length: 0.081

ENSG00000118271 exon 1

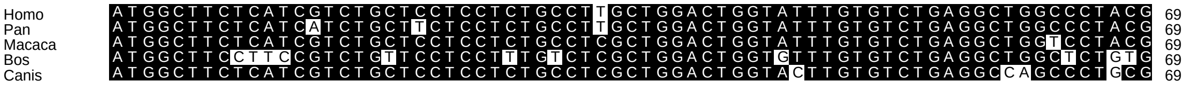

ENSG00000118271 exon 2

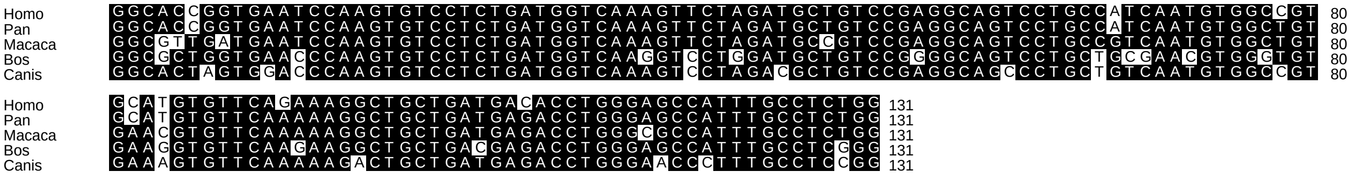

ENSG00000118271 intron 1

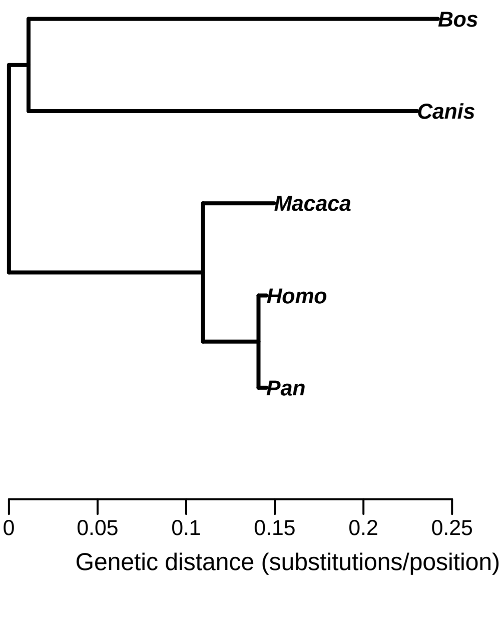

ENSG00000118271 intron 1

```
Homo    GTGAGTGT----TTCTGTGACATCCCATTCCTACATTTAAGATTCACGCTAAATGAAGTAGAAGTGACTCCTTCCAGCTTTGCCAACCAGCTTTTATTAC 96
Pan     GTGAGTGT----TTCTGTGACATCCCATTCCTACATTTAAGATTCACGCTAAATGAAGTAGAAGTGACTCCTTCCAGCTTTGCCAACCAGCTTTTGTTAC 96
Macaca  GTGAGTGT----TTCTGTGACATCTCGCTCCTACATTTAAAATGCACGCTAAATGAGGTAGGAGTGACTCCTTCCAGCTTTGCCAAGCAGCTTTTGTTAC 96
Bos     GTGAGTGTCTGACTCTCTGATGTTCT-TTCATCTGTCTCCTATG-------GATGAAGTATAAGTGACTTCTTCCTGCTTTGCCAACCCACTTTTGTTAC 92
Canis   GTGAGTGTTTGATTCTGTGAAGTCCT-TTTGTATGATCCTTATGTACACTAGTTGAAGTGGAAGTGACTTCTTTTGGCTCTGCCAGCTGGCTTTTGTTGC 99

Homo    TAGGGCAAGGGTACCCAGCATCTATTTTT-AATATAATTAATTCAAACTTCAAAAAGAATGAAGTTCCACTGAGCTTACTGAGCTGGGACTTGAACTCTG 195
Pan     TAGGGCAAGGGTACCCAGCATCTATTTTT-AATATAATTAATTCAAACTTCAAAAAGAATGAAGTTCCACTGAGCTTACTGAGCTGGGACTTGAACTCTG 195
Macaca  TAGGGCAAGGGTACCCAGCATCTATTTTT-AATATCATTTATTCAAACTTCAAAAAGAATGAAGTTCCACTGAGCTTACTGAGCTGGGACTTGAACTCTG 195
Bos     TGGGGCCAGGGCAGCCGGCATCTATTTTTAAATGTCATTGATCCAAACTTCAAAAAGAATGAAGTTCTGCAGAGCTTACTAAACTGGGGCTCAGATCCTG 192
Canis   TAGGACAAGGGTACCCAGCATCAACTCTTAAATCTCATTGATGCAAACCTCAGACTGAATCAAGTTCTGCAGAGCTCACTGAACTGAGGCTTGGACCCTG 199

Homo    AGCATTCTACCTCATTGCTTTGGTGCATTAGGTTTGTAATATCTGGTAC-----CTCTGTTTCCTCAGATAGATGATAGAAATAAAGATATGATATTAAG 290
Pan     AGCATTCTACCTCATTGCTTTGGTGCATTAGGTTTGTAATATCTGGTAC-----CTCTGTTTCCTCAGATAGATGATAGAAATAAAGATATGATATTAAG 290
Macaca  GGGATTCTGCCTCATTGCTTTGGTGCATTGGGTTTGTAATGTCTGGTAT-----CTCCACTTCCTCAGATCGATGATAGAGATAAAGATATGATATTAAG 290
Bos     GGCGCCCTCCTCTATGACTTTGCAG------------------TGGTGC-----CTCAGTTCCCTCAGATGAATGGTAGAAATAAAGATCTGATATTCTG 269
Canis   GGTGCTCCCTGCTCTTGCTTTGGTGCCCTGGCTTTACAGTGCCTGGCTCCTCCACTCCATTCCCTCAGCGAGATGGTAGAACTAAAGGTGTGATATTCAT 299

Homo    GAAGCTGTTAATACTGAATTTTCAGAAAAGTATCCCTCCATAAAATGTATTTGGGGGACAAACTGCAGGAGATTATATTCTGGCCCTATAGTTATTCAAA 390
Pan     GAAGCTGTTAATACTGAATTTTCAGAAAAGTATCCCTCCATAAAATGTATTTGGGGGGCAAACTGCAGGAGATTATATTCTGGCCCTATGGTTATTCAAA 390
Macaca  GAAGCTGTTAATCCTGAATTTTCAGAAAAGTATCCCTTTATAAAATGTATTTGGGGGGCAAACTGCATGAGATTATATTCTGGCCCTATAGCTAATCAAA 390
Bos     GAAGCTGTGACTACTGAATTTTCAGAAGCTTGACCTTTGATAAAGCTAATTT-------------------------------CCCTTTAGCTAATCAAA 338
Canis   GAAGCCACCGATATTATGTTTTCAGAAATGCATCCTTTCATAAAGCTTGTTT-------------------------------CTTTTCAGCTAATCTAA 368

Homo    ACGTATTTATTGATTAATCTTTAAAAGGCTTAGTGAACAATATTCTAGTCAGATATCTAATTCTTAAATCCTCTAGAAGAATTAAC-------------- 476
Pan     ACGTATTTATTGATTAATCTTTAAAAGGCTTAGTGAACAATATTCTAGTCAGATATCTAATTCTTAAATCCTCTAGAAGAATTAAC-------------- 476
Macaca  ATGTATTTACCGATTAATCTTTAAAAGGCTTAGTGAACAATATTTTAGTCAGATATCTAATTCTTAAATACTCTAGAAGGATTAAC-------------- 476
Bos     AAGTATGTACCGATTAATTCTTCAAAGGCTTGGTGCACAACCTTCTAGTCCAATATCTAATTCTTAAATCCCCTAGGACAGTGGACCCCAACCTTTTTGG 438
Canis   AGGTATTGATTGATTGATTTTTGCAAGGCTTGGCAGACACTGTTCTAATCAAATACCTAATTCTTAAGTCCTCTAAAAGAATTAAC-------------- 454

Homo    ---------------------------------------------------------------------------------------------------- 476
Pan     ---------------------------------------------------------------------------------------------------- 476
Macaca  ---------------------------------------------------------------------------------------------------- 476
Bos     CACCAGGAACTGGTTTCACGGAAGACAATTTTTCCACAGACTGGAAGGTGGGGGTTGGGGGATGGTTGGGGGATGATTTAAGAGTATTATATTTATCGTG 538
Canis   ---------------------------------------------------------------------------------------------------- 454

Homo    ----------------------------------------------------------------------------------------TAATACTATAAA 488
Pan     ----------------------------------------------------------------------------------------TAATACTATAAA 488
Macaca  ----------------------------------------------------------------------------------------TAAT-CTATAAA 487
Bos     CACTTTATTTCTATCATTATAACATCAGCTCCACCTCAGATCATCAGGCATTAGATCCCAGAAGTTGGGGATGCTTGGCTTAGAAGAATAAAACTGTGAA 638
Canis   ----------------------------------------------------------------------------------------TCATACTATAAA 466

Homo    ATGGGTCTGGATGTAGTTCTGACATTATTTTATAACAACTGGTAAGAGGGAGTGACTATAGCAACA----ACTAAAATGATCTCAGGAAAACCTG-TT-- 581
Pan     ATGGGTCTGGATGCAGTTCTGACATTATTTTATAACAACTGGTAAGAGGGAGTGACTATAGCAACA----ACTAAAATGATCTCAGGAAAACCTG-TT-- 581
Macaca  ATGGGTCTGGATATAG-TCTGACATAATTTTATAACAACCGGTAAGAGGGAGTGACTAGAGCAACA----ACTAAAATCATCTCAGGAAAAACTGTTT-- 580
Bos     AAGGATCATGAAATCA-TCTGGCTTTATTTTACAGCAACTAGCAAGAAGGAACTGCTGAAGCAACAGCTGAGTAAAATGTTCTCAGGAAAAGCTATTTGG 737
Canis   ATGGCCCATGAAACAG-CCTGACATTATTTT---GCAGCCAGTGAGAGGGAATTACTACAGCAACAGCCGACTACAATGCTCTCGGGGAAAAACTAAC-- 560

Homo    ---------------------------------------------------------------------------------------------------- 581
Pan     ---------------------------------------------------------------------------------------------------- 581
Macaca  ---------------------------------------------------------------------------------------------------- 580
Bos     TCCTGTGCTATACTTAGTCACTCAGTTGTGTCCGACTCTTTGGGACCCCATGGACCATAGCCCACCAGACTCCTCTGTCCATGGGATTCTCCTAGCAAGA 837
Canis   ---------------------------------------------------------------------------------------------------- 560

Homo    ---------------------------------------------------------------------------------------------------- 581
Pan     ---------------------------------------------------------------------------------------------------- 581
Macaca  ---------------------------------------------------------------------------------------------------- 580
Bos     ATATCGGAGTGGGTTGCCATGCCCTCCTCCAGGGGATCTTCCCGACTCAGGGATCGAACCCAGGTCTCCTGTGCTGCAGGTGGCTTCTTTACCAGTTGAG 937
Canis   ---------------------------------------------------------------------------------------------------- 560

Homo    ----------------TGGCCCTATGTATGGTACATTACATCTTTTCAGTAATTCCACTCAAATGGAGACTTTTAACAAAGCAACTGTTCTCAGGGGACC 665
Pan     ----------------TGGCCCTATGTATGGTACATTACATCTTTTCAGTAATTCCACTCAAATGGAGACTTTTAACAAAGCAACTGTTCTCAGGGG-TC 664
Macaca  ----------------TGGTCCTATGTATGGTACGTTACATCTTTTCAGTAATTCCATTCAAATGGAGAAGTTTAACAAGGCAACTGTTCTCAGGGGGCC 664
Bos     CTACCAGGGAAGCTCCTATTTGGTCCTGTGGCATATTATATCTTTGCAGTAACTCCACTCAGATGAGGAGGCTTAACAAAACCATTCTTTTCAGAGGGAC 1037
Canis   ----------------TATTTGGTCCTATGGCACATTATACCTTTTCAGTAATTCCACTCAAATAGTGAGGCTTAACAAAGCAACTCTTCTCAAAGGGCC 644

Homo    TATTTTCTCCCTTAAAATTCATTATACACATCCCTGGTTGATAGCAGTGTGTCTGGAGGCAGAAACCATTCTTGCTTTGGAAACAATTACGTCTGTGTTA 765
Pan     TATTTTCTCCCTTAAAATTCATTATACACATCCCTGGTTGATAGCAGTGTGTCTGGAGGCAGAAACCATTCTTGCTTTGGAAACAATTATGTCTGTGTTA 764
Macaca  TATTCTCTCCCTTAAAATTCATTATACACATCCCTGGTTGATAGCAGTGTGTCTGGAGGCAGAAACCATTCTTGCTTTGGAAACAATTATGTCTGTGTTA 764
Bos     CATTATCTCCCTTCAAGTTCATAATACACAACCCCAGTAGAGCACAGCATGTCCGGAGGCAGAAACTATTCTTTCTTTGGAGACAATTGTGTCTGTGTTA 1137
Canis   TACTTTCTCCCTTAAAATTCATTATACACGTCCCCGGTCAATTGTAGCGTGTCTGGAGACAGAAATCAGCTTTGCTGTGGAAGCAATCATGTCTG-GTCA 743

Homo    TACTGAGT-AGGGAAGCTCATTAATTGTCGACACTTACGTTCCTGATAATGGGATCAGTGTGTAATTCTTGTTTCGCTCCAGATTTCTAATACCACAAAG 864
Pan     TACTGAGT-AGGGAAGCTCATTAATTGCCGACACTTACGTTCCTGATAATGGGATCAGTGTGTAATTCTTGTTTCGCTCCAGATTTCTAATACCATAAAG 863
Macaca  TACTGAGT-AGGGAAGCTCATTAACTGTCAACACTTATGTTCTTCATAATGGAATCAGTGTGTAATTCTTGTTTTGTTCCAGATTTCTAACACCATAAAG 863
Bos     CACTGAGTAAGGGAAGTTCATTAACCGTCAACCCATCTGTCGCTGAAAATGGGATTCTTGCGTCCCTGCTGCCGTGTTCCTGGTCTC--ACACCACAAAG 1235
Canis   CACTGAGA-AGGGGAGCTCATTAACTATCAACACTTGCATTGCTGGAAATGGAATTGGCATGTACTTTTTACCTTGTTCCAGATTTCTAGCACCATAAAG 842

Homo    AATAAATCCTTTCA-------------------------------------------------------------------------------------- 878
Pan     AATAAATCCTTTCA-------------------------------------------------------------------------------------- 877
Macaca  AATAAATCCTTTCA-------------------------------------------------------------------------------------- 877
Bos     GATGCAGCCTGTGG-------------------------------------------------------------------------------------- 1249
Canis   AACAGATCCTTTTAGGGGCACCTGGATAGCTCAGTTGGTTAAGCATCTGCCTTCGGCTCGGGTCATGGTCCTGAGGTCTTGAGATCAAGTCCCACATCGG 942

Homo    ---------------------------------------------------------------------------------------------------- 878
Pan     ---------------------------------------------------------------------------------------------------- 877
Macaca  ---------------------------------------------------------------------------------------------------- 877
Bos     ---------------------------------------------------------------------------------------------------- 1249
Canis   GCTCCCTGCTCCACGGGAAGCCTGCTTCTCCCTCTCCCTCTGCCTGTCACTCCCCCAGGTTGTGCTCTCCCTCCTTCTGTCAAATAAATAAATGAAATCT 1042

Homo    -------------------------------------CTCTGATCAATTTTGTTAACTTCTCACGTGTCTTCTCTACACCCAG 924
Pan     -------------------------------------CTCTGATCAATTTTGTTAACTTCTCACGTGTCTTCTCTACACCCAG 923
Macaca  -------------------------------------CTCTGATCAATGTTGTTAACTTCTCACTTGTCTTCTCTATACCCAG 923
Bos     -------------------------------------CTCTGACCAACTTTATTGACTTGTCACTTGTCTTCTCTCCACCCAG 1295
Canis   GAAAATTTTTTAAAAATGAAAAGAATAGATCCTTTCCCTCTTATCACTTTTGTTGACCTCTTTATGGCCTTCTCTATGCCCAG 1125
```

ENSG00000131323 intron 1

Description: TNF receptor-associated factor 3 (TRAF3)
Intron number: 1
Human chromosome: 14
Intron start (bp): 102406537
Human intron length : 1470
Intron alignment length: 2420
Flanking exons length (upstream/downstream): 245/52
SNP density: 0.002041
K tree score: 0.0989
Scaling factor: 0.5556
Human-chimpanzee distance: 0.007557
Total primate branch length: 0.081

ENSG00000131323 exon 1

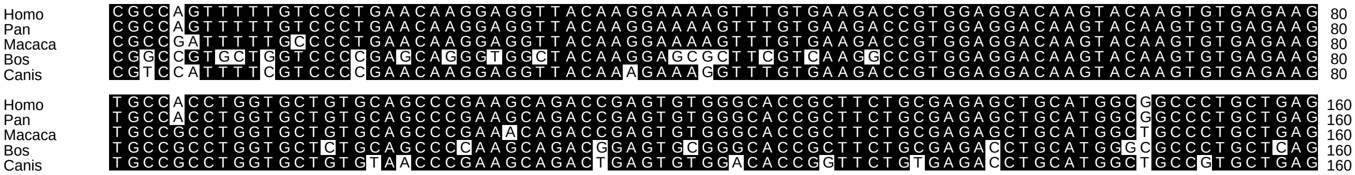

ENSG00000131323 exon 2

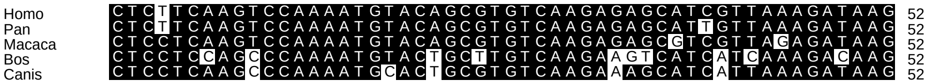

ENSG00000131323 intron 1

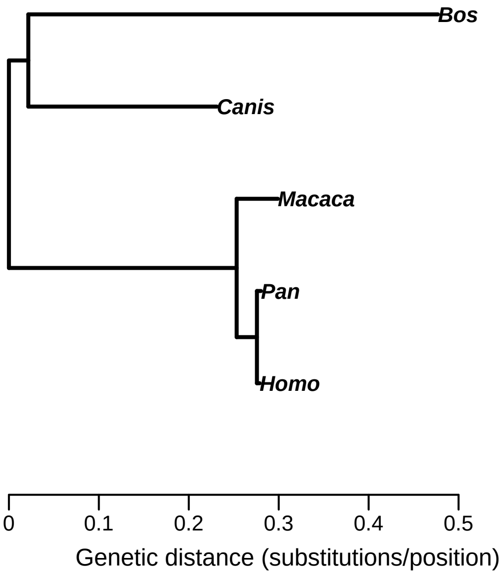

ENSG00000131323 intron 1

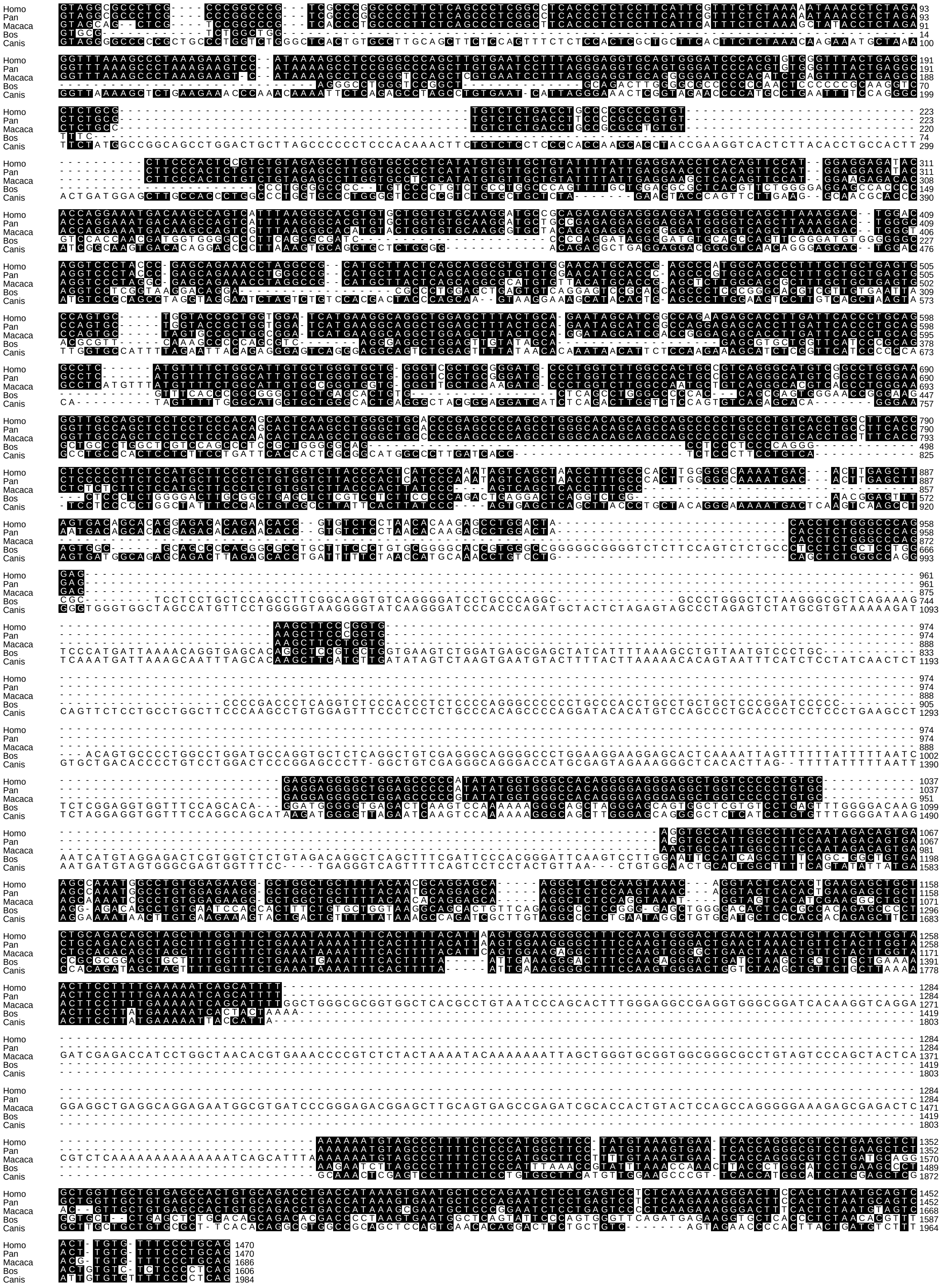

ENSG00000110455 intron 3

Description: 1-aminocyclopropane-1-carboxylate synthase (ACCS)
Intron number: 3
Human chromosome: 11
Intron start (bp): 44051644
Human intron length : 1094
Intron alignment length: 1270
Flanking exons length (upstream/downstream): 71/70
SNP density: 0.001828
K tree score: 0.0693
Scaling factor: 0.9765
Human-chimpanzee distance: 0.012925
Total primate branch length: 0.0809

ENSG00000110455 exon 3

```
Homo    CTGAGTCAGCGCGACATGCAGAGGGTGGAGCCATCCCTGCTGCAGTATGCTGACTGGAGGGGACATCTGTT  71
Pan     CTGAGTCAGAGCGACATGCAGAGGGTGGAGCCATCCCTGCTGCAGTATGCTGACTGGAGGGGACATCTGTT  71
Macaca  CTGAGTCAGAGCGACATGCAGAGAGTGGAGCCGTCCCTGCTGCAGTACGCTGACTGGAGGGGACATCTGTT  71
Bos     CTGAGTCAGAGTGACATGCTGCAGGTGGAGCCGGCCCTGTTGCAGTACCCCGACTGGAGGGGACATCTGTT  71
Canis   CTGAGTCAGAGTGACATGCTGCGGGTGGAGCCATCATTGCTGCAGTACCCTGACTGGAGGGGACATCTGTT  71
```

ENSG00000110455 exon 4

```
Homo    CCTCCGGGAGGAAGTGGCCAAGTTCCTGTCTTTCTACTGCAAGAGCCCAGTACCCCTCAGACCAGAGAAT  70
Pan     CCTCCGGGAGGAAGTGGCCAAGTTCCTGTCTTTCTACTGCAAGAGCCCAGTACCCCTCAGACCAGAGAAT  70
Macaca  CCTCCGGGAGGAAGTGGCCAAGTTCCTGTCTTTCTACTGCAAGAGCCCAGCGCCACTGAGACCAGAGAAT  70
Bos     CCTCCGGGAGGAAGTGGCCAGGTTCCTATCTTTCTACTGCAGAAGCCCCGCACCCCTTAAACCGGAGAAT  70
Canis   TCTCCGGGAGGAAGTGGCCAGATTCCTGTCTTTCTACTGCAAGAGCCCTGCACCCCTTAAACCAGAGAAC  70
```

ENSG00000110455 intron 3

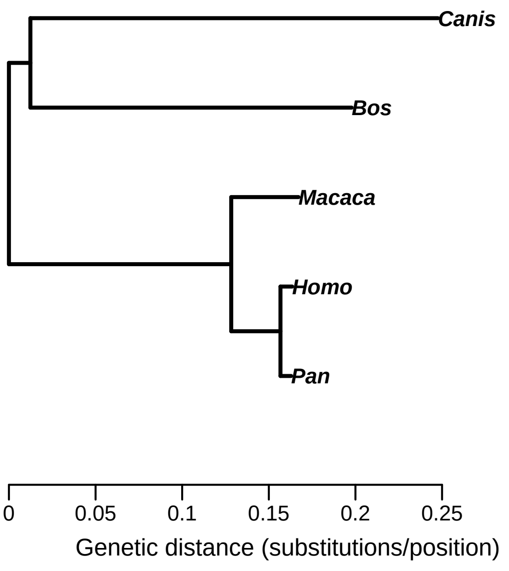

ENSG00000110455 intron 3

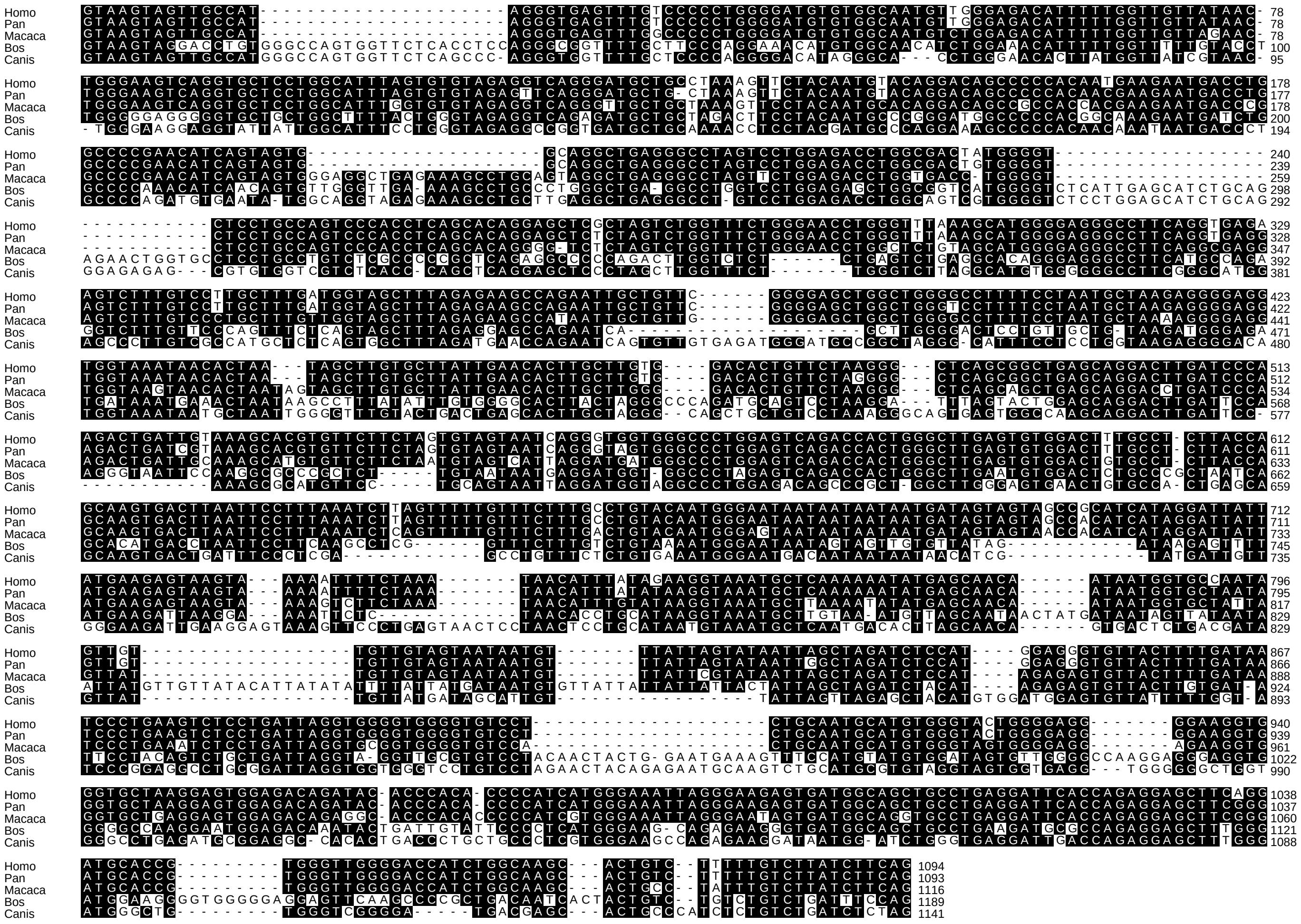

ENSG00000103042 intron 8

Description: amino acid transporter (SLC38A7)
Intron number: 8
Human chromosome: 16
Intron start (bp): 57261573
Human intron length : 877
Intron alignment length: 945
Flanking exons length (upstream/downstream): 200/55
SNP density: 0.002280
K tree score: 0.0565
Scaling factor: 0.8852
Human-chimpanzee distance: 0.009245
Total primate branch length: 0.0809

ENSG00000103042 exon 8

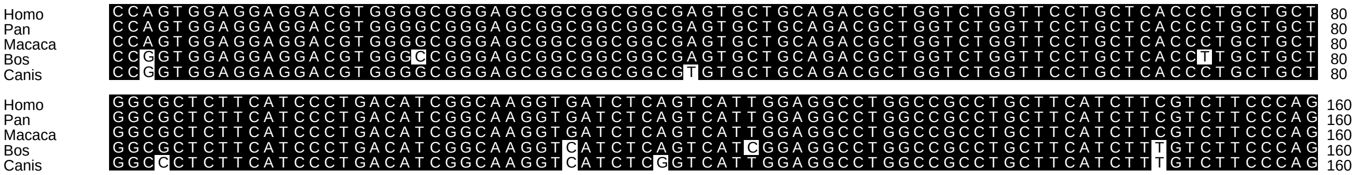

ENSG00000103042 exon 9

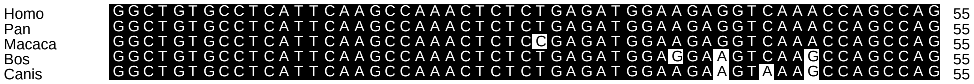

ENSG00000103042 intron 8

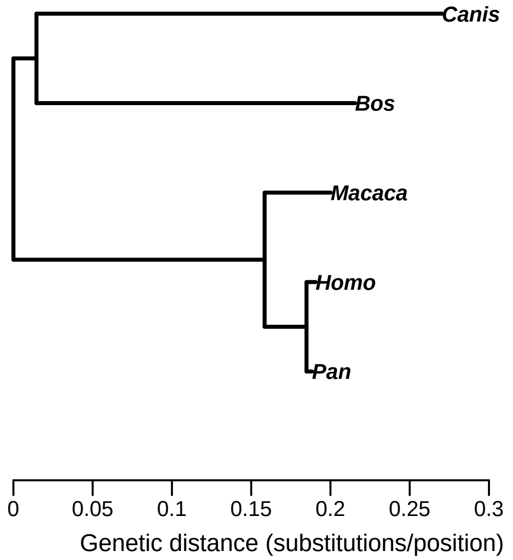

ENSG00000103042 intron 8

```
Homo    GTGCGGACCCCTGGCGCCCCATCTCACAGCCCCACAGCACAGACTCCCAGGGCCTGCCTAGCCTCAGCTCCCTACTTGCTCAAAACATGCAGGAGAGAGT 100
Pan     GTGCGGACCCCTGGCGCCCCATCTCACAGCCCCACAGCACAGACTCCCAGGGCCTGCCTAGCCTCAGCTCCCTACTTGCTCAAAACATGCAGGAGAGAGT 100
Macaca  GTGCGGACCCCTGGCGCCCCA--TCACAGCCCCACAGCACAGACTCCCAGGGCCTCCCTAGCCTCTGCTCCCTACTTAATCCAAACATGCAGGAGAGAGT 98
Bos     GTGTGGACCCCCGGTGT------------CCCCACAGCACTCACTCCCAGACCCT-CCTGGCCTCAGCTCCTGTCTTGCTC---ACCTGCGGGGGAGAAT 84
Canis   GTGGGCACTCCCGGTAC------------CCCCACAGCATTGGCCCCCAGGCTCT-CCCAGCCTCAGTACCTCCCTTGCTT---TCCTGCCTGA-AGAAC 83

Homo    AACCACCAGCACTAATTGATCACCTG-TCTCGCCTCTGTACTAGCCCCTATCCAAGCATTATCT-----------------------------------C 164
Pan     AACCGCCAGCACTAATTGATCACCTG-CCTCGCCTCTGTACTAGCCCCTATCCAAGCATTATCT-----------------------------------C 164
Macaca  AACCACCATCACTGACTAATCACCTG-CCTCGCCTCTGTGATGGCCCCTGTCCAAGCATTATCTCCTGGAAAGAAGGTGTAGTTGTGATCTCCAGTTTAC 197
Bos     AATCACCAGCATTTATCAATCACCT---TCAGGCTCTGTGGGAACCCCTGTTTGAGCATCACCT-----------------------------------C 146
Canis   TACCACTAGTGCTTATTAATCACCCGACTTAGGCTCTGTGCTGATCCCTGCCC---------CT-----------------------------------C 139

Homo    CTGGAAAGAAGGTATAGTTGTGATCTCCAGTTTAC---TGATGGCACGTGGGGCTCAGAAAGGGTCACAGACTTGCCTAGAGCTTAACAAACTCAACTAG 261
Pan     CTGGAAAGAAGGTATAGTTGTGATCTCCAGTTTAC---TGATGGCACGTGGGGCTCAGAAAGGGTCACAGACTTGCCTAGAGCTTAACAAACTCAACTAG 261
Macaca  CTGGAAAGAAGGTGTAGTTGTGATCTCCAGTTTAC---TGATGGCACGTGGGGCTCAGAAAGGGTCACAGACTTGCCTAGAGCTTAACAAACTCGACTAG 294
Bos     CTACAGAGAAGGTGTAATTGTCATCTCCATTTTACCCATGAGGAGGTGTGAGG-TCAGAGAGAGTAACAAACTTCCCTAGAGGTTAACAAACTCAACTAA 245
Canis   CTGAAAAGAAGCTACAGGTGTTATCTCCACTTTGC---TGGTGAGATGGGAGG---------------------------AAGTTAACATACTCAAGTAT 209

Homo    T----AA------CCTTTTCCATCCCACGCTGTCTGGGTCCAGAGTCTATTC--TTTTAACCATTCATTCAGGTGTCTGCTGCTCCTTGACTATGAGGGA 349
Pan     TAACAAA------CCTTTTCCATCCCACGCTGTCTGGGTCCAGAGTCTATTC--TTTTAACCATTCATTCAGGTGTCTGCTGCTCTTTGACTATGAGGGA 353
Macaca  TAACAAA------CCTTTTCCATCCCACACTGTCTGGGTTCAGAGTCTGTTC--TCTTAACCATTCATTCAGGTGTCTGCTGCTCCCTAAC--TGAGGGA 384
Bos     AGAAGAATAGAATCTTTTCAAACTGAAGTCTGTCCAGATCCACAGTCTATATAGTCTTAACTCTTATTTCAAGAACCTGTTCCTCCATGACTGTCAGGGA 345
Canis   TGAGTAACAG---CTTTTCTGATGCAGGTCTGTCTGGGTGTGCAGCCTGCAC--TCATCACCTTTCATGCACATACTTGTTGCTGCCTGACTGTGAGGGG 304

Homo    TCTATCTGGCTGGTTCAGCCCATTACAGCCCAGAGGGCAGTGGGGTCT-GGGGTGGCACCCTGACACAGACATCAGGGAACCTGGGTTCCAGACACAGAT 448
Pan     TCTATCTGGCTGGTTCAGCCCATTATAGCCCAGAGGGCAGTGGGGTCT-GGGGTGGCACCCTGACACAGACATCAGGGAACCTGGGTTCCAGACACAGAT 452
Macaca  TCTGTCTGGCTGGTTCAGCCCATTACAGCCCAGAGAGCAGTAGGGTCTAGGGGTGACAGCCTGACACAGACATCAGAGGACCTGGGTTCCAGACACAGAT 484
Bos     ----TCTGGCTGGGTCAGCCCACGACAG------GGTAATTAGGGTCTGGCAGTGGCACCCTGACACGGAGGTTGGGAGACTTGAGTTTCAGACA---GT 432
Canis   ----TTGAGCTGGTTCAGCCCATTATAGGCAAGACGGCCATGGGGTCT--------------GGTGCAGAAGTCGGGAGACCTGTGTTTCAGACACGATT 386

Homo    CTACCGCCAGATCCCTGGAGAACT-GTCCTGTCTGCCCAGCCATGAGTTCCACCCTTTTCCAGCTTGTCTCTATCCAGGAAGCCACCCTGAAAGCCCCAG 547
Pan     CTACCGCCAGATCCCTGGAGAACT-GTCCTGTCTGCCCAGCCATGAGTTCCACCCTTTTCCAGCTTGTCTCTATCCAGGAAGCCACCCTCAAAGCCCCAG 551
Macaca  CTACCACCAGATCCCTGGAGAAGT-GTCCTGTTGGCCCAGCCATGAGTTCTACCCTTACCCAACTTGTCTTTATCCAGGAAGCCACCCTCAAAGCTCCAG 583
Bos     CTGCCACCGGATCCCTGNNNNNNN-NNNNNNNNNNNNNAGTCACGAGTTCTGCCTATACCCAGCTTATTTCTACCCAAGAGGCCAGCTGGAAGGCCCTTG 531
Canis   CTGCCGCCAGATCTCCAAGGAAGTGGTCTCGTGTGCCCAGCCACGAGTTCCGCCTATACCCAGCTTATTCCTCTCCAAGAGGCCAGCTTGAAAGCTGCTG 486

Homo    CTGCTTTGCTTGGAGCAGGGGAGCCAGATCTGCTGGGAAGTGTACTAGGAAGACCCCTGGGGT----TGGGCCACCACTGAGTCACCATTGAGTGCTGTC 643
Pan     CTGCTTTGCTTGGAGCAGGGGAGCCAGATCTGCTGGGAAGTGTA-TAGGAAGACCCCTGGGGT----TGGGCCACCACTGAGTCACCATTGAGTGCTGTC 646
Macaca  CTACTTTGC-TGGAGCAGGGGAGCCAGATCTGCTCGGAAGTGTACTGGGAAGACCCCTGGGGT----TGGGCCACCACTGAGTCACCATTGAGTGCTGTC 678
Bos     CCACTTGGCCTGGAGCAAGGGGGCCACACCTGCTCAGAAGTGCATTGGGAAAG-CCCTGCAGC----GAGGGCACCACAAAGTCGCCTCCCAGTGCTGCC 626
Canis   CCACTTTGCCTGGACCAGGGGGACCA---CTTCTTAGAAGTA-ACTGGAAGGGCCCCGGGTATGGTGGGGGCTACTGCTATATTATCTTCCACGGCTGCC 582

Homo    CCTTTAG--CTGAGTCTAGCATGCTGCCCAAGGGGT-CTTGTCAGATGCCAGCCTGTCATGGGAGAGCAGATGCCAGATGACCCCAGCAGGATGTGGTGT 740
Pan     CCTTTAG--CTGAGTCTAGCATGCTGCCCAAGGGGT-CTTGTCAGATGCCAGCCTGTCATGGGAGAGCAGATGCCAGACGACCCCAGCAGGATGTGGCGT 743
Macaca  CCTTTAGCTCTGAGTCTAGCATGCTGCCCAAGGGAT-CTTATCAGATGCTGGCCTGTCATGGGAGAGCAGATGCCAGACGTCCCCAGGTGGATGTGGCAT 777
Bos     CCTTTAGCTTGAGGTCTTATGTGCCACCCAAGG------------------ACCTCTCCTGGGAGCCCAGTTGCCAGGC--TTCCAGCTGGGTGCAGAGT 706
Canis   CTGTTAGCTCTGAGGTTCCTGTGCAGCCTAGGAGGTGCCTGTCAGATGCTGGCCTCTTCTGGAGGAGCAGATGCAAGGC--CTCCAGCTGTGTGTGGATT 680

Homo    GGGGGCAGAGATGGAATTTCTAGAAGAGTAAACATGGGCCTTTCTCCATCCA---GCACTGCTTCTTGGGAAAGGAGGTGGCTAG-----GAGGGAGGGA 832
Pan     GGGGGCAGAGATGGAATTTCTAGAAGAGTAAACATGGGCCTTTCTCCATCCA---GCACTGCTTCTTGGGAAAGGGGGTGGCTAG-----GAGGGAGGGA 835
Macaca  GGGGGCAGAGATGGAATTTCTAGAAGAGTAAACATGGGCCTTTCTCCATCCA---GCACTGCTTCTTGGGAAAGGGGAAGGTTAG-----GAGGGAGGGA 869
Bos     GGAGGCAGAGATGGAAAATGTGGAGGAGGAAACACAGGCCTTTCTCTGTCCACCTCCCCTGTCTCCTGGCAAAGGTGGAGGCTGGCCGGTTATGGGGGA 806
Canis   GAGGACAGAGATGGAATATCTGGACGAGTACACAGAGGCCTTTGTCCATTCATTGCCACCACCTCTTGGTAAAGGGGGTGGGTGG-GCCTGAGGGAAGGA 779

Homo    GCCCTCTCCCCTCACAAATGCTCAGGGCCACTTCTTCTCTTTCAG 877
Pan     GCCCTCTCCCCTCACAAATGCTCAGGGCCACTTCTTCTCTTTCAG 880
Macaca  GCCCTCTCCCCTCACGAATGCTCAGGGCCACTTCTTCTCTTCTAG 914
Bos     ACCCAGC-------CAAGCTCTCAGGGCCACCTCTTCTCTTCCAG 844
Canis   GCCCACC-------TGAGTTCTTGGGGCCACCTCCTCTCTTCCAG 817
```

ENSG00000166394 intron 5

Description: cytochrome b5 reductase b5R (CYB5R2)
Intron number: 5
Human chromosome: 11
Intron start (bp): 7645621
Human intron length : 664
Intron alignment length: 766
Flanking exons length (upstream/downstream): 84/86
SNP density: 0.006024
K tree score: 0.0689
Scaling factor: 0.9462
Human-chimpanzee distance: 0.024261
Total primate branch length: 0.0807

ENSG00000166394 exon 5

```
Homo    GGAATCTTGGAATCAGACCAGACCAGACGAGTGAGCCTAAAAAAACACTGGCCGATCACCTGGGAATGATTGCTGGGGGC 80
Pan     GGAATCTTGGAATCAGACCAGACCAGACGAGTGAGCCTAAAAAAAAAACTGGCCGATCACCTGGGAATGATTGCTGGGGGC 80
Macaca  GGAATCTTGGAATCAGACCAGACCACATGAGTGAGCCTAAAAAAAAAACTGGCCCATCACCTGGGAATGATTGCTGGGGGC 80
Bos     GGAAGTTTGTATTCAAGCCATACAAAACGAGTGAGCCCGAAACTAAACTGGTGCATCACCTGGGAATGATTGCTGGGGGC 80
Canis   GGAACTTTTCAATCAAGCCATACAAAACAAGTGAGCCTGAAAAGAAGCTGGTCAGTCACCTGGGAATGATTGCTGGGGGC 80

Homo    ACAG 84
Pan     ACAG 84
Macaca  ACAG 84
Bos     ACAG 84
Canis   ACAG 84
```

ENSG00000166394 exon 6

```
Homo    GCATCACACCCATGTTGCAGCTCATTCGCCACATCACCAAGGACCCCAGTGACAGGACCAGGATGTCCCTCATCTTTGCC 80
Pan     GCATCACACCCATGTTGCAGCTCATTCGCCACATCACCAAGGACCCCAGTGACATGACCAGGATGTCCCTCATCTTTGCC 80
Macaca  GCATCACACCCATGTTGCAGCTCATTCGCCACATCACCAAGGACCCCAGTGACAGCACCAGGATGTCCCTCATCTTTGCC 80
Bos     GGATCACGCCTATGCTGCAGCTCATCCGCTGCATCGCCAGGAAGCCCAGTGACAAGACCGTGATGTCCCTCATCTTCGCC 80
Canis   GGATCACGCCCATGCTGCAGCTCATTCGCCACATCACCAAGAACCCCAACGACAGGACCAGGATGTCCCTCATCTTTGCC 80

Homo    AACCAG 86
Pan     AACCAG 86
Macaca  AACCAG 86
Bos     AACCAG 86
Canis   AACCAG 86
```

ENSG00000166394 intron 5

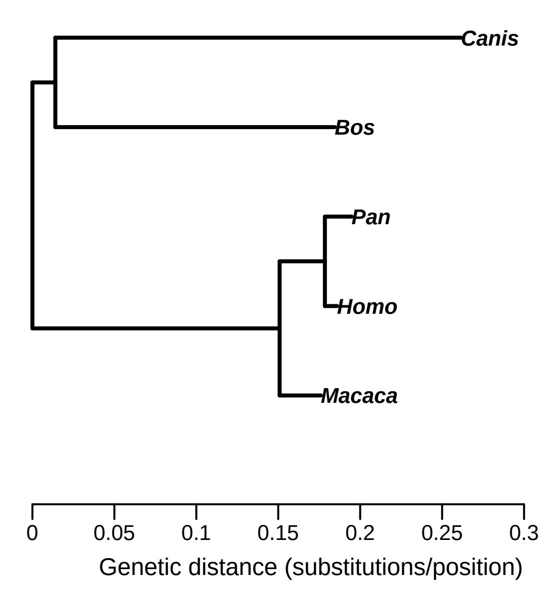

ENSG00000166394 intron 5

```
Homo    GTAAGAGGCTTCTAGGGGCTCCATCCAGAGAGTGAGCCCCGAGACCCATTCTGACTTGGGAGTCATTTGATCCTCTAAGGGACAACATGAGAGATTTCGC 100
Pan     GTAAGAGGCTTCTAGGGGCTCCATCCAGAGAGTGAGCCCTGAGA-CCATTCTGACTTGGGAGTCATTTGATCTTCGAAGGGACAACATGAGAGATTTCGC 99
Macaca  GTAAGAGGCTTCTAGGGGCTCCATCCGGAGAATGAGCCCTGAGA-CCATTCTGACTTGGGAGTCATTTGATCCTCGAAGGGACAACATGAGCGATTTCGC 99
Bos     GTAAGAGGGAGCTGGGGGCTTCATCC--AGGGTGAACCCTGAGA-CCTCTCTCACTTTGGAGTCA--TGGTCCTGGAAGAGGCGATGGAAGAGATTCTTT 95
Canis   GTAAGAGGCTTCT-GGGGCTTTGTCCACAGGGTGGGCCTTTGGA---------ACTTAGGAGTCATTTGATCCTAGAGGAGA-------------TCCTT 77

Homo    CTCCAGCTGTAGAAGGTGGGAACGATGAACCAGTGACAGGTCTTGAAACTGACCCTGTGTACCCTGCTTCCCCTAACCAATATGGTGGCTGCTCAGGAGC 200
Pan     CTCCAGCTGTAGAAGGTGGGAACGATGAACCAGTGACAGGTCTTGAAACTGACCCTGTGTACCCTGCTTCCCCTAACCAATATGGTGGCTGCTCAGGAGC 199
Macaca  CCCCAGCTGTAGAAGGTGGGAATGATGAATCAGTGACAGGTCTTGAAACTGACCCTGTGTGCCCTGCTTCCCCTAACCAACATGGTGGTTGCTCAGGAGC 199
Bos     CCCTGGCTGTTTGAAATGGGGATACAAAGTCAATGACAGTTCTTGACTCTGACCCTGGATGCCCTGCTCCCCTTAACCAACATGGGGGCTACACCAGGGG 195
Canis   CCATGGCTGTTTTAGATGCGGGTGCAGGGTTGATGACAGGCCTGGAAACTGACCCCACGTGCCCTGCTCCCCTGACCCAACAGGGTGGCTAGCCCAGGAA 177

Homo    CTTGTGGGGACCTTTCAGGTCAGGCTTGGGGG-CAAGCTGGGCAGGCCCACCCATGCTGTCCCAGGGGCTCCTAGATCCAGTTCAAGCCTGACTCTCCCC 299
Pan     CTTGTGGGGACCTCTCAGGTCAGGCTGGGGGG-CAAGCTGGGCAGGCCCACCCGTGCTGTCCCAGGAGCTCCTAGATCCAGTTCAAGCCTGACTCTCCCC 298
Macaca  CTTGCGGGGACCTCTCAGGTCAGGCTGAGGGC-CAAGCTGGGCAGGCCCACCCATGCTGTCCCAGGAGCGCCTGGATCCGGTTCAAGTCTGACTCTCCCC 298
Bos     CCCCTGGAG-CTTCTAAGGTGAGTTTGGGGGGGCAGGCTGGGCTGGCCCACACGTGGGCTCCCAAGGGCTCCTGGATCCTATTCAAGCCCGATGCTCCCT 294
Canis   GTCAGGCCGGGGAACATGGAGGAGTCTGGGGATCGAGCCAAATAGGCCCAAACATGGTCCCCCAAGGGCTCTTGGATCCAGGTCAAGCCTGATACTCCTC 277

Homo    ATCCCTTGAAAATGACTGCCTGAAGAAAGAGAGCAGTCTTTGAAGGAGACATACAATGTGCCAGGCACTGTAATAGGCATTCT--TAAAACGATCACATT 397
Pan     ATCCCTTGAAAATGACGGCCTGAAGAAAGAGAGCAGTCTTTGAAGGAGACATACAATGTGCCAGGCACTGTAATAGGCATTCT--TAAAACGATCACATT 396
Macaca  ATCCCTTGAAAATGACTGCCTGAAGGAAGAGAACAATCTTTGAAGGAGACATACAATGTGCCAGGCACTGTAATAGGCACTCT--TAAAGCTATCACATT 396
Bos     ATCCCTTGAAAATGAATGCCTGAAAGAGGAGAACAATCATGTAATGTGAACTACTACGCGCCAGGCACCATAATGGGCATTTTAAAAAAACTACCACATT 394
Canis   ATCCCTTGAAAATAAATATCTGAGAGAATAGAACCAATGTGTACGGATGCCTACAACGTGCCAGGCACCATAATAGGCGCTTT--AAAAACAACCACATT 375

Homo    TACCCGTC----AT--TGTTTGATGAATGAGGACACTGAAGAACTTA----------------------------------------------------- 438
Pan     TACCCGTC----AT--TAATTG---------------------TTTA----------------------------------------------------- 416
Macaca  TACCCGTCATTAAT--TGTTTGATGAATGAGGACACGGAAGAACTTA----------------------------------------------------- 441
Bos     TACTTGTCATTGGTTCTCTTTTACAAACGAAGACACTAAAAAGCTCAGTGAGG----------------------------------------------- 447
Canis   TACTTGTCGTTAATTCTGTTGTACAAACGTGGACACTAAAGAGCTCAGACAGGCACTGCACTCACCCTGCTCAGACGGCAAGTGGGGAGGCTGGGTGTGA 475

Homo    -------------------GAGTGTTCTTTCC-------------ATCTTGCCATATGAAAATGAGAAAGACTACCTGACTTACTGTTGCATTAGAAAGG 506
Pan     -------------------GAGTGTTCTTTCC-------------ATCTTGCCATATGAAAATGAGAAAGACGACCTGACTTACTGTTGCATTAGAAAGG 484
Macaca  -------------------GAGTGTTCTTTCC-------------ATCTTGCCATATGAAAATGAGAAAGACCTTCTGACTTACTGTTGCATTAGAAAGG 509
Bos     ---------GAGCTGGACGGAACATGCAGTCCAGTTTCAAAGCCGATGCTGCCATGTGAAAATGAGAAGGGCT-TTTGATGGACTATTGCATTAGAAGAG 537
Canis   AACGCAGCCAGTTCAGAGCCAGTGCTCCTTCC-------------ATCTTGCCACGTGAACACGAGAAAGGGCTTTGGACTTACTGTTGTGTTAGAAGGG 562

Homo    AAAACCAAAATGAAGGGCTGTTTCCTGC-TTTGAGTTTTGGGGACAGAAGTGAGAAAAGGATAACTTGGGCTGGGGGAAGGTATGAAGCTTGCTGCAGGT 605
Pan     AAAACCAAAATGTAGGGCTGTTTCCTGC-TTTGAGTTTTGGGGACAGAAGTGAGAAAAGGATAACTTGGGCCGGGGGAAGGTATGAAGCTTGCTGCAGGT 583
Macaca  AAAACCAAAATGAAGGGCTGTTTCCCACTTTTGAGTTTTGGGGACAGAAGTGAGAAAAGGATAACTGGGGCTGGGGGCAGGT---AAGCTTGCTGCAGGT 606
Bos     AAAACCAAGATGAAGGGTAGTTCTGCAC-TTTGAGTTTTGGGCACAGAAGTGAGAAAAG--TAATTGAGATCAAGGCAACCTGTGAAGC-TGCTGCAGAG 633
Canis   AAAACCAAAATGCAGGGCAGTTTTCCAC-TTTGAGCTTTGGGGGCAGGCACGAGAAAAGTAGGATTGGAGCTAAGG-AAGCTGTTAAGG-GCCTGCAGGC 659

Homo    CAGGGACTGTGGGCTTCAGAGACAGGGAGAAAG---CTGGCTGCTCGCTCACTG----TGTTCCAG 664
Pan     CGGGGACTGTGGGCTTCAGAGACAGGGAGAAAG---CTGGCTGCTCGCTCACTG----TGTTCCAG 642
Macaca  CAGGGACTGTGGGCTTCAGAGACAGGGAGAAAG---CTGGCTGCCCACTCACTA----TGTTCCAG 665
Bos     CAGAGACTATGGGCATCAGGGGGAGGG-GAAGC---CAGCCTGCCTGCTCTCTG----TGTTCCAG 691
Canis   CAGGGCTTGTGGGCATCGGAGAGGGGGGGAGAGCCCCTAGCTGTGTCCTGCCTGCTGCTGTTCCAG 725
```

ENSG00000181092 intron 1

Description: Adiponectin precursor (ADIPOQ)
Intron number: 1
Human chromosome: 03
Intron start (bp): 188053756
Human intron length : 911
Intron alignment length: 1173
Flanking exons length (upstream/downstream): 214/521
SNP density: 0.007684
K tree score: 0.0766
Scaling factor: 1.0066
Human-chimpanzee distance: 0.015732
Total primate branch length: 0.0806

ENSG00000181092 exon 1

```
Homo    GAAACCACGACTCAAGGGCCCGGAGTCCTGCTTCCCCTGCCCAAGGGGGCCTGCACAGGTTGGATGGCGGGCATCCCAGG 80
Pan     GAAACCACGACTCAAGGGCCCGGAGTCCTGCTTCCCCTGCCCAAGGGGGCCTGCACAGGTTGGATGGCGGGCATCCCAGG 80
Macaca  GATACCACAACTCAAGGGCCCGGAGTCCTGCTTCCTCTGCCCAAGGGGGCCTGCACAGGTTGGATGGCAGGCATCCCAGG 80
Bos     GACAACATGGAAGA---------------TCCCCCGCTGCCCAAGGGGGCCTGCGCAGGTTGGATGGCAGGCATTCCAGG 65
Canis   GACTCCGTGGCAGAAGGGCCTGGAGTCCTGCTTCCCCTGCCGAAGGGGGCCTGCCCAGGTTGGATGGCAGGCATCCCAGG 80

Homo    GCATCCGGGCCATAATGGGGCCCCAGGCCGTGATGGCAGAGATGGCACCCCTGGTGAGAAGGGTGAGAAAGGAGATCCAG 160
Pan     GCATCCGGGCCATAATGGGGCCCCAGGCCGTGATGGCAGAGATGGCACCCCTGGTGAGAAGGGTGAGAAAGGAGATCCAG 160
Macaca  GCATCCAGGCCATAATGGGGTCCCAGGTCGTGATGGCAGAGATGGCACCGCTGGCGAGAAGGGTGAGAAAGGAGATCCAG 160
Bos     GCATCCTGGCCACAACGGGACCCCAGGCCGGGATGGCAGAGATGGCACCCCTGGTGAGAAGGGTGAGAAAGGAGATCCAG 145
Canis   GCATCCTGGCCACAATGGGACCCCAGGCCGTGATGGCAGAGATGGCACCCCTGGTGAAAAGGGTGAGAAAGGAGATCCAG 160
```

ENSG00000181092 exon 2

```
Homo    GTCTTATTGGTCCTAAGGGAGACATCGGTGAAACCGGAGTACCCGGGGCTGAAGGTCCCCGAGGCTTTCCGGGAATCCAA 80
Pan     GTCTTATTGGTCCTAAGGGAGACATCGGTGAAACCGGAGTACCCGGGGCTGAAGGTCCCCGAGGCTTTCCGGGAATCCAA 80
Macaca  GTCTTATTGGTCCTAAGGGAGACACTGGTGAAACTGGAGTAACCGGGGCTGAAGGTCCCCGAGGCTTTCCGGGAATCCAA 80
Bos     GTCTTGTTGGTCCTAAGGGTGACACTGGTGAAACTGGAATCACTGGGATCGAAGGTCCCCGAGGCTTTCCAGGAACCCCA 80
Canis   GTCTTGTTGGTCCTAAGGGTGACACTGGTGAAACTGGAGTAACTGGGGTTGAAGGTCCCCGAGGCTTTCCAGGAACCCCA 80

Homo    GGCAGGAAAGGAGAACCTGGAGAAGGTGCCTATGTATACCGCTCAGCATTCAGTGTGGGATTGGAGACTTACGTTACTAT 160
Pan     GGCAGGAAAGGAGAACCTGGAGAAGGTGCCTATGTATACCGCTCAGCATTCAGTGTGGGATTGGAGACTTACATTACTGT 160
Macaca  GGCAGGAAAGGAGAACCTGGAGAAGGTGCCTATGTATACCGCTCAGCATTCAGTGTGGGATTGGAGACCTACGTTACTGT 160
Bos     GGCAGAAAGGGAGAACCTGGAGAAAGTGCCTATGTATACCGCTCAGCATTCAGTGTGGGACTGGAGAGACAGGTCACTGT 160
Canis   GGAAGGAAAGGAGAACCTGGGGAAAGTGCCTATGTACACCGTTCGGCATTCAGTGTGGGGTTGGAGAGCCGGATCACTGT 160
```

ENSG00000181092 intron 1

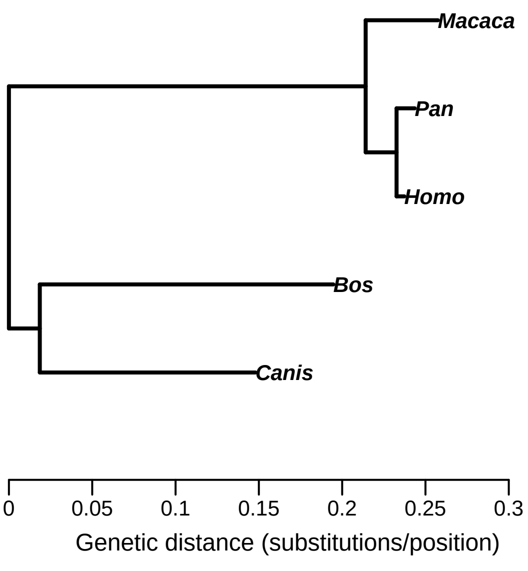

ENSG00000181092 intron 1

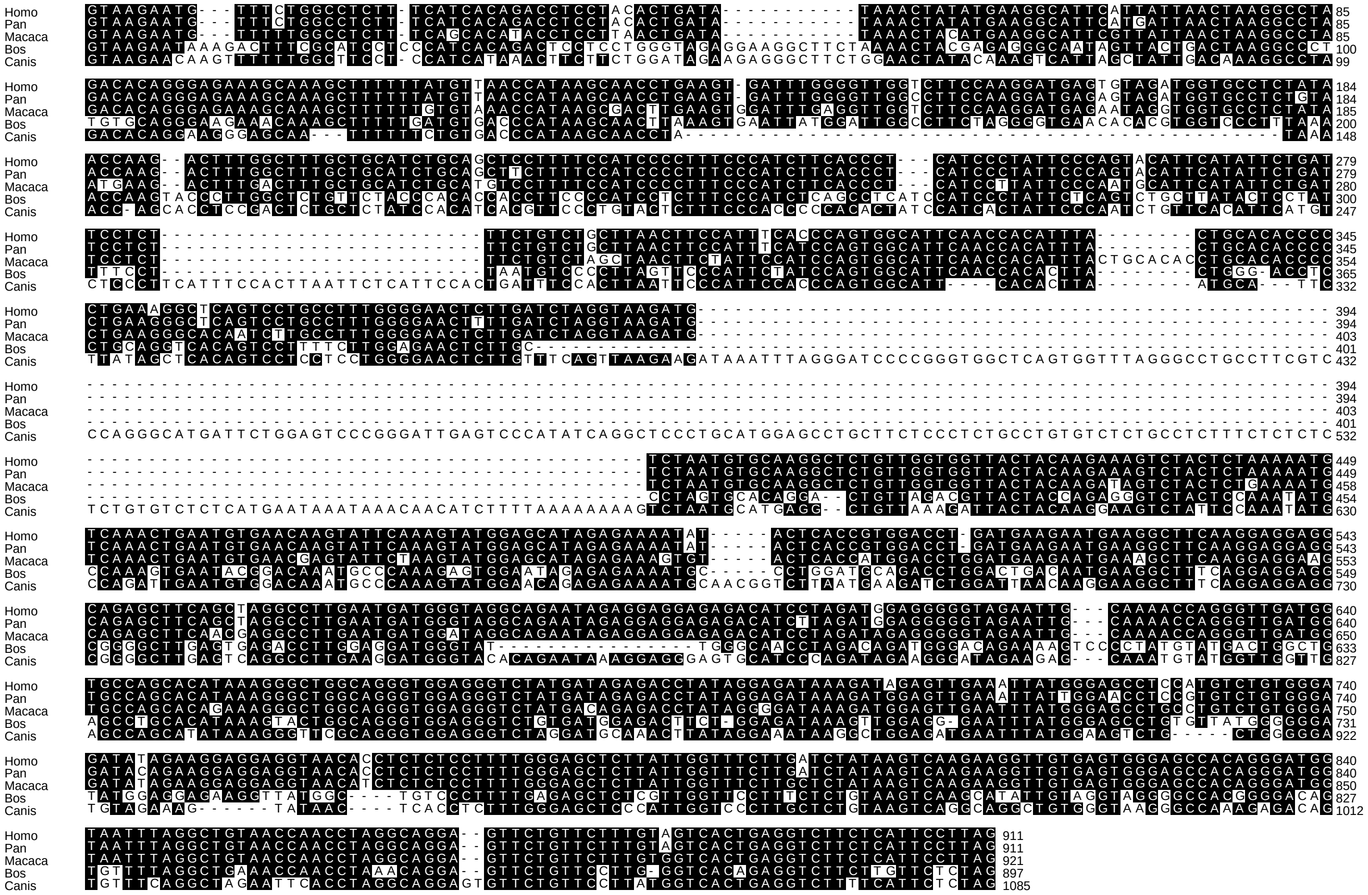

ENSG00000108405 intron 2

Description: P2X purinoceptor 1 (P2RX1)
Intron number: 2
Human chromosome: 17
Intron start (bp): 3754973
Human intron length : 290
Intron alignment length: 298
Flanking exons length (upstream/downstream): 148/72
SNP density: 0.006897
K tree score: 0.0759
Scaling factor: 0.8843
Human-chimpanzee distance: 0.010398
Total primate branch length: 0.0806

ENSG00000108405 exon 2

```
Homo    GTGGGTGTTTCTCTATGAGAAGGGCTACCAGACCTCGAGCGGCCTCATCAGCAGTGTCTCTGTGAAACTCAAGGGCCTGG 80
Pan     GTGGGTGTTTCTCTACGAGAAGGGCTACCAGACCTCGAGCGGCCTCATCAGCAGTGTCTCTGTGAAACTCAAGGGCCTGG 80
Macaca  GTGGGTGTTCCTCTACGAGAAGGGCTACCAGACCTCGAGCGGCCTCATCAGCAGTGTGTCTGTGAAACTCAAGGGCCTGG 80
Bos     GTGGGTGTTTGTCTACGAGAAGGGCTACCAGACCTCGGGCAGCCTCATCAGCAGTGTGTCTGTTAAACTCAAAGGTCTGG 80
Canis   GTGGGTGTTTGTCTACGAGAAGGGCTATCAGACTTCAAGTGGCCTCATCAGCAGCGTGTCTGTGAAACTCAAAGGTCTAG 80

Homo    CCGTGACCCAGCTCCCTGGCCTCGGCCCCCAGGTCTGGGATGTGGCTGACTACGTCTTCCCAGCCCAG 148
Pan     CCGTGACCCAGCTCCCTGGCCTCGGCCCCCAGGTCTGGGACGTGGCTGACTACGTCTTCCCAGCCCAG 148
Macaca  CCGTGACCCAGCTCCCTGGCCTCGGCCCCCAGGTCTGGGACGTGGCTGACTACGTCTTCCCAGCCCAG 148
Bos     CTGTGACACAGCTCCCAAGCCTGGGCCCCCAGGTCTGGGATGTGGCCGACTACGTCTTCCCAGTGCAG 148
Canis   CCGTGACACAGCTCCCAGGCCTGGGCTCCCAGGTCTGGGATGTAGCTGACTACGTCTTCCCAGCCCAG 148
```

ENSG00000108405 exon 3

```
Homo    GGGGACAACTCCTTCGTGGTCATGACCAATTTCATCGTGACCCCGAAGCAGACTCAAGGCTACTGCGCAGAG 72
Pan     GGGGACAACTCCTTCGTGGTCATGACCAATTTCATCGTGACCCCGAAGCAGACTCAAGGCTACTGCGCAGAG 72
Macaca  GGGGACAATTCCTTCGTGGTCATGACCAATTTCATCGTGACCCCAAAGCAGACTCAAGGCTACTGCGCAGAG 72
Bos     GGGGACAGTTCCTTCGTAGTCATGACCAATTTTATCGCCACCCCCCGGCAGGCTCAAGGTCACTGTGCAGAG 72
Canis   GGGGACAGCTCCTTCGTGATCATGACCAATTTCATTGCGACCCTCCAGCAGGTTCAAGGCTACTGTGCGGAG 72
```

ENSG00000108405 intron 2

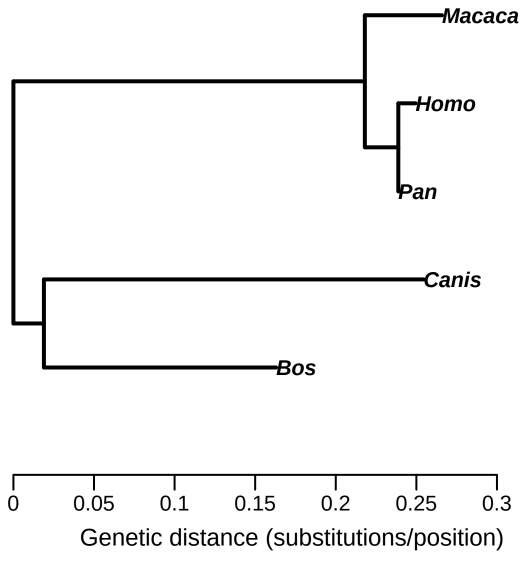

ENSG00000108405 intron 2

```
Homo     GTGAGCTGGC--TCAGGTTTGCACCTTCCCCTCACAGGGCCTGT---CTCAGAGTTGGGTGGGACTCTGTCCCACGTCCCTGTGGGACCAGCCCAGCTCC 95
Pan      GTGAGCTGGC--TCAGGGCTGCACCTTCCCCTCACAGGGCCTGT---CTCAGAGTTGGGTGGGACTCTGTCCCACGTCCCTGTGGGACCAGCCCAGCTCC 95
Macaca   GTGAGCTGAC--TCGGGGCTGCGCCTTCCCCTCGCAGGGCCTGT---CTCAGAGCTGG-----------------------GTGGGACCAGCCCAGCTCC 72
Bos      GTGAGCTGACTGTGCGGGGGGCACCTCCCACCCACAGGGCGTGCAGGCTCAGAGCTGGGTTAGGCTCTGTCCCGCCTCCCTGTGGG-------------- 86
Canis    GTGAGCTGACT-CAGGGGTAATACCTTCCACCCACAGGGTGG-----CTCAGAGCGGACTTGGGCTCTGTCCAATCTCCCTATTGCACTAGCCCAGG--- 91

Homo     TCTTTCTCTCTGGACCTCTGTGAATGCCGGGCAGAAGCAG-CCTGGTGATATCACCCCATGTTGGTGGCAGGGGGTGTGGTGGCTGCTGCGTGTCCTGTG 194
Pan      TCTTTCTCTCTGGACCTCTGTGAATGCCGGGCAGAAGCAG-CCTGGTGATATCACCCCATGTTGATGGCAGGGGGTGTGGTGGCTGCTGCGTGTCCTGTG 194
Macaca   TCTTTCTCTCTGGACCTC------TGCCGGGCAAAGGCAGCCCTGGTGATATCACCCCATGTGGGTGGCAGGGGGTGTGGTGGCTGCTGCGTGTCCTGTG 166
Bos      ------CCTCAGTTTCCCTGCGAACGAGGGACAGATGCAG-CCCCGTACCATCAGCCCACTCTGATGTCA-TGAGTGTTGAGGCTGCTGGGGGTCCTGTG 178
Canis    ------CCCCAGCTCCCCT---AATGAGGGGCAGAAATGG--GTTGTAATATCATCCCCCTCTGATATCATGGGGTGTGGAGGCTGCTGGGTATCATGTG 180

Homo     GGCTCTAGGGGCCTCTGCTGGGGCAGCGGTGGGGCCCTT-GGGGCTCTTCTCCCAGCTCCCAGCCC-ACCCCACCCTCCCCCTGCCCCTCACTTCTAG 290
Pan      GGCTCTAGGGGCCTCTGCTGGGGCAGCGGTGGGGCCCTT-GGGGCTCTTCTCCCAGCTCCCAGCCC-ACCCCACCCTCCCCCTGCCCCTCACTTCTAG 290
Macaca   GGCTCTAGGG--TCCTGCTGGGGCAGCATTGGGGCCCTT-GAGGCTGTT-------CTCCCAGCCC-ACCCTGCCCTCCCCCTGCCCCT--CTTCTAG 251
Bos      GGTTCTGGGG--GCTGGCTGGGACCATGCTGGTGTTCTC-CGGGCTCTT---CCAGCTCAAAACCCTGTCTTGCCCTCCCCCTGCCTCTCACTTCCAG 270
Canis    GATTCTAGGC--ACCTATTGGGACTGTGCTTGGGTTCTCGGGGGCTGTTCTCCCAACCCAGAGCCCTCCCCCTTCCCTCCCCCTGCCTGTCGTTTCCAG 276
```

ENSG00000082516 intron 14

Description: Gem-associated protein 5 (GEMIN5)
Intron number: 14
Human chromosome: 05
Intron start (bp): 154271652
Human intron length : 1000
Intron alignment length: 1623
Flanking exons length (upstream/downstream): 140/172
SNP density: 0.002000
K tree score: 0.0536
Scaling factor: 1.0982
Human-chimpanzee distance: 0.012124
Total primate branch length: 0.0805

ENSG00000082516 exon 14

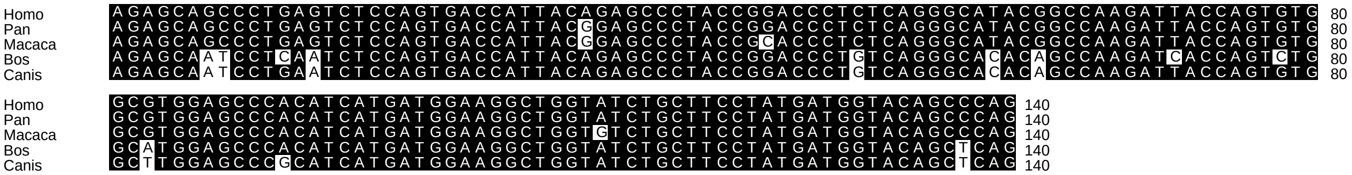

ENSG00000082516 exon 15

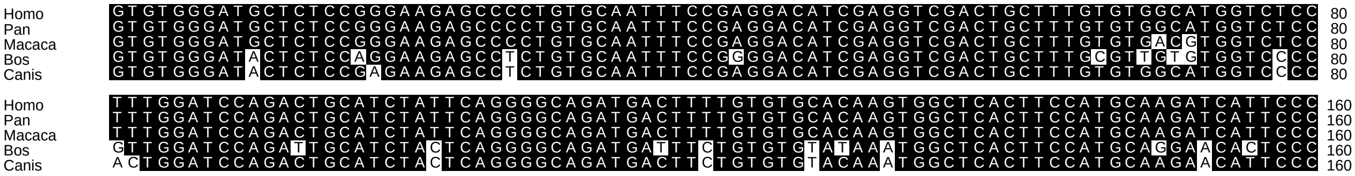

ENSG00000082516 intron 14

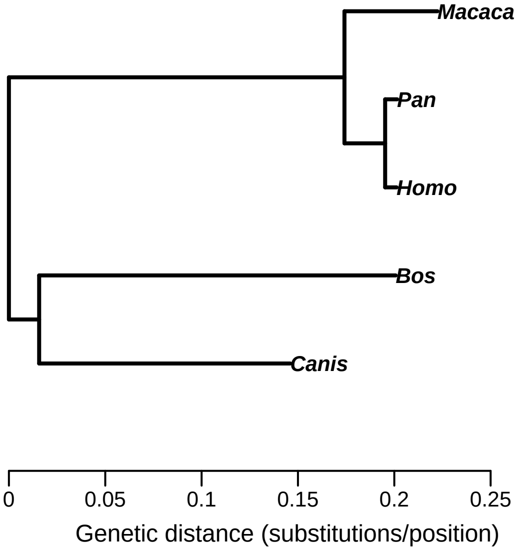

ENSG00000082516 intron 14

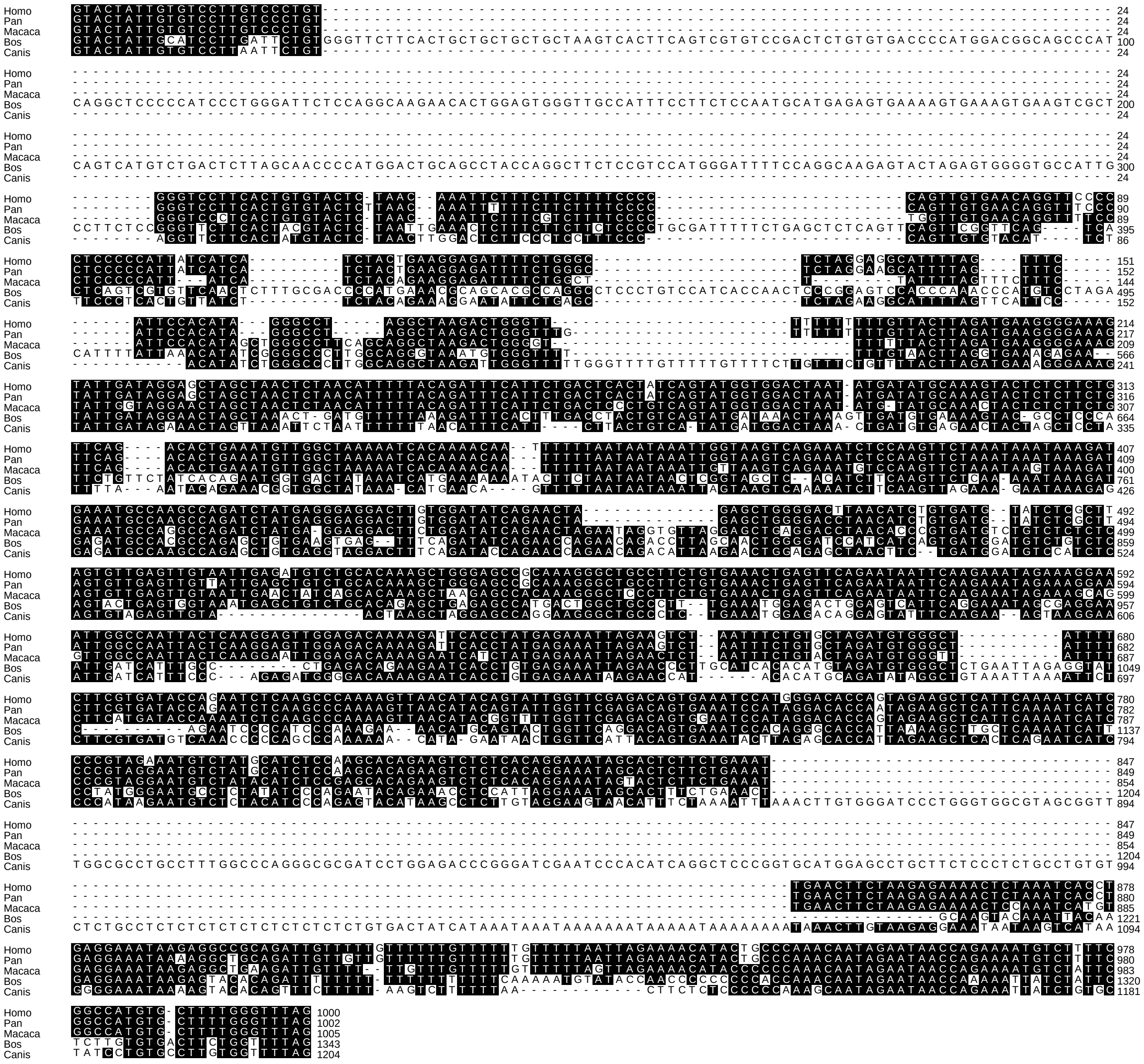

ENSG00000135966 intron 8

Description: TGF beta receptor associated protein -1 (TGFBRAP1)
Intron number: 8
Human chromosome: 02
Intron start (bp): 105255904
Human intron length : 529
Intron alignment length: 1013
Flanking exons length (upstream/downstream): 147/160
SNP density: 0.009452
K tree score: 0.091
Scaling factor: 0.5887
Human-chimpanzee distance: 0.023022
Total primate branch length: 0.0805

ENSG00000135966 exon 8

```
Homo    GTCGGAGTTCAGGTTTTCACCAAGAGACCTTTGGATGAACAGCAGAAGAACAGTTTTAATCCAGACGACATTATCAATTG 80
Pan     GTCGGAGTTCAGGTTTTCACCAAGAGACCTTTGGATGAACAGCAGAAGAACAGTTTTAATCCAGACGACATTATCCATTG 80
Macaca  GTCGGAGTTCAGGTTTTCTTTAAGAGACCTTTGGATGAACAGCAGAATAACAGTTTTAATCCCGATGACATTATCAATTG 80
Bos     GTCGGAGTTCAGGTTTTCACCAAGAGACCACTAGATGAACAGCAG---AGTGGTTTTAACCCAGATGATATCATCAGTTG 77
Canis   GTTGGAGTTCAAGTTTTCACCAAGAGACCTTTGGATGAACAGCAG---AACAGTTTTAATCCAGACAGTATAATCACTTG 77

Homo    CCTTAAAAAATACCCTAAAGCCCTTGTGAAGTATCTGGAACATCTTGTGATAGACAAGAGACTGCAG 147
Pan     CCTTAAAAAATACCCTAAAGCCCTTGTGAAGTATCTGGAACATCTTGTGATAGACAAGAGACTGCAG 147
Macaca  CCTTCACAAATACCCTAAAGCCCTTGTGAAGTATCTGGAACATCTTGTGATAGACAAGAGACTGCAG 147
Bos     CCTTAAGAAATACCCTCAAGCCCTGGTTAAGTACCTGGAGCATCTTGTCACAGAAAGGAGACTGCAG 144
Canis   CCTTAAGAAATACCCTAAAGCCCTTGTCAAGTACCTGGAACATCTAGTCATAGACAGAAGACTGCAG 144
```

ENSG00000135966 exon 9

```
Homo    AAAGAAGAGTATCACACCCACTTAGCTGTGCTGTACCTGGAAGAGGTGCTGCTGCAGAGGGCCTCCGCCAGTGGCAAGGG 80
Pan     AAAGAAGAGTATCACACCCACTTAGCTGTGCTGTACCTGGAAGAGGTGCTGCTGCAGAGGGCCTCCGCCAGCGGCAAGGG 80
Macaca  AAGGAAGAGTATCACACTCACTTAGCTGTGCTCTACCTGGAAGAGGTGCTGCAGCAGAGGGCCTCCACCAGCGGCAAGGG 80
Bos     AAGGAGGAGTACCATACGCACCTGGCTGTCCTCTACCTGGACGAGGTGCTGCAGCAGAGGCCCTGCACCCCTGACAAGGA 80
Canis   AAGGAAGAGTACCACACGCACCTGGCCCTCCTTTACCTGGACAAGGTGCTGCAGGAGAGGTCTGGTGCCAACAGCAAGGG 80

Homo    TGCAGAGGCCACCGAGACGCAGGCCAAGCTGCGGCGGCTGCTCCAGAAATCTGATTTATACCGAGTCCACTTTCTTCTCG 160
Pan     TGCAGAGGCCACCGAGACGCAGGCCAAGCTGCGGCGGCTGCTCCAGAAATCTGATTTATACCGAGTCCACTTTCTTCTCG 160
Macaca  CGCAGAGGCCTCTGAGACACAGGCCAAGCTTCGGCGGCTGCTCCAGAAGTCCGATTTATACCGAGTTCACTTTCTCCTTG 160
Bos     TGCAGAAGTGACTGAGACGCAGGCGAAGCTGCGACGCCTGCTCCAGGAATCTGATCTCTACCGAGTCCACTTTCTGATGG 160
Canis   TGTGGAAGCGACAGAGACACAGGTGAAGCTCCAGCACCTGCTCCAGAAGTCCGATCTGTACCGAGTCCACATCCTGATAG 160
```

ENSG00000135966 intron 8

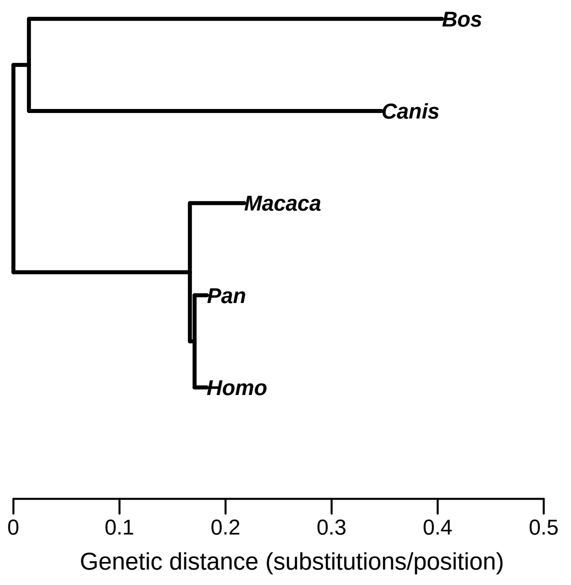

ENSG00000135966 intron 8

```
Homo    GTGAGCACTGCAGAAGTTAGACTAAAGAGTGTGGG---------------------CTGGAGAGACGGAATTGAACTAGAACACTTTTTGGCATGTGA-T 78
Pan     GTGAGCACTGCAGAACTTAGACTAAAGAGTGTGGG---------------------CTGGAGAGACGGAATTGAGCTAGAACACTTTTTGGCATGCGA-T 78
Macaca  GTGAGCACTGCAGAACTTAGACTAAAGAGTGTGGG---------------------CTGGAGAGACGGAATTGAACTAGAGCACTTTTTGGTGTGTGGTT 79
Bos     GTGAGGCCTG--GCTCTCAGGCCAATAT-CATGGGTTTGCTGGGGCCCTATGGGAACCTGAA------------------------CTTGGCCAGTGGGT 73
Canis   GTGAGCACCTCCAAACTTAGACTAACATGTGCAGGGCTGCCTGGTCATAGTAGTGACCTAAGAGATAGAGTTGGACTAGAAGGCTTGTTGGCAGATGGCT 100

Homo    TCTGTGTAATG------------------TCTGTTCCCTGG--GTGATCCATCCGTACTTGTTGATG--------------------------------- 125
Pan     TCTGTGTAATG------------------TCTGTTCCCCGG--GTGATCCATCCATACTTGTTGATG--------------------------------- 125
Macaca  TCTGTGTAATG------------------TCTGTTCCCCGGGTGTGATCCATCCGTACTTGTTGATG--------------------------------- 128
Bos     CTAACTGAACG-------------------TCTTATGTTCCTGGCGCTCAATCTGTACTTGCGGGAG--------------------------------- 121
Canis   CTAATGCAATATTTTAGGTGTAGGTTTTTTTTTTTTTTTTTTAAAGATTTTTATGTAGTTATTCATGAGAGACACAGAGAGAAGCAGAGACACAGGCAGA 200

Homo    -------------------------GTCTGATGGCGGCCTTGA-----GGCTTGCGGAAAGAGACCTGGAGCAATAGGTAGCTTTA-------------- 181
Pan     -------------------------GTCTGATGGGGGCCTTGA-----GGCTTGCGGAAAGAGACCTGGAGCAATAGGTAGCTTTA-------------- 181
Macaca  -------------------------GTCTGATGGGGGCCTTGA-----GGCTTGGGGAAAGAGACCTGGACCAATAGGTAGCTTTA-------------- 184
Bos     -------------------------GTCAGAGAGGGAATCAGA-------CATTTGTGAAAGAGACCGGAGGCCCAGGTGGC------------------ 171
Canis   GGGAGAAGCAGGCTCCATGCAGGGAGCCTGATGTGAGACTCAATCCCGGGTCTCCAGGATCACACCCTAGGCTGAAGGCGGCGCTAAACCGCTGAGCCAC 300

Homo    ------------------------------------------TTTTTTTCTCCGTCCGTCTCACACCCACTCAGCTTTTATTT----------------- 222
Pan     ------------------------------------------TTTTTTTCTCCGTCCGTCTCACACCCACTCAGCTTTTATTT----------------- 222
Macaca  ------------------------------------------TTTTTTTCTCCTTCTATCTCACCCCCACTTAGCTTTCATTT----------------- 225
Bos     ------------------------------------------TTGTTTTCACCCTCCACCTCTTGTCCCGTCCACTTTCATTTTT--------------- 214
Canis   CGGGGCTGCCCAGGTGTAGGTGTTTTAAATGTTCTAATGTAGTATTTTAAATGCCCTGTTCCCAGGATGCTCAGCCTGTATTTGTGGATGGCCTAGGGCG 400

Homo    ------------------------------TTCCTCTCTTTTCCTTCTTTGTTATTATCCTTAAAGGAAATAAAGGGAGCTATACCTTTTTGTAGG---- 288
Pan     ------------------------------TTCCTCTCTTTTCCTTCTTTGTTATTATCCTTAAAGGAAATAAAGGGAGCTATACCTTTTTGTAGG---- 288
Macaca  ------------------------------TCCCTCTCTTTTCCTTATTTGTTATTATCTGTAAAGGAAATAAAGGGAGCTATACCTTTTTGTAGG---- 291
Bos     ------------------------------CTTCTTTGTTTTTCTTCCTTGTTATTATTCTTAAAGGAAGTGAGTGAAGCTTCCTTTCTTTGGAGGCAGC 284
Canis   CTGGAGGTTTCTGCAGCCTGGAGTGCATGGCTTTGTCTCTATCTTCCTTTGTTATTATCCTT-AGGGAAATGAGGGAAGCTTCACCTTTCTGGAAA---- 495

Homo    ---------------TTATCTAAATTCTGTGGCCCTTACTTCTTGAAATAAGA----------------------------GGTGACATTTTGTTC---- 341
Pan     ---------------TTATCTAAATTCTGTGGCCCTTACTTCTCAAAATAAGA----------------------------GGTGACATTTTGTTC---- 341
Macaca  ---------------TTATCTACATTCTGTGGCCCTTACTTCTCAAAATAAGA----------------------------GGTGACTTTTTGTTC---- 344
Bos     TAAATCTTGTGGCCCTTACCTAAATCCTGTGGCCCTTACTTCTCAGAATGAGATACTTTCCCCTAGATTCTGTTTCATTCTGCTTATTATTTATTCGGCT 384
Canis   ---------------ATACCTAAATCCGGTGGCCCTCACTTCTCAAATGAGAA----------------------------GCGACCCTTTTGCAT---- 548

Homo    -----------------------------TATTCCCCATAGATGGTAAACTCATCTCAGT---------------------------------------- 372
Pan     -----------------------------TACTCCCCATAGATGGTAAACTCATCTCAGT---------------------------------------- 372
Macaca  -----------------------------TATTGCC--------------------CAGT---------------------------------------- 355
Bos     GCATTGGGTCTCAGTTGTGGCACGTGGCATCTTCCTTGTGGCATGTGGGCTCTTTCCTGTGGGCTTAGTTGCCCCCCAGCATGTGGGATCCTAGCTCCCC 484
Canis   -----------------------------TTTTTCTCATAGAT-------TTTTCTCCGT---------------------------------------- 572

Homo    -------------------------------------------------------------------------------------------------ATC 375
Pan     -------------------------------------------------------------------------------------------------ATC 375
Macaca  -------------------------------------------------------------------------------------------------ATC 358
Bos     GACTAGGGATCAAACCTGCGTCCCCTGCATTGGAAGGTGAAGGCTTGACCACCGGACCCCAGGGCAGTCTCCCAAAAAGTTTATATTCAGGTCCTCCATC 584
Canis   -------------------------------------------------------------------------------------------------ATT 575

Homo    ATGGTTTCTGAGAGGGTTTCGATCCAGCAAGT----------------GAGCATCTGCCCAGCTCATGTCAGTGCAGCCGC------------------- 440
Pan     ATGGTTTCTGAGAGGGTTTCGATCCAGCAAGT----------------GAGCATCTGCCCAGCTCATGTCAGTGCAGACCC------------------- 440
Macaca  GTGGTTTCTGAGAGGGTTTTGATCCAGTGAGT----------------GAG----TGCCCAGCTCATGTCAGCGCAGCCCC------------------- 419
Bos     ATGCTTTCTGAAA-GCTTTCTCTCCAGTACTTGCATTCTGTTGACCTGGAGCCCCTGCTCACATCACACATCTGGGCTCCCACACATGCCATCCTGGGCG 683
Canis   GTGGTTTCTGGGATGTTTTCACTC-----AGT----------------GAGCA--TGTCCTGTTAACTGCCCCAGAGACCC------------------- 633

Homo    -------------GGTC--------TCCAAGCCCTGGCTGACAGGGGAGAGCTTTGACTTTC---CCACTTGAAAACACTGTCTGTCTGCTTGGCTCCCC 516
Pan     -------------GGTC--------TCCAAGCCCTGGCTGACAGGGGAGAGCTTTGACTTTA---CCACTTGAAAACACTGTCTGTCTGCTTGGCTCCCC 516
Macaca  -------------GGGC--------TCCAAGCCCTGGCTGACGGGGGAGACCTTTGACTTTC---CCACTTGAAAACACTGTCTGTCTGCTTGGCTCCCC 495
Bos     GGGGGTGGGGTGGTCTCTGGGCTCCTGCACACGTCGTCAGGGGCGGGGGTGGGGTGGTCTCCTGGCCCCCTGCACATGCTGCCCGTCTGCTCA------C 777
Canis   -------------TGCCCTGGCTTGCCTTGGCGCAGTCCTGAGCTGGGGAGCTCCCAC-TTC---CCGCCTACAAACACTATCTGTCTGCTTGGATTCCC 716

Homo    CTGTTTCCTGCAG 529
Pan     CTGTTTCCTGCAG 529
Macaca  CTGTTTCCTGCAG 508
Bos     CCCTGTCCTGCAG 790
Canis   --GTCTCCTGCAG 727
```

ENSG00000100162 intron 4

Description: Centromere protein M (CENPM)
Intron number: 4
Human chromosome: 22
Intron start (bp): 40669652
Human intron length : 1523
Intron alignment length: 1807
Flanking exons length (upstream/downstream): 80/92
SNP density: 0.003283
K tree score: 0.0211
Scaling factor: 0.5596
Human-chimpanzee distance: 0.010775
Total primate branch length: 0.0804

ENSG00000100162 exon 4

```
Homo    TCTCCAGAACACAGAGGAGTCCCTGCGCCATGTGGATGCCAGCTTCTTCTTGGGGAAGGTGTGTTTCCTCGCCACAGGTG 80
Pan     CCTCCAGAACACAGAGGAGTCCCTGCGCCATGTGGATGCCAGCTTCTTCTTGGGGAAGGTGTGTTTCCTCGCCACAGGTG 80
Macaca  CCTCCAGAACACTGAGGAGTCCCTGCGCCATGTGGATGCCAGCTTCTTCTTGGGGAAGGTGTGTTTCCTTGCCACAGGTG 80
Bos     CCTGCAGAGCGTGGAGGAGTCCCTGTGCCACCTGGACGCAGCCTTCTTCCTGGGGAAGGTGGCCTTCCTCGCCACGGGGG 80
Canis   CCTCCAGAACGTGGAGGAGTCCCTGCGCCATGTGGACGCCACCTTCTTCCTGGGGAAGGTGGGCTTCCTCGCCATGGGTG 80
```

ENSG00000100162 exon 5

```
Homo    CTGGGCGGGAGAGCCACTGCAGCATTCACCGGCACACCGTGGTGAAGCTGGCCCACACCTATCAAAGCCCCCTGCTCTAC 80
Pan     CTGGGCGGGAGAGCCACTGCAGCATTCACCGGCACACCGTGGTGAAGCTGGCCCACACCTATCAAAGCCCCCTGCTCTAC 80
Macaca  CTGGGCGGGAGAGCCACTGCAGCATTCACCGGCACACCGTGGTGAAGCTGGCCCACACCTACCAAAGCCCCCTGCTCTAC 80
Bos     CTGGGCGGGACAGCCACTGCAGCATCCACCGGAACACCGTGGTCAAGCTGGCCCACACCTACCGGAGCCCTCTGCTCTTC 80
Canis   CTGGGCAGGAGAGCCACTGTAGCATCCATCGGAACACTGTCACGAAGCTGGCCAACACCTACAGAAGCCCTCTGTTCTTC 80

Homo    TGTGACCTGGAG 92
Pan     TGTGACCTGGAG 92
Macaca  TGTGACCTGGAG 92
Bos     TGTGACCTAGAG 92
Canis   TGTGACCTGGAG 92
```

ENSG00000100162 intron 4

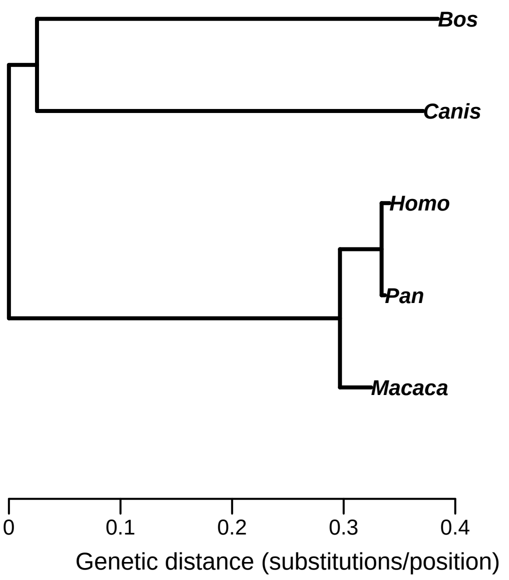

ENSG00000100162 intron 4

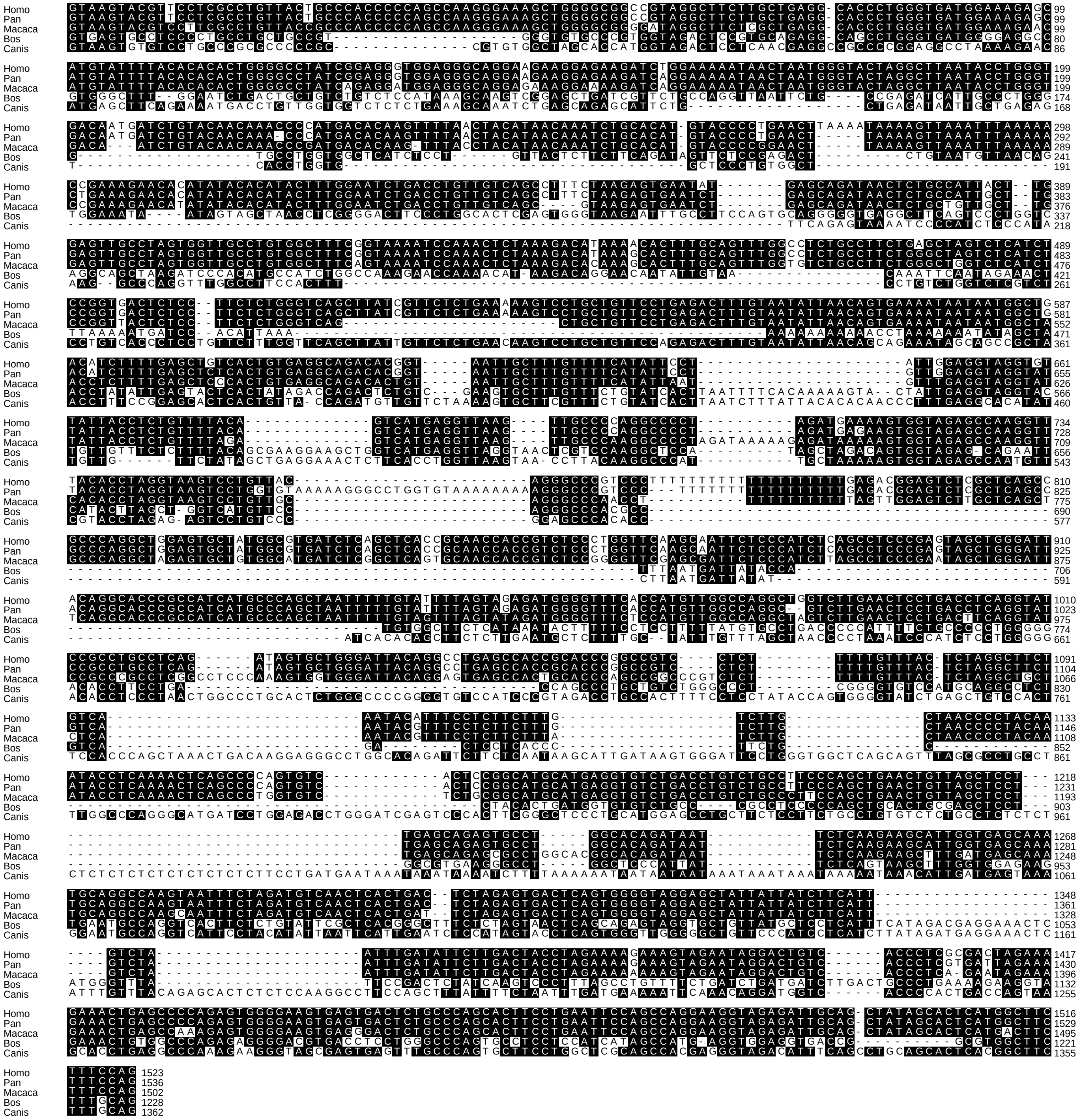

ENSG00000111537 intron 1

Description: Interferon gamma precursor (IFNG)
Intron number: 1
Human chromosome: 12
Intron start (bp): 66838307
Human intron length : 1242
Intron alignment length: 1325
Flanking exons length (upstream/downstream): 114/69
SNP density: 0.006441
K tree score: 0.0354
Scaling factor: 1.0214
Human-chimpanzee distance: 0.014895
Total primate branch length: 0.0804

ENSG00000111537 exon 1

```
Homo    ATGAAATATACAAGTTATATCTTGGCTTTTCAGCTCTGCATCGTTTTGGGTTCTCTTGGCTGTTACTGCCAGGACCCATA 80
Pan     ATGAAATATACAAGTTATATCTTGGCTTTTCAGCTCTGCATCGTTTTGGGTTCTCTTGGCTGTTACTGCCAGGACCCATA 80
Macaca  ATGAAATACACAAGTTATATCTTGGCTTTTCAGCTCTGCATTGTTTTGGGTTCTCTTGGCTGTTACTGCCAGGACCCATA 80
Bos     ATGAAATATACAAGCTATTTCTTAGCTTTACTGCTCTGTGGGCTTTTGGGTTTTTCTGGTTCTTATGGCCAGGGCCAATT 80
Canis   ATGAATTATACAAGCTATATCTTAGCTTTTCAGCTTTGCGTGATTTTGTGTTCTTCTGGCTGTAACTGTCAGGCCATGTT 80

Homo    TGTAAAAGAAGCAGAAAACCTTAAGAAATATTTT 114
Pan     TGTAAAAGAAGCAGAAAACCTTAAGAAATATTTT 114
Macaca  TGTAAAAGAAGCAGAAAACCTTAAGAAATATTTT 114
Bos     TTTTAGAGAAATAGAAAACTTAAAGGAGTATTTT 114
Canis   TTTTAAAGAAATAGAAAACCTAAAGGAATATTTT 114
```

ENSG00000111537 exon 2

```
Homo    AATGCAGGTCATTCAGATGTAGCGGATAATGGAACTCTTTTCTTAGGCATTTTGAAGAATTGGAAAGAG 69
Pan     AATGCAGGTCATTCAGATGTAGCAGATAATGGAACTCTTTTCTTAGGCATTTTGAAGAATTGGAAAGAG 69
Macaca  AATGCAGGTGATCCAGATGTAGCAGATAATGGAACTCTTTTCTTAGACATCTTGAGGAATTGGAAAGAG 69
Bos     AATGCAAGTAGCCCAGATGTAGCTAAGGGTGGGCCTCTCTTCTCAGAAATTTTGAAGAATTGGAAAGAT 69
Canis   AATGCAAGTAATCCAGATGTATCGGACGGTGGGTCTCTTTTCGTAGATATTTTGAAGAAATGGAGAGAG 69
```

ENSG00000111537 intron 1

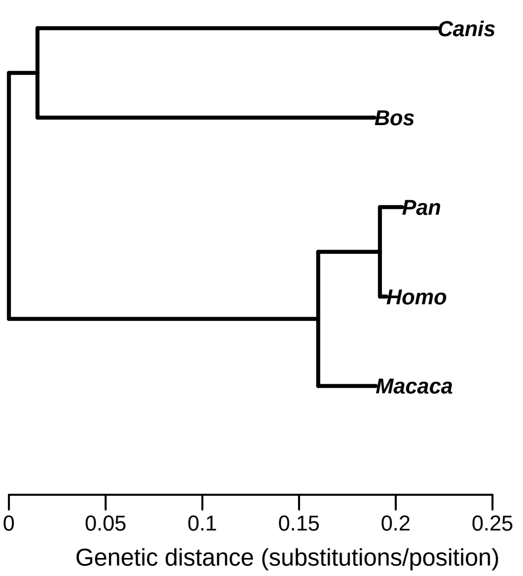

ENSG00000111537 intron 1

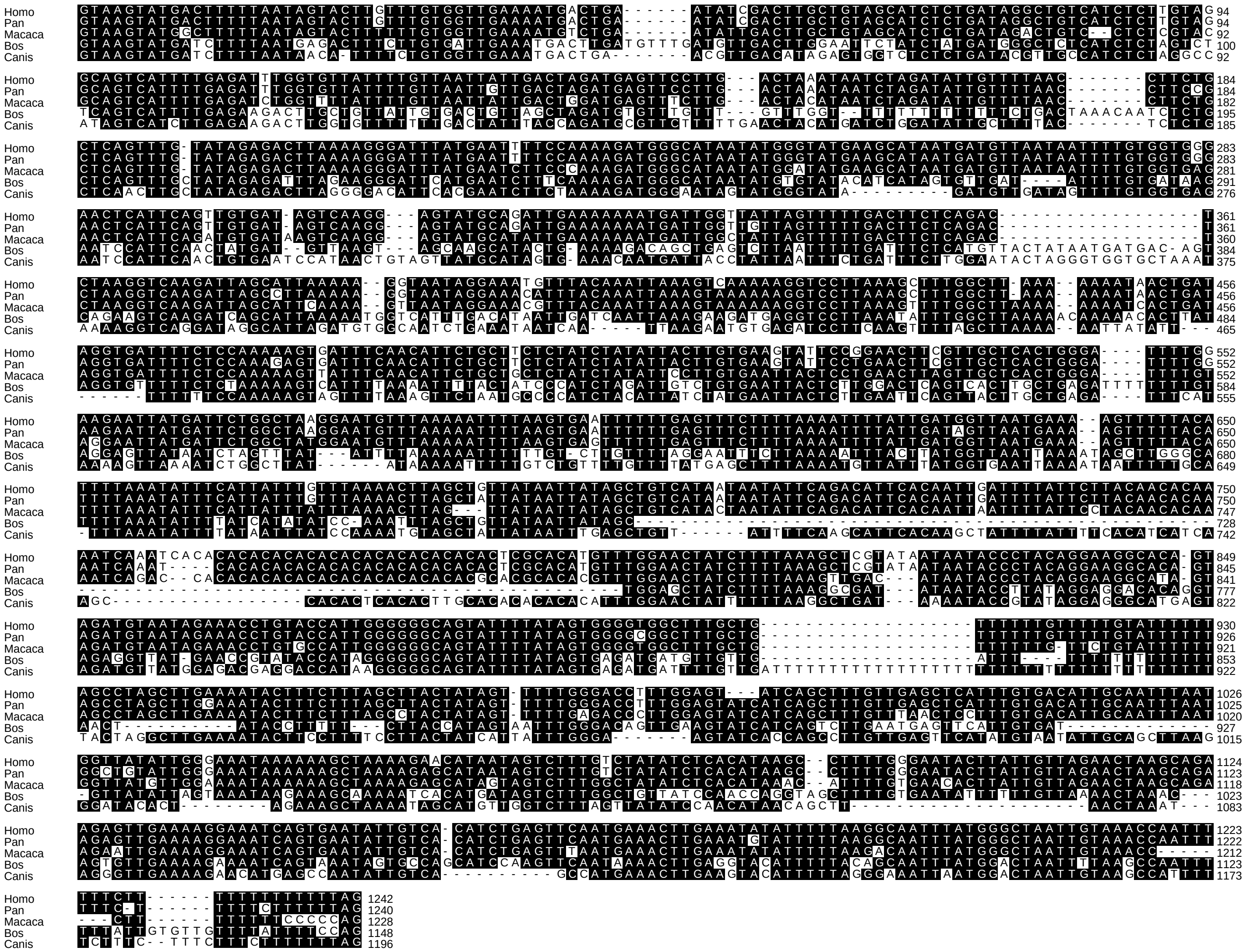

ENSG00000164400 intron 2

Description: Granulocyte-macrophage colony-stimulating factor precursor (CSF2)
Intron number: 2
Human chromosome: 05
Intron start (bp): 131437715
Human intron length : 687
Intron alignment length: 770
Flanking exons length (upstream/downstream): 42/126
SNP density: 0.010189
K tree score: 0.0542
Scaling factor: 1.1456
Human-chimpanzee distance: 0.007325
Total primate branch length: 0.0804

ENSG00000164400 exon 2

```
Homo    AATGAAACAGTAGAAGTCATCTCAGAAATGTTTGACCTCCAG 42
Pan     AATGAAACAGTAGAAGTTGTCTCAGAAATGTTTGACCTCCAG 42
Macaca  AATAAAACCGTAGAAGTCGTCTCAGAAATGTTTGACCTCCAG 42
Bos     AATGACAC---AGAAGTCGTCTCTGAAAAGTTTGACTCCCAG 39
Canis   AATAAAGCAGTAAAAGTGGTCTCTGAAGTGTTTGACCCTGAG 42
```

ENSG00000164400 exon 3

```
Homo    GAGCCGACCTGCCTACAGACCCGCCTGGAGCTGTACAAGCAGGGCCTGCGGGGCAGCCTCACCAAGCTCAAGGGCCCCTT 80
Pan     GAGCCGACCTGCCTACAGACCCGCCTGGAGCTGTACAAGCAGGGCCTGCGGGGCAGCCTCACCAAGCTCAAGGGCCCCTT 80
Macaca  GAGCCGAGCTGCCTACAGACCCGCCTGGAGCTGTACAAGCAGGGCCTGCAGGGCAGCCTCACCAAGCTCAAGGGCCCCTT 80
Bos     GAACCAACGTGCCTGCAGACTCGCCTGAAGCTGTACAAGAACGGCCTGCAGGGCAGCCTCACTAGTCTCATGGGCTCCTT 80
Canis   GGGCCAACATGCCTGGAGACCCGCCTACAGCTGTACAAGGAGGGCCTGCAGGGCAGCCTCACCAGCCTCAAGAATCCCTT 80

Homo    GACCATGATGGCCAGCCACTACAAGCAGCACTGCCCTCCAACCCCG 126
Pan     GACCATGATGGCCAGCCACTACAAGCAGCACTGCCCTCCAACCCCG 126
Macaca  GACCATGATGGCCAGCCACTACAAGCAGCACTGCCCTCCAACCCCG 126
Bos     GACCATGATGGCCACCCACTACGAGAAACACTGCCCACCCACCCCG 126
Canis   AACCATGATGGCCAATCACTATAAGCAGCACTGTCCCCCTACCCCG 126
```

ENSG00000164400 intron 2

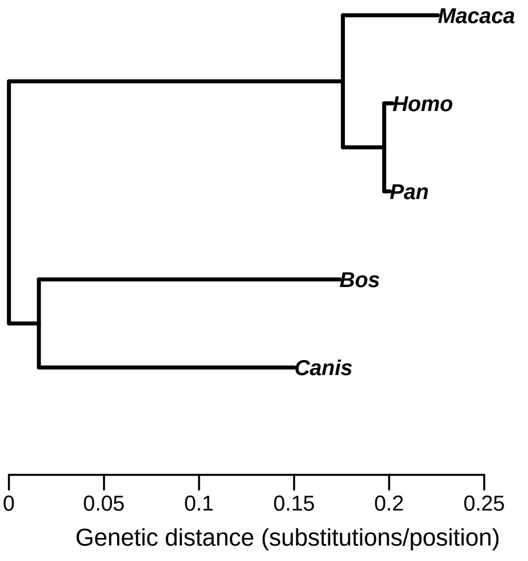

ENSG00000164400 intron 2

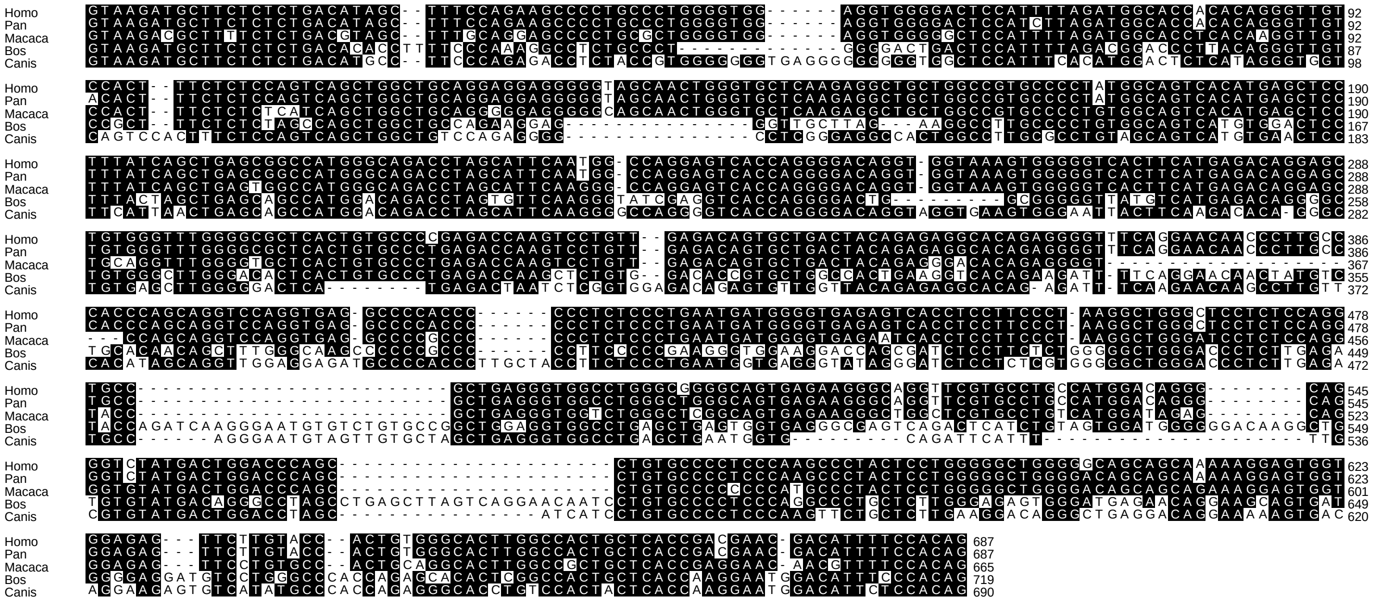

ENSG00000151360 intron 3

Description: Probable allantoicase (ALLC)
Intron number: 3
Human chromosome: 02
Intron start (bp): 3704021
Human intron length : 1313
Intron alignment length: 1656
Flanking exons length (upstream/downstream): 88/126
SNP density: 0.006855
K tree score: 0.0992
Scaling factor: 0.3987
Human-chimpanzee distance: 0.013874
Total primate branch length: 0.0804

ENSG00000151360 exon 3

```
Homo    AGTGACAGCCCGTGCTTCAAAGAGCATGAATATACGGAGTTTGGGAAATGGATGGATGGCTGGGAGACCAGGAGGAAAAG 80
Pan     AGTGACAGCCCGTGCTTCAAAGAGCATGAATATACGGAGTTTGGGAAATGGATGGATGGCTGGGAGACCAGGAGGAAAAG 80
Macaca  AGTGACAGCCCGTGCTTTAAAGAGCGTGAATATACTGAGTTTGGGAAATGGATGGATGGCTGGGAGACCAGAAGGAAAAG 80
Bos     AGGGACAACCCAAGTTTTAAAGAACACGAGTACACTGAGTTTGGGAAATGGATGGATGGATGGCAGACCAGAAGGAAAAG 80
Canis   AGTGACAGCCCAAGCTTCAGAGAACATGAATACACTGAGTTTGGGAAGTGGATGGATGGATGGGAGACCAGAAGGAAGAG 80

Homo    GATTCCAG 88
Pan     GATTCCAG 88
Macaca  GATTCCAG 88
Bos     GATTCCAG 88
Canis   GATTCCAG 88
```

ENSG00000151360 exon 4

```
Homo    GTCACGACTGGTGTGTCCTCAGGCTGGGGATCCAAGGAGTCATCCGGGGCTTCGACGTGGACGTTTCTTACTTCACGGGA 80
Pan     GTCATGACTGGTGTGTCCTCAGGCTGGGGATCCAAGGAGTCATCCGGGGCTTCGACGTGGACGTTTCTTACTTCACGGGA 80
Macaca  GTCACGACTGGTGCGTCCTCAGGTTGGGGATCCAAGGAGTCATCCGGGCCTTCGACGTGGACGTTTCTTATTTCACGGGA 80
Bos     GTCACGACTGGTGCGTGGTCCAGCTGGGCATCCAGGGAGTTATCCGGGGCTTCGACGTGGACACGTCCTACTTCACGGGA 80
Canis   GTCACGACTGGTGCATCATCAAGCTGGGGATACAAGGGATTATCCGGGGCTTTGATGTGGATATTTCTTACTTCATGGGA 80

Homo    GATTACGCTCCTCGAGTGTCCATTCAAGCAGCAAACTTGGAAGAAG 126
Pan     GATTATGCTCCTCGAGTGTCCATTCAAGCAGCAAACTTGGAAGAAG 126
Macaca  GATTACGCTCCTCGAATGTCAATTCAAGCAGCAAACTTGGAAGAAG 126
Bos     GATCACGCCCCTCGAGTGTCGATTCAAGCGGCAAACTTCGAAGAAG 126
Canis   GATTATGCTCCTCGAATGTCAATTCAAGCAGCAAACTTGGAAGAAG 126
```

ENSG00000151360 intron 3

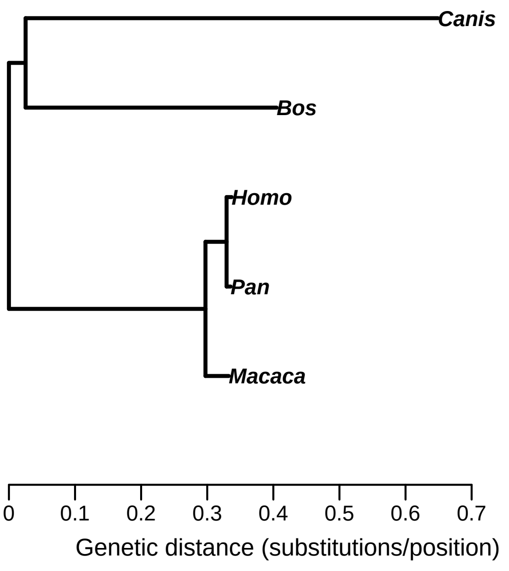

ENSG00000151360 intron 3

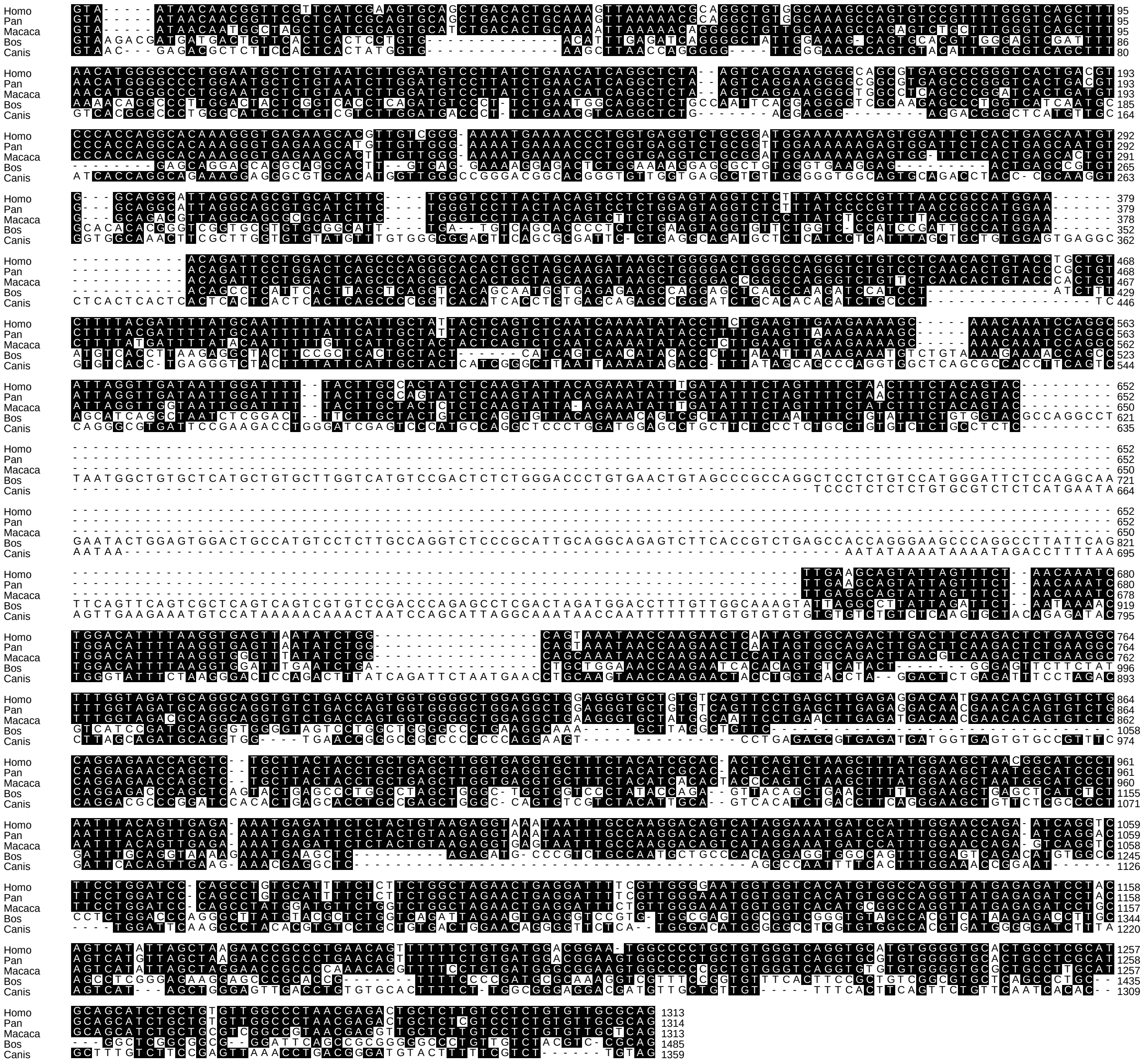

ENSG00000151576 intron 1

Description: queuine tRNA-ribosyltransferase domain containing 1 (QTRTD1)
Intron number: 1
Human chromosome: 03
Intron start (bp): 115266995
Human intron length : 770
Intron alignment length: 802
Flanking exons length (upstream/downstream): 200/56
SNP density: 0.001299
K tree score: 0.0738
Scaling factor: 1.0979
Human-chimpanzee distance: 0.018467
Total primate branch length: 0.0803

ENSG00000151576 exon 1

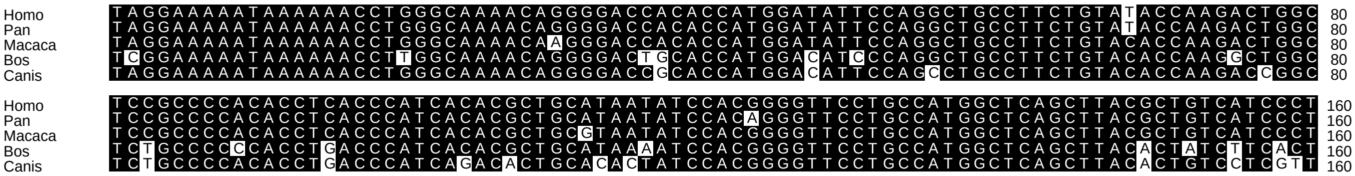

ENSG00000151576 exon 2

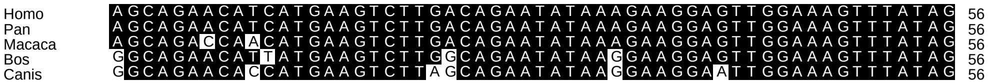

ENSG00000151576 intron 1

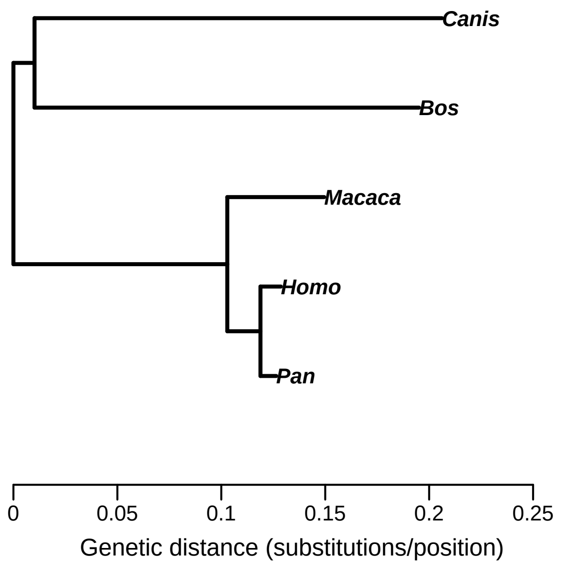

ENSG00000151576 intron 1

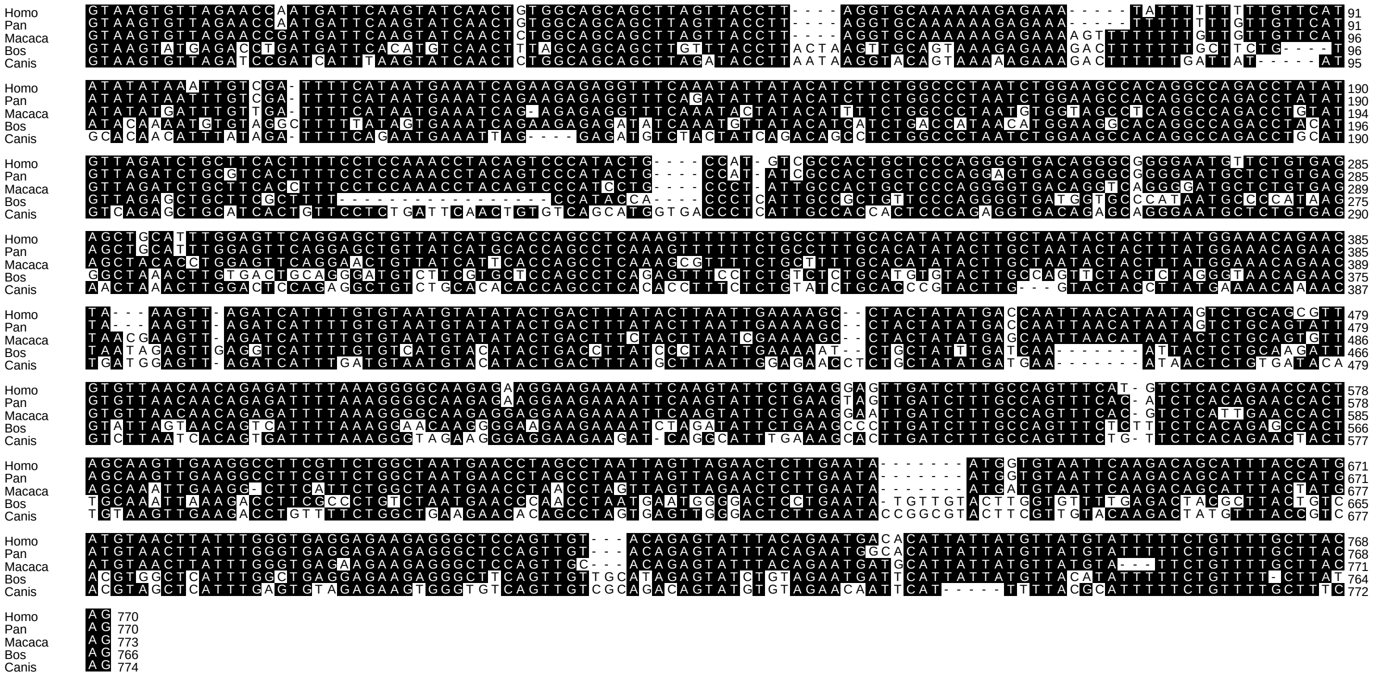

ENSG00000142546 intron 1

Description: eNOS interacting protein (NOSIP)
Intron number: 1
Human chromosome: 19
Intron start (bp): 54755109
Human intron length : 582
Intron alignment length: 628
Flanking exons length (upstream/downstream): 70/106
SNP density: 0.000000
K tree score: 0.0916
Scaling factor: 0.6241
Human-chimpanzee distance: 0.010401
Total primate branch length: 0.0802

ENSG00000142546 exon 1

```
Homo    ATGACGCGGCATGGCAAGAACTGCACCGCAGGGGCCGTCTACACCTACCACGAGAAGAAGAAGGACACAG  70
Pan     ATGACGCGGCATGGCAAGAACTGCACCGCAGGGGCCGTCTACACCTACCACGAGAAGAAGAAGGACACAG  70
Macaca  ATGACACGGCACGGCAAGAACTGCACCGCGGGGGCCGTCTACACCTACCACGAGAAGAAGAAGGACACAG  70
Bos     ATGACGCGGCACGGCAAGAACTGCACAGCAGGGGCCGTCTACACCTACCATGAGAAAAAGAAGGACACAG  70
Canis   ATGACACGACATGGCAAGAATTGCACGGCAGGGGCCGTCTATACCTACCACGAGAAGAAGAAGGACACAG  70
```

ENSG00000142546 exon 2

```
Homo    CGGCCTCGGGCTATGGGACCCAGAACATTCGACTGAGCCGGGATGCCGTGAAGGACTTCGACTGCTGTTGTCTCTCCCTG  80
Pan     CGGCCTCGGGCTACGGGACCCAGAACATTCGACTGAGCCGGGATGCCGTGAAGGACTTCGACTGCTGTTGTCTCTCCCTG  80
Macaca  CGGCCTCAGGCTATGGGACCCAGAACATTCGACTGAGCCGGGATGCCGTGAAGGACTTCGACTGCTGTTGTCTGTCCCTG  80
Bos     CGGCCTCAGGCTATGGCACCCAGAACATTCGACTGAGCCGGGATGCCGTCAAGGACTTCGACTGCTGCTGCCTCTCGCTG  80
Canis   CGGCCTCAGGGTATGGGACCCAGAACATTCAACTGATCCGGGATGCCGTCAAGGACTTCAATTGCTGCTGCCTCTCTCTG  80

Homo    CAGCCTTGCCACGATCCTGTTGTCAC  106
Pan     CAGCCTTGCCACGATCCTGTTGTCAC  106
Macaca  CAGCCTTGCCACGATCCTGTTGTCAC  106
Bos     CAGCCCTGCCACGACCCTGTCGTCAC  106
Canis   CAGGCCTGCCATGATCCTGTCGTCAC  106
```

ENSG00000142546 intron 1

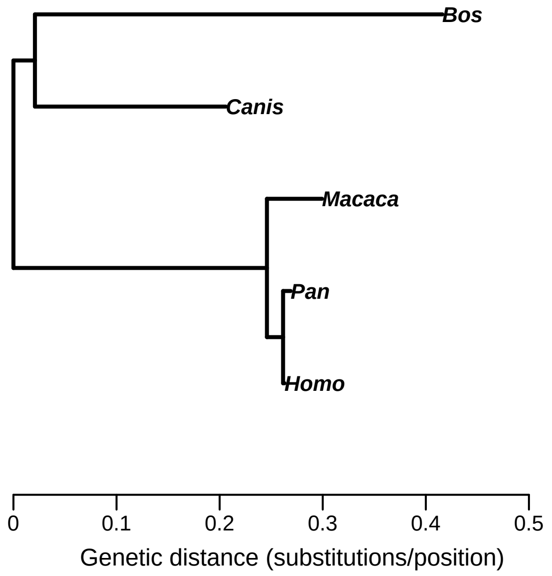

ENSG00000142546 intron 1

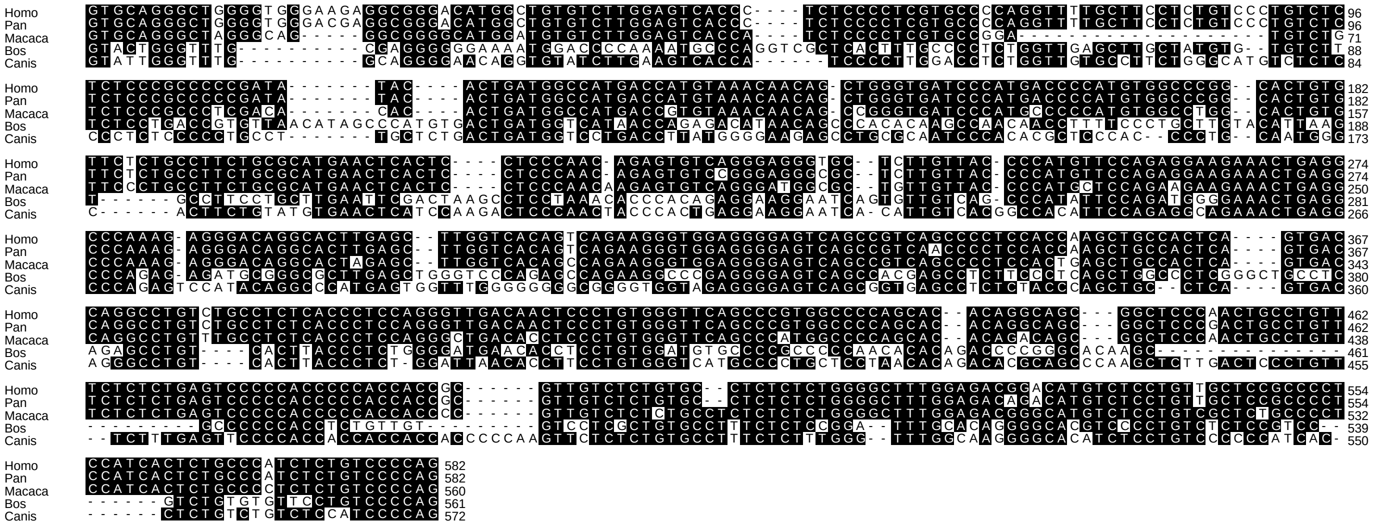

ENSG00000105948 intron 13

Description: tetratricopeptide repeat domain 26 (TTC26)
Intron number: 13
Human chromosome: 07
Intron start (bp): 138513589
Human intron length : 212
Intron alignment length: 217
Flanking exons length (upstream/downstream): 89/84
SNP density: 0.000000
K tree score: 0.0907
Scaling factor: 0.4632
Human-chimpanzee distance: 0.009454
Total primate branch length: 0.0802

ENSG00000105948 exon 13

```
Homo    ATACAATACCAGGGAGGCAGTGCATGGCTTCCTGTTTCTTCCTGCTTAAGCAATTTGATGATGTTTTGATTTACCTCAAC 80
Pan     ATACAATACCAGGGAGGCAGTGCATGGCTTCCTGTTTCTTCCTGCTTAAGCAATTTGATGATGTTTTGATTTACCTCAAC 80
Macaca  ATACAATACCAGGGAGGCAGTGCATGGCTTCCTGTTTCTTCCTGCTTAAGCAATTTGATGATGTCTTGATTTACCTCAAC 80
Bos     ATACAATACCAGGAAGGCAGTGCATGGCTTCCTGTTTCTTCCTGCTTAAGCAATTTGATGATGTCTTGATTTACCTCAAC 80
Canis   ATACAATACCTGGGAGGCAGTGCATGGCTTCTTGTTTCTTTCTGCTTAAGCAATTCGATGATGTCTTGATTTACCTCAAC 80

Homo    TCATTTAAG 89
Pan     TCATTTAAG 89
Macaca  TCATTTAAG 89
Bos     TCATTCAAG 89
Canis   TCATTTAAG 89
```

ENSG00000105948 exon 14

```
Homo    AGTTACTTCTATAATGATGACATCTTTAACTTTAATTATGCCCAAGCCAAAGCTGCAACAGGCAATACCAGTGAGGGCGA 80
Pan     AGTTACTTCTATAATGATGACATCTTTAACTTTAATTATGCCCAAGCCAAAGCTGCAACAGGCAATACCAGTGAGGGCGA 80
Macaca  AGTTACTTCTATAATGATGACATCTTTAACTTTAATTATGCCCAAGCCAAAGCTGCAACAGGCAATACCAGTGAGGGTGA 80
Bos     AGTTACTTCTATAATGATGACATCTTTAACTTTAATTATGCCCAAGCCAAAGCTGCAACTGGCAATACCAGTGAGGGTGA 80
Canis   AGTTACTTTTACAATGATGACATCTTTAACTTTAATTATGCCCAAGCCAAAGCTGCAACAGGCAATACCAGCGAGGGTGA 80

Homo    AGAG 84
Pan     AGAG 84
Macaca  AGAG 84
Bos     AGAG 84
Canis   AGAG 84
```

ENSG00000105948 intron 13

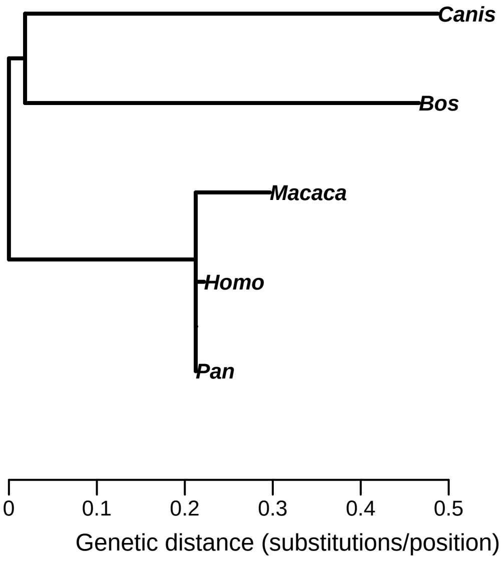

ENSG00000105948 intron 13

```
Homo    GTCAGTATAGAATTATCTATAAGACTTTATATACAAAATACTA--TGGAATGTGTCAGAGAAGATAAGATACCCTTAGAGATGGCCCATTTTCTGTGGTG 98
Pan     GTCAGTATAGAATTATCTACAAGACTTTATATACAAAATACTA--TAGAATGTGTCAGAGAAGATAAGATACCCTTAGAGATGGCCCATTTTCTGTGGTG 98
Macaca  GTCAGTATAGAATTATCTACAAGATTTTATATACAAAATACTG--TACAATGTATCAAAAAAAATAAGATATCGTTAGAGATGGCCCATTTTCTATGGTG 98
Bos     GTCAGTGTAGAGTTTTCTAGGTGATTTTGTATACAAAATACTC--TAATATGTCAC-----AGATAAGATACTTTTGGAGATGACCCTTTTACTGTGGGA 93
Canis   GTCAGTATAGAAGTTTCTAGATGAGTTTGTCTACCAAATACTATTTATAATGTGTCAG---AAGTAAGATAGTTTTAGAGATGGCCCTTTTACTACAATG 97

Homo    GCATTAGTGGCATCAACTAA-TCCTTAAGGTTAGTTTTGTGTTTATCTGGTATAACTGATGG--TTATATTTTATATGTTAATTCTCAGTGTGACCTGTT 195
Pan     GCATTAGTGGCATCAACTAA-TCCTTAAGGTTAGTTTTGTGTTTATCTGGTATAACTGATGG--TTATATTTTATATGTTAATTCTCAGTGTGACCTGTT 195
Macaca  GCATTAGTGGCATCAACTAA-TCCTTAAGGTTAGTTTTGTGTTTACCTGGTATAACTGATGGTTTTATATTTTGTATGTTCATTCTCAATGTGACCTGTT 197
Bos     GTAGTAGTGG------------------------------------------TAACTGTTGTTTTTATATTTTATATGTTAAAGCTCAATGTGATCTGTT 151
Canis   GCATTGATGGTGTCTGCTAATTCCTTACGATTATCTTTGAGTTTACCCAGTATAACCGATAT-GTTATATATTCTATGTTAATACTCAATGTGACCTATT 196

Homo    CTTGGGATGTTTTTCAG 212
Pan     CTTGGGATGTTTTTCAG 212
Macaca  CTTGGGATGTTTTTCAG 214
Bos     CTTGGGATGTTTTTCAG 168
Canis   CTTGGGATGTTTTTCAG 213
```

ENSG00000138231 intron 1

Description: Lariat debranching enzyme (DBR1)
Intron number: 1
Human chromosome: 03
Intron start (bp): 139375159
Human intron length : 972
Intron alignment length: 1247
Flanking exons length (upstream/downstream): 197/125
SNP density: 0.003086
K tree score: 0.0638
Scaling factor: 0.7128
Human-chimpanzee distance: 0.012956
Total primate branch length: 0.08

ENSG00000138231 exon 1

```
Homo    TGGATAAGATCTATGAGACGCTGGCGCTGGCAGAGCGGCGCGGCCCGGGGCCTGTCGACCTCTTGCTGTGCTGCGGCGAC 80
Pan     TGGATAAGATCTATGAGACGCTGGCGCTGGCAGAGCGGCGCGGCCCGGGGCCTGTCGACCTCTTGCTGTGCTGCGGCGAC 80
Macaca  TGGATAAAATCTATGAGACGCTGGCGCTGGCGGAGCGGCGCGGCCCGGGGCCTGTCGACCTCTTGCTGTGCTGCGGCGAC 80
Bos     TGGACAAGATCTATGAGACGCTGGCGCTGGCGGAGCGGCGCGGCCCGGGGCGGATAGACCTTCTGCTCTGCTGCGGCGAC 80
Canis   TAGACAAGATCTATGAGACGCTGGCGCTGGCGGAGAGGCGCGGCCCCGGGCCGATAGATCTTCTGCTGTGCTGCGGCGAC 80

Homo    TTCCAGGCGGTGCGCAACGAGGCGGATCTACGCTGCATGGCCGTGCCGCCCAAGTATCGTCACATGCAAACCTTCTACAG 160
Pan     TTCCAGGCGGTGCGCAACGAGGCGGATCTACGCTGCATGGCCGTGCCGCCCAAGTATCGTCACATGCAAACCTTCTACAG 160
Macaca  TTCCAGGCGGTGCGCAACGAGGCGGACCTACGCTGCATGGCCGTGCCGCCCAAGTACCGCCACATGCAAACCTTCTACAG 160
Bos     TTCCAGGCAGTGCGCAACGAGGCCGACCTGCGTTGCATGGCCGTGCCGCCCAAGTACCGCCACATGCAAACCTTCTACAG 160
Canis   TTCCAGGCGGTGCGCAACGAGGCCGACCTGCGTTGCATGGCCGTCCCGCCCAAGTACCGCCACATGCAGACCTTCTACAG 160
```

ENSG00000138231 exon 2

```
Homo    GTATTACTCTGGAGAGAAAAAGGCTCCAGTTCTCACGCTCTTCATTGGGGGAAACCATGAAGCCTCAAATCATTTGCAAG 80
Pan     GTATTACTCTGGAGAGAAAAAGGCCCCAGTTCTCACACTGTTCATTGGGGGAAACCATGAAGCCTCAAATCATTTGCAAG 80
Macaca  GTATTACTCTGGAGAGAAAAAGGCCCCAGTTCTCACGCTCTTCATTGGGGGAAACCATGAAGCCTCAAATCATTTGCAAG 80
Bos     GTATTACTCTGGGGAGAAGAAGGCCCCAGTTCTCACGATTTTCATTGGGGGAAACCATGAAGCCTCAAATCATTTGCAAG 80
Canis   GTATTACTCTGGAGAGAAAAAGGCCCCAGTTCTCACAATCTTCATTGGGGGAAACCATGAAGCCTCAAATCATTTGCAAG 80

Homo    AGTTACCCTATGGTGGCTGGGTGGCACCAAACATTTATTATTTAG 125
Pan     AGTTACCCTATGGTGGCTGGGTGGCACCAAACATTTATTATTTAG 125
Macaca  AGTTACCCTATGGTGGCTGGGTGGCACCAAACATTTATTATTTAG 125
Bos     AGTTACCCTACGGTGGCTGGGTAGCACCAAACATTTATTATTTAG 125
Canis   AGTTACCCTATGGTGGCTGGGTGGCACCAAACATTTATTATTTAG 125
```

ENSG00000138231 intron 1

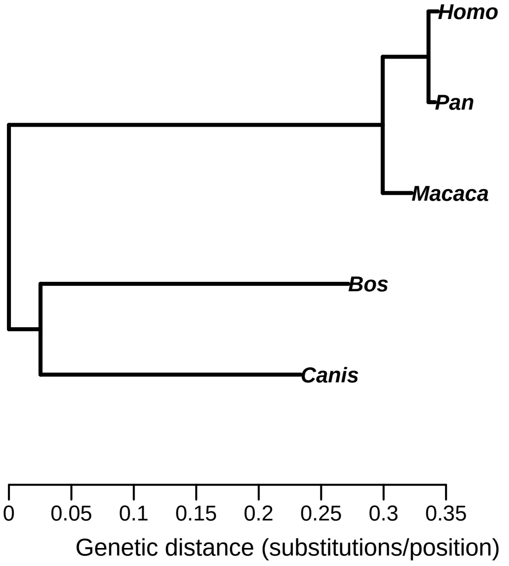

ENSG00000138231 intron 1

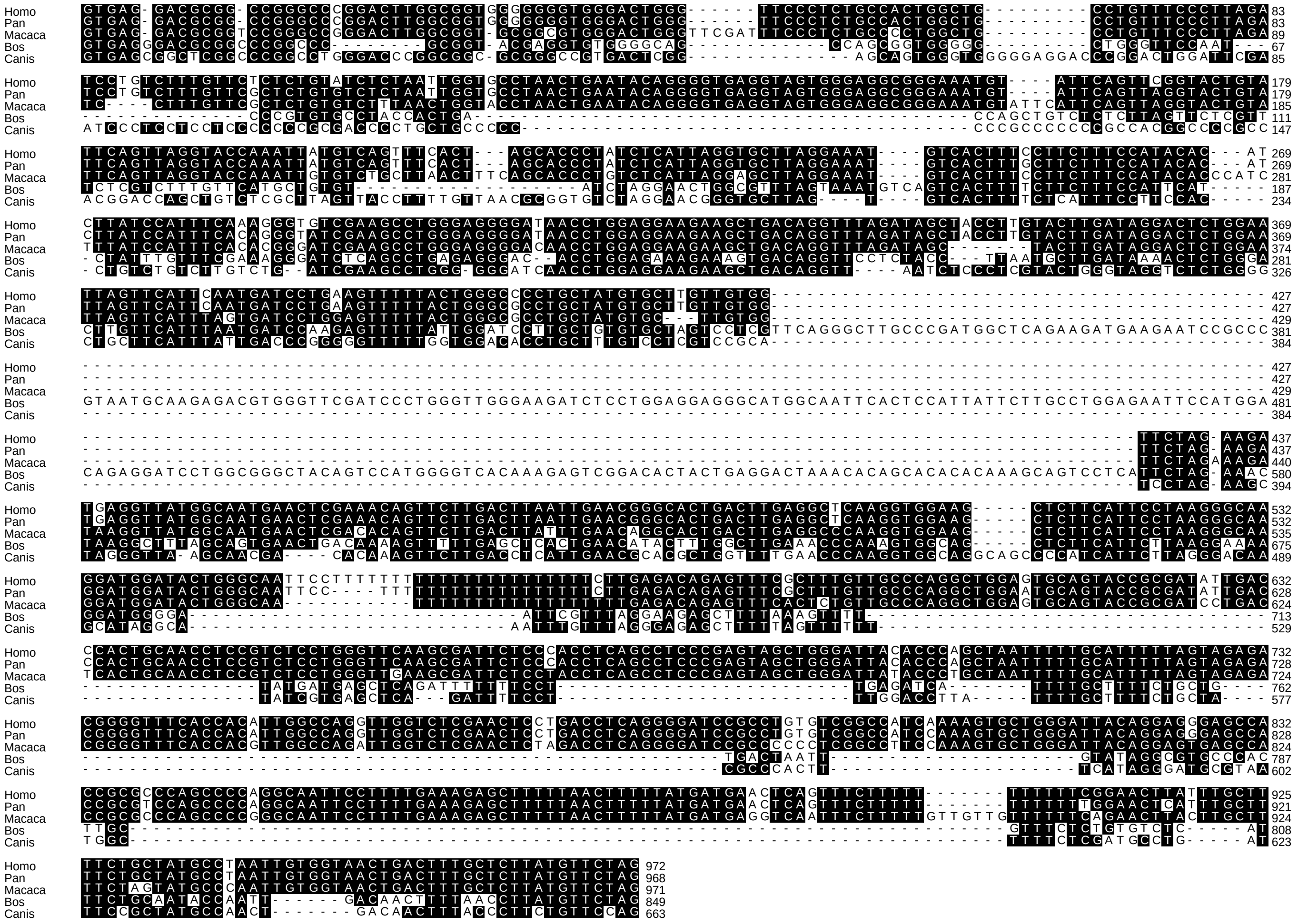

ENSG00000124207 intron 12

Description: Exportin-2 (CSE1L)
Intron number: 12
Human chromosome: 20
Intron start (bp): 47127009
Human intron length : 1496
Intron alignment length: 1625
Flanking exons length (upstream/downstream): 85/62
SNP density: 0.004679
K tree score: 0.0459
Scaling factor: 0.545
Human-chimpanzee distance: 0.008764
Total primate branch length: 0.0799

ENSG00000124207 exon 12

```
Homo    CATGGAATTACACAAGCAAATGAACTTGTAAACCTAACTGAGTTCTTTGTGAATCACATCCTCCCTGATTTAAAATCAGC 80
Pan     CATGGAATTACACAAGCAAATGAACTTGTAAACCTAACTGAGTTCTTTGTGAATCACATCCTCCCTGATTTAAAATCAGC 80
Macaca  CATGGAATTACACAAGCAAATGAACTTGTAAACCTAACTGAGTTCTTTGTGAATCACATCCTCCCTGATTTAAAATCAGC 80
Bos     CATGGGATTACACAAGCAAATGAGCTTGTAAACCTCACTGAATTCTTCGTGAATCACATTCTCCCTGATTTAAAATCAGC 80
Canis   CATGGAATTACACAAGCAAATGAACTTGTAAACCTGACTGAGTTCTTTGTGAATCATATTCTTCCTGATTTAAAATCTGC 80

Homo    TAATG 85
Pan     TAATG 85
Macaca  TAATG 85
Bos     CAATG 85
Canis   TAATG 85
```

ENSG00000124207 exon 13

```
Homo    TGAATGAATTTCCTGTCCTTAAAGCTGACGGTATCAAATATATTATGATTTTTAGAAATCAA 62
Pan     TGAATGAATTTCCTGTCCTTAAAGCTGACGGTATCAAATATATTATGATTTTTAGAAATCAA 62
Macaca  TGAATGAATTTCCTGTCCTTAAAGCTGATGGTATCAAATATATTATGATTTTTAGAAATCAA 62
Bos     TAAATGAGTTTCCTGTTCTTAAAGCTGATGGCATCAAATACATTATGATTTTTAGGAATCAA 62
Canis   TGAATGAATTCCCTGTCCTTAAAGCTGATGGTATCAAATACATTATGATTTTTAGGAATCAA 62
```

ENSG00000124207 intron 12

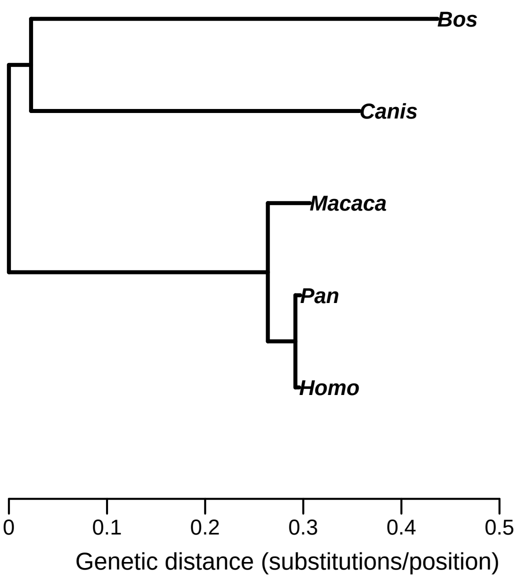

ENSG00000124207 intron 12

```
Homo    GTGAGTT----GTGAAATTCTTGCTTTTTTTACAGCTTGCCACACTATACCTG-AATTCAAAAAACAGCTTCCTATCCTTGTTCCCTTTTGTTCTTTTTT 95
Pan     GTGAGTT----GTGAAATTCTTGCTTTTTTTACAGCTTGCCACACTATACCTG-AATTCAAAAAACGGCTTCCTATCCTTGTTCCCTTTTGTTC--TTTT 93
Macaca  GTGAGTT----GTGAAATTCTTGCTTTTTTTACAGCTTACCACACTATATCTGCAATTAAAAACATGGCTTCCTATCCTTGTTCCCATTCATTC------ 90
Bos     GTGAGTT----GTGAAA----TGTTTTTAATACAAC---TTACAGTGTACCTGTAATT------------------------------------------ 47
Canis   GTAAGTTTGTGAAAAAATTTTTATGCTATGTACAAC-----------AATTTTTACTTGAGGG------------------------------------- 52

Homo    TTTTTTTTTTTTTTCTTTTGAGATGGTGTCTTACTCTGTTACCCAGGCTGGAGTGCAGTGGCACAGTCTCTGCTCACTGCAACTCCTGCCTCCTGGGTTCA 195
Pan     TTTTTTTTTTTTTTCTTTTGAGACGGTGTCTTACTCTGTTACCCAGGCTGGAGTGCAGTGGCACAGTCTCTGCTCACTGCAACTCCTGCCTCCTGGGTTCA 193
Macaca  -TTTTTTTCTTTTCTTTTGAGATGGTGTCTCACTC--TTACCCAGGCTGGAGTGCGGTGGCGTGGTCTCCACTCACTGCAACCCCTGCCTCCTGCGTTCA 187
Bos     -------------------------------------------------------------------------CTTA----------------------- 51
Canis   --------TTTTTGTTTTTAAGTAATTTTT-------GCACCCAACGTGGGGCTCAG-------------ACTCA---CAACCCTGAACATCAAGATTCA 121

Homo    AGCGATTCTCCTGCCTCAGCTTCCCAAGCAGCTGGGACTACAGGCACGTACCACCATGCCCGGCTAATTTTTGTATTTTTAGTAGAGACGGGGTTTCCCC 295
Pan     AGCGATTCTCCTGCCTCAGCTTCCCAAGCAGCTGGGACTACAGGCACGTACCACCATGCCCGGCTAATTTTTGTATTTTTAGTAGAGACGGGGTTTCCCC 293
Macaca  AATTATTCTCCTGCCTCAGCTTACCAAGCAGCTGGGACTACAGGCACATACCAGTATGCCTGGCTAATTTTTATATTTTTTGTAGAGACAGGGTTTCCCC 287
Bos     ---------------------------------------------CGTGCACTGTTATTTTAAACTA-------CCAAATGAGTGC--------------T 86
Canis   CAT-ACTCTACTGACTGAGCCAGCCAGGTGCC-------------CCTGCACTGTTATTTTAGACTA-------CAAAATAAGTAA--------------C 187

Homo    ATGTTGGCCAGGCTGGTCTCAAACTCCTGACCTCAGGTGATCCACCTGCGTCGGCCTGCCAAAGTGCTGGGATTGCAGGCGTGAGCCTCCATGCCCTGCC 395
Pan     ATGTTGGCCAGGCTGGTCTCAAACTCCTGACCTCAGGTGATCCACCTGCGTCGGCCTGCCAAAGTGCTGGGATTGCAGGCGTGAGCCTCCATGCCCTGCC 393
Macaca  TTGTTGGCCAGGCTGGTCTCGAACTCCTAACCTCAGGTGATCCACCTGCGTTGGCCTCCCAAAGTGCTGGGATTGCAGGCATGAGCCTCCATGCCCTGCC 387
Bos     GTCGTGG-------------------CTCTGTATCA------------------------TTCATGATACTGAAAACAGTGGCTCCTATCCTTG---------- 137
Canis   ATTATGGCAAAAGTCATAATGGGGACTGTACATCA------------------------TCAGGATACTCAGAAAATTGGCCTCTACCCTTG-----TTGC 259

Homo    TATTATTTCTTTTATTGAATAAAATCTCAAATGGTTTCCCCAGATCTTGTCCATGTGAAATTAATTATTGATAAAGAAAATATTGCTTGATTAGGAGGAA 495
Pan     CATTATTTCTTTTATTGAATAAAATCTCAAATGGTTTCCCCAGATCTTGTCCATGTGAAATTAATTATTGATAAAGAAAATATTGCTTGATTAGGAGTAA 493
Macaca  TATTATTTCTTTTATT-AATAAAATCTCAAATGGTTTCCCCAGGTCTTGTCCATGTGAAATTAATTATTGAT----AAAATATTGCTTGATTAGGAAGAA 482
Bos     --CAGTTACTTCTGTCGGATAAAATTTCTAATGGGTGCCTTCAATCTTGCCCAGGTGAAATTAACTTTGCCTAAAGAAAATGTTGTTTATATAGAAGGAA 235
Canis   AGTTATTTCTTTTATTGAATAAAATCTCAGATGCTTTCCCCAGATCTTGCCTAGTTGAAATCAACTTTCTCTA---ATGATATTGCTTGTGTAGAAGGAA 356

Homo    AATAGGAATAAATACAACTAAACTGTAACTCAGCTTTGCTAC-CTAGTGTAT---AAATAAGGATTATACATTGGC-----TTCCTTTTCTTTTTTATTT 586
Pan     AATAGGAATAAATACAACTAAACTGTAACTCAGCTTTGCTAC-CTAGTGTAT---AAATAAGGATTATACATTGGC-----TTCCTTTTCTTTTTTATTT 584
Macaca  AATAGGAATAAGTACAACTAAACTGTAACTCAGTTTTGCTAC-CTAGTGGAT---AAATA---ATTATACATTGGC-----TTC-----CTTTTTTATTT 565
Bos     AATAGAAACAAGTGAGACTGAATTGTCATTCAGCTTTGTTACCCGAATAATTTCACACTAGAAATCATACTTTGGTTTTTGTTTTCCTTCTTTCTCATTT 335
Canis   AATAGAAACAACTAAGATGGAACTGTAGCTCAGCTTTGTTACGCTGACGCAT---AATCTAGGAAT-TATACTTGT-----TTTCTTGCCCTTCTCATTT 447

Homo    ----GAAAGACGAATTCTTAAAAAATTGGAGTACATTTCAAAGTACTAGAAAATTGATAAATATTAAGGATATCTTTTGGCCGGGTGCGGTGGCTCACACC 682
Pan     ----GAAAAACGAATTCTTAAAAAATTGGAGTACATTTCAAAGTACTAGAAAATTGATAAATATTAAGGATATCTTTTGGCCAGGTGCAGTGGCTCACACC 680
Macaca  ----GAAAAATGAATTCTTAAAAAATTGGAGTACAGTTCAAAGAACTAGAAAATTGATAAATATTAAGGATATCTTTTGGCTGGGCGCAGTGGCTCACCCT 661
Bos     AAAAAAAAAAAGAACTCTTGGGAATTCCCTGG-CGGTCAAGTGGTTGGTACTCTGTGCTTCACTGCTGACGTCCTGGGTTCAGT------------------ 418
Canis   ----GAAAAATGAAATATTAAAAGTTGAAATA-AATTTAAAGTACT-TCAAACTGATAATTAAGGATAATTTCCTATGATTA----------------- 523

Homo    TGTAATCCCAGCACTTTGGGAGACTGAGGCGGGAGGATCGTGAGGTCAAGAGATTGAGACCAGCCTGGTCAAAATGGTGAAACCCCGTCTCTACTAAGAA 782
Pan     TGTAATCCCAGCACTTTGGGAGACTGAGGCGGGAGGATCGTGAGGTCAAGAGATTGAGACCAGCCTGGTCAAAATGGTGAAACCCCGTCTCTACTAAGAA 780
Macaca  TGTAATCCCAGCACTTTGGGAGGCCAAGGTGGGCGGATCATGAGATCAAGAGATCGAGACCAACCTGGCCAAAATGGTGAAACCCCGTCTCTACTAAAAA 761
Bos     ---------------------------------------------------------ATCTGGTCAGGGAA------------------TTAAGAG 439
Canis   ------------------------------------------------TGAAAGTATATTGATCAAGATA---------CATTATATATTCCCTT 561

Homo    TACAAAAACTAGCCGGGCGTGGTGGCGTGTGCCTGTAATCCCAGCTATTTGGGAGGCTGAGGCAGGAGAATCGCTTGAATCCAGGAGGCAGGGGTTGCAG 882
Pan     TACAAAAACTAGCCGGGCGTGGTGGCGTGTGCCTGTAATCCCAGCTATTTGGGAGGCTGAGGCAGGAGAATCGCTTGAATCCAGGAGGCAGGGGTTGCAG 880
Macaca  TAAAAAAACTAGCCGGACGTGGTGGCACATGCCTGTAAGCCCAGCTACTCGGGAGGCTGAGGCAGGAGAATCGCTTGAATCCAGGAGGCAGGGGTTGCAG 861
Bos     CTCACAACATGGCCAAAAATA---------------------------------------------------------------AAATAAAACTTGAAA 475
Canis   TTAAAAAACTTTTTGTATGTG--------------------AGGCACCTGGGTGGCTCAGTCAGTAAAGTGTC---------------CAGTTCTTGATC 626

Homo    TGAGCCGAGATCGTGCCACTGCACTCTAGCCTGGGATACAGAGCGAGACTCTGTCTC-----AAAAAAAAAAAATTAAGGATAGTTTCTGATGTTTATGAA 977
Pan     TGAGCCGAGATCGTGCCACTGCACTCTAGCCTGGGATACAGAGCGAGACTCTGTCTC-----AAAAAAAAAAAATTAAGGATAGTTTCTGATGTTTATGAA 975
Macaca  TGAGCTGAGATCGTGCCACTGCACTGTAGCCTGGGAGGCAGAGCGAGACTCTGTCTCAAAAAAAAAAAAAAAAATTAAGGATAGTTTCTGATGTTTATGAA 961
Bos     AAAAATGAACTCATAAAAATGGAA-----------------GTAAGTTTAAAGCAGTTGAGACTAATAGATGCTCAGGATACTTCCATATGTTTATGAA 557
Canis   TCAGCTCAGGTCTTGATCTTAGGTTT-----------------GTGAGTTCAAGCCCC-------------AAGTTAGGCTCCATGCTTGG---------- 687

Homo    TGTATTGATCTAGATCCAGATATATTCCTTTTTAAAAAAAAATA---TTTTAATGTAGGAAATTTATGTACACCCAAAGGTGAAGAGAATATTGTAATGAG 1074
Pan     TGTATTGATCTAGATCTAGATATATTCCTTTTTAAAAAAAAATA---TTTTAATGTAGGAAATTTATATACACCCAAAGGTGAAGAGAATATTGTAATGAG 1072
Macaca  TGTATTCCTCTAGATTCAGATATATTCTTTAAAAAAAAAAAAAAATTTTTAATGTGGGAAATTTATATACACCTAAAGGTGAAGAGAATATTGT--TACG 1059
Bos     AGTATG----TTAATCAAGACAGAC-----------------AGACATGTAAAGTTGTGGAGACTTAAAATCACTGCACAGC---GGGCATAACATAGAGGA 635
Canis   -GTGTGGGGCCTATCCTAAAAACAC----------AAAAAGGAAGCTTTCTGTGTGGAAATTTTAAGTACACTAAAAGG----GAGACTATTGTAATGAA 772

Homo    C--TCCCATGTGACCATCATTT----AGCTTCAAGAATTATTAACATTTGGTGATCTATTCCTTTCATTAATTTATTTC---------------------- 1147
Pan     C--TCCCATGTGACCATCATTT----AGCTTCAAGAATTATTAACATTTGGTGATCTATTCCTTTCATTAATTTATTTC---------------------- 1145
Macaca  C--TCCTATGTGACTATCATCT----AGCTTCAAGAATTATTAACATTTAGTGATCTATTCCTTTC----ATTTATTTC--------------------- 1128
Bos     C--TCCCTCGTCACCGTCACTTCAAGAGCTTTGACAACT------GCCCGATACTCTGTCCCTTTCACTAGTCAGCGT--------------------- 705
Canis   CAATCGTGTTTTACTGTCATCT----AGCTTTAATAATTTAAAATAACTGACCAATATTCTCTTTT--TCATTAATTTCCCATTAAAACAGTTTAAAGGA 866

Homo    ---CCTACCCACTA---------------GAATAATGTAAAGCAAATTCCAGCTAGAGAGACATGTTTCTAATTCAGAATAGCTTCACTTCTGGGAATGTA 1230
Pan     ---CCTACCCACTA---------------GAATAATGTAAAGCAAATTCCAGCTAGACAGACATGTTTCTAATTCAGAATAGCTTCACTTCTGGGAATGTA 1228
Macaca  ---CCTA-CCACTG---------------GAATAATGTAAAGCAAATTCCAGCTAGAGAGACGTGTTTCTAATTCAGAATAGCTTCACTTCTGGGAATGTA 1210
Bos     ---GCTTCCCACTGCTCGTGT-------------------------AAGCTCATCTGGAGAGGCCTGCTCCTGATTCAGAATAGCTTCGGTTCTGGGA----- 775
Canis   AATCCTAGCCATTGCTCACTTTGGCAGCATATATATTAAAGCAAATCTCAGCCAGAGAGACATGCTCCTGTTTCAATATAGCTTCACTTCTAGGAAAAAG 966

Homo    G-GTAGACATGCTCACCGATGC--ATGGTCCAGCTCATTTTTACTCAAT-----TATGTAATATGTGTTCCTTAGTCATGAAA---------CAGGCATG 1313
Pan     G-GTAGACATGCTCACCGATGC--ATGGTCCAGCTCATTTTTACTCAAT-----TATATAATATGTGTTCCTTTGTCATGAAA---------CAGGCATG 1311
Macaca  G-GTAGACATGCTCACCGATGC--ATGGTCCAGCTCATTTTTACTCAAT-----TATATAATATGTGTTCCTTTGTCATGAAA---------TAGGCATG 1293
Bos     --AGAGATACTGTCATTATTTCCTGTGGCCCAAATCATTTTTGTTCAAT-TACATAAGTAACGTATGTTCCTTTGTAGTGAAAATTAAAGTTAAAG---G 869
Canis   ATGATTATATGGTCATTAGTTT--ATGATCTAAATCATATTTATTCAGTTAATATAAGTAATGCATGTTCATTTGTAGTGAAA---------CAAG---G 1052

Homo    GGGAAAAATAAAATCATTTATAATCCTACCAAAATTAATGACTAAATAGTAAATCATAATATAATTAACTGTGC-------------------------- 1387
Pan     GGGAAAAATAAAATCATTTATAATCCTACCAAAATTAATGACTAAATAGTAAATCATAATATAATTAACCGTGC-------------------------- 1385
Macaca  AGGAAAAATAAAATTATTTATAATCCTACCAAAATTAATGGCTAAATAGTAAATCATTATATAATTAACTGTGC-------------------------- 1367
Bos     TGAAAAAACAGAATTATTCATAAACTTACCCAAGTTAATAGTTGTGTGGTGGATCGTTATATAATTAACTTGACATTATATAACTTAAATATAACTTGAC 969
Canis   AGAAAAAATAAAATTATTCATAATCTTACCAGAACTAATAGCAACATAGTAGATATTTAAATAATTAATTAAGC-------------------------- 1126

Homo    ----TTTAATTACTTGACATTT--------TATC-TTTTTTTATAACTCTGATGATGTC---ACTAATTTGGAAGTAATGATCCTTACCACTTAAGTAAC 1471
Pan     ----TTTAATTACTTGACATTT--------TATC-TTTTTTTATAACTCTGATGATGTC---ACTAATTTGGAAGTAATGATCCTTACCACTTAAGTAAC 1469
Macaca  ----TTTAATTACTTGACATTT--------TATCATTTTTTTGTAACTCTGATGATATC---ACTAATTTGGAAGTAATGATCCTTACCACT----TAAC 1448
Bos     GTTATATAATTACTTGGCATTTGCTTCCCAGAACCATGTTTTTAAAATCTGATAATATTGTGACTGATTTGAGAGTAG----------CAGTTAAATAAC 1059
Canis   ----TCCAATTACTTGACT----------------ACGTTTCAAAAATCTGATAAAATC---ACCAATCTGGTAGTAATAGTCCTTAGCACTTAAATAAC 1203

Homo    TGTGGCTTTCTGTTTTTTTATATAG 1496
Pan     TGTGGCTTTCTGTTTTTTTATATAG 1494
Macaca  TATGGCTTTCTGTTTTTTTA-ATAG 1472
Bos     TGTGACTTTTTATTTCTTTTAATAG 1084
Canis   TATGGTTTTATTTTTTTA--ATAG 1226
```

ENSG00000124827 intron 4

Description: Chorion-specific transcription factor GCMb (GCM2)
Intron number: 4
Human chromosome: 06
Intron start (bp): 10983153
Human intron length : 957
Intron alignment length: 1023
Flanking exons length (upstream/downstream): 126/939
SNP density: 0.004180
K tree score: 0.0355
Scaling factor: 0.9833
Human-chimpanzee distance: 0.025057
Total primate branch length: 0.0798

ENSG00000124827 exon 4

```
Homo    GCCAAGGGAGTTCATGATCATCCAAGACCAGAGAGCAAATCAGAGACAGAAGCTAGAAGAAGCGCCATCAAGAGACAAAT 80
Pan     GCCAAGGGAGTTCATGATCATCCAAGACCAGAGAGCAAATCAGAGACAGAAGCTAGAAGAAGCGCCATCAAGAGACAAAT 80
Macaca  GCCAAGGGAGTTCATGATCACCCAAGACCGGAGAGCAAATCGGAGACAGAAGCTAGAAGAAGCGCCATCAAGAGACAAAT 80
Bos     GCCAAGGGAGTTCATGATCACCCAAGACCAGAGAGCAAATCAGAGGCGGAAGCTAGAAGAAGTGCAATCAAGAGACAGAT 80
Canis   GCCAAAGGAGTTCACGACCACCCAAGACCAGAGAGCAAATCAGAGACGGAAGCTAGAAGAAGCGCCATCAAGAGACAAAT 80

Homo    GGCCTCTTTCTACCAACCCCAGAAAAAGAGAATTCGAGAATCCGAG 126
Pan     GGCCTCTTTCTACCAACCCCAGAAAAAGAGAATTCGAGAATCCGAG 126
Macaca  GGCCTCTTTCTACCAACCCCAGAAAAAGAGAATTCGAGAATCCGAG 126
Bos     GGCTTCTTTTTACCAACCACAGAAAAGGAGAATTCGAGAACCTGAG 126
Canis   GGCCTCTTTTTACCAACCCCAGAAAAAGAGAATTCGAGAACCAGAG 126
```

ENSG00000124827 exon 5

```
Homo    GCAGAAGAAAATCAAGACAGCAGTGGTCATTTCAGCAACATACCTCCCTTGGAAAATCCAGAAGACTTTGATATAGTTAC 80
Pan     GCAGAAGAAAATCAAGACAGCAGTGGTCATTTCAGCAACATACCTCCCTTGGAAAATCCAGAAGACTTTGATATAGTTAC 80
Macaca  GCAGAAGAAAATCAAGACAGCAGTGGTCATTTCAGCAACATACCTCCCTTGGAAAATCCAGAAGACTTTGATATAGCTGC 80
Bos     GCAGGAGAGAATCAGGATCACAGCAGACATTTCAACAACATCCCTTCCCTGGAGAACCCAGAGGGCTTGGATGTGATTCC 80
Canis   GCCGGAGAGAATCAAGACAACGGTGGACATTTCAGCAACATACCTCCCTTGGAAAATCCAGAAGAGTTTGATATCATTAC 80

Homo    TGAAACCAGCTTCCCTATTCCAGGGCAGCCTTGCCCTTCCTTCCCAAAGTCTGATGTTTACAAAGCTACCTGTGACCTAG 160
Pan     TGAAACCAGCTTCCCTATTCCAGGGCAGCCTTGCCCTTCCTTCCCAAAGTCTGATGTTTACAAAGCTACCTGTGACCTAG 160
Macaca  TGAAACCAGCTTCCCTATTCCGGGGCAGCCTTGCCCTTCCTTCCCAAACTCTGATTCTTACAAAGCTACCTGTGACCTAG 160
Bos     TGACGCTGGCTTCCCTGTTTCGGGCCAGCACTGCCTTTCCTTTCCAAACTCGGATGCTTGCAAAGCTACCTGCGACCTCA 160
Canis   TGACACTGGCTTCCCTATTTCAGGGCAGCCTTGCTTTTCCTTCCCAAACTCCGATGCTTACAAAGCTACCTGTGACCTAG 160
```

ENSG00000124827 intron 4

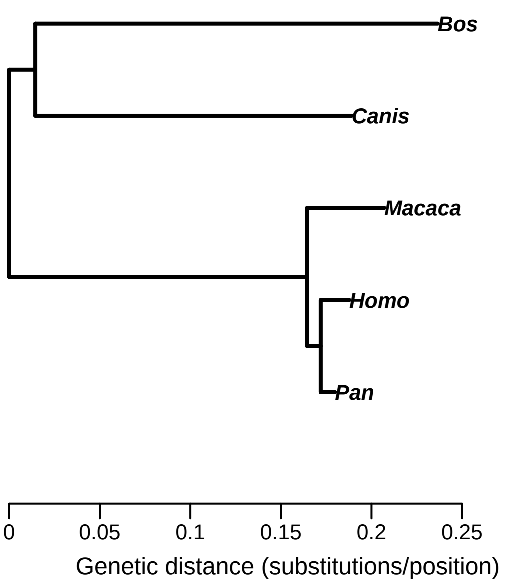

ENSG00000124827 intron 4

```
Homo    GTAACAGGGAGCTGATAGTGCAC-AGTGGTCTCAGCTCATTTTCATCCCAAGATACGCTGATCGTTATGCAAAATATGAAGGTCATACGTTAATTACAAG 99
Pan     GTAACAGGGAGCTGATAGCGCAC-AGTGGTCTCAGCTCATTTTCATCCCAAGATACGCTGATAGTTATGCAAAATATGAAGGTCATACATTAATTACAAG 99
Macaca  GTAACAGGGAGCTGACAGTGCAC-AGCGGTCTCAGCTCATTTTCATCCCAAGATACGCTGATCATTATGCAAAATATGAAGGTCATACATTAATTACAAG 99
Bos     GTAATGGGGAGCTGATGTTACACTTGTGGTCTGTATTTGTCGTCATCAGAAAATACACTGAGAATTACGCTAAATAGGAATGCCATAACTTAATTTAAAA 100
Canis   GTAATGGGGAGCTGACATGACAC-TGTGGTCTGTGTTCATTTCCGTCACAAAATACACTGCTAATTATGTAAAATATGAATGCTACAAATTAATTA---- 95

Homo    GGTTTGATTTCATCTGAAAATTATACGTCTGTAACAAAATATCATTAAATCAACA-TTTGGACAGGGTTTCTAATCC---CTTCTCCTT--------AAA 187
Pan     GGTTTGATTTCATGTGAAAATTATATGTCTGTAACAAAATATCATTAAATCAACA-TTTGGACAGGGTTTCTAATCC---CTTCTCCTT--------AAA 187
Macaca  GGTTTGATTTCATGTGAAAATTATATGTCTGTAACAAAATATCATTAAGTCAACA-TTTGGATAGGGTTTCTAATCCCTTCTTCTCCTT--------AAA 190
Bos     GGTTTGATTTTATGTGAAAGTTCTATGTCTCTAACAAAATGTCAATGAATCAAATTTTTGAATAGGGTTTCTAATCCCTTCTTCTCCTTGGGGAAAAAAA 200
Canis   GGTTTTATTTTATGGGAAAATTATATTTC--CAACAAAATGTCATTGAATCCACATTTTGGATAGGGTTTCTAATTTCTTCTTCTTCTT--------AAA 185

Homo    ACAAAAAGCAACAATCTCTACAGGCAATGATACAGTCCTGATATGTCAGTGTGCTGAGCTGACATCCTAATATAAGGG-CGATTATGCAACAGTAATAAA 286
Pan     ACAAAAAGCAACAATCTCTACAGGCAATGATACAGTCCTGATATGTCAGTGTGCTGAGCTGACATCCTAATATAAGGG-TGATTATGCAACAGTAATAAA 286
Macaca  AGAAAAAGCAACAATCTCTACAAGCAATGATACAGTCCTTATATGTCAGTGT-----GCTGACATCCTAATATAAGGC-CGATTATGCAACAGTAATAAA 284
Bos     AAAAGAGCTGAATATCTCTACAGTTAATGATAGACTGGGGATATGA-----------GCTGACATTCTACTACAAGGG-CAATTATCCAACGGTCATACA 288
Canis   AAAAAGGCAAACTGTCTCTACAGTCAATGATATAGTGAGGATGTGT-----------GCTGACACCCTGACATAAGGGCCAGGTATCCAGTTTTACTAAA 274

Homo    GATAGAAATATGTGCCTACCAACAGGAAGTTAACAGTGCACCCTTGAGAGGGTCCTTTCCATTATTTCATCCACAAGAATGGATAAAATTTC---TCTGC 383
Pan     GATAGAAATATGTGCCTACCAACAGGAAGTTAACAGTGCACCCTTGAGAGGGTCCTTTCCATTATTTCATCCACAAGAATGGATAAAATTTC---TCTGC 383
Macaca  GACAGAAATATGTGCCTACCAACAGGACATTGTCAGTGTACCCTTGAGAGGGTCCTTTCCATTATTTCATCCATAAGAATGGATAAAATTTC---TCTGT 381
Bos     GAAAAACATAGGTGCATCC---CAGTGAGTTAACAGTACAGCTTTGAGAGGGCCCTTTGCATTATTAACGT----AGAACAGATAAAAGTTC---TTTGT 378
Canis   AAGAGAAATACGTGCATACCAACAGTCAGTTAATAGCACATCCTTGAGAGCATCCTTTTATTATTTACAC-----AGAGCAAATAAAACTTCCAGTTGGG 369

Homo    GCAATTTAATTCAGTTTGGCTTTTCAACCAAAGCACGCCAAGCAGGCAGAGTGTTAGTGTAAGGATAACAGAAGCTGA-CATTTTCCAGCACTTCCTATG 482
Pan     GCAATTTAATTCAGTTTGGCTTTTCAACCAAAGCACGCCAAGCAGGCAGAGTGTTAGTGTAAGGATAACAGAAACTGA-CATTCTCCAGCACTTCCTATG 482
Macaca  GCAATTTAATTCAGTTTTCCTTTTCAACCAAAGCTTGCCAAGCAGGCAGAGTGTTAGTGTAAGGATAACAGAAACTGA-CACTTTCCAGCACTTCCTATG 480
Bos     GCAACTTGAGCCAGTTTGGCTTCTCTACCAAAGTATGCCAAGCAGGCAGAATATTTGCCTGAAGATAAGAGGCACTGGTCTTGTCTGAGCACTTTCTATG 478
Canis   GCAACTTGATCCAGTTTGGCT--------------------------------TTAGTCTGGGGACGAGAGAAGCT---CGTGTTTGAGCACTT------ 428

Homo    TGCCAGGCACT--GGTCTAAGAGTTTCACACATTTAATGGTTGCAGAAACCTTATAATGAGGTTACTGTTATTAAGTTCCATTTTTAAGATGAAGGATCT 580
Pan     TGCCAGGCACT--GGTCTAAGAGTTTCACACATTTAATGGTTGCAGAAACCCTATAATGAGGTTACTATTATTAAGTTCCATTTTTAAGATGAAGAATCT 580
Macaca  TGCCAGGTACT--GGTCGAAGAGTTTCACACATTTAATGGTTGCAAAAACTCTATAATGAGGTTACTATGATTAAGTTCCATTTTTAACATGAAGAATCT 578
Bos     TGCCATGACATACAGGCAAAGAGATTTACACTTCTTAAGATCACAAAAGCCTTAAGAAGAAACTACGGTTATTGAGCCCCATTTTTAAG-TGAGGAACCT 577
Canis   ---------------TCTAAGAGTTTCACACGTGAAATGGTCACAAAAGCCTTACGAAGAGGTGGCTGTTAT----TTACATTTTTTAAATTAGGAATCT 509

Homo    GAGGCTCAGAGAGGTTAAGTGGCTTGTACAATTTGTGAGGGACAGAGTCAGGAATTGAAC----------CCAGGCC--CGTGTGGCTCCTAAGTCTCCA 668
Pan     GAGGCTCAGAGAGGTTAAGTGGCTTGTACAATTTGTGAGGGACAGAGTCAGGAATTGAAC----------CCGGGCC--TGTGTGGCTTCTAAGTCTCCA 668
Macaca  GAGGCTCAGAGAGGTTAAGTGGCTTGTACAATTCGTGAGGGACAGAGTCAGGAATTGAAC----------NNNNNNN--NNNNNNNNNNNNNNNNNNNNN 666
Bos     GAGGCTCAGAGAGAGTAGGTAACTTGTCTAACTCATGAGGGACAGG--CAGGATTTGAAC----------CCACGTCTGCGGCAGGCTCCTAAGCCTCTG 665
Canis   GAGGTTCAGAAAGGTTAAGTAACTAGTTTGATTAATGAGGGACAGAGTCAGGATTTGAACAGAGTCAGGTCTATGTC--AGGCAGGAGCCTGTGTCTCCG 607

Homo    CTATTAGGAAGA------------CAGTATAGGTGAGAACAGGACCATCTGCCACTCAGAAGCATCCTACCTGGCTACCATGGACCTTTG---------- 746
Pan     CTATTAGGAACA------------CAGTATAGGTGAGAACAGGACCATCTGTCACTCAGAAGCATCCTACCTGGCTACCATGGACCTTTG---------- 746
Macaca  NNNNNNNNNNNN------------NNNNNNNNNNNNNNNNNNNNNNNNNNNNNNNNNNNNNNNNNNNNNNNNNNNNNNNNNNNNNNNNNNNNNNNNNNNNNC 754
Bos     CTCTCCTGCACA------------TAGTGTGCGGAAG-ACAGGATCGTCCACCACTGAGACCCACCCTCCCAGGCTATCCTGGACCCTCT---------- 742
Canis   TTATTATAAATAGAAATCCATTATTATGATCAGGCACCACAGAGCCATCTGCCACTCAGCCCCAGCCTGCCTGGCTACCGTGGACTCTCT---------- 697

Homo    TCATTCAGTCGT-TGGTATTTATGTCTGAGTGAGCCCAAGCTTAGCAACCCCTGGACCACGTGCTTACATCACGTACCTGAGCAGTGCCATGTTCACAAC 845
Pan     TCATTCAGTTGT-TGGTATTTATGTCTGAGCGAGCCCAAGCTTAGCAACCCCTGGACCATGTGCTTAAATCACGTACCTGAGCAGTGCCATGTTCACAAC 845
Macaca  TCATTCAGTTGT-TGGTATTTATATCTGAGAGAGCTCAAGCTTAGCAACCCTTGGACCATGTGCTTAAATCACGTACCTGGGCAGTGCCATGTTCACAAC 853
Bos     GAGGTCAGCAGCGTGGAATTCAAGACTGA----GCTCAAGCCGTGAAATTCCTGGACTGGGTGCCCGGAAAGTGT------------------------- 813
Canis   TCATTCGGCAGCATGGTGTTGGTGCCTGAGCAGGCTCAAGTGATGCAGCCCTTGGACCACGGGCCCAAAGCATGTACCTGAGCGGTGCAGTGCTCACAAC 797

Homo    ACAGAGCCCAGCACATGGATGTCTGTTTTTACTTACTT-CTAGGCTATTGTT-ACAGGTGAGGGTGTCCACACGATTTACATAGCGTGTGTCT------- 936
Pan     ACAGAGCCCAGCACATGGACGTCTGTTTTTACTTACTT-CTTGGCTATTGTT-ACAGGTGAGGGTGTCCACACGATTTACATAGCATGTGTCT------- 936
Macaca  ACAGAGCCCAGCGCATGGATGTCTGTTTTTACTTACTT-GTTGGCTATTGTT-ACGGGTGAGGGTGTCCATACGATTTACATAGCATGTGTCT------- 944
Bos     ---------------------TTGCTTGTTATTTGGTTGGTTGGTGACTGTTGACAGGTGAAGCTGTCCATGTAATTTACATCTC---TATTTACAAATA 889
Canis   ACACAACTGGCCAACTGGAAGTTTGTTTTCACTTGGTTGGTTGGCCACTGTT-CCAGATGAGGGTGACCATGTAATTTACATTTCTGTTGACCAAAAAAA 896

Homo    --ATGTTTGTATCATTTTTCTAG 957
Pan     --ATGTTTGTATCATTTTTCTAG 957
Macaca  --GTGTTTGTATCATTTTTCTAG 965
Bos     AAAACTTTGGGTCATTTCTTTAG 912
Canis   AAAAATGTATATCATTTTTTTAG 919
```

ENSG00000197563 intron 5

Description: GPI ethanolamine phosphate transferase 1 (PIGN)
Intron number: 5
Human chromosome: 18
Intron start (bp): 57965315
Human intron length : 1112
Intron alignment length: 1241
Flanking exons length (upstream/downstream): 125/131
SNP density: 0.011691
K tree score: 0.0668
Scaling factor: 1.0595
Human-chimpanzee distance: 0.016505
Total primate branch length: 0.0798

ENSG00000197563 exon 5

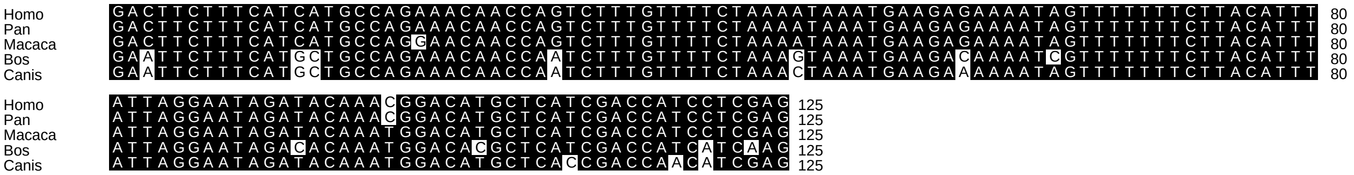

ENSG00000197563 exon 6

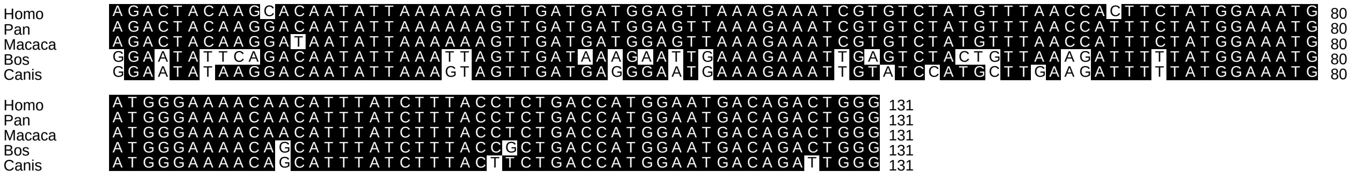

ENSG00000197563 intron 5

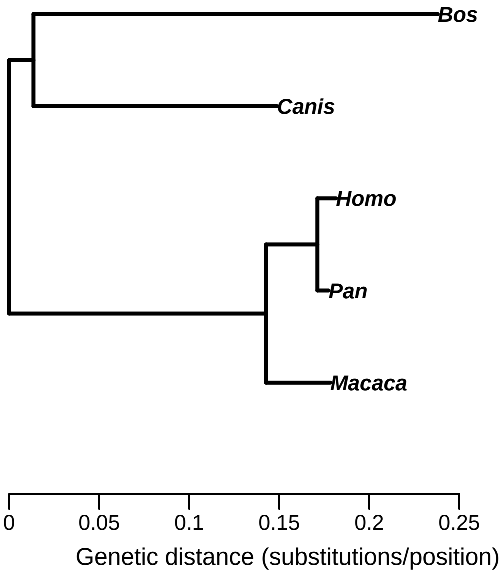

ENSG00000197563 intron 5

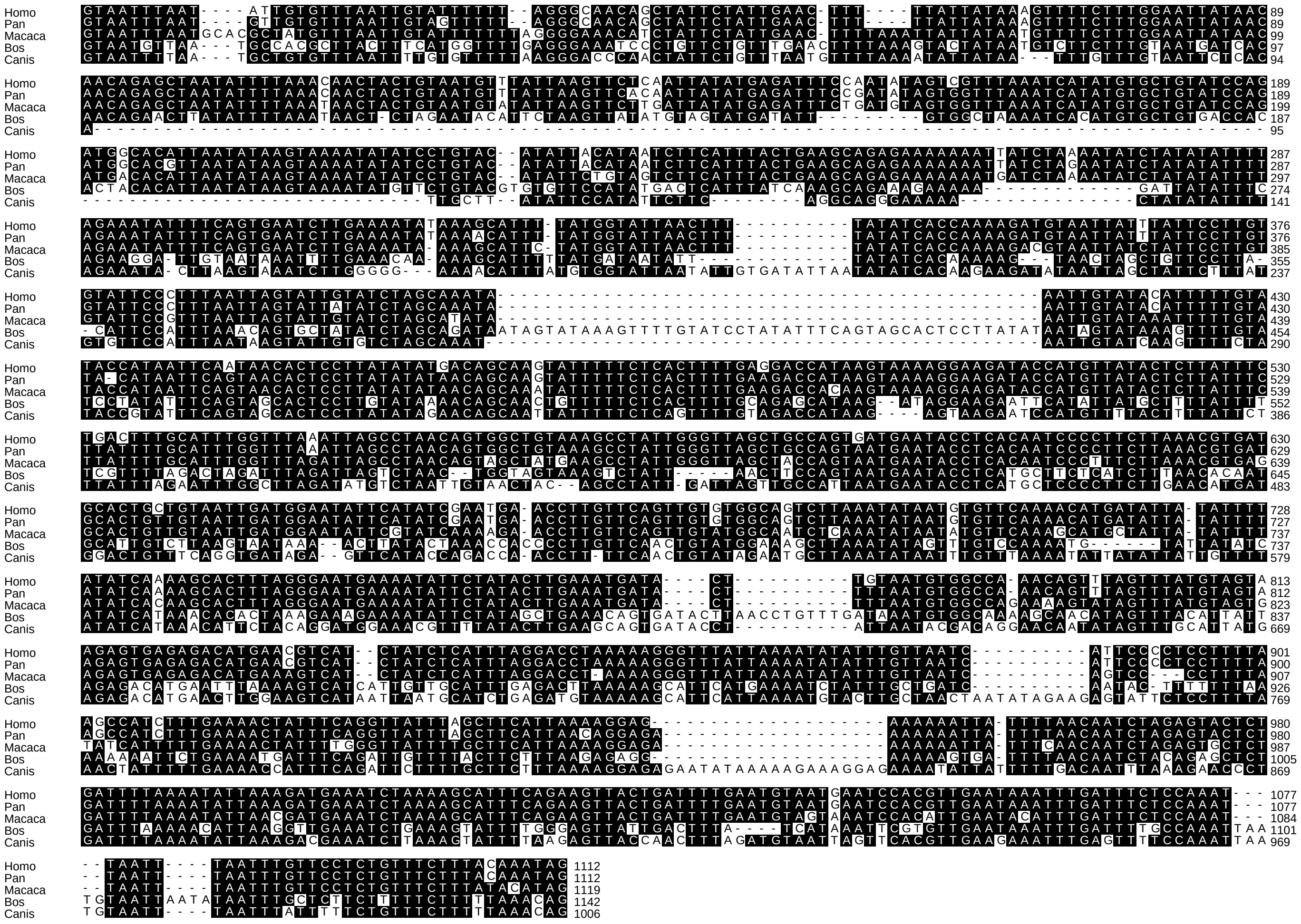

ENSG00000136750 intron 3

Description: Glutamate decarboxylase 2 (GAD2)
Intron number: 3
Human chromosome: 10
Intron start (bp): 26546927
Human intron length : 1051
Intron alignment length: 1189
Flanking exons length (upstream/downstream): 150/234
SNP density: 0.011418
K tree score: 0.04
Scaling factor: 1.0753
Human-chimpanzee distance: 0.022407
Total primate branch length: 0.0796

ENSG00000136750 exon 3

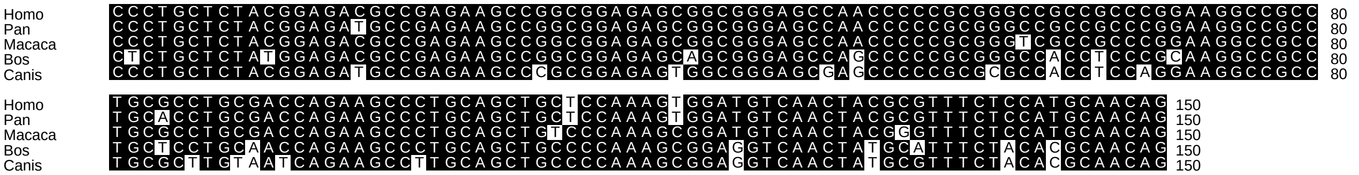

ENSG00000136750 exon 4

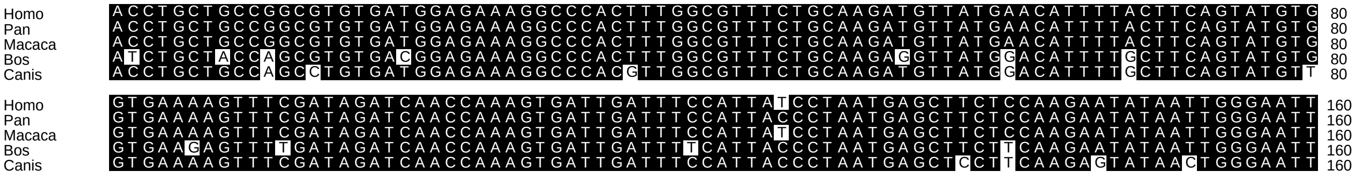

ENSG00000136750 intron 3

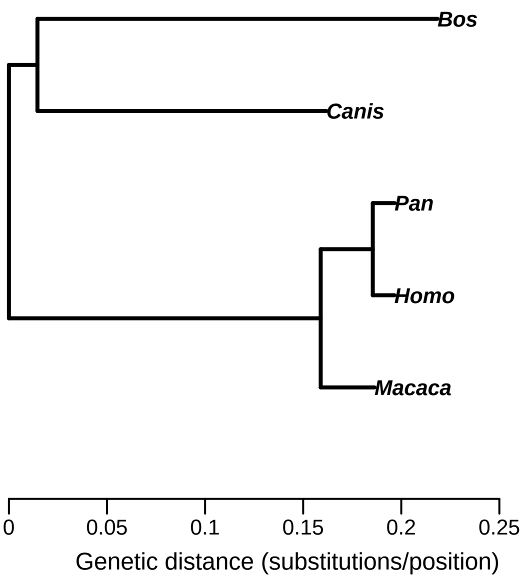

ENSG00000136750 intron 3

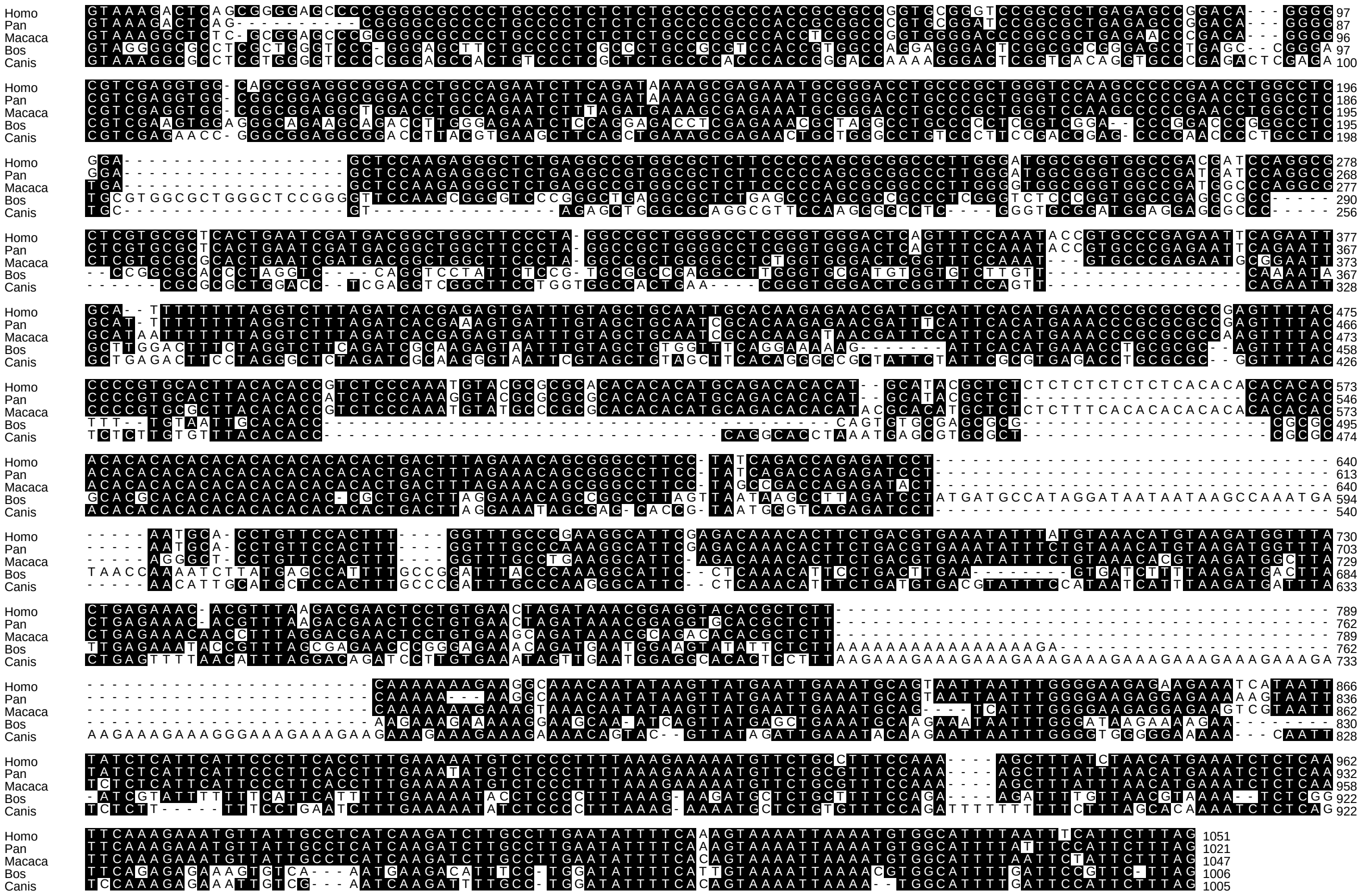

ENSG00000148297 intron 2

Description: Surfeit locus protein 5 (MED22)
Intron number: 2
Human chromosome: 09
Intron start (bp): 135201010
Human intron length : 838
Intron alignment length: 934
Flanking exons length (upstream/downstream): 81/209
SNP density: 0.008353
K tree score: 0.0391
Scaling factor: 0.582
Human-chimpanzee distance: 0.012263
Total primate branch length: 0.0796

ENSG00000148297 exon 2

```
Homo    ATTGAGGACGAGACGCAGGTGTCACGGGCCACTCAGGGTGAACAGGACAATTACGAGATGCATGTGCGAGCCGCCAACAT 80
Pan     ATTGAGGACGAGACGCAGGTGTCACGGGCCACTCAGGGTGAACAGGACAATTACGAGATGCATGTGCGAGCCGCCAACAT 80
Macaca  ATTGAGGACGAGACGCAGGTGTCACGGGCCACTCAGGGTGAACAGGACAATTACGAGATGCACGTGCGAGCCGCCAACAT 80
Bos     ATTGAGGACGAGACGCAGGTGTCAAGGGCTACTCAGGGCGAGCAGGACAACTACGAGATGCACGTGAAGAGCTGCCAACAT 80
Canis   ATCGAGGACGAGACACAAGTGTCGAGAGCCACTCAGGGCGAGCAGGATAATTACGAGATGCATGTTCGAGCTGCCAACAT 80

Homo    C 81
Pan     C 81
Macaca  T 81
Bos     C 81
Canis   C 81
```

ENSG00000148297 exon 3

```
Homo    GTCCGAGCCGGCGAGTCCCTGATGAAGCTGGTGTCCGACCTCAAGCAGTTCCTGATCCTCAATGACTTCCCCTCCGTGAA 80
Pan     GTCCGAGCCGGCGAGTCCCTGATGAAGCTGGTGTCCGATCTCAAGCAGTTCCTGATCCTCAATGACTTCCCCTCTGTGAA 80
Macaca  GTCCGAGCCGGTGAGTCCCTGATGAAGCTGGTGTCCGACCTCAAGCAGTTCCTGATCCTCAACGACTTCCCTTCAGTGAA 80
Bos     GTCCGAGCCGGCGAGTCCCTGATGAAGCTGGTGTCTGACCTCAAGCAGTTTCTCATCCTCAACGACTTCCCATCGGTGAA 80
Canis   GTCCGAGCCGGTGAGTCCCTGATGAAGCTGGTGTCTGACCTCAAGCAGTTCCTCATCCTCAATGACTTCCCATCGGTGAA 80

Homo    CGAGGCCATTGACCAGCGCAACCAGCAGCTGCGCACACTGCAGGAGGAGTGCGACCGGAAGCTCATCACGCTGCGAGACG 160
Pan     CGAGGCCATTGACCAGCGCAACCAGCAGCTGCGCGCACTGCAGGAGGAGTGCGACCGGAAGCTCATCACGCTGCGAGACG 160
Macaca  TGAGGCCATCGACCAGCGCAATCAGCAGCTGCGCGCGCTGCAGGAGGAGTGTGACCGGAAGCTCATCACGCTGCGAGACG 160
Bos     CGAGGCCATCGACCAGCGTAACCAGCAGCTGCGAGCCCTGCAGGAGGAGTGTGACCGGAAGCTCATCGCACTGCGGGACG 160
Canis   TGAGGCCATCGACCAGCGCAACCAGCAGCTGCGAGCCCTGCAGGAGGAATGTGACCGGAAGCTCATCGCCCTGCGGGACG 160
```

ENSG00000148297 intron 2

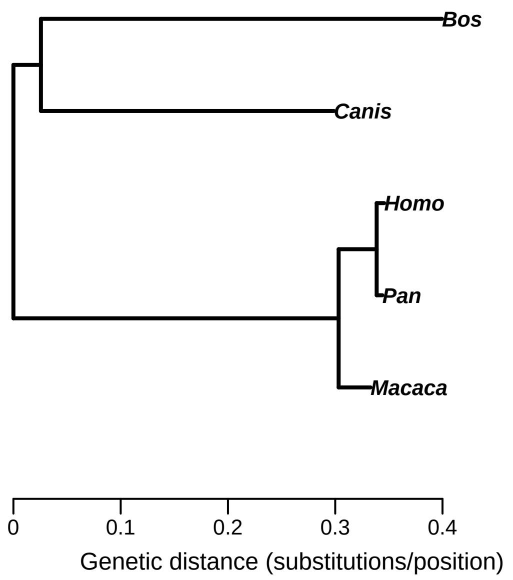

ENSG00000148297 intron 2

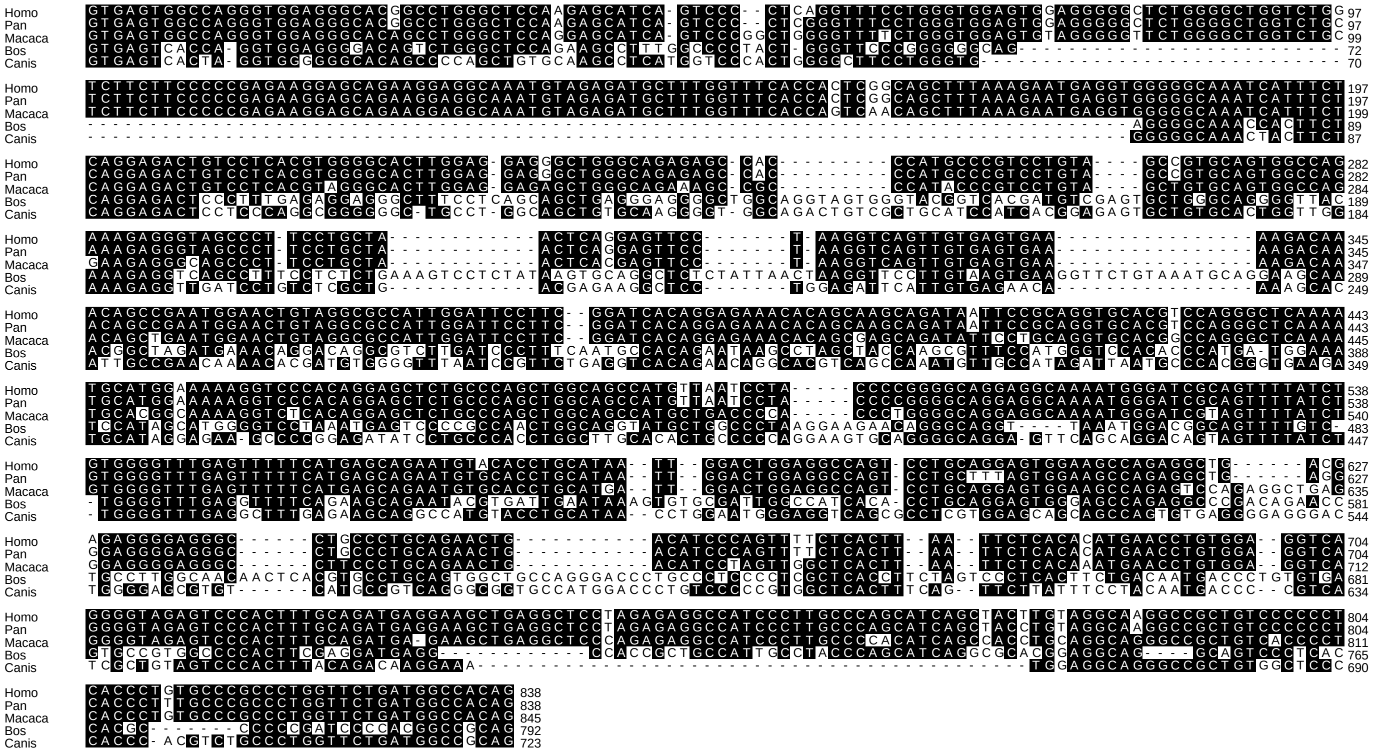

ENSG00000113161 intron 4

Description: 3-hydroxy-3-methylglutaryl-coenzyme A reductase (HMGCR)
Intron number: 4
Human chromosome: 05
Intron start (bp): 74677240
Human intron length : 1545
Intron alignment length: 1586
Flanking exons length (upstream/downstream): 85/106
SNP density: 0.005178
K tree score: 0.0574
Scaling factor: 1.0295
Human-chimpanzee distance: 0.009875
Total primate branch length: 0.0796

ENSG00000113161 exon 4

```
Homo    TGAAGCTTTGCCCTTTTTCCTACTTTTGATTGACCTTTCCAGAGCAAGCACATTAGCAAAGTTTGCCCTCAGTTCCAACT 80
Pan     TGAAGCTTTGCCCTTTTTCCTACTTTTGATTGACCTTTCCAGAGCAAGCACATTAGCAAAGTTTGCCCTCAGTTCCAACT 80
Macaca  TGAAGCTTTGCCCTTTTTCCTACTTCTGATTGACCTTTCCAGAGCAAGTGCATTAGCAAAGTTTGCCCTCAGTTCCAACT 80
Bos     TGAAGCTTTGCCCTTTTTCCTGCTGCTGGTCGACCTTTCTAGAGCAAGTGCGCTAGCCAAGTTCGCCCTCAGTTCTAACT 80
Canis   TGAAGCTTTACCCTTCTTCCTACTTCTGATTGACCTTTCCAGAGCAAGTGCTCTAGCAAAATTTGCTCTCAGTTCCAACT 80

Homo    CACAG 85
Pan     CACAG 85
Macaca  CACAG 85
Bos     CACAG 85
Canis   CACAG 85
```

ENSG00000113161 exon 5

```
Homo    GATGAAGTAAGGGAAAATATTGCTCGTGGAATGGCAATTTTAGGTCCTACGTTTACCCTCGATGCTCTTGTTGAATGTCT 80
Pan     GATGAAGTAAGGGAAAATATTGCTCGTGGAATGGCAATTTTAGGTCCTACGTTTACCCTCGATGCTCTTGTTGAATGTCT 80
Macaca  GATGAAGTAAGGGAAAATATTGCTCGTGGAATGGCAATTTTAGGTCCAACGTTTACCCTCGATGCTCTTGTTGAATGTCT 80
Bos     GATGAAGTGAGGGAGAACATTGCTCGTGGGATGGCGATTTTAGGTCCCACCTTCACCCTGGATGCTCTTGTAGAATGTCT 80
Canis   GATGAAGTACGGGAAAATATTGCTCGTGGAATGGCAATTTTAGGTCCCACGTTCACCCTTGATGCTCTTGTAGAGTGTCT 80

Homo    TGTGATTGGAGTTGGTACCATGTCAG 106
Pan     TGTGATTGGAGTTGGTACCATGTCAG 106
Macaca  TGTGATTGGAGTTGGTACCATGTCAG 106
Bos     TGTGATTGGAGTTGGTACCATGTCAG 106
Canis   GGTGATTGGAGTTGGTACCATGTCAG 106
```

ENSG00000113161 intron 4

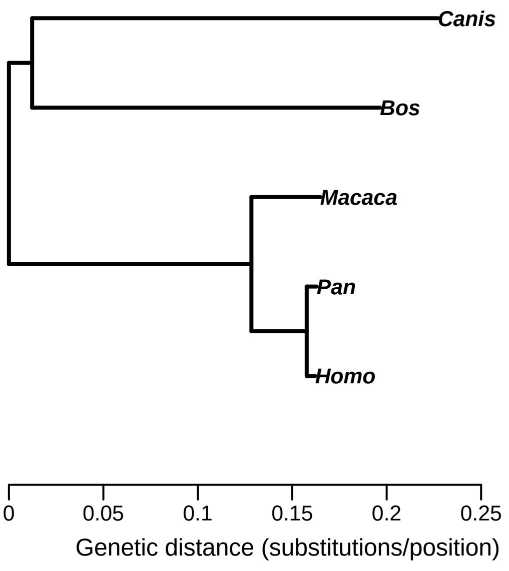

ENSG00000113161 intron 4

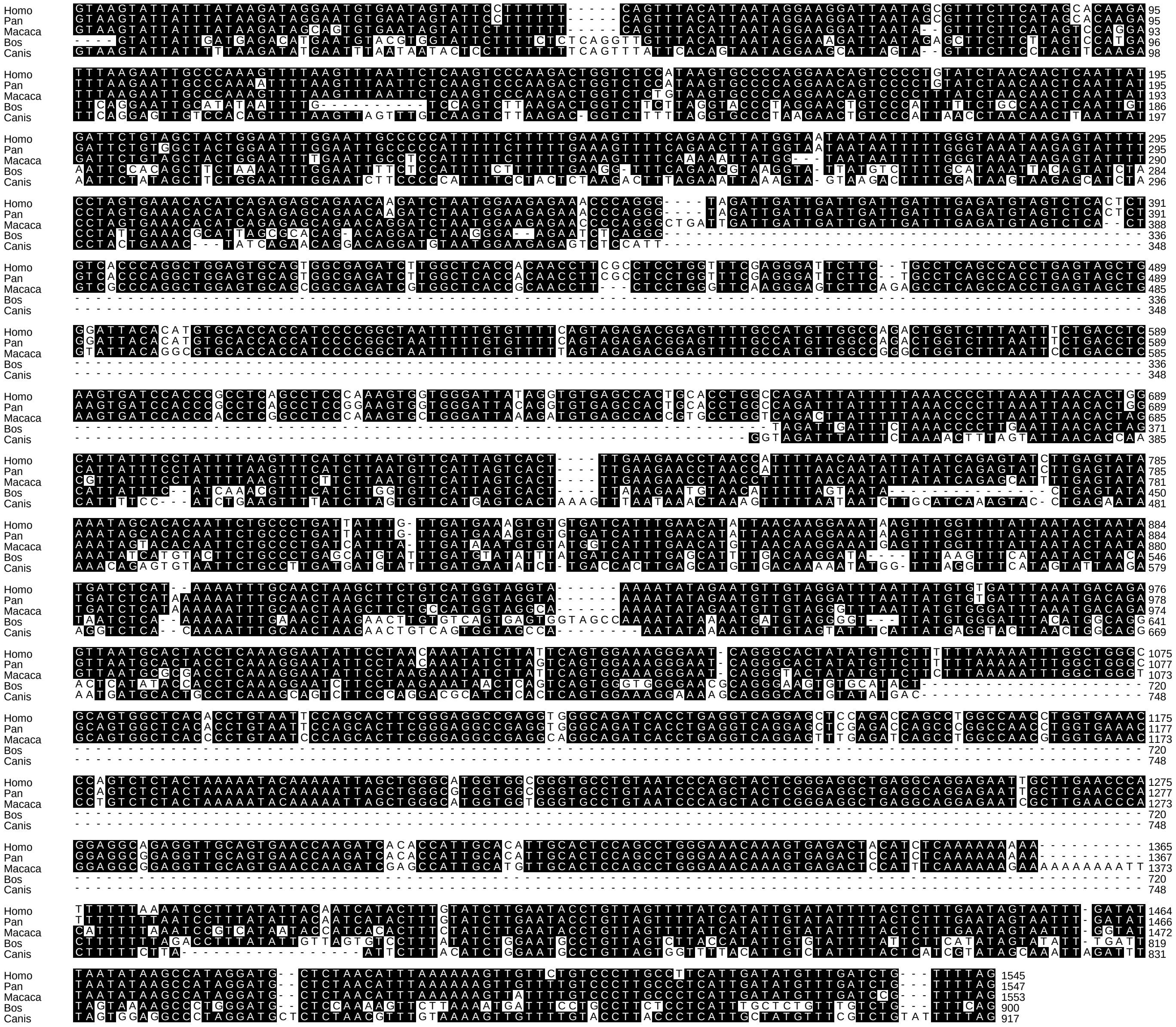

ENSG00000128165 intron 1

Description: ADM2 precursor (ADM2)
Intron number: 1
Human chromosome: 22
Intron start (bp): 49267253
Human intron length : 609
Intron alignment length: 786
Flanking exons length (upstream/downstream): 110/337
SNP density: 0.000000
K tree score: 0.0687
Scaling factor: 0.5794
Human-chimpanzee distance: 0.011702
Total primate branch length: 0.0794

ENSG00000128165 exon 1

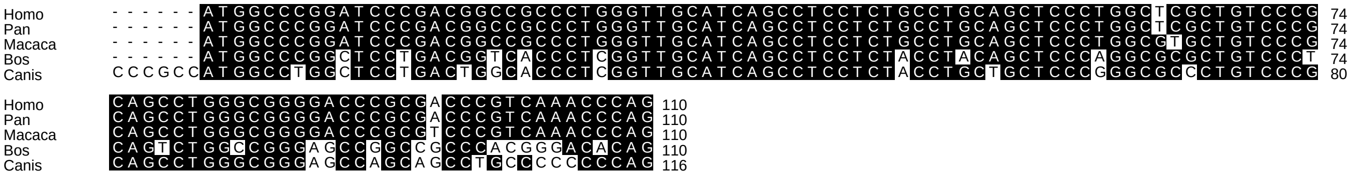

ENSG00000128165 exon 2

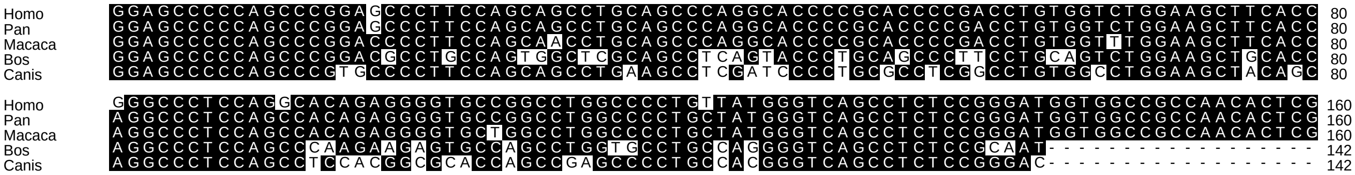

ENSG00000128165 intron 1

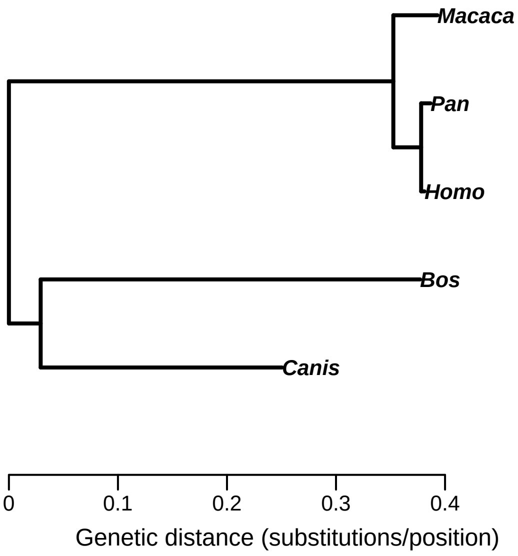

ENSG00000128165 intron 1

```
Homo    GTGAGTCCAGGTCTTGGGCCAGCCAACCCCTCTGGCCCCCGCAGGGAAGCAGA--GTGCCGGGGGCCGCGGGGCCGCCCTCCCTCCACGCC--------- 89
Pan     GTGAGTCCTGGTCTTGGGCCAGCCAACCCCTCTGGCCCCCGCAGGGAAGCAGA--GTGCGGGGGGCCGCGGGGCCGCCCTCCCTCCACGCC--------- 89
Macaca  GTGAGTCCAGGTCTTGGGCCAGCCAGCCCCTCTGGCCCCCACAGGGAAGCAGA--GTACCG--------GGGGCCGCCCTCCCTCCA-ACC--------- 80
Bos     GTGAG-------CAGGTCTCAGACCACCATGGATGCCGGGGCAGGAAATCAGATGCTCCGGAAGGCCACACCCCGCCGCTGCCCCAGGACCCCGAACTGG 93
Canis   GTGAG-------CGGGGTTC--ACAGCTGCCCACGCTGTGGC-------------CCACGGGGAGGCATCCCCCCAACATTCCCCAGCACT--------G 70

Homo    --------TCCACCTGCTGCTAGGACTCCCCTCCCAAACAAAGGCCCCTACCCCAGGCTCAGGAGCGCCTGAGCAGGGCCAGAGGCCCCTCTTTCTCTGC 181
Pan     --------TCCACCTGCTGCTAGGACTCCCCTCCCAAACAAAGGCCCCTACCCCAGGCTCAGGAGCGCCTGAGCAGGGCCAGAGGCCCCTCTTTCTCTGC 181
Macaca  --------GCCACCTGCTGCTAGGGCTCCCGTCCCAAACCAAGGCCCCTGCCCCAGGCTCAGCAGCGCCTGAGCAGGGCCAGAGGACCCTCTTCCTCTGC 172
Bos     GGGAAACCCGCTTCTGCTGCTAGGGCTCACCTGCTAAATCCAGCCCC-------AGGCCATGGGCACCCCCCGCAGGGCAGAGGGCTCCTTTTTCTTTGC 186
Canis   GGGCCCCCTCCACCTACTGCTAGGGCTTTCCTCCTAAATCAAGGGCCC--ATGTAGGCCTTCAAATCCTGAGGGGGGGCAAGCGGCCCCTCCTCCTTTGT 168

Homo    CTCCCTACACAGGGCAGTTGC----TCCTCCCCACACACATGGGCACAGCAGCCCCTCTTCTGGGGGTGTCTGCTGGGGGAGGGGAGGCTGGCGGTATCC 277
Pan     CTCCCTACACAGGGCAGTTGC----TCCTCCCCACACACATGGGTACAGCAGCCCCTCTTCTGGGGGTGTCTGCTGGGGGAGGGGAGGCTGGCGGTATCC 277
Macaca  CTCCCTGCACAGGGCAGTTGC----TTCTCCCCACACAGGTGGACACAGCAGCCCCTTTTCTGGGGGTGTCTGCTGGGGGAGGGACGGCTGGCGGTATCC 268
Bos     CTGCCCTTCTAAGGCATGCCC----------ACTGCCCCACTGGCAGAGCAGACCCCTCAGAAGGAGGGTCAGCTGTGGGAGAGGAGGTTAAGGGGCTTC 276
Canis   CT-CCCCCCAAGGACACTTCCCCCATCCCCGACCCCCCTGTTGGTAGAGCCATCCCCTCA-AGGAGAGGTCAGC--CAGGAGAGGGAGGTGGAGGGATGC 264

Homo    CT-GGCCGGCCTGTGACCACCCTAAAGGGAGGGCAGAAGCCGAGTCACCCTGAGCCCTGAGAGTGGTGACCCGCAGAGGCTGGGCCGCCGGGGTGCCCTT 376
Pan     CT-GGCCGGCCTGTGACCACCCTAAAGGGAGGGCAGAAGCCGAGTCACCCTGAGCCCTGAGAGTGGTGACCCACAGAGGCTGGGCCGCCGGGGTGCCCTT 376
Macaca  CCAGGCCCACCTGTGACC-----AAAGGGAGGGCAGAAGCTGAGTCACCCTGAGCCCTGAGAGTGGTGACCCGCAGAGGCTGGGCCGCGGGGGTGCCCTT 363
Bos     CCACACCTGCCCACCTTCCCCCACCCCAAGGGAAGGGCGT-------AACTTAGTCCTGAGCAGAGTAGCCCAGGGAGGT-------CAG-GCAGCTGCT 361
Canis   CCAGGACTGCCCACCTCTCCCACCCAGGGGAAGGGGGAGC-------ATGTGGGCTGCGAGCATAGTGACCCACGGGGGC-------CAGTGGCGCCCTT 350

Homo    TGGCCAAGGAGGTGCCGGTTCCCGGGACACGTGACCCGGGCCACTGAGATGGTC-----CCAGG-CCCAAGCCCAGAG---------------------- 448
Pan     TGGCCAAGGAGGTGCCGGTTCCCGGGACACGTGACCCGGGCCACTGAGATGGTC-----CCAGG-CCCAAGCCCAGAG---------------------- 448
Macaca  TGCTCAAGGAGGTGCCGGTTCCCAGGACACGTGACCCGGGCCACTGAGATGGTC-----CCAGGCCCCAAGCCCAGAG---------------------- 436
Bos     TGCCCAG-------CCCTTGAA--------------TGGGCTACTGAGATGGCTGAGGGCCAGAACCCCAGCCCTGAGCTCAGTGGGCTTCCCCTGGCCT 440
Canis   TGCCCGGGGAGGTTCAGGTGAAT-GGACGTGGGACCTGGGCTGCCGAGGTGGCTGAGGGCCAGGACCCAAGCCCAGGGCTCAGAAGGC------------ 437

Homo    ------------------------------------------------------------------------------------------------GGGA 452
Pan     ------------------------------------------------------------------------------------------------GAGA 452
Macaca  ------------------------------------------------------------------------------------------------GGGA 440
Bos     ACTGGCCCCATGGCATGAGGTCTGTGGGCTTTCCCTGGCCCGCTGGCCCATGGCGTGGGGTCTGTGGGCTTCCCCTGGCCTGCTGGCCCATGGCGTGGGG 540
Canis   ---------------------------------------------------------------------------------------------------- 437

Homo    CGC--GGGCTTCCCCTGGCCGTGCTCCAGGCTGTGTGTGGATGGGGGCTGTGGGCTTCCCCTGGC--AGTGACCCAGGCCTGTGTAGAGGCAAAAGTGGG 548
Pan     CGC--GGGCTTCCCCTGGCCGTGCTCCAGGCTGTGTGTGGATGGGGGCCGTGGGCTTCCCCTGGC--AGTGACCCAGGCCTGTGTAGAGGCAAAAGTGGG 548
Macaca  CGCGTGGGCTTCCCCTGGCTATGCTCCAGGC--TGTGTGGGTGGGGGCCGTGGGCTTCCCCTGGC--AGTGACCCAGGCCTGTGTAGAGGCAAAAGTGGG 536
Bos     TCTGTGGGCTTCCCCTGGCCGTGCTCCAGCCTGT-------GGGGTGGGGTGGGCTTCCCCTGGCTCGCTGGCCAGTGACTTGGGGCTGTGGGCTTCCC 633
Canis   GCCGTGGGCTTCCCCTGGCAGCACTCCAGGGCTC-------TGGGGAAACTGGGCTTCCCCT-GTCGGTGACCCCATGCCTCTGTGGAAGGGAGAGTGGC 529

Homo    CAGGT-------------------------GTGAGTTCTCCAAAAGGCTGAGCCATCTGGTTGACATTCTCCATCTGCCTCTGCAG 609
Pan     CAGGT-------------------------GTGAGTTCTCCGAAAGGCTGAGCCATCTGGTTGACATTCTCCATCTGCCTCTGCAG 609
Macaca  TAGGT-------------------------GTGAGGTCTCTGAAAGGCTGAGCCATCCAGTTGACATTCTCCATCTGCCTCTGCAG 597
Bos     CTGGCCATGCTCCAGGCTGTTGGAAAGCTGTGGGCTTCCCTGAAAGGCTGGACCACTGGGTTTACTTCCTCCATCTCCCTGTGCAG 719
Canis   TGGGC----------------------TTGAGGGGTCCCCTGAAAGGTTGGGCCACCAGGTTTCCTTCCTCC-CCCCCCACTGCAG 592
```

ENSG00000164062 intron 17

Description: Acylamino-acid-releasing enzyme (APEH)
Intron number: 17
Human chromosome: 03
Intron start (bp): 49694405
Human intron length : 380
Intron alignment length: 381
Flanking exons length (upstream/downstream): 81/89
SNP density: 0.005263
K tree score: 0.0732
Scaling factor: 1.0144
Human-chimpanzee distance: 0.010818
Total primate branch length: 0.0793

ENSG00000164062 exon 17

```
Homo    GGGGGCCCCATTCATCCTTTGTCACTGCCTGGATGCTGTTCCCAGCCATGCTTTGCAAGATGGGCTTTGCGGTACTACTA  80
Pan     GGGGGCCCCATTCATCCTTTGTCACTGCCTGGATGCTGTTCCCAGCCATGCTTTGCAAGATGGGCTTTGCGGTACTACTA  80
Macaca  GGGGGCCCCATTCATCCTTTGTCACTGCCTGGATGCTGTTCCCAGCCATGCTTTGCAAGATGGGCTTTGCGGTACTACTA  80
Bos     GGGGACCCCATTCATCCTTTGTCACTTCCTGGATGCTACTCCCAGCCATGCTTTGCAAGATGGGCTTTGCAGCTCTACTA  80
Canis   GGGGACCCCATTCATCCTTTGTCACTGCCTGGATGCTGTTCCCAGCCATGCTTTGCAAGATGGGTTTTGCAGTACTCCTA  80

Homo    G  81
Pan     G  81
Macaca  G  81
Bos     G  81
Canis   G  81
```

ENSG00000164062 exon 18

```
Homo    TGAACTATCGTGGCTCCACGGGCTTTGGCCAGGACAGCATCCTCTCCCTCCCAGGCAATGTGGGCCACCAGGATGTGAAG  80
Pan     TGAACTATCGTGGCTCCACGGGCTTTGGCCAGGACAGCATCCTCTCCCTCCCAGGCAATGTGGGCCACCAGGATGTGAAG  80
Macaca  TGAACTATCGTGGCTCCACGGGCTTTGGCCAGGACAGCATCCTCTCCCTCCCAGGCAATGTGGGCCACCAGGATGTGAAG  80
Bos     TGAACTATCGTGGCTCCACTGGCTTTGGCCAGGACAGCATCCTCTCCCTCCCCGGCAATGTGGGCAGCCAGGATGTGAAG  80
Canis   TGAACTATCGAGGCTCCACGGGTTTTGGCCAGGACAGCATCCTCTCCCTTCCTGGCAAAGTGGGCCACCAGGATGTGAAG  80

Homo    GATGTCCAG  89
Pan     GATGTCCAG  89
Macaca  GATGTCCAG  89
Bos     GATGTCCAG  89
Canis   GATGTCCAG  89
```

ENSG00000164062 intron 17

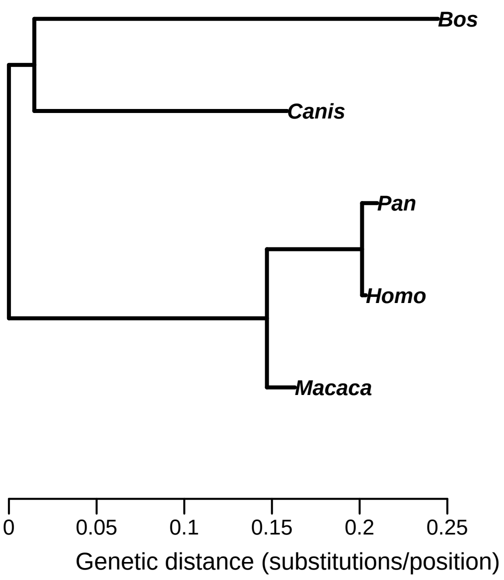

ENSG00000164062 intron 17

```
Homo    GTGAGTGAGCAGGGACCCACAGTTCTGTTAGGACTACAGTGGAGTGACGGGGGTGGACCTGGTTTGTATAAGTACCATGGGTGCCACTCTAGTCTCCAGG 100
Pan     GTGAGTGAGCAGGGACCCACAGTTCTGTTAGGACTACGGTGGAGTGACGGGGGTGGACCTGGTTTGTATAAGTACCATGGGTGCCACTCTAGTCTCCAGG 100
Macaca  GTGAGTGAGCAGGGACCCACAGCTCTGTTAGGACTGTGGTGGAGTGAGAGGGGTGGACCTGGTTTGTACAAGCACCATGGGTGCCACTCTAGTCTCCAGG 100
Bos     GTGAGGGGGCAGGGCCCTGCAGCTCTGGTAGGGCCGT-GGGGTGGTCATGGGGTGGGCCTGGTTGGTGCAAGTGTTGTGGGTACTGCCCTCATCTCCAGG 99
Canis   GTGAGTAAGCAGAGCCCTACAGCAGTGGTAGGGCT---GTGGAGGGTGATGGGTGG-CCTGGTTTGT--AAGCACCACGGGTAGCACCCTAGTCTCCGGG 94

Homo    ACTTTCTCTAGCCTAGACCTAGTAACCACTACCAGGAACTGCTGGGGCCCAGCCTCTGGTTCTGTGGCATAGTGATAACAAGGACTAACTCGGGACTTCC 200
Pan     ACTTTCTCTAGCCTAGACCTAGTAACCACTACCAGGAACTGCTGGGGCCCAGCCTCTGGTTCTGTGGCATAGTGATAACAAGGACTAACTCGGGACTCCC 200
Macaca  TCTTTCTGTAGCCCAGACCTAGTAACCACTACCAGGAACTGCCGGGGCCCAGCCTCTGGTTCTGTGGCATAGTGATAACAAGGACTAACTTGGGATTTCC 200
Bos     CCTCTCTGCAGCCCTGAACTGGTAATCACTATCAGAAACTGCCCTGACCCAGCCTCAGGCACCATGG----------------ACTAACTTGGAACTCCC 183
Canis   CCTGTCTGCAGCCTGGCACCAGCAGCC-----------TGGTAATGGCCCAGCCTCAGGCTCCACGGCGTGGTCACAACATGAAATAACATGGGACTTCC 183

Homo    AAGGAAAGGGCGCTGTGGTCACTGGGTGGCAGCCTCAAATCTCTGAGTCCCTTCCCCTTGACCCCTTGACAGTCTGTGGTATAGCTGGCCCCAGGGCCTA 300
Pan     AAGGAAAGGGCGCTGTGGTCATTGGGTGGCAGCCTCAAATCTCTGAGTCCCTT--------CCCCTTGACAGTCTGTGGTATAGCTGACCCCAGGGCCTA 292
Macaca  AGGGGAAGGGCACTGTGGTTACTCGGTGGCAGCCTCAAATCTCTGGGTCCCTT--------CCCCTTGACAGTCTGTGGTATAGCTGGCCCCAGGGCCTA 292
Bos     ATGAAAGGG--CATGTGGCCACTGGGCCAT---CTCCAACCTCTTGGCCCCTT---------CCCCTGGCA---TCTGATGCAGCTAGCCCCAAGGCCTA 266
Canis   ATGGAAATGGTACTGTGGTCACTGGGCAATAGCCTCAAATCTCTTGGCCCCAT---------GCCTTGACAGTCTGTGGTGCAGCTGGCCCTAAGGCCTA 274

Homo    CTTCACAGATGTGTGAAAAGAGCGTCGAGGGGGCTGGGAATGTTGCCTGACTACACTGTCTGGTCCCTCCCT-GCCCTCAG 380
Pan     CTTCACAGATGTGTGAAAAGAGCGTCGAGGGGGCTGGGAATGTTGCCTGACTACACTGTCTGGTCCCTCCCT-GCCCTCAG 372
Macaca  CTTCACAGATGTGTGGAAAGGGTGTCCAGGGGGCTGGGAATGTTGCCTGACTACACTGTCTGGTCCCTCCCC-GCCCTCAG 372
Bos     CTTCACATA-AAGGTGAAAGGGTATCCAGGGCTCCAGGAATGTCACTTGACCACCCTGTCTGGTCCCTCCTTGGCTTCCAG 346
Canis   CTTCACAA--GGGATGAAAGTGCATCCAGGGGGCTGGGAATGTCA-CTGACCACCTTATCTGCTTCCTCCCTGGCCTCCAG 352
```

ENSG00000136750 intron 1

Description: Glutamate decarboxylase 2 (GAD2)
Intron number: 1
Human chromosome: 10
Intron start (bp): 26545821
Human intron length : 724
Intron alignment length: 748
Flanking exons length (upstream/downstream): 76/60
SNP density: 0.004144
K tree score: 0.0331
Scaling factor: 0.8949
Human-chimpanzee distance: 0.016778
Total primate branch length: 0.0793

ENSG00000136750 exon 1

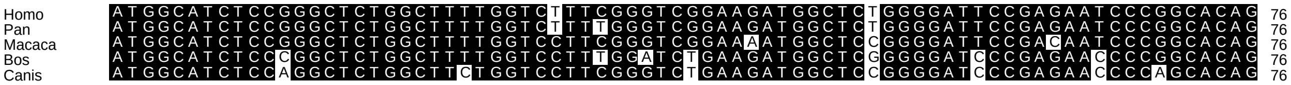

ENSG00000136750 exon 2

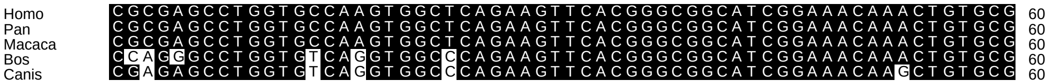

ENSG00000136750 intron 1

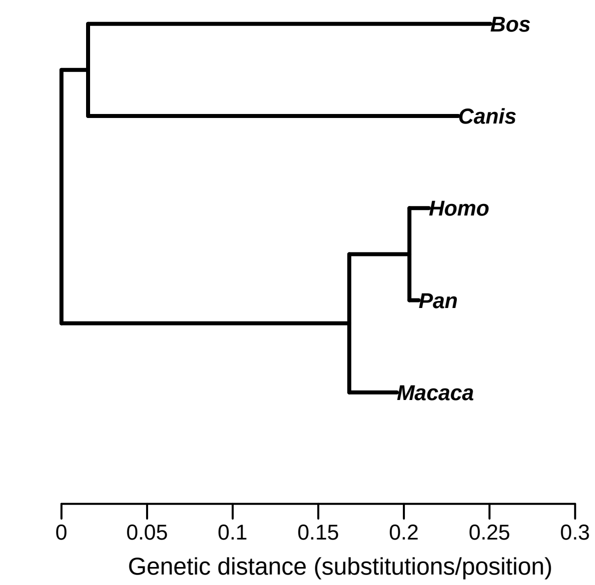

ENSG00000136750 intron 1

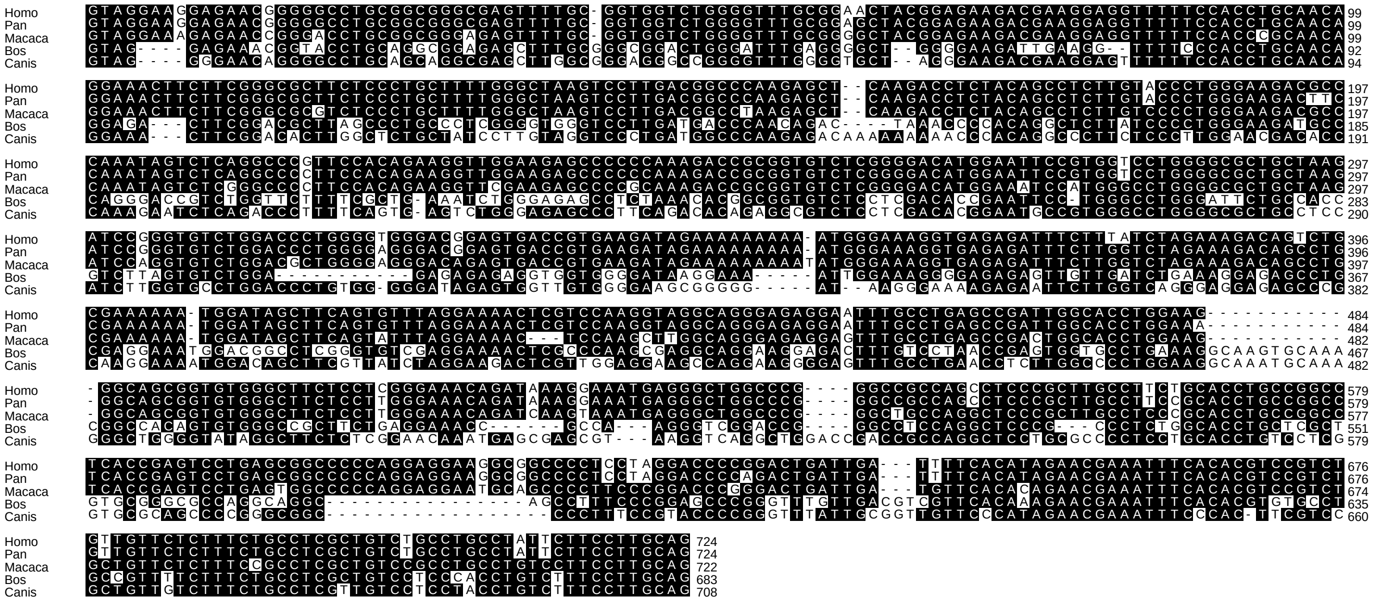

ENSG00000078898 intron 9

Description: Bactericidal/permeability-increasing protein-like 1 precursor (BPIL1)
Intron number: 9
Human chromosome: 20
Intron start (bp): 31070583
Human intron length : 464
Intron alignment length: 576
Flanking exons length (upstream/downstream): 54/171
SNP density: 0.004310
K tree score: 0.0615
Scaling factor: 0.7318
Human-chimpanzee distance: 0.002188
Total primate branch length: 0.079

ENSG00000078898 exon 9

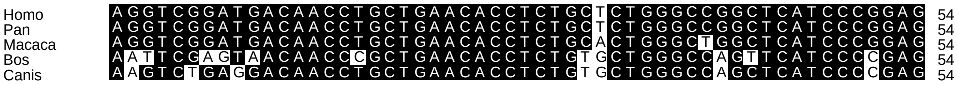

ENSG00000078898 exon 10

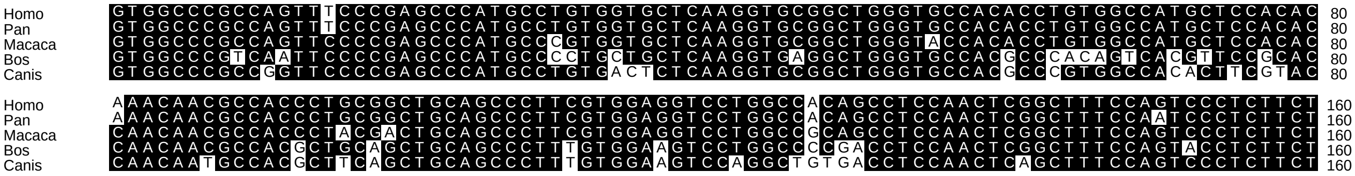

ENSG00000078898 intron 9

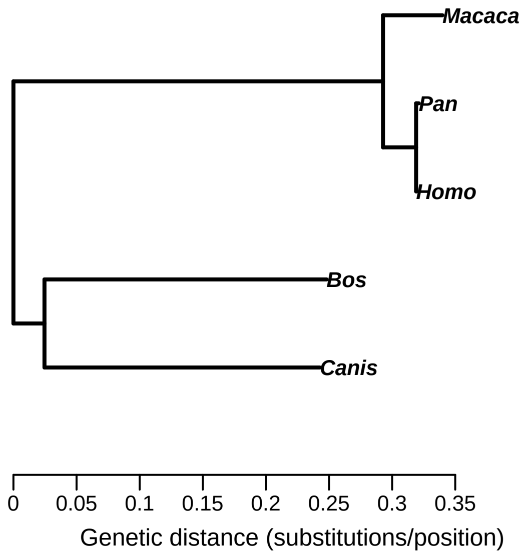

ENSG00000078898 intron 9

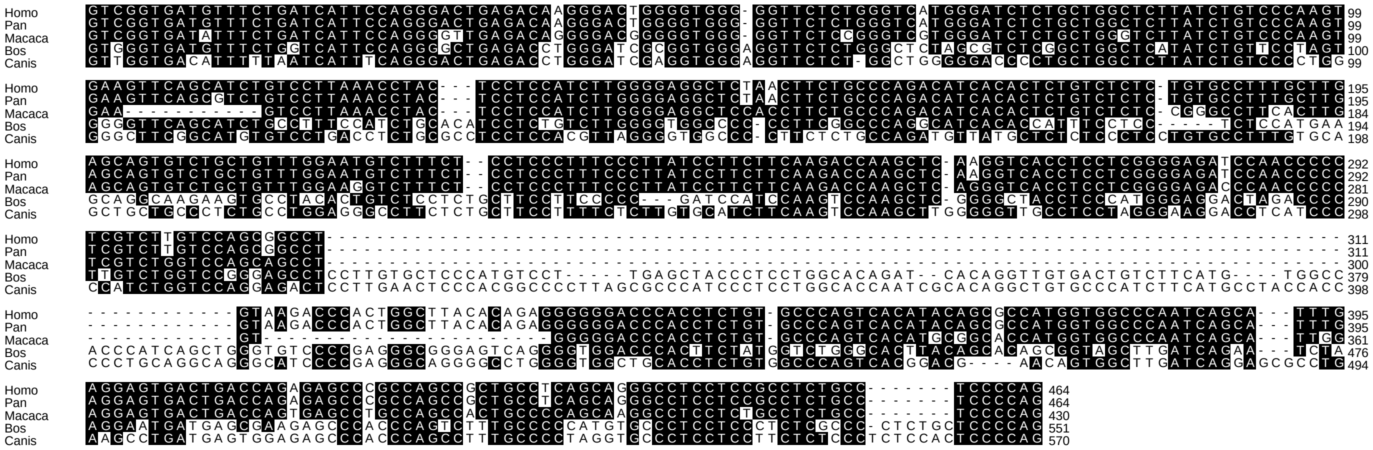

ENSG00000174996 intron 10

Description: Kinesin light chain 2 (KLC2)
Intron number: 10
Human chromosome: 11
Intron start (bp): 65789283
Human intron length : 419
Intron alignment length: 511
Flanking exons length (upstream/downstream): 68/109
SNP density: 0.000000
K tree score: 0.0935
Scaling factor: 0.8032
Human-chimpanzee distance: 0.009631
Total primate branch length: 0.0789

ENSG00000174996 exon 10

```
Homo    GATAAGCGCCGGGACAGCGCCCCCTATGGGGAATACGGCAGCTGGTACAAGGCCTGTAAAGTAGACAG 68
Pan     GATAAGCGCCGGGACAGCGCCCCCTATGGGGAATATGGCAGCTGGTACAAGGCCTGTAAAGTAGACAG 68
Macaca  GATAAGCGCCGGGACAGCGCCCCCTATGGGGAATATGGCAGCTGGTACAAGGCCTGTAAAGTAGACAG 68
Bos     GATAAGCGCCGGGACAGCACCCCCTATGGGGAATATGGCAGCTGGTACAAGGCCTGTAAAGTAGACAG 68
Canis   GACAAGCGCCGGGACAGCACCCCCTATGGGGAATATGGCAGCTGGTACAAAGCCTGTAAAGTAGACAG 68
```

ENSG00000174996 exon 11

```
Homo    CCCCACAGTCAACACCACCCTGCGCAGCTTGGGGGCCCTATACCGGCGCCAGGGCAAGCTGGAAGCCGCGCACACACTAG 80
Pan     CCCCACAGTCAACACCACCCTGCGCAGCTTGGGGGCCCTATACCGGCGCCAGGGCAAGCTGGAAGCCGCGCACACGCTAG 80
Macaca  CCCCACAGTCAACACCACCCTGCGCAGCTTGGGGGCCCTATACCGACGCCAGGGCAAGCTGGAAGCCGCACACACACTGG 80
Bos     CCCCACGGTCAACACCACGCTGCGCAGCTTGGGGGCCCTGTACCGGCGCCAGGGCAAACTGGAAGCCGCACACACGCTGG 80
Canis   CCCCACAGTCAACACTACCCTGCGCAGCTTGGGAGCCCTATACCGGCGCCAGGGGAAACTAGAAGCTGCACATACCCTGG 80

Homo    AGGACTGTGCCAGCCGTAACCGCAAGCAG 109
Pan     AGGACTGTGCCAGCCGTAACCGCAAGCAG 109
Macaca  AGGACTGTGCCAGCCGCAACCGCAAGCAG 109
Bos     AGGACTGTGCCAGCCGCAGCCGCAAGCAG 109
Canis   AGGACTGTGCCAGCCGCAGCCGCAAACAG 109
```

ENSG00000174996 intron 10

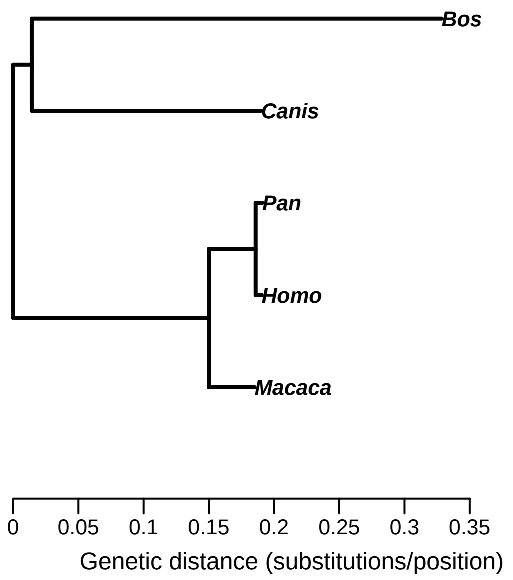

ENSG00000174996 intron 10

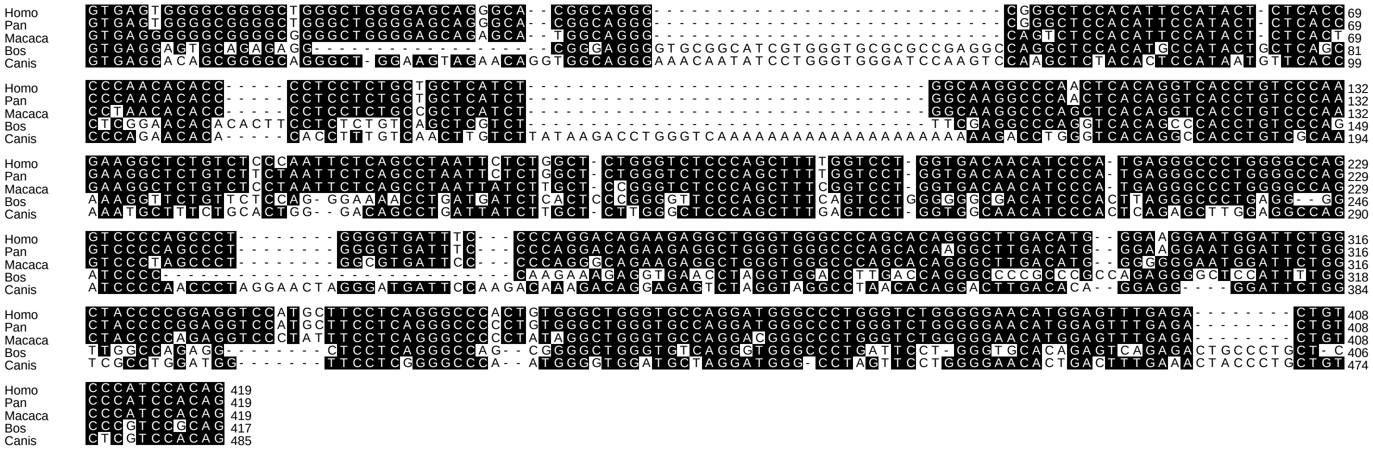

ENSG00000123213 intron 5

Description: Neurolysin, mitochondrial precursor (NLN)
Intron number: 5
Human chromosome: 05
Intron start (bp): 65111865
Human intron length : 979
Intron alignment length: 1033
Flanking exons length (upstream/downstream): 103/161
SNP density: 0.000000
K tree score: 0.0758
Scaling factor: 0.4917
Human-chimpanzee distance: 0.009301
Total primate branch length: 0.0788

ENSG00000123213 exon 5

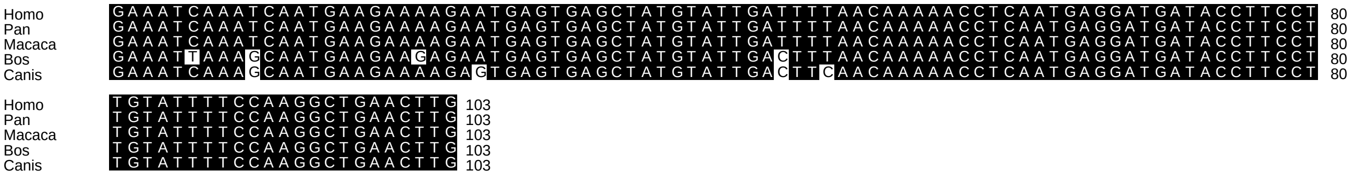

ENSG00000123213 exon 6

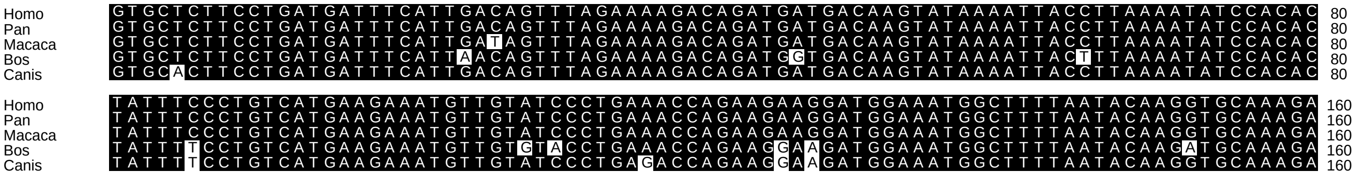

ENSG00000123213 intron 5

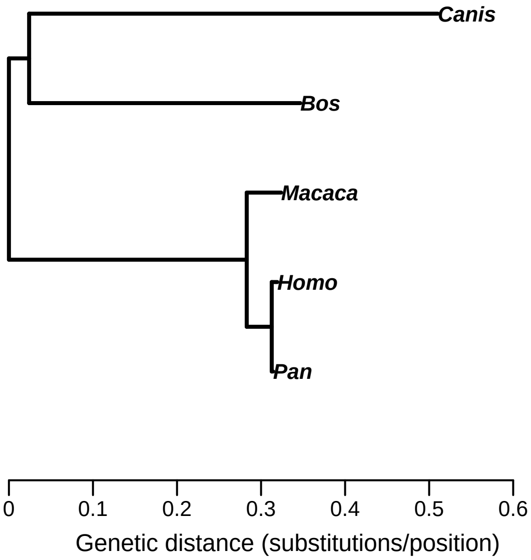

ENSG00000123213 intron 5

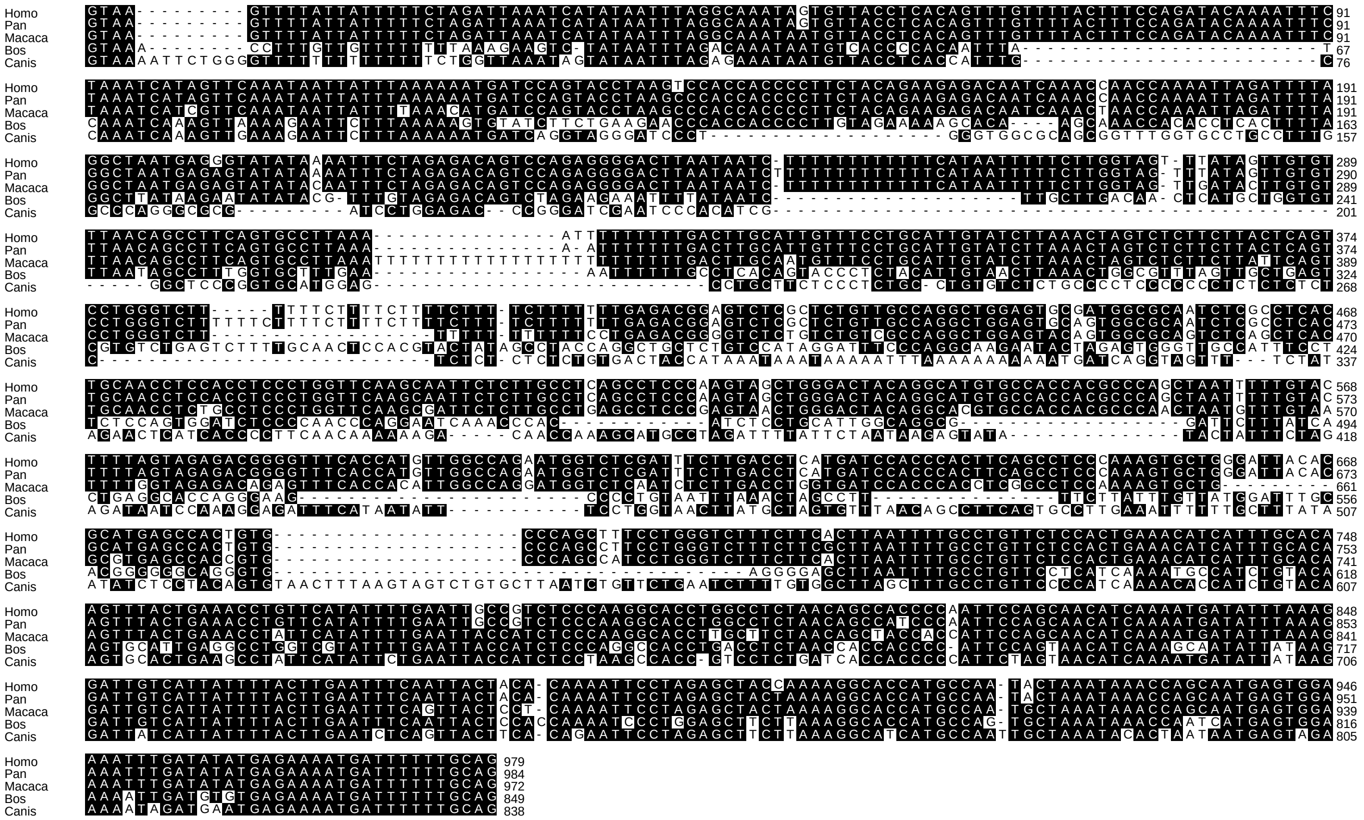

ENSG00000094804 intron 6

Description: Cell division control protein 6 homolog (CDC6)
Intron number: 6
Human chromosome: 17
Intron start (bp): 35704282
Human intron length : 852
Intron alignment length: 1584
Flanking exons length (upstream/downstream): 140/101
SNP density: 0.005869
K tree score: 0.0911
Scaling factor: 0.5206
Human-chimpanzee distance: 0.013071
Total primate branch length: 0.0787

ENSG00000094804 exon 6

```
Homo    GTATTGCTAATACCCTGGATCTCACAGATAGAATTCTACCTAGGCTTCAAGCTAGAGAAAAATGTAAGCCACAGCTGTTG 80
Pan     GTATTGCTAATACCCTGGATCTCACAGATAGAATTCTACCTAGGCTTCAAGCTAGAGAAAAATGTAAGCCACAGCTGTTG 80
Macaca  GTATTGCTAATACCCTGGATCTCACAGATAGAATTCTGCCTAGGCTTCAAGCTAGAGAAAACTGTAAGCCACAGCTGTTG 80
Bos     GTATTGCTAATACTCTGGATCTCACAGACAGAATTCTGCCGAGGCTTCAAGCTAGAGAAAAATGCAAGCCACAGCTGTTG 80
Canis   GTATTGCTAACACTCTAGATCTCACGGACAGAATTCTGCCGAGGCTTCAAGCTAGAGAAAAATGTAAGCCGCAGCTGTTG 80

Homo    AACTTCCCACCTTATACCAGAAATCAGATAGTCACTATTTTGCAAGATCGACTTAATCAG 140
Pan     AACTTCCCACCTTATACCAGAAATCAGATAGTCACTATTTTGCAAGATCGACTTAATCAG 140
Macaca  AACTTCCCACCTTATACCAGAAATCAGATAGTCGCTATTTTGCAAGATCGACTTACTCAG 140
Bos     AACTTCCCACCTTATACCAAAAATCAGATAGCCACCATCTTGCAGGATCGACTGAATCAG 140
Canis   AACTTCCCACCTTATACCAAAAATCAGATAGCTACTATCTTGCAGGAGCGGCTTGATCTG 140
```

ENSG00000094804 exon 7

```
Homo    GTATCTAGAGATCAGGTTCTGGACAATGCTGCAGTTCAATTCTGTGCCCGCAAAGTCTCTGCTGTTTCAGGAGATGTTCG 80
Pan     GTATCTAGAGATCAGGTTCTGGACAATGCTGCAATTCAATTCTGTGCCCGCAAAGTCTCTGCTGTTTCAGGAGATGTTCG 80
Macaca  GTATCTAGAGATCAGGTTCTGGACAATGCTGCAATTCAATTCTGTGCCCGCAAAGTCTCTGCTGTTTCAGGAGATGTTCG 80
Bos     GCATCTAATGACCAGGTCCTAGACAATGCTGCCATTCAGTTCTGCGCCCGCAAAGTCTCTGCTGTTTCGGGAGATGTTCG 80
Canis   GTATCTAGAGCTCAGGTTCTGGATAATGCTGCCATTCAGTTCTGTGCCCGAAAAGTCTCTGCTCTTTCAGGAGACGTTCG 80

Homo    CAAAGCACTGGATGTTTGCAG 101
Pan     CAAAGCACTGGATGTTTGCAG 101
Macaca  CAAAGCACTAGATGTTTGCAG 101
Bos     CAAAGCGCTTGATATTTGCAG 101
Canis   AAAAGCGCTAGATGTTTGCAG 101
```

ENSG00000094804 intron 6

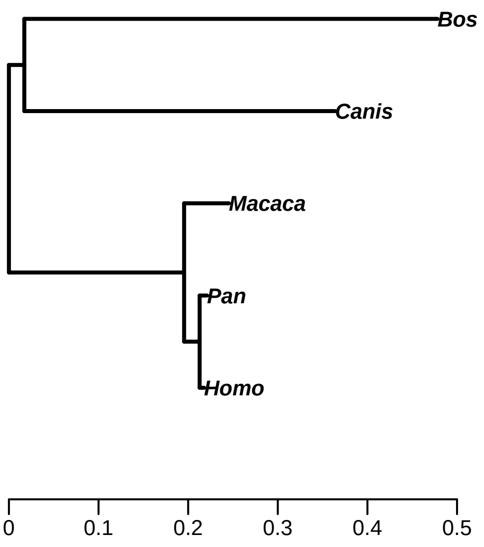

Genetic distance (substitutions/position)

ENSG00000094804 intron 6

```
Homo   GTCAGTGCCAACTATTTTGCCAAATTATGTGTTCCTTGGATCACCTGTGCTAGGAAAATTTAACAATAGTTCTAAAATATTTTT------------GCCT 88
Pan    GTCAGTGCCAAATATTTTGCCAAATTATGTGTTCCTTGGATCACCTGTGCTAGGAAAATTTAACAATCGTTCTAAAATATTTTT------------GCCT 88
Macaca GTCAGTGCCAACTATTTTGCCAAATTATGTGTTTCTTGGATCACCTGTGCTAGGAAAATTTAACAATAGTTTTAAAATATTTTT------------GCCT 88
Bos    GTGAGTGCCAAGCACTGTACCCAGGTGTCTGTTCCTTGTATCACCTGTGAAAGGGAAGCCTAACAATAGTTCTAAAATATTTT-------------GCTT 87
Canis  GTAAGTGCCCCCCATTGTACCACATTATGTGTCCCTTGGATCACCTCTGGTGGGGAAACCTAACA---GTCCTAAAATATTTTTGCTTAGAGGGGCGCCT 97

Homo   TGATTGCTTTATTAATGG---------------------------------------------------------------------------------- 106
Pan    TGATTGCTTTATTAATGG---------------------------------------------------------------------------------- 106
Macaca TAATTGCTTTATTAATGG---------------------------------------------------------------------------------- 106
Bos    TGACTGCATGGCTA-------------------------------------------------------------------------------------- 101
Canis  GGGTGGCTCAGTCAGTTGAGGATCTGACTCTTGATCTCAGCTCAGATCTCAGTCTTAGGGGTGTGAGTTCAAGCCCCACACTGGGCTCCACAATGGGTGT 197

Homo   ----------------------------------------------AGCAAGGAGTTCCTATGGACCATAG----------------------------- 131
Pan    ----------------------------------------------AGCAAGGAGTTCCTGTGGACCATAG----------------------------- 131
Macaca ----------------------------------------------AGCAAGAAGTTCCTGTGGACCATAG----------------------------- 131
Bos    ----------------------------------------------AACAAGGCATTCCTTTGGGCCATAG----------------------------- 126
Canis  GGAGCTTACTTAAAATTTTTTATTTTTTGCTTAGATTGCTTTCCTCAGCAGGGAGTTACTGTGGGCCATAGTGAGAACATTTTTTTTCCCCCAAAGATTT 297

Homo   ---------------------------------------------------------------------------------------------------- 131
Pan    ---------------------------------------------------------------------------------------------------- 131
Macaca ---------------------------------------------------------------------------------------------------- 131
Bos    ---------------------------------------------------------------------------------------------------- 126
Canis  TATTTATCCATTCATGAGAAACACAGAGAGAGGCAGAGATACAGGCAGAGGGAGAAGCTGGCTCCATGCAGGGAGTCGGACGTGGGACTCAATCCTGGGA 397

Homo   --------------------------------------------------------------------TGAGAACATTTTTATGTGAATTTCAGCTTCA- 162
Pan    --------------------------------------------------------------------TGAGAACATTTTTATGTGAATTTCTGCTTCA- 162
Macaca --------------------------------------------------------------------AGAGAACATTTTTATGTGAATTTCTGCTTCA- 162
Bos    --------------------------------------------------------------------TGAGAACA-TTTGGTGTGAATTTCCACCCATG 157
Canis  CTCCAGGATCACGCCCTGGGCTGAAGGCAGGCACCAAACCGCCGAACCACCCAGGGATCCCCCAATATTGAGAACATTTTTATATGAATTTCCACTCAAG 497

Homo   --TTCATTTACTGGGTGTCTCAGTGTTGAAATAAAAGCAGGA--------GTTAGAGGAATCACCCAAGCGCTTTTTTTTTTTTTTTTAGAT-GAGTTTT 251
Pan    --TTCATTTACTGGGTGTCTCAGTGTTGAAATAAAAGCAGGA--------GTTAGAGGAATCACCCAAGTGC-TTTTTTTTTTTTTTTAGAT-GAGTTTT 250
Macaca --TTCATTTACTGGATGTCTCAGTGTTGAAATAAAATCAGGA--------GTTAGAGGAA-----------------------TTTTGAGATGGAGTTTT 229
Bos    GCTCCATTTATTAGGTGCTTCAGTATTGGAATAAACATAAGA------------GAGGAGTCACTCAAATGCTTCTCTAATAGTCTCAT----GGAGCAG 241
Canis  GCTCCATTTATTGGATGCCTCACTGTTGGAACAAAAATCAGAGAGGCCTTGGTTGCTCAGTCAGTTAAGCATCCCACTATTGATTTCAGCTTGGGGTTTG 597

Homo   GCTCTTATCGCCC-----------------------------------------------AGGCTGGAGTGCAATGGCGTGATCTCGGCTCTCTACAGCC 304
Pan    GCTCTTATCGCCC-----------------------------------------------AGGCTGGAGTGCAATGGCGTGATCTCGGCTCTCTACAGCC 303
Macaca GCTCTTGTAGCCC-----------------------------------------------AGGCTGGAATGCAATGGCATGATCTCAGCTCTCTACAACC 282
Bos    GAAATGGCAACCCACTCCAGTATTCTTGCCTGGAGAATTTCATGGACAGAGGAGCCTGGCAGGCTGCAGTCTATGGGGTTGCAAACAGTTGGACATGA-- 339
Canis  GTTTTTTTTTTTTTTTTTTTTTTTTTTTTTTTTAGGATTTTATTTATTTATTCATGAGAGACACAGAGAGGCAGAGACATAGGCAGAGGGAGAAGCAGGC 697

Homo   TCTGCCTCACGGGTTC----------------------------------AAGCGATTCTCCTGC--CTCAGCCTCCCCAGTAGC--------------- 353
Pan    TCTGCCTCACGGGTTC----------------------------------AAACGATTCTCCTGC--CTCAGCCTCCCCAGTAGC--------------- 352
Macaca TCTGCCTCCCGGGTTC----------------------------------AAGCAATTCTTCTGC--CTCAGCCTCCCGAGTAAC--------------- 331
Bos    -CTGAGCGACGAACAC-----ACACACAATAGTCTTAAAATAGGACCTGAGGGGGCTTCCCTGGTGGCTCAGTGGTAAAGA---------------ATCT 418
Canis  TCCCTGTGGTGAGCCCAGTGCGGGGACCCCAGGATGAAGACGTGAGCCAAAGACAGATGCTCAACCACTGAGCCACCCAGGCATCCCTCAGCTCAGATCT 797

Homo   ------TAGGACTACAGGCACCTGCCACCACGCCCAGCTAATTGTTG-------TATTTTTAGTAGAGCTGGGGTTTCACCGTCTTGGCCAGGCTGGTCT 440
Pan    ------TAGGACTACAGGCACCTGCCACCACGCCCAGCTAATTGTTG-------TATTTTTAGTAGAGCTGGGGTTTCACCGTCTTGGCCAGGCTGGTCT 439
Macaca ------TGGGGTTACA-GCACCCGCCACCACGCCCAGCTAATTGTTA-------TATTTTTAGTAGAGCTCGGGTTTCACCATGTTGGCCAGGCTGGTCT 417
Bos    GCCTGCCAGTGCAGGAGACACGGGTT-CCATCCCTGATCCGGGA--GGATCCCACAAGCCTTGGAGCAATTGAGCCTGGCAACCGCAACTACTGGAGTCT 515
Canis  TGATCTCAGGGTTGTGAGTTCAAGCC-CCATGCTGGGCTCTGTATTGGGTTGCACACTCAGTGTAGAGTCTGATTCTCCCTCTGCTCTTCCCCCTGCTCC 896

Homo   CGAACTC-----------------------------------CTAACCTCAGGTGATATGCCTGTCTCGGCCTCT------------------------- 480
Pan    CAAACTC-----------------------------------CTAACCTCAGGTGATATGCCTGTCTGGGCCTCT------------------------- 479
Macaca CGAACTC-----------------------------------CTAACCTCAGGTGATATGCCTGTCTCGGCCTC-------------------------- 456
Bos    GGGCTTC------------------ACAACAGGAGAAGCCCGTGCACCACAGTAGAGCGTAGCTGCTGCTTGCCACAACCACAGAAAGCCCACGCAGCAG 598
Canis  TTCCCTCTCCCCGCTTTTGTATGGACACTTTTTAAGTTTATTTGCTTTTAGCAATCTCTCTCTCTCTCTCTCTTTTTTTTTAAAGATGTTCTTTATTCAT 996

Homo   --------------------------------------------------------------------------------CAAAGTGCTGGGATTAC--- 497
Pan    --------------------------------------------------------------------------------CAAAGTGCTGGGATTAC--- 496
Macaca --------------------------------------------------------------------------------CAAAGTGCTCAGATTAC--- 473
Bos    TGAAGACCCAGCACAGCCGAAAAGAAACAGAAAAAAAAAAAA--------------------------------------ATAGGACCTGGGACTTCCCT 660
Canis  GAGAGACACAGAGAGAGGCAGAGGCTTAGTCAGAGGAAGAAGCAGGCTCCCCACAGGGAGCCCAATGTGGGACTCGATTCCAGAACTCTGGGATCACGCC 1096

Homo   ---------AGGCATGAGCCATCGCACCCAGCCTCAAGTGCT-----TTCTTTAATGACCTTTA------------------------------------ 547
Pan    ---------AGGCATGAGCCATCGCACCCGGCCTCAAGTGCT-----TTCTTTAATGACCTTTA------------------------------------ 546
Macaca ---------AGGTGTGAGCCACTGTACCCGGCCTCAAGTGCT-----TTCTTTAATGACTTTTA------------------------------------ 523
Bos    GGCGGTCCAGTGGCTAAGACTTTGCACTTCAATGCAGGGGGG----------CACAGCTCCTATCCCTGGTCGGGGAACTAAGAT--------------- 735
Canis  CTGGGCCAAAGGCAGATGCTCAGCCGCTGAGCCACACAGGCCTCCCATTTTTTAGTAATCTCTACGCCCAATGTGAGGCTCAAGCTCATTACCCCAAGAT 1196

Homo   ---------------------------------------------------------------AAAAATAGGGCCTATGAAAAAACTTTTTCTGTTGAAA 584
Pan    ---------------------------------------------------------------AAAAATAGGGCCTATGAAAAAACTTTTTCTGTTGAAA 583
Macaca ---------------------------------------------------------------AAAAATAGGGCCTATGAAAAAACTTTTTCTGTTGAAA 560
Bos    -----CCCATATGTCTCATGGTCTGGCCAAAAAATTTTTTTT---------------------AAAAATAGGACC-AAGAAAACACTTTCTC-------- 800
Canis  CAAGACTCGCATGCTCTTCCAACTGCCAGCGAGGCACCTCTCAATAGTTTTATAATGGCCTGTAAAAATGGGGCCTTTGAGAAAACTTTCTT--TTGAAA 1294

Homo   ATGACTAAATAGATTTT------TTAT--TTTTGCTAATTTC-AGTTCTGATTGC-TTTTTTCCATTTATGAACTTCTACTGCATCCGTACAAAAAGCTT 674
Pan    GTGACTAAATAGATTTT------TTAT--TTTTGCTAATTTC-AGTTCTGATTGC-TTTTTTCCATTTGTGAACTTCTACTGCATCCGTACAAAAAGCTT 673
Macaca GTAACTAAATAGATATT------TTAT--TTTTGCTAATTTC-AGTTCTGATTGCTTTTTTTCCATTTATGAACTTCTACCACATCCATACAAAATGCTT 651
Bos    TTGACCAAGTAGATTTTAGTATAGTGTAGTTTTGCTAATTTA-AGTTTCAGTTGCTTTTTTCCCCTTTGTGAACCTCCACCACATTCCTACAAAATGCTT 899
Canis  ATGACCAAATAGATTTTATTTTATTCA--TTTTGCTAATTTCTGGTTGCTCTCCCCCCCCCCCCATTCCTGAGCTTCTACCACATGCCTACAAAATGTAT 1392

Homo   AAATGTGTGAAAACTCTCAAACGTCTCCATTATTTATGAGAAGAAAAGGCATCTTTTTAACCCTAAAGAGTTGCCAATCAAGTTTAGGGTTAAATGTGAA 774
Pan    AAATGTGTGAAAACTCTCAAACGTCTCCATTATTTATGAGAAGAAAAGGCATCTTTTTAACCCTAAAGAGTTGCCAATCAAGTTTAGGGTTAAATGTGAA 773
Macaca AAATGTGTGAAAACTCTCAAATATCTCCATTATTTATGAGAAGAAAAGGCATCTTTTTGACCCTAAGGAGTTGCCAATCAAGTTTAGGGTTAAATGTGAA 751
Bos    CAATGTATGAAATTCCC-------------ATTCCTAAGCAGAAAAAGGCATCTCTTAGACCCTAAAGTTTGGTCAATCAAGTTTAGAATTAAGTGTGAA 985
Canis  AAATGCATAAATTTTTC----------------------------------TCTCTTGGACCCTAAAGGATGGCCAGTCAAGTTTAGACTT-AATGTGAA 1457

Homo   AATATTTATGCTTGGGACCTAAATTACAGAG--GGAGAATCT----TATGGTTCAAACTGTCATCTTCTATGTCTTGTCTGAAG 852
Pan    AATATTTATGCTTGGGACCTAAATTACAGAG--GGAGAATCT----TATGGTTCAAACTGTCATCTTCTATGTCTTGTCTGAAG 851
Macaca AATATTTATGCTTGGGACATAAATTACCGAG--GGAGAATCT----TATAGTTCAAACTGTCATATTCTATGTCTTGTCTTAAG 829
Bos    AGTACTCATGTCTGAGACCTAAATTGCAGAA--GAGGAGCTG----TGTACTTTCAACTGTAAACTTCTGTGTCTTGTCCAAAG 1063
Canis  AATATTTATGTCTGGGACCTAAATTACAGAGAAGGGGAGTCTCATATATAGTTTATGCTGTCATCTTCTATGTCTTGTCCAAAG 1541
```

ENSG00000140274 intron 1

Description: dual oxidase activator 2 (DUOXA2)
Intron number: 1
Human chromosome: 15
Intron start (bp): 43194243
Human intron length : 1046
Intron alignment length: 1181
Flanking exons length (upstream/downstream): 147/58
SNP density: 0.001912
K tree score: 0.0313
Scaling factor: 0.8591
Human-chimpanzee distance: 0.016899
Total primate branch length: 0.0786

ENSG00000140274 exon 1

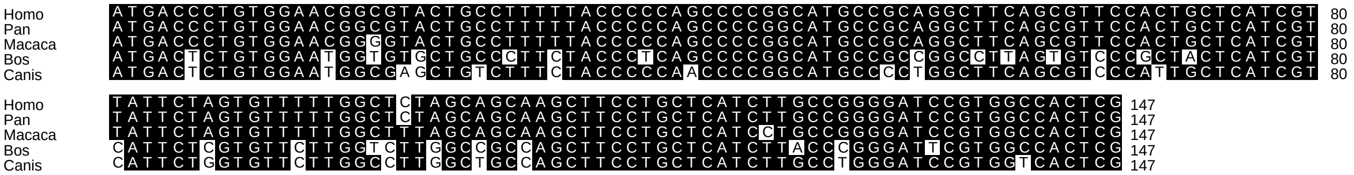

ENSG00000140274 exon 2

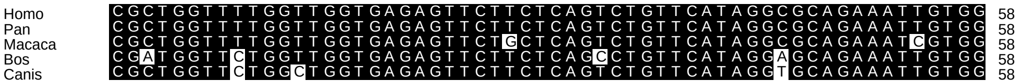

ENSG00000140274 intron 1

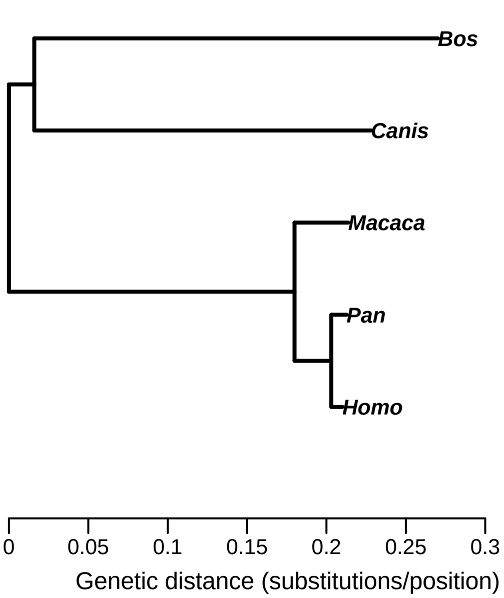

ENSG00000140274 intron 1

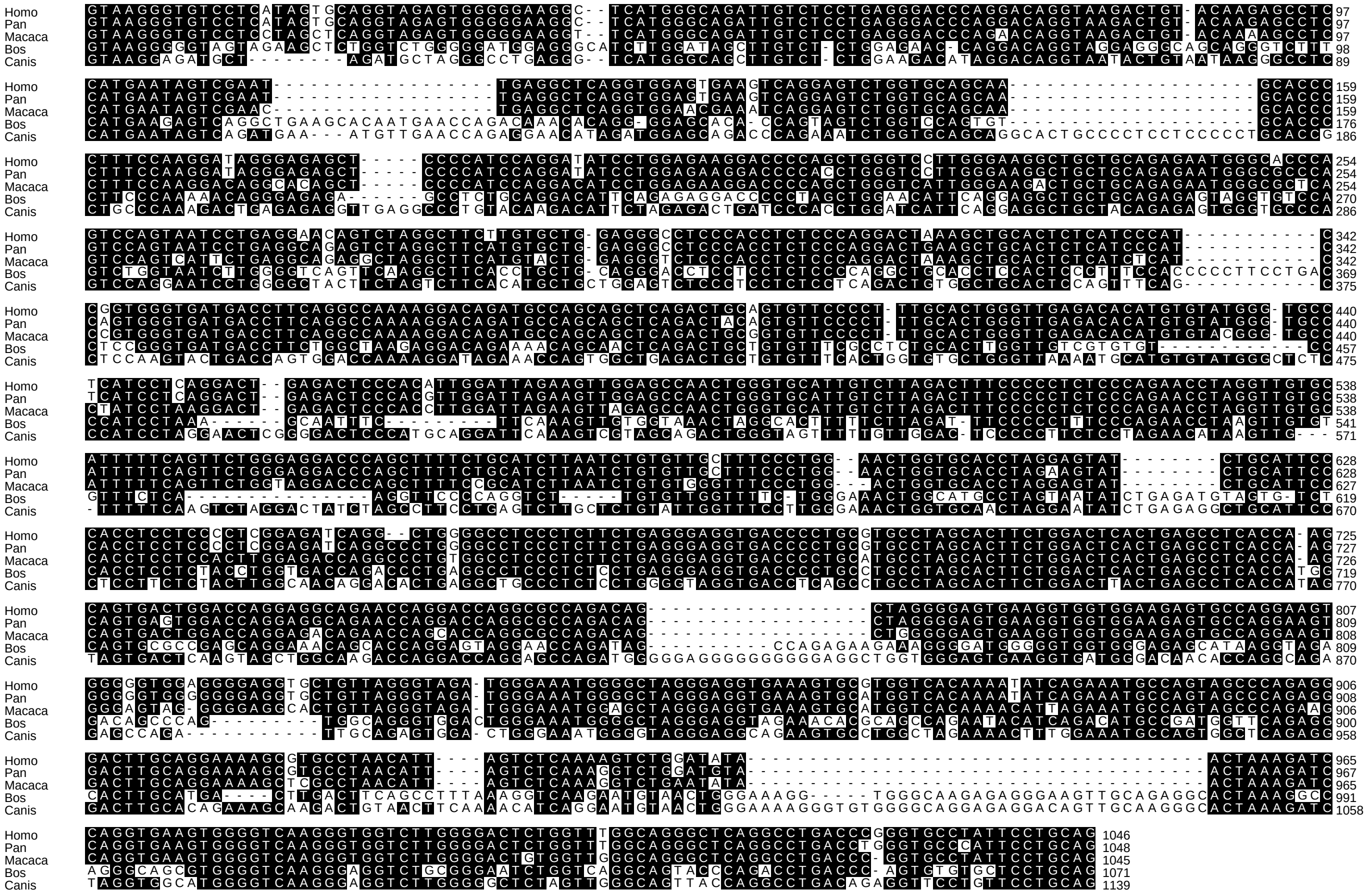

ENSG00000134028 intron 7

Description: ADAM DEC1 precursor (ADAMDEC1)
Intron number: 7
Human chromosome: 08
Intron start (bp): 24311204
Human intron length : 734
Intron alignment length: 768
Flanking exons length (upstream/downstream): 63/72
SNP density: 0.002725
K tree score: 0.0204
Scaling factor: 0.9127
Human-chimpanzee distance: 0.020836
Total primate branch length: 0.0786

ENSG00000134028 exon 7

```
Homo    AAAGAAGACTTTCTTCGGGCACAGAAATACATTGATCTCTATTTGGTGCTGGATAATGCCTTT 63
Pan     AAAGAAGACTTTCTTCGGGCACAGAAATACATTGATCTCTATTTGGTGCTGGATAATGCCTTT 63
Macaca  CAAGAAGACTTTCTTCAGGCACAGAAATACATTGATCTCTATTTGGTGCTGGATAATGCCTTT 63
Bos     ATAGAAGAATTTCTTCAGGGTGAGAAATACATTGATCTGCTTTTGGTGCTGGATAATGCCTTT 63
Canis   CAAGAAGATTTTCTTCAGGCTGAGAAATATATTGATCTCTTTTTGGTGCTGGATAATGCCTTT 63
```

ENSG00000134028 exon 8

```
Homo    TATAAGAACTATAATGAGAATCTAACTCTGATAAGAAGCTTTGTGTTTGATGTGATGAACCTACTCAATGTG 72
Pan     TATAAGAACTATAATGAGAATCTAACTCTGATAAGAAGCTTTGTGTTTGATGTGATGAACCTACTCAATGTG 72
Macaca  TATAAGAACTATAATGAGAATCTAACTCTGATAAGAAGCTTTGTGTTTGATGTGATGAACCTACTCAATGTG 72
Bos     TATAAGATGTATAATGAGAATATAACTGTGATAAGAAGCTTTGTGTTTGATGTGATAAACCTACTCAATGTG 72
Canis   TATAACATATACAAGGGGAATCTAACTTCGATAAGAAGCTTTGTGTTTGATGTGATGAACCTACTCAATGTG 72
```

ENSG00000134028 intron 7

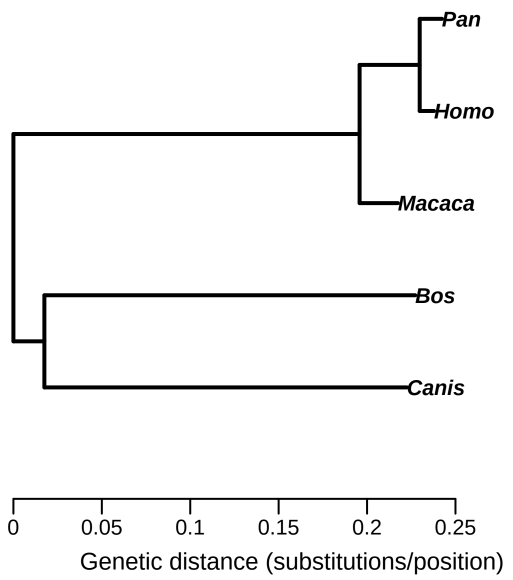

ENSG00000134028 intron 7

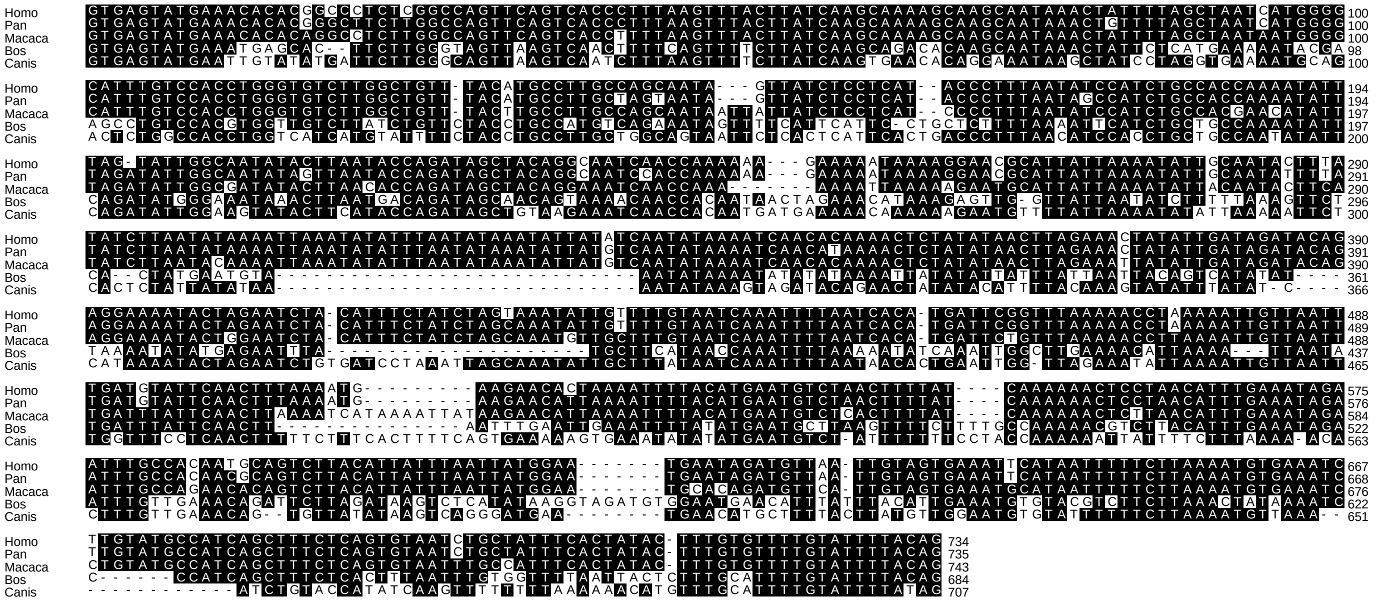

ENSG00000011021 intron 9

Description: Chloride channel protein 6 (CLCN6)
Intron number: 9
Human chromosome: 01
Intron start (bp): 11808859
Human intron length : 874
Intron alignment length: 938
Flanking exons length (upstream/downstream): 59/133
SNP density: 0.011442
K tree score: 0.0463
Scaling factor: 0.7421
Human-chimpanzee distance: 0.016185
Total primate branch length: 0.0784

ENSG00000011021 exon 9

```
Homo    TTTCAGAGCATCTCCTTACGGAAGATCCAGTTTAACTTCCCCTATTTCCGAAGCGACAG 59
Pan     TTTCAGAGCATCTCCTTACGGAAGATCCAGTTTAACTTCCCCTATTTCCGAAGCGACAG 59
Macaca  TTTCAGAGCATCTCCTTACGGAAGATCCAGTTTAACTTCCCCTATTTCCGAAGCGACAG 59
Bos     TTTCAGAGCATCTCCTTACGAAAGATCCAGTTTAACTTTCCCTATTTTCGAAGTGACAG 59
Canis   TTTCAGAGCATCTCCTTACGGAAGATCCAGTTTAACTTCCCCTACTTCCGAAGTGACAG 59
```

ENSG00000011021 exon 10

```
Homo    AGACAAGAGAGACTTTGTATCAGCAGGAGCGGCTGCTGGAGTTGCTGCAGCTTTCGGGGCGCCAATCGGGGGTACCTTGT 80
Pan     AGACAAGAGAGACTTTGTATCAGCAGGAGCGGCTGCTGGAGTTGCTGCAGCTTTCGGGGCGCCAATCGGGGGTACCTTGT 80
Macaca  AGACAAGAGAGACTTTGTATCAGCAGGAGCAGCTGCTGGAGTTGCTGCAGCTTTCGGGGCCCCAATCGGGGGTACCTTGT 80
Bos     AGACAAGCGGGACTTTGTATCAGCAGGGGCAGCTGCCGGAGTTGCTGCGGCTTTTGGGGCCCCCATTGGGGGCACCCTGT 80
Canis   AGACAAGCGAGACTTTGTATCAGCAGGGGCGGCTGCTGGAGTTGCTGCAGCTTTCGGGGCTCCCATCGGGGGTACCCTGT 80

Homo    TCAGTCTAGAGGAGGGTTCGTCCTTCTGGAACCAAGGGCTCACGTGGAAAGTG 133
Pan     TCAGTCTAGAGGAGGGATCGTCCTTCTGGAACCAAGGGCTCACGTGGAAAGTG 133
Macaca  TCAGTCTAGAGGAGGGTTCGTCCTTCTGGAACCAAGGGCTCACATGGAAAGTG 133
Bos     TCAGTCTGGAGGAGGGTTCGTCCTTCTGGAACCAAGGGCTCACATGGAGAGTG 133
Canis   TCAGTCTGGAAGAGGGCTCGTCCTTCTGGAACCAGGGGCTCACGTGGAAAGTG 133
```

ENSG00000011021 intron 9

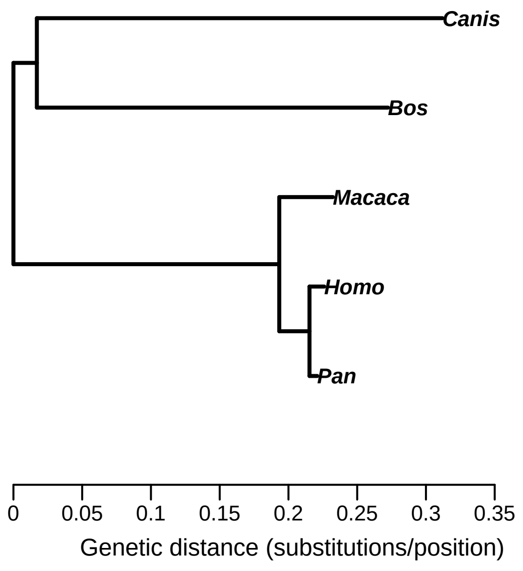

ENSG00000011021 intron 9

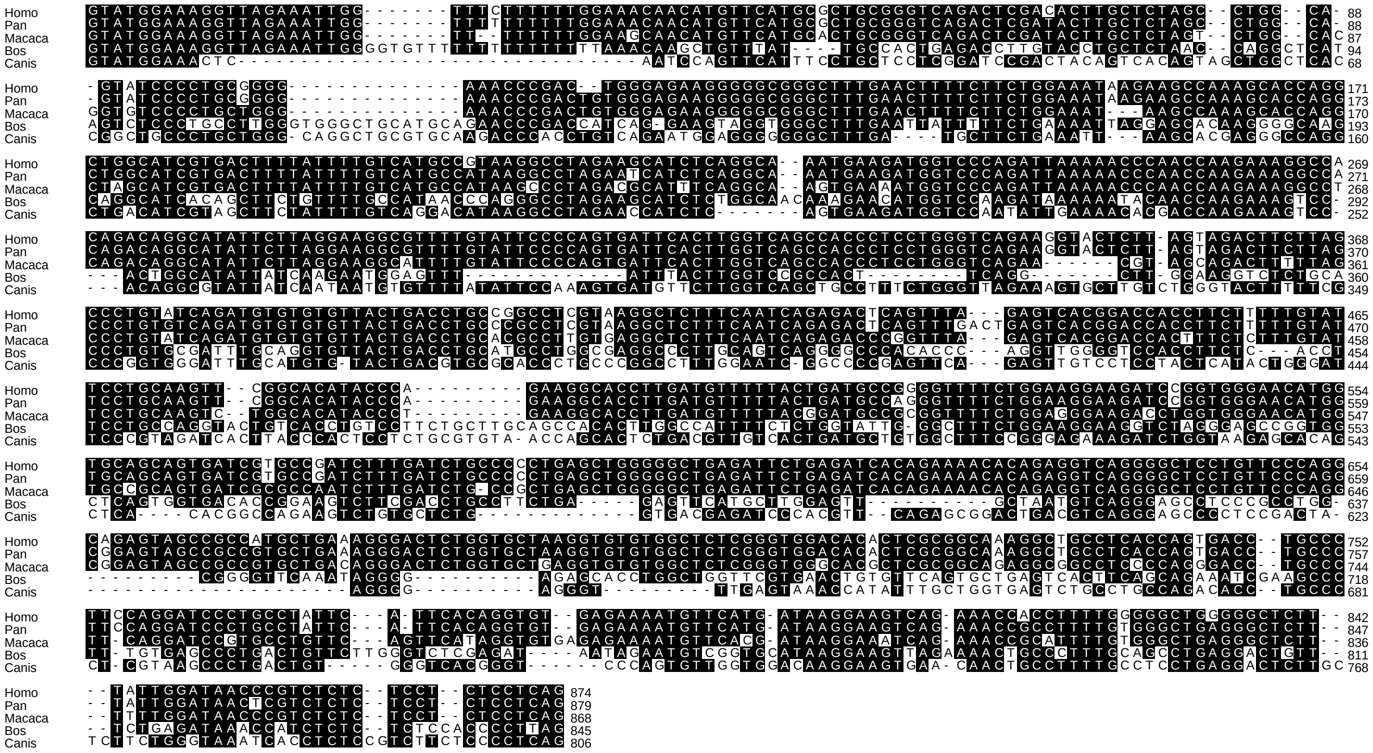

ENSG00000132323 intron 4

Description: integrin-linked kinase-associated protein phosphatase 2C isoform (ILKAP)
Intron number: 4
Human chromosome: 02
Intron start (bp): 238761638
Human intron length : 1595
Intron alignment length: 1710
Flanking exons length (upstream/downstream): 120/127
SNP density: 0.003135
K tree score: 0.0755
Scaling factor: 0.645
Human-chimpanzee distance: 0.012113
Total primate branch length: 0.0784

ENSG00000132323 exon 4

```
Homo    GTTCTCTTGCCACATCAATATCCCAGATGGTAAAGACTGAAGGGAAAGGAGCAAAGAGAAAAACCTCCGAGGAAGAGAAG 80
Pan     GTTCTCTTGCCACATCAATATCCCAGATGGTAAAGACTGAAGGGAAAGGAGCAAAGAGAAAAACCTCCGAGGAAGAGAAG 80
Macaca  GTTCTCTTGCCCCGTCAATATCCCAGATGGTAAAGAATGAAGGGAAAGGAGCAAAGAGAAAAACCTCCGAGGAAGAGAAG 80
Bos     GTTCTCTTGACACTTCGTTATCCGAGGAGGTAAAGAATGAAGGGAAAGGAGCAAAGAGAAAAGCCTCTGATGAAGAGAAG 80
Canis   GTCCTCTTGACACGTCGATGTCCCAGACGGTAAAGAGTGAAGGGAAAGGAGCAAAGAGAAAAGCCTCTGAAGAAGAGAAG 80

Homo    AATGGCAGTGAAGAGCTTGTGGAAAAAGAAAGTTTGTAAAG 120
Pan     AATGGCAGTGAAGAGCTTGTGGAAAAAGAAAGTTTGTAAAG 120
Macaca  AATGGCAGTGAAGAGCTTGTGGAAAAAGAAAGTTTGTAAAG 120
Bos     AATGGGAGTGAAGAGCTTGTGGAAAAAGAAAGTTTGTAAAG 120
Canis   AATGGCAGTGAAGAGCTTGTGGAAAAAGAAAGTTTGTAAAG 120
```

ENSG00000132323 exon 5

```
Homo    CCTCTTCGGTGATCTTTGGTCTGAAGGGCTATGTGGCTGAGCGGAAGGGTGAGAGGGAGGAGATGCAGGATGCCCACGTC 80
Pan     CCTCTTCGGTGATCTTTGGTCTGAAGGGCTATGTGGCTGAGCGGAAGGGTGAGAGGGAGGAGATGCAGGATGCCCACGTC 80
Macaca  CCTCTTCGGTGATCTTTGGTCTGAAGGGCTATGTGGCTGAGCGGAAGGGTGAGAGGGAGGAGATGCAGGATGCCCACGTC 80
Bos     CCTCTTCGGTGATCTTCAGCCTGAAAGGCTACGTGGCTGAGCGGAAGGGCGAGCGGGAAGAGATGCAGGATGCCCACGTC 80
Canis   CCTCCTCAGTGATCTTTGGTTTGAAAGGCTATGTGGCGGAGCGGAAGGGTGAGCGGGAGGAGATGCAGGACGCCCACGTC 80

Homo    ATCCTGAACGACATCACCGAGGAGTGTAGGCCCCCATCGTCCCTCAT 127
Pan     ATTCTGAACGACATCACCGAGGAGTGTAGGCCCCCATCGTCCCTCAT 127
Macaca  ATCCTGAACGACATCACCGAGGAGTGTAGGCCCCCATCGTCCCTCAT 127
Bos     ATCCTGAACGACATCACCGCGGAGTGCCAGCCCCCGTCCGCTCTCGT 127
Canis   ATCCTGAACGACATCACGGAGGAATGTAGGCCCCCATCGTCCCTCAT 127
```

ENSG00000132323 intron 4

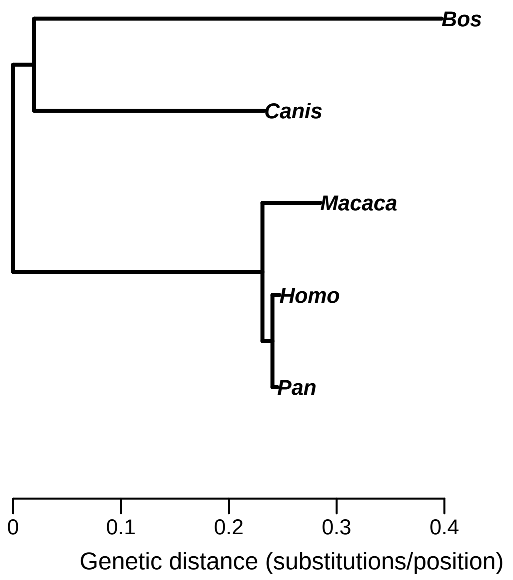

ENSG00000132323 intron 4

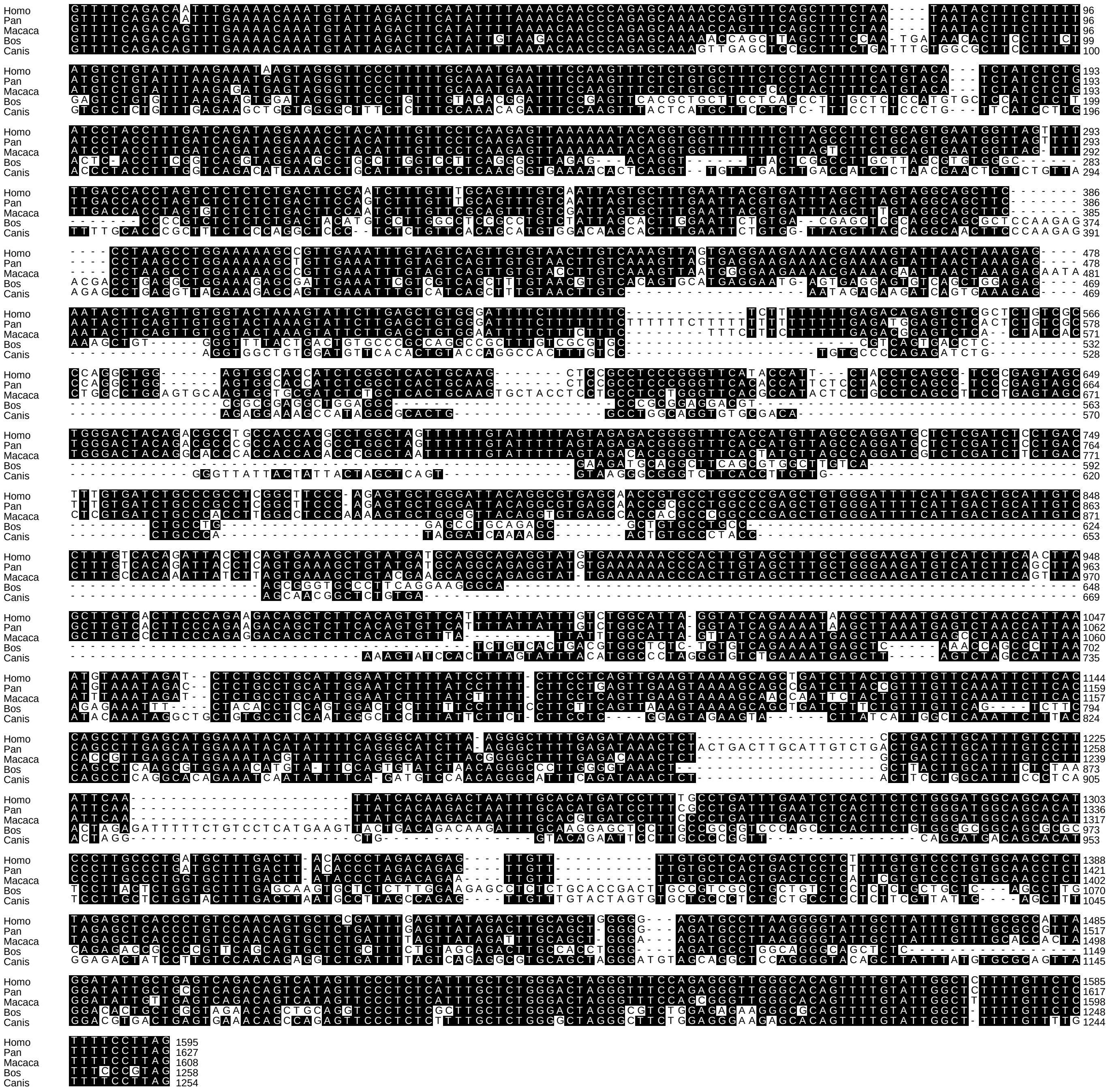

ENSG00000019505 intron 3

Description: Synaptotagmin-13 (SYT13)
Intron number: 3
Human chromosome: 11
Intron start (bp): 45230850
Human intron length : 1547
Intron alignment length: 1683
Flanking exons length (upstream/downstream): 135/302
SNP density: 0.001939
K tree score: 0.0539
Scaling factor: 0.8837
Human-chimpanzee distance: 0.013135
Total primate branch length: 0.0783

ENSG00000019505 exon 3

```
Homo    GTGTGGTGGAGGATGTCTGTGTCATGGAGACCTGGAACCCAGAGAAGGCTGCCAGTTGGAACCAGGCCCCCAAACTCCAC 80
Pan     GTGTGGTGGAGGATGTCTGTGTCATGGAGACCTGGAACCCAGAGAAGGCTGCCAGTTGGAACCAGGCCCCCAAACTCCAC 80
Macaca  GTGTGGTGGAGGATGTCTGTGTCATGGAGACCTGGAATCCAGAAAAGGCTGCCAGTTGGAACCAGGCCCCCAAACTCCAC 80
Bos     GTGTGGTGGAGGATGTCTGTGTCATGGAGACCTGGAACCCAGAGAAGGCTGCCAGTTGGAACCAAGCTCCCAAACTTCAC 80
Canis   GTGTGGTGGAGGATGTGTGTGTCATGGAGGCCTGGAACCCAGAAAAGGCTGCCAGTTGGAACCAAGCCCCCAAACTCCAC 80

Homo    TACTGCCTGGACTATGACTGTCAGAAGGCAGAATTGTTTGTGACTCGCCTGGAAG 135
Pan     TACTGCCTGGACTATGACTGTCAGAAGGCTGAATTGTTTGTGACTCGCCTGGAAG 135
Macaca  TACTGCCTGGACTATGACCGTCAGAAGGCTGAATTGTTTGTGACTAGCCTGGAAG 135
Bos     TACTGCCTGGACTATGACAGGCAGAAGGCCGAACTATTTGTGACTCGTCTGGAAG 135
Canis   TACCGCTTGGACTATGACCAGCAGAAAGCAGAACTGTTTGTGACTCGTCTGGAAG 135
```

ENSG00000019505 exon 4

```
Homo    CTGTGACCAGCAACCACGACGGAGGCTGTGACTGCTACGTCCAAGGGAGTGTGGCCAATAGGACCGGCTCTGTGGAGGCT 80
Pan     CTGTGACCAGCAACCACGACGGAGGCTGTGACTGCTACGTCCAAGGGAGTGTGGCCAATAGGACCGGCTCTGTGGAGGCT 80
Macaca  CTGTGACCAGCAACCATGATGGAGGCTGTGACTGCTATGTCCAAGGGAGTGTGGCCAATAGGACTGGCTCTGTGGAGGCG 80
Bos     CTGTGACCAGCGACCACGATGGAGGCTGTGACTGCTACGTCCAGGGCAGCGTGGCCAACAGCATGGGCTCCGTGGAGGCC 80
Canis   CTGTGACCAGCGGCCACGACGGAGGCTGTGACTGCTACATCCAAGGCAGCGTGACCAACAGGACCCGGACCGTGGAGGCC 80

Homo    CAGACAGCCCTAAAGAAGCGGCAGCTGCACACCACCTGGGAGGAGGGCCTGGTGCTCCCCCTGGCGGAGGAGGAGCTCCC 160
Pan     CAGACAGCCCTAAAGAAGCGGCAGCTGCACACCACCTGGGAGGAGGGCCTGGTGCTCCCCCTGGCGGAGGAGGAGCTCCC 160
Macaca  CAGACAGCCCTAAAGAAGCGGCAGCTGCACACCACCTGGGAGGAGGGCCTGGTGCTCCCCCTGGCGGAGGAGGAGCTCCC 160
Bos     CAGACGGCCCTCAAGAAGCGGGAGTTGCACACCATGTGGGAGGAAGGCCTGGTGCTCCCCCTGGCCGAGGAGGAGCTCCC 160
Canis   CAGACGGCTCTGAGGAAGCGGCAGCTGCACACCACCTGGGAGGAAGGCCTGGTGCTCCCCCTGGCGGAGGAGGAGCTGCC 160
```

ENSG00000019505 intron 3

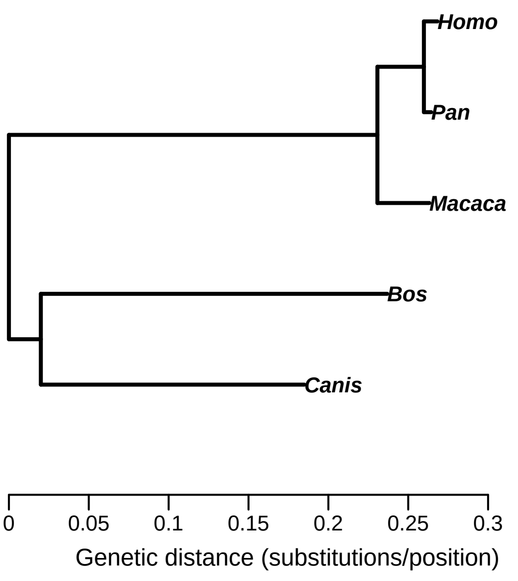

ENSG00000019505 intron 3

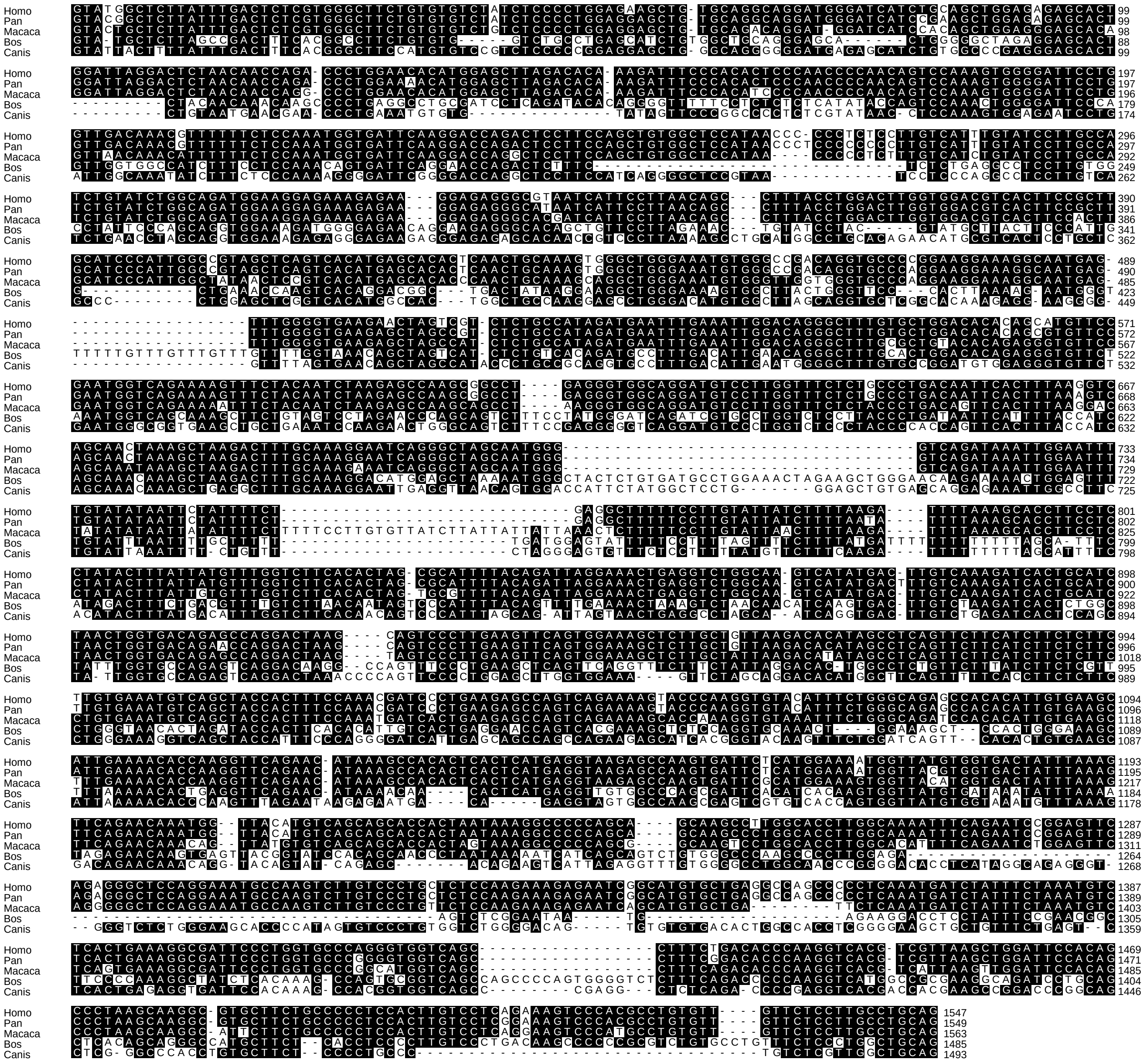

ENSG00000138434 intron 13

Description: Sperm-specific antigen 2 (SSFA2)
Intron number: 13
Human chromosome: 02
Intron start (bp): 182491855
Human intron length : 413
Intron alignment length: 430
Flanking exons length (upstream/downstream): 128/151
SNP density: 0.007264
K tree score: 0.0847
Scaling factor: 0.7144
Human-chimpanzee distance: 0.012206
Total primate branch length: 0.0782

ENSG00000138434 exon 13

```
Homo    AATACTTTTCAGGAGCTCCAGGTAATGAGGCGGAGCCTGAATTTGTTTAGAACACAAATGATGGATTTAGAATTGGCAAT 80
Pan     AATACTTTTCAGGAGCTCCAGGTAATGAGGCGGAGCCTGAATTTGTTTAGAACACAAATGATGGATTTAGAATTGGCAAT 80
Macaca  AATACTTTTCAGGAGCTCCAGGTAATGAGGCGGAGCCTGAATTTGTTTAGAACACAAATGATGGATTTAGAATTGGCAAT 80
Bos     AATACTTTCCAGGAGCTCCAAGTAATGAGGCGGAGTCTGAATTTGTTTAGAACGCAAATGATGGATTTAGAATTGGCAAT 80
Canis   AATACTTTTCAGGAGCTCCAGGTAATGAGGCGGAGCCTGAATTTATTTAGAACGCAAATGATGGATTTAGAGTTGGCAAT 80

Homo    GCTGCGTCAGCAAACCATGGTTTATCATCATATGACTGAGGAGGAGAG 128
Pan     GCTGCGTCAGCAAACCATGGTTTATCATCATATGACTGAGGAGGAGAG 128
Macaca  GCTGCGTCAGCAAACCATGGTTTATCATCATATGACTGAGGAGGAAAG 128
Bos     GCTGCGTCAGCAAACCATGGTTTATCATCATATGACTGAGGAGGAAAG 128
Canis   GCTGCGTCAGCAAACCATGGTTTATCATCATATGACCGAGGAGGAAAG 128
```

ENSG00000138434 exon 14

```
Homo    GTTTGAAGTTGATCAGCTCCAGGGTTTGAGAAATTCAGTCCGAATGGAACTTCAGGACCTGGAACTGCAGCTGGAGGAGC 80
Pan     GTTTGAAGTTGATCAGCTCCAGGGTTTGAGAAATTCAGTCCGAATGGAACTTCAGGACCTGGAACTGCAGCTGGAAGAGC 80
Macaca  GTTTGAAGTTGATCAGCTCCAGAGTTTGAGAAATTCAGTCCGAATGGAACTTCAGGACCTGGAACTGCAGCTAGAGGAGC 80
Bos     GTATGAAGTTGATCAGCTGCAGAGTTTGAGAAATTCAGTCCGAATGGAACTCCAGGACCTGGAGCTGCAGCTGGAGGAGC 80
Canis   GTATGAAGTGGATCAGCTTCAGAGTTTGAGAAATTCTGTGCGAATGGAACTTCAAGACCTGGAACTGCAGCTGGAGGAGC 80

Homo    GCCTGCTGGGCCTGGAGGAGCAGCTTCGTGCTGTGCGCATGCCTTCACCCTTCCGCTCCTCCGCACTCATG 151
Pan     GCCTGCTGGGCCTGGAGGAGCAGCTTCGTGCTGTGCGCATGCCTTCACCCTTCCGCTCCTCCGCACTCATG 151
Macaca  GCCTCCTGGGCCTGGAGGAGCAGCTTCGTGCTGTGCGCATGCCTTCACCCTTCCGCTCTTCTGCACTCATG 151
Bos     GCCTGCTGGGGCTGGAGGAGCAGCTCCGCCCTGTGCACTTGCCGTCAGCCTTCCGCCCCTCGGCACTCATG 151
Canis   GCCTGCTGGCCCTGGAGGAGCAGTTCCGTGTCGTGCGCATGCCTTCTCCCTTCCGTTCCTCAGCTCTTATG 151
```

ENSG00000138434 intron 13

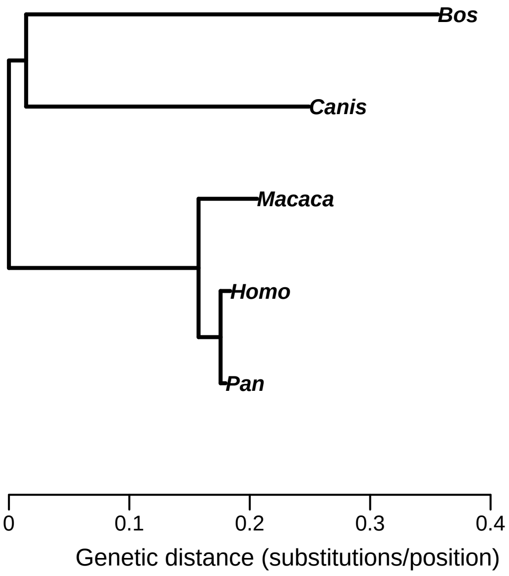

ENSG00000138434 intron 13

```
Homo    GTAAAAGTTCATTTTTGTCTTAGCACACCTCAAAAAAGCTTTTCATTC-----AAAGTCTTCTCAACTTATAT-TTTTTAAAATTAGGTTTTGATTCTACTG 94
Pan     GTAAAAGTTCATTTTTGTCTTAGCACACCTCAAAAAAGCTTTTCATTC-----AAAGTCTTCTCAACTTATAT-TTTTTAAAATTAGGTTTTGATTCCACTG 94
Macaca  GTAAAAGTTCATTTTTGTCTTAGCACTCCTGAAAAAAACTTTTCATTC-----AAAGTCTTTTCAACTTATAT-TTTTAAAAAATTAGGTTTTGATTTTACTG 94
Bos     GTAACAGTTCATATGTCTTAACGTTGACTTAAAATATTTTTCATTCTAAAAAGCATTTTAACTTTTTTTTT-TCTTGGAAGCTGACTTTTATTTACACTG 99
Canis   GTAAAAGTTCATTTTTGTCTTAATGTAACTCAAAATATTCTTCATGCTAAAAAAAGCATTGCCAACTTTTTTATTTTTAAAGCTTCATTTTATGTCTGCTG 100

Homo    CTGTGTACCTTGTATCTGTTTTGTGAACCAGTGTAGATAAGATTAGTCATAGATAGTGTTTTACTAAGTAAACTTGTATGCCTTCAATATCCTAAGG--- 191
Pan     CTGTGTACCTTGTATCTGTTTTGTGAACCAGTGTAGATAAGATTAGTCATAGATAGTGTTTTACTAAGTAAACTTGTATGCCTTCAGTATCCTAAGG--- 191
Macaca  CTATGTACCTTATATCCGTTTTGTGAACCAGTGTAGATAAAA-TAGTCATAGATAGTGTTTTACTAAGTAAACTTGTGTGCCTTCAGTATCCTAAGG--- 190
Bos     CTGTGTACCCTTTATCTAGTCTGTGAACCAGTGTAAATAAAATTGG----GCACAATGTTTTGCTAAGTAAACTTCTCTATCTTCGCTGAACTGAGGAAG 195
Canis   GTGTGTAC----TGTTATTTTGTGAACCACTGTAAATTAGG--------GCCTCGTGTTTTACTAATTAAGC-----TGCTTTCAATGTATTAAGGAAG 183

Homo    ---GTAGTGGTAACCTGCTTTCCCGTGTTTGAGATATTTGTGA--GAGGATAGGGGAAGAAAGACTA-ATGTCCTTGTGGATACACCTATTGTAGTTGAT 285
Pan     ---GTAGTGGTAACCTGCTTTCCCGTGTTTGAGATATTTGTGA--GAGGATAGAGGAAGAAAGACCA-ATGTCCTTGTGGATACACCTATTGTAGTTGAT 285
Macaca  ---GTGGTTGTAACCAGTTTTCCAGTGTTTGAGATATTTGTGA--GAGGATGGGGGAAGAAAGACCA-ATATCCTTGTGGATATACCTATTGTAGTTGAT 284
Bos     ACAGTGGTTGGAATCTGTTTCTCATTGTCTGGAATATTTGTGATGGAAGCTAGAGGAAGAAAAAGCA---GTTATAATGGTTATACTCATTGCAGACGCT 292
Canis   CCAGTGGTTGTAATCTGTTTTTTAGTTCGGAATATTTGGGAGA--GAAGGTAGAGGAAGAAAGACTGTTTGTTCTACTGGTCACACCTATGGTTTTCGCT 281

Homo    ATTGTACTAAT--TTCTAGAACCTGAGATTTCCATGCCTTCTGTTAGCATATAGTAGTTGTTGAAAGATTTGTGATTAGGAATCTCCAAACTGCATTTTT 383
Pan     ATTGTACTAAT--TTCTAGAACCTGAGATTTCCATGCCTTCTGTTAGCATATAGTAGTTGTTGAAAGATTTGTGATTAGGAATCTCCAAACTGCATTTTT 383
Macaca  ATTGTACTAATTATTATAGAACCTGAGATTTCTGTACCTTCTGTTAGCATATAGTAGTTGTTGAAAGATATGTGATTAGGAATCTCCAAACTGCATTTTT 384
Bos     GTTGTGCTAAT--CTATAGAACTTGGAGTTTCAGTAACTTCCGAAAGCT------------TGTGAGAGTTGTGACACTGAGTTTCC------------- 365
Canis   GTTGTGCTAAT--TTTTAGAACTTGCGCTCTCAGTGCCTTCCACTGGCTTG----------TGAGAGAGTGATGAATATGAATCTCCAAACTGCTTTTTT 369

Homo    CCAATTTTGTGCCCTTCACCATACCAATAG 413
Pan     CAAATTTTGTGCCCTTCACCATACCAATAG 413
Macaca  CAAACTTTGTGCCCTTCACCATACCAATAG 414
Bos     ---------------------TACTAATAG 374
Canis   C-AACCTTGCGCCTGTCATCATACCAATAG 398
```

ENSG00000133055 intron 2

Description: Myosin-binding protein H (MYBPH)
Intron number: 2
Human chromosome: 01
Intron start (bp): 201410349
Human intron length : 729
Intron alignment length: 772
Flanking exons length (upstream/downstream): 135/168
SNP density: 0.005487
K tree score: 0.0644
Scaling factor: 0.7499
Human-chimpanzee distance: 0.011160
Total primate branch length: 0.0781

ENSG00000133055 exon 2

```
Homo    ATGTCCCCAGTGCCCCACTGCTGCTGACCCTGGATGATGTGAGCAGCAGCTCTGTGACTGTGAGCTGGGAGCCCCCAGAG 80
Pan     ATGTCCCCAGTGCCCCACTGCTGCTGACCCTGGAGGATGTGAGCAGCAGCTCTGTGACTGTGAGCTGGGAGCCCCCAGAG 80
Macaca  ATGTCCCCAGTGCCCCACTGCTGCTGACTCTGGAGGATGTGAGCAACAGCTCTGTGACTGTGAGCTGGGAGCCCCCAGAG 80
Bos     ATGTCCCCAGTGCCCCGCTGCTGCTGGCCGTGGAGGATGTGAGTGACAGCTCGGTGACTGTGAGCTGGGAGCCTCCAGAG 80
Canis   ACGTGCCCAGTGCCCCGCTGCTGCTGGCCGTGGAGGATGTGAGTGACAGCTCGGTGACCGTGAGCTGGGAGCCTCCGGAG 80

Homo    AGGCTGGGGAGGCTGGGCCTCCAGGGCTATGTGCTGGAGCTCTGCAGAGAGGGAG 135
Pan     AGGCTGGGGAGGCTGGGCCTCCAGGGCTATGTGCTGGAGCTCTGCAGAGAGGGAG 135
Macaca  AGGCTGGGGAGGCTGGGCCTCCAGGGCTATGTGCTGGAGCTCTGCAGAGAGGGAG 135
Bos     AGGCTGGGGAGGCTGGGGCTTCAGGGCTATGTGCTGGAACTCCGTCGAGAGGGAG 135
Canis   AGCCTGGGGAGGCTGGGACTCCAGGGCTACGTGCTGGAGCTCTGCCGAGAGGGAG 135
```

ENSG00000133055 exon 3

```
Homo    CCTCGGAGTGGGTGCCTGTGAGTGCCCGGCCCATGATGGTGACCCAGCAGACTGTGCGGAACCTGGCTCTGGGAGACAAG 80
Pan     GCTCGGAGTGGGTGCCTGCGAGTGCCCGGCCCATGATGGTGACCCAGCAGACTGTGCGGAACCTGGCTCTGGGAGACAAG 80
Macaca  CCTCGGAGTGGGTGCCTGTGAGTGCCCGGCCCATGATGGTGACCCAGCAGACCGTGCGGAACCTGGCTCTGGGAGACAAG 80
Bos     CCTTGGACTGGGTGCCTGTGAATGCCCGGCCCATGATGGTGACCCAGCAGACCTTGCGGAACCTGGCTGTAGGTGACAAG 80
Canis   CCTCGGAGTGGGTGCCTGTGAACGCCCGGCCCGTGATGGTGACTCAGCAGACCGTGCGGAACCTGGCGCTGGGAGACAAG 80

Homo    TTCCTCCTGCGCGTGTCTGCAGTGAGTTCTGCAGGGGCTGGCCCGCCGGCCATGCTGGACCAGCCCATCCACATCCGAGA 160
Pan     TTCCTCCTGCGTGTGTCTGCAGTGAGTTCTGCAGGGGCTGGCCCGCCGGCCATGCTGGACCAGCCCATCCACATCCGAGA 160
Macaca  CTCCTCCTGCGGGTGTCTGCAGTGAGTTCCGCAGGGGCTGGCCCGCCGGCCATGCTGGACCAGCCCGTCCACATCCGAGA 160
Bos     TTCTTCGTGCGCGTGGCTGCAGTGAGCTCTGCAGGGGCTGGCCCACCGGCGGTGCTGGAGCGGCTCATCCACATCCAGGA 160
Canis   TTCTTCCTGCGTGTGGCTGCAGTGAGCTCTGCGGGGGCCGGCCCACCAGCGGTGCTCAAACAGCCCGTCCACATCCAGCA 160
```

ENSG00000133055 intron 2

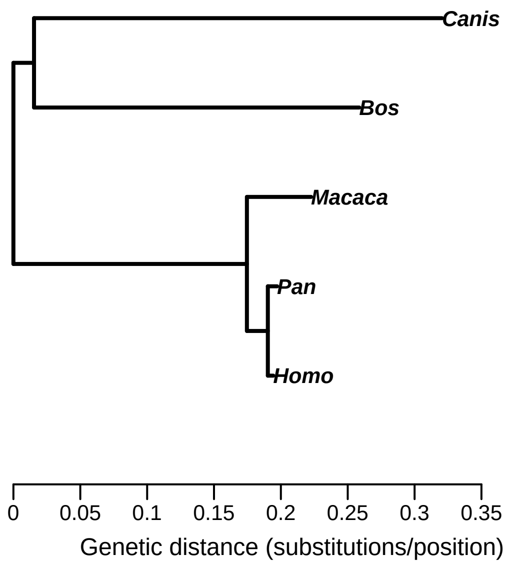

ENSG00000133055 intron 2

```
Homo    GTGAGTGCTGGGCTAAGTCTTGCATACACCCAGGGGAGGTTGTCATGGAGGGCTGCAGGGACAAGTGCACAGGCCCTGTGGTTGGTCAGTCTGGGAGAGA 100
Pan     GTGAGTGCTGGGCTAAGTCTTGCATACACCCAGGGGAAGTTGTCATGGAGGGCTGCAGGGACAAGTGCACAGGCCCTGTGGTTGGTCAGTCTGGGAGAGA 100
Macaca  GTGAGTTCTGGGCTAAGTCTTGCATACACCCAGGGGAGGTTGTCATGGAGAGTTGCAGGGACAAGTGCGTAGGCCATGTTGTTGGTCAGTCTGGGAGAGA 100
Bos     GTGAGCTCCGGGTTAAGCCCT-CATATACGGAGGGGAGGTGGCCTGGGGGGCAGTAGGGACAAGCCCCCAGACTCTGCAGTTGAGTCAGAACAGAGGGA 99
Canis   GTGAGCTCTGGGCTAAGCCCTGCGGACACC--GGGGAGGTGGGCTTG---------------------------------GCTGGGCCAGCCTGGGGGCA 65

Homo    TACAGAAGGAGGGGATGCGAGCAGTAGGCTCCTCAACCCACCCACCTGCCCTTTCTTGCTCCACCAGCAGGAGAGCAAG--AAAGGGTAGGTGGGTGGGT 198
Pan     TACAGAAGGAGGGGATGCGAGCAGTGGGCTCCTCAACCCACCCACATGCCCTTTCTTGCTCCACCAGCAGGAGAGCAAG--AAAGGGTAGGTGGGTGGGT 198
Macaca  CACAGAAGGGGGTGATGCAAGCGGTGGGCTCCTCAACCCACT----TATCCTTTCTTGCTCCACCAGCAGGAGAGTAAG--AAAGGGTAGGTGGGTGGGT 194
Bos     GACGCAGAGGGGGAGTGGAGGCAGTCTGCT--TAAGCCTCATGGACAGCGTCTTGCTCACCTACACATCTCTGAAAGCA--GGAGGGCATCCAAGGGGCC 195
Canis   GATACAGAGGAAGGAAGTGCAC-GTGGGCCCTCCAGCCACTCACCTGCCACCTGTTGCTCGCCCCATGTGCCGCTGAGGCAGGGGGTGTCCAAATGACC 164

Homo    TGAGGGGCTC-----CCTTGTCCT-CAGCTCCAAGGAGCTTC----TGAGTAAGGGAAGGGTAA-----GGGGACACTGGGTTCTCCTTTGGTGAGAAGG 283
Pan     TGAGGGGCTC-----CCTTGTCCT-CAGCTCCAAGGAGCTTC----TGAGTAAGGGAAGGGTAA-----GGGGACACTGGGTTCTCCTTTGGTAAGAAGG 283
Macaca  TGAGGGGCTC-----CCTTGTCCT-CAGTTCCAAGGAGTCTC----TGAGTAAGAGAAGGGTAA-----GGGGACTCTGGGTTCTCCTTTGGCGAGAAGG 279
Bos     TCAGCAGGTG-----CCTTGTCCTCCAGCTTCAGGAAGCCTC----TGAGTAACAAGGGTAAAATGGTCGGTGACACTGCGGTTCTCCTCTGTAAGAA-T 285
Canis   TTGGGAGATCCCCCACCCGTCCTCCAGCTCCGGGGAGCCTCTGGGTGAGTAACAGAGAGAGAAGGGTTGGGGCCAGTGGGTGCTGCTCTCATGAGAA-- 262

Homo    GAAGGAAGTGCTAAGTCTCAGAGGACCCCGAACTGTAGGAAGTGGAAAGGGAGGAGCTGGAGAGGGGAATGTTAGCGTAAGAGACAGTGAGGAGAA--AG 381
Pan     GAAGGAAGTGCTAAGTCTCAGAGGACCCCGAACTGTAGGAAGTGGAGAGGGAGGAGCTGGAGAGGGGAATGTTAGCGTAAGAGACAGTGAGGAGAA--AG 381
Macaca  GAAGGAAGTGCTAAGTCTCAGAGGACCCTGAAGTGTAGGAAGTGGAGAGGGAGGAGCAGGAGAGAGGAATGTTAGCATAAGAGACAGTGAGGAGAA--AG 377
Bos     TAAGGAGGTCCTGAGTCTCAGAGGACCCTGCTTGTAAAGAAGCTGAG-----------GGAGAGGGCTAAGTTAGAACAAGAGACAGTGAGGAGAAGGAG 374
Canis   -GTGGAGACGCTGGGTCGCAGAGGAGCCTGCTTGGGGA--------------------GGGCAGGCAGGAGGTAGAGGCCAGGGAAGCCAGGAGTTGAGG 341

Homo    CGGGAATGGGGTGGGGGTGTGGGGGCCA----CATCCCTGCTGCCA--CTGTCCCCCTCCATGGC--CCAAGTCCAGCCTG------GCCTCTGCCTCTG 467
Pan     CGGGAATGGGGTGGGGGTGTGGGGGCCA----CATCCCTGCTGCCA--CTGTCCCCCTCCATGGC--CCAAGTCCAGCCTG------GCCTCTGCCTCTG 467
Macaca  CAGGAATGGGGTGGGAGTATGGGGGCTA----CATCCCTGATGCCA--CTGTCCCCCTCCATGGC--CCAAGTCCAGCCTGGCCTCTGCCTCTGCCTCTG 469
Bos     CGGGGCTGTGGCCAAACTCCTGATGCCGCCATCTTGTCCGCTGCCGTCTTGTCCACCTCCGCGGCAGGCAAGTTCAGTCTG------GCTCCTGCCTTTG 468
Canis   GGAGACAGTGGGGAGAATCAGGGGACCAGGGCCGCGGCCCACGTC---CTGTTCACCCCCGTGGTGGCCAAGGTCAGCCTG------GCTTCTGCCCTTG 432

Homo    CCCCTACCTTGCTGTGTGACTTTGCAAATGTGTCCAACCCTCTCTGGGCTTCAACAACCTCATCTCTAGCCACAATTTCAGGGCTCTAACATCAGGTGTC 567
Pan     CCCCTACCTTGCTGTGTGACTTTGCAAATGTGTCCAACCCTCTCTGGGCTTCAACAACCTCATCTCTGGCCACAGTTTCAGGGCTCTAACATCAGGTGTC 567
Macaca  CCCCTACCTTGCTGTGTGACTTTGCAATTGTGTCCAACCCTCTCTGGGCTTCAACAACCTCATCTCTGGCCATGATTTCAGGGCTCTAACATCAGGTGTC 569
Bos     CCTCCGCCTTGCTGTGTGACTTGGTAAACGTGTCCAAGCCTCTCTGGGCTTCAGGAAACCCGTTTCTGGCCACCACTCAAAGGTTCTGACACAAGGTGTC 568
Canis   CCC--ACCCTGCGGTA-GACTTGGCACATGTGTCCTGCCCTCTCCAGGCCGTAGCCACACCTCCCCGGCCACCTTCCCTGGGTTTAACACCAGGTGTC 529

Homo    TTTGATGCCATTTGACA----AGAAGTCAGGGAAGGCAAACAAGGGGAAAATGAGGCCCA----GAGAGGGAGGCTGATTTCCCCAGATCACCAGCAGGA 659
Pan     TTTGATGCCATTTGACA----TGAAGTCAGGGAAGGCAAACAAGGGGAAAATGAGGCCCA----GAGAGGGAGGCTGATTTCCCCAGATCACCAGCAGGA 659
Macaca  TTTTATGCCATTTGACA----AGAAGTCAGGGAAGGTAGAGAAGGGAAAAATGAGGCCCA----GAGAGGGAGGCCGATTTCCCCAGATCACCAGCAGGC 661
Bos     TTTGATGCCATTTGATACATGAGAAGTCAAGGAGGGACAGCAGAAGGAAAATAAGGCCCG----GAAAGGGAGGCCAGCTTCCCCAGACCGTCAGTGAGA 664
Canis   TTTGGTGCCATCTGACA----AGAAGTCAGAGAAGGAGAGCAAGAGAGAAATAAGGCCCAGGATGGGAGGAGGTCAGCTTCCCCAGGTCACCAGGGAGA 625

Homo    AAAGAAGCCCCA-TGGAGAGGGGCGCCTGTGGCCACATTGCAAAGACCTCACACTCTCTCCA-TCCCCTCAG 729
Pan     AAAGAAGCCCCA-TGGAGAGGGGCGCCTGTGGCCACATTGCAAAGACCTCACACTCTCTCCA-TCCCCTCAG 729
Macaca  AAAGAAGCCCCA-TGGAGAGGGGCGCCTGTGGCCACACTGCCAAGACCTCACACTCTCTCCA-TCCCCTCAG 731
Bos     AGGGAAGCCCCACTGAGAAAGGGTGCCGGTGGCCATGGGGCCAGGGCCTTGTACTGTATTCATTCCTCTCAG 736
Canis   AGGAAAACCCCG-GTGAGTAGGGCGCCCATGGTCACGAAGCCGGGGCCTTCGTGCCGTCGC-TTTCCCCAG 695
```

ENSG00000183763 intron 8

Description: TRAF-interacting protein (TRAIP)
Intron number: 8
Human chromosome: 03
Intron start (bp): 49852811
Human intron length : 611
Intron alignment length: 818
Flanking exons length (upstream/downstream): 88/90
SNP density: 0.006547
K tree score: 0.0673
Scaling factor: 1.0103
Human-chimpanzee distance: 0.011626
Total primate branch length: 0.0781

ENSG00000183763 exon 8

```
Homo    AGAGTACGAGAATCTAAAAGAGGCACGGAAGGCCTCAGGGGAGGTGGCTGACAAGCTGAGGAAGGATTTGTTTTCCTCCA  80
Pan     AGAGTACGAGAATCTAAAAGAGGCACGGAAGGCCTCAGGGGAGGTGGCTGACAAGCTGAGGAAGGATTTGTTTTCCTCCA  80
Macaca  AGAGTACGAGAATCTAAAAGAGGCACGGAAGGCCTCAGGGGAGCTGGCTGAGAAGCTGAGGAAGGATTTGTTTTCCTCCA  80
Bos     AGAGTACGAGAATCTAAAAGAGGCACGGAAGGCCTCAGGGGAGCTAGCTGACAAACTGAAGAAGGACTTGCTTTCTTCCA  80
Canis   AGAGTATGAGAACCTTAAAGAGGCACGGAAGGCCTCAGGGGAGCTGGCTGACAGACTGAAGAAGGACTTGTTTTCTTCTA  80

Homo    GAAGCAAG  88
Pan     GAAGCAAG  88
Macaca  GAAGCAAG  88
Bos     GAAGCAAG  88
Canis   AAAACAAG  88
```

ENSG00000183763 exon 9

```
Homo    TTGCAGACAGTCTACTCTGAATTGGATCAGGCCAAGTTAGAACTGAAGTCAGCCCAGAAGGACTTACAGAGTGCTGACAA  80
Pan     ---CAGACAGTCTACTCTGAATTGGATCAGGCCAAGTTAGAACTGAAGTCAGCCCAGAAGGACTTACAGAGTGCTGACAA  77
Macaca  TTGCAGACAGTCTACTCTGAATTGGATCAGGCCAAGTTAGAACTGAAGTCAGCCCAGAAGGACTTACAGAGTGCTGACAA  80
Bos     TTGCAGACAGTCTGCTCTGAACTGGACCAGGCCAAGTTGGAGCTGAAGTCGTCTCAGAAGGACTTACAGAGTGCTGACAA  80
Canis   TTGCAGACAGTCTACTCTGAACTAGATCAGACCAAGTTGGAGCTGAAGTCGGCTCAGAAGGATTTACAGAGTGCTGACAA  80

Homo    GGAAATCATG  90
Pan     GGAAATCATG  87
Macaca  GGAAATCATG  90
Bos     GGAAATTGCG  90
Canis   AGAAATCATG  90
```

ENSG00000183763 intron 8

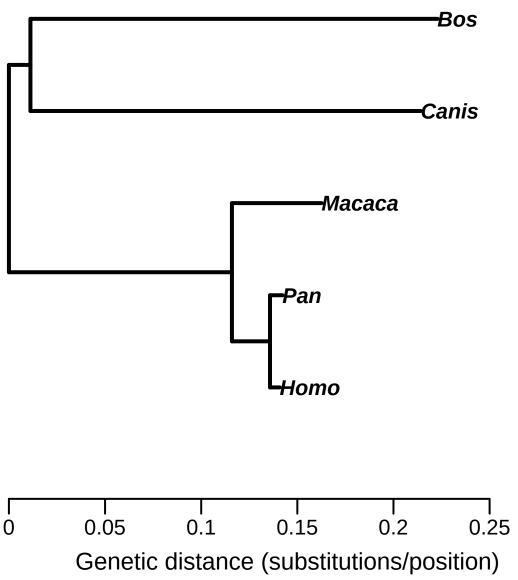

ENSG00000183763 intron 8

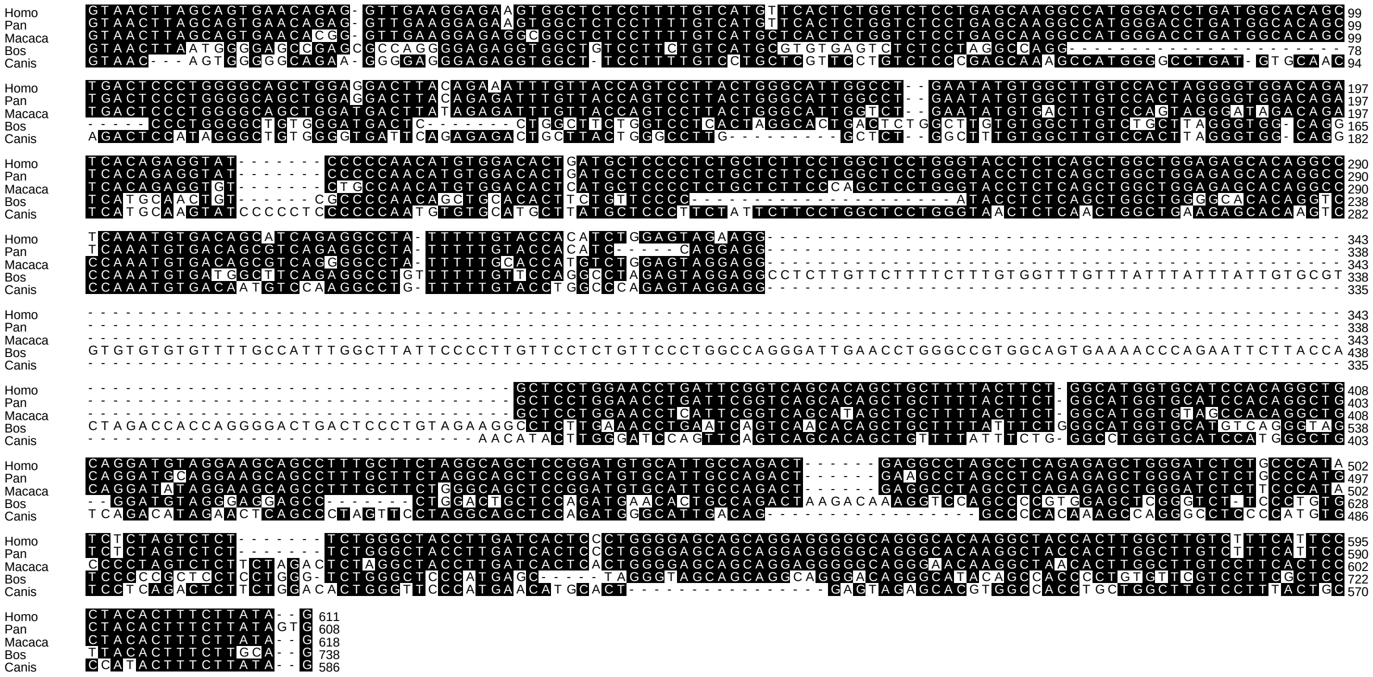

ENSG00000143921 intron 9

Description: ATP-binding cassette sub-family G member 8 (ABCG8)
Intron number: 9
Human chromosome: 02
Intron start (bp): 43954630
Human intron length : 420
Intron alignment length: 476
Flanking exons length (upstream/downstream): 200/77
SNP density: 0.014286
K tree score: 0.0898
Scaling factor: 1.1024
Human-chimpanzee distance: 0.025154
Total primate branch length: 0.0781

ENSG00000143921 exon 9

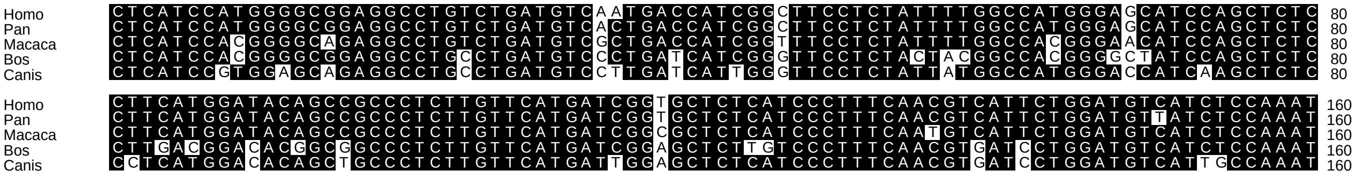

ENSG00000143921 exon 10

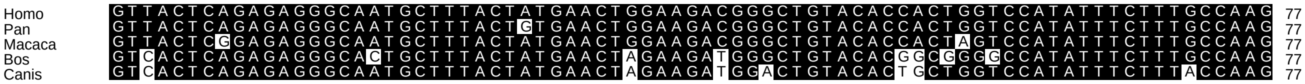

ENSG00000143921 intron 9

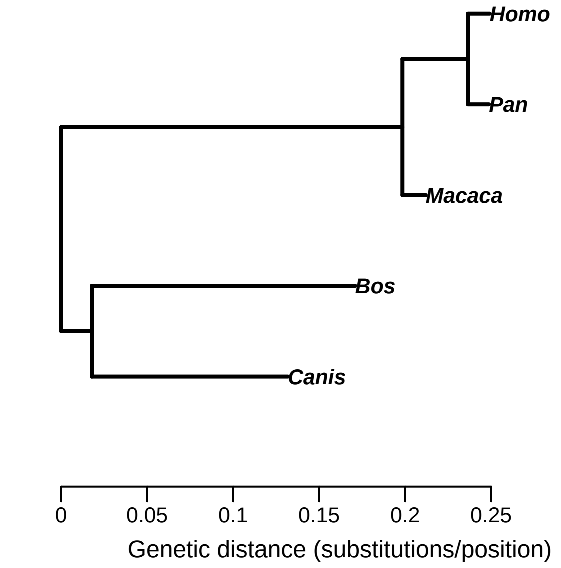

ENSG00000143921 intron 9

```
Homo    GTGAGTGTGGCCCACTGGCATGGGCAGGCA---------------------------------------GGACCTCAGCCACCTCCAAGCTGTGTTCCTC 61
Pan     GTGAGTGTGGCCCACTGGCATGGCCAGGCA---------------------------------------GGACCTCAGCCACCTCCAAGCTGTGTTCCTC 61
Macaca  GTGAGTGTGGC-------CGTGGCCACGCA---------------------------------------GGACCTCAGCCACCTCTGAGCTGTGTTCCTC 54
Bos     GTGAGTGTGGCCTGCTCTCTGTGACCCACA-----------TGCAGGGCCGTG----TCAGCCACGCTCTGAACTGGATCAGGGCTGGCCTGAGTTCCGT 85
Canis   GTGAGTATGGCTTGCTCTCTGTAACTCACACTTGGAACAGCCAGAGGACCATGGGATACATCACCTTTCTGAGCTGATCCAAAGCTGACCTCTGTTCCTC 100

Homo    TGAGCTCCTGGGGAG--CGGGTTTG-------ATTTCATTGTGATTGTGATATATAAGACAATAA-TGTTTTTAAAGTTTGCATGTTAATATTAGCA--T 149
Pan     TGAGCTCCTGGGGAG--CGGGTTTG-------ATTTTATTGTGATTGTGATATATAAGACAATAA-TGTTTTTAAAGTTTGCATGTTAATATTAGCA--T 149
Macaca  TGAGCTC----------TGGGTTTG-------ATTTCATCGTGATTATGATATACAAGACAATAA-TGTTTTTAAAGTTTGCATGTTAATGTTAGTA--T 134
Bos     ATAGCTCCTGGAGGAGACAGGTCTGCTGAACAATTTCACCATGAGTGCTAAATAAAAGCTGAGGGTTGTTTTTAAACTTCATATATTAATATCAGTATTT 185
Canis   CAAACTTCTGGGGAGAATAGGTTTGCTGAATGGTTTCACCATGATTGCAAAATAAAAGGTGATGATTGTTTTTAAAGTTCGTACATTAATATTAGTA--T 198

Homo    ACAAATGAAAGTAAATTACC---GAGTCTTAGCTGTTCCTATCCTAGCAGGATTTATATTTGACAGCAGAACACGAGCTACAGACTTGCAAAACCTGAAG 246
Pan     ACAAATGAAAGTAAATTACC---GAGTCTTAGCTGTTCCTATCCTAGCAGGATTTAAATTTGACAGCAGAACACGAGCTACAGACTTGGAAAACCTGAGG 246
Macaca  GCAAATGAAAGCAAATTACC---GAGTCTTAGCTGTTCCTATCCTAGCAGGATTTATATTTGACAGCAGAACACGAGCTACAGACTTGCAAAACCTGAAG 231
Bos     GCAAATGAGAACAAATGACCTAGGAGTCTGAGGCTTTTCTCTCCTAGCAGGAGTTATA-TTGACAGCAATGCACAAGTCACAGAATTATAA--------- 275
Canis   GCAAATGAAAGACGATTACCCAGGAGTCTTAGGCATTCCTATTATAACAGGAATTATACTTGACAGCAATGCACAAGTCAGAGAATTACAA--------- 289

Homo    AGCCTCATCAAACATCTAAATTAGGATGGCTTTACTGTGCCTA--TTTAAAAAAAAAAAATAGAGACTTGGGCAATATGATAACTACTTTGAATTGTATT 344
Pan     AGCCTCATCAAACATCTAAATTAGGATGGCTTTACTGTGCCTA--TTT-AAAAAAAAAAATAAGAGACTTGGGCAATATGATAACTACTTTGAATTGTATT 343
Macaca  AGCCTCATCAGACATCTAAATTAGGATGGCTTTACTGTGCCTGTTTTTTAAAAAAAAACCTAAGAGACTTGGGCAATATGATAACTGCTTTGAATTGTATT 331
Bos     -----------ACATCTAAATTAGGA---TCAAAATTGCCTTTTTTTT--TAATA---TATGACATTGAGGCAAT---AAAACTAGTTTTAATTGTACA 353
Canis   -----------ACGTCTATATTAGGATGGTTTAACTCTGCCTGCTTTTTAAAAATA---GAAGACATTTAGGCAAT---ATGACTATTTATAATTGTACT 372

Homo    AAGAGAGTCTCCAAAACAGAAGCACTGTAGATTTATTCTACTTCTTCATTCTCTTTTCCTTTCCCTTACTTTTTAG 420
Pan     AAGAGAGTCTCCAAAACAGAAACACTGTAGATTTATTCTACTTTTTTATTCTCTTTTCCTTTCCCTTATTTTTTAG 419
Macaca  GAGAGAATCTCCAAAACAGAAACACTGTAGATTTATTCTACTTTTTCATTCTCTTTTCCTTTCCCTTATTTTTTAG 407
Bos     AAGAGAGACTC--AAACAGCAACACTGTAATTTCAT-----TTTTTCATTCTCCTTTTCTCCCC----TTT-TTAG 417
Canis   A----AGACTC--AAAAGAAAACACTAAAAATTAATGT--CATTTTCATTCTCCTTTTCTCCCT----TTTGTTAG 436
```

ENSG00000164253 intron 10

Description: WD repeat protein 41 (WDR41)
Intron number: 10
Human chromosome: 05
Intron start (bp): 76769006
Human intron length : 815
Intron alignment length: 1178
Flanking exons length (upstream/downstream): 122/89
SNP density: 0.002454
K tree score: 0.0966
Scaling factor: 1.01
Human-chimpanzee distance: 0.004926
Total primate branch length: 0.078

ENSG00000164253 exon 10

```
Homo    AATGTATTTGCTGCAGTTGGAAGGGGTTTATACGTGTATAGCCTTCAAATGAAGCGTGTGATTGCCTGCCAGAAAACTGC 80
Pan     AATGTATTTGCTGCAGTTGGAAGGGGTTTATACGTGTATAGCCTTCAAATGAAGCGTGTGATTGCCTGCCAGAAAACTGC 80
Macaca  AATGTATTTGCTGCAGTTGGAAGGGGTTTATACGTGTATAACCTTCACATGAAGCGTGTGATTGCCTGCCAGAAAACTGC 80
Bos     AATGTATTTGTTGCAGTTGGAAGGGGTTTATATGTGTATAACCTTCCAATGAAGCGTGTGATTGCCTGCCAGAAAGCTGC 80
Canis   AATGTATTTGCCGCAGTTGGAAGGGGTTTATATGTGTATAACCTTCAAATGAAGCGTGTGATTGCTTGCCAGAAAACTGC 80

Homo    ACATGACTCCAATGTCCTGCACGTTGCCAGACTTCCAAACAG 122
Pan     ACATGACTCCAATGTCCTGCACATTGCCAGACTTCCAAACAG 122
Macaca  ACATGACTCCAGCGTCCTGCACATCGCCAAACTTCCGAACAG 122
Bos     GCATGACTCCAGTGTCCTGCACGTTGCCAAACTCCCAAACAG 122
Canis   ACATGACTCCAGTGTCCTGCACGTTGCCAAACTTCCAAACAG 122
```

ENSG00000164253 exon 11

```
Homo    GCAGTTAATCTCATGCTCAGAAGATGGCAGTGTACGCATTTGGGAGTTAAGAGAAAAACAGCAGCTTGCAGCTGAGCCTG 80
Pan     GCAGTTAATCTCATGCTCAGAAGATGGCAGTGTACGCATTTGGGAGTTAAGAGAAAAACAGCAGCTTGCAGCTGAGCCTG 80
Macaca  GCAGTTAATCTCATGCTCAGAAGATGGCAGTGTACGCATTTGGGAGTTAAGAGAAAAACAGCAGCTTGCAGCTGAGCCTG 80
Bos     GCAGGTTATCTCATGCTCAGAAGATGGCAGTGTACGCATTTGGGAGCTACGAGAAAAACAGCAGCTTTCAGTGGAGCCGG 80
Canis   GCAGTTAATCTCATGCTCAGAAGATGGCAGTGTACGCATTTGGGAGTTAAGAGAAAAACAGCAGCTTGCAGTTGAACCTG 80

Homo    TACCAACAG 89
Pan     TACCAACAG 89
Macaca  TCCCAACAG 89
Bos     CGCCAACGG 89
Canis   TACCAACAG 89
```

ENSG00000164253 intron 10

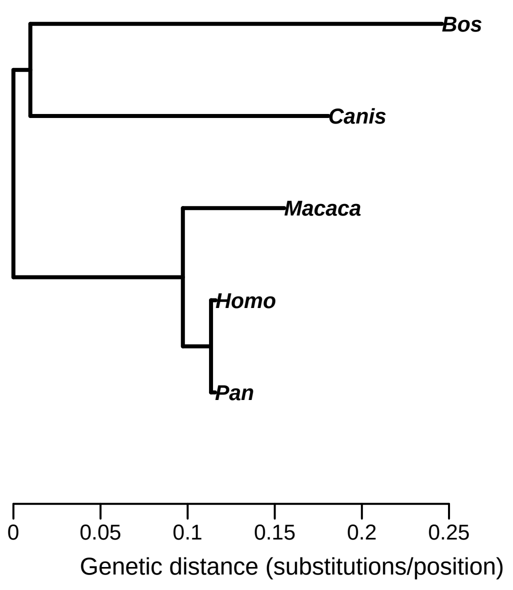

ENSG00000164253 intron 10

```
Homo    GTACAAGTTCCCATCTGTTCTGCAATAGATGAAAGCAAGTCACAGTTCCCAGGGCTACCAGTGTAATTTCTCAAGTGGCCAAGTTACCTGGCTTCATGGC 100
Pan     GTACAAGTTCCCATCTGTTCTGCAATAGATGAAAGCAAGTCACAGTTCCCAGGGCTACCAGTGTAATTTCTCAAGTGGCCAAGTTACCTGGCTTCATGGC 100
Macaca  GTACAAGTTCCCATCTGTTCTGCAGCAGATGAAAGCAAGTCACGGTTCCCAGGGCTTCCATTGTAATTTCTCAAGTGGCCAAGTTACCTGGCTTCATGGC 100
Bos     GTACGAGTCTCCATCCACTCCGTAACAGGTGGGAGAAACGCACAGTTCCCAGGGCCACCAGTG-AACTGCTCAGGGCGCAAAGTCGCCTGGCTCTGTTGC 99
Canis   GTACAAGTTCCCATCTGTTCTACAACAAATGAAAGATAATCCCCTTTCCCAGGGCCACCAGGGTAATTTCTCAGGTGGCAAAGTTATCTGGCTCCATGGC 100

Homo    TTGACCTGCTGTGTTCTTTTGGAAATGGAGAATGCCTGAGGGCTGTCTCTAACA---GAAGGGTATGTGG---CAGTATATTACAGAATTTACAAGAAGC 194
Pan     TTGACCTGCTGTGTTCTTTTGGAAATGGAGAATGCCTGAGGGCTGTCTCTAACA---GAAGGGTATGTGG---CAGTATATTACAGAATTTACAAGAAGC 194
Macaca  TTGACCTGCTGTGTTCTTTTGGAAATGGAGAATGCCTGAGGGCTGTCTCAGGCATTTAAATTAAATTTAAATTCTGTAAATTACAGAATTTACAAGAAGA 200
Bos     TTGACCAAC-AGGTTCTTTTGAAAATGGAGACTACCCTATGGCTGTCTCTAACA---GAAGAGTTTGTGG---CAATATATTACAAAATTTACAAACAGC 192
Canis   TTGACTCGTTAGGTTCTTTTGGGAATGGCGAATACCC-ATGGCTATCTCTAACA---AAAGAGTTTGTGG---CAATATATTAAAAAATTTATAAGAGGC 193

Homo    AAGGTGCAAATGAATTACATTGAATTTCATTGTTAAGAAGAGATGA-TTTAAAATTTGGTATTTA---ACAACATAA-ACGACTTTGGGGCCACTTACAT 289
Pan     AAGGTGCAAATGAATTACATTGAATTTCATTGTTAAGAAGAGATGA-TTTAAAATTTGGTATTTA---ACAACATAA-ACGACTTTGGGGCCACTTACAG 289
Macaca  AAGGTTCAAATGAATTACGTCGAATTTCATTG-TAAGAAGAGATGA-TTTAAAATTTGGTATTTC---ACAATGAAA-ATGACTTTGGGGCCACTTACAG 294
Bos     AGGGTTCAAACAAATTATTCTGAATTTCATTG-TAAGAAGAGAGAA-TTTAAAAGTTGGTATTTATTTTAATAGTTAGAAGACTTTGGGGC-----ACTA 285
Canis   AGAGTTCAAATGAATTACATTGAGTTTTATTA-CAGGAAGGGATAACTTTAAAATTTGGTATTTATTTAAAAAGACA-AAGACTTGTGGGC-----ACTA 286

Homo    TGTACCAATTTTTATAT--TTCTCATAAACCTACCTTCTT----T--TTGTTTATGGCCAGAATCAAATGGTTGCTGGTCTCCTTGTTGTGTGAGGCTAC 381
Pan     TGTACCAATTTTTATAT--TTCTCATAAACCTACCTTCTT----T--TTGTTTATGGCCAGAATCAAATGGTTGCTGGTCTCCTTGTTGTGTGAGGCTAC 381
Macaca  TGTACCAATTTTTATGT--TTCTCATAAACCTACCTTCTTTTTGT--TTGTTTATGTCCAGAATCAAATGGCTGCTAGTCTCCTTGTTATATGAGGCTAC 390
Bos     TGTTCTAATTTTTTCATATTTTCCACAAAGCCACCTTTTTGGTAGTGTTGCTTATGGCCAGAATTAAATGGTG----GTCTCCTTGTTGGCTGAGGCTAC 381
Canis   TGTGCTAATTTTTCTTCTGTTTTTCTCAACTCATTTTTTTGTTGTAGTTATTGATGGCCAGAAAGAAGTGATC----ATCTGCTTGTTGTATAAGGCTAC 382

Homo    TAAGTGAGTGGTTTCCTGTCAGGGTAGATTT-GCTGCTGAGCTTCTAGGGACGAA---CATATGGACAGGGACTATCATTATTTTCT------------- 464
Pan     TAAGTGAGTGGTTTCCTGTCAGGGTAGATTT-GCTGCTGAGCTTCTAGGGACGAA---CATATGGACAGGGACTATCATTATTTTCT------------- 464
Macaca  TAAGAGAGTGGTTTCCTCTCAGGGTAGATTT-GCTGCTGAGCTTCTAGGGACGAA---CATATGGACAGGGAATGTCGTTATCTTCT------------- 473
Bos     TAACTGAGGGCTTTCCTCTCAGGGCAGATGTGGCCATTGAGCTTCTAGGGATGAATTCTAT----GAAGGAAATGGTAGTATTTTCGGGGTTTCCCTGGT 477
Canis   TAAATGAGGGCTTTCCTGTCAGGATACATTTGGCTGCTGAGTTTCTAGGCATGAAATTTATATAGGAAGGGAATGCAATATTTTTTTGGATTAAAAAAAA 482

Homo    ------------------------------ATGCAGGATA------------------------------------------------------------ 474
Pan     ------------------------------ATGCAGGATA------------------------------------------------------------ 474
Macaca  ------------------------------ATGCAGGATA------------------------------------------------------------ 483
Bos     GGCTCAGCTGGTAAAGAATCTGCCAGCA--ATGCAGGAGA---CCCCGGTTCGATTCCTGGGTCAGGAAGTTCCCCTGGAGAAGGCACAGGCTACCCACT 572
Canis   ATTTTTTTTATTGGAGTTCAATTTGCCAACATATAGCATAATACCCAATGCTCATCCCATCAAGCATAATACCCAATGCTCATCCCATCAAATGCCCCCC 582

Homo    ---------------------------------------------------------------------------------------------------- 474
Pan     ---------------------------------------------------------------------------------------------------- 474
Macaca  ---------------------------------------------------------------------------------------------------- 483
Bos     CCAGTGTTCTTGGGCTTCCCTGGTGGCTCAGATGACAAAGAATCTGCCTGCAATGTGGGAGGCCTGGGTTGGATCCCTGGGTTGGGAAGATCCCCTTGAG 672
Canis   TCAGTGTCCATCACCCAGTCATCCCATCCCCCTG--------CCCACCTCCCCTTTCACTACCCCTTGTTCATTTCCCAGAGTTAGGAGTCTCTCATGTT 674

Homo    ---------------------------------------------------------------------------------------------------- 474
Pan     ---------------------------------------------------------------------------------------------------- 474
Macaca  ---------------------------------------------------------------------------------------------------- 483
Bos     GAGGGCATGGCAACCCACTCCAGTATTCAGGCCTGGAGAATCCCATGGACAGAAGAACCTGGTGGGCTACAGTCTGTGGGGTTGCAGAGAGCTGGACACA 772
Canis   CTGTCCCCCTCACTGATATTTCCCACTCATTTTCTCTCCTTTCC---------------------CCTTTAATCCCTTTCACTATTTTTTATATTCCCCA 753

Homo    ---------------------------TTTTTATAGT--GAAGCACCCAGAGAAGAGAGGTAGCAAGAGGAGGGAAGGGGGCTGCTTAGGACATTGTG-A 544
Pan     ---------------------------TTTTTATAGT--GAAGCACCCAGAGAAGAGAGGTAGCAAGAGGAGGGAAGGGGGCTGCTTAGGACATTGTG-A 544
Macaca  ---------------------------TTTTTGGAGTGAGAGGCACCCAGAGAAGAGA-GTAGCAAGAGGAGGGGAGGGGGCTGCTTAGGACATTGTG-A 554
Bos     ACTGAGCAGCTAAGCACAGCACAGCAGTATTTACTCTGCAGGATACTTTCAGAGTGA--GAGGGGAGAGAAAGGAATGGGGCTTCTCAGGATATTGTGAG 870
Canis   AATGAATGAGACCATATAATGTTTGTCCTTTTAGCACAAGAGCCACCTTGAGAAGACACACAGTGAGAGGAG----------------GGATATTGTGAA 837

Homo    AGTATAATTCACA--CTGTTTTCCCAAGGGCATTTTTTTAAATGGAGAGAATC--AATTTATAGCATTTGAGCTAATGTAAC-GAGTAACATGTGGCTTT 639
Pan     AGTGTAATTCACA--CTGTTTTCCCAAGTGCATTTTTTTAAATGGAGAGAATC--AATTTATAGCATTTGAGCTAATGTAAC-GAGTAACATGTGGCTTT 639
Macaca  AGTACAATTCACA----GTTTTCCCAAGTGCATTTTTTTAAATGAAGAGAATA--AATTTATAGCATTTGAGCTAATGTAAT-GTATAACATGTGGCTTT 647
Bos     AGTATAATTCATAACCTGTTTTCCTGCATGCATTTTTTAAAATGGAAGCGATACGTGTTTGAAACATTTGAGCTAGTCTCACACCATAATGCATGCCTTT 970
Canis   AGTGTAATTTATA--CTGTTTTCCTGAATGTGTTT-----AAGAAATGGGATA--GATTTATCACATTTGAGCCAGTGTAACAGTATAACAGGTGCCCTT 928

Homo    CCTAAAGGGTGCTTTTAGTTTGCGTTGTAACGTCATGCTAAATTGTGATTTTT--GTCAGTTCCTGCTAAGAGCTGAATAAAAGCAGTTGAGGGCTCCAC 737
Pan     CCTAAAGGGTGCTTTTAGTTTGCGTTGTAATGTCATGCTAAATTGTGATTTTT--GTCAGTTCCTGCTAAGAGCTGAATAAAAGCAGTTGAGGGCTCCAC 737
Macaca  CCTAAAAGGTGCTTTTAGTTTGTGTTGTAACGTCATGTTAAATTGTGATTTTT--GTCAGTTCCTGCTAAGAGCTGAATAAAAGCAGTTGAGGGCTCCAC 745
Bos     CC----------------TTTGTATTGTAAAGCCATGTTAAAGTGTGATTTTTTAATCAGCTCCCGCCAGGAGCTGGATGAAAGCAGATAAAGGCTGCGC 1054
Canis   CCCAAAGGACACTTTTACTTTGCATTGTGAAGCCATGTTA------------C--ATGAGCTCCTGCCCAGAGCTGAAGAGAAGTAGTTGAGGACTTCAC 1014

Homo    TGATTTCTTTCACTGCAAATTTAGCATTGGCCCTCAGTGGTGCGCTCTTTTTGTATTACTGATTTTCTGTTCTGACAG 815
Pan     TGATTTCTTTCACTGCAAATTTAGCATTGGCCCTCAGTGGTGCGCTCTTTTTGTATTACTGATTTTCTGTTCTGACAG 815
Macaca  TGATTTCTTTCACTGCAAATTTAGCATTGGTCCTCAGTGGTGCGCTCTCTTTGTGTTACTGATTTTCTGTTCTGACAG 823
Bos     TGATGGTTTTCCCTGCGCTCCAAGCACTTGCCCTCCATGGTGCTCTCTC------------------TCTCCTGACAG 1114
Canis   TGATTTCTTGCACTGCAAATTTAGCACTGGCCTTCCATGGTGCATTCTC----TATCACTGATTTTCTATTCTGACAG 1088
```

ENSG00000137558 intron 2

Description: protease inhibitor 15 preproprotein (PI15)
Intron number: 2
Human chromosome: 08
Intron start (bp): 75918890
Human intron length : 1028
Intron alignment length: 1240
Flanking exons length (upstream/downstream): 119/133
SNP density: 0.001946
K tree score: 0.0602
Scaling factor: 0.9243
Human-chimpanzee distance: 0.011804
Total primate branch length: 0.078

ENSG00000137558 exon 2

```
Homo    GTTTGGATGAAAATCTTGCAAAATCGGCAGAGGCTTGGGCGGCTACTTGCATTTGGGACCATGGACCTTCTTACTTACT 80
Pan     GTTTGGATGAAAATCTTGCGAAATCGGCAGAGGCTTGGGCGGCTACTTGCATTTGGGACCATGGACCTTCTTACTTACT 80
Macaca  GTTTGGATGAAAATCTTGCGAAATCGGCAGAGGCTTGGGCCGCTACTTGCATTTGGGACCATGGACCTTCTTACTTACT 80
Bos     GTTTGGATGAAAATCTTGCAAAGTCTGCAGAGGCTTGGGCAGCTACCTGCATTTGGGACCATGGACCTTCTTACTTATT 80
Canis   GTTTGGATGAAAATCTTGCAAAATCTGCAGAGGCTTGGGCAGCTACTTGCATTTGGGATCATGGACCTTCTTACTTACT 80

Homo    GAGATTTTTGGGCCAAAATCTATCTGTACGCACTGGAAG 119
Pan     GAGATTTTTGGGCCAAAATCTATCTGTACGCACTGGAAG 119
Macaca  GAGATTTTTGGGCCAAAATCTATCTGTACGCACTGGAAG 119
Bos     GAGATTTTTGGGTCAAAATCTATCTGTACGAACTGGAAG 119
Canis   GAGATTTTTGGGCCAAAATCTATCTGTACGAACTGGAAG 119
```

ENSG00000137558 exon 3

```
Homo    ATATCGCTCTATTCTCCAGTTGGTCAAGCCATGGTATGATGAAGTGAAAGATTATGCTTTTCCATATCCCCAGGATTGCA 80
Pan     ATATCGCTCTATTCTCCAGTTGGTCAAGCCATGGTATGATGAAGTGAAAGATTATGCTTTTCCATATCCCCAGGATTGCA 80
Macaca  ATATCGCTCTATTCTCCAGTTGGTCAAGCCATGGTATGATGAAGTGAAAGATTATGCTTTTCCATATCCCCAGGATTGCA 80
Bos     ATACCGCTCTATTCTCCAGTTGGTCAAGCCATGGTATGATGAAGTGAAAGACTACGCCTTTCCATATCCCCAGGATTGCA 80
Canis   ATATCGCTCTATTCTCCAGTTGGTCAAGCCATGGTATGATGAAGTGAAAGATTATGCTTTTCCATATCCTCAGGATTGCA 80

Homo    ACCCCAGATGTCCTATGAGATGTTTTGGTCCCATGTGCACACATTATACGCAG 133
Pan     ACCCCAGATGTCCTATGAGATGTTTTGGTCCCATGTGCACACATTATACGCAG 133
Macaca  ACCCCAGATGTCCTATGAGATGTTTTGGTCCCATGTGCACACATTATACGCAG 133
Bos     ATCCCCGATGTCCTATGAGATGTTTCGGCCCCATGTGCACACATTATACACAG 133
Canis   ATCCCAGATGTCCTATGAGATGTTTCGGTCCTATGTGCACACATTATACGCAG 133
```

ENSG00000137558 intron 2

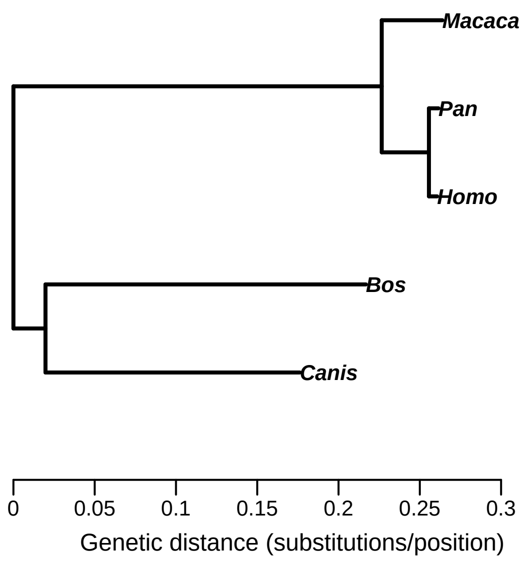

ENSG00000137558 intron 2

```
Homo    GTAGGAAGTAATTTCACTGATGCAGTTCATCCACAT------------GGCTTTATTAATTATGGACTCTAGGAGGGAATTTCATCAATTTCAAGTAATA 88
Pan     GTAGGAAGTAATTTCACTGATGCAGTTCATCCACAT------------GGCTTTATTTATTATGGACTCTAGGAGGGAATTACATCAATTTCAAGTAATA 88
Macaca  GTAGGAAGTAGTTTCACTGATGCAGTTTATCCATAT------------GGCTTTATTAATTATGGACTCTAGGAGGGAATTACATCAGTTTCAAATAATA 88
Bos     GTAAGAAATAGTTTCACTGATACTGTTTATCCAGATAACTTTATTGATAACTTTATTGCCTATGGACTCCAAGTGTTAGTAGTGTCAATTTCAAGTAACA 100
Canis   GTAGGAATCAGTTTCATTGATACTGTTCATCCACAC-------------ACTTTGTTACTTATGGATCCCAAG-GGGAGTGACACCACTTTCAAGTAATA 86

Homo    TCAACAAATTCTTA-TTGAATGCTCACTATGTGTCAGGTACTAACGCTTGGCCTATGAATATTTTAGATGTCTTCCAGAATTACTTAATTAGGCTAAAGG 187
Pan     TCAACAAATTCTTA-TTGAATGCTCACTATGTGTCAGGCACTAACGCTTGGCCTATGAATATTTTAGATGTCTTCCACAATTACTTAATTAGGCTAAAGG 187
Macaca  TCAACAAATACCTA-TTGAATGCTCACTATGTGTCAGGTACTAATGCTTGGTCTACGAATATTTTAGATGTCTTCCAGAATTATTTAATTGGGTTAAAGG 187
Bos     TCTACAGGTATTTATTTGAGAACTCACTATATGCCAGTTGCTGATGCTTGGCCTATGACTGTTTTAGATGTCTTAAAAAATTATTTAAATTTTTAAAAAA 200
Canis   TCAGCAGATATATA-TTGAGTGCTCACTCTGTGTCAGGCAATGATACTTGGCCTGTGACTGTTTCAGATAGTTTCAAGGAGTATTTAATTTGTTAAAAAG 185

Homo    G-------------CCAGTTCCCCTTTTCACCCTTGGAACACCTTCCCTTAGC----------------------------------------------- 227
Pan     G-------------CCAGTTCCCCTTTTCACCCTTGGAACACCTTCCCTTAGC----------------------------------------------- 227
Macaca  G-------------CCAGTTCCCCTTTTCACCCCTGGAACACCTTCCCTTAGC----------------------------------------------- 227
Bos     GCCTAAAAAAACCCCCAGTTTTCTCTTTCACCCTTGGAACACCATCCCTTAATCTTTTCTACACTGCTGCTGCTGCTGCTAAGTCACTTCGGTCGTGTCT 300
Canis   ACCTAAAAAGA---CCAATCTCCCCTTTCACTTTTGGAACACCCTTTCTTAGT----------------------------------------------- 235

Homo    ---------------------------------------------------------------------------------------------------- 227
Pan     ---------------------------------------------------------------------------------------------------- 227
Macaca  ---------------------------------------------------------------------------------------------------- 227
Bos     GACTCTGTGCGACCCATAGACCGCAGCCCACCAGGCTCCCACGTCCCAGGGATTCTCCAGGCAAGAACACTGGAGTGGGTTGCCATTTCCTTCTCCAATG 400
Canis   --------------------------------------CCATGTC------------------------------------------------------- 242

Homo    --------------CTGGATTTTTCTTTGAGCTCGCAGAATCTTAGAAAGGGTCAAGCC------TGTGCTGTATCTTGCCCCCTCCATCTCAGGAAAAA 307
Pan     --------------CTGGATTTTTCTTTGAGCTCGCAGAATCTTAGAAAGGGTCAAGCC------TGTGCGGTATCTTGCCCCCTCCATCTCAGGAAAAA 307
Macaca  --------------CTGGACTTTTCTTTGAGCTCACAGAATCTTAGAAAGGGTTAAGCC------TGTGCTGTATCTTGCCCCCTCCATCTCAGGAAAAA 307
Bos     CATGAAAGTGAAAAGTGAATTCTACACTAATCTCACAGAATCTTTGAAAGAATCAGGTTGTAT------CCTTATCCTATCGCTTCTAGTTCAGAAAAAT 494
Canis   -------------------------------CTCCCAGAATCTTTGAAAGGATCAAGCGATGCTATATACTGCACCCTGATTTTCCCACGTAAGGAAAAA 311

Homo    GCCCCATGACTGTGCAGAGGCTGTGTGTGT--GTGTGTGTGTGTGTGTGTGTGTGTGTGTGTGTGTATGCATATGAGTGCAACTTTCCCAAAGTTGCATA 405
Pan     GCCCCATGACTGTGCAGAGGCTGTGTGTGTGCGTGTGTATGTGTGTGTGTGTGTGTGTGTGTGTGTATGCATATGAGTGCAACTTTCCCAAAGTTGCATA 407
Macaca  GCTCCATGACTGTGCAGAGGTTGTGTGTGT----------------------GCATGTGTGTGCATATGTACATGCATGAAACTTTCCCATAGTTGCATA 385
Bos     CCCTTGATAGTG-GCAGAAGCCATTATTAT------------------------------------------G--TATGCAGCTTTCCCATGTTTGCATA 549
Canis   ATTTTGAATGTA-GCAGAGGCCATTCTTGT------------------------------------------GTTTATTCAGCTTTCCCGTGGTTACATC 368

Homo    TACCCGCATTTACAT-----AAACATATACAATAAAAATGTAGGCATTTACATATGTTT--ATACACATACATATACATGCAGGTATATGCAGCTATGGG 498
Pan     TACCCGCATTTACAT-----AAATATATACAATAAAAATGTAGGCATTTACATATGTTT--ATACACACACATATACATGCAGGTATATGCAGCTATGGG 500
Macaca  TACCTGCATATACCT-----AAATGTATACAATAAAAATGTATGCATTTACATATATTT--ATACACGCACCTATACATGCAGGTATAGGCAGCTATGGG 478
Bos     TTCCTATATATGTGTTATACAAATTCAATGTATTTACATGCATATG--TACATATACTT--ATGCACATATATGTATATGCAAGTATATGCAACTGTGAA 645
Canis   TATCTGTATGTCTGT-----GTGTGTTATATATTTACATATATATGAATACACGCACTTACACACACACACATGTATAGGCAAGTATGTGCAACTATGAG 463

Homo    AAAAGCTATTCCCATTTGCTAGGATAATTTTTCTGCCACTGTGCAAAGTCGTTATCCAACACTCCAAATACCCA-TCTCCCCCAAATAGGGAAGGAAAAT 597
Pan     AAAAGCTATTCCCATTTGCTAGGATAATTTTTCTGCCACTGTGCAATGTCGTTATCCAACACTCCAAATACCCA-TCTCCCCCAAATAGGGAAGGAAAAT 599
Macaca  AAAAGCTATTCCAATTTGCTAGGATAATTTTTCTGCCACTGTGCAATGTCATTATCCAACATTTCAAAGACCCA-TCTCCCCCAAATAAGGAAGGAAAAT 577
Bos     TGAAGCTGCTTCCG-GTAACAGGATAATTTTTCTG-CACTGTGAGAGGTCATCATCCAACATT-CAAAGACTCATTCCCTTCCATAGAGGGGAGAAAAAT 742
Canis   AAAGGCCGTACCCA-GTAATAGGATAATTTTTCCACCACTGTGGAATGTCATCATCCAACATTCCAAAGACCTATTCTCTTCCATATACAGGAGAAAAAT 562

Homo    CAACTGTGCTATTATTAAAACTAAGGGAATTCAGACTGAAGTGAAAAAAATGCACTGGGTTCTAATATTGTGACTACTTTTATACATCTAATTCTGGATC 697
Pan     CAACTGTGCTATTATTAAAACTAAGGGAATTCAGACTGAAGTGAAAAAAATGCACTGGGTTCTAATATTGTGACTACTTTTATACATCTAATTCTGGATC 699
Macaca  CAACTGTGCTATTATTAAAACTAAGGGAATTCAAACTGAAGTGAAAAAAATGCACTGGGTTCTACTATTGTGACTACTTTTATACATCTAATTCTGAATT 677
Bos     CGGGTGAATTCTCACT-AAATTAAAATGATACAACTTTAAGGGGGAAATCACTCTGAG-TCTAGTATCACAGTTTTTTTTA-ATATCTAATTTTTAATT 839
Canis   CAGTTGTGCTATTGTTAAAATTAAAGTGATTCAAGTTTAAGGAAAAAA--CACACTGAGTTCTAGTATCACAACTACTTTTATATATCTAATTTTTAGTT 660

Homo    ATTTATAAAATTCTGATGGAGGAAGTTTTCGACATGGATTAA-ATAAGACA---TTGATGATTCCTGAAATTATTTGCATTTTCTTCGTGGAATGACCAG 793
Pan     GTTTATAAAATTCTGATGGAGGAAATTTTCGACATGGATTAA-ATAAGACA---TTGACGATTCCTGAAATTATTTGCATTTTCTTCGTGGAATGACCAG 795
Macaca  GCTTATAAATTTCTGATGGAGGAAGTTTTCGACATGGATTAA-ACAAGACA---TTGATGATTCCTGAAATTATTTGCATTTTCTTTGTGGAATGACCAG 773
Bos     ATGTATAAGATTCCAATGGAAGACATTTCTGACATTGGTAAATATAAGGTAAAGATGATAATTCCCGAAAGATTTGCATTTCTCTTTTTGAAACAACCAG 939
Canis   GCATATATGATTCTGGTGGGAGAGATTT-TGACATTGATAAA-ACAAGAAAAATATGAAGATTCCTGAAATATTTGTATTTCTCTTTTTGAAACAACCAG 758

Homo    ACACATGTTTAGACAAATACAGAATCACTTACTCCAGCCCTGGCCATTTCCATGCTTGGCCCTAGTTGGAGGAATAGAGGACCCGACTAGGTGGTGATGG 893
Pan     ACACATGTTTAGACAAATACAGAATCACTTACTCCAGCCCTGGCCATTTCCATGCTTGGCCCTAGTTGGAGGAATAGAGGACCCGACTAGGTGGTGATGG 895
Macaca  ACACATGTTTAGACAAATACAGAATCACTTACTCCAGCCCTGGCCATTTCCATGCTTGGCCCCACTTGGAGGAATAGAGGACCTGACTAGGTGGTGATCG 873
Bos     ACTCATACTTAAACAAATATAGGATCACTTGCTCCAGGCCTGAACATTTTCATGCTTGGCTCTACTGGGAGGAACAGAGGATTCTGCTGGGTGGTGATGG 1039
Canis   ACATGTACTTAAACAAATGTAGGCTCACTTATGTTAGTCCCGAGCATTTTCATGCTTGGCCCTACTTGGAAGAA--------------TAGGTGATGATGG 845

Homo    TGATTGTGA-GGGGTGGATGTGCTCTTGGATCATCATGAATAATCTGC-AAAAAGAACAATCAATTTACAGTGTAACATGAGTCCCTTATATATTACCAC 991
Pan     TGATTGTGA-GGGGTGGATGTGCTCTTGGATCATCATGAATAATCTGC-AAAAAGAACAATCAATTTACAGTGTAACATGAGTCCCTTATATATTACCAC 993
Macaca  TGATTGTGA-GGGGTGGACGTGCTCTTGGATCTTCATGAATAATCTGT-AAAAAGAATAATCAATTTACAGTGTAACATGAGTCCCTTATATATTACCAC 971
Bos     TTATTGTGAGGGGGTGGATATGCTCTTGATTTGCCATTAATAGCCTGC--AAAAGAACAATTAACTTGTAATTTAGTGTGAGTCCACTACATATTGCCAC 1137
Canis   TAATTGTGA-GGCTTGGATTTGCTCTTGATTCATCATGAATAATCTGCAAAAAAGAACTATTAACTTACAATTTAACTTGAGTCCACTATAAATTACCCC 944

Homo    TGCTGCACTCTGATTTCTTTCT---TTTCTTTATATGCAG 1028
Pan     TGCTGCACTCTGATTTCTTTCT---TTTCTTTATATGCAG 1030
Macaca  TGCTGCACTCTGATTTCTTTAT---TTTCTTGATATGCAG 1008
Bos     TGCTGAACTGTGAGTTCTCATTGTGTTTCTTGATATTTAG 1177
Canis   TGCTACACTCTAAGTTCTCATTGTTTTTCTTGGTATCTAG 984
```

ENSG00000110455 intron 6

Description: 1-aminocyclopropane-1-carboxylate synthase (ACCS)
Intron number: 6
Human chromosome: 11
Intron start (bp): 44055503
Human intron length : 468
Intron alignment length: 556
Flanking exons length (upstream/downstream): 98/78
SNP density: 0.004274
K tree score: 0.0306
Scaling factor: 0.8403
Human-chimpanzee distance: 0.015073
Total primate branch length: 0.0773

ENSG00000110455 exon 6

```
Homo    AGGCTTTCCTGATCCCCACCCCTTACTATGGCGCTATCACACAGCACGTGTGTCTCTATGGCAACATCCGGCTGGCCTAT 80
Pan     AGGCTTTCCTGATCCCCACCCCTTACTATGGCGCTATCACACAGCACGTGTGTCTCTATGGCAACATCCGGCTGGCCTAT 80
Macaca  AGGCTTTCCTGATCCCCACCCCTTACTATGGCGCCATCACACAGCACGTGTGTCTCTACGGCAATGTCCGACTGGCCTGT 80
Bos     AGGCTTTCCTGATCCCTGCCCCTTACTATGGAGCCATCACACAGCACGTGTATCTCTATGGCAACGTCCGACTGGTCTGT 80
Canis   AGGCTTTCCTGATCCCCGCCCCTTACTATGGAGCCATCACACAGCATGTCTGTCTCTATGGCAATGTCCGACTGGTCTGT 80

Homo    GTCTACCTGGACAGTGAG 98
Pan     GTCTACCTGGACAGTGAG 98
Macaca  GTCTACTTGGACAGTGAG 98
Bos     GTCTATCTGGACAGTGAG 98
Canis   GTCCATCTGGACAGTGAG 98
```

ENSG00000110455 exon 7

```
Homo    GTCACTGGGCTAGACACACGCCCCTTCCAGCTCACAGTGGAGAAGCTGGAGATGGCCCTGAGAGAAGCTCACTCTGAG 78
Pan     GTCACTGGGCTAGACACACGCCCCTTCCAGCTCACAGTGGAGAAGCTGGAGATGGCCCTGAGAGAAGCTCACTCTGAG 78
Macaca  GTCACTGGGCTAGACACACGCCCCTTCCAGCTCACAGTGGAGAAGCTGGAGATGGCCCTGCGAGAAGCTCACTCTGAG 78
Bos     GTCACTGGGCTGGAAACACGCCCCTTCCAGCTCACAGTGGAGAAGCTGGAGATGGCCCTGCAAGGAGCTAATTCTGAG 78
Canis   GTCACTGGGCTGGACACACGTCCCTTCCAGCTCACAGTGGAGAAGCTGGAGATGGCCCTGCAAAGAGCTCATTCTGAG 78
```

ENSG00000110455 intron 6

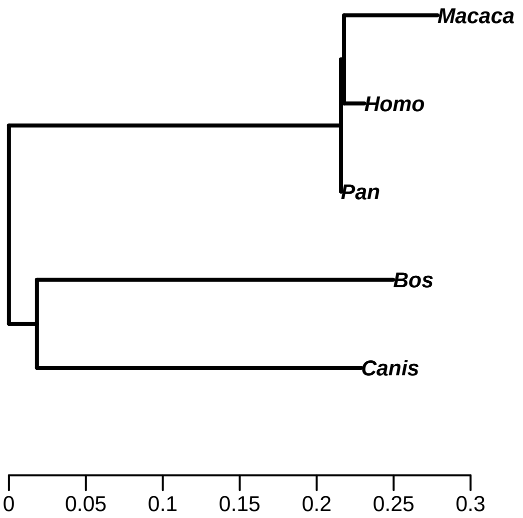

Genetic distance (substitutions/position)

ENSG00000110455 intron 6

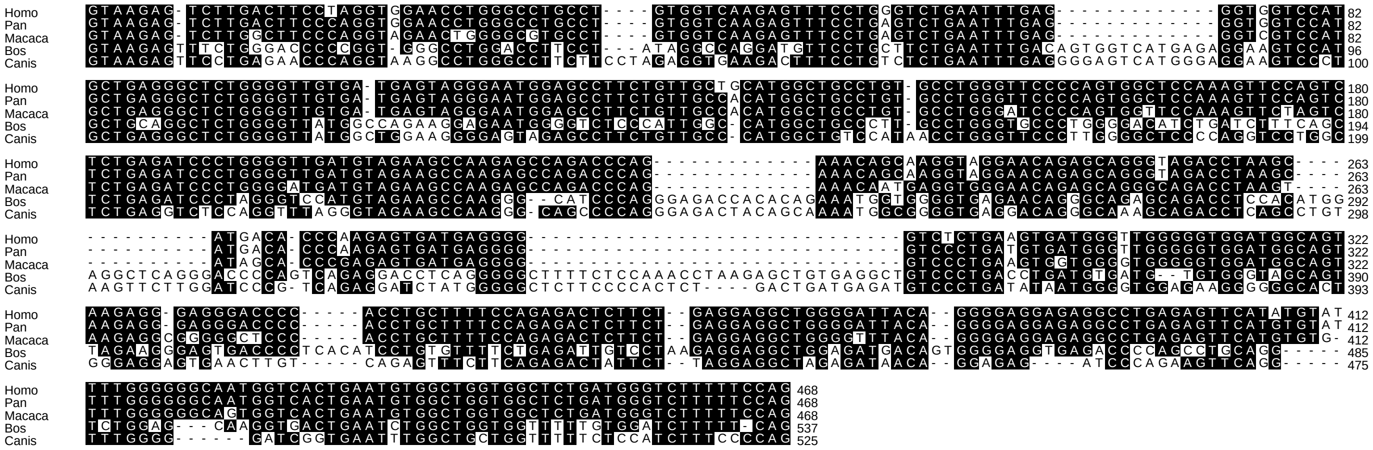

ENSG00000137857 intron 9

Description: Dual oxidase 1 precursor (DUOX1)
Intron number: 9
Human chromosome: 15
Intron start (bp): 43217505
Human intron length : 1051
Intron alignment length: 1353
Flanking exons length (upstream/downstream): 91/103
SNP density: 0.000000
K tree score: 0.057
Scaling factor: 1.0401
Human-chimpanzee distance: 0.018474
Total primate branch length: 0.0773

ENSG00000137857 exon 9

```
Homo    AAATGCCAGCTGCCACTTCCAGGGGGTCATCAATCGGAACTCAAGTGTCTCCAGAGCTCTCCGGGTCTGCAACAGCTACT 80
Pan     AAATGCCAGCTGCCACTTCCAGGGGGTCATCAATCGGAACTCAAGTGTCTCCAGAGCTCTCCGGGTCTGCAACAGCTACT 80
Macaca  AAATGCCAGCTGCCACTTCCAGGTGGTCATCAATCGGAACTCAAGTGTCTCCAGAGCTCTCCGGGTCTGCAATAGCTACT 80
Bos     AAATGCCAGCTGCCACTTCCAGGGGGTCATCAATAGGAACTCAAGTGTCTCCAGAGCTCTCCGGGTCTGCAACAGCTACT 80
Canis   AAATGCCAGTTGTCACTTCCAAGAGGTCATCAATAGGAACTCAAGTATCTCCAGAGCTCTCCGGGTCTGCAATAGCTACT 80

Homo    GGAGCCGTGAG 91
Pan     GGAGCCGTGAG 91
Macaca  GGAGCCGTGAG 91
Bos     GGAGCCGAGAG 91
Canis   GGAGTCGAAAG 91
```

ENSG00000137857 exon 10

```
Homo    CACCCAAGCCTACAAAGTGCTGAAGATGTGGATGCACTGCTGCTGGGCATGGCCTCCCAGATCGCAGAGCGAGAGGACCA 80
Pan     CACCCAAGCCTACAAAGTGCTGAAGATGTGGATGCACTGCTGCTGGGCATGGCCTCCCAGATCGCAGAGCGAGAGGACCA 80
Macaca  CACCCAAGCCTACAAAGTGCTGAAGATGTGGATGCACTGCTGCTGGGCATGGCCTCCCAGATCGCAGAGCGAGAGGACCA 80
Bos     CACCCCAACCTACAAAGAGCTGAAGATGTGGATGCGCTACTGCTGGGCATGGCCTCCCAGATCGCTGAGCGAGAGGACCA 80
Canis   CATCCAAACCTACGAAGAGCTGAAGATGTGGACGCGCTACTGCTAGGCATGGCCTCCCAGATCGCTGAGCGAGAGGACCA 80

Homo    TGTGTTGGTTGAAGATGTGCGGG 103
Pan     TGTGTTGGTTGAAGATGTGCGGG 103
Macaca  CGTGGTGGTTGAAGATGTACGGG 103
Bos     TGTGGTGGTGGAGGACGTGCGAG 103
Canis   TGTGGTGGTTGAGGATGTGCTAG 103
```

ENSG00000137857 intron 9

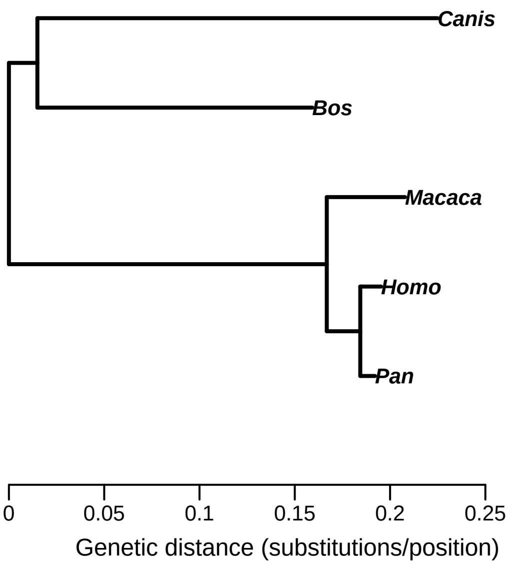

ENSG00000137857 intron 9

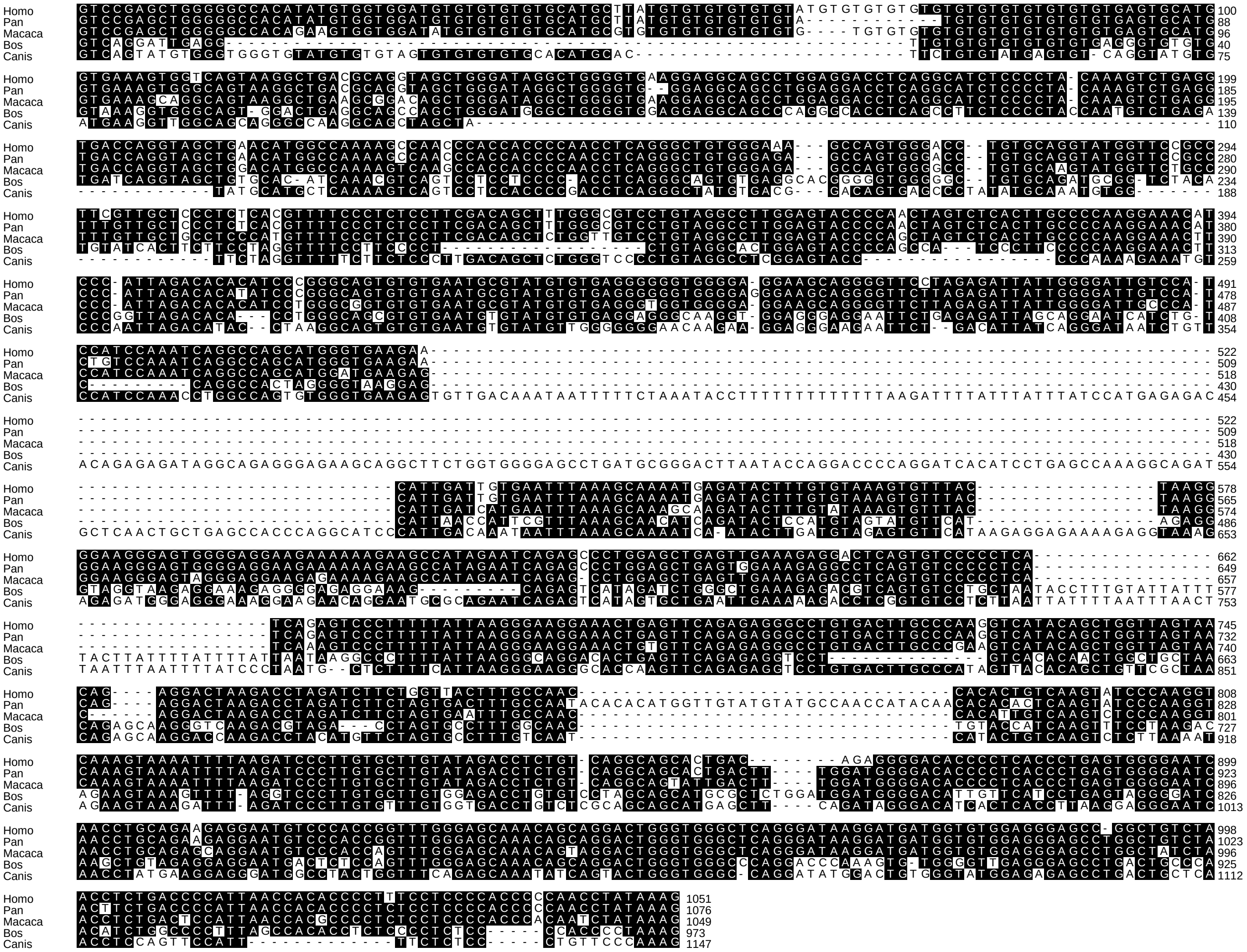

ENSG00000153975 intron 7

Description: Zinc finger protein C6orf113 (ZUFSP)
Intron number: 7
Human chromosome: 06
Intron start (bp): 117073791
Human intron length : 1524
Intron alignment length: 1944
Flanking exons length (upstream/downstream): 152/221
SNP density: 0.004593
K tree score: 0.0314
Scaling factor: 0.8547
Human-chimpanzee distance: 0.010725
Total primate branch length: 0.0772

ENSG00000153975 exon 7

```
Homo    GTGTCATATTGTTGATTTTCACAAATCAACTGGTCCTTTGGGTACACACCCTCGCTTATTTGAATGGATATTGAACTATT 80
Pan     GTGTCATATTGTTGATTTTCACAAATCAACTGGTCCTTTGGGTACACACCCTCGCTTATTTGAATGGATATTGAACTATT 80
Macaca  GTGTCATATTGTTGATTTTCATAAGTCAACTGGTCCTTTGGGTACACACCCTCGCTTATTTGAATGGATATTGAACTATT 80
Bos     GTGCCGTATTGTTGACTTTCACGAGTCAACTGGTCCTTTGGGTACACACCCTCGCTTATTTGAGTGGATATTGAGCTACT 80
Canis   ATGCCACATTGTTGATTTTCATAAGTCAACTGGTCCTTTGGGTACCCATCCTCGCTTATTTGAATGGATATTGAACTATT 80

Homo    ATTCTTCAGAGGGAGAAGGGAGTCCAAAGGTAGTGTGTACATCTAAACCTCCTATCTATCTTCAGCATCAAG 152
Pan     ATTCTTCAGAGGGAGAAGGGAGTCCAAAGGTAGTGTGTACATCTAAACCTCCTATCTATCTTCAGCATCAAG 152
Macaca  ATTCTTCAGAGGGGGAAGGGAGTCCAAAGGTAGTGTGTACATCTAAACCTCCTATCTATCTTCAGCATCAAG 152
Bos     ATGCTTCAGAGAGAGAAGGAAGTCCAAAAGTAGTGTGTACATCTAAACCTCCTATCTATCTTCAGCATCAAG 152
Canis   ATTCTTCAGAGAGAGAAGGGAATCCAAAAGTAGTGTGTACATCTAAACCTCCTATCTATCTTCAGCATCAAG 152
```

ENSG00000153975 exon 8

```
Homo    GTCACAGTCGAACTGTTATTGGAATTGAAGAGAAAAAAAAACCGAACATTATGCTTACTAATACTTGATCCTGGATGTCCT 80
Pan     GTCACAGTCGAACTGTTATTGGAATTGAAGAGAAAAAAAAACCGAACATTATGCTTACTAATATTTGATCCTGGATGTCCT 80
Macaca  GTCACAGTCGAACTGTTATTGGAATTGAAGAGAAAAAAAAACCGAACATTATGCTTACTAATACTTGATCCTGGATGTCCT 80
Bos     GTCATAGTCGAACAGTTGTTGGAATTGAAGAGAAAAAAAAACCGAACATTGTGTTTACTAGTATTTGATCCTGGGTGTCCT 80
Canis   GTCATAGTCGAACAGTTGTTGGAATTGAAGAGAGAAAAAAATCGAACATTATGTTTACTAATATTTGATCCTGGATGTCCT 80

Homo    TCTCGAGAAATGCAGAAATTATTAAAGCAAGACATAGAGGCTAGCAGTCTCAAGCAACTTCGGAAATCTATGGGAAATTT 160
Pan     TCTCGAGAAATGCAGAAATTATTAAAGCAAGACATAGAGGCTAGCAGTCTCAAGCAACTTCGGAAATCTATGGGAAATTT 160
Macaca  TCTCGAGAAATGCAGAAATTGTTAAAGCAAGACGTAGAGGCTAGCAGTCTCAAGCAACTTCGAAAATCTATGGGAAATTT 160
Bos     TCTCGAGAAATGCAGAAACTCTTAAAGCAAGACATGGAAGTTAGCAGTCTCAAGCAACTACGGAAGTTTGTGGGGAATTT 160
Canis   TCTCGGGAAATGCAGAAACTATTAAAGCAAGATACAGAGGCTAGCAATCTCAAACAACTTCGGAAATTTGTGGGAAATTT 160
```

ENSG00000153975 intron 7

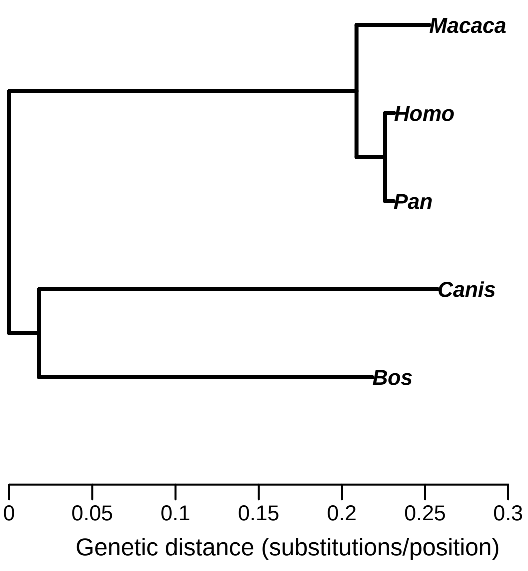

ENSG00000153975 intron 7

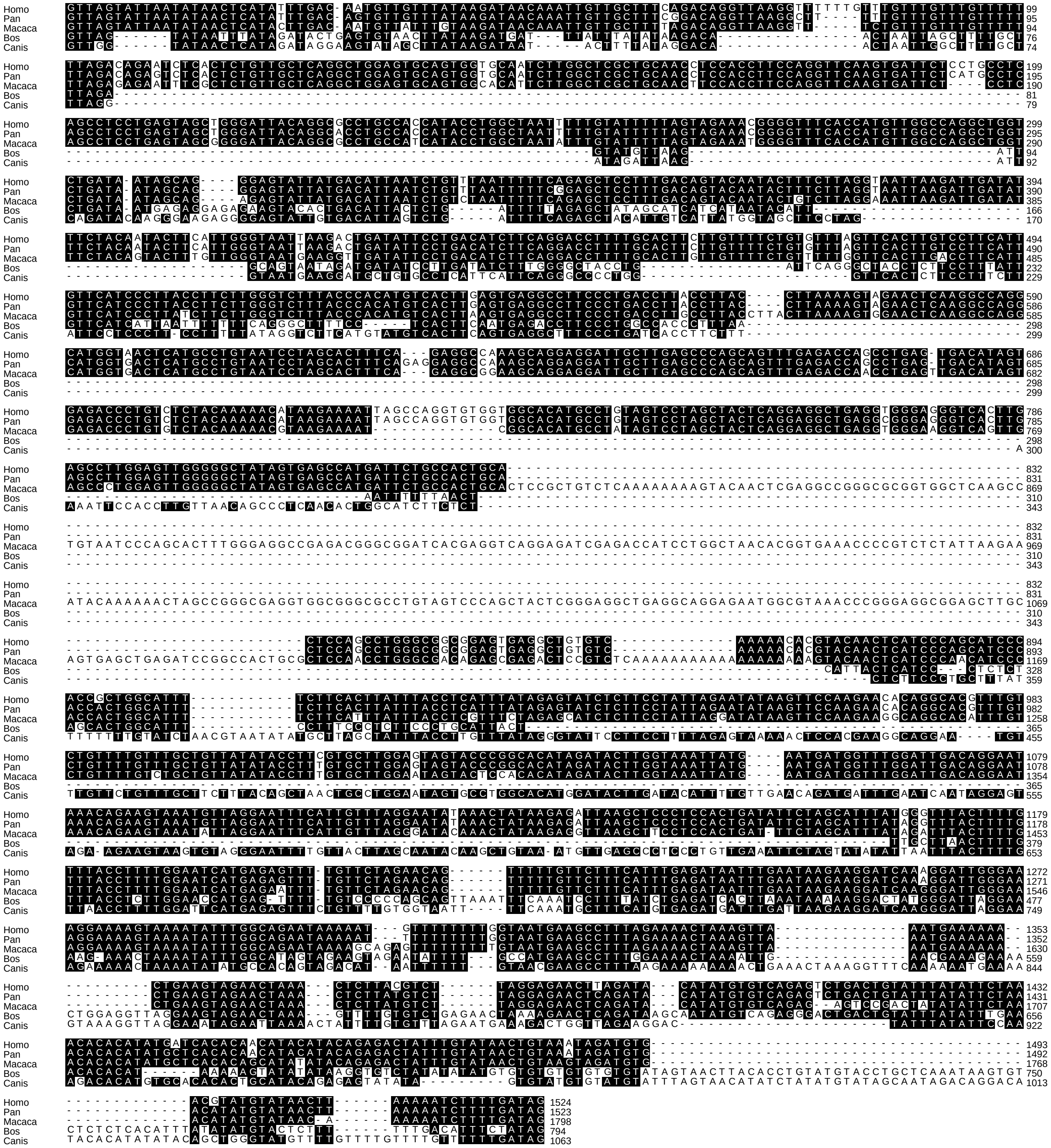

ENSG00000105771 intron 9

Description: Protein smg-9 homolog (C19orf61)
Intron number: 9
Human chromosome: 19
Intron start (bp): 48929726
Human intron length : 655
Intron alignment length: 846
Flanking exons length (upstream/downstream): 107/108
SNP density: 0.004580
K tree score: 0.0949
Scaling factor: 0.664
Human-chimpanzee distance: 0.012485
Total primate branch length: 0.0772

ENSG00000105771 exon 9

```
Homo    GTTCCTGCAGACAGCAGAGATGGTGAAGCCCTCCACCCCATCCCCCAGCCACGAGTCCAGCAGCTCATCGGGCTCCGATG 80
Pan     GTTCCTGCAGACAGCAGAGATGGTGAAGCCCTCCACCCCATCCCCCAGCCACGAGTCCAGCAGCTCATCGGGCTCCGATG 80
Macaca  GTTCCTGCAGACAGCAGAGATGGTGAAGCCCTCCACCCCATCCCCCAGCCACGAGTCCAGCAGCTCATCAGGCTCCGAGG 80
Bos     GTTCCTCCAGACAGCAGAGATGGTGAAACCCTCCACCCCATCTCCCAGCCACGAGTCCAGCAGCTCGTCAGGCTCCGAGG 80
Canis   GTTCCTCCAGACAGCAGAGATGGTGAAGCCCTCCACCCCGTCCCCCAGCCATGAGTCCAGCAGCTCCTCAGGCTCCGATG 80

Homo    AAGGCACCGAGTACTACCCCCACCTAG 107
Pan     AAGGCACCGAGTACTACCCCCACCTAG 107
Macaca  AAGGCACCGAGTACTACCCCCACCTGG 107
Bos     AAGGCGCTGAGTACTACCCCCACCTGG 107
Canis   AAGGCACGGAGTACTACCCCCACCTGG 107
```

ENSG00000105771 exon 10

```
Homo    TCTTCTTGCAGAACAAAGCTCGCCGAGAGGACTTCTGTCCTCGGAAGCTGCGGCAGATGCACCTGATGATTGACCAGCTC 80
Pan     TCTTCTTGCAGAACAAAGCTCGCCGAGAGGACTTTTGTCCTCGGAAGCTGCGGCAGATGCACCTGATGATTGACCAGCTC 80
Macaca  TCTTCTTGCAGAACAAAGCTCGCCGAGAGGACTTCTGTCCTCGGAAGCTGCGGCAGATGCATCTGATGATCGACCAGCTC 80
Bos     TCTTCCTGCAGAATAAAGCTCGCCGGGAGGATTTCTGTCCCCGCAAGCTACGTCAGATGCACCTGATGATCGACCAGCTC 80
Canis   TCTTCCTGCAGAACAAAGCTCGCCGAGAGGACTTCTGTCCTCGAAAGCTGCGACAGATGCACCTGATGATTGACCAGCTC 80

Homo    ATGGCCCACTCCCACCTGCGTTACAAGG 108
Pan     ATGGCCCACTCCCACCTGCGTTACAAGG 108
Macaca  ATGGCCCACTCCCACCTGCGTTACAAGG 108
Bos     ATGGCCCACTCCCACCTGCGGTACAAGG 108
Canis   ATGGCTCACTCCCACTTGCGTTACAAGG 108
```

ENSG00000105771 intron 9

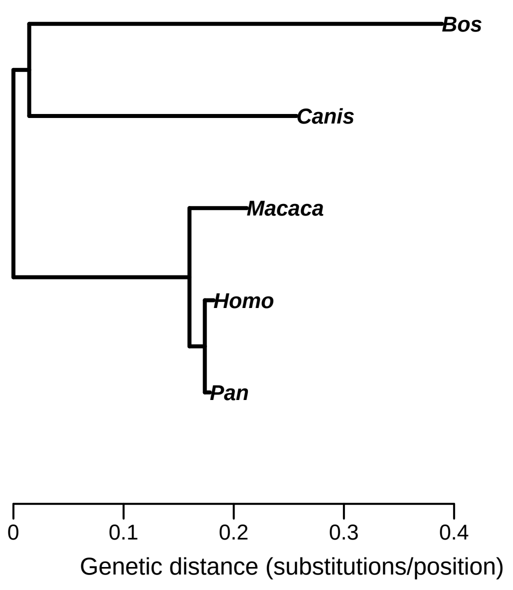

ENSG00000105771 intron 9

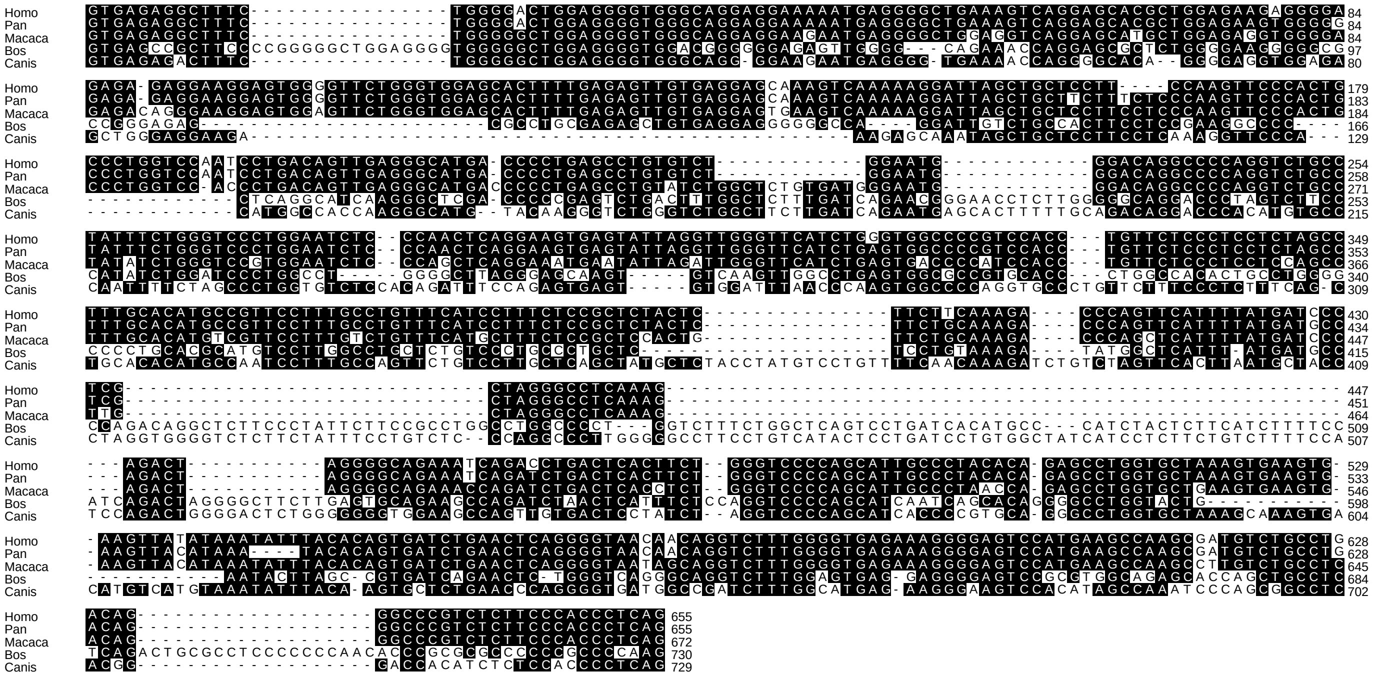

ENSG00000167139 intron 8

Description: TBC1 domain family member 21 (TBC1D21)
Intron number: 8
Human chromosome: 15
Intron start (bp): 71966007
Human intron length : 1007
Intron alignment length: 1365
Flanking exons length (upstream/downstream): 101/117
SNP density: 0.003972
K tree score: 0.0536
Scaling factor: 0.8102
Human-chimpanzee distance: 0.010038
Total primate branch length: 0.0771

ENSG00000167139 exon 8

```
Homo    AAGGGAAGGGTGCAGGGGCTGTGCAGTCCCTCTTCCCCTGGTTCTGCTTCTGCTTCCAGCGTGCCTTCAAGTCCTTCGAT 80
Pan     AAGGGAAGGGTGCAGGGGCTGTGCAGTCCCTCTTCCCCTGGTTCTGCTTCTGCTTCCAGCGTGCCTTCAAGTCCTTCGAT 80
Macaca  AAGGGAAGGGTGCGGGAGCTGTGCAGTCCCTCTTCCCCTGGTTCTGCTTCTGCTTCCAGCGTGCCTTCAAGTCCTTCGAT 80
Bos     AAGGGAAGGGTGCGGGGGCTGTGCCATCCCTCTTCCCCTGGTTCTGCCTCTGCTTTCAACGTGCCTTCAAGACCTTTGAC 80
Canis   GAGGGAAGGGTGCGGGGACTGTGCAGTCCCTCTTCCCCTGGTTCTGTCTCTGCTTCCAGCACGCCTTCAAGTCCTTCGAC 80

Homo    GATGTCTGGAGGCTCTGGGAG 101
Pan     GATGTCTGGAGGCTCTGGGAG 101
Macaca  GACGTCTGGAGGCTCTGGGAG 101
Bos     GACGTCTGGAGGCTCTGGGAG 101
Canis   GATGTCTGGAGGCTCTGGGAG 101
```

ENSG00000167139 exon 9

```
Homo    GTTCTGCTGACGGGGAAGCCCTGCAGGAACTTCCAGGTGCTGGTGGCCTACAGCATGCTGCAGATGGTGCGGGAGCAGGT 80
Pan     GTTCTGCTGACGGGGAAGCCCTGCAGGAACTTCCAGGTGCTGGTGGCCTACAGCATGCTGCAGATGGTGCGGGAGCAGGT 80
Macaca  GTTCTGCTGACGGGGAAACCCTGCAGGAACTTCCAGGTGCTGGTGGCCTACAGCATGCTACAGATGGTGCGGGAGCAGGT 80
Bos     GTGCTGCTCACAGGGAAACCCTGCAGGAACTTCCAGGTGCTGGTGGCCTACAGCATGCTGCAGATGGTGCGCCAGCAGGT 80
Canis   GTTCTGCTGACAGGGAAGCCGTGCAGGAACTTCCAGGTGCTGGTGGCCTACAGCATGCTGCAGATGGTGCGGGAGCAGGT 80

Homo    GCTGCAGGAAAGCATGGGCGGGGATGACATCCTCCTG 117
Pan     GCTGCAGGAAAGCATGGGCGGGGATGACATCCTCCTG 117
Macaca  GCTGCAGGAAAGCATGGGCGGGGACGACATCCTCCTG 117
Bos     GCTACAGGAGAGCATGACGGGCGACGATATCCTCCTG 117
Canis   GCTGCAGGAGAGCATGACCAGCAATGACATCCTCCTG 117
```

ENSG00000167139 intron 8

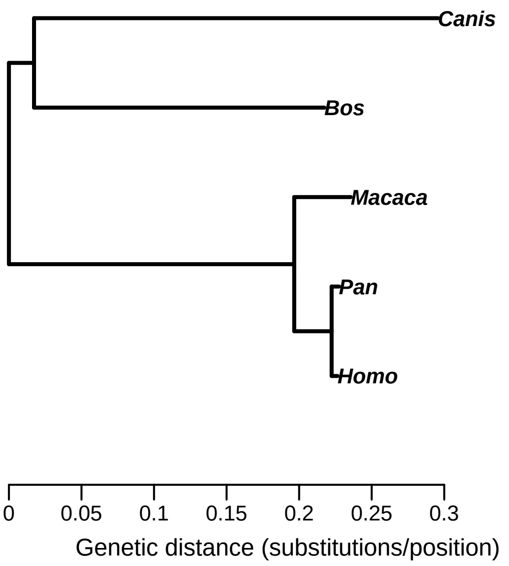

ENSG00000167139 intron 8

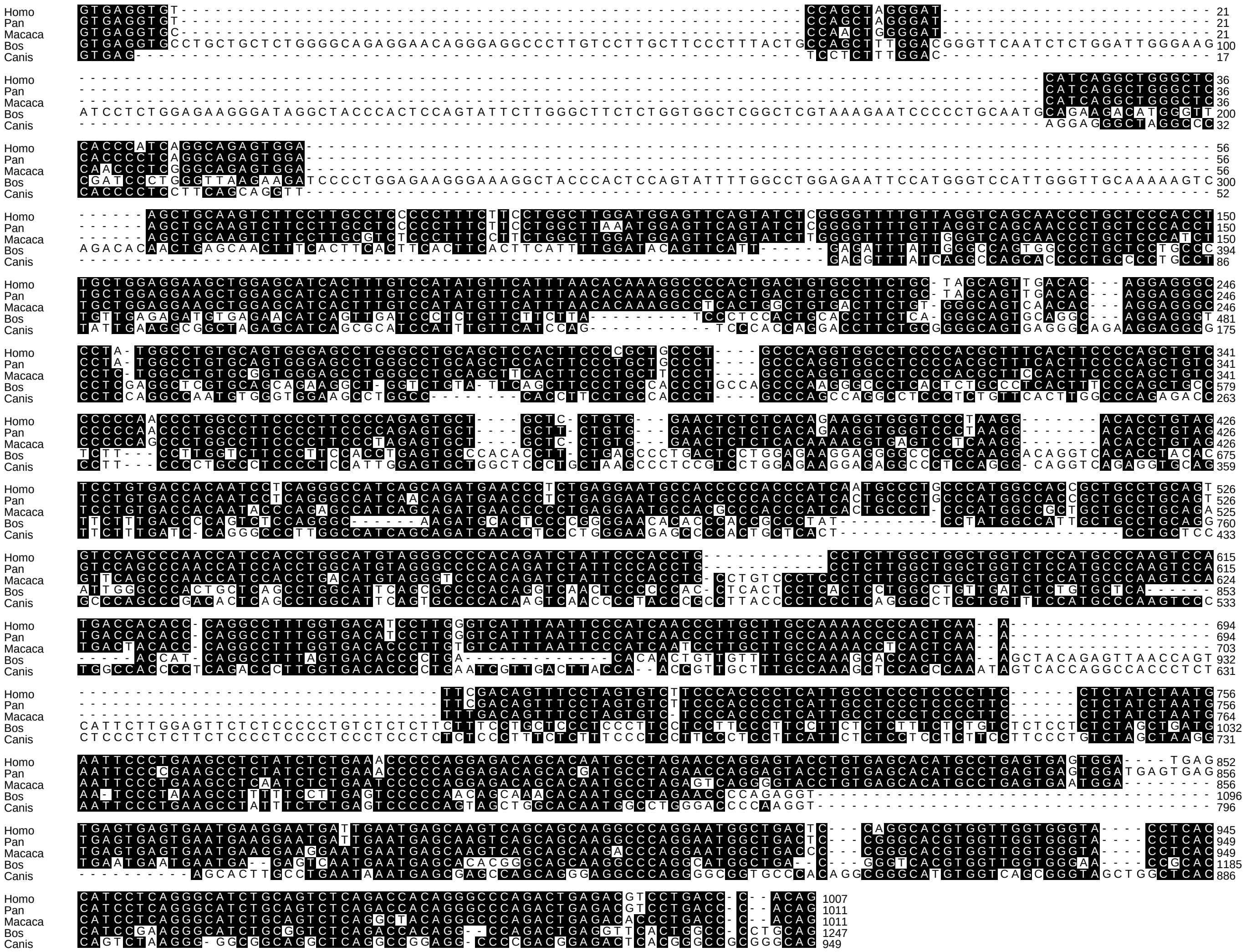

ENSG00000162782 intron 11

Description: Tudor domain-containing protein 5 (TDRD5)
Intron number: 11
Human chromosome: 01
Intron start (bp): 177886826
Human intron length : 971
Intron alignment length: 1015
Flanking exons length (upstream/downstream): 201/159
SNP density: 0.007209
K tree score: 0.0788
Scaling factor: 0.8884
Human-chimpanzee distance: 0.011503
Total primate branch length: 0.077

ENSG00000162782 exon 11

```
Homo    GTGCGGTTTGAAGCCATTAGTGGGGGTAGTGGATGAATATGTAGATGGAATCCTTAACATTTTTTTGTGTGACACATCCT  80
Pan     GTGCGGTTTGAAGCCATTAGTGGGGGTAGTGGATGAATATGTAGATGGAATCCTTAACATTTTTTTGTGTGACACATCCT  80
Macaca  GTGCGGTTTGAAGCCATTAGTAGGGGTAGTGGATGAATATGTAGATGGAATCCTTAACATTTTTTTGTGTGACACATCCT  80
Bos     GTGTGGCTTGAAGCCATTAGTAGGGGTAGTGGATGAATATGTAGATGGGATCCTGAACATTTTCCTGTGTGACACGTCCT  80
Canis   GTGTGGTTTGAAGCCATTAGTGGGGGTAGTGGATGAATATGTAGATGGAATCCTTAACATTTTTCTGTGTGATACATCCT  80

Homo    CAAACGAAGATGTCTATTTCCATCATGTCTTGAGAACAGAGGGCCATGCTATTGTATGCCGAGAAAATATCTCTTCTAAG  160
Pan     CAAACGAAGATGTCTATTTCCATCATGTCTTGAGAACAGAGGGCCATGCTATTGTATGCCGAGAAAATATCTCTTCTAAG  160
Macaca  CAAACGAAGATATCTATTTCCATCATGTCTTGAGAACAGAGGGCCATGCTATTGTATGCCGAGAAAATATCTCTTCTAAG  160
Bos     CAAATGAAGATGTCTATTTCCACCATGTTTTGAGGACAGAGGGTCATGCTATTGTATGCCGAGAAAATGTCCCTTCTAAG  160
Canis   CAAATGAAGATGTCTATTTCCATCATGTCTTGAGAACAGAGGGCCATGCTATTGTATGCCGAGAAAATGTCCCTTCTAAG  160
```

ENSG00000162782 exon 12

```
Homo    GGTTTCAGTGAGCTCAACCCTTTAGCTTTATACACGACATCCAGTGGAGGGCCAGAGGACATTGTCTTGACAGAACTGGG  80
Pan     GGTTTCAGTGAGCTCAACCCTTTAGCTTTATACACGACATCCAGTGGAGGGCCAGAGGACATTGTCTTGACAGAACTGGG  80
Macaca  GGTTTCAGTGAGCTCAACCCTTTAGCTTTATACACGAAATCCAGTGGAGGGCCAGAGGACATTGTCTTGACAGAACTGGG  80
Bos     GGTTTCAGAGACCTCAACCCTTTAGCTTTATACACTAAATCCAGTGTGACGCCTGAGGACATTGTCCTGAAAGAGCTGGG  80
Canis   GGTTTCAGAGAACTCAACCCTTTAGCTTTATATACTAAATCCAGTGCAGGGCCAGAAGATGTGGTCTTGACAGAACTGGG  80

Homo    TTATCCTTCCCAGCAGCACTATTTTAATGAAGACCGAAAGATAAGTCCACAGTCAAAAGAGAGTGAGTTACGTATCTTG  159
Pan     TTATCCTTCCCAGCAGCACTATTTTAATGAAGACCGAAAGATAAGTCCACAGTCAAAAGAGAGTGAGTTACATATCCTG  159
Macaca  TTATCCTTCCCAGCAGCACTATTTTAATGAAGACCGAAAGATAAGTCCACAGTCAAAAGAGAGTGAGTTACATATCCTG  159
Bos     CTATCATTCCCAGCAGCACTATTTTAATGAAGACCGAGAGATCAGTCCACAATCAAAAGAGAGTGACTTATATACCCTG  159
Canis   TTGTCCTTCCCAGCAGCACTATTTTAATGAAGACCGAGAGATAAGTCCACAGTCAAAGGAGAATGAATTACCTACCCTG  159
```

ENSG00000162782 intron 11

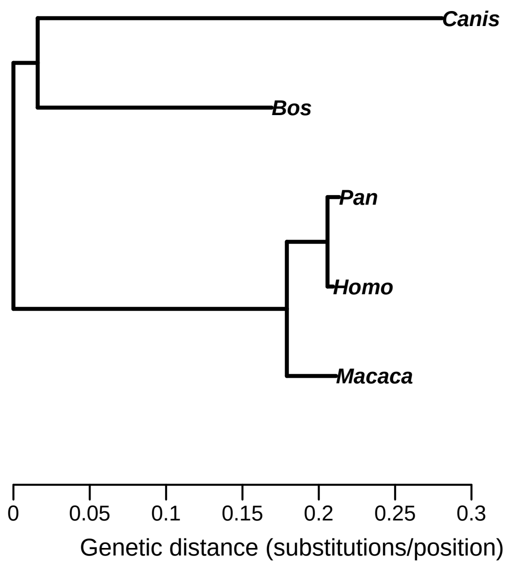

ENSG00000162782 intron 11

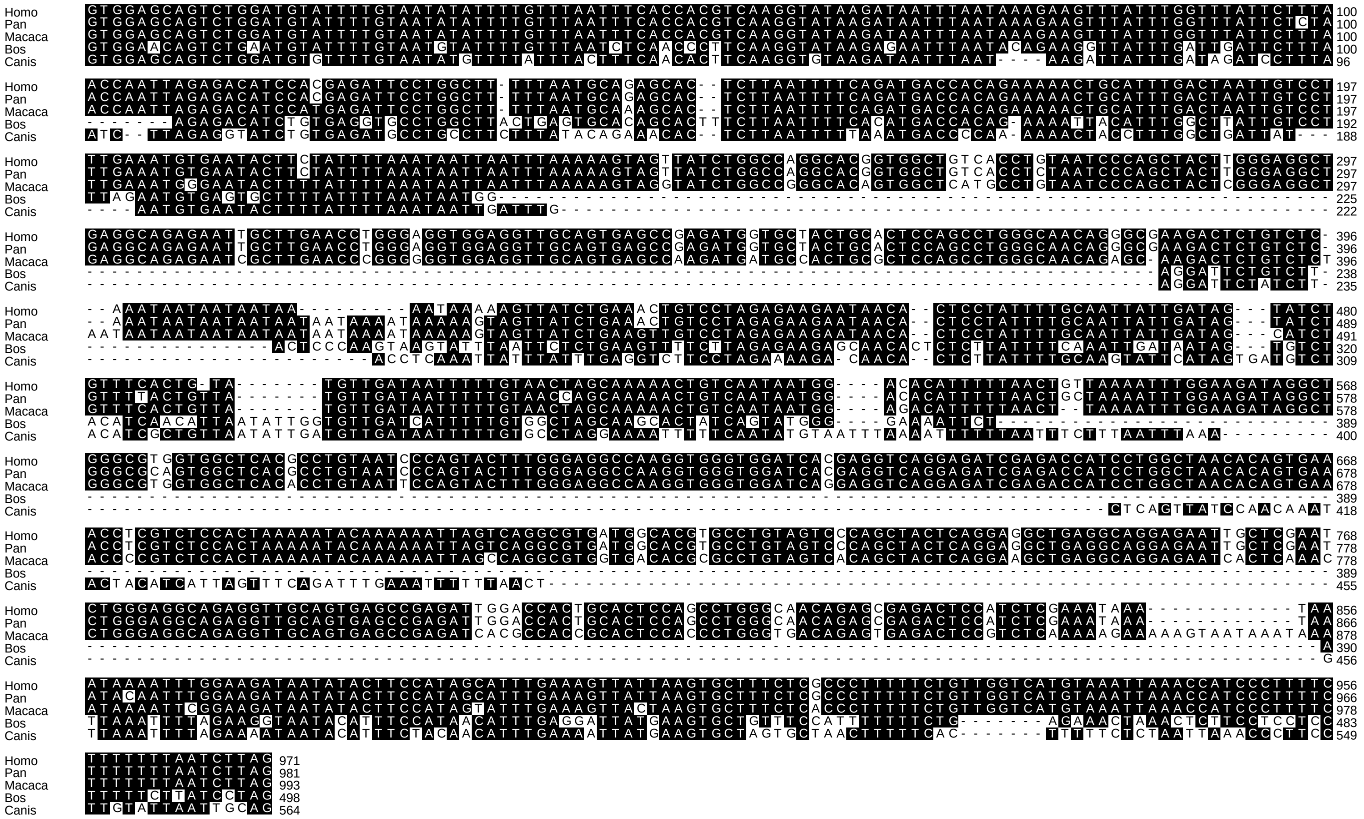

ENSG00000100985 intron 10

Description: Matrix metalloproteinase-9 precursor (MMP9)
Intron number: 10
Human chromosome: 20
Intron start (bp): 44075843
Human intron length : 327
Intron alignment length: 458
Flanking exons length (upstream/downstream): 140/151
SNP density: 0.009174
K tree score: 0.0627
Scaling factor: 0.6765
Human-chimpanzee distance: 0.003091
Total primate branch length: 0.077

ENSG00000100985 exon 10

```
Homo    GAAGTACTGGCGATTCTCTGAGGGCAGGGGGAGCCGGCCGCAGGGCCCCTTCCTTATCGCCGACAAGTGGCCCGCGCTGC 80
Pan     GAAGTACTGGCGATTCTCTGAGGGCAGGGGGAGCCGGCCGCAGGGCCCCTTCCTTATCGCCGACAAGTGGCCCGCGCTGC 80
Macaca  GAGGTACTGGCGATTCTCCGAGCGCAGGGGGAGCCGGCTGCAGGGCCCCTTCCTTATCGCCGACACGTGGCCCGCGTTGC 80
Bos     GAAGTACTGGAGACTTTCTGAGGGAGGGGGCCGCCGGGTGCAGGGTCCCTTCCTTGTCAAGAGCAAGTGGCCTGCGCTGC 80
Canis   GAAGTACTGGCGATTCTCCAAGGGCAAGGGACGCCGGGTGCAGGGCCCCTTCCTTATCACCGACACGTGGCCTGCGCTGC 80

Homo    CCCGCAAGCTGGACTCGGTCTTTGAGGAGCGGCTCTCCAAGAAGCTTTTCTTCTTCTCTG 140
Pan     CCCGCAAGCTGGACTCGGTCTTTGAGGAGCCGCTCTCCAAGAAGCTTTTCTTCTTCTCTG 140
Macaca  CCCGCAAGCTGGACTCGGCCTTTGAGGAGCCGCTCTCCAAGAAGCTTTTCTTCTTCTCTG 140
Bos     CCCGCAAGCTGGACTCCGCCTTCGAGGATCCGCTCACCAAGAAGATTTTCTTCTTCTCTG 140
Canis   CCCGCAAGCTGGACTCCGCCTTTGAGGACGGGCTCACCAAGAAGACTTTCTTCTTCTCTG 140
```

ENSG00000100985 exon 11

```
Homo    GGCGCCAGGTGTGGGTGTACACAGGCGCGTCGGTGCTGGGCCCGAGGCGTCTGGACAAGCTGGGCCTGGGAGCCGACGTG 80
Pan     GGCGCCAGGTGTGGGTGTACACAGGCGCGTCGGTGCTGGGCCCGAGGCGTTTGGACAAGCTGGGCCTGGGCGCCGACGTG 80
Macaca  GGCGCCAGGTGTGGGTGTACACAGGCGCGTCGGTGCTGGGCCCGAGGCGTCTAGACAAGCTGGGCCTGGGCGCCGACGTG 80
Bos     GGCGCCAAGTATGGGTGTACACCGGCGCGTCGTTGCTAGGCCCGAGGCGTCTGGACAAGTTGGGCCTGGGCCCGGAAGTG 80
Canis   GGCGCCAAGTGTGGGTGTACACAGGCACGTCGGTGGTAGGCCCGAGGCGTCTGGACAAGCTGGGCCTGGGCCCGGAGGTT 80

Homo    GCCCAGGTGACCGGGGCCCTCCGGAGTGGCAGGGGGAAGATGCTGCTGTTCAGCGGGCGGCGCCTCTGGAG 151
Pan     GCCCAGGTGACCGGGGCCCTCCGGAGTGGCAGGGGGAAGATGCTGCTGTTCAGCGGGCGGCGCCTCTGGAG 151
Macaca  GCCCAGGTGACCGGGGCCCTCCGGCGTGGCGCGGGGAAGATGCTGCTATTCAGCGGGCGGCGCTTCTGGAG 151
Bos     GCCCAGGTCACCGGGGCCCTCCCGCGCCCTGAGGGTAAGGTGCTGCTGTTCAGCGGGCAGAGCTTCTGGAG 151
Canis   ACCCAAGTCACCGGCGCCCTCCCGCAAGGCGGGGGTAAGGTGCTGCTGTTCAGCAGGCAGCGCTTCTGGAG 151
```

ENSG00000100985 intron 10

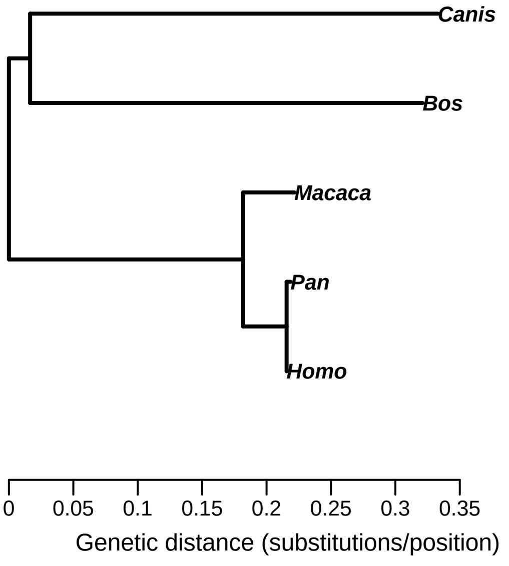

ENSG00000100985 intron 10

```
Homo    GTTAGTTACCTACTTTCCCTCCCCC---------GCCCGGTCAATC------------------------------------------------------ 37
Pan     GTTAGTTACCTACTTTCCCTCCCCC---------GCCCGGTCAATC------------------------------------------------------ 37
Macaca  GTTAGTTACCTTCTACGCCTCCCT---------------GTCAATC------------------------------------------------------ 31
Bos     GTTAGTTTTCGTCTTTCTCCCTCCCCTGTGCTGTGCTTGGTCGCTCAGTCGTGTCCGACTCTTTGCGACCCTGTGGACTGTAGCCCACCAGGTTCCTCTG 100
Canis   GTTAGTTTCCTCCTTTTTCTTCCTCCGCTCCC--GCCATCCTCATC------------------------------------------------------ 44

Homo    ----------CCCATCAGTCAAGGAGGCTCAAGAGA---------------------------------CCATCGATAACCCACGAAACGTCTTGTGCGT 94
Pan     ----------CCCATCAGTCAAGGAGGCTCAAGAGA---------------------------------CCATCGATAACCCACGAAACGTCTTGTGCGT 94
Macaca  ----------CCCATCAGTCAAGGAGGCTCAAGAGA---------------------------------CCGTCGATAACCCAGGAAATGTCTTGTGCAT 88
Bos     TCCATGGGGATTCTCCAGGCAAGAAGACTGGAGTGGGTTGCCATTTCTTCTTTCTCCTGGGGATCTTCCCCACCCAGGGATCGAACTGGGTATCCCGCAT 200
Canis   ----------CTCGCCAATCTAGGAGGGCCAAGAGA--------------------------------CCGAGTATAAATCCGTGAAACAGTTTACGCGG 102

Homo    TTTAGAAAAA------------TACGCCCCCTGGCGGACGCAGTTTAGCAAACGTAGGGGCGGCTGAGTTTCTGCCCCCTCCTCTCCACGCCCTCGCGTC 182
Pan     TTTAGAAAAA------------TACGCCCCCTGGCGGACGCAGTTTAGCAAACGTAGGGGCGGCTGAGTTTCTGCCCCCTCCTCTCCACGCCCTCGCGTC 182
Macaca  TTTAGAAAAA------------TACGCCCCCTGGCGGACTCAGTTTAGCAAACGTAGGGACGGCTGAGTTTCTGCCCTTTCCTCTCCACGCCCTCGCGCC 176
Bos     TGCAGGCAGATTATTTACCGTCTGAGCCACCAGGGGAGCCC---TGAACAAATTTAGCAG-GGCTGAATTTCAGCCC--TCCTCACAGCACCCTGGCACA 294
Canis   ATTAGGAAAA-----------------------------------------------AGGCGCCCCCTTCTGGCGCCGATCGTCACCGTCCCCTTGCACA 155

Homo    GCTCTACCCAGCGCCTCTGCCCCTGGGTTGCAGGGACTGCGGGCA----------CGCGGGCTAGGAAAGGCCTCGCCGGAATCTCCCTCCTCGCGTTCT 272
Pan     GCTCTACCCAGCGCCTCTGCCCCTGGGTTGCAGGGACTGCGGGCA----------CGCGGGCTAGGAAAGGCCTCGCCGGAATCTCCCTCCTCGCGTTCT 272
Macaca  GCTCTACCCAGCGCCTCTGCCCCTGGGGTGCAGGGACTGCGGGCA----------CGCGGGCTA-GAAAGGCCTCGCCGGGATCTCCCTCCTAGCGTTCT 265
Bos     GTCCTACCGAGGGGCTCTGCTCCTTGGGCACAGAGACTGCGGGCA----------CTC-------CTGGACCGTGCCTGCGTTTTCCCTCTTGACATTCA 377
Canis   GTCCGATGGAGCCGGTCTCCTCCGTGGGCACCGAGTCTGCGGGCACCTCGGGCACCTCGGGCGCAGAAGGGCCTCGCTGGGTTCTTCTCCTTGACATTCA 255

Homo    AG--GAGTACGTGCTCC-CTCTGCGCCCCCAAACCGACGTGACCCTCCTCCCCTGCAG 327
Pan     AG--GAGTACGTGCTCC-CTCCGCGCCCCCAAACCGACGTGACCCTCCTCCCCTGCAG 327
Macaca  AGAAGAGTACGGGCTCT-CTCTGCGCCTCCAGACCGATGTGACCCTCCTCCCCTGCAG 322
Bos     AGGGGGGTGTGTACCCCACTCCCTGCCCCCAGACCGATGTGAGCCT-CTCTTCGGCAG 434
Canis   AGAGCAATGTGTGTCCA-CTCCCTGCCCCCAGACCGACGCGGCTCTTCTCCCCTGCAG 312
```

ENSG00000115884 intron 3

Description: Syndecan-1 precursor (SDC1)
Intron number: 3
Human chromosome: 02
Intron start (bp): 20266465
Human intron length : 590
Intron alignment length: 676
Flanking exons length (upstream/downstream): 479/136
SNP density: 0.000000
K tree score: 0.0587
Scaling factor: 0.7151
Human-chimpanzee distance: 0.014078
Total primate branch length: 0.077

ENSG00000115884 exon 3

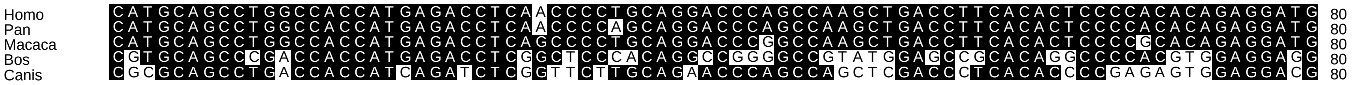

ENSG00000115884 exon 4

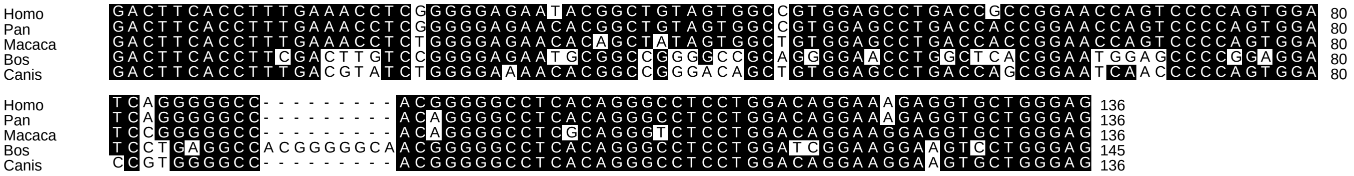

ENSG00000115884 intron 3

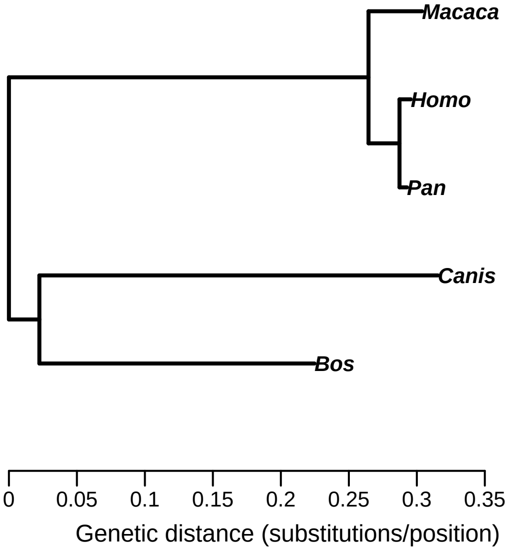

ENSG00000115884 intron 3

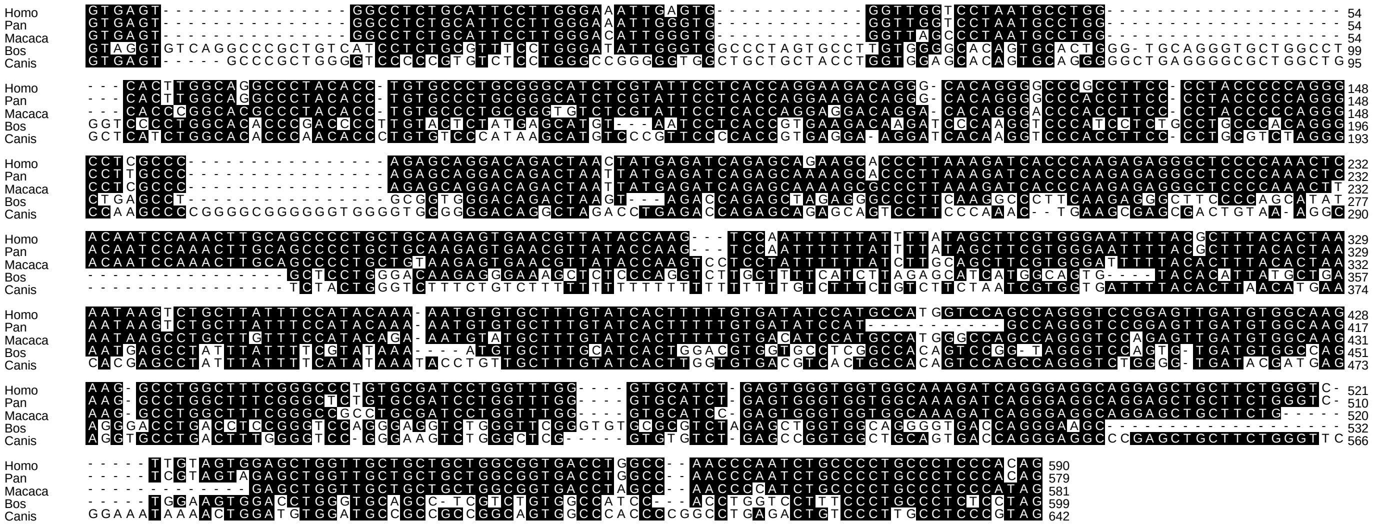

ENSG00000011021 intron 18

Description: Chloride channel protein 6 (CLCN6)
Intron number: 18
Human chromosome: 01
Intron start (bp): 11818798
Human intron length : 845
Intron alignment length: 1053
Flanking exons length (upstream/downstream): 187/158
SNP density: 0.005917
K tree score: 0.0525
Scaling factor: 0.6044
Human-chimpanzee distance: 0.013142
Total primate branch length: 0.0769

ENSG00000011021 exon 18

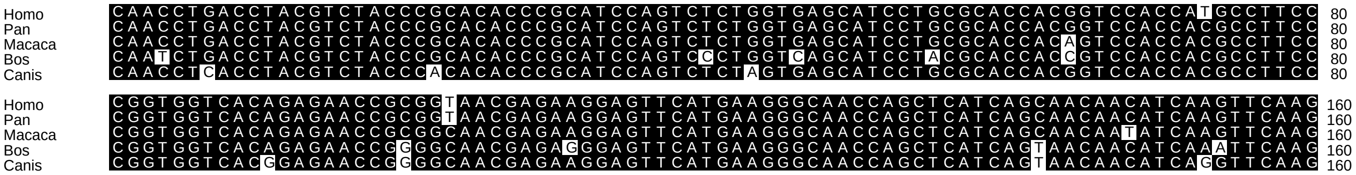

ENSG00000011021 exon 19

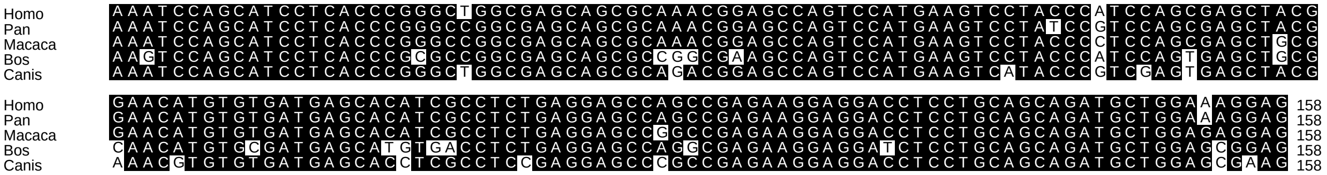

ENSG00000011021 intron 18

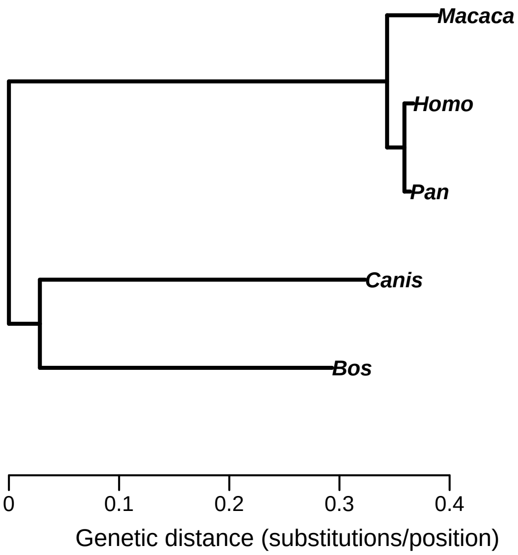

ENSG00000011021 intron 18

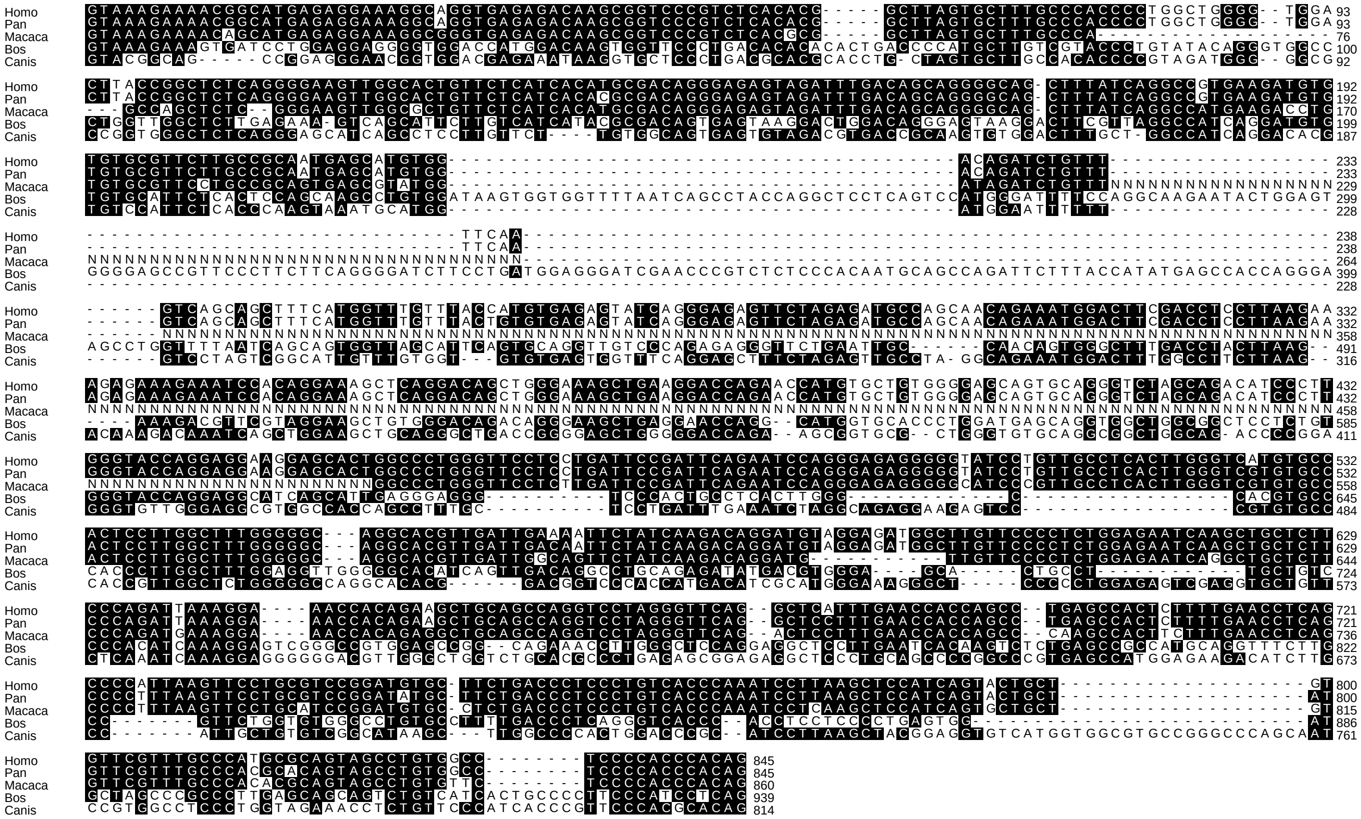

ENSG00000115267 intron 7

Description: Interferon-induced helicase C domain-containing protein 1 (IFIH1)
Intron number: 7
Human chromosome: 02
Intron start (bp): 162844869
Human intron length : 1215
Intron alignment length: 1303
Flanking exons length (upstream/downstream): 218/117
SNP density: 0.011523
K tree score: 0.0546
Scaling factor: 1.1409
Human-chimpanzee distance: 0.018833
Total primate branch length: 0.0769

ENSG00000115267 exon 7

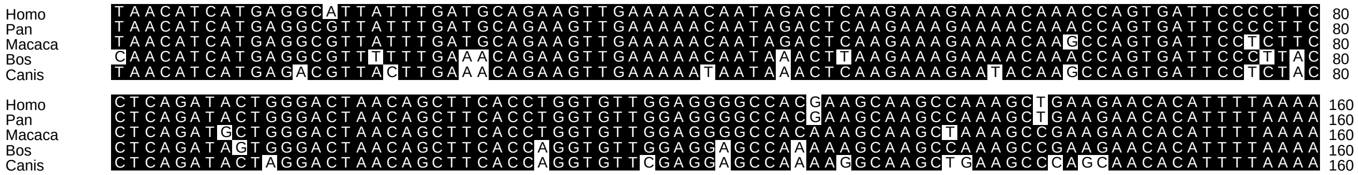

ENSG00000115267 exon 8

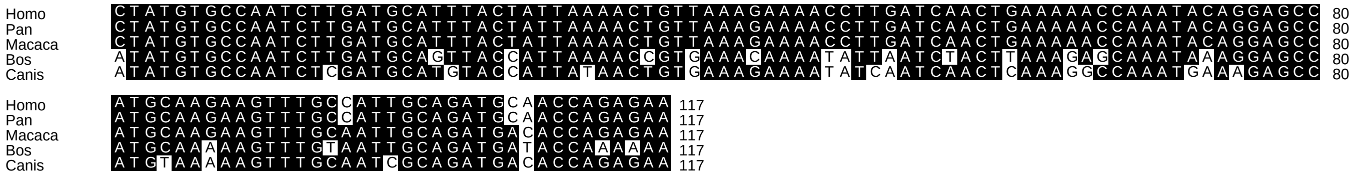

ENSG00000115267 intron 7

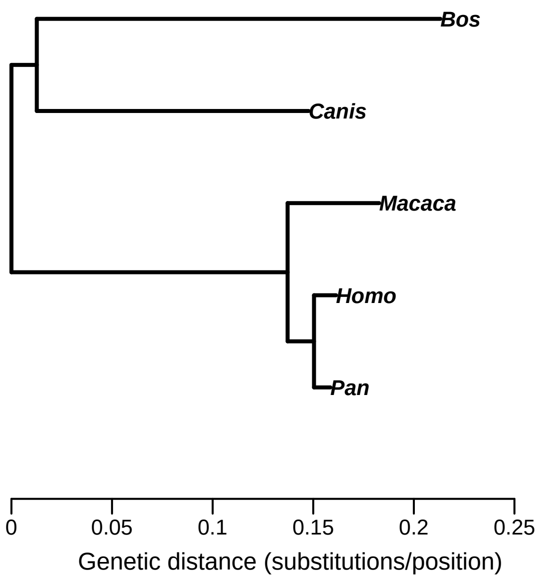

ENSG00000115267 intron 7

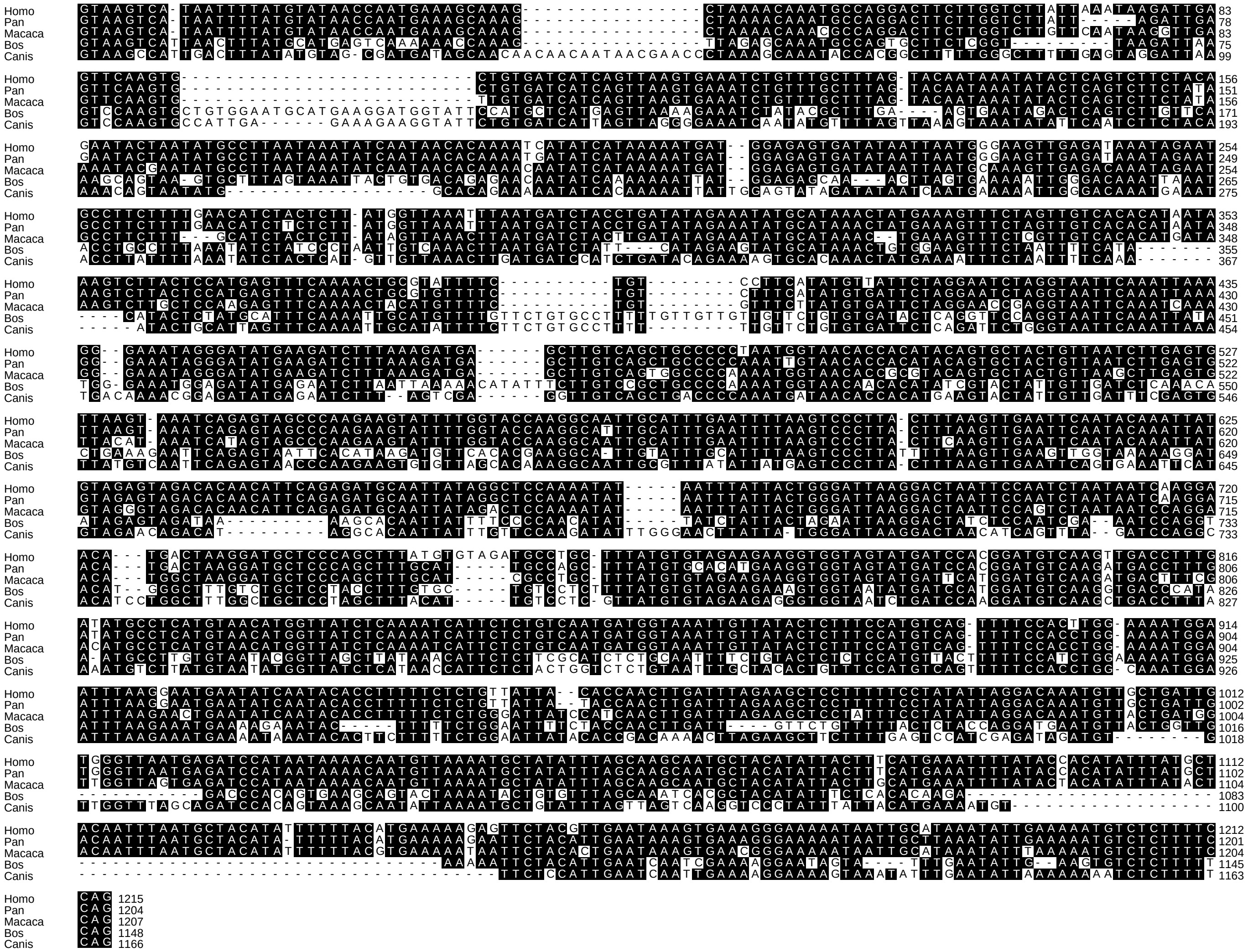

ENSG00000160917 intron 6

Description: Cleavage and polyadenylation specificity factor 30 kDa subunit (CPSF4)
Intron number: 6
Human chromosome: 07
Intron start (bp): 98888000
Human intron length : 1525
Intron alignment length: 1752
Flanking exons length (upstream/downstream): 73/171
SNP density: 0.000000
K tree score: 0.076
Scaling factor: 0.9453
Human-chimpanzee distance: 0.009354
Total primate branch length: 0.0768

ENSG00000160917 exon 6

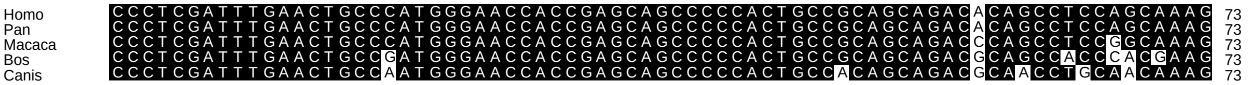

ENSG00000160917 exon 7

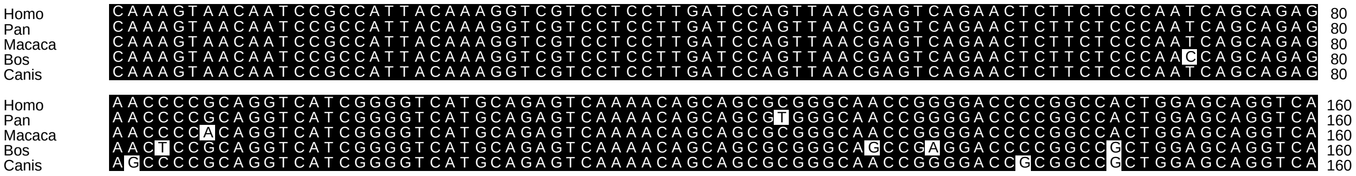

ENSG00000160917 intron 6

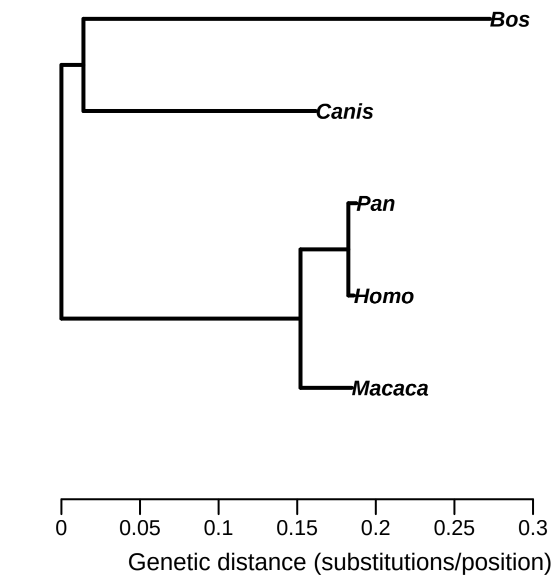

ENSG00000160917 intron 6

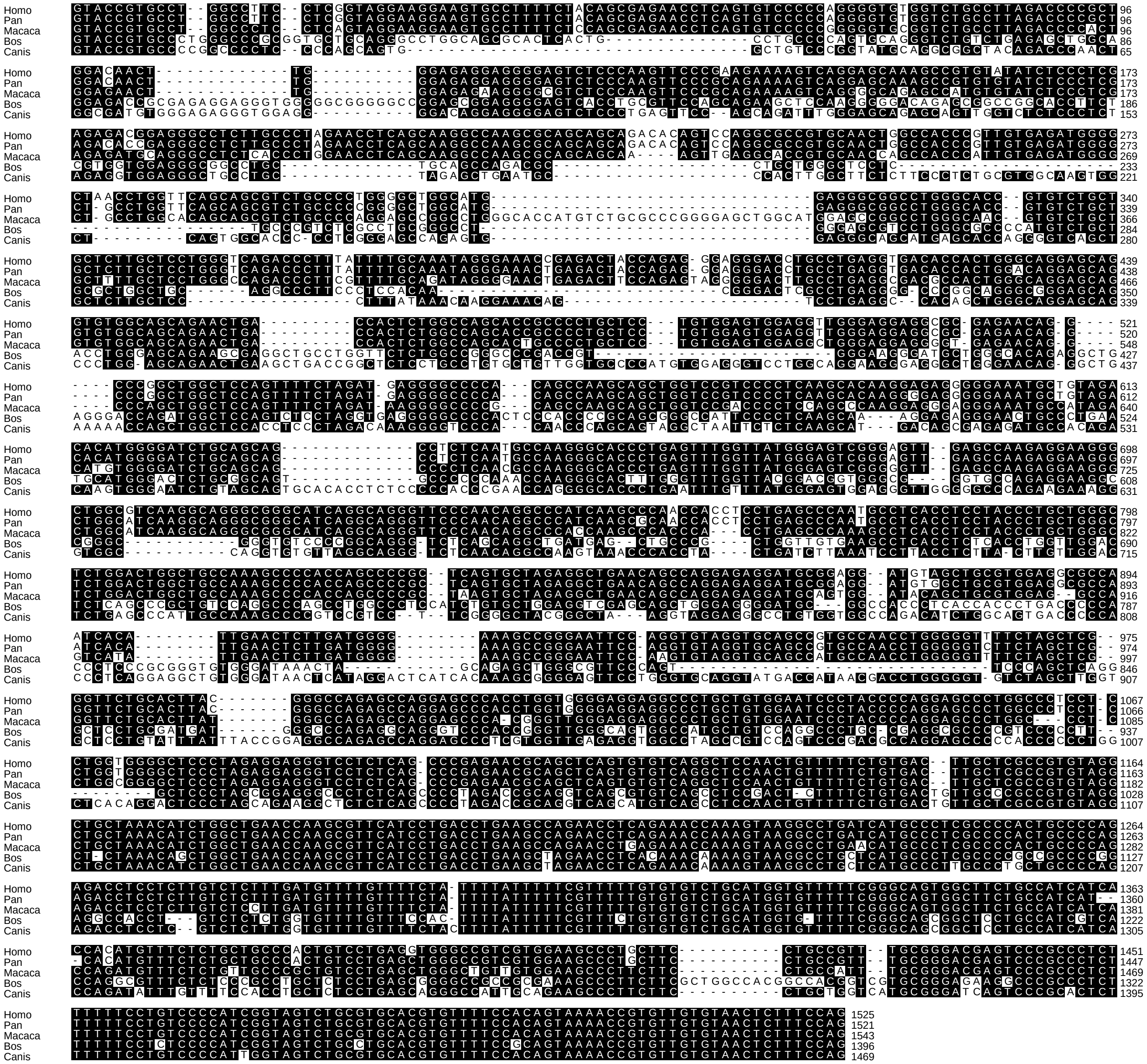

ENSG00000081791 intron 5

Description: KIAA0141 (KIAA0141)
Intron number: 5
Human chromosome: 05
Intron start (bp): 141289490
Human intron length : 248
Intron alignment length: 275
Flanking exons length (upstream/downstream): 159/86
SNP density: 0.008065
K tree score: 0.0892
Scaling factor: 0.9469
Human-chimpanzee distance: 0.016359
Total primate branch length: 0.0768

ENSG00000081791 exon 5

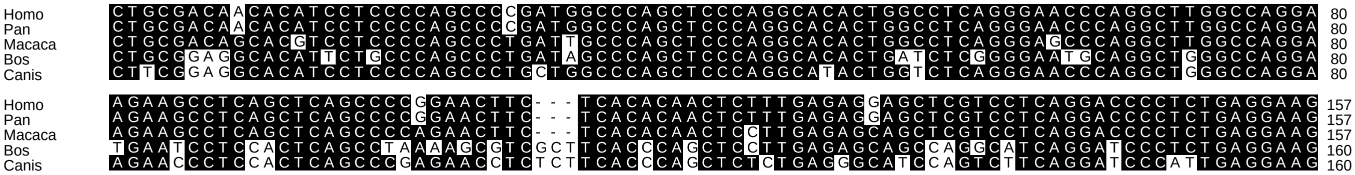

ENSG00000081791 exon 6

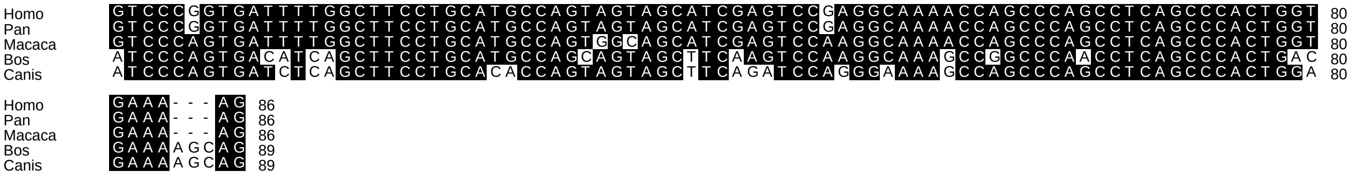

ENSG00000081791 intron 5

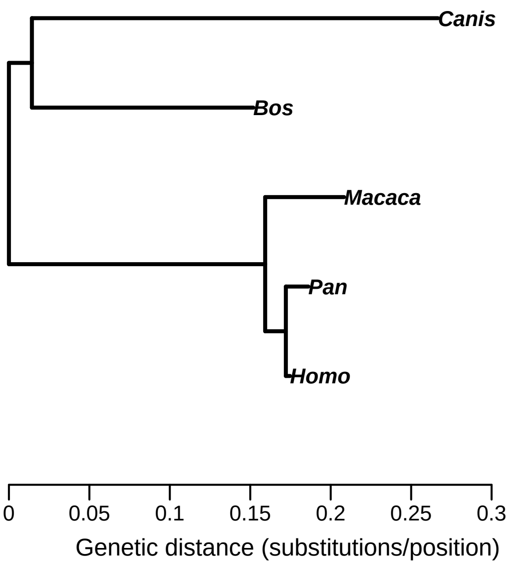

ENSG00000081791 intron 5

```
Homo    GTATGTCCCTGCCTCATGGAATCAGCAGA------------AGGCAGGGGGCGGTGGTGGTGAGCTGGGCAAGATGGACGTTGTTTGCTGCG-------- 80
Pan     GTATGTCCCTGCCTCATGGAATCAGCAGA------------AGGCAGGGGGCGGTGGTGGCGAGCTGGGCAAGATGGACGTTGTTTGCTGCG-------- 80
Macaca  GTATGTCCCTGCCTCATGGAATCAGCAGA------------AGGCAGGGGGTGGTGGTGGCAAACTGGGC-AGAGGGATGTTGGTTGCTGTG-------- 79
Bos     GTATGGCCCCAGCTACTAAAAGCAGAAGATCTGTGGTGGGGTGGGGTGGGGTGGGGGTGGGGAATTGGGC-AGATGGACTTTTGTTGCTGCATATGCCGG 99
Canis   GTATGTTTCTACCTTCTAGAATCAGCAAA----------------AGGTAGAGGGGGTGGGTAATTGGAC-AGATGGACATTTGTTGCTATGTATTCTGG 83

Homo    --AAGGGAATTGGCACTCCTGTGAGGTCCCATGCCTTGTGAGGTGATTCAGGGTTATTTCGGTTCCTGGCAAGGGTGCTGGCTGGAAAGCCTGGGTAGAC 178
Pan     --AAGGGAATTGGCACTCCTGTGAGGTCCCATGCCTTGTGAGGTGATTCAGGGTTATTTCGGTTCCTGGCAAGGGTGCTGGCTGGAAAGCCTGGATAGAC 178
Macaca  --AAGGGAATTGGCATTCCTGTGAGGTCCCATGCCTTGTGAGGTGATTCAGGGTTATTTCGGTTCCTGGTGAGGGTGCTGGATGGAAAGCCTGGGTAGAT 177
Bos     CTAAGGGAATTGTCATTCTTGTGAGTCCTCATGCTTTCTGGGGCAATTTGGAATTATTTTGGTTCTTGGCGGTGGTGCTGGCTGGAAGG-CTGGGGGCAC 198
Canis   ATAAGGTAATTGTCATTATTGCAGGGCCCC-TGCTTTCTGG--------GGAATGATTTTGTTTCCTGGCAGTGGTGCTGCTGGAGGGG-CTAGTGAGAC 173

Homo    TGGGAGAGTCGGAGAAAAGAAG----TACAACTTTCTCCTTTGCCCTGGCCGGGTGTCGGCC-TTTCCCTTGCAG 248
Pan     TGGGAGAGTTGGAGAAAAGAAG----TACAACTTTCTCCTTTGCCCTGGCCGGGTGTCGGCC-TTTGCCTTGCAG 248
Macaca  TGGAAGAGTCGGAGAAAAGAAG----TACAACTTTCTCCTTTGCCCTGGCCAGGTGTCGGCC-TTTCCCTTGCAG 247
Bos     TGGGAGAGTGGGAGAAAA-AAGAAATAACACCTTCCTCCTTTGCCC-----------------TTTCCCTCGCAG 255
Canis   TGGGATGGTGGGAGAAAAGGAGAAACCACATTCTTTCTCCTTGCCCTGACTGGGTGTCACCCATTTTCCTTGCAG 248
```

ENSG00000122188 intron 3

Description: Lymphocyte transmembrane adapter 1 (LAX1)
Intron number: 3
Human chromosome: 01
Intron start (bp): 202007229
Human intron length : 590
Intron alignment length: 936
Flanking exons length (upstream/downstream): 111/80
SNP density: 0.006780
K tree score: 0.0911
Scaling factor: 0.6989
Human-chimpanzee distance: 0.010369
Total primate branch length: 0.0764

ENSG00000122188 exon 3

```
Homo    GACAAGTTCCTTACCTCCGAGTTACCGTCATGCCCTTGCTGACTTTGCCACAAACCAGACAAAGAGCCAAAAATATTTAT 80
Pan     GACAAGTTCCTTACCTCCGAGTTACCGTCATGCCCTTGCTGACTTTGCCACAAACCAGACAAAGAGCCAAAAATATTTAT 80
Macaca  GGCAAGTTCCTTACCTCCGAGTTACCGTCATGCCCTTGCTGACTCTGCCACAAAACAGACAAAGAGCCAAAAATATTTAT 80
Bos     GGCAAGTTCCCTACCTCCGAGTTACCATCATGCCCTTGTTGACTCTGCCTCGGCCCAGACAGCGAGCCAAAAATATTTAT 80
Canis   GACACATTCCTTACTTCCGAGTTACCATCACGCCCTTACTTTCTCTGTCTCGACCCAGACAAGGAGCCAAAAATATTTAT 80

Homo    GACATCTTGCCTTGGCGACAGGAAGACCTGG 111
Pan     GACATCTTGCCTTGGCGACAGGAAGACCTGG 111
Macaca  GACATCTTGCCTTGGCGACAGGAAGACCTGG 111
Bos     GACCTCTTGCCCCGAAGACAAGAAGAGCTGG 111
Canis   GACTCCTTACCCCAGAGGCAAGGAGAGCTGG 111
```

ENSG00000122188 exon 4

```
Homo    GGAGACATGAGTCGAGGAGTATGCGCATTTTCAGTACTGAGAGCCTCCTCTCCAGAAATTCTGAGAGCC------CGGAG 74
Pan     GGAGACATGAGTCAAGGAGTATGCGCATTTTCAGTACTGAGAGCCTCCTCTCCAGAAATTCTGAGAGCC------CAGAG 74
Macaca  GGAGACATGAGTCAAGGAGTATGCGCATTTTCAGTACTGAGAGCCTCCTCTCCAGAAATTCTGAGAGCC------CTGAG 74
Bos     GAAGACATCCCTCAAGGAGCATCCGTATTGTCAGCACGGAAAGCCTCCTCTCCAGGAATTCTGACAGCCCTTCCTCTGAG 80
Canis   GGAGACGGCAGTCAAGGAGTAACCATATTTTCAGTACCGAGAGCCTCCTTTCCAGAAATTATGATAGCCCTCCTTCTGAT 80

Homo    CATGTG 80
Pan     CATGTG 80
Macaca  CATGTG 80
Bos     CATGTG 86
Canis   CCTGTG 86
```

ENSG00000122188 intron 3

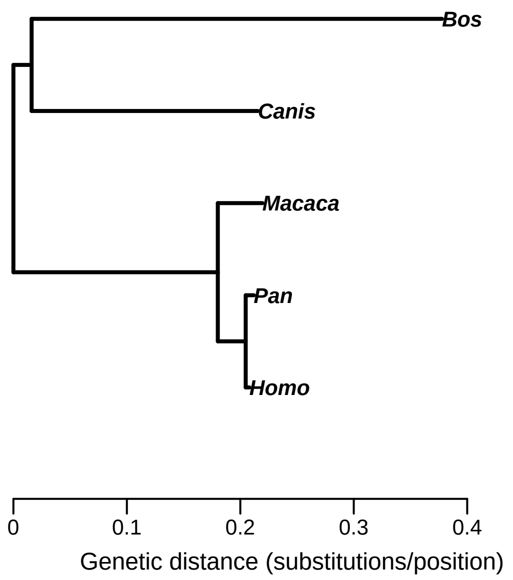

ENSG00000122188 intron 3

```
Homo    GTAGGTTTTTCCTTCTAATCTATGGAGTCCAGATATT--CGAACTTCTACTCCCAGAATGTGCCTCTCA------------------------------- 67
Pan     GTAGGTTTTTCCTTCTAATCTATGGAGTCCAGATATT--CGAACTTCTACTCCCAGAATGTGCCTCTCA------------------------------- 67
Macaca  GTAGGTTTTTCCTTCTAATCTATGGAGTCCAGATGTT--CAAACTTCCACTCCCAGAATGTGCCTCTCA------------------------------- 67
Bos     GTAGGTTTTTCTTTCTCATCCATGGAATCCAGGC-CTACAGAACTTCTGCCCCCAGAATATAGGTCTCA------------------------------- 68
Canis   GTAGGTTTTTCTTTCTAATCTGCAGAGCACAAATGTTACTGAACTT---CATCCAGAATGCACCTCTCATACCCAGAAGCACCTTTTTTCTTTTTTTAAG 97

Homo    ---------------------------------------------------------------------------------------------------- 67
Pan     ---------------------------------------------------------------------------------------------------- 67
Macaca  ---------------------------------------------------------------------------------------------------- 67
Bos     ---------------------------------------------------------------------------------------------------- 68
Canis   ATTTTATTTGTTTATTCATGAGAGACACAGAGCAAGAGAGAGAAAGGCAGAGGCACAGGCAGAGGGAGGAGCAGGCTCCATGCAGGGAGCCTGACGTGGG 197

Homo    --------------------------------------------------------------------------------------------CCCTCCAG 75
Pan     --------------------------------------------------------------------------------------------CCCTCCAG 75
Macaca  --------------------------------------------------------------------------------------------CCCTCCAG 75
Bos     ---------------------------------------------------------------------------------------------CCTGCAA 75
Canis   ACTCGATCCTGGATCTCCAGGATCACGCCCTGGGCTGAAGGCTGCACTAAACTGCTCAGCTGCCCCAGAAGCAACTTCTTCTTTTTTTTTTTTTTTTCAG 297

Homo    AAGCAATTTTCTTTAAGTTCTCAAACCTGTTTGCCCTTCAGAGAGTATCAGGGAGAATGGGATAGATAGACTGTCAGCTGCAACTCCA-GGGGGGTCTGG 174
Pan     AAGCAATTTTCTTTAAGTTCTCAAACCTGTTTGCCCTTCAGAGAGTATCAGGAAAAATGGGATAGAGAGACTGTCAGCTGCAACTCCA-GGGGGGTCTGG 174
Macaca  AAGCAACTTTCTCTAAGTTCTCAAACCTGTTTGCCCTTCAGAGAGTATCAGGGAGAATGGGATAGACAGACCGTCAGCTGCAACTCCG-GGGGAACCTGG 174
Bos     AAGCGGCTTTTTCTAAGCATGC-TACCATCCTGCTCTTCAGGGAGCAGGCGAGAGGACGGGACAGACAGACGGCAAGGTTTACCTCCA-GTGGGGCCTGG 173
Canis   AAGCAACTTTCTTTACATTTTCAGACCTTTCTGCCCTTCAGGGAGCGTGGAGGAGGACGGGATAGTCAGA-AGCAGGCTGTAACTCCATTAGGGGCCTAC 396

Homo    AGGGCT-CCAAAAAGGGAGCAGAGGGTCATCTGGGCTGGGAGTTT------------------------------------------------------- 218
Pan     AGGGCT-CCAAAAAGGGAGCAGAGGGTCATCTGGGCTGGGAGTTT------------------------------------------------------- 218
Macaca  AGGGCT-CCAAAGAGGGAGCAGAGGGTCATCTGGGCTGGGAGTTT------------------------------------------------------- 218
Bos     GGGCTT---GAAGAAGGAGCAGGAAGTCATCAGGGTCAGGGATCTGGTTTGGGGTTTTCTCCAACTTCATTGAGAAATGATTGACATATGTCACTGTATG 270
Canis   AGAGCTAAAAAAAAGGGAGCTGGGAGGTGTCTGGGTTGGGTTTTT------------------------------------------------------- 441

Homo    -------------------------------------------TGT--TTTTTCTTAATGTTAAGGCAACAGAAGAAAGCCAAGTGTGTCCAGGCATAGG 273
Pan     -------------------------------------------TGT--TTTTTCTTAATGTTAAGGCAACAGAAGAAAGCCAAGTGTGTCCAGGCATGGG 273
Macaca  -------------------------------------------TGT--TTTTTCTTAATATTAAGGCAACAGAAGAAAGCCAAGTGTGTCCAGGCATGGG 273
Bos     AGTTTACAGAGTACAGCATGATAATTTGATTACATAGACTAAATTTGGTTTTTCTTGATGTAG------------TTAGCTGGGTATGTCTAAAGTTGGG 358
Canis   -------------------------------------------TTTTATTTGTCCTGATATGAAGTC--------CTGGATGGGTATGTCCAGGAATGGG 490

Homo    GAAGACACCAAGGAAGAGAACTCGGTGCCAGCATCACAGGCAGAGGATGGGGCAGGGAGGAGGTTCTCACACAGGAGCTTGTTTCTCAGCC-TCCCAACC 372
Pan     GAAGACACCAAGGAAGAGAACTCGGTGCCAGCATCACAGGCAGAGGATGGGGTGGGGAGGAGGTTCTCACACAGGAGCTTGTTTCTCAGCC-TCCCAACC 372
Macaca  G-AGACACAAAGGAGGAGAACTCAGTGCCAGCATCACAGGCAGAGGA-GGGGCGGGGAGGAGGTTCTCCCACAGGACCGTGTTTCTCAGCC-TCCCAATC 370
Bos     AAAGACACACAG---GAGAACATGG--------CCTCCAGCC----TCAAGTCAGGGAGGGCTCACCCGAGAAGACTCCGCCCAGCCAGCCTCCTCCACT 443
Canis   GGACACATACAGAAGAAGAGCATGG--------CCTCTGGCA---------------------------TTACAGGAGCCTCCTCCCAGCTGCTCTAAAC 556

Homo    CCTGTG----ATAGCATCCTCCAGTGCC--ACAGAAGCTGAGGG-GCTTCCGGTTCTCAGAGTG---TCATCCCCATAACTCTGACCTCAGAACATGAGA 462
Pan     CCTGTG----ATAGCATCCTCCAGTGCC--ACAGAAGCTGAGGG-GCTTCCGGTTCTCAGAGTG---TCATCCCCATAACTCTGACCTCAGAACATGAGA 462
Macaca  CCCGTG----ATAGCATCCGCCAATGCC--ACAGAAGCTGAGGG-GCTTCCGGTTCTCAGAGTG---TCATCCCCATAGCTCTGACCTCAGAACACAGGA 460
Bos     CTTGTG----TTAGTGTCCATTTGTCCT--CAGCGAGCTGAGGAGTGTTCCTGGTCTCCCAGCTCA-TCATCCTTGAAGCCCTGACCCT--CTCCCCACA 534
Canis   TCTGTGCTAATTAGTGTCCTCTGGTCCTTGTTAAAACAGAGGACAGTCCAGGCCTCAGAGCACATCATTCTCCCTAGCTCTGCCCCT--CTCACCACA 654

Homo    GAACACCAGACCCAAGGCTGGAAATTCCAGACACCACGCAAACCCGCTTCATCCATGATGCAGGGATTATTTGGACTGAAGCACTCAGAGGA----CTGG 558
Pan     GAACACCAGACCCAAGGCTGGAAATTCCAGACACCACGCAAACCCGCTTCATCCATGATGCAGGGATTATTTGGACTGAAGCACTCAGAGGA----CTGG 558
Macaca  GAACACAGGACCCAAGGCTGGAAATTCCAGACACCATACAAACCCAGTTCATCCATGATGCAGGGATTATTTGGGCTGAAGCACTCAGAGGA----CTGG 556
Bos     GAACACTGGCCCCCAA-------ATACCAGGTATCAGACAGGTCCACTTCACCCAGAATGTGAGGACTGAACAGGCTGAAGCACTTGGAGGACTGGCTGA 627
Canis   GCACACCGGCCTCAAA---------TACCGACACCACACAAACCCACATCAACCACGATGCGGGGACCATGGAGGTGGAAGCGCTCAGGGGACTGGCTGG 745

Homo    CTATCTTCAGGATTTGTTCTCTGTCCTTTCAG 590
Pan     CTATCTTCAGGATTTGTTCTCTGTCCTTTCAG 590
Macaca  CTATCTTCAGAATTTGTTCTCTGTCCTTTCAG 588
Bos     CTATGCTCAGGGTTTGCTCTCTGACCTTTCAG 659
Canis   CTATCTTCAGGATTTGTTCTCTGTCCTTTCAG 777
```

ENSG00000186815 intron 24

Description: two pore segment channel 1 (TPCN1)
Intron number: 24
Human chromosome: 12
Intron start (bp): 112214147
Human intron length : 975
Intron alignment length: 1020
Flanking exons length (upstream/downstream): 85/140
SNP density: 0.005128
K tree score: 0.0762
Scaling factor: 0.693
Human-chimpanzee distance: 0.013543
Total primate branch length: 0.0764

ENSG00000186815 exon 24

```
Homo    GTGGTGATGACGATCATTGTCGCCTTTATCCTCGAGGCCTTCGTCTTCCGAATGAACTACAGCCGCAAGAACCAGGACTC 80
Pan     GTGGTGATGACGATCATTGTCGCCTTTATCCTCGAGGCCTTCGTCTTCCGAATGAACTACAGCCGCAAGAACCAGGACTC 80
Macaca  GTGGTGATGACGATCATTGTCGCCTTCATCCTCGAGGCCTTCGTCTTCCGAATGAACTACAGCCGCAAGAACCAGGACTC 80
Bos     GTGGTGATGACCATCATTGTGGCCTTCATCCTCGAAGCCTTTGTCTTCCGAATGAACTACAGCCGCAAGAACCAGGACTC 80
Canis   GTGGTGATGACTATCATTGTCGCCTTCATCCTTGAAGCCTTCGTCTTCCGAATGAACTACAGCCGCAAGAACCAGGACTC 80

Homo    GGAAG 85
Pan     GGAAG 85
Macaca  GGAAG 85
Bos     GGAAG 85
Canis   GGAAG 85
```

ENSG00000186815 exon 25

```
Homo    TTGATGGTGGCATCACCCTTGAGAAGGAAATCTCCAAAGAAGAGCTGGTTGCCGTCCTGGAGCTCTACCGGGAGGCACGG 80
Pan     TTGATGGTGGCATCACCCTTGAGAAGGAAATCTCCAAAGAAGAGCTGGTTGCCGTCCTGGAGCTCTACCGGGAGGCACGG 80
Macaca  TTGATGGTGGCATCACCCTTGAGAAGGAAATCTCCAAAGAAGAGCTGGTTGCTGTCCTGGAGCTCTACCGGGAGGCACAG 80
Bos     TGGACGACGGCATCACCCTGGAGAAGGAGCTCTCCAAAGACGAGCTGGTCGCAGTTCTGAAGCTCTACCGGGAGGCAGGG 80
Canis   TTGATGGTGGCATCACCCTTGAGAAGGAAATCTCCAAAGATGAGCTGCTTGCTGTCCTGGAGCTCTACCGGGAGGTGCGA 80

Homo    GGGGCCTCCTCGGATGTCACCAGGCTGCTGGAGACCCTCTCCCAGATGGAGAGATACCAG 140
Pan     GGGGCCTCCTCGGATGTCACCCGGCTGCTGGAGACCCTCTCCCAGATGGAGAGATACCAG 140
Macaca  GGGGCCTCCTCGGACGTCACCCGGCTGCTGGAGACCCTCTCCCAGATGGAGAGATACCAG 140
Bos     GGAGCCAACTCAGACATTGCCCAGCTGCTCAAGATCCTCTCCCAGCTGGAGAGATACGAG 140
Canis   GGAGCCACCTCGGATATCACCCGGCTGCTCACAGTCCTCTCTCAGATGGAGAAACACGAG 140
```

ENSG00000186815 intron 24

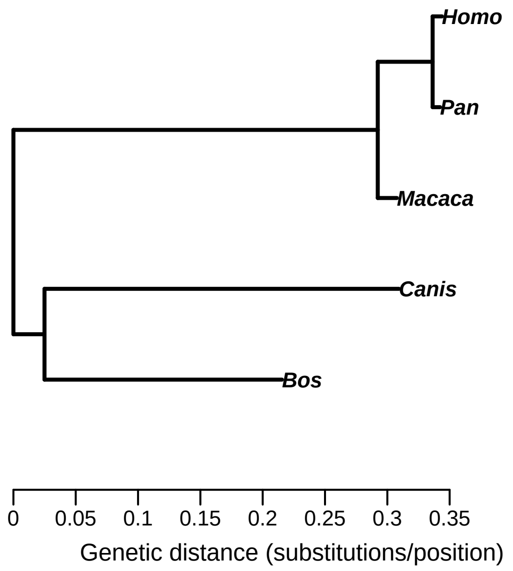

ENSG00000186815 intron 24

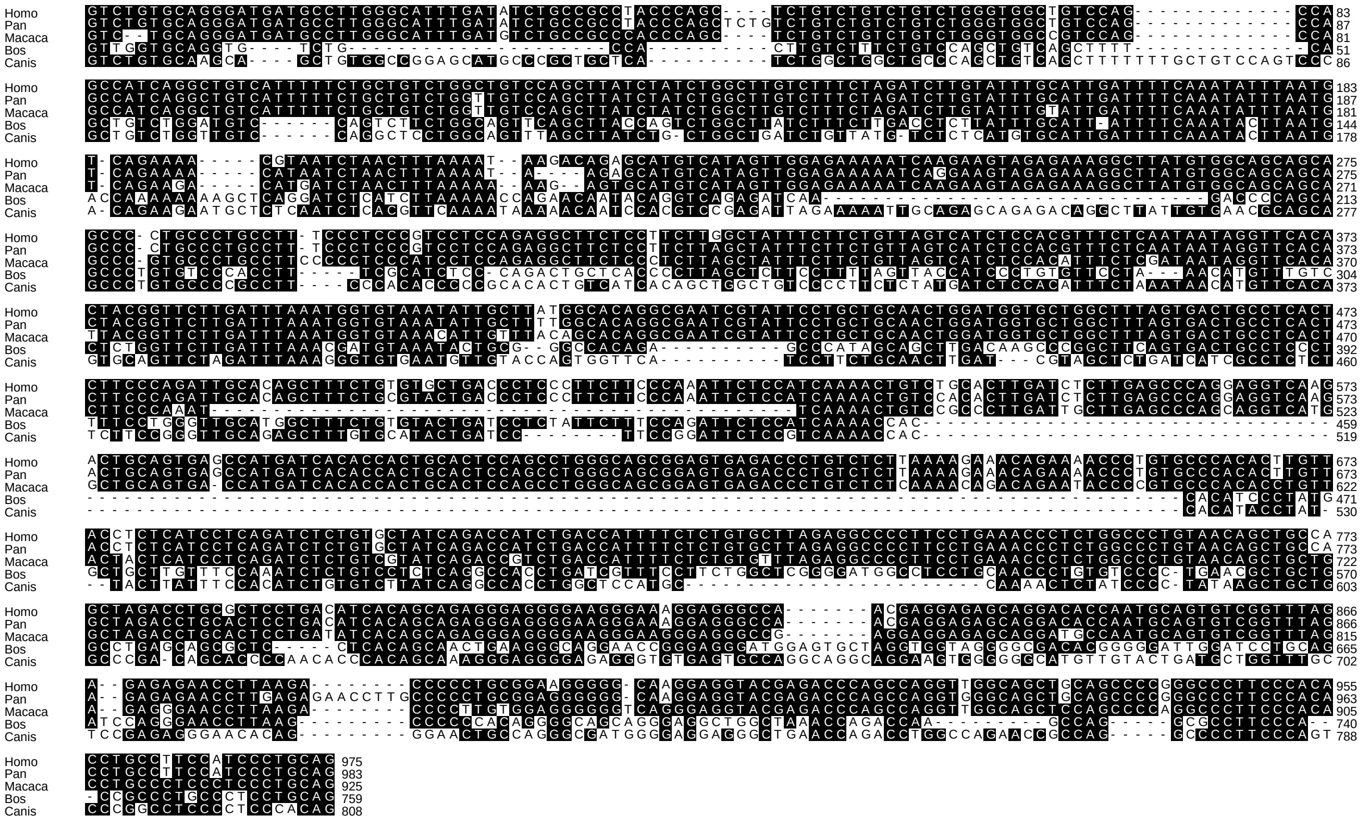

ENSG00000106333 intron 2

Description: Procollagen C-endopeptidase enhancer 1 precursor (PCOLCE)
Intron number: 2
Human chromosome: 07
Intron start (bp): 100039098
Human intron length : 420
Intron alignment length: 461
Flanking exons length (upstream/downstream): 109/259
SNP density: 0.000000
K tree score: 0.0595
Scaling factor: 0.7781
Human-chimpanzee distance: 0.017239
Total primate branch length: 0.0763

ENSG00000106333 exon 2

```
Homo    ACCCGTGTTCCTGTGCGGAGGGGATGTGAAGGGGGAATCAGGTTACGTGGCAAGTGAGGGGTTCCCCAACCTCTACCCCC 80
Pan     ACCCGTGTTCCTGTGCGGAGGGGATGTGAAGGGGGAATCAGGTTACGTGGCAAGTGAGGGGTTCCCCAACCTCTACCCCC 80
Macaca  ACCCGTGTTCCTGTGCGGAGGGGATGTGAAGGGGGAATCAGGTTACGTGGCAAGTGAGGGGTTCCCCAACCTCTACCCCC 80
Bos     ACCCGTGTTCCTGTGCGGAGGGGATGTGACTGGGGAGTCGGGTTACGTGGCAAGTGAGGGTTTCCCCAACCTCTACCCTC 80
Canis   ACCTGTGTTCCTGTGTGGAGGGGATGTGACCGGGGAGTCAGGTTACGTGGCAAGTGAGGGTTTCCCCAACCTGTACCCCC 80

Homo    CTAATAAGGAGTGCATCTGGACCATAACG 109
Pan     CTAATAAGGAGTGCATCTGGACCATAACG 109
Macaca  CTAATAAGGAGTGCATCTGGACCATAACG 109
Bos     CCAATAAGGAGTGCATCTGGACAATAACG 109
Canis   CCAATAAGGAGTGCATCTGGACAATAACG 109
```

ENSG00000106333 exon 3

```
Homo    GTCCCCGAGGGCCAGACTGTGTCCCTCTCATTCCGAGTCTTCGACCTGGAGCTGCACCCCGCCTGCCGCTACGATGCTCT 80
Pan     GTCCCCGAGGGCCAGACTGTGTCCCTCTCATTCCGAGTCTTCGACCTGGAGCTGCACCCCGCCTGCCGCTACGATGCTCT 80
Macaca  GTCCCCGAGGGTCAGACTGTGTCCCTCTCATTCCGAGTCTTCGACCTGGAGCTGCACCCCGCCTGCCGCTACGACGCACT 80
Bos     GTCCCTAAGAGCCAGACTGTGTCCCTCTCCTTCCGAGTCTTTGACCTGGAGCAGCACCCCAGCTGCCGGTACGATGCTCT 80
Canis   GTCCCTGAGGGCCAGACTGTGTCCCTTTCCTTCCGAGTCTTTGATTTGGAGCTGCACCCCGCCTGCCGTTATGATGCTCT 80

Homo    GGAGGTCTTCGCTGGGTCTGGGACTTCCGGCCAGCGGCTCGGACGCTTTTGTGGGACCTTCCGGCCTGCGCCCCTAGTCG 160
Pan     GGAGGTCTTCGCTGGGTCTGGGACTTCCGGCCAGCGGCTCGGACGCTTTTGTGGGACCTTCCGGCCTGCGCCCCTAGTCG 160
Macaca  GGAGGTCTTCGCTGGGTCTGGGACTTCTGGCCAGCGGCTCGGACGCTTTTGCGGGACCTTCCGGCCTGCGCCCCTAGTCG 160
Bos     GGAGATCTTTGCCGGGTCTGGAACCTCGGGCCAGCAACTCGGACGCTTCTGTGGGACCTTCCGACCCGCGCCTGTAGTCG 160
Canis   GGAGGTCTTTGCCGGGTCTGGAACCTCGGGCCAGCGGCTTGGACGCTTCTGCGGGACCTTCCGGCCTGCGCCCTTAGTCG 160
```

ENSG00000106333 intron 2

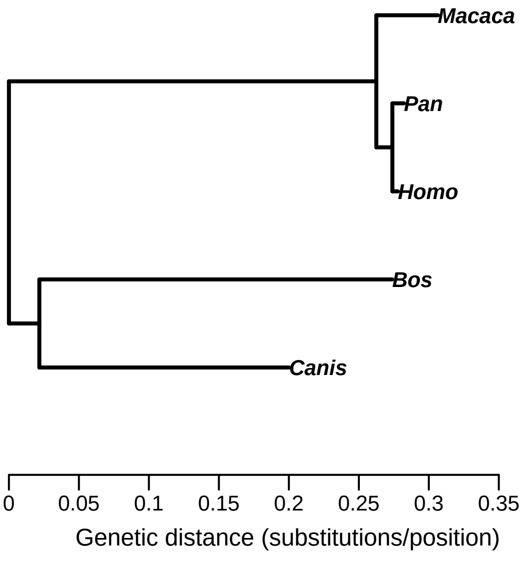

ENSG00000106333 intron 2

```
Homo    GTGAGAAA-CCCCTCTGGGCACTA-CCCCTTGTTAAAGTCTCAGAGGCAAAAAGGCCTGACTGCGAAGGGA-----------CCCCC--------CCCCC 79
Pan     GTGAGAAA-CCCCTCTGGGCACTA-CCCCTTGTTAAAGTCTCAGAGGCAAAAGGGCCTGACTGCGAAGGGA-----------CCCCC--------CCCCC 79
Macaca  GTGAGAAG-CCCCTCTGGGCACTA-CCGCTTGTCAAAGTCTCAGAGGCAAA--GGCCTGACTGCGAAGGGAGCGCCCCCAGGCCCCC--------ACCCC 88
Bos     GTGAGAAGGCCCTTCTGGCCACTCTTCCCTCCTCCAAGTGTCTGA--------GGCCTGGGTCCAGGCAGCCAAAGGCCAGACTGCTAAGGGAATTCCCT 92
Canis   GTGAGAAGTTCCTTCCTATCACTACCCCCTTGTTGTAGTCTCCCA--------GGCCCAACTAAGGGCACC------TCAGTCTGCCGG------CCCCC 80

Homo    GTCCCCCCCGCACCACCTTCTCAACCTGGGGCAGCTCCAAGTGTCTTCCCTCTGTCACTGAAGGCATCATTCCCACCGTAACACGCATC------ATCTT 173
Pan     CCCCCCCCCGCACCACCTTCTCAACCTGGGGCAGCTCCAAGTGTCTTCCCTCTGTCACTGAAGGCATCATTCCCACCGTAACACGCATC------CTCTT 173
Macaca  CAGTCCCCGGCACCACCTTCTCAACCTGGGGCAGCTCCAAGTGTCTTCCCTCTGTCACTAAAGGCATCATTCCCACCGTAACAAGCACC------CTCTT 182
Bos     CCCTGCCAAGTCCCACCATTTCTCCCTGGGGAAGTCCCA------GCCCCCTCCCCTCTG----GTCAGTGCCCACAATACCACGTGGC-AAGTGACCTC 181
Canis   TGCTCCCACCACCTCCCATCCCTGAACCACCAAGCCTTA------TCCCCTCTGTCACTGAGGCAGCAGTGCCCATAATAACACGGGGCAAAGCGTCCTC 174

Homo    AACCCTTCCCTCAAATCTTCATTCTCCA-----------GTCCCCGGCC-CTCCCTCCTCTTCCAAAGCCTCAGCTCTTGTCCCCTGCACAGGCTACCAG 261
Pan     AACCCTTCCCTCAAATCTTCATTCTCCA-----------GTCCCCGGCC-CTCCCTCCTCTTCCAAAGCCTCAGCTCTTGTCCCCTGCACAGCCTACCAG 261
Macaca  AACCCTTCCCTCAAATCCTCATTCTCCA-----------GTCTCCGGCC-CTCCCTCCTCTTCCAAAGCCTCAGCTCTTGTCCCCTGCACAGGCCACCAG 270
Bos     ACACCTTAATGCAGAGCCTGCTTGTCCATTCCCACCTTCATCCACACCCGCTCTGGTCTCTCCACAGACCTCATTTGGGTCTTGGTGCA----------- 270
Canis   ACACCTTACT-TAAGGTCTGCTTCTCCACTTCCAGCTTCATTCCCAGTC-CTCCAATCTCTCCA-GGACCTCAGGTCTTGTCCCGTGCACAAGCCAAAAG 271

Homo    CAGAGCTGCAGAGACCAGGCCCTCTGCTCTGCACTCACATGGAGGCCCCTTCCCATTCAGTCC-GTTTCTGCAAGAAACGCTT-GGGGCAGGGGAGCTGC 359
Pan     CAGAGCTGCAGAGACCAGGCCCTCTGCTCTGCACTCACGTGGAGGCCCCTTCCCATTCGGTCC-GTTTCTGCAAGAAACGCTT-GGGGCAGGGGAGCTGC 359
Macaca  CAGAGCTGCAGAGACCAGGCCCTCTGCCCTACACTGGCATGGGGGCCTCTTCCCGTTCAGTCC-GTTTCTGCAAGAAACGCTT-GGGGCAGGGGAGTTGC 368
Bos     -------------------------GTCATGCACCATCAGACTGGCCCT-----TTTAGGCCCAGTCTCTGTAGGAAACGCTTGGGGGAAAGAGAGGTGC 340
Canis   C--TGCTGCAGAGCCCAGGTCCTCTGCTCTGCACGCTGATGGGGTCCCTTTCTGTTCAGGCCCAGTATCTGTATGAAATGCTT-GGGGAAGGACAGGTAC 368

Homo    CCTCTGGCTGGGTTGTGTGGGGCCTGACTCTGTGGGTCCCCGCCTCTGTCCCCGCTATCAG 420
Pan     CCTCTGGCTGGGTTGTGTGGGGCCTGACTCTGTGGGTCCCCGCCTCTGTCCCCGCTATCAG 420
Macaca  CCTCTGGCTGGGTTGTGTGGGGCCTGACTCTGTGGGTCCCCGCCTCTGTCCCCGCTATCAG 429
Bos     CCTCTGCCT-GGTGGTGTTTGGCCTGACTTTGCAGGACCCCGCCCCTGTCCCCACCATCAG 400
Canis   CCTCTGCCTGGGTTGTGTGTGGCCTGACTTTGCAGGTCCTCTTCCCTGTCCCCGCCTTCAG 429
```

ENSG00000146233 intron 4

Description: Cytochrome P450 39A1 (CYP39A1)
Intron number: 4
Human chromosome: 06
Intron start (bp): 46712179
Human intron length : 1346
Intron alignment length: 1449
Flanking exons length (upstream/downstream): 150/94
SNP density: 0.002972
K tree score: 0.097
Scaling factor: 0.8819
Human-chimpanzee distance: 0.008997
Total primate branch length: 0.0762

ENSG00000146233 exon 4

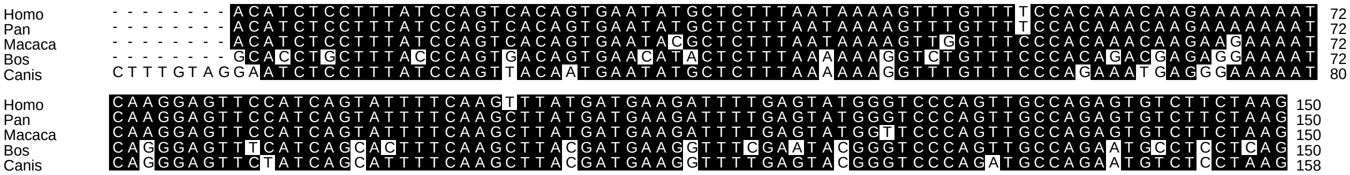

ENSG00000146233 exon 5

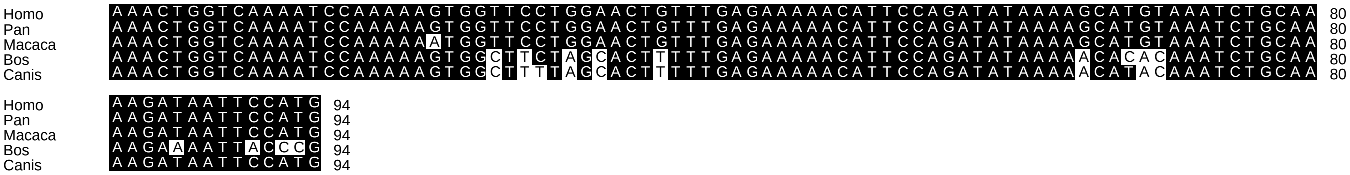

ENSG00000146233 intron 4

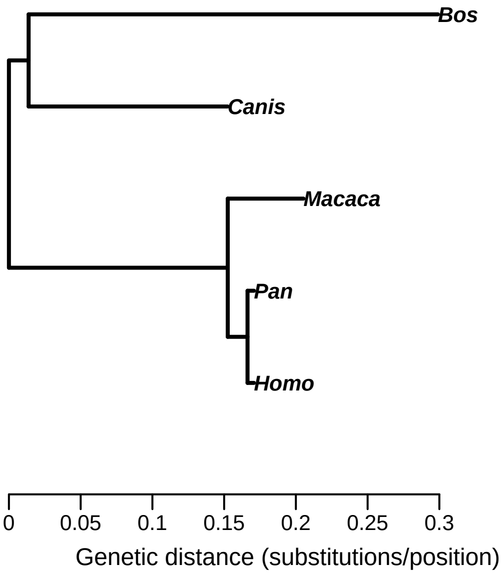

ENSG00000146233 intron 4

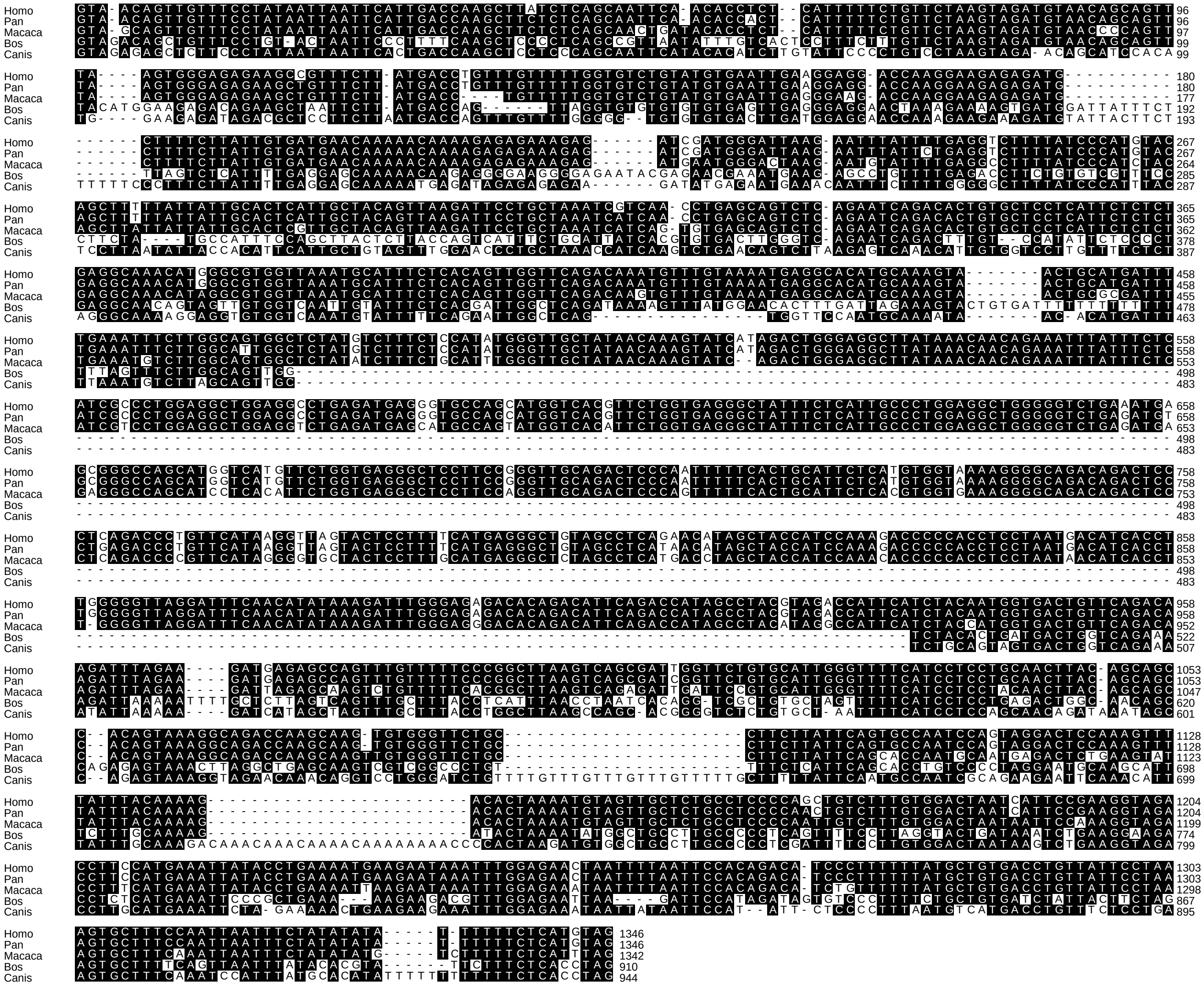

ENSG00000103978 intron 16

Description: transmembrane protein 87A (TMEM87A)
Intron number: 16
Human chromosome: 15
Intron start (bp): 40299133
Human intron length : 419
Intron alignment length: 499
Flanking exons length (upstream/downstream): 74/62
SNP density: 0.000000
K tree score: 0.0664
Scaling factor: 1.0461
Human-chimpanzee distance: 0.004829
Total primate branch length: 0.0761

ENSG00000103978 exon 16

```
Homo    GTTTGCCTTTTCACCATTGTCTGAGGAAGAGGAGGAGGATGAACAAAAGGAGCCTATGCTGAAAGAAAGCTTTG 74
Pan     GTTTGCCTTTTCACCATTGTCTGAGGAAGAGGAGGAGGATGAACAAAAGGAGCCTATGCTGAAAGAAAGCTTTG 74
Macaca  GTTTGCCTTTTCACCATTGTCTGAGGAAGAGGAGGAAGATGAACAAAAGGAACCTATGCTGAAAGAAAGCTTTG 74
Bos     GTTTGCCTTTTCACCATTGTCTGAGGAAGAGGAGGAGGATGAACAAAAGGAACCTATGCTGAAAGAAAGCTTTG 74
Canis   ATTTGCCTTTTCACCATTGTCTGAGGAAGAGGAAGAGGATGAACAAAAAGAACCTATGCTAAAAGAAAGCTTTG 74
```

ENSG00000103978 exon 17

```
Homo    AAGGAATGAAAATGAGAAGTACCAAACAAGAACCCAATGGAAATAGTAAAGTTAACAAAGCA 62
Pan     AAGGAATGAAAATGAGAAGTACCAAACAAGAACCCAATGGAAATAGTAAAGTCAACAAAGCA 62
Macaca  AGGGAATGAAAATGAGAAGTACCAAACAAGAACCCAATGGAAATAGTAAAGTTAACAAAGCA 62
Bos     AAGGGATGAAAATGAGAAGTACCAAACAAGAACCAAATGGAAATAGTAAAGTAAATAAAGCA 62
Canis   AAGGAATGAAAATGAGAAGTACTAAACAAGAACCAAATGGAAATAGTAAAGTAAACAAAGCA 62
```

ENSG00000103978 intron 16

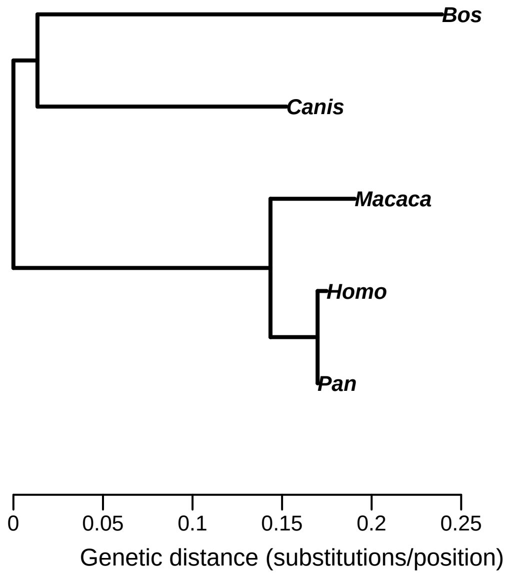

ENSG00000103978 intron 16

```
Homo    GTAATAATAAAATTCACCTCTTATTAGTACTTTAAATATTATTTTT--------------------------------C--TGTAAGT------------ 54
Pan     GTAATAATAAAATTCACCTCTTATTAGTACTTTAAATATTATTTTT--------------------------------C--TGTAAGT------------ 54
Macaca  GTAGTCATAAAATTCACGTCTCATTAGTACTTTAAAT-TTTTTTTT--------------------------------C--TATAAGT------------ 53
Bos     GTAGTGATAAAATTCACCTTTTATTTGTGCTTTTAATATTCTTTTCTGGTTTTTAGAAATACAGTAAATGAAGTATAACATTGTGAGTATCAAAATCAAG 100
Canis   GTAGTGTAAAA-------TTTTATTTGTGCTTTAAATATTATTTTCTGGTTTTTAGGAATAGAGTAGAGCAAGTATAAT--TGTGAGTATCCAAATCAAG 91

Homo    --------ACATT-----GTTTATTATTTTATAACTGT------GTTGTTCCTATATTTGGTGTGGGATGAAGTCAGGG--TTAAGAAAATAAGGGTGTG 133
Pan     --------ACATT-----GTTTATTATTTTATAACTGT------GTTGTTCCTATATTTGGTATGGGATGAAGTTAGGG--TTAAGAAAATAAGGGTGTG 133
Macaca  --------ACATT-----GTTTATCATTTTGTAACTAT------GTTGTTCCTATATTTGGTATGGGATGAAGTTAGGG--TAAAGAAAATAAGGGTGTG 132
Bos     AGACTTTGATATAAGTACGTTTACTCTTTTACAACAT-------GTTATTCTTCCGTTGGGTATGTGGTAAAGTTAGAGGAAAGCAAAAGTAAGGGTATC 193
Canis   ATAGTTGGATACA-----GTTTATTATTTTGCAGCCGTTTTATAGCTGTGTCTCAGTTTGGTCTGTGGTATAGTTAGGGGACTGCAAAAATAAGGGTATT 186

Homo    AATCTTAAGATGAAGGCATGGTAAA-----TTCAGTAAGTAATTGCACATTAAGTATCCATTTTACTTGCTAGAAGTGAGCTAAAAATTTTGCTTTGAAC 228
Pan     AATCTTAAGATGAAGGCATGGTAAA-----TTCAGTAAGTAATTGCACATTAAGTATCCATTTTACTTGCTAGAAGTGAGCTAAAAATTTTGCTTTGAAC 228
Macaca  ACTCTTAAGATGAAGCCATGGTAAA-----TTCAGTAAGTAATTGCACATTAAGTATCCATTTTACTTGCTAGAAGTGAGCTAAAAATTTTGCTTTGAAC 227
Bos     AGTCTTTAGATGAAACTTCATCTAAAGATGTTCAGTGATTAATGGCTCATTAAATGTCCAG------TGCTATAGATGAGCTGAAAATTTTACTTTGAAC 287
Canis   AATTTTAAGGTGAAGCTGTGGCAAA-----TTCAGTAAATAATTGCACATTAAATGTCCAGTTTGCTTACTGGAA--GAGCTAAAAATCTGGCTTTGAAC 279

Homo    TTCCCAGCTTACATATAAAGATGGAGAAGC--ATCTGTTGTATACTTCACAAGTATTAGGAAGCTTAAAATAAAACTATGATAAGTAACTTTGGGGCTTT 326
Pan     TTCCCAGCTTACATATAAAGATGGAGAAGC--ATCTGTTGTATACTTCACAAGTATTAGGAAGCTTAAAATAAAACTATGATAAGTAACTTTGGGGCTTT 326
Macaca  TTCCCAGCATACATGTAAAGATGGAAAAAC--ATCTGTTGTGTACTTCACAAGTATTAGAAAGCTTA---------------AACTAACCTTGGGGCTTT 310
Bos     TTTCCAGCACAAATGTAAAGATGAAGAAACTTACCTGTAGTATAATTT---AGTATCAGTAAGCTTA--------------------------------- 351
Canis   TTCCTAGC------ATAAAGATGGAGAAAC--ATTTGTTTTATAATTC---AGCTTCAATAAACTTA--------------------------------- 335

Homo    TAATTTAAAAAAAG-----AAAAAGTTCTGAATCCGTTTTGCCAA-CATTGTTGGTCTGTTCACAAAGTTAAACAGTGTATATTTATTTTCTTCTACAG 419
Pan     TAATTTAAAAAAAG-----AAAAAGTTCTGAATCCGTTTTGCCAA-CATTGTTGGTCTGTTCACAAAGTTAAACAGTGTATATTTATTTTCTTCTACAG 419
Macaca  TAATTTAAAAAAAGAAAAAAAAAAGTTCTGAATCCATTTTGCCAT-CATTGTCAGTCTGTTCACAAAGTTAACCAGTGTACGTTTATTTTCTTCTACAG 408
Bos     -----------------AAATGAAGGTCTTGATATATTTTTGCTGTCAGTGT--GGTCACTGACATAGTTAACCATATTTATTTTTTTTCTTCTACAG 431
Canis   ----------------------AAGTTCTGAATCCATGTTGGAAATCATTGCTGGTCTACTGATATACTTAACCCACCTACTTTGTTTTCCCTTTACAG 412
```

ENSG00000163935 intron 5

Description: Scm-like with four MBT domains protein 1 (SFMBT1)
Intron number: 5
Human chromosome: 03
Intron start (bp): 52940092
Human intron length : 1026
Intron alignment length: 1421
Flanking exons length (upstream/downstream): 247/95
SNP density: 0.001949
K tree score: 0.0886
Scaling factor: 0.9371
Human-chimpanzee distance: 0.008882
Total primate branch length: 0.076

ENSG00000163935 exon 5

```
Homo    ATTGTTACTGTAGTAGAAAACATTGGAGGAAGGCTGAAGCTACGTTATGAAGGACTTGAAAGTTCTGACAATTATGAACA 80
Pan     ATTGTTACTGTAGTAGAAAACATTGGAGGAAGGCTGAAGCTACGTTATGAAGGACTTGAAAGTTCTGACAATTATGAACA 80
Macaca  ATTGTTACTGTAGTAGAAAACATTGGGGGAAGGCTGAAGCTACGTTATGAAGGACTTGAAAGTTCTGACAATTACGAACA 80
Bos     ATTGTTACTGTGGTAGACAACATTGGGGGAAGGCTGAAGCTGCGTTATGAAGGACTTGAGGGTTCTGATAATGTTGAGCA 80
Canis   ATTGTTACTGTAGTAGAAAACATTGGGGGAAGGCTGAAGCTGCGTTATGAAGGACTTGCAAGTTCTGACAGTTTTGAGCT 80

Homo    TTGGTTGTATTACTTGGATCCATTTCTTCATCACGTTGGTTGGGCTGCTCAACAGGGATATGAGCTTCAGCCCCCTTCAG 160
Pan     TTGGTTGTATTACTTGGATCCATTTCTTCATCACGTTGGTTGGGCTGCTCAACAGGGATATGAGCTTCAGCCCCCTTCAG 160
Macaca  TTGGTTGTATTACTTGGATCCATTTCTTCATCACGTTGGTTGGGCTGCTCAACAGGGATATGAGCTTCAGCCTCCTTCAG 160
Bos     TTGGTTGTATTACTTGGATCCGTTTCTTCATCACGTTGGTTGGGCTGCTCAACAAGGGTATGAGCTTCAACCCCCATCAG 160
Canis   TTGGTTGTATTACTTGGATCCGTTTCTTCATCACGTTGGTTGGGCTGCTCAGCATGGATATGAACTGCAGCCCCCATTAG 160
```

ENSG00000163935 exon 6

```
Homo    CCATTAGACATCTAAAAAATGAAGCTGAGTGGCAAGAGATTTTGGCCAAAGTGAAAGAGGAAGAGGAAGAGCCATTACCA 80
Pan     CCATCAGACATCTAAAAAATGAAGCTGAGTGGCAAGAGATTTTGGCCAAAGTGAAAGAGGAAGAGGAAGAGCCATTACCA 80
Macaca  CCATCAGACATCTAAAAAATGAAGCTGAGTGGCAAGAGATTTTGGCCAAAGTGAAAGAGGAAGAGGAAGAGCCATTACCA 80
Bos     CCATCAGACATCTGAAAAATGAAGCTGAGTGGCAAGAGATCTTGGCCAAAGTCAAAGAG---GAGGAAGAGCCATTACCA 77
Canis   CCATCCGGCATCTGAAAACTGAAGCTGAGTGGCAAGATATCTTGGCCAAAGTGAAAGAGGAGGAGGAAGAGCCATTGCCA 80

Homo    TCTTACTTATTTAAG 95
Pan     TCTTACTTATTTAAG 95
Macaca  TCTTACTTATTTAAG 95
Bos     TCTTATTTGTTTAAG 92
Canis   TCCTACTTATTTAAG 95
```

ENSG00000163935 intron 5

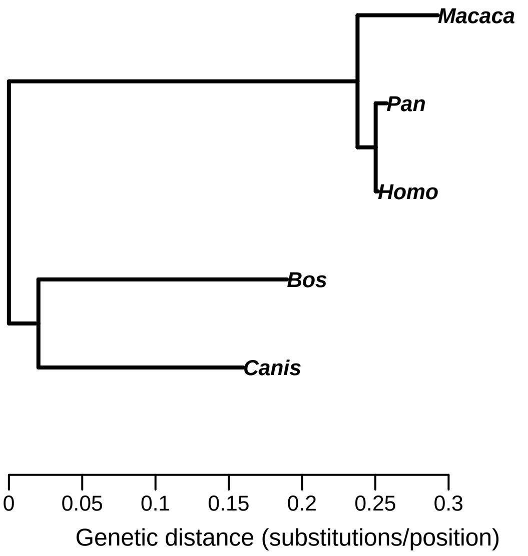

ENSG00000163935 intron 5

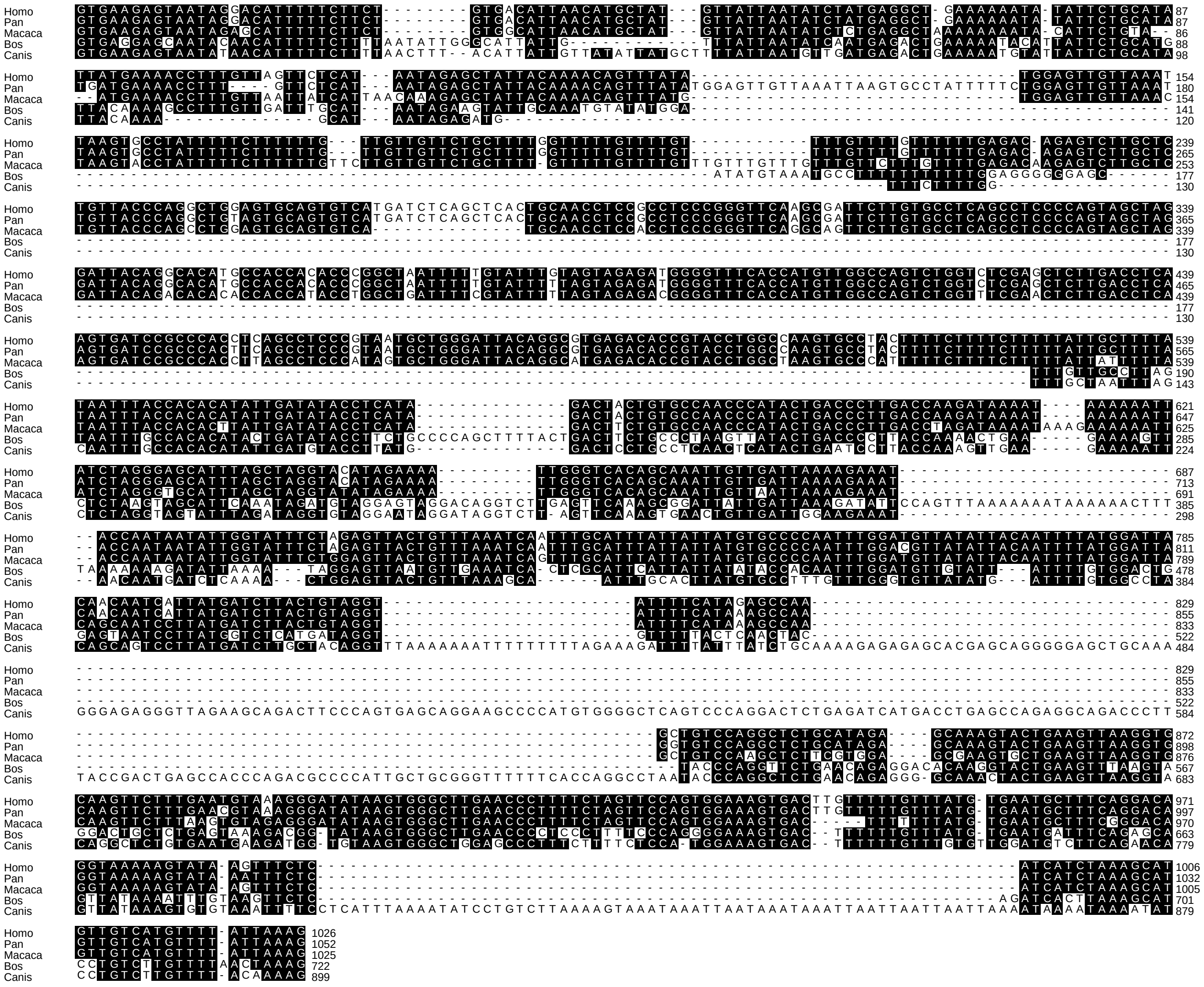

ENSG00000104381 intron 1

Description: Ganglioside-induced differentiation-associated protein 1 (GDAP1)
Intron number: 1
Human chromosome: 08
Intron start (bp): 75425369
Human intron length : 695
Intron alignment length: 947
Flanking exons length (upstream/downstream): 117/193
SNP density: 0.005755
K tree score: 0.0942
Scaling factor: 0.9556
Human-chimpanzee distance: 0.014690
Total primate branch length: 0.076

ENSG00000104381 exon 1

```
Homo    ATGGCTGAGAGGCAGGAAGAGCAGAGAGGGAGCCCGCCCTTGAGGGCGGAAGGCAAGGCCGACGCGGAGGTTAAGCTCAT 80
Pan     ATGGCTGAGAGGCAGGAAGAGCAGAGAGGGAGCCCGCCCTTGAGGGCAGAAGGCAAGACCGACGCGGAGGTTAAGCTCAT 80
Macaca  ATGGCTGGGAGGCAGGAAGAGCAGAGAGGGAGCCCGCCCTTGAGGGCGGAAGGCAAGGCCGACGCGGAGGTTAAGCTTAT 80
Bos     ATGGCTCGGAGGCAGGACGAGCAGAGAGGGGGAGCGCCCTTGATTGCGGAAGGCAAGTCGGACGCAGAGGTTAAGCTCAT 80
Canis   ATGGCTCGGAGGCAGGAGGAGCAGACAGGGGGCGCGCCCTTGATGGCGGAAGGCAAGGCGGACGCGGAGGTTAGGCTCAT 80

Homo    TCTGTACCATTGGACGCATTCGTTCAGCTCTCAAAAG 117
Pan     TCTGTACCACTGGACGCATTCCTTCAGCTCTCAAAAG 117
Macaca  TCTGTACCACTGGACGCATTCCTTCAGCTCTCAAAAG 117
Bos     TCTGTACCACTGGACGCATTCCTTCAGCTCTCAAAAG 117
Canis   TCTGTACCACTGGACACATTCTTTCAGCTCTCAAAAG 117
```

ENSG00000104381 exon 2

```
Homo    GTGCGCTTGGTAATTGCTGAAAAGGCATTGAAGTGCGAGGAACATGATGTAAGTCTGCCCTTGAGTGAGCACAATGAGCC 80
Pan     GTGCGCTTGGTAATTGCTGAAAAGGCATTGAAGTGCGAGGAACATGATGTAAGTCTGCCCTTGAGTGAGCACAATGAGCC 80
Macaca  GTGCGCTTGGTAATTGCTGAAAAGGCATTGAAGTGCGAGGAACATGATGTAAGTCTGCCCTTGAGTGAGCACAATGAGCC 80
Bos     GTGCGCTTGGTAATTGCTGAAAAGGCATTGAAGTGCGAGGAACATGATGTAAGTCTGCCCCTGAGTGAGCACAATGAGCC 80
Canis   GTGCGCTTGGTAATTGCTGAAAAGGCATTGAAGTGCGAGGAACATGATGTAAGTCTGCCCCTGAGTGAACACAATGAGCC 80

Homo    TTGGTTTATGCGTTTGAACTCAACTGGAGAAGTGCCTGTCCTTATCCACGGGGAAAACATAATTTGTGAGGCCACTCAGA 160
Pan     TTGGTTTATGCGTTTGAACTCAACTGGAGAAGTGCCTGTCCTTATCCACGGGGAAAACATAATTTGTGAGGCCACTCAGA 160
Macaca  TTGGTTTATGCGTTTGAACTCAACTGGAGAAGTGCCTGTCCTTATCCACGGGGAAAACATAATTTGTGAGGCCACTCAGA 160
Bos     TTGGTTTATGCGTTTGAACTCAACTGGAGAAGTGCCCGTCCTTATCCACGGGGAAAACATTATTTGTGAGGCCACTCAGA 160
Canis   TTGGTTTATGCGTTTGAACTCAACTGGAGAAGTGCCTGTCCTTATCCACGGGGAAAACATTATTTGTGAGGCCACTCAGA 160
```

ENSG00000104381 intron 1

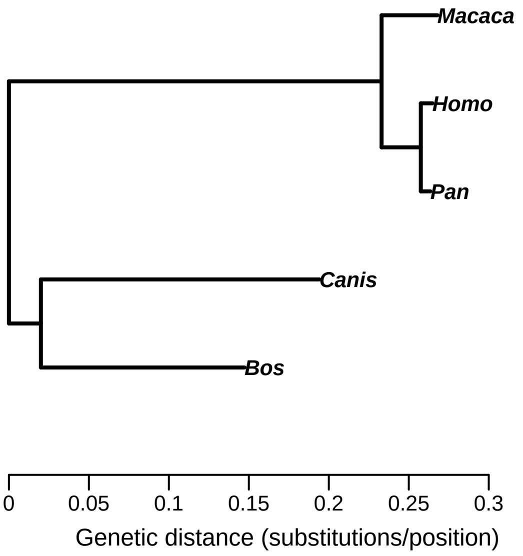

ENSG00000104381 intron 1

```
Homo    GTACAACAGGCCTTGGCGGCGGAGGGTGGCGCGGATCGGGCTTCAGCACTGGGACAGCTCCTTCTGAGTCCCGCCCAGGGAAGCCGGTCCACCTAGAAAT 100
Pan     GT---ACAGGCCTTGGCGGCGGAGGGTGGCGCGGATCGGGCTTCAGCACTGGGACAGCTCCTTCTGAGTCCCGCCCAGGGAAGCCGGTCCACCTAGAAAT 97
Macaca  GT---ACAGGCCTTGGCGGCGGAGGGTGACGCGGATCGGGCTTCAGCACTGGGACAGCTCCTGCTGGGTCCGGCTCAGAGAATCCGTTCCACCTAGAAAC 97
Bos     GT---AAAGGCCTTGGCC-GGGCGGGCGGCGCAGATCGGGTTTCAGCACCTGGACAGCTCCCGCTGGGTGCCGCCCGGGGAAGCCCGCCCACCGAGACCT 96
Canis   GT---AAAGGCCTCTGCCGGGGCGGGCGGCGCCGATCAGGTTTCAGCACCGGGACAGCTCCC---CGGGGCCCTCCGGGCA--CTCGGCCTCCAG----- 87

Homo    CCGCCTCCAGTGTCCTGACCCCGGGCAGGCGCTCCCTCCAGGCGGGGACGCGCCC--GGGTGGGGAGCGGGGAGAATCGATCGGAGATGATCCTATGTGG 198
Pan     CCGCCTCCAGTGTCCTGACCCCGGGCAGGCGCTCCCTCCAGGCGGGGACGCGCCC--GGGTGGGGAGCGGGGAGAATCGATCGGAGATGATCCTATGTGG 195
Macaca  CCGCCTCCAGCGTCCTGACCCTGGGCAGGCCCTCCCTCCA-----GGATGCCCCC--CGGTGGGGAGCGGGTAGAACCGGTCTGAGATAATCTTATGTGG 190
Bos     CCAACTCAACC------CCTGCGGGCGGCGCGCCCTGTGGGGCAGGGGTGCGCTTTATATGGAGGAGCAGGGAAAATCAGTCTGAGATG-----GGAAGG 185
Canis   --AACCGGGCC------TCCCCGGGCGCAGCATCCCTTCGAGC-----------------------------GCACTTAGTCTGAGATG-----CAAAGG 145

Homo    CTATGGCCCAGGCTCCGCCTCTCGCGAAATGCGAC-AAAAAAGCATC-ATTTTCAGTATTTGGTCTGGAGGCCTTGCCTGCTGGTCT-TCGGAGA--GAG 293
Pan     CTATGGCCCAGGCTCCGCCTCTCGCGAAATGCGAC-AAAAAAGCGTC-ATTTTCAGTATTTGATCTGGAGGCCTTGCCTGCTGGGCT-TCGGAGA--GAG 290
Macaca  CTATGACCCAGGCTCTGCCTCTCGCGAAATGCGACAAAAAAAGCATC-ATTTTCAGTATTTAGCCTGGAGGCCTTGCCTGCTGGGCT-TCGGAGA--GAG 286
Bos     CCGTGACTCAGGCACCGCCACTCATGAAATGCGAC-AGAAAA-CATC-ACTTTCGGCGTTGGGGCGGGAGGCCTCGTCTCCGGGGCCTTTGGAGAGTGAG 282
Canis   CTATGACTCAGGCTCTGCCGCGGATGAAATGCGAC-AGAAAAGCATCTACTTTAGGTGTTTGGGCGGAAGGCCTTGTCCCCTGGGCC-TTGGAGAGCGAG 243

Homo    GCAGAGGGAGGTGCCTTTGTGGACCCTCATAATC----------------------------------------------------------TGCGATCT 335
Pan     GCAGAGGGAGGTGCCTTTGTGCACCCTCATAATC----------------------------------------------------------TGCGATCT 332
Macaca  GCAGAGGGAGGTGCCTTCGTGCACCCTTATCATC----------------------------------------------------------TGCGATCT 328
Bos     GCAGAGGGAAGCGCCTTCCGGCAACCATCCCATC----------------------------------------------------------CAGGGTTC 324
Canis   GCAGAGGAAAGCGCCTTCCTGCAACCGCATCATCTTCTTTTACCCTTTGTACGCATTTGCTCTCCTCCACAAGGCCTAGCACATCTAGATATCAGATTCT 343

Homo    CTTCTTTTG------------CTCTCTCGCATTCACTGTCTTCAGCAAGAGC------------------------------------------------ 375
Pan     CTTCTTTTG------------CTCTCTCGCATTCACTGTCTTCAGCAAGAGC------------------------------------------------ 372
Macaca  CTTC---TG------------CTCTCTCGCATTCACTTTCTTCAGCAAGAGC------------------------------------------------ 365
Bos     CTTCTTTTA------------CTCTTGGTTGATTGACTTTTCAACAGG---------------------------------------------------- 360
Canis   TTTTTTTTAAATAATTTATTTTTTGTTGGTGTTCAATTTGCCAACATAGAGCATAACACTCAGTGCTCATCCCATCAAGTGCCCCCCTCAGTGCCCGTCA 443

Homo    ---------------------------------------------------------------------------------TGATGTACCTACATGTCAG 394
Pan     ---------------------------------------------------------------------------------TGATGTACCTACATGTCAG 391
Macaca  ---------------------------------------------------------------------------------TGATGTACGTACATGTCAG 384
Bos     ---------------------------------------------------------------------------------CCTAGTATATCTGTTTCAG 379
Canis   CCCAGTCACCCCCACCCACCTCCCTTTCTACCACCCCTTGTTCGTTTCCCAGAGTTAGGAGTCTCTCACGTTCTGTCTCCCTCTGATATTTCCCACTCAT 543

Homo    GTTCTTGTCTG----------------------------------GGGAGGACCCATAATCAGGTTTAGGACACCGAG-AAAGTGGTAGTGATGAGGAGG 459
Pan     GTTCTTGTCTG----------------------------------GGGAGGACCCGTAATCAGGTTTAGGACACCGAG-AAAGTGGTAGTGATGAGGAGG 456
Macaca  GTTCTTTTCTG----------------------------------GGGAGGGCGCATAATCAGGTTTAGGACGCCAAG-AAAGTGGTGGTGATGAGGAGG 449
Bos     GTTCTTACTAT------------------------------------GGGGACGCATCATAAGGTTTAGGACACAAAGAAAAGTAGTAGTGATGAAGAGG 443
Canis   TTTTTCTCCTTTCTCCTTTATTCCCTTTCACTATTTTTTTGTCTGGGGAGGACACATAATTAGGTTTAGGACGCAAAG-AAAGTACTGATGATG-GGAGA 641

Homo    GTGACTGTTTAATG----AATACAGTCAGAATCATT-TCATTACTTATTTTGTGTTCTTCTCTTAATTTGTTGA------GTATTGTTCTTTCCTTACTT 548
Pan     GTGACTGTTTAATG----AATACAGTCAGAATCATT-TCATTACTTATTTTGTGTTCTTCTCTTAATTTGTTGA------ATATTGTTCTGTACTTACTT 545
Macaca  GTGACTGTTTAATG----AATACAGTAAGAATCATT-TCATTACTTATTTTGTG---TTCTCTTAATTTGTTGA------ATATTGTTCTG----TACTT 531
Bos     GTGACCGTGGACTG----AATATTGCCAGAATCATTCTCATTACGTATCTT----TTCTAACTTACAAGATTGAGGTGCGAGAATATGTCT----CACTT 531
Canis   GTCACTGTGGAATAAATCAATACAGCCAGAATTATTTTCGTTCCTCATTTTGTTGTCCTAATTTATATGATTAAGATGCTAGAATATATCT----CACTT 737

Homo    GTTAATTCCAGGAATAGGAATTGTTAAAACGACTGTTGAAAATGTCGGTAACACAGGGAAGCCCAGAAAGGCTGCTTAGCGGTGTCCAGGGAAGTCATTT 648
Pan     GTTAATTCCAGGAATAGGAATTGTTAAAACGACTGTTGAAAATGTCAGTAACACAGGGAAGCCCAGAAAGGCTGCTTAGCGGTGTCCAGGGAAGTCATTT 645
Macaca  GTTAATTCCAGGAATAGGAATTGTTAAAACGACTGTTGAAAATGTCAGTAACACAGGGGAGCCCAGAAAGGCTGCTTAGCGGTGTCCAGGGAAGTCATTT 631
Bos     GTTAATTCCAGGAATAG----AGTTGAAAGGGGTGTTGAAAATGTTAATAACTCAGGGAAGCACAAAAAGGCTGCTGGCTGGTATCCAGGGATGTCATTT 627
Canis   GTTAATTCCGAGGACAGGAATAGTTAAAGAGGTTGTTGATAA---TAATAACTTAGGGAAACACAGAAAGGCTACTTAATGGTATCCAGGGGTGTCAGTT 834

Homo    AATTGAAAGCTTACATGTGTTGTAGTAACCAGTGTGAACTCTTCCAG 695
Pan     AATTGAAAGCTTTCATGTGTTGTAGTAACCAGTGTGAACTCTTCCAG 692
Macaca  AATTGAAAGCTTACACGTGTTGTAGTAACCAGTGTGAACTCTTCCAG 678
Bos     AATTAAAAGCCTACCTGCGTTGTGGTAACCAATGTGAACTCTTCCAG 674
Canis   -----AAAGCCTGCATGTGTTGTAGTAACCAGTGTGAACTCTTTCAG 876
```

ENSG00000164062 intron 14

Description: Acylamino-acid-releasing enzyme (APEH)
Intron number: 14
Human chromosome: 03
Intron start (bp): 49693067
Human intron length : 471
Intron alignment length: 472
Flanking exons length (upstream/downstream): 89/139
SNP density: 0.000000
K tree score: 0.0887
Scaling factor: 0.9719
Human-chimpanzee distance: 0.011061
Total primate branch length: 0.0759

ENSG00000164062 exon 14

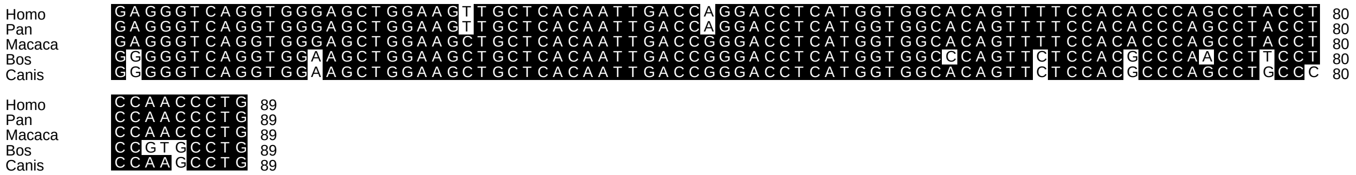

ENSG00000164062 exon 15

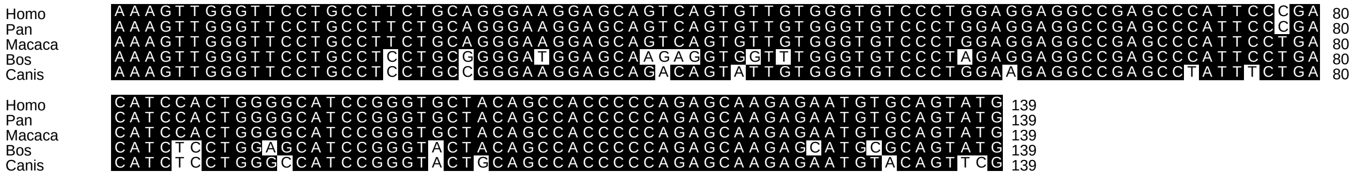

ENSG00000164062 intron 14

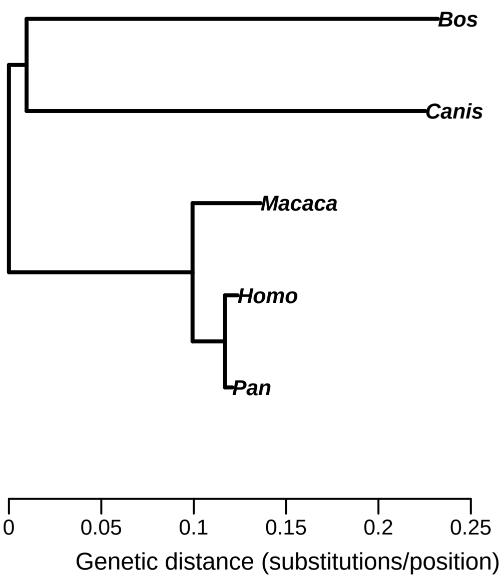

ENSG00000164062 intron 14

```
Homo    GTGAGTGTCGGTGGGTCCCAGGCTGTGGAGGCAGGGGTTCTGGGCAGGCAGAGCTCACCAGGTCAGGGAATTGGCCCCAGAGTCTGACC-TGGCTGGTCA 99
Pan     GTGAGTGTCGGTGGGTCCCAGGCTGTGGAGGCAGGGGTTCTGGGCAGGCAGAGCTCACCAGGTCAGGGAATTGGCCCCGGAGTCTGACC-TGGCTGGTCA 99
Macaca  GTGAGTGTCGGTGGGTCCCAGGCCGTGGAGGCAGGGGGTCTGGGCAGGCCAAGCTCATCAGGTCAGGGAATTGGCCCCCGAGTCTGACCATGGCTGGTCA 100
Bos     GTGAGTGTTGGTGGGCCACAGACCA-GGAGGCAGGGATTCTGTGCAGGTGGAGTTC-CCAGGTCAAGGAACTGACCC--AAGTCTTGAC-TGGCTGGTCA 95
Canis   GTGAGTGTTGGTGGGCCCCGGACCA-GGAAGTGAGGGTTCTGTGTAGGCAGAGCTCCCCAGGTCAGGGAGCTGGCCT--GAGTCTGACTGCGGTTGGTCA 97

Homo    GCATACAGACGGGCCAAGACACAGGCCCAGCTTGGCATGGTCCATGAGGGTTTGGCACTCACAGGGACGAACTACTAGGCTAGAATCTGCTCCGTACAAA 199
Pan     GCATACAGACGGGCCAAGACACAGGCCTAGCTTGGCATGGTCCATGAGGGTTTGGCACTCACAGGGACGAACTACTAGGCTAGAATCTGCTCCGTACAAA 199
Macaca  GCATACAGATGGGCCAAGACACAGGCCCAGCTTGGCATGGTCCATGAGGGTTTGGCACTCACAGGGACGAACTACTAGGCTAGAATCTGCTCTGTACAGA 200
Bos     GTTCATGAGT-AGCCAGGATGCAGACTCAGCTCAGGATAGTCCACGA-GATCTGGCAC-CATGGGGAGGAATGGCTGAGCTGAAGTTTGCTCCGTACAGA 192
Canis   GCATGTGGGA-GGCCGAGAAGTGGTCCCAGCCCAGCATGGTCCATAGGGGTCTGGCACCCATGGGGAGCTACTACCAGGTGTAGGTCTGCTCTATAAAGA 196

Homo    ACAAGGTGCCTGGGGAAGTGCTCTGTCTTGGTATTACAGAGCAAAGGAGACAGCAGAGCCTAGAAGCTGAGGAGGGAGGGGTGTGCCTGTGTGTGATTGT 299
Pan     ACAAGGTGCCTGGGGAAGTGCTCTGTCTTGGTATTACAGAGCAAAGGAGACAGCAGAGCCTAGAAGCTGAGGAGGGAGGGGTGTGCCTGTGTGTGATTGT 299
Macaca  ACAAGGTGCCTGGGGAAGTACCCAGTCCTGGTATTATAGATCTAAGGAGACAACAGAGCCTAAAAGCTGAGGAGGGAAGGGTGTGCCTGTGTGTGATTGT 300
Bos     ACAGGGTGCCTGGGAAAGTGCTCAGTCCT---------------CTGGCGTAAAATAGCCTAGAGGCTGAGGAGGGAGGG--GTACCTCTGTGTGGTTGG 275
Canis   ATAGGGTACCCAGGAAAGTGCCCAGTCCT---CTGGCACAGCAGAGGACACAGCAGAGCCTAAAAGTTCAGGAGGGAAGGGTGTGTGTTATGTGG---- 289

Homo    ATGAGTATGAATACCTTGGTATATACCATCCCACAACAGGAAGCCATGCTGTACCTTCTGGAGGTGGGTAGAGGGCCCAGCATAGTGAGAGCTGCCTATT 399
Pan     ATGAGTATGAATACCTTGGTATATACCATCCCACAACAGGAAGCCATGCTATACCCTCTGGAGGTGGGTAGAGGGCCCAGCATAGTGAGAGCTGCCTATT 399
Macaca  ATGAGTGTGAATACCTTGGTATATACCATCCCACAACAGGAAGCCATGCTATACCCTCTGGAGGTGGGTGGAGGGCCCAGCATAGTGAGAGCTGCCTATT 400
Bos     GTGAGTG------CCCGTGTATATAACAGCTTACGACAGGAGTCCACGCCGTGCCCTCTGGAGGTGGGCAGACAGCCTAGTAGAGCGAGGGCTGTCCA-T 368
Canis   --GAGTG------CTTCTGCATATACCGGCTCACAGCATTAGTCTATGATGTACCCTC--GAGGT-GGTAAAAGGCCTAGCATAGTGAGAGCTGCCCACC 378

Homo    TCCCACTGTGGGCAGAAGGGAAGGGCAGGGAGGCAGCCAGGCCACCTATGACACATTCTTTCCCCTTGGCAG 471
Pan     TCCCACTGTGGGCAGAAGGGAAGGGCAGGGAGGCAGCCAGGCCACCTATGACACATTCCTTCCCCTTGGCAG 471
Macaca  TCCCACTGTGGGCAAAAGGGAAGGGCAGGGAGGCAGCCAGGCCACCTGTGACATATTCCTTCCCCTTGGCAG 472
Bos     GCTCACAGTGG------TAGGAAGGCTGGGAGGCAGCCAGGCCACCTGTGACCCACTCTTTTCCCTTGGCAG 434
Canis   TCCCACGTGGC-----------------AGAGGCAGCCAAGCCACCTATGACACACTTTTTCCCCTTGGCAG 433
```

ENSG00000133800 intron 5

Description: Lymphatic vessel endothelial hyaluronic acid receptor 1 precursor (LYVE1)
Intron number: 5
Human chromosome: 11
Intron start (bp): 10537421
Human intron length : 518
Intron alignment length: 551
Flanking exons length (upstream/downstream): 79/187
SNP density: 0.005792
K tree score: 0.0807
Scaling factor: 1.0776
Human-chimpanzee distance: 0.011739
Total primate branch length: 0.0759

ENSG00000133800 exon 5

```
Homo    GTGTCCCCACGGCTCTGCTAGTGCTTGCTCTCCTCTTCTTTGGTGCTGCAGCTGGTCTTGGATTTTGCTATGTCAAAAG 79
Pan     GTGTCCCCACGGCTCTGCTAGTGCTTGCTCTCCTCTTCTTTGGTGCTGCAGCTGGTCTTGGATTTTGCTATGTCAAAAG 79
Macaca  GTGTCCCCACGGCTCTGCTAGTGCTTGCTCTCCTCTTTTTTGGTGCTGCAGCTGGTCTTGGATTTTGCTATGTCAAAAG 79
Bos     GTGTTCCCACTGCCCTGCTGGTGCTTGCGCTCCTCTTCTTTGCTGCGGCAGCTGGCCTCGCAGTTTGCTACGTCAAAAG 79
Canis   GTGTCCCCACGGCTCTGCTAGTGCTTGCACTCCTCTTCTTTGCTGCCGCAGCTGGTTTGGCGGTTTGCTACGTCAAAAG 79
```

ENSG00000133800 exon 6

```
Homo    GTATGTGAAGGCCTTCCCTTTTACAAACAAGAATCAGCAGAAGGAAATGATCGAAACCAAAGTAGTAAAGGAGGAGAAGG 80
Pan     GTATGTGAAGGCCTTCCCTTTTACAAACAAGAATCAGCAGAAGGAAATGATCGAAACCATAGTAGTAAAGGAGGAGAAGG 80
Macaca  GTATGTGAAGGCCTTCCCTTTTACAAACAAGAATCAGCAGAAGGAAATGATTGAAACCAAAGTAGTAAAGGAGGAGAAGG 80
Bos     GTATGTGAAGGCATTCCCTTTTACAAATAAGAACCAGCAGAAGGAAATGATAGAAACTAAAGTAGTAAAAGAGGAGAAGG 80
Canis   GTATGTGAAGACCTTTCCTTTTACAAACAAGAATCAGCAGAAGGAAATGATTGAAACCAAAGTAGTAAAGGAAGAGAAAG 80

Homo    CCAATGATAGCAACCCTAATGAGGAATCAAAGAAAACTGATAAAAACCCAGAAGAGTCCAAGAGTCCAAGCAAAACTACC 160
Pan     CCAATGATAGCAACCCTAATGAGGAATTGAAGAAAACTGATAAAAACCCAGAAGAGCCCAAGAGTCCAAGCAAAACTACC 160
Macaca  CCGATGATATCAACCCTAATGAAGAATCAAAGAAAACGGATAACAACCCAGAAGAGCCCAAGAGTCCAAGCAAAACTACC 160
Bos     CCGATGATAGCAACCCTAATGAGGAATCAAAGAAAATGAATAAAACGCCAGAGGAGCCCAAGAGTCCACCCAAAACCACA 160
Canis   CCGATGATAGCAACCCTAATGAAGAATCAAAGAAAACTGATAAAAAGCCAGAAGAGCCCAAGAGTCCAACCAAAACCACA 160
```

ENSG00000133800 intron 5

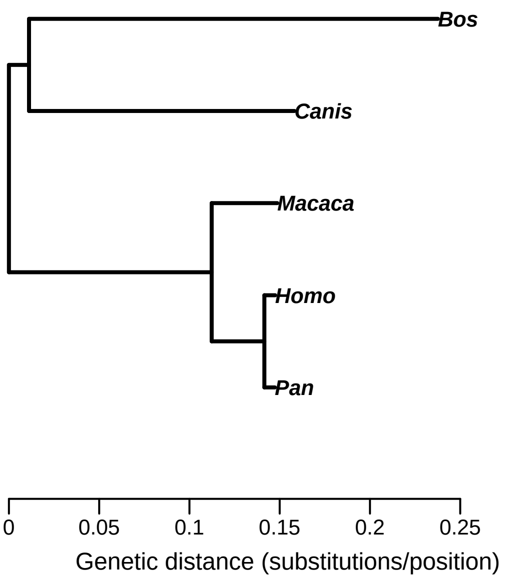

ENSG00000133800 intron 5

```
Homo    GTAGGGTTGTTGGTGCACTGCTAGTCTTGTAATCTTTGTCATGTTTCTATCCTGTGCTTTTTCAGGGTCACTGTTGGCATTTTGCTGATCAAAATGGAGA 100
Pan     GTAGGGTTGTTGGTGCACTGCTAGTCTTGTAATCTTTGTCATGTTTCTATCCTGTGCTTTTTCAGGGTCACTGTTGGCATTTTGCTGATCAAAATGGAGA 100
Macaca  GTAGGGTTGCTGGTACACTGCTAGTCTTGTAATCTTTGTCAAGTTTCTATCCTGTGCTTTTTCAAGGTCGCTATTGGCATTTTGCTGATCAAAATGGAGA 100
Bos     GTGGGGTTGTTGGCAGATGGTCGGTCTCATCATTTTTGTCGCATCTGTATCTCATGTTATCCAGGTT-GCCATTAGCATTTCCCTTTCCCAAATGGAGA 99
Canis   GTGAGGTTGTTGGCCCATTGTTAGTCTTATAATTTATGTCACATTCCTATCTTGTGTTTTCCAGGGT---TTTTAGCATTTTCTTAGTCAAAACAGAAA 97

Homo    TAGCAGTCCTCTGATA---TAAA-TTTCTCCCTCATCTCATCTCT-TTCCCTTCAGCCCAATCTTTT---CTATTTCAGGACATGCATGTCGTCCAGCCA 192
Pan     TAGCAGTCCTCTTATA---TAAA-TTTCTCCCTCATCTCATCTCT-TTCCCTTCAGCCCAATCTTTT---CTATTTCAGCACATGCATGTCGTCCAGCCA 192
Macaca  TAGCAGTCCTCTTATACTGTAAA-TTTCTCTCTCATCTCATCTCT-CTCCCTTCCGCCCAGTCTTTT---CTATTTCAGCACATGCATGTCATCTAGCCA 195
Bos     TGGCAGTGCTCATACACTGTACATGTTCCTCCCCACCTCATTTGTCCTCCCTACCACCCAAACTTCCCTACTGTTTCAGCACATGCGTGTTATCCGGCTT 199
Canis   TGAGAGTCTTCTTATATTGTAAATTTTCTTCTTCATCTCACCTCTCCTCCCCACCACCCAATCTTTCCTACTATTTCAGCACATGCATGTCATCTAGACT 197

Homo    TCA-------GTCAATGACTGT-CCTTTGAAAATCAAGAAAT---TAAAAAAAATTGACATCTGGG-GAAAATTTATATGCTTTA-TTTGATTTTTGGTT 279
Pan     TCA-------GTCAATGACTGT-CCTTTGAAAATCAAGAAAT---TAAAAAAAATTGACACCTGGG-GAAAATTTATATGCTTTA-TTTGATTTTTGGTT 279
Macaca  TCA-------GTCAATGACTGT-CCTTTGAAAATCAAGAAA----TAAAAAACATTGACATCTGGG-GAAAATGTATATGCTCTA-TTTGATTTTTGGTT 281
Bos     GAAGATTTAGCTCAATGGCTGTCCCTTTGAATATCAAGAAAT-----GAAAAGACTGGCATCTAGGAGGAAATGGATATGCTTTA-TTTGATTTTCAATT 293
Canis   TTAGAAACAGGTCAATGCTGTC-CCTTTGAAAATTAAGAGATAAGAAGAAAAGACTGACATCTAGG-GGGAAAATATATGTTTTATTTTGATTCTCAATT 295

Homo    CTTTGTTTTTGTTGGAAGAGTTATGTGGTCAGCCTAAAGTCTGGCCTGCAAACATATTGTCCAATTTAAATGAATCCATTAAGGTGGTGATGTCTTAATG 379
Pan     CTTTGTTTTTGTTGGAAGAGTTATGTGGTCAGCCTAAAGTCTGGCCTGCAAACATATTGTCCAATTTAAATGAATCCATTAAGGTGGTGATGTCTTAATG 379
Macaca  CTTTGTTTTATGGGAAGAATTATGTGGTCAGCCTAAAGTCTGGCCTGCAAACATATTTTCCCACTTAAATGAATCCATTAAGGTGGTGATGTGTTAATG 381
Bos     CTTTGCTTTGATGAGAGGGTTTTGAGAGCAGGCTAAAGTTTGGCCTGTGAACCTACCATCCCATCTAAATGAATCCATTCAGGTCAGGATGGGTGGGGG 393
Canis   CTTTGTTGTTGGTGGGAGGGTTATGAGGTCAGCCTCAAGTTTGGCCTGCAAACTTATTGCCTCATCTAACTGAATCCATTAAGGTGGGAATGCATTAATA 395

Homo    TATTAGA----GCATGCATGTGCTGGCATCACTCTGAAATAAAGGTATCCAGTGTACTGTGCCAATATGTACCATGGCC-AAAAAGTGTTATCTATCGTT 474
Pan     TATTAGA----GCATCCATGTGCTGGCATCACTCTGAAATAAAGGTATCCAGTGTACTGTGCCAATATGTACCATGGCC-AAAAAGTGTTATCTATCGTT 474
Macaca  TATTCGA----GCATGCATGTGCTAGCATCACCCTGAAATAAAGGTATCCAGTGTACTGTGCCAATATGTACCATGACCAAAAAAGTGTTATCTATTATT 477
Bos     TATTTGAGCATGCATGCATGTGTTGGCATCTCTCTGAAATAGAGGAACCCAGTGTACTCCGCCAGTGTAGGTCTTTAC----AAAGTATCATCTGTTGTT 489
Canis   TATTTGA----GTGTGCATGTGTTAGAATCTCTCTGAAATAAAGATACCCAGTGTACTGTGCTAATATGAGCTATCACAGACAAAGCATTACCTATTGTC 491

Homo    ATGTGTG----AGAG---TCTCTCACTTTAATTTCATGTTTCTGCCTAAAG 518
Pan     ATGTGTG----AGAG---TCTCTCAATTTAATTTCATGTTTATGCCTAAAG 518
Macaca  ATGTGTGTTACACAG---TCTCTCACTTTTATTTCATGTTTATGCCCAAAG 525
Bos     CTATGTGTTATATAGAAG--TCTCATTTTAATTTTGTGTTTATGCCCACAG 538
Canis   ATATGTTTTACATAGAAATCTCTCATTTTAATTTTATGATTATGCCTTTAG 542
```

ENSG00000139193 intron 5

Description: Tumor necrosis factor receptor superfamily member 7 precursor (CD27)
Intron number: 5
Human chromosome: 12
Intron start (bp): 6430442
Human intron length : 253
Intron alignment length: 286
Flanking exons length (upstream/downstream): 120/125
SNP density: 0.000000
K tree score: 0.0689
Scaling factor: 0.725
Human-chimpanzee distance: 0.016124
Total primate branch length: 0.0759

ENSG00000139193 exon 5

```
Homo    CCCAAAGATCCCTGTGCAGCTCCGATTTTATTCGCATCCTTGTGATCTTCTCTGGAATGTTCCTTGTTTTCACCCTGGCC 80
Pan     CCCAAAGATCCCTGTGCAGCTCCGATTTTATTCGCATCCTTGTGATCTTCTCTGGAATGTTCCTTGTTTTCACCCTGGCC 80
Macaca  CCCAAAGATCCCTGTGCAGCTCAGATTTTATTCGTATCCTTGTGATCTTCTCCGGAATGTTTCTTGTTTTCACCCTGGCC 80
Bos     CCCAAAGGTCCCTGTGCAGCGCGAACTGCATCCGCATCTTTGTGCTCCTCTCTGGAATGTTTCTTGCTTTCACCATAGTC 80
Canis   CCCAAAGGTCTCTGTGCAGCTCAGATTGCATCCGCATCTTTGTGATCCTCTCTGGAATGTTTCTTGCTTTCACCATGATC 80

Homo    GGGGCCCTGTTCCTCCATCAACGAAGGAAATATAGATCAA 120
Pan     GGGGCCCTGTTCCTCCATCAACGAAGGAAATATAGATCAA 120
Macaca  GGAACCCTGTTCCTCCATCAACAAAGGAAATATAGATCAA 120
Bos     GGAGCCTTGTTCCTCCATCAACAAAGAAAAT------TAA 114
Canis   GGAGCTCTGTTCTTCCACCAACAAAGAAAATACAGGCTAA 120
```

ENSG00000139193 exon 6

```
Homo    ACAAAGGAGAAAGTCCTGTGGAGCCTGCAGAGCCTTGTCATTACAGCTGCCCCAGGGAGGAGGAGGGCAGCACCATCCCC 80
Pan     ACAAAGGAGAAAGTCCTGTGGAGCCTGCAGAGCCTTGTCGTTACAGCTGCCCCAGGGAGGAGGAGGGCAGCACCATCCCC 80
Macaca  ACAAAGGAGAAAGTCCCATGGAGCCTGCAGAACCTTGTCCTTACAGCTGCCCCAGGGAGGAGGAAGGCAGCACCATCCCC 80
Bos     ACGCAGGAGAAAGTCCAGTGGCACCCGCTGAGCCTTGTCCTTACACCTGTCCCAGCGAGGAAGAGGGCAGTGCCATCCCC 80
Canis   ACAAAGAAGAATACCCAGTGGTGCCTGTGGAGCCTTGTCCTTACAGCTGCCCCAGGGAGGAAGAGGGTGGCGCCATCCCC 80

Homo    ATCCAGGAGGATTACCGAAAACCGGAGCCTGCCTGCTCCCCCTGA 125
Pan     ATCCAGGAGGATTACCGAAAACCGGAGCCTGCCTGCTCCCCCTGA 125
Macaca  ATCCAAGAGGATTACCGAAAACCGGAGCCTGCCTCCTCCCCGTGA 125
Bos     ATTCAGGAGGATTACCGAAAACCAGAGCCTACTTCCTACTTCTGA 125
Canis   ATTCAGGAAGATTACCGAAAACCAGAACCCGCTTCCTACTGCTGA 125
```

ENSG00000139193 intron 5

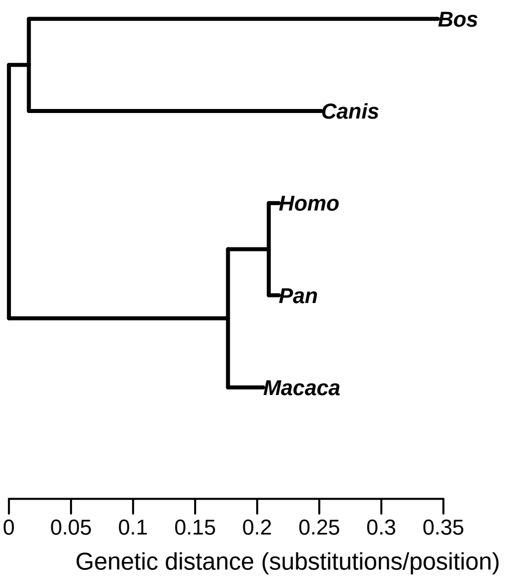

ENSG00000139193 intron 5

```
Homo    GTAAGAGACAGAACACAGGCCT--------------------CCGGTCCTGCCCCTGCACCACACCCCACTGGCTCAACCCCACTGCCCACGCCTGGAAT 80
Pan     GTAAGAGACAGAACACAGGTCT--------------------CCGGTCCTGCCCTTGCACCACACCCCACTGGCTCAACTCCACTGCCCACGCCTGGAAT 80
Macaca  GTAAGAGACAGAATACAGGTCT--------------------CCGGTCCTGCCCTTGCATCACACCCCAACAGCTCAGCCCCACTGCCCACGCCTGGAAT 80
Bos     GTAAGAGACAAAATACAGGTCCTAAAAAAAAAAAAAAAAAATACAGGTCCTGCACCC-CACCTCACCTCACCATCTTGGCTTTGCCACCCA-ATCTGGAAC 98
Canis   GTAAGAGACAACATACAGGTTT--------------------CCAGTCCTGTCCCTGTTCCCTACCCTACCATCCCAGGCCCCCTGCCCATGCCTGGAAC 80

Homo    CTCACTGAAACCCACC--AGCTCCACTTCACCAGCCTTGGTCCTACCCCT-----TCTCTCCCCTTAGCTGGCGTGCTCCTGACACCCCTCCCCCAAGCG 173
Pan     CTCACTGAAACCCACC--AGCTCCACTTCACCAGCCTTGGTCCTACCCCT-----TCTCTCCCCTTAGCTGGCGTGCTCCTGACACCCCTCCCCCAAGGG 173
Macaca  CTCACTGAAACCCACC--AGTTCCACTTCACCAGCCTTGGTCCTAGCGCT-----TCTCTCCCCTTAGCTGG-GTGCTCCTGACACCCCTCCCCCAAGCA 172
Bos     CTCTGGG---CCTCTCCAGCTCCATCTTTACCAGC-CTGTCGCCTCTCTT-----CCCCTCTACTATGCTGGGGCCCTCCTGACA--------------- 174
Canis   CTCAGGGAAGCTTTCCTTACTCCATCTTTCCCAACGCTGGCTCCCACCTTCTCTGTCTTCCCTCTTAGCTGGCAACCCCCTGACA-------GCTAAGCT 173

Homo    CACCCGCCTCTACCCATCTCCTTCTCCCGTC----TCCCCCTGCCCCCACTGCTGGCCAA-GACTCATCGGA-TCTCCTTCTGCAG 253
Pan     CACCCGCCTCTACCCATCTCCTTCTCCCGTC----TCCCCCTGCCCCCACTGCTGGCCAA-GACTCATCGGA-TCTCCTTCTGCAG 253
Macaca  CACCTGCCTCTGCCCATCTCCTTCTCCCGTC----TCCCCCGGCCCCCACTGCTGGCCAA-GACTCATCAGA-TCTCCTTCTGCAG 252
Bos     -GCCCGCCTTCACTCACCCCTTCTCCCT-------TCACCCCTCCTCTACCACGGGCCAACAACTCCTCTGATTCTCCT-CTGCAG 251
Canis   GACCCTCCTTGGCTCATCTCC-ACTCCTGTCACCACCCCCCACCCTCACTGCCAGTGAA-GGTTCATCTGATTCTCCTCCCGCAG 257
```

ENSG00000100650 intron 1

Description: Splicing factor, arginine/serine-rich 5 (SFRS5)
Intron number: 1
Human chromosome: 14
Intron start (bp): 69304753
Human intron length : 321
Intron alignment length: 343
Flanking exons length (upstream/downstream): 126/71
SNP density: 0.003115
K tree score: 0.0609
Scaling factor: 0.6994
Human-chimpanzee distance: 0.009423
Total primate branch length: 0.0758

ENSG00000100650 exon 1

```
Homo    ATGAGTGGCTGTCGGGTATTCATCGGGAGACTAAATCCAGCGGCCAGGGAGAAGGACGTGGAAAGATTCTTCAAGGGATA 80
Pan     ATGAGTGGCTGTCGGGTATTCATCGGGAGACTAAATCCAGCGGCCAGGGAGAAGGACGTGGAAAGATTCTTCAAGGGATA 80
Macaca  ATGAGTGGCTGTCGAGTATTCATCGGGAGACTAAATCCAGCGGCCAGGGAGAAGGACGTGGAAAGATTCTTCAAGGGATA 80
Bos     ATGAGTGGCTGTCGAGTATTCATCGGGAGGCTAAATCCGGCGGCCAGGGAGAAAGACGTGGAAAGATTCTTCAAGGGGTA 80
Canis   ATGAGCGGCTGTCGAGTATTCATCGGGAGGCTAAATCCAGCGGCCAGGGAGAAAGACGTGGAAAGATTCTTCAAGGGGTA 80

Homo    TGGACGGATAAGAGATATTGATCTGAAAAGAGGCTTTGGTTTTGTG 126
Pan     TGGACGGATAAGAGATATTGATCTGAAAAGAGGCTTTGGTTTTGTG 126
Macaca  TGGACGGATAAGAGATATTGATCTGAAAAGAGGCTTTGGTTTTGTG 126
Bos     TGGACGAATAAGAGATATTGATCTGAAAAGAGGCTTTGGTTTTGTG 126
Canis   TGGACGAATAAGAGATATTGATCTGAAAAGAGGCTTTGGGTTTGTG 126
```

ENSG00000100650 exon 2

```
Homo    GAATTTGAGGATCCAAGGGATGCAGATGATGCTGTGTATGAGCTTGATGGAAAAGAACTCTGTAGTGAAAG 71
Pan     GAATTTGAGGATCCAAGGGATGCAGATGATGCTGTGTATGAGCTTGATGGAAAAGAACTCTGTAGTGAAAG 71
Macaca  GAATTTGAGGATCCGAGGGATGCAGATGATGCTGTGTATGAGCTTGATGGAAAAGAACTCTGTAGTGAAAG 71
Bos     GAATTTGAAGATCCCAGGGATGCGGATGATGCTGTATATGAACTTGATGGAAAAGAACTCTGCAGTGAAAG 71
Canis   GAATTTGAGGATCCCAGGGATGCGGATGATGCTGTATATGAACTTGATGGAAAAGAACTCTGCAGTGAAAG 71
```

ENSG00000100650 intron 1

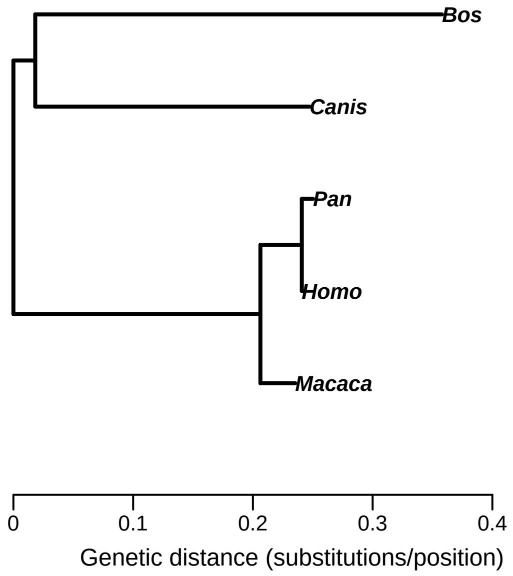

ENSG00000100650 intron 1

```
Homo    GTAAGTATTTAGAACTGG----GTGAATTATCTGCTA--AGTAGGTAGAG-TTTTGATAAGCAG----TTGTTTTCGATCTTGAGTCCGGGTGGAATATT 89
Pan     GTAAGTATTTAGAACTGG----GTGAATTATCTGCTA--AGTAGGTAGAG-TTTTGATAAGCAG----TTGTTTTCGATCTTGAGTCCGGGTGGAATATT 89
Macaca  GTAAGTATTTAGAACTGG----GTGAATTATCTGCTA--AGTAGGTAGAG-TTTTGATAAGCAG----TTGTTTTCGATCTTGAGTCCAGGTAGAATATT 89
Bos     GTAAGTGTTTACAACTGG----TTGAAATATCTATTGTTAGTGGGTAAAGATTGTTATAAGCAGTGCTTTTGAGTTT--TTTGAGTCCCAATAGAGTATG 94
Canis   GTAAGTATTGACCGTTGGTGGTTTAGAACTACTGTTA--AGTCGGTAAAGATCTTGATAAGTAGTGTCTTGATTTCCA-CTGAAGTTCAGGTAGAGTATT 97

Homo    TGCCTTCAGAAAATGTTACCCAGCCTTATGTCTGAATTTTATTGAGGCTGAAAACAACTTTAACTTGCCGGCTCACTGGAACCCCACTCCCAGTGGCTCC 189
Pan     TGCCTTCAGAAAATGTTACCCAGCCTTATGTCTGAATTTTATTGAGGCTGAAAACAACTTTAACTCGCCGGCTCACTGGAACCCCACTCCCAGTGGCTCC 189
Macaca  TGCGTTCAGAAAATGTTACCCAGCCTTGTGTGTGAGTTTTATTGAGGCTGAAAACAACTTGAACTTGCCGGCTCACTGGAA-CCCACTCCTAGTGGCTCC 188
Bos     TTTGGGACACATGCATTCTTAAGGT-----AGTCAGTTTTCTTAAAGGAGAAAAAGAATTT---CACTCCGGGCTGGAGAAA--TCATTTCCGGTCCCTTT 184
Canis   T----------------------------------------TTAAGGGATAAAAGAAC-----CTCGCTGTTGGAGGGAAA--CCACTCCTGATCACTTC 150

Homo    TTGATTAGGTTTGA-CTGTATGGCAGTGTCGTTAAGATA---CTCTTCCCATTCTGTCTCCTGATTTCAGTG-CTCTTAATGTGTTGTAGAATACTCTTC 284
Pan     TTGATTAGGTTTGA-CTGTATGGCAGTGTCGTTAAGATA---CTCTTCCCGTTCTGTCTCCTGATTTCAGTG-CTCTTAATGTGTTGTAGAATACTCTTC 284
Macaca  TTGATTAGGTTTGA-CGATGTGGCAGTGTTGTTAAGATACTTCTTTTCCTATTCTGTCTCCTGATTTCAATG-CTC--ATTATGTTGTAGAATACTCTTC 284
Bos     TTGATTGGGCTTAATCTATATGCTGTTGTCATTAACATA------------CTCTTTCACAGGCATTTAGTG-GTTTTAATGTGTTGTAGAATAGACTTT 271
Canis   TCAGTTCAACTTGT-GTACGTGGCACTGTTCTTAAGATA-------CTCAATCCTGCCACACGCTTTTAGTGATTTTTAGTATGTTGTAGAACACGCTTT 242

Homo    TAAAGCCAATGTTAAGAG------TCTTATGCTTACATTTTAG 321
Pan     TAAAGCCAATGTTAAGAG------TCTTACGCTTACATTTTAG 321
Macaca  TAAAGCCCATGT---------------TTATACTTACATTTTAG 313
Bos     T-AAAGCAGTGTTAAGCATTAT--GTATATGTTTGTGTTTTAG 311
Canis   --AGAGCCACGTTAAGCACTGTTTTTTCATGTTTACGTATTAG 283
```

ENSG00000136878 intron 9

Description: Ubiquitin carboxyl-terminal hydrolase 20 (USP20)
Intron number: 9
Human chromosome: 09
Intron start (bp): 131670550
Human intron length : 412
Intron alignment length: 553
Flanking exons length (upstream/downstream): 445/141
SNP density: 0.004854
K tree score: 0.0626
Scaling factor: 0.4896
Human-chimpanzee distance: 0.009780
Total primate branch length: 0.0757

ENSG00000136878 exon 9

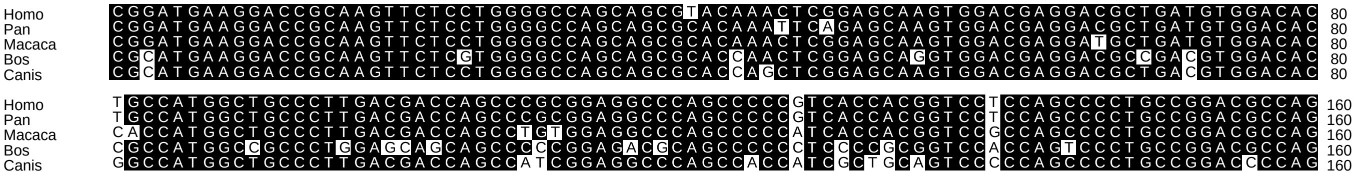

ENSG00000136878 exon 10

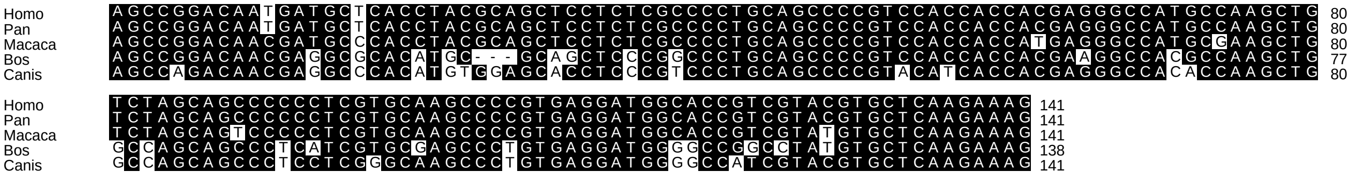

ENSG00000136878 intron 9

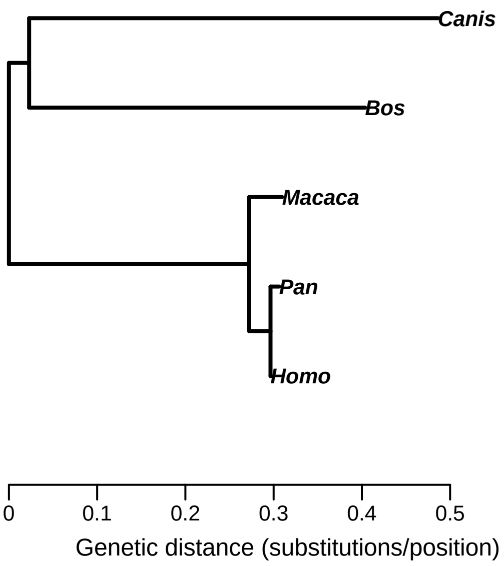

ENSG00000136878 intron 9

```
Homo    GTATCAGCTGGCCGGGG----ACTGCG---------------------------------------GGAGGAACCTCAGCCTATGGCCCAGTACCTACCG 57
Pan     GTATCAGCTGGCCAGGG----ACTGCG---------------------------------------GGAGGAACCTCAGCCTATGGCCCAGTACCTACCG 57
Macaca  GTATCAGCTGGCTGGGG----GCTGCG---------------------------------------GGAGGAACCTCAGCCTGTGGCCCAGTACCTACTG 57
Bos     GTACCAGCCAGCTGCGGGAGCACCGCGGCCCTCTTGGGCCGCAGCCCTACCCTGGGGGGTTTAGACGGGAAAGAATCTGCCTGCAGTGCAGGAGAC-CTG 99
Canis   GTGCCAGACAGCTGCTT-------------------------------------------------GGGGATCCCCACCCGCTTTGTCCAGCACGTCCCA 51

Homo    GGTGCTGAGCGCCGACCTGCAGTAGCCCCCGGGGGACGG-------------------GTTCTTCACTAGA---CCCGAATGACAGTGG--------GGA 127
Pan     GGTGCTGAGCGCCGACCTGCAGTAGCCCCCGGGGGACAG-------------------GTTCTTCACTAGA---CCCGAATGACAGTGG--------GGA 127
Macaca  GGTGCTGAGCGCCAACCTGCAGTGGCCCCCAGGGGATGG-------------------GTTTTTCACTAGA---CCCGAATGACAGTGG--------GGA 127
Bos     GGTTTGATCCATGGATCTGGAAGGTCCCCTTGAGAAGGGAATGGCAACCCACTCCAGTATCCTTGCCTGGAGAGTCCCATGGACAGAGGAGCCTGGTGGG 199
Canis   CGTGCCAGGCACCGACTTGTGCTGGCCCGCTGGCCT----------------------GTCCCTCCCTGTAGGGCCCC-------------------CCA 110

Homo    AGTCAGCCTCCGGGGG------------------GGCTCAGTGAT--GTGCCCAGGGTCCCCTAGCTCATAATGCT------------------------ 183
Pan     AGTCAGCCTCCGGGGG------------------GGCTCAGTGAT--GTGCCCAGGGTCCCCTAGCTCATAATGCT------------------------ 183
Macaca  AGTCAGCCT-CGGCGG------------------GGGTCAGTGAT--GTGCCCAGGGTCCCCTAGCTCATAACGCT------------------------ 182
Bos     CTACAGTCCATGGGGGTCGCAAAGAGTCGGACACGACTCAGTGACTAACACTAGGGGGCTGCTCTCCCACTAGACCTGGCCTGTAGGTGAGCAGTCAGGT 299
Canis   TCAGAGCCCCTCTGGG------------------------GCGAGGACCACGTAGTGTGCAGACTCACTCAGGA-------------------------- 160

Homo    ---------------------GGAGCCCAATTCAGAGCCCTCTCCCAGAGCCCTGAGCCTTCCAGCCGGACATGGATTGGACAGGT-CTGCTCCGGCCTG 261
Pan     ---------------------GGAGCCCAATTCAGAGCCCTCTCCCAGAGCCCTGAGCCTTCCAGCCAGACATGGATTGGACAGGT-CTGCTCCGGCCTG 261
Macaca  ---------------------GGAGCCCAATTCAGAGCCCTCTCCCAGAGCCTTGAGCCTTCC-GCCGGAAGTGGAATGGACAGCT-CTGCTCCGGCCTG 259
Bos     GAATAGCATGTCACTTGGTGAAGGAGTGAGATAGGAGCCTTCCCCGAG---TTCAAGCCCCCAGCGGGAATGGGCAGTGTGTGGACACAGGTCCTGTGTA 396
Canis   ---------------------GGGGTGTGGCTC--AGCCTGCTCTGTG--------GCCCCCACTGGACACGAGGACATGTGTCCTGCAGGTTTGG--TG 227

Homo    TGTGCTGGGGCCAGGAGTGGGCACATGACAGAGGAGACACATTAGGGTCAGGCTTTCGTCCCTTTAGGAGGGGCTGGCAGGTCATCTTCCTGCCCACT-C 360
Pan     TGTGCTGGGGCCAGGAGTGGGCACATGACAGAGGAGACACATTAGGGTCAGGCTTTCGTCCCTTTAGGAGGGGCTGGCAGGTCATCTTCCTGCTCACT-C 360
Macaca  TGTGCTGGGGCCAGGAGTGGGCACGTGACAGATGAGACACATTGGGGTCAGGCCTTCGTCCCTTTAGGAGGGGCTGGCAGGTCATCTTCCTGCCCACT-C 358
Bos     GGGGCTGGGGAGTGGGCTGGGCTCAGGACAGAGGAGACGTGGTGGGTTCAGGCCTTGGTGCCCTTCGGAGGTGCTGGAGG----TCTTCTTGACCAGG-C 491
Canis   GGCACCAGGGCGGCGGG-GGTCAAAAGGCAGAGGACACCTGGTGGGCTCTGGCCTCGGGCCAC--AGTAGATGCTGGCAGGTCCCCTTCCTGATCAGGCC 324

Homo    GTGGAGCTGGCCTGGCTGCCCTGGCCCAGCATGGT-ACCCTCTCTGCCCCCAG 412
Pan     GTGGAGCTGGCCTGGCTGCCCTGGCCCAGCATGGT-ACCCTCTCTGCCCCCAG 412
Macaca  ACGGAGCCGGCCTGGCTGCCCTGGCCCAGCATGGT-GCCCTCTCTGCCCCCAG 410
Bos     TAGGGGCTGGCTCAGCCGCCCAGCACTGTCCCCCTCGCTCCGTCCGCCCCTAG 544
Canis   ACGGAGCTGGAGTGGCTCCCCAGGCCCAGCAATGGTCTCCTGTCTGCCCGTAG 377
```

ENSG00000136449 intron 11

Description: Myc-binding protein-associated protein (MYCBPAP)
Intron number: 11
Human chromosome: 17
Intron start (bp): 45955489
Human intron length : 468
Intron alignment length: 514
Flanking exons length (upstream/downstream): 190/188
SNP density: 0.002137
K tree score: 0.0699
Scaling factor: 0.7742
Human-chimpanzee distance: 0.006509
Total primate branch length: 0.0756

ENSG00000136449 exon 11

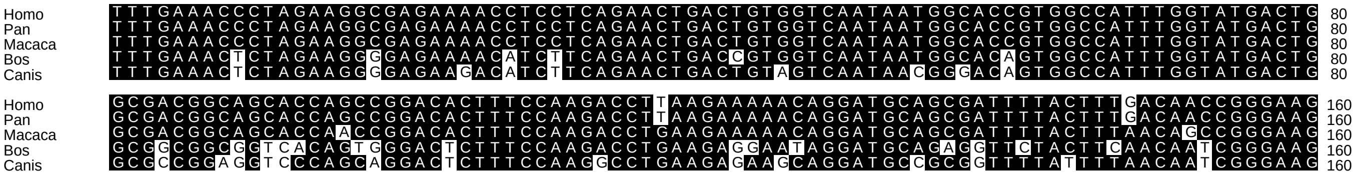

ENSG00000136449 exon 12

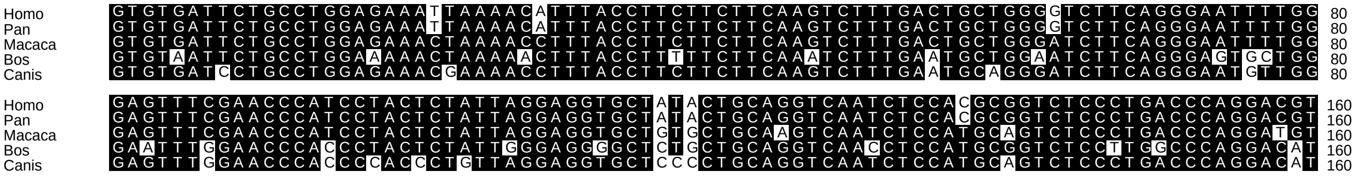

ENSG00000136449 intron 11

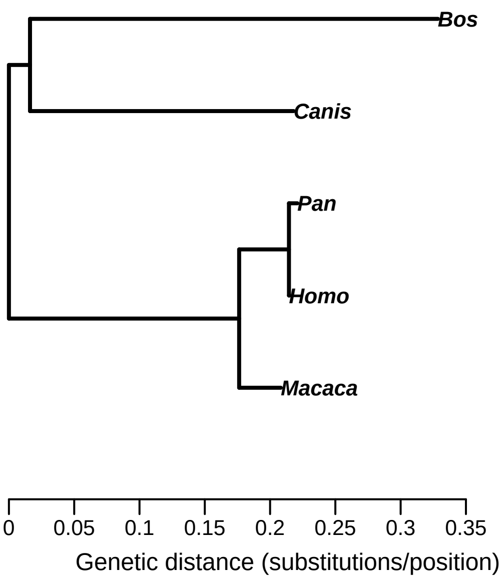

ENSG00000136449 intron 11

```
Homo    GTACTCGGGAGAAGCCACCCTATGTGCTAGCTCCTGTCTGGGGCTGGTTTTGTCCTCCGTGAGACATCAAAGTTTAGTTATGGCCCAGCTCCTGTAAGTT 100
Pan     GTACTCAGGAGAAGCCACCCTATGTGCTAGCTCCTGTCTGGGGCTGGTTTTGTCCTCCGTGAGACATCAAAGTTTAGTTATGGCCCAGCTCCTGTAAGTT 100
Macaca  GTACTTGGGAGAAGCCACCCTACACGCTGGCTCCTGTCTGGGGCTGGTTTTATCCT-CGTGAGACATCAAAATTTAGTTATGACCCAGCTCCTGTAAGTT 99
Bos     GTACCAGGGGGAAGCTGCCC-----ACTCACCCCTCCT------------------------ACAGCCCAAGTCTCGCTCATGCCCAGGCCCTGCAAGCA 71
Canis   GTACCT------AGCTGCACTGCTCCCTGGCTTTTGCC----------CTCGTTCT--GTGAGGAGCCAGAGTTTGGCTG-----ATGCTCCTGAAAGTT 77

Homo    TGAGCCCTGCTTCCCTGCCTTGTCCCTCTCATTCCTTCTGTGAAATGTG---TGGCCTGACTACTCATGTCTGTTGAGAGACTGATGGAA--CTTGAGAA 195
Pan     TGAGCCCTGCTTCCCTGCCTTGTCCCTCTCATTCCTTCTGTGAAATGTG---TGGCCTGACTACTCATGTCTATTGAGAGACTGATGGAA--CTTGAGAA 195
Macaca  TGA------------------GTCCCTCTCATTCCTTCCGTAAAATGTG---TGGCCTGACTATTCATGTCTGTTGAGAGACTGACGGAA--CTTGAGAA 176
Bos     TGAGCCCCTCTTCCCA--CCTGTCCACCCCATGTCTTCCATGAAGTGTGCCGTCTC----CTGTTCCCATCAGCTGGGGGAATGGCAGAA---------- 155
Canis   TGAGCCCCCCTCCCTGGCCTCATCCCTCCCATTTCTTCCATGAATTGTGTCCTCTCCCAACTGTCCATGTCTTTTGGTGGAACGGTCATACTCTTGAGAT 177

Homo    CCTGGGGAGGTGAGCCACCAGGGAGCAGAAGCGTTCTAGGGGCTCAGTTATCTAGGTGCACAGGCCTGGGGCTAGGATGGCCAGCTGTCTCTGTTTGCCT 295
Pan     CCTGGGGAGGTGAGCCACCAGGGAGCAGAAGCGTTCTAGGGGCTCAGTTATCTAGGTGCACAGGCCTGGGGCTAGGATGGCCAGCTGTCTCTGTTTGCCT 295
Macaca  CCCGGGGACGTGAGCCACCTGGGAGCAGAAACGTTCTAGGGGCTCAGTTATCTAGATGCACAGGCCTGGAGCTAGGGTGGCCAGCTGTCTCTGTTTGCCT 276
Bos     --CGCAGGAGTGAGCCACCAGGG-GCAGAAA-------------CAGTTGCCCATATACCTGGGCCTGAGGCTAGAG----------------------- 216
Canis   CACAGAGAGGAGAGTCATCAGGGAGCAGAAACTTTCTAGAGGTTCAGTTACCTATATGCTCAGACCTGAGGCTAGGA--ACCCTCAATCCCTGTTTGCCT 275

Homo    GGGACTTT----------------AGCACTCAAATTCCCACATTCCAGGAAACCTCTCAGCCCTGGGCAAACTGGGATGGTTGGCCAGCCACCTGGCCTG 379
Pan     GGGACTTT----------------AGCACTCAAATTCCCACATTCCAGGAAACCTCTCAGCCCTGGGCAAACTGGGATGGTTGGCCAGCCACCTGGCCTT 379
Macaca  GGGACTTT----------------AGCACTCAAAATCCCCCTTTCCAGGAAACCTCTCAGCCCTGGGCAAACTGGGATGGTTGGCCAGTCACCTGGCCTG 360
Bos     -----------------------------------TCCCACATCCCAAGAACGCCCTCAGGTCTTCACAAGCCGGGACA----GCTGGTCACCCTAACCA 277
Canis   GAGACTTCCCCAGTTTTTGCACAGAAAGTTGAAAGTCCTACGTCCCAGGAGACCCCTTGGTTCTGGGCAAATCGGGATGGT--GTCGGTCACTCTGCCTG 373

Homo    GGACTCAGTTAACTGCATGTAGGA-------------------------GCATGTAGGCCTGGGGCTCAGCCAGCTCCTGCATGTTGAAAACCTCGGGTC 454
Pan     GGACTCAGTTAACTGCATGTAGGA-------------------------GCATGTAGGCCTGGGGCTCAGCCAGCTCCTGCATGTTGAAAACCTCGGGTC 454
Macaca  GGGCTCAGTTAACTGCATGTAGCA-------------------------GCATACAGGTCTGGGGCTCAGCCAGCTCCTGCATGTTGAAAACCTC-AGTC 434
Bos     AGGCTTAGTTAACTTTCT-------------------------------ACCCACAGGCTTGGGGCTCAGC-AGCTCCCACCTGGTGAAAAACCTCTGTC 345
Canis   GGGCTCAGTTAACTGTGTATGCAAAGGTTTAGGGTTCAGTTAACTTTATACACACAGGCTTAAGGCCCAGCTGACTCCTGTGTGGTGAAAACCTC-TGTC 472

Homo    TCTGTCCCTCTCAG 468
Pan     TCTGTCCCTCTCAG 468
Macaca  TCTGTCCCTCTCAG 448
Bos     TCTGTCCCTCTCAG 359
Canis   TTGGTCCCTCTCAG 486
```

ENSG00000151224 intron 8

Description: S-adenosylmethionine synthetase isoform type-1 (MAT1A)
Intron number: 8
Human chromosome: 10
Intron start (bp): 82023620
Human intron length : 636
Intron alignment length: 780
Flanking exons length (upstream/downstream): 134/103
SNP density: 0.003145
K tree score: 0.0884
Scaling factor: 0.7006
Human-chimpanzee distance: 0.017546
Total primate branch length: 0.0755

ENSG00000151224 exon 8

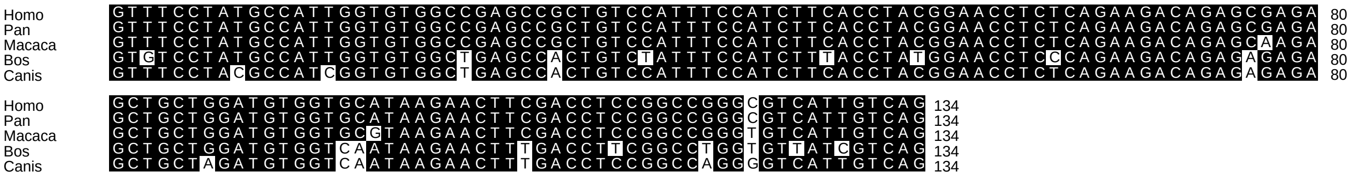

ENSG00000151224 exon 9

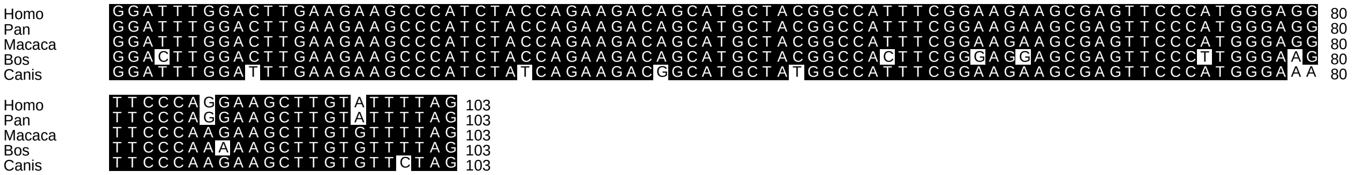

ENSG00000151224 intron 8

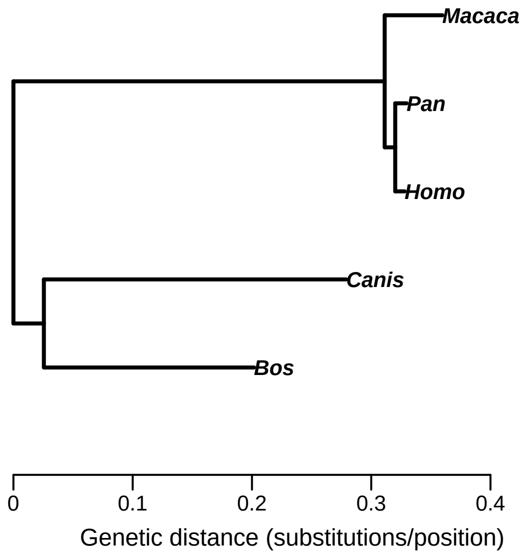

ENSG00000151224 intron 8

```
Homo    GTAAAGTGCCATGCGCTGCTATTACA--CAGGACCTTGCTCCTTCCTTGCCCAGATGCTCACCTCACCTGAAAGGGAGGCAGAAAGGAGGATGTTTCTGC 98
Pan     GTAAAGTGCCATGCGCTGCTATTACA--CAGGACCTTGCTCCTTCCTTGCCCAGATGCTCACCTCACCTGAAAGGGAGGCAGAAAGGAGGATGTTTCTGC 98
Macaca  GTAAAGTGCCATGCTCCGCTATTACA--CAGGACCTTGCTCCCTCCTTGCCCAGTTGCTCACCTCACCTGAAAGGGAGGCAGAAAGGAGGATGTTTCTGC 98
Bos     GTAAAAGGGCCGCCCCTGCAGTTG-GGCAAAGCC----CTCTTTCCCCACCTGGTGCTT--GCTTATTGGAAAGAGAATCAGAAGGAAGGAT-GTTCAGC 92
Canis   GTAAGAGTGCCACCACTGTCACTGCTGCCCGGCCCTTGCTCCTCCCTCGCCCAGAGCCTCGGCTCCCCTGGAAGGGAAGCAGGGGGAAGGAC-TTTGAGC 99

Homo    CTCTCAGAAGTGGGAGGGTCTGTGAGCTCTTGGTACAGCACAGTGC--ATCCCCAGTGCAGAAAGCCTGGTTGTCAGGTTAAAGGAGGCTGCCCCAGGTT 196
Pan     CTCTCAGAAGTGGGAGGGTCTGTGAGCTCTTGGTCCAGCACAGTGC--ATCCCCAGTGCAGAAAGCCTGGTGGTCAGGTTAAAGGAGGCTGCCCCAGGTT 196
Macaca  CTCTCAGAAGTGGGAGGGTCTGTGAGCTCCTGGTACAGCACACTGC--ATCCTCAGGGCAGAAAGCCTGGCTGTCAGGTTGAAGGAGGCCGCCCCAGGTT 196
Bos     CTCCCAGAAGCAGGAGAGGCTTCAAGTTCTTGGCGCAGCACAGTGTAAACCCCAAATGCAGAATGTCTAGTTTTCAGGTTGAAGGAGCCC---------- 182
Canis   CTCCCCGAAGCAAGAG-GGCCTGCGGCCCCTGGCCCAGCACAACACACACCCCAAATGCAGGATGTCTGCTTTTCGGGCGGAAGGAGGCTGTGTAAGGTT 198

Homo    CCTGCTGGCCCAGTCAACACCAACTCCAGTGGGGCCATCTCTGAGCAACTGGGAGGCAGGCGGGGATCTTAGGAAAATAATTATTAATATAAGGCCAAAT 296
Pan     CCCGCTGGCCCAGTCAGCACCAACTCCAGTGGGGCCATCTCTGAGCAGCTGGGAGGCAGGCGGGGATCTTAGGAAAATAATTATTAATATAAGGCCAAAT 296
Macaca  CCTGCTGACCCAGTCAACACCAACTCTGGTGGGGCCATCTCTGAGCAGCTGGGAGGCAGGCGGGGATCTTAGGAAAATAATTATTAATATAAAGCCAAAT 296
Bos     ---------------AGCATTGACTTCAGGGGGCCCATCTTTGAGCAGGTG----------GAGGATCTTAGGAAGATAACT---------------GCA 242
Canis   CCTACTAGCCCAG-AGACCCCGACGCCAGTGAGGCCATCCTGGAGCAGGTG----------GGGGATCTTAGGAAAGTCACC---------------AGA 272

Homo    AGCTCAAATGGCTGGGAATCAAGTCAGCAAGTCT------------------------------ATTCC--GTTTCTAGAGCTTAGTGGTGTCAACATTG 364
Pan     AGCTCAAATGGCTGGGAATCGAGTCAGCAAGTCT------------------------------ATTCC--GTTTCTAGAGCTTAGTGGTGTCAACATTG 364
Macaca  GGCTCAAATGGCTGGGAATCGAGTCAGCAAGTCT------------------------------ATTCCCTGTTTCCAGAGCTTGGTGGTGTCAACACTG 366
Bos     AGACCGGATGGGGGGGAATTGAGTCAGCAAGTCTGGCCCCAACTGCTCTCGCTTCTAATGCC--ACCCCTGCTTCTTGGAGCTT-GCGGTGTCGATGTTG 339
Canis   GGGCCGAAT-GCTGAGAATTGAGCCGGCTGGTCCGG-CCCAGCTGCCCTTGCTTCTGATGCCGTATCCCCACTTTCTGGAGCTT---GGCGACGCAGTTG 367

Homo    GCCTGGGCCAGGTAAGTGGAGTGGG-------------------------------------------------------AGGAGAAAAGCACCGGAAGG 409
Pan     GCCTGGGCCAGGTAAGTGGAGTGGG-------------------------------------------------------AGGAGAAAAGCACCGGAAGG 409
Macaca  GCCTGGGCCAGGTAAGTGGAGTGGG-------------------------------------------------------AGGAGAAAGGCATCCGAAGG 411
Bos     GGCTGTGCCAGGTCAGTGGAGCAGA--------------------------------------------------------------------GTGTAGG 371
Canis   GTGTGCTCCAGGCCGGTGGAGTGGATGGAGAGGAGAGGAGAGGAGAGGAGTGGAGTGGAGAGGAGTGGAGAGGAGAGGAGAGGAGAGGAGCCCAGGGAGG 467

Homo    CTGGAGTT-AGATCCTG------------------------------------GTCCCTGAACCTCTAA-------------GCATCCGTGGGGCTATAA 459
Pan     CTGGAGTT-AGATCCTG------------------------------------GTCCCTGAACCGCTAA-------------GCATCCGTGGGGCTATAA 459
Macaca  CTGGAGTT-AGGTCCTG------------------------------------GTCCGTGAACCTCTGA-------------GCATCCGTGGGGCTATAA 461
Bos     CCAGAGTTCAAATCCTGATTCCTGGTGCATCTGTCTGTCTGGTTGATCAGGCTGTCCTGGAACC-CTGAGACTGTTTCTTAAGCGCCAGTGGCAGTCACC 470
Canis   CCAGGGCTCAAGGTCTGGGGCCTGGGGTGTGTGTC--------TGATCGGACTGTCTCCGAGCCTCTGAGCCTGTTTCCTTCGGGCCGGTGGTAGTTCCC 559

Homo    AACGAGACCAGGGTATGGCACAGATAGGATGGGTGCGCGCAACTGCGGTGGGGAGGCGTAGTGCCATGCGAGGAGGGCTGTGTTACAGTTCGTTGCTCCC 559
Pan     AACGAGACCAGGGTATGGCACAGATAGGATGTGTGTGTGCAACTGCGGTGGGGAGGCGTAGTGCCATGCGAGGAGGGCTATGTTACAGTTCGTTGCTCCC 559
Macaca  AACGAGACCGTGGTATGGCACAGATAGGATGTGTGTGTGCAACCGCTGGGGGGAGGTGTAGT-------GAGGAGGGCTGTGCTACAGTTCATTGCTCCC 554
Bos     TTGTAAGGTGTGGCATAGCAGAGATAGGAAATTTAGGGAGAATTGCTCTTGAGTGGTTAATACCCCAC----------ACATTCATAGTCTGTTAATTAC 560
Canis   GCAGAAGATGTGGTGTGGTGTAGACAGCAAGTTGATGGGAGATCGCTC-AGAGTAG-TGACACTCCACGTGGACGAAGACCATCACACACTGTTAATTAT 657

Homo    TCCTGTCCTTGAAATGGAGAAAGA---AACCCTGCTCCTGTTCCATCTTCCAATGCCCTTCCTTTCTGCTGGACATGCAG 636
Pan     TCCTGTCCTTGAAATGGAGAAAGA---AACCCTGCTCCTGTTCCATCTTCCAATGCCCTTCCTTTCTGCTGGACATGCAG 636
Macaca  TCCTGTCCTTGAAATGGAGAAAGA---AACCCTGCTCCTGTTCCATCTTCCAATGCCCTTCCCTTCTGCTGGACATGCAG 631
Bos     TCCTGTGCCCCAAATTCAGGGAAA-GACACTCCACTCCTTTTCCAT-TAAAACTGACCCTTTGTTCTACTGAACGTGCAG 638
Canis   TCCTGTGCCCAAAATTGAGAAAAACTGTTCCTTTTCCCTTTTCCTT-TTAAAATGACCCTTTTCTGTGCTGACTGTGCAG 736
```

ENSG00000094755 intron 1

Description: Gamma-aminobutyric-acid receptor subunit pi precursor (GABRP)
Intron number: 1
Human chromosome: 05
Intron start (bp): 170148251
Human intron length : 460
Intron alignment length: 679
Flanking exons length (upstream/downstream): 53/119
SNP density: 0.008696
K tree score: 0.0689
Scaling factor: 0.9026
Human-chimpanzee distance: 0.004369
Total primate branch length: 0.0755

ENSG00000094755 exon 1

```
Homo    ATGAACTACAGCCTCCACTTGGCCTTCGTGTGTCTGAGTCTCTTCACTGAGAG 53
Pan     ATGAACTACAGCCTCCACTTGGCCTTCGTGTGTCTGAGTCTCTTCACTGAAAG 53
Macaca  ATGAACTACAGCCTCCACTTGGCCTTCGTGTGTCTGAGTCTCTTCACTGAAAG 53
Bos     ATGAAGCGCAGTCTCCACCTGACCTTCGTGTGTCTGAGTCTCTTCTCTGCACG 53
Canis   ATGAAATATAGTCTCTACTTGACCTTCATGTGTCTGAGTCTCTTCACTCCAAG 53
```

ENSG00000094755 exon 2

```
Homo    GATGTGCATCCAGGGGAGTCAGTTCAACGTCGAGGTCGGCAGAAGTGACAAGCTTTCCCTGCCTGGCTTTGAGAACCTCA 80
Pan     GATGTGCATCCAGGGGAATCAGTTCAACGTCGAGGTCGGCAGAAGTGACAAGCTTTCCCTGCCTGGCTTTGAGAACCTCA 80
Macaca  GATGTGCATCCAGGGGAATCAGCTCAACGTCGAGGTCGGCAGAAGTGACAAGCTTTCCCTGCCTGGCTTTGAGAACCTCA 80
Bos     GATGTGCGTCCAGGGGAATCAGTTTAACATCGAGGTCAGCAGAAGCAACAAGCTGTCCCTGCCTGGCTTTGAGAATCTCA 80
Canis   GATATGTGTCCAGGGGAATCAGTTCAACATCGAGACCAGCAGAAGTGACAAGCTCTCCCTGCCTGGCTTTGAGAATCTCA 80

Homo    CAGCAGGATATAACAAATTTCTCAGGCCCAATTTTGGTG 119
Pan     CAGCAGGATATAACAAATTTCTCAGGCCCAATTTTGGTG 119
Macaca  CAGCAGGATATAACAAATTTCTCAGGCCCAATTTTGGTG 119
Bos     CAGCAGGATATAACAAGTTTCTCAGGCCCAATTTTGGCG 119
Canis   CAGCAGGATATAACAAATTTCTCAGGCCAAATTTTGGCG 119
```

ENSG00000094755 intron 1

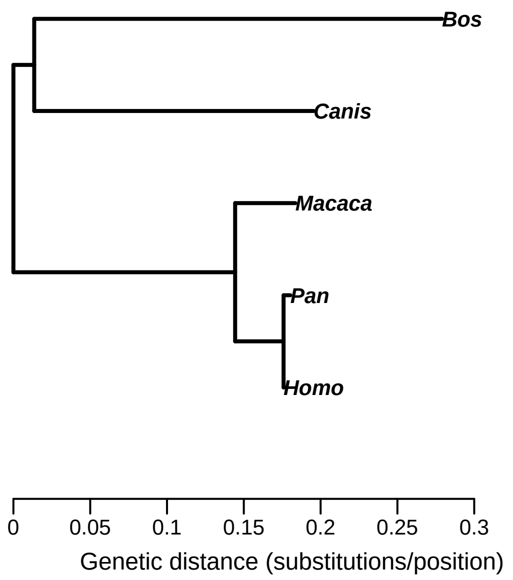

ENSG00000094755 intron 1

```
Homo    GTGAGCTTTGCTACCCCCAGAATGGCCTCCATC-TGCGTTGCTTTGCATTCGGGCATGTGGTGGGAGGG--GGCAGCTCCTCCTATTCACCCACAGCAAG 97
Pan     GTGAGCTTTGCCACCCCCAGAATGGCCTCCATC-TGCGTTGCTTTGCATTCGGGCATGTGGTGGGAGGG--GGCAGCTCCTCTTATTCACCCACAGCAAG 97
Macaca  GTGAGCCTTGCTACCCCCAGGATGGCCCCCATCTTGAGTTGCTTTGCATTCAGGCATGTGGTGGGAGGG--GCCAGCTCTTCCTACTCACCCACAGCCAG 98
Bos     GTGAGCTCTGCCA--CCCACAGTGGCCTTGGTC-TGAACTGCTCTGTGTTT-TGCACTCGGTGGGAGGGGTGGCAGTCCTTTCTACTTACCGACGACAAG 96
Canis   GTGAG-TTTGCTG-CCTCACAATGACCTGGGTCTGAATTGTTTTTGCATATGTGCTCAAAATAGGAAGAGTGGCAGGTCCTCCTACTTTCCAACTGCAAG 98

Homo    GCAAAACCCGGTCCACTCACTTCCCTGGAGCA-CCGAACAATGAAACTTCCTGCCATT-TACTCTATTTACAGAACACAAGCTTCCAATATGGTCATAGA 195
Pan     GCAAAACCCGGTCCACTCACTTCCCTGGAGCA-CCGAACAATGAAACTTCCTGCCATT-TACTCTATTTACAGAACACAAGCTTCCAATATGGTCATAGA 195
Macaca  GCAAAACCTGGTCCCCTCACTTCCCTGGAGCA-CCGAACAATAAAACTTCCCGCCATG-TACTCTCTTTACAGAACACAAGCTTCCAATATGGTCATAGA 196
Bos     ACAGATCCTAGTGCCTTCACCCCGCTGACGCA-CCAGGACGTACAGTGTCATGCCATGTTTTTCCGTCTGGAGAAGTCAAGCTTCCAAAATGGTCAGAGA 195
Canis   ACAAAGCCTAGTCCATTCATCTCCCTGATATACCCAGACAACACAATTTCCTGCCATG-TATTTTATTTGGAGAATGCAAGCTTCCAAAATGGTCATAGA 197

Homo    TGAATG-AACCCTTATGTTTTTAAAG-AAGAGACTTGGAGGGTTTC-CAAATTACCATGATATTTTGATCTATGGCAAATGGGTAA-AAAATGGAG---- 287
Pan     TGAATG-AACCCTTATGTTTTTAAAG-AAGAGACTTGGAGGGTTTC-CAAATTACCATGATATTTTGATCTATGGCAAATGGGTAA-AAAATGGAG---- 287
Macaca  TGGATG-AACCCTTATGTTTTTAAAT-AAGACATTTGGAGGGTCCC-CAAATTACCATGATATTTTGATCTATTGCAAATGGGTAA-AAAATGGAG---- 288
Bos     TGGATAAATCCCTTTTGTTTTGGGGTGAAG--GTTTTGAGTGTTTC-CAAATTACCACGACATTATGATACATGGCAAATGCATAATAAAGTGGGGTGAG 292
Canis   TGGATGAAACCCTTGTGTTTTTTTCTAAAGACACTTGAAGGGTTTCTCAAATTACCTCAACATTCTGATGTATGGCAAATGGGTAATAAAATGGAGCAGG 297

Homo    --------ATA-CTAACTCAATAGCTGAAAACTAAGAGATTTTTCTGTAGGAATGTCACTGCTGGTACATGGGCAA------------------------ 354
Pan     --------ATA-CTAACTCAATAGCTGAAAACTAAGAGATTTTTCTGTAGGAATGTCACTGCTGGTACATGGGCAA------------------------ 354
Macaca  --------ATA-CTAACTCAATAGCTGAAAACTAAGAGATTTTTCTGTAGGAATGTCACTGCTGGTACATGGGCAC------------------------ 355
Bos     ATAGAGATATGTTTAATTCAGCAGCTGAAAATT-GGAGGGTTCTCTTTGGAAGAGTCACTGCTGGTACATGAGCACATGCACACGTATGTGCGTGCTTGT 391
Canis   ATAGGGACATG-TTAACTCAGCAGCTTAAAATT-AGAGATTTCTCTCTGGGCAAGTCACGGCTGGTACATGGGCAC------------------------ 371

Homo    ---------------------------------------------------------------------------------------------------- 354
Pan     ---------------------------------------------------------------------------------------------------- 354
Macaca  ---------------------------------------------------------------------------------------------------- 355
Bos     GTGCTCAGTTGTGTCTGACTCTTTGCAACCCATGAATTTTCCAGGCAAGAATACTGGAGTGGGTTGCCTTTTCCTACTCCAGAGTCCTGACCCAGGGATC 491
Canis   ---------------------------------------------------------------------------------------------------- 371

Homo    ---------------------------------------------------------------GGCACATACACCCACACACACGTGGGCAAACACAAGC 391
Pan     ---------------------------------------------------------------GGCACATACACCCACACACACGTGGGCAAACACAAGC 391
Macaca  ---------------------------------------------------------------GGCACATACATCCACACACACATGGGCAAACACAAGC 392
Bos     AAACCTGTGTCTCCTGCGTCTTTTGCATTGGCAAACAGATTCTTTACCACTGAGCCACTTGGAAGCCTATATAACCACACACATACATGCAAACACAAGC 591
Canis   ---------------------------------------------------------------AACACTTATACATGCAAACA-------GAGCTCAAGT 401

Homo    TCAAG---------GGTGTGTACACGTGTTGATTCACACATAAACAAGCAAACACAACAAAGCCCATTTCTT-TTCCAG 460
Pan     TCAAG---------GGTGTGTACACGTGTTGATTCACACATAAACAAGCAAACACAACAAAGCCCATTTCTT-TTCCAG 460
Macaca  TCAAG---------CGTGTGTACACGTGTGGATTCACACATAAGCAAGCAAATGCAACAAAGTCCATTTCTT-TTCCAG 461
Bos     TCAAGTTTCAAGTTTGTGTGTGTGCATGTTGACCCGCACATCAGCACACATGTACACTGACACCTGTTTTCTTCTGCAG 670
Canis   TTA-------------TGTGTATACGTGCTTATTCACACATAAATAAGCACGTGCACCAACATCCATTTTCTGTTCCAG 467
```

ENSG00000127129 intron 3

Description: Endothelin-2 precursor (EDN2)
Intron number: 3
Human chromosome: 01
Intron start (bp): 41719452
Human intron length : 1272
Intron alignment length: 1518
Flanking exons length (upstream/downstream): 123/99
SNP density: 0.012579
K tree score: 0.0814
Scaling factor: 1.0786
Human-chimpanzee distance: 0.007927
Total primate branch length: 0.0755

ENSG00000127129 exon 3

```
Homo    ACAGACAGCTCCTTACGGCCTGGGAAACCCGCCAAGACGCCGGCGCCGCTCCCTGCCAAGGCGCTGTCAGTGCTCCAGTG 80
Pan     ACAGACAGCTCCTTACGGCCTGGGAAACCCGCCAAGACGCCGGCGCCGCTCCCTGCCAAGGCGCTGTCAGTGCTCCAGTG 80
Macaca  ACAGACAGCTCCTTACGGCCTGGGAAACCCGCCAAGACGCCGGCGCCGCTCCCTGCCAAGGCGCTGTCAGTGCTCCAGCG 80
Bos     ACAGACAGCTCCTTACGGCCTGGGAAACCCGCCAAGACGCCGGCGCCGCTCTTCTGCCAAGACGCTGTGAGTGCTACAGTG 80
Canis   ACAGACAGCTCCTTACGGCCTGGGAAACCCGCCAAGACGCCGGCGCCGCTCCCTGCCAAAGCGCTGCGAATGCTCCAGTG 80

Homo    CCAGGGACCCCGCCTGTGCCACCTTCTGCCTTCGAAGGCCCTG 123
Pan     CCAGGGACCCCGCCTGTGCCACCTTCTGCCTTCGAAGGCCCTG 123
Macaca  CCAGGGACCCCTCCTGTGCCACCTTCTGCCTTCGAAGGCCCTG 123
Bos     CCAGGGACCCCGCCTGTGCCACCTTCTGCCATCAAAGGCCCTG 123
Canis   GCGGGGACCCCGCCTGTGCCACCTTCTGCCATCGACGGCCCTG 123
```

ENSG00000127129 exon 4

```
Homo    GACTGAAGCCGGGGCAGTCCCAAGCCGGAAGTCCCCTGCAGACGTGTTCCAGACTGGCAAGACAGGGGCCACTACAGGAG 80
Pan     GACTGAAGCCGGGGCAGTCCCAAGCCGGAAGTCCCCTGCAGACGTGTTCCAGACTGGCAAGACAGGGGCCACTACAGGAG 80
Macaca  GACTGAAGCCCGGGCAGTCCCAAGCCGGAAGTCCCCAGCAGACGTGTTCCAGACTGGCAAGACAGGGGCCACTAGAGGAG 80
Bos     GACTGATGCGGTGGCAGTCCCAGGCAGCGGGTCCCCTGCAGCTGCGTTCCAGGATGGCAAGACACAGGCCACAGCAGGAG 80
Canis   GGCCGAAGCTGTGGTCGTCCCAGGCAGCAGGTCCCCCGCAGACGTGTTCCAGGCTGGCAGGACGTGGACCTCCGCAGGAG 80

Homo    AGCTTCTCCAAAGGCTGAG 99
Pan     AGCTTCTCCAAAGGCTGAG 99
Macaca  AGCTTCTCCAAAGGCTAAG 99
Bos     AGCTCCTCCAGCGGCTGAG 99
Canis   AGCTCCTCCGGCAGCTGCG 99
```

ENSG00000127129 intron 3

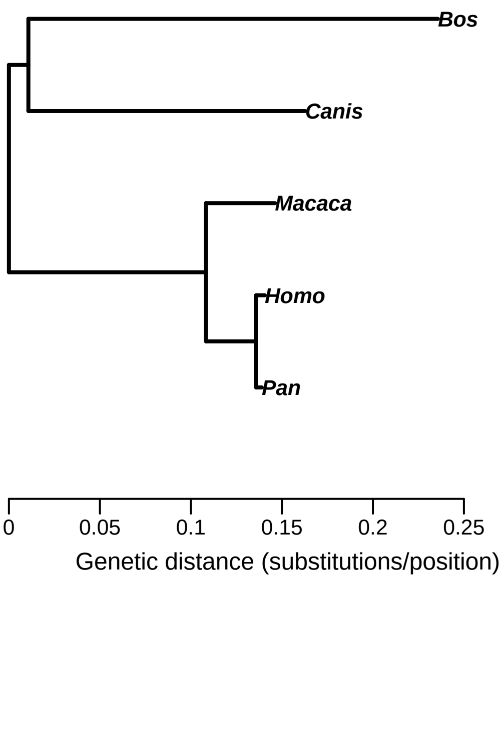

ENSG00000127129 intron 3

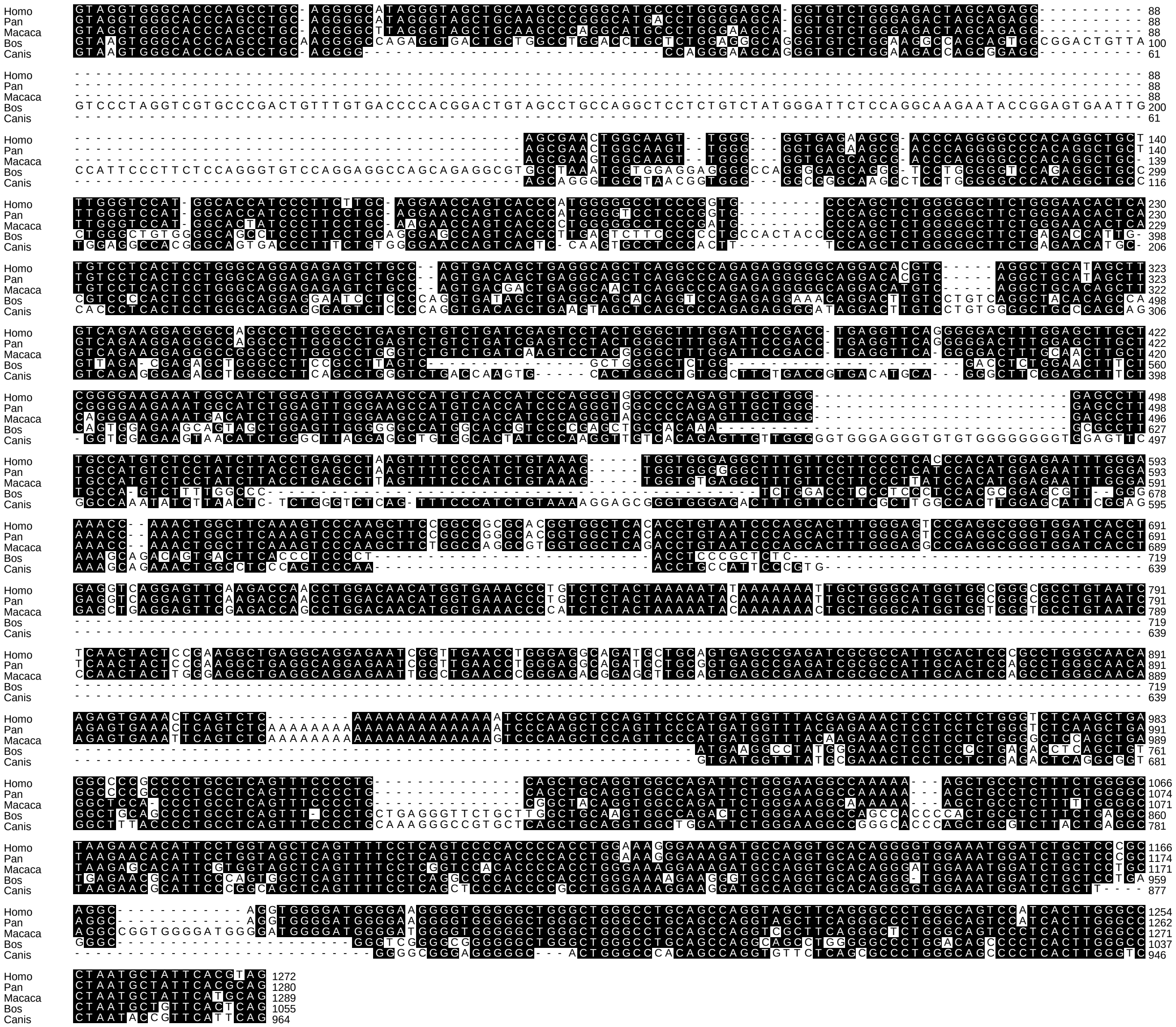

ENSG00000021826 intron 17

Description: Carbamoyl-phosphate synthase (CPS1)
Intron number: 17
Human chromosome: 02
Intron start (bp): 211178216
Human intron length : 1484
Intron alignment length: 2076
Flanking exons length (upstream/downstream): 145/211
SNP density: 0.003369
K tree score: 0.039
Scaling factor: 1.0958
Human-chimpanzee distance: 0.015106
Total primate branch length: 0.0753

ENSG00000021826 exon 17

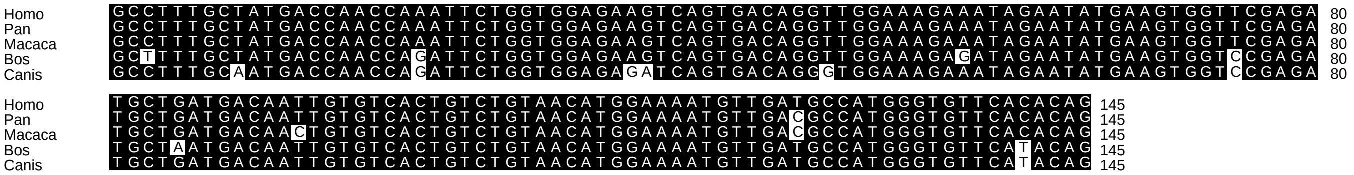

ENSG00000021826 exon 18

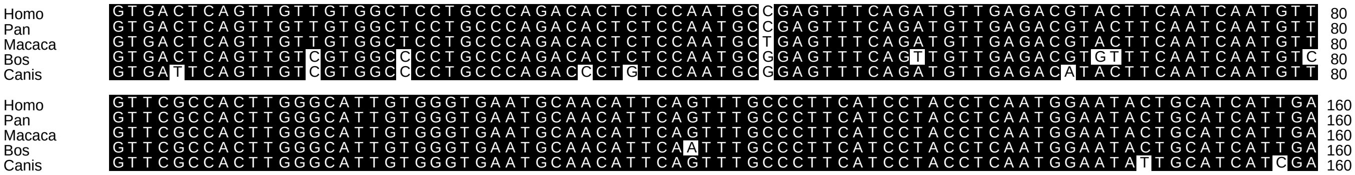

ENSG00000021826 intron 17

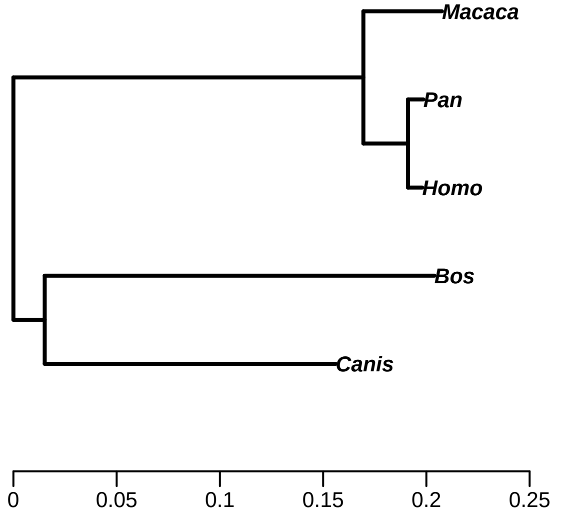

Genetic distance (substitutions/position)

ENSG00000021826 intron 17

Species	Sequence	Position
Homo	GTAGGCAAAGTATCTTCAAGAACTATAGTAATGCTTTCAGTTC---------ATGTCTTTGATGGC-CAAGGCAGTCTTTATAAAGTTGTTGCCTTTAAA	90
Pan	GTAGGCAAAGTATCTTCAAGAACTATAGTAATGCTTTCAGTTC---------ATGTCTTTGATGGC-CAAGGCAGTCTTTATAAAGTTGTTGCCTTTAAA	90
Macaca	GTAGGCAAAGCATATTCAAGAACTACCGCAATGCTTTCAGCTCATTTTAGCAATGTCTTTGATGGC-CATGGCAGTCTTTATAAAGTTGTTGTCTTTAAA	99
Bos	GTAGGCAAGGAATCTTCAAGCATTATGGTAATGCCTTCAACTC---------ATGTATCTGATGGCTCAAAGCACTTGTTATTAAGTTCCTTTCTTTAAA	91
Canis	GTAGGCAAAGAATCTTCGGGAATTATAGTAATGACTTCAGCTC---------ATGCTTCTGATGATGCGTAGCAGTCTTTATTAAGTTATTTTCTTTAAA	91
Homo	TTACTATTTCTAGTTGACAGACTAGTGATAACATTTTGGACAAGCATAGTATTTTACAGTATTGAAAATGTTCTCACATTTAATTTGATCTTCACACAAA	190
Pan	TTACTATTTCTAGTTGACAGACTCGTGATAACATTTTAGACAAGCATAGTATTTTACAGTATTGAAAATGTTCTCACATTTAATTTGATCTTCACACAAA	190
Macaca	TTACTATTTCTAGTAGACAGACTAGTGATAACATTTTGGACAAGTATAGTATTTTACAGTATTGAAAATATTCTCACATTTAATTTGATCTTTACACAAA	199
Bos	TTAGTATTCTTAGCGGGCACAATAGTGATACAATTGTATACATA---CATATTTCACAGGATTCAAAATACTCCCATATTTAATTTAATCTTCACAAAAA	188
Canis	TTACTATTCTTAGCTGACAGACTAGTGATAAAGTTTCACAGAAA-ATAGTATATCACAGTATTGAAAATAACCTCACATTCATTTTGATCTTCACAAAAA	190
Homo	CCCTGTGAAGTAAGGCGGA---TATTACACTGAGTTAATT--GAGGAAGAGCCAAAGGTAAGCAATATGGTAGAAATAAGATCCATAAAACTTAGTGTTG	285
Pan	CCCTGTGAAGTAAGGCAGA---TATTACACTGAGTTAATT--GAGGAAGAGCCAAAGGTAAGCAATATGGTAGAAATAAGATCCATAAAACTTGGTGTTG	285
Macaca	CCCTGTGAAGTAAGG----------AACCCTGATTTAACT--GAGGAAAAGCCA-----AAGCAATATGGTAGAAATAAGATCCATAAAACCTGATGTTG	282
Bos	CTCTCTGAAGTAGGGGATGTATTATTATCTTGATTTCACTTGAGGAAGGGGTCAAACATAAGCAATATAGTGAAGATGGAATATGTAAGACTTGATGTTG	288
Canis	CCCTGTGAAGTAAGGCAGG---TATTATCTTGATTTTATTTGAGGAAGAGGTCAAATGTAAGCAATG-AGTGATGGTAAAATCTGTAAGACTTGGTGTTG	286
Homo	AGAAATAAGAAGGGAGGTTGAGACAGTTTAAA--GCAGACTGAGCTGGTAATCACCCAGTAAATGAGAAAATAGGCACAA-TAATAG-------------	369
Pan	AGAAATAAGAAGGGAGGTTGAGACAGTTTAAA--GCAGACTGAGCTGGTAATCACCCAGTAAATGAGAAAATAGGCAGAA-TAATAG-------------	369
Macaca	AAAAATAAGAAGGGAAGTTGAGAAGGTTTAAA--GCAGACTGAGATGTTAGTCACCCAGTAAATGAGAAAATAGGCACAA-TAATAG-------------	366
Bos	AGAAGTAGGGCATGAGATTAAGAAGGCTTAAA--GCAGACTGAG-TTTTAGTCACCAGGTGAATAAGAAAATAGCCACAGTAAATAGAAATAGATGAAAT	385
Canis	AAAAGAA----ATGAGGTTAAGAAGGCTTAAATGAGAGGATGAG-TATTAGTTACCACGTGAATGAAAAAAGGGCCACAAGTAATAGAAACAGGTAAAAT	381
Homo	--	369
Pan	--	369
Macaca	--	366
Bos	--	385
Canis	AAGTTTTTTTTTGGTAAATTTTATTTATTTATTTATTTTTAATAATATTTTTTTTTATTGGTGTTCAGTTTGCCAACATACAGAATAACACCCAGTGCTC	481
Homo	--	369
Pan	--	369
Macaca	--	366
Bos	--	385
Canis	ATCCCGTCAAGTGCCCCACTAAGTGCCTGCCACCCAGTCACCCCACCCCCCGCCATCCTCCCCTTCCATCACCCCTAGTTCGTTTCCCAGAGTTAGGAGT	581
Homo	---------------------AAATAAGTTTTTCAGAAAAGTACACTAGTTAGATAAGAGGGCAAAGATGATAACTTAGGATTTAAAAGTGCTAAATTTG	448
Pan	---------------------AAATAAGTTTTTCAGGAAAGTACACTAGTTAGATAAGAGGGTAAAGATGATAACTTAGGATTTAAAAGTGCTAAATTTG	448
Macaca	---------------------AAATAAGTTTTTCAGGGAAGTAAATTAGTTAGATAAGAGGGCAAAGATGATAACTTAGGATTCAAAAGTGCTAAATTTG	445
Bos	---------------------AAATAGGTTTCTCAGCAAGTGAAGCTAGTTAGACAAGAGGGCAAAGATGATGACTTAGGATTTAGAAATGCTAAATTTG	464
Canis	CTCTCCTGTTCTGTCTCCCTAAAATAAGTTTTTTAGGAAGAGAAACTAATTAGATAAGACGGCAAAAATAATAACTTAGGATTTAGAAATGCTAAGTTTG	681
Homo	GGGTGTGAGCAGAGCTGTCAAAAAAGGGAGTTGAGAATGTGGAGATGGAATTTAAGAAAT-AAAATAGAAATAAATAAGTTGATACAAAGACACTGGAAA	547
Pan	AGGTGTGAGCAGAGCTGTCAAAAAAGGGAGTTGAGAATGTGGAGATGGAATTTAAGAAAC-AAAATAGAAATAAAGAAGTTGATACAAAGACACTGGAAA	547
Macaca	AGGTGTGAGCAGAACTGTCAAAAAAGGGAGTTGAGAATGTGGAGATGGAATTTAAGAAAT-AAAATAGAAATAAAGAAATTGATACAAAGACATTGGAAA	544
Bos	AAGGGTGGGTGGAAATCTC-TAAAAGAGGTTTGAGAATATGGAGTTGTAATTCAAGAAAA-AGGATAAAAATAAAGAAGTTAATGCAAGGTTATTGGAAG	562
Canis	AGGCATGGGTGGAATTCTC-AAGAAGAGGGTTGGTAATAGGGAGCTGGAATTCAAGAAAAGAGAATACAAATAAAGATGCTGATACAAAGTCATTGGGAG	780
Homo	CTGTTGGCATAAAAGGGGAAATTGAAGTTGTAGGCATAGACAAAGTATTCAAGGACATAGGTATAGAAAGAGGAGAGAATAGGGTTGAACTTTGAACTCT	647
Pan	CTGTTGGCATAAAAGGGGAAATTGAAGTCGTAGGCATAGACAAAGCATTCAAGGACATAGGTATAGAAAGAGGAGAGAATAGGGTTGAACTTTGAACTCT	647
Macaca	CTGTTGGCATAAAGGGGATAATTGAAGTCATAGGCATAGACAAAGTATCCAAGGGCATAGGTATAGAAAGAGGAGAGAATAGGGTTGAACTTTGAACTCT	644
Bos	TC-TTGGTATAAAGGAAATAGTGGAAACTGGAAGCATAGACTGAGTATTCAAGACCATAGATGTAGAAAGA-AA---ACTAGAGTCAATGATCAAAC-CC	656
Canis	TCATTGGTGTGAAGGACATAATTGAAACTGGAAGCATAGGCTAAATATTCAAGGGCATGGGCATAGAAAGA-AAGACGGTAGGGTCAAGAACTGAAC-TT	878
Homo	GGGAAACATTTAGATTTATAGGGCAGGGGAAAGAGGAAGTATTACTGTTGAAGATAGAAGGGGAGAATAAAGAGTTAAGAGGAAAATCA-GGTAGTACTG	746
Pan	GGGAAACGTTTAGATTTATAGGGCAGGGGAAAGAGGAAGTGTTACTGTTGAAGATAGAAGGGGAGAATAAAGAGTTAAGAGGAAAATCAGGGTAGTACTG	747
Macaca	GGGAAACATTTAGATTTGTAGGGCAGGGGAAAGAGGAAGTATTACTGTTGAAGATAGAAGGGGAGAATAAAGAGTTAAGAGGAAAATCAGGGTAGAACTG	744
Bos	TGGGA---AACAGATTTATAGG-TGGAAGAAAAATAAAGGATTGGTGTTGAAGAAAAAGAAGG------GAGTGATAG---------------------T	725
Canis	GGGAACTGTTTAGATTTACAGGACAGGAGTAAGAGGAAGGGTTAGTGTTGAAGATGAAGAGAG------AAGTGATAGGAAGAAAACCAGAATGGTACAC	972
Homo	TACCAACTAAGCCAATGCAAGAAC-TATTTTATAAAAGAGCTTTGTTTTTGGCAGTATCAAATGCTATGCAAGACCAAGACTGATTATTTAATTTGGCTC	845
Pan	TACCAACTAAGCCAATGCAAGAAC-TATTTTATAAAAGAGCTTTGTTTTTGGCAGTATCAAATGCTGTGCAAGACCAAGACTGATTATTTAATTTGGCTC	846
Macaca	TAACAACTAAGCCAATGCAAGAAC-TATTTTATCAAAGAGCTTTGTTTTTGGCAGTTTCAAATGCTATGCAAGACCAAGGCTGATTAACTAATTTGGCTC	843
Bos	TACCAAGTAAGCCAATGTAGGCACAAGTTTTATAAAAGAGCTTTGATTTTGACTGTAGCAAATACTATGAAAGGTCAAGAATGAGGA--TTCAATGTTGC	823
Canis	TACCAAGTAAGCCAATACAAGAAAATATTTTGTAAAACAGCTTTGGTTTTGGCCAAATCAAATGATGTGAAAGGTCAAGAATTAGGATTTTCATGTGGGT	1072
Homo	TTTTGGATGTCCAAGAGAACAATTTCATTGCAAAGTCAGTTCTGTGAGCTCTATTAAAAGGAATTACATGGTATCAAGTGGTAAGCAGAATTGGCAAACT	945
Pan	TTTTGGATGTCCAAGAGAACAATTTCATTGCAAAGTCAGTTCTGTGAGCTCTATTAAAAGGAATTACATGGTATCGAGTGGTAAGCAGAATTGGCAAACT	946
Macaca	TTTTGGATGTCCAAGAGAACAATTTCATTGCAAAGTCAGTTCTGGGAGCTCTGTTGAAAGGAATTATATGGTATTGAGTGGTAAGCAGAATTGGCAAACT	943
Bos	TTTTGGATATTTAAGAGAACAGTTTGAATGCAAGGTTGGTTATGTGAGCCATGTGGAAAGGAATTATATGGCTGCGAGTGATGAGTAGAACTGGTAAACT	923
Canis	TTTTGGAGGTTCAAGAGGACAATTTCGATGCAAAGTTGGTTATGGGAGCCGTATTGAAAAGAATTATATGGCAATAAGGAATATGCAGAAATGGGAAACT	1172
Homo	TTCAAGAAGCTCATTCATGTAAAGAATGAGAGAGAAGCTTTGTATGGTTGTCTGGCAATGTTGGGAGAAGACTTCCGGAGGCAGTGAAGATCTGAGCGTG	1045
Pan	TTCAAGAAGCTCATTCATGTAAAGAATGAGAGAGAAGCTTTGTACGGTTGTCTGGCAATGTTGGGAGAAGACTTTTGGAGGCAGTGAAGATCTGAGCGTG	1046
Macaca	TTCAAGAAGCTCATTTATGTAAAGAATGAGAGAGAAGCTTTGTATGGTCCTCTGACAACATTGGGAGAAGACTTCTGGAGGCAATGATGATCTGACCGTG	1043
Bos	TTCAAGAGGTTTTGTCAT-TAAGGAATGAGAGAGATGTTTTTTGTAACCATCTGGCAGT-TGGGGAAGGGGCTTTTTGAGCTTGTAATGATTAGAGCTTG	1021
Canis	TTCAAGAAGTGTAGTCATGTAAAGAATAAGACAGATG-TTCTTGTGACT-------------GAGGAGGAGCTTCTTGAGCCAGTAGAAATCTGAGCATG	1258
Homo	TTTGAAGGCAGAAGGGATGGAGACTAAG---------ATTGCAGATATAAGGGGCTATGATGAGGGAA----GTGAAGATGGAAAGGGATGAGATACAGA	1132
Pan	TTTGAAGGCAGAAGGGATGGAGACTAAG---------ATTGCAGATATAAAGGGCTATGATGAGAGAA----GTGAAGATGGAAAGGGATGAGATACAGA	1133
Macaca	TTTGAAGGCAGAAGGGATGGAGACTAAG---------ATTGCAGATATAAGGGGCTATGATGAGAGAA----GTGAGGATGGAAAGGGATGAGATACAGA	1130
Bos	TTTGTAGGTGGAAAGGATGGTGACTAAGGAATAACATATTGAAGATACAGGGGGCTGTGATGGGAGAGAAGTGAGAAAATGGGAAGGGATGTGATACAGA	1121
Canis	TTTGTAGGCAGAAGGAATGGTGACTAAAGATTAAGAGATTGAAGACATA--GGGCAGTAGTTAGAGAGGAGTAAGACAATGGGAAAGGATGAGATGTGGA	1356
Homo	AAGCCAGCTGATGAGCCAGTCTGAGTGA--------TAATATTCAATGAAAGGAAGAACTT--------CAGAAATAATTTGAGATGAAGAGCAGGGAAG	1216
Pan	AAGCCAGCTGATGAGCCAGTCTGAGTGA--------TAATATTCAATGAAAGGAAGAACTT--------CAGAAATAATTTGAGATGAAGAGCAGGGAAG	1217
Macaca	AAGCCAAGTGATGAGCCAGTCTGAGTGA--------TAATATTCTGTGAAAGGAAGAACTT--------GAGAAATAATTTGAGATGAACAGCAGGGAAG	1214
Bos	AAATTGATGGATGAGCCTGTATAGGTGAAGAGGAGTTGATATTTTATGGAAGGAATTAGTT--------GAGAGATGATTTATGATACAGAGAAGAGAAA	1213
Canis	AAACAGGGGGATGAACCAGTCTGGGTGAAGAGGAGATCATAGTTTGTAGAAGGGAGAACTTGACACAGAGAGAGAGATTTTGAAATGAAGAGCAGGGAAA	1456
Homo	TTGAA---------GTCAATCTAGTAAGTAGACGATTGAAGCCTTGATAGCTACTTACTAGATAGATGAACTTGGGAAAGGTTAGAGAGGTTTCTAA---	1304
Pan	TTGAA---------GTCAATCTAGTAAGTAGATGATTGAAGCCTTGATAGCTACTTACTAGATAGATGAACTTGGGAAAGGTTAGAGAGGTTTCTAA---	1305
Macaca	TTGAA---------GTCATTCTAGTAAGTAGATGATTGAAGCCTTGATAGCTACTTATTAGATAGATGAACTTGGGAAAAGTTAGAGAGGTTTCTAA---	1302
Bos	CTGAAGAAGTTAATGTCAATCGAGTAAGTAGATGACT-AAGCCTTGATTGC-----------TGGATACATTTGGGAAAGCTTAGAGTGATTTCTGTAAA	1301
Canis	TTGAAGAATTTAGTGTCGATCTGGTAAGTAGATGACTGAGGCCTTGATAGC-----------TAGGTGAACTTGGGAAAGTGTAGAGGGGTTTTTGT---	1542
Homo	--AAGTACTG--	1312
Pan	--AAGTACTG--	1313
Macaca	--AAGTACTG--	1310
Bos	CGTGTCTGAGCTATCATGGCTTCCCTGATAGTTCAGTTGGTAAAGAATCCACCTGCAATGCAGGAGACCCTGGTTTGATTCCTGGGTTGGGAAGGTCCGC	1401
Canis	--CAGTAATG--	1550
Homo	--	1312
Pan	--	1313
Macaca	--	1310
Bos	TGGAGAAGGGATAGGCTACCTGCTACAGTATTCATGGGCTTCCCCTGTGGCTCAGCTGGTAAAGAATCTGCCCACAATGTGGGAGACCTGGGTTCTATTC	1501
Canis	--	1550
Homo	--TATCAGAGTAATAT	1326
Pan	--TATCAGAGTAATAT	1327
Macaca	--TATCAGAATAACAC	1324
Bos	CTGGGTTGGGAAGATCCCCTGGAGAAGGGAAAGGCTACTCACTCCAGTATTCTGACCTGGATAATTCCATGGATTGTATAGTCTATTGTCCGAGTAATAT	1601
Canis	--TGTCAGAGTAGTAC	1564
Homo	GGGCAAAACAGGAATTGG----GGGAATACCTGATAT--------------CACATATATATCCCAACAAACCCTCAGACTGTTCTATGTCTTGCATCGT	1408
Pan	GGGCAAAACAGGAATTGG----GGGAATACCTGATAT--------------CACATATATATCCCAACAAACCCTCAGACTGTTC--TGTCTTGCATCGT	1407
Macaca	GGG-GAAACAGGAATTGG----GGGAATACTTGATTT--------------CACATATATATCCCAACCAACCCTCAGACTGTTCTGTGTCTTACATCAT	1405
Bos	AGGTGAAACAATCAGAGG----GAACGTGCTTTGTATGTATTTCCTTAAGATAATTCTAT-TCCCAGCAAACCCTTAGTCTATTCTGTGTCTTGGGTCAC	1696
Canis	AGACAAAACAAGAATCAGTTTCTGATGTGCCTTATATGTATTGCCTTGAGACAGTTATTT-TCCCAACAAACCCATAGACTAATCTGTGTCATGTATCAC	1663
Homo	GCAAGTAGATGGTTAAGTCGCTGAAGTTGGTTATTTCAATATGCCACCAAGTAATGAGTGCTTCTTGGATATATAG	1484
Pan	GCAAGTAGATGGTTAAGTCGCTGAAGTTGGTTATTTCAATATGCCACCAAGTAATGAGTGCTTCTTGGATATATAG	1483
Macaca	GCAAGTAGA-GGTTAAGTCGCTGAAGTTGGTTATTTCAATATGCCACCAAGTAATGAGTGCTTCTTGGATATACAG	1480
Bos	GCAAATGGATTGTTAAGTCACTGAGCTG----ATTTATATATGCTTCCAAATAACAAGTGTTTCCTGGATGTACAG	1768
Canis	ACAAATCAATCG---AGTCACCAAAATGAGTTATTTAGATATGCTACCAAATGACAAGTGTTGCTTGGATGTACAG	1736

ENSG00000106077 intron 2

Description: abhydrolase domain containing 11 isoform 1 (ABHD11)
Intron number: 2
Human chromosome: 07
Intron start (bp): 72790002
Human intron length : 592
Intron alignment length: 728
Flanking exons length (upstream/downstream): 136/174
SNP density: 0.000000
K tree score: 0.0362
Scaling factor: 0.8915
Human-chimpanzee distance: 0.003380
Total primate branch length: 0.0752

ENSG00000106077 exon 2

```
Homo    GCCGCTTCCGCTTTCCTACAGGCTTCTGGACGGGGAGGCAGCCCTCCCGGCCGTCGTCTTTTTGCACGGGCTCTTCGGCA 80
Pan     GCCGCTTCCGCTTTCCTACAGGCTTCTGGACGGGGAGGCAGCCCTCCCGGCCGTCGTCTTTTTGCACGGGCTCTTCGGCA 80
Macaca  GCCGCTTCCGCTTTCCTACAGCCTTCTGGACGGGGAGGCAGCCCTCCCGGCCGTCATTTTTCTGCACGGGCTCTTCGGCT 80
Bos     ACCGGTGCGGCTTTCCTACAAGCTTCTGGACGGCGAGGCAGCTAGCCCCGCCCTTGTCTTTCTGCATGGGCTCTTCGGCA 80
Canis   GTCGGTGCTGCTTTCCTACAAGCTTCTGGACGGAGAGGCGGCTCGCCCGGCCCTCGTGTTTCTGCACGGGCTCTTCGGCT 80

Homo    GCAAAACTAACTTCAACTCCATCGCCAAGATCTTGGCCCAGCAGACAGGCCGTAGG 136
Pan     GCAAAACTAACTTCAACTCCATCGCCAAGATCTTGGCCCAGCAGACAGGCCGTAGG 136
Macaca  GCAAAACTAACTTCAACTCCATCGCCAAGGCCTTCGCCCAGCAGACAGGACGTAGG 136
Bos     GCAAAACCAACTTTAACTTCGTCGCCAAGACCCTGGCTCAGCAGACAGGCCGGAGG 136
Canis   CCAAAACCAACTTCAACTCCATCGCCAAGGCCCTGGCCCAGCAGACCGGCCGGAGA 136
```

ENSG00000106077 exon 3

```
Homo    GTGCTGACGGTGGATGCTCGTAACCACGGTGACAGCCCCCACAGCCCAGACATGAGCTACGAGATCATGAGCCAGGACCT 80
Pan     GTGCTGACGGTGGATGCTCGTAACCACGGTGACAGCCCCCACAGCCCAGACATGAGCTACGAGATCATGAGCCAGGACCT 80
Macaca  GTGCTGACGGTGGATGCTCGTAACCACGGTAACAGCCCCCACAGCCCAGACATGAGCTACGAGATCATGAGCCAGGACCT 80
Bos     GTGCTGACAGTGGATGCTCGGAACCATGGTGAGAGCTCCCACAGCCCAGACATGAGCTACGAGGCCATGAGCAAGGATCT 80
Canis   GTGCTTACAGTGGATGCTCGGAACCATGGTGACAGCCCCCACAGCCCAGAAATGAGCTACGAGGCCATGAGCCAGGACCT 80

Homo    GCAGGACCTTCTGCCCCAGCTGGGCCTGGTGCCCTGCGTCGTCGTTGGCCACAGCATGGGAGGAAAGACAGCCATGCTGC 160
Pan     GCAGGACCTTCTGCCCCAGCTGGGCCTGGTGCCCTGCGTCGTCGTTGGCCACAGCATGGGAGGAAAGACAGCCATGCTGC 160
Macaca  GCAGGACCTCCTGCCCCAGCTGGACCTGGTGCCCTGTGTCGTCGTTGGCCACAGCATGGGAGGGAGGACAGCCATGCTGC 160
Bos     GCAAGACCTCCTACCCCACCTGGGCCTGGTGCCCTGCGTCCTCATTGGCCACAGCATGGGAGGCAGGACCGCCATGCTGC 160
Canis   GCAGGACCTCCTGCCCCAGCTGGGCCTAGTGCCCTGCGTCCTCATTGGCCACAGCATGGGAGGCAAGACAGCCATGCTGC 160
```

ENSG00000106077 intron 2

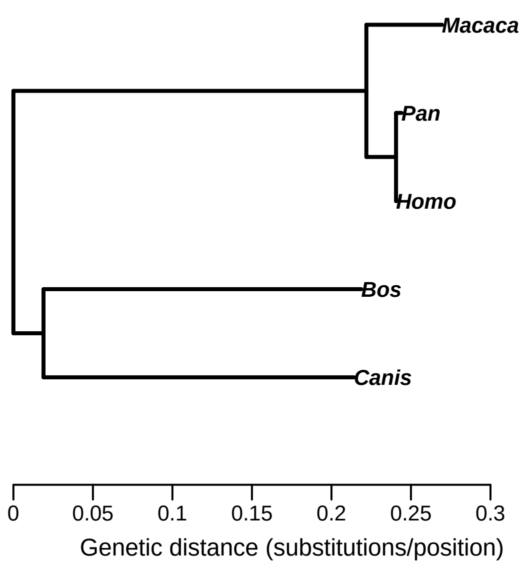

ENSG00000106077 intron 2

```
Homo    GTGAGCTTTCGAGTGGCGGGGCTCCTTTGG--GGCTGGGCGGGAGTGGGCGGGGCCTGAGAGGAGGCGGGGCAGGGGGGGTCCCGCTTCTC-------- 90
Pan     GTGAGCTTTCGAGTGGCGGGGCTCCTTTGG--GGCTGGGCGGGAGTGGGCGGGGCCTGAGAGGAGGCAGGGCA--GGGGGTCCCGCTTCTC-------- 88
Macaca  GTGCGCTTCC------------------------------GAGTGGGCGGGGCCTGGGAGGAGGCGGGGCT-GGGGCGTCCCGCTTCTC-------- 58
Bos     GTGAGCTTCTGGAGTCGGCGGGGCCCTGAGGGAGGTGGGGTCTGGGTGGGCGGGGCCCTAGAGGAGGCCGAGGT----GTGGCCAGCTTCTCTGTCCCTA 96
Canis   GTGAGCTTCCGGGAGAGGCGGGGTCCCGCAGGAGGTGGGGCCTGGCTGGGCGGGA-AGAGGGGGAGGCCGAGGC----GGGGCCCGCTTCTCTGCCCTTC 95

Homo    ----------------------------------ACGCTCACTTCCAGATCCCTCACTCTGAGTATGGACCCTGAACGGC------------------AACCCC 142
Pan     ----------------------------------ACGCTCACTTCCAGATCCCTCACTCTGAGTATGGACCCTGAACGGC------------------AACCCC 140
Macaca  ----------------------------------GGGGTCCC------GCTCCTCACGCTGAGTATGGACCCTGAACGGC------------------AACCCC 104
Bos     ACGCTTGGGAGCGGCGGGTCTCTCTGATAAGAAATCAAAGAAGAGATTGACCTTCTGAGGTTGGAGGCCCCAGGGCAGACCCAGGCCCACGACGAACCCC 196
Canis   CTGC----------------------------------------------AGGCCACCTGCCCAGGAGTACCGGCGCACCGGTACTGACTGACTCTGGGTGGACTTC 156

Homo    ATTTATTCCCTCACGTTGCCATCT------TCATTAATTCATGTTGTAGATCTCCATCGGGGCTGGCCC---------------------------- 205
Pan     ATTTATTCCCTCACGTTGCCATCT------TCATTAATTCATGTTGTAGATCTCCATCGGGGCTGGCCC---------------------------- 203
Macaca  GTTTGTTCCCTCACGTTGCCATCT------TCATTAATTCATGTTGTAGATCTCAATCGGCGCTGGCCA---------------------------- 167
Bos     ATTCATTCCCTGGTGCTTTCACCCACCCAGTCCTTCATTCGTATTATAGATCTCCGTCTGTGTGATCCACCCTGACTT-----------------AGCCT 279
Canis   ATTCATTCTCTCAGGCTTGCATCTATCCTGTCCTTCATTCGTGTTATAGACCTCCACGTGTGTGCTCCACGCTGAGCTTAGATGTCCTCATCATAAGGCT 256

Homo    ----------ATGCCTAGGCGCCTGCACCCTTTCCATGAAGGGG-------CCTGCTTGTTCACTAGGGATGACCGGGAGCTCACCACCTCTTATGGCAG 288
Pan     ----------ATGCCTAGGCGCCTGCACCCTTTCCATGAAGGGG-------CCTGCTTGTTCACTAGGGATGACCGGGAGCTCACCACCTCTTATGGCAG 286
Macaca  ----------ATGACTAGGCGCCTGCACCCTTTCCATGAAGGGG-------CCTGCTTGTTTCCTAGGGATGACCTGGAGCTCACCACCTCTTATGGCAG 250
Bos     CTGCTCTCAGATACCTAGGCATCAACATCCTGCCCGCTAAGGGGGCCAGACCCTGCTTGTTCACCAGGG--GATGACGGGCTTTCCACCTCCTGC----- 372
Canis   CAGCCGTCCAATGCCTAAACGCCAATAGCTTTTCGGTTAAGGGGGCCAGCCCCTGTTCGTTCACCCGGGATGACAGGGGGCTTACCACCTTTTACAGTAG 356

Homo    CCCATTCTTCTGTGCTCCTACTGTTGGAAATTCCTGGAGTCTCCTTCCTGGCATTCTCCGAGATTGCCACCCAGCCAGGCCAGCCCTTCAGAGACGTGGT 388
Pan     CCCATTCTTCTGTGCTCCTACTGTTGGAAATTCCTGGAGTCTCCTTCCTGGCATTCTCCGAGATTGCCACCCAGCCAGGCCAGCCCTTCAGAGACGTGGT 386
Macaca  CCCGTTCTTCTGTGCTCCTACTGTTGGAAATTCCTGGAGTCTCGTTCCTGGCATTCTCCGGGATTACCCCCAGCCTGGCCAGCCCTTCAGAGACGTGGT 350
Bos     CCCGCCCTGCTGTGCTCCAGCTGTTGCCAGTTCTTGCTGTCT--------GCTTTCTCTGTAGCTGCCAGCCAGCTAGGCCAAATGTCTGGAGATGTGGT 464
Canis   TCCATCCTTCTGTGCTCCAACTGTTGGAAATTCTTGGCACCT--------GCTTACTCTG-GGTGTTCACTCAACCAGGCCAAACCTTTAGAGACAGGGC 447

Homo    CACAACCCTGGGGCCTGCTCTTCTCTGGCAGAGTGGCCCCAGGTTCTTCATCTGGGCCTCCTAGGTCTGGAAA------------------CTGGGCCCT 470
Pan     CACAACCCTGGGGCCTGCTCTTCTCTGGCAGAGTGGCCCCAGGTTCTTCATCTGGGCCTCCTAGGTCTGGAAA------------------CTGGGCCCT 468
Macaca  CACAACCCTGGGGCCTGCTCTTCTCTGGCAGAGTGGCCCCAGGTTCTTCATCTGGGCCTTCTAGGTCTGGAAAC-TGGGCCCTCCCACCATCTGGGCCCT 449
Bos     CACACTCCTTGGGCCT-------TCTGGCACAGTGGCTCC-GGTTCTTCATCTGGGCCACCAAGGTCTGAGAACGGGGGTCTTCTCACCATCCGGGCCCT 556
Canis   CACACTCCTTGGGCCT-----TCTCTAGCTGAGTGACTCCATGTTCTTTATTT-GGTCTCCTAGGTCTGAGAAC-TGGGCCCCTCACCATTGGGTCTCT 540

Homo    TCCCCT-CCACAGGGTGTAT----CCCCAGGCTCTCCACACCTTTGACCTGCTGTCCCTACAA-GCCTTTGGCTGTCTACCCCCAGAATGGGGATATCAA 564
Pan     TCCCCT-CCACAGGGTGTAT----CCCCAGGCTCTCCACACCTTTGACCTGCTGTCCCTACAA-GCCTTTGGCTGTCTACCCCCAGAATGGGGATATCAA 562
Macaca  TCCCCT-CTGCAGGGTGTAT----CCCCAGGCTCTCCACACCCTTGACCTGCTGTCCCTACAAGGCCTTTGGCTGTCTA-CCCTAGAATGGGGATACCAG 543
Bos     TTCCCC-CTTCTGGATCTCCCCCACCCCGGGCTCTCCACCTCCCTCAGCCTGCAGATCTGCCAGGCTTTTGGCTCT----GCCCTGACT--GGATGCTAG 649
Canis   TCTTCCGCTTCAGGATCTTT----CTCCAGGCTCTCTACTCCCCTCAGCCTGCGCAAATGTCATGCCTTAGGCTGTCTGTGCCCTGAATGTGGGTGCCAG 636

Homo    CGCAGCCCCGTCTCACCCCACTCCCCAG 592
Pan     CGCAGCCCCGTCTCACCCCACTCCCTAG 590
Macaca  CGCAGCTCTGTCTCACCCCACTCCCCAG 571
Bos     CGCTGCTCCCCACCTCCCGCTTCCCCAG 677
Canis   CTCTGCCCACCCTCACTGCACTCTGCAG 664
```
